# The Spanish Polygenic Score reference distribution: a resource for personalized medicine

**DOI:** 10.1038/s41431-025-01850-9

**Published:** 2025-04-24

**Authors:** Rosario Carmona, Gema Roldán, Jose L. Fernández-Rueda, Arcadi Navarro, María Peña-Chilet, CSVS Crowdsourcing Group, Angel Alonso, Josefa Salgado-Garrido, Sara Pasalodos-Sanchez, Virginia Aquino, Javier Perez-Florido, Gerrit Bostelmann, Carmen Ayuso, Pablo Minguez, Almudena Avila-Fernandez, Marta Corton, Rafael Artuch, Salud Borrego, Guillermo Antiñolo, Angel Carracedo, Jorge Amigo, Luis Antonio Castaño, Isabel Tejada, Aitor Delmiro, Carmina Espinos, Daniel Grinberg, Encarnación Guillén, Pablo Lapunzina, Jose Antonio Lopez-Escámez, Alvaro Gallego-Martinez, Ramón Martí, Eulalia Rovira, José Mª Millán, Miguel Angel Moreno, Matías Morin, Antonio Moreno-Galdó, Mónica Fernández-Cancio, Beatriz Morte, Victoriano Mulero, Diana García, Virginia Nunes, Francesc Palau, Belén Perez, Rosario Perona, Aurora Pujol, Feliciano Ramos, Esther Lopez, Antonia Ribes, Jordi Rosell, Jordi Surrallés, Joaquín Dopazo, Daniel López-López

**Affiliations:** 1 Computational Medicine Platform, Andalusian Public Foundation Progress and Health-FPS, Sevilla, Spain; 2 Institute of Biomedicine of Seville, IBiS, University Hospital Virgen del Rocío/CSIC/University of Seville, Sevilla, Spain; 3 Centro de Investigación Biomédica en Red en Enfermedades Raras (CIBERER), ISCIII, Madrid, Spain; 4 IBE, Institute of Evolutionary Biology (UPF-CSIC), Department of Medicine and Life Sciences, Universitat Pompeu Fabra. PRBB, Barcelona, Spain; 5 Institució Catalana de Recerca i Estudis Avançats (ICREA) and Universitat Pompeu Fabra, Barcelona, Spain; 6 Center for Genomic Regulation (CRG), The Barcelona Institute of Science and Technology, Barcelona, Spain; 7 BarcelonaBeta Brain Research Center, Pasqual Maragall Foundation, Barcelona, Spain; 8 FPS/ELIXIR-ES, Andalusian Public Foundation Progress and Health-FPS, Sevilla, Spain; 9 Navarrabiomed-IdiSNA, Complejo Hospitalario de Navarra, Universidad Pública de Navarra (UPNA), IdiSNA (Navarra Institute for Health Research), Pamplona, Navarra, Spain; 10 Department of Genetics, Instituto de Investigación Sanitaria-Fundación Jiménez Díaz University Hospital, Universidad Autónoma de Madrid (IIS-FJD, UAM), Madrid, Spain; 11 Fundación para la Investigación y Docencia Sant Joan de Deu, Barcelona, Spain; 12 University Hospital Virgen del Rocío, Sevilla, Spain; 13 Fundación Pública Galega de Medicina Xenómica, SERGAS, IDIS, Santiago de Compostela, Spain; 14 Asociación Instituto de Investigación Sanitaria de Biocruces, Vizcaya, Spain; 15 Hospital Univ. 12 de Octubre, Madrid, Spain; 16 Centro de Investigación Príncipe Felipe, valencia, Spain; 17 Universidad de Barcelona, Barcelona, Spain; 18 Hospital Virgen de la Arrixaca, Murcia, Spain; 19 Servicio Madrileño de Salud, Madrid, Spain; 20 Department of Genomic Medicine, Centre for Genomics and Oncological Research (GENYO), Pfizer University of Granada, Granada, Spain; 21 Vall d’Hebron Institut de Recerca, Barcelona, Spain; 22 Fundación para la Investigación del Hospital la Fe, Valencia, Spain; 23 Servicio de Genética, Ramón y Cajal Institute of Health Research (IRYCIS) and Biomedical Network Research Centre on Rare Diseases (CIBERER), Madrid, Spain; 24 Vall d’Hebron Institut de Recerca (VHIR), Hospital Universitari Vall d’Hebron, Barcelona, Spain; 25 Undiagnosed Rare Diseases Programme (ENoD). Center for Biomedical Research on Rare Diseases (CIBERER), ISCIII, Madrid, Spain; 26 Universidad de Murcia, Murcia, Spain; 27 Fundación IDIBELL, Barcelona, Spain; 28 Universidad Autónoma de Madrid, Madrid, Spain; 29 Agencia Estatal Consejo Superior de Investigaciones Científicas, Madrid, Spain; 30 30Universidad de Zaragoza, Zaragoza, Spain; 31 Hospital Clínico y Provincial de Barcelona, Barcelona, Spain; 32 Fundación Instituto de Investigación Sanitaria Illes Baleares (IdISBa), Palma de Mallorca, Spain; 33 Universidad Autónoma de Barcelona, Barcelona, Spain

**Keywords:** Genome-wide association studies, Genome informatics, Genetic predisposition to disease, Risk factors, Predictive markers

## Abstract

Here we present the Polygenic Score (PGS) distributions for 3124 common diseases and quantitative traits observed in the Spanish population. To achieve so, the genomes and exomes of 2190 unrelated individuals of Spanish ancestry were used. The analysis covered a wide range of diseases and traits, including both complex disorders, such as various types of cancer, and disorders associated with the digestive, cardiovascular, neuronal, and immune systems, as well as quantitative traits like hematological and anthropometric measurements. The resulting PGS distributions provide valuable insights into the genetic architecture of the Spanish population, offering a comprehensive framework for investigating disease susceptibility and potential risk factors in this specific population. The study has also explored potential relationships between diseases and traits based on PGS pairwise correlations, revealing significant correlations that warrant further investigation. These findings have contributed to increase our understanding of the genetic basis of human traits and have implications for personalized medicine and public health interventions in the Spanish population. In addition, for the sake of reproducibility, we provide a data processing pipeline, enabling the computation of PGS for external genomes and exomes. The pipeline, accessible on GitHub, supports parallel tasks on various computing platforms and contributes to the standardization of PGS comparisons globally. Lastly, a user-friendly web interface facilitates the exploration of PGS reference distributions, featuring a detailed table, distribution plots, and filtering options. This interface enhances accessibility for researchers and clinicians, fostering informed decision-making based on population-specific PGS distributions. The PGS reference distributions can be explored at the SpPGS Atlas repository through the web interface: https://csvs.clinbioinfosspa.es/?tab=pgs.

## Introduction

Polygenic Scores (PGSs) have emerged as the method of choice for predicting genetic susceptibilities to a particular disease or trait [1]. PGSs are constructed by aggregating genetic variants, typically single nucleotide polymorphisms (SNPs) across the genome, from genome-wide association studies (GWAS), to produce a score related to the risk of developing specific diseases or phenotypes [2]. Thus, an analysis of PGS involves two steps, each involving different data: the base data, which includes the weights of the genotype-phenotype associations at the variant level, obtained during the PGS development, and the target data, which correspond to the PGS score for a specific individual, obtained from the sum of that individual’s variants weights [3].

In contrast to the traditional paradigm that associates disease risk primarily with specific mutations (e.g. *ATM*, *BRCA1/2*, *CHEK2*, and *PALB2* in breast cancer diagnosis [4]), the concept of PGS shifts this perspective. In the PGS framework, disease risk (or liability) arises from the additive effects of numerous genetic loci, each exerting a small, often subtle influence on the phenotype [5]. These loci function collectively within a complex network, together with environmental factors, ultimately shaping an individual’s susceptibility to a disease [2]. Interestingly, recent evidence suggested that in many common diseases, polygenic inheritance may exert a more substantial influence than rare monogenic, high-risk mutations [6, 7]. Moreover, it has been proposed that the variation in polygenic background explains the range of penetrance observed in carriers of high-risk variants [8]. Consequently, PGSs offer a quantitative measure of the cumulative effects of these common, small-effect genetic variants, shedding light on the collective influence of multiple pathways rather than a solitary mechanism [9].

PGSs have the potential to enhance the prediction of disease risks like breast cancer [10], prostate cancer [11], and type 1 diabetes [12], particularly in individuals of European descent, and may offer improvements over some current clinical models [13]. In particular, PGS have proven to be invaluable for risk stratification, as exemplified by a PGS developed for schizophrenia, where the top decile of the PGS distribution signifies a 7.8 to 20.3 times higher risk compared to the bottom decile [14]. A recent study on the UK Biobank data highlighted how individuals in the top 8%, 6.1%, 3.5%, and 1.5% of the PGS distribution face at least a threefold increased risk for coronary artery disease, atrial fibrillation, type 2 diabetes, inflammatory bowel disease, and breast cancer, respectively [1]. This relative risk estimation compared an individual’s genetic probability of developing a disease with that of the general population, which helped in making clinical and lifestyle decisions. For example, women in the top 10% of the PGS distribution may have reached the clinical risk threshold for mammographic screening in their early 30 s, while those in the bottom 10% may never have crossed that threshold [15]. Actually, combining PGS with family history proves useful for estimating absolute risk, providing a direct probability of an individual developing a disease and considering both their genetic profile and the baseline population risk in determining when to initiate screening [16]. Additionally, PGS can influence treatment decisions, as taking statins offers greater absolute risk reduction for coronary heart disease in individuals with a higher polygenic risk [17]. These findings provide clinicians with additional criteria for preventive treatment decisions [18].

The integration of PGS into clinical practice is a dynamic research frontier across numerous diseases [19]. By the end of 2022, a total of 22 active clinical trials were exploring PGS applications for various conditions [20]. Numerous experts anticipate that PGS of traits related to risk of disease phenotypes (polygenic risk scores, PRS) will evolve into a valuable tool for clinical care and personalized medicine [2, 18]. Nevertheless, several limitations must be addressed before the widespread adoption of PGS in clinical practice [1]. For instance, a recent study revealed that just 1.7% of white participants in the Mass General Brigham Biobank exceeded the PGS threshold linked to a 2-fold increased risk of type 2 diabetes, previously described [1], while the vast majority of black MGBB participants (88.9%) surpassed this threshold. These disparities stem from differences in allele frequencies and linkage disequilibrium patterns among different ethnic groups. Moreover, substantial variations in PGS distributions attributed to population ancestry have been highlighted by numerous studies [21–24]. However, quantiles of PGS distributions exhibit a strong correlation with the log(odds) of disease, and standardizing PGS can mitigate this variability [22]. This underscores the need to tailor PGS to population-specific structures and address observed differences in PGS distributions across various populations to establish ethnicity-specific reference ranges. At the individual level, comparing one’s PGS to a population-specific distribution is essential for robust interpretation [18]. Regrettably, reference distributions are currently lacking for most population groups.

Here, the Spanish PGS Reference Distribution Atlas (SpPGS Atlas), a comprehensive resource tailored for the Spanish population, is presented. The SpPGS Atlas encompasses 3124 PGS distributions covering a wide range of common diseases, quantitative traits, and more. These include PGS for cancer, disorders affecting the digestive, cardiovascular, neuronal, and immune systems, as well as quantitative traits like hematological measurements and anthropometric levels. To estimate these PGS, genomic or exomic data corresponding to 2190 unrelated Spanish individuals from the Collaborative Spanish Variant Server (CSVS) initiative [25], was used. To better support its utility, the pipeline employed for preprocessing, phasing, and imputing samples in our reference cohort, optimized for parallel processing of large cohorts, was also made available. Together, these resources enable the computation of PGS for new genomes or exomes, aligning them with specific reference distributions from the SpPGS Atlas in a standardized manner. This aids in PGS selection, patient risk stratification, and precise calibration of absolute risk values for the Spanish population, facilitating and fostering the integration of PGS into the Spanish healthcare system. Moreover, this adaptable approach can be also used to establish population-specific PGS distributions for other populations, streamlining the global adoption of PGS in healthcare systems.

## Materials and methods

### Data preprocessing

This study used data from the Collaborative Spanish Variant Server (CSVS), which stores genomic and exomic data on 2190 unrelated individuals of Spanish ancestry, with an approximate composition of 50% of each sex, and stratified by ICD10 upper categoriesy [25]. The dataset includes contributions from various Spanish consortiums and projects, such as the Medical Genome Project [26], the EnoD project from the Spanish Network for Research in Rare Diseases (CIBERER) [27], the Project Genome 1000 Navarra [28], the RareGenomics project, and other research groups and initiatives across Spain. To ensure methodological accuracy, an initial data cleaning and a VCF correction process were carried out. This implied the removal of contig prefix names such as “chr” and the INFO and FORMAT fields, filtering out non-pass variants, and subsequently sorting the VCFs using bcftools v1.14 [29]. Additionally, the VCFs were left-normalized to eliminate duplicate variants, and non-standard chromosomes along with the Y chromosome were excluded. For exome data, variants beyond the shared captured region were excluded to mitigate potential biases in allele frequencies arising from slight disparities in the captured regions among different technologies and versions. Lastly, samples were consolidated by chromosome, resulting in the creation of two distinct sets of multisample VCFs: one for genomes and another for exome samples.

### Missing variants imputation

Various factors impact the accuracy of Next-generation sequencing (NGS), including the sequencing kit, sequencing depth, software tools utilized for sequence variant detection, and inherent DNA sequence characteristics (*e.g*., GC content, presence of repetitive elements). To mitigate potential biases stemming from the diverse origins of the samples in the Collaborative Spanish Variant Server (CSVS) database, a standardized protocol for variant imputation was implemented. Initially, haplotype samples were estimated using SHAPEIT v4 [30], with adjustments made to the default parameters to suit the sequencing data. The genetic map b37 was directly acquired from the SHAPEIT gitlab repository and used in the analysis. Subsequently, pre-phased VCFs for pseudoautosomal regions (PAR1 and PAR2) were concatenated using bcftools v1.14 [29]. Finally, imputation of autosomal, pseudoautosomal, and non-pseudoautosomal VCFs was conducted using minimac v4 [31] and the 1000 genomes Phase 3 reference panel [32].

### PGS source and calculations

The Polygenic Score (PGS) catalog [33] is used as a source of PGSs. At the moment of carrying out this work (February 2024) the PGS catalog contained a total of 3138 PGSs corresponding to 538 traits. Several diseases or traits are represented by numerous PGS within the catalog, often resulting from diverse training methods or cohorts with similar population structures, leading to redundancy. For this work, all PGSs were computed for the imputed VCF files using the pgsc_calc v1.3.1 software [34].

### PGS pairwise correlations

Pearson correlation coefficients and *p*-values for the PGS distributions for the Spanish population were calculated with SciPy v1.9.3 [35]. *P*-values were then adjusted for multiple testing (q-value) using the Benjamini-Hochberg false discovery rate method [36] implemented in the Python package Statsmodels v0.14.0 [37]. For further analysis, we considered only pairwise correlations with FDR-adjusted *p*-value < 10^−6^.

### Web architecture

In order to enhance the exploratory analysis of polygenic score reference distributions, a specialized web server was designed. The front end of this server was constructed using the highcharts v4.2.5 responsive JavaScript library along with the Polymer v1.9 framework.

For the back-end infrastructure, the Java programming language was used. Data were stored and managed within a MongoDB server, a document-based NoSQL database known for its efficiency and flexibility in storing a wide array of dataset types. The choice of MongoDB was particularly apt for accommodating the diverse nature of datasets associated with polygenic scores.

## Results

### PGS calculation

All the 3138 PGSs available in the PGS catalog [33] at the moment of carrying out this work were computed for the imputed VCFs through the utilization of pgsc_calc v1.3.1 software [34]. However, 14 PGS calculations proved unfeasible due to restricted variability within the CSVS cohort. Consequently, a total of 3124 PGSs, corresponding to a total of 538 traits, were successfully generated for both, genome and exome data, within the CSVS dataset. Supplementary Table S1 lists the PGS used in this study.

### Spanish PGS reference distribution

**Fig. 1:**
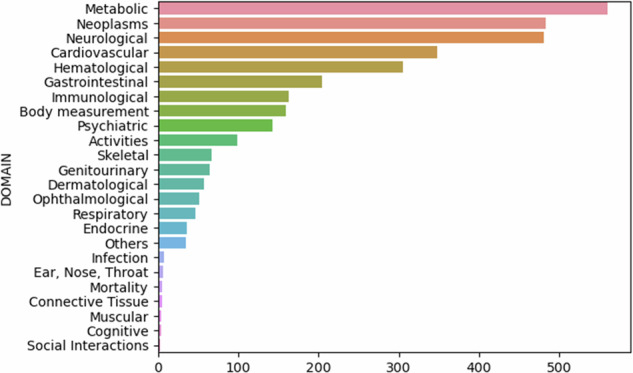
Number of PGSs in the SpPGS Atlas aggregated by disease domain.

The Spanish PGS reference presented here comprises a dataset of 3124 PGS distributions derived from the CSVS cohort of unrelated Spanish individuals (Fig. 1). This dataset encompasses a wide range of PGS predicting risks for diverse diseases and quantitative traits, sourced from the PGS catalog [33]. It includes over 500 PGS related to various cancer types, such as breast, prostate, colorectal, lung, and ovarian carcinoma. Furthermore, the dataset contains PGS designed to assess the likelihood of disorders associated with the digestive, cardiovascular, neuronal, and immune systems, such as celiac disease, coronary artery disease, or Alzheimer’s disease. Additionally, it covers PGS for metabolic conditions like obesity and diabetes, as well as those predicting quantitative traits like hematological measurements, anthropometric levels, and behavioral tendencies. Supplementary Table S1 contains the complete list of PGSs included in the current reference release. Notably, it’s important to highlight the underrepresentation of PGS related to drug response in the PGS catalog, despite the crucial role of pharmacogenomics in personalized medicine.

**Fig. 2:**
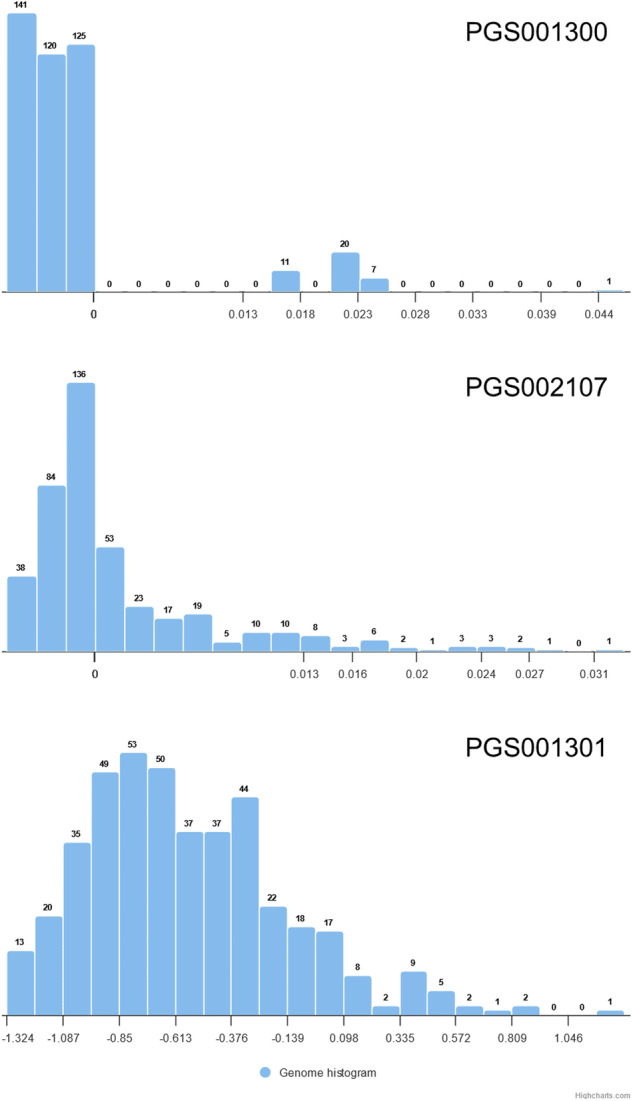
Individual PGS reference distributions observed in the Spanish samples analyzed for the three celiac disease PGS that appear in Table 1.

For each PGS from the PGS catalog, the distribution of scores obtained for individuals tends to follow a normal pattern, mirroring the trend observed in other studies [1]. This normal distribution indicates that the majority of individuals possess PGS values close to the mean, with only a limited number exhibiting significant deviations. Notably, significant diversity exists in both the mean and standard deviation of these distributions, even when considering PGS designed for the same trait or disease, as observed in general PGS repositories, like the PGS catalog [33]. To illustrate how different such values can be, Fig. 2 depicts the score distributions in the Spanish population obtained for three PGS of celiac disease (PGS001300, PGS001301 and PGS002107), that yielded distinct means (−0.0013, −0.504 and 0.0012, respectively) and standard deviations (0.0073, 0.423 and 0.0059, respectively).

As previously mentioned, many diseases or traits in the PGS catalog are redundantly represented by multiple PGS. For example, there are currently 76 PGS for breast carcinoma, all trained and evaluated in a European cohort. This redundancy extends to PGS associated with different Experimental Factor Ontology labels [38], such as those linked to fat body mass. This highlights the need to unify the PGS by disease or condition, combining PGS with tools like MultiPRS [39] or choosing a consensus PGS based on performance metrics.

### Potential relationships between diseases and traits based on PGS pairwise correlations

To investigate potential relationships between different PGSs, pairwise correlations were calculated within the entire PGS dataset. The results showed high correlation values, either positive or negative, between certain clinical phenotypes and traits that are commonly used in the diagnosis of the disease. A total of 71,723 pairwise correlations (1.46% of the total number of PGS pairs) showed a significant (FDR-adjusted *p*-value < 10^−6^) correlation (See Supplementary Table S2).

While evaluating all possible pairwise correlations falls beyond the scope of this study, some interesting examples of the most significant (FDR-adjusted *p*-value < 10^−9^) and highest positive and negative correlations, excluding pairs of traits with an obvious conceptual relationship (like, for example, obesity and body mass index, *r* = 0.9945) are listed in Table 1. Also, other known relationships, like myocardial infarction and aspirin consumption (*r* = 0.3303) emerged from this study [40]. Additionally, traits can be negatively correlated as, for example, the calcaneal bone quantitative ultrasound measurement (*r* = −0.8153), used to estimate bone density and strength, was negatively correlated with osteoporosis. Some unexpected and recently described relationships between mental diseases and longevity (*r* = −0.6893) arose [41]. An in-depth exploration of the whole pairwise correlations table (refer to Supplementary Table S2) might reveal intriguing and previously unknown relationships that could be further investigated in subsequent research.

**Table 1:** Correlations among traits with high statistical significance (FDR-adjusted *p*-value < 10^−9^) and are not conceptually related.

Mapped trait(s) (EFO label) 1	Mapped trait(s) (EFO label) 2	PGS 1 ID	PGS 2 ID	CC	*p*-value
Celiac disease	Varicella zoster virus seropositivity	PGS001300	PGS001399	0.8537	1.4 × 10^−127^
Vitamin supplement exposure measurement	Multiple sclerosis	PGS001045	PGS001271	−0.8158	5.6 × 10^−107^
Multiple sclerosis	Folic acid measurement	PGS001271	PGS001153	−0.8148	1.8 × 10^−106^
Dementia	Longevity	PGS000929	PGS002795	−0.6893	8.8 × 10^−63^
Alzheimer disease	Longevity	PGS001349	PGS002795	−0.6706	3.6 × 10^−58^
Autoimmune disease	Myxedema / hypothyroidism	PGS002629	PGS000965	0.6306	2.5 × 10^−49^
Varicella zoster virus seropositivity	Lung carcinoma	PGS001399	PGS003391	0.5678	7.8 × 10^−38^
Atrial fibrillation	Appendicitis	PGS002773	PGS001369	0.5670	1.0 × 10^−37^
Celiac disease	Insulin use measurement	PGS002107	PGS001117	0.5640	3.3 × 10^−37^
Connective tissue disease	Polymyalgia rheumatica	PGS000960	PGS001051	0.4678	6.6 × 10^−24^
Alzheimer disease	Apolipoprotein B measurement	PGS000779	PGS000672	0.3921	4.5 × 10^−16^
Alkaline phosphatase measurement	Thrombotic disease	PGS000670	PGS000930	−0.3763	1.1 × 10^−14^
Estrogen-receptor-positive breast cancer	Age at death / female	PGS000002	PGS000318	0.3696	4.1 × 10^−14^
Vitiligo	Folic acid measurement	PGS001536	PGS001153	0.3484	2.2 × 10^−12^
Aspirin use measurement	Myocardial infarction	PGS001112	PGS001314	0.3303	5.0 × 10^−11^
Celiac disease	Multiple sclerosis	PGS001301	PGS001270	0.3266	9.2 × 10^−11^

Correlations were obtained using only the 476 individuals for which the whole genome was available.

### Pipeline of data processing

A comprehensive pipeline has been developed for preprocessing, phasing, and imputing genomes and exomes (see Material and Methods for details on these procedures). The objective of this pipeline is to promote reproducibility by facilitating the standardization of data preprocessing so that results can be comparable. This pipeline ensures the inclusion of non-coding variants and those missed due to technology limitations or sample quality. Developed with Nextflow, it supports parallel tasks on various computing platforms, including GridEngine, SLURM, LSF, PBS, Moab, HTCondor batch schedulers, as well as cloud-based platforms like Kubernetes, Amazon AWS, Google Cloud, and Microsoft Azure, using Docker/Singularity containers for easy setup and reproducibility. The Nextflow DSL2 implementation employs a single container per process, simplifying software management. The pipeline was used to compute polygenic score reference distributions for a Spanish cohort but is applicable to external samples. This facilitates standardized PGS comparisons and the creation of population-specific PGS distributions for global healthcare integration.

The pipeline is freely accessible at: https://github.com/babelomics/SpPGS.

### Web interface: features and functionalities

**Fig. 3:**
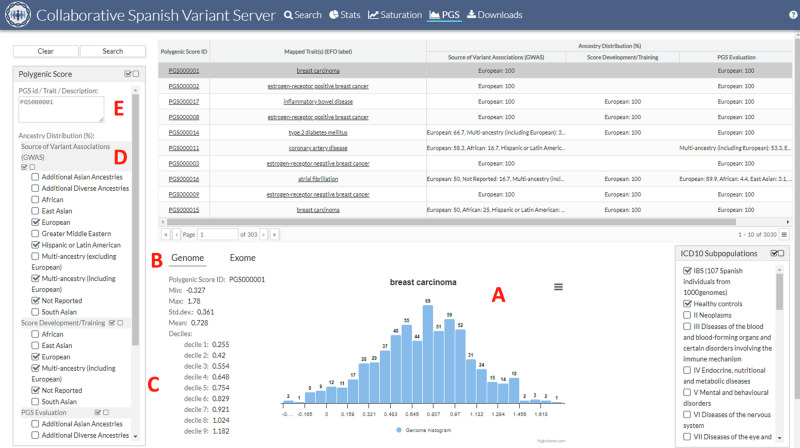
The PGS web interface. **A** Frequency histogram plot. **B** Selector for genome and exome distributions. **C** Various distribution parameters such as mean, standard deviation, and deciles. **D** Search and filtering panel, **E** PGS panel.

A user-friendly web interface was developed to simplify the exploration of polygenic score (PGS) reference distributions, consisting of three key sections portrayed in Fig. 3. Firstly, the top section of the page displays a table that provides the whole list of PGS, along with their associated Experimental Factor Ontology labels [38], the population ancestries used for extracting genome-wide variant associations, and the population origin employed for PGS training and evaluation. By default, only PGS trained and evaluated in the European population are displayed. Secondly, in the lower section of the page, users can access specific PGS distribution details, including a frequency histogram plot (Fig. 3A). Users can switch between genome and exome distributions using the tab button (Fig. 3B). Various distribution parameters such as mean, standard deviation, and deciles are also displayed (Fig. 3C). The individuals used to plot the frequency histogram and calculate the distribution parameters can be filtered by selecting the corresponding subpopulations (Fig. 3D). Lastly, the left side of the page contains the search and filtering panel (Fig. 3E), allowing users to search for PGS using PGS Catalog identification (e.g., PGP000198) or disease/trait description (*e.g*., breast cancer). Further filtering options are available based on the population ancestries used during development, training, and evaluation phases.

## Discussion

Despite the clinical utility of PGS being increasingly recognized [23], the transition from research to clinical application remains to be made. Recent years have witnessed a gradual shift from PGS research to their clinical integration, marked by notable methodological refinements [42], reporting standards [43] and comprehensive cataloging efforts [33, 44]. However, and notwithstanding these advancements, the clinical implementation of PGS is still in its early stages. One key challenge stems from the population-specific nature of PGS, which necessitates precise calibration and interpretation at populational level. Research has demonstrated that translating absolute risk estimates across populations with varying allele frequencies and linkage disequilibrium patterns is problematic [22]. Actually, even within the same ancestry group differences can arise due to individual characteristics such as socio-economic status, age or sex [45]. Thus, several authors have advocated for the stratification of individuals based on quantiles within the PGS distribution, offering a relative risk perspective [1, 15, 22]. Therefore, when assessing an individual’s PGS, it is imperative to benchmark it against a population-specific distribution for meaningful insights. However, to our knowledge, large-scale initiatives dedicated to such population-specific resources other than the UK Biobank are lacking and, specifically, there is a notable absence of PGS reference datasets tailored to the Spanish population. This knowledge gap underscores the pressing need for population-specific PGS references and resources, essential for the effective deployment of PGS in clinical settings and healthcare systems.

Here we introduce the SpPGS Atlas, a comprehensive database containing 3124 distinct PGS distributions encompassing common diseases and quantitative traits. These scores were derived from a cohort of 2105 unrelated Spanish individuals. Additionally, we are providing access to the pipeline employed for generating these distributions, optimized for high-performance computing and cloud-based environments. The use of a bioinformatics pipeline enhances the reproducibility, portability, modularity, and scalability of the analysis, providing a more robust and efficient approach to processing and analyzing biological data, especially for large-scale and complex analyses. These combined resources offer invaluable support for relative risk assessment in patient stratification and hold the potential to serve as a cornerstone for the widespread integration of PGS within the Spanish healthcare system. Furthermore, this pioneering framework can be readily extended to formulate population-specific PGS references for other populations, streamlining the global adoption of PGS within healthcare systems.

In the Spanish population, most PGS conform to a normal distribution, aligning with broader research indicating that the majority of individuals cluster around the mean, with fewer exhibiting significant deviations [1]. Notably, there’s considerable variability in both the mean and the standard deviation of these distributions, even within PGS developed for the same disease. For example, breast carcinoma PGS like PGS001778 and PGS002242 display distinct means (0.572 and 3.04) and standard deviations (0.307 and 0.397). Factors contributing to these differences encompass PGS generation methodologies, specific genetic variants, associated weights, and variations in sample preprocessing. Consequently, it’s imperative to compare an individual’s PGS with a reference distribution for the same PGS.

Remarkably, the PGS catalog lacks comprehensive representation of PGS related to drug response, a critical aspect in personalized medicine. Studies carried out in the Estonian biobank, which found 99.8% of individuals with genotypes linked to adverse reactions [46], or in the Spanish population, where 98% of individuals carry alleles associated with therapeutic change [47] highlight the importance of this type of genetic variability and underscore this gap. The limited focus on drug response genetics may stem from higher associated costs, particularly for follow-up studies, compared to disease association genetics.

Beyond the relevance of PGS distributions, their correlations also reveal interesting relationships shedding light on interconnected health indicators. Many of the correlated traits listed in Supplementary Table 2 reflect known relationships among them as, for example, the serum alanine aminotransferase measurement (PGS002732), which is a common test used to assess liver health, is almost perfectly correlated (*r* = 0.9906) with non-alcoholic fatty liver disease (PGS002282). Similarly, the HbA1c measurement (PGS002403), used to evaluate average blood sugar levels, is highly correlated (*r* = 0.9425) with diabetes mellitus (PGS002543). Also, Alzheimer disease (PGS000779) and apolipoprotein B measurement (PGS000672) are known to be positively correlated [48]. An example of negatively correlated traits is the calcaneal bone quantitative ultrasound measurement, used to estimate bone density and strength, displaying a negative correlation (*r* = −0.8153) with osteoporosis (PGS002768). Interestingly, some diseases known to have a significant heritability component are highly correlated with the trait family history of the own disease. Examples include Alzheimer’s disease (PGS001347, PGS001349, *r* = 0.9684), breast cancer (PGS001337, PGS001336, *r* = 0.7952) or cardiovascular disease (PGS001324, PGS001335, *r* = 0.6221). Even more interesting are other highly correlated traits that do not reflect obvious connections among them [41]. In the top range of correlations, Table 1 shows how Varicella zoster virus seropositivity (PGS001399) displays a high correlation (0.8537) with celiac disease (PGS001300). Actually, it has been described that individuals with celiac disease have a 1.62 times higher risk for herpes zoster [49]. Moreover, there is growing evidence linking viral infections, such as herpesviruses, with the development of multiple autoimmune disorders [49]. Figure 2 shows the individual reference distributions of scores in the Spanish population for the three celiac disease PGS that appear in Table 1. While the first one, corresponding to the PGS001399, might result in a higher correlation because of the skewness, the other two ones present distributions closer to a normal one, which would support the relationship between celiac disease and risk for herpes zoster already described [49]. Other relevant correlations, supported by previous studies are the correlation between Alzheimer disease and longevity [41] and the correlation between dementia and longevity [41], also supported by previous studies. However, caution is advised when drawing conclusions from the correlations found in this study between PGS, as artifacts and significant biases may artificially inflate these correlations, necessitating further detailed investigation.

The informative potential of PGSs on disease risk suggests that they are likely to become a valuable tool in clinical care and contribute to personalized medicine, as highlighted by various authors [2, 18, 21, 23]. The Spanish PGS reference distributions database presented here facilitates the calibration of PGSs within the Spanish population for numerous common diseases, including coronary artery disease, atrial fibrillation, type 2 diabetes, inflammatory bowel disease, breast cancer, or schizophrenia, among others. Nevertheless, achieving accurate calibration of PS at the individual level necessitates substantial effort. A model may consistently over- or underestimate risks for different risk strata if the underlying disease incidence rate used is not representative of the desired population for prediction [50]. Consequently, after constructing a model for relative risk, evaluating absolute risk requires estimating the baseline hazard against which relative risks are assessed. Utilizing data from representative cohort studies or population-based registries can aid in estimating the baseline rate from the overall rate of disease prevalence.

The integration of PGS into predictive risk models is akin to combining clinical and biochemical data, lifestyle factors, and medical history to predict the risk of cardiovascular diseases and diabetes. With regular updates planned for the SpPGS Atlas, the inclusion of more diseases and more accurate predictors can be anticipated, paving the way for widespread implementation in the Spanish clinical health system. Furthermore, PGS can aid decisions regarding participation in screening programs, lifestyle modifications, preventive treatments, and can be relevant at various stages of disease diagnosis and progression.

Although this study has focused on the Spanish population, the strategy applied can easily be replicated to develop population-specific PGS distributions for other populations, facilitating the adoption of PGS in healthcare systems worldwide.

## Supplementary information

Supplementary Table S1

Supplementary Table S2

## Data Availability

The web server is part of the CSVS initiative and can be found at https://csvs.clinbioinfosspa.es/?tab=prs. Trait pairwise correlations (Supplementary Table 2) can be found in Zenodo (https://zenodo.org/doi/10.5281/zenodo.10000253).

## Code availability

The pipeline used is available in Zenodo (10.5281/zenodo.7919159).
