# Supplementary material for: The Spanish Polygenic Score reference distribution: a resource for personalized medicine
Source: Eur J Hum Genet. 2025 Apr 24;34(2):270–7. doi: 10.1038/s41431-025-01850-9 (PMC12859145; doi:10.1038/s41431-025-01850-9)
Supplement: Supplementary file 1 — Supplementary Table S1 [file 41431_2025_1850_MOESM1_ESM.pdf]

Polygenic Score (PGS) ID	Mapped Trait(s) (EFO ID)	Mapped Trait(s) (EFO label)	Trait (ontology term label)	Trait Identifier (ontology ID)	Trait Category	DOMAIN
PGS000001	EFO_0000305	breast carcinoma	breast carcinoma	EFO_0000305	Cancer	Neoplasms
PGS000004	EFO_0000305	breast carcinoma	breast carcinoma	EFO_0000305	Cancer	Neoplasms
PGS000007	EFO_0000305	breast carcinoma	breast carcinoma	EFO_0000305	Cancer	Neoplasms
PGS000015	EFO_0000305	breast carcinoma	breast carcinoma	EFO_0000305	Cancer	Neoplasms
PGS000028	EFO_0000305	breast carcinoma	breast carcinoma	EFO_0000305	Cancer	Neoplasms
PGS000045	EFO_0000305	breast carcinoma	breast carcinoma	EFO_0000305	Cancer	Neoplasms
PGS000051	EFO_0000305	breast carcinoma	breast carcinoma	EFO_0000305	Cancer	Neoplasms
PGS000052	EFO_0000305	breast carcinoma	breast carcinoma	EFO_0000305	Cancer	Neoplasms
PGS000072	EFO_0000305	breast carcinoma	breast carcinoma	EFO_0000305	Cancer	Neoplasms
PGS000153	EFO_0000305	breast carcinoma	breast carcinoma	EFO_0000305	Cancer	Neoplasms
PGS000317	EFO_0000305	breast carcinoma	breast carcinoma	EFO_0000305	Cancer	Neoplasms
PGS000332	EFO_0000305	breast carcinoma	breast carcinoma	EFO_0000305	Cancer	Neoplasms
PGS000335	EFO_0000305	breast carcinoma	breast carcinoma	EFO_0000305	Cancer	Neoplasms
PGS000344	EFO_0000305	breast carcinoma	breast carcinoma	EFO_0000305	Cancer	Neoplasms
PGS000345	EFO_0000305	breast carcinoma	breast carcinoma	EFO_0000305	Cancer	Neoplasms
PGS000472	EFO_0000305	breast carcinoma	breast carcinoma	EFO_0000305	Cancer	Neoplasms
PGS000473	EFO_0000305	breast carcinoma	breast carcinoma	EFO_0000305	Cancer	Neoplasms
PGS000474	EFO_0000305	breast carcinoma	breast carcinoma	EFO_0000305	Cancer	Neoplasms
PGS000475	EFO_0000305	breast carcinoma	breast carcinoma	EFO_0000305	Cancer	Neoplasms
PGS000476	EFO_0000305	breast carcinoma	breast carcinoma	EFO_0000305	Cancer	Neoplasms
PGS000477	EFO_0000305	breast carcinoma	breast carcinoma	EFO_0000305	Cancer	Neoplasms
PGS000478	EFO_0000305	breast carcinoma	breast carcinoma	EFO_0000305	Cancer	Neoplasms
PGS000479	EFO_0000305	breast carcinoma	breast carcinoma	EFO_0000305	Cancer	Neoplasms
PGS000480	EFO_0000305	breast carcinoma	breast carcinoma	EFO_0000305	Cancer	Neoplasms
PGS000481	EFO_0000305	breast carcinoma	breast carcinoma	EFO_0000305	Cancer	Neoplasms
PGS000482	EFO_0000305	breast carcinoma	breast carcinoma	EFO_0000305	Cancer	Neoplasms
PGS000483	EFO_0000305	breast carcinoma	breast carcinoma	EFO_0000305	Cancer	Neoplasms
PGS000484	EFO_0000305	breast carcinoma	breast carcinoma	EFO_0000305	Cancer	Neoplasms
PGS000485	EFO_0000305	breast carcinoma	breast carcinoma	EFO_0000305	Cancer	Neoplasms
PGS000486	EFO_0000305	breast carcinoma	breast carcinoma	EFO_0000305	Cancer	Neoplasms
PGS000487	EFO_0000305	breast carcinoma	breast carcinoma	EFO_0000305	Cancer	Neoplasms
PGS000488	EFO_0000305	breast carcinoma	breast carcinoma	EFO_0000305	Cancer	Neoplasms
PGS000489	EFO_0000305	breast carcinoma	breast carcinoma	EFO_0000305	Cancer	Neoplasms
PGS000490	EFO_0000305	breast carcinoma	breast carcinoma	EFO_0000305	Cancer	Neoplasms
PGS000491	EFO_0000305	breast carcinoma	breast carcinoma	EFO_0000305	Cancer	Neoplasms
PGS000492	EFO_0000305	breast carcinoma	breast carcinoma	EFO_0000305	Cancer	Neoplasms
PGS000493	EFO_0000305	breast carcinoma	breast carcinoma	EFO_0000305	Cancer	Neoplasms
PGS000494	EFO_0000305	breast carcinoma	breast carcinoma	EFO_0000305	Cancer	Neoplasms
PGS000495	EFO_0000305	breast carcinoma	breast carcinoma	EFO_0000305	Cancer	Neoplasms
PGS000496	EFO_0000305	breast carcinoma	breast carcinoma	EFO_0000305	Cancer	Neoplasms
PGS000497	EFO_0000305	breast carcinoma	breast carcinoma	EFO_0000305	Cancer	Neoplasms
PGS000498	EFO_0000305	breast carcinoma	breast carcinoma	EFO_0000305	Cancer	Neoplasms
PGS000499	EFO_0000305	breast carcinoma	breast carcinoma	EFO_0000305	Cancer	Neoplasms
PGS000500	EFO_0000305	breast carcinoma	breast carcinoma	EFO_0000305	Cancer	Neoplasms
PGS000501	EFO_0000305	breast carcinoma	breast carcinoma	EFO_0000305	Cancer	Neoplasms
PGS000502	EFO_0000305	breast carcinoma	breast carcinoma	EFO_0000305	Cancer	Neoplasms
PGS000503	EFO_0000305	breast carcinoma	breast carcinoma	EFO_0000305	Cancer	Neoplasms
PGS000504	EFO_0000305	breast carcinoma	breast carcinoma	EFO_0000305	Cancer	Neoplasms

PGS000505	EFO_0000305	breast carcinoma	breast carcinoma	EFO_0000305	Cancer	Neoplasms
PGS000506	EFO_0000305	breast carcinoma	breast carcinoma	EFO_0000305	Cancer	Neoplasms
PGS000507	EFO_0000305	breast carcinoma	breast carcinoma	EFO_0000305	Cancer	Neoplasms
PGS000508	EFO_0000305	breast carcinoma	breast carcinoma	EFO_0000305	Cancer	Neoplasms
PGS000509	EFO_0000305	breast carcinoma	breast carcinoma	EFO_0000305	Cancer	Neoplasms
PGS000510	EFO_0000305	breast carcinoma	breast carcinoma	EFO_0000305	Cancer	Neoplasms
PGS000511	EFO_0000305	breast carcinoma	breast carcinoma	EFO_0000305	Cancer	Neoplasms
PGS000512	EFO_0000305	breast carcinoma	breast carcinoma	EFO_0000305	Cancer	Neoplasms
PGS000513	EFO_0000305	breast carcinoma	breast carcinoma	EFO_0000305	Cancer	Neoplasms
PGS000514	EFO_0000305	breast carcinoma	breast carcinoma	EFO_0000305	Cancer	Neoplasms
PGS000515	EFO_0000305	breast carcinoma	breast carcinoma	EFO_0000305	Cancer	Neoplasms
PGS000516	EFO_0000305	breast carcinoma	breast carcinoma	EFO_0000305	Cancer	Neoplasms
PGS000517	EFO_0000305	breast carcinoma	breast carcinoma	EFO_0000305	Cancer	Neoplasms
PGS000518	EFO_0000305	breast carcinoma	breast carcinoma	EFO_0000305	Cancer	Neoplasms
PGS000519	EFO_0000305	breast carcinoma	breast carcinoma	EFO_0000305	Cancer	Neoplasms
PGS000520	EFO_0000305	breast carcinoma	breast carcinoma	EFO_0000305	Cancer	Neoplasms
PGS000521	EFO_0000305	breast carcinoma	breast carcinoma	EFO_0000305	Cancer	Neoplasms
PGS000522	EFO_0000305	breast carcinoma	breast carcinoma	EFO_0000305	Cancer	Neoplasms
PGS000523	EFO_0000305	breast carcinoma	breast carcinoma	EFO_0000305	Cancer	Neoplasms
PGS000524	EFO_0000305	breast carcinoma	breast carcinoma	EFO_0000305	Cancer	Neoplasms
PGS000525	EFO_0000305	breast carcinoma	breast carcinoma	EFO_0000305	Cancer	Neoplasms
PGS000526	EFO_0000305	breast carcinoma	breast carcinoma	EFO_0000305	Cancer	Neoplasms
PGS000527	EFO_0000305	breast carcinoma	breast carcinoma	EFO_0000305	Cancer	Neoplasms
PGS000528	EFO_0000305	breast carcinoma	breast carcinoma	EFO_0000305	Cancer	Neoplasms
PGS000529	EFO_0000305	breast carcinoma	breast carcinoma	EFO_0000305	Cancer	Neoplasms
PGS000530	EFO_0000305	breast carcinoma	breast carcinoma	EFO_0000305	Cancer	Neoplasms
PGS000531	EFO_0000305	breast carcinoma	breast carcinoma	EFO_0000305	Cancer	Neoplasms
PGS000532	EFO_0000305	breast carcinoma	breast carcinoma	EFO_0000305	Cancer	Neoplasms
PGS000533	EFO_0000305	breast carcinoma	breast carcinoma	EFO_0000305	Cancer	Neoplasms
PGS000534	EFO_0000305	breast carcinoma	breast carcinoma	EFO_0000305	Cancer	Neoplasms
PGS000535	EFO_0000305	breast carcinoma	breast carcinoma	EFO_0000305	Cancer	Neoplasms
PGS000536	EFO_0000305	breast carcinoma	breast carcinoma	EFO_0000305	Cancer	Neoplasms
PGS000537	EFO_0000305	breast carcinoma	breast carcinoma	EFO_0000305	Cancer	Neoplasms
PGS000538	EFO_0000305	breast carcinoma	breast carcinoma	EFO_0000305	Cancer	Neoplasms
PGS000539	EFO_0000305	breast carcinoma	breast carcinoma	EFO_0000305	Cancer	Neoplasms
PGS000540	EFO_0000305	breast carcinoma	breast carcinoma	EFO_0000305	Cancer	Neoplasms
PGS000773	EFO_0000305	breast carcinoma	breast carcinoma	EFO_0000305	Cancer	Neoplasms
PGS000783	EFO_0000305	breast carcinoma	breast carcinoma	EFO_0000305	Cancer	Neoplasms
PGS000873	EFO_0000305	breast carcinoma	breast carcinoma	EFO_0000305	Cancer	Neoplasms
PGS001336	EFO_0000305	breast carcinoma	breast carcinoma	EFO_0000305	Cancer	Neoplasms
PGS001778	EFO_0000305	breast carcinoma	breast carcinoma	EFO_0000305	Cancer	Neoplasms
PGS001804	EFO_0000305	breast carcinoma	breast carcinoma	EFO_0000305	Cancer	Neoplasms
PGS002015	EFO_0000305	breast carcinoma	breast carcinoma	EFO_0000305	Cancer	Neoplasms
PGS002242	EFO_0000305	breast carcinoma	breast carcinoma	EFO_0000305	Cancer	Neoplasms
PGS002294	EFO_0000305	breast carcinoma	breast carcinoma	EFO_0000305	Cancer	Neoplasms
PGS003380	EFO_0000305	breast carcinoma	breast carcinoma	EFO_0000305	Cancer	Neoplasms
PGS003398	EFO_0000305	breast carcinoma	breast carcinoma	EFO_0000305	Cancer	Neoplasms
PGS003399	EFO_0000305	breast carcinoma	breast carcinoma	EFO_0000305	Cancer	Neoplasms
PGS000002	EFO_1000649	estrogen-receptor positive breast cancer	estrogen-receptor positive breast cancer	EFO_1000649	Cancer	Neoplasms
PGS000005	EFO_1000649	estrogen-receptor positive breast cancer	estrogen-receptor positive breast cancer	EFO_1000649	Cancer	Neoplasms

PGS000008	EFO_1000649	estrogen-receptor positive breast cancer	estrogen-receptor positive breast cancer	EFO_1000649	Cancer	Neoplasms
PGS000046	EFO_1000649	estrogen-receptor positive breast cancer	estrogen-receptor positive breast cancer	EFO_1000649	Cancer	Neoplasms
PGS000347	EFO_1000649	estrogen-receptor positive breast cancer	estrogen-receptor positive breast cancer	EFO_1000649	Cancer	Neoplasms
PGS000774	EFO_1000649	estrogen-receptor positive breast cancer	estrogen-receptor positive breast cancer	EFO_1000649	Cancer	Neoplasms
PGS000003	EFO_1000650	estrogen-receptor negative breast cancer	estrogen-receptor negative breast cancer	EFO_1000650	Cancer	Neoplasms
PGS000006	EFO_1000650	estrogen-receptor negative breast cancer	estrogen-receptor negative breast cancer	EFO_1000650	Cancer	Neoplasms
PGS000009	EFO_1000650	estrogen-receptor negative breast cancer	estrogen-receptor negative breast cancer	EFO_1000650	Cancer	Neoplasms
PGS000047	EFO_1000650	estrogen-receptor negative breast cancer	estrogen-receptor negative breast cancer	EFO_1000650	Cancer	Neoplasms
PGS000346	EFO_1000650	estrogen-receptor negative breast cancer	estrogen-receptor negative breast cancer	EFO_1000650	Cancer	Neoplasms
PGS000775	EFO_1000650	estrogen-receptor negative breast cancer	estrogen-receptor negative breast cancer	EFO_1000650	Cancer	Neoplasms
PGS000011	EFO_0001645	coronary artery disease	coronary artery disease	EFO_0001645	Cardiovascular disease	Cardiovascular
PGS000012	EFO_0001645	coronary artery disease	coronary artery disease	EFO_0001645	Cardiovascular disease	Cardiovascular
PGS000013	EFO_0001645	coronary artery disease	coronary artery disease	EFO_0001645	Cardiovascular disease	Cardiovascular
PGS000018	EFO_0001645	coronary artery disease	coronary artery disease	EFO_0001645	Cardiovascular disease	Cardiovascular
PGS000019	EFO_0001645	coronary artery disease	coronary artery disease	EFO_0001645	Cardiovascular disease	Cardiovascular
PGS000058	EFO_0001645	coronary artery disease	coronary artery disease	EFO_0001645	Cardiovascular disease	Cardiovascular
PGS000059	EFO_0001645	coronary artery disease	coronary artery disease	EFO_0001645	Cardiovascular disease	Cardiovascular
PGS000116	EFO_0001645	coronary artery disease	coronary artery disease	EFO_0001645	Cardiovascular disease	Cardiovascular
PGS000200	EFO_0001645	coronary artery disease	coronary artery disease	EFO_0001645	Cardiovascular disease	Cardiovascular
PGS000296	EFO_0001645	coronary artery disease	coronary artery disease	EFO_0001645	Cardiovascular disease	Cardiovascular
PGS000329	EFO_0001645	coronary artery disease	coronary artery disease	EFO_0001645	Cardiovascular disease	Cardiovascular
PGS000349	EFO_0001645	coronary artery disease	coronary artery disease	EFO_0001645	Cardiovascular disease	Cardiovascular
PGS000746	EFO_0001645	coronary artery disease	coronary artery disease	EFO_0001645	Cardiovascular disease	Cardiovascular
PGS000747	EFO_0001645	coronary artery disease	coronary artery disease	EFO_0001645	Cardiovascular disease	Cardiovascular
PGS000748	EFO_0001645	coronary artery disease	coronary artery disease	EFO_0001645	Cardiovascular disease	Cardiovascular
PGS000749	EFO_0001645	coronary artery disease	coronary artery disease	EFO_0001645	Cardiovascular disease	Cardiovascular
PGS000798	EFO_0001645	coronary artery disease	coronary artery disease	EFO_0001645	Cardiovascular disease	Cardiovascular
PGS000818	EFO_0001645	coronary artery disease	coronary artery disease	EFO_0001645	Cardiovascular disease	Cardiovascular
PGS000899	EFO_0001645	coronary artery disease	coronary artery disease	EFO_0001645	Cardiovascular disease	Cardiovascular
PGS001355	EFO_0001645	coronary artery disease	coronary artery disease	EFO_0001645	Cardiovascular disease	Cardiovascular
PGS001780	EFO_0001645	coronary artery disease	coronary artery disease	EFO_0001645	Cardiovascular disease	Cardiovascular
PGS002244	EFO_0001645	coronary artery disease	coronary artery disease	EFO_0001645	Cardiovascular disease	Cardiovascular
PGS002775	EFO_0001645	coronary artery disease	coronary artery disease	EFO_0001645	Cardiovascular disease	Cardiovascular
PGS002776	EFO_0001645	coronary artery disease	coronary artery disease	EFO_0001645	Cardiovascular disease	Cardiovascular
PGS002777	EFO_0001645	coronary artery disease	coronary artery disease	EFO_0001645	Cardiovascular disease	Cardiovascular
PGS002778	EFO_0001645	coronary artery disease	coronary artery disease	EFO_0001645	Cardiovascular disease	Cardiovascular
PGS003355	EFO_0001645	coronary artery disease	coronary artery disease	EFO_0001645	Cardiovascular disease	Cardiovascular
PGS003356	EFO_0001645	coronary artery disease	coronary artery disease	EFO_0001645	Cardiovascular disease	Cardiovascular
PGS000014	MONDO_0005148	type 2 diabetes mellitus	type 2 diabetes mellitus	MONDO_0005148	Digestive system disorder	Gastrointestinal
PGS000014	MONDO_0005148	type 2 diabetes mellitus	type 2 diabetes mellitus	MONDO_0005148	Metabolic disorder	Metabolic
PGS000020	MONDO_0005148	type 2 diabetes mellitus	type 2 diabetes mellitus	MONDO_0005148	Digestive system disorder	Gastrointestinal
PGS000020	MONDO_0005148	type 2 diabetes mellitus	type 2 diabetes mellitus	MONDO_0005148	Metabolic disorder	Metabolic
PGS000036	MONDO_0005148	type 2 diabetes mellitus	type 2 diabetes mellitus	MONDO_0005148	Digestive system disorder	Gastrointestinal
PGS000036	MONDO_0005148	type 2 diabetes mellitus	type 2 diabetes mellitus	MONDO_0005148	Metabolic disorder	Metabolic

PGS000125	MONDO_0005148	type 2 diabetes mellitus	type 2 diabetes mellitus	MONDO_0005148	Digestive system disorder	Gastrointestinal
PGS000125	MONDO_0005148	type 2 diabetes mellitus	type 2 diabetes mellitus	MONDO_0005148	Metabolic disorder	Metabolic
PGS000330	MONDO_0005148	type 2 diabetes mellitus	type 2 diabetes mellitus	MONDO_0005148	Digestive system disorder	Gastrointestinal
PGS000330	MONDO_0005148	type 2 diabetes mellitus	type 2 diabetes mellitus	MONDO_0005148	Metabolic disorder	Metabolic
PGS000712	MONDO_0005148	type 2 diabetes mellitus	type 2 diabetes mellitus	MONDO_0005148	Digestive system disorder	Gastrointestinal
PGS000712	MONDO_0005148	type 2 diabetes mellitus	type 2 diabetes mellitus	MONDO_0005148	Metabolic disorder	Metabolic
PGS000713	MONDO_0005148	type 2 diabetes mellitus	type 2 diabetes mellitus	MONDO_0005148	Digestive system disorder	Gastrointestinal
PGS000713	MONDO_0005148	type 2 diabetes mellitus	type 2 diabetes mellitus	MONDO_0005148	Metabolic disorder	Metabolic
PGS000729	MONDO_0005148	type 2 diabetes mellitus	type 2 diabetes mellitus	MONDO_0005148	Digestive system disorder	Gastrointestinal
PGS000729	MONDO_0005148	type 2 diabetes mellitus	type 2 diabetes mellitus	MONDO_0005148	Metabolic disorder	Metabolic
PGS000804	MONDO_0005148	type 2 diabetes mellitus	type 2 diabetes mellitus	MONDO_0005148	Digestive system disorder	Gastrointestinal
PGS000804	MONDO_0005148	type 2 diabetes mellitus	type 2 diabetes mellitus	MONDO_0005148	Metabolic disorder	Metabolic
PGS000805	MONDO_0005148	type 2 diabetes mellitus	type 2 diabetes mellitus	MONDO_0005148	Digestive system disorder	Gastrointestinal
PGS000805	MONDO_0005148	type 2 diabetes mellitus	type 2 diabetes mellitus	MONDO_0005148	Metabolic disorder	Metabolic
PGS000807	MONDO_0005148	type 2 diabetes mellitus	type 2 diabetes mellitus	MONDO_0005148	Digestive system disorder	Gastrointestinal
PGS000807	MONDO_0005148	type 2 diabetes mellitus	type 2 diabetes mellitus	MONDO_0005148	Metabolic disorder	Metabolic
PGS000832	MONDO_0005148	type 2 diabetes mellitus	type 2 diabetes mellitus	MONDO_0005148	Digestive system disorder	Gastrointestinal
PGS000832	MONDO_0005148	type 2 diabetes mellitus	type 2 diabetes mellitus	MONDO_0005148	Metabolic disorder	Metabolic
PGS000849	MONDO_0005148	type 2 diabetes mellitus	type 2 diabetes mellitus	MONDO_0005148	Digestive system disorder	Gastrointestinal
PGS000849	MONDO_0005148	type 2 diabetes mellitus	type 2 diabetes mellitus	MONDO_0005148	Metabolic disorder	Metabolic
PGS000856	MONDO_0005148	type 2 diabetes mellitus	type 2 diabetes mellitus	MONDO_0005148	Digestive system disorder	Gastrointestinal
PGS000856	MONDO_0005148	type 2 diabetes mellitus	type 2 diabetes mellitus	MONDO_0005148	Metabolic disorder	Metabolic
PGS000857	MONDO_0005148	type 2 diabetes mellitus	type 2 diabetes mellitus	MONDO_0005148	Digestive system disorder	Gastrointestinal
PGS000857	MONDO_0005148	type 2 diabetes mellitus	type 2 diabetes mellitus	MONDO_0005148	Metabolic disorder	Metabolic
PGS000868	MONDO_0005148	type 2 diabetes mellitus	type 2 diabetes mellitus	MONDO_0005148	Digestive system disorder	Gastrointestinal

PGS000868	MONDO_0005148	type 2 diabetes mellitus	type 2 diabetes mellitus	MONDO_0005148	Metabolic disorder	Metabolic
PGS001294	MONDO_0005148	type 2 diabetes mellitus	type 2 diabetes mellitus	MONDO_0005148	Digestive system disorder	Gastrointestinal
PGS001294	MONDO_0005148	type 2 diabetes mellitus	type 2 diabetes mellitus	MONDO_0005148	Metabolic disorder	Metabolic
PGS001295	MONDO_0005148	type 2 diabetes mellitus	type 2 diabetes mellitus	MONDO_0005148	Digestive system disorder	Gastrointestinal
PGS001295	MONDO_0005148	type 2 diabetes mellitus	type 2 diabetes mellitus	MONDO_0005148	Metabolic disorder	Metabolic
PGS001357	MONDO_0005148	type 2 diabetes mellitus	type 2 diabetes mellitus	MONDO_0005148	Digestive system disorder	Gastrointestinal
PGS001357	MONDO_0005148	type 2 diabetes mellitus	type 2 diabetes mellitus	MONDO_0005148	Metabolic disorder	Metabolic
PGS001781	MONDO_0005148	type 2 diabetes mellitus	type 2 diabetes mellitus	MONDO_0005148	Digestive system disorder	Gastrointestinal
PGS001781	MONDO_0005148	type 2 diabetes mellitus	type 2 diabetes mellitus	MONDO_0005148	Metabolic disorder	Metabolic
PGS001818	MONDO_0005148	type 2 diabetes mellitus	type 2 diabetes mellitus	MONDO_0005148	Digestive system disorder	Gastrointestinal
PGS001818	MONDO_0005148	type 2 diabetes mellitus	type 2 diabetes mellitus	MONDO_0005148	Metabolic disorder	Metabolic
PGS002026	MONDO_0005148	type 2 diabetes mellitus	type 2 diabetes mellitus	MONDO_0005148	Digestive system disorder	Gastrointestinal
PGS002026	MONDO_0005148	type 2 diabetes mellitus	type 2 diabetes mellitus	MONDO_0005148	Metabolic disorder	Metabolic
PGS002243	MONDO_0005148	type 2 diabetes mellitus	type 2 diabetes mellitus	MONDO_0005148	Digestive system disorder	Gastrointestinal
PGS002243	MONDO_0005148	type 2 diabetes mellitus	type 2 diabetes mellitus	MONDO_0005148	Metabolic disorder	Metabolic
PGS002308	MONDO_0005148	type 2 diabetes mellitus	type 2 diabetes mellitus	MONDO_0005148	Digestive system disorder	Gastrointestinal
PGS002308	MONDO_0005148	type 2 diabetes mellitus	type 2 diabetes mellitus	MONDO_0005148	Metabolic disorder	Metabolic
PGS002354	MONDO_0005148	type 2 diabetes mellitus	type 2 diabetes mellitus	MONDO_0005148	Digestive system disorder	Gastrointestinal
PGS002354	MONDO_0005148	type 2 diabetes mellitus	type 2 diabetes mellitus	MONDO_0005148	Metabolic disorder	Metabolic
PGS002379	MONDO_0005148	type 2 diabetes mellitus	type 2 diabetes mellitus	MONDO_0005148	Digestive system disorder	Gastrointestinal
PGS002379	MONDO_0005148	type 2 diabetes mellitus	type 2 diabetes mellitus	MONDO_0005148	Metabolic disorder	Metabolic
PGS002426	MONDO_0005148	type 2 diabetes mellitus	type 2 diabetes mellitus	MONDO_0005148	Digestive system disorder	Gastrointestinal
PGS002426	MONDO_0005148	type 2 diabetes mellitus	type 2 diabetes mellitus	MONDO_0005148	Metabolic disorder	Metabolic
PGS002475	MONDO_0005148	type 2 diabetes mellitus	type 2 diabetes mellitus	MONDO_0005148	Digestive system disorder	Gastrointestinal
PGS002475	MONDO_0005148	type 2 diabetes mellitus	type 2 diabetes mellitus	MONDO_0005148	Metabolic disorder	Metabolic

PGS002524	MONDO_0005148	type 2 diabetes mellitus	type 2 diabetes mellitus	MONDO_0005148	Digestive system disorder	Gastrointestinal
PGS002524	MONDO_0005148	type 2 diabetes mellitus	type 2 diabetes mellitus	MONDO_0005148	Metabolic disorder	Metabolic
PGS002573	MONDO_0005148	type 2 diabetes mellitus	type 2 diabetes mellitus	MONDO_0005148	Digestive system disorder	Gastrointestinal
PGS002573	MONDO_0005148	type 2 diabetes mellitus	type 2 diabetes mellitus	MONDO_0005148	Metabolic disorder	Metabolic
PGS002622	MONDO_0005148	type 2 diabetes mellitus	type 2 diabetes mellitus	MONDO_0005148	Digestive system disorder	Gastrointestinal
PGS002622	MONDO_0005148	type 2 diabetes mellitus	type 2 diabetes mellitus	MONDO_0005148	Metabolic disorder	Metabolic
PGS002733	MONDO_0005148	type 2 diabetes mellitus	type 2 diabetes mellitus	MONDO_0005148	Digestive system disorder	Gastrointestinal
PGS002733	MONDO_0005148	type 2 diabetes mellitus	type 2 diabetes mellitus	MONDO_0005148	Metabolic disorder	Metabolic
PGS002771	MONDO_0005148	type 2 diabetes mellitus	type 2 diabetes mellitus	MONDO_0005148	Digestive system disorder	Gastrointestinal
PGS002771	MONDO_0005148	type 2 diabetes mellitus	type 2 diabetes mellitus	MONDO_0005148	Metabolic disorder	Metabolic
PGS002779	MONDO_0005148	type 2 diabetes mellitus	type 2 diabetes mellitus	MONDO_0005148	Digestive system disorder	Gastrointestinal
PGS002779	MONDO_0005148	type 2 diabetes mellitus	type 2 diabetes mellitus	MONDO_0005148	Metabolic disorder	Metabolic
PGS002780	MONDO_0005148	type 2 diabetes mellitus	type 2 diabetes mellitus	MONDO_0005148	Digestive system disorder	Gastrointestinal
PGS002780	MONDO_0005148	type 2 diabetes mellitus	type 2 diabetes mellitus	MONDO_0005148	Metabolic disorder	Metabolic
PGS003089	MONDO_0005148	type 2 diabetes mellitus	type 2 diabetes mellitus	MONDO_0005148	Digestive system disorder	Gastrointestinal
PGS003089	MONDO_0005148	type 2 diabetes mellitus	type 2 diabetes mellitus	MONDO_0005148	Metabolic disorder	Metabolic
PGS003090	MONDO_0005148	type 2 diabetes mellitus	type 2 diabetes mellitus	MONDO_0005148	Digestive system disorder	Gastrointestinal
PGS003090	MONDO_0005148	type 2 diabetes mellitus	type 2 diabetes mellitus	MONDO_0005148	Metabolic disorder	Metabolic
PGS003091	MONDO_0005148	type 2 diabetes mellitus	type 2 diabetes mellitus	MONDO_0005148	Digestive system disorder	Gastrointestinal
PGS003091	MONDO_0005148	type 2 diabetes mellitus	type 2 diabetes mellitus	MONDO_0005148	Metabolic disorder	Metabolic
PGS003092	MONDO_0005148	type 2 diabetes mellitus	type 2 diabetes mellitus	MONDO_0005148	Digestive system disorder	Gastrointestinal
PGS003092	MONDO_0005148	type 2 diabetes mellitus	type 2 diabetes mellitus	MONDO_0005148	Metabolic disorder	Metabolic
PGS003093	MONDO_0005148	type 2 diabetes mellitus	type 2 diabetes mellitus	MONDO_0005148	Digestive system disorder	Gastrointestinal
PGS003093	MONDO_0005148	type 2 diabetes mellitus	type 2 diabetes mellitus	MONDO_0005148	Metabolic disorder	Metabolic
PGS003094	MONDO_0005148	type 2 diabetes mellitus	type 2 diabetes mellitus	MONDO_0005148	Digestive system disorder	Gastrointestinal

PGS003094	MONDO_0005148	type 2 diabetes mellitus	type 2 diabetes mellitus	MONDO_0005148	Metabolic disorder	Metabolic
PGS003095	MONDO_0005148	type 2 diabetes mellitus	type 2 diabetes mellitus	MONDO_0005148	Digestive system disorder	Gastrointestinal
PGS003095	MONDO_0005148	type 2 diabetes mellitus	type 2 diabetes mellitus	MONDO_0005148	Metabolic disorder	Metabolic
PGS003096	MONDO_0005148	type 2 diabetes mellitus	type 2 diabetes mellitus	MONDO_0005148	Digestive system disorder	Gastrointestinal
PGS003096	MONDO_0005148	type 2 diabetes mellitus	type 2 diabetes mellitus	MONDO_0005148	Metabolic disorder	Metabolic
PGS003097	MONDO_0005148	type 2 diabetes mellitus	type 2 diabetes mellitus	MONDO_0005148	Digestive system disorder	Gastrointestinal
PGS003097	MONDO_0005148	type 2 diabetes mellitus	type 2 diabetes mellitus	MONDO_0005148	Metabolic disorder	Metabolic
PGS003098	MONDO_0005148	type 2 diabetes mellitus	type 2 diabetes mellitus	MONDO_0005148	Digestive system disorder	Gastrointestinal
PGS003098	MONDO_0005148	type 2 diabetes mellitus	type 2 diabetes mellitus	MONDO_0005148	Metabolic disorder	Metabolic
PGS003099	MONDO_0005148	type 2 diabetes mellitus	type 2 diabetes mellitus	MONDO_0005148	Digestive system disorder	Gastrointestinal
PGS003099	MONDO_0005148	type 2 diabetes mellitus	type 2 diabetes mellitus	MONDO_0005148	Metabolic disorder	Metabolic
PGS003100	MONDO_0005148	type 2 diabetes mellitus	type 2 diabetes mellitus	MONDO_0005148	Digestive system disorder	Gastrointestinal
PGS003100	MONDO_0005148	type 2 diabetes mellitus	type 2 diabetes mellitus	MONDO_0005148	Metabolic disorder	Metabolic
PGS003101	MONDO_0005148	type 2 diabetes mellitus	type 2 diabetes mellitus	MONDO_0005148	Digestive system disorder	Gastrointestinal
PGS003101	MONDO_0005148	type 2 diabetes mellitus	type 2 diabetes mellitus	MONDO_0005148	Metabolic disorder	Metabolic
PGS003102	MONDO_0005148	type 2 diabetes mellitus	type 2 diabetes mellitus	MONDO_0005148	Digestive system disorder	Gastrointestinal
PGS003102	MONDO_0005148	type 2 diabetes mellitus	type 2 diabetes mellitus	MONDO_0005148	Metabolic disorder	Metabolic
PGS003103	MONDO_0005148	type 2 diabetes mellitus	type 2 diabetes mellitus	MONDO_0005148	Digestive system disorder	Gastrointestinal
PGS003103	MONDO_0005148	type 2 diabetes mellitus	type 2 diabetes mellitus	MONDO_0005148	Metabolic disorder	Metabolic
PGS003104	MONDO_0005148	type 2 diabetes mellitus	type 2 diabetes mellitus	MONDO_0005148	Digestive system disorder	Gastrointestinal
PGS003104	MONDO_0005148	type 2 diabetes mellitus	type 2 diabetes mellitus	MONDO_0005148	Metabolic disorder	Metabolic
PGS003105	MONDO_0005148	type 2 diabetes mellitus	type 2 diabetes mellitus	MONDO_0005148	Digestive system disorder	Gastrointestinal
PGS003105	MONDO_0005148	type 2 diabetes mellitus	type 2 diabetes mellitus	MONDO_0005148	Metabolic disorder	Metabolic
PGS003106	MONDO_0005148	type 2 diabetes mellitus	type 2 diabetes mellitus	MONDO_0005148	Digestive system disorder	Gastrointestinal
PGS003106	MONDO_0005148	type 2 diabetes mellitus	type 2 diabetes mellitus	MONDO_0005148	Metabolic disorder	Metabolic

PGS003107	MONDO_0005148	type 2 diabetes mellitus	type 2 diabetes mellitus	MONDO_0005148	Digestive system disorder	Gastrointestinal
PGS003107	MONDO_0005148	type 2 diabetes mellitus	type 2 diabetes mellitus	MONDO_0005148	Metabolic disorder	Metabolic
PGS003108	MONDO_0005148	type 2 diabetes mellitus	type 2 diabetes mellitus	MONDO_0005148	Digestive system disorder	Gastrointestinal
PGS003108	MONDO_0005148	type 2 diabetes mellitus	type 2 diabetes mellitus	MONDO_0005148	Metabolic disorder	Metabolic
PGS003109	MONDO_0005148	type 2 diabetes mellitus	type 2 diabetes mellitus	MONDO_0005148	Digestive system disorder	Gastrointestinal
PGS003109	MONDO_0005148	type 2 diabetes mellitus	type 2 diabetes mellitus	MONDO_0005148	Metabolic disorder	Metabolic
PGS003110	MONDO_0005148	type 2 diabetes mellitus	type 2 diabetes mellitus	MONDO_0005148	Digestive system disorder	Gastrointestinal
PGS003110	MONDO_0005148	type 2 diabetes mellitus	type 2 diabetes mellitus	MONDO_0005148	Metabolic disorder	Metabolic
PGS003111	MONDO_0005148	type 2 diabetes mellitus	type 2 diabetes mellitus	MONDO_0005148	Digestive system disorder	Gastrointestinal
PGS003111	MONDO_0005148	type 2 diabetes mellitus	type 2 diabetes mellitus	MONDO_0005148	Metabolic disorder	Metabolic
PGS003112	MONDO_0005148	type 2 diabetes mellitus	type 2 diabetes mellitus	MONDO_0005148	Digestive system disorder	Gastrointestinal
PGS003112	MONDO_0005148	type 2 diabetes mellitus	type 2 diabetes mellitus	MONDO_0005148	Metabolic disorder	Metabolic
PGS003113	MONDO_0005148	type 2 diabetes mellitus	type 2 diabetes mellitus	MONDO_0005148	Digestive system disorder	Gastrointestinal
PGS003113	MONDO_0005148	type 2 diabetes mellitus	type 2 diabetes mellitus	MONDO_0005148	Metabolic disorder	Metabolic
PGS003114	MONDO_0005148	type 2 diabetes mellitus	type 2 diabetes mellitus	MONDO_0005148	Digestive system disorder	Gastrointestinal
PGS003114	MONDO_0005148	type 2 diabetes mellitus	type 2 diabetes mellitus	MONDO_0005148	Metabolic disorder	Metabolic
PGS003115	MONDO_0005148	type 2 diabetes mellitus	type 2 diabetes mellitus	MONDO_0005148	Digestive system disorder	Gastrointestinal
PGS003115	MONDO_0005148	type 2 diabetes mellitus	type 2 diabetes mellitus	MONDO_0005148	Metabolic disorder	Metabolic
PGS003116	MONDO_0005148	type 2 diabetes mellitus	type 2 diabetes mellitus	MONDO_0005148	Digestive system disorder	Gastrointestinal
PGS003116	MONDO_0005148	type 2 diabetes mellitus	type 2 diabetes mellitus	MONDO_0005148	Metabolic disorder	Metabolic
PGS003117	MONDO_0005148	type 2 diabetes mellitus	type 2 diabetes mellitus	MONDO_0005148	Digestive system disorder	Gastrointestinal
PGS003117	MONDO_0005148	type 2 diabetes mellitus	type 2 diabetes mellitus	MONDO_0005148	Metabolic disorder	Metabolic
PGS003118	MONDO_0005148	type 2 diabetes mellitus	type 2 diabetes mellitus	MONDO_0005148	Digestive system disorder	Gastrointestinal
PGS003118	MONDO_0005148	type 2 diabetes mellitus	type 2 diabetes mellitus	MONDO_0005148	Metabolic disorder	Metabolic
PGS003119	MONDO_0005148	type 2 diabetes mellitus	type 2 diabetes mellitus	MONDO_0005148	Digestive system disorder	Gastrointestinal

PGS003119	MONDO_0005148	type 2 diabetes mellitus	type 2 diabetes mellitus	MONDO_0005148	Metabolic disorder	Metabolic
PGS003120	MONDO_0005148	type 2 diabetes mellitus	type 2 diabetes mellitus	MONDO_0005148	Digestive system disorder	Gastrointestinal
PGS003120	MONDO_0005148	type 2 diabetes mellitus	type 2 diabetes mellitus	MONDO_0005148	Metabolic disorder	Metabolic
PGS003121	MONDO_0005148	type 2 diabetes mellitus	type 2 diabetes mellitus	MONDO_0005148	Digestive system disorder	Gastrointestinal
PGS003121	MONDO_0005148	type 2 diabetes mellitus	type 2 diabetes mellitus	MONDO_0005148	Metabolic disorder	Metabolic
PGS003122	MONDO_0005148	type 2 diabetes mellitus	type 2 diabetes mellitus	MONDO_0005148	Digestive system disorder	Gastrointestinal
PGS003122	MONDO_0005148	type 2 diabetes mellitus	type 2 diabetes mellitus	MONDO_0005148	Metabolic disorder	Metabolic
PGS003123	MONDO_0005148	type 2 diabetes mellitus	type 2 diabetes mellitus	MONDO_0005148	Digestive system disorder	Gastrointestinal
PGS003123	MONDO_0005148	type 2 diabetes mellitus	type 2 diabetes mellitus	MONDO_0005148	Metabolic disorder	Metabolic
PGS003402	MONDO_0005148	type 2 diabetes mellitus	type 2 diabetes mellitus	MONDO_0005148	Digestive system disorder	Gastrointestinal
PGS003402	MONDO_0005148	type 2 diabetes mellitus	type 2 diabetes mellitus	MONDO_0005148	Metabolic disorder	Metabolic
PGS000016	EFO_0000275	atrial fibrillation	atrial fibrillation	EFO_0000275	Cardiovascular disease	Cardiovascular
PGS000035	EFO_0000275	atrial fibrillation	atrial fibrillation	EFO_0000275	Cardiovascular disease	Cardiovascular
PGS000331	EFO_0000275	atrial fibrillation	atrial fibrillation	EFO_0000275	Cardiovascular disease	Cardiovascular
PGS000727	EFO_0000275	atrial fibrillation	atrial fibrillation	EFO_0000275	Cardiovascular disease	Cardiovascular
PGS001339	EFO_0000275	atrial fibrillation|atrial flutter	atrial fibrillation	EFO_0000275	Cardiovascular disease	Cardiovascular
PGS001340	EFO_0000275	atrial fibrillation	atrial fibrillation	EFO_0000275	Cardiovascular disease	Cardiovascular
PGS001356	EFO_0000275	atrial fibrillation	atrial fibrillation	EFO_0000275	Cardiovascular disease	Cardiovascular
PGS002756	EFO_0000275	atrial fibrillation	atrial fibrillation	EFO_0000275	Cardiovascular disease	Cardiovascular
PGS002773	EFO_0000275	atrial fibrillation	atrial fibrillation	EFO_0000275	Cardiovascular disease	Cardiovascular
PGS002774	EFO_0000275	atrial fibrillation	atrial fibrillation	EFO_0000275	Cardiovascular disease	Cardiovascular
PGS002814	EFO_0000275	atrial fibrillation	atrial fibrillation	EFO_0000275	Cardiovascular disease	Cardiovascular
PGS000017	EFO_0003767	inflammatory bowel disease	inflammatory bowel disease	EFO_0003767	Digestive system disorder	Gastrointestinal
PGS000017	EFO_0003767	inflammatory bowel disease	inflammatory bowel disease	EFO_0003767	Immune system disorder	Immunological
PGS001288	EFO_0003767	inflammatory bowel disease	inflammatory bowel disease	EFO_0003767	Digestive system disorder	Gastrointestinal
PGS001288	EFO_0003767	inflammatory bowel disease	inflammatory bowel disease	EFO_0003767	Immune system disorder	Immunological
PGS000021	MONDO_0005147	type 1 diabetes mellitus	type 1 diabetes mellitus	MONDO_0005147	Digestive system disorder	Gastrointestinal
PGS000021	MONDO_0005147	type 1 diabetes mellitus	type 1 diabetes mellitus	MONDO_0005147	Immune system disorder	Immunological
PGS000021	MONDO_0005147	type 1 diabetes mellitus	type 1 diabetes mellitus	MONDO_0005147	Metabolic disorder	Metabolic
PGS000023	MONDO_0005147	type 1 diabetes mellitus	type 1 diabetes mellitus	MONDO_0005147	Digestive system disorder	Gastrointestinal

PGS000023	MONDO_0005147	type 1 diabetes mellitus	type 1 diabetes mellitus	MONDO_0005147	Immune system disorder	Immunological
PGS000023	MONDO_0005147	type 1 diabetes mellitus	type 1 diabetes mellitus	MONDO_0005147	Metabolic disorder	Metabolic
PGS000869	MONDO_0005147	type 1 diabetes mellitus	type 1 diabetes mellitus	MONDO_0005147	Digestive system disorder	Gastrointestinal
PGS000869	MONDO_0005147	type 1 diabetes mellitus	type 1 diabetes mellitus	MONDO_0005147	Immune system disorder	Immunological
PGS000869	MONDO_0005147	type 1 diabetes mellitus	type 1 diabetes mellitus	MONDO_0005147	Metabolic disorder	Metabolic
PGS001296	MONDO_0005147	type 1 diabetes mellitus	type 1 diabetes mellitus	MONDO_0005147	Digestive system disorder	Gastrointestinal
PGS001296	MONDO_0005147	type 1 diabetes mellitus	type 1 diabetes mellitus	MONDO_0005147	Immune system disorder	Immunological
PGS001296	MONDO_0005147	type 1 diabetes mellitus	type 1 diabetes mellitus	MONDO_0005147	Metabolic disorder	Metabolic
PGS001297	MONDO_0005147	type 1 diabetes mellitus	type 1 diabetes mellitus	MONDO_0005147	Digestive system disorder	Gastrointestinal
PGS001297	MONDO_0005147	type 1 diabetes mellitus	type 1 diabetes mellitus	MONDO_0005147	Immune system disorder	Immunological
PGS001297	MONDO_0005147	type 1 diabetes mellitus	type 1 diabetes mellitus	MONDO_0005147	Metabolic disorder	Metabolic
PGS001817	MONDO_0005147	type 1 diabetes mellitus	type 1 diabetes mellitus	MONDO_0005147	Digestive system disorder	Gastrointestinal
PGS001817	MONDO_0005147	type 1 diabetes mellitus	type 1 diabetes mellitus	MONDO_0005147	Immune system disorder	Immunological
PGS001817	MONDO_0005147	type 1 diabetes mellitus	type 1 diabetes mellitus	MONDO_0005147	Metabolic disorder	Metabolic
PGS002025	MONDO_0005147	type 1 diabetes mellitus	type 1 diabetes mellitus	MONDO_0005147	Digestive system disorder	Gastrointestinal
PGS002025	MONDO_0005147	type 1 diabetes mellitus	type 1 diabetes mellitus	MONDO_0005147	Immune system disorder	Immunological
PGS002025	MONDO_0005147	type 1 diabetes mellitus	type 1 diabetes mellitus	MONDO_0005147	Metabolic disorder	Metabolic
PGS000025	MONDO_0004975	Alzheimer disease	Alzheimer disease	MONDO_0004975	Neurological disorder	Neurological
PGS000026	MONDO_0004975	Alzheimer disease	Alzheimer disease	MONDO_0004975	Neurological disorder	Neurological
PGS000779	MONDO_0004975	Alzheimer disease	Alzheimer disease	MONDO_0004975	Neurological disorder	Neurological
PGS000823	MONDO_0004975	Alzheimer disease	Alzheimer disease	MONDO_0004975	Neurological disorder	Neurological
PGS000898	MONDO_0004975	Alzheimer disease	Alzheimer disease	MONDO_0004975	Neurological disorder	Neurological
PGS001348	MONDO_0004975	Alzheimer disease	Alzheimer disease	MONDO_0004975	Neurological disorder	Neurological
PGS001349	MONDO_0004975	Alzheimer disease	Alzheimer disease	MONDO_0004975	Neurological disorder	Neurological
PGS001775	MONDO_0004975	Alzheimer disease	Alzheimer disease	MONDO_0004975	Neurological disorder	Neurological

PGS001828	MONDO_0004975	Alzheimer disease	Alzheimer disease	MONDO_0004975	Neurological disorder	Neurological
PGS002249	MONDO_0004975	Alzheimer disease	Alzheimer disease	MONDO_0004975	Neurological disorder	Neurological
PGS002280	MONDO_0004975	Alzheimer disease	Alzheimer disease	MONDO_0004975	Neurological disorder	Neurological
PGS002753	MONDO_0004975	Alzheimer disease	Alzheimer disease	MONDO_0004975	Neurological disorder	Neurological
PGS000027	EFO_0004340	body mass index	body mass index	EFO_0004340	Body measurement	Body measurement
PGS000034	EFO_0004340	body mass index	body mass index	EFO_0004340	Body measurement	Body measurement
PGS000298	EFO_0004340	body mass index	body mass index	EFO_0004340	Body measurement	Body measurement
PGS000320	EFO_0004340	body mass index	body mass index	EFO_0004340	Body measurement	Body measurement
PGS000717	EFO_0004340	body mass index	body mass index	EFO_0004340	Body measurement	Body measurement
PGS000770	EFO_0004340	body mass index	body mass index	EFO_0004340	Body measurement	Body measurement
PGS000829	EFO_0004340	body mass index|male	body mass index	EFO_0004340	Body measurement	Body measurement
PGS000830	EFO_0004340	body mass index|female	body mass index	EFO_0004340	Body measurement	Body measurement
PGS000841	EFO_0004340	body mass index	body mass index	EFO_0004340	Body measurement	Body measurement
PGS000921	EFO_0004340	body mass index	body mass index	EFO_0004340	Body measurement	Body measurement
PGS001228	EFO_0004340	body mass index	body mass index	EFO_0004340	Body measurement	Body measurement
PGS001943	EFO_0004340	body mass index	body mass index	EFO_0004340	Body measurement	Body measurement
PGS002161	EFO_0004340	body mass index	body mass index	EFO_0004340	Body measurement	Body measurement
PGS002251	EFO_0004340	body mass index	body mass index	EFO_0004340	Body measurement	Body measurement
PGS002313	EFO_0004340	body mass index	body mass index	EFO_0004340	Body measurement	Body measurement
PGS002360	EFO_0004340	body mass index	body mass index	EFO_0004340	Body measurement	Body measurement
PGS002385	EFO_0004340	body mass index	body mass index	EFO_0004340	Body measurement	Body measurement
PGS002434	EFO_0004340	body mass index	body mass index	EFO_0004340	Body measurement	Body measurement
PGS002483	EFO_0004340	body mass index	body mass index	EFO_0004340	Body measurement	Body measurement
PGS002532	EFO_0004340	body mass index	body mass index	EFO_0004340	Body measurement	Body measurement
PGS002581	EFO_0004340	body mass index	body mass index	EFO_0004340	Body measurement	Body measurement
PGS002630	EFO_0004340	body mass index	body mass index	EFO_0004340	Body measurement	Body measurement
PGS002751	EFO_0004340	body mass index	body mass index	EFO_0004340	Body measurement	Body measurement
PGS002840	EFO_0004340	body mass index	body mass index	EFO_0004340	Body measurement	Body measurement
PGS002841	EFO_0004340	body mass index	body mass index	EFO_0004340	Body measurement	Body measurement
PGS002842	EFO_0004340	body mass index	body mass index	EFO_0004340	Body measurement	Body measurement
PGS002843	EFO_0004340	body mass index	body mass index	EFO_0004340	Body measurement	Body measurement
PGS002844	EFO_0004340	body mass index	body mass index	EFO_0004340	Body measurement	Body measurement
PGS002845	EFO_0004340	body mass index	body mass index	EFO_0004340	Body measurement	Body measurement
PGS002846	EFO_0004340	body mass index	body mass index	EFO_0004340	Body measurement	Body measurement
PGS002847	EFO_0004340	body mass index	body mass index	EFO_0004340	Body measurement	Body measurement
PGS002848	EFO_0004340	body mass index	body mass index	EFO_0004340	Body measurement	Body measurement
PGS002849	EFO_0004340	body mass index	body mass index	EFO_0004340	Body measurement	Body measurement
PGS002850	EFO_0004340	body mass index	body mass index	EFO_0004340	Body measurement	Body measurement
PGS002851	EFO_0004340	body mass index	body mass index	EFO_0004340	Body measurement	Body measurement
PGS002852	EFO_0004340	body mass index	body mass index	EFO_0004340	Body measurement	Body measurement
PGS002853	EFO_0004340	body mass index	body mass index	EFO_0004340	Body measurement	Body measurement
PGS002854	EFO_0004340	body mass index	body mass index	EFO_0004340	Body measurement	Body measurement
PGS002855	EFO_0004340	body mass index	body mass index	EFO_0004340	Body measurement	Body measurement
PGS002856	EFO_0004340	body mass index	body mass index	EFO_0004340	Body measurement	Body measurement
PGS002857	EFO_0004340	body mass index	body mass index	EFO_0004340	Body measurement	Body measurement
PGS002858	EFO_0004340	body mass index	body mass index	EFO_0004340	Body measurement	Body measurement

PGS002859	EFO_0004340	body mass index	body mass index	EFO_0004340	Body measurement	Body measurement
PGS003124	EFO_0004340	body mass index	body mass index	EFO_0004340	Body measurement	Body measurement
PGS003125	EFO_0004340	body mass index	body mass index	EFO_0004340	Body measurement	Body measurement
PGS003126	EFO_0004340	body mass index	body mass index	EFO_0004340	Body measurement	Body measurement
PGS003127	EFO_0004340	body mass index	body mass index	EFO_0004340	Body measurement	Body measurement
PGS003128	EFO_0004340	body mass index	body mass index	EFO_0004340	Body measurement	Body measurement
PGS003129	EFO_0004340	body mass index	body mass index	EFO_0004340	Body measurement	Body measurement
PGS003130	EFO_0004340	body mass index	body mass index	EFO_0004340	Body measurement	Body measurement
PGS003131	EFO_0004340	body mass index	body mass index	EFO_0004340	Body measurement	Body measurement
PGS003132	EFO_0004340	body mass index	body mass index	EFO_0004340	Body measurement	Body measurement
PGS003133	EFO_0004340	body mass index	body mass index	EFO_0004340	Body measurement	Body measurement
PGS000030	EFO_0001663	prostate carcinoma	prostate carcinoma	EFO_0001663	Cancer	Neoplasms
PGS000044	EFO_0001663	prostate carcinoma	prostate carcinoma	EFO_0001663	Cancer	Neoplasms
PGS000049	EFO_0001663	prostate carcinoma	prostate carcinoma	EFO_0001663	Cancer	Neoplasms
PGS000067	EFO_0001663	prostate carcinoma	prostate carcinoma	EFO_0001663	Cancer	Neoplasms
PGS000084	EFO_0001663	prostate carcinoma	prostate carcinoma	EFO_0001663	Cancer	Neoplasms
PGS000160	EFO_0001663	prostate carcinoma	prostate carcinoma	EFO_0001663	Cancer	Neoplasms
PGS000333	EFO_0001663	prostate carcinoma	prostate carcinoma	EFO_0001663	Cancer	Neoplasms
PGS000348	EFO_0001663	prostate carcinoma	prostate carcinoma	EFO_0001663	Cancer	Neoplasms
PGS000565	EFO_0001663	prostate carcinoma	prostate carcinoma	EFO_0001663	Cancer	Neoplasms
PGS000566	EFO_0001663	prostate carcinoma	prostate carcinoma	EFO_0001663	Cancer	Neoplasms
PGS000567	EFO_0001663	prostate carcinoma	prostate carcinoma	EFO_0001663	Cancer	Neoplasms
PGS000568	EFO_0001663	prostate carcinoma	prostate carcinoma	EFO_0001663	Cancer	Neoplasms
PGS000569	EFO_0001663	prostate carcinoma	prostate carcinoma	EFO_0001663	Cancer	Neoplasms
PGS000570	EFO_0001663	prostate carcinoma	prostate carcinoma	EFO_0001663	Cancer	Neoplasms
PGS000571	EFO_0001663	prostate carcinoma	prostate carcinoma	EFO_0001663	Cancer	Neoplasms
PGS000572	EFO_0001663	prostate carcinoma	prostate carcinoma	EFO_0001663	Cancer	Neoplasms
PGS000573	EFO_0001663	prostate carcinoma	prostate carcinoma	EFO_0001663	Cancer	Neoplasms
PGS000574	EFO_0001663	prostate carcinoma	prostate carcinoma	EFO_0001663	Cancer	Neoplasms
PGS000575	EFO_0001663	prostate carcinoma	prostate carcinoma	EFO_0001663	Cancer	Neoplasms
PGS000576	EFO_0001663	prostate carcinoma	prostate carcinoma	EFO_0001663	Cancer	Neoplasms
PGS000577	EFO_0001663	prostate carcinoma	prostate carcinoma	EFO_0001663	Cancer	Neoplasms
PGS000578	EFO_0001663	prostate carcinoma	prostate carcinoma	EFO_0001663	Cancer	Neoplasms
PGS000579	EFO_0001663	prostate carcinoma	prostate carcinoma	EFO_0001663	Cancer	Neoplasms
PGS000580	EFO_0001663	prostate carcinoma	prostate carcinoma	EFO_0001663	Cancer	Neoplasms
PGS000581	EFO_0001663	prostate carcinoma	prostate carcinoma	EFO_0001663	Cancer	Neoplasms
PGS000582	EFO_0001663	prostate carcinoma	prostate carcinoma	EFO_0001663	Cancer	Neoplasms
PGS000583	EFO_0001663	prostate carcinoma	prostate carcinoma	EFO_0001663	Cancer	Neoplasms
PGS000584	EFO_0001663	prostate carcinoma	prostate carcinoma	EFO_0001663	Cancer	Neoplasms
PGS000585	EFO_0001663	prostate carcinoma	prostate carcinoma	EFO_0001663	Cancer	Neoplasms
PGS000586	EFO_0001663	prostate carcinoma	prostate carcinoma	EFO_0001663	Cancer	Neoplasms
PGS000587	EFO_0001663	prostate carcinoma	prostate carcinoma	EFO_0001663	Cancer	Neoplasms
PGS000588	EFO_0001663	prostate carcinoma	prostate carcinoma	EFO_0001663	Cancer	Neoplasms
PGS000589	EFO_0001663	prostate carcinoma	prostate carcinoma	EFO_0001663	Cancer	Neoplasms
PGS000590	EFO_0001663	prostate carcinoma	prostate carcinoma	EFO_0001663	Cancer	Neoplasms
PGS000591	EFO_0001663	prostate carcinoma	prostate carcinoma	EFO_0001663	Cancer	Neoplasms
PGS000592	EFO_0001663	prostate carcinoma	prostate carcinoma	EFO_0001663	Cancer	Neoplasms
PGS000662	EFO_0001663	prostate carcinoma	prostate carcinoma	EFO_0001663	Cancer	Neoplasms
PGS000714	EFO_0001663	prostate carcinoma	prostate carcinoma	EFO_0001663	Cancer	Neoplasms
PGS000719	EFO_0001663	prostate carcinoma	prostate carcinoma	EFO_0001663	Cancer	Neoplasms

PGS000733	EFO_0001663	prostate carcinoma	prostate carcinoma	EFO_0001663	Cancer	Neoplasms
PGS000741	EFO_0001663	prostate carcinoma	prostate carcinoma	EFO_0001663	Cancer	Neoplasms
PGS000742	EFO_0001663	prostate carcinoma	prostate carcinoma	EFO_0001663	Cancer	Neoplasms
PGS000751	EFO_0001663	prostate carcinoma	prostate carcinoma	EFO_0001663	Cancer	Neoplasms
PGS000795	EFO_0001663	prostate carcinoma	prostate carcinoma	EFO_0001663	Cancer	Neoplasms
PGS001291	EFO_0001663	prostate carcinoma	prostate carcinoma	EFO_0001663	Cancer	Neoplasms
PGS001805	EFO_0001663	prostate carcinoma	prostate carcinoma	EFO_0001663	Cancer	Neoplasms
PGS002016	EFO_0001663	prostate carcinoma	prostate carcinoma	EFO_0001663	Cancer	Neoplasms
PGS002240	EFO_0001663	prostate carcinoma	prostate carcinoma	EFO_0001663	Cancer	Neoplasms
PGS002241	EFO_0001663	prostate carcinoma	prostate carcinoma	EFO_0001663	Cancer	Neoplasms
PGS002791	EFO_0001663	prostate carcinoma	prostate carcinoma	EFO_0001663	Cancer	Neoplasms
PGS002792	EFO_0001663	prostate carcinoma	prostate carcinoma	EFO_0001663	Cancer	Neoplasms
PGS002796	EFO_0001663	prostate carcinoma	prostate carcinoma	EFO_0001663	Cancer	Neoplasms
PGS002798	EFO_0001663	prostate carcinoma	prostate carcinoma	EFO_0001663	Cancer	Neoplasms
PGS003331	EFO_0001663	prostate carcinoma	prostate carcinoma	EFO_0001663	Cancer	Neoplasms
PGS003415	EFO_0001663	prostate carcinoma	prostate carcinoma	EFO_0001663	Cancer	Neoplasms
PGS000037	MONDO_0004979	asthma	asthma	MONDO_0004979	Other disease	Respiratory
PGS001341	MONDO_0004979	asthma	asthma	MONDO_0004979	Other disease	Respiratory
PGS001343	MONDO_0004979	asthma	asthma	MONDO_0004979	Other disease	Respiratory
PGS001344	MONDO_0004979	asthma	asthma	MONDO_0004979	Other disease	Respiratory
PGS001345	MONDO_0004979	asthma	asthma	MONDO_0004979	Other disease	Respiratory
PGS001346	MONDO_0004979	asthma	asthma	MONDO_0004979	Other disease	Respiratory
PGS001370	MONDO_0004979	asthma	asthma	MONDO_0004979	Other disease	Respiratory
PGS001782	MONDO_0004979	asthma	asthma	MONDO_0004979	Other disease	Respiratory
PGS001787	MONDO_0004979	asthma	asthma	MONDO_0004979	Other disease	Respiratory
PGS001849	MONDO_0004979	asthma	asthma	MONDO_0004979	Other disease	Respiratory
PGS002061	MONDO_0004979	asthma	asthma	MONDO_0004979	Other disease	Respiratory
PGS002311	MONDO_0004979	asthma	asthma	MONDO_0004979	Other disease	Respiratory
PGS002358	MONDO_0004979	asthma	asthma	MONDO_0004979	Other disease	Respiratory
PGS002383	MONDO_0004979	asthma	asthma	MONDO_0004979	Other disease	Respiratory
PGS002432	MONDO_0004979	asthma	asthma	MONDO_0004979	Other disease	Respiratory
PGS002481	MONDO_0004979	asthma	asthma	MONDO_0004979	Other disease	Respiratory
PGS002530	MONDO_0004979	asthma	asthma	MONDO_0004979	Other disease	Respiratory

PGS002579	MONDO_0004979	asthma	asthma	MONDO_0004979	Other disease	Respiratory
PGS002628	MONDO_0004979	asthma	asthma	MONDO_0004979	Other disease	Respiratory
PGS002727	MONDO_0004979	asthma	asthma	MONDO_0004979	Other disease	Respiratory
PGS002754	MONDO_0004979	asthma	asthma	MONDO_0004979	Other disease	Respiratory
PGS000038	EFO_0000712	stroke	stroke	EFO_0000712	Cardiovascular disease	Cardiovascular
PGS000038	EFO_0000712	stroke	stroke	EFO_0000712	Neurological disorder	Neurological
PGS000039	EFO_0000712	stroke|Ischemic stroke	stroke	EFO_0000712	Cardiovascular disease	Cardiovascular
PGS000039	EFO_0000712	stroke|Ischemic stroke	stroke	EFO_0000712	Neurological disorder	Neurological
PGS001793	EFO_0000712	stroke	stroke	EFO_0000712	Cardiovascular disease	Cardiovascular
PGS001793	EFO_0000712	stroke	stroke	EFO_0000712	Neurological disorder	Neurological
PGS001798	EFO_0000712	stroke	stroke	EFO_0000712	Cardiovascular disease	Cardiovascular
PGS001798	EFO_0000712	stroke	stroke	EFO_0000712	Neurological disorder	Neurological
PGS002259	EFO_0000712	stroke	stroke	EFO_0000712	Cardiovascular disease	Cardiovascular
PGS002259	EFO_0000712	stroke	stroke	EFO_0000712	Neurological disorder	Neurological
PGS002770	EFO_0000712	stroke	stroke	EFO_0000712	Cardiovascular disease	Cardiovascular
PGS002770	EFO_0000712	stroke	stroke	EFO_0000712	Neurological disorder	Neurological
PGS000040	EFO_0001060	celiac disease	celiac disease	EFO_0001060	Digestive system disorder	Gastrointestinal
PGS000040	EFO_0001060	celiac disease	celiac disease	EFO_0001060	Immune system disorder	Immunological
PGS000041	EFO_0001060	celiac disease	celiac disease	EFO_0001060	Digestive system disorder	Gastrointestinal
PGS000041	EFO_0001060	celiac disease	celiac disease	EFO_0001060	Immune system disorder	Immunological
PGS000042	EFO_0001060	celiac disease	celiac disease	EFO_0001060	Digestive system disorder	Gastrointestinal
PGS000042	EFO_0001060	celiac disease	celiac disease	EFO_0001060	Immune system disorder	Immunological
PGS001300	EFO_0001060	celiac disease	celiac disease	EFO_0001060	Digestive system disorder	Gastrointestinal
PGS001300	EFO_0001060	celiac disease	celiac disease	EFO_0001060	Immune system disorder	Immunological
PGS001301	EFO_0001060	celiac disease	celiac disease	EFO_0001060	Digestive system disorder	Gastrointestinal
PGS001301	EFO_0001060	celiac disease	celiac disease	EFO_0001060	Immune system disorder	Immunological
PGS001856	EFO_0001060	celiac disease	celiac disease	EFO_0001060	Digestive system disorder	Gastrointestinal
PGS001856	EFO_0001060	celiac disease	celiac disease	EFO_0001060	Immune system disorder	Immunological
PGS001894	EFO_0001060	celiac disease	celiac disease	EFO_0001060	Digestive system disorder	Gastrointestinal
PGS001894	EFO_0001060	celiac disease	celiac disease	EFO_0001060	Immune system disorder	Immunological
PGS002067	EFO_0001060	celiac disease	celiac disease	EFO_0001060	Digestive system disorder	Gastrointestinal

PGS002067	EFO_0001060	celiac disease	celiac disease	EFO_0001060	Immune system disorder	Immunological
PGS002107	EFO_0001060	celiac disease	celiac disease	EFO_0001060	Digestive system disorder	Gastrointestinal
PGS002107	EFO_0001060	celiac disease	celiac disease	EFO_0001060	Immune system disorder	Immunological
PGS000043	EFO_0004286	venous thromboembolism	venous thromboembolism	EFO_0004286	Cardiovascular disease	Cardiovascular
PGS001796	EFO_0004286	venous thromboembolism	venous thromboembolism	EFO_0004286	Cardiovascular disease	Cardiovascular
PGS002772	EFO_0004286	venous thromboembolism	venous thromboembolism	EFO_0004286	Cardiovascular disease	Cardiovascular
PGS003332	EFO_0004286	venous thromboembolism	venous thromboembolism	EFO_0004286	Cardiovascular disease	Cardiovascular
PGS000048	EFO_0001075	ovarian carcinoma	ovarian carcinoma	EFO_0001075	Cancer	Neoplasms
PGS000068	EFO_0001075	ovarian carcinoma	ovarian carcinoma	EFO_0001075	Cancer	Neoplasms
PGS000082	EFO_0001075	ovarian carcinoma	ovarian carcinoma	EFO_0001075	Cancer	Neoplasms
PGS000351	EFO_0001075	ovarian carcinoma	ovarian carcinoma	EFO_0001075	Cancer	Neoplasms
PGS000793	EFO_0001075	ovarian carcinoma	ovarian carcinoma	EFO_0001075	Cancer	Neoplasms
PGS002250	EFO_0001075	ovarian carcinoma	ovarian carcinoma	EFO_0001075	Cancer	Neoplasms
PGS003394	EFO_0001075	ovarian carcinoma	ovarian carcinoma	EFO_0001075	Cancer	Neoplasms
PGS000056	MONDO_0005180	Parkinson disease	Parkinson disease	MONDO_0005180	Metabolic disorder	Metabolic
PGS000056	MONDO_0005180	Parkinson disease	Parkinson disease	MONDO_0005180	Neurological disorder	Neurological
PGS000123	MONDO_0005180	Parkinson disease	Parkinson disease	MONDO_0005180	Metabolic disorder	Metabolic
PGS000123	MONDO_0005180	Parkinson disease	Parkinson disease	MONDO_0005180	Neurological disorder	Neurological
PGS000750	MONDO_0005180	Parkinson disease	Parkinson disease	MONDO_0005180	Metabolic disorder	Metabolic
PGS000750	MONDO_0005180	Parkinson disease	Parkinson disease	MONDO_0005180	Neurological disorder	Neurological
PGS000903	MONDO_0005180	Parkinson disease	Parkinson disease	MONDO_0005180	Metabolic disorder	Metabolic
PGS000903	MONDO_0005180	Parkinson disease	Parkinson disease	MONDO_0005180	Neurological disorder	Neurological
PGS001774	MONDO_0005180	Parkinson disease	Parkinson disease	MONDO_0005180	Metabolic disorder	Metabolic
PGS001774	MONDO_0005180	Parkinson disease	Parkinson disease	MONDO_0005180	Neurological disorder	Neurological
PGS000060	EFO_0004612	high density lipoprotein cholesterol measurement	high density lipoprotein cholesterol measurement	EFO_0004612	Lipid or lipoprotein measurement	Metabolic
PGS000064	EFO_0004612	high density lipoprotein cholesterol measurement	high density lipoprotein cholesterol measurement	EFO_0004612	Lipid or lipoprotein measurement	Metabolic
PGS000192	EFO_0004612	high density lipoprotein cholesterol measurement|low density lipoprotein cholesterol measurement	high density lipoprotein cholesterol measurement	EFO_0004612	Lipid or lipoprotein measurement	Metabolic
PGS000309	EFO_0004612	high density lipoprotein cholesterol measurement	high density lipoprotein cholesterol measurement	EFO_0004612	Lipid or lipoprotein measurement	Metabolic
PGS000686	EFO_0004612	high density lipoprotein cholesterol measurement	high density lipoprotein cholesterol measurement	EFO_0004612	Lipid or lipoprotein measurement	Metabolic
PGS000825	EFO_0004612	high density lipoprotein cholesterol measurement	high density lipoprotein cholesterol measurement	EFO_0004612	Lipid or lipoprotein measurement	Metabolic

PGS001954	EFO_0004612	high density lipoprotein cholesterol measurement	high density lipoprotein cholesterol measurement	EFO_0004612	Lipid or lipoprotein measurement	Metabolic
PGS002172	EFO_0004612	high density lipoprotein cholesterol measurement	high density lipoprotein cholesterol measurement	EFO_0004612	Lipid or lipoprotein measurement	Metabolic
PGS002284	EFO_0004612	high density lipoprotein cholesterol measurement	high density lipoprotein cholesterol measurement	EFO_0004612	Lipid or lipoprotein measurement	Metabolic
PGS002329	EFO_0004612	high density lipoprotein cholesterol measurement	high density lipoprotein cholesterol measurement	EFO_0004612	Lipid or lipoprotein measurement	Metabolic
PGS002366	EFO_0004612	high density lipoprotein cholesterol measurement	high density lipoprotein cholesterol measurement	EFO_0004612	Lipid or lipoprotein measurement	Metabolic
PGS002548	EFO_0004612	high density lipoprotein cholesterol measurement	high density lipoprotein cholesterol measurement	EFO_0004612	Lipid or lipoprotein measurement	Metabolic
PGS002597	EFO_0004612	high density lipoprotein cholesterol measurement	high density lipoprotein cholesterol measurement	EFO_0004612	Lipid or lipoprotein measurement	Metabolic
PGS002781	EFO_0004612	high density lipoprotein cholesterol measurement	high density lipoprotein cholesterol measurement	EFO_0004612	Lipid or lipoprotein measurement	Metabolic
PGS002954	EFO_0004612	high density lipoprotein cholesterol measurement	high density lipoprotein cholesterol measurement	EFO_0004612	Lipid or lipoprotein measurement	Metabolic
PGS002955	EFO_0004612	high density lipoprotein cholesterol measurement	high density lipoprotein cholesterol measurement	EFO_0004612	Lipid or lipoprotein measurement	Metabolic
PGS002956	EFO_0004612	high density lipoprotein cholesterol measurement	high density lipoprotein cholesterol measurement	EFO_0004612	Lipid or lipoprotein measurement	Metabolic
PGS002957	EFO_0004612	high density lipoprotein cholesterol measurement	high density lipoprotein cholesterol measurement	EFO_0004612	Lipid or lipoprotein measurement	Metabolic
PGS002958	EFO_0004612	high density lipoprotein cholesterol measurement	high density lipoprotein cholesterol measurement	EFO_0004612	Lipid or lipoprotein measurement	Metabolic
PGS002959	EFO_0004612	high density lipoprotein cholesterol measurement	high density lipoprotein cholesterol measurement	EFO_0004612	Lipid or lipoprotein measurement	Metabolic
PGS002960	EFO_0004612	high density lipoprotein cholesterol measurement	high density lipoprotein cholesterol measurement	EFO_0004612	Lipid or lipoprotein measurement	Metabolic
PGS002961	EFO_0004612	high density lipoprotein cholesterol measurement	high density lipoprotein cholesterol measurement	EFO_0004612	Lipid or lipoprotein measurement	Metabolic
PGS002962	EFO_0004612	high density lipoprotein cholesterol measurement	high density lipoprotein cholesterol measurement	EFO_0004612	Lipid or lipoprotein measurement	Metabolic
PGS002963	EFO_0004612	high density lipoprotein cholesterol measurement	high density lipoprotein cholesterol measurement	EFO_0004612	Lipid or lipoprotein measurement	Metabolic
PGS000061	EFO_0004611	low density lipoprotein cholesterol measurement	low density lipoprotein cholesterol measurement	EFO_0004611	Cardiovascular measurement	Cardiovascular
PGS000061	EFO_0004611	low density lipoprotein cholesterol measurement	low density lipoprotein cholesterol measurement	EFO_0004611	Lipid or lipoprotein measurement	Metabolic
PGS000065	EFO_0004611	low density lipoprotein cholesterol measurement	low density lipoprotein cholesterol measurement	EFO_0004611	Cardiovascular measurement	Cardiovascular
PGS000065	EFO_0004611	low density lipoprotein cholesterol measurement	low density lipoprotein cholesterol measurement	EFO_0004611	Lipid or lipoprotein measurement	Metabolic
PGS000115	EFO_0004611	low density lipoprotein cholesterol measurement	low density lipoprotein cholesterol measurement	EFO_0004611	Cardiovascular measurement	Cardiovascular
PGS000115	EFO_0004611	low density lipoprotein cholesterol measurement	low density lipoprotein cholesterol measurement	EFO_0004611	Lipid or lipoprotein measurement	Metabolic
PGS000310	EFO_0004611	low density lipoprotein cholesterol measurement	low density lipoprotein cholesterol measurement	EFO_0004611	Cardiovascular measurement	Cardiovascular

PGS000310	EFO_0004611	low density lipoprotein cholesterol measurement	low density lipoprotein cholesterol measurement	EFO_0004611	Lipid or lipoprotein measurement	Metabolic
PGS000340	EFO_0004611	low density lipoprotein cholesterol measurement	low density lipoprotein cholesterol measurement	EFO_0004611	Cardiovascular measurement	Cardiovascular
PGS000340	EFO_0004611	low density lipoprotein cholesterol measurement	low density lipoprotein cholesterol measurement	EFO_0004611	Lipid or lipoprotein measurement	Metabolic
PGS000688	EFO_0004611	low density lipoprotein cholesterol measurement	low density lipoprotein cholesterol measurement	EFO_0004611	Cardiovascular measurement	Cardiovascular
PGS000688	EFO_0004611	low density lipoprotein cholesterol measurement	low density lipoprotein cholesterol measurement	EFO_0004611	Lipid or lipoprotein measurement	Metabolic
PGS000814	EFO_0004611	low density lipoprotein cholesterol measurement	low density lipoprotein cholesterol measurement	EFO_0004611	Cardiovascular measurement	Cardiovascular
PGS000814	EFO_0004611	low density lipoprotein cholesterol measurement	low density lipoprotein cholesterol measurement	EFO_0004611	Lipid or lipoprotein measurement	Metabolic
PGS000824	EFO_0004611	low density lipoprotein cholesterol measurement	low density lipoprotein cholesterol measurement	EFO_0004611	Cardiovascular measurement	Cardiovascular
PGS000824	EFO_0004611	low density lipoprotein cholesterol measurement	low density lipoprotein cholesterol measurement	EFO_0004611	Lipid or lipoprotein measurement	Metabolic
PGS000875	EFO_0004611	low density lipoprotein cholesterol measurement	low density lipoprotein cholesterol measurement	EFO_0004611	Cardiovascular measurement	Cardiovascular
PGS000875	EFO_0004611	low density lipoprotein cholesterol measurement	low density lipoprotein cholesterol measurement	EFO_0004611	Lipid or lipoprotein measurement	Metabolic
PGS000886	EFO_0004611	low density lipoprotein cholesterol measurement	low density lipoprotein cholesterol measurement	EFO_0004611	Cardiovascular measurement	Cardiovascular
PGS000886	EFO_0004611	low density lipoprotein cholesterol measurement	low density lipoprotein cholesterol measurement	EFO_0004611	Lipid or lipoprotein measurement	Metabolic
PGS000887	EFO_0004611	low density lipoprotein cholesterol measurement	low density lipoprotein cholesterol measurement	EFO_0004611	Cardiovascular measurement	Cardiovascular
PGS000887	EFO_0004611	low density lipoprotein cholesterol measurement	low density lipoprotein cholesterol measurement	EFO_0004611	Lipid or lipoprotein measurement	Metabolic
PGS000888	EFO_0004611	low density lipoprotein cholesterol measurement	low density lipoprotein cholesterol measurement	EFO_0004611	Cardiovascular measurement	Cardiovascular
PGS000888	EFO_0004611	low density lipoprotein cholesterol measurement	low density lipoprotein cholesterol measurement	EFO_0004611	Lipid or lipoprotein measurement	Metabolic
PGS000889	EFO_0004611	low density lipoprotein cholesterol measurement	low density lipoprotein cholesterol measurement	EFO_0004611	Cardiovascular measurement	Cardiovascular
PGS000889	EFO_0004611	low density lipoprotein cholesterol measurement	low density lipoprotein cholesterol measurement	EFO_0004611	Lipid or lipoprotein measurement	Metabolic
PGS000890	EFO_0004611	low density lipoprotein cholesterol measurement	low density lipoprotein cholesterol measurement	EFO_0004611	Cardiovascular measurement	Cardiovascular
PGS000890	EFO_0004611	low density lipoprotein cholesterol measurement	low density lipoprotein cholesterol measurement	EFO_0004611	Lipid or lipoprotein measurement	Metabolic
PGS000891	EFO_0004611	low density lipoprotein cholesterol measurement	low density lipoprotein cholesterol measurement	EFO_0004611	Cardiovascular measurement	Cardiovascular
PGS000891	EFO_0004611	low density lipoprotein cholesterol measurement	low density lipoprotein cholesterol measurement	EFO_0004611	Lipid or lipoprotein measurement	Metabolic
PGS000892	EFO_0004611	low density lipoprotein cholesterol measurement	low density lipoprotein cholesterol measurement	EFO_0004611	Cardiovascular measurement	Cardiovascular
PGS000892	EFO_0004611	low density lipoprotein cholesterol measurement	low density lipoprotein cholesterol measurement	EFO_0004611	Lipid or lipoprotein measurement	Metabolic

PGS000893	EFO_0004611	low density lipoprotein cholesterol measurement	low density lipoprotein cholesterol measurement	EFO_0004611	Cardiovascular measurement	Cardiovascular
PGS000893	EFO_0004611	low density lipoprotein cholesterol measurement	low density lipoprotein cholesterol measurement	EFO_0004611	Lipid or lipoprotein measurement	Metabolic
PGS000895	EFO_0004611	low density lipoprotein cholesterol measurement	low density lipoprotein cholesterol measurement	EFO_0004611	Cardiovascular measurement	Cardiovascular
PGS000895	EFO_0004611	low density lipoprotein cholesterol measurement	low density lipoprotein cholesterol measurement	EFO_0004611	Lipid or lipoprotein measurement	Metabolic
PGS000897	EFO_0004611	low density lipoprotein cholesterol measurement	low density lipoprotein cholesterol measurement	EFO_0004611	Cardiovascular measurement	Cardiovascular
PGS000897	EFO_0004611	low density lipoprotein cholesterol measurement	low density lipoprotein cholesterol measurement	EFO_0004611	Lipid or lipoprotein measurement	Metabolic
PGS001933	EFO_0004611	low density lipoprotein cholesterol measurement	low density lipoprotein cholesterol measurement	EFO_0004611	Cardiovascular measurement	Cardiovascular
PGS001933	EFO_0004611	low density lipoprotein cholesterol measurement	low density lipoprotein cholesterol measurement	EFO_0004611	Lipid or lipoprotein measurement	Metabolic
PGS002150	EFO_0004611	low density lipoprotein cholesterol measurement	low density lipoprotein cholesterol measurement	EFO_0004611	Cardiovascular measurement	Cardiovascular
PGS002150	EFO_0004611	low density lipoprotein cholesterol measurement	low density lipoprotein cholesterol measurement	EFO_0004611	Lipid or lipoprotein measurement	Metabolic
PGS002274	EFO_0004611	low density lipoprotein cholesterol measurement	low density lipoprotein cholesterol measurement	EFO_0004611	Cardiovascular measurement	Cardiovascular
PGS002274	EFO_0004611	low density lipoprotein cholesterol measurement	low density lipoprotein cholesterol measurement	EFO_0004611	Lipid or lipoprotein measurement	Metabolic
PGS002285	EFO_0004611	low density lipoprotein cholesterol measurement	low density lipoprotein cholesterol measurement	EFO_0004611	Cardiovascular measurement	Cardiovascular
PGS002285	EFO_0004611	low density lipoprotein cholesterol measurement	low density lipoprotein cholesterol measurement	EFO_0004611	Lipid or lipoprotein measurement	Metabolic
PGS002337	EFO_0004611	low density lipoprotein cholesterol measurement	low density lipoprotein cholesterol measurement	EFO_0004611	Cardiovascular measurement	Cardiovascular
PGS002337	EFO_0004611	low density lipoprotein cholesterol measurement	low density lipoprotein cholesterol measurement	EFO_0004611	Lipid or lipoprotein measurement	Metabolic
PGS002369	EFO_0004611	low density lipoprotein cholesterol measurement	low density lipoprotein cholesterol measurement	EFO_0004611	Cardiovascular measurement	Cardiovascular
PGS002369	EFO_0004611	low density lipoprotein cholesterol measurement	low density lipoprotein cholesterol measurement	EFO_0004611	Lipid or lipoprotein measurement	Metabolic
PGS002409	EFO_0004611	low density lipoprotein cholesterol measurement	low density lipoprotein cholesterol measurement	EFO_0004611	Cardiovascular measurement	Cardiovascular
PGS002409	EFO_0004611	low density lipoprotein cholesterol measurement	low density lipoprotein cholesterol measurement	EFO_0004611	Lipid or lipoprotein measurement	Metabolic
PGS002458	EFO_0004611	low density lipoprotein cholesterol measurement	low density lipoprotein cholesterol measurement	EFO_0004611	Cardiovascular measurement	Cardiovascular
PGS002458	EFO_0004611	low density lipoprotein cholesterol measurement	low density lipoprotein cholesterol measurement	EFO_0004611	Lipid or lipoprotein measurement	Metabolic
PGS002507	EFO_0004611	low density lipoprotein cholesterol measurement	low density lipoprotein cholesterol measurement	EFO_0004611	Cardiovascular measurement	Cardiovascular
PGS002507	EFO_0004611	low density lipoprotein cholesterol measurement	low density lipoprotein cholesterol measurement	EFO_0004611	Lipid or lipoprotein measurement	Metabolic
PGS002556	EFO_0004611	low density lipoprotein cholesterol measurement	low density lipoprotein cholesterol measurement	EFO_0004611	Cardiovascular measurement	Cardiovascular

PGS002556	EFO_0004611	low density lipoprotein cholesterol measurement	low density lipoprotein cholesterol measurement	EFO_0004611	Lipid or lipoprotein measurement	Metabolic
PGS002605	EFO_0004611	low density lipoprotein cholesterol measurement	low density lipoprotein cholesterol measurement	EFO_0004611	Cardiovascular measurement	Cardiovascular
PGS002605	EFO_0004611	low density lipoprotein cholesterol measurement	low density lipoprotein cholesterol measurement	EFO_0004611	Lipid or lipoprotein measurement	Metabolic
PGS002654	EFO_0004611	low density lipoprotein cholesterol measurement	low density lipoprotein cholesterol measurement	EFO_0004611	Cardiovascular measurement	Cardiovascular
PGS002654	EFO_0004611	low density lipoprotein cholesterol measurement	low density lipoprotein cholesterol measurement	EFO_0004611	Lipid or lipoprotein measurement	Metabolic
PGS003029	EFO_0004611	low density lipoprotein cholesterol measurement	low density lipoprotein cholesterol measurement	EFO_0004611	Cardiovascular measurement	Cardiovascular
PGS003029	EFO_0004611	low density lipoprotein cholesterol measurement	low density lipoprotein cholesterol measurement	EFO_0004611	Lipid or lipoprotein measurement	Metabolic
PGS003030	EFO_0004611	low density lipoprotein cholesterol measurement	low density lipoprotein cholesterol measurement	EFO_0004611	Cardiovascular measurement	Cardiovascular
PGS003030	EFO_0004611	low density lipoprotein cholesterol measurement	low density lipoprotein cholesterol measurement	EFO_0004611	Lipid or lipoprotein measurement	Metabolic
PGS003031	EFO_0004611	low density lipoprotein cholesterol measurement	low density lipoprotein cholesterol measurement	EFO_0004611	Cardiovascular measurement	Cardiovascular
PGS003031	EFO_0004611	low density lipoprotein cholesterol measurement	low density lipoprotein cholesterol measurement	EFO_0004611	Lipid or lipoprotein measurement	Metabolic
PGS003032	EFO_0004611	low density lipoprotein cholesterol measurement	low density lipoprotein cholesterol measurement	EFO_0004611	Cardiovascular measurement	Cardiovascular
PGS003032	EFO_0004611	low density lipoprotein cholesterol measurement	low density lipoprotein cholesterol measurement	EFO_0004611	Lipid or lipoprotein measurement	Metabolic
PGS003033	EFO_0004611	low density lipoprotein cholesterol measurement	low density lipoprotein cholesterol measurement	EFO_0004611	Cardiovascular measurement	Cardiovascular
PGS003033	EFO_0004611	low density lipoprotein cholesterol measurement	low density lipoprotein cholesterol measurement	EFO_0004611	Lipid or lipoprotein measurement	Metabolic
PGS003034	EFO_0004611	low density lipoprotein cholesterol measurement	low density lipoprotein cholesterol measurement	EFO_0004611	Cardiovascular measurement	Cardiovascular
PGS003034	EFO_0004611	low density lipoprotein cholesterol measurement	low density lipoprotein cholesterol measurement	EFO_0004611	Lipid or lipoprotein measurement	Metabolic
PGS003035	EFO_0004611	low density lipoprotein cholesterol measurement	low density lipoprotein cholesterol measurement	EFO_0004611	Cardiovascular measurement	Cardiovascular
PGS003035	EFO_0004611	low density lipoprotein cholesterol measurement	low density lipoprotein cholesterol measurement	EFO_0004611	Lipid or lipoprotein measurement	Metabolic
PGS003036	EFO_0004611	low density lipoprotein cholesterol measurement	low density lipoprotein cholesterol measurement	EFO_0004611	Cardiovascular measurement	Cardiovascular
PGS003036	EFO_0004611	low density lipoprotein cholesterol measurement	low density lipoprotein cholesterol measurement	EFO_0004611	Lipid or lipoprotein measurement	Metabolic
PGS003037	EFO_0004611	low density lipoprotein cholesterol measurement	low density lipoprotein cholesterol measurement	EFO_0004611	Cardiovascular measurement	Cardiovascular
PGS003037	EFO_0004611	low density lipoprotein cholesterol measurement	low density lipoprotein cholesterol measurement	EFO_0004611	Lipid or lipoprotein measurement	Metabolic
PGS003038	EFO_0004611	low density lipoprotein cholesterol measurement	low density lipoprotein cholesterol measurement	EFO_0004611	Cardiovascular measurement	Cardiovascular
PGS003038	EFO_0004611	low density lipoprotein cholesterol measurement	low density lipoprotein cholesterol measurement	EFO_0004611	Lipid or lipoprotein measurement	Metabolic

PGS003403	EFO_0004611	low density lipoprotein cholesterol measurement	low density lipoprotein cholesterol measurement	EFO_0004611	Cardiovascular measurement	Cardiovascular
PGS003403	EFO_0004611	low density lipoprotein cholesterol measurement	low density lipoprotein cholesterol measurement	EFO_0004611	Lipid or lipoprotein measurement	Metabolic
PGS003404	EFO_0004611	low density lipoprotein cholesterol measurement	low density lipoprotein cholesterol measurement	EFO_0004611	Cardiovascular measurement	Cardiovascular
PGS003404	EFO_0004611	low density lipoprotein cholesterol measurement	low density lipoprotein cholesterol measurement	EFO_0004611	Lipid or lipoprotein measurement	Metabolic
PGS003405	EFO_0004611	low density lipoprotein cholesterol measurement	low density lipoprotein cholesterol measurement	EFO_0004611	Cardiovascular measurement	Cardiovascular
PGS003405	EFO_0004611	low density lipoprotein cholesterol measurement	low density lipoprotein cholesterol measurement	EFO_0004611	Lipid or lipoprotein measurement	Metabolic
PGS000062	EFO_0004574	total cholesterol measurement	total cholesterol measurement	EFO_0004574	Lipid or lipoprotein measurement	Metabolic
PGS000311	EFO_0004574	total cholesterol measurement	total cholesterol measurement	EFO_0004574	Lipid or lipoprotein measurement	Metabolic
PGS000677	EFO_0004574	total cholesterol measurement	total cholesterol measurement	EFO_0004574	Lipid or lipoprotein measurement	Metabolic
PGS000831	EFO_0004574	total cholesterol measurement	total cholesterol measurement	EFO_0004574	Lipid or lipoprotein measurement	Metabolic
PGS001895	EFO_0004574	total cholesterol measurement	total cholesterol measurement	EFO_0004574	Lipid or lipoprotein measurement	Metabolic
PGS002108	EFO_0004574	total cholesterol measurement	total cholesterol measurement	EFO_0004574	Lipid or lipoprotein measurement	Metabolic
PGS002286	EFO_0004574	total cholesterol measurement	total cholesterol measurement	EFO_0004574	Lipid or lipoprotein measurement	Metabolic
PGS002352	EFO_0004574	total cholesterol measurement	total cholesterol measurement	EFO_0004574	Lipid or lipoprotein measurement	Metabolic
PGS002377	EFO_0004574	total cholesterol measurement	total cholesterol measurement	EFO_0004574	Lipid or lipoprotein measurement	Metabolic
PGS002424	EFO_0004574	total cholesterol measurement	total cholesterol measurement	EFO_0004574	Lipid or lipoprotein measurement	Metabolic
PGS002473	EFO_0004574	total cholesterol measurement	total cholesterol measurement	EFO_0004574	Lipid or lipoprotein measurement	Metabolic
PGS002571	EFO_0004574	total cholesterol measurement	total cholesterol measurement	EFO_0004574	Lipid or lipoprotein measurement	Metabolic
PGS002620	EFO_0004574	total cholesterol measurement	total cholesterol measurement	EFO_0004574	Lipid or lipoprotein measurement	Metabolic
PGS002783	EFO_0004574	total cholesterol measurement	total cholesterol measurement	EFO_0004574	Lipid or lipoprotein measurement	Metabolic
PGS003134	EFO_0004574	total cholesterol measurement	total cholesterol measurement	EFO_0004574	Lipid or lipoprotein measurement	Metabolic
PGS003135	EFO_0004574	total cholesterol measurement	total cholesterol measurement	EFO_0004574	Lipid or lipoprotein measurement	Metabolic
PGS003136	EFO_0004574	total cholesterol measurement	total cholesterol measurement	EFO_0004574	Lipid or lipoprotein measurement	Metabolic
PGS003137	EFO_0004574	total cholesterol measurement	total cholesterol measurement	EFO_0004574	Lipid or lipoprotein measurement	Metabolic
PGS003138	EFO_0004574	total cholesterol measurement	total cholesterol measurement	EFO_0004574	Lipid or lipoprotein measurement	Metabolic

PGS003139	EFO_0004574	total cholesterol measurement	total cholesterol measurement	EFO_0004574	Lipid or lipoprotein measurement	Metabolic
PGS003140	EFO_0004574	total cholesterol measurement	total cholesterol measurement	EFO_0004574	Lipid or lipoprotein measurement	Metabolic
PGS003141	EFO_0004574	total cholesterol measurement	total cholesterol measurement	EFO_0004574	Lipid or lipoprotein measurement	Metabolic
PGS003142	EFO_0004574	total cholesterol measurement	total cholesterol measurement	EFO_0004574	Lipid or lipoprotein measurement	Metabolic
PGS003143	EFO_0004574	total cholesterol measurement	total cholesterol measurement	EFO_0004574	Lipid or lipoprotein measurement	Metabolic
PGS000063	EFO_0004530	triglyceride measurement	triglyceride measurement	EFO_0004530	Lipid or lipoprotein measurement	Metabolic
PGS000066	EFO_0004530	triglyceride measurement	triglyceride measurement	EFO_0004530	Lipid or lipoprotein measurement	Metabolic
PGS000312	EFO_0004530	triglyceride measurement	triglyceride measurement	EFO_0004530	Lipid or lipoprotein measurement	Metabolic
PGS000699	EFO_0004530	triglyceride measurement	triglyceride measurement	EFO_0004530	Lipid or lipoprotein measurement	Metabolic
PGS000826	EFO_0004530	triglyceride measurement	triglyceride measurement	EFO_0004530	Lipid or lipoprotein measurement	Metabolic
PGS000847	EFO_0004530	triglyceride measurement	triglyceride measurement	EFO_0004530	Lipid or lipoprotein measurement	Metabolic
PGS001979	EFO_0004530	triglyceride measurement	triglyceride measurement	EFO_0004530	Lipid or lipoprotein measurement	Metabolic
PGS002197	EFO_0004530	triglyceride measurement	triglyceride measurement	EFO_0004530	Lipid or lipoprotein measurement	Metabolic
PGS002287	EFO_0004530	triglyceride measurement	triglyceride measurement	EFO_0004530	Lipid or lipoprotein measurement	Metabolic
PGS002353	EFO_0004530	triglyceride measurement	triglyceride measurement	EFO_0004530	Lipid or lipoprotein measurement	Metabolic
PGS002378	EFO_0004530	triglyceride measurement	triglyceride measurement	EFO_0004530	Lipid or lipoprotein measurement	Metabolic
PGS002425	EFO_0004530	triglyceride measurement	triglyceride measurement	EFO_0004530	Lipid or lipoprotein measurement	Metabolic
PGS002474	EFO_0004530	triglyceride measurement	triglyceride measurement	EFO_0004530	Lipid or lipoprotein measurement	Metabolic
PGS002523	EFO_0004530	triglyceride measurement	triglyceride measurement	EFO_0004530	Lipid or lipoprotein measurement	Metabolic
PGS002572	EFO_0004530	triglyceride measurement	triglyceride measurement	EFO_0004530	Lipid or lipoprotein measurement	Metabolic
PGS002621	EFO_0004530	triglyceride measurement	triglyceride measurement	EFO_0004530	Lipid or lipoprotein measurement	Metabolic
PGS002784	EFO_0004530	triglyceride measurement	triglyceride measurement	EFO_0004530	Lipid or lipoprotein measurement	Metabolic
PGS003144	EFO_0004530	triglyceride measurement	triglyceride measurement	EFO_0004530	Lipid or lipoprotein measurement	Metabolic
PGS003145	EFO_0004530	triglyceride measurement	triglyceride measurement	EFO_0004530	Lipid or lipoprotein measurement	Metabolic
PGS003146	EFO_0004530	triglyceride measurement	triglyceride measurement	EFO_0004530	Lipid or lipoprotein measurement	Metabolic

PGS003147	EFO_0004530	triglyceride measurement	triglyceride measurement	EFO_0004530	Lipid or lipoprotein measurement	Metabolic
PGS003148	EFO_0004530	triglyceride measurement	triglyceride measurement	EFO_0004530	Lipid or lipoprotein measurement	Metabolic
PGS003149	EFO_0004530	triglyceride measurement	triglyceride measurement	EFO_0004530	Lipid or lipoprotein measurement	Metabolic
PGS003150	EFO_0004530	triglyceride measurement	triglyceride measurement	EFO_0004530	Lipid or lipoprotein measurement	Metabolic
PGS003151	EFO_0004530	triglyceride measurement	triglyceride measurement	EFO_0004530	Lipid or lipoprotein measurement	Metabolic
PGS003152	EFO_0004530	triglyceride measurement	triglyceride measurement	EFO_0004530	Lipid or lipoprotein measurement	Metabolic
PGS003153	EFO_0004530	triglyceride measurement	triglyceride measurement	EFO_0004530	Lipid or lipoprotein measurement	Metabolic
PGS003401	EFO_0004530	triglyceride measurement	triglyceride measurement	EFO_0004530	Lipid or lipoprotein measurement	Metabolic
PGS000069	EFO_1001516	ovarian serous carcinoma	ovarian serous carcinoma	EFO_1001516	Cancer	Neoplasms
PGS003385	EFO_1001516	ovarian serous carcinoma	ovarian serous carcinoma	EFO_1001516	Cancer	Neoplasms
PGS000070	EFO_0000571	lung adenocarcinoma	lung adenocarcinoma	EFO_0000571	Cancer	Neoplasms
PGS003393	EFO_0000571	lung adenocarcinoma	lung adenocarcinoma	EFO_0000571	Cancer	Neoplasms
PGS000071	MONDO_0004986	urinary bladder carcinoma	urinary bladder carcinoma	MONDO_0004986	Cancer	Neoplasms
PGS000607	MONDO_0004986	urinary bladder carcinoma	urinary bladder carcinoma	MONDO_0004986	Cancer	Neoplasms
PGS000608	MONDO_0004986	urinary bladder carcinoma	urinary bladder carcinoma	MONDO_0004986	Cancer	Neoplasms
PGS000609	MONDO_0004986	urinary bladder carcinoma	urinary bladder carcinoma	MONDO_0004986	Cancer	Neoplasms
PGS000610	MONDO_0004986	urinary bladder carcinoma	urinary bladder carcinoma	MONDO_0004986	Cancer	Neoplasms
PGS000611	MONDO_0004986	urinary bladder carcinoma	urinary bladder carcinoma	MONDO_0004986	Cancer	Neoplasms
PGS000612	MONDO_0004986	urinary bladder carcinoma	urinary bladder carcinoma	MONDO_0004986	Cancer	Neoplasms
PGS000613	MONDO_0004986	urinary bladder carcinoma	urinary bladder carcinoma	MONDO_0004986	Cancer	Neoplasms
PGS000614	MONDO_0004986	urinary bladder carcinoma	urinary bladder carcinoma	MONDO_0004986	Cancer	Neoplasms
PGS000615	MONDO_0004986	urinary bladder carcinoma	urinary bladder carcinoma	MONDO_0004986	Cancer	Neoplasms
PGS000616	MONDO_0004986	urinary bladder carcinoma	urinary bladder carcinoma	MONDO_0004986	Cancer	Neoplasms
PGS000782	MONDO_0004986	urinary bladder carcinoma	urinary bladder carcinoma	MONDO_0004986	Cancer	Neoplasms
PGS001807	MONDO_0004986	urinary bladder carcinoma	urinary bladder carcinoma	MONDO_0004986	Cancer	Neoplasms
PGS002017	MONDO_0004986	urinary bladder carcinoma	urinary bladder carcinoma	MONDO_0004986	Cancer	Neoplasms
PGS003335	MONDO_0004986	urinary bladder carcinoma	urinary bladder carcinoma	MONDO_0004986	Cancer	Neoplasms

PGS000073	EFO_0001061	cervical carcinoma	cervical carcinoma	EFO_0001061	Cancer	Neoplasms
PGS000784	EFO_0001061	cervical carcinoma	cervical carcinoma	EFO_0001061	Cancer	Neoplasms
PGS001299	EFO_0001061	cervical carcinoma	cervical carcinoma	EFO_0001061	Cancer	Neoplasms
PGS003389	EFO_0001061	cervical carcinoma	cervical carcinoma	EFO_0001061	Cancer	Neoplasms
PGS000075	EFO_1001512	endometrial carcinoma	endometrial carcinoma	EFO_1001512	Cancer	Neoplasms
PGS000786	EFO_1001512	endometrial carcinoma	endometrial carcinoma	EFO_1001512	Cancer	Neoplasms
PGS002735	EFO_1001512	endometrial carcinoma	endometrial carcinoma	EFO_1001512	Cancer	Neoplasms
PGS002737	EFO_1001512	endometrial carcinoma	endometrial carcinoma	EFO_1001512	Cancer	Neoplasms
PGS003381	EFO_1001512	endometrial carcinoma	endometrial carcinoma	EFO_1001512	Cancer	Neoplasms
PGS000076	EFO_0000681	renal cell carcinoma	renal cell carcinoma	EFO_0000681	Cancer	Neoplasms
PGS000787	EFO_0000681	renal cell carcinoma	renal cell carcinoma	EFO_0000681	Cancer	Neoplasms
PGS000077	EFO_0004289	lymphoid leukemia	lymphoid leukemia	EFO_0004289	Cancer	Neoplasms
PGS000077	EFO_0004289	lymphoid leukemia	lymphoid leukemia	EFO_0004289	Immune system disorder	Immunological
PGS000645	EFO_0004289	lymphoid leukemia	lymphoid leukemia	EFO_0004289	Cancer	Neoplasms
PGS000645	EFO_0004289	lymphoid leukemia	lymphoid leukemia	EFO_0004289	Immune system disorder	Immunological
PGS000788	EFO_0004289	lymphoid leukemia	lymphoid leukemia	EFO_0004289	Cancer	Neoplasms
PGS000788	EFO_0004289	lymphoid leukemia	lymphoid leukemia	EFO_0004289	Immune system disorder	Immunological
PGS000078	EFO_0001071	lung carcinoma	lung carcinoma	EFO_0001071	Cancer	Neoplasms
PGS000156	EFO_0001071	lung carcinoma	lung carcinoma	EFO_0001071	Cancer	Neoplasms
PGS000740	EFO_0001071	lung carcinoma	lung carcinoma	EFO_0001071	Cancer	Neoplasms
PGS000789	EFO_0001071	lung carcinoma	lung carcinoma	EFO_0001071	Cancer	Neoplasms
PGS000880	EFO_0001071	lung carcinoma	lung carcinoma	EFO_0001071	Cancer	Neoplasms
PGS002270	EFO_0001071	lung carcinoma	lung carcinoma	EFO_0001071	Cancer	Neoplasms
PGS002808	EFO_0001071	lung carcinoma	lung carcinoma	EFO_0001071	Cancer	Neoplasms
PGS003391	EFO_0001071	lung carcinoma	lung carcinoma	EFO_0001071	Cancer	Neoplasms
PGS000079	EFO_0000756	melanoma	melanoma	EFO_0000756	Cancer	Neoplasms
PGS000408	EFO_0000756	melanoma	melanoma	EFO_0000756	Cancer	Neoplasms
PGS000409	EFO_0000756	melanoma	melanoma	EFO_0000756	Cancer	Neoplasms
PGS000410	EFO_0000756	melanoma	melanoma	EFO_0000756	Cancer	Neoplasms
PGS000411	EFO_0000756	melanoma	melanoma	EFO_0000756	Cancer	Neoplasms
PGS000412	EFO_0000756	melanoma	melanoma	EFO_0000756	Cancer	Neoplasms
PGS000413	EFO_0000756	melanoma	melanoma	EFO_0000756	Cancer	Neoplasms
PGS000414	EFO_0000756	melanoma	melanoma	EFO_0000756	Cancer	Neoplasms
PGS000415	EFO_0000756	melanoma	melanoma	EFO_0000756	Cancer	Neoplasms
PGS000416	EFO_0000756	melanoma	melanoma	EFO_0000756	Cancer	Neoplasms
PGS000417	EFO_0000756	melanoma	melanoma	EFO_0000756	Cancer	Neoplasms
PGS000418	EFO_0000756	melanoma	melanoma	EFO_0000756	Cancer	Neoplasms
PGS000419	EFO_0000756	melanoma	melanoma	EFO_0000756	Cancer	Neoplasms
PGS000420	EFO_0000756	melanoma	melanoma	EFO_0000756	Cancer	Neoplasms
PGS000421	EFO_0000756	melanoma	melanoma	EFO_0000756	Cancer	Neoplasms
PGS000422	EFO_0000756	melanoma	melanoma	EFO_0000756	Cancer	Neoplasms
PGS000423	EFO_0000756	melanoma	melanoma	EFO_0000756	Cancer	Neoplasms
PGS000424	EFO_0000756	melanoma	melanoma	EFO_0000756	Cancer	Neoplasms
PGS000425	EFO_0000756	melanoma	melanoma	EFO_0000756	Cancer	Neoplasms
PGS000426	EFO_0000756	melanoma	melanoma	EFO_0000756	Cancer	Neoplasms
PGS000427	EFO_0000756	melanoma	melanoma	EFO_0000756	Cancer	Neoplasms
PGS000428	EFO_0000756	melanoma	melanoma	EFO_0000756	Cancer	Neoplasms

PGS000429	EFO_0000756	melanoma	melanoma	EFO_0000756	Cancer	Neoplasms
PGS000430	EFO_0000756	melanoma	melanoma	EFO_0000756	Cancer	Neoplasms
PGS000431	EFO_0000756	melanoma	melanoma	EFO_0000756	Cancer	Neoplasms
PGS000432	EFO_0000756	melanoma	melanoma	EFO_0000756	Cancer	Neoplasms
PGS000732	EFO_0000756	melanoma	melanoma	EFO_0000756	Cancer	Neoplasms
PGS000743	EFO_0000756	melanoma	melanoma	EFO_0000756	Cancer	Neoplasms
PGS000744	EFO_0000756	melanoma	melanoma	EFO_0000756	Cancer	Neoplasms
PGS000745	EFO_0000756	melanoma	melanoma	EFO_0000756	Cancer	Neoplasms
PGS000790	EFO_0000756	melanoma	melanoma	EFO_0000756	Cancer	Neoplasms
PGS000813	EFO_0000756	melanoma	melanoma	EFO_0000756	Cancer	Neoplasms
PGS001304	EFO_0000756	melanoma	melanoma	EFO_0000756	Cancer	Neoplasms
PGS002246	EFO_0000756	melanoma	melanoma	EFO_0000756	Cancer	Neoplasms
PGS002247	EFO_0000756	melanoma	melanoma	EFO_0000756	Cancer	Neoplasms
PGS002253	EFO_0000756	melanoma	melanoma	EFO_0000756	Cancer	Neoplasms
PGS003412	EFO_0000756	melanoma	melanoma	EFO_0000756	Cancer	Neoplasms
PGS000080	EFO_0005952	non-Hodgkins lymphoma	non-Hodgkins lymphoma	EFO_0005952	Cancer	Neoplasms
PGS000641	EFO_0005952	non-Hodgkins lymphoma	non-Hodgkins lymphoma	EFO_0005952	Cancer	Neoplasms
PGS000642	EFO_0005952	non-Hodgkins lymphoma	non-Hodgkins lymphoma	EFO_0005952	Cancer	Neoplasms
PGS000791	EFO_0005952	non-Hodgkins lymphoma	non-Hodgkins lymphoma	EFO_0005952	Cancer	Neoplasms
PGS000081	EFO_0005570	oral cavity cancer|pharynx cancer	oral cavity cancer	EFO_0005570	Cancer	Neoplasms
PGS000081	EFO_0005570	oral cavity cancer|pharynx cancer	oral cavity cancer	EFO_0005570	Digestive system disorder	Gastrointestinal
PGS000357	EFO_0005570	oral cavity cancer	oral cavity cancer	EFO_0005570	Cancer	Neoplasms
PGS000357	EFO_0005570	oral cavity cancer	oral cavity cancer	EFO_0005570	Digestive system disorder	Gastrointestinal
PGS000358	EFO_0005570	oral cavity cancer	oral cavity cancer	EFO_0005570	Cancer	Neoplasms
PGS000358	EFO_0005570	oral cavity cancer	oral cavity cancer	EFO_0005570	Digestive system disorder	Gastrointestinal
PGS000083	EFO_0002618	pancreatic carcinoma	pancreatic carcinoma	EFO_0002618	Cancer	Neoplasms
PGS000083	EFO_0002618	pancreatic carcinoma	pancreatic carcinoma	EFO_0002618	Digestive system disorder	Gastrointestinal
PGS000385	EFO_0002618	pancreatic carcinoma	pancreatic carcinoma	EFO_0002618	Cancer	Neoplasms
PGS000385	EFO_0002618	pancreatic carcinoma	pancreatic carcinoma	EFO_0002618	Digestive system disorder	Gastrointestinal
PGS000386	EFO_0002618	pancreatic carcinoma	pancreatic carcinoma	EFO_0002618	Cancer	Neoplasms
PGS000386	EFO_0002618	pancreatic carcinoma	pancreatic carcinoma	EFO_0002618	Digestive system disorder	Gastrointestinal
PGS000794	EFO_0002618	pancreatic carcinoma	pancreatic carcinoma	EFO_0002618	Cancer	Neoplasms
PGS000794	EFO_0002618	pancreatic carcinoma	pancreatic carcinoma	EFO_0002618	Digestive system disorder	Gastrointestinal
PGS000086	EFO_0005088	testicular carcinoma|Testicular Germ Cell Tumor	testicular carcinoma	EFO_0005088	Cancer	Neoplasms
PGS000595	EFO_0005088	testicular carcinoma	testicular carcinoma	EFO_0005088	Cancer	Neoplasms
PGS000596	EFO_0005088	testicular carcinoma	testicular carcinoma	EFO_0005088	Cancer	Neoplasms
PGS000597	EFO_0005088	testicular carcinoma	testicular carcinoma	EFO_0005088	Cancer	Neoplasms
PGS000598	EFO_0005088	testicular carcinoma	testicular carcinoma	EFO_0005088	Cancer	Neoplasms
PGS000599	EFO_0005088	testicular carcinoma	testicular carcinoma	EFO_0005088	Cancer	Neoplasms
PGS000600	EFO_0005088	testicular carcinoma	testicular carcinoma	EFO_0005088	Cancer	Neoplasms
PGS000601	EFO_0005088	testicular carcinoma	testicular carcinoma	EFO_0005088	Cancer	Neoplasms
PGS000602	EFO_0005088	testicular carcinoma	testicular carcinoma	EFO_0005088	Cancer	Neoplasms

PGS000603	EFO_0005088	testicular carcinoma	testicular carcinoma	EFO_0005088	Cancer	Neoplasms
PGS000604	EFO_0005088	testicular carcinoma	testicular carcinoma	EFO_0005088	Cancer	Neoplasms
PGS000796	EFO_0005088	testicular carcinoma|Testicular Germ Cell Tumor	testicular carcinoma	EFO_0005088	Cancer	Neoplasms
PGS001164	EFO_0005088	testicular carcinoma	testicular carcinoma	EFO_0005088	Cancer	Neoplasms
PGS000087	EFO_0002892	thyroid carcinoma	thyroid carcinoma	EFO_0002892	Cancer	Neoplasms
PGS000207	EFO_0002892	thyroid carcinoma	thyroid carcinoma	EFO_0002892	Cancer	Neoplasms
PGS000208	EFO_0002892	thyroid carcinoma	thyroid carcinoma	EFO_0002892	Cancer	Neoplasms
PGS000209	EFO_0002892	thyroid carcinoma	thyroid carcinoma	EFO_0002892	Cancer	Neoplasms
PGS000626	EFO_0002892	thyroid carcinoma	thyroid carcinoma	EFO_0002892	Cancer	Neoplasms
PGS000627	EFO_0002892	thyroid carcinoma	thyroid carcinoma	EFO_0002892	Cancer	Neoplasms
PGS000628	EFO_0002892	thyroid carcinoma	thyroid carcinoma	EFO_0002892	Cancer	Neoplasms
PGS000629	EFO_0002892	thyroid carcinoma	thyroid carcinoma	EFO_0002892	Cancer	Neoplasms
PGS000630	EFO_0002892	thyroid carcinoma	thyroid carcinoma	EFO_0002892	Cancer	Neoplasms
PGS000631	EFO_0002892	thyroid carcinoma	thyroid carcinoma	EFO_0002892	Cancer	Neoplasms
PGS000632	EFO_0002892	thyroid carcinoma	thyroid carcinoma	EFO_0002892	Cancer	Neoplasms
PGS000633	EFO_0002892	thyroid carcinoma	thyroid carcinoma	EFO_0002892	Cancer	Neoplasms
PGS000634	EFO_0002892	thyroid carcinoma	thyroid carcinoma	EFO_0002892	Cancer	Neoplasms
PGS000635	EFO_0002892	thyroid carcinoma	thyroid carcinoma	EFO_0002892	Cancer	Neoplasms
PGS000636	EFO_0002892	thyroid carcinoma	thyroid carcinoma	EFO_0002892	Cancer	Neoplasms
PGS000797	EFO_0002892	thyroid carcinoma	thyroid carcinoma	EFO_0002892	Cancer	Neoplasms
PGS001289	EFO_0002892	thyroid carcinoma	thyroid carcinoma	EFO_0002892	Cancer	Neoplasms
PGS001794	EFO_0002892	thyroid carcinoma	thyroid carcinoma	EFO_0002892	Cancer	Neoplasms
PGS001799	EFO_0002892	thyroid carcinoma	thyroid carcinoma	EFO_0002892	Cancer	Neoplasms
PGS001809	EFO_0002892	thyroid carcinoma	thyroid carcinoma	EFO_0002892	Cancer	Neoplasms
PGS002018	EFO_0002892	thyroid carcinoma	thyroid carcinoma	EFO_0002892	Cancer	Neoplasms
PGS000088	EFO_0005090	basophil count	basophil count	EFO_0005090	Hematological measurement	Hematological
PGS000088	EFO_0005090	basophil count	basophil count	EFO_0005090	Inflammatory measurement	Immunological
PGS000163	EFO_0005090	basophil count	basophil count	EFO_0005090	Hematological measurement	Hematological
PGS000163	EFO_0005090	basophil count	basophil count	EFO_0005090	Inflammatory measurement	Immunological
PGS001378	EFO_0005090	basophil count	basophil count	EFO_0005090	Hematological measurement	Hematological
PGS001378	EFO_0005090	basophil count	basophil count	EFO_0005090	Inflammatory measurement	Immunological
PGS000089	EFO_0007992	basophil percentage of leukocytes	basophil percentage of leukocytes	EFO_0007992	Hematological measurement	Hematological
PGS000089	EFO_0007992	basophil percentage of leukocytes	basophil percentage of leukocytes	EFO_0007992	Inflammatory measurement	Immunological
PGS000164	EFO_0007992	basophil percentage of leukocytes	basophil percentage of leukocytes	EFO_0007992	Hematological measurement	Hematological
PGS000164	EFO_0007992	basophil percentage of leukocytes	basophil percentage of leukocytes	EFO_0007992	Inflammatory measurement	Immunological
PGS001377	EFO_0007992	basophil percentage of leukocytes	basophil percentage of leukocytes	EFO_0007992	Hematological measurement	Hematological
PGS001377	EFO_0007992	basophil percentage of leukocytes	basophil percentage of leukocytes	EFO_0007992	Inflammatory measurement	Immunological

PGS000090	EFO_0004842	eosinophil count	eosinophil count	EFO_0004842	Hematological measurement	Hematological
PGS000165	EFO_0004842	eosinophil count	eosinophil count	EFO_0004842	Hematological measurement	Hematological
PGS001172	EFO_0004842	eosinophil count	eosinophil count	EFO_0004842	Hematological measurement	Hematological
PGS002325	EFO_0004842	eosinophil count	eosinophil count	EFO_0004842	Hematological measurement	Hematological
PGS002364	EFO_0004842	eosinophil count	eosinophil count	EFO_0004842	Hematological measurement	Hematological
PGS002397	EFO_0004842	eosinophil count	eosinophil count	EFO_0004842	Hematological measurement	Hematological
PGS002446	EFO_0004842	eosinophil count	eosinophil count	EFO_0004842	Hematological measurement	Hematological
PGS002495	EFO_0004842	eosinophil count	eosinophil count	EFO_0004842	Hematological measurement	Hematological
PGS002544	EFO_0004842	eosinophil count	eosinophil count	EFO_0004842	Hematological measurement	Hematological
PGS002593	EFO_0004842	eosinophil count	eosinophil count	EFO_0004842	Hematological measurement	Hematological
PGS002642	EFO_0004842	eosinophil count	eosinophil count	EFO_0004842	Hematological measurement	Hematological
PGS000091	EFO_0007991	eosinophil percentage of leukocytes	eosinophil percentage of leukocytes	EFO_0007991	Hematological measurement	Hematological
PGS000166	EFO_0007991	eosinophil percentage of leukocytes	eosinophil percentage of leukocytes	EFO_0007991	Hematological measurement	Hematological
PGS001076	EFO_0007991	eosinophil percentage of leukocytes	eosinophil percentage of leukocytes	EFO_0007991	Hematological measurement	Hematological
PGS001949	EFO_0007991	eosinophil percentage of leukocytes	eosinophil percentage of leukocytes	EFO_0007991	Hematological measurement	Hematological
PGS002167	EFO_0007991	eosinophil percentage of leukocytes	eosinophil percentage of leukocytes	EFO_0007991	Hematological measurement	Hematological
PGS000092	EFO_0004348	hematocrit	hematocrit	EFO_0004348	Hematological measurement	Hematological
PGS000167	EFO_0004348	hematocrit	hematocrit	EFO_0004348	Hematological measurement	Hematological
PGS001225	EFO_0004348	hematocrit	hematocrit	EFO_0004348	Hematological measurement	Hematological
PGS001925	EFO_0004348	hematocrit	hematocrit	EFO_0004348	Hematological measurement	Hematological
PGS002141	EFO_0004348	hematocrit	hematocrit	EFO_0004348	Hematological measurement	Hematological
PGS000093	EFO_0004509	hemoglobin measurement	hemoglobin measurement	EFO_0004509	Hematological measurement	Hematological
PGS000168	EFO_0004509	hemoglobin measurement	hemoglobin measurement	EFO_0004509	Hematological measurement	Hematological
PGS001400	EFO_0004509	hemoglobin measurement	hemoglobin measurement	EFO_0004509	Hematological measurement	Hematological
PGS001926	EFO_0004509	hemoglobin measurement	hemoglobin measurement	EFO_0004509	Hematological measurement	Hematological

PGS002142	EFO_0004509	hemoglobin measurement	hemoglobin measurement	EFO_0004509	Hematological measurement	Hematological
PGS000094	EFO_0007986	reticulocyte count	reticulocyte count	EFO_0007986	Hematological measurement	Hematological
PGS000095	EFO_0007986	reticulocyte count	reticulocyte count	EFO_0007986	Hematological measurement	Hematological
PGS000096	EFO_0007986	reticulocyte count	reticulocyte count	EFO_0007986	Hematological measurement	Hematological
PGS000111	EFO_0007986	reticulocyte count	reticulocyte count	EFO_0007986	Hematological measurement	Hematological
PGS000112	EFO_0007986	reticulocyte count	reticulocyte count	EFO_0007986	Hematological measurement	Hematological
PGS000169	EFO_0007986	reticulocyte count	reticulocyte count	EFO_0007986	Hematological measurement	Hematological
PGS000170	EFO_0007986	reticulocyte count	reticulocyte count	EFO_0007986	Hematological measurement	Hematological
PGS000171	EFO_0007986	reticulocyte count	reticulocyte count	EFO_0007986	Hematological measurement	Hematological
PGS000189	EFO_0007986	reticulocyte count	reticulocyte count	EFO_0007986	Hematological measurement	Hematological
PGS000190	EFO_0007986	reticulocyte count	reticulocyte count	EFO_0007986	Hematological measurement	Hematological
PGS001528	EFO_0007986	reticulocyte count	reticulocyte count	EFO_0007986	Hematological measurement	Hematological
PGS001976	EFO_0007986	reticulocyte count	reticulocyte count	EFO_0007986	Hematological measurement	Hematological
PGS002194	EFO_0007986	reticulocyte count	reticulocyte count	EFO_0007986	Hematological measurement	Hematological
PGS002333	EFO_0007986	reticulocyte count	reticulocyte count	EFO_0007986	Hematological measurement	Hematological
PGS002405	EFO_0007986	reticulocyte count	reticulocyte count	EFO_0007986	Hematological measurement	Hematological
PGS002454	EFO_0007986	reticulocyte count	reticulocyte count	EFO_0007986	Hematological measurement	Hematological
PGS002503	EFO_0007986	reticulocyte count	reticulocyte count	EFO_0007986	Hematological measurement	Hematological
PGS002552	EFO_0007986	reticulocyte count	reticulocyte count	EFO_0007986	Hematological measurement	Hematological
PGS002601	EFO_0007986	reticulocyte count	reticulocyte count	EFO_0007986	Hematological measurement	Hematological
PGS000097	EFO_0004587	lymphocyte count	lymphocyte count	EFO_0004587	Hematological measurement	Hematological
PGS000172	EFO_0004587	lymphocyte count	lymphocyte count	EFO_0004587	Hematological measurement	Hematological
PGS001199	EFO_0004587	lymphocyte count	lymphocyte count	EFO_0004587	Hematological measurement	Hematological
PGS001965	EFO_0004587	lymphocyte count	lymphocyte count	EFO_0004587	Hematological measurement	Hematological
PGS002183	EFO_0004587	lymphocyte count	lymphocyte count	EFO_0004587	Hematological measurement	Hematological

PGS002338	EFO_0004587	lymphocyte count	lymphocyte count	EFO_0004587	Hematological measurement	Hematological
PGS002370	EFO_0004587	lymphocyte count	lymphocyte count	EFO_0004587	Hematological measurement	Hematological
PGS002410	EFO_0004587	lymphocyte count	lymphocyte count	EFO_0004587	Hematological measurement	Hematological
PGS002459	EFO_0004587	lymphocyte count	lymphocyte count	EFO_0004587	Hematological measurement	Hematological
PGS002508	EFO_0004587	lymphocyte count	lymphocyte count	EFO_0004587	Hematological measurement	Hematological
PGS002557	EFO_0004587	lymphocyte count	lymphocyte count	EFO_0004587	Hematological measurement	Hematological
PGS002606	EFO_0004587	lymphocyte count	lymphocyte count	EFO_0004587	Hematological measurement	Hematological
PGS002655	EFO_0004587	lymphocyte count	lymphocyte count	EFO_0004587	Hematological measurement	Hematological
PGS000098	EFO_0007993	lymphocyte percentage of leukocytes	lymphocyte percentage of leukocytes	EFO_0007993	Hematological measurement	Hematological
PGS000173	EFO_0007993	lymphocyte percentage of leukocytes	lymphocyte percentage of leukocytes	EFO_0007993	Hematological measurement	Hematological
PGS001414	EFO_0007993	lymphocyte percentage of leukocytes	lymphocyte percentage of leukocytes	EFO_0007993	Hematological measurement	Hematological
PGS001986	EFO_0007993	lymphocyte percentage of leukocytes	lymphocyte percentage of leukocytes	EFO_0007993	Hematological measurement	Hematological
PGS002203	EFO_0007993	lymphocyte percentage of leukocytes	lymphocyte percentage of leukocytes	EFO_0007993	Hematological measurement	Hematological
PGS000099	EFO_0004527	mean corpuscular hemoglobin	mean corpuscular hemoglobin	EFO_0004527	Hematological measurement	Hematological
PGS000174	EFO_0004527	mean corpuscular hemoglobin	mean corpuscular hemoglobin	EFO_0004527	Hematological measurement	Hematological
PGS001219	EFO_0004527	mean corpuscular hemoglobin	mean corpuscular hemoglobin	EFO_0004527	Hematological measurement	Hematological
PGS001989	EFO_0004527	mean corpuscular hemoglobin	mean corpuscular hemoglobin	EFO_0004527	Hematological measurement	Hematological
PGS002206	EFO_0004527	mean corpuscular hemoglobin	mean corpuscular hemoglobin	EFO_0004527	Hematological measurement	Hematological
PGS002339	EFO_0004527	mean corpuscular hemoglobin	mean corpuscular hemoglobin	EFO_0004527	Hematological measurement	Hematological
PGS002371	EFO_0004527	mean corpuscular hemoglobin	mean corpuscular hemoglobin	EFO_0004527	Hematological measurement	Hematological
PGS002411	EFO_0004527	mean corpuscular hemoglobin	mean corpuscular hemoglobin	EFO_0004527	Hematological measurement	Hematological
PGS002460	EFO_0004527	mean corpuscular hemoglobin	mean corpuscular hemoglobin	EFO_0004527	Hematological measurement	Hematological
PGS002509	EFO_0004527	mean corpuscular hemoglobin	mean corpuscular hemoglobin	EFO_0004527	Hematological measurement	Hematological
PGS002558	EFO_0004527	mean corpuscular hemoglobin	mean corpuscular hemoglobin	EFO_0004527	Hematological measurement	Hematological
PGS002607	EFO_0004527	mean corpuscular hemoglobin	mean corpuscular hemoglobin	EFO_0004527	Hematological measurement	Hematological

PGS002656	EFO_0004527	mean corpuscular hemoglobin	mean corpuscular hemoglobin	EFO_0004527	Hematological measurement	Hematological
PGS000100	EFO_0004528	mean corpuscular hemoglobin concentration	mean corpuscular hemoglobin concentration	EFO_0004528	Hematological measurement	Hematological
PGS000175	EFO_0004528	mean corpuscular hemoglobin concentration	mean corpuscular hemoglobin concentration	EFO_0004528	Hematological measurement	Hematological
PGS001218	EFO_0004528	mean corpuscular hemoglobin concentration	mean corpuscular hemoglobin concentration	EFO_0004528	Hematological measurement	Hematological
PGS000101	EFO_0004526	mean corpuscular volume	mean corpuscular volume	EFO_0004526	Hematological measurement	Hematological
PGS000176	EFO_0004526	mean corpuscular volume	mean corpuscular volume	EFO_0004526	Hematological measurement	Hematological
PGS000181	EFO_0004526	mean corpuscular volume	mean corpuscular volume	EFO_0004526	Hematological measurement	Hematological
PGS001220	EFO_0004526	mean corpuscular volume	mean corpuscular volume	EFO_0004526	Hematological measurement	Hematological
PGS001990	EFO_0004526	mean corpuscular volume	mean corpuscular volume	EFO_0004526	Hematological measurement	Hematological
PGS002207	EFO_0004526	mean corpuscular volume	mean corpuscular volume	EFO_0004526	Hematological measurement	Hematological
PGS000102	EFO_0005091	monocyte count	monocyte count	EFO_0005091	Hematological measurement	Hematological
PGS000177	EFO_0005091	monocyte count	monocyte count	EFO_0005091	Hematological measurement	Hematological
PGS001163	EFO_0005091	monocyte count	monocyte count	EFO_0005091	Hematological measurement	Hematological
PGS001968	EFO_0005091	monocyte count	monocyte count	EFO_0005091	Hematological measurement	Hematological
PGS002186	EFO_0005091	monocyte count	monocyte count	EFO_0005091	Hematological measurement	Hematological
PGS002341	EFO_0005091	monocyte count	monocyte count	EFO_0005091	Hematological measurement	Hematological
PGS002372	EFO_0005091	monocyte count	monocyte count	EFO_0005091	Hematological measurement	Hematological
PGS002413	EFO_0005091	monocyte count	monocyte count	EFO_0005091	Hematological measurement	Hematological
PGS002462	EFO_0005091	monocyte count	monocyte count	EFO_0005091	Hematological measurement	Hematological
PGS002560	EFO_0005091	monocyte count	monocyte count	EFO_0005091	Hematological measurement	Hematological
PGS002609	EFO_0005091	monocyte count	monocyte count	EFO_0005091	Hematological measurement	Hematological
PGS002658	EFO_0005091	monocyte count	monocyte count	EFO_0005091	Hematological measurement	Hematological
PGS000103	EFO_0007989	monocyte percentage of leukocytes	monocyte percentage of leukocytes	EFO_0007989	Hematological measurement	Hematological
PGS000178	EFO_0007989	monocyte percentage of leukocytes	monocyte percentage of leukocytes	EFO_0007989	Hematological measurement	Hematological
PGS001078	EFO_0007989	monocyte percentage of leukocytes	monocyte percentage of leukocytes	EFO_0007989	Hematological measurement	Hematological

PGS001991	EFO_0007989	monocyte percentage of leukocytes	monocyte percentage of leukocytes	EFO_0007989	Hematological measurement	Hematological
PGS002208	EFO_0007989	monocyte percentage of leukocytes	monocyte percentage of leukocytes	EFO_0007989	Hematological measurement	Hematological
PGS000104	EFO_0004584	mean platelet volume	mean platelet volume	EFO_0004584	Hematological measurement	Hematological
PGS000179	EFO_0004584	mean platelet volume	mean platelet volume	EFO_0004584	Hematological measurement	Hematological
PGS001200	EFO_0004584	mean platelet volume	mean platelet volume	EFO_0004584	Hematological measurement	Hematological
PGS001971	EFO_0004584	mean platelet volume	mean platelet volume	EFO_0004584	Hematological measurement	Hematological
PGS002189	EFO_0004584	mean platelet volume	mean platelet volume	EFO_0004584	Hematological measurement	Hematological
PGS002340	EFO_0004584	mean platelet volume	mean platelet volume	EFO_0004584	Hematological measurement	Hematological
PGS002412	EFO_0004584	mean platelet volume	mean platelet volume	EFO_0004584	Hematological measurement	Hematological
PGS002461	EFO_0004584	mean platelet volume	mean platelet volume	EFO_0004584	Hematological measurement	Hematological
PGS002559	EFO_0004584	mean platelet volume	mean platelet volume	EFO_0004584	Hematological measurement	Hematological
PGS002608	EFO_0004584	mean platelet volume	mean platelet volume	EFO_0004584	Hematological measurement	Hematological
PGS000105	EFO_0004833	neutrophil count	neutrophil count	EFO_0004833	Hematological measurement	Hematological
PGS000182	EFO_0004833	neutrophil count	neutrophil count	EFO_0004833	Hematological measurement	Hematological
PGS001173	EFO_0004833	neutrophil count	neutrophil count	EFO_0004833	Hematological measurement	Hematological
PGS001969	EFO_0004833	neutrophil count	neutrophil count	EFO_0004833	Hematological measurement	Hematological
PGS002187	EFO_0004833	neutrophil count	neutrophil count	EFO_0004833	Hematological measurement	Hematological
PGS000106	EFO_0007990	neutrophil percentage of leukocytes	neutrophil percentage of leukocytes	EFO_0007990	Hematological measurement	Hematological
PGS000183	EFO_0007990	neutrophil percentage of leukocytes	neutrophil percentage of leukocytes	EFO_0007990	Hematological measurement	Hematological
PGS001077	EFO_0007990	neutrophil percentage of leukocytes	neutrophil percentage of leukocytes	EFO_0007990	Hematological measurement	Hematological
PGS001997	EFO_0007990	neutrophil percentage of leukocytes	neutrophil percentage of leukocytes	EFO_0007990	Hematological measurement	Hematological
PGS002214	EFO_0007990	neutrophil percentage of leukocytes	neutrophil percentage of leukocytes	EFO_0007990	Hematological measurement	Hematological
PGS000107	EFO_0007985	platelet crit	platelet crit	EFO_0007985	Hematological measurement	Hematological
PGS000184	EFO_0007985	platelet crit	platelet crit	EFO_0007985	Hematological measurement	Hematological
PGS001517	EFO_0007985	platelet crit	platelet crit	EFO_0007985	Hematological measurement	Hematological

PGS001970	EFO_0007985	platelet crit	platelet crit	EFO_0007985	Hematological measurement	Hematological
PGS002188	EFO_0007985	platelet crit	platelet crit	EFO_0007985	Hematological measurement	Hematological
PGS000108	EFO_0007984	platelet component distribution width	platelet component distribution width	EFO_0007984	Hematological measurement	Hematological
PGS000185	EFO_0007984	platelet component distribution width	platelet component distribution width	EFO_0007984	Hematological measurement	Hematological
PGS001079	EFO_0007984	platelet component distribution width	platelet component distribution width	EFO_0007984	Hematological measurement	Hematological
PGS001972	EFO_0007984	platelet component distribution width	platelet component distribution width	EFO_0007984	Hematological measurement	Hematological
PGS002190	EFO_0007984	platelet component distribution width	platelet component distribution width	EFO_0007984	Hematological measurement	Hematological
PGS000110	EFO_0004305	erythrocyte count	erythrocyte count	EFO_0004305	Hematological measurement	Hematological
PGS000187	EFO_0004305	erythrocyte count	erythrocyte count	EFO_0004305	Hematological measurement	Hematological
PGS001240	EFO_0004305	erythrocyte count	erythrocyte count	EFO_0004305	Hematological measurement	Hematological
PGS001909	EFO_0004305	erythrocyte count	erythrocyte count	EFO_0004305	Hematological measurement	Hematological
PGS002123	EFO_0004305	erythrocyte count	erythrocyte count	EFO_0004305	Hematological measurement	Hematological
PGS002345	EFO_0004305	erythrocyte count	erythrocyte count	EFO_0004305	Hematological measurement	Hematological
PGS002374	EFO_0004305	erythrocyte count	erythrocyte count	EFO_0004305	Hematological measurement	Hematological
PGS002417	EFO_0004305	erythrocyte count	erythrocyte count	EFO_0004305	Hematological measurement	Hematological
PGS002466	EFO_0004305	erythrocyte count	erythrocyte count	EFO_0004305	Hematological measurement	Hematological
PGS002515	EFO_0004305	erythrocyte count	erythrocyte count	EFO_0004305	Hematological measurement	Hematological
PGS002564	EFO_0004305	erythrocyte count	erythrocyte count	EFO_0004305	Hematological measurement	Hematological
PGS002613	EFO_0004305	erythrocyte count	erythrocyte count	EFO_0004305	Hematological measurement	Hematological
PGS002662	EFO_0004305	erythrocyte count	erythrocyte count	EFO_0004305	Hematological measurement	Hematological
PGS000113	EFO_0004308	leukocyte count	leukocyte count	EFO_0004308	Hematological measurement	Hematological
PGS000191	EFO_0004308	leukocyte count	leukocyte count	EFO_0004308	Hematological measurement	Hematological
PGS001239	EFO_0004308	leukocyte count	leukocyte count	EFO_0004308	Hematological measurement	Hematological
PGS001962	EFO_0004308	leukocyte count	leukocyte count	EFO_0004308	Hematological measurement	Hematological
PGS002180	EFO_0004308	leukocyte count	leukocyte count	EFO_0004308	Hematological measurement	Hematological

PGS002357	EFO_0004308	leukocyte count	leukocyte count	EFO_0004308	Hematological measurement	Hematological
PGS002380	EFO_0004308	leukocyte count	leukocyte count	EFO_0004308	Hematological measurement	Hematological
PGS002429	EFO_0004308	leukocyte count	leukocyte count	EFO_0004308	Hematological measurement	Hematological
PGS002478	EFO_0004308	leukocyte count	leukocyte count	EFO_0004308	Hematological measurement	Hematological
PGS002527	EFO_0004308	leukocyte count	leukocyte count	EFO_0004308	Hematological measurement	Hematological
PGS002576	EFO_0004308	leukocyte count	leukocyte count	EFO_0004308	Hematological measurement	Hematological
PGS002625	EFO_0004308	leukocyte count	leukocyte count	EFO_0004308	Hematological measurement	Hematological
PGS000114	EFO_0002609	juvenile idiopathic arthritis	juvenile idiopathic arthritis	EFO_0002609	Immune system disorder	Immunological
PGS000117	EFO_0000319	cardiovascular disease	cardiovascular disease	EFO_0000319	Cardiovascular disease	Cardiovascular
PGS000863	EFO_0000319	cardiovascular disease	cardiovascular disease	EFO_0000319	Cardiovascular disease	Cardiovascular
PGS001335	EFO_0000319	cardiovascular disease	cardiovascular disease	EFO_0000319	Cardiovascular disease	Cardiovascular
PGS002316	EFO_0000319	cardiovascular disease	cardiovascular disease	EFO_0000319	Cardiovascular disease	Cardiovascular
PGS002361	EFO_0000319	cardiovascular disease	cardiovascular disease	EFO_0000319	Cardiovascular disease	Cardiovascular
PGS002388	EFO_0000319	cardiovascular disease	cardiovascular disease	EFO_0000319	Cardiovascular disease	Cardiovascular
PGS002437	EFO_0000319	cardiovascular disease	cardiovascular disease	EFO_0000319	Cardiovascular disease	Cardiovascular
PGS002486	EFO_0000319	cardiovascular disease	cardiovascular disease	EFO_0000319	Cardiovascular disease	Cardiovascular
PGS002535	EFO_0000319	cardiovascular disease	cardiovascular disease	EFO_0000319	Cardiovascular disease	Cardiovascular
PGS002584	EFO_0000319	cardiovascular disease	cardiovascular disease	EFO_0000319	Cardiovascular disease	Cardiovascular
PGS002633	EFO_0000319	cardiovascular disease	cardiovascular disease	EFO_0000319	Cardiovascular disease	Cardiovascular
PGS000124	EFO_0004695	intraocular pressure measurement	intraocular pressure measurement	EFO_0004695	Other measurement	Ophthalmological
PGS000879	EFO_0004695	intraocular pressure measurement	intraocular pressure measurement	EFO_0004695	Other measurement	Ophthalmological
PGS001185	EFO_0004695	intraocular pressure measurement	intraocular pressure measurement	EFO_0004695	Other measurement	Ophthalmological
PGS001409	EFO_0004695	intraocular pressure measurement	intraocular pressure measurement	EFO_0004695	Other measurement	Ophthalmological
PGS001410	EFO_0004695	intraocular pressure measurement	intraocular pressure measurement	EFO_0004695	Other measurement	Ophthalmological
PGS001411	EFO_0004695	intraocular pressure measurement	intraocular pressure measurement	EFO_0004695	Other measurement	Ophthalmological
PGS003413	EFO_0004695	intraocular pressure measurement	intraocular pressure measurement	EFO_0004695	Other measurement	Ophthalmological
PGS000126	EFO_0004531	urate measurement	urate measurement	EFO_0004531	Other measurement	Metabolic
PGS000700	EFO_0004531	urate measurement	urate measurement	EFO_0004531	Other measurement	Metabolic
PGS002010	EFO_0004531	urate measurement	urate measurement	EFO_0004531	Other measurement	Metabolic
PGS002229	EFO_0004531	urate measurement	urate measurement	EFO_0004531	Other measurement	Metabolic
PGS000127	EFO_0004541	HbA1c measurement	HbA1c measurement	EFO_0004541	Hematological measurement	Hematological
PGS000130	EFO_0004541	HbA1c measurement	HbA1c measurement	EFO_0004541	Hematological measurement	Hematological
PGS000304	EFO_0004541	HbA1c measurement	HbA1c measurement	EFO_0004541	Hematological measurement	Hematological
PGS000685	EFO_0004541	HbA1c measurement	HbA1c measurement	EFO_0004541	Hematological measurement	Hematological
PGS001352	EFO_0004541	HbA1c measurement	HbA1c measurement	EFO_0004541	Hematological measurement	Hematological
PGS001953	EFO_0004541	HbA1c measurement	HbA1c measurement	EFO_0004541	Hematological measurement	Hematological

PGS002171	EFO_0004541	HbA1c measurement	HbA1c measurement	EFO_0004541	Hematological measurement	Hematological
PGS002331	EFO_0004541	HbA1c measurement	HbA1c measurement	EFO_0004541	Hematological measurement	Hematological
PGS002367	EFO_0004541	HbA1c measurement	HbA1c measurement	EFO_0004541	Hematological measurement	Hematological
PGS002403	EFO_0004541	HbA1c measurement	HbA1c measurement	EFO_0004541	Hematological measurement	Hematological
PGS002452	EFO_0004541	HbA1c measurement	HbA1c measurement	EFO_0004541	Hematological measurement	Hematological
PGS002501	EFO_0004541	HbA1c measurement	HbA1c measurement	EFO_0004541	Hematological measurement	Hematological
PGS002550	EFO_0004541	HbA1c measurement	HbA1c measurement	EFO_0004541	Hematological measurement	Hematological
PGS002599	EFO_0004541	HbA1c measurement	HbA1c measurement	EFO_0004541	Hematological measurement	Hematological
PGS002648	EFO_0004541	HbA1c measurement	HbA1c measurement	EFO_0004541	Hematological measurement	Hematological
PGS000133	MONDO_0005090	schizophrenia	schizophrenia	MONDO_0005090	Neurological disorder	Neurological
PGS000134	MONDO_0005090	schizophrenia	schizophrenia	MONDO_0005090	Neurological disorder	Neurological
PGS000135	MONDO_0005090	schizophrenia	schizophrenia	MONDO_0005090	Neurological disorder	Neurological
PGS000136	MONDO_0005090	schizophrenia	schizophrenia	MONDO_0005090	Neurological disorder	Neurological
PGS002785	MONDO_0005090	schizophrenia	schizophrenia	MONDO_0005090	Neurological disorder	Neurological
PGS000137	MONDO_0005041	glaucoma	glaucoma	MONDO_0005041	Other disease	Ophthalmological
PGS001321	MONDO_0005041	glaucoma	glaucoma	MONDO_0005041	Other disease	Ophthalmological
PGS001322	MONDO_0005041	glaucoma	glaucoma	MONDO_0005041	Other disease	Ophthalmological
PGS001323	MONDO_0005041	glaucoma	glaucoma	MONDO_0005041	Other disease	Ophthalmological
PGS001792	MONDO_0005041	glaucoma	glaucoma	MONDO_0005041	Other disease	Ophthalmological
PGS001836	MONDO_0005041	glaucoma	glaucoma	MONDO_0005041	Other disease	Ophthalmological
PGS002043	MONDO_0005041	glaucoma	glaucoma	MONDO_0005041	Other disease	Ophthalmological
PGS002761	MONDO_0005041	glaucoma	glaucoma	MONDO_0005041	Other disease	Ophthalmological
PGS000138	MONDO_0002009	major depressive disorder	major depressive disorder	MONDO_0002009	Neurological disorder	Neurological
PGS000139	MONDO_0002009	major depressive disorder|recurrent	major depressive disorder	MONDO_0002009	Neurological disorder	Neurological
PGS000145	MONDO_0002009	major depressive disorder	major depressive disorder	MONDO_0002009	Neurological disorder	Neurological

PGS000193	MONDO_0002009	major depressive disorder	major depressive disorder	MONDO_0002009	Neurological disorder	Neurological
PGS002789	MONDO_0002009	major depressive disorder	major depressive disorder	MONDO_0002009	Neurological disorder	Neurological
PGS003333	MONDO_0002009	major depressive disorder	major depressive disorder	MONDO_0002009	Neurological disorder	Neurological
PGS000140	EFO_0009820	seeing a general practitioner for nerves, anxiety, tension or depression, self-reported|depressive disorder	seeing a general practitioner for nerves, anxiety, tension or depression, self-reported	EFO_0009820	Other trait	Psychiatric
PGS000142	EFO_0009820	seeing a general practitioner for nerves, anxiety, tension or depression, self-reported|seeing a psychiatrist for nerves, anxiety, tension or depression, self-reported|depressive disorder	seeing a general practitioner for nerves, anxiety, tension or depression, self-reported	EFO_0009820	Other trait	Psychiatric
PGS000143	EFO_0009820	seeing a general practitioner for nerves, anxiety, tension or depression, self-reported	seeing a general practitioner for nerves, anxiety, tension or depression, self-reported	EFO_0009820	Other trait	Psychiatric
PGS000141	EFO_0009821	seeing a psychiatrist for nerves, anxiety, tension or depression, self-reported|depressive disorder	seeing a psychiatrist for nerves, anxiety, tension or depression, self-reported	EFO_0009821	Other trait	Psychiatric
PGS000144	EFO_0009799	self-reported trait|depressive symptom measurement	self-reported trait	EFO_0009799	Other trait	Psychiatric
PGS001006	EFO_0009799	self-reported trait|body weight	self-reported trait	EFO_0009799	Other trait	Body measurement
PGS000180	EFO_0010701	mean reticulocyte volume	mean reticulocyte volume	EFO_0010701	Hematological measurement	Hematological
PGS000987	EFO_0010701	mean reticulocyte volume	mean reticulocyte volume	EFO_0010701	Hematological measurement	Hematological
PGS002003	EFO_0010701	mean reticulocyte volume	mean reticulocyte volume	EFO_0010701	Hematological measurement	Hematological
PGS002221	EFO_0010701	mean reticulocyte volume	mean reticulocyte volume	EFO_0010701	Hematological measurement	Hematological
PGS000186	EFO_0004309	platelet count	platelet count	EFO_0004309	Hematological measurement	Hematological
PGS001238	EFO_0004309	platelet count	platelet count	EFO_0004309	Hematological measurement	Hematological
PGS001973	EFO_0004309	platelet count	platelet count	EFO_0004309	Hematological measurement	Hematological
PGS002191	EFO_0004309	platelet count	platelet count	EFO_0004309	Hematological measurement	Hematological
PGS002343	EFO_0004309	platelet count	platelet count	EFO_0004309	Hematological measurement	Hematological
PGS002373	EFO_0004309	platelet count	platelet count	EFO_0004309	Hematological measurement	Hematological
PGS002415	EFO_0004309	platelet count	platelet count	EFO_0004309	Hematological measurement	Hematological
PGS002464	EFO_0004309	platelet count	platelet count	EFO_0004309	Hematological measurement	Hematological
PGS002513	EFO_0004309	platelet count	platelet count	EFO_0004309	Hematological measurement	Hematological
PGS002562	EFO_0004309	platelet count	platelet count	EFO_0004309	Hematological measurement	Hematological

PGS002611	EFO_0004309	platelet count	platelet count	EFO_0004309	Hematological measurement	Hematological
PGS002660	EFO_0004309	platelet count	platelet count	EFO_0004309	Hematological measurement	Hematological
PGS000188	EFO_0005192	red blood cell distribution width	red blood cell distribution width	EFO_0005192	Hematological measurement	Hematological
PGS001152	EFO_0005192	red blood cell distribution width	red blood cell distribution width	EFO_0005192	Hematological measurement	Hematological
PGS001908	EFO_0005192	red blood cell distribution width	red blood cell distribution width	EFO_0005192	Hematological measurement	Hematological
PGS002122	EFO_0005192	red blood cell distribution width	red blood cell distribution width	EFO_0005192	Hematological measurement	Hematological
PGS002346	EFO_0005192	red blood cell distribution width	red blood cell distribution width	EFO_0005192	Hematological measurement	Hematological
PGS002418	EFO_0005192	red blood cell distribution width	red blood cell distribution width	EFO_0005192	Hematological measurement	Hematological
PGS002467	EFO_0005192	red blood cell distribution width	red blood cell distribution width	EFO_0005192	Hematological measurement	Hematological
PGS002516	EFO_0005192	red blood cell distribution width	red blood cell distribution width	EFO_0005192	Hematological measurement	Hematological
PGS002565	EFO_0005192	red blood cell distribution width	red blood cell distribution width	EFO_0005192	Hematological measurement	Hematological
PGS002614	EFO_0005192	red blood cell distribution width	red blood cell distribution width	EFO_0005192	Hematological measurement	Hematological
PGS002663	EFO_0005192	red blood cell distribution width	red blood cell distribution width	EFO_0005192	Hematological measurement	Hematological
PGS000194	EFO_0009459	ACPA-positive rheumatoid arthritis	ACPA-positive rheumatoid arthritis	EFO_0009459	Immune system disorder	Immunological
PGS000195	EFO_0009460	ACPA-negative rheumatoid arthritis	ACPA-negative rheumatoid arthritis	EFO_0009460	Immune system disorder	Immunological
PGS000196	MONDO_0007915	systemic lupus erythematosus	systemic lupus erythematosus	MONDO_0007915	Immune system disorder	Immunological
PGS000754	MONDO_0007915	systemic lupus erythematosus	systemic lupus erythematosus	MONDO_0007915	Immune system disorder	Immunological
PGS000771	MONDO_0007915	systemic lupus erythematosus	systemic lupus erythematosus	MONDO_0007915	Immune system disorder	Immunological
PGS000772	MONDO_0007915	systemic lupus erythematosus	systemic lupus erythematosus	MONDO_0007915	Immune system disorder	Immunological
PGS000803	MONDO_0007915	systemic lupus erythematosus	systemic lupus erythematosus	MONDO_0007915	Immune system disorder	Immunological
PGS000197	EFO_0000706	spondyloarthropathy	spondyloarthropathy	EFO_0000706	Immune system disorder	Immunological
PGS000198	EFO_0003778	psoriatic arthritis	psoriatic arthritis	EFO_0003778	Immune system disorder	Immunological
PGS000342	EFO_0003778	psoriatic arthritis	psoriatic arthritis	EFO_0003778	Immune system disorder	Immunological
PGS001287	EFO_0003778	psoriatic arthritis	psoriatic arthritis	EFO_0003778	Immune system disorder	Immunological
PGS000199	EFO_0004274	gout	gout	EFO_0004274	Metabolic disorder	Metabolic
PGS000711	EFO_0004274	gout	gout	EFO_0004274	Metabolic disorder	Metabolic

<table>
<tr><td>PGS001248</td><td>EFO_0004274</td><td>gout</td><td>gout</td><td>EFO_0004274</td><td>Metabolic disorder</td><td>Metabolic</td></tr>
<tr><td>PGS001249</td><td>EFO_0004274</td><td>gout</td><td>gout</td><td>EFO_0004274</td><td>Metabolic disorder</td><td>Metabolic</td></tr>
<tr><td>PGS001789</td><td>EFO_0004274</td><td>gout</td><td>gout</td><td>EFO_0004274</td><td>Metabolic disorder</td><td>Metabolic</td></tr>
<tr><td>PGS001822</td><td>EFO_0004274</td><td>gout</td><td>gout</td><td>EFO_0004274</td><td>Metabolic disorder</td><td>Metabolic</td></tr>
<tr><td>PGS002030</td><td>EFO_0004274</td><td>gout</td><td>gout</td><td>EFO_0004274</td><td>Metabolic disorder</td><td>Metabolic</td></tr>
<tr><td>PGS002307</td><td>EFO_0004274</td><td>gout</td><td>gout</td><td>EFO_0004274</td><td>Metabolic disorder</td><td>Metabolic</td></tr>
<tr><td>PGS002762</td><td>EFO_0004274</td><td>gout</td><td>gout</td><td>EFO_0004274</td><td>Metabolic disorder</td><td>Metabolic</td></tr>
<tr><td>PGS003329</td><td>EFO_0004274</td><td>gout</td><td>gout</td><td>EFO_0004274</td><td>Metabolic disorder</td><td>Metabolic</td></tr>
<tr><td>PGS000201</td><td>EFO_0007835</td><td>alcohol dependence measurement</td><td>alcohol dependence measurement</td><td>EFO_0007835</td><td>Other measurement</td><td>Psychiatric</td></tr>
<tr><td>PGS000202</td><td>EFO_0007835</td><td>alcohol dependence measurement</td><td>alcohol dependence measurement</td><td>EFO_0007835</td><td>Other measurement</td><td>Psychiatric</td></tr>
<tr><td>PGS000203</td><td>EFO_0007878</td><td>alcohol consumption measurement</td><td>alcohol consumption measurement</td><td>EFO_0007878</td><td>Other measurement</td><td>Psychiatric</td></tr>
<tr><td>PGS000204</td><td>EFO_0007878</td><td>alcohol consumption measurement</td><td>alcohol consumption measurement</td><td>EFO_0007878</td><td>Other measurement</td><td>Psychiatric</td></tr>
<tr><td>PGS001085</td><td>EFO_0007878</td><td>alcohol consumption measurement|longitudinal
alcohol consumption measurement</td><td>alcohol consumption measurement</td><td>EFO_0007878</td><td>Other measurement</td><td>Psychiatric</td></tr>
<tr><td>PGS001086</td><td>EFO_0007878</td><td>alcohol consumption measurement</td><td>alcohol consumption measurement</td><td>EFO_0007878</td><td>Other measurement</td><td>Psychiatric</td></tr>
<tr><td>PGS001087</td><td>EFO_0007878</td><td>alcohol consumption measurement</td><td>alcohol consumption measurement</td><td>EFO_0007878</td><td>Other measurement</td><td>Psychiatric</td></tr>
<tr><td>PGS001088</td><td>EFO_0007878</td><td>alcohol consumption measurement</td><td>alcohol consumption measurement</td><td>EFO_0007878</td><td>Other measurement</td><td>Psychiatric</td></tr>
<tr><td>PGS001394</td><td>EFO_0007878</td><td>alcohol consumption measurement</td><td>alcohol consumption measurement</td><td>EFO_0007878</td><td>Other measurement</td><td>Psychiatric</td></tr>
<tr><td>PGS001934</td><td>EFO_0007878</td><td>alcohol consumption measurement</td><td>alcohol consumption measurement</td><td>EFO_0007878</td><td>Other measurement</td><td>Psychiatric</td></tr>
<tr><td>PGS002152</td><td>EFO_0007878</td><td>alcohol consumption measurement</td><td>alcohol consumption measurement</td><td>EFO_0007878</td><td>Other measurement</td><td>Psychiatric</td></tr>
<tr><td>PGS002752</td><td>EFO_0007878</td><td>alcohol consumption measurement</td><td>alcohol consumption measurement</td><td>EFO_0007878</td><td>Other measurement</td><td>Psychiatric</td></tr>
<tr><td>PGS002825</td><td>EFO_0007878</td><td>alcohol consumption measurement</td><td>alcohol consumption measurement</td><td>EFO_0007878</td><td>Other measurement</td><td>Psychiatric</td></tr>
<tr><td>PGS002826</td><td>EFO_0007878</td><td>alcohol consumption measurement</td><td>alcohol consumption measurement</td><td>EFO_0007878</td><td>Other measurement</td><td>Psychiatric</td></tr>
<tr><td>PGS002827</td><td>EFO_0007878</td><td>alcohol consumption measurement</td><td>alcohol consumption measurement</td><td>EFO_0007878</td><td>Other measurement</td><td>Psychiatric</td></tr>
<tr><td>PGS002828</td><td>EFO_0007878</td><td>alcohol consumption measurement</td><td>alcohol consumption measurement</td><td>EFO_0007878</td><td>Other measurement</td><td>Psychiatric</td></tr>
<tr><td>PGS002829</td><td>EFO_0007878</td><td>alcohol consumption measurement</td><td>alcohol consumption measurement</td><td>EFO_0007878</td><td>Other measurement</td><td>Psychiatric</td></tr>
<tr><td>PGS002830</td><td>EFO_0007878</td><td>alcohol consumption measurement</td><td>alcohol consumption measurement</td><td>EFO_0007878</td><td>Other measurement</td><td>Psychiatric</td></tr>
<tr><td>PGS002831</td><td>EFO_0007878</td><td>alcohol consumption measurement</td><td>alcohol consumption measurement</td><td>EFO_0007878</td><td>Other measurement</td><td>Psychiatric</td></tr>
<tr><td>PGS002832</td><td>EFO_0007878</td><td>alcohol consumption measurement</td><td>alcohol consumption measurement</td><td>EFO_0007878</td><td>Other measurement</td><td>Psychiatric</td></tr>
<tr><td>PGS002833</td><td>EFO_0007878</td><td>alcohol consumption measurement</td><td>alcohol consumption measurement</td><td>EFO_0007878</td><td>Other measurement</td><td>Psychiatric</td></tr>
<tr><td>PGS002834</td><td>EFO_0007878</td><td>alcohol consumption measurement</td><td>alcohol consumption measurement</td><td>EFO_0007878</td><td>Other measurement</td><td>Psychiatric</td></tr>
<tr><td>PGS002835</td><td>EFO_0007878</td><td>alcohol consumption measurement</td><td>alcohol consumption measurement</td><td>EFO_0007878</td><td>Other measurement</td><td>Psychiatric</td></tr>
<tr><td>PGS002836</td><td>EFO_0007878</td><td>alcohol consumption measurement</td><td>alcohol consumption measurement</td><td>EFO_0007878</td><td>Other measurement</td><td>Psychiatric</td></tr>
<tr><td>PGS002837</td><td>EFO_0007878</td><td>alcohol consumption measurement</td><td>alcohol consumption measurement</td><td>EFO_0007878</td><td>Other measurement</td><td>Psychiatric</td></tr>
<tr><td>PGS002838</td><td>EFO_0007878</td><td>alcohol consumption measurement</td><td>alcohol consumption measurement</td><td>EFO_0007878</td><td>Other measurement</td><td>Psychiatric</td></tr>
<tr><td>PGS002839</td><td>EFO_0007878</td><td>alcohol consumption measurement</td><td>alcohol consumption measurement</td><td>EFO_0007878</td><td>Other measurement</td><td>Psychiatric</td></tr>
<tr><td>PGS003376</td><td>EFO_0007878</td><td>alcohol consumption measurement</td><td>alcohol consumption measurement</td><td>EFO_0007878</td><td>Other measurement</td><td>Psychiatric</td></tr>
<tr><td>PGS000205</td><td>EFO_0008579</td><td>risk-taking behaviour</td><td>risk-taking behaviour</td><td>EFO_0008579</td><td>Biological process</td><td>Activities</td></tr>
<tr><td>PGS000206</td><td>EFO_0008579</td><td>risk-taking behaviour</td><td>risk-taking behaviour</td><td>EFO_0008579</td><td>Biological process</td><td>Activities</td></tr>
<tr><td>PGS001049</td><td>EFO_0008579</td><td>risk-taking behaviour</td><td>risk-taking behaviour</td><td>EFO_0008579</td><td>Biological process</td><td>Activities</td></tr>
<tr><td>PGS000210</td><td>EFO_0004713</td><td>FEV|FEC ratio</td><td>FEV|FEC ratio</td><td>EFO_0004713</td><td>Other measurement</td><td>Respiratory</td></tr>
<tr><td>PGS001180</td><td>EFO_0004713</td><td>FEV|FEC ratio</td><td>FEV|FEC ratio</td><td>EFO_0004713</td><td>Other measurement</td><td>Respiratory</td></tr>
<tr><td>PGS001801</td><td>EFO_0004713</td><td>FEV|FEC ratio</td><td>FEV|FEC ratio</td><td>EFO_0004713</td><td>Other measurement</td><td>Respiratory</td></tr>
<tr><td>PGS002326</td><td>EFO_0004713</td><td>FEV|FEC ratio</td><td>FEV|FEC ratio</td><td>EFO_0004713</td><td>Other measurement</td><td>Respiratory</td></tr>
<tr><td>PGS002398</td><td>EFO_0004713</td><td>FEV|FEC ratio</td><td>FEV|FEC ratio</td><td>EFO_0004713</td><td>Other measurement</td><td>Respiratory</td></tr>
<tr><td>PGS002447</td><td>EFO_0004713</td><td>FEV|FEC ratio</td><td>FEV|FEC ratio</td><td>EFO_0004713</td><td>Other measurement</td><td>Respiratory</td></tr>
<tr><td>PGS002496</td><td>EFO_0004713</td><td>FEV|FEC ratio</td><td>FEV|FEC ratio</td><td>EFO_0004713</td><td>Other measurement</td><td>Respiratory</td></tr>
<tr><td>PGS002545</td><td>EFO_0004713</td><td>FEV|FEC ratio</td><td>FEV|FEC ratio</td><td>EFO_0004713</td><td>Other measurement</td><td>Respiratory</td></tr>
<tr><td>PGS002594</td><td>EFO_0004713</td><td>FEV|FEC ratio</td><td>FEV|FEC ratio</td><td>EFO_0004713</td><td>Other measurement</td><td>Respiratory</td></tr>
</table>

PGS002643	EFO_0004713	FEV|FEC ratio	FEV|FEC ratio	EFO_0004713	Other measurement	Respiratory
PGS000212	MONDO_0021116	luminal A breast carcinoma	luminal A breast carcinoma	MONDO_0021116	Cancer	Neoplasms
PGS000213	EFO_0009780	HER2 negative breast carcinoma	HER2 negative breast carcinoma	EFO_0009780	Cancer	Neoplasms
PGS000214	MONDO_0021115	luminal B breast carcinoma	luminal B breast carcinoma	MONDO_0021115	Cancer	Neoplasms
PGS000215	EFO_1000294	HER2 Positive Breast Carcinoma	HER2 Positive Breast Carcinoma	EFO_1000294	Cancer	Neoplasms
PGS000216	EFO_0005537	triple-negative breast cancer	triple-negative breast cancer	EFO_0005537	Cancer	Neoplasms
PGS000217	EFO_0007937	blood protein measurement	blood protein measurement	EFO_0007937	Other measurement	Hematological
PGS000218	EFO_0007937	blood protein measurement	blood protein measurement	EFO_0007937	Other measurement	Hematological
PGS000219	EFO_0007937	blood protein measurement	blood protein measurement	EFO_0007937	Other measurement	Hematological
PGS000220	EFO_0007937	blood protein measurement	blood protein measurement	EFO_0007937	Other measurement	Hematological
PGS000221	EFO_0007937	blood protein measurement	blood protein measurement	EFO_0007937	Other measurement	Hematological
PGS000222	EFO_0007937	blood protein measurement	blood protein measurement	EFO_0007937	Other measurement	Hematological
PGS000223	EFO_0007937	blood protein measurement	blood protein measurement	EFO_0007937	Other measurement	Hematological
PGS000224	EFO_0007937	blood protein measurement	blood protein measurement	EFO_0007937	Other measurement	Hematological
PGS000225	EFO_0007937	blood protein measurement	blood protein measurement	EFO_0007937	Other measurement	Hematological
PGS000226	EFO_0007937	blood protein measurement	blood protein measurement	EFO_0007937	Other measurement	Hematological
PGS000227	EFO_0007937	blood protein measurement	blood protein measurement	EFO_0007937	Other measurement	Hematological
PGS000228	EFO_0007937	blood protein measurement	blood protein measurement	EFO_0007937	Other measurement	Hematological
PGS000229	EFO_0007937	blood protein measurement	blood protein measurement	EFO_0007937	Other measurement	Hematological
PGS000230	EFO_0007937	blood protein measurement	blood protein measurement	EFO_0007937	Other measurement	Hematological
PGS000231	EFO_0007937	blood protein measurement	blood protein measurement	EFO_0007937	Other measurement	Hematological
PGS000232	EFO_0007937	blood protein measurement	blood protein measurement	EFO_0007937	Other measurement	Hematological
PGS000233	EFO_0007937	blood protein measurement	blood protein measurement	EFO_0007937	Other measurement	Hematological
PGS000234	EFO_0007937	blood protein measurement	blood protein measurement	EFO_0007937	Other measurement	Hematological
PGS000235	EFO_0007937	blood protein measurement	blood protein measurement	EFO_0007937	Other measurement	Hematological
PGS000236	EFO_0007937	blood protein measurement	blood protein measurement	EFO_0007937	Other measurement	Hematological
PGS000237	EFO_0007937	blood protein measurement	blood protein measurement	EFO_0007937	Other measurement	Hematological
PGS000238	EFO_0007937	blood protein measurement	blood protein measurement	EFO_0007937	Other measurement	Hematological
PGS000239	EFO_0007937	blood protein measurement	blood protein measurement	EFO_0007937	Other measurement	Hematological
PGS000240	EFO_0007937	blood protein measurement	blood protein measurement	EFO_0007937	Other measurement	Hematological
PGS000241	EFO_0007937	blood protein measurement	blood protein measurement	EFO_0007937	Other measurement	Hematological
PGS000242	EFO_0007937	blood protein measurement	blood protein measurement	EFO_0007937	Other measurement	Hematological
PGS000243	EFO_0007937	blood protein measurement	blood protein measurement	EFO_0007937	Other measurement	Hematological
PGS000244	EFO_0007937	blood protein measurement	blood protein measurement	EFO_0007937	Other measurement	Hematological
PGS000245	EFO_0007937	blood protein measurement	blood protein measurement	EFO_0007937	Other measurement	Hematological
PGS000246	EFO_0007937	blood protein measurement	blood protein measurement	EFO_0007937	Other measurement	Hematological
PGS000247	EFO_0007937	blood protein measurement	blood protein measurement	EFO_0007937	Other measurement	Hematological
PGS000248	EFO_0007937	blood protein measurement	blood protein measurement	EFO_0007937	Other measurement	Hematological
PGS000249	EFO_0007937	blood protein measurement	blood protein measurement	EFO_0007937	Other measurement	Hematological
PGS000250	EFO_0007937	blood protein measurement	blood protein measurement	EFO_0007937	Other measurement	Hematological
PGS000251	EFO_0007937	blood protein measurement	blood protein measurement	EFO_0007937	Other measurement	Hematological
PGS000252	EFO_0007937	blood protein measurement	blood protein measurement	EFO_0007937	Other measurement	Hematological
PGS000253	EFO_0007937	blood protein measurement	blood protein measurement	EFO_0007937	Other measurement	Hematological
PGS000254	EFO_0007937	blood protein measurement	blood protein measurement	EFO_0007937	Other measurement	Hematological
PGS000255	EFO_0007937	blood protein measurement	blood protein measurement	EFO_0007937	Other measurement	Hematological
PGS000256	EFO_0007937	blood protein measurement	blood protein measurement	EFO_0007937	Other measurement	Hematological
PGS000257	EFO_0007937	blood protein measurement	blood protein measurement	EFO_0007937	Other measurement	Hematological
PGS000258	EFO_0007937	blood protein measurement	blood protein measurement	EFO_0007937	Other measurement	Hematological

PGS000259	EFO_0007937	blood protein measurement	blood protein measurement	EFO_0007937	Other measurement	Hematological
PGS000260	EFO_0007937	blood protein measurement	blood protein measurement	EFO_0007937	Other measurement	Hematological
PGS000261	EFO_0007937	blood protein measurement	blood protein measurement	EFO_0007937	Other measurement	Hematological
PGS000262	EFO_0007937	blood protein measurement	blood protein measurement	EFO_0007937	Other measurement	Hematological
PGS000263	EFO_0007937	blood protein measurement	blood protein measurement	EFO_0007937	Other measurement	Hematological
PGS000264	EFO_0007937	blood protein measurement	blood protein measurement	EFO_0007937	Other measurement	Hematological
PGS000265	EFO_0007937	blood protein measurement	blood protein measurement	EFO_0007937	Other measurement	Hematological
PGS000266	EFO_0007937	blood protein measurement	blood protein measurement	EFO_0007937	Other measurement	Hematological
PGS000267	EFO_0007937	blood protein measurement	blood protein measurement	EFO_0007937	Other measurement	Hematological
PGS000268	EFO_0007937	blood protein measurement	blood protein measurement	EFO_0007937	Other measurement	Hematological
PGS000269	EFO_0007937	blood protein measurement	blood protein measurement	EFO_0007937	Other measurement	Hematological
PGS000270	EFO_0007937	blood protein measurement	blood protein measurement	EFO_0007937	Other measurement	Hematological
PGS000271	EFO_0007937	blood protein measurement	blood protein measurement	EFO_0007937	Other measurement	Hematological
PGS000272	EFO_0007937	blood protein measurement	blood protein measurement	EFO_0007937	Other measurement	Hematological
PGS000273	EFO_0007937	blood protein measurement	blood protein measurement	EFO_0007937	Other measurement	Hematological
PGS000274	EFO_0007937	blood protein measurement	blood protein measurement	EFO_0007937	Other measurement	Hematological
PGS000275	EFO_0007937	blood protein measurement	blood protein measurement	EFO_0007937	Other measurement	Hematological
PGS000276	EFO_0007937	blood protein measurement	blood protein measurement	EFO_0007937	Other measurement	Hematological
PGS000277	EFO_0007937	blood protein measurement	blood protein measurement	EFO_0007937	Other measurement	Hematological
PGS000278	EFO_0007937	blood protein measurement	blood protein measurement	EFO_0007937	Other measurement	Hematological
PGS000279	EFO_0007937	blood protein measurement	blood protein measurement	EFO_0007937	Other measurement	Hematological
PGS000280	EFO_0007937	blood protein measurement	blood protein measurement	EFO_0007937	Other measurement	Hematological
PGS000281	EFO_0007937	blood protein measurement	blood protein measurement	EFO_0007937	Other measurement	Hematological
PGS000282	EFO_0007937	blood protein measurement	blood protein measurement	EFO_0007937	Other measurement	Hematological
PGS000283	EFO_0007937	blood protein measurement	blood protein measurement	EFO_0007937	Other measurement	Hematological
PGS000284	EFO_0007937	blood protein measurement	blood protein measurement	EFO_0007937	Other measurement	Hematological
PGS000285	EFO_0007937	blood protein measurement	blood protein measurement	EFO_0007937	Other measurement	Hematological
PGS000286	EFO_0007937	blood protein measurement	blood protein measurement	EFO_0007937	Other measurement	Hematological
PGS000287	EFO_0007937	blood protein measurement	blood protein measurement	EFO_0007937	Other measurement	Hematological
PGS000288	EFO_0007937	blood protein measurement	blood protein measurement	EFO_0007937	Other measurement	Hematological
PGS000289	EFO_0007937	blood protein measurement	blood protein measurement	EFO_0007937	Other measurement	Hematological
PGS000290	EFO_0007937	blood protein measurement	blood protein measurement	EFO_0007937	Other measurement	Hematological
PGS000291	EFO_0007937	blood protein measurement	blood protein measurement	EFO_0007937	Other measurement	Hematological
PGS000292	EFO_0007937	blood protein measurement	blood protein measurement	EFO_0007937	Other measurement	Hematological
PGS000293	EFO_0007937	blood protein measurement	blood protein measurement	EFO_0007937	Other measurement	Hematological
PGS000294	EFO_0007937	blood protein measurement	blood protein measurement	EFO_0007937	Other measurement	Hematological
PGS000295	EFO_0007937	blood protein measurement	blood protein measurement	EFO_0007937	Other measurement	Hematological
PGS001081	EFO_0007937	blood protein measurement	blood protein measurement	EFO_0007937	Other measurement	Hematological
PGS001082	EFO_0007937	blood protein measurement	blood protein measurement	EFO_0007937	Other measurement	Hematological
PGS001407	EFO_0007937	blood protein measurement	blood protein measurement	EFO_0007937	Other measurement	Hematological
PGS000297	EFO_0004339	body height	body height	EFO_0004339	Body measurement	Body measurement
PGS000758	EFO_0004339	body height	body height	EFO_0004339	Body measurement	Body measurement
PGS001229	EFO_0004339	body height	body height	EFO_0004339	Body measurement	Body measurement
PGS001405	EFO_0004339	body height	body height	EFO_0004339	Body measurement	Body measurement
PGS001929	EFO_0004339	body height	body height	EFO_0004339	Body measurement	Body measurement
PGS002146	EFO_0004339	body height	body height	EFO_0004339	Body measurement	Body measurement
PGS002332	EFO_0004339	body height	body height	EFO_0004339	Body measurement	Body measurement
PGS002368	EFO_0004339	body height	body height	EFO_0004339	Body measurement	Body measurement
PGS002404	EFO_0004339	body height	body height	EFO_0004339	Body measurement	Body measurement
PGS002453	EFO_0004339	body height	body height	EFO_0004339	Body measurement	Body measurement

PGS002502	EFO_0004339	body height	body height	EFO_0004339	Body measurement	Body measurement
PGS002551	EFO_0004339	body height	body height	EFO_0004339	Body measurement	Body measurement
PGS002600	EFO_0004339	body height	body height	EFO_0004339	Body measurement	Body measurement
PGS002802	EFO_0004339	body height	body height	EFO_0004339	Body measurement	Body measurement
PGS002964	EFO_0004339	body height	body height	EFO_0004339	Body measurement	Body measurement
PGS002965	EFO_0004339	body height	body height	EFO_0004339	Body measurement	Body measurement
PGS002966	EFO_0004339	body height	body height	EFO_0004339	Body measurement	Body measurement
PGS002967	EFO_0004339	body height	body height	EFO_0004339	Body measurement	Body measurement
PGS002968	EFO_0004339	body height	body height	EFO_0004339	Body measurement	Body measurement
PGS002969	EFO_0004339	body height	body height	EFO_0004339	Body measurement	Body measurement
PGS002970	EFO_0004339	body height	body height	EFO_0004339	Body measurement	Body measurement
PGS002971	EFO_0004339	body height	body height	EFO_0004339	Body measurement	Body measurement
PGS002972	EFO_0004339	body height	body height	EFO_0004339	Body measurement	Body measurement
PGS002973	EFO_0004339	body height	body height	EFO_0004339	Body measurement	Body measurement
PGS002974	EFO_0004339	body height	body height	EFO_0004339	Body measurement	Body measurement
PGS002975	EFO_0004339	body height	body height	EFO_0004339	Body measurement	Body measurement
PGS002976	EFO_0004339	body height	body height	EFO_0004339	Body measurement	Body measurement
PGS002977	EFO_0004339	body height	body height	EFO_0004339	Body measurement	Body measurement
PGS002978	EFO_0004339	body height	body height	EFO_0004339	Body measurement	Body measurement
PGS002979	EFO_0004339	body height	body height	EFO_0004339	Body measurement	Body measurement
PGS002980	EFO_0004339	body height	body height	EFO_0004339	Body measurement	Body measurement
PGS002981	EFO_0004339	body height	body height	EFO_0004339	Body measurement	Body measurement
PGS002982	EFO_0004339	body height	body height	EFO_0004339	Body measurement	Body measurement
PGS002983	EFO_0004339	body height	body height	EFO_0004339	Body measurement	Body measurement
PGS002984	EFO_0004339	body height	body height	EFO_0004339	Body measurement	Body measurement
PGS002985	EFO_0004339	body height	body height	EFO_0004339	Body measurement	Body measurement
PGS002986	EFO_0004339	body height	body height	EFO_0004339	Body measurement	Body measurement
PGS002987	EFO_0004339	body height	body height	EFO_0004339	Body measurement	Body measurement
PGS002988	EFO_0004339	body height	body height	EFO_0004339	Body measurement	Body measurement
PGS002989	EFO_0004339	body height	body height	EFO_0004339	Body measurement	Body measurement
PGS002990	EFO_0004339	body height	body height	EFO_0004339	Body measurement	Body measurement
PGS002991	EFO_0004339	body height	body height	EFO_0004339	Body measurement	Body measurement
PGS002992	EFO_0004339	body height	body height	EFO_0004339	Body measurement	Body measurement
PGS002993	EFO_0004339	body height	body height	EFO_0004339	Body measurement	Body measurement
PGS000299	EFO_0007788	BMI-adjusted waist-hip ratio	BMI-adjusted waist-hip ratio	EFO_0007788	Body measurement	Metabolic
PGS000843	EFO_0007788	BMI-adjusted waist-hip ratio	BMI-adjusted waist-hip ratio	EFO_0007788	Body measurement	Metabolic
PGS000300	EFO_0004351	resting heart rate	resting heart rate	EFO_0004351	Cardiovascular measurement	Cardiovascular
PGS000301	EFO_0006335	systolic blood pressure	systolic blood pressure	EFO_0006335	Other measurement	Cardiovascular
PGS000900	EFO_0006335	systolic blood pressure|female	systolic blood pressure	EFO_0006335	Other measurement	Cardiovascular
PGS000901	EFO_0006335	systolic blood pressure|male	systolic blood pressure	EFO_0006335	Other measurement	Cardiovascular
PGS000913	EFO_0006335	systolic blood pressure	systolic blood pressure	EFO_0006335	Other measurement	Cardiovascular
PGS001134	EFO_0006335	systolic blood pressure	systolic blood pressure	EFO_0006335	Other measurement	Cardiovascular
PGS002009	EFO_0006335	systolic blood pressure	systolic blood pressure	EFO_0006335	Other measurement	Cardiovascular
PGS002228	EFO_0006335	systolic blood pressure	systolic blood pressure	EFO_0006335	Other measurement	Cardiovascular
PGS002238	EFO_0006335	systolic blood pressure	systolic blood pressure	EFO_0006335	Other measurement	Cardiovascular
PGS002257	EFO_0006335	systolic blood pressure	systolic blood pressure	EFO_0006335	Other measurement	Cardiovascular
PGS002275	EFO_0006335	systolic blood pressure	systolic blood pressure	EFO_0006335	Other measurement	Cardiovascular
PGS002349	EFO_0006335	systolic blood pressure	systolic blood pressure	EFO_0006335	Other measurement	Cardiovascular
PGS002376	EFO_0006335	systolic blood pressure	systolic blood pressure	EFO_0006335	Other measurement	Cardiovascular

PGS002421	EFO_0006335	systolic blood pressure	systolic blood pressure	EFO_0006335	Other measurement	Cardiovascular
PGS002470	EFO_0006335	systolic blood pressure	systolic blood pressure	EFO_0006335	Other measurement	Cardiovascular
PGS002568	EFO_0006335	systolic blood pressure	systolic blood pressure	EFO_0006335	Other measurement	Cardiovascular
PGS002617	EFO_0006335	systolic blood pressure	systolic blood pressure	EFO_0006335	Other measurement	Cardiovascular
PGS002734	EFO_0006335	systolic blood pressure	systolic blood pressure	EFO_0006335	Other measurement	Cardiovascular
PGS002807	EFO_0006335	systolic blood pressure	systolic blood pressure	EFO_0006335	Other measurement	Cardiovascular
PGS003069	EFO_0006335	systolic blood pressure	systolic blood pressure	EFO_0006335	Other measurement	Cardiovascular
PGS003070	EFO_0006335	systolic blood pressure	systolic blood pressure	EFO_0006335	Other measurement	Cardiovascular
PGS003071	EFO_0006335	systolic blood pressure	systolic blood pressure	EFO_0006335	Other measurement	Cardiovascular
PGS003072	EFO_0006335	systolic blood pressure	systolic blood pressure	EFO_0006335	Other measurement	Cardiovascular
PGS003073	EFO_0006335	systolic blood pressure	systolic blood pressure	EFO_0006335	Other measurement	Cardiovascular
PGS003074	EFO_0006335	systolic blood pressure	systolic blood pressure	EFO_0006335	Other measurement	Cardiovascular
PGS003075	EFO_0006335	systolic blood pressure	systolic blood pressure	EFO_0006335	Other measurement	Cardiovascular
PGS003076	EFO_0006335	systolic blood pressure	systolic blood pressure	EFO_0006335	Other measurement	Cardiovascular
PGS003077	EFO_0006335	systolic blood pressure	systolic blood pressure	EFO_0006335	Other measurement	Cardiovascular
PGS003078	EFO_0006335	systolic blood pressure	systolic blood pressure	EFO_0006335	Other measurement	Cardiovascular
PGS003079	EFO_0006335	systolic blood pressure	systolic blood pressure	EFO_0006335	Other measurement	Cardiovascular
PGS003080	EFO_0006335	systolic blood pressure	systolic blood pressure	EFO_0006335	Other measurement	Cardiovascular
PGS003081	EFO_0006335	systolic blood pressure	systolic blood pressure	EFO_0006335	Other measurement	Cardiovascular
PGS003082	EFO_0006335	systolic blood pressure	systolic blood pressure	EFO_0006335	Other measurement	Cardiovascular
PGS003083	EFO_0006335	systolic blood pressure	systolic blood pressure	EFO_0006335	Other measurement	Cardiovascular
PGS003084	EFO_0006335	systolic blood pressure	systolic blood pressure	EFO_0006335	Other measurement	Cardiovascular
PGS003085	EFO_0006335	systolic blood pressure	systolic blood pressure	EFO_0006335	Other measurement	Cardiovascular
PGS003086	EFO_0006335	systolic blood pressure	systolic blood pressure	EFO_0006335	Other measurement	Cardiovascular
PGS003087	EFO_0006335	systolic blood pressure	systolic blood pressure	EFO_0006335	Other measurement	Cardiovascular
PGS003088	EFO_0006335	systolic blood pressure	systolic blood pressure	EFO_0006335	Other measurement	Cardiovascular
PGS000302	EFO_0006336	diastolic blood pressure	diastolic blood pressure	EFO_0006336	Other measurement	Cardiovascular
PGS000912	EFO_0006336	diastolic blood pressure	diastolic blood pressure	EFO_0006336	Other measurement	Cardiovascular
PGS001133	EFO_0006336	diastolic blood pressure	diastolic blood pressure	EFO_0006336	Other measurement	Cardiovascular
PGS001900	EFO_0006336	diastolic blood pressure	diastolic blood pressure	EFO_0006336	Other measurement	Cardiovascular
PGS002114	EFO_0006336	diastolic blood pressure	diastolic blood pressure	EFO_0006336	Other measurement	Cardiovascular
PGS002239	EFO_0006336	diastolic blood pressure	diastolic blood pressure	EFO_0006336	Other measurement	Cardiovascular
PGS002258	EFO_0006336	diastolic blood pressure	diastolic blood pressure	EFO_0006336	Other measurement	Cardiovascular
PGS002322	EFO_0006336	diastolic blood pressure	diastolic blood pressure	EFO_0006336	Other measurement	Cardiovascular
PGS002362	EFO_0006336	diastolic blood pressure	diastolic blood pressure	EFO_0006336	Other measurement	Cardiovascular
PGS002394	EFO_0006336	diastolic blood pressure	diastolic blood pressure	EFO_0006336	Other measurement	Cardiovascular
PGS002443	EFO_0006336	diastolic blood pressure	diastolic blood pressure	EFO_0006336	Other measurement	Cardiovascular
PGS002541	EFO_0006336	diastolic blood pressure	diastolic blood pressure	EFO_0006336	Other measurement	Cardiovascular
PGS002590	EFO_0006336	diastolic blood pressure	diastolic blood pressure	EFO_0006336	Other measurement	Cardiovascular
PGS002889	EFO_0006336	diastolic blood pressure	diastolic blood pressure	EFO_0006336	Other measurement	Cardiovascular
PGS002890	EFO_0006336	diastolic blood pressure	diastolic blood pressure	EFO_0006336	Other measurement	Cardiovascular
PGS002891	EFO_0006336	diastolic blood pressure	diastolic blood pressure	EFO_0006336	Other measurement	Cardiovascular
PGS002892	EFO_0006336	diastolic blood pressure	diastolic blood pressure	EFO_0006336	Other measurement	Cardiovascular
PGS002893	EFO_0006336	diastolic blood pressure	diastolic blood pressure	EFO_0006336	Other measurement	Cardiovascular
PGS002894	EFO_0006336	diastolic blood pressure	diastolic blood pressure	EFO_0006336	Other measurement	Cardiovascular
PGS002895	EFO_0006336	diastolic blood pressure	diastolic blood pressure	EFO_0006336	Other measurement	Cardiovascular
PGS002896	EFO_0006336	diastolic blood pressure	diastolic blood pressure	EFO_0006336	Other measurement	Cardiovascular
PGS002897	EFO_0006336	diastolic blood pressure	diastolic blood pressure	EFO_0006336	Other measurement	Cardiovascular
PGS002898	EFO_0006336	diastolic blood pressure	diastolic blood pressure	EFO_0006336	Other measurement	Cardiovascular
PGS002899	EFO_0006336	diastolic blood pressure	diastolic blood pressure	EFO_0006336	Other measurement	Cardiovascular

PGS002900	EFO_0006336	diastolic blood pressure	diastolic blood pressure	EFO_0006336	Other measurement	Cardiovascular
PGS002901	EFO_0006336	diastolic blood pressure	diastolic blood pressure	EFO_0006336	Other measurement	Cardiovascular
PGS002902	EFO_0006336	diastolic blood pressure	diastolic blood pressure	EFO_0006336	Other measurement	Cardiovascular
PGS002903	EFO_0006336	diastolic blood pressure	diastolic blood pressure	EFO_0006336	Other measurement	Cardiovascular
PGS002904	EFO_0006336	diastolic blood pressure	diastolic blood pressure	EFO_0006336	Other measurement	Cardiovascular
PGS002905	EFO_0006336	diastolic blood pressure	diastolic blood pressure	EFO_0006336	Other measurement	Cardiovascular
PGS002906	EFO_0006336	diastolic blood pressure	diastolic blood pressure	EFO_0006336	Other measurement	Cardiovascular
PGS002907	EFO_0006336	diastolic blood pressure	diastolic blood pressure	EFO_0006336	Other measurement	Cardiovascular
PGS002908	EFO_0006336	diastolic blood pressure	diastolic blood pressure	EFO_0006336	Other measurement	Cardiovascular
PGS000303	EFO_0005208	glomerular filtration rate	glomerular filtration rate	EFO_0005208	Other measurement	Genitourinary
PGS000682	EFO_0005208	glomerular filtration rate	glomerular filtration rate	EFO_0005208	Other measurement	Genitourinary
PGS000860	EFO_0005208	glomerular filtration rate	glomerular filtration rate	EFO_0005208	Other measurement	Genitourinary
PGS000883	EFO_0005208	glomerular filtration rate	glomerular filtration rate	EFO_0005208	Other measurement	Genitourinary
PGS000884	EFO_0005208	glomerular filtration rate	glomerular filtration rate	EFO_0005208	Other measurement	Genitourinary
PGS000885	EFO_0005208	glomerular filtration rate	glomerular filtration rate	EFO_0005208	Other measurement	Genitourinary
PGS002810	EFO_0005208	glomerular filtration rate	glomerular filtration rate	EFO_0005208	Other measurement	Genitourinary
PGS003219	EFO_0005208	glomerular filtration rate	glomerular filtration rate	EFO_0005208	Other measurement	Genitourinary
PGS003220	EFO_0005208	glomerular filtration rate	glomerular filtration rate	EFO_0005208	Other measurement	Genitourinary
PGS003221	EFO_0005208	glomerular filtration rate	glomerular filtration rate	EFO_0005208	Other measurement	Genitourinary
PGS003222	EFO_0005208	glomerular filtration rate	glomerular filtration rate	EFO_0005208	Other measurement	Genitourinary
PGS003223	EFO_0005208	glomerular filtration rate	glomerular filtration rate	EFO_0005208	Other measurement	Genitourinary
PGS003224	EFO_0005208	glomerular filtration rate	glomerular filtration rate	EFO_0005208	Other measurement	Genitourinary
PGS003225	EFO_0005208	glomerular filtration rate	glomerular filtration rate	EFO_0005208	Other measurement	Genitourinary
PGS003226	EFO_0005208	glomerular filtration rate	glomerular filtration rate	EFO_0005208	Other measurement	Genitourinary
PGS003227	EFO_0005208	glomerular filtration rate	glomerular filtration rate	EFO_0005208	Other measurement	Genitourinary
PGS003228	EFO_0005208	glomerular filtration rate	glomerular filtration rate	EFO_0005208	Other measurement	Genitourinary
PGS003377	EFO_0005208	glomerular filtration rate	glomerular filtration rate	EFO_0005208	Other measurement	Genitourinary
PGS000305	EFO_0004465	fasting blood glucose measurement	fasting blood glucose measurement	EFO_0004465	Other measurement	Metabolic
PGS000838	EFO_0004465	fasting blood glucose measurement	fasting blood glucose measurement	EFO_0004465	Other measurement	Metabolic
PGS001350	EFO_0004465	fasting blood glucose measurement	fasting blood glucose measurement	EFO_0004465	Other measurement	Metabolic
PGS002924	EFO_0004465	fasting blood glucose measurement	fasting blood glucose measurement	EFO_0004465	Other measurement	Metabolic
PGS002925	EFO_0004465	fasting blood glucose measurement	fasting blood glucose measurement	EFO_0004465	Other measurement	Metabolic
PGS002926	EFO_0004465	fasting blood glucose measurement	fasting blood glucose measurement	EFO_0004465	Other measurement	Metabolic
PGS002927	EFO_0004465	fasting blood glucose measurement	fasting blood glucose measurement	EFO_0004465	Other measurement	Metabolic
PGS002928	EFO_0004465	fasting blood glucose measurement	fasting blood glucose measurement	EFO_0004465	Other measurement	Metabolic
PGS002929	EFO_0004465	fasting blood glucose measurement	fasting blood glucose measurement	EFO_0004465	Other measurement	Metabolic
PGS002930	EFO_0004465	fasting blood glucose measurement	fasting blood glucose measurement	EFO_0004465	Other measurement	Metabolic
PGS002931	EFO_0004465	fasting blood glucose measurement	fasting blood glucose measurement	EFO_0004465	Other measurement	Metabolic
PGS002932	EFO_0004465	fasting blood glucose measurement	fasting blood glucose measurement	EFO_0004465	Other measurement	Metabolic
PGS002933	EFO_0004465	fasting blood glucose measurement	fasting blood glucose measurement	EFO_0004465	Other measurement	Metabolic
PGS002944	EFO_0004465	fasting blood glucose measurement	fasting blood glucose measurement	EFO_0004465	Other measurement	Metabolic
PGS002945	EFO_0004465	fasting blood glucose measurement	fasting blood glucose measurement	EFO_0004465	Other measurement	Metabolic
PGS002946	EFO_0004465	fasting blood glucose measurement	fasting blood glucose measurement	EFO_0004465	Other measurement	Metabolic
PGS002947	EFO_0004465	fasting blood glucose measurement	fasting blood glucose measurement	EFO_0004465	Other measurement	Metabolic
PGS002948	EFO_0004465	fasting blood glucose measurement	fasting blood glucose measurement	EFO_0004465	Other measurement	Metabolic
PGS002949	EFO_0004465	fasting blood glucose measurement	fasting blood glucose measurement	EFO_0004465	Other measurement	Metabolic
PGS002950	EFO_0004465	fasting blood glucose measurement	fasting blood glucose measurement	EFO_0004465	Other measurement	Metabolic
PGS002951	EFO_0004465	fasting blood glucose measurement	fasting blood glucose measurement	EFO_0004465	Other measurement	Metabolic
PGS002952	EFO_0004465	fasting blood glucose measurement	fasting blood glucose measurement	EFO_0004465	Other measurement	Metabolic
PGS002953	EFO_0004465	fasting blood glucose measurement	fasting blood glucose measurement	EFO_0004465	Other measurement	Metabolic

PGS000306	EFO_0008036	BMI-adjusted fasting blood glucose measurement	BMI-adjusted fasting blood glucose measurement	EFO_0008036	Other measurement	Metabolic
PGS000307	EFO_0004466	fasting blood insulin measurement	fasting blood insulin measurement	EFO_0004466	Other measurement	Metabolic
PGS000836	EFO_0004466	fasting blood insulin measurement	fasting blood insulin measurement	EFO_0004466	Other measurement	Metabolic
PGS001351	EFO_0004466	fasting blood insulin measurement	fasting blood insulin measurement	EFO_0004466	Other measurement	Metabolic
PGS000308	EFO_0008037	BMI-adjusted fasting blood insulin measurement	BMI-adjusted fasting blood insulin measurement	EFO_0008037	Other measurement	Metabolic
PGS000313	EFO_0006925	lipoprotein A measurement	lipoprotein A measurement	EFO_0006925	Lipid or lipoprotein measurement	Metabolic
PGS000667	EFO_0006925	lipoprotein A measurement	lipoprotein A measurement	EFO_0006925	Lipid or lipoprotein measurement	Metabolic
PGS000689	EFO_0006925	lipoprotein A measurement	lipoprotein A measurement	EFO_0006925	Lipid or lipoprotein measurement	Metabolic
PGS000752	EFO_0006925	lipoprotein A measurement	lipoprotein A measurement	EFO_0006925	Lipid or lipoprotein measurement	Metabolic
PGS001963	EFO_0006925	lipoprotein A measurement	lipoprotein A measurement	EFO_0006925	Lipid or lipoprotein measurement	Metabolic
PGS002181	EFO_0006925	lipoprotein A measurement	lipoprotein A measurement	EFO_0006925	Lipid or lipoprotein measurement	Metabolic
PGS002297	EFO_0006925	lipoprotein A measurement	lipoprotein A measurement	EFO_0006925	Lipid or lipoprotein measurement	Metabolic
PGS000314	EFO_0004458	C-reactive protein measurement	C-reactive protein measurement	EFO_0004458	Inflammatory measurement	Immunological
PGS000675	EFO_0004458	C-reactive protein measurement	C-reactive protein measurement	EFO_0004458	Inflammatory measurement	Immunological
PGS001946	EFO_0004458	C-reactive protein measurement	C-reactive protein measurement	EFO_0004458	Inflammatory measurement	Immunological
PGS002164	EFO_0004458	C-reactive protein measurement	C-reactive protein measurement	EFO_0004458	Inflammatory measurement	Immunological
PGS002860	EFO_0004458	C-reactive protein measurement	C-reactive protein measurement	EFO_0004458	Inflammatory measurement	Immunological
PGS002861	EFO_0004458	C-reactive protein measurement	C-reactive protein measurement	EFO_0004458	Inflammatory measurement	Immunological
PGS002862	EFO_0004458	C-reactive protein measurement	C-reactive protein measurement	EFO_0004458	Inflammatory measurement	Immunological
PGS002863	EFO_0004458	C-reactive protein measurement	C-reactive protein measurement	EFO_0004458	Inflammatory measurement	Immunological
PGS002864	EFO_0004458	C-reactive protein measurement	C-reactive protein measurement	EFO_0004458	Inflammatory measurement	Immunological
PGS002865	EFO_0004458	C-reactive protein measurement	C-reactive protein measurement	EFO_0004458	Inflammatory measurement	Immunological
PGS002866	EFO_0004458	C-reactive protein measurement	C-reactive protein measurement	EFO_0004458	Inflammatory measurement	Immunological
PGS002867	EFO_0004458	C-reactive protein measurement	C-reactive protein measurement	EFO_0004458	Inflammatory measurement	Immunological
PGS002868	EFO_0004458	C-reactive protein measurement	C-reactive protein measurement	EFO_0004458	Inflammatory measurement	Immunological
PGS002869	EFO_0004458	C-reactive protein measurement	C-reactive protein measurement	EFO_0004458	Inflammatory measurement	Immunological

PGS002870	EFO_0004458	C-reactive protein measurement	C-reactive protein measurement	EFO_0004458	Inflammatory measurement	Immunological
PGS002871	EFO_0004458	C-reactive protein measurement	C-reactive protein measurement	EFO_0004458	Inflammatory measurement	Immunological
PGS002872	EFO_0004458	C-reactive protein measurement	C-reactive protein measurement	EFO_0004458	Inflammatory measurement	Immunological
PGS002873	EFO_0004458	C-reactive protein measurement	C-reactive protein measurement	EFO_0004458	Inflammatory measurement	Immunological
PGS002874	EFO_0004458	C-reactive protein measurement	C-reactive protein measurement	EFO_0004458	Inflammatory measurement	Immunological
PGS002875	EFO_0004458	C-reactive protein measurement	C-reactive protein measurement	EFO_0004458	Inflammatory measurement	Immunological
PGS002876	EFO_0004458	C-reactive protein measurement	C-reactive protein measurement	EFO_0004458	Inflammatory measurement	Immunological
PGS002877	EFO_0004458	C-reactive protein measurement	C-reactive protein measurement	EFO_0004458	Inflammatory measurement	Immunological
PGS002878	EFO_0004458	C-reactive protein measurement	C-reactive protein measurement	EFO_0004458	Inflammatory measurement	Immunological
PGS002879	EFO_0004458	C-reactive protein measurement	C-reactive protein measurement	EFO_0004458	Inflammatory measurement	Immunological
PGS002880	EFO_0004458	C-reactive protein measurement	C-reactive protein measurement	EFO_0004458	Inflammatory measurement	Immunological
PGS002881	EFO_0004458	C-reactive protein measurement	C-reactive protein measurement	EFO_0004458	Inflammatory measurement	Immunological
PGS002882	EFO_0004458	C-reactive protein measurement	C-reactive protein measurement	EFO_0004458	Inflammatory measurement	Immunological
PGS002883	EFO_0004458	C-reactive protein measurement	C-reactive protein measurement	EFO_0004458	Inflammatory measurement	Immunological
PGS002884	EFO_0004458	C-reactive protein measurement	C-reactive protein measurement	EFO_0004458	Inflammatory measurement	Immunological
PGS002885	EFO_0004458	C-reactive protein measurement	C-reactive protein measurement	EFO_0004458	Inflammatory measurement	Immunological
PGS002886	EFO_0004458	C-reactive protein measurement	C-reactive protein measurement	EFO_0004458	Inflammatory measurement	Immunological
PGS002887	EFO_0004458	C-reactive protein measurement	C-reactive protein measurement	EFO_0004458	Inflammatory measurement	Immunological
PGS002888	EFO_0004458	C-reactive protein measurement	C-reactive protein measurement	EFO_0004458	Inflammatory measurement	Immunological
PGS000315	EFO_0004579	serum IgE measurement	serum IgE measurement	EFO_0004579	Other measurement	Metabolic
PGS000318	EFO_0005056	age at death|female	age at death	EFO_0005056	Other measurement	Mortality
PGS000319	EFO_0005056	age at death|male	age at death	EFO_0005056	Other measurement	Mortality
PGS000321	EFO_0004908	testosterone measurement	testosterone measurement	EFO_0004908	Lipid or lipoprotein measurement	Metabolic
PGS000322	EFO_0004908	testosterone measurement|female	testosterone measurement	EFO_0004908	Lipid or lipoprotein measurement	Metabolic
PGS000323	EFO_0004908	testosterone measurement|male	testosterone measurement	EFO_0004908	Lipid or lipoprotein measurement	Metabolic
PGS000696	EFO_0004908	testosterone measurement	testosterone measurement	EFO_0004908	Lipid or lipoprotein measurement	Metabolic

PGS001914	EFO_0004908	testosterone measurement|female	testosterone measurement	EFO_0004908	Lipid or lipoprotein measurement	Metabolic
PGS001988	EFO_0004908	testosterone measurement|male	testosterone measurement	EFO_0004908	Lipid or lipoprotein measurement	Metabolic
PGS002130	EFO_0004908	testosterone measurement|female	testosterone measurement	EFO_0004908	Lipid or lipoprotein measurement	Metabolic
PGS002205	EFO_0004908	testosterone measurement|male	testosterone measurement	EFO_0004908	Lipid or lipoprotein measurement	Metabolic
PGS000324	EFO_0009732	enthesitis-related juvenile idiopathic arthritis	enthesitis-related juvenile idiopathic arthritis	EFO_0009732	Immune system disorder	Immunological
PGS000325	EFO_1002019	oligoarticular juvenile idiopathic arthritis	oligoarticular juvenile idiopathic arthritis	EFO_1002019	Immune system disorder	Immunological
PGS000326	EFO_1002020	polyarticular juvenile idiopathic arthritis, rheumatoid factor negative	polyarticular juvenile idiopathic arthritis, rheumatoid factor negative	EFO_1002020	Immune system disorder	Immunological
PGS000327	EFO_0003756	autism spectrum disorder	autism spectrum disorder	EFO_0003756	Neurological disorder	Neurological
PGS002790	EFO_0003756	autism spectrum disorder	autism spectrum disorder	EFO_0003756	Neurological disorder	Neurological
PGS000336	EFO_0008328	chronotype measurement	chronotype measurement	EFO_0008328	Other measurement	Psychiatric
PGS001055	EFO_0008328	chronotype measurement	chronotype measurement	EFO_0008328	Other measurement	Psychiatric
PGS001992	EFO_0008328	chronotype measurement	chronotype measurement	EFO_0008328	Other measurement	Psychiatric
PGS002209	EFO_0008328	chronotype measurement	chronotype measurement	EFO_0008328	Other measurement	Psychiatric
PGS002318	EFO_0008328	chronotype measurement	chronotype measurement	EFO_0008328	Other measurement	Psychiatric
PGS002390	EFO_0008328	chronotype measurement	chronotype measurement	EFO_0008328	Other measurement	Psychiatric
PGS002439	EFO_0008328	chronotype measurement	chronotype measurement	EFO_0008328	Other measurement	Psychiatric
PGS002488	EFO_0008328	chronotype measurement	chronotype measurement	EFO_0008328	Other measurement	Psychiatric
PGS002537	EFO_0008328	chronotype measurement	chronotype measurement	EFO_0008328	Other measurement	Psychiatric
PGS002586	EFO_0008328	chronotype measurement	chronotype measurement	EFO_0008328	Other measurement	Psychiatric
PGS002635	EFO_0008328	chronotype measurement	chronotype measurement	EFO_0008328	Other measurement	Psychiatric
PGS000339	EFO_0000389	cutaneous melanoma	cutaneous melanoma	EFO_0000389	Cancer	Neoplasms
PGS003382	EFO_0000389	cutaneous melanoma	cutaneous melanoma	EFO_0000389	Cancer	Neoplasms
PGS000341	EFO_0000717	systemic scleroderma	systemic scleroderma	EFO_0000717	Cardiovascular disease	Cardiovascular
PGS000341	EFO_0000717	systemic scleroderma	systemic scleroderma	EFO_0000717	Immune system disorder	Immunological
PGS000352	EFO_1001958	high grade ovarian serous adenocarcinoma	high grade ovarian serous adenocarcinoma	EFO_1001958	Cancer	Neoplasms
PGS000353	MONDO_0004992	cancer	cancer	MONDO_0004992	Cancer	Neoplasms
PGS000354	MONDO_0004992	cancer	cancer	MONDO_0004992	Cancer	Neoplasms
PGS000355	MONDO_0004992	cancer	cancer	MONDO_0004992	Cancer	Neoplasms
PGS000356	MONDO_0004992	cancer	cancer	MONDO_0004992	Cancer	Neoplasms
PGS000359	EFO_0003871	tongue neoplasm	tongue neoplasm	EFO_0003871	Cancer	Neoplasms
PGS000359	EFO_0003871	tongue neoplasm	tongue neoplasm	EFO_0003871	Digestive system disorder	Gastrointestinal
PGS000360	EFO_1000354	Malignant Laryngeal Neoplasm	Malignant Laryngeal Neoplasm	EFO_1000354	Cancer	Neoplasms
PGS000361	EFO_1000354	Malignant Laryngeal Neoplasm	Malignant Laryngeal Neoplasm	EFO_1000354	Cancer	Neoplasms
PGS000362	EFO_1000354	Malignant Laryngeal Neoplasm	Malignant Laryngeal Neoplasm	EFO_1000354	Cancer	Neoplasms
PGS000363	MONDO_0007576	esophageal cancer	esophageal cancer	MONDO_0007576	Cancer	Neoplasms

PGS000363	MONDO_0007576	esophageal cancer	esophageal cancer	MONDO_0007576	Digestive system disorder	Gastrointestinal
PGS000364	MONDO_0007576	esophageal cancer	esophageal cancer	MONDO_0007576	Cancer	Neoplasms
PGS000364	MONDO_0007576	esophageal cancer	esophageal cancer	MONDO_0007576	Digestive system disorder	Gastrointestinal
PGS000365	MONDO_0007576	esophageal cancer	esophageal cancer	MONDO_0007576	Cancer	Neoplasms
PGS000365	MONDO_0007576	esophageal cancer	esophageal cancer	MONDO_0007576	Digestive system disorder	Gastrointestinal
PGS000366	MONDO_0007576	esophageal cancer	esophageal cancer	MONDO_0007576	Cancer	Neoplasms
PGS000366	MONDO_0007576	esophageal cancer	esophageal cancer	MONDO_0007576	Digestive system disorder	Gastrointestinal
PGS000376	EFO_1001950	colon carcinoma	colon carcinoma	EFO_1001950	Cancer	Neoplasms
PGS000376	EFO_1001950	colon carcinoma	colon carcinoma	EFO_1001950	Digestive system disorder	Gastrointestinal
PGS000377	EFO_1001950	colon carcinoma	colon carcinoma	EFO_1001950	Cancer	Neoplasms
PGS000377	EFO_1001950	colon carcinoma	colon carcinoma	EFO_1001950	Digestive system disorder	Gastrointestinal
PGS000378	EFO_1001950	colon carcinoma	colon carcinoma	EFO_1001950	Cancer	Neoplasms
PGS000378	EFO_1001950	colon carcinoma	colon carcinoma	EFO_1001950	Digestive system disorder	Gastrointestinal
PGS000379	EFO_1001950	colon carcinoma	colon carcinoma	EFO_1001950	Cancer	Neoplasms
PGS000379	EFO_1001950	colon carcinoma	colon carcinoma	EFO_1001950	Digestive system disorder	Gastrointestinal
PGS000380	EFO_1001950	colon carcinoma	colon carcinoma	EFO_1001950	Cancer	Neoplasms
PGS000380	EFO_1001950	colon carcinoma	colon carcinoma	EFO_1001950	Digestive system disorder	Gastrointestinal
PGS000381	EFO_1001950	colon carcinoma	colon carcinoma	EFO_1001950	Cancer	Neoplasms
PGS000381	EFO_1001950	colon carcinoma	colon carcinoma	EFO_1001950	Digestive system disorder	Gastrointestinal
PGS000382	EFO_1001950	colon carcinoma	colon carcinoma	EFO_1001950	Cancer	Neoplasms
PGS000382	EFO_1001950	colon carcinoma	colon carcinoma	EFO_1001950	Digestive system disorder	Gastrointestinal
PGS000383	EFO_1000657	rectum cancer|rectosigmoid junction neoplasm	rectum cancer	EFO_1000657	Cancer	Neoplasms
PGS000383	EFO_1000657	rectum cancer|rectosigmoid junction neoplasm	rectum cancer	EFO_1000657	Digestive system disorder	Gastrointestinal
PGS000384	EFO_1000657	rectum cancer|rectosigmoid junction neoplasm	rectum cancer	EFO_1000657	Cancer	Neoplasms
PGS000384	EFO_1000657	rectum cancer|rectosigmoid junction neoplasm	rectum cancer	EFO_1000657	Digestive system disorder	Gastrointestinal
PGS000387	MONDO_0000376	respiratory system cancer	respiratory system cancer	MONDO_0000376	Cancer	Neoplasms
PGS000388	MONDO_0001672	bronchus cancer|lung cancer|tracheal cancer	bronchus cancer	MONDO_0001672	Cancer	Neoplasms
PGS000389	MONDO_0001672	bronchus cancer|lung cancer|tracheal cancer	bronchus cancer	MONDO_0001672	Cancer	Neoplasms
PGS000390	MONDO_0001672	bronchus cancer|lung cancer|tracheal cancer	bronchus cancer	MONDO_0001672	Cancer	Neoplasms

PGS000391	MONDO_0001672	bronchus cancer|lung cancer|tracheal cancer	bronchus cancer	MONDO_0001672	Cancer	Neoplasms
PGS000392	MONDO_0001672	bronchus cancer|lung cancer|tracheal cancer	bronchus cancer	MONDO_0001672	Cancer	Neoplasms
PGS000393	MONDO_0001672	bronchus cancer|lung cancer|tracheal cancer	bronchus cancer	MONDO_0001672	Cancer	Neoplasms
PGS000394	MONDO_0001672	bronchus cancer|lung cancer|tracheal cancer	bronchus cancer	MONDO_0001672	Cancer	Neoplasms
PGS000395	MONDO_0001672	bronchus cancer|lung cancer|tracheal cancer	bronchus cancer	MONDO_0001672	Cancer	Neoplasms
PGS000396	MONDO_0001672	bronchus cancer|lung cancer|tracheal cancer	bronchus cancer	MONDO_0001672	Cancer	Neoplasms
PGS000397	MONDO_0001672	bronchus cancer|lung cancer|tracheal cancer	bronchus cancer	MONDO_0001672	Cancer	Neoplasms
PGS000398	EFO_0009259	skin carcinoma	skin carcinoma	EFO_0009259	Cancer	Neoplasms
PGS000399	EFO_0009259	skin carcinoma	skin carcinoma	EFO_0009259	Cancer	Neoplasms
PGS000400	EFO_0009259	skin carcinoma	skin carcinoma	EFO_0009259	Cancer	Neoplasms
PGS000401	EFO_0009259	skin carcinoma	skin carcinoma	EFO_0009259	Cancer	Neoplasms
PGS000402	EFO_0009259	skin carcinoma	skin carcinoma	EFO_0009259	Cancer	Neoplasms
PGS000403	EFO_0009259	skin carcinoma	skin carcinoma	EFO_0009259	Cancer	Neoplasms
PGS000404	EFO_0009259	skin carcinoma	skin carcinoma	EFO_0009259	Cancer	Neoplasms
PGS000405	EFO_0009259	skin carcinoma	skin carcinoma	EFO_0009259	Cancer	Neoplasms
PGS000406	EFO_0009259	skin carcinoma	skin carcinoma	EFO_0009259	Cancer	Neoplasms
PGS000407	EFO_0009259	skin carcinoma	skin carcinoma	EFO_0009259	Cancer	Neoplasms
PGS001041	EFO_0009259	skin carcinoma	skin carcinoma	EFO_0009259	Cancer	Neoplasms
PGS001803	EFO_0009259	skin carcinoma	skin carcinoma	EFO_0009259	Cancer	Neoplasms
PGS002014	EFO_0009259	skin carcinoma	skin carcinoma	EFO_0009259	Cancer	Neoplasms
PGS000433	MONDO_0002898	skin cancer	skin cancer	MONDO_0002898	Cancer	Neoplasms
PGS000434	MONDO_0002898	skin cancer	skin cancer	MONDO_0002898	Cancer	Neoplasms
PGS000435	MONDO_0002898	skin cancer	skin cancer	MONDO_0002898	Cancer	Neoplasms
PGS000436	MONDO_0002898	skin cancer	skin cancer	MONDO_0002898	Cancer	Neoplasms
PGS000437	MONDO_0002898	skin cancer	skin cancer	MONDO_0002898	Cancer	Neoplasms
PGS000438	MONDO_0002898	skin cancer	skin cancer	MONDO_0002898	Cancer	Neoplasms
PGS000439	MONDO_0002898	skin cancer	skin cancer	MONDO_0002898	Cancer	Neoplasms
PGS000440	MONDO_0002898	skin cancer	skin cancer	MONDO_0002898	Cancer	Neoplasms
PGS000441	MONDO_0002898	skin cancer	skin cancer	MONDO_0002898	Cancer	Neoplasms
PGS000442	MONDO_0002898	skin cancer	skin cancer	MONDO_0002898	Cancer	Neoplasms
PGS000443	MONDO_0002898	skin cancer	skin cancer	MONDO_0002898	Cancer	Neoplasms

PGS000444	MONDO_0002898	skin cancer	skin cancer	MONDO_0002898	Cancer	Neoplasms
PGS000445	EFO_0004193	basal cell carcinoma	basal cell carcinoma	EFO_0004193	Cancer	Neoplasms
PGS000446	EFO_0004193	basal cell carcinoma	basal cell carcinoma	EFO_0004193	Cancer	Neoplasms
PGS000447	EFO_0004193	basal cell carcinoma	basal cell carcinoma	EFO_0004193	Cancer	Neoplasms
PGS000448	EFO_0004193	basal cell carcinoma	basal cell carcinoma	EFO_0004193	Cancer	Neoplasms
PGS000449	EFO_0004193	basal cell carcinoma	basal cell carcinoma	EFO_0004193	Cancer	Neoplasms
PGS000450	EFO_0004193	basal cell carcinoma	basal cell carcinoma	EFO_0004193	Cancer	Neoplasms
PGS000451	EFO_0004193	basal cell carcinoma	basal cell carcinoma	EFO_0004193	Cancer	Neoplasms
PGS000452	EFO_0004193	basal cell carcinoma	basal cell carcinoma	EFO_0004193	Cancer	Neoplasms
PGS000453	EFO_0004193	basal cell carcinoma	basal cell carcinoma	EFO_0004193	Cancer	Neoplasms
PGS000454	EFO_0004193	basal cell carcinoma	basal cell carcinoma	EFO_0004193	Cancer	Neoplasms
PGS000455	EFO_0004193	basal cell carcinoma	basal cell carcinoma	EFO_0004193	Cancer	Neoplasms
PGS000456	EFO_0004193	basal cell carcinoma	basal cell carcinoma	EFO_0004193	Cancer	Neoplasms
PGS000457	EFO_0004193	basal cell carcinoma	basal cell carcinoma	EFO_0004193	Cancer	Neoplasms
PGS000458	EFO_0004193	basal cell carcinoma	basal cell carcinoma	EFO_0004193	Cancer	Neoplasms
PGS000459	EFO_0004193	basal cell carcinoma	basal cell carcinoma	EFO_0004193	Cancer	Neoplasms
PGS000730	EFO_0004193	basal cell carcinoma	basal cell carcinoma	EFO_0004193	Cancer	Neoplasms
PGS003416	EFO_0004193	basal cell carcinoma	basal cell carcinoma	EFO_0004193	Cancer	Neoplasms
PGS003417	EFO_0004193	basal cell carcinoma	basal cell carcinoma	EFO_0004193	Cancer	Neoplasms
PGS000460	EFO_0000707	squamous cell carcinoma	squamous cell carcinoma	EFO_0000707	Cancer	Neoplasms
PGS000461	EFO_0000707	squamous cell carcinoma	squamous cell carcinoma	EFO_0000707	Cancer	Neoplasms
PGS000462	EFO_0000707	squamous cell carcinoma	squamous cell carcinoma	EFO_0000707	Cancer	Neoplasms
PGS000463	EFO_0000707	squamous cell carcinoma	squamous cell carcinoma	EFO_0000707	Cancer	Neoplasms
PGS000464	EFO_0000707	squamous cell carcinoma	squamous cell carcinoma	EFO_0000707	Cancer	Neoplasms
PGS000465	EFO_0000707	squamous cell carcinoma	squamous cell carcinoma	EFO_0000707	Cancer	Neoplasms
PGS000466	EFO_0000707	squamous cell carcinoma	squamous cell carcinoma	EFO_0000707	Cancer	Neoplasms
PGS000467	EFO_0000707	squamous cell carcinoma	squamous cell carcinoma	EFO_0000707	Cancer	Neoplasms
PGS000468	EFO_0000707	squamous cell carcinoma	squamous cell carcinoma	EFO_0000707	Cancer	Neoplasms
PGS000731	EFO_0000707	squamous cell carcinoma	squamous cell carcinoma	EFO_0000707	Cancer	Neoplasms
PGS000469	MONDO_0004641	skin carcinoma in situ	skin carcinoma in situ	MONDO_0004641	Cancer	Neoplasms
PGS000470	MONDO_0004641	skin carcinoma in situ	skin carcinoma in situ	MONDO_0004641	Cancer	Neoplasms
PGS000471	MONDO_0004641	skin carcinoma in situ	skin carcinoma in situ	MONDO_0004641	Cancer	Neoplasms
PGS000541	MONDO_0002715	uterine cancer	uterine cancer	MONDO_0002715	Cancer	Neoplasms
PGS000542	MONDO_0002715	uterine cancer	uterine cancer	MONDO_0002715	Cancer	Neoplasms
PGS000543	MONDO_0021148	female reproductive system neoplasm	female reproductive system neoplasm	MONDO_0021148	Cancer	Neoplasms
PGS000544	EFO_0003893	ovarian neoplasm	ovarian neoplasm	EFO_0003893	Cancer	Neoplasms
PGS000545	EFO_0003893	ovarian neoplasm	ovarian neoplasm	EFO_0003893	Cancer	Neoplasms
PGS000546	EFO_0003893	ovarian neoplasm	ovarian neoplasm	EFO_0003893	Cancer	Neoplasms
PGS000547	EFO_0003893	ovarian neoplasm	ovarian neoplasm	EFO_0003893	Cancer	Neoplasms
PGS000548	EFO_0003893	ovarian neoplasm	ovarian neoplasm	EFO_0003893	Cancer	Neoplasms
PGS000549	EFO_0003893	ovarian neoplasm	ovarian neoplasm	EFO_0003893	Cancer	Neoplasms
PGS000550	EFO_0003893	ovarian neoplasm	ovarian neoplasm	EFO_0003893	Cancer	Neoplasms
PGS000551	EFO_0003893	ovarian neoplasm	ovarian neoplasm	EFO_0003893	Cancer	Neoplasms

PGS000552	EFO_0003893	ovarian neoplasm	ovarian neoplasm	EFO_0003893	Cancer	Neoplasms
PGS000553	EFO_0003893	ovarian neoplasm	ovarian neoplasm	EFO_0003893	Cancer	Neoplasms
PGS000554	EFO_0003893	ovarian neoplasm	ovarian neoplasm	EFO_0003893	Cancer	Neoplasms
PGS000555	EFO_0003893	ovarian neoplasm	ovarian neoplasm	EFO_0003893	Cancer	Neoplasms
PGS000556	EFO_0003893	ovarian neoplasm	ovarian neoplasm	EFO_0003893	Cancer	Neoplasms
PGS000557	EFO_0003893	ovarian neoplasm	ovarian neoplasm	EFO_0003893	Cancer	Neoplasms
PGS000558	EFO_0003893	ovarian neoplasm	ovarian neoplasm	EFO_0003893	Cancer	Neoplasms
PGS000559	EFO_0003893	ovarian neoplasm	ovarian neoplasm	EFO_0003893	Cancer	Neoplasms
PGS000560	EFO_0003893	ovarian neoplasm	ovarian neoplasm	EFO_0003893	Cancer	Neoplasms
PGS000561	EFO_0003893	ovarian neoplasm	ovarian neoplasm	EFO_0003893	Cancer	Neoplasms
PGS000562	EFO_0003893	ovarian neoplasm	ovarian neoplasm	EFO_0003893	Cancer	Neoplasms
PGS000563	EFO_0003893	ovarian neoplasm	ovarian neoplasm	EFO_0003893	Cancer	Neoplasms
PGS000564	EFO_0003893	ovarian neoplasm	ovarian neoplasm	EFO_0003893	Cancer	Neoplasms
PGS000593	EFO_0007355	male reproductive organ cancer	male reproductive organ cancer	EFO_0007355	Cancer	Neoplasms
PGS000594	EFO_0007355	male reproductive organ cancer	male reproductive organ cancer	EFO_0007355	Cancer	Neoplasms
PGS001111	EFO_0007355	male reproductive organ cancer	male reproductive organ cancer	EFO_0007355	Cancer	Neoplasms
PGS000605	MONDO_0002367	kidney cancer	kidney cancer	MONDO_0002367	Cancer	Neoplasms
PGS000606	MONDO_0002367	kidney cancer	kidney cancer	MONDO_0002367	Cancer	Neoplasms
PGS000617	MONDO_0002236	ocular cancer	ocular cancer	MONDO_0002236	Cancer	Neoplasms
PGS000617	MONDO_0002236	ocular cancer	ocular cancer	MONDO_0002236	Neurological disorder	Neurological
PGS000618	EFO_0000326	central nervous system cancer	central nervous system cancer	EFO_0000326	Cancer	Neoplasms
PGS000618	EFO_0000326	central nervous system cancer	central nervous system cancer	EFO_0000326	Neurological disorder	Neurological
PGS000619	EFO_0000326	central nervous system cancer	central nervous system cancer	EFO_0000326	Cancer	Neoplasms
PGS000619	EFO_0000326	central nervous system cancer	central nervous system cancer	EFO_0000326	Neurological disorder	Neurological
PGS000620	MONDO_0001657	brain cancer	brain cancer	MONDO_0001657	Cancer	Neoplasms
PGS000620	MONDO_0001657	brain cancer	brain cancer	MONDO_0001657	Neurological disorder	Neurological
PGS000621	MONDO_0001657	brain cancer	brain cancer	MONDO_0001657	Cancer	Neoplasms
PGS000621	MONDO_0001657	brain cancer	brain cancer	MONDO_0001657	Neurological disorder	Neurological
PGS000622	MONDO_0001657	brain cancer	brain cancer	MONDO_0001657	Cancer	Neoplasms
PGS000622	MONDO_0001657	brain cancer	brain cancer	MONDO_0001657	Neurological disorder	Neurological
PGS000623	MONDO_0001657	brain cancer	brain cancer	MONDO_0001657	Cancer	Neoplasms
PGS000623	MONDO_0001657	brain cancer	brain cancer	MONDO_0001657	Neurological disorder	Neurological
PGS000624	MONDO_0001657	brain cancer	brain cancer	MONDO_0001657	Cancer	Neoplasms
PGS000624	MONDO_0001657	brain cancer	brain cancer	MONDO_0001657	Neurological disorder	Neurological
PGS000625	MONDO_0001657	brain cancer	brain cancer	MONDO_0001657	Cancer	Neoplasms

PGS000625	MONDO_0001657	brain cancer	brain cancer	MONDO_0001657	Neurological disorder	Neurological
PGS001808	MONDO_0001657	brain cancer	brain cancer	MONDO_0001657	Cancer	Neoplasms
PGS001808	MONDO_0001657	brain cancer	brain cancer	MONDO_0001657	Neurological disorder	Neurological
PGS000637	EFO_0000183	Hodgkins lymphoma	Hodgkins lymphoma	EFO_0000183	Cancer	Neoplasms
PGS000638	EFO_0000183	Hodgkins lymphoma	Hodgkins lymphoma	EFO_0000183	Cancer	Neoplasms
PGS000639	EFO_0000183	Hodgkins lymphoma	Hodgkins lymphoma	EFO_0000183	Cancer	Neoplasms
PGS000640	EFO_0000183	Hodgkins lymphoma	Hodgkins lymphoma	EFO_0000183	Cancer	Neoplasms
PGS000643	MONDO_0018906	follicular lymphoma	follicular lymphoma	MONDO_0018906	Cancer	Neoplasms
PGS000643	MONDO_0018906	follicular lymphoma	follicular lymphoma	MONDO_0018906	Immune system disorder	Immunological
PGS000644	MONDO_0018906	follicular lymphoma	follicular lymphoma	MONDO_0018906	Cancer	Neoplasms
PGS000644	MONDO_0018906	follicular lymphoma	follicular lymphoma	MONDO_0018906	Immune system disorder	Immunological
PGS000646	EFO_0000095	chronic lymphocytic leukemia	chronic lymphocytic leukemia	EFO_0000095	Cancer	Neoplasms
PGS000646	EFO_0000095	chronic lymphocytic leukemia	chronic lymphocytic leukemia	EFO_0000095	Immune system disorder	Immunological
PGS000647	EFO_0000095	chronic lymphocytic leukemia	chronic lymphocytic leukemia	EFO_0000095	Cancer	Neoplasms
PGS000647	EFO_0000095	chronic lymphocytic leukemia	chronic lymphocytic leukemia	EFO_0000095	Immune system disorder	Immunological
PGS000648	EFO_0000095	chronic lymphocytic leukemia	chronic lymphocytic leukemia	EFO_0000095	Cancer	Neoplasms
PGS000648	EFO_0000095	chronic lymphocytic leukemia	chronic lymphocytic leukemia	EFO_0000095	Immune system disorder	Immunological
PGS000649	EFO_0000095	chronic lymphocytic leukemia	chronic lymphocytic leukemia	EFO_0000095	Cancer	Neoplasms
PGS000649	EFO_0000095	chronic lymphocytic leukemia	chronic lymphocytic leukemia	EFO_0000095	Immune system disorder	Immunological
PGS000650	EFO_0000095	chronic lymphocytic leukemia	chronic lymphocytic leukemia	EFO_0000095	Cancer	Neoplasms
PGS000650	EFO_0000095	chronic lymphocytic leukemia	chronic lymphocytic leukemia	EFO_0000095	Immune system disorder	Immunological
PGS000651	EFO_0000095	chronic lymphocytic leukemia	chronic lymphocytic leukemia	EFO_0000095	Cancer	Neoplasms
PGS000651	EFO_0000095	chronic lymphocytic leukemia	chronic lymphocytic leukemia	EFO_0000095	Immune system disorder	Immunological
PGS000652	EFO_0001378	multiple myeloma	multiple myeloma	EFO_0001378	Cancer	Neoplasms
PGS000652	EFO_0001378	multiple myeloma	multiple myeloma	EFO_0001378	Immune system disorder	Immunological
PGS000653	EFO_0001378	multiple myeloma	multiple myeloma	EFO_0001378	Cancer	Neoplasms
PGS000653	EFO_0001378	multiple myeloma	multiple myeloma	EFO_0001378	Immune system disorder	Immunological
PGS000654	EFO_0001378	multiple myeloma	multiple myeloma	EFO_0001378	Cancer	Neoplasms
PGS000654	EFO_0001378	multiple myeloma	multiple myeloma	EFO_0001378	Immune system disorder	Immunological
PGS002281	EFO_0001378	multiple myeloma	multiple myeloma	EFO_0001378	Cancer	Neoplasms
PGS002281	EFO_0001378	multiple myeloma	multiple myeloma	EFO_0001378	Immune system disorder	Immunological
PGS000664	EFO_0006829	GFR change measurement	GFR change measurement	EFO_0006829	Other measurement	Genitourinary
PGS000665	HP_0002140	Ischemic stroke|stroke	Ischemic stroke	HP_0002140	Other trait	Cardiovascular

PGS000911	HP_0002140	Ischemic stroke|stroke	Ischemic stroke	HP_0002140	Other trait	Cardiovascular
PGS002724	HP_0002140	Ischemic stroke|stroke	Ischemic stroke	HP_0002140	Other trait	Cardiovascular
PGS002725	HP_0002140	Ischemic stroke|stroke	Ischemic stroke	HP_0002140	Other trait	Cardiovascular
PGS000666	EFO_0008206	left ventricular systolic function measurement	left ventricular systolic function measurement	EFO_0008206	Cardiovascular measurement	Cardiovascular
PGS000668	EFO_0004735	serum alanine aminotransferase measurement	serum alanine aminotransferase measurement	EFO_0004735	Liver enzyme measurement	Gastrointestinal
PGS000816	EFO_0004735	serum alanine aminotransferase measurement	serum alanine aminotransferase measurement	EFO_0004735	Liver enzyme measurement	Gastrointestinal
PGS001940	EFO_0004735	serum alanine aminotransferase measurement	serum alanine aminotransferase measurement	EFO_0004735	Liver enzyme measurement	Gastrointestinal
PGS002158	EFO_0004735	serum alanine aminotransferase measurement	serum alanine aminotransferase measurement	EFO_0004735	Liver enzyme measurement	Gastrointestinal
PGS002732	EFO_0004735	serum alanine aminotransferase measurement	serum alanine aminotransferase measurement	EFO_0004735	Liver enzyme measurement	Gastrointestinal
PGS000669	EFO_0004535	serum albumin measurement	serum albumin measurement	EFO_0004535	Other measurement	Metabolic
PGS001886	EFO_0004535	serum albumin measurement	serum albumin measurement	EFO_0004535	Other measurement	Metabolic
PGS002099	EFO_0004535	serum albumin measurement	serum albumin measurement	EFO_0004535	Other measurement	Metabolic
PGS000670	EFO_0004533	alkaline phosphatase measurement	alkaline phosphatase measurement	EFO_0004533	Liver enzyme measurement	Gastrointestinal
PGS000815	EFO_0004533	alkaline phosphatase measurement	alkaline phosphatase measurement	EFO_0004533	Liver enzyme measurement	Gastrointestinal
PGS001939	EFO_0004533	alkaline phosphatase measurement	alkaline phosphatase measurement	EFO_0004533	Liver enzyme measurement	Gastrointestinal
PGS002157	EFO_0004533	alkaline phosphatase measurement	alkaline phosphatase measurement	EFO_0004533	Liver enzyme measurement	Gastrointestinal
PGS000671	EFO_0004614	apolipoprotein A 1 measurement	apolipoprotein A 1 measurement	EFO_0004614	Cardiovascular measurement	Cardiovascular
PGS000671	EFO_0004614	apolipoprotein A 1 measurement	apolipoprotein A 1 measurement	EFO_0004614	Inflammatory measurement	Immunological
PGS000671	EFO_0004614	apolipoprotein A 1 measurement	apolipoprotein A 1 measurement	EFO_0004614	Lipid or lipoprotein measurement	Metabolic
PGS001888	EFO_0004614	apolipoprotein A 1 measurement	apolipoprotein A 1 measurement	EFO_0004614	Cardiovascular measurement	Cardiovascular
PGS001888	EFO_0004614	apolipoprotein A 1 measurement	apolipoprotein A 1 measurement	EFO_0004614	Inflammatory measurement	Immunological
PGS001888	EFO_0004614	apolipoprotein A 1 measurement	apolipoprotein A 1 measurement	EFO_0004614	Lipid or lipoprotein measurement	Metabolic
PGS002101	EFO_0004614	apolipoprotein A 1 measurement	apolipoprotein A 1 measurement	EFO_0004614	Cardiovascular measurement	Cardiovascular
PGS002101	EFO_0004614	apolipoprotein A 1 measurement	apolipoprotein A 1 measurement	EFO_0004614	Inflammatory measurement	Immunological
PGS002101	EFO_0004614	apolipoprotein A 1 measurement	apolipoprotein A 1 measurement	EFO_0004614	Lipid or lipoprotein measurement	Metabolic
PGS000672	EFO_0004615	apolipoprotein B measurement	apolipoprotein B measurement	EFO_0004615	Lipid or lipoprotein measurement	Metabolic
PGS001889	EFO_0004615	apolipoprotein B measurement	apolipoprotein B measurement	EFO_0004615	Lipid or lipoprotein measurement	Metabolic
PGS002102	EFO_0004615	apolipoprotein B measurement	apolipoprotein B measurement	EFO_0004615	Lipid or lipoprotein measurement	Metabolic

PGS000673	EFO_0004736	aspartate aminotransferase measurement	aspartate aminotransferase measurement	EFO_0004736	Liver enzyme measurement	Gastrointestinal
PGS001941	EFO_0004736	aspartate aminotransferase measurement	aspartate aminotransferase measurement	EFO_0004736	Liver enzyme measurement	Gastrointestinal
PGS002159	EFO_0004736	aspartate aminotransferase measurement	aspartate aminotransferase measurement	EFO_0004736	Liver enzyme measurement	Gastrointestinal
PGS000674	EFO_0010934	aspartate aminotransferase to alanine aminotransferase ratio	aspartate aminotransferase to alanine aminotransferase ratio	EFO_0010934	Other measurement	Metabolic
PGS000676	EFO_0004838	calcium measurement	calcium measurement	EFO_0004838	Other measurement	Metabolic
PGS001893	EFO_0004838	calcium measurement	calcium measurement	EFO_0004838	Other measurement	Metabolic
PGS002106	EFO_0004838	calcium measurement	calcium measurement	EFO_0004838	Other measurement	Metabolic
PGS000678	EFO_0004518	creatinine measurement	creatinine measurement	EFO_0004518	Other measurement	Metabolic
PGS001945	EFO_0004518	creatinine measurement	creatinine measurement	EFO_0004518	Other measurement	Metabolic
PGS002163	EFO_0004518	creatinine measurement	creatinine measurement	EFO_0004518	Other measurement	Metabolic
PGS000679	EFO_0007934	creatinine clearance measurement	creatinine clearance measurement	EFO_0007934	Other measurement	Metabolic
PGS001944	EFO_0007934	creatinine clearance measurement	creatinine clearance measurement	EFO_0007934	Other measurement	Metabolic
PGS002162	EFO_0007934	creatinine clearance measurement	creatinine clearance measurement	EFO_0007934	Other measurement	Metabolic
PGS000680	EFO_0004617	cystatin C measurement	cystatin C measurement	EFO_0004617	Other measurement	Metabolic
PGS001947	EFO_0004617	cystatin C measurement	cystatin C measurement	EFO_0004617	Other measurement	Metabolic
PGS002165	EFO_0004617	cystatin C measurement	cystatin C measurement	EFO_0004617	Other measurement	Metabolic
PGS003279	EFO_0004617	cystatin C measurement	cystatin C measurement	EFO_0004617	Other measurement	Metabolic
PGS003280	EFO_0004617	cystatin C measurement	cystatin C measurement	EFO_0004617	Other measurement	Metabolic
PGS003281	EFO_0004617	cystatin C measurement	cystatin C measurement	EFO_0004617	Other measurement	Metabolic
PGS003282	EFO_0004617	cystatin C measurement	cystatin C measurement	EFO_0004617	Other measurement	Metabolic
PGS003283	EFO_0004617	cystatin C measurement	cystatin C measurement	EFO_0004617	Other measurement	Metabolic
PGS003284	EFO_0004617	cystatin C measurement	cystatin C measurement	EFO_0004617	Other measurement	Metabolic
PGS003285	EFO_0004617	cystatin C measurement	cystatin C measurement	EFO_0004617	Other measurement	Metabolic
PGS003286	EFO_0004617	cystatin C measurement	cystatin C measurement	EFO_0004617	Other measurement	Metabolic
PGS003287	EFO_0004617	cystatin C measurement	cystatin C measurement	EFO_0004617	Other measurement	Metabolic
PGS003288	EFO_0004617	cystatin C measurement	cystatin C measurement	EFO_0004617	Other measurement	Metabolic
PGS003289	EFO_0004617	cystatin C measurement|glomerular filtration rate	cystatin C measurement	EFO_0004617	Other measurement	Metabolic
PGS003290	EFO_0004617	cystatin C measurement|glomerular filtration rate	cystatin C measurement	EFO_0004617	Other measurement	Metabolic
PGS003291	EFO_0004617	cystatin C measurement|glomerular filtration rate	cystatin C measurement	EFO_0004617	Other measurement	Metabolic
PGS003292	EFO_0004617	cystatin C measurement|glomerular filtration rate	cystatin C measurement	EFO_0004617	Other measurement	Metabolic
PGS003293	EFO_0004617	cystatin C measurement|glomerular filtration rate	cystatin C measurement	EFO_0004617	Other measurement	Metabolic
PGS003294	EFO_0004617	cystatin C measurement|glomerular filtration rate	cystatin C measurement	EFO_0004617	Other measurement	Metabolic
PGS003295	EFO_0004617	cystatin C measurement|glomerular filtration rate	cystatin C measurement	EFO_0004617	Other measurement	Metabolic
PGS003296	EFO_0004617	cystatin C measurement|glomerular filtration rate	cystatin C measurement	EFO_0004617	Other measurement	Metabolic
PGS003297	EFO_0004617	cystatin C measurement|glomerular filtration rate	cystatin C measurement	EFO_0004617	Other measurement	Metabolic
PGS003298	EFO_0004617	cystatin C measurement|glomerular filtration rate	cystatin C measurement	EFO_0004617	Other measurement	Metabolic

PGS003299	EFO_0004617	cystatin C measurement|glomerular filtration rate	cystatin C measurement	EFO_0004617	Other measurement	Metabolic
PGS003300	EFO_0004617	cystatin C measurement|glomerular filtration rate	cystatin C measurement	EFO_0004617	Other measurement	Metabolic
PGS003301	EFO_0004617	cystatin C measurement|glomerular filtration rate	cystatin C measurement	EFO_0004617	Other measurement	Metabolic
PGS003302	EFO_0004617	cystatin C measurement|glomerular filtration rate	cystatin C measurement	EFO_0004617	Other measurement	Metabolic
PGS003303	EFO_0004617	cystatin C measurement|glomerular filtration rate	cystatin C measurement	EFO_0004617	Other measurement	Metabolic
PGS003304	EFO_0004617	cystatin C measurement|glomerular filtration rate	cystatin C measurement	EFO_0004617	Other measurement	Metabolic
PGS003305	EFO_0004617	cystatin C measurement|glomerular filtration rate	cystatin C measurement	EFO_0004617	Other measurement	Metabolic
PGS003306	EFO_0004617	cystatin C measurement|glomerular filtration rate	cystatin C measurement	EFO_0004617	Other measurement	Metabolic
PGS003307	EFO_0004617	cystatin C measurement|glomerular filtration rate	cystatin C measurement	EFO_0004617	Other measurement	Metabolic
PGS003308	EFO_0004617	cystatin C measurement|glomerular filtration rate	cystatin C measurement	EFO_0004617	Other measurement	Metabolic
PGS003309	EFO_0004617	cystatin C measurement|glomerular filtration rate	cystatin C measurement	EFO_0004617	Other measurement	Metabolic
PGS003310	EFO_0004617	cystatin C measurement|glomerular filtration rate	cystatin C measurement	EFO_0004617	Other measurement	Metabolic
PGS003311	EFO_0004617	cystatin C measurement|glomerular filtration rate	cystatin C measurement	EFO_0004617	Other measurement	Metabolic
PGS003312	EFO_0004617	cystatin C measurement|glomerular filtration rate	cystatin C measurement	EFO_0004617	Other measurement	Metabolic
PGS003313	EFO_0004617	cystatin C measurement|glomerular filtration rate	cystatin C measurement	EFO_0004617	Other measurement	Metabolic
PGS003314	EFO_0004617	cystatin C measurement|glomerular filtration rate	cystatin C measurement	EFO_0004617	Other measurement	Metabolic
PGS003315	EFO_0004617	cystatin C measurement|glomerular filtration rate	cystatin C measurement	EFO_0004617	Other measurement	Metabolic
PGS003316	EFO_0004617	cystatin C measurement|glomerular filtration rate	cystatin C measurement	EFO_0004617	Other measurement	Metabolic
PGS003317	EFO_0004617	cystatin C measurement|glomerular filtration rate	cystatin C measurement	EFO_0004617	Other measurement	Metabolic
PGS003318	EFO_0004617	cystatin C measurement|glomerular filtration rate	cystatin C measurement	EFO_0004617	Other measurement	Metabolic
PGS000681	EFO_0004570	bilirubin measurement	bilirubin measurement	EFO_0004570	Other measurement	Metabolic
PGS000697	EFO_0004570	bilirubin measurement	bilirubin measurement	EFO_0004570	Other measurement	Metabolic
PGS001942	EFO_0004570	bilirubin measurement	bilirubin measurement	EFO_0004570	Other measurement	Metabolic
PGS002160	EFO_0004570	bilirubin measurement	bilirubin measurement	EFO_0004570	Other measurement	Metabolic
PGS000683	EFO_0004532	serum gamma-glutamyl transferase measurement	serum gamma-glutamyl transferase measurement	EFO_0004532	Liver enzyme measurement	Gastrointestinal
PGS000817	EFO_0004532	serum gamma-glutamyl transferase measurement	serum gamma-glutamyl transferase measurement	EFO_0004532	Liver enzyme measurement	Gastrointestinal
PGS001964	EFO_0004532	serum gamma-glutamyl transferase measurement	serum gamma-glutamyl transferase measurement	EFO_0004532	Liver enzyme measurement	Gastrointestinal

PGS002182	EFO_0004532	serum gamma-glutamyl transferase measurement	serum gamma-glutamyl transferase measurement	EFO_0004532	Liver enzyme measurement	Gastrointestinal
PGS000684	EFO_0004468	glucose measurement	glucose measurement	EFO_0004468	Other measurement	Metabolic
PGS001952	EFO_0004468	glucose measurement	glucose measurement	EFO_0004468	Other measurement	Metabolic
PGS002170	EFO_0004468	glucose measurement	glucose measurement	EFO_0004468	Other measurement	Metabolic
PGS002328	EFO_0004468	glucose measurement	glucose measurement	EFO_0004468	Other measurement	Metabolic
PGS002365	EFO_0004468	glucose measurement	glucose measurement	EFO_0004468	Other measurement	Metabolic
PGS002400	EFO_0004468	glucose measurement	glucose measurement	EFO_0004468	Other measurement	Metabolic
PGS002449	EFO_0004468	glucose measurement	glucose measurement	EFO_0004468	Other measurement	Metabolic
PGS002547	EFO_0004468	glucose measurement	glucose measurement	EFO_0004468	Other measurement	Metabolic
PGS002596	EFO_0004468	glucose measurement	glucose measurement	EFO_0004468	Other measurement	Metabolic
PGS002645	EFO_0004468	glucose measurement	glucose measurement	EFO_0004468	Other measurement	Metabolic
PGS002934	EFO_0004468	glucose measurement	glucose measurement	EFO_0004468	Other measurement	Metabolic
PGS002935	EFO_0004468	glucose measurement	glucose measurement	EFO_0004468	Other measurement	Metabolic
PGS002936	EFO_0004468	glucose measurement	glucose measurement	EFO_0004468	Other measurement	Metabolic
PGS002937	EFO_0004468	glucose measurement	glucose measurement	EFO_0004468	Other measurement	Metabolic
PGS002938	EFO_0004468	glucose measurement	glucose measurement	EFO_0004468	Other measurement	Metabolic
PGS002939	EFO_0004468	glucose measurement	glucose measurement	EFO_0004468	Other measurement	Metabolic
PGS002940	EFO_0004468	glucose measurement	glucose measurement	EFO_0004468	Other measurement	Metabolic
PGS002941	EFO_0004468	glucose measurement	glucose measurement	EFO_0004468	Other measurement	Metabolic
PGS002942	EFO_0004468	glucose measurement	glucose measurement	EFO_0004468	Other measurement	Metabolic
PGS002943	EFO_0004468	glucose measurement	glucose measurement	EFO_0004468	Other measurement	Metabolic
PGS000687	EFO_0004627	IGF-1 measurement	IGF-1 measurement	EFO_0004627	Other measurement	Metabolic
PGS001960	EFO_0004627	IGF-1 measurement	IGF-1 measurement	EFO_0004627	Other measurement	Metabolic
PGS002178	EFO_0004627	IGF-1 measurement	IGF-1 measurement	EFO_0004627	Other measurement	Metabolic
PGS000690	EFO_0010967	urinary microalbumin measurement	urinary microalbumin measurement	EFO_0010967	Other measurement	Metabolic
PGS000972	EFO_0010967	urinary microalbumin measurement	urinary microalbumin measurement	EFO_0010967	Other measurement	Metabolic
PGS001967	EFO_0010967	urinary microalbumin measurement	urinary microalbumin measurement	EFO_0010967	Other measurement	Metabolic
PGS002185	EFO_0010967	urinary microalbumin measurement	urinary microalbumin measurement	EFO_0010967	Other measurement	Metabolic
PGS000691	EFO_0004568	serum non-albumin protein measurement	serum non-albumin protein measurement	EFO_0004568	Other measurement	Metabolic
PGS000692	EFO_0010968	phosphate measurement	phosphate measurement	EFO_0010968	Other measurement	Metabolic
PGS001998	EFO_0010968	phosphate measurement	phosphate measurement	EFO_0010968	Other measurement	Metabolic
PGS002216	EFO_0010968	phosphate measurement	phosphate measurement	EFO_0010968	Other measurement	Metabolic
PGS000693	EFO_0010952	urinary potassium measurement	urinary potassium measurement	EFO_0010952	Other measurement	Metabolic
PGS001974	EFO_0010952	urinary potassium measurement	urinary potassium measurement	EFO_0010952	Other measurement	Metabolic
PGS002192	EFO_0010952	urinary potassium measurement	urinary potassium measurement	EFO_0010952	Other measurement	Metabolic
PGS000694	EFO_0004696	sex hormone-binding globulin measurement	sex hormone-binding globulin measurement	EFO_0004696	Other measurement	Metabolic
PGS001977	EFO_0004696	sex hormone-binding globulin measurement	sex hormone-binding globulin measurement	EFO_0004696	Other measurement	Metabolic
PGS002195	EFO_0004696	sex hormone-binding globulin measurement	sex hormone-binding globulin measurement	EFO_0004696	Other measurement	Metabolic
PGS000695	EFO_0009282	sodium measurement|urinary metabolite measurement	sodium measurement	EFO_0009282	Other measurement	Metabolic
PGS002007	EFO_0009282	sodium measurement	sodium measurement	EFO_0009282	Other measurement	Metabolic
PGS002226	EFO_0009282	sodium measurement	sodium measurement	EFO_0009282	Other measurement	Metabolic
PGS000698	EFO_0004536	total blood protein measurement	total blood protein measurement	EFO_0004536	Hematological measurement	Hematological
PGS002001	EFO_0004536	total blood protein measurement	total blood protein measurement	EFO_0004536	Hematological measurement	Hematological
PGS002219	EFO_0004536	total blood protein measurement	total blood protein measurement	EFO_0004536	Hematological measurement	Hematological
PGS000701	EFO_0009795	serum urea measurement	serum urea measurement	EFO_0009795	Other measurement	Metabolic

PGS000702	EFO_0004631	vitamin D measurement	vitamin D measurement	EFO_0004631	Lipid or lipoprotein measurement	Metabolic
PGS001982	EFO_0004631	vitamin D measurement	vitamin D measurement	EFO_0004631	Lipid or lipoprotein measurement	Metabolic
PGS002200	EFO_0004631	vitamin D measurement	vitamin D measurement	EFO_0004631	Lipid or lipoprotein measurement	Metabolic
PGS002355	EFO_0004631	vitamin D measurement	vitamin D measurement	EFO_0004631	Lipid or lipoprotein measurement	Metabolic
PGS002427	EFO_0004631	vitamin D measurement	vitamin D measurement	EFO_0004631	Lipid or lipoprotein measurement	Metabolic
PGS002476	EFO_0004631	vitamin D measurement	vitamin D measurement	EFO_0004631	Lipid or lipoprotein measurement	Metabolic
PGS002525	EFO_0004631	vitamin D measurement	vitamin D measurement	EFO_0004631	Lipid or lipoprotein measurement	Metabolic
PGS002574	EFO_0004631	vitamin D measurement	vitamin D measurement	EFO_0004631	Lipid or lipoprotein measurement	Metabolic
PGS002623	EFO_0004631	vitamin D measurement	vitamin D measurement	EFO_0004631	Lipid or lipoprotein measurement	Metabolic
PGS002672	EFO_0004631	vitamin D measurement	vitamin D measurement	EFO_0004631	Lipid or lipoprotein measurement	Metabolic
PGS002721	EFO_0004631	vitamin D measurement	vitamin D measurement	EFO_0004631	Lipid or lipoprotein measurement	Metabolic
PGS003174	EFO_0004631	vitamin D measurement	vitamin D measurement	EFO_0004631	Lipid or lipoprotein measurement	Metabolic
PGS003175	EFO_0004631	vitamin D measurement	vitamin D measurement	EFO_0004631	Lipid or lipoprotein measurement	Metabolic
PGS003176	EFO_0004631	vitamin D measurement	vitamin D measurement	EFO_0004631	Lipid or lipoprotein measurement	Metabolic
PGS003177	EFO_0004631	vitamin D measurement	vitamin D measurement	EFO_0004631	Lipid or lipoprotein measurement	Metabolic
PGS003178	EFO_0004631	vitamin D measurement	vitamin D measurement	EFO_0004631	Lipid or lipoprotein measurement	Metabolic
PGS003179	EFO_0004631	vitamin D measurement	vitamin D measurement	EFO_0004631	Lipid or lipoprotein measurement	Metabolic
PGS003180	EFO_0004631	vitamin D measurement	vitamin D measurement	EFO_0004631	Lipid or lipoprotein measurement	Metabolic
PGS003181	EFO_0004631	vitamin D measurement	vitamin D measurement	EFO_0004631	Lipid or lipoprotein measurement	Metabolic
PGS003182	EFO_0004631	vitamin D measurement	vitamin D measurement	EFO_0004631	Lipid or lipoprotein measurement	Metabolic
PGS003183	EFO_0004631	vitamin D measurement	vitamin D measurement	EFO_0004631	Lipid or lipoprotein measurement	Metabolic
PGS003184	EFO_0004631	vitamin D measurement	vitamin D measurement	EFO_0004631	Lipid or lipoprotein measurement	Metabolic
PGS003185	EFO_0004631	vitamin D measurement	vitamin D measurement	EFO_0004631	Lipid or lipoprotein measurement	Metabolic
PGS003186	EFO_0004631	vitamin D measurement	vitamin D measurement	EFO_0004631	Lipid or lipoprotein measurement	Metabolic
PGS003187	EFO_0004631	vitamin D measurement	vitamin D measurement	EFO_0004631	Lipid or lipoprotein measurement	Metabolic

PGS003188	EFO_0004631	vitamin D measurement	vitamin D measurement	EFO_0004631	Lipid or lipoprotein measurement	Metabolic
PGS003189	EFO_0004631	vitamin D measurement	vitamin D measurement	EFO_0004631	Lipid or lipoprotein measurement	Metabolic
PGS003190	EFO_0004631	vitamin D measurement	vitamin D measurement	EFO_0004631	Lipid or lipoprotein measurement	Metabolic
PGS003191	EFO_0004631	vitamin D measurement	vitamin D measurement	EFO_0004631	Lipid or lipoprotein measurement	Metabolic
PGS003192	EFO_0004631	vitamin D measurement	vitamin D measurement	EFO_0004631	Lipid or lipoprotein measurement	Metabolic
PGS003193	EFO_0004631	vitamin D measurement	vitamin D measurement	EFO_0004631	Lipid or lipoprotein measurement	Metabolic
PGS003194	EFO_0004631	vitamin D measurement	vitamin D measurement	EFO_0004631	Lipid or lipoprotein measurement	Metabolic
PGS003195	EFO_0004631	vitamin D measurement	vitamin D measurement	EFO_0004631	Lipid or lipoprotein measurement	Metabolic
PGS003196	EFO_0004631	vitamin D measurement	vitamin D measurement	EFO_0004631	Lipid or lipoprotein measurement	Metabolic
PGS003197	EFO_0004631	vitamin D measurement	vitamin D measurement	EFO_0004631	Lipid or lipoprotein measurement	Metabolic
PGS003198	EFO_0004631	vitamin D measurement	vitamin D measurement	EFO_0004631	Lipid or lipoprotein measurement	Metabolic
PGS003199	EFO_0004631	vitamin D measurement	vitamin D measurement	EFO_0004631	Lipid or lipoprotein measurement	Metabolic
PGS003200	EFO_0004631	vitamin D measurement	vitamin D measurement	EFO_0004631	Lipid or lipoprotein measurement	Metabolic
PGS003201	EFO_0004631	vitamin D measurement	vitamin D measurement	EFO_0004631	Lipid or lipoprotein measurement	Metabolic
PGS003202	EFO_0004631	vitamin D measurement	vitamin D measurement	EFO_0004631	Lipid or lipoprotein measurement	Metabolic
PGS003203	EFO_0004631	vitamin D measurement	vitamin D measurement	EFO_0004631	Lipid or lipoprotein measurement	Metabolic
PGS000703	EFO_0003913	angina pectoris	angina pectoris	EFO_0003913	Other trait	Cardiovascular
PGS001260	EFO_0003913	angina pectoris	angina pectoris	EFO_0003913	Other trait	Cardiovascular
PGS001261	EFO_0003913	angina pectoris	angina pectoris	EFO_0003913	Other trait	Cardiovascular
PGS001262	EFO_0003913	angina pectoris	angina pectoris	EFO_0003913	Other trait	Cardiovascular
PGS000704	EFO_1000802	alcoholic liver cirrhosis	alcoholic liver cirrhosis	EFO_1000802	Cancer	Neoplasms
PGS000704	EFO_1000802	alcoholic liver cirrhosis	alcoholic liver cirrhosis	EFO_1000802	Digestive system disorder	Gastrointestinal
PGS001777	EFO_1000802	alcoholic liver cirrhosis	alcoholic liver cirrhosis	EFO_1000802	Cancer	Neoplasms
PGS001777	EFO_1000802	alcoholic liver cirrhosis	alcoholic liver cirrhosis	EFO_1000802	Digestive system disorder	Gastrointestinal
PGS000705	EFO_0004210	gallstones	gallstones	EFO_0004210	Digestive system disorder	Gastrointestinal
PGS001256	EFO_0004210	gallstones	gallstones	EFO_0004210	Digestive system disorder	Gastrointestinal
PGS000706	EFO_0000537	hypertension	hypertension	EFO_0000537	Cardiovascular disease	Cardiovascular
PGS001320	EFO_0000537	hypertension	hypertension	EFO_0000537	Cardiovascular disease	Cardiovascular
PGS001838	EFO_0000537	hypertension	hypertension	EFO_0000537	Cardiovascular disease	Cardiovascular
PGS002047	EFO_0000537	hypertension	hypertension	EFO_0000537	Cardiovascular disease	Cardiovascular

PGS002296	EFO_0000537	hypertension	hypertension	EFO_0000537	Cardiovascular disease	Cardiovascular
PGS002335	EFO_0000537	hypertension	hypertension	EFO_0000537	Cardiovascular disease	Cardiovascular
PGS002407	EFO_0000537	hypertension	hypertension	EFO_0000537	Cardiovascular disease	Cardiovascular
PGS002456	EFO_0000537	hypertension	hypertension	EFO_0000537	Cardiovascular disease	Cardiovascular
PGS002505	EFO_0000537	hypertension	hypertension	EFO_0000537	Cardiovascular disease	Cardiovascular
PGS002554	EFO_0000537	hypertension	hypertension	EFO_0000537	Cardiovascular disease	Cardiovascular
PGS002603	EFO_0000537	hypertension	hypertension	EFO_0000537	Cardiovascular disease	Cardiovascular
PGS002652	EFO_0000537	hypertension	hypertension	EFO_0000537	Cardiovascular disease	Cardiovascular
PGS002765	EFO_0000537	hypertension	hypertension	EFO_0000537	Cardiovascular disease	Cardiovascular
PGS002994	EFO_0000537	hypertension	hypertension	EFO_0000537	Cardiovascular disease	Cardiovascular
PGS002995	EFO_0000537	hypertension	hypertension	EFO_0000537	Cardiovascular disease	Cardiovascular
PGS002996	EFO_0000537	hypertension	hypertension	EFO_0000537	Cardiovascular disease	Cardiovascular
PGS002997	EFO_0000537	hypertension	hypertension	EFO_0000537	Cardiovascular disease	Cardiovascular
PGS002998	EFO_0000537	hypertension	hypertension	EFO_0000537	Cardiovascular disease	Cardiovascular
PGS002999	EFO_0000537	hypertension	hypertension	EFO_0000537	Cardiovascular disease	Cardiovascular
PGS003000	EFO_0000537	hypertension	hypertension	EFO_0000537	Cardiovascular disease	Cardiovascular
PGS003001	EFO_0000537	hypertension	hypertension	EFO_0000537	Cardiovascular disease	Cardiovascular
PGS003002	EFO_0000537	hypertension	hypertension	EFO_0000537	Cardiovascular disease	Cardiovascular
PGS003003	EFO_0000537	hypertension	hypertension	EFO_0000537	Cardiovascular disease	Cardiovascular
PGS003004	EFO_0000537	hypertension	hypertension	EFO_0000537	Cardiovascular disease	Cardiovascular
PGS003005	EFO_0000537	hypertension	hypertension	EFO_0000537	Cardiovascular disease	Cardiovascular
PGS003006	EFO_0000537	hypertension	hypertension	EFO_0000537	Cardiovascular disease	Cardiovascular
PGS003007	EFO_0000537	hypertension	hypertension	EFO_0000537	Cardiovascular disease	Cardiovascular
PGS003008	EFO_0000537	hypertension	hypertension	EFO_0000537	Cardiovascular disease	Cardiovascular
PGS003009	EFO_0000537	hypertension	hypertension	EFO_0000537	Cardiovascular disease	Cardiovascular
PGS003010	EFO_0000537	hypertension	hypertension	EFO_0000537	Cardiovascular disease	Cardiovascular
PGS003011	EFO_0000537	hypertension	hypertension	EFO_0000537	Cardiovascular disease	Cardiovascular
PGS003012	EFO_0000537	hypertension	hypertension	EFO_0000537	Cardiovascular disease	Cardiovascular
PGS003013	EFO_0000537	hypertension	hypertension	EFO_0000537	Cardiovascular disease	Cardiovascular
PGS003014	EFO_0000537	hypertension	hypertension	EFO_0000537	Cardiovascular disease	Cardiovascular
PGS003015	EFO_0000537	hypertension	hypertension	EFO_0000537	Cardiovascular disease	Cardiovascular
PGS003016	EFO_0000537	hypertension	hypertension	EFO_0000537	Cardiovascular disease	Cardiovascular
PGS003017	EFO_0000537	hypertension	hypertension	EFO_0000537	Cardiovascular disease	Cardiovascular
PGS003018	EFO_0000537	hypertension	hypertension	EFO_0000537	Cardiovascular disease	Cardiovascular
PGS003019	EFO_0000537	hypertension	hypertension	EFO_0000537	Cardiovascular disease	Cardiovascular
PGS003020	EFO_0000537	hypertension	hypertension	EFO_0000537	Cardiovascular disease	Cardiovascular
PGS003021	EFO_0000537	hypertension	hypertension	EFO_0000537	Cardiovascular disease	Cardiovascular
PGS003022	EFO_0000537	hypertension	hypertension	EFO_0000537	Cardiovascular disease	Cardiovascular
PGS003023	EFO_0000537	hypertension	hypertension	EFO_0000537	Cardiovascular disease	Cardiovascular
PGS003024	EFO_0000537	hypertension	hypertension	EFO_0000537	Cardiovascular disease	Cardiovascular
PGS003025	EFO_0000537	hypertension	hypertension	EFO_0000537	Cardiovascular disease	Cardiovascular
PGS003026	EFO_0000537	hypertension	hypertension	EFO_0000537	Cardiovascular disease	Cardiovascular
PGS003027	EFO_0000537	hypertension	hypertension	EFO_0000537	Cardiovascular disease	Cardiovascular
PGS003028	EFO_0000537	hypertension	hypertension	EFO_0000537	Cardiovascular disease	Cardiovascular
PGS000707	HP_0001082	Cholecystitis	Cholecystitis	HP_0001082	Other trait	Gastrointestinal
PGS000942	HP_0001082	Cholecystitis	Cholecystitis	HP_0001082	Other trait	Gastrointestinal
PGS000943	HP_0001082	Cholecystitis	Cholecystitis	HP_0001082	Other trait	Gastrointestinal
PGS000708	EFO_1002048	kidney failure	kidney failure	EFO_1002048	Other disease	Genitourinary
PGS000709	EFO_0003144	heart failure	heart failure	EFO_0003144	Cardiovascular disease	Cardiovascular
PGS001790	EFO_0003144	heart failure	heart failure	EFO_0003144	Cardiovascular disease	Cardiovascular

PGS000710	EFO_0000612	myocardial infarction	myocardial infarction	EFO_0000612	Cardiovascular disease	Cardiovascular
PGS001314	EFO_0000612	myocardial infarction	myocardial infarction	EFO_0000612	Cardiovascular disease	Cardiovascular
PGS001315	EFO_0000612	myocardial infarction	myocardial infarction	EFO_0000612	Cardiovascular disease	Cardiovascular
PGS001316	EFO_0000612	myocardial infarction	myocardial infarction	EFO_0000612	Cardiovascular disease	Cardiovascular
PGS001317	EFO_0000612	myocardial infarction	myocardial infarction	EFO_0000612	Cardiovascular disease	Cardiovascular
PGS000716	EFO_0009819	comparative body size at age 10, self-reported|body mass index	comparative body size at age 10, self-reported	EFO_0009819	Other trait	Body measurement
PGS000998	EFO_0009819	comparative body size at age 10, self-reported	comparative body size at age 10, self-reported	EFO_0009819	Other trait	Body measurement
PGS000999	EFO_0009819	comparative body size at age 10, self-reported	comparative body size at age 10, self-reported	EFO_0009819	Other trait	Body measurement
PGS000718	EFO_0007766	response to beta blocker	response to beta blocker	EFO_0007766	Biological process	Cardiovascular
PGS000718	EFO_0007766	response to beta blocker	response to beta blocker	EFO_0007766	Response to drug	Cardiovascular
PGS000722	EFO_0002890	renal carcinoma	renal carcinoma	EFO_0002890	Cancer	Neoplasms
PGS000726	EFO_0001422	cirrhosis of liver	cirrhosis of liver	EFO_0001422	Cancer	Neoplasms
PGS000726	EFO_0001422	cirrhosis of liver	cirrhosis of liver	EFO_0001422	Digestive system disorder	Gastrointestinal
PGS000776	EFO_0001422	cirrhosis of liver	cirrhosis of liver	EFO_0001422	Cancer	Neoplasms
PGS000776	EFO_0001422	cirrhosis of liver	cirrhosis of liver	EFO_0001422	Digestive system disorder	Gastrointestinal
PGS000728	EFO_0003884	chronic kidney disease	chronic kidney disease	EFO_0003884	Other disease	Metabolic
PGS000859	EFO_0003884	chronic kidney disease	chronic kidney disease	EFO_0003884	Other disease	Metabolic
PGS001272	EFO_0003884	chronic kidney disease	chronic kidney disease	EFO_0003884	Other disease	Metabolic
PGS002237	EFO_0003884	chronic kidney disease	chronic kidney disease	EFO_0003884	Other disease	Metabolic
PGS002757	EFO_0003884	chronic kidney disease	chronic kidney disease	EFO_0003884	Other disease	Metabolic
PGS000735	EFO_0004462	PR interval	PR interval	EFO_0004462	Cardiovascular measurement	Cardiovascular
PGS001521	EFO_0004462	PR interval	PR interval	EFO_0004462	Cardiovascular measurement	Cardiovascular
PGS001904	EFO_0004462	PR interval	PR interval	EFO_0004462	Cardiovascular measurement	Cardiovascular
PGS002118	EFO_0004462	PR interval	PR interval	EFO_0004462	Cardiovascular measurement	Cardiovascular
PGS000736	EFO_0005055	QRS duration	QRS duration	EFO_0005055	Cardiovascular measurement	Cardiovascular
PGS001525	EFO_0005055	QRS duration	QRS duration	EFO_0005055	Cardiovascular measurement	Cardiovascular
PGS001948	EFO_0005055	QRS duration	QRS duration	EFO_0005055	Cardiovascular measurement	Cardiovascular
PGS002166	EFO_0005055	QRS duration	QRS duration	EFO_0005055	Cardiovascular measurement	Cardiovascular
PGS000737	MONDO_0015263	Brugada syndrome	Brugada syndrome	MONDO_0015263	Cardiovascular disease	Cardiovascular
PGS001779	MONDO_0015263	Brugada syndrome	Brugada syndrome	MONDO_0015263	Cardiovascular disease	Cardiovascular
PGS000738	EFO_0004208	Vitiligo	Vitiligo	EFO_0004208	Immune system disorder	Immunological
PGS000760	EFO_0004208	Vitiligo	Vitiligo	EFO_0004208	Immune system disorder	Immunological
PGS001536	EFO_0004208	Vitiligo	Vitiligo	EFO_0004208	Immune system disorder	Immunological
PGS000739	EFO_0000538	hypertrophic cardiomyopathy	hypertrophic cardiomyopathy	EFO_0000538	Cardiovascular disease	Cardiovascular

PGS000778	EFO_0000538	hypertrophic cardiomyopathy	hypertrophic cardiomyopathy	EFO_0000538	Cardiovascular disease	Cardiovascular
PGS000753	EFO_0004214	Abdominal Aortic Aneurysm	Abdominal Aortic Aneurysm	EFO_0004214	Cardiovascular disease	Cardiovascular
PGS001784	EFO_0004214	Abdominal Aortic Aneurysm	Abdominal Aortic Aneurysm	EFO_0004214	Cardiovascular disease	Cardiovascular
PGS002054	EFO_0004214	Abdominal Aortic Aneurysm	Abdominal Aortic Aneurysm	EFO_0004214	Cardiovascular disease	Cardiovascular
PGS000756	MONDO_0016158	narcolepsy-cataplexy syndrome	narcolepsy-cataplexy syndrome	MONDO_0016158	Neurological disorder	Neurological
PGS000759	EFO_0004705	hypothyroidism	hypothyroidism	EFO_0004705	Other disease	Endocrine
PGS000761	EFO_0004705	hypothyroidism	hypothyroidism	EFO_0004705	Other disease	Endocrine
PGS000820	EFO_0004705	hypothyroidism	hypothyroidism	EFO_0004705	Other disease	Endocrine
PGS001181	EFO_0004705	hypothyroidism	hypothyroidism	EFO_0004705	Other disease	Endocrine
PGS001816	EFO_0004705	hypothyroidism	hypothyroidism	EFO_0004705	Other disease	Endocrine
PGS002024	EFO_0004705	hypothyroidism	hypothyroidism	EFO_0004705	Other disease	Endocrine
PGS002336	EFO_0004705	hypothyroidism	hypothyroidism	EFO_0004705	Other disease	Endocrine
PGS002408	EFO_0004705	hypothyroidism	hypothyroidism	EFO_0004705	Other disease	Endocrine
PGS002457	EFO_0004705	hypothyroidism	hypothyroidism	EFO_0004705	Other disease	Endocrine
PGS002506	EFO_0004705	hypothyroidism	hypothyroidism	EFO_0004705	Other disease	Endocrine
PGS002555	EFO_0004705	hypothyroidism	hypothyroidism	EFO_0004705	Other disease	Endocrine
PGS002604	EFO_0004705	hypothyroidism	hypothyroidism	EFO_0004705	Other disease	Endocrine
PGS002766	EFO_0004705	hypothyroidism	hypothyroidism	EFO_0004705	Other disease	Endocrine
PGS000762	EFO_0005782	age-related hearing impairment	age-related hearing impairment	EFO_0005782	Neurological disorder	Neurological
PGS000763	EFO_0005782	age-related hearing impairment	age-related hearing impairment	EFO_0005782	Neurological disorder	Neurological
PGS000767	EFO_0003761	unipolar depression|depressive symptom measurement	unipolar depression	EFO_0003761	Neurological disorder	Neurological
PGS002759	EFO_0003761	unipolar depression	unipolar depression	EFO_0003761	Neurological disorder	Neurological
PGS000768	EFO_0004682	QT interval	QT interval	EFO_0004682	Cardiovascular measurement	Cardiovascular
PGS001526	EFO_0004682	QT interval	QT interval	EFO_0004682	Cardiovascular measurement	Cardiovascular
PGS001527	EFO_0004682	QT interval	QT interval	EFO_0004682	Cardiovascular measurement	Cardiovascular
PGS001905	EFO_0004682	QT interval	QT interval	EFO_0004682	Cardiovascular measurement	Cardiovascular
PGS001906	EFO_0004682	QT interval	QT interval	EFO_0004682	Cardiovascular measurement	Cardiovascular
PGS002119	EFO_0004682	QT interval	QT interval	EFO_0004682	Cardiovascular measurement	Cardiovascular
PGS002120	EFO_0004682	QT interval	QT interval	EFO_0004682	Cardiovascular measurement	Cardiovascular
PGS002276	EFO_0004682	QT interval	QT interval	EFO_0004682	Cardiovascular measurement	Cardiovascular
PGS000777	EFO_0007710	cognitive decline measurement|Parkinson disease	cognitive decline measurement	EFO_0007710	Other measurement	Neurological
PGS000780	MONDO_0005271	allergic disease	allergic disease	MONDO_0005271	Immune system disorder	Immunological
PGS000810	MONDO_0005271	allergic disease	allergic disease	MONDO_0005271	Immune system disorder	Immunological
PGS001283	MONDO_0005271	allergic disease	allergic disease	MONDO_0005271	Immune system disorder	Immunological
PGS001284	MONDO_0005271	allergic disease	allergic disease	MONDO_0005271	Immune system disorder	Immunological

PGS001285	MONDO_0005271	allergic disease	allergic disease	MONDO_0005271	Immune system disorder	Immunological
PGS001286	MONDO_0005271	allergic disease	allergic disease	MONDO_0005271	Immune system disorder	Immunological
PGS001372	MONDO_0005271	allergic disease	allergic disease	MONDO_0005271	Immune system disorder	Immunological
PGS000781	EFO_0003833	brain neoplasm|glioma	brain neoplasm	EFO_0003833	Cancer	Neoplasms
PGS000781	EFO_0003833	brain neoplasm|glioma	brain neoplasm	EFO_0003833	Neurological disorder	Neurological
PGS000792	EFO_0005577	pharynx cancer|oral cavity cancer	pharynx cancer	EFO_0005577	Cancer	Neoplasms
PGS000792	EFO_0005577	pharynx cancer|oral cavity cancer	pharynx cancer	EFO_0005577	Digestive system disorder	Gastrointestinal
PGS000819	EFO_0003770	diabetic retinopathy	diabetic retinopathy	EFO_0003770	Cardiovascular disease	Cardiovascular
PGS000819	EFO_0003770	diabetic retinopathy	diabetic retinopathy	EFO_0003770	Metabolic disorder	Metabolic
PGS000819	EFO_0003770	diabetic retinopathy	diabetic retinopathy	EFO_0003770	Neurological disorder	Neurological
PGS000862	EFO_0003770	diabetic retinopathy	diabetic retinopathy	EFO_0003770	Cardiovascular disease	Cardiovascular
PGS000862	EFO_0003770	diabetic retinopathy	diabetic retinopathy	EFO_0003770	Metabolic disorder	Metabolic
PGS000862	EFO_0003770	diabetic retinopathy	diabetic retinopathy	EFO_0003770	Neurological disorder	Neurological
PGS001819	EFO_0003770	diabetic retinopathy	diabetic retinopathy	EFO_0003770	Cardiovascular disease	Cardiovascular
PGS001819	EFO_0003770	diabetic retinopathy	diabetic retinopathy	EFO_0003770	Metabolic disorder	Metabolic
PGS001819	EFO_0003770	diabetic retinopathy	diabetic retinopathy	EFO_0003770	Neurological disorder	Neurological
PGS002027	EFO_0003770	diabetic retinopathy	diabetic retinopathy	EFO_0003770	Cardiovascular disease	Cardiovascular
PGS002027	EFO_0003770	diabetic retinopathy	diabetic retinopathy	EFO_0003770	Metabolic disorder	Metabolic
PGS002027	EFO_0003770	diabetic retinopathy	diabetic retinopathy	EFO_0003770	Neurological disorder	Neurological
PGS000822	EFO_0007778	urinary albumin to creatinine ratio	urinary albumin to creatinine ratio	EFO_0007778	Other measurement	Metabolic
PGS001107	EFO_0007778	urinary albumin to creatinine ratio	urinary albumin to creatinine ratio	EFO_0007778	Other measurement	Metabolic
PGS000827	EFO_0004342	waist circumference|male	waist circumference	EFO_0004342	Body measurement	Body measurement
PGS000828	EFO_0004342	waist circumference|female	waist circumference	EFO_0004342	Body measurement	Body measurement
PGS001227	EFO_0004342	waist circumference	waist circumference	EFO_0004342	Body measurement	Body measurement
PGS001983	EFO_0004342	waist circumference	waist circumference	EFO_0004342	Body measurement	Body measurement
PGS002201	EFO_0004342	waist circumference	waist circumference	EFO_0004342	Body measurement	Body measurement
PGS000834	EFO_0008473	insulin response measurement	insulin response measurement	EFO_0008473	Other measurement	Metabolic
PGS000835	EFO_0008001	insulin secretion measurement	insulin secretion measurement	EFO_0008001	Other measurement	Metabolic
PGS000871	EFO_0008001	insulin secretion measurement	insulin secretion measurement	EFO_0008001	Other measurement	Metabolic
PGS000837	EFO_0004471	insulin sensitivity measurement	insulin sensitivity measurement	EFO_0004471	Other measurement	Metabolic
PGS000839	EFO_0004307	glucose tolerance test	glucose tolerance test	EFO_0004307	Other measurement	Metabolic
PGS000840	EFO_0010814	proinsulin measurement	proinsulin measurement	EFO_0010814	Other measurement	Metabolic
PGS000842	EFO_0004343	waist-hip ratio	waist-hip ratio	EFO_0004343	Body measurement	Body measurement
PGS002356	EFO_0004343	waist-hip ratio	waist-hip ratio	EFO_0004343	Body measurement	Body measurement
PGS002428	EFO_0004343	waist-hip ratio	waist-hip ratio	EFO_0004343	Body measurement	Body measurement
PGS002477	EFO_0004343	waist-hip ratio	waist-hip ratio	EFO_0004343	Body measurement	Body measurement
PGS002575	EFO_0004343	waist-hip ratio	waist-hip ratio	EFO_0004343	Body measurement	Body measurement
PGS002624	EFO_0004343	waist-hip ratio	waist-hip ratio	EFO_0004343	Body measurement	Body measurement
PGS002673	EFO_0004343	waist-hip ratio	waist-hip ratio	EFO_0004343	Body measurement	Body measurement
PGS000844	EFO_0004765	visceral adipose tissue measurement	visceral adipose tissue measurement	EFO_0004765	Other measurement	Metabolic
PGS002813	EFO_0004765	visceral adipose tissue measurement	visceral adipose tissue measurement	EFO_0004765	Other measurement	Metabolic
PGS000865	EFO_0010821	liver fat measurement	liver fat measurement	EFO_0010821	Other measurement	Gastrointestinal
PGS000866	EFO_0010821	liver fat measurement	liver fat measurement	EFO_0010821	Other measurement	Gastrointestinal
PGS002245	EFO_0010821	liver fat measurement	liver fat measurement	EFO_0010821	Other measurement	Gastrointestinal
PGS000872	EFO_0003095	non-alcoholic fatty liver disease	non-alcoholic fatty liver disease	EFO_0003095	Digestive system disorder	Gastrointestinal

PGS002282	EFO_0003095	non-alcoholic fatty liver disease	non-alcoholic fatty liver disease	EFO_0003095	Digestive system disorder	Gastrointestinal
PGS002283	EFO_0003095	non-alcoholic fatty liver disease	non-alcoholic fatty liver disease	EFO_0003095	Digestive system disorder	Gastrointestinal
PGS000877	EFO_0002614	insulin resistance	insulin resistance	EFO_0002614	Other trait	Metabolic
PGS000906	EFO_0004300	longevity	longevity	EFO_0004300	Other measurement	Mortality
PGS002795	EFO_0004300	longevity	longevity	EFO_0004300	Other measurement	Mortality
PGS000922	MONDO_0019338	sarcoidosis	sarcoidosis	MONDO_0019338	Other disease	Others
PGS000923	MONDO_0019338	sarcoidosis	sarcoidosis	MONDO_0019338	Other disease	Others
PGS000924	MONDO_0037821	porphyrin metabolism disease|bilirubin metabolism disease	porphyrin metabolism disease	MONDO_0037821	Metabolic disorder	Metabolic
PGS000925	MONDO_0024355	respiratory tract infectious disorder	respiratory tract infectious disorder	MONDO_0024355	Other disease	Skeletal
PGS000926	MONDO_0021107	narcolepsy	narcolepsy	MONDO_0021107	Neurological disorder	Neurological
PGS001995	MONDO_0021107	narcolepsy	narcolepsy	MONDO_0021107	Neurological disorder	Neurological
PGS002212	MONDO_0021107	narcolepsy	narcolepsy	MONDO_0021107	Neurological disorder	Neurological
PGS000927	MONDO_0002406	dermatitis	dermatitis	MONDO_0002406	Other disease	Dermatological
PGS000928	MONDO_0001658	nontoxic goiter	nontoxic goiter	MONDO_0001658	Other disease	Endocrine
PGS000929	MONDO_0001627	dementia	dementia	MONDO_0001627	Neurological disorder	Neurological
PGS001827	MONDO_0001627	dementia	dementia	MONDO_0001627	Neurological disorder	Neurological
PGS002035	MONDO_0001627	dementia	dementia	MONDO_0001627	Neurological disorder	Neurological
PGS000930	MONDO_0000831	thrombotic disease	thrombotic disease	MONDO_0000831	Cardiovascular disease	Cardiovascular
PGS000931	MONDO_0000831	thrombotic disease|deep vein thrombosis	thrombotic disease	MONDO_0000831	Cardiovascular disease	Cardiovascular
PGS000932	MONDO_0000812	vertebral column disorder	vertebral column disorder	MONDO_0000812	Other disease	Skeletal
PGS000933	HP_0100582	Nasal polyposis	Nasal polyposis	HP_0100582	Other trait	Ear, Nose, Throat
PGS000934	HP_0100582	Nasal polyposis	Nasal polyposis	HP_0100582	Other trait	Ear, Nose, Throat
PGS001848	HP_0100582	Nasal polyposis	Nasal polyposis	HP_0100582	Other trait	Ear, Nose, Throat
PGS002060	HP_0100582	Nasal polyposis	Nasal polyposis	HP_0100582	Other trait	Ear, Nose, Throat
PGS000935	HP_0032263	Increased blood pressure|age at diagnosis	Increased blood pressure	HP_0032263	Other trait	Cardiovascular
PGS000936	HP_0003124	Hypercholesterolemia	Hypercholesterolemia	HP_0003124	Other trait	Metabolic
PGS002334	HP_0003124	Hypercholesterolemia	Hypercholesterolemia	HP_0003124	Other trait	Metabolic
PGS002406	HP_0003124	Hypercholesterolemia	Hypercholesterolemia	HP_0003124	Other trait	Metabolic
PGS002455	HP_0003124	Hypercholesterolemia	Hypercholesterolemia	HP_0003124	Other trait	Metabolic
PGS002504	HP_0003124	Hypercholesterolemia	Hypercholesterolemia	HP_0003124	Other trait	Metabolic
PGS002553	HP_0003124	Hypercholesterolemia	Hypercholesterolemia	HP_0003124	Other trait	Metabolic
PGS002602	HP_0003124	Hypercholesterolemia	Hypercholesterolemia	HP_0003124	Other trait	Metabolic
PGS002651	HP_0003124	Hypercholesterolemia	Hypercholesterolemia	HP_0003124	Other trait	Metabolic

PGS002764	HP_0003124	Hypercholesterolemia	Hypercholesterolemia	HP_0003124	Other trait	Metabolic
PGS000937	HP_0002619	Varicose veins	Varicose veins	HP_0002619	Other trait	Cardiovascular
PGS000938	HP_0002619	Varicose veins	Varicose veins	HP_0002619	Other trait	Cardiovascular
PGS001845	HP_0002619	Varicose veins	Varicose veins	HP_0002619	Other trait	Cardiovascular
PGS002057	HP_0002619	Varicose veins	Varicose veins	HP_0002619	Other trait	Cardiovascular
PGS000939	HP_0002036	Hiatus hernia	Hiatus hernia	HP_0002036	Other trait	Gastrointestinal
PGS000940	HP_0002024	Malabsorption	Malabsorption	HP_0002024	Other trait	Gastrointestinal
PGS000944	HP_0000964	Eczema|dermatitis	Eczema	HP_0000964	Other trait	Dermatological
PGS002363	HP_0000964	Eczema	Eczema	HP_0000964	Other trait	Dermatological
PGS002395	HP_0000964	Eczema	Eczema	HP_0000964	Other trait	Dermatological
PGS002444	HP_0000964	Eczema	Eczema	HP_0000964	Other trait	Dermatological
PGS002493	HP_0000964	Eczema	Eczema	HP_0000964	Other trait	Dermatological
PGS002542	HP_0000964	Eczema	Eczema	HP_0000964	Other trait	Dermatological
PGS002591	HP_0000964	Eczema	Eczema	HP_0000964	Other trait	Dermatological
PGS002640	HP_0000964	Eczema	Eczema	HP_0000964	Other trait	Dermatological
PGS000945	HP_0000726	Dementia|Alzheimer disease	Dementia	HP_0000726	Other trait	Psychiatric
PGS000946	HP_0000726	Dementia	Dementia	HP_0000726	Other trait	Psychiatric
PGS003334	HP_0000726	Dementia	Dementia	HP_0000726	Other trait	Psychiatric
PGS000947	HP_0000155	Oral ulcer	Oral ulcer	HP_0000155	Other trait	Ear, Nose, Throat
PGS000948	HP_0000023	Inguinal hernia	Inguinal hernia	HP_0000023	Other trait	Gastrointestinal
PGS000949	HP_0000023	Inguinal hernia	Inguinal hernia	HP_0000023	Other trait	Gastrointestinal
PGS001854	HP_0000023	Inguinal hernia	Inguinal hernia	HP_0000023	Other trait	Gastrointestinal
PGS002065	HP_0000023	Inguinal hernia	Inguinal hernia	HP_0000023	Other trait	Gastrointestinal
PGS000950	GO_0009314	response to radiation	response to radiation	GO_0009314	Biological process	Others
PGS000951	EFO_0004514	bone quantitative ultrasound measurement	bone quantitative ultrasound measurement	EFO_0004514	Other measurement	Skeletal
PGS000952	EFO_0004514	bone quantitative ultrasound measurement	bone quantitative ultrasound measurement	EFO_0004514	Other measurement	Skeletal
PGS000953	EFO_0004514	bone quantitative ultrasound measurement	bone quantitative ultrasound measurement	EFO_0004514	Other measurement	Skeletal
PGS000954	EFO_0004514	bone quantitative ultrasound measurement	bone quantitative ultrasound measurement	EFO_0004514	Other measurement	Skeletal
PGS000955	EFO_0004514	bone quantitative ultrasound measurement	bone quantitative ultrasound measurement	EFO_0004514	Other measurement	Skeletal
PGS000956	EFO_0004514	bone quantitative ultrasound measurement	bone quantitative ultrasound measurement	EFO_0004514	Other measurement	Skeletal
PGS000957	MONDO_0001134	essential hypertension	essential hypertension	MONDO_0001134	Cardiovascular disease	Cardiovascular
PGS000958	MONDO_0001134	essential hypertension	essential hypertension	MONDO_0001134	Cardiovascular disease	Cardiovascular
PGS000959	EFO_1001986	connective tissue disease	connective tissue disease	EFO_1001986	Other disease	Connective Tissue
PGS000960	EFO_1001986	connective tissue disease	connective tissue disease	EFO_1001986	Other disease	Connective Tissue
PGS000961	EFO_1001395	Phlebitis|Thrombophlebitis	Phlebitis	EFO_1001395	Cardiovascular disease	Cardiovascular
PGS001844	EFO_1001395	Phlebitis|Thrombophlebitis	Phlebitis	EFO_1001395	Cardiovascular disease	Cardiovascular
PGS002056	EFO_1001395	Phlebitis|Thrombophlebitis	Phlebitis	EFO_1001395	Cardiovascular disease	Cardiovascular
PGS000962	EFO_1001375	Myocardial Ischemia	Myocardial Ischemia	EFO_1001375	Cardiovascular disease	Cardiovascular
PGS000963	EFO_1001329	Follicular Cyst	Follicular Cyst	EFO_1001329	Other disease	Genitourinary
PGS000964	EFO_1001317	Epiphyses, Slipped	Epiphyses, Slipped	EFO_1001317	Other disease	Skeletal
PGS000965	EFO_1001055	myxedema|hypothyroidism	myxedema	EFO_1001055	Other disease	Endocrine
PGS000967	EFO_1000786	osteoarthritis, hip	osteoarthritis, hip	EFO_1000786	Other disease	Skeletal
PGS002728	EFO_1000786	osteoarthritis, hip	osteoarthritis, hip	EFO_1000786	Other disease	Skeletal
PGS002763	EFO_1000786	osteoarthritis, hip	osteoarthritis, hip	EFO_1000786	Other disease	Skeletal
PGS000969	EFO_0011011	sitting height measurement	sitting height measurement	EFO_0011011	Other measurement	Skeletal
PGS002005	EFO_0011011	sitting height measurement	sitting height measurement	EFO_0011011	Other measurement	Skeletal
PGS002224	EFO_0011011	sitting height measurement	sitting height measurement	EFO_0011011	Other measurement	Skeletal
PGS000976	EFO_0010907	polyomavirus 2 seropositivity	polyomavirus 2 seropositivity	EFO_0010907	Other measurement	Infection

PGS000977	EFO_0010906	merkel cell virus seropositivity	merkel cell virus seropositivity	EFO_0010906	Other measurement	Infection
PGS000978	EFO_0010811	carbohydrate intake measurement	carbohydrate intake measurement	EFO_0010811	Other measurement	Metabolic
PGS000980	EFO_0010729	sun exposure measurement	sun exposure measurement	EFO_0010729	Other measurement	Activities
PGS000982	EFO_0010729	sun exposure measurement	sun exposure measurement	EFO_0010729	Other measurement	Activities
PGS001535	EFO_0010729	sun exposure measurement	sun exposure measurement	EFO_0010729	Other measurement	Activities
PGS001993	EFO_0010729	sun exposure measurement	sun exposure measurement	EFO_0010729	Other measurement	Activities
PGS002210	EFO_0010729	sun exposure measurement	sun exposure measurement	EFO_0010729	Other measurement	Activities
PGS000984	EFO_0010724	lifestyle measurement	lifestyle measurement	EFO_0010724	Other measurement	Activities
PGS001923	EFO_0010724	lifestyle measurement	lifestyle measurement	EFO_0010724	Other measurement	Activities
PGS001999	EFO_0010724	lifestyle measurement	lifestyle measurement	EFO_0010724	Other measurement	Activities
PGS002139	EFO_0010724	lifestyle measurement	lifestyle measurement	EFO_0010724	Other measurement	Activities
PGS002217	EFO_0010724	lifestyle measurement	lifestyle measurement	EFO_0010724	Other measurement	Activities
PGS000988	EFO_0010700	reticulocyte measurement	reticulocyte measurement	EFO_0010700	Hematological measurement	Hematological
PGS000989	EFO_0010700	reticulocyte measurement	reticulocyte measurement	EFO_0010700	Hematological measurement	Hematological
PGS001406	EFO_0010700	reticulocyte measurement	reticulocyte measurement	EFO_0010700	Hematological measurement	Hematological
PGS001959	EFO_0010700	reticulocyte measurement	reticulocyte measurement	EFO_0010700	Hematological measurement	Hematological
PGS002177	EFO_0010700	reticulocyte measurement	reticulocyte measurement	EFO_0010700	Hematological measurement	Hematological
PGS000990	EFO_0010698	retinal break|retinal detachment	retinal break	EFO_0010698	Neurological disorder	Neurological
PGS000991	EFO_0010158	sugar consumption measurement	sugar consumption measurement	EFO_0010158	Biological process	Metabolic
PGS000993	EFO_0010139	fish consumption measurement	fish consumption measurement	EFO_0010139	Other measurement	Activities
PGS000994	EFO_0010091	tea consumption measurement	tea consumption measurement	EFO_0010091	Other measurement	Activities
PGS000995	EFO_0010078	dentures	dentures	EFO_0010078	Other trait	Others
PGS000996	EFO_0009959	diverticular disease|diverticulitis	diverticular disease	EFO_0009959	Digestive system disorder	Gastrointestinal
PGS000997	EFO_0009959	diverticular disease	diverticular disease	EFO_0009959	Digestive system disorder	Gastrointestinal
PGS001000	EFO_0009818	nap during day, self-reported	nap during day, self-reported	EFO_0009818	Other trait	Activities
PGS001001	EFO_0009817	ease of getting up in the morning, self-reported	ease of getting up in the morning, self-reported	EFO_0009817	Other trait	Activities
PGS001002	EFO_0009805	whole body water mass	whole body water mass	EFO_0009805	Other measurement	Body measurement
PGS001984	EFO_0009805	whole body water mass	whole body water mass	EFO_0009805	Other measurement	Body measurement
PGS002202	EFO_0009805	whole body water mass	whole body water mass	EFO_0009805	Other measurement	Body measurement
PGS001003	EFO_0009803	number of treatments or medications taken, self-reported	number of treatments or medications taken, self-reported	EFO_0009803	Other trait	Activities
PGS001004	EFO_0009801	number of non-cancer illnesses, self-reported	number of non-cancer illnesses, self-reported	EFO_0009801	Other trait	Activities
PGS001005	EFO_0009800	number of cancers, self-reported	number of cancers, self-reported	EFO_0009800	Other trait	Activities
PGS001007	EFO_0009749	age at first sexual intercourse measurement	age at first sexual intercourse measurement	EFO_0009749	Other measurement	Genitourinary
PGS001938	EFO_0009749	age at first sexual intercourse measurement	age at first sexual intercourse measurement	EFO_0009749	Other measurement	Genitourinary
PGS002156	EFO_0009749	age at first sexual intercourse measurement	age at first sexual intercourse measurement	EFO_0009749	Other measurement	Genitourinary
PGS002215	EFO_0009749	age at first sexual intercourse measurement	age at first sexual intercourse measurement	EFO_0009749	Other measurement	Genitourinary
PGS001008	EFO_0009724	health satisfaction measurement	health satisfaction measurement	EFO_0009724	Other measurement	Activities
PGS001401	EFO_0009724	health satisfaction measurement	health satisfaction measurement	EFO_0009724	Other measurement	Activities
PGS001935	EFO_0009724	health satisfaction measurement	health satisfaction measurement	EFO_0009724	Other measurement	Activities
PGS002153	EFO_0009724	health satisfaction measurement	health satisfaction measurement	EFO_0009724	Other measurement	Activities
PGS001009	EFO_0009718	peak expiratory flow	peak expiratory flow	EFO_0009718	Other measurement	Respiratory
PGS001010	EFO_0009718	peak expiratory flow	peak expiratory flow	EFO_0009718	Other measurement	Respiratory

PGS001011	EFO_0009662	common wart	common wart	EFO_0009662	Cancer	Neoplasms
PGS001012	EFO_0009631	fall	fall	EFO_0009631	Other trait	Others
PGS001916	EFO_0009631	fall	fall	EFO_0009631	Other trait	Others
PGS002132	EFO_0009631	fall	fall	EFO_0009631	Other trait	Others
PGS001013	EFO_0009606	macular degeneration	macular degeneration	EFO_0009606	Neurological disorder	Neurological
PGS001014	EFO_0009605	pancreas disease	pancreas disease	EFO_0009605	Digestive system disorder	Gastrointestinal
PGS001015	EFO_0009602	prostate disease	prostate disease	EFO_0009602	Other disease	Genitourinary
PGS001016	EFO_0009599	feeling emotionally hurt measurement	feeling emotionally hurt measurement	EFO_0009599	Other measurement	Psychiatric
PGS001017	EFO_0009597	feeling nervous measurement	feeling nervous measurement	EFO_0009597	Other measurement	Psychiatric
PGS001018	EFO_0009592	social interaction measurement	social interaction measurement	EFO_0009592	Other measurement	Social Interactions
PGS001019	EFO_0009592	social interaction measurement	social interaction measurement	EFO_0009592	Other measurement	Social Interactions
PGS001020	EFO_0009592	social interaction measurement	social interaction measurement	EFO_0009592	Other measurement	Social Interactions
PGS001021	EFO_0009589	worry measurement|anxiety measurement	worry measurement	EFO_0009589	Other measurement	Psychiatric
PGS001022	EFO_0009589	worry measurement	worry measurement	EFO_0009589	Other measurement	Psychiatric
PGS001920	EFO_0009589	worry measurement	worry measurement	EFO_0009589	Other measurement	Psychiatric
PGS002136	EFO_0009589	worry measurement	worry measurement	EFO_0009589	Other measurement	Psychiatric
PGS001023	EFO_0009556	mineral metabolism disease	mineral metabolism disease	EFO_0009556	Metabolic disorder	Metabolic
PGS001024	EFO_0009552	hemorrhoid	hemorrhoid	EFO_0009552	Cardiovascular disease	Cardiovascular
PGS001846	EFO_0009552	hemorrhoid	hemorrhoid	EFO_0009552	Cardiovascular disease	Cardiovascular
PGS002058	EFO_0009552	hemorrhoid	hemorrhoid	EFO_0009552	Cardiovascular disease	Cardiovascular
PGS001025	EFO_0009531	aortic valve disease	aortic valve disease	EFO_0009531	Cardiovascular disease	Cardiovascular
PGS001026	EFO_0009514	upper extremity fracture	upper extremity fracture	EFO_0009514	Other disease	Skeletal
PGS001027	EFO_0009507	knee injury	knee injury	EFO_0009507	Other disease	Others
PGS001028	EFO_0009486	diabetic eye disease	diabetic eye disease	EFO_0009486	Other disease	Metabolic
PGS001029	EFO_0009464	corneal disease	corneal disease	EFO_0009464	Other disease	Ophthalmological
PGS001030	EFO_0009448	pulmonary fibrosis	pulmonary fibrosis	EFO_0009448	Cancer	Neoplasms
PGS001031	EFO_0009432	fibroblastic disorder	fibroblastic disorder	EFO_0009432	Other disease	Connective Tissue
PGS001032	EFO_0000731	uterine fibroid	uterine fibroid	EFO_0000731	Cancer	Neoplasms
PGS002263	EFO_0000731	uterine fibroid	uterine fibroid	EFO_0000731	Cancer	Neoplasms
PGS001033	EFO_0009314	blood coagulation disease	blood coagulation disease	EFO_0009314	Other disease	Hematological
PGS001034	EFO_0008111	diet measurement	diet measurement	EFO_0008111	Other measurement	Activities
PGS001056	EFO_0008111	diet measurement	diet measurement	EFO_0008111	Other measurement	Activities
PGS001057	EFO_0008111	diet measurement	diet measurement	EFO_0008111	Other measurement	Activities
PGS001058	EFO_0008111	diet measurement	diet measurement	EFO_0008111	Other measurement	Activities
PGS001059	EFO_0008111	diet measurement	diet measurement	EFO_0008111	Other measurement	Activities
PGS001060	EFO_0008111	diet measurement	diet measurement	EFO_0008111	Other measurement	Activities
PGS001061	EFO_0008111	diet measurement	diet measurement	EFO_0008111	Other measurement	Activities
PGS001062	EFO_0008111	diet measurement	diet measurement	EFO_0008111	Other measurement	Activities
PGS001063	EFO_0008111	diet measurement	diet measurement	EFO_0008111	Other measurement	Activities
PGS001064	EFO_0008111	diet measurement	diet measurement	EFO_0008111	Other measurement	Activities
PGS001065	EFO_0008111	diet measurement	diet measurement	EFO_0008111	Other measurement	Activities
PGS001066	EFO_0008111	diet measurement	diet measurement	EFO_0008111	Other measurement	Activities
PGS001067	EFO_0008111	diet measurement	diet measurement	EFO_0008111	Other measurement	Activities
PGS001068	EFO_0008111	diet measurement	diet measurement	EFO_0008111	Other measurement	Activities
PGS001069	EFO_0008111	diet measurement	diet measurement	EFO_0008111	Other measurement	Activities
PGS001389	EFO_0008111	diet measurement	diet measurement	EFO_0008111	Other measurement	Activities
PGS001518	EFO_0008111	diet measurement	diet measurement	EFO_0008111	Other measurement	Activities
PGS001036	EFO_0009271	Epstein Barr virus nuclear antigen-1 seropositivity	Epstein Barr virus nuclear antigen-1 seropositivity	EFO_0009271	Other measurement	Infection

PGS001037	EFO_0009270	heel bone mineral density	heel bone mineral density	EFO_0009270	Other measurement	Skeletal
PGS001038	EFO_0009270	heel bone mineral density	heel bone mineral density	EFO_0009270	Other measurement	Skeletal
PGS001039	EFO_0009270	heel bone mineral density	heel bone mineral density	EFO_0009270	Other measurement	Skeletal
PGS001402	EFO_0009270	heel bone mineral density	heel bone mineral density	EFO_0009270	Other measurement	Skeletal
PGS001403	EFO_0009270	heel bone mineral density	heel bone mineral density	EFO_0009270	Other measurement	Skeletal
PGS001404	EFO_0009270	heel bone mineral density	heel bone mineral density	EFO_0009270	Other measurement	Skeletal
PGS001955	EFO_0009270	heel bone mineral density	heel bone mineral density	EFO_0009270	Other measurement	Skeletal
PGS002173	EFO_0009270	heel bone mineral density	heel bone mineral density	EFO_0009270	Other measurement	Skeletal
PGS001040	EFO_0009260	non-melanoma skin carcinoma	non-melanoma skin carcinoma	EFO_0009260	Cancer	Neoplasms
PGS001042	EFO_0009190	Thyrotoxicosis	Thyrotoxicosis	EFO_0009190	Immune system disorder	Immunological
PGS001815	EFO_0009190	Thyrotoxicosis	Thyrotoxicosis	EFO_0009190	Immune system disorder	Immunological
PGS002023	EFO_0009190	Thyrotoxicosis	Thyrotoxicosis	EFO_0009190	Immune system disorder	Immunological
PGS001043	EFO_0009189	hyperthyroidism|Thyrotoxicosis	hyperthyroidism	EFO_0009189	Other disease	Endocrine
PGS001044	EFO_0009116	vitamin supplement exposure measurement	vitamin supplement exposure measurement	EFO_0009116	Other measurement	Activities
PGS001045	EFO_0009116	vitamin supplement exposure measurement	vitamin supplement exposure measurement	EFO_0009116	Other measurement	Activities
PGS001046	EFO_0009115	tobacco smoke exposure measurement	tobacco smoke exposure measurement	EFO_0009115	Other measurement	Psychiatric
PGS001047	EFO_0009115	tobacco smoke exposure measurement	tobacco smoke exposure measurement	EFO_0009115	Other measurement	Psychiatric
PGS001048	EFO_0008586	Non-ST Elevation Myocardial Infarction	Non-ST Elevation Myocardial Infarction	EFO_0008586	Cardiovascular disease	Cardiovascular
PGS001050	EFO_0008561	diaphragmatic hernia	diaphragmatic hernia	EFO_0008561	Other trait	Gastrointestinal
PGS001051	EFO_0008518	polymyalgia rheumatica	polymyalgia rheumatica	EFO_0008518	Other disease	Immunological
PGS001878	EFO_0008518	polymyalgia rheumatica	polymyalgia rheumatica	EFO_0008518	Other disease	Immunological
PGS002091	EFO_0008518	polymyalgia rheumatica	polymyalgia rheumatica	EFO_0008518	Other disease	Immunological
PGS001052	EFO_0600081	time spent outdoors measurement	time spent outdoors measurement	EFO_0600081	Other measurement	Activities
PGS001053	EFO_0600081	time spent outdoors measurement	time spent outdoors measurement	EFO_0600081	Other measurement	Activities
PGS001054	EFO_0008341	snoring measurement	snoring measurement	EFO_0008341	Other measurement	Psychiatric
PGS002006	EFO_0008341	snoring measurement	snoring measurement	EFO_0008341	Other measurement	Psychiatric
PGS002225	EFO_0008341	snoring measurement	snoring measurement	EFO_0008341	Other measurement	Psychiatric
PGS001070	EFO_0008080	cerebrospinal fluid volume measurement	cerebrospinal fluid volume measurement	EFO_0008080	Other measurement	Neurological
PGS001639	EFO_0008080	cerebrospinal fluid volume measurement|neuroimaging measurement	cerebrospinal fluid volume measurement	EFO_0008080	Other measurement	Neurological
PGS001071	EFO_0008006	skin aging measurement	skin aging measurement	EFO_0008006	Other measurement	Dermatological
PGS001072	EFO_0008006	skin aging measurement	skin aging measurement	EFO_0008006	Other measurement	Dermatological
PGS001073	EFO_0008002	physical activity measurement	physical activity measurement	EFO_0008002	Other measurement	Activities
PGS001074	EFO_0008002	physical activity measurement	physical activity measurement	EFO_0008002	Other measurement	Activities
PGS001075	EFO_0008002	physical activity measurement	physical activity measurement	EFO_0008002	Other measurement	Activities
PGS001397	EFO_0008002	physical activity measurement	physical activity measurement	EFO_0008002	Other measurement	Activities
PGS002254	EFO_0008002	physical activity measurement|self-reported trait	physical activity measurement	EFO_0008002	Other measurement	Activities
PGS002255	EFO_0008002	physical activity measurement	physical activity measurement	EFO_0008002	Other measurement	Activities
PGS001080	EFO_0007946	tiredness measurement	tiredness measurement	EFO_0007946	Other measurement	Psychiatric
PGS001090	EFO_0007869	wellbeing measurement	wellbeing measurement	EFO_0007869	Other measurement	Activities
PGS001936	EFO_0007869	wellbeing measurement	wellbeing measurement	EFO_0007869	Other measurement	Activities
PGS002154	EFO_0007869	wellbeing measurement	wellbeing measurement	EFO_0007869	Other measurement	Activities
PGS001091	EFO_0007865	loneliness measurement	loneliness measurement	EFO_0007865	Other measurement	Psychiatric
PGS001092	EFO_0007822	hair colour measurement	hair colour measurement	EFO_0007822	Other measurement	Dermatological
PGS001093	EFO_0007822	hair colour measurement	hair colour measurement	EFO_0007822	Other measurement	Dermatological
PGS001094	EFO_0007822	hair colour measurement	hair colour measurement	EFO_0007822	Other measurement	Dermatological

PGS001095	EFO_0007822	hair colour measurement	hair colour measurement	EFO_0007822	Other measurement	Dermatological
PGS001096	EFO_0007822	hair colour measurement	hair colour measurement	EFO_0007822	Other measurement	Dermatological
PGS001097	EFO_0007822	hair colour measurement	hair colour measurement	EFO_0007822	Other measurement	Dermatological
PGS001098	EFO_0007822	hair colour measurement	hair colour measurement	EFO_0007822	Other measurement	Dermatological
PGS001896	EFO_0007822	hair colour measurement	hair colour measurement	EFO_0007822	Other measurement	Dermatological
PGS002002	EFO_0007822	hair colour measurement	hair colour measurement	EFO_0007822	Other measurement	Dermatological
PGS002109	EFO_0007822	hair colour measurement	hair colour measurement	EFO_0007822	Other measurement	Dermatological
PGS002220	EFO_0007822	hair colour measurement	hair colour measurement	EFO_0007822	Other measurement	Dermatological
PGS002330	EFO_0007822	hair colour measurement	hair colour measurement	EFO_0007822	Other measurement	Dermatological
PGS002402	EFO_0007822	hair colour measurement	hair colour measurement	EFO_0007822	Other measurement	Dermatological
PGS002451	EFO_0007822	hair colour measurement	hair colour measurement	EFO_0007822	Other measurement	Dermatological
PGS002500	EFO_0007822	hair colour measurement	hair colour measurement	EFO_0007822	Other measurement	Dermatological
PGS002549	EFO_0007822	hair colour measurement	hair colour measurement	EFO_0007822	Other measurement	Dermatological
PGS002598	EFO_0007822	hair colour measurement	hair colour measurement	EFO_0007822	Other measurement	Dermatological
PGS002647	EFO_0007822	hair colour measurement	hair colour measurement	EFO_0007822	Other measurement	Dermatological
PGS001099	EFO_0007814	refractive error measurement	refractive error measurement	EFO_0007814	Other measurement	Ophthalmological
PGS001100	EFO_0007814	refractive error measurement	refractive error measurement	EFO_0007814	Other measurement	Ophthalmological
PGS001101	EFO_0007800	body fat percentage	body fat percentage	EFO_0007800	Body measurement	Body measurement
PGS001102	EFO_0007800	body fat percentage	body fat percentage	EFO_0007800	Body measurement	Body measurement
PGS001103	EFO_0007800	body fat percentage	body fat percentage	EFO_0007800	Body measurement	Body measurement
PGS001104	EFO_0007800	body fat percentage	body fat percentage	EFO_0007800	Body measurement	Body measurement
PGS001105	EFO_0007800	body fat percentage	body fat percentage	EFO_0007800	Body measurement	Body measurement
PGS001106	EFO_0007800	body fat percentage	body fat percentage	EFO_0007800	Body measurement	Body measurement
PGS001917	EFO_0007800	body fat percentage	body fat percentage	EFO_0007800	Body measurement	Body measurement
PGS002133	EFO_0007800	body fat percentage	body fat percentage	EFO_0007800	Body measurement	Body measurement
PGS001108	EFO_0007777	base metabolic rate measurement	base metabolic rate measurement	EFO_0007777	Other measurement	Metabolic
PGS001109	EFO_0007533	vasomotor rhinitis|allergic rhinitis	vasomotor rhinitis	EFO_0007533	Immune system disorder	Immunological
PGS001110	EFO_0007460	reactive arthritis	reactive arthritis	EFO_0007460	Immune system disorder	Immunological
PGS001112	EFO_0007013	aspirin use measurement	aspirin use measurement	EFO_0007013	Other measurement	Activities
PGS001113	EFO_0007013	aspirin use measurement	aspirin use measurement	EFO_0007013	Other measurement	Activities
PGS001114	EFO_0007012	NSAID use measurement|self-reported trait	NSAID use measurement	EFO_0007012	Other measurement	Activities
PGS001115	EFO_0007012	NSAID use measurement|self-reported trait	NSAID use measurement	EFO_0007012	Other measurement	Activities
PGS001116	EFO_0007012	NSAID use measurement	NSAID use measurement	EFO_0007012	Other measurement	Activities
PGS001117	EFO_0600082	insulin use measurement	insulin use measurement	EFO_0600082	Other measurement	Metabolic
PGS001118	EFO_0007010	drug use measurement	drug use measurement	EFO_0007010	Other measurement	Activities
PGS001119	EFO_0006941	grip strength measurement	grip strength measurement	EFO_0006941	Other measurement	Muscular
PGS001120	EFO_0006941	grip strength measurement	grip strength measurement	EFO_0006941	Other measurement	Muscular
PGS001927	EFO_0006941	grip strength measurement	grip strength measurement	EFO_0006941	Other measurement	Muscular
PGS002143	EFO_0006941	grip strength measurement	grip strength measurement	EFO_0006941	Other measurement	Muscular
PGS001123	EFO_0006781	coffee consumption measurement	coffee consumption measurement	EFO_0006781	Other measurement	Psychiatric
PGS001124	EFO_0006781	coffee consumption measurement	coffee consumption measurement	EFO_0006781	Other measurement	Psychiatric
PGS001125	EFO_0006781	coffee consumption measurement	coffee consumption measurement	EFO_0006781	Other measurement	Psychiatric
PGS001126	EFO_0006781	coffee consumption measurement	coffee consumption measurement	EFO_0006781	Other measurement	Psychiatric
PGS001127	EFO_0006527	smoking status measurement	smoking status measurement	EFO_0006527	Other measurement	Psychiatric
PGS001128	EFO_0006527	smoking status measurement	smoking status measurement	EFO_0006527	Other measurement	Psychiatric
PGS001129	EFO_0006527	smoking status measurement	smoking status measurement	EFO_0006527	Other measurement	Psychiatric
PGS001911	EFO_0006527	smoking status measurement	smoking status measurement	EFO_0006527	Other measurement	Psychiatric
PGS002126	EFO_0006527	smoking status measurement	smoking status measurement	EFO_0006527	Other measurement	Psychiatric

PGS002348	EFO_0006527	smoking status measurement	smoking status measurement	EFO_0006527	Other measurement	Psychiatric
PGS002375	EFO_0006527	smoking status measurement	smoking status measurement	EFO_0006527	Other measurement	Psychiatric
PGS002420	EFO_0006527	smoking status measurement	smoking status measurement	EFO_0006527	Other measurement	Psychiatric
PGS002469	EFO_0006527	smoking status measurement	smoking status measurement	EFO_0006527	Other measurement	Psychiatric
PGS002518	EFO_0006527	smoking status measurement	smoking status measurement	EFO_0006527	Other measurement	Psychiatric
PGS002567	EFO_0006527	smoking status measurement	smoking status measurement	EFO_0006527	Other measurement	Psychiatric
PGS002616	EFO_0006527	smoking status measurement	smoking status measurement	EFO_0006527	Other measurement	Psychiatric
PGS003044	EFO_0006527	smoking status measurement	smoking status measurement	EFO_0006527	Other measurement	Psychiatric
PGS003045	EFO_0006527	smoking status measurement	smoking status measurement	EFO_0006527	Other measurement	Psychiatric
PGS003046	EFO_0006527	smoking status measurement	smoking status measurement	EFO_0006527	Other measurement	Psychiatric
PGS003047	EFO_0006527	smoking status measurement	smoking status measurement	EFO_0006527	Other measurement	Psychiatric
PGS003048	EFO_0006527	smoking status measurement	smoking status measurement	EFO_0006527	Other measurement	Psychiatric
PGS003049	EFO_0006527	smoking status measurement	smoking status measurement	EFO_0006527	Other measurement	Psychiatric
PGS003050	EFO_0006527	smoking status measurement	smoking status measurement	EFO_0006527	Other measurement	Psychiatric
PGS003051	EFO_0006527	smoking status measurement	smoking status measurement	EFO_0006527	Other measurement	Psychiatric
PGS003052	EFO_0006527	smoking status measurement	smoking status measurement	EFO_0006527	Other measurement	Psychiatric
PGS003053	EFO_0006527	smoking status measurement	smoking status measurement	EFO_0006527	Other measurement	Psychiatric
PGS003054	EFO_0006527	smoking status measurement	smoking status measurement	EFO_0006527	Other measurement	Psychiatric
PGS003055	EFO_0006527	smoking status measurement	smoking status measurement	EFO_0006527	Other measurement	Psychiatric
PGS003056	EFO_0006527	smoking status measurement	smoking status measurement	EFO_0006527	Other measurement	Psychiatric
PGS003057	EFO_0006527	smoking status measurement	smoking status measurement	EFO_0006527	Other measurement	Psychiatric
PGS003058	EFO_0006527	smoking status measurement	smoking status measurement	EFO_0006527	Other measurement	Psychiatric
PGS003059	EFO_0006527	smoking status measurement	smoking status measurement	EFO_0006527	Other measurement	Psychiatric
PGS003060	EFO_0006527	smoking status measurement	smoking status measurement	EFO_0006527	Other measurement	Psychiatric
PGS003061	EFO_0006527	smoking status measurement	smoking status measurement	EFO_0006527	Other measurement	Psychiatric
PGS003062	EFO_0006527	smoking status measurement	smoking status measurement	EFO_0006527	Other measurement	Psychiatric
PGS003063	EFO_0006527	smoking status measurement	smoking status measurement	EFO_0006527	Other measurement	Psychiatric
PGS003064	EFO_0006527	smoking status measurement	smoking status measurement	EFO_0006527	Other measurement	Psychiatric
PGS003065	EFO_0006527	smoking status measurement	smoking status measurement	EFO_0006527	Other measurement	Psychiatric
PGS003066	EFO_0006527	smoking status measurement	smoking status measurement	EFO_0006527	Other measurement	Psychiatric
PGS003067	EFO_0006527	smoking status measurement	smoking status measurement	EFO_0006527	Other measurement	Psychiatric
PGS003068	EFO_0006527	smoking status measurement	smoking status measurement	EFO_0006527	Other measurement	Psychiatric
PGS001130	EFO_0006525	cigarettes per day measurement	cigarettes per day measurement	EFO_0006525	Other measurement	Psychiatric
PGS001515	EFO_0006525	cigarettes per day measurement	cigarettes per day measurement	EFO_0006525	Other measurement	Psychiatric
PGS003368	EFO_0006525	cigarettes per day measurement	cigarettes per day measurement	EFO_0006525	Other measurement	Psychiatric
PGS001131	EFO_0006510	Herpes Zoster	Herpes Zoster	EFO_0006510	Other disease	Infection
PGS001132	EFO_0000493	family history	family history	EFO_0000493	Other trait	Others
PGS001324	EFO_0000493	family history	family history	EFO_0000493	Other trait	Others
PGS001328	EFO_0000493	family history	family history	EFO_0000493	Other trait	Others
PGS001135	EFO_0005856	arthritis	arthritis	EFO_0005856	Other disease	Immunological
PGS001136	EFO_0005803	hematologic disease	hematologic disease	EFO_0005803	Other disease	Hematological
PGS001137	EFO_0005773	retinal detachment	retinal detachment	EFO_0005773	Neurological disorder	Neurological
PGS001833	EFO_0005773	retinal detachment	retinal detachment	EFO_0005773	Neurological disorder	Neurological
PGS002040	EFO_0005773	retinal detachment	retinal detachment	EFO_0005773	Neurological disorder	Neurological
PGS001138	EFO_0005767	rheumatoid factor measurement	rheumatoid factor measurement	EFO_0005767	Other measurement	Connective Tissue
PGS001139	EFO_0005671	smoking behaviour measurement	smoking behaviour measurement	EFO_0005671	Other measurement	Psychiatric
PGS001388	EFO_0005671	smoking behaviour measurement	smoking behaviour measurement	EFO_0005671	Other measurement	Psychiatric
PGS001532	EFO_0005671	smoking behaviour measurement	smoking behaviour measurement	EFO_0005671	Other measurement	Psychiatric
PGS001140	EFO_0005584	seborrheic keratosis	seborrheic keratosis	EFO_0005584	Other disease	Dermatological
PGS002085	EFO_0005584	seborrheic keratosis	seborrheic keratosis	EFO_0005584	Other disease	Dermatological

PGS001141	EFO_0005422	skin aging	skin aging	EFO_0005422	Biological process	Dermatological
PGS001142	EFO_0005420	grey matter volume measurement	grey matter volume measurement	EFO_0005420	Other measurement	Neurological
PGS001143	EFO_0005420	grey matter volume measurement	grey matter volume measurement	EFO_0005420	Other measurement	Neurological
PGS001544	EFO_0005420	grey matter volume measurement|neuroimaging measurement	grey matter volume measurement	EFO_0005420	Other measurement	Neurological
PGS001545	EFO_0005420	grey matter volume measurement|neuroimaging measurement	grey matter volume measurement	EFO_0005420	Other measurement	Neurological
PGS001546	EFO_0005420	grey matter volume measurement|neuroimaging measurement	grey matter volume measurement	EFO_0005420	Other measurement	Neurological
PGS001547	EFO_0005420	grey matter volume measurement|neuroimaging measurement	grey matter volume measurement	EFO_0005420	Other measurement	Neurological
PGS001548	EFO_0005420	grey matter volume measurement|neuroimaging measurement	grey matter volume measurement	EFO_0005420	Other measurement	Neurological
PGS001549	EFO_0005420	grey matter volume measurement|neuroimaging measurement	grey matter volume measurement	EFO_0005420	Other measurement	Neurological
PGS001550	EFO_0005420	grey matter volume measurement|neuroimaging measurement	grey matter volume measurement	EFO_0005420	Other measurement	Neurological
PGS001551	EFO_0005420	grey matter volume measurement|neuroimaging measurement	grey matter volume measurement	EFO_0005420	Other measurement	Neurological
PGS001552	EFO_0005420	grey matter volume measurement|neuroimaging measurement	grey matter volume measurement	EFO_0005420	Other measurement	Neurological
PGS001553	EFO_0005420	grey matter volume measurement|neuroimaging measurement	grey matter volume measurement	EFO_0005420	Other measurement	Neurological
PGS001554	EFO_0005420	grey matter volume measurement|neuroimaging measurement	grey matter volume measurement	EFO_0005420	Other measurement	Neurological
PGS001555	EFO_0005420	grey matter volume measurement|neuroimaging measurement	grey matter volume measurement	EFO_0005420	Other measurement	Neurological
PGS001556	EFO_0005420	grey matter volume measurement|neuroimaging measurement	grey matter volume measurement	EFO_0005420	Other measurement	Neurological
PGS001557	EFO_0005420	grey matter volume measurement|neuroimaging measurement	grey matter volume measurement	EFO_0005420	Other measurement	Neurological
PGS001558	EFO_0005420	grey matter volume measurement|neuroimaging measurement	grey matter volume measurement	EFO_0005420	Other measurement	Neurological
PGS001559	EFO_0005420	grey matter volume measurement|neuroimaging measurement	grey matter volume measurement	EFO_0005420	Other measurement	Neurological
PGS001560	EFO_0005420	grey matter volume measurement|neuroimaging measurement	grey matter volume measurement	EFO_0005420	Other measurement	Neurological
PGS001561	EFO_0005420	grey matter volume measurement|neuroimaging measurement	grey matter volume measurement	EFO_0005420	Other measurement	Neurological
PGS001562	EFO_0005420	grey matter volume measurement|neuroimaging measurement	grey matter volume measurement	EFO_0005420	Other measurement	Neurological
PGS001563	EFO_0005420	grey matter volume measurement|neuroimaging measurement	grey matter volume measurement	EFO_0005420	Other measurement	Neurological
PGS001564	EFO_0005420	grey matter volume measurement|neuroimaging measurement	grey matter volume measurement	EFO_0005420	Other measurement	Neurological
PGS001565	EFO_0005420	grey matter volume measurement|neuroimaging measurement	grey matter volume measurement	EFO_0005420	Other measurement	Neurological
PGS001566	EFO_0005420	grey matter volume measurement|neuroimaging measurement	grey matter volume measurement	EFO_0005420	Other measurement	Neurological

PGS001567	EFO_0005420	grey matter volume measurement|neuroimaging measurement	grey matter volume measurement	EFO_0005420	Other measurement	Neurological
PGS001568	EFO_0005420	grey matter volume measurement|neuroimaging measurement	grey matter volume measurement	EFO_0005420	Other measurement	Neurological
PGS001569	EFO_0005420	grey matter volume measurement|neuroimaging measurement	grey matter volume measurement	EFO_0005420	Other measurement	Neurological
PGS001570	EFO_0005420	grey matter volume measurement|neuroimaging measurement	grey matter volume measurement	EFO_0005420	Other measurement	Neurological
PGS001571	EFO_0005420	grey matter volume measurement|neuroimaging measurement	grey matter volume measurement	EFO_0005420	Other measurement	Neurological
PGS001572	EFO_0005420	grey matter volume measurement|neuroimaging measurement	grey matter volume measurement	EFO_0005420	Other measurement	Neurological
PGS001573	EFO_0005420	grey matter volume measurement|neuroimaging measurement	grey matter volume measurement	EFO_0005420	Other measurement	Neurological
PGS001574	EFO_0005420	grey matter volume measurement|neuroimaging measurement	grey matter volume measurement	EFO_0005420	Other measurement	Neurological
PGS001575	EFO_0005420	grey matter volume measurement|neuroimaging measurement	grey matter volume measurement	EFO_0005420	Other measurement	Neurological
PGS001576	EFO_0005420	grey matter volume measurement|neuroimaging measurement	grey matter volume measurement	EFO_0005420	Other measurement	Neurological
PGS001577	EFO_0005420	grey matter volume measurement|neuroimaging measurement	grey matter volume measurement	EFO_0005420	Other measurement	Neurological
PGS001578	EFO_0005420	grey matter volume measurement|neuroimaging measurement	grey matter volume measurement	EFO_0005420	Other measurement	Neurological
PGS001579	EFO_0005420	grey matter volume measurement|neuroimaging measurement	grey matter volume measurement	EFO_0005420	Other measurement	Neurological
PGS001580	EFO_0005420	grey matter volume measurement|neuroimaging measurement	grey matter volume measurement	EFO_0005420	Other measurement	Neurological
PGS001581	EFO_0005420	grey matter volume measurement|neuroimaging measurement	grey matter volume measurement	EFO_0005420	Other measurement	Neurological
PGS001582	EFO_0005420	grey matter volume measurement|neuroimaging measurement	grey matter volume measurement	EFO_0005420	Other measurement	Neurological
PGS001583	EFO_0005420	grey matter volume measurement|neuroimaging measurement	grey matter volume measurement	EFO_0005420	Other measurement	Neurological
PGS001584	EFO_0005420	grey matter volume measurement|neuroimaging measurement	grey matter volume measurement	EFO_0005420	Other measurement	Neurological
PGS001585	EFO_0005420	grey matter volume measurement|neuroimaging measurement	grey matter volume measurement	EFO_0005420	Other measurement	Neurological
PGS001586	EFO_0005420	grey matter volume measurement|neuroimaging measurement	grey matter volume measurement	EFO_0005420	Other measurement	Neurological
PGS001587	EFO_0005420	grey matter volume measurement|neuroimaging measurement	grey matter volume measurement	EFO_0005420	Other measurement	Neurological
PGS001588	EFO_0005420	grey matter volume measurement|neuroimaging measurement	grey matter volume measurement	EFO_0005420	Other measurement	Neurological
PGS001589	EFO_0005420	grey matter volume measurement|neuroimaging measurement	grey matter volume measurement	EFO_0005420	Other measurement	Neurological
PGS001590	EFO_0005420	grey matter volume measurement|neuroimaging measurement	grey matter volume measurement	EFO_0005420	Other measurement	Neurological
PGS001591	EFO_0005420	grey matter volume measurement|neuroimaging measurement	grey matter volume measurement	EFO_0005420	Other measurement	Neurological

PGS001592	EFO_0005420	grey matter volume measurement|neuroimaging measurement	grey matter volume measurement	EFO_0005420	Other measurement	Neurological
PGS001593	EFO_0005420	grey matter volume measurement|neuroimaging measurement	grey matter volume measurement	EFO_0005420	Other measurement	Neurological
PGS001594	EFO_0005420	grey matter volume measurement|neuroimaging measurement	grey matter volume measurement	EFO_0005420	Other measurement	Neurological
PGS001595	EFO_0005420	grey matter volume measurement|neuroimaging measurement	grey matter volume measurement	EFO_0005420	Other measurement	Neurological
PGS001596	EFO_0005420	grey matter volume measurement|neuroimaging measurement	grey matter volume measurement	EFO_0005420	Other measurement	Neurological
PGS001599	EFO_0005420	grey matter volume measurement|neuroimaging measurement	grey matter volume measurement	EFO_0005420	Other measurement	Neurological
PGS001600	EFO_0005420	grey matter volume measurement|neuroimaging measurement	grey matter volume measurement	EFO_0005420	Other measurement	Neurological
PGS001601	EFO_0005420	grey matter volume measurement|neuroimaging measurement	grey matter volume measurement	EFO_0005420	Other measurement	Neurological
PGS001602	EFO_0005420	grey matter volume measurement|neuroimaging measurement	grey matter volume measurement	EFO_0005420	Other measurement	Neurological
PGS001603	EFO_0005420	grey matter volume measurement|neuroimaging measurement	grey matter volume measurement	EFO_0005420	Other measurement	Neurological
PGS001604	EFO_0005420	grey matter volume measurement|neuroimaging measurement	grey matter volume measurement	EFO_0005420	Other measurement	Neurological
PGS001605	EFO_0005420	grey matter volume measurement|neuroimaging measurement	grey matter volume measurement	EFO_0005420	Other measurement	Neurological
PGS001606	EFO_0005420	grey matter volume measurement|neuroimaging measurement	grey matter volume measurement	EFO_0005420	Other measurement	Neurological
PGS001607	EFO_0005420	grey matter volume measurement|neuroimaging measurement	grey matter volume measurement	EFO_0005420	Other measurement	Neurological
PGS001608	EFO_0005420	grey matter volume measurement|neuroimaging measurement	grey matter volume measurement	EFO_0005420	Other measurement	Neurological
PGS001609	EFO_0005420	grey matter volume measurement|neuroimaging measurement	grey matter volume measurement	EFO_0005420	Other measurement	Neurological
PGS001610	EFO_0005420	grey matter volume measurement|neuroimaging measurement	grey matter volume measurement	EFO_0005420	Other measurement	Neurological
PGS001611	EFO_0005420	grey matter volume measurement|neuroimaging measurement	grey matter volume measurement	EFO_0005420	Other measurement	Neurological
PGS001612	EFO_0005420	grey matter volume measurement|neuroimaging measurement	grey matter volume measurement	EFO_0005420	Other measurement	Neurological
PGS001613	EFO_0005420	grey matter volume measurement|neuroimaging measurement	grey matter volume measurement	EFO_0005420	Other measurement	Neurological
PGS001614	EFO_0005420	grey matter volume measurement|neuroimaging measurement	grey matter volume measurement	EFO_0005420	Other measurement	Neurological
PGS001615	EFO_0005420	grey matter volume measurement|neuroimaging measurement	grey matter volume measurement	EFO_0005420	Other measurement	Neurological
PGS001616	EFO_0005420	grey matter volume measurement|neuroimaging measurement	grey matter volume measurement	EFO_0005420	Other measurement	Neurological
PGS001617	EFO_0005420	grey matter volume measurement|neuroimaging measurement	grey matter volume measurement	EFO_0005420	Other measurement	Neurological
PGS001618	EFO_0005420	grey matter volume measurement|neuroimaging measurement	grey matter volume measurement	EFO_0005420	Other measurement	Neurological

PGS001619	EFO_0005420	grey matter volume measurement|neuroimaging measurement	grey matter volume measurement	EFO_0005420	Other measurement	Neurological
PGS001620	EFO_0005420	grey matter volume measurement|neuroimaging measurement	grey matter volume measurement	EFO_0005420	Other measurement	Neurological
PGS001621	EFO_0005420	grey matter volume measurement|neuroimaging measurement	grey matter volume measurement	EFO_0005420	Other measurement	Neurological
PGS001622	EFO_0005420	grey matter volume measurement|neuroimaging measurement	grey matter volume measurement	EFO_0005420	Other measurement	Neurological
PGS001623	EFO_0005420	grey matter volume measurement|neuroimaging measurement	grey matter volume measurement	EFO_0005420	Other measurement	Neurological
PGS001624	EFO_0005420	grey matter volume measurement|neuroimaging measurement	grey matter volume measurement	EFO_0005420	Other measurement	Neurological
PGS001625	EFO_0005420	grey matter volume measurement|neuroimaging measurement	grey matter volume measurement	EFO_0005420	Other measurement	Neurological
PGS001626	EFO_0005420	grey matter volume measurement|neuroimaging measurement	grey matter volume measurement	EFO_0005420	Other measurement	Neurological
PGS001627	EFO_0005420	grey matter volume measurement|neuroimaging measurement	grey matter volume measurement	EFO_0005420	Other measurement	Neurological
PGS001628	EFO_0005420	grey matter volume measurement|neuroimaging measurement	grey matter volume measurement	EFO_0005420	Other measurement	Neurological
PGS001629	EFO_0005420	grey matter volume measurement|neuroimaging measurement	grey matter volume measurement	EFO_0005420	Other measurement	Neurological
PGS001633	EFO_0005420	grey matter volume measurement|neuroimaging measurement	grey matter volume measurement	EFO_0005420	Other measurement	Neurological
PGS001634	EFO_0005420	grey matter volume measurement|neuroimaging measurement	grey matter volume measurement	EFO_0005420	Other measurement	Neurological
PGS001144	EFO_0005409	fat body mass	fat body mass	EFO_0005409	Other measurement	Body measurement
PGS001145	EFO_0005409	fat body mass	fat body mass	EFO_0005409	Other measurement	Body measurement
PGS001146	EFO_0005409	fat body mass	fat body mass	EFO_0005409	Other measurement	Body measurement
PGS001147	EFO_0005409	fat body mass	fat body mass	EFO_0005409	Other measurement	Body measurement
PGS001148	EFO_0005409	fat body mass	fat body mass	EFO_0005409	Other measurement	Body measurement
PGS001149	EFO_0005409	fat body mass	fat body mass	EFO_0005409	Other measurement	Body measurement
PGS001951	EFO_0005409	fat body mass	fat body mass	EFO_0005409	Other measurement	Body measurement
PGS002169	EFO_0005409	fat body mass	fat body mass	EFO_0005409	Other measurement	Body measurement
PGS001150	EFO_0005271	sleep duration	sleep duration	EFO_0005271	Other measurement	Psychiatric
PGS001978	EFO_0005271	sleep duration	sleep duration	EFO_0005271	Other measurement	Psychiatric
PGS002196	EFO_0005271	sleep duration	sleep duration	EFO_0005271	Other measurement	Psychiatric
PGS001153	EFO_0005111	folic acid measurement	folic acid measurement	EFO_0005111	Other measurement	Metabolic
PGS001154	EFO_0005106	body composition measurement	body composition measurement	EFO_0005106	Body measurement	Body measurement
PGS001155	EFO_0005106	body composition measurement	body composition measurement	EFO_0005106	Body measurement	Body measurement
PGS001156	EFO_0005106	body composition measurement	body composition measurement	EFO_0005106	Body measurement	Body measurement
PGS001157	EFO_0005106	body composition measurement	body composition measurement	EFO_0005106	Body measurement	Body measurement
PGS001158	EFO_0005106	body composition measurement	body composition measurement	EFO_0005106	Body measurement	Body measurement
PGS001159	EFO_0005106	body composition measurement	body composition measurement	EFO_0005106	Body measurement	Body measurement
PGS001160	EFO_0005106	body composition measurement	body composition measurement	EFO_0005106	Body measurement	Body measurement
PGS001161	EFO_0005106	body composition measurement	body composition measurement	EFO_0005106	Body measurement	Body measurement
PGS001961	EFO_0005106	body composition measurement	body composition measurement	EFO_0005106	Body measurement	Body measurement
PGS002179	EFO_0005106	body composition measurement	body composition measurement	EFO_0005106	Body measurement	Body measurement
PGS001162	EFO_0005093	hip circumference	hip circumference	EFO_0005093	Body measurement	Body measurement
PGS001958	EFO_0005093	hip circumference	hip circumference	EFO_0005093	Body measurement	Body measurement

PGS002176	EFO_0005093	hip circumference	hip circumference	EFO_0005093	Body measurement	Body measurement
PGS001165	EFO_0004995	lean body mass	lean body mass	EFO_0004995	Other measurement	Skeletal
PGS001166	EFO_0004995	lean body mass	lean body mass	EFO_0004995	Other measurement	Skeletal
PGS001167	EFO_0004995	lean body mass	lean body mass	EFO_0004995	Other measurement	Skeletal
PGS001168	EFO_0004995	lean body mass	lean body mass	EFO_0004995	Other measurement	Skeletal
PGS001169	EFO_0004995	lean body mass	lean body mass	EFO_0004995	Other measurement	Skeletal
PGS001950	EFO_0004995	lean body mass	lean body mass	EFO_0004995	Other measurement	Skeletal
PGS002168	EFO_0004995	lean body mass	lean body mass	EFO_0004995	Other measurement	Skeletal
PGS001174	EFO_0004799	cholelithiasis	cholelithiasis	EFO_0004799	Digestive system disorder	Gastrointestinal
PGS001861	EFO_0004799	cholelithiasis|Cholecystitis	cholelithiasis	EFO_0004799	Digestive system disorder	Gastrointestinal
PGS002072	EFO_0004799	cholelithiasis|Cholecystitis	cholelithiasis	EFO_0004799	Digestive system disorder	Gastrointestinal
PGS001179	EFO_0004718	vascular dementia	vascular dementia	EFO_0004718	Cardiovascular disease	Cardiovascular
PGS001179	EFO_0004718	vascular dementia	vascular dementia	EFO_0004718	Neurological disorder	Neurological
PGS001182	EFO_0004697	estradiol measurement	estradiol measurement	EFO_0004697	Other measurement	Endocrine
PGS002914	EFO_0004697	estradiol measurement	estradiol measurement	EFO_0004697	Other measurement	Endocrine
PGS002915	EFO_0004697	estradiol measurement	estradiol measurement	EFO_0004697	Other measurement	Endocrine
PGS002916	EFO_0004697	estradiol measurement	estradiol measurement	EFO_0004697	Other measurement	Endocrine
PGS002917	EFO_0004697	estradiol measurement	estradiol measurement	EFO_0004697	Other measurement	Endocrine
PGS002918	EFO_0004697	estradiol measurement	estradiol measurement	EFO_0004697	Other measurement	Endocrine
PGS002919	EFO_0004697	estradiol measurement	estradiol measurement	EFO_0004697	Other measurement	Endocrine
PGS002920	EFO_0004697	estradiol measurement	estradiol measurement	EFO_0004697	Other measurement	Endocrine
PGS002921	EFO_0004697	estradiol measurement	estradiol measurement	EFO_0004697	Other measurement	Endocrine
PGS002922	EFO_0004697	estradiol measurement	estradiol measurement	EFO_0004697	Other measurement	Endocrine
PGS002923	EFO_0004697	estradiol measurement	estradiol measurement	EFO_0004697	Other measurement	Endocrine
PGS001192	EFO_0004616	osteoarthritis, knee	osteoarthritis, knee	EFO_0004616	Other disease	Skeletal
PGS002729	EFO_0004616	osteoarthritis, knee	osteoarthritis, knee	EFO_0004616	Other disease	Skeletal
PGS002767	EFO_0004616	osteoarthritis, knee	osteoarthritis, knee	EFO_0004616	Other disease	Skeletal
PGS001226	EFO_0004344	birth weight	birth weight	EFO_0004344	Body measurement	Body measurement
PGS001892	EFO_0004344	birth weight	birth weight	EFO_0004344	Body measurement	Body measurement
PGS002105	EFO_0004344	birth weight	birth weight	EFO_0004344	Body measurement	Body measurement
PGS001230	EFO_0004338	body weight	body weight	EFO_0004338	Body measurement	Body measurement
PGS001232	EFO_0004337	intelligence	intelligence	EFO_0004337	Biological process	Cognitive
PGS001919	EFO_0004337	intelligence	intelligence	EFO_0004337	Biological process	Cognitive
PGS002135	EFO_0004337	intelligence	intelligence	EFO_0004337	Biological process	Cognitive
PGS001233	EFO_0004326	heart rate	heart rate	EFO_0004326	Cardiovascular measurement	Cardiovascular
PGS001375	EFO_0004326	heart rate	heart rate	EFO_0004326	Cardiovascular measurement	Cardiovascular
PGS001523	EFO_0004326	heart rate	heart rate	EFO_0004326	Cardiovascular measurement	Cardiovascular
PGS001524	EFO_0004326	heart rate	heart rate	EFO_0004326	Cardiovascular measurement	Cardiovascular
PGS001975	EFO_0004326	heart rate	heart rate	EFO_0004326	Cardiovascular measurement	Cardiovascular
PGS002193	EFO_0004326	heart rate	heart rate	EFO_0004326	Cardiovascular measurement	Cardiovascular
PGS001234	EFO_0004324	body weights and measures	body weights and measures	EFO_0004324	Body measurement	Body measurement

PGS001235	EFO_0004324	body weights and measures	body weights and measures	EFO_0004324	Body measurement	Body measurement
PGS001236	EFO_0004314	forced expiratory volume	forced expiratory volume	EFO_0004314	Other measurement	Respiratory
PGS001800	EFO_0004314	forced expiratory volume	forced expiratory volume	EFO_0004314	Other measurement	Respiratory
PGS001918	EFO_0004314	forced expiratory volume	forced expiratory volume	EFO_0004314	Other measurement	Respiratory
PGS002134	EFO_0004314	forced expiratory volume	forced expiratory volume	EFO_0004314	Other measurement	Respiratory
PGS001237	EFO_0004312	vital capacity	vital capacity	EFO_0004312	Other measurement	Activities
PGS001922	EFO_0004312	vital capacity	vital capacity	EFO_0004312	Other measurement	Activities
PGS002138	EFO_0004312	vital capacity	vital capacity	EFO_0004312	Other measurement	Activities
PGS002327	EFO_0004312	vital capacity	vital capacity	EFO_0004312	Other measurement	Activities
PGS002399	EFO_0004312	vital capacity	vital capacity	EFO_0004312	Other measurement	Activities
PGS002448	EFO_0004312	vital capacity	vital capacity	EFO_0004312	Other measurement	Activities
PGS002497	EFO_0004312	vital capacity	vital capacity	EFO_0004312	Other measurement	Activities
PGS002546	EFO_0004312	vital capacity	vital capacity	EFO_0004312	Other measurement	Activities
PGS002595	EFO_0004312	vital capacity	vital capacity	EFO_0004312	Other measurement	Activities
PGS002644	EFO_0004312	vital capacity	vital capacity	EFO_0004312	Other measurement	Activities
PGS001241	EFO_0004302	anthropometric measurement	anthropometric measurement	EFO_0004302	Other measurement	Body measurement
PGS001242	EFO_0004302	anthropometric measurement	anthropometric measurement	EFO_0004302	Other measurement	Body measurement
PGS001243	EFO_0004302	anthropometric measurement	anthropometric measurement	EFO_0004302	Other measurement	Body measurement
PGS001379	EFO_0004302	anthropometric measurement	anthropometric measurement	EFO_0004302	Other measurement	Body measurement
PGS001887	EFO_0004302	anthropometric measurement	anthropometric measurement	EFO_0004302	Other measurement	Body measurement
PGS002100	EFO_0004302	anthropometric measurement	anthropometric measurement	EFO_0004302	Other measurement	Body measurement
PGS001244	EFO_0004279	suntan	suntan	EFO_0004279	Other trait	Dermatological
PGS001245	EFO_0004279	suntan	suntan	EFO_0004279	Other trait	Dermatological
PGS001246	EFO_0004279	suntan	suntan	EFO_0004279	Other trait	Dermatological
PGS001247	EFO_0004279	suntan	suntan	EFO_0004279	Other trait	Dermatological
PGS001937	EFO_0004279	suntan	suntan	EFO_0004279	Other trait	Dermatological
PGS002155	EFO_0004279	suntan	suntan	EFO_0004279	Other trait	Dermatological
PGS002350	EFO_0004279	suntan	suntan	EFO_0004279	Other trait	Dermatological
PGS002422	EFO_0004279	suntan	suntan	EFO_0004279	Other trait	Dermatological
PGS002471	EFO_0004279	suntan	suntan	EFO_0004279	Other trait	Dermatological
PGS002520	EFO_0004279	suntan	suntan	EFO_0004279	Other trait	Dermatological
PGS002569	EFO_0004279	suntan	suntan	EFO_0004279	Other trait	Dermatological
PGS002618	EFO_0004279	suntan	suntan	EFO_0004279	Other trait	Dermatological
PGS002667	EFO_0004279	suntan	suntan	EFO_0004279	Other trait	Dermatological
PGS001250	EFO_0004253	nephrolithiasis|ureterolithiasis	nephrolithiasis	EFO_0004253	Other disease	Genitourinary
PGS001251	EFO_0004244	interstitial lung disease	interstitial lung disease	EFO_0004244	Other disease	Respiratory
PGS001252	EFO_0004238	hearing loss|deafness	hearing loss	EFO_0004238	Neurological disorder	Neurological
PGS001253	EFO_0004238	hearing loss	hearing loss	EFO_0004238	Neurological disorder	Neurological
PGS001254	EFO_0004229	Dupuytren Contracture	Dupuytren Contracture	EFO_0004229	Other disease	Others
PGS001880	EFO_0004229	Dupuytren Contracture	Dupuytren Contracture	EFO_0004229	Other disease	Others
PGS002092	EFO_0004229	Dupuytren Contracture	Dupuytren Contracture	EFO_0004229	Other disease	Others
PGS001255	EFO_0004213	otosclerosis	otosclerosis	EFO_0004213	Neurological disorder	Neurological
PGS002046	EFO_0004213	otosclerosis	otosclerosis	EFO_0004213	Neurological disorder	Neurological
PGS001257	EFO_0003958	sunburn	sunburn	EFO_0003958	Other disease	Dermatological
PGS001258	EFO_0003957	radius fracture	radius fracture	EFO_0003957	Other disease	Skeletal
PGS001259	EFO_0003956	seasonal allergic rhinitis	seasonal allergic rhinitis	EFO_0003956	Immune system disorder	Immunological
PGS001263	EFO_0003911	atrial flutter	atrial flutter	EFO_0003911	Cardiovascular disease	Cardiovascular
PGS001841	EFO_0003911	atrial flutter|atrial fibrillation	atrial flutter	EFO_0003911	Cardiovascular disease	Cardiovascular
PGS002050	EFO_0003911	atrial flutter|atrial fibrillation	atrial flutter	EFO_0003911	Cardiovascular disease	Cardiovascular

PGS001264	EFO_0003907	deep vein thrombosis	deep vein thrombosis	EFO_0003907	Other trait	Cardiovascular
PGS001265	EFO_0003907	deep vein thrombosis	deep vein thrombosis	EFO_0003907	Other trait	Cardiovascular
PGS001266	EFO_0003907	deep vein thrombosis	deep vein thrombosis	EFO_0003907	Other trait	Cardiovascular
PGS001267	EFO_0003898	ankylosing spondylitis	ankylosing spondylitis	EFO_0003898	Immune system disorder	Immunological
PGS001268	EFO_0003898	ankylosing spondylitis	ankylosing spondylitis	EFO_0003898	Immune system disorder	Immunological
PGS001876	EFO_0003898	ankylosing spondylitis	ankylosing spondylitis	EFO_0003898	Immune system disorder	Immunological
PGS002089	EFO_0003898	ankylosing spondylitis	ankylosing spondylitis	EFO_0003898	Immune system disorder	Immunological
PGS001270	MONDO_0005301	multiple sclerosis	multiple sclerosis	MONDO_0005301	Immune system disorder	Immunological
PGS001270	MONDO_0005301	multiple sclerosis	multiple sclerosis	MONDO_0005301	Neurological disorder	Neurological
PGS001271	MONDO_0005301	multiple sclerosis	multiple sclerosis	MONDO_0005301	Immune system disorder	Immunological
PGS001271	MONDO_0005301	multiple sclerosis	multiple sclerosis	MONDO_0005301	Neurological disorder	Neurological
PGS001831	MONDO_0005301	multiple sclerosis	multiple sclerosis	MONDO_0005301	Immune system disorder	Immunological
PGS001831	MONDO_0005301	multiple sclerosis	multiple sclerosis	MONDO_0005301	Neurological disorder	Neurological
PGS002038	MONDO_0005301	multiple sclerosis	multiple sclerosis	MONDO_0005301	Immune system disorder	Immunological
PGS002038	MONDO_0005301	multiple sclerosis	multiple sclerosis	MONDO_0005301	Neurological disorder	Neurological
PGS002726	MONDO_0005301	multiple sclerosis	multiple sclerosis	MONDO_0005301	Immune system disorder	Immunological
PGS002726	MONDO_0005301	multiple sclerosis	multiple sclerosis	MONDO_0005301	Neurological disorder	Neurological
PGS001273	EFO_0003882	osteoporosis	osteoporosis	EFO_0003882	Other disease	Skeletal
PGS001274	EFO_0003882	osteoporosis	osteoporosis	EFO_0003882	Other disease	Skeletal
PGS001883	EFO_0003882	osteoporosis	osteoporosis	EFO_0003882	Other disease	Skeletal
PGS002095	EFO_0003882	osteoporosis	osteoporosis	EFO_0003882	Other disease	Skeletal
PGS002768	EFO_0003882	osteoporosis	osteoporosis	EFO_0003882	Other disease	Skeletal
PGS001275	EFO_0003839	retinopathy	retinopathy	EFO_0003839	Neurological disorder	Neurological
PGS001276	EFO_0003839	retinopathy	retinopathy	EFO_0003839	Neurological disorder	Neurological
PGS001277	EFO_0003827	pulmonary embolism|deep vein thrombosis	pulmonary embolism	EFO_0003827	Cardiovascular disease	Cardiovascular
PGS001278	EFO_0003827	pulmonary embolism|deep vein thrombosis	pulmonary embolism	EFO_0003827	Cardiovascular disease	Cardiovascular
PGS001279	EFO_0003827	pulmonary embolism|deep vein thrombosis	pulmonary embolism	EFO_0003827	Cardiovascular disease	Cardiovascular
PGS001280	EFO_0003827	pulmonary embolism	pulmonary embolism	EFO_0003827	Cardiovascular disease	Cardiovascular
PGS001281	MONDO_0005277	migraine disorder	migraine disorder	MONDO_0005277	Cardiovascular disease	Cardiovascular
PGS001281	MONDO_0005277	migraine disorder	migraine disorder	MONDO_0005277	Neurological disorder	Neurological
PGS001282	MONDO_0005277	migraine disorder	migraine disorder	MONDO_0005277	Cardiovascular disease	Cardiovascular
PGS001282	MONDO_0005277	migraine disorder	migraine disorder	MONDO_0005277	Neurological disorder	Neurological

PGS001290	MONDO_0005178	osteoarthritis	osteoarthritis	MONDO_0005178	Other disease	Skeletal
PGS001882	MONDO_0005178	osteoarthritis	osteoarthritis	MONDO_0005178	Other disease	Skeletal
PGS002094	MONDO_0005178	osteoarthritis	osteoarthritis	MONDO_0005178	Other disease	Skeletal
PGS001292	EFO_0600083	family history of prostate cancer	family history of prostate cancer	EFO_0600083	Other trait	Neoplasms
PGS001293	EFO_0001421	liver disease	liver disease	EFO_0001421	Digestive system disorder	Gastrointestinal
PGS001860	EFO_0001421	liver disease	liver disease	EFO_0001421	Digestive system disorder	Gastrointestinal
PGS002071	EFO_0001421	liver disease	liver disease	EFO_0001421	Digestive system disorder	Gastrointestinal
PGS001298	EFO_0001073	obesity	obesity	EFO_0001073	Metabolic disorder	Metabolic
PGS003400	EFO_0001073	obesity	obesity	EFO_0001073	Metabolic disorder	Metabolic
PGS001302	MONDO_0005129	cataract	cataract	MONDO_0005129	Other disease	Ophthalmological
PGS001303	MONDO_0005129	cataract	cataract	MONDO_0005129	Other disease	Ophthalmological
PGS001837	MONDO_0005129	cataract	cataract	MONDO_0005129	Other disease	Ophthalmological
PGS002044	MONDO_0005129	cataract	cataract	MONDO_0005129	Other disease	Ophthalmological
PGS001305	EFO_0000734	vitamin B12 deficiency|anemia	vitamin B12 deficiency	EFO_0000734	Metabolic disorder	Metabolic
PGS001306	EFO_0000729	ulcerative colitis	ulcerative colitis	EFO_0000729	Digestive system disorder	Gastrointestinal
PGS001306	EFO_0000729	ulcerative colitis	ulcerative colitis	EFO_0000729	Immune system disorder	Immunological
PGS001307	EFO_0000729	ulcerative colitis	ulcerative colitis	EFO_0000729	Digestive system disorder	Gastrointestinal
PGS001307	EFO_0000729	ulcerative colitis	ulcerative colitis	EFO_0000729	Immune system disorder	Immunological
PGS001855	EFO_0000729	ulcerative colitis	ulcerative colitis	EFO_0000729	Digestive system disorder	Gastrointestinal
PGS001855	EFO_0000729	ulcerative colitis	ulcerative colitis	EFO_0000729	Immune system disorder	Immunological
PGS002066	EFO_0000729	ulcerative colitis	ulcerative colitis	EFO_0000729	Digestive system disorder	Gastrointestinal
PGS002066	EFO_0000729	ulcerative colitis	ulcerative colitis	EFO_0000729	Immune system disorder	Immunological
PGS001308	EFO_0000699	Sjogren syndrome	Sjogren syndrome	EFO_0000699	Digestive system disorder	Gastrointestinal
PGS001308	EFO_0000699	Sjogren syndrome	Sjogren syndrome	EFO_0000699	Immune system disorder	Immunological
PGS001309	EFO_0000685	rheumatoid arthritis	rheumatoid arthritis	EFO_0000685	Immune system disorder	Immunological
PGS001310	EFO_0000685	rheumatoid arthritis	rheumatoid arthritis	EFO_0000685	Immune system disorder	Immunological
PGS001311	EFO_0000685	rheumatoid arthritis	rheumatoid arthritis	EFO_0000685	Immune system disorder	Immunological

PGS001875	EFO_0000685	rheumatoid arthritis	rheumatoid arthritis	EFO_0000685	Immune system disorder	Immunological
PGS002088	EFO_0000685	rheumatoid arthritis	rheumatoid arthritis	EFO_0000685	Immune system disorder	Immunological
PGS002745	EFO_0000685	rheumatoid arthritis	rheumatoid arthritis	EFO_0000685	Immune system disorder	Immunological
PGS002769	EFO_0000685	rheumatoid arthritis	rheumatoid arthritis	EFO_0000685	Immune system disorder	Immunological
PGS001312	EFO_0000676	psoriasis	psoriasis	EFO_0000676	Immune system disorder	Immunological
PGS001313	EFO_0000676	psoriasis	psoriasis	EFO_0000676	Immune system disorder	Immunological
PGS001871	EFO_0000676	psoriasis	psoriasis	EFO_0000676	Immune system disorder	Immunological
PGS002083	EFO_0000676	psoriasis	psoriasis	EFO_0000676	Immune system disorder	Immunological
PGS002344	EFO_0000676	psoriasis	psoriasis	EFO_0000676	Immune system disorder	Immunological
PGS002416	EFO_0000676	psoriasis	psoriasis	EFO_0000676	Immune system disorder	Immunological
PGS002465	EFO_0000676	psoriasis	psoriasis	EFO_0000676	Immune system disorder	Immunological
PGS002514	EFO_0000676	psoriasis	psoriasis	EFO_0000676	Immune system disorder	Immunological
PGS002563	EFO_0000676	psoriasis	psoriasis	EFO_0000676	Immune system disorder	Immunological
PGS002612	EFO_0000676	psoriasis	psoriasis	EFO_0000676	Immune system disorder	Immunological
PGS001318	EFO_0000589	metabolic disease	metabolic disease	EFO_0000589	Metabolic disorder	Metabolic
PGS001319	EFO_0000589	metabolic disease	metabolic disease	EFO_0000589	Metabolic disorder	Metabolic
PGS001326	EFO_0000464	emphysema	emphysema	EFO_0000464	Other disease	Respiratory
PGS001327	EFO_0000400	diabetes mellitus	diabetes mellitus	EFO_0000400	Digestive system disorder	Gastrointestinal
PGS001327	EFO_0000400	diabetes mellitus	diabetes mellitus	EFO_0000400	Metabolic disorder	Metabolic
PGS001329	EFO_0000400	diabetes mellitus	diabetes mellitus	EFO_0000400	Digestive system disorder	Gastrointestinal
PGS001329	EFO_0000400	diabetes mellitus	diabetes mellitus	EFO_0000400	Metabolic disorder	Metabolic
PGS002321	EFO_0000400	diabetes mellitus	diabetes mellitus	EFO_0000400	Digestive system disorder	Gastrointestinal
PGS002321	EFO_0000400	diabetes mellitus	diabetes mellitus	EFO_0000400	Metabolic disorder	Metabolic
PGS002393	EFO_0000400	diabetes mellitus	diabetes mellitus	EFO_0000400	Digestive system disorder	Gastrointestinal
PGS002393	EFO_0000400	diabetes mellitus	diabetes mellitus	EFO_0000400	Metabolic disorder	Metabolic
PGS002442	EFO_0000400	diabetes mellitus	diabetes mellitus	EFO_0000400	Digestive system disorder	Gastrointestinal
PGS002442	EFO_0000400	diabetes mellitus	diabetes mellitus	EFO_0000400	Metabolic disorder	Metabolic
PGS002491	EFO_0000400	diabetes mellitus	diabetes mellitus	EFO_0000400	Digestive system disorder	Gastrointestinal
PGS002491	EFO_0000400	diabetes mellitus	diabetes mellitus	EFO_0000400	Metabolic disorder	Metabolic

PGS002540	EFO_0000400	diabetes mellitus	diabetes mellitus	EFO_0000400	Digestive system disorder	Gastrointestinal
PGS002540	EFO_0000400	diabetes mellitus	diabetes mellitus	EFO_0000400	Metabolic disorder	Metabolic
PGS002589	EFO_0000400	diabetes mellitus	diabetes mellitus	EFO_0000400	Digestive system disorder	Gastrointestinal
PGS002589	EFO_0000400	diabetes mellitus	diabetes mellitus	EFO_0000400	Metabolic disorder	Metabolic
PGS002638	EFO_0000400	diabetes mellitus	diabetes mellitus	EFO_0000400	Digestive system disorder	Gastrointestinal
PGS002638	EFO_0000400	diabetes mellitus	diabetes mellitus	EFO_0000400	Metabolic disorder	Metabolic
PGS001330	EFO_0000384	Crohn's disease	Crohn's disease	EFO_0000384	Digestive system disorder	Gastrointestinal
PGS001330	EFO_0000384	Crohn's disease	Crohn's disease	EFO_0000384	Immune system disorder	Immunological
PGS001331	EFO_0000384	Crohn's disease	Crohn's disease	EFO_0000384	Digestive system disorder	Gastrointestinal
PGS001331	EFO_0000384	Crohn's disease	Crohn's disease	EFO_0000384	Immune system disorder	Immunological
PGS001332	EFO_0000341	chronic obstructive pulmonary disease	chronic obstructive pulmonary disease	EFO_0000341	Other disease	Respiratory
PGS001333	EFO_0000341	chronic obstructive pulmonary disease	chronic obstructive pulmonary disease	EFO_0000341	Other disease	Respiratory
PGS001334	EFO_0000341	chronic obstructive pulmonary disease	chronic obstructive pulmonary disease	EFO_0000341	Other disease	Respiratory
PGS001783	EFO_0000341	chronic obstructive pulmonary disease	chronic obstructive pulmonary disease	EFO_0000341	Other disease	Respiratory
PGS001788	EFO_0000341	chronic obstructive pulmonary disease	chronic obstructive pulmonary disease	EFO_0000341	Other disease	Respiratory
PGS001850	EFO_0000341	chronic obstructive pulmonary disease	chronic obstructive pulmonary disease	EFO_0000341	Other disease	Respiratory
PGS002062	EFO_0000341	chronic obstructive pulmonary disease	chronic obstructive pulmonary disease	EFO_0000341	Other disease	Respiratory
PGS001337	EFO_0005606	family history of breast cancer	family history of breast cancer	EFO_0005606	Other trait	Neoplasms
PGS001338	EFO_0000284	benign prostatic hyperplasia	benign prostatic hyperplasia	EFO_0000284	Other disease	Genitourinary
PGS001865	EFO_0000284	benign prostatic hyperplasia	benign prostatic hyperplasia	EFO_0000284	Other disease	Genitourinary
PGS002076	EFO_0000284	benign prostatic hyperplasia	benign prostatic hyperplasia	EFO_0000284	Other disease	Genitourinary
PGS001347	EFO_0009268	family history of Alzheimerâ€™s disease	family history of Alzheimerâ€™s disease	EFO_0009268	Other measurement	Neurological
PGS001358	EFO_0004731	eye measurement	eye measurement	EFO_0004731	Other measurement	Ophthalmological
PGS001359	EFO_0004731	eye measurement	eye measurement	EFO_0004731	Other measurement	Ophthalmological
PGS001360	EFO_0004731	eye measurement	eye measurement	EFO_0004731	Other measurement	Ophthalmological
PGS001361	EFO_0004731	eye measurement	eye measurement	EFO_0004731	Other measurement	Ophthalmological
PGS001362	EFO_0004731	eye measurement	eye measurement	EFO_0004731	Other measurement	Ophthalmological
PGS001363	EFO_0004731	eye measurement	eye measurement	EFO_0004731	Other measurement	Ophthalmological
PGS001364	EFO_0004731	eye measurement	eye measurement	EFO_0004731	Other measurement	Ophthalmological
PGS001365	EFO_0004731	eye measurement	eye measurement	EFO_0004731	Other measurement	Ophthalmological
PGS001366	EFO_0004731	eye measurement	eye measurement	EFO_0004731	Other measurement	Ophthalmological
PGS001367	EFO_0004731	eye measurement	eye measurement	EFO_0004731	Other measurement	Ophthalmological
PGS001368	EFO_0004731	eye measurement	eye measurement	EFO_0004731	Other measurement	Ophthalmological
PGS001373	EFO_0004731	eye measurement	eye measurement	EFO_0004731	Other measurement	Ophthalmological
PGS001924	EFO_0004731	eye measurement	eye measurement	EFO_0004731	Other measurement	Ophthalmological
PGS002140	EFO_0004731	eye measurement	eye measurement	EFO_0004731	Other measurement	Ophthalmological
PGS001369	EFO_0007149	appendicitis	appendicitis	EFO_0007149	Digestive system disorder	Gastrointestinal
PGS001369	EFO_0007149	appendicitis	appendicitis	EFO_0007149	Immune system disorder	Immunological
PGS001785	EFO_0007149	appendicitis	appendicitis	EFO_0007149	Digestive system disorder	Gastrointestinal

PGS001785	EFO_0007149	appendicitis	appendicitis	EFO_0007149	Immune system disorder	Immunological
PGS001371	EFO_0004918	age at diagnosis|diabetes mellitus	age at diagnosis	EFO_0004918	Other measurement	Metabolic
PGS001374	EFO_0004319	smoking cessation	smoking cessation	EFO_0004319	Biological process	Psychiatric
PGS003372	EFO_0004319	smoking cessation	smoking cessation	EFO_0004319	Biological process	Psychiatric
PGS001376	EFO_0600084	bowel opening frequency	bowel opening frequency	EFO_0600084	Other measurement	Gastrointestinal
PGS002232	EFO_0600084	bowel opening frequency	bowel opening frequency	EFO_0600084	Other measurement	Gastrointestinal
PGS001380	EFO_0010066	corneal hysteresis	corneal hysteresis	EFO_0010066	Other measurement	Ophthalmological
PGS001381	EFO_0010066	corneal hysteresis	corneal hysteresis	EFO_0010066	Other measurement	Ophthalmological
PGS001382	EFO_0010067	corneal resistance factor	corneal resistance factor	EFO_0010067	Other measurement	Ophthalmological
PGS001383	EFO_0010067	corneal resistance factor	corneal resistance factor	EFO_0010067	Other measurement	Ophthalmological
PGS001384	EFO_0011014	health-related quality of life measurement	health-related quality of life measurement	EFO_0011014	Other measurement	Activities
PGS001385	EFO_0011014	health-related quality of life measurement	health-related quality of life measurement	EFO_0011014	Other measurement	Activities
PGS001386	EFO_0011014	health-related quality of life measurement	health-related quality of life measurement	EFO_0011014	Other measurement	Activities
PGS001387	EFO_0011014	health-related quality of life measurement	health-related quality of life measurement	EFO_0011014	Other measurement	Activities
PGS001390	EFO_0004607	duodenal ulcer	duodenal ulcer	EFO_0004607	Digestive system disorder	Gastrointestinal
PGS001391	EFO_0007585	Cannabis use	Cannabis use	EFO_0007585	Biological process	Psychiatric
PGS001910	EFO_0007585	Cannabis use	Cannabis use	EFO_0007585	Biological process	Psychiatric
PGS002125	EFO_0007585	Cannabis use	Cannabis use	EFO_0007585	Biological process	Psychiatric
PGS001392	EFO_0006953	family history of lung cancer	family history of lung cancer	EFO_0006953	Other measurement	Respiratory
PGS001393	EFO_0009712	father's age at death	father's age at death	EFO_0009712	Other measurement	Mortality
PGS001395	HP_0002027	Abdominal pain	Abdominal pain	HP_0002027	Other trait	Neurological
PGS001884	HP_0002027	Abdominal pain	Abdominal pain	HP_0002027	Other trait	Neurological
PGS002096	HP_0002027	Abdominal pain	Abdominal pain	HP_0002027	Other trait	Neurological
PGS001396	EFO_0007803	emotional symptom measurement	emotional symptom measurement	EFO_0007803	Other measurement	Activities
PGS001398	EFO_0009725	friendship satisfaction measurement	friendship satisfaction measurement	EFO_0009725	Other measurement	Psychiatric
PGS001399	EFO_0009340	Varicella zoster virus seropositivity	Varicella zoster virus seropositivity	EFO_0009340	Other measurement	Infection
PGS001408	EFO_0009253	Immature Reticulocyte Fraction Measurement	Immature Reticulocyte Fraction Measurement	EFO_0009253	Hematological measurement	Hematological
PGS001930	EFO_0009253	Immature Reticulocyte Fraction Measurement	Immature Reticulocyte Fraction Measurement	EFO_0009253	Hematological measurement	Hematological
PGS002147	EFO_0009253	Immature Reticulocyte Fraction Measurement	Immature Reticulocyte Fraction Measurement	EFO_0009253	Hematological measurement	Hematological
PGS001412	EFO_0008373	left ventricular ejection fraction measurement	left ventricular ejection fraction measurement	EFO_0008373	Cardiovascular measurement	Cardiovascular
PGS002278	EFO_0008373	left ventricular ejection fraction measurement	left ventricular ejection fraction measurement	EFO_0008373	Cardiovascular measurement	Cardiovascular
PGS002279	EFO_0008373	left ventricular ejection fraction measurement	left ventricular ejection fraction measurement	EFO_0008373	Cardiovascular measurement	Cardiovascular
PGS001413	EFO_0010555	left ventricular stroke volume measurement	left ventricular stroke volume measurement	EFO_0010555	Cardiovascular measurement	Cardiovascular
PGS001415	EFO_0006930	brain volume measurement|neuroimaging measurement	brain volume measurement	EFO_0006930	Other measurement	Neurological
PGS001416	EFO_0006930	brain volume measurement|neuroimaging measurement	brain volume measurement	EFO_0006930	Other measurement	Neurological
PGS001417	EFO_0006930	brain volume measurement|neuroimaging measurement	brain volume measurement	EFO_0006930	Other measurement	Neurological
PGS001418	EFO_0006930	brain volume measurement|neuroimaging measurement	brain volume measurement	EFO_0006930	Other measurement	Neurological

PGS001419	EFO_0006930	brain volume measurement|neuroimaging measurement	brain volume measurement	EFO_0006930	Other measurement	Neurological
PGS001420	EFO_0006930	brain volume measurement|neuroimaging measurement	brain volume measurement	EFO_0006930	Other measurement	Neurological
PGS001421	EFO_0006930	brain volume measurement|neuroimaging measurement	brain volume measurement	EFO_0006930	Other measurement	Neurological
PGS001422	EFO_0006930	brain volume measurement|neuroimaging measurement	brain volume measurement	EFO_0006930	Other measurement	Neurological
PGS001423	EFO_0006930	brain volume measurement|neuroimaging measurement	brain volume measurement	EFO_0006930	Other measurement	Neurological
PGS001424	EFO_0006930	brain volume measurement|neuroimaging measurement	brain volume measurement	EFO_0006930	Other measurement	Neurological
PGS001425	EFO_0006930	brain volume measurement|neuroimaging measurement	brain volume measurement	EFO_0006930	Other measurement	Neurological
PGS001426	EFO_0006930	brain volume measurement|neuroimaging measurement	brain volume measurement	EFO_0006930	Other measurement	Neurological
PGS001427	EFO_0006930	brain volume measurement|neuroimaging measurement	brain volume measurement	EFO_0006930	Other measurement	Neurological
PGS001428	EFO_0006930	brain volume measurement|neuroimaging measurement	brain volume measurement	EFO_0006930	Other measurement	Neurological
PGS001429	EFO_0006930	brain volume measurement|neuroimaging measurement	brain volume measurement	EFO_0006930	Other measurement	Neurological
PGS001430	EFO_0006930	brain volume measurement|neuroimaging measurement	brain volume measurement	EFO_0006930	Other measurement	Neurological
PGS001431	EFO_0006930	brain volume measurement|neuroimaging measurement	brain volume measurement	EFO_0006930	Other measurement	Neurological
PGS001432	EFO_0006930	brain volume measurement|neuroimaging measurement	brain volume measurement	EFO_0006930	Other measurement	Neurological
PGS001433	EFO_0006930	brain volume measurement|neuroimaging measurement	brain volume measurement	EFO_0006930	Other measurement	Neurological
PGS001434	EFO_0006930	brain volume measurement|neuroimaging measurement	brain volume measurement	EFO_0006930	Other measurement	Neurological
PGS001435	EFO_0006930	brain volume measurement|neuroimaging measurement	brain volume measurement	EFO_0006930	Other measurement	Neurological
PGS001436	EFO_0006930	brain volume measurement|neuroimaging measurement	brain volume measurement	EFO_0006930	Other measurement	Neurological
PGS001437	EFO_0006930	brain volume measurement|neuroimaging measurement	brain volume measurement	EFO_0006930	Other measurement	Neurological
PGS001438	EFO_0006930	brain volume measurement|neuroimaging measurement	brain volume measurement	EFO_0006930	Other measurement	Neurological
PGS001439	EFO_0006930	brain volume measurement|neuroimaging measurement	brain volume measurement	EFO_0006930	Other measurement	Neurological
PGS001440	EFO_0006930	brain volume measurement|neuroimaging measurement	brain volume measurement	EFO_0006930	Other measurement	Neurological
PGS001441	EFO_0006930	brain volume measurement|neuroimaging measurement	brain volume measurement	EFO_0006930	Other measurement	Neurological
PGS001442	EFO_0006930	brain volume measurement|neuroimaging measurement	brain volume measurement	EFO_0006930	Other measurement	Neurological
PGS001443	EFO_0006930	brain volume measurement|neuroimaging measurement	brain volume measurement	EFO_0006930	Other measurement	Neurological

PGS001444	EFO_0006930	brain volume measurement|neuroimaging measurement	brain volume measurement	EFO_0006930	Other measurement	Neurological
PGS001445	EFO_0006930	brain volume measurement|neuroimaging measurement	brain volume measurement	EFO_0006930	Other measurement	Neurological
PGS001446	EFO_0006930	brain volume measurement|neuroimaging measurement	brain volume measurement	EFO_0006930	Other measurement	Neurological
PGS001447	EFO_0006930	brain volume measurement|neuroimaging measurement	brain volume measurement	EFO_0006930	Other measurement	Neurological
PGS001448	EFO_0006930	brain volume measurement|neuroimaging measurement	brain volume measurement	EFO_0006930	Other measurement	Neurological
PGS001449	EFO_0006930	brain volume measurement|neuroimaging measurement	brain volume measurement	EFO_0006930	Other measurement	Neurological
PGS001450	EFO_0006930	brain volume measurement|neuroimaging measurement	brain volume measurement	EFO_0006930	Other measurement	Neurological
PGS001451	EFO_0006930	brain volume measurement|neuroimaging measurement	brain volume measurement	EFO_0006930	Other measurement	Neurological
PGS001452	EFO_0006930	brain volume measurement|neuroimaging measurement	brain volume measurement	EFO_0006930	Other measurement	Neurological
PGS001453	EFO_0006930	brain volume measurement|neuroimaging measurement	brain volume measurement	EFO_0006930	Other measurement	Neurological
PGS001454	EFO_0006930	brain volume measurement|neuroimaging measurement	brain volume measurement	EFO_0006930	Other measurement	Neurological
PGS001455	EFO_0006930	brain volume measurement|neuroimaging measurement	brain volume measurement	EFO_0006930	Other measurement	Neurological
PGS001456	EFO_0006930	brain volume measurement|neuroimaging measurement	brain volume measurement	EFO_0006930	Other measurement	Neurological
PGS001457	EFO_0006930	brain volume measurement|neuroimaging measurement	brain volume measurement	EFO_0006930	Other measurement	Neurological
PGS001458	EFO_0006930	brain volume measurement|neuroimaging measurement	brain volume measurement	EFO_0006930	Other measurement	Neurological
PGS001459	EFO_0006930	brain volume measurement|neuroimaging measurement	brain volume measurement	EFO_0006930	Other measurement	Neurological
PGS001460	EFO_0006930	brain volume measurement|neuroimaging measurement	brain volume measurement	EFO_0006930	Other measurement	Neurological
PGS001461	EFO_0006930	brain volume measurement|neuroimaging measurement	brain volume measurement	EFO_0006930	Other measurement	Neurological
PGS001462	EFO_0006930	brain volume measurement|neuroimaging measurement	brain volume measurement	EFO_0006930	Other measurement	Neurological
PGS001463	EFO_0006930	brain volume measurement|neuroimaging measurement	brain volume measurement	EFO_0006930	Other measurement	Neurological
PGS001464	EFO_0006930	brain volume measurement|neuroimaging measurement	brain volume measurement	EFO_0006930	Other measurement	Neurological
PGS001465	EFO_0006930	brain volume measurement|neuroimaging measurement	brain volume measurement	EFO_0006930	Other measurement	Neurological
PGS001466	EFO_0006930	brain volume measurement|neuroimaging measurement	brain volume measurement	EFO_0006930	Other measurement	Neurological
PGS001467	EFO_0006930	brain volume measurement|neuroimaging measurement	brain volume measurement	EFO_0006930	Other measurement	Neurological
PGS001468	EFO_0006930	brain volume measurement|neuroimaging measurement	brain volume measurement	EFO_0006930	Other measurement	Neurological

PGS001469	EFO_0006930	brain volume measurement|neuroimaging measurement	brain volume measurement	EFO_0006930	Other measurement	Neurological
PGS001470	EFO_0006930	brain volume measurement|neuroimaging measurement	brain volume measurement	EFO_0006930	Other measurement	Neurological
PGS001471	EFO_0006930	brain volume measurement|neuroimaging measurement	brain volume measurement	EFO_0006930	Other measurement	Neurological
PGS001472	EFO_0006930	brain volume measurement|neuroimaging measurement	brain volume measurement	EFO_0006930	Other measurement	Neurological
PGS001473	EFO_0006930	brain volume measurement|neuroimaging measurement	brain volume measurement	EFO_0006930	Other measurement	Neurological
PGS001474	EFO_0006930	brain volume measurement|neuroimaging measurement	brain volume measurement	EFO_0006930	Other measurement	Neurological
PGS001475	EFO_0006930	brain volume measurement|neuroimaging measurement	brain volume measurement	EFO_0006930	Other measurement	Neurological
PGS001476	EFO_0006930	brain volume measurement|neuroimaging measurement	brain volume measurement	EFO_0006930	Other measurement	Neurological
PGS001477	EFO_0006930	brain volume measurement|neuroimaging measurement	brain volume measurement	EFO_0006930	Other measurement	Neurological
PGS001478	EFO_0006930	brain volume measurement|neuroimaging measurement	brain volume measurement	EFO_0006930	Other measurement	Neurological
PGS001479	EFO_0006930	brain volume measurement|neuroimaging measurement	brain volume measurement	EFO_0006930	Other measurement	Neurological
PGS001480	EFO_0006930	brain volume measurement|neuroimaging measurement	brain volume measurement	EFO_0006930	Other measurement	Neurological
PGS001481	EFO_0006930	brain volume measurement|neuroimaging measurement	brain volume measurement	EFO_0006930	Other measurement	Neurological
PGS001482	EFO_0006930	brain volume measurement|neuroimaging measurement	brain volume measurement	EFO_0006930	Other measurement	Neurological
PGS001483	EFO_0006930	brain volume measurement|neuroimaging measurement	brain volume measurement	EFO_0006930	Other measurement	Neurological
PGS001484	EFO_0006930	brain volume measurement|neuroimaging measurement	brain volume measurement	EFO_0006930	Other measurement	Neurological
PGS001485	EFO_0006930	brain volume measurement|neuroimaging measurement	brain volume measurement	EFO_0006930	Other measurement	Neurological
PGS001486	EFO_0006930	brain volume measurement|neuroimaging measurement	brain volume measurement	EFO_0006930	Other measurement	Neurological
PGS001487	EFO_0006930	brain volume measurement|neuroimaging measurement	brain volume measurement	EFO_0006930	Other measurement	Neurological
PGS001488	EFO_0006930	brain volume measurement|neuroimaging measurement	brain volume measurement	EFO_0006930	Other measurement	Neurological
PGS001489	EFO_0006930	brain volume measurement|neuroimaging measurement	brain volume measurement	EFO_0006930	Other measurement	Neurological
PGS001490	EFO_0006930	brain volume measurement|neuroimaging measurement	brain volume measurement	EFO_0006930	Other measurement	Neurological
PGS001491	EFO_0006930	brain volume measurement|neuroimaging measurement	brain volume measurement	EFO_0006930	Other measurement	Neurological
PGS001492	EFO_0006930	brain volume measurement|neuroimaging measurement	brain volume measurement	EFO_0006930	Other measurement	Neurological
PGS001493	EFO_0006930	brain volume measurement|neuroimaging measurement	brain volume measurement	EFO_0006930	Other measurement	Neurological

PGS001494	EFO_0006930	brain volume measurement|neuroimaging measurement	brain volume measurement	EFO_0006930	Other measurement	Neurological
PGS001495	EFO_0006930	brain volume measurement|neuroimaging measurement	brain volume measurement	EFO_0006930	Other measurement	Neurological
PGS001496	EFO_0006930	brain volume measurement|neuroimaging measurement	brain volume measurement	EFO_0006930	Other measurement	Neurological
PGS001497	EFO_0006930	brain volume measurement|neuroimaging measurement	brain volume measurement	EFO_0006930	Other measurement	Neurological
PGS001498	EFO_0006930	brain volume measurement|neuroimaging measurement	brain volume measurement	EFO_0006930	Other measurement	Neurological
PGS001499	EFO_0006930	brain volume measurement|neuroimaging measurement	brain volume measurement	EFO_0006930	Other measurement	Neurological
PGS001500	EFO_0006930	brain volume measurement|neuroimaging measurement	brain volume measurement	EFO_0006930	Other measurement	Neurological
PGS001501	EFO_0006930	brain volume measurement|neuroimaging measurement	brain volume measurement	EFO_0006930	Other measurement	Neurological
PGS001502	EFO_0006930	brain volume measurement|neuroimaging measurement	brain volume measurement	EFO_0006930	Other measurement	Neurological
PGS001503	EFO_0006930	brain volume measurement|neuroimaging measurement	brain volume measurement	EFO_0006930	Other measurement	Neurological
PGS001504	EFO_0006930	brain volume measurement|neuroimaging measurement	brain volume measurement	EFO_0006930	Other measurement	Neurological
PGS001505	EFO_0006930	brain volume measurement|neuroimaging measurement	brain volume measurement	EFO_0006930	Other measurement	Neurological
PGS001506	EFO_0006930	brain volume measurement|neuroimaging measurement	brain volume measurement	EFO_0006930	Other measurement	Neurological
PGS001642	EFO_0006930	brain volume measurement|neuroimaging measurement	brain volume measurement	EFO_0006930	Other measurement	Neurological
PGS001643	EFO_0006930	brain volume measurement|neuroimaging measurement	brain volume measurement	EFO_0006930	Other measurement	Neurological
PGS001644	EFO_0006930	brain volume measurement|neuroimaging measurement	brain volume measurement	EFO_0006930	Other measurement	Neurological
PGS001645	EFO_0006930	brain volume measurement|neuroimaging measurement	brain volume measurement	EFO_0006930	Other measurement	Neurological
PGS001646	EFO_0006930	brain volume measurement|neuroimaging measurement	brain volume measurement	EFO_0006930	Other measurement	Neurological
PGS001647	EFO_0006930	brain volume measurement|neuroimaging measurement	brain volume measurement	EFO_0006930	Other measurement	Neurological
PGS001648	EFO_0006930	brain volume measurement|neuroimaging measurement	brain volume measurement	EFO_0006930	Other measurement	Neurological
PGS001649	EFO_0006930	brain volume measurement|neuroimaging measurement	brain volume measurement	EFO_0006930	Other measurement	Neurological
PGS001650	EFO_0006930	brain volume measurement|neuroimaging measurement	brain volume measurement	EFO_0006930	Other measurement	Neurological
PGS001651	EFO_0006930	brain volume measurement|neuroimaging measurement	brain volume measurement	EFO_0006930	Other measurement	Neurological
PGS001652	EFO_0006930	brain volume measurement|neuroimaging measurement	brain volume measurement	EFO_0006930	Other measurement	Neurological
PGS001653	EFO_0006930	brain volume measurement|neuroimaging measurement	brain volume measurement	EFO_0006930	Other measurement	Neurological

PGS001654	EFO_0006930	brain volume measurement|neuroimaging measurement	brain volume measurement	EFO_0006930	Other measurement	Neurological
PGS001655	EFO_0006930	brain volume measurement|neuroimaging measurement	brain volume measurement	EFO_0006930	Other measurement	Neurological
PGS001656	EFO_0006930	brain volume measurement|neuroimaging measurement	brain volume measurement	EFO_0006930	Other measurement	Neurological
PGS001657	EFO_0006930	brain volume measurement|neuroimaging measurement	brain volume measurement	EFO_0006930	Other measurement	Neurological
PGS001658	EFO_0006930	brain volume measurement|neuroimaging measurement	brain volume measurement	EFO_0006930	Other measurement	Neurological
PGS001659	EFO_0006930	brain volume measurement|neuroimaging measurement	brain volume measurement	EFO_0006930	Other measurement	Neurological
PGS001660	EFO_0006930	brain volume measurement|neuroimaging measurement	brain volume measurement	EFO_0006930	Other measurement	Neurological
PGS001661	EFO_0006930	brain volume measurement|neuroimaging measurement	brain volume measurement	EFO_0006930	Other measurement	Neurological
PGS001662	EFO_0006930	brain volume measurement|neuroimaging measurement	brain volume measurement	EFO_0006930	Other measurement	Neurological
PGS001663	EFO_0006930	brain volume measurement|neuroimaging measurement	brain volume measurement	EFO_0006930	Other measurement	Neurological
PGS001664	EFO_0006930	brain volume measurement|neuroimaging measurement	brain volume measurement	EFO_0006930	Other measurement	Neurological
PGS001665	EFO_0006930	brain volume measurement|neuroimaging measurement	brain volume measurement	EFO_0006930	Other measurement	Neurological
PGS001666	EFO_0006930	brain volume measurement|neuroimaging measurement	brain volume measurement	EFO_0006930	Other measurement	Neurological
PGS001667	EFO_0006930	brain volume measurement|neuroimaging measurement	brain volume measurement	EFO_0006930	Other measurement	Neurological
PGS001668	EFO_0006930	brain volume measurement|neuroimaging measurement	brain volume measurement	EFO_0006930	Other measurement	Neurological
PGS001669	EFO_0006930	brain volume measurement|neuroimaging measurement	brain volume measurement	EFO_0006930	Other measurement	Neurological
PGS001670	EFO_0006930	brain volume measurement|neuroimaging measurement	brain volume measurement	EFO_0006930	Other measurement	Neurological
PGS001671	EFO_0006930	brain volume measurement|neuroimaging measurement	brain volume measurement	EFO_0006930	Other measurement	Neurological
PGS001672	EFO_0006930	brain volume measurement|neuroimaging measurement	brain volume measurement	EFO_0006930	Other measurement	Neurological
PGS001673	EFO_0006930	brain volume measurement|neuroimaging measurement	brain volume measurement	EFO_0006930	Other measurement	Neurological
PGS001674	EFO_0006930	brain volume measurement|neuroimaging measurement	brain volume measurement	EFO_0006930	Other measurement	Neurological
PGS001675	EFO_0006930	brain volume measurement|neuroimaging measurement	brain volume measurement	EFO_0006930	Other measurement	Neurological
PGS001676	EFO_0006930	brain volume measurement|neuroimaging measurement	brain volume measurement	EFO_0006930	Other measurement	Neurological
PGS001677	EFO_0006930	brain volume measurement|neuroimaging measurement	brain volume measurement	EFO_0006930	Other measurement	Neurological
PGS001678	EFO_0006930	brain volume measurement|neuroimaging measurement	brain volume measurement	EFO_0006930	Other measurement	Neurological

PGS001679	EFO_0006930	brain volume measurement|neuroimaging measurement	brain volume measurement	EFO_0006930	Other measurement	Neurological
PGS001680	EFO_0006930	brain volume measurement|neuroimaging measurement	brain volume measurement	EFO_0006930	Other measurement	Neurological
PGS001681	EFO_0006930	brain volume measurement|neuroimaging measurement	brain volume measurement	EFO_0006930	Other measurement	Neurological
PGS001682	EFO_0006930	brain volume measurement|neuroimaging measurement	brain volume measurement	EFO_0006930	Other measurement	Neurological
PGS001683	EFO_0006930	brain volume measurement|neuroimaging measurement	brain volume measurement	EFO_0006930	Other measurement	Neurological
PGS001684	EFO_0006930	brain volume measurement|neuroimaging measurement	brain volume measurement	EFO_0006930	Other measurement	Neurological
PGS001685	EFO_0006930	brain volume measurement|neuroimaging measurement	brain volume measurement	EFO_0006930	Other measurement	Neurological
PGS001686	EFO_0006930	brain volume measurement|neuroimaging measurement	brain volume measurement	EFO_0006930	Other measurement	Neurological
PGS001687	EFO_0006930	brain volume measurement|neuroimaging measurement	brain volume measurement	EFO_0006930	Other measurement	Neurological
PGS001688	EFO_0006930	brain volume measurement|neuroimaging measurement	brain volume measurement	EFO_0006930	Other measurement	Neurological
PGS001689	EFO_0006930	brain volume measurement|neuroimaging measurement	brain volume measurement	EFO_0006930	Other measurement	Neurological
PGS001690	EFO_0006930	brain volume measurement|neuroimaging measurement	brain volume measurement	EFO_0006930	Other measurement	Neurological
PGS001691	EFO_0006930	brain volume measurement|neuroimaging measurement	brain volume measurement	EFO_0006930	Other measurement	Neurological
PGS001692	EFO_0006930	brain volume measurement|neuroimaging measurement	brain volume measurement	EFO_0006930	Other measurement	Neurological
PGS001693	EFO_0006930	brain volume measurement|neuroimaging measurement	brain volume measurement	EFO_0006930	Other measurement	Neurological
PGS001694	EFO_0006930	brain volume measurement|neuroimaging measurement	brain volume measurement	EFO_0006930	Other measurement	Neurological
PGS001695	EFO_0006930	brain volume measurement|neuroimaging measurement	brain volume measurement	EFO_0006930	Other measurement	Neurological
PGS001696	EFO_0006930	brain volume measurement|neuroimaging measurement	brain volume measurement	EFO_0006930	Other measurement	Neurological
PGS001697	EFO_0006930	brain volume measurement|neuroimaging measurement	brain volume measurement	EFO_0006930	Other measurement	Neurological
PGS001698	EFO_0006930	brain volume measurement|neuroimaging measurement	brain volume measurement	EFO_0006930	Other measurement	Neurological
PGS001699	EFO_0006930	brain volume measurement|neuroimaging measurement	brain volume measurement	EFO_0006930	Other measurement	Neurological
PGS001700	EFO_0006930	brain volume measurement|neuroimaging measurement	brain volume measurement	EFO_0006930	Other measurement	Neurological
PGS001701	EFO_0006930	brain volume measurement|neuroimaging measurement	brain volume measurement	EFO_0006930	Other measurement	Neurological
PGS001702	EFO_0006930	brain volume measurement|neuroimaging measurement	brain volume measurement	EFO_0006930	Other measurement	Neurological
PGS001703	EFO_0006930	brain volume measurement|neuroimaging measurement	brain volume measurement	EFO_0006930	Other measurement	Neurological

PGS001704	EFO_0006930	brain volume measurement|neuroimaging measurement	brain volume measurement	EFO_0006930	Other measurement	Neurological
PGS001705	EFO_0006930	brain volume measurement|neuroimaging measurement	brain volume measurement	EFO_0006930	Other measurement	Neurological
PGS001706	EFO_0006930	brain volume measurement|neuroimaging measurement	brain volume measurement	EFO_0006930	Other measurement	Neurological
PGS001707	EFO_0006930	brain volume measurement|neuroimaging measurement	brain volume measurement	EFO_0006930	Other measurement	Neurological
PGS001708	EFO_0006930	brain volume measurement|neuroimaging measurement	brain volume measurement	EFO_0006930	Other measurement	Neurological
PGS001709	EFO_0006930	brain volume measurement|neuroimaging measurement	brain volume measurement	EFO_0006930	Other measurement	Neurological
PGS001710	EFO_0006930	brain volume measurement|neuroimaging measurement	brain volume measurement	EFO_0006930	Other measurement	Neurological
PGS001711	EFO_0006930	brain volume measurement|neuroimaging measurement	brain volume measurement	EFO_0006930	Other measurement	Neurological
PGS001712	EFO_0006930	brain volume measurement|neuroimaging measurement	brain volume measurement	EFO_0006930	Other measurement	Neurological
PGS001713	EFO_0006930	brain volume measurement|neuroimaging measurement	brain volume measurement	EFO_0006930	Other measurement	Neurological
PGS001714	EFO_0006930	brain volume measurement|neuroimaging measurement	brain volume measurement	EFO_0006930	Other measurement	Neurological
PGS001715	EFO_0006930	brain volume measurement|neuroimaging measurement	brain volume measurement	EFO_0006930	Other measurement	Neurological
PGS001716	EFO_0006930	brain volume measurement|neuroimaging measurement	brain volume measurement	EFO_0006930	Other measurement	Neurological
PGS001717	EFO_0006930	brain volume measurement|neuroimaging measurement	brain volume measurement	EFO_0006930	Other measurement	Neurological
PGS001718	EFO_0006930	brain volume measurement|neuroimaging measurement	brain volume measurement	EFO_0006930	Other measurement	Neurological
PGS001719	EFO_0006930	brain volume measurement|neuroimaging measurement	brain volume measurement	EFO_0006930	Other measurement	Neurological
PGS001720	EFO_0006930	brain volume measurement|neuroimaging measurement	brain volume measurement	EFO_0006930	Other measurement	Neurological
PGS001721	EFO_0006930	brain volume measurement|neuroimaging measurement	brain volume measurement	EFO_0006930	Other measurement	Neurological
PGS001722	EFO_0006930	brain volume measurement|neuroimaging measurement	brain volume measurement	EFO_0006930	Other measurement	Neurological
PGS001723	EFO_0006930	brain volume measurement|neuroimaging measurement	brain volume measurement	EFO_0006930	Other measurement	Neurological
PGS001724	EFO_0006930	brain volume measurement|neuroimaging measurement	brain volume measurement	EFO_0006930	Other measurement	Neurological
PGS001725	EFO_0006930	brain volume measurement|neuroimaging measurement	brain volume measurement	EFO_0006930	Other measurement	Neurological
PGS001726	EFO_0006930	brain volume measurement|neuroimaging measurement	brain volume measurement	EFO_0006930	Other measurement	Neurological
PGS001727	EFO_0006930	brain volume measurement|neuroimaging measurement	brain volume measurement	EFO_0006930	Other measurement	Neurological
PGS001728	EFO_0006930	brain volume measurement|neuroimaging measurement	brain volume measurement	EFO_0006930	Other measurement	Neurological

PGS001729	EFO_0006930	brain volume measurement|neuroimaging measurement	brain volume measurement	EFO_0006930	Other measurement	Neurological
PGS001730	EFO_0006930	brain volume measurement|neuroimaging measurement	brain volume measurement	EFO_0006930	Other measurement	Neurological
PGS001731	EFO_0006930	brain volume measurement|neuroimaging measurement	brain volume measurement	EFO_0006930	Other measurement	Neurological
PGS001732	EFO_0006930	brain volume measurement|neuroimaging measurement	brain volume measurement	EFO_0006930	Other measurement	Neurological
PGS001733	EFO_0006930	brain volume measurement|neuroimaging measurement	brain volume measurement	EFO_0006930	Other measurement	Neurological
PGS001734	EFO_0006930	brain volume measurement|neuroimaging measurement	brain volume measurement	EFO_0006930	Other measurement	Neurological
PGS001735	EFO_0006930	brain volume measurement|neuroimaging measurement	brain volume measurement	EFO_0006930	Other measurement	Neurological
PGS001736	EFO_0006930	brain volume measurement|neuroimaging measurement	brain volume measurement	EFO_0006930	Other measurement	Neurological
PGS001737	EFO_0006930	brain volume measurement|neuroimaging measurement	brain volume measurement	EFO_0006930	Other measurement	Neurological
PGS001738	EFO_0006930	brain volume measurement|neuroimaging measurement	brain volume measurement	EFO_0006930	Other measurement	Neurological
PGS001739	EFO_0006930	brain volume measurement|neuroimaging measurement	brain volume measurement	EFO_0006930	Other measurement	Neurological
PGS001740	EFO_0006930	brain volume measurement|neuroimaging measurement	brain volume measurement	EFO_0006930	Other measurement	Neurological
PGS001741	EFO_0006930	brain volume measurement|neuroimaging measurement	brain volume measurement	EFO_0006930	Other measurement	Neurological
PGS001742	EFO_0006930	brain volume measurement|neuroimaging measurement	brain volume measurement	EFO_0006930	Other measurement	Neurological
PGS001743	EFO_0006930	brain volume measurement|neuroimaging measurement	brain volume measurement	EFO_0006930	Other measurement	Neurological
PGS001744	EFO_0006930	brain volume measurement|neuroimaging measurement	brain volume measurement	EFO_0006930	Other measurement	Neurological
PGS001745	EFO_0006930	brain volume measurement|neuroimaging measurement	brain volume measurement	EFO_0006930	Other measurement	Neurological
PGS001746	EFO_0006930	brain volume measurement|neuroimaging measurement	brain volume measurement	EFO_0006930	Other measurement	Neurological
PGS001747	EFO_0006930	brain volume measurement|neuroimaging measurement	brain volume measurement	EFO_0006930	Other measurement	Neurological
PGS001748	EFO_0006930	brain volume measurement|neuroimaging measurement	brain volume measurement	EFO_0006930	Other measurement	Neurological
PGS001749	EFO_0006930	brain volume measurement|neuroimaging measurement	brain volume measurement	EFO_0006930	Other measurement	Neurological
PGS001750	EFO_0006930	brain volume measurement|neuroimaging measurement	brain volume measurement	EFO_0006930	Other measurement	Neurological
PGS001751	EFO_0006930	brain volume measurement|neuroimaging measurement	brain volume measurement	EFO_0006930	Other measurement	Neurological
PGS001752	EFO_0006930	brain volume measurement|neuroimaging measurement	brain volume measurement	EFO_0006930	Other measurement	Neurological
PGS001753	EFO_0006930	brain volume measurement|neuroimaging measurement	brain volume measurement	EFO_0006930	Other measurement	Neurological

PGS001754	EFO_0006930	brain volume measurement|neuroimaging measurement	brain volume measurement	EFO_0006930	Other measurement	Neurological
PGS001755	EFO_0006930	brain volume measurement|neuroimaging measurement	brain volume measurement	EFO_0006930	Other measurement	Neurological
PGS001756	EFO_0006930	brain volume measurement|neuroimaging measurement	brain volume measurement	EFO_0006930	Other measurement	Neurological
PGS001757	EFO_0006930	brain volume measurement|neuroimaging measurement	brain volume measurement	EFO_0006930	Other measurement	Neurological
PGS001758	EFO_0006930	brain volume measurement|neuroimaging measurement	brain volume measurement	EFO_0006930	Other measurement	Neurological
PGS001759	EFO_0006930	brain volume measurement|neuroimaging measurement	brain volume measurement	EFO_0006930	Other measurement	Neurological
PGS001760	EFO_0006930	brain volume measurement|neuroimaging measurement	brain volume measurement	EFO_0006930	Other measurement	Neurological
PGS001761	EFO_0006930	brain volume measurement|neuroimaging measurement	brain volume measurement	EFO_0006930	Other measurement	Neurological
PGS001762	EFO_0006930	brain volume measurement|neuroimaging measurement	brain volume measurement	EFO_0006930	Other measurement	Neurological
PGS001763	EFO_0006930	brain volume measurement|neuroimaging measurement	brain volume measurement	EFO_0006930	Other measurement	Neurological
PGS001764	EFO_0006930	brain volume measurement|neuroimaging measurement	brain volume measurement	EFO_0006930	Other measurement	Neurological
PGS001765	EFO_0006930	brain volume measurement|neuroimaging measurement	brain volume measurement	EFO_0006930	Other measurement	Neurological
PGS001766	EFO_0006930	brain volume measurement|neuroimaging measurement	brain volume measurement	EFO_0006930	Other measurement	Neurological
PGS001767	EFO_0006930	brain volume measurement|neuroimaging measurement	brain volume measurement	EFO_0006930	Other measurement	Neurological
PGS001768	EFO_0006930	brain volume measurement|neuroimaging measurement	brain volume measurement	EFO_0006930	Other measurement	Neurological
PGS001769	EFO_0006930	brain volume measurement|neuroimaging measurement	brain volume measurement	EFO_0006930	Other measurement	Neurological
PGS001770	EFO_0006930	brain volume measurement|neuroimaging measurement	brain volume measurement	EFO_0006930	Other measurement	Neurological
PGS001771	EFO_0006930	brain volume measurement|neuroimaging measurement	brain volume measurement	EFO_0006930	Other measurement	Neurological
PGS001507	EFO_0004346	neuroimaging measurement	neuroimaging measurement	EFO_0004346	Other measurement	Neurological
PGS001508	EFO_0004346	neuroimaging measurement	neuroimaging measurement	EFO_0004346	Other measurement	Neurological
PGS001509	EFO_0004346	neuroimaging measurement	neuroimaging measurement	EFO_0004346	Other measurement	Neurological
PGS001510	EFO_0004346	neuroimaging measurement	neuroimaging measurement	EFO_0004346	Other measurement	Neurological
PGS001511	EFO_0004346	neuroimaging measurement	neuroimaging measurement	EFO_0004346	Other measurement	Neurological
PGS001512	EFO_0004346	neuroimaging measurement	neuroimaging measurement	EFO_0004346	Other measurement	Neurological
PGS001513	EFO_0004346	neuroimaging measurement	neuroimaging measurement	EFO_0004346	Other measurement	Neurological
PGS001514	EFO_0000408	disease	disease	EFO_0000408	Other disease	Others
PGS001516	EFO_0009431	intestinal disease	intestinal disease	EFO_0009431	Digestive system disorder	Gastrointestinal
PGS001519	EFO_0004517	arterial stiffness measurement	arterial stiffness measurement	EFO_0004517	Cardiovascular measurement	Cardiovascular
PGS001520	EFO_0004517	arterial stiffness measurement	arterial stiffness measurement	EFO_0004517	Cardiovascular measurement	Cardiovascular

PGS001522	HP_0003040	Arthropathy	Arthropathy	HP_0003040	Other trait	Skeletal
PGS001877	HP_0003040	Arthropathy	Arthropathy	HP_0003040	Other trait	Skeletal
PGS002090	HP_0003040	Arthropathy	Arthropathy	HP_0003040	Other trait	Skeletal
PGS001529	EFO_0007772	calcaneal bone quantitative ultrasound measurement	calcaneal bone quantitative ultrasound measurement	EFO_0007772	Other measurement	Skeletal
PGS001530	EFO_0007772	calcaneal bone quantitative ultrasound measurement	calcaneal bone quantitative ultrasound measurement	EFO_0007772	Other measurement	Skeletal
PGS001531	EFO_0007772	calcaneal bone quantitative ultrasound measurement	calcaneal bone quantitative ultrasound measurement	EFO_0007772	Other measurement	Skeletal
PGS001956	EFO_0007772	calcaneal bone quantitative ultrasound measurement	calcaneal bone quantitative ultrasound measurement	EFO_0007772	Other measurement	Skeletal
PGS002174	EFO_0007772	calcaneal bone quantitative ultrasound measurement	calcaneal bone quantitative ultrasound measurement	EFO_0007772	Other measurement	Skeletal
PGS001533	HP_0000360	Tinnitus	Tinnitus	HP_0000360	Other trait	Ear, Nose, Throat
PGS001534	EFO_0005665	white matter hyperintensity measurement|neuroimaging measurement	white matter hyperintensity measurement	EFO_0005665	Other measurement	Neurological
PGS001537	EFO_0006931	nucleus accumbens volume|neuroimaging measurement	nucleus accumbens volume	EFO_0006931	Other measurement	Neurological
PGS001538	EFO_0006931	nucleus accumbens volume|neuroimaging measurement	nucleus accumbens volume	EFO_0006931	Other measurement	Neurological
PGS001539	EFO_0010303	fourth ventricle volume measurement|brain stem volume measurement|neuroimaging measurement	fourth ventricle volume measurement	EFO_0010303	Other measurement	Neurological
PGS001540	EFO_0008320	white matter volume measurement|grey matter volume measurement|neuroimaging measurement	white matter volume measurement	EFO_0008320	Other measurement	Neurological
PGS001541	EFO_0008320	white matter volume measurement|grey matter volume measurement|neuroimaging measurement	white matter volume measurement	EFO_0008320	Other measurement	Neurological
PGS001640	EFO_0008320	white matter volume measurement|neuroimaging measurement	white matter volume measurement	EFO_0008320	Other measurement	Neurological
PGS001641	EFO_0008320	white matter volume measurement|neuroimaging measurement	white matter volume measurement	EFO_0008320	Other measurement	Neurological
PGS001542	EFO_0004830	caudate nucleus volume|neuroimaging measurement	caudate nucleus volume	EFO_0004830	Other measurement	Neurological
PGS001543	EFO_0004830	caudate nucleus volume|neuroimaging measurement	caudate nucleus volume	EFO_0004830	Other measurement	Neurological
PGS001597	EFO_0006516	superior frontal gyrus grey matter volume measurement|neuroimaging measurement	superior frontal gyrus grey matter volume measurement	EFO_0006516	Other measurement	Neurological
PGS001598	EFO_0006516	superior frontal gyrus grey matter volume measurement|neuroimaging measurement	superior frontal gyrus grey matter volume measurement	EFO_0006516	Other measurement	Neurological
PGS001630	EFO_0005035	hippocampal volume|neuroimaging measurement	hippocampal volume	EFO_0005035	Other measurement	Neurological
PGS001631	EFO_0006933	pallidum volume|neuroimaging measurement	pallidum volume	EFO_0006933	Other measurement	Neurological
PGS001632	EFO_0006933	pallidum volume|neuroimaging measurement	pallidum volume	EFO_0006933	Other measurement	Neurological
PGS001635	EFO_0006932	putamen volume|neuroimaging measurement	putamen volume	EFO_0006932	Other measurement	Neurological
PGS001636	EFO_0006932	putamen volume|neuroimaging measurement	putamen volume	EFO_0006932	Other measurement	Neurological
PGS001637	EFO_0006935	thalamus volume|neuroimaging measurement	thalamus volume	EFO_0006935	Other measurement	Neurological
PGS001638	EFO_0006935	thalamus volume|neuroimaging measurement	thalamus volume	EFO_0006935	Other measurement	Neurological
PGS001772	EFO_0009273	anti-Epstein Barr virus antibody measurement	anti-Epstein Barr virus antibody measurement	EFO_0009273	Other measurement	Infection

PGS001773	EFO_0000274	atopic eczema	atopic eczema	EFO_0000274	Immune system disorder	Immunological
PGS002755	EFO_0000274	atopic eczema	atopic eczema	EFO_0000274	Immune system disorder	Immunological
PGS001786	EFO_0600086	appendectomy	appendectomy	EFO_0600086	Other trait	Gastrointestinal
PGS001791	EFO_0000768	idiopathic pulmonary fibrosis	idiopathic pulmonary fibrosis	EFO_0000768	Cancer	Neoplasms
PGS001795	EFO_0002919	uterine carcinoma	uterine carcinoma	EFO_0002919	Cancer	Neoplasms
PGS001797	EFO_0004190	open-angle glaucoma	open-angle glaucoma	EFO_0004190	Other disease	Ophthalmological
PGS002741	EFO_0004190	open-angle glaucoma	open-angle glaucoma	EFO_0004190	Other disease	Ophthalmological
PGS001806	EFO_0004281	testicular neoplasm	testicular neoplasm	EFO_0004281	Other disease	Neoplasms
PGS001810	EFO_0002429	polycythemia vera	polycythemia vera	EFO_0002429	Cancer	Neoplasms
PGS001810	EFO_0002429	polycythemia vera	polycythemia vera	EFO_0002429	Immune system disorder	Immunological
PGS001811	MONDO_0002278	benign colon neoplasm	benign colon neoplasm	MONDO_0002278	Cancer	Neoplasms
PGS001811	MONDO_0002278	benign colon neoplasm	benign colon neoplasm	MONDO_0002278	Digestive system disorder	Gastrointestinal
PGS002019	MONDO_0002278	benign colon neoplasm	benign colon neoplasm	MONDO_0002278	Cancer	Neoplasms
PGS002019	MONDO_0002278	benign colon neoplasm	benign colon neoplasm	MONDO_0002278	Digestive system disorder	Gastrointestinal
PGS001812	MONDO_0000385	benign digestive system neoplasm	benign digestive system neoplasm	MONDO_0000385	Cancer	Neoplasms
PGS001812	MONDO_0000385	benign digestive system neoplasm	benign digestive system neoplasm	MONDO_0000385	Digestive system disorder	Gastrointestinal
PGS002020	MONDO_0000385	benign digestive system neoplasm	benign digestive system neoplasm	MONDO_0000385	Cancer	Neoplasms
PGS002020	MONDO_0000385	benign digestive system neoplasm	benign digestive system neoplasm	MONDO_0000385	Digestive system disorder	Gastrointestinal
PGS001813	MONDO_0000632	uterine benign neoplasm	uterine benign neoplasm	MONDO_0000632	Cancer	Neoplasms
PGS002021	MONDO_0000632	uterine benign neoplasm	uterine benign neoplasm	MONDO_0000632	Cancer	Neoplasms
PGS001814	MONDO_0000334	multinodular goiter|nontoxic goiter	multinodular goiter	MONDO_0000334	Other disease	Endocrine
PGS002022	MONDO_0000334	multinodular goiter|nontoxic goiter	multinodular goiter	MONDO_0000334	Other disease	Endocrine
PGS001820	HP_0001943	Hypoglycemia	Hypoglycemia	HP_0001943	Other trait	Metabolic
PGS002028	HP_0001943	Hypoglycemia	Hypoglycemia	HP_0001943	Other trait	Metabolic
PGS001821	HP_0003119	Abnormal circulating lipid concentration	Abnormal circulating lipid concentration	HP_0003119	Other trait	Metabolic
PGS002029	HP_0003119	Abnormal circulating lipid concentration	Abnormal circulating lipid concentration	HP_0003119	Other trait	Metabolic
PGS001823	MONDO_0002279	iron metabolism disease	iron metabolism disease	MONDO_0002279	Metabolic disorder	Metabolic
PGS002031	MONDO_0002279	iron metabolism disease	iron metabolism disease	MONDO_0002279	Metabolic disorder	Metabolic
PGS001824	Orphanet_309816	Disorder of bilirubin metabolism and excretion	Disorder of bilirubin metabolism and excretion	Orphanet_309816	Metabolic disorder	Metabolic
PGS002032	Orphanet_309816	Disorder of bilirubin metabolism and excretion	Disorder of bilirubin metabolism and excretion	Orphanet_309816	Metabolic disorder	Metabolic

PGS001825	MONDO_0003916	overnutrition|overweight body mass index status|obesity	overnutrition	MONDO_0003916	Metabolic disorder	Metabolic
PGS002033	MONDO_0003916	overnutrition|overweight body mass index status|obesity	overnutrition	MONDO_0003916	Metabolic disorder	Metabolic
PGS001826	MONDO_0015722	congenital vitamin K-dependent coagulation factors deficiency	congenital vitamin K-dependent coagulation factors deficiency	MONDO_0015722	Other disease	Hematological
PGS002034	MONDO_0015722	congenital vitamin K-dependent coagulation factors deficiency	congenital vitamin K-dependent coagulation factors deficiency	MONDO_0015722	Other disease	Hematological
PGS001829	MONDO_0002050	depressive disorder	depressive disorder	MONDO_0002050	Neurological disorder	Neurological
PGS002036	MONDO_0002050	depressive disorder	depressive disorder	MONDO_0002050	Neurological disorder	Neurological
PGS001830	EFO_0003768	nicotine dependence	nicotine dependence	EFO_0003768	Neurological disorder	Neurological
PGS002037	EFO_0003768	nicotine dependence	nicotine dependence	EFO_0003768	Neurological disorder	Neurological
PGS001832	EFO_0009387	peripheral nervous system disease	peripheral nervous system disease	EFO_0009387	Neurological disorder	Neurological
PGS002039	EFO_0009387	peripheral nervous system disease	peripheral nervous system disease	EFO_0009387	Neurological disorder	Neurological
PGS001834	EFO_0001365	age-related macular degeneration	age-related macular degeneration	EFO_0001365	Neurological disorder	Neurological
PGS002041	EFO_0001365	age-related macular degeneration	age-related macular degeneration	EFO_0001365	Neurological disorder	Neurological
PGS001835	MONDO_0018102	corneal dystrophy	corneal dystrophy	MONDO_0018102	Other disease	Ophthalmological
PGS002042	MONDO_0018102	corneal dystrophy	corneal dystrophy	MONDO_0018102	Other disease	Ophthalmological
PGS001839	MONDO_0021661	coronary atherosclerosis	coronary atherosclerosis	MONDO_0021661	Cardiovascular disease	Cardiovascular
PGS002048	MONDO_0021661	coronary atherosclerosis	coronary atherosclerosis	MONDO_0021661	Cardiovascular disease	Cardiovascular
PGS001840	MONDO_0004596	cor pulmonale	cor pulmonale	MONDO_0004596	Cardiovascular disease	Cardiovascular
PGS002049	MONDO_0004596	cor pulmonale	cor pulmonale	MONDO_0004596	Cardiovascular disease	Cardiovascular
PGS001842	EFO_0000373	congestive heart failure	congestive heart failure	EFO_0000373	Cardiovascular disease	Cardiovascular
PGS002051	EFO_0000373	congestive heart failure	congestive heart failure	EFO_0000373	Cardiovascular disease	Cardiovascular
PGS001843	EFO_0003875	peripheral vascular disease	peripheral vascular disease	EFO_0003875	Cardiovascular disease	Cardiovascular
PGS002055	EFO_0003875	peripheral vascular disease	peripheral vascular disease	EFO_0003875	Cardiovascular disease	Cardiovascular
PGS001847	EFO_0004264	vascular disease	vascular disease	EFO_0004264	Cardiovascular disease	Cardiovascular
PGS002059	EFO_0004264	vascular disease	vascular disease	EFO_0004264	Cardiovascular disease	Cardiovascular
PGS001851	EFO_0003948	gastroesophageal reflux disease|esophagitis	gastroesophageal reflux disease	EFO_0003948	Digestive system disorder	Gastrointestinal
PGS002063	EFO_0003948	gastroesophageal reflux disease|esophagitis	gastroesophageal reflux disease	EFO_0003948	Digestive system disorder	Gastrointestinal
PGS001852	MONDO_0004627	duodenitis	duodenitis	MONDO_0004627	Digestive system disorder	Gastrointestinal
PGS001853	EFO_0009542	disorder of appendix	disorder of appendix	EFO_0009542	Digestive system disorder	Gastrointestinal
PGS002064	EFO_0009542	disorder of appendix	disorder of appendix	EFO_0009542	Digestive system disorder	Gastrointestinal
PGS001857	HP_0002253	Colonic diverticula	Colonic diverticula	HP_0002253	Other trait	Gastrointestinal
PGS002068	HP_0002253	Colonic diverticula	Colonic diverticula	HP_0002253	Other trait	Gastrointestinal
PGS001858	EFO_0000405	digestive system disease	digestive system disease	EFO_0000405	Digestive system disorder	Gastrointestinal

PGS002069	EFO_0000405	digestive system disease	digestive system disease	EFO_0000405	Digestive system disorder	Gastrointestinal
PGS001859	MONDO_0060766	anal polyp|polyp of rectum	anal polyp	MONDO_0060766	Digestive system disorder	Gastrointestinal
PGS002070	MONDO_0060766	anal polyp|polyp of rectum	anal polyp	MONDO_0060766	Digestive system disorder	Gastrointestinal
PGS001862	EFO_0009534	biliary tract disease	biliary tract disease	EFO_0009534	Digestive system disorder	Gastrointestinal
PGS002073	EFO_0009534	biliary tract disease	biliary tract disease	EFO_0009534	Digestive system disorder	Gastrointestinal
PGS001863	HP_0000790	Hematuria	Hematuria	HP_0000790	Other trait	Others
PGS002074	HP_0000790	Hematuria	Hematuria	HP_0000790	Other trait	Others
PGS001864	MONDO_0024647	urolithiasis	urolithiasis	MONDO_0024647	Other disease	Genitourinary
PGS002075	MONDO_0024647	urolithiasis	urolithiasis	MONDO_0024647	Other disease	Genitourinary
PGS001866	EFO_0001065	endometriosis	endometriosis	EFO_0001065	Other disease	Genitourinary
PGS002077	EFO_0001065	endometriosis	endometriosis	EFO_0001065	Other disease	Genitourinary
PGS001867	MONDO_0001592	prolapse of female genital organ	prolapse of female genital organ	MONDO_0001592	Other disease	Others
PGS002078	MONDO_0001592	prolapse of female genital organ	prolapse of female genital organ	MONDO_0001592	Other disease	Others
PGS001868	EFO_1001159	Rh isoimmunization	Rh isoimmunization	EFO_1001159	Other disease	Immunological
PGS002080	EFO_1001159	Rh isoimmunization	Rh isoimmunization	EFO_1001159	Other disease	Immunological
PGS001869	EFO_0003035	cellulitis	cellulitis	EFO_0003035	Other disease	Others
PGS002081	EFO_0003035	cellulitis	cellulitis	EFO_0003035	Other disease	Others
PGS001870	MONDO_0004670	lupus erythematosus	lupus erythematosus	MONDO_0004670	Immune system disorder	Immunological
PGS002082	MONDO_0004670	lupus erythematosus	lupus erythematosus	MONDO_0004670	Immune system disorder	Immunological
PGS001872	EFO_1000767	skin sarcoidosis	skin sarcoidosis	EFO_1000767	Other disease	Others
PGS002084	EFO_1000767	skin sarcoidosis	skin sarcoidosis	EFO_1000767	Other disease	Others
PGS001873	MONDO_0002917	disorder of pilosebaceous unit	disorder of pilosebaceous unit	MONDO_0002917	Other disease	Dermatological
PGS002086	MONDO_0002917	disorder of pilosebaceous unit	disorder of pilosebaceous unit	MONDO_0002917	Other disease	Dermatological
PGS001874	EFO_1000243	Epidermal Inclusion Cyst	Epidermal Inclusion Cyst	EFO_1000243	Cancer	Neoplasms
PGS002087	EFO_1000243	Epidermal Inclusion Cyst	Epidermal Inclusion Cyst	EFO_1000243	Cancer	Neoplasms
PGS001879	MONDO_0004874	ganglion or cyst of synovium|tendon|bursa	ganglion or cyst of synovium/tendon/bursa	MONDO_0004874	Other disease	Connective Tissue
PGS001881	HP_0001822	Hallux valgus	Hallux valgus	HP_0001822	Other trait	Others
PGS002093	HP_0001822	Hallux valgus	Hallux valgus	HP_0001822	Other trait	Others
PGS001885	EFO_0009482	drug allergy	drug allergy	EFO_0009482	Immune system disorder	Immunological
PGS002097	EFO_0009482	drug allergy	drug allergy	EFO_0009482	Immune system disorder	Immunological
PGS001890	EFO_0008385	visual acuity measurement	visual acuity measurement	EFO_0008385	Other measurement	Ophthalmological
PGS001985	EFO_0008385	visual acuity measurement	visual acuity measurement	EFO_0008385	Other measurement	Ophthalmological
PGS002103	EFO_0008385	visual acuity measurement	visual acuity measurement	EFO_0008385	Other measurement	Ophthalmological

PGS001891	MONDO_0021945	hearing disorder	hearing disorder	MONDO_0021945	Neurological disorder	Neurological
PGS002104	MONDO_0021945	hearing disorder	hearing disorder	MONDO_0021945	Neurological disorder	Neurological
PGS001897	EFO_0003784	skin pigmentation	skin pigmentation	EFO_0003784	Other trait	Dermatological
PGS002110	EFO_0003784	skin pigmentation	skin pigmentation	EFO_0003784	Other trait	Dermatological
PGS001898	EFO_0007006	depressive symptom measurement	depressive symptom measurement	EFO_0007006	Other measurement	Psychiatric
PGS001899	EFO_0007006	depressive symptom measurement	depressive symptom measurement	EFO_0007006	Other measurement	Psychiatric
PGS002111	EFO_0007006	depressive symptom measurement	depressive symptom measurement	EFO_0007006	Other measurement	Psychiatric
PGS002112	EFO_0007006	depressive symptom measurement	depressive symptom measurement	EFO_0007006	Other measurement	Psychiatric
PGS001901	EFO_0004329	alcohol drinking	alcohol drinking	EFO_0004329	Biological process	Psychiatric
PGS002115	EFO_0004329	alcohol drinking	alcohol drinking	EFO_0004329	Biological process	Psychiatric
PGS002909	EFO_0004329	alcohol drinking	alcohol drinking	EFO_0004329	Biological process	Psychiatric
PGS002910	EFO_0004329	alcohol drinking	alcohol drinking	EFO_0004329	Biological process	Psychiatric
PGS002911	EFO_0004329	alcohol drinking	alcohol drinking	EFO_0004329	Biological process	Psychiatric
PGS002912	EFO_0004329	alcohol drinking	alcohol drinking	EFO_0004329	Biological process	Psychiatric
PGS002913	EFO_0004329	alcohol drinking	alcohol drinking	EFO_0004329	Biological process	Psychiatric
PGS001902	EFO_0005094	P wave duration	P wave duration	EFO_0005094	Cardiovascular measurement	Cardiovascular
PGS002116	EFO_0005094	P wave duration	P wave duration	EFO_0005094	Cardiovascular measurement	Cardiovascular
PGS001903	EFO_0600085	PP interval	PP interval	EFO_0600085	Cardiovascular measurement	Cardiovascular
PGS002117	EFO_0600085	PP interval	PP interval	EFO_0600085	Cardiovascular measurement	Cardiovascular
PGS001907	EFO_0004831	RR interval	RR interval	EFO_0004831	Cardiovascular measurement	Cardiovascular
PGS002121	EFO_0004831	RR interval	RR interval	EFO_0004831	Cardiovascular measurement	Cardiovascular
PGS001912	EFO_0009101	age at first birth measurement	age at first birth measurement	EFO_0009101	Other measurement	Genitourinary
PGS002127	EFO_0009101	age at first birth measurement	age at first birth measurement	EFO_0009101	Other measurement	Genitourinary
PGS001913	EFO_0007786	menstrual cycle measurement	menstrual cycle measurement	EFO_0007786	Other measurement	Genitourinary
PGS002128	EFO_0007786	menstrual cycle measurement	menstrual cycle measurement	EFO_0007786	Other measurement	Genitourinary
PGS002129	EFO_0007786	menstrual cycle measurement	menstrual cycle measurement	EFO_0007786	Other measurement	Genitourinary
PGS001915	EFO_0004703	age at menarche	age at menarche	EFO_0004703	Other measurement	Genitourinary
PGS002131	EFO_0004703	age at menarche	age at menarche	EFO_0004703	Other measurement	Genitourinary
PGS002310	EFO_0004703	age at menarche	age at menarche	EFO_0004703	Other measurement	Genitourinary
PGS002382	EFO_0004703	age at menarche	age at menarche	EFO_0004703	Other measurement	Genitourinary
PGS002431	EFO_0004703	age at menarche	age at menarche	EFO_0004703	Other measurement	Genitourinary
PGS002529	EFO_0004703	age at menarche	age at menarche	EFO_0004703	Other measurement	Genitourinary
PGS002578	EFO_0004703	age at menarche	age at menarche	EFO_0004703	Other measurement	Genitourinary
PGS002815	EFO_0004703	age at menarche	age at menarche	EFO_0004703	Other measurement	Genitourinary
PGS002816	EFO_0004703	age at menarche	age at menarche	EFO_0004703	Other measurement	Genitourinary
PGS002817	EFO_0004703	age at menarche	age at menarche	EFO_0004703	Other measurement	Genitourinary
PGS002818	EFO_0004703	age at menarche	age at menarche	EFO_0004703	Other measurement	Genitourinary
PGS002819	EFO_0004703	age at menarche	age at menarche	EFO_0004703	Other measurement	Genitourinary
PGS001921	EFO_0003931	bone fracture	bone fracture	EFO_0003931	Other disease	Skeletal
PGS002137	EFO_0003931	bone fracture	bone fracture	EFO_0003931	Other disease	Skeletal
PGS001928	EFO_0009550	headache disorder	headache disorder	EFO_0009550	Neurological disorder	Neurological
PGS002145	EFO_0009550	headache disorder	headache disorder	EFO_0009550	Neurological disorder	Neurological

PGS001931	EFO_0009695	household income	household income	EFO_0009695	Other trait	Others
PGS002148	EFO_0009695	household income	household income	EFO_0009695	Other trait	Others
PGS001932	EFO_0004698	insomnia	insomnia	EFO_0004698	Neurological disorder	Neurological
PGS002149	EFO_0004698	insomnia	insomnia	EFO_0004698	Neurological disorder	Neurological
PGS003319	EFO_0004698	insomnia	insomnia	EFO_0004698	Neurological disorder	Neurological
PGS003320	EFO_0004698	insomnia	insomnia	EFO_0004698	Neurological disorder	Neurological
PGS003321	EFO_0004698	insomnia	insomnia	EFO_0004698	Neurological disorder	Neurological
PGS003322	EFO_0004698	insomnia	insomnia	EFO_0004698	Neurological disorder	Neurological
PGS003323	EFO_0004698	insomnia	insomnia	EFO_0004698	Neurological disorder	Neurological
PGS003324	EFO_0004698	insomnia	insomnia	EFO_0004698	Neurological disorder	Neurological
PGS003325	EFO_0004698	insomnia	insomnia	EFO_0004698	Neurological disorder	Neurological
PGS003326	EFO_0004698	insomnia	insomnia	EFO_0004698	Neurological disorder	Neurological
PGS003327	EFO_0004698	insomnia	insomnia	EFO_0004698	Neurological disorder	Neurological
PGS003328	EFO_0004698	insomnia	insomnia	EFO_0004698	Neurological disorder	Neurological
PGS001957	EFO_0005654	velocity of sound measurement	velocity of sound measurement	EFO_0005654	Other measurement	Others
PGS002175	EFO_0005654	velocity of sound measurement	velocity of sound measurement	EFO_0005654	Other measurement	Others
PGS001966	EFO_0007117	carotid artery intima media thickness	carotid artery intima media thickness	EFO_0007117	Other measurement	Cardiovascular
PGS002184	EFO_0007117	carotid artery intima media thickness	carotid artery intima media thickness	EFO_0007117	Other measurement	Cardiovascular
PGS001980	EFO_0011005	urea measurement	urea measurement	EFO_0011005	Other measurement	Metabolic
PGS002198	EFO_0011005	urea measurement	urea measurement	EFO_0011005	Other measurement	Metabolic
PGS001981	EFO_0007928	ventricular rate measurement	ventricular rate measurement	EFO_0007928	Cardiovascular measurement	Cardiovascular
PGS002199	EFO_0007928	ventricular rate measurement	ventricular rate measurement	EFO_0007928	Cardiovascular measurement	Cardiovascular
PGS001987	EFO_0007825	balding measurement	balding measurement	EFO_0007825	Other measurement	Activities
PGS002204	EFO_0007825	balding measurement	balding measurement	EFO_0007825	Other measurement	Activities
PGS002314	EFO_0007825	balding measurement	balding measurement	EFO_0007825	Other measurement	Activities
PGS002386	EFO_0007825	balding measurement	balding measurement	EFO_0007825	Other measurement	Activities
PGS002435	EFO_0007825	balding measurement	balding measurement	EFO_0007825	Other measurement	Activities
PGS002484	EFO_0007825	balding measurement	balding measurement	EFO_0007825	Other measurement	Activities
PGS002533	EFO_0007825	balding measurement	balding measurement	EFO_0007825	Other measurement	Activities
PGS002582	EFO_0007825	balding measurement	balding measurement	EFO_0007825	Other measurement	Activities
PGS002631	EFO_0007825	balding measurement	balding measurement	EFO_0007825	Other measurement	Activities
PGS001994	HP_0000545	Myopia	Myopia	HP_0000545	Other trait	Ophthalmological
PGS002211	HP_0000545	Myopia	Myopia	HP_0000545	Other trait	Ophthalmological
PGS001996	EFO_0007660	neuroticism measurement	neuroticism measurement	EFO_0007660	Other measurement	Psychiatric
PGS002213	EFO_0007660	neuroticism measurement	neuroticism measurement	EFO_0007660	Other measurement	Psychiatric
PGS002342	EFO_0007660	neuroticism measurement	neuroticism measurement	EFO_0007660	Other measurement	Psychiatric
PGS002414	EFO_0007660	neuroticism measurement	neuroticism measurement	EFO_0007660	Other measurement	Psychiatric
PGS002463	EFO_0007660	neuroticism measurement	neuroticism measurement	EFO_0007660	Other measurement	Psychiatric
PGS002561	EFO_0007660	neuroticism measurement	neuroticism measurement	EFO_0007660	Other measurement	Psychiatric
PGS002610	EFO_0007660	neuroticism measurement	neuroticism measurement	EFO_0007660	Other measurement	Psychiatric
PGS002000	EFO_0007652	health trait	health trait	EFO_0007652	Other trait	Others
PGS002004	EFO_0007652	health trait	health trait	EFO_0007652	Other trait	Others
PGS002218	EFO_0007652	health trait	health trait	EFO_0007652	Other trait	Others
PGS002223	EFO_0007652	health trait	health trait	EFO_0007652	Other trait	Others
PGS002008	EFO_0004503	hematological measurement	hematological measurement	EFO_0004503	Hematological measurement	Hematological
PGS002227	EFO_0004503	hematological measurement	hematological measurement	EFO_0004503	Hematological measurement	Hematological

PGS002011	EFO_0004315	drinking behavior	drinking behavior	EFO_0004315	Biological process	Psychiatric
PGS002230	EFO_0004315	drinking behavior	drinking behavior	EFO_0004315	Biological process	Psychiatric
PGS002012	EFO_0011015	educational attainment	educational attainment	EFO_0011015	Other measurement	Activities
PGS002231	EFO_0011015	educational attainment	educational attainment	EFO_0011015	Other measurement	Activities
PGS002319	EFO_0011015	educational attainment	educational attainment	EFO_0011015	Other measurement	Activities
PGS002391	EFO_0011015	educational attainment	educational attainment	EFO_0011015	Other measurement	Activities
PGS002440	EFO_0011015	educational attainment	educational attainment	EFO_0011015	Other measurement	Activities
PGS002489	EFO_0011015	educational attainment	educational attainment	EFO_0011015	Other measurement	Activities
PGS002538	EFO_0011015	educational attainment	educational attainment	EFO_0011015	Other measurement	Activities
PGS002587	EFO_0011015	educational attainment	educational attainment	EFO_0011015	Other measurement	Activities
PGS002045	EFO_0003966	eye disease|eye inflammation	eye disease	EFO_0003966	Other disease	Ophthalmological
PGS002052	EFO_0009677	occlusion precerebral artery	occlusion precerebral artery	EFO_0009677	Cardiovascular disease	Cardiovascular
PGS002052	EFO_0009677	occlusion precerebral artery	occlusion precerebral artery	EFO_0009677	Neurological disorder	Neurological
PGS002053	EFO_0003763	cerebrovascular disorder	cerebrovascular disorder	EFO_0003763	Cardiovascular disease	Cardiovascular
PGS002053	EFO_0003763	cerebrovascular disorder	cerebrovascular disorder	EFO_0003763	Neurological disorder	Neurological
PGS002079	MONDO_0015860	anomaly of puberty or|and menstrual cycle	anomaly of puberty or|and menstrual cycle	MONDO_0015860	Other disease	Genitourinary
PGS002098	EFO_0005203	eating disorder	eating disorder	EFO_0005203	Metabolic disorder	Metabolic
PGS002098	EFO_0005203	eating disorder	eating disorder	EFO_0005203	Neurological disorder	Neurological
PGS002113	EFO_0010703	trauma exposure measurement|depressive episode measurement	trauma exposure measurement	EFO_0010703	Other measurement	Psychiatric
PGS002124	EFO_0004347	addictive behaviour	addictive behaviour	EFO_0004347	Biological process	Psychiatric
PGS002144	EFO_0008568	Sleep Disorder	Sleep Disorder	EFO_0008568	Neurological disorder	Neurological
PGS002151	EFO_0009902	handedness	handedness	EFO_0009902	Other trait	Cognitive
PGS002222	HP_0100716	Self-injurious behavior	Self-injurious behavior	HP_0100716	Other trait	Psychiatric
PGS002233	EFO_0007794	nicotine metabolite ratio	nicotine metabolite ratio	EFO_0007794	Other measurement	Psychiatric
PGS002234	EFO_0007794	nicotine metabolite ratio	nicotine metabolite ratio	EFO_0007794	Other measurement	Psychiatric
PGS002236	EFO_0020865	aortic measurement	aortic measurement	EFO_0020865	Cardiovascular measurement	Cardiovascular
PGS002261	EFO_0000621	neuroblastoma	neuroblastoma	EFO_0000621	Cancer	Neoplasms
PGS002261	EFO_0000621	neuroblastoma	neuroblastoma	EFO_0000621	Neurological disorder	Neurological
PGS002264	EFO_0002517	pancreatic ductal adenocarcinoma	pancreatic ductal adenocarcinoma	EFO_0002517	Cancer	Neoplasms
PGS002264	EFO_0002517	pancreatic ductal adenocarcinoma	pancreatic ductal adenocarcinoma	EFO_0002517	Digestive system disorder	Gastrointestinal
PGS002266	EFO_0003959	cleft lip|Cleft palate	cleft lip	EFO_0003959	Other trait	Others
PGS002267	EFO_0005278	cardiovascular disease biomarker measurement|aortic measurement	cardiovascular disease biomarker measurement	EFO_0005278	Cardiovascular measurement	Cardiovascular
PGS002271	HP_0001634	Mitral valve prolapse	Mitral valve prolapse	HP_0001634	Cardiovascular disease	Cardiovascular
PGS002272	MONDO_0100096	COVID-19	COVID-19	MONDO_0100096	Other disease	Infection
PGS002273	MONDO_0100096	COVID-19	COVID-19	MONDO_0100096	Other disease	Infection
PGS002288	EFO_0004710	pelvic organ prolapse	pelvic organ prolapse	EFO_0004710	Other disease	Others
PGS002292	MONDO_0015486	keratoconus	keratoconus	MONDO_0015486	Other disease	Ophthalmological
PGS002295	EFO_0004628	insulin like growth factor measurement	insulin like growth factor measurement	EFO_0004628	Other measurement	Metabolic
PGS002299	MONDO_0001056	gastric cancer	gastric cancer	MONDO_0001056	Cancer	Neoplasms
PGS002299	MONDO_0001056	gastric cancer	gastric cancer	MONDO_0001056	Digestive system disorder	Gastrointestinal

PGS002309	EFO_0009102	number of children ever born measurement	number of children ever born measurement	EFO_0009102	Other measurement	Genitourinary
PGS002381	EFO_0009102	number of children ever born measurement	number of children ever born measurement	EFO_0009102	Other measurement	Genitourinary
PGS002430	EFO_0009102	number of children ever born measurement	number of children ever born measurement	EFO_0009102	Other measurement	Genitourinary
PGS002479	EFO_0009102	number of children ever born measurement	number of children ever born measurement	EFO_0009102	Other measurement	Genitourinary
PGS002528	EFO_0009102	number of children ever born measurement	number of children ever born measurement	EFO_0009102	Other measurement	Genitourinary
PGS002577	EFO_0009102	number of children ever born measurement	number of children ever born measurement	EFO_0009102	Other measurement	Genitourinary
PGS002626	EFO_0009102	number of children ever born measurement	number of children ever born measurement	EFO_0009102	Other measurement	Genitourinary
PGS002312	EFO_0005140	autoimmune disease	autoimmune disease	EFO_0005140	Immune system disorder	Immunological
PGS002359	EFO_0005140	autoimmune disease	autoimmune disease	EFO_0005140	Immune system disorder	Immunological
PGS002384	EFO_0005140	autoimmune disease	autoimmune disease	EFO_0005140	Immune system disorder	Immunological
PGS002433	EFO_0005140	autoimmune disease	autoimmune disease	EFO_0005140	Immune system disorder	Immunological
PGS002482	EFO_0005140	autoimmune disease	autoimmune disease	EFO_0005140	Immune system disorder	Immunological
PGS002531	EFO_0005140	autoimmune disease	autoimmune disease	EFO_0005140	Immune system disorder	Immunological
PGS002580	EFO_0005140	autoimmune disease	autoimmune disease	EFO_0005140	Immune system disorder	Immunological
PGS002629	EFO_0005140	autoimmune disease	autoimmune disease	EFO_0005140	Immune system disorder	Immunological
PGS002315	EFO_0003923	bone density	bone density	EFO_0003923	Other measurement	Skeletal
PGS002387	EFO_0003923	bone density	bone density	EFO_0003923	Other measurement	Skeletal
PGS002436	EFO_0003923	bone density	bone density	EFO_0003923	Other measurement	Skeletal
PGS002485	EFO_0003923	bone density	bone density	EFO_0003923	Other measurement	Skeletal
PGS002534	EFO_0003923	bone density	bone density	EFO_0003923	Other measurement	Skeletal
PGS002583	EFO_0003923	bone density	bone density	EFO_0003923	Other measurement	Skeletal
PGS002632	EFO_0003923	bone density	bone density	EFO_0003923	Other measurement	Skeletal
PGS002317	EFO_0007862	reproductive behaviour measurement	reproductive behaviour measurement	EFO_0007862	Other measurement	Activities
PGS002389	EFO_0007862	reproductive behaviour measurement	reproductive behaviour measurement	EFO_0007862	Other measurement	Activities
PGS002438	EFO_0007862	reproductive behaviour measurement	reproductive behaviour measurement	EFO_0007862	Other measurement	Activities
PGS002487	EFO_0007862	reproductive behaviour measurement	reproductive behaviour measurement	EFO_0007862	Other measurement	Activities
PGS002536	EFO_0007862	reproductive behaviour measurement	reproductive behaviour measurement	EFO_0007862	Other measurement	Activities
PGS002585	EFO_0007862	reproductive behaviour measurement	reproductive behaviour measurement	EFO_0007862	Other measurement	Activities
PGS002634	EFO_0007862	reproductive behaviour measurement	reproductive behaviour measurement	EFO_0007862	Other measurement	Activities
PGS002320	EFO_0000701	skin disease	skin disease	EFO_0000701	Other disease	Dermatological
PGS002392	EFO_0000701	skin disease	skin disease	EFO_0000701	Other disease	Dermatological
PGS002441	EFO_0000701	skin disease	skin disease	EFO_0000701	Other disease	Dermatological
PGS002490	EFO_0000701	skin disease	skin disease	EFO_0000701	Other disease	Dermatological
PGS002539	EFO_0000701	skin disease	skin disease	EFO_0000701	Other disease	Dermatological
PGS002588	EFO_0000701	skin disease	skin disease	EFO_0000701	Other disease	Dermatological
PGS002637	EFO_0000701	skin disease	skin disease	EFO_0000701	Other disease	Dermatological
PGS002324	EFO_0001379	endocrine system disease|diabetes mellitus	endocrine system disease	EFO_0001379	Other disease	Metabolic
PGS002396	EFO_0001379	endocrine system disease|diabetes mellitus	endocrine system disease	EFO_0001379	Other disease	Metabolic
PGS002445	EFO_0001379	endocrine system disease|diabetes mellitus	endocrine system disease	EFO_0001379	Other disease	Metabolic
PGS002543	EFO_0001379	endocrine system disease|diabetes mellitus	endocrine system disease	EFO_0001379	Other disease	Metabolic
PGS002592	EFO_0001379	endocrine system disease|diabetes mellitus	endocrine system disease	EFO_0001379	Other disease	Metabolic
PGS002347	EFO_0000684	respiratory system disease	respiratory system disease	EFO_0000684	Other disease	Skeletal

PGS002419	EFO_0000684	respiratory system disease	respiratory system disease	EFO_0000684	Other disease	Skeletal
PGS002468	EFO_0000684	respiratory system disease	respiratory system disease	EFO_0000684	Other disease	Skeletal
PGS002517	EFO_0000684	respiratory system disease	respiratory system disease	EFO_0000684	Other disease	Skeletal
PGS002566	EFO_0000684	respiratory system disease	respiratory system disease	EFO_0000684	Other disease	Skeletal
PGS002615	EFO_0000684	respiratory system disease	respiratory system disease	EFO_0000684	Other disease	Skeletal
PGS002664	EFO_0000684	respiratory system disease	respiratory system disease	EFO_0000684	Other disease	Skeletal
PGS002351	EFO_1000627	thyroid disease	thyroid disease	EFO_1000627	Other disease	Endocrine
PGS002423	EFO_1000627	thyroid disease	thyroid disease	EFO_1000627	Other disease	Endocrine
PGS002472	EFO_1000627	thyroid disease	thyroid disease	EFO_1000627	Other disease	Endocrine
PGS002521	EFO_1000627	thyroid disease	thyroid disease	EFO_1000627	Other disease	Endocrine
PGS002570	EFO_1000627	thyroid disease	thyroid disease	EFO_1000627	Other disease	Endocrine
PGS002619	EFO_1000627	thyroid disease	thyroid disease	EFO_1000627	Other disease	Endocrine
PGS002668	EFO_1000627	thyroid disease	thyroid disease	EFO_1000627	Other disease	Endocrine
PGS002730	GO_0036273	response to statin|low density lipoprotein cholesterol measurement	response to statin	GO_0036273	Biological process	Metabolic
PGS002730	GO_0036273	response to statin|low density lipoprotein cholesterol measurement	response to statin	GO_0036273	Response to drug	Metabolic
PGS002746	EFO_0003888	attention deficit hyperactivity disorder	attention deficit hyperactivity disorder	EFO_0003888	Neurological disorder	Neurological
PGS002747	EFO_1000218	Digestive System Carcinoma	Digestive System Carcinoma	EFO_1000218	Cancer	Neoplasms
PGS002747	EFO_1000218	Digestive System Carcinoma	Digestive System Carcinoma	EFO_1000218	Digestive system disorder	Gastrointestinal
PGS002760	EFO_0000474	epilepsy	epilepsy	EFO_0000474	Neurological disorder	Neurological
PGS002782	EFO_0005689	non-high density lipoprotein cholesterol measurement	non-high density lipoprotein cholesterol measurement	EFO_0005689	Cardiovascular measurement	Cardiovascular
PGS002782	EFO_0005689	non-high density lipoprotein cholesterol measurement	non-high density lipoprotein cholesterol measurement	EFO_0005689	Lipid or lipoprotein measurement	Metabolic
PGS002786	MONDO_0004985	bipolar disorder	bipolar disorder	MONDO_0004985	Neurological disorder	Neurological
PGS002787	EFO_0009963	bipolar I disorder	bipolar I disorder	EFO_0009963	Neurological disorder	Neurological
PGS002788	EFO_0009964	bipolar II disorder	bipolar II disorder	EFO_0009964	Neurological disorder	Neurological
PGS002811	EFO_0004764	adipose tissue measurement	adipose tissue measurement	EFO_0004764	Other measurement	Body measurement
PGS002812	EFO_0004764	adipose tissue measurement	adipose tissue measurement	EFO_0004764	Other measurement	Body measurement
PGS002820	EFO_0003922	menopause	menopause	EFO_0003922	Other trait	Genitourinary
PGS002821	EFO_0003922	menopause	menopause	EFO_0003922	Other trait	Genitourinary
PGS002822	EFO_0003922	menopause	menopause	EFO_0003922	Other trait	Genitourinary
PGS002823	EFO_0003922	menopause	menopause	EFO_0003922	Other trait	Genitourinary
PGS002824	EFO_0003922	menopause	menopause	EFO_0003922	Other trait	Genitourinary
PGS003039	EFO_0010733	polyunsaturated fatty acid measurement	polyunsaturated fatty acid measurement	EFO_0010733	Lipid or lipoprotein measurement	Metabolic
PGS003040	EFO_0010733	polyunsaturated fatty acid measurement	polyunsaturated fatty acid measurement	EFO_0010733	Lipid or lipoprotein measurement	Metabolic
PGS003041	EFO_0010733	polyunsaturated fatty acid measurement	polyunsaturated fatty acid measurement	EFO_0010733	Lipid or lipoprotein measurement	Metabolic
PGS003042	EFO_0010733	polyunsaturated fatty acid measurement	polyunsaturated fatty acid measurement	EFO_0010733	Lipid or lipoprotein measurement	Metabolic
PGS003043	EFO_0010733	polyunsaturated fatty acid measurement	polyunsaturated fatty acid measurement	EFO_0010733	Lipid or lipoprotein measurement	Metabolic
PGS003154	EFO_0004620	vitamin B12 measurement	vitamin B12 measurement	EFO_0004620	Other measurement	Metabolic
PGS003155	EFO_0004620	vitamin B12 measurement	vitamin B12 measurement	EFO_0004620	Other measurement	Metabolic
PGS003156	EFO_0004620	vitamin B12 measurement	vitamin B12 measurement	EFO_0004620	Other measurement	Metabolic

PGS003157	EFO_0004620	vitamin B12 measurement	vitamin B12 measurement	EFO_0004620	Other measurement	Metabolic
PGS003158	EFO_0004620	vitamin B12 measurement	vitamin B12 measurement	EFO_0004620	Other measurement	Metabolic
PGS003159	EFO_0004620	vitamin B12 measurement	vitamin B12 measurement	EFO_0004620	Other measurement	Metabolic
PGS003160	EFO_0004620	vitamin B12 measurement	vitamin B12 measurement	EFO_0004620	Other measurement	Metabolic
PGS003161	EFO_0004620	vitamin B12 measurement	vitamin B12 measurement	EFO_0004620	Other measurement	Metabolic
PGS003162	EFO_0004620	vitamin B12 measurement	vitamin B12 measurement	EFO_0004620	Other measurement	Metabolic
PGS003163	EFO_0004620	vitamin B12 measurement	vitamin B12 measurement	EFO_0004620	Other measurement	Metabolic
PGS003164	EFO_0004620	vitamin B12 measurement	vitamin B12 measurement	EFO_0004620	Other measurement	Metabolic
PGS003165	EFO_0004620	vitamin B12 measurement	vitamin B12 measurement	EFO_0004620	Other measurement	Metabolic
PGS003166	EFO_0004620	vitamin B12 measurement	vitamin B12 measurement	EFO_0004620	Other measurement	Metabolic
PGS003167	EFO_0004620	vitamin B12 measurement	vitamin B12 measurement	EFO_0004620	Other measurement	Metabolic
PGS003168	EFO_0004620	vitamin B12 measurement	vitamin B12 measurement	EFO_0004620	Other measurement	Metabolic
PGS003169	EFO_0004620	vitamin B12 measurement	vitamin B12 measurement	EFO_0004620	Other measurement	Metabolic
PGS003170	EFO_0004620	vitamin B12 measurement	vitamin B12 measurement	EFO_0004620	Other measurement	Metabolic
PGS003171	EFO_0004620	vitamin B12 measurement	vitamin B12 measurement	EFO_0004620	Other measurement	Metabolic
PGS003172	EFO_0004620	vitamin B12 measurement	vitamin B12 measurement	EFO_0004620	Other measurement	Metabolic
PGS003173	EFO_0004620	vitamin B12 measurement	vitamin B12 measurement	EFO_0004620	Other measurement	Metabolic
PGS003204	EFO_0003877	sleep apnea	sleep apnea	EFO_0003877	Neurological disorder	Neurological
PGS003205	EFO_0003877	sleep apnea	sleep apnea	EFO_0003877	Neurological disorder	Neurological
PGS003206	EFO_0003877	sleep apnea	sleep apnea	EFO_0003877	Neurological disorder	Neurological
PGS003207	EFO_0003877	sleep apnea	sleep apnea	EFO_0003877	Neurological disorder	Neurological
PGS003208	EFO_0003877	sleep apnea	sleep apnea	EFO_0003877	Neurological disorder	Neurological
PGS003209	EFO_0003877	sleep apnea	sleep apnea	EFO_0003877	Neurological disorder	Neurological
PGS003210	EFO_0003877	sleep apnea	sleep apnea	EFO_0003877	Neurological disorder	Neurological
PGS003211	EFO_0003877	sleep apnea	sleep apnea	EFO_0003877	Neurological disorder	Neurological
PGS003212	EFO_0003877	sleep apnea	sleep apnea	EFO_0003877	Neurological disorder	Neurological
PGS003213	EFO_0003877	sleep apnea	sleep apnea	EFO_0003877	Neurological disorder	Neurological
PGS003214	EFO_0003877	sleep apnea	sleep apnea	EFO_0003877	Neurological disorder	Neurological
PGS003215	EFO_0003877	sleep apnea	sleep apnea	EFO_0003877	Neurological disorder	Neurological
PGS003216	EFO_0003877	sleep apnea	sleep apnea	EFO_0003877	Neurological disorder	Neurological
PGS003217	EFO_0003877	sleep apnea	sleep apnea	EFO_0003877	Neurological disorder	Neurological
PGS003218	EFO_0003877	sleep apnea	sleep apnea	EFO_0003877	Neurological disorder	Neurological
PGS003229	EFO_0010472	creatine measurement	creatine measurement	EFO_0010472	Other measurement	Metabolic
PGS003230	EFO_0010472	creatine measurement	creatine measurement	EFO_0010472	Other measurement	Metabolic
PGS003231	EFO_0010472	creatine measurement	creatine measurement	EFO_0010472	Other measurement	Metabolic
PGS003232	EFO_0010472	creatine measurement	creatine measurement	EFO_0010472	Other measurement	Metabolic
PGS003233	EFO_0010472	creatine measurement	creatine measurement	EFO_0010472	Other measurement	Metabolic
PGS003234	EFO_0010472	creatine measurement	creatine measurement	EFO_0010472	Other measurement	Metabolic
PGS003235	EFO_0010472	creatine measurement	creatine measurement	EFO_0010472	Other measurement	Metabolic
PGS003236	EFO_0010472	creatine measurement	creatine measurement	EFO_0010472	Other measurement	Metabolic
PGS003237	EFO_0010472	creatine measurement	creatine measurement	EFO_0010472	Other measurement	Metabolic
PGS003238	EFO_0010472	creatine measurement	creatine measurement	EFO_0010472	Other measurement	Metabolic
PGS003239	EFO_0010472	creatine measurement	creatine measurement	EFO_0010472	Other measurement	Metabolic
PGS003240	EFO_0010472	creatine measurement	creatine measurement	EFO_0010472	Other measurement	Metabolic
PGS003241	EFO_0010472	creatine measurement	creatine measurement	EFO_0010472	Other measurement	Metabolic
PGS003242	EFO_0010472	creatine measurement	creatine measurement	EFO_0010472	Other measurement	Metabolic
PGS003243	EFO_0010472	creatine measurement	creatine measurement	EFO_0010472	Other measurement	Metabolic
PGS003244	EFO_0010472	creatine measurement	creatine measurement	EFO_0010472	Other measurement	Metabolic
PGS003245	EFO_0010472	creatine measurement	creatine measurement	EFO_0010472	Other measurement	Metabolic
PGS003246	EFO_0010472	creatine measurement	creatine measurement	EFO_0010472	Other measurement	Metabolic

PGS003247	EFO_0010472	creatine measurement	creatine measurement	EFO_0010472	Other measurement	Metabolic
PGS003248	EFO_0010472	creatine measurement	creatine measurement	EFO_0010472	Other measurement	Metabolic
PGS003249	EFO_0010472	creatine measurement|glomerular filtration rate	creatine measurement	EFO_0010472	Other measurement	Metabolic
PGS003250	EFO_0010472	creatine measurement|glomerular filtration rate	creatine measurement	EFO_0010472	Other measurement	Metabolic
PGS003251	EFO_0010472	creatine measurement|glomerular filtration rate	creatine measurement	EFO_0010472	Other measurement	Metabolic
PGS003252	EFO_0010472	creatine measurement|glomerular filtration rate	creatine measurement	EFO_0010472	Other measurement	Metabolic
PGS003253	EFO_0010472	creatine measurement|glomerular filtration rate	creatine measurement	EFO_0010472	Other measurement	Metabolic
PGS003254	EFO_0010472	creatine measurement|glomerular filtration rate	creatine measurement	EFO_0010472	Other measurement	Metabolic
PGS003255	EFO_0010472	creatine measurement|glomerular filtration rate	creatine measurement	EFO_0010472	Other measurement	Metabolic
PGS003256	EFO_0010472	creatine measurement|glomerular filtration rate	creatine measurement	EFO_0010472	Other measurement	Metabolic
PGS003257	EFO_0010472	creatine measurement|glomerular filtration rate	creatine measurement	EFO_0010472	Other measurement	Metabolic
PGS003258	EFO_0010472	creatine measurement|glomerular filtration rate	creatine measurement	EFO_0010472	Other measurement	Metabolic
PGS003259	EFO_0010472	creatine measurement|glomerular filtration rate	creatine measurement	EFO_0010472	Other measurement	Metabolic
PGS003260	EFO_0010472	creatine measurement|glomerular filtration rate	creatine measurement	EFO_0010472	Other measurement	Metabolic
PGS003261	EFO_0010472	creatine measurement|glomerular filtration rate	creatine measurement	EFO_0010472	Other measurement	Metabolic
PGS003262	EFO_0010472	creatine measurement|glomerular filtration rate	creatine measurement	EFO_0010472	Other measurement	Metabolic
PGS003263	EFO_0010472	creatine measurement|glomerular filtration rate	creatine measurement	EFO_0010472	Other measurement	Metabolic
PGS003264	EFO_0010472	creatine measurement|glomerular filtration rate	creatine measurement	EFO_0010472	Other measurement	Metabolic
PGS003265	EFO_0010472	creatine measurement|glomerular filtration rate	creatine measurement	EFO_0010472	Other measurement	Metabolic
PGS003266	EFO_0010472	creatine measurement|glomerular filtration rate	creatine measurement	EFO_0010472	Other measurement	Metabolic
PGS003267	EFO_0010472	creatine measurement|glomerular filtration rate	creatine measurement	EFO_0010472	Other measurement	Metabolic
PGS003268	EFO_0010472	creatine measurement|glomerular filtration rate	creatine measurement	EFO_0010472	Other measurement	Metabolic
PGS003269	EFO_0010472	creatine measurement|glomerular filtration rate	creatine measurement	EFO_0010472	Other measurement	Metabolic
PGS003270	EFO_0010472	creatine measurement|glomerular filtration rate	creatine measurement	EFO_0010472	Other measurement	Metabolic
PGS003271	EFO_0010472	creatine measurement|glomerular filtration rate	creatine measurement	EFO_0010472	Other measurement	Metabolic
PGS003272	EFO_0010472	creatine measurement|glomerular filtration rate	creatine measurement	EFO_0010472	Other measurement	Metabolic

PGS003273	EFO_0010472	creatine measurement|glomerular filtration rate	creatine measurement	EFO_0010472	Other measurement	Metabolic
PGS003274	EFO_0010472	creatine measurement|glomerular filtration rate	creatine measurement	EFO_0010472	Other measurement	Metabolic
PGS003275	EFO_0010472	creatine measurement|glomerular filtration rate	creatine measurement	EFO_0010472	Other measurement	Metabolic
PGS003276	EFO_0010472	creatine measurement|glomerular filtration rate	creatine measurement	EFO_0010472	Other measurement	Metabolic
PGS003277	EFO_0010472	creatine measurement|glomerular filtration rate	creatine measurement	EFO_0010472	Other measurement	Metabolic
PGS003278	EFO_0010472	creatine measurement|glomerular filtration rate	creatine measurement	EFO_0010472	Other measurement	Metabolic
PGS003330	EFO_0004630	factor VIII measurement	factor VIII measurement	EFO_0004630	Hematological measurement	Hematological
PGS003354	EFO_0004255	nephrotic syndrome	nephrotic syndrome	EFO_0004255	Other disease	Genitourinary
PGS003360	EFO_0005670	smoking initiation	smoking initiation	EFO_0005670	Biological process	Psychiatric
PGS003364	EFO_0021784	age at initiation of smoking	age at initiation of smoking	EFO_0021784	Other measurement	Psychiatric
PGS003383	EFO_0000673	prostate adenocarcinoma	prostate adenocarcinoma	EFO_0000673	Cancer	Neoplasms
PGS003384	MONDO_0018177	glioblastoma	glioblastoma	MONDO_0018177	Cancer	Neoplasms
PGS003384	MONDO_0018177	glioblastoma	glioblastoma	MONDO_0018177	Neurological disorder	Neurological
PGS003386	EFO_1001951	colorectal carcinoma	colorectal carcinoma	EFO_1001951	Cancer	Neoplasms
PGS003386	EFO_1001951	colorectal carcinoma	colorectal carcinoma	EFO_1001951	Digestive system disorder	Gastrointestinal
PGS003387	EFO_0000478	esophageal adenocarcinoma|Barrett's esophagus	esophageal adenocarcinoma	EFO_0000478	Cancer	Neoplasms
PGS003387	EFO_0000478	esophageal adenocarcinoma|Barrett's esophagus	esophageal adenocarcinoma	EFO_0000478	Digestive system disorder	Gastrointestinal
PGS003388	EFO_0000478	esophageal adenocarcinoma	esophageal adenocarcinoma	EFO_0000478	Cancer	Neoplasms
PGS003388	EFO_0000478	esophageal adenocarcinoma	esophageal adenocarcinoma	EFO_0000478	Digestive system disorder	Gastrointestinal
PGS003390	EFO_0000181	head and neck squamous cell carcinoma	head and neck squamous cell carcinoma	EFO_0000181	Cancer	Neoplasms
PGS003392	EFO_0000708	squamous cell lung carcinoma	squamous cell lung carcinoma	EFO_0000708	Cancer	Neoplasms
PGS003406	EFO_0003870	brain aneurysm	brain aneurysm	EFO_0003870	Cardiovascular disease	Cardiovascular
PGS003406	EFO_0003870	brain aneurysm	brain aneurysm	EFO_0003870	Neurological disorder	Neurological
PGS003407	EFO_0003870	brain aneurysm|male	brain aneurysm	EFO_0003870	Cardiovascular disease	Cardiovascular
PGS003407	EFO_0003870	brain aneurysm|male	brain aneurysm	EFO_0003870	Neurological disorder	Neurological
PGS003408	EFO_0003870	brain aneurysm|female	brain aneurysm	EFO_0003870	Cardiovascular disease	Cardiovascular
PGS003408	EFO_0003870	brain aneurysm|female	brain aneurysm	EFO_0003870	Neurological disorder	Neurological
PGS003409	EFO_0003870	brain aneurysm	brain aneurysm	EFO_0003870	Cardiovascular disease	Cardiovascular
PGS003409	EFO_0003870	brain aneurysm	brain aneurysm	EFO_0003870	Neurological disorder	Neurological
PGS003410	EFO_0003870	brain aneurysm|male	brain aneurysm	EFO_0003870	Cardiovascular disease	Cardiovascular
PGS003410	EFO_0003870	brain aneurysm|male	brain aneurysm	EFO_0003870	Neurological disorder	Neurological
PGS003411	EFO_0003870	brain aneurysm|female	brain aneurysm	EFO_0003870	Cardiovascular disease	Cardiovascular
PGS003411	EFO_0003870	brain aneurysm|female	brain aneurysm	EFO_0003870	Neurological disorder	Neurological
PGS003418	MONDO_0008315	prostate cancer	prostate cancer	MONDO_0008315	Cancer	Neoplasms
PGS003419	MONDO_0008315	prostate cancer	prostate cancer	MONDO_0008315	Cancer	Neoplasms
