# Supplementary material for: The Spanish Polygenic Score reference distribution: a resource for personalized medicine
Source: Eur J Hum Genet. 2025 Apr 24;34(2):270–7. doi: 10.1038/s41431-025-01850-9 (PMC12859145; doi:10.1038/s41431-025-01850-9)
Supplement: Supplementary file 2 — Supplementary Table S2 [file 41431_2025_1850_MOESM2_ESM.pdf]

corr_coef	p_value	fdr	Polygenic Score (PGS) ID	Mapped Trait(s) (EFO ID)	Mapped Trait(s) (EFO label)	Trait (ontology term label)	Trait Identifier (ontology ID)	Trait Category	DOMAIN	Polygenic Score (PGS) ID_2	Mapped Trait(s) (EFO ID)_2	Mapped Trait(s) (EFO label)_2	Trait (ontology term label)_2	Trait Identifier (ontology ID)_2	Trait Category_2	DOMAIN_2
0.9871	0.00E+00	0.00E+00	PGS000008	EFO_1000649	estrogen-receptor positive breast cancer	estrogen-receptor positive breast cancer	EFO_1000649	Cancer	Neoplasms	PGS000007	EFO_0000305	breast carcinoma	breast carcinoma	EFO_0000305	Cancer	Neoplasms
1.0	0.00E+00	0.00E+00	PGS003121	MONDO_0005148	type 2 diabetes mellitus	type 2 diabetes mellitus	MONDO_0005148	Digestive system disorder	Gastrointestinal	PGS003120	MONDO_0005148	type 2 diabetes mellitus	type 2 diabetes mellitus	MONDO_0005148	Metabolic disorder	Metabolic
1.0	0.00E+00	0.00E+00	PGS000455	EFO_0004193	basal cell carcinoma	basal cell carcinoma	EFO_0004193	Cancer	Neoplasms	PGS000453	EFO_0004193	basal cell carcinoma	basal cell carcinoma	EFO_0004193	Cancer	Neoplasms
0.9865	0.00E+00	0.00E+00	PGS002426	MONDO_0005148	type 2 diabetes mellitus	type 2 diabetes mellitus	MONDO_0005148	Digestive system disorder	Gastrointestinal	PGS002393	EFO_0000400	diabetes mellitus	diabetes mellitus	EFO_0000400	Digestive system disorder	Gastrointestinal
0.9865	0.00E+00	0.00E+00	PGS002426	MONDO_0005148	type 2 diabetes mellitus	type 2 diabetes mellitus	MONDO_0005148	Digestive system disorder	Gastrointestinal	PGS002393	EFO_0000400	diabetes mellitus	diabetes mellitus	EFO_0000400	Metabolic disorder	Metabolic
1.0	0.00E+00	0.00E+00	PGS000487	EFO_0000305	breast carcinoma	breast carcinoma	EFO_0000305	Cancer	Neoplasms	PGS000485	EFO_0000305	breast carcinoma	breast carcinoma	EFO_0000305	Cancer	Neoplasms
0.9865	0.00E+00	0.00E+00	PGS002426	MONDO_0005148	type 2 diabetes mellitus	type 2 diabetes mellitus	MONDO_0005148	Metabolic disorder	Metabolic	PGS002393	EFO_0000400	diabetes mellitus	diabetes mellitus	EFO_0000400	Digestive system disorder	Gastrointestinal
0.9865	0.00E+00	0.00E+00	PGS002426	MONDO_0005148	type 2 diabetes mellitus	type 2 diabetes mellitus	MONDO_0005148	Metabolic disorder	Metabolic	PGS002393	EFO_0000400	diabetes mellitus	diabetes mellitus	EFO_0000400	Metabolic disorder	Metabolic
0.995	0.00E+00	0.00E+00	PGS000743	EFO_0000756	melanoma	melanoma	EFO_0000756	Cancer	Neoplasms	PGS002253	EFO_0000756	melanoma	melanoma	EFO_0000756	Cancer	Neoplasms
1.0	0.00E+00	0.00E+00	PGS003021	EFO_0000537	hypertension	hypertension	EFO_0000537	Cardiovascular disease	Cardiovascular	PGS003020	EFO_0000537	hypertension	hypertension	EFO_0000537	Cardiovascular disease	Cardiovascular
1.0	0.00E+00	0.00E+00	PGS000497	EFO_0000305	breast carcinoma	breast carcinoma	EFO_0000305	Cancer	Neoplasms	PGS000498	EFO_0000305	breast carcinoma	breast carcinoma	EFO_0000305	Cancer	Neoplasms
0.9881	0.00E+00	0.00E+00	PGS000468	EFO_0000707	squamous cell carcinoma	squamous cell carcinoma	EFO_0000707	Cancer	Neoplasms	PGS000465	EFO_0000707	squamous cell carcinoma	squamous cell carcinoma	EFO_0000707	Cancer	Neoplasms
0.9974	0.00E+00	0.00E+00	PGS001160	EFO_0005106	body composition measurement	body composition measurement	EFO_0005106	Body measurement	Body measurement	PGS001168	EFO_0004995	lean body mass	lean body mass	EFO_0004995	Other measurement	Skeletal
0.988	0.00E+00	0.00E+00	PGS001234	EFO_0004324	body weights and measures	body weights and measures	EFO_0004324	Body measurement	Body measurement	PGS001165	EFO_0004995	lean body mass	lean body mass	EFO_0004995	Other measurement	Skeletal
1.0	0.00E+00	0.00E+00	PGS002881	EFO_0004458	C-reactive protein measurement	C-reactive protein measurement	EFO_0004458	Inflammatory measurement	Immunological	PGS002880	EFO_0004458	C-reactive protein measurement	C-reactive protein measurement	EFO_0004458	Inflammatory measurement	Immunological
1.0	0.00E+00	0.00E+00	PGS003166	EFO_0004620	vitamin B12 measurement	vitamin B12 measurement	EFO_0004620	Other measurement	Metabolic	PGS003165	EFO_0004620	vitamin B12 measurement	vitamin B12 measurement	EFO_0004620	Other measurement	Metabolic
0.9884	0.00E+00	0.00E+00	PGS002991	EFO_0004339	body height	body height	EFO_0004339	Body measurement	Body measurement	PGS002990	EFO_0004339	body height	body height	EFO_0004339	Body measurement	Body measurement
0.9845	0.00E+00	0.00E+00	PGS003105	MONDO_0005148	type 2 diabetes mellitus	type 2 diabetes mellitus	MONDO_0005148	Digestive system disorder	Gastrointestinal	PGS003120	MONDO_0005148	type 2 diabetes mellitus	type 2 diabetes mellitus	MONDO_0005148	Digestive system disorder	Gastrointestinal
0.9968	0.00E+00	0.00E+00	PGS000930	MONDO_0000831	thrombotic disease	thrombotic disease	MONDO_0000831	Cardiovascular disease	Cardiovascular	PGS001279	EFO_0003827	pulmonary embolism|deep vein thrombosis	pulmonary embolism	EFO_0003827	Cardiovascular disease	Cardiovascular
1.0	0.00E+00	0.00E+00	PGS002862	EFO_0004458	C-reactive protein measurement	C-reactive protein measurement	EFO_0004458	Inflammatory measurement	Immunological	PGS002861	EFO_0004458	C-reactive protein measurement	C-reactive protein measurement	EFO_0004458	Inflammatory measurement	Immunological
1.0	0.00E+00	0.00E+00	PGS003102	MONDO_0005148	type 2 diabetes mellitus	type 2 diabetes mellitus	MONDO_0005148	Digestive system disorder	Gastrointestinal	PGS003117	MONDO_0005148	type 2 diabetes mellitus	type 2 diabetes mellitus	MONDO_0005148	Digestive system disorder	Gastrointestinal
1.0	0.00E+00	0.00E+00	PGS003102	MONDO_0005148	type 2 diabetes mellitus	type 2 diabetes mellitus	MONDO_0005148	Digestive system disorder	Gastrointestinal	PGS003117	MONDO_0005148	type 2 diabetes mellitus	type 2 diabetes mellitus	MONDO_0005148	Metabolic disorder	Metabolic
0.9852	0.00E+00	0.00E+00	PGS000774	EFO_1000649	estrogen-receptor positive breast cancer	estrogen-receptor positive breast cancer	EFO_1000649	Cancer	Neoplasms	PGS000773	EFO_0000305	breast carcinoma	breast carcinoma	EFO_0000305	Cancer	Neoplasms
1.0	0.00E+00	0.00E+00	PGS003116	MONDO_0005148	type 2 diabetes mellitus	type 2 diabetes mellitus	MONDO_0005148	Digestive system disorder	Gastrointestinal	PGS003115	MONDO_0005148	type 2 diabetes mellitus	type 2 diabetes mellitus	MONDO_0005148	Digestive system disorder	Gastrointestinal
1.0	0.00E+00	0.00E+00	PGS003116	MONDO_0005148	type 2 diabetes mellitus	type 2 diabetes mellitus	MONDO_0005148	Digestive system disorder	Gastrointestinal	PGS003115	MONDO_0005148	type 2 diabetes mellitus	type 2 diabetes mellitus	MONDO_0005148	Metabolic disorder	Metabolic
1.0	0.00E+00	0.00E+00	PGS000418	EFO_0000756	melanoma	melanoma	EFO_0000756	Cancer	Neoplasms	PGS000416	EFO_0000756	melanoma	melanoma	EFO_0000756	Cancer	Neoplasms
1.0	0.00E+00	0.00E+00	PGS003116	MONDO_0005148	type 2 diabetes mellitus	type 2 diabetes mellitus	MONDO_0005148	Metabolic disorder	Metabolic	PGS003115	MONDO_0005148	type 2 diabetes mellitus	type 2 diabetes mellitus	MONDO_0005148	Digestive system disorder	Gastrointestinal
1.0	0.00E+00	0.00E+00	PGS003116	MONDO_0005148	type 2 diabetes mellitus	type 2 diabetes mellitus	MONDO_0005148	Metabolic disorder	Metabolic	PGS003115	MONDO_0005148	type 2 diabetes mellitus	type 2 diabetes mellitus	MONDO_0005148	Metabolic disorder	Metabolic
1.0	0.00E+00	0.00E+00	PGS002876	EFO_0004458	C-reactive protein measurement	C-reactive protein measurement	EFO_0004458	Inflammatory measurement	Immunological	PGS002875	EFO_0004458	C-reactive protein measurement	C-reactive protein measurement	EFO_0004458	Inflammatory measurement	Immunological
0.989	0.00E+00	0.00E+00	PGS000791	EFO_0005952	non-Hodgkins lymphoma	non-Hodgkins lymphoma	EFO_0005952	Cancer	Neoplasms	PGS000080	EFO_0005952	non-Hodgkins lymphoma	non-Hodgkins lymphoma	EFO_0005952	Cancer	Neoplasms
0.9845	0.00E+00	0.00E+00	PGS003105	MONDO_0005148	type 2 diabetes mellitus	type 2 diabetes mellitus	MONDO_0005148	Digestive system disorder	Gastrointestinal	PGS003120	MONDO_0005148	type 2 diabetes mellitus	type 2 diabetes mellitus	MONDO_0005148	Metabolic disorder	Metabolic
0.9813	0.00E+00	0.00E+00	PGS002564	EFO_0004305	erythrocyte count	erythrocyte count	EFO_0004305	Hematological measurement	Hematological	PGS002613	EFO_0004305	erythrocyte count	erythrocyte count	EFO_0004305	Hematological measurement	Hematological
0.9834	0.00E+00	0.00E+00	PGS003136	EFO_0004574	total cholesterol measurement	total cholesterol measurement	EFO_0004574	Lipid or lipoprotein measurement	Metabolic	PGS003141	EFO_0004574	total cholesterol measurement	total cholesterol measurement	EFO_0004574	Lipid or lipoprotein measurement	Metabolic
0.9854	0.00E+00	0.00E+00	PGS002286	EFO_0004574	total cholesterol measurement	total cholesterol measurement	EFO_0004574	Lipid or lipoprotein measurement	Metabolic	PGS002285	EFO_0004611	low density lipoprotein cholesterol measurement	measurement	EFO_0004611	Lipid or lipoprotein measurement	Metabolic
0.9854	0.00E+00	0.00E+00	PGS002286	EFO_0004574	total cholesterol measurement	total cholesterol measurement	EFO_0004574	Lipid or lipoprotein measurement	Metabolic	PGS002285	EFO_0004611	low density lipoprotein cholesterol measurement	measurement	EFO_0004611	Cardiovascular measurement	Cardiovascular
1.0	0.00E+00	0.00E+00	PGS000560	EFO_0003893	ovarian neoplasm	ovarian neoplasm	EFO_0003893	Cancer	Neoplasms	PGS000559	EFO_0003893	ovarian neoplasm	ovarian neoplasm	EFO_0003893	Cancer	Neoplasms
1.0	0.00E+00	0.00E+00	PGS003102	MONDO_0005148	type 2 diabetes mellitus	type 2 diabetes mellitus	MONDO_0005148	Metabolic disorder	Metabolic	PGS003117	MONDO_0005148	type 2 diabetes mellitus	type 2 diabetes mellitus	MONDO_0005148	Metabolic disorder	Metabolic
0.9983	0.00E+00	0.00E+00	PGS002569	EFO_0004279	suntan	suntan	EFO_0004279	Other trait	Dermatological	PGS002618	EFO_0004279	suntan	suntan	EFO_0004279	Other trait	Dermatological
1.0	0.00E+00	0.00E+00	PGS003102	MONDO_0005148	type 2 diabetes mellitus	type 2 diabetes mellitus	MONDO_0005148	Metabolic disorder	Metabolic	PGS003117	MONDO_0005148	type 2 diabetes mellitus	type 2 diabetes mellitus	MONDO_0005148	Digestive system disorder	Gastrointestinal
0.989	0.00E+00	0.00E+00	PGS000005	EFO_1000649	estrogen-receptor positive breast cancer	estrogen-receptor positive breast cancer	EFO_1000649	Cancer	Neoplasms	PGS000004	EFO_0000305	breast carcinoma	breast carcinoma	EFO_0000305	Cancer	Neoplasms
0.9828	0.00E+00	0.00E+00	PGS003186	EFO_0004631	vitamin D measurement	vitamin D measurement	EFO_0004631	Lipid or lipoprotein measurement	Metabolic	PGS003185	EFO_0004631	vitamin D measurement	vitamin D measurement	EFO_0004631	Lipid or lipoprotein measurement	Metabolic
0.988	0.00E+00	0.00E+00	PGS002571	EFO_0004574	total cholesterol measurement	total cholesterol measurement	EFO_0004574	Lipid or lipoprotein measurement	Metabolic	PGS002620	EFO_0004574	total cholesterol measurement	total cholesterol measurement	EFO_0004574	Lipid or lipoprotein measurement	Metabolic
0.9845	0.00E+00	0.00E+00	PGS003105	MONDO_0005148	type 2 diabetes mellitus	type 2 diabetes mellitus	MONDO_0005148	Metabolic disorder	Metabolic	PGS003120	MONDO_0005148	type 2 diabetes mellitus	type 2 diabetes mellitus	MONDO_0005148	Digestive system disorder	Gastrointestinal
0.9997	0.00E+00	0.00E+00	PGS000630	EFO_0002892	thyroid carcinoma	thyroid carcinoma	EFO_0002892	Cancer	Neoplasms	PGS000209	EFO_0002892	thyroid carcinoma	thyroid carcinoma	EFO_0002892	Cancer	Neoplasms
0.9938	0.00E+00	0.00E+00	PGS000455	EFO_0004193	basal cell carcinoma	basal cell carcinoma	EFO_0004193	Cancer	Neoplasms	PGS000454	EFO_0004193	basal cell carcinoma	basal cell carcinoma	EFO_0004193	Cancer	Neoplasms
0.9824	0.00E+00	0.00E+00	PGS003224	EFO_0005208	glomerular filtration rate	glomerular filtration rate	EFO_0005208	Other measurement	Genitourinary	PGS003219	EFO_0005208	glomerular filtration rate	glomerular filtration rate	EFO_0005208	Other measurement	Genitourinary
1.0	0.00E+00	0.00E+00	PGS003403	EFO_0004611	low density lipoprotein cholesterol measurement	low density lipoprotein cholesterol measurement	EFO_0004611	Cardiovascular measurement	Cardiovascular	PGS000340	EFO_0004611	low density lipoprotein cholesterol measurement	measurement	EFO_0004611	Lipid or lipoprotein measurement	Metabolic
1.0	0.00E+00	0.00E+00	PGS003403	EFO_0004611	low density lipoprotein cholesterol measurement	low density lipoprotein cholesterol measurement	EFO_0004611	Lipid or lipoprotein measurement	Metabolic	PGS000340	EFO_0004611	low density lipoprotein cholesterol measurement	measurement	EFO_0004611	Cardiovascular measurement	Cardiovascular
0.9823	0.00E+00	0.00E+00	PGS001531	EFO_0007772	calcaneal bone quantitative ultrasound measurement	calcaneal bone quantitative ultrasound measurement	EFO_0007772	Other measurement	Skeletal	PGS000956	EFO_0004514	bone quantitative ultrasound measurement	measurement	EFO_0004514	Other measurement	Skeletal
0.9833	0.00E+00	0.00E+00	PGS000604	EFO_0005088	testicular carcinoma	testicular carcinoma	EFO_0005088	Cancer	Neoplasms	PGS000602	EFO_0005088	testicular carcinoma	testicular carcinoma	EFO_0005088	Cancer	Neoplasms
0.9845	0.00E+00	0.00E+00	PGS003106	MONDO_0005148	type 2 diabetes mellitus	type 2 diabetes mellitus	MONDO_0005148	Digestive system disorder	Gastrointestinal	PGS003120	MONDO_0005148	type 2 diabetes mellitus	type 2 diabetes mellitus	MONDO_0005148	Metabolic disorder	Metabolic
1.0	0.00E+00	0.00E+00	PGS003251	EFO_0010472	creatine measurement|glomerular filtration rate	creatine measurement	EFO_0010472	Other measurement	Metabolic	PGS003250	EFO_0010472	creatine measurement|glomerular filtration rate	creatine measurement	EFO_0010472	Other measurement	Metabolic
0.996	0.00E+00	0.00E+00	PGS002964	EFO_0004339	body height	body height	EFO_0004339	Body measurement	Body measurement	PGS002969	EFO_0004339	body height	body height	EFO_0004339	Body measurement	Body measurement
0.9828	0.00E+00	0.00E+00	PGS001530	EFO_0007772	calcaneal bone quantitative ultrasound measurement	calcaneal bone quantitative ultrasound measurement	EFO_0007772	Other measurement	Skeletal	PGS000954	EFO_0004514	bone quantitative ultrasound measurement	measurement	EFO_0004514	Other measurement	Skeletal
0.9906	0.00E+00	0.00E+00	PGS002986	EFO_0004339	body height	body height	EFO_0004339	Body measurement	Body measurement	PGS002985	EFO_0004339	body height	body height	EFO_0004339	Body measurement	Body measurement
1.0	0.00E+00	0.00E+00	PGS000611	MONDO_0004986	urinary bladder carcinoma	urinary bladder carcinoma	MONDO_0004986	Cancer	Neoplasms	PGS000610	MONDO_0004986	urinary bladder carcinoma	urinary bladder carcinoma	MONDO_0004986	Cancer	Neoplasms
1.0	0.00E+00	0.00E+00	PGS000630	EFO_0002892	thyroid carcinoma	thyroid carcinoma	EFO_0002892	Cancer	Neoplasms	PGS000631	EFO_0002892	thyroid carcinoma	thyroid carcinoma	EFO_0002892	Cancer	Neoplasms
0.9876	0.00E+00	0.00E+00	PGS002551	EFO_0004339	body height	body height	EFO_0004339	Body measurement	Body measurement	PGS002600	EFO_0004339	body height	body height	EFO_0004339	Body measurement	Body measurement
0.9926	0.00E+00	0.00E+00	PGS001349	MONDO_0004975	Alzheimer disease	Alzheimer disease	MONDO_0004975	Neurological disorder	Neurological	PGS000929	MONDO_0001627	dementia	dementia	MONDO_0001627	Neurological disorder	Neurological
0.9919	0.00E+00	0.00E+00	PGS002840	EFO_0004340	body mass index	body mass index	EFO_0004340	Body measurement	Body measurement	PGS002850	EFO_0004340	body mass index	body mass index	EFO_0004340	Body measurement	Body measurement
0.9924	0.00E+00	0.00E+00	PGS002558	EFO_0004527	mean corpuscular hemoglobin	mean corpuscular hemoglobin	EFO_0004527	Hematological measurement	Hematological	PGS002607	EFO_0004527	mean corpuscular hemoglobin	mean corpuscular hemoglobin	EFO_0004527	Hematological measurement	Hematological
1.0	0.00E+00	0.00E+00	PGS003105	MONDO_0005148	type 2 diabetes mellitus	type 2 diabetes mellitus	MONDO_0005148	Metabolic disorder	Metabolic	PGS003106	MONDO_0005148	type 2 diabetes mellitus	type 2 diabetes mellitus	MONDO_0005148	Metabolic disorder	Metabolic
1.0	0.00E+00	0.00E+00	PGS003403	EFO_0004611	low density lipoprotein cholesterol measurement	low density lipoprotein cholesterol measurement	EFO_0004611	Lipid or lipoprotein measurement	Metabolic	PGS000340	EFO_0004611	low density lipoprotein cholesterol measurement	measurement	EFO_0004611	Lipid or lipoprotein measurement	Metabolic
0.9979	0.00E+00	0.00E+00	PGS002549	EFO_0007822	hair colour measurement	hair colour measurement	EFO_0007822	Other measurement	Dermatological	PGS002598	EFO_0007822	hair colour measurement	hair colour measurement	EFO_0007822	Other measurement	Dermatological
0.9958	0.00E+00	0.00E+00	PGS001039	EFO_0009270	heel bone mineral density	heel bone mineral density	EFO_0009270	Other measurement	Skeletal	PGS001404	EFO_0009270	heel bone mineral density	heel bone mineral density	EFO_0009270	Other measurement	Skeletal
0.9993	0.00E+00	0.00E+00	PGS002996	EFO_0000537	hypertension	hypertension	EFO_0000537	Cardiovascular disease	Cardiovascular	PGS002995	EFO_0000537	hypertension	hypertension	EFO_0000537	Cardiovascular disease	Cardiovascular
1.0	0.00E+00	0.00E+00	PGS000427	EFO_0000756	melanoma	melanoma	EFO_0000756	Cancer	Neoplasms	PGS000428	EFO_0000756	melanoma	melanoma	EFO_0000756	Cancer	Neoplasms
0.9845	0.00E+00	0.00E+00	PGS003105	MONDO_0005148	type 2 diabetes mellitus	type 2 diabetes mellitus	MONDO_0005148	Metabolic disorder	Metabolic	PGS003120	MONDO_0005148	type 2 diabetes mellitus	type 2 diabetes mellitus	MONDO_0005148	Metabolic disorder	Metabolic
0.9881	0.00E+00	0.00E+00	PGS002993	EFO_0004339	body height	body height	EFO_0004339	Body measurement	Body measurement	PGS002983	EFO_0004339	body height	body height	EFO_0004339	Body measurement	Body measurement
0.9904	0.00E+00	0.00E+00	PGS002593	EFO_0004842	eosinophil count	eosinophil count	EFO_0004842	Hematological measurement	Hematological	PGS002544	EFO_0004842	eosinophil count	eosinophil count	EFO_0004842	Hematological measurement	Hematological
0.9837	0.00E+00	0.00E+00	PGS001267	EFO_0003898	ankylosing spondylitis	ankylosing spondylitis	EFO_0003898	Immune system disorder	Immunological	PGS002089	EFO_0003898	ankylosing spondylitis	ankylosing spondylitis	EFO_0003898	Immune system disorder	Immunological
0.9922	0.00E+00	0.00E+00	PGS001278	EFO_0003827	pulmonary embolism|deep vein thrombosis	pulmonary embolism	EFO_0003827	Cardiovascular disease	Cardiovascular	PGS000931	MONDO_0000831	thrombotic disease|deep vein thrombosis	thrombotic disease	MONDO_0000831	Cardiovascular disease	Cardiovascular
0.985	0.00E+00	0.00E+00	PGS001824	Orphanet_309816	Disorder of bilirubin metabolism and excretion	Disorder of bilirubin metabolism and excretion	Orphanet_309816	Metabolic disorder	Metabolic	PGS000924	MONDO_0037821	disease	porphyrin metabolism disease	MONDO_0037821	Metabolic disorder	Metabolic
0.9826	0.00E+00	0.00E+00	PGS001349	MONDO_0004975	Alzheimer disease	Alzheimer disease	MONDO_0004975	Neurological disorder	Neurological	PGS000946	HP_0000726	Dementia	Dementia	HP_0000726	Other trait	Psychiatric
1.0	0.00E+00	0.00E+00	PGS003276	EFO_0010472	creatine measurement|glomerular filtration rate	creatine measurement	EFO_0010472	Other measurement	Metabolic	PGS003275	EFO_0010472	creatine measurement|glomerular filtration rate	creatine measurement	EFO_0010472	Other measurement	Metabolic
0.9917	0.00E+00	0.00E+00	PGS000697	EFO_0004570	bilirubin measurement	bilirubin measurement	EFO_0004570	Other measurement	Metabolic	PGS000681	EFO_0004570	bilirubin measurement	bilirubin measurement	EFO_0004570	Other measurement	Metabolic
0.983	0.00E+00	0.00E+00	PGS001179	EFO_0004718	vascular dementia	vascular dementia	EFO_0004718	Cardiovascular disease	Cardiovascular	PGS001348	MONDO_0004975	Alzheimer disease	Alzheimer disease	MONDO_0004975	Neurological disorder	Neurological
0.983	0.00E+00	0.00E+00	PGS001179	EFO_0004718	vascular dementia	vascular dementia	EFO_0004718	Neurological disorder	Neurological	PGS001348	MONDO_0004975	Alzheimer disease	Alzheimer disease	MONDO_0004975	Neurological disorder	Neurological
1.0	0.00E+00	0.00E+00	PGS001180	EFO_0004713	FEV|FEC ratio	FEV|FEC ratio	EFO_0004713	Other measurement	Respiratory	PGS001010	EFO_0009718	peak expiratory flow	peak expiratory flow	EFO_0009718	Other measurement	Respiratory
1.0	0.00E+00	0.00E+00	PGS003315	EFO_0004617	cystatin C measurement|glomerular filtration rate	cystatin C measurement	EFO_0004617	Other measurement	Metabolic	PGS003316	EFO_0004617	cystatin C measurement|glomerular filtration rate	cystatin C measurement	EFO_0004617	Other measurement	Metabolic
0.9916	0.00E+00	0.00E+00	PGS003221	EFO_0005208	glomerular filtration rate	glomerular filtration rate	EFO_0005208	Other measurement	Genitourinary	PGS003220	EFO_0005208	glomerular filtration rate	glomerular filtration rate	EFO_0005208	Other measurement	Genitourinary
1.0	0.00E+00	0.00E+00	PGS003403	EFO_0004611	low density lipoprotein cholesterol measurement	low density lipoprotein cholesterol measurement	EFO_0004611	Cardiovascular measurement	Cardiovascular	PGS000340	EFO_0004611	low density lipoprotein cholesterol measurement	measurement	EFO_0004611	Cardiovascular measurement	Cardiovascular
0.9905	0.00E+00	0.00E+00	PGS002614	EFO_0005192	red blood cell distribution width	red blood cell distribution width	EFO_0005192	Hematological measurement	Hematological	PGS002565	EFO_0005192	red blood cell distribution width	red blood cell distribution width	EFO_0005192	Hematological measurement	Hematological
1.0	0.00E+00	0.00E+00	PGS002817	EFO_0004703	age at menarche	age at menarche	EFO_0004703	Other measurement	Genitourinary	PGS002816	EFO_0004703	age at menarche	age at menarche	EFO_0004703	Other measurement	Genitourinary
0.993	0.00E+00	0.00E+00	PGS002548	EFO_0004612	high density lipoprotein cholesterol measurement	high density lipoprotein cholesterol measurement	EFO_0004612	Lipid or lipoprotein measurement	Metabolic	PGS002597	EFO_0004612	high density lipoprotein cholesterol measurement	measurement	EFO_0004612	Lipid or lipoprotein measurement	Metabolic
1.0	0.00E+00	0.00E+00	PGS000427	EFO_0000756	melanoma	melanoma	EFO_0000756	Cancer	Neoplasms	PGS000425	EFO_0000756	melanoma	melanoma	EFO_0000756	Cancer	Neoplasms
0.9916	0.00E+00	0.00E+00	PGS002904	EFO_0006336	diastolic blood pressure	diastolic blood pressure	EFO_0006336	Other measurement	Cardiovascular	PGS002899	EFO_0006336	diastolic blood pressure	diastolic blood pressure	EFO_0006336	Other measurement	Cardiovascular
1.0	0.00E+00	0.00E+00	PGS003025	EFO_0000537	hypertension	hypertension	EFO_0000537	Cardiovascular disease	Cardiovascular	PGS003026	EFO_0000537	hypertension	hypertension	EFO_0000537	Cardiovascular disease	Cardiovascular
0.9883	0.00E+00	0.00E+00	PGS002609	EFO_0005091	monocyte count	monocyte count	EFO_0005091	Hematological measurement	Hematological	PGS002560	EFO_0005091	monocyte count	monocyte count	EFO_0005091	Hematological measurement	Hematological
0.9835	0.00E+00	0.00E+00	PGS000929	MONDO_0001627	dementia	dementia	MONDO_0001627	Neurological disorder	Neurological	PGS001348	MONDO_0004975	Alzheimer disease	Alzheimer disease	MONDO_0004975	Neurological disorder	Neurological
0.9997	0.00E+00	0.00E+00	PGS003266	EFO_0010472	creatine measurement|glomerular filtration rate	creatine measurement	EFO_0010472	Other measurement	Metabolic	PGS003265	EFO_0010472	creatine measurement|glomerular filtration rate	creatine measurement	EFO_0010472	Other measurement	Metabolic
0.9921	0.00E+00	0.00E+00	PGS002605	EFO_0004611	low density lipoprotein cholesterol measurement	low density lipoprotein cholesterol measurement	EFO_0004611	Cardiovascular measurement	Cardiovascular	PGS002556	EFO_0004611	low density lipoprotein cholesterol measurement	measurement	EFO_0004611	Cardiovascular measurement	Cardiovascular
0.981	0.00E+00	0.00E+00	PGS002393	EFO_0000400	diabetes mellitus	diabetes mellitus	EFO_0000400	Digestive system disorder	Gastrointestinal	PGS002573	MONDO_0005148	type 2 diabetes mellitus	type 2 diabetes mellitus	MONDO_0005148	Metabolic disorder	Metabolic
1.0	0.00E+00	0.00E+00	PGS003121	MONDO_0005148	type 2 diabetes mellitus	type 2 diabetes mellitus	MONDO_0005148	Metabolic disorder	Metabolic	PGS003120	MONDO_0005148	type 2 diabetes mellitus	type 2 diabetes mellitus	MONDO_0005148	Metabolic disorder	Metabolic
0.9921	0.00E+00	0.00E+00	PGS002605	EFO_0004611	low density lipoprotein cholesterol measurement	low density lipoprotein cholesterol measurement	EFO_0004611	Cardiovascular measurement	Cardiovascular	PGS002556	EFO_0004611	low density lipoprotein cholesterol measurement	measurement	EFO_0004611	Lipid or lipoprotein measurement	Metabolic
0.985	0.00E+00	0.00E+00	PGS001169	EFO_0004995	lean body mass	lean body mass	EFO_0004995	Other measurement	Skeletal	PGS001108	EFO_0007777	base metabolic rate measurement	base metabolic rate measurement	EFO_0007777	Other measurement	Metabolic
1.0	0.00E+00	0.00E+00	PGS000612	MONDO_0004986	urinary bladder carcinoma	urinary bladder carcinoma	MONDO_0004986	Cancer	Neoplasms	PGS000610	MONDO_0004986	urinary bladder carcinoma	urinary bladder carcinoma	MONDO_0004986	Cancer	Neoplasms
1.0	0.00E+00	0.00E+00	PGS003121	MONDO_0005148	type 2 diabetes mellitus	type 2 diabetes mellitus	MONDO_0005148	Metabolic disorder	Metabolic	PGS003120	MONDO_0005148	type 2 diabetes mellitus	type 2 diabetes mellitus	MONDO_0005148	Digestive system disorder	Gastrointestinal
0.9839	0.00E+00	0.00E+00	PGS002958	EFO_0004612	high density lipoprotein cholesterol measurement	high density lipoprotein cholesterol measurement	EFO_0004612	Lipid or lipoprotein measurement	Metabolic	PGS002963	EFO_0004612	high density lipoprotein cholesterol measurement	measurement	EFO_0004612	Lipid or lipoprotein measurement	Metabolic
1.0	0.00E+00	0.00E+00	PGS000456	EFO_0004193	basal cell carcinoma	basal cell carcinoma	EFO_0004193	Cancer	Neoplasms	PGS000457	EFO_0004193	basal cell carcinoma	basal cell carcinoma	EFO_0004193	Cancer	Neoplasms
0.9994	0.00E+00	0.00E+00	PGS001158	EFO_0005106	body composition measurement	body composition measurement	EFO_0005106	Body measurement	Body measurement	PGS001166	EFO_0004995	lean body mass	lean body mass	EFO_0004995	Other measurement	Skeletal

1.0	0.00E+00	0.00E+00	PGS000421	EFO_0000756	melanoma	melanoma	EFO_0000756	Cancer	Neoplasms	PGS000422	EFO_0000756	melanoma	melanoma	EFO_0000756	Cancer	Neoplasms
0.9915	0.00E+00	0.00E+00	PGS001145	EFO_0005409	fat body mass	fat body mass	EFO_0005409	Other measurement	Body measurement	PGS001144	EFO_0005409	fat body mass	fat body mass	EFO_0005409	Other measurement	Body measurement
0.9921	0.00E+00	0.00E+00	PGS002605	EFO_0004611	low density lipoprotein cholesterol measurement	low density lipoprotein cholesterol measurement	EFO_0004611	Lipid or lipoprotein measurement	Metabolic	PGS002556	EFO_0004611	low density lipoprotein cholesterol measurement	measurement	EFO_0004611	Lipid or lipoprotein measurement	Metabolic
0.9904	0.00E+00	0.00E+00	PGS002601	EFO_0007986	reticulocyte count	reticulocyte count	EFO_0007986	Hematological measurement	Hematological	PGS002552	EFO_0007986	reticulocyte count	reticulocyte count	EFO_0007986	Hematological measurement	Hematological
0.9969	0.00E+00	0.00E+00	PGS000952	EFO_0004514	bone quantitative ultrasound measurement	bone quantitative ultrasound measurement	EFO_0004514	Other measurement	Skeletal	PGS001402	EFO_0009270	heel bone mineral density	heel bone mineral density	EFO_0009270	Other measurement	Skeletal
0.9921	0.00E+00	0.00E+00	PGS002605	EFO_0004611	low density lipoprotein cholesterol measurement	low density lipoprotein cholesterol measurement	EFO_0004611	Lipid or lipoprotein measurement	Metabolic	PGS002556	EFO_0004611	low density lipoprotein cholesterol measurement	measurement	EFO_0004611	Cardiovascular measurement	Cardiovascular
1.0	0.00E+00	0.00E+00	PGS000578	EFO_0001663	prostate carcinoma	prostate carcinoma	EFO_0001663	Cancer	Neoplasms	PGS000577	EFO_0001663	prostate carcinoma	prostate carcinoma	EFO_0001663	Cancer	Neoplasms
1.0	0.00E+00	0.00E+00	PGS003320	EFO_0004698	insomnia	insomnia	EFO_0004698	Neurological disorder	Neurological	PGS003321	EFO_0004698	insomnia	insomnia	EFO_0004698	Neurological disorder	Neurological
0.9829	0.00E+00	0.00E+00	PGS002422	EFO_0004279	suntan	suntan	EFO_0004279	Other trait	Dermatological	PGS002618	EFO_0004279	suntan	suntan	EFO_0004279	Other trait	Dermatological
0.9845	0.00E+00	0.00E+00	PGS003106	MONDO_0005148	type 2 diabetes mellitus	type 2 diabetes mellitus	MONDO_0005148	Metabolic disorder	Metabolic	PGS003120	MONDO_0005148	type 2 diabetes mellitus	type 2 diabetes mellitus	MONDO_0005148	Metabolic disorder	Metabolic
0.9812	0.00E+00	0.00E+00	PGS002393	EFO_0000400	diabetes mellitus	diabetes mellitus	EFO_0000400	Metabolic disorder	Metabolic	PGS002442	EFO_0000400	diabetes mellitus	diabetes mellitus	EFO_0000400	Metabolic disorder	Metabolic
0.9996	0.00E+00	0.00E+00	PGS003274	EFO_0010472	creatine measurement|glomerular filtration rate	creatine measurement	EFO_0010472	Other measurement	Metabolic	PGS003259	EFO_0010472	creatine measurement|glomerular filtration rate	creatine measurement	EFO_0010472	Other measurement	Metabolic
0.9923	0.00E+00	0.00E+00	PGS000866	EFO_0010821	liver fat measurement	liver fat measurement	EFO_0010821	Other measurement	Gastrointestinal	PGS000872	EFO_0003095	non-alcoholic fatty liver disease	non-alcoholic fatty liver disease	EFO_0003095	Digestive system disorder	Gastrointestinal
0.9845	0.00E+00	0.00E+00	PGS003106	MONDO_0005148	type 2 diabetes mellitus	type 2 diabetes mellitus	MONDO_0005148	Digestive system disorder	Gastrointestinal	PGS003120	MONDO_0005148	type 2 diabetes mellitus	type 2 diabetes mellitus	MONDO_0005148	Digestive system disorder	Gastrointestinal
0.9812	0.00E+00	0.00E+00	PGS002393	EFO_0000400	diabetes mellitus	diabetes mellitus	EFO_0000400	Metabolic disorder	Metabolic	PGS002442	EFO_0000400	diabetes mellitus	diabetes mellitus	EFO_0000400	Digestive system disorder	Gastrointestinal
0.9812	0.00E+00	0.00E+00	PGS002393	EFO_0000400	diabetes mellitus	diabetes mellitus	EFO_0000400	Digestive system disorder	Gastrointestinal	PGS002442	EFO_0000400	diabetes mellitus	diabetes mellitus	EFO_0000400	Metabolic disorder	Metabolic
0.9812	0.00E+00	0.00E+00	PGS002393	EFO_0000400	diabetes mellitus	diabetes mellitus	EFO_0000400	Digestive system disorder	Gastrointestinal	PGS002442	EFO_0000400	diabetes mellitus	diabetes mellitus	EFO_0000400	Digestive system disorder	Gastrointestinal
1.0	0.00E+00	0.00E+00	PGS000489	EFO_0000305	breast carcinoma	breast carcinoma	EFO_0000305	Cancer	Neoplasms	PGS000490	EFO_0000305	breast carcinoma	breast carcinoma	EFO_0000305	Cancer	Neoplasms
0.9985	0.00E+00	0.00E+00	PGS003029	EFO_0004611	low density lipoprotein cholesterol measurement	low density lipoprotein cholesterol measurement	EFO_0004611	Lipid or lipoprotein measurement	Metabolic	PGS003034	EFO_0004611	low density lipoprotein cholesterol measurement	measurement	EFO_0004611	Lipid or lipoprotein measurement	Metabolic
0.9985	0.00E+00	0.00E+00	PGS003029	EFO_0004611	low density lipoprotein cholesterol measurement	low density lipoprotein cholesterol measurement	EFO_0004611	Lipid or lipoprotein measurement	Metabolic	PGS003034	EFO_0004611	low density lipoprotein cholesterol measurement	measurement	EFO_0004611	Cardiovascular measurement	Cardiovascular
0.9845	0.00E+00	0.00E+00	PGS003105	MONDO_0005148	type 2 diabetes mellitus	type 2 diabetes mellitus	MONDO_0005148	Digestive system disorder	Gastrointestinal	PGS003121	MONDO_0005148	type 2 diabetes mellitus	type 2 diabetes mellitus	MONDO_0005148	Metabolic disorder	Metabolic
0.9985	0.00E+00	0.00E+00	PGS003029	EFO_0004611	low density lipoprotein cholesterol measurement	low density lipoprotein cholesterol measurement	EFO_0004611	Cardiovascular measurement	Cardiovascular	PGS003034	EFO_0004611	low density lipoprotein cholesterol measurement	measurement	EFO_0004611	Lipid or lipoprotein measurement	Metabolic
0.9985	0.00E+00	0.00E+00	PGS003029	EFO_0004611	low density lipoprotein cholesterol measurement	low density lipoprotein cholesterol measurement	EFO_0004611	Cardiovascular measurement	Cardiovascular	PGS003034	EFO_0004611	low density lipoprotein cholesterol measurement	measurement	EFO_0004611	Cardiovascular measurement	Cardiovascular
0.9845	0.00E+00	0.00E+00	PGS003105	MONDO_0005148	type 2 diabetes mellitus	type 2 diabetes mellitus	MONDO_0005148	Metabolic disorder	Metabolic	PGS003121	MONDO_0005148	type 2 diabetes mellitus	type 2 diabetes mellitus	MONDO_0005148	Digestive system disorder	Gastrointestinal
0.9845	0.00E+00	0.00E+00	PGS003105	MONDO_0005148	type 2 diabetes mellitus	type 2 diabetes mellitus	MONDO_0005148	Metabolic disorder	Metabolic	PGS003121	MONDO_0005148	type 2 diabetes mellitus	type 2 diabetes mellitus	MONDO_0005148	Metabolic disorder	Metabolic
0.9946	0.00E+00	0.00E+00	PGS002559	EFO_0004584	mean platelet volume	mean platelet volume	EFO_0004584	Hematological measurement	Hematological	PGS002608	EFO_0004584	mean platelet volume	mean platelet volume	EFO_0004584	Hematological measurement	Hematological
0.981	0.00E+00	0.00E+00	PGS002393	EFO_0000400	diabetes mellitus	diabetes mellitus	EFO_0000400	Digestive system disorder	Gastrointestinal	PGS002573	MONDO_0005148	type 2 diabetes mellitus	type 2 diabetes mellitus	MONDO_0005148	Digestive system disorder	Gastrointestinal
0.9845	0.00E+00	0.00E+00	PGS003105	MONDO_0005148	type 2 diabetes mellitus	type 2 diabetes mellitus	MONDO_0005148	Digestive system disorder	Gastrointestinal	PGS003121	MONDO_0005148	type 2 diabetes mellitus	type 2 diabetes mellitus	MONDO_0005148	Digestive system disorder	Gastrointestinal
1.0	0.00E+00	0.00E+00	PGS003105	MONDO_0005148	type 2 diabetes mellitus	type 2 diabetes mellitus	MONDO_0005148	Metabolic disorder	Metabolic	PGS003106	MONDO_0005148	type 2 diabetes mellitus	type 2 diabetes mellitus	MONDO_0005148	Digestive system disorder	Gastrointestinal
0.9881	0.00E+00	0.00E+00	PGS000468	EFO_0000707	squamous cell carcinoma	squamous cell carcinoma	EFO_0000707	Cancer	Neoplasms	PGS000466	EFO_0000707	squamous cell carcinoma	squamous cell carcinoma	EFO_0000707	Cancer	Neoplasms
0.9964	0.00E+00	0.00E+00	PGS003096	MONDO_0005148	type 2 diabetes mellitus	type 2 diabetes mellitus	MONDO_0005148	Digestive system disorder	Gastrointestinal	PGS003095	MONDO_0005148	type 2 diabetes mellitus	type 2 diabetes mellitus	MONDO_0005148	Digestive system disorder	Gastrointestinal
1.0	0.00E+00	0.00E+00	PGS000461	EFO_0000707	squamous cell carcinoma	squamous cell carcinoma	EFO_0000707	Cancer	Neoplasms	PGS000462	EFO_0000707	squamous cell carcinoma	squamous cell carcinoma	EFO_0000707	Cancer	Neoplasms
0.9898	0.00E+00	0.00E+00	PGS001388	EFO_0005671	smoking behaviour measurement	smoking behaviour measurement	EFO_0005671	Other measurement	Psychiatric	PGS001515	EFO_0006525	cigarettes per day measurement	cigarettes per day measurement	EFO_0006525	Other measurement	Psychiatric
1.0	0.00E+00	0.00E+00	PGS001038	EFO_0009270	heel bone mineral density	heel bone mineral density	EFO_0009270	Other measurement	Skeletal	PGS000954	EFO_0004514	bone quantitative ultrasound measurement	measurement	EFO_0004514	Other measurement	Skeletal
1.0	0.00E+00	0.00E+00	PGS000647	EFO_0000095	chronic lymphocytic leukemia	chronic lymphocytic leukemia	EFO_0000095	Cancer	Neoplasms	PGS000646	EFO_0000095	chronic lymphocytic leukemia	chronic lymphocytic leukemia	EFO_0000095	Cancer	Neoplasms
1.0	0.00E+00	0.00E+00	PGS000647	EFO_0000095	chronic lymphocytic leukemia	chronic lymphocytic leukemia	EFO_0000095	Cancer	Neoplasms	PGS000646	EFO_0000095	chronic lymphocytic leukemia	chronic lymphocytic leukemia	EFO_0000095	Immune system disorder	Immunological
1.0	0.00E+00	0.00E+00	PGS000647	EFO_0000095	chronic lymphocytic leukemia	chronic lymphocytic leukemia	EFO_0000095	Immune system disorder	Immunological	PGS000646	EFO_0000095	chronic lymphocytic leukemia	chronic lymphocytic leukemia	EFO_0000095	Cancer	Neoplasms
0.9992	0.00E+00	0.00E+00	PGS000662	EFO_0001663	prostate carcinoma	prostate carcinoma	EFO_0001663	Cancer	Neoplasms	PGS003415	EFO_0001663	prostate carcinoma	prostate carcinoma	EFO_0001663	Cancer	Neoplasms
0.9899	0.00E+00	0.00E+00	PGS000347	EFO_1000649	estrogen-receptor positive breast cancer	estrogen-receptor positive breast cancer	EFO_1000649	Cancer	Neoplasms	PGS000344	EFO_0000305	breast carcinoma	breast carcinoma	EFO_0000305	Cancer	Neoplasms
0.9984	0.00E+00	0.00E+00	PGS002202	EFO_0009805	whole body water mass	whole body water mass	EFO_0009805	Other measurement	Body measurement	PGS002168	EFO_0004995	lean body mass	lean body mass	EFO_0004995	Other measurement	Skeletal
0.9928	0.00E+00	0.00E+00	PGS001253	EFO_0004238	hearing loss	hearing loss	EFO_0004238	Neurological disorder	Neurological	PGS001252	EFO_0004238	hearing loss|deafness	hearing loss	EFO_0004238	Neurological disorder	Neurological
1.0	0.00E+00	0.00E+00	PGS000647	EFO_0000095	chronic lymphocytic leukemia	chronic lymphocytic leukemia	EFO_0000095	Immune system disorder	Immunological	PGS000646	EFO_0000095	chronic lymphocytic leukemia	chronic lymphocytic leukemia	EFO_0000095	Immune system disorder	Immunological
0.9897	0.00E+00	0.00E+00	PGS001828	MONDO_0004975	Alzheimer disease	Alzheimer disease	MONDO_0004975	Neurological disorder	Neurological	PGS001827	MONDO_0001627	dementia	dementia	MONDO_0001627	Neurological disorder	Neurological
0.991	0.00E+00	0.00E+00	PGS000207	EFO_0002892	thyroid carcinoma	thyroid carcinoma	EFO_0002892	Cancer	Neoplasms	PGS000209	EFO_0002892	thyroid carcinoma	thyroid carcinoma	EFO_0002892	Cancer	Neoplasms
0.9876	0.00E+00	0.00E+00	PGS000069	EFO_1001516	ovarian serous carcinoma	ovarian serous carcinoma	EFO_1001516	Cancer	Neoplasms	PGS000068	EFO_0001075	ovarian carcinoma	ovarian carcinoma	EFO_0001075	Cancer	Neoplasms
1.0	0.00E+00	0.00E+00	PGS000517	EFO_0000305	breast carcinoma	breast carcinoma	EFO_0000305	Cancer	Neoplasms	PGS000518	EFO_0000305	breast carcinoma	breast carcinoma	EFO_0000305	Cancer	Neoplasms
0.9823	0.00E+00	0.00E+00	PGS001531	EFO_0007772	calcaneal bone quantitative ultrasound measurement	calcaneal bone quantitative ultrasound measurement	EFO_0007772	Other measurement	Skeletal	PGS001039	EFO_0009270	heel bone mineral density	heel bone mineral density	EFO_0009270	Other measurement	Skeletal
0.9918	0.00E+00	0.00E+00	PGS002968	EFO_0004339	body height	body height	EFO_0004339	Body measurement	Body measurement	PGS002973	EFO_0004339	body height	body height	EFO_0004339	Body measurement	Body measurement
0.9949	0.00E+00	0.00E+00	PGS000207	EFO_0002892	thyroid carcinoma	thyroid carcinoma	EFO_0002892	Cancer	Neoplasms	PGS000208	EFO_0002892	thyroid carcinoma	thyroid carcinoma	EFO_0002892	Cancer	Neoplasms
1.0	0.00E+00	0.00E+00	PGS000956	EFO_0004514	bone quantitative ultrasound measurement	bone quantitative ultrasound measurement	EFO_0004514	Other measurement	Skeletal	PGS001039	EFO_0009270	heel bone mineral density	heel bone mineral density	EFO_0009270	Other measurement	Skeletal
0.9938	0.00E+00	0.00E+00	PGS000454	EFO_0004193	basal cell carcinoma	basal cell carcinoma	EFO_0004193	Cancer	Neoplasms	PGS000453	EFO_0004193	basal cell carcinoma	basal cell carcinoma	EFO_0004193	Cancer	Neoplasms
0.9835	0.00E+00	0.00E+00	PGS002936	EFO_0004468	glucose measurement	glucose measurement	EFO_0004468	Other measurement	Metabolic	PGS002935	EFO_0004468	glucose measurement	glucose measurement	EFO_0004468	Other measurement	Metabolic
0.9921	0.00E+00	0.00E+00	PGS000458	EFO_0004193	basal cell carcinoma	basal cell carcinoma	EFO_0004193	Cancer	Neoplasms	PGS000459	EFO_0004193	basal cell carcinoma	basal cell carcinoma	EFO_0004193	Cancer	Neoplasms
1.0	0.00E+00	0.00E+00	PGS000534	EFO_0000305	breast carcinoma	breast carcinoma	EFO_0000305	Cancer	Neoplasms	PGS000533	EFO_0000305	breast carcinoma	breast carcinoma	EFO_0000305	Cancer	Neoplasms
1.0	0.00E+00	0.00E+00	PGS000425	EFO_0000756	melanoma	melanoma	EFO_0000756	Cancer	Neoplasms	PGS000428	EFO_0000756	melanoma	melanoma	EFO_0000756	Cancer	Neoplasms
0.9939	0.00E+00	0.00E+00	PGS001349	MONDO_0004975	Alzheimer disease	Alzheimer disease	MONDO_0004975	Neurological disorder	Neurological	PGS001348	MONDO_0004975	Alzheimer disease	Alzheimer disease	MONDO_0004975	Neurological disorder	Neurological
0.9964	0.00E+00	0.00E+00	PGS003096	MONDO_0005148	type 2 diabetes mellitus	type 2 diabetes mellitus	MONDO_0005148	Digestive system disorder	Gastrointestinal	PGS003095	MONDO_0005148	type 2 diabetes mellitus	type 2 diabetes mellitus	MONDO_0005148	Metabolic disorder	Metabolic
1.0	0.00E+00	0.00E+00	PGS000426	EFO_0000756	melanoma	melanoma	EFO_0000756	Cancer	Neoplasms	PGS000428	EFO_0000756	melanoma	melanoma	EFO_0000756	Cancer	Neoplasms
0.9964	0.00E+00	0.00E+00	PGS003096	MONDO_0005148	type 2 diabetes mellitus	type 2 diabetes mellitus	MONDO_0005148	Metabolic disorder	Metabolic	PGS003095	MONDO_0005148	type 2 diabetes mellitus	type 2 diabetes mellitus	MONDO_0005148	Digestive system disorder	Gastrointestinal
0.9906	0.00E+00	0.00E+00	PGS002732	EFO_0004735	serum alanine aminotransferase measurement	serum alanine aminotransferase measurement	EFO_0004735	Liver enzyme measurement	Gastrointestinal	PGS002282	EFO_0003095	non-alcoholic fatty liver disease	non-alcoholic fatty liver disease	EFO_0003095	Digestive system disorder	Gastrointestinal
0.9964	0.00E+00	0.00E+00	PGS003096	MONDO_0005148	type 2 diabetes mellitus	type 2 diabetes mellitus	MONDO_0005148	Metabolic disorder	Metabolic	PGS003095	MONDO_0005148	type 2 diabetes mellitus	type 2 diabetes mellitus	MONDO_0005148	Metabolic disorder	Metabolic
0.981	0.00E+00	0.00E+00	PGS002393	EFO_0000400	diabetes mellitus	diabetes mellitus	EFO_0000400	Metabolic disorder	Metabolic	PGS002573	MONDO_0005148	type 2 diabetes mellitus	type 2 diabetes mellitus	MONDO_0005148	Digestive system disorder	Gastrointestinal
0.9825	0.00E+00	0.00E+00	PGS002598	EFO_0007822	hair colour measurement	hair colour measurement	EFO_0007822	Other measurement	Dermatological	PGS002402	EFO_0007822	hair colour measurement	hair colour measurement	EFO_0007822	Other measurement	Dermatological
1.0	0.00E+00	0.00E+00	PGS000865	EFO_0010821	liver fat measurement	liver fat measurement	EFO_0010821	Other measurement	Gastrointestinal	PGS000872	EFO_0003095	non-alcoholic fatty liver disease	non-alcoholic fatty liver disease	EFO_0003095	Digestive system disorder	Gastrointestinal
0.9969	0.00E+00	0.00E+00	PGS001037	EFO_0009270	heel bone mineral density	heel bone mineral density	EFO_0009270	Other measurement	Skeletal	PGS001402	EFO_0009270	heel bone mineral density	heel bone mineral density	EFO_0009270	Other measurement	Skeletal
1.0	0.00E+00	0.00E+00	PGS000427	EFO_0000756	melanoma	melanoma	EFO_0000756	Cancer	Neoplasms	PGS000426	EFO_0000756	melanoma	melanoma	EFO_0000756	Cancer	Neoplasms
0.9986	0.00E+00	0.00E+00	PGS003306	EFO_0004617	cystatin C measurement|glomerular filtration rate	cystatin C measurement	EFO_0004617	Other measurement	Metabolic	PGS003305	EFO_0004617	cystatin C measurement|glomerular filtration rate	cystatin C measurement	EFO_0004617	Other measurement	Metabolic
1.0	0.00E+00	0.00E+00	PGS000525	EFO_0000305	breast carcinoma	breast carcinoma	EFO_0000305	Cancer	Neoplasms	PGS000526	EFO_0000305	breast carcinoma	breast carcinoma	EFO_0000305	Cancer	Neoplasms
0.9845	0.00E+00	0.00E+00	PGS003311	EFO_0004617	cystatin C measurement|glomerular filtration rate	cystatin C measurement	EFO_0004617	Other measurement	Metabolic	PGS003310	EFO_0004617	cystatin C measurement|glomerular filtration rate	cystatin C measurement	EFO_0004617	Other measurement	Metabolic
0.9999	0.00E+00	0.00E+00	PGS003325	EFO_0004698	insomnia	insomnia	EFO_0004698	Neurological disorder	Neurological	PGS003326	EFO_0004698	insomnia	insomnia	EFO_0004698	Neurological disorder	Neurological
0.9828	0.00E+00	0.00E+00	PGS001530	EFO_0007772	calcaneal bone quantitative ultrasound measurement	calcaneal bone quantitative ultrasound measurement	EFO_0007772	Other measurement	Skeletal	PGS001038	EFO_0009270	heel bone mineral density	heel bone mineral density	EFO_0009270	Other measurement	Skeletal
1.0	0.00E+00	0.00E+00	PGS000466	EFO_0000707	squamous cell carcinoma	squamous cell carcinoma	EFO_0000707	Cancer	Neoplasms	PGS000465	EFO_0000707	squamous cell carcinoma	squamous cell carcinoma	EFO_0000707	Cancer	Neoplasms
0.9832	0.00E+00	0.00E+00	PGS001265	EFO_0003907	deep vein thrombosis	deep vein thrombosis	EFO_0003907	Other trait	Cardiovascular	PGS001266	EFO_0003907	deep vein thrombosis	deep vein thrombosis	EFO_0003907	Other trait	Cardiovascular
1.0	0.00E+00	0.00E+00	PGS003121	MONDO_0005148	type 2 diabetes mellitus	type 2 diabetes mellitus	MONDO_0005148	Digestive system disorder	Gastrointestinal	PGS003120	MONDO_0005148	type 2 diabetes mellitus	type 2 diabetes mellitus	MONDO_0005148	Digestive system disorder	Gastrointestinal
1.0	0.00E+00	0.00E+00	PGS000426	EFO_0000756	melanoma	melanoma	EFO_0000756	Cancer	Neoplasms	PGS000425	EFO_0000756	melanoma	melanoma	EFO_0000756	Cancer	Neoplasms
0.9932	0.00E+00	0.00E+00	PGS000946	HP_0000726	Dementia	Dementia	HP_0000726	Other trait	Psychiatric	PGS001179	EFO_0004718	vascular dementia	vascular dementia	EFO_0004718	Neurological disorder	Neurological
1.0	0.00E+00	0.00E+00	PGS000621	MONDO_0001657	brain cancer	brain cancer	MONDO_0001657	Neurological disorder	Neurological	PGS000622	MONDO_0001657	brain cancer	brain cancer	MONDO_0001657	Cancer	Neoplasms
0.9932	0.00E+00	0.00E+00	PGS000946	HP_0000726	Dementia	Dementia	HP_0000726	Other trait	Psychiatric	PGS001179	EFO_0004718	vascular dementia	vascular dementia	EFO_0004718	Cardiovascular disease	Cardiovascular
0.9845	0.00E+00	0.00E+00	PGS003121	MONDO_0005148	type 2 diabetes mellitus	type 2 diabetes mellitus	MONDO_0005148	Metabolic disorder	Metabolic	PGS003106	MONDO_0005148	type 2 diabetes mellitus	type 2 diabetes mellitus	MONDO_0005148	Metabolic disorder	Metabolic
0.993	0.00E+00	0.00E+00	PGS003290	EFO_0004617	cystatin C measurement|glomerular filtration rate	cystatin C measurement	EFO_0004617	Other measurement	Metabolic	PGS003291	EFO_0004617	cystatin C measurement|glomerular filtration rate	cystatin C measurement	EFO_0004617	Other measurement	Metabolic
1.0	0.00E+00	0.00E+00	PGS000506	EFO_0000305	breast carcinoma	breast carcinoma	EFO_0000305	Cancer	Neoplasms	PGS000505	EFO_0000305	breast carcinoma	breast carcinoma	EFO_0000305	Cancer	Neoplasms
0.9922	0.00E+00	0.00E+00	PGS003226	EFO_0005208	glomerular filtration rate	glomerular filtration rate	EFO_0005208	Other measurement	Genitourinary	PGS003225	EFO_0005208	glomerular filtration rate	glomerular filtration rate	EFO_0005208	Other measurement	Genitourinary
0.9845	0.00E+00	0.00E+00	PGS003121	MONDO_0005148	type 2 diabetes mellitus	type 2 diabetes mellitus	MONDO_0005148	Metabolic disorder	Metabolic	PGS003106	MONDO_0005148	type 2 diabetes mellitus	type 2 diabetes mellitus	MONDO_0005148	Digestive system disorder	Gastrointestinal
0.9902	0.00E+00	0.00E+00	PGS002845	EFO_0004340	body mass index	body mass index	EFO_0004340	Body measurement	Body measurement	PGS002855	EFO_0004340	body mass index	body mass index	EFO_0004340	Body measurement	Body measurement
0.9844	0.00E+00	0.00E+00	PGS003142	EFO_0004574	total cholesterol measurement	total cholesterol measurement	EFO_0004574	Lipid or lipoprotein measurement	Metabolic	PGS003137	EFO_0004574	total cholesterol measurement	total cholesterol measurement	EFO_0004574	Lipid or lipoprotein measurement	Metabolic
1.0	0.00E+00	0.00E+00	PGS000605	MONDO_0002367	kidney cancer	kidney cancer	MONDO_0002367	Cancer	Neoplasms	PGS000606	MONDO_0002367	kidney cancer	kidney cancer	MONDO_0002367	Cancer	Neoplasms
0.9923	0.00E+00	0.00E+00	PGS002426	MONDO_0005148	type 2 diabetes mellitus	type 2 diabetes mellitus	MONDO_0005148	Metabolic disorder	Metabolic	PGS002573	MONDO_0005148	type 2 diabetes mellitus	type 2 diabetes mellitus	MONDO_0005148	Digestive system disorder	Gastrointestinal
0.9955	0.00E+00	0.00E+00	PGS002623	EFO_0004631	vitamin D measurement	vitamin D measurement	EFO_0004631	Lipid or lipoprotein measurement	Metabolic	PGS002574	EFO_0004631	vitamin D measurement	vitamin D measurement	EFO_0004631	Lipid or lipoprotein measurement	Metabolic
0.9886	0.00E+00	0.00E+00	PGS000353	MONDO_0004992	cancer	cancer	MONDO_0004992	Cancer	Neoplasms	PGS000355	MONDO_0004992	cancer	cancer	MONDO_0004992	Cancer	Neoplasms
0.9845	0.00E+00	0.00E+00	PGS003121	MONDO_0005148	type 2 diabetes mellitus	type 2 diabetes mellitus	MONDO_0005148	Digestive system disorder	Gastrointestinal	PGS003106	MONDO_0005148	type 2 diabetes mellitus	type 2 diabetes mellitus	MONDO_0005148	Metabolic disorder	Metabolic
1.0	0.00E+00	0.00E+00	PGS000455	EFO_0004193	basal cell carcinoma	basal cell carcinoma	EFO_0004193	Cancer	Neoplasms	PGS000452	EFO_0004193	basal cell carcinoma	basal cell carcinoma	EFO_0004193	Cancer	Neoplasms
0.9898	0.00E+00	0.00E+00	PGS001343	MONDO_0004979	asthma	asthma	MONDO_0004979	Other disease	Respiratory	PGS001341	MONDO_0004979	asthma	asthma	MONDO_0004979	Other disease	Respiratory
0.9972	0.00E+00	0.00E+00	PGS001950	EFO_0004995	lean body mass	lean body mass	EFO_0004995	Other measurement	Skeletal	PGS001984	EFO_0009805	whole body water mass	whole body water mass	EFO_0009805	Other measurement	Body measurement
0.9923	0.00E+00	0.00E+00	PGS002426	MONDO_0005148	type 2 diabetes mellitus	type 2 diabetes mellitus	MONDO_0005148	Digestive system disorder	Gastrointestinal	PGS002573	MONDO_0005148	type 2 diabetes mellitus	type 2 diabetes mellitus	MONDO_0005148	Metabolic disorder	Metabolic
0.986	0.00E+00	0.00E+00	PGS002543	EFO_0001379	endocrine system disease|diabetes mellitus	endocrine system disease	EFO_0001379	Other disease	Metabolic	PGS002592	EFO_0001379	endocrine system disease|diabetes mellitus	endocrine system disease	EFO_0001379	Other disease	Metabolic
0.9838	0.00E+00	0.00E+00	PGS003201	EFO_0004631	vitamin D measurement	vitamin D measurement	EFO_0004631	Lipid or lipoprotein measurement	Metabolic	PGS003200	EFO_0004631	vitamin D measurement	vitamin D measurement	EFO_0004631	Lipid or lipoprotein measurement	Metabolic
0.9866	0.00E+00	0.00E+00	PGS001824	Orphanet_309816	Disorder of bilirubin metabolism and excretion	Disorder of bilirubin metabolism and excretion	Orphanet_309816	Metabolic disorder	Metabolic	PGS002032	Orphanet_309816	Disorder of bilirubin metabolism and excretion	excretion	Orphanet_309816	Metabolic disorder	Metabolic
0.9951	0.00E+00	0.00E+00	PGS000804	MONDO_0005148	type 2 diabetes mellitus	type 2 diabetes mellitus	MONDO_0005148	Digestive system disorder	Gastrointestinal	PGS000805	MONDO_0005148	type 2 diabetes mellitus	type 2 diabetes mellitus	MONDO_0005148	Digestive system disorder	Gastrointestinal
0.9951	0.00E+00	0.00E+00	PGS000804	MONDO_0005148	type 2 diabetes mellitus	type 2 diabetes mellitus	MONDO_0005148	Digestive system disorder	Gastrointestinal	PGS000805	MONDO_0005148	type 2 diabetes mellitus	type 2 diabetes mellitus	MONDO_0005148	Metabolic disorder	Metabolic
0.9951	0.00E+00	0.00E+00	PGS000804	MONDO_0005148	type 2 diabetes mellitus	type 2 diabetes mellitus	MONDO_0005148	Metabolic disorder	Metabolic	PGS000805	MONDO_0005148	type 2 diabetes mellitus	type 2 diabetes mellitus	MONDO_0005148	Digestive system disorder	Gastrointestinal
0.9912	0.00E+00	0.00E+00	PGS000207	EFO_0002892	thyroid carcinoma	thyroid carcinoma	EFO_0002892	Cancer	Neoplasms	PGS000630	EFO_0002892	thyroid carcinoma	thyroid carcinoma	EFO_0002892	Cancer	Neoplasms
0.9951	0.00E+00	0.00E+00	PGS000804	MONDO_0005148	type 2 diabetes mellitus	type 2 diabetes mellitus	MONDO_0005148	Metabolic disorder	Metabolic	PGS000805	MONDO_0005148	type 2 diabetes mellitus	type 2 diabetes mellitus	MONDO_0005148	Metabolic disorder	Metabolic
1.0	0.00E+00	0.00E+00	PGS003171	EFO_0004620	vitamin B12 measurement	vitamin B12 measurement	EFO_0004620	Other measurement	Metabolic	PGS003170	EFO_0004620	vitamin B12 measurement	vitamin B12 measurement	EFO_0004620	Other measurement	Metabolic

0.9999	0.00E+00	0.00E+00	PGS003211	EFO_0003877	sleep apnea	sleep apnea	EFO_0003877	Neurological disorder	Neurological	PGS003210	EFO_0003877	sleep apnea	sleep apnea	EFO_0003877	Neurological disorder	Neurological
0.9923	0.00E+00	0.00E+00	PGS002426	MONDO_0005148	type 2 diabetes mellitus	type 2 diabetes mellitus	MONDO_0005148	Digestive system disorder	Gastrointestinal	PGS002573	MONDO_0005148	type 2 diabetes mellitus	type 2 diabetes mellitus	MONDO_0005148	Digestive system disorder	Gastrointestinal
0.9985	0.00E+00	0.00E+00	PGS003300	EFO_0004617	cystatin C measurement|glomerular filtration rate	cystatin C measurement	EFO_0004617	Other measurement	Metabolic	PGS003301	EFO_0004617	cystatin C measurement|glomerular filtration rate	cystatin C measurement	EFO_0004617	Other measurement	Metabolic
0.9997	0.00E+00	0.00E+00	PGS000631	EFO_0002892	thyroid carcinoma	thyroid carcinoma	EFO_0002892	Cancer	Neoplasms	PGS000209	EFO_0002892	thyroid carcinoma	thyroid carcinoma	EFO_0002892	Cancer	Neoplasms
1.0	0.00E+00	0.00E+00	PGS000623	MONDO_0001657	brain cancer	brain cancer	MONDO_0001657	Neurological disorder	Neurological	PGS000622	MONDO_0001657	brain cancer	brain cancer	MONDO_0001657	Cancer	Neoplasms
0.9833	0.00E+00	0.00E+00	PGS001349	MONDO_0004975	Alzheimer disease	Alzheimer disease	MONDO_0004975	Neurological disorder	Neurological	PGS001828	MONDO_0004975	Alzheimer disease	Alzheimer disease	MONDO_0004975	Neurological disorder	Neurological
0.9863	0.00E+00	0.00E+00	PGS003201	EFO_0004631	vitamin D measurement	vitamin D measurement	EFO_0004631	Lipid or lipoprotein measurement	Metabolic	PGS003186	EFO_0004631	vitamin D measurement	vitamin D measurement	EFO_0004631	Lipid or lipoprotein measurement	Metabolic
0.9876	0.00E+00	0.00E+00	PGS002569	EFO_0004279	suntan	suntan	EFO_0004279	Other trait	Dermatological	PGS002422	EFO_0004279	suntan	suntan	EFO_0004279	Other trait	Dermatological
1.0	0.00E+00	0.00E+00	PGS000623	MONDO_0001657	brain cancer	brain cancer	MONDO_0001657	Cancer	Neoplasms	PGS000622	MONDO_0001657	brain cancer	brain cancer	MONDO_0001657	Neurological disorder	Neurological
0.9833	0.00E+00	0.00E+00	PGS000604	EFO_0005088	testicular carcinoma	testicular carcinoma	EFO_0005088	Cancer	Neoplasms	PGS000601	EFO_0005088	testicular carcinoma	testicular carcinoma	EFO_0005088	Cancer	Neoplasms
1.0	0.00E+00	0.00E+00	PGS000621	MONDO_0001657	brain cancer	brain cancer	MONDO_0001657	Neurological disorder	Neurological	PGS000622	MONDO_0001657	brain cancer	brain cancer	MONDO_0001657	Neurological disorder	Neurological
0.9884	0.00E+00	0.00E+00	PGS000389	MONDO_0001672	bronchus cancer|lung cancer|tracheal cancer	bronchus cancer	MONDO_0001672	Cancer	Neoplasms	PGS000392	MONDO_0001672	bronchus cancer|lung cancer|tracheal cancer	bronchus cancer	MONDO_0001672	Cancer	Neoplasms
0.9999	0.00E+00	0.00E+00	PGS003001	EFO_0000537	hypertension	hypertension	EFO_0000537	Cardiovascular disease	Cardiovascular	PGS003000	EFO_0000537	hypertension	hypertension	EFO_0000537	Cardiovascular disease	Cardiovascular
1.0	0.00E+00	0.00E+00	PGS000623	MONDO_0001657	brain cancer	brain cancer	MONDO_0001657	Cancer	Neoplasms	PGS000622	MONDO_0001657	brain cancer	brain cancer	MONDO_0001657	Cancer	Neoplasms
0.9923	0.00E+00	0.00E+00	PGS002426	MONDO_0005148	type 2 diabetes mellitus	type 2 diabetes mellitus	MONDO_0005148	Metabolic disorder	Metabolic	PGS002573	MONDO_0005148	type 2 diabetes mellitus	type 2 diabetes mellitus	MONDO_0005148	Metabolic disorder	Metabolic
0.9951	0.00E+00	0.00E+00	PGS003119	MONDO_0005148	type 2 diabetes mellitus	type 2 diabetes mellitus	MONDO_0005148	Metabolic disorder	Metabolic	PGS003104	MONDO_0005148	type 2 diabetes mellitus	type 2 diabetes mellitus	MONDO_0005148	Metabolic disorder	Metabolic
0.9856	0.00E+00	0.00E+00	PGS000073	EFO_0001061	cervical carcinoma	cervical carcinoma	EFO_0001061	Cancer	Neoplasms	PGS000784	EFO_0001061	cervical carcinoma	cervical carcinoma	EFO_0001061	Cancer	Neoplasms
0.9884	0.00E+00	0.00E+00	PGS000390	MONDO_0001672	bronchus cancer|lung cancer|tracheal cancer	bronchus cancer	MONDO_0001672	Cancer	Neoplasms	PGS000392	MONDO_0001672	bronchus cancer|lung cancer|tracheal cancer	bronchus cancer	MONDO_0001672	Cancer	Neoplasms
0.9924	0.00E+00	0.00E+00	PGS002621	EFO_0004530	triglyceride measurement	triglyceride measurement	EFO_0004530	Lipid or lipoprotein measurement	Metabolic	PGS002572	EFO_0004530	triglyceride measurement	triglyceride measurement	EFO_0004530	Lipid or lipoprotein measurement	Metabolic
0.9864	0.00E+00	0.00E+00	PGS001349	MONDO_0004975	Alzheimer disease	Alzheimer disease	MONDO_0004975	Neurological disorder	Neurological	PGS001179	EFO_0004718	vascular dementia	vascular dementia	EFO_0004718	Cardiovascular disease	Cardiovascular
0.9859	0.00E+00	0.00E+00	PGS001037	EFO_0009270	heel bone mineral density	heel bone mineral density	EFO_0009270	Other measurement	Skeletal	PGS001529	EFO_0007772	measurement	measurement	EFO_0007772	Other measurement	Skeletal
0.9869	0.00E+00	0.00E+00	PGS003185	EFO_0004631	vitamin D measurement	vitamin D measurement	EFO_0004631	Lipid or lipoprotein measurement	Metabolic	PGS003200	EFO_0004631	vitamin D measurement	vitamin D measurement	EFO_0004631	Lipid or lipoprotein measurement	Metabolic
0.9972	0.00E+00	0.00E+00	PGS001280	EFO_0003827	pulmonary embolism	pulmonary embolism	EFO_0003827	Cardiovascular disease	Cardiovascular	PGS001277	EFO_0003827	pulmonary embolism|deep vein thrombosis	pulmonary embolism	EFO_0003827	Cardiovascular disease	Cardiovascular
0.9956	0.00E+00	0.00E+00	PGS001146	EFO_0005409	fat body mass	fat body mass	EFO_0005409	Other measurement	Body measurement	PGS001235	EFO_0004324	body weights and measures	body weights and measures	EFO_0004324	Body measurement	Body measurement
1.0	0.00E+00	0.00E+00	PGS002821	EFO_0003922	menopause	menopause	EFO_0003922	Other trait	Genitourinary	PGS002822	EFO_0003922	menopause	menopause	EFO_0003922	Other trait	Genitourinary
0.9945	0.00E+00	0.00E+00	PGS003400	EFO_0001073	obesity	obesity	EFO_0001073	Metabolic disorder	Metabolic	PGS000034	EFO_0004340	body mass index	body mass index	EFO_0004340	Body measurement	Body measurement
0.9827	0.00E+00	0.00E+00	PGS000773	EFO_0000305	breast carcinoma	breast carcinoma	EFO_0000305	Cancer	Neoplasms	PGS000052	EFO_0000305	breast carcinoma	breast carcinoma	EFO_0000305	Cancer	Neoplasms
0.9886	0.00E+00	0.00E+00	PGS003094	MONDO_0005148	type 2 diabetes mellitus	type 2 diabetes mellitus	MONDO_0005148	Digestive system disorder	Gastrointestinal	PGS003109	MONDO_0005148	type 2 diabetes mellitus	type 2 diabetes mellitus	MONDO_0005148	Digestive system disorder	Gastrointestinal
0.9864	0.00E+00	0.00E+00	PGS000324	EFO_0009732	enthesitis-related juvenile idiopathic arthritis	enthesitis-related juvenile idiopathic arthritis	EFO_0009732	Immune system disorder	Immunological	PGS002089	EFO_0003898	ankylosing spondylitis	ankylosing spondylitis	EFO_0003898	Immune system disorder	Immunological
0.9886	0.00E+00	0.00E+00	PGS003094	MONDO_0005148	type 2 diabetes mellitus	type 2 diabetes mellitus	MONDO_0005148	Digestive system disorder	Gastrointestinal	PGS003109	MONDO_0005148	type 2 diabetes mellitus	type 2 diabetes mellitus	MONDO_0005148	Metabolic disorder	Metabolic
0.9886	0.00E+00	0.00E+00	PGS003094	MONDO_0005148	type 2 diabetes mellitus	type 2 diabetes mellitus	MONDO_0005148	Metabolic disorder	Metabolic	PGS003109	MONDO_0005148	type 2 diabetes mellitus	type 2 diabetes mellitus	MONDO_0005148	Digestive system disorder	Gastrointestinal
0.9886	0.00E+00	0.00E+00	PGS003094	MONDO_0005148	type 2 diabetes mellitus	type 2 diabetes mellitus	MONDO_0005148	Metabolic disorder	Metabolic	PGS003109	MONDO_0005148	type 2 diabetes mellitus	type 2 diabetes mellitus	MONDO_0005148	Metabolic disorder	Metabolic
1.0	0.00E+00	0.00E+00	PGS000611	MONDO_0004986	urinary bladder carcinoma	urinary bladder carcinoma	MONDO_0004986	Cancer	Neoplasms	PGS000612	MONDO_0004986	urinary bladder carcinoma	urinary bladder carcinoma	MONDO_0004986	Cancer	Neoplasms
1.0	0.00E+00	0.00E+00	PGS000489	EFO_0000305	breast carcinoma	breast carcinoma	EFO_0000305	Cancer	Neoplasms	PGS000494	EFO_0000305	breast carcinoma	breast carcinoma	EFO_0000305	Cancer	Neoplasms
1.0	0.00E+00	0.00E+00	PGS003261	EFO_0010472	creatine measurement|glomerular filtration rate	creatine measurement	EFO_0010472	Other measurement	Metabolic	PGS003260	EFO_0010472	creatine measurement|glomerular filtration rate	creatine measurement	EFO_0010472	Other measurement	Metabolic
0.9837	0.00E+00	0.00E+00	PGS001402	EFO_0009270	heel bone mineral density	heel bone mineral density	EFO_0009270	Other measurement	Skeletal	PGS001529	EFO_0007772	measurement	measurement	EFO_0007772	Other measurement	Skeletal
0.9864	0.00E+00	0.00E+00	PGS001349	MONDO_0004975	Alzheimer disease	Alzheimer disease	MONDO_0004975	Neurological disorder	Neurological	PGS001179	EFO_0004718	vascular dementia	vascular dementia	EFO_0004718	Neurological disorder	Neurological
0.9946	0.00E+00	0.00E+00	PGS000654	EFO_0001378	multiple myeloma	multiple myeloma	EFO_0001378	Immune system disorder	Immunological	PGS000653	EFO_0001378	multiple myeloma	multiple myeloma	EFO_0001378	Immune system disorder	Immunological
0.9825	0.00E+00	0.00E+00	PGS002559	EFO_0004584	mean platelet volume	mean platelet volume	EFO_0004584	Hematological measurement	Hematological	PGS002412	EFO_0004584	mean platelet volume	mean platelet volume	EFO_0004584	Hematological measurement	Hematological
0.9951	0.00E+00	0.00E+00	PGS003119	MONDO_0005148	type 2 diabetes mellitus	type 2 diabetes mellitus	MONDO_0005148	Digestive system disorder	Gastrointestinal	PGS003104	MONDO_0005148	type 2 diabetes mellitus	type 2 diabetes mellitus	MONDO_0005148	Digestive system disorder	Gastrointestinal
1.0	0.00E+00	0.00E+00	PGS000623	MONDO_0001657	brain cancer	brain cancer	MONDO_0001657	Cancer	Neoplasms	PGS000621	MONDO_0001657	brain cancer	brain cancer	MONDO_0001657	Cancer	Neoplasms
1.0	0.00E+00	0.00E+00	PGS000623	MONDO_0001657	brain cancer	brain cancer	MONDO_0001657	Cancer	Neoplasms	PGS000621	MONDO_0001657	brain cancer	brain cancer	MONDO_0001657	Neurological disorder	Neurological
0.9979	0.00E+00	0.00E+00	PGS001775	MONDO_0004975	Alzheimer disease	Alzheimer disease	MONDO_0004975	Neurological disorder	Neurological	PGS000898	MONDO_0004975	Alzheimer disease	Alzheimer disease	MONDO_0004975	Neurological disorder	Neurological
0.9845	0.00E+00	0.00E+00	PGS003121	MONDO_0005148	type 2 diabetes mellitus	type 2 diabetes mellitus	MONDO_0005148	Digestive system disorder	Gastrointestinal	PGS003106	MONDO_0005148	type 2 diabetes mellitus	type 2 diabetes mellitus	MONDO_0005148	Digestive system disorder	Gastrointestinal
0.9882	0.00E+00	0.00E+00	PGS002954	EFO_0004612	high density lipoprotein cholesterol measurement	high density lipoprotein cholesterol measurement	EFO_0004612	Lipid or lipoprotein measurement	Metabolic	PGS002959	EFO_0004612	high density lipoprotein cholesterol measurement	measurement	EFO_0004612	Lipid or lipoprotein measurement	Metabolic
0.9852	0.00E+00	0.00E+00	PGS001316	EFO_0000612	myocardial infarction	myocardial infarction	EFO_0000612	Cardiovascular disease	Cardiovascular	PGS001315	EFO_0000612	myocardial infarction	myocardial infarction	EFO_0000612	Cardiovascular disease	Cardiovascular
0.9845	0.00E+00	0.00E+00	PGS002547	EFO_0004468	glucose measurement	glucose measurement	EFO_0004468	Other measurement	Metabolic	PGS002596	EFO_0004468	glucose measurement	glucose measurement	EFO_0004468	Other measurement	Metabolic
0.988	0.00E+00	0.00E+00	PGS002533	EFO_0007825	balding measurement	balding measurement	EFO_0007825	Other measurement	Activities	PGS002582	EFO_0007825	balding measurement	balding measurement	EFO_0007825	Other measurement	Activities
1.0	0.00E+00	0.00E+00	PGS000621	MONDO_0001657	brain cancer	brain cancer	MONDO_0001657	Cancer	Neoplasms	PGS000622	MONDO_0001657	brain cancer	brain cancer	MONDO_0001657	Neurological disorder	Neurological
1.0	0.00E+00	0.00E+00	PGS000623	MONDO_0001657	brain cancer	brain cancer	MONDO_0001657	Neurological disorder	Neurological	PGS000621	MONDO_0001657	brain cancer	brain cancer	MONDO_0001657	Cancer	Neoplasms
0.9874	0.00E+00	0.00E+00	PGS002549	EFO_0007822	hair colour measurement	hair colour measurement	EFO_0007822	Other measurement	Dermatological	PGS002402	EFO_0007822	hair colour measurement	hair colour measurement	EFO_0007822	Other measurement	Dermatological
1.0	0.00E+00	0.00E+00	PGS000623	MONDO_0001657	brain cancer	brain cancer	MONDO_0001657	Neurological disorder	Neurological	PGS000621	MONDO_0001657	brain cancer	brain cancer	MONDO_0001657	Neurological disorder	Neurological
0.9951	0.00E+00	0.00E+00	PGS003119	MONDO_0005148	type 2 diabetes mellitus	type 2 diabetes mellitus	MONDO_0005148	Metabolic disorder	Metabolic	PGS003104	MONDO_0005148	type 2 diabetes mellitus	type 2 diabetes mellitus	MONDO_0005148	Digestive system disorder	Gastrointestinal
0.9999	0.00E+00	0.00E+00	PGS003015	EFO_0000537	hypertension	hypertension	EFO_0000537	Cardiovascular disease	Cardiovascular	PGS003016	EFO_0000537	hypertension	hypertension	EFO_0000537	Cardiovascular disease	Cardiovascular
1.0	0.00E+00	0.00E+00	PGS000621	MONDO_0001657	brain cancer	brain cancer	MONDO_0001657	Cancer	Neoplasms	PGS000622	MONDO_0001657	brain cancer	brain cancer	MONDO_0001657	Cancer	Neoplasms
0.9991	0.00E+00	0.00E+00	PGS001267	EFO_0003898	ankylosing spondylitis	ankylosing spondylitis	EFO_0003898	Immune system disorder	Immunological	PGS001268	EFO_0003898	ankylosing spondylitis	ankylosing spondylitis	EFO_0003898	Immune system disorder	Immunological
0.9951	0.00E+00	0.00E+00	PGS003119	MONDO_0005148	type 2 diabetes mellitus	type 2 diabetes mellitus	MONDO_0005148	Digestive system disorder	Gastrointestinal	PGS003104	MONDO_0005148	type 2 diabetes mellitus	type 2 diabetes mellitus	MONDO_0005148	Metabolic disorder	Metabolic
1.0	0.00E+00	0.00E+00	PGS000602	EFO_0005088	testicular carcinoma	testicular carcinoma	EFO_0005088	Cancer	Neoplasms	PGS000601	EFO_0005088	testicular carcinoma	testicular carcinoma	EFO_0005088	Cancer	Neoplasms
0.9815	0.00E+00	0.00E+00	PGS001827	MONDO_0001627	dementia	dementia	MONDO_0001627	Neurological disorder	Neurological	PGS001348	MONDO_0004975	Alzheimer disease	Alzheimer disease	MONDO_0004975	Neurological disorder	Neurological
1.0	0.00E+00	0.00E+00	PGS002886	EFO_0004458	C-reactive protein measurement	C-reactive protein measurement	EFO_0004458	Inflammatory measurement	Immunological	PGS002885	EFO_0004458	C-reactive protein measurement	C-reactive protein measurement	EFO_0004458	Inflammatory measurement	Immunological
0.9946	0.00E+00	0.00E+00	PGS000654	EFO_0001378	multiple myeloma	multiple myeloma	EFO_0001378	Immune system disorder	Immunological	PGS000653	EFO_0001378	multiple myeloma	multiple myeloma	EFO_0001378	Cancer	Neoplasms
1.0	0.00E+00	0.00E+00	PGS000623	MONDO_0001657	brain cancer	brain cancer	MONDO_0001657	Neurological disorder	Neurological	PGS000622	MONDO_0001657	brain cancer	brain cancer	MONDO_0001657	Neurological disorder	Neurological
1.0	0.00E+00	0.00E+00	PGS002951	EFO_0004465	fasting blood glucose measurement	fasting blood glucose measurement	EFO_0004465	Other measurement	Metabolic	PGS002950	EFO_0004465	fasting blood glucose measurement	fasting blood glucose measurement	EFO_0004465	Other measurement	Metabolic
1.0	0.00E+00	0.00E+00	PGS001045	EFO_0009116	vitamin supplement exposure measurement	vitamin supplement exposure measurement	EFO_0009116	Other measurement	Activities	PGS001153	EFO_0005111	folic acid measurement	folic acid measurement	EFO_0005111	Other measurement	Metabolic
0.9958	0.00E+00	0.00E+00	PGS000956	EFO_0004514	bone quantitative ultrasound measurement	bone quantitative ultrasound measurement	EFO_0004514	Other measurement	Skeletal	PGS001404	EFO_0009270	heel bone mineral density	heel bone mineral density	EFO_0009270	Other measurement	Skeletal
0.9859	0.00E+00	0.00E+00	PGS000952	EFO_0004514	bone quantitative ultrasound measurement	bone quantitative ultrasound measurement	EFO_0004514	Other measurement	Skeletal	PGS001529	EFO_0007772	measurement	measurement	EFO_0007772	Other measurement	Skeletal
0.9887	0.00E+00	0.00E+00	PGS002611	EFO_0004309	platelet count	platelet count	EFO_0004309	Hematological measurement	Hematological	PGS002562	EFO_0004309	platelet count	platelet count	EFO_0004309	Hematological measurement	Hematological
0.9835	0.00E+00	0.00E+00	PGS000424	EFO_0000756	melanoma	melanoma	EFO_0000756	Cancer	Neoplasms	PGS000423	EFO_0000756	melanoma	melanoma	EFO_0000756	Cancer	Neoplasms
0.9912	0.00E+00	0.00E+00	PGS000207	EFO_0002892	thyroid carcinoma	thyroid carcinoma	EFO_0002892	Cancer	Neoplasms	PGS000631	EFO_0002892	thyroid carcinoma	thyroid carcinoma	EFO_0002892	Cancer	Neoplasms
0.9846	0.00E+00	0.00E+00	PGS003143	EFO_0004574	total cholesterol measurement	total cholesterol measurement	EFO_0004574	Lipid or lipoprotein measurement	Metabolic	PGS003138	EFO_0004574	total cholesterol measurement	total cholesterol measurement	EFO_0004574	Lipid or lipoprotein measurement	Metabolic
0.9808	0.00E+00	0.00E+00	PGS003198	EFO_0004631	vitamin D measurement	vitamin D measurement	EFO_0004631	Lipid or lipoprotein measurement	Metabolic	PGS003193	EFO_0004631	vitamin D measurement	vitamin D measurement	EFO_0004631	Lipid or lipoprotein measurement	Metabolic
0.996	0.00E+00	0.00E+00	PGS002940	EFO_0004468	glucose measurement	glucose measurement	EFO_0004468	Other measurement	Metabolic	PGS002941	EFO_0004468	glucose measurement	glucose measurement	EFO_0004468	Other measurement	Metabolic
1.0	0.00E+00	0.00E+00	PGS003040	EFO_0010733	polyunsaturated fatty acid measurement	polyunsaturated fatty acid measurement	EFO_0010733	Lipid or lipoprotein measurement	Metabolic	PGS003041	EFO_0010733	polyunsaturated fatty acid measurement	polyunsaturated fatty acid measurement	EFO_0010733	Lipid or lipoprotein measurement	Metabolic
1.0	0.00E+00	0.00E+00	PGS000464	EFO_0000707	squamous cell carcinoma	squamous cell carcinoma	EFO_0000707	Cancer	Neoplasms	PGS000462	EFO_0000707	squamous cell carcinoma	squamous cell carcinoma	EFO_0000707	Cancer	Neoplasms
0.9938	0.00E+00	0.00E+00	PGS000452	EFO_0004193	basal cell carcinoma	basal cell carcinoma	EFO_0004193	Cancer	Neoplasms	PGS000454	EFO_0004193	basal cell carcinoma	basal cell carcinoma	EFO_0004193	Cancer	Neoplasms
0.984	0.00E+00	0.00E+00	PGS002041	EFO_0001365	age-related macular degeneration	age-related macular degeneration	EFO_0001365	Neurological disorder	Neurological	PGS001834	EFO_0001365	age-related macular degeneration	age-related macular degeneration	EFO_0001365	Neurological disorder	Neurological
1.0	0.00E+00	0.00E+00	PGS000582	EFO_0001663	prostate carcinoma	prostate carcinoma	EFO_0001663	Cancer	Neoplasms	PGS000581	EFO_0001663	prostate carcinoma	prostate carcinoma	EFO_0001663	Cancer	Neoplasms
1.0	0.00E+00	0.00E+00	PGS000194	EFO_0009459	ACPA-positive rheumatoid arthritis	ACPA-positive rheumatoid arthritis	EFO_0009459	Immune system disorder	Immunological	PGS000195	EFO_0009460	ACPA-negative rheumatoid arthritis	ACPA-negative rheumatoid arthritis	EFO_0009460	Immune system disorder	Immunological
0.9927	0.00E+00	0.00E+00	PGS001403	EFO_0009270	heel bone mineral density	heel bone mineral density	EFO_0009270	Other measurement	Skeletal	PGS000954	EFO_0004514	bone quantitative ultrasound measurement	measurement	EFO_0004514	Other measurement	Skeletal
1.0	0.00E+00	0.00E+00	PGS002926	EFO_0004465	fasting blood glucose measurement	fasting blood glucose measurement	EFO_0004465	Other measurement	Metabolic	PGS002925	EFO_0004465	fasting blood glucose measurement	fasting blood glucose measurement	EFO_0004465	Other measurement	Metabolic
1.0	0.00E+00	0.00E+00	PGS003101	MONDO_0005148	type 2 diabetes mellitus	type 2 diabetes mellitus	MONDO_0005148	Metabolic disorder	Metabolic	PGS003100	MONDO_0005148	type 2 diabetes mellitus	type 2 diabetes mellitus	MONDO_0005148	Metabolic disorder	Metabolic
0.9859	0.00E+00	0.00E+00	PGS002534	EFO_0003923	bone density	bone density	EFO_0003923	Other measurement	Skeletal	PGS002583	EFO_0003923	bone density	bone density	EFO_0003923	Other measurement	Skeletal
1.0	0.00E+00	0.00E+00	PGS003101	MONDO_0005148	type 2 diabetes mellitus	type 2 diabetes mellitus	MONDO_0005148	Metabolic disorder	Metabolic	PGS003100	MONDO_0005148	type 2 diabetes mellitus	type 2 diabetes mellitus	MONDO_0005148	Digestive system disorder	Gastrointestinal
1.0	0.00E+00	0.00E+00	PGS003101	MONDO_0005148	type 2 diabetes mellitus	type 2 diabetes mellitus	MONDO_0005148	Digestive system disorder	Gastrointestinal	PGS003100	MONDO_0005148	type 2 diabetes mellitus	type 2 diabetes mellitus	MONDO_0005148	Digestive system disorder	Gastrointestinal
1.0	0.00E+00	0.00E+00	PGS002952	EFO_0004465	fasting blood glucose measurement	fasting blood glucose measurement	EFO_0004465	Other measurement	Metabolic	PGS002947	EFO_0004465	fasting blood glucose measurement	fasting blood glucose measurement	EFO_0004465	Other measurement	Metabolic
1.0	0.00E+00	0.00E+00	PGS003105	MONDO_0005148	type 2 diabetes mellitus	type 2 diabetes mellitus	MONDO_0005148	Digestive system disorder	Gastrointestinal	PGS003106	MONDO_0005148	type 2 diabetes mellitus	type 2 diabetes mellitus	MONDO_0005148	Metabolic disorder	Metabolic
1.0	0.00E+00	0.00E+00	PGS003105	MONDO_0005148	type 2 diabetes mellitus	type 2 diabetes mellitus	MONDO_0005148	Digestive system disorder	Gastrointestinal	PGS003106	MONDO_0005148	type 2 diabetes mellitus	type 2 diabetes mellitus	MONDO_0005148	Digestive system disorder	Gastrointestinal
0.9907	0.00E+00	0.00E+00	PGS002976	EFO_0004339	body height	body height	EFO_0004339	Body measurement	Body measurement	PGS002975	EFO_0004339	body height	body height	EFO_0004339	Body measurement	Body measurement
-0.9822	0.00E+00	0.00E+00	PGS001388	EFO_0005671	smoking behaviour measurement	smoking behaviour measurement	EFO_0005671	Other measurement	Psychiatric	PGS001139	EFO_0005671	smoking behaviour measurement	smoking behaviour measurement	EFO_0005671	Other measurement	Psychiatric
0.9861	0.00E+00	0.00E+00	PGS003038	EFO_0004611	low density lipoprotein cholesterol measurement	low density lipoprotein cholesterol measurement	EFO_0004611	Lipid or lipoprotein measurement	Metabolic	PGS003033	EFO_0004611	low density lipoprotein cholesterol measurement	measurement	EFO_0004611	Lipid or lipoprotein measurement	Metabolic
0.9991	0.00E+00	0.00E+00	PGS000578	EFO_0001663	prostate carcinoma	prostate carcinoma	EFO_0001663	Cancer	Neoplasms	PGS000580	EFO_0001663	prostate carcinoma	prostate carcinoma	EFO_0001663	Cancer	Neoplasms
0.9841	0.00E+00	0.00E+00	PGS000001	EFO_0000305	breast carcinoma	breast carcinoma	EFO_0000305	Cancer	Neoplasms	PGS000002	EFO_1000649	estrogen-receptor positive breast cancer	cancer	EFO_1000649	Cancer	Neoplasms
0.9861	0.00E+00	0.00E+00	PGS003038	EFO_0004611	low density lipoprotein cholesterol measurement	low density lipoprotein cholesterol measurement	EFO_0004611	Lipid or lipoprotein measurement	Metabolic	PGS003033	EFO_0004611	low density lipoprotein cholesterol measurement	measurement	EFO_0004611	Cardiovascular measurement	Cardiovascular
1.0	0.00E+00	0.00E+00	PGS000490	EFO_0000305	breast carcinoma	breast carcinoma	EFO_0000305	Cancer	Neoplasms	PGS000494	EFO_0000305	breast carcinoma	breast carcinoma	EFO_0000305	Cancer	Neoplasms
1.0	0.00E+00	0.00E+00	PGS003199	EFO_0004631	vitamin D measurement	vitamin D measurement	EFO_0004631	Lipid or lipoprotein measurement	Metabolic	PGS003184	EFO_0004631	vitamin D measurement	vitamin D measurement	EFO_0004631	Lipid or lipoprotein measurement	Metabolic
0.9861	0.00E+00	0.00E+00	PGS003038	EFO_0004611	low density lipoprotein cholesterol measurement	low density lipoprotein cholesterol measurement	EFO_0004611	Cardiovascular measurement	Cardiovascular	PGS003033	EFO_0004611	low density lipoprotein cholesterol measurement	measurement	EFO_0004611	Lipid or lipoprotein measurement	Metabolic
0.9861	0.00E+00	0.00E+00	PGS003038	EFO_0004611	low density lipoprotein cholesterol measurement	low density lipoprotein cholesterol measurement	EFO_0004611	Cardiovascular measurement	Cardiovascular	PGS003033	EFO_0004611	low density lipoprotein cholesterol measurement	measurement	EFO_0004611	Cardiovascular measurement	Cardiovascular
0.9865	0.00E+00	0.00E+00	PGS002557	EFO_0004587	lymphocyte count	lymphocyte count	EFO_0004587	Hematological measurement	Hematological	PGS002606	EFO_0004587	lymphocyte count	lymphocyte count	EFO_0004587	Hematological measurement	Hematological
0.981	0.00E+00	0.00E+00	PGS002393	EFO_0000400	diabetes mellitus	diabetes mellitus	EFO_0000400	Metabolic disorder	Metabolic	PGS002573	MONDO_0005148	type 2 diabetes mellitus	type 2 diabetes mellitus	MONDO_0005148	Metabolic disorder	Metabolic
0.9991	0.00E+00	0.00E+00	PGS000577	EFO_0001663	prostate carcinoma	prostate carcinoma	EFO_0001663	Cancer	Neoplasms	PGS000580	EFO_0001663	prostate carcinoma	prostate carcinoma	EFO_0001663	Cancer	Neoplasms
0.9921	0.00E+00	0.00E+00	PGS002981	EFO_0004339	body height	body height	EFO_0004339	Body measurement	Body measurement	PGS002980	EFO_0004339	body height	body height	EFO_0004339	Body measurement	Body measurement
1.0	0.00E+00	0.00E+00	PGS003101	MONDO_0005148	type 2 diabetes mellitus	type 2 diabetes mellitus	MONDO_0005148	Digestive system disorder	Gastrointestinal	PGS003100	MONDO_0005148	type 2 diabetes mellitus	type 2 diabetes mellitus	MONDO_0005148	Metabolic disorder	Metabolic
0.9915	0.00E+00	0.00E+00	PGS002894	EFO_0006336	diastolic blood pressure	diastolic blood pressure	EFO_0006336	Other measurement	Cardiovascular	PGS002889	EFO_0006336	diastolic blood pressure	diastolic blood pressure	EFO_0006336	Other measurement	Cardiovascular

0.9845	0.00E+00	0.00E+00	PGS003106	MONDO_0005148	type 2 diabetes mellitus	type 2 diabetes mellitus	MONDO_0005148	Metabolic disorder	Metabolic	PGS003120	MONDO_0005148	type 2 diabetes mellitus	type 2 diabetes mellitus	MONDO_0005148	Digestive system disorder	Gastrointestinal
1.0	0.00E+00	0.00E+00	PGS000393	MONDO_0001672	bronchus cancer|lung cancer|tracheal cancer	bronchus cancer	MONDO_0001672	Cancer	Neoplasms	PGS000394	MONDO_0001672	bronchus cancer|lung cancer|tracheal cancer	bronchus cancer	MONDO_0001672	Cancer	Neoplasms
0.9968	0.00E+00	0.00E+00	PGS001169	EFO_0004995	lean body mass	lean body mass	EFO_0004995	Other measurement	Skeletal	PGS001002	EFO_0009805	whole body water mass	whole body water mass	EFO_0009805	Other measurement	Body measurement
0.9874	0.00E+00	0.00E+00	PGS001349	MONDO_0004975	Alzheimer disease	Alzheimer disease	MONDO_0004975	Neurological disorder	Neurological	PGS001827	MONDO_0001627	dementia	dementia	MONDO_0001627	Neurological disorder	Neurological
0.9819	0.00E+00	0.00E+00	PGS000321	EFO_0004908	testosterone measurement	testosterone measurement	EFO_0004908	Lipid or lipoprotein measurement	Metabolic	PGS000696	EFO_0004908	testosterone measurement	testosterone measurement	EFO_0004908	Lipid or lipoprotein measurement	Metabolic
1.0	0.00E+00	0.00E+00	PGS000452	EFO_0004193	basal cell carcinoma	basal cell carcinoma	EFO_0004193	Cancer	Neoplasms	PGS000453	EFO_0004193	basal cell carcinoma	basal cell carcinoma	EFO_0004193	Cancer	Neoplasms
0.9847	0.00E+00	0.00E+00	PGS001108	EFO_0007777	base metabolic rate measurement	base metabolic rate measurement	EFO_0007777	Other measurement	Metabolic	PGS001002	EFO_0009805	whole body water mass	whole body water mass	EFO_0009805	Other measurement	Body measurement
0.9927	0.00E+00	0.00E+00	PGS001038	EFO_0009270	heel bone mineral density	heel bone mineral density	EFO_0009270	Other measurement	Skeletal	PGS001403	EFO_0009270	heel bone mineral density	heel bone mineral density	EFO_0009270	Other measurement	Skeletal
1.0	0.00E+00	0.00E+00	PGS000464	EFO_0000707	squamous cell carcinoma	squamous cell carcinoma	EFO_0000707	Cancer	Neoplasms	PGS000461	EFO_0000707	squamous cell carcinoma	squamous cell carcinoma	EFO_0000707	Cancer	Neoplasms
0.9881	0.00E+00	0.00E+00	PGS003139	EFO_0004574	total cholesterol measurement	total cholesterol measurement	EFO_0004574	Lipid or lipoprotein measurement	Metabolic	PGS003134	EFO_0004574	total cholesterol measurement	total cholesterol measurement	EFO_0004574	Lipid or lipoprotein measurement	Metabolic
0.9923	0.00E+00	0.00E+00	PGS000866	EFO_0010821	liver fat measurement	liver fat measurement	EFO_0010821	Other measurement	Gastrointestinal	PGS000865	EFO_0010821	liver fat measurement	liver fat measurement	EFO_0010821	Other measurement	Gastrointestinal
0.982	0.00E+00	0.00E+00	PGS000929	MONDO_0001627	dementia	dementia	MONDO_0001627	Neurological disorder	Neurological	PGS001827	MONDO_0001627	dementia	dementia	MONDO_0001627	Neurological disorder	Neurological
1.0	0.00E+00	0.00E+00	PGS000545	EFO_0003893	ovarian neoplasm	ovarian neoplasm	EFO_0003893	Cancer	Neoplasms	PGS000544	EFO_0003893	ovarian neoplasm	ovarian neoplasm	EFO_0003893	Cancer	Neoplasms
1.0	0.00E+00	0.00E+00	PGS000952	EFO_0004514	bone quantitative ultrasound measurement	bone quantitative ultrasound measurement	EFO_0004514	Other measurement	Skeletal	PGS001037	EFO_0009270	heel bone mineral density	heel bone mineral density	EFO_0009270	Other measurement	Skeletal
0.9946	0.00E+00	0.00E+00	PGS000654	EFO_0001378	multiple myeloma	multiple myeloma	EFO_0001378	Cancer	Neoplasms	PGS000653	EFO_0001378	multiple myeloma	multiple myeloma	EFO_0001378	Cancer	Neoplasms
1.0	0.00E+00	0.00E+00	PGS000389	MONDO_0001672	bronchus cancer|lung cancer|tracheal cancer	bronchus cancer	MONDO_0001672	Cancer	Neoplasms	PGS000390	MONDO_0001672	bronchus cancer|lung cancer|tracheal cancer	bronchus cancer	MONDO_0001672	Cancer	Neoplasms
0.9999	0.00E+00	0.00E+00	PGS003297	EFO_0004617	cystatin C measurement|glomerular filtration rate	cystatin C measurement	EFO_0004617	Other measurement	Metabolic	PGS003312	EFO_0004617	cystatin C measurement|glomerular filtration rate	cystatin C measurement	EFO_0004617	Other measurement	Metabolic
0.9946	0.00E+00	0.00E+00	PGS000654	EFO_0001378	multiple myeloma	multiple myeloma	EFO_0001378	Cancer	Neoplasms	PGS000653	EFO_0001378	multiple myeloma	multiple myeloma	EFO_0001378	Immune system disorder	Immunological
0.9993	0.00E+00	0.00E+00	PGS001167	EFO_0004995	lean body mass	lean body mass	EFO_0004995	Other measurement	Skeletal	PGS001159	EFO_0005106	body composition measurement	body composition measurement	EFO_0005106	Body measurement	Body measurement
0.9805	0.00E+00	0.00E+00	PGS000456	EFO_0004193	basal cell carcinoma	basal cell carcinoma	EFO_0004193	Cancer	Neoplasms	PGS000459	EFO_0004193	basal cell carcinoma	basal cell carcinoma	EFO_0004193	Cancer	Neoplasms
0.9805	0.00E+00	0.00E+00	PGS000457	EFO_0004193	basal cell carcinoma	basal cell carcinoma	EFO_0004193	Cancer	Neoplasms	PGS000459	EFO_0004193	basal cell carcinoma	basal cell carcinoma	EFO_0004193	Cancer	Neoplasms
0.9804	0.00E+00	0.00E+00	PGS002854	EFO_0004340	body mass index	body mass index	EFO_0004340	Body measurement	Body measurement	PGS002844	EFO_0004340	body mass index	body mass index	EFO_0004340	Body measurement	Body measurement
0.9804	0.00E+00	0.00E+00	PGS001285	MONDO_0005271	allergic disease	allergic disease	MONDO_0005271	Immune system disorder	Immunological	PGS001284	MONDO_0005271	allergic disease	allergic disease	MONDO_0005271	Immune system disorder	Immunological
0.9804	0.00E+00	0.00E+00	PGS002840	EFO_0004340	body mass index	body mass index	EFO_0004340	Body measurement	Body measurement	PGS002845	EFO_0004340	body mass index	body mass index	EFO_0004340	Body measurement	Body measurement
0.9804	0.00E+00	0.00E+00	PGS002859	EFO_0004340	body mass index	body mass index	EFO_0004340	Body measurement	Body measurement	PGS002849	EFO_0004340	body mass index	body mass index	EFO_0004340	Body measurement	Body measurement
0.9802	0.00E+00	0.00E+00	PGS002850	EFO_0004340	body mass index	body mass index	EFO_0004340	Body measurement	Body measurement	PGS002855	EFO_0004340	body mass index	body mass index	EFO_0004340	Body measurement	Body measurement
0.9799	0.00E+00	0.00E+00	PGS002089	EFO_0003898	ankylosing spondylitis	ankylosing spondylitis	EFO_0003898	Immune system disorder	Immunological	PGS001268	EFO_0003898	ankylosing spondylitis	ankylosing spondylitis	EFO_0003898	Immune system disorder	Immunological
0.9797	0.00E+00	0.00E+00	PGS001179	EFO_0004718	vascular dementia	vascular dementia	EFO_0004718	Neurological disorder	Neurological	PGS001828	MONDO_0004975	Alzheimer disease	Alzheimer disease	MONDO_0004975	Neurological disorder	Neurological
0.9797	0.00E+00	0.00E+00	PGS001179	EFO_0004718	vascular dementia	vascular dementia	EFO_0004718	Cardiovascular disease	Cardiovascular	PGS001828	MONDO_0004975	Alzheimer disease	Alzheimer disease	MONDO_0004975	Neurological disorder	Neurological
0.9796	0.00E+00	0.00E+00	PGS001942	EFO_0004570	bilirubin measurement	bilirubin measurement	EFO_0004570	Other measurement	Metabolic	PGS002160	EFO_0004570	bilirubin measurement	bilirubin measurement	EFO_0004570	Other measurement	Metabolic
0.9796	0.00E+00	0.00E+00	PGS002589	EFO_0000400	diabetes mellitus	diabetes mellitus	EFO_0000400	Metabolic disorder	Metabolic	PGS002622	MONDO_0005148	type 2 diabetes mellitus	type 2 diabetes mellitus	MONDO_0005148	Metabolic disorder	Metabolic
0.9796	0.00E+00	0.00E+00	PGS002589	EFO_0000400	diabetes mellitus	diabetes mellitus	EFO_0000400	Metabolic disorder	Metabolic	PGS002622	MONDO_0005148	type 2 diabetes mellitus	type 2 diabetes mellitus	MONDO_0005148	Digestive system disorder	Gastrointestinal
0.9796	0.00E+00	0.00E+00	PGS002589	EFO_0000400	diabetes mellitus	diabetes mellitus	EFO_0000400	Digestive system disorder	Gastrointestinal	PGS002622	MONDO_0005148	type 2 diabetes mellitus	type 2 diabetes mellitus	MONDO_0005148	Metabolic disorder	Metabolic
0.9796	0.00E+00	0.00E+00	PGS002589	EFO_0000400	diabetes mellitus	diabetes mellitus	EFO_0000400	Digestive system disorder	Gastrointestinal	PGS002622	MONDO_0005148	type 2 diabetes mellitus	type 2 diabetes mellitus	MONDO_0005148	Digestive system disorder	Gastrointestinal
0.9795	0.00E+00	0.00E+00	PGS000946	HP_0000726	Dementia	Dementia	HP_0000726	Other trait	Psychiatric	PGS001348	MONDO_0004975	Alzheimer disease	Alzheimer disease	MONDO_0004975	Neurological disorder	Neurological
0.9794	0.00E+00	0.00E+00	PGS000499	EFO_0000305	breast carcinoma	breast carcinoma	EFO_0000305	Cancer	Neoplasms	PGS000500	EFO_0000305	breast carcinoma	breast carcinoma	EFO_0000305	Cancer	Neoplasms
0.9794	0.00E+00	0.00E+00	PGS000945	HP_0000726	Dementia|Alzheimer disease	Dementia	HP_0000726	Other trait	Psychiatric	PGS001348	MONDO_0004975	Alzheimer disease	Alzheimer disease	MONDO_0004975	Neurological disorder	Neurological
0.9794	0.00E+00	0.00E+00	PGS001828	MONDO_0004975	Alzheimer disease	Alzheimer disease	MONDO_0004975	Neurological disorder	Neurological	PGS001348	MONDO_0004975	Alzheimer disease	Alzheimer disease	MONDO_0004975	Neurological disorder	Neurological
0.9791	0.00E+00	0.00E+00	PGS001179	EFO_0004718	vascular dementia	vascular dementia	EFO_0004718	Cardiovascular disease	Cardiovascular	PGS000945	HP_0000726	Dementia|Alzheimer disease	Dementia	HP_0000726	Other trait	Psychiatric
0.9791	0.00E+00	0.00E+00	PGS001179	EFO_0004718	vascular dementia	vascular dementia	EFO_0004718	Neurological disorder	Neurological	PGS000945	HP_0000726	Dementia|Alzheimer disease	Dementia	HP_0000726	Other trait	Psychiatric
0.9788	0.00E+00	0.00E+00	PGS000583	EFO_0001663	prostate carcinoma	prostate carcinoma	EFO_0001663	Cancer	Neoplasms	PGS000584	EFO_0001663	prostate carcinoma	prostate carcinoma	EFO_0001663	Cancer	Neoplasms
0.9788	0.00E+00	0.00E+00	PGS001530	EFO_0007772	calcaneal bone quantitative ultrasound measurement	calcaneal bone quantitative ultrasound measurement	EFO_0007772	Other measurement	Skeletal	PGS001403	EFO_0009270	heel bone mineral density	heel bone mineral density	EFO_0009270	Other measurement	Skeletal
0.9787	0.00E+00	0.00E+00	PGS000508	EFO_0000305	breast carcinoma	breast carcinoma	EFO_0000305	Cancer	Neoplasms	PGS000507	EFO_0000305	breast carcinoma	breast carcinoma	EFO_0000305	Cancer	Neoplasms
0.9787	0.00E+00	0.00E+00	PGS002888	EFO_0004458	C-reactive protein measurement	C-reactive protein measurement	EFO_0004458	Inflammatory measurement	Immunological	PGS002878	EFO_0004458	C-reactive protein measurement	C-reactive protein measurement	EFO_0004458	Inflammatory measurement	Immunological
0.9786	0.00E+00	0.00E+00	PGS001531	EFO_0007772	calcaneal bone quantitative ultrasound measurement	calcaneal bone quantitative ultrasound measurement	EFO_0007772	Other measurement	Skeletal	PGS001404	EFO_0009270	heel bone mineral density	heel bone mineral density	EFO_0009270	Other measurement	Skeletal
0.9786	0.00E+00	0.00E+00	PGS000631	EFO_0002892	thyroid carcinoma	thyroid carcinoma	EFO_0002892	Cancer	Neoplasms	PGS000208	EFO_0002892	thyroid carcinoma	thyroid carcinoma	EFO_0002892	Cancer	Neoplasms
0.9786	0.00E+00	0.00E+00	PGS000630	EFO_0002892	thyroid carcinoma	thyroid carcinoma	EFO_0002892	Cancer	Neoplasms	PGS000208	EFO_0002892	thyroid carcinoma	thyroid carcinoma	EFO_0002892	Cancer	Neoplasms
0.9784	0.00E+00	0.00E+00	PGS000946	HP_0000726	Dementia	Dementia	HP_0000726	Other trait	Psychiatric	PGS001828	MONDO_0004975	Alzheimer disease	Alzheimer disease	MONDO_0004975	Neurological disorder	Neurological
0.9783	0.00E+00	0.00E+00	PGS002411	EFO_0004527	mean corpuscular hemoglobin	mean corpuscular hemoglobin	EFO_0004527	Hematological measurement	Hematological	PGS002558	EFO_0004527	mean corpuscular hemoglobin	mean corpuscular hemoglobin	EFO_0004527	Hematological measurement	Hematological
0.9783	0.00E+00	0.00E+00	PGS000209	EFO_0002892	thyroid carcinoma	thyroid carcinoma	EFO_0002892	Cancer	Neoplasms	PGS000208	EFO_0002892	thyroid carcinoma	thyroid carcinoma	EFO_0002892	Cancer	Neoplasms
0.9783	0.00E+00	0.00E+00	PGS000786	EFO_1001512	endometrial carcinoma	endometrial carcinoma	EFO_1001512	Cancer	Neoplasms	PGS000075	EFO_1001512	endometrial carcinoma	endometrial carcinoma	EFO_1001512	Cancer	Neoplasms
0.9781	0.00E+00	5.24E-308	PGS001158	EFO_0005106	body composition measurement	body composition measurement	EFO_0005106	Body measurement	Body measurement	PGS001167	EFO_0004995	lean body mass	lean body mass	EFO_0004995	Other measurement	Skeletal
0.9779	0.00E+00	3.42E-307	PGS001271	MONDO_0005301	multiple sclerosis	multiple sclerosis	MONDO_0005301	Immune system disorder	Immunological	PGS001270	MONDO_0005301	multiple sclerosis	multiple sclerosis	MONDO_0005301	Immune system disorder	Immunological
0.9779	0.00E+00	3.42E-307	PGS001271	MONDO_0005301	multiple sclerosis	multiple sclerosis	MONDO_0005301	Neurological disorder	Neurological	PGS001270	MONDO_0005301	multiple sclerosis	multiple sclerosis	MONDO_0005301	Immune system disorder	Immunological
0.9779	0.00E+00	3.42E-307	PGS001271	MONDO_0005301	multiple sclerosis	multiple sclerosis	MONDO_0005301	Immune system disorder	Immunological	PGS001270	MONDO_0005301	multiple sclerosis	multiple sclerosis	MONDO_0005301	Neurological disorder	Neurological
0.9779	0.00E+00	3.42E-307	PGS001271	MONDO_0005301	multiple sclerosis	multiple sclerosis	MONDO_0005301	Neurological disorder	Neurological	PGS001270	MONDO_0005301	multiple sclerosis	multiple sclerosis	MONDO_0005301	Neurological disorder	Neurological
0.9776	0.00E+00	1.15E-305	PGS001158	EFO_0005106	body composition measurement	body composition measurement	EFO_0005106	Body measurement	Body measurement	PGS001159	EFO_0005106	body composition measurement	body composition measurement	EFO_0005106	Body measurement	Body measurement
0.9775	0.00E+00	1.37E-305	PGS001166	EFO_0004995	lean body mass	lean body mass	EFO_0004995	Other measurement	Skeletal	PGS001167	EFO_0004995	lean body mass	lean body mass	EFO_0004995	Other measurement	Skeletal
0.9772	0.00E+00	3.61E-304	PGS003140	EFO_0004574	total cholesterol measurement	total cholesterol measurement	EFO_0004574	Lipid or lipoprotein measurement	Metabolic	PGS003135	EFO_0004574	total cholesterol measurement	total cholesterol measurement	EFO_0004574	Lipid or lipoprotein measurement	Metabolic
0.9772	2.24E-308	3.79E-304	PGS000774	EFO_1000649	estrogen-receptor positive breast cancer	estrogen-receptor positive breast cancer	EFO_1000649	Cancer	Neoplasms	PGS000052	EFO_0000305	breast carcinoma	breast carcinoma	EFO_0000305	Cancer	Neoplasms
0.9772	2.27E-308	3.84E-304	PGS000780	MONDO_0005271	allergic disease	allergic disease	MONDO_0005271	Immune system disorder	Immunological	PGS000810	MONDO_0005271	allergic disease	allergic disease	MONDO_0005271	Immune system disorder	Immunological
0.9771	7.81E-308	1.31E-303	PGS001166	EFO_0004995	lean body mass	lean body mass	EFO_0004995	Other measurement	Skeletal	PGS001159	EFO_0005106	body composition measurement	body composition measurement	EFO_0005106	Body measurement	Body measurement
0.977	1.07E-307	1.79E-303	PGS002543	EFO_0001379	endocrine system disease|diabetes mellitus	endocrine system disease	EFO_0001379	Other disease	Metabolic	PGS002540	EFO_0000400	diabetes mellitus	diabetes mellitus	EFO_0000400	Metabolic disorder	Metabolic
0.977	1.07E-307	1.79E-303	PGS002543	EFO_0001379	endocrine system disease|diabetes mellitus	endocrine system disease	EFO_0001379	Other disease	Metabolic	PGS002540	EFO_0000400	diabetes mellitus	diabetes mellitus	EFO_0000400	Digestive system disorder	Gastrointestinal
0.977	1.08E-307	1.79E-303	PGS000946	HP_0000726	Dementia	Dementia	HP_0000726	Other trait	Psychiatric	PGS001827	MONDO_0001627	dementia	dementia	MONDO_0001627	Neurological disorder	Neurological
0.977	1.07E-307	1.79E-303	PGS002543	EFO_0001379	endocrine system disease|diabetes mellitus	endocrine system disease	EFO_0001379	Other disease	Metabolic	PGS002396	EFO_0001379	endocrine system disease|diabetes mellitus	endocrine system disease	EFO_0001379	Other disease	Metabolic
0.977	2.08E-307	3.45E-303	PGS002426	MONDO_0005148	type 2 diabetes mellitus	type 2 diabetes mellitus	MONDO_0005148	Digestive system disorder	Gastrointestinal	PGS002475	MONDO_0005148	type 2 diabetes mellitus	type 2 diabetes mellitus	MONDO_0005148	Metabolic disorder	Metabolic
0.977	2.08E-307	3.45E-303	PGS002426	MONDO_0005148	type 2 diabetes mellitus	type 2 diabetes mellitus	MONDO_0005148	Metabolic disorder	Metabolic	PGS002475	MONDO_0005148	type 2 diabetes mellitus	type 2 diabetes mellitus	MONDO_0005148	Digestive system disorder	Gastrointestinal
0.977	2.08E-307	3.45E-303	PGS002426	MONDO_0005148	type 2 diabetes mellitus	type 2 diabetes mellitus	MONDO_0005148	Metabolic disorder	Metabolic	PGS002475	MONDO_0005148	type 2 diabetes mellitus	type 2 diabetes mellitus	MONDO_0005148	Metabolic disorder	Metabolic
0.977	2.08E-307	3.45E-303	PGS002426	MONDO_0005148	type 2 diabetes mellitus	type 2 diabetes mellitus	MONDO_0005148	Digestive system disorder	Gastrointestinal	PGS002475	MONDO_0005148	type 2 diabetes mellitus	type 2 diabetes mellitus	MONDO_0005148	Digestive system disorder	Gastrointestinal
0.9769	3.42E-307	5.64E-303	PGS001349	MONDO_0004975	Alzheimer disease	Alzheimer disease	MONDO_0004975	Neurological disorder	Neurological	PGS000945	HP_0000726	Dementia|Alzheimer disease	Dementia	HP_0000726	Other trait	Psychiatric
0.9769	5.50E-307	9.06E-303	PGS002989	EFO_0004339	body height	body height	EFO_0004339	Body measurement	Body measurement	PGS002979	EFO_0004339	body height	body height	EFO_0004339	Body measurement	Body measurement
0.9768	9.81E-307	1.61E-302	PGS001106	EFO_0007800	body fat percentage	body fat percentage	EFO_0007800	Body measurement	Body measurement	PGS001102	EFO_0007800	body fat percentage	body fat percentage	EFO_0007800	Body measurement	Body measurement
0.9767	2.43E-306	3.98E-302	PGS000794	EFO_0002618	pancreatic carcinoma	pancreatic carcinoma	EFO_0002618	Digestive system disorder	Gastrointestinal	PGS000083	EFO_0002618	pancreatic carcinoma	pancreatic carcinoma	EFO_0002618	Digestive system disorder	Gastrointestinal
0.9767	2.43E-306	3.98E-302	PGS000794	EFO_0002618	pancreatic carcinoma	pancreatic carcinoma	EFO_0002618	Cancer	Neoplasms	PGS000083	EFO_0002618	pancreatic carcinoma	pancreatic carcinoma	EFO_0002618	Digestive system disorder	Gastrointestinal
0.9767	2.43E-306	3.98E-302	PGS000794	EFO_0002618	pancreatic carcinoma	pancreatic carcinoma	EFO_0002618	Cancer	Neoplasms	PGS000083	EFO_0002618	pancreatic carcinoma	pancreatic carcinoma	EFO_0002618	Cancer	Neoplasms
0.9767	2.43E-306	3.98E-302	PGS000794	EFO_0002618	pancreatic carcinoma	pancreatic carcinoma	EFO_0002618	Digestive system disorder	Gastrointestinal	PGS000083	EFO_0002618	pancreatic carcinoma	pancreatic carcinoma	EFO_0002618	Cancer	Neoplasms
0.9764	4.34E-305	7.07E-301	PGS002576	EFO_0004308	leukocyte count	leukocyte count	EFO_0004308	Hematological measurement	Hematological	PGS002625	EFO_0004308	leukocyte count	leukocyte count	EFO_0004308	Hematological measurement	Hematological
0.9758	2.27E-302	3.69E-298	PGS002844	EFO_0004340	body mass index	body mass index	EFO_0004340	Body measurement	Body measurement	PGS002849	EFO_0004340	body mass index	body mass index	EFO_0004340	Body measurement	Body measurement
0.9755	1.58E-301	2.55E-297	PGS003035	EFO_0004611	low density lipoprotein cholesterol measurement	low density lipoprotein cholesterol measurement	EFO_0004611	Cardiovascular measurement	Cardiovascular	PGS003030	EFO_0004611	low density lipoprotein cholesterol measurement	measurement	EFO_0004611	Lipid or lipoprotein measurement	Metabolic
0.9755	1.58E-301	2.55E-297	PGS003035	EFO_0004611	low density lipoprotein cholesterol measurement	low density lipoprotein cholesterol measurement	EFO_0004611	Cardiovascular measurement	Cardiovascular	PGS003030	EFO_0004611	low density lipoprotein cholesterol measurement	measurement	EFO_0004611	Cardiovascular measurement	Cardiovascular
0.9755	1.58E-301	2.55E-297	PGS003035	EFO_0004611	low density lipoprotein cholesterol measurement	low density lipoprotein cholesterol measurement	EFO_0004611	Lipid or lipoprotein measurement	Metabolic	PGS003030	EFO_0004611	low density lipoprotein cholesterol measurement	measurement	EFO_0004611	Lipid or lipoprotein measurement	Metabolic
0.9755	1.58E-301	2.55E-297	PGS003035	EFO_0004611	low density lipoprotein cholesterol measurement	low density lipoprotein cholesterol measurement	EFO_0004611	Lipid or lipoprotein measurement	Metabolic	PGS003030	EFO_0004611	low density lipoprotein cholesterol measurement	measurement	EFO_0004611	Cardiovascular measurement	Cardiovascular
0.9753	1.42E-300	2.28E-296	PGS002884	EFO_0004458	C-reactive protein measurement	C-reactive protein measurement	EFO_0004458	Inflammatory measurement	Immunological	PGS002885	EFO_0004458	C-reactive protein measurement	C-reactive protein measurement	EFO_0004458	Inflammatory measurement	Immunological
0.9753	1.42E-300	2.28E-296	PGS002886	EFO_0004458	C-reactive protein measurement	C-reactive protein measurement	EFO_0004458	Inflammatory measurement	Immunological	PGS002884	EFO_0004458	C-reactive protein measurement	C-reactive protein measurement	EFO_0004458	Inflammatory measurement	Immunological
0.9752	3.36E-300	5.37E-296	PGS002840	EFO_0004340	body mass index	body mass index	EFO_0004340	Body measurement	Body measurement	PGS002859	EFO_0004340	body mass index	body mass index	EFO_0004340	Body measurement	Body measurement
0.975	1.63E-299	2.60E-295	PGS002445	EFO_0001379	endocrine system disease|diabetes mellitus	endocrine system disease	EFO_0001379	Other disease	Metabolic	PGS002396	EFO_0001379	endocrine system disease|diabetes mellitus	endocrine system disease	EFO_0001379	Other disease	Metabolic
0.9749	6.37E-299	1.01E-294	PGS003228	EFO_0005208	glomerular filtration rate	glomerular filtration rate	EFO_0005208	Other measurement	Genitourinary	PGS003223	EFO_0005208	glomerular filtration rate	glomerular filtration rate	EFO_0005208	Other measurement	Genitourinary
0.9747	2.32E-298	3.68E-294	PGS000507	EFO_0000305	breast carcinoma	breast carcinoma	EFO_0000305	Cancer	Neoplasms	PGS000335	EFO_0000305	breast carcinoma	breast carcinoma	EFO_0000305	Cancer	Neoplasms
0.9747	2.53E-298	4.00E-294	PGS002840	EFO_0004340	body mass index	body mass index	EFO_0004340	Body measurement	Body measurement	PGS002855	EFO_0004340	body mass index	body mass index	EFO_0004340	Body measurement	Body measurement
0.9744	4.27E-297	6.73E-293	PGS002854	EFO_0004340	body mass index	body mass index	EFO_0004340	Body measurement	Body measurement	PGS002859	EFO_0004340	body mass index	body mass index	EFO_0004340	Body measurement	Body measurement
0.9744	4.34E-297	6.82E-293	PGS001179	EFO_0004718	vascular dementia	vascular dementia	EFO_0004718	Neurological disorder	Neurological	PGS001827	MONDO_0001627	dementia	dementia	MONDO_0001627	Neurological disorder	Neurological
0.9744	4.34E-297	6.82E-293	PGS001179	EFO_0004718	vascular dementia	vascular dementia	EFO_0004718	Cardiovascular disease	Cardiovascular	PGS001827	MONDO_0001627	dementia	dementia	MONDO_0001627	Neurological disorder	Neurological
0.9743	1.05E-296	1.64E-292	PGS003191	EFO_0004631	vitamin D measurement	vitamin D measurement	EFO_0004631	Lipid or lipoprotein measurement	Metabolic	PGS003196	EFO_0004631	vitamin D measurement	vitamin D measurement	EFO_0004631	Lipid or lipoprotein measurement	Metabolic
0.9743	1.17E-296	1.83E-292	PGS001897	EFO_0003784	skin pigmentation	skin pigmentation	EFO_0003784	Other trait	Dermatological	PGS002110	EFO_0003784	skin pigmentation	skin pigmentation	EFO_0003784	Other trait	Dermatological
0.9741	5.18E-296	8.05E-292	PGS002412	EFO_0004584	mean platelet volume	mean platelet volume	EFO_0004584	Hematological measurement	Hematological	PGS002461	EFO_0004584	mean platelet volume	mean platelet volume	EFO_0004584	Hematological measurement	Hematological
0.9741	6.20E-296	9.61E-292	PGS001878	EFO_0008518	polymyalgia rheumatica	polymyalgia rheumatica	EFO_0008518	Other disease	Immunological	PGS002091	EFO_0008518	polymyalgia rheumatica	polymyalgia rheumatica	EFO_0008518	Other disease	Immunological
0.9741	7.32E-296	1.13E-291	PGS002592	EFO_0001379	endocrine system disease|diabetes mellitus	endocrine system disease	EFO_0001379	Other disease	Metabolic	PGS002622	MONDO_0005148	type 2 diabetes mellitus	type 2 diabetes mellitus	MONDO_0005148	Metabolic disorder	Metabolic
0.9741	7.32E-296	1.13E-291	PGS002592	EFO_0001379	endocrine system disease|diabetes mellitus	endocrine system disease	EFO_0001379	Other disease	Metabolic	PGS002622	MONDO_0005148	type 2 diabetes mellitus	type 2 diabetes mellitus	MONDO_0005148	Digestive system disorder	Gastrointestinal
0.9739	3.90E-295	6.00E-291	PGS001349	MONDO_0004975	Alzheimer disease	Alzheimer disease	MONDO_0004975	Neurological disorder	Neurological	PGS002035	MONDO_0001627	dementia	dementia	MONDO_0001627	Neurological disorder	Neurological
0.9739	3.99E-295	6.13E-291	PGS003103	MONDO_0005148	type 2 diabetes mellitus	type 2 diabetes mellitus	MONDO_0005148	Metabolic disorder	Metabolic	PGS003118	MONDO_0005148	type 2 diabetes mellitus	type 2 diabetes mellitus	MONDO_0005148	Digestive system disorder	Gastrointestinal
0.9739	3.99E-295	6.13E-291	PGS003103	MONDO_0005148	type 2 diabetes mellitus	type 2 diabetes mellitus	MONDO_0005148	Metabolic disorder	Metabolic	PGS003118	MONDO_0005148	type 2 diabetes mellitus	type 2 diabetes mellitus	MONDO_0005148	Metabolic disorder	Metabolic
0.9739	3.99E-295	6.13E-291	PGS003103	MONDO_0005148	type 2 diabetes mellitus	type 2 diabetes mellitus	MONDO_0005148	Digestive system disorder	Gastrointestinal	PGS003118	MONDO_0005148	type 2 diabetes mellitus	type 2 diabetes mellitus	MONDO_0005148	Metabolic disorder	Metabolic

0.9739	3.99E-295	6.13E-291	PGS003103	MONDO_0005148	type 2 diabetes mellitus	type 2 diabetes mellitus	MONDO_0005148	Digestive system disorder	Gastrointestinal	PGS003118	MONDO_0005148	type 2 diabetes mellitus	type 2 diabetes mellitus	MONDO_0005148	Digestive system disorder	Gastrointestinal
0.9738	7.25E-295	1.11E-290	PGS003069	EFO_0006335	systolic blood pressure	systolic blood pressure	EFO_0006335	Other measurement	Cardiovascular	PGS003074	EFO_0006335	systolic blood pressure	systolic blood pressure	EFO_0006335	Other measurement	Cardiovascular
0.9737	1.34E-294	2.04E-290	PGS000946	HP_0000726	Dementia	Dementia	HP_0000726	Other trait	Psychiatric	PGS000945	HP_0000726	Dementia|Alzheimer disease	Dementia	HP_0000726	Other trait	Psychiatric
0.9736	5.39E-294	8.19E-290	PGS000945	HP_0000726	Dementia|Alzheimer disease	Dementia	HP_0000726	Other trait	Psychiatric	PGS001828	MONDO_0004975	Alzheimer disease	Alzheimer disease	MONDO_0004975	Neurological disorder	Neurological
0.9735	7.27E-294	1.10E-289	PGS000508	EFO_0000305	breast carcinoma	breast carcinoma	EFO_0000305	Cancer	Neoplasms	PGS000335	EFO_0000305	breast carcinoma	breast carcinoma	EFO_0000305	Cancer	Neoplasms
0.9735	1.05E-293	1.59E-289	PGS000713	MONDO_0005148	type 2 diabetes mellitus	type 2 diabetes mellitus	MONDO_0005148	Digestive system disorder	Gastrointestinal	PGS000712	MONDO_0005148	type 2 diabetes mellitus	type 2 diabetes mellitus	MONDO_0005148	Digestive system disorder	Gastrointestinal
0.9735	1.05E-293	1.59E-289	PGS000713	MONDO_0005148	type 2 diabetes mellitus	type 2 diabetes mellitus	MONDO_0005148	Digestive system disorder	Gastrointestinal	PGS000712	MONDO_0005148	type 2 diabetes mellitus	type 2 diabetes mellitus	MONDO_0005148	Metabolic disorder	Metabolic
0.9735	1.05E-293	1.59E-289	PGS000713	MONDO_0005148	type 2 diabetes mellitus	type 2 diabetes mellitus	MONDO_0005148	Metabolic disorder	Metabolic	PGS000712	MONDO_0005148	type 2 diabetes mellitus	type 2 diabetes mellitus	MONDO_0005148	Digestive system disorder	Gastrointestinal
0.9735	1.05E-293	1.59E-289	PGS000713	MONDO_0005148	type 2 diabetes mellitus	type 2 diabetes mellitus	MONDO_0005148	Metabolic disorder	Metabolic	PGS000712	MONDO_0005148	type 2 diabetes mellitus	type 2 diabetes mellitus	MONDO_0005148	Metabolic disorder	Metabolic
0.9734	3.11E-293	4.68E-289	PGS002475	MONDO_0005148	type 2 diabetes mellitus	type 2 diabetes mellitus	MONDO_0005148	Digestive system disorder	Gastrointestinal	PGS002442	EFO_0000400	diabetes mellitus	diabetes mellitus	EFO_0000400	Metabolic disorder	Metabolic
0.9734	3.11E-293	4.68E-289	PGS002475	MONDO_0005148	type 2 diabetes mellitus	type 2 diabetes mellitus	MONDO_0005148	Digestive system disorder	Gastrointestinal	PGS002442	EFO_0000400	diabetes mellitus	diabetes mellitus	EFO_0000400	Digestive system disorder	Gastrointestinal
0.9734	3.11E-293	4.68E-289	PGS002475	MONDO_0005148	type 2 diabetes mellitus	type 2 diabetes mellitus	MONDO_0005148	Metabolic disorder	Metabolic	PGS002442	EFO_0000400	diabetes mellitus	diabetes mellitus	EFO_0000400	Metabolic disorder	Metabolic
0.9734	3.11E-293	4.68E-289	PGS002475	MONDO_0005148	type 2 diabetes mellitus	type 2 diabetes mellitus	MONDO_0005148	Metabolic disorder	Metabolic	PGS002442	EFO_0000400	diabetes mellitus	diabetes mellitus	EFO_0000400	Digestive system disorder	Gastrointestinal
0.9733	4.18E-293	6.26E-289	PGS000457	EFO_0004193	basal cell carcinoma	basal cell carcinoma	EFO_0004193	Cancer	Neoplasms	PGS000458	EFO_0004193	basal cell carcinoma	basal cell carcinoma	EFO_0004193	Cancer	Neoplasms
0.9733	4.18E-293	6.26E-289	PGS000456	EFO_0004193	basal cell carcinoma	basal cell carcinoma	EFO_0004193	Cancer	Neoplasms	PGS000458	EFO_0004193	basal cell carcinoma	basal cell carcinoma	EFO_0004193	Cancer	Neoplasms
0.9733	5.45E-293	8.13E-289	PGS002845	EFO_0004340	body mass index	body mass index	EFO_0004340	Body measurement	Body measurement	PGS002850	EFO_0004340	body mass index	body mass index	EFO_0004340	Body measurement	Body measurement
0.9733	6.76E-293	1.01E-288	PGS000046	EFO_1000649	estrogen-receptor positive breast cancer	estrogen-receptor positive breast cancer	EFO_1000649	Cancer	Neoplasms	PGS000045	EFO_0000305	breast carcinoma	breast carcinoma	EFO_0000305	Cancer	Neoplasms
0.9731	3.08E-292	4.56E-288	PGS000929	MONDO_0001627	dementia	dementia	MONDO_0001627	Neurological disorder	Neurological	PGS001347	EFO_0009268	family history of Alzheimer's disease	family history of Alzheimer's disease	EFO_0009268	Other measurement	Neurological
0.9729	2.09E-291	3.09E-287	PGS002545	EFO_0004713	FEV|FEC ratio	FEV|FEC ratio	EFO_0004713	Other measurement	Respiratory	PGS002594	EFO_0004713	FEV|FEC ratio	FEV|FEC ratio	EFO_0004713	Other measurement	Respiratory
0.9728	3.51E-291	5.17E-287	PGS000912	EFO_0006336	diastolic blood pressure	diastolic blood pressure	EFO_0006336	Other measurement	Cardiovascular	PGS002898	EFO_0006336	diastolic blood pressure	diastolic blood pressure	EFO_0006336	Other measurement	Cardiovascular
0.9726	1.51E-290	2.21E-286	PGS002427	EFO_0004631	vitamin D measurement	vitamin D measurement	EFO_0004631	Lipid or lipoprotein measurement	Metabolic	PGS002574	EFO_0004631	vitamin D measurement	vitamin D measurement	EFO_0004631	Lipid or lipoprotein measurement	Metabolic
0.9726	1.59E-290	2.33E-286	PGS002898	EFO_0006336	diastolic blood pressure	diastolic blood pressure	EFO_0006336	Other measurement	Cardiovascular	PGS002893	EFO_0006336	diastolic blood pressure	diastolic blood pressure	EFO_0006336	Other measurement	Cardiovascular
0.9725	4.38E-290	6.40E-286	PGS002451	EFO_0007822	hair colour measurement	hair colour measurement	EFO_0007822	Other measurement	Dermatological	PGS002402	EFO_0007822	hair colour measurement	hair colour measurement	EFO_0007822	Other measurement	Dermatological
0.9724	1.37E-289	2.00E-285	PGS002757	EFO_0003884	chronic kidney disease	chronic kidney disease	EFO_0003884	Other disease	Metabolic	PGS003223	EFO_0005208	glomerular filtration rate	glomerular filtration rate	EFO_0005208	Other measurement	Genitourinary
0.9723	2.01E-289	2.92E-285	PGS000583	EFO_0001663	prostate carcinoma	prostate carcinoma	EFO_0001663	Cancer	Neoplasms	PGS002240	EFO_0001663	prostate carcinoma	prostate carcinoma	EFO_0001663	Cancer	Neoplasms
0.9723	2.57E-289	3.72E-285	PGS003313	EFO_0004617	cystatin C measurement|glomerular filtration rate	cystatin C measurement	EFO_0004617	Other measurement	Metabolic	PGS003298	EFO_0004617	cystatin C measurement|glomerular filtration rate	cystatin C measurement	EFO_0004617	Other measurement	Metabolic
0.9723	3.11E-289	4.48E-285	PGS003190	EFO_0004631	vitamin D measurement	vitamin D measurement	EFO_0004631	Lipid or lipoprotein measurement	Metabolic	PGS003195	EFO_0004631	vitamin D measurement	vitamin D measurement	EFO_0004631	Lipid or lipoprotein measurement	Metabolic
0.9723	3.27E-289	4.70E-285	PGS002970	EFO_0004339	body height	body height	EFO_0004339	Body measurement	Body measurement	PGS002965	EFO_0004339	body height	body height	EFO_0004339	Body measurement	Body measurement
0.9722	4.19E-289	6.00E-285	PGS000913	EFO_0006335	systolic blood pressure	systolic blood pressure	EFO_0006335	Other measurement	Cardiovascular	PGS003073	EFO_0006335	systolic blood pressure	systolic blood pressure	EFO_0006335	Other measurement	Cardiovascular
0.9722	4.98E-289	7.12E-285	PGS002221	EFO_0010701	mean reticulocyte volume	mean reticulocyte volume	EFO_0010701	Hematological measurement	Hematological	PGS002003	EFO_0010701	mean reticulocyte volume	mean reticulocyte volume	EFO_0010701	Hematological measurement	Hematological
0.9721	1.56E-288	2.22E-284	PGS002080	EFO_1001159	Rh isoimmunization	Rh isoimmunization	EFO_1001159	Other disease	Immunological	PGS001868	EFO_1001159	Rh isoimmunization	Rh isoimmunization	EFO_1001159	Other disease	Immunological
0.9721	1.58E-288	2.25E-284	PGS003078	EFO_0006335	systolic blood pressure	systolic blood pressure	EFO_0006335	Other measurement	Cardiovascular	PGS003073	EFO_0006335	systolic blood pressure	systolic blood pressure	EFO_0006335	Other measurement	Cardiovascular
0.972	2.04E-288	2.90E-284	PGS000945	HP_0000726	Dementia|Alzheimer disease	Dementia	HP_0000726	Other trait	Psychiatric	PGS001827	MONDO_0001627	dementia	dementia	MONDO_0001627	Neurological disorder	Neurological
0.972	2.06E-288	2.91E-284	PGS002547	EFO_0004468	glucose measurement	glucose measurement	EFO_0004468	Other measurement	Metabolic	PGS002592	EFO_0001379	endocrine system disease|diabetes mellitus	endocrine system disease	EFO_0001379	Other disease	Metabolic
0.9718	1.05E-287	1.47E-283	PGS002624	EFO_0004343	waist-hip ratio	waist-hip ratio	EFO_0004343	Body measurement	Body measurement	PGS002575	EFO_0004343	waist-hip ratio	waist-hip ratio	EFO_0004343	Body measurement	Body measurement
-0.9717	2.53E-287	3.55E-283	PGS001139	EFO_0005671	smoking behaviour measurement	smoking behaviour measurement	EFO_0005671	Other measurement	Psychiatric	PGS001515	EFO_0006525	cigarettes per day measurement	cigarettes per day measurement	EFO_0006525	Other measurement	Psychiatric
0.9715	1.07E-286	1.49E-282	PGS000646	EFO_0000095	chronic lymphocytic leukemia	chronic lymphocytic leukemia	EFO_0000095	Cancer	Neoplasms	PGS000649	EFO_0000095	chronic lymphocytic leukemia	chronic lymphocytic leukemia	EFO_0000095	Cancer	Neoplasms
0.9715	1.07E-286	1.49E-282	PGS000647	EFO_0000095	chronic lymphocytic leukemia	chronic lymphocytic leukemia	EFO_0000095	Cancer	Neoplasms	PGS000649	EFO_0000095	chronic lymphocytic leukemia	chronic lymphocytic leukemia	EFO_0000095	Cancer	Neoplasms
0.9715	1.07E-286	1.49E-282	PGS000647	EFO_0000095	chronic lymphocytic leukemia	chronic lymphocytic leukemia	EFO_0000095	Cancer	Neoplasms	PGS000649	EFO_0000095	chronic lymphocytic leukemia	chronic lymphocytic leukemia	EFO_0000095	Immune system disorder	Immunological
0.9715	1.07E-286	1.49E-282	PGS000646	EFO_0000095	chronic lymphocytic leukemia	chronic lymphocytic leukemia	EFO_0000095	Immune system disorder	Immunological	PGS000649	EFO_0000095	chronic lymphocytic leukemia	chronic lymphocytic leukemia	EFO_0000095	Cancer	Neoplasms
0.9715	1.07E-286	1.49E-282	PGS000646	EFO_0000095	chronic lymphocytic leukemia	chronic lymphocytic leukemia	EFO_0000095	Immune system disorder	Immunological	PGS000649	EFO_0000095	chronic lymphocytic leukemia	chronic lymphocytic leukemia	EFO_0000095	Immune system disorder	Immunological
0.9715	1.07E-286	1.49E-282	PGS000646	EFO_0000095	chronic lymphocytic leukemia	chronic lymphocytic leukemia	EFO_0000095	Cancer	Neoplasms	PGS000649	EFO_0000095	chronic lymphocytic leukemia	chronic lymphocytic leukemia	EFO_0000095	Immune system disorder	Immunological
0.9715	1.07E-286	1.49E-282	PGS000647	EFO_0000095	chronic lymphocytic leukemia	chronic lymphocytic leukemia	EFO_0000095	Immune system disorder	Immunological	PGS000649	EFO_0000095	chronic lymphocytic leukemia	chronic lymphocytic leukemia	EFO_0000095	Cancer	Neoplasms
0.9715	1.07E-286	1.49E-282	PGS000647	EFO_0000095	chronic lymphocytic leukemia	chronic lymphocytic leukemia	EFO_0000095	Immune system disorder	Immunological	PGS000649	EFO_0000095	chronic lymphocytic leukemia	chronic lymphocytic leukemia	EFO_0000095	Immune system disorder	Immunological
0.9715	1.77E-286	2.47E-282	PGS002721	EFO_0004631	vitamin D measurement	vitamin D measurement	EFO_0004631	Lipid or lipoprotein measurement	Metabolic	PGS002355	EFO_0004631	vitamin D measurement	vitamin D measurement	EFO_0004631	Lipid or lipoprotein measurement	Metabolic
0.9715	1.98E-286	2.75E-282	PGS002173	EFO_0009270	heel bone mineral density	heel bone mineral density	EFO_0009270	Other measurement	Skeletal	PGS002175	EFO_0005654	velocity of sound measurement	velocity of sound measurement	EFO_0005654	Other measurement	Others
0.9714	3.23E-286	4.47E-282	PGS003031	EFO_0004611	low density lipoprotein cholesterol measurement	low density lipoprotein cholesterol measurement	EFO_0004611	Cardiovascular measurement	Cardiovascular	PGS003036	EFO_0004611	low density lipoprotein cholesterol measurement	measurement	EFO_0004611	Lipid or lipoprotein measurement	Metabolic
0.9714	3.23E-286	4.47E-282	PGS003031	EFO_0004611	low density lipoprotein cholesterol measurement	low density lipoprotein cholesterol measurement	EFO_0004611	Lipid or lipoprotein measurement	Metabolic	PGS003036	EFO_0004611	low density lipoprotein cholesterol measurement	measurement	EFO_0004611	Lipid or lipoprotein measurement	Metabolic
0.9714	3.23E-286	4.47E-282	PGS003031	EFO_0004611	low density lipoprotein cholesterol measurement	low density lipoprotein cholesterol measurement	EFO_0004611	Lipid or lipoprotein measurement	Metabolic	PGS003036	EFO_0004611	low density lipoprotein cholesterol measurement	measurement	EFO_0004611	Cardiovascular measurement	Cardiovascular
0.9714	3.23E-286	4.47E-282	PGS003031	EFO_0004611	low density lipoprotein cholesterol measurement	low density lipoprotein cholesterol measurement	EFO_0004611	Cardiovascular measurement	Cardiovascular	PGS003036	EFO_0004611	low density lipoprotein cholesterol measurement	measurement	EFO_0004611	Cardiovascular measurement	Cardiovascular
0.9713	8.11E-286	1.12E-281	PGS003186	EFO_0004631	vitamin D measurement	vitamin D measurement	EFO_0004631	Lipid or lipoprotein measurement	Metabolic	PGS003200	EFO_0004631	vitamin D measurement	vitamin D measurement	EFO_0004631	Lipid or lipoprotein measurement	Metabolic
0.9712	1.90E-285	2.62E-281	PGS002608	EFO_0004584	mean platelet volume	mean platelet volume	EFO_0004584	Hematological measurement	Hematological	PGS002412	EFO_0004584	mean platelet volume	mean platelet volume	EFO_0004584	Hematological measurement	Hematological
0.9711	2.36E-285	3.24E-281	PGS000697	EFO_0004570	bilirubin measurement	bilirubin measurement	EFO_0004570	Other measurement	Metabolic	PGS002160	EFO_0004570	bilirubin measurement	bilirubin measurement	EFO_0004570	Other measurement	Metabolic
0.9711	3.42E-285	4.67E-281	PGS000924	MONDO_0037821	disease	porphyrin metabolism disease	MONDO_0037821	Metabolic disorder	Metabolic	PGS002032	Orphanet_309816	Disorder of bilirubin metabolism and excretion	excretion	Orphanet_309816	Metabolic disorder	Metabolic
0.971	6.79E-285	9.26E-281	PGS002568	EFO_0006335	systolic blood pressure	systolic blood pressure	EFO_0006335	Other measurement	Cardiovascular	PGS002617	EFO_0006335	systolic blood pressure	systolic blood pressure	EFO_0006335	Other measurement	Cardiovascular
0.971	8.77E-285	1.19E-280	PGS002948	EFO_0004465	fasting blood glucose measurement	fasting blood glucose measurement	EFO_0004465	Other measurement	Metabolic	PGS002928	EFO_0004465	fasting blood glucose measurement	fasting blood glucose measurement	EFO_0004465	Other measurement	Metabolic
0.9707	6.62E-284	8.97E-280	PGS000929	MONDO_0001627	dementia	dementia	MONDO_0001627	Neurological disorder	Neurological	PGS001828	MONDO_0004975	Alzheimer disease	Alzheimer disease	MONDO_0004975	Neurological disorder	Neurological
0.9706	1.01E-283	1.37E-279	PGS003228	EFO_0005208	glomerular filtration rate	glomerular filtration rate	EFO_0005208	Other measurement	Genitourinary	PGS002757	EFO_0003884	chronic kidney disease	chronic kidney disease	EFO_0003884	Other disease	Metabolic
0.9706	1.63E-283	2.20E-279	PGS001181	EFO_0004705	hypothyroidism	hypothyroidism	EFO_0004705	Other disease	Endocrine	PGS000965	EFO_1001055	myxedema|hypothyroidism	myxedema	EFO_1001055	Other disease	Endocrine
0.9705	4.16E-283	5.60E-279	PGS002240	EFO_0001663	prostate carcinoma	prostate carcinoma	EFO_0001663	Cancer	Neoplasms	PGS000584	EFO_0001663	prostate carcinoma	prostate carcinoma	EFO_0001663	Cancer	Neoplasms
0.9704	5.04E-283	6.76E-279	PGS002253	EFO_0000756	melanoma	melanoma	EFO_0000756	Cancer	Neoplasms	PGS000813	EFO_0000756	melanoma	melanoma	EFO_0000756	Cancer	Neoplasms
0.9704	6.86E-283	9.17E-279	PGS001267	EFO_0003898	ankylosing spondylitis	ankylosing spondylitis	EFO_0003898	Immune system disorder	Immunological	PGS000324	EFO_0009732	enthesitis-related juvenile idiopathic arthritis	arthritis	EFO_0009732	Immune system disorder	Immunological
0.9703	1.44E-282	1.92E-278	PGS002854	EFO_0004340	body mass index	body mass index	EFO_0004340	Body measurement	Body measurement	PGS002849	EFO_0004340	body mass index	body mass index	EFO_0004340	Body measurement	Body measurement
0.9703	1.51E-282	2.01E-278	PGS002542	HP_0000964	Eczema	Eczema	HP_0000964	Other trait	Dermatological	PGS002591	HP_0000964	Eczema	Eczema	HP_0000964	Other trait	Dermatological
0.97	1.92E-281	2.55E-277	PGS002972	EFO_0004339	body height	body height	EFO_0004339	Body measurement	Body measurement	PGS002967	EFO_0004339	body height	body height	EFO_0004339	Body measurement	Body measurement
0.9699	3.51E-281	4.64E-277	PGS001340	EFO_0000275	atrial fibrillation	atrial fibrillation	EFO_0000275	Cardiovascular disease	Cardiovascular	PGS001263	EFO_0003911	atrial flutter	atrial flutter	EFO_0003911	Cardiovascular disease	Cardiovascular
0.9696	2.96E-280	3.91E-276	PGS000912	EFO_0006336	diastolic blood pressure	diastolic blood pressure	EFO_0006336	Other measurement	Cardiovascular	PGS002893	EFO_0006336	diastolic blood pressure	diastolic blood pressure	EFO_0006336	Other measurement	Cardiovascular
0.9694	9.07E-280	1.19E-275	PGS001179	EFO_0004718	vascular dementia	vascular dementia	EFO_0004718	Neurological disorder	Neurological	PGS002035	MONDO_0001627	dementia	dementia	MONDO_0001627	Neurological disorder	Neurological
0.9694	9.07E-280	1.19E-275	PGS001179	EFO_0004718	vascular dementia	vascular dementia	EFO_0004718	Cardiovascular disease	Cardiovascular	PGS002035	MONDO_0001627	dementia	dementia	MONDO_0001627	Neurological disorder	Neurological
0.9694	1.20E-279	1.57E-275	PGS000333	EFO_0001663	prostate carcinoma	prostate carcinoma	EFO_0001663	Cancer	Neoplasms	PGS002241	EFO_0001663	prostate carcinoma	prostate carcinoma	EFO_0001663	Cancer	Neoplasms
0.9694	1.27E-279	1.67E-275	PGS002541	EFO_0006336	diastolic blood pressure	diastolic blood pressure	EFO_0006336	Other measurement	Cardiovascular	PGS002590	EFO_0006336	diastolic blood pressure	diastolic blood pressure	EFO_0006336	Other measurement	Cardiovascular
0.9694	1.29E-279	1.68E-275	PGS002403	EFO_0004541	HbA1c measurement	HbA1c measurement	EFO_0004541	Hematological measurement	Hematological	PGS002452	EFO_0004541	HbA1c measurement	HbA1c measurement	EFO_0004541	Hematological measurement	Hematological
0.9693	2.33E-279	3.03E-275	PGS002551	EFO_0004339	body height	body height	EFO_0004339	Body measurement	Body measurement	PGS002404	EFO_0004339	body height	body height	EFO_0004339	Body measurement	Body measurement
0.9692	4.12E-279	5.34E-275	PGS002157	EFO_0004533	alkaline phosphatase measurement	alkaline phosphatase measurement	EFO_0004533	Liver enzyme measurement	Gastrointestinal	PGS001939	EFO_0004533	alkaline phosphatase measurement	alkaline phosphatase measurement	EFO_0004533	Liver enzyme measurement	Gastrointestinal
0.969	2.02E-278	2.61E-274	PGS000946	HP_0000726	Dementia	Dementia	HP_0000726	Other trait	Psychiatric	PGS002035	MONDO_0001627	dementia	dementia	MONDO_0001627	Neurological disorder	Neurological
0.9689	3.20E-278	4.12E-274	PGS002554	EFO_0000537	hypertension	hypertension	EFO_0000537	Cardiovascular disease	Cardiovascular	PGS002603	EFO_0000537	hypertension	hypertension	EFO_0000537	Cardiovascular disease	Cardiovascular
0.9686	2.76E-277	3.54E-273	PGS000521	EFO_0000305	breast carcinoma	breast carcinoma	EFO_0000305	Cancer	Neoplasms	PGS000518	EFO_0000305	breast carcinoma	breast carcinoma	EFO_0000305	Cancer	Neoplasms
0.9686	2.76E-277	3.54E-273	PGS000517	EFO_0000305	breast carcinoma	breast carcinoma	EFO_0000305	Cancer	Neoplasms	PGS000521	EFO_0000305	breast carcinoma	breast carcinoma	EFO_0000305	Cancer	Neoplasms
0.9686	3.85E-277	4.92E-273	PGS003201	EFO_0004631	vitamin D measurement	vitamin D measurement	EFO_0004631	Lipid or lipoprotein measurement	Metabolic	PGS003185	EFO_0004631	vitamin D measurement	vitamin D measurement	EFO_0004631	Lipid or lipoprotein measurement	Metabolic
0.9686	3.92E-277	5.00E-273	PGS003270	EFO_0010472	creatine measurement|glomerular filtration rate	creatine measurement	EFO_0010472	Other measurement	Metabolic	PGS003255	EFO_0010472	creatine measurement|glomerular filtration rate	creatine measurement	EFO_0010472	Other measurement	Metabolic
0.9685	6.69E-277	8.52E-273	PGS002393	EFO_0000400	diabetes mellitus	diabetes mellitus	EFO_0000400	Digestive system disorder	Gastrointestinal	PGS002475	MONDO_0005148	type 2 diabetes mellitus	type 2 diabetes mellitus	MONDO_0005148	Digestive system disorder	Gastrointestinal
0.9685	6.69E-277	8.52E-273	PGS002393	EFO_0000400	diabetes mellitus	diabetes mellitus	EFO_0000400	Metabolic disorder	Metabolic	PGS002475	MONDO_0005148	type 2 diabetes mellitus	type 2 diabetes mellitus	MONDO_0005148	Metabolic disorder	Metabolic
0.9685	6.69E-277	8.52E-273	PGS002393	EFO_0000400	diabetes mellitus	diabetes mellitus	EFO_0000400	Metabolic disorder	Metabolic	PGS002475	MONDO_0005148	type 2 diabetes mellitus	type 2 diabetes mellitus	MONDO_0005148	Digestive system disorder	Gastrointestinal
0.9685	6.69E-277	8.52E-273	PGS002393	EFO_0000400	diabetes mellitus	diabetes mellitus	EFO_0000400	Digestive system disorder	Gastrointestinal	PGS002475	MONDO_0005148	type 2 diabetes mellitus	type 2 diabetes mellitus	MONDO_0005148	Metabolic disorder	Metabolic
0.9685	7.01E-277	8.83E-273	PGS003021	EFO_0000537	hypertension	hypertension	EFO_0000537	Cardiovascular disease	Cardiovascular	PGS003025	EFO_0000537	hypertension	hypertension	EFO_0000537	Cardiovascular disease	Cardiovascular
0.9685	7.01E-277	8.83E-273	PGS003020	EFO_0000537	hypertension	hypertension	EFO_0000537	Cardiovascular disease	Cardiovascular	PGS003026	EFO_0000537	hypertension	hypertension	EFO_0000537	Cardiovascular disease	Cardiovascular
0.9685	7.01E-277	8.83E-273	PGS003021	EFO_0000537	hypertension	hypertension	EFO_0000537	Cardiovascular disease	Cardiovascular	PGS003026	EFO_0000537	hypertension	hypertension	EFO_0000537	Cardiovascular disease	Cardiovascular
0.9685	7.01E-277	8.83E-273	PGS003020	EFO_0000537	hypertension	hypertension	EFO_0000537	Cardiovascular disease	Cardiovascular	PGS003025	EFO_0000537	hypertension	hypertension	EFO_0000537	Cardiovascular disease	Cardiovascular
0.9684	1.37E-276	1.73E-272	PGS002397	EFO_0004842	eosinophil count	eosinophil count	EFO_0004842	Hematological measurement	Hematological	PGS002544	EFO_0004842	eosinophil count	eosinophil count	EFO_0004842	Hematological measurement	Hematological
0.9684	1.48E-276	1.85E-272	PGS001349	MONDO_0004975	Alzheimer disease	Alzheimer disease	MONDO_0004975	Neurological disorder	Neurological	PGS001347	EFO_0009268	family history of Alzheimer's disease	family history of Alzheimer's disease	EFO_0009268	Other measurement	Neurological
0.9683	4.13E-276	5.17E-272	PGS001955	EFO_0009270	heel bone mineral density	heel bone mineral density	EFO_0009270	Other measurement	Skeletal	PGS001957	EFO_0005654	velocity of sound measurement	velocity of sound measurement	EFO_0005654	Other measurement	Others
0.9681	1.40E-275	1.74E-271	PGS000585	EFO_0001663	prostate carcinoma	prostate carcinoma	EFO_0001663	Cancer	Neoplasms	PGS000586	EFO_0001663	prostate carcinoma	prostate carcinoma	EFO_0001663	Cancer	Neoplasms
0.968	1.89E-275	2.35E-271	PGS003063	EFO_0006527	smoking status measurement	smoking status measurement	EFO_0006527	Other measurement	Psychiatric	PGS003068	EFO_0006527	smoking status measurement	smoking status measurement	EFO_0006527	Other measurement	Psychiatric
0.968	2.55E-275	3.17E-271	PGS000792	EFO_0005577	pharynx cancer|oral cavity cancer	pharynx cancer	EFO_0005577	Digestive system disorder	Gastrointestinal	PGS000081	EFO_0005570	oral cavity cancer|pharynx cancer	oral cavity cancer	EFO_0005570	Digestive system disorder	Gastrointestinal
0.968	2.55E-275	3.17E-271	PGS000792	EFO_0005577	pharynx cancer|oral cavity cancer	pharynx cancer	EFO_0005577	Cancer	Neoplasms	PGS000081	EFO_0005570	oral cavity cancer|pharynx cancer	oral cavity cancer	EFO_0005570	Cancer	Neoplasms
0.968	2.55E-275	3.17E-271	PGS000792	EFO_0005577	pharynx cancer|oral cavity cancer	pharynx cancer	EFO_0005577	Cancer	Neoplasms	PGS000081	EFO_0005570	oral cavity cancer|pharynx cancer	oral cavity cancer	EFO_0005570	Digestive system disorder	Gastrointestinal
0.968	2.55E-275	3.17E-271	PGS000792	EFO_0005577	pharynx cancer|oral cavity cancer	pharynx cancer	EFO_0005577	Digestive system disorder	Gastrointestinal	PGS000081	EFO_0005570	oral cavity cancer|pharynx cancer	oral cavity cancer	EFO_0005570	Cancer	Neoplasms
0.9678	8.75E-275	1.08E-270	PGS000789	EFO_0001071	lung carcinoma	lung carcinoma	EFO_0001071	Cancer	Neoplasms	PGS000078	EFO_0001071	lung carcinoma	lung carcinoma	EFO_0001071	Cancer	Neoplasms
0.9678	1.01E-274	1.25E-270	PGS002008	EFO_0004503	hematological measurement	hematological measurement	EFO_0004503	Hematological measurement	Hematological	PGS002227	EFO_0004503	hematological measurement	hematological measurement	EFO_0004503	Hematological measurement	Hematological
0.9677	2.06E-274	2.53E-270	PGS003078	EFO_0006335	systolic blood pressure	systolic blood pressure	EFO_0006335	Other measurement	Cardiovascular	PGS000913	EFO_0006335	systolic blood pressure	systolic blood pressure	EFO_0006335	Other measurement	Cardiovascular
0.9677	2.18E-274	2.67E-270	PGS002426	MONDO_0005148	type 2 diabetes mellitus	type 2 diabetes mellitus	MONDO_0005148	Metabolic disorder	Metabolic	PGS002442	EFO_0000400	diabetes mellitus	diabetes mellitus	EFO_0000400	Metabolic disorder	Metabolic
0.9677	2.18E-274	2.67E-270	PGS002426	MONDO_0005148	type 2 diabetes mellitus	type 2 diabetes mellitus	MONDO_0005148	Digestive system disorder	Gastrointestinal	PGS002442	EFO_0000400	diabetes mellitus	diabetes mellitus	EFO_0000400	Digestive system disorder	Gastrointestinal
0.9677	2.18E-274	2.67E-270	PGS002426	MONDO_0005148	type 2 diabetes mellitus	type 2 diabetes mellitus	MONDO_0005148	Digestive system disorder	Gastrointestinal	PGS002442	EFO_0000400	diabetes mellitus	diabetes mellitus	EFO_0000400	Metabolic disorder	Metabolic

0.9677	2.18E-274	2.67E-270	PGS002426	MONDO_0005148	type 2 diabetes mellitus	type 2 diabetes mellitus	MONDO_0005148	Metabolic disorder	Metabolic	PGS002442	EFO_0000400	diabetes mellitus	diabetes mellitus	EFO_0000400	Digestive system disorder	Gastrointestinal
0.9676	3.24E-274	3.97E-270	PGS000324	EFO_0009732	enthesitis-related juvenile idiopathic arthritis	enthesitis-related juvenile idiopathic arthritis	EFO_0009732	Immune system disorder	Immunological	PGS001268	EFO_0003898	ankylosing spondylitis	ankylosing spondylitis	EFO_0003898	Immune system disorder	Immunological
0.9674	1.67E-273	2.04E-269	PGS002035	MONDO_0001627	dementia	dementia	MONDO_0001627	Neurological disorder	Neurological	PGS001348	MONDO_0004975	Alzheimer disease	Alzheimer disease	MONDO_0004975	Neurological disorder	Neurological
0.9673	2.89E-273	3.52E-269	PGS002035	MONDO_0001627	dementia	dementia	MONDO_0001627	Neurological disorder	Neurological	PGS001827	MONDO_0001627	dementia	dementia	MONDO_0001627	Neurological disorder	Neurological
0.9672	4.55E-273	5.53E-269	PGS000743	EFO_0000756	melanoma	melanoma	EFO_0000756	Cancer	Neoplasms	PGS000813	EFO_0000756	melanoma	melanoma	EFO_0000756	Cancer	Neoplasms
0.9671	1.34E-272	1.63E-268	PGS000136	MONDO_0005090	schizophrenia	schizophrenia	MONDO_0005090	Neurological disorder	Neurological	PGS000135	MONDO_0005090	schizophrenia	schizophrenia	MONDO_0005090	Neurological disorder	Neurological
0.9671	1.62E-272	1.96E-268	PGS003148	EFO_0004530	triglyceride measurement	triglyceride measurement	EFO_0004530	Lipid or lipoprotein measurement	Metabolic	PGS003153	EFO_0004530	triglyceride measurement	triglyceride measurement	EFO_0004530	Lipid or lipoprotein measurement	Metabolic
0.967	1.76E-272	2.12E-268	PGS002835	EFO_0007878	alcohol consumption measurement	alcohol consumption measurement	EFO_0007878	Other measurement	Psychiatric	PGS002830	EFO_0007878	alcohol consumption measurement	alcohol consumption measurement	EFO_0007878	Other measurement	Psychiatric
0.967	3.19E-272	3.83E-268	PGS001234	EFO_0004324	body weights and measures	body weights and measures	EFO_0004324	Body measurement	Body measurement	PGS001235	EFO_0004324	body weights and measures	body weights and measures	EFO_0004324	Body measurement	Body measurement
0.9669	5.39E-272	6.47E-268	PGS002170	EFO_0004468	glucose measurement	glucose measurement	EFO_0004468	Other measurement	Metabolic	PGS001952	EFO_0004468	glucose measurement	glucose measurement	EFO_0004468	Other measurement	Metabolic
0.9668	8.47E-272	1.01E-267	PGS002562	EFO_0004309	platelet count	platelet count	EFO_0004309	Hematological measurement	Hematological	PGS002415	EFO_0004309	platelet count	platelet count	EFO_0004309	Hematological measurement	Hematological
0.9666	3.07E-271	3.67E-267	PGS003009	EFO_0000537	hypertension	hypertension	EFO_0000537	Cardiovascular disease	Cardiovascular	PGS003004	EFO_0000537	hypertension	hypertension	EFO_0000537	Cardiovascular disease	Cardiovascular
0.9665	5.68E-271	6.76E-267	PGS001146	EFO_0005409	fat body mass	fat body mass	EFO_0005409	Other measurement	Body measurement	PGS001234	EFO_0004324	body weights and measures	body weights and measures	EFO_0004324	Body measurement	Body measurement
0.9664	1.06E-270	1.25E-266	PGS003273	EFO_0010472	creatine measurement|glomerular filtration rate	creatine measurement	EFO_0010472	Other measurement	Metabolic	PGS003258	EFO_0010472	creatine measurement|glomerular filtration rate	creatine measurement	EFO_0010472	Other measurement	Metabolic
0.9663	3.10E-270	3.67E-266	PGS002405	EFO_0007986	reticulocyte count	reticulocyte count	EFO_0007986	Hematological measurement	Hematological	PGS002552	EFO_0007986	reticulocyte count	reticulocyte count	EFO_0007986	Hematological measurement	Hematological
0.9662	4.03E-270	4.76E-266	PGS000681	EFO_0004570	bilirubin measurement	bilirubin measurement	EFO_0004570	Other measurement	Metabolic	PGS002160	EFO_0004570	bilirubin measurement	bilirubin measurement	EFO_0004570	Other measurement	Metabolic
0.9656	1.93E-268	2.28E-264	PGS002592	EFO_0001379	endocrine system disease|diabetes mellitus	endocrine system disease	EFO_0001379	Other disease	Metabolic	PGS002396	EFO_0001379	endocrine system disease|diabetes mellitus	endocrine system disease	EFO_0001379	Other disease	Metabolic
0.9655	3.69E-268	4.34E-264	PGS003222	EFO_0005208	glomerular filtration rate	glomerular filtration rate	EFO_0005208	Other measurement	Genitourinary	PGS003227	EFO_0005208	glomerular filtration rate	glomerular filtration rate	EFO_0005208	Other measurement	Genitourinary
0.9655	4.01E-268	4.70E-264	PGS001103	EFO_0007800	body fat percentage	body fat percentage	EFO_0007800	Body measurement	Body measurement	PGS001104	EFO_0007800	body fat percentage	body fat percentage	EFO_0007800	Body measurement	Body measurement
0.9655	4.04E-268	4.73E-264	PGS001942	EFO_0004570	bilirubin measurement	bilirubin measurement	EFO_0004570	Other measurement	Metabolic	PGS000697	EFO_0004570	bilirubin measurement	bilirubin measurement	EFO_0004570	Other measurement	Metabolic
0.9652	2.62E-267	3.06E-263	PGS002056	EFO_1001395	Phlebitis|Thrombophlebitis	Phlebitis	EFO_1001395	Cardiovascular disease	Cardiovascular	PGS001844	EFO_1001395	Phlebitis|Thrombophlebitis	Phlebitis	EFO_1001395	Cardiovascular disease	Cardiovascular
0.9651	6.62E-267	7.71E-263	PGS001169	EFO_0004995	lean body mass	lean body mass	EFO_0004995	Other measurement	Skeletal	PGS001168	EFO_0004995	lean body mass	lean body mass	EFO_0004995	Other measurement	Skeletal
0.9651	7.05E-267	8.19E-263	PGS002971	EFO_0004339	body height	body height	EFO_0004339	Body measurement	Body measurement	PGS002966	EFO_0004339	body height	body height	EFO_0004339	Body measurement	Body measurement
0.9649	2.50E-266	2.90E-262	PGS001908	EFO_0005192	red blood cell distribution width	red blood cell distribution width	EFO_0005192	Hematological measurement	Hematological	PGS002122	EFO_0005192	red blood cell distribution width	red blood cell distribution width	EFO_0005192	Hematological measurement	Hematological
0.9647	8.08E-266	9.33E-262	PGS000929	MONDO_0001627	dementia	dementia	MONDO_0001627	Neurological disorder	Neurological	PGS001179	EFO_0004718	vascular dementia	vascular dementia	EFO_0004718	Cardiovascular disease	Cardiovascular
0.9647	8.08E-266	9.33E-262	PGS000929	MONDO_0001627	dementia	dementia	MONDO_0001627	Neurological disorder	Neurological	PGS001179	EFO_0004718	vascular dementia	vascular dementia	EFO_0004718	Neurological disorder	Neurological
0.9647	8.22E-266	9.48E-262	PGS002206	EFO_0004527	mean corpuscular hemoglobin	mean corpuscular hemoglobin	EFO_0004527	Hematological measurement	Hematological	PGS001989	EFO_0004527	mean corpuscular hemoglobin	mean corpuscular hemoglobin	EFO_0004527	Hematological measurement	Hematological
0.9647	1.02E-265	1.18E-261	PGS002471	EFO_0004279	suntan	suntan	EFO_0004279	Other trait	Dermatological	PGS002422	EFO_0004279	suntan	suntan	EFO_0004279	Other trait	Dermatological
0.9646	1.29E-265	1.47E-261	PGS000632	EFO_0002892	thyroid carcinoma	thyroid carcinoma	EFO_0002892	Cancer	Neoplasms	PGS000631	EFO_0002892	thyroid carcinoma	thyroid carcinoma	EFO_0002892	Cancer	Neoplasms
0.9646	1.29E-265	1.47E-261	PGS000630	EFO_0002892	thyroid carcinoma	thyroid carcinoma	EFO_0002892	Cancer	Neoplasms	PGS000632	EFO_0002892	thyroid carcinoma	thyroid carcinoma	EFO_0002892	Cancer	Neoplasms
0.9645	3.08E-265	3.52E-261	PGS001149	EFO_0005409	fat body mass	fat body mass	EFO_0005409	Other measurement	Body measurement	PGS001148	EFO_0005409	fat body mass	fat body mass	EFO_0005409	Other measurement	Body measurement
0.9645	3.94E-265	4.49E-261	PGS000929	MONDO_0001627	dementia	dementia	MONDO_0001627	Neurological disorder	Neurological	PGS002035	MONDO_0001627	dementia	dementia	MONDO_0001627	Neurological disorder	Neurological
0.9644	4.74E-265	5.38E-261	PGS001160	EFO_0005106	body composition measurement	body composition measurement	EFO_0005106	Body measurement	Body measurement	PGS001169	EFO_0004995	lean body mass	lean body mass	EFO_0004995	Other measurement	Skeletal
0.9642	1.52E-264	1.73E-260	PGS002121	EFO_0004831	RR interval	RR interval	EFO_0004831	Cardiovascular measurement	Cardiovascular	PGS002117	EFO_0600085	PP interval	PP interval	EFO_0600085	Cardiovascular measurement	Cardiovascular
0.9641	4.13E-264	4.67E-260	PGS001023	EFO_0009556	mineral metabolism disease	mineral metabolism disease	EFO_0009556	Metabolic disorder	Metabolic	PGS002031	MONDO_0002279	iron metabolism disease	iron metabolism disease	MONDO_0002279	Metabolic disorder	Metabolic
0.964	8.48E-264	9.57E-260	PGS002543	EFO_0001379	endocrine system disease|diabetes mellitus	endocrine system disease	EFO_0001379	Other disease	Metabolic	PGS002622	MONDO_0005148	type 2 diabetes mellitus	type 2 diabetes mellitus	MONDO_0005148	Metabolic disorder	Metabolic
0.964	8.48E-264	9.57E-260	PGS002543	EFO_0001379	endocrine system disease|diabetes mellitus	endocrine system disease	EFO_0001379	Other disease	Metabolic	PGS002622	MONDO_0005148	type 2 diabetes mellitus	type 2 diabetes mellitus	MONDO_0005148	Digestive system disorder	Gastrointestinal
0.9639	1.16E-263	1.31E-259	PGS002171	EFO_0004541	HbA1c measurement	HbA1c measurement	EFO_0004541	Hematological measurement	Hematological	PGS001953	EFO_0004541	HbA1c measurement	HbA1c measurement	EFO_0004541	Hematological measurement	Hematological
0.9639	1.32E-263	1.48E-259	PGS000632	EFO_0002892	thyroid carcinoma	thyroid carcinoma	EFO_0002892	Cancer	Neoplasms	PGS000209	EFO_0002892	thyroid carcinoma	thyroid carcinoma	EFO_0002892	Cancer	Neoplasms
0.9637	5.85E-263	6.56E-259	PGS001041	EFO_0009259	skin carcinoma	skin carcinoma	EFO_0009259	Cancer	Neoplasms	PGS001040	EFO_0009260	non-melanoma skin carcinoma	non-melanoma skin carcinoma	EFO_0009260	Cancer	Neoplasms
0.9635	1.15E-262	1.29E-258	PGS000467	EFO_0000707	squamous cell carcinoma	squamous cell carcinoma	EFO_0000707	Cancer	Neoplasms	PGS000468	EFO_0000707	squamous cell carcinoma	squamous cell carcinoma	EFO_0000707	Cancer	Neoplasms
0.9635	1.47E-262	1.64E-258	PGS003226	EFO_0005208	glomerular filtration rate	glomerular filtration rate	EFO_0005208	Other measurement	Genitourinary	PGS003221	EFO_0005208	glomerular filtration rate	glomerular filtration rate	EFO_0005208	Other measurement	Genitourinary
0.9635	1.93E-262	2.14E-258	PGS001168	EFO_0004995	lean body mass	lean body mass	EFO_0004995	Other measurement	Skeletal	PGS001002	EFO_0009805	whole body water mass	whole body water mass	EFO_0009805	Other measurement	Body measurement
0.9634	3.24E-262	3.60E-258	PGS002107	EFO_0001060	celiac disease	celiac disease	EFO_0001060	Digestive system disorder	Gastrointestinal	PGS001894	EFO_0001060	celiac disease	celiac disease	EFO_0001060	Immune system disorder	Immunological
0.9634	3.24E-262	3.60E-258	PGS002107	EFO_0001060	celiac disease	celiac disease	EFO_0001060	Immune system disorder	Immunological	PGS001894	EFO_0001060	celiac disease	celiac disease	EFO_0001060	Immune system disorder	Immunological
0.9634	3.24E-262	3.60E-258	PGS002107	EFO_0001060	celiac disease	celiac disease	EFO_0001060	Immune system disorder	Immunological	PGS001894	EFO_0001060	celiac disease	celiac disease	EFO_0001060	Digestive system disorder	Gastrointestinal
0.9634	3.24E-262	3.60E-258	PGS002107	EFO_0001060	celiac disease	celiac disease	EFO_0001060	Digestive system disorder	Gastrointestinal	PGS001894	EFO_0001060	celiac disease	celiac disease	EFO_0001060	Digestive system disorder	Gastrointestinal
0.9634	3.59E-262	3.97E-258	PGS002453	EFO_0004339	body height	body height	EFO_0004339	Body measurement	Body measurement	PGS002404	EFO_0004339	body height	body height	EFO_0004339	Body measurement	Body measurement
0.9629	7.59E-261	8.39E-257	PGS003173	EFO_0004620	vitamin B12 measurement	vitamin B12 measurement	EFO_0004620	Other measurement	Metabolic	PGS003168	EFO_0004620	vitamin B12 measurement	vitamin B12 measurement	EFO_0004620	Other measurement	Metabolic
0.9628	1.16E-260	1.28E-256	PGS001261	EFO_0003913	angina pectoris	angina pectoris	EFO_0003913	Other trait	Cardiovascular	PGS001260	EFO_0003913	angina pectoris	angina pectoris	EFO_0003913	Other trait	Cardiovascular
0.9628	1.29E-260	1.41E-256	PGS001160	EFO_0005106	body composition measurement	body composition measurement	EFO_0005106	Body measurement	Body measurement	PGS001002	EFO_0009805	whole body water mass	whole body water mass	EFO_0009805	Other measurement	Body measurement
0.9628	1.37E-260	1.51E-256	PGS000929	MONDO_0001627	dementia	dementia	MONDO_0001627	Neurological disorder	Neurological	PGS000946	HP_0000726	Dementia	Dementia	HP_0000726	Other trait	Psychiatric
0.9627	1.86E-260	2.03E-256	PGS003220	EFO_0005208	glomerular filtration rate	glomerular filtration rate	EFO_0005208	Other measurement	Genitourinary	PGS003225	EFO_0005208	glomerular filtration rate	glomerular filtration rate	EFO_0005208	Other measurement	Genitourinary
0.9626	3.70E-260	4.04E-256	PGS002623	EFO_0004631	vitamin D measurement	vitamin D measurement	EFO_0004631	Lipid or lipoprotein measurement	Metabolic	PGS002427	EFO_0004631	vitamin D measurement	vitamin D measurement	EFO_0004631	Lipid or lipoprotein measurement	Metabolic
0.9626	4.38E-260	4.76E-256	PGS000533	EFO_0000305	breast carcinoma	breast carcinoma	EFO_0000305	Cancer	Neoplasms	PGS000538	EFO_0000305	breast carcinoma	breast carcinoma	EFO_0000305	Cancer	Neoplasms
0.9626	4.38E-260	4.76E-256	PGS000534	EFO_0000305	breast carcinoma	breast carcinoma	EFO_0000305	Cancer	Neoplasms	PGS000538	EFO_0000305	breast carcinoma	breast carcinoma	EFO_0000305	Cancer	Neoplasms
0.9624	9.97E-260	1.08E-255	PGS001942	EFO_0004570	bilirubin measurement	bilirubin measurement	EFO_0004570	Other measurement	Metabolic	PGS000681	EFO_0004570	bilirubin measurement	bilirubin measurement	EFO_0004570	Other measurement	Metabolic
0.9621	7.36E-259	7.96E-255	PGS001971	EFO_0004584	mean platelet volume	mean platelet volume	EFO_0004584	Hematological measurement	Hematological	PGS002189	EFO_0004584	mean platelet volume	mean platelet volume	EFO_0004584	Hematological measurement	Hematological
0.9621	7.87E-259	8.50E-255	PGS002102	EFO_0004615	apolipoprotein B measurement	apolipoprotein B measurement	EFO_0004615	Lipid or lipoprotein measurement	Metabolic	PGS001889	EFO_0004615	apolipoprotein B measurement	apolipoprotein B measurement	EFO_0004615	Lipid or lipoprotein measurement	Metabolic
0.9621	7.97E-259	8.59E-255	PGS001896	EFO_0007822	hair colour measurement	hair colour measurement	EFO_0007822	Other measurement	Dermatological	PGS002109	EFO_0007822	hair colour measurement	hair colour measurement	EFO_0007822	Other measurement	Dermatological
0.962	1.32E-258	1.42E-254	PGS000536	EFO_0000305	breast carcinoma	breast carcinoma	EFO_0000305	Cancer	Neoplasms	PGS000535	EFO_0000305	breast carcinoma	breast carcinoma	EFO_0000305	Cancer	Neoplasms
0.9619	1.98E-258	2.12E-254	PGS002578	EFO_0004703	age at menarche	age at menarche	EFO_0004703	Other measurement	Genitourinary	PGS002529	EFO_0004703	age at menarche	age at menarche	EFO_0004703	Other measurement	Genitourinary
0.9618	4.26E-258	4.55E-254	PGS001990	EFO_0004526	mean corpuscular volume	mean corpuscular volume	EFO_0004526	Hematological measurement	Hematological	PGS002207	EFO_0004526	mean corpuscular volume	mean corpuscular volume	EFO_0004526	Hematological measurement	Hematological
0.9617	6.11E-258	6.51E-254	PGS002411	EFO_0004527	mean corpuscular hemoglobin	mean corpuscular hemoglobin	EFO_0004527	Hematological measurement	Hematological	PGS002607	EFO_0004527	mean corpuscular hemoglobin	mean corpuscular hemoglobin	EFO_0004527	Hematological measurement	Hematological
0.9615	2.24E-257	2.39E-253	PGS000541	MONDO_0002715	uterine cancer	uterine cancer	MONDO_0002715	Cancer	Neoplasms	PGS000542	MONDO_0002715	uterine cancer	uterine cancer	MONDO_0002715	Cancer	Neoplasms
0.9615	2.45E-257	2.60E-253	PGS002524	MONDO_0005148	type 2 diabetes mellitus	type 2 diabetes mellitus	MONDO_0005148	Digestive system disorder	Gastrointestinal	PGS002491	EFO_0000400	diabetes mellitus	diabetes mellitus	EFO_0000400	Metabolic disorder	Metabolic
0.9615	2.45E-257	2.60E-253	PGS002524	MONDO_0005148	type 2 diabetes mellitus	type 2 diabetes mellitus	MONDO_0005148	Metabolic disorder	Metabolic	PGS002491	EFO_0000400	diabetes mellitus	diabetes mellitus	EFO_0000400	Metabolic disorder	Metabolic
0.9615	2.45E-257	2.60E-253	PGS002524	MONDO_0005148	type 2 diabetes mellitus	type 2 diabetes mellitus	MONDO_0005148	Metabolic disorder	Metabolic	PGS002491	EFO_0000400	diabetes mellitus	diabetes mellitus	EFO_0000400	Digestive system disorder	Gastrointestinal
0.9615	2.45E-257	2.60E-253	PGS002524	MONDO_0005148	type 2 diabetes mellitus	type 2 diabetes mellitus	MONDO_0005148	Digestive system disorder	Gastrointestinal	PGS002491	EFO_0000400	diabetes mellitus	diabetes mellitus	EFO_0000400	Digestive system disorder	Gastrointestinal
0.9612	1.60E-256	1.69E-252	PGS002543	EFO_0001379	endocrine system disease|diabetes mellitus	endocrine system disease	EFO_0001379	Other disease	Metabolic	PGS002547	EFO_0004468	glucose measurement	glucose measurement	EFO_0004468	Other measurement	Metabolic
0.9611	2.10E-256	2.21E-252	PGS001895	EFO_0004574	total cholesterol measurement	total cholesterol measurement	EFO_0004574	Lipid or lipoprotein measurement	Metabolic	PGS002108	EFO_0004574	total cholesterol measurement	total cholesterol measurement	EFO_0004574	Lipid or lipoprotein measurement	Metabolic
0.961	3.04E-256	3.20E-252	PGS003123	MONDO_0005148	type 2 diabetes mellitus	type 2 diabetes mellitus	MONDO_0005148	Metabolic disorder	Metabolic	PGS003108	MONDO_0005148	type 2 diabetes mellitus	type 2 diabetes mellitus	MONDO_0005148	Digestive system disorder	Gastrointestinal
0.961	3.04E-256	3.20E-252	PGS003123	MONDO_0005148	type 2 diabetes mellitus	type 2 diabetes mellitus	MONDO_0005148	Metabolic disorder	Metabolic	PGS003108	MONDO_0005148	type 2 diabetes mellitus	type 2 diabetes mellitus	MONDO_0005148	Metabolic disorder	Metabolic
0.961	3.04E-256	3.20E-252	PGS003123	MONDO_0005148	type 2 diabetes mellitus	type 2 diabetes mellitus	MONDO_0005148	Digestive system disorder	Gastrointestinal	PGS003108	MONDO_0005148	type 2 diabetes mellitus	type 2 diabetes mellitus	MONDO_0005148	Digestive system disorder	Gastrointestinal
0.961	3.04E-256	3.20E-252	PGS003123	MONDO_0005148	type 2 diabetes mellitus	type 2 diabetes mellitus	MONDO_0005148	Digestive system disorder	Gastrointestinal	PGS003108	MONDO_0005148	type 2 diabetes mellitus	type 2 diabetes mellitus	MONDO_0005148	Metabolic disorder	Metabolic
0.961	3.99E-256	4.17E-252	PGS000611	MONDO_0004986	urinary bladder carcinoma	urinary bladder carcinoma	MONDO_0004986	Cancer	Neoplasms	PGS000613	MONDO_0004986	urinary bladder carcinoma	urinary bladder carcinoma	MONDO_0004986	Cancer	Neoplasms
0.961	3.99E-256	4.17E-252	PGS000613	MONDO_0004986	urinary bladder carcinoma	urinary bladder carcinoma	MONDO_0004986	Cancer	Neoplasms	PGS000612	MONDO_0004986	urinary bladder carcinoma	urinary bladder carcinoma	MONDO_0004986	Cancer	Neoplasms
0.961	3.99E-256	4.17E-252	PGS000613	MONDO_0004986	urinary bladder carcinoma	urinary bladder carcinoma	MONDO_0004986	Cancer	Neoplasms	PGS000610	MONDO_0004986	urinary bladder carcinoma	urinary bladder carcinoma	MONDO_0004986	Cancer	Neoplasms
0.961	4.49E-256	4.69E-252	PGS000883	EFO_0005208	glomerular filtration rate	glomerular filtration rate	EFO_0005208	Other measurement	Genitourinary	PGS003377	EFO_0005208	glomerular filtration rate	glomerular filtration rate	EFO_0005208	Other measurement	Genitourinary
0.9607	2.25E-255	2.34E-251	PGS000823	MONDO_0004975	Alzheimer disease	Alzheimer disease	MONDO_0004975	Neurological disorder	Neurological	PGS000025	MONDO_0004975	Alzheimer disease	Alzheimer disease	MONDO_0004975	Neurological disorder	Neurological
0.9607	2.29E-255	2.38E-251	PGS002393	EFO_0000400	diabetes mellitus	diabetes mellitus	EFO_0000400	Digestive system disorder	Gastrointestinal	PGS002396	EFO_0001379	endocrine system disease|diabetes mellitus	endocrine system disease	EFO_0001379	Other disease	Metabolic
0.9607	2.29E-255	2.38E-251	PGS002393	EFO_0000400	diabetes mellitus	diabetes mellitus	EFO_0000400	Metabolic disorder	Metabolic	PGS002396	EFO_0001379	endocrine system disease|diabetes mellitus	endocrine system disease	EFO_0001379	Other disease	Metabolic
0.9604	9.27E-255	9.62E-251	PGS000697	EFO_0004570	bilirubin measurement	bilirubin measurement	EFO_0004570	Other measurement	Metabolic	PGS001824	Orphanet_309816	Disorder of bilirubin metabolism and excretion	excretion	Orphanet_309816	Metabolic disorder	Metabolic
0.9604	1.57E-254	1.62E-250	PGS000929	MONDO_0001627	dementia	dementia	MONDO_0001627	Neurological disorder	Neurological	PGS000945	HP_0000726	Dementia|Alzheimer disease	Dementia	HP_0000726	Other trait	Psychiatric
0.9603	1.73E-254	1.78E-250	PGS000206	EFO_0008579	risk-taking behaviour	risk-taking behaviour	EFO_0008579	Biological process	Activities	PGS000205	EFO_0008579	risk-taking behaviour	risk-taking behaviour	EFO_0008579	Biological process	Activities
0.9603	2.29E-254	2.35E-250	PGS001105	EFO_0007800	body fat percentage	body fat percentage	EFO_0007800	Body measurement	Body measurement	PGS001101	EFO_0007800	body fat percentage	body fat percentage	EFO_0007800	Body measurement	Body measurement
0.9601	6.58E-254	6.74E-250	PGS002015	EFO_0000305	breast carcinoma	breast carcinoma	EFO_0000305	Cancer	Neoplasms	PGS001804	EFO_0000305	breast carcinoma	breast carcinoma	EFO_0000305	Cancer	Neoplasms
0.96	1.18E-253	1.20E-249	PGS003196	EFO_0004631	vitamin D measurement	vitamin D measurement	EFO_0004631	Lipid or lipoprotein measurement	Metabolic	PGS003195	EFO_0004631	vitamin D measurement	vitamin D measurement	EFO_0004631	Lipid or lipoprotein measurement	Metabolic
0.9599	2.18E-253	2.22E-249	PGS002155	EFO_0004279	suntan	suntan	EFO_0004279	Other trait	Dermatological	PGS001937	EFO_0004279	suntan	suntan	EFO_0004279	Other trait	Dermatological
0.9598	3.80E-253	3.86E-249	PGS002425	EFO_0004530	triglyceride measurement	triglyceride measurement	EFO_0004530	Lipid or lipoprotein measurement	Metabolic	PGS002572	EFO_0004530	triglyceride measurement	triglyceride measurement	EFO_0004530	Lipid or lipoprotein measurement	Metabolic
0.9597	4.62E-253	4.69E-249	PGS003254	EFO_0010472	creatine measurement|glomerular filtration rate	creatine measurement	EFO_0010472	Other measurement	Metabolic	PGS003269	EFO_0010472	creatine measurement|glomerular filtration rate	creatine measurement	EFO_0010472	Other measurement	Metabolic
0.9597	5.27E-253	5.34E-249	PGS002839	EFO_0007878	alcohol consumption measurement	alcohol consumption measurement	EFO_0007878	Other measurement	Psychiatric	PGS000204	EFO_0007878	alcohol consumption measurement	alcohol consumption measurement	EFO_0007878	Other measurement	Psychiatric
0.9597	7.25E-253	7.33E-249	PGS002475	MONDO_0005148	type 2 diabetes mellitus	type 2 diabetes mellitus	MONDO_0005148	Digestive system disorder	Gastrointestinal	PGS002573	MONDO_0005148	type 2 diabetes mellitus	type 2 diabetes mellitus	MONDO_0005148	Digestive system disorder	Gastrointestinal
0.9597	7.25E-253	7.33E-249	PGS002475	MONDO_0005148	type 2 diabetes mellitus	type 2 diabetes mellitus	MONDO_0005148	Digestive system disorder	Gastrointestinal	PGS002573	MONDO_0005148	type 2 diabetes mellitus	type 2 diabetes mellitus	MONDO_0005148	Metabolic disorder	Metabolic
0.9597	7.25E-253	7.33E-249	PGS002475	MONDO_0005148	type 2 diabetes mellitus	type 2 diabetes mellitus	MONDO_0005148	Metabolic disorder	Metabolic	PGS002573	MONDO_0005148	type 2 diabetes mellitus	type 2 diabetes mellitus	MONDO_0005148	Metabolic disorder	Metabolic
0.9597	7.25E-253	7.33E-249	PGS002475	MONDO_0005148	type 2 diabetes mellitus	type 2 diabetes mellitus	MONDO_0005148	Metabolic disorder	Metabolic	PGS002573	MONDO_0005148	type 2 diabetes mellitus	type 2 diabetes mellitus	MONDO_0005148	Digestive system disorder	Gastrointestinal
0.9594	2.89E-252	2.91E-248	PGS000923	MONDO_0019338	sarcoidosis	sarcoidosis	MONDO_0019338	Other disease	Others	PGS000922	MONDO_0019338	sarcoidosis	sarcoidosis	MONDO_0019338	Other disease	Others
0.9592	9.04E-252	9.10E-248	PGS002150	EFO_0004611	low density lipoprotein cholesterol measurement	low density lipoprotein cholesterol measurement	EFO_0004611	Cardiovascular measurement	Cardiovascular	PGS001933	EFO_0004611	low density lipoprotein cholesterol measurement	measurement	EFO_0004611	Lipid or lipoprotein measurement	Metabolic
0.9592	9.04E-252	9.10E-248	PGS002150	EFO_0004611	low density lipoprotein cholesterol measurement	low density lipoprotein cholesterol measurement	EFO_0004611	Lipid or lipoprotein measurement	Metabolic	PGS001933	EFO_0004611	low density lipoprotein cholesterol measurement	measurement	EFO_0004611	Lipid or lipoprotein measurement	Metabolic
0.9592	9.04E-252	9.10E-248	PGS002150	EFO_0004611	low density lipoprotein cholesterol measurement	low density lipoprotein cholesterol measurement	EFO_0004611	Lipid or lipoprotein measurement	Metabolic	PGS001933	EFO_0004611	low density lipoprotein cholesterol measurement	measurement	EFO_0004611	Cardiovascular measurement	Cardiovascular
0.9592	9.04E-252	9.10E-248	PGS002150	EFO_0004611	low density lipoprotein cholesterol measurement	low density lipoprotein cholesterol measurement	EFO_0004611	Cardiovascular measurement	Cardiovascular	PGS001933	EFO_0004611	low density lipoprotein cholesterol measurement	measurement	EFO_0004611	Cardiovascular measurement	Cardiovascular
0.959	3.26E-251	3.27E-247	PGS002930	EFO_0004465	fasting blood glucose measurement	fasting blood glucose measurement	EFO_0004465	Other measurement	Metabolic	PGS002931	EFO_0004465	fasting blood glucose measurement	fasting blood glucose measurement	EFO_0004465	Other measurement	Metabolic
0.959	3.50E-251	3.51E-247	PGS002035	MONDO_0001627	dementia	dementia	MONDO_0001627	Neurological disorder	Neurological	PGS001828	MONDO_0004975	Alzheimer disease	Alzheimer disease	MONDO_0004975	Neurological disorder	Neurological

0.9588	6.99E-251	6.99E-247	PGS001991	EFO_0007989	monocyte percentage of leukocytes	monocyte percentage of leukocytes	EFO_0007989	Hematological measurement	Hematological	PGS002208	EFO_0007989	monocyte percentage of leukocytes	monocyte percentage of leukocytes	EFO_0007989	Hematological measurement	Hematological
0.9587	1.35E-250	1.35E-246	PGS000076	EFO_0000681	renal cell carcinoma	renal cell carcinoma	EFO_0000681	Cancer	Neoplasms	PGS000787	EFO_0000681	renal cell carcinoma	renal cell carcinoma	EFO_0000681	Cancer	Neoplasms
0.9585	4.87E-250	4.85E-246	PGS000632	EFO_0002892	thyroid carcinoma	thyroid carcinoma	EFO_0002892	Cancer	Neoplasms	PGS000208	EFO_0002892	thyroid carcinoma	thyroid carcinoma	EFO_0002892	Cancer	Neoplasms
0.9584	5.94E-250	5.91E-246	PGS000414	EFO_0000756	melanoma	melanoma	EFO_0000756	Cancer	Neoplasms	PGS000412	EFO_0000756	melanoma	melanoma	EFO_0000756	Cancer	Neoplasms
0.9582	1.83E-249	1.82E-245	PGS001820	HP_0001943	Hypoglycemia	Hypoglycemia	HP_0001943	Other trait	Metabolic	PGS002028	HP_0001943	Hypoglycemia	Hypoglycemia	HP_0001943	Other trait	Metabolic
0.958	5.72E-249	5.66E-245	PGS002543	EFO_0001379	endocrine system disease|diabetes mellitus	endocrine system disease	EFO_0001379	Other disease	Metabolic	PGS002573	MONDO_0005148	type 2 diabetes mellitus	type 2 diabetes mellitus	MONDO_0005148	Digestive system disorder	Gastrointestinal
0.958	5.72E-249	5.66E-245	PGS002543	EFO_0001379	endocrine system disease|diabetes mellitus	endocrine system disease	EFO_0001379	Other disease	Metabolic	PGS002573	MONDO_0005148	type 2 diabetes mellitus	type 2 diabetes mellitus	MONDO_0005148	Metabolic disorder	Metabolic
0.9579	7.91E-249	7.82E-245	PGS002560	EFO_0005091	monocyte count	monocyte count	EFO_0005091	Hematological measurement	Hematological	PGS002413	EFO_0005091	monocyte count	monocyte count	EFO_0005091	Hematological measurement	Hematological
0.9578	1.40E-248	1.38E-244	PGS002853	EFO_0004340	body mass index	body mass index	EFO_0004340	Body measurement	Body measurement	PGS002843	EFO_0004340	body mass index	body mass index	EFO_0004340	Body measurement	Body measurement
0.9577	2.52E-248	2.48E-244	PGS000796	EFO_0005088	testicular carcinoma|Testicular Germ Cell Tumor	testicular carcinoma	EFO_0005088	Cancer	Neoplasms	PGS000086	EFO_0005088	testicular carcinoma|Testicular Germ Cell Tumor	testicular carcinoma	EFO_0005088	Cancer	Neoplasms
0.9577	3.69E-248	3.62E-244	PGS003221	EFO_0005208	glomerular filtration rate	glomerular filtration rate	EFO_0005208	Other measurement	Genitourinary	PGS003225	EFO_0005208	glomerular filtration rate	glomerular filtration rate	EFO_0005208	Other measurement	Genitourinary
0.9576	4.16E-248	4.08E-244	PGS001339	EFO_0000275	atrial fibrillation|atrial flutter	atrial fibrillation	EFO_0000275	Cardiovascular disease	Cardiovascular	PGS001263	EFO_0003911	atrial flutter	atrial flutter	EFO_0003911	Cardiovascular disease	Cardiovascular
0.9574	1.44E-247	1.41E-243	PGS003123	MONDO_0005148	type 2 diabetes mellitus	type 2 diabetes mellitus	MONDO_0005148	Digestive system disorder	Gastrointestinal	PGS001781	MONDO_0005148	type 2 diabetes mellitus	type 2 diabetes mellitus	MONDO_0005148	Digestive system disorder	Gastrointestinal
0.9574	1.44E-247	1.41E-243	PGS003123	MONDO_0005148	type 2 diabetes mellitus	type 2 diabetes mellitus	MONDO_0005148	Metabolic disorder	Metabolic	PGS001781	MONDO_0005148	type 2 diabetes mellitus	type 2 diabetes mellitus	MONDO_0005148	Digestive system disorder	Gastrointestinal
0.9574	1.44E-247	1.41E-243	PGS003123	MONDO_0005148	type 2 diabetes mellitus	type 2 diabetes mellitus	MONDO_0005148	Digestive system disorder	Gastrointestinal	PGS001781	MONDO_0005148	type 2 diabetes mellitus	type 2 diabetes mellitus	MONDO_0005148	Metabolic disorder	Metabolic
0.9574	1.44E-247	1.41E-243	PGS003123	MONDO_0005148	type 2 diabetes mellitus	type 2 diabetes mellitus	MONDO_0005148	Metabolic disorder	Metabolic	PGS001781	MONDO_0005148	type 2 diabetes mellitus	type 2 diabetes mellitus	MONDO_0005148	Metabolic disorder	Metabolic
0.9573	2.35E-247	2.29E-243	PGS000207	EFO_0002892	thyroid carcinoma	thyroid carcinoma	EFO_0002892	Cancer	Neoplasms	PGS000632	EFO_0002892	thyroid carcinoma	thyroid carcinoma	EFO_0002892	Cancer	Neoplasms
0.9572	3.26E-247	3.17E-243	PGS003144	EFO_0004530	triglyceride measurement	triglyceride measurement	EFO_0004530	Lipid or lipoprotein measurement	Metabolic	PGS003149	EFO_0004530	triglyceride measurement	triglyceride measurement	EFO_0004530	Lipid or lipoprotein measurement	Metabolic
0.9572	4.18E-247	4.06E-243	PGS002531	EFO_0005140	autoimmune disease	autoimmune disease	EFO_0005140	Immune system disorder	Immunological	PGS002580	EFO_0005140	autoimmune disease	autoimmune disease	EFO_0005140	Immune system disorder	Immunological
0.9571	7.45E-247	7.22E-243	PGS001973	EFO_0004309	platelet count	platelet count	EFO_0004309	Hematological measurement	Hematological	PGS002191	EFO_0004309	platelet count	platelet count	EFO_0004309	Hematological measurement	Hematological
0.9571	8.36E-247	8.09E-243	PGS001158	EFO_0005106	body composition measurement	body composition measurement	EFO_0005106	Body measurement	Body measurement	PGS001108	EFO_0007777	base metabolic rate measurement	base metabolic rate measurement	EFO_0007777	Other measurement	Metabolic
0.957	1.21E-246	1.17E-242	PGS001235	EFO_0004324	body weights and measures	body weights and measures	EFO_0004324	Body measurement	Body measurement	PGS001165	EFO_0004995	lean body mass	lean body mass	EFO_0004995	Other measurement	Skeletal
0.9569	1.45E-246	1.39E-242	PGS002546	EFO_0004312	vital capacity	vital capacity	EFO_0004312	Other measurement	Activities	PGS002595	EFO_0004312	vital capacity	vital capacity	EFO_0004312	Other measurement	Activities
0.9569	1.90E-246	1.83E-242	PGS001166	EFO_0004995	lean body mass	lean body mass	EFO_0004995	Other measurement	Skeletal	PGS001108	EFO_0007777	base metabolic rate measurement	base metabolic rate measurement	EFO_0007777	Other measurement	Metabolic
0.9568	2.78E-246	2.67E-242	PGS002426	MONDO_0005148	type 2 diabetes mellitus	type 2 diabetes mellitus	MONDO_0005148	Digestive system disorder	Gastrointestinal	PGS002396	EFO_0001379	endocrine system disease|diabetes mellitus	endocrine system disease	EFO_0001379	Other disease	Metabolic
0.9568	2.78E-246	2.67E-242	PGS002426	MONDO_0005148	type 2 diabetes mellitus	type 2 diabetes mellitus	MONDO_0005148	Metabolic disorder	Metabolic	PGS002396	EFO_0001379	endocrine system disease|diabetes mellitus	endocrine system disease	EFO_0001379	Other disease	Metabolic
0.9567	4.65E-246	4.45E-242	PGS000528	EFO_0000305	breast carcinoma	breast carcinoma	EFO_0000305	Cancer	Neoplasms	PGS000527	EFO_0000305	breast carcinoma	breast carcinoma	EFO_0000305	Cancer	Neoplasms
0.9566	6.93E-246	6.62E-242	PGS002387	EFO_0003923	bone density	bone density	EFO_0003923	Other measurement	Skeletal	PGS002436	EFO_0003923	bone density	bone density	EFO_0003923	Other measurement	Skeletal
0.9566	7.07E-246	6.74E-242	PGS003203	EFO_0004631	vitamin D measurement	vitamin D measurement	EFO_0004631	Lipid or lipoprotein measurement	Metabolic	PGS003188	EFO_0004631	vitamin D measurement	vitamin D measurement	EFO_0004631	Lipid or lipoprotein measurement	Metabolic
0.9566	9.40E-246	8.96E-242	PGS000688	EFO_0004611	low density lipoprotein cholesterol measurement	low density lipoprotein cholesterol measurement	EFO_0004611	Lipid or lipoprotein measurement	Metabolic	PGS000672	EFO_0004615	apolipoprotein B measurement	apolipoprotein B measurement	EFO_0004615	Lipid or lipoprotein measurement	Metabolic
0.9566	9.40E-246	8.96E-242	PGS000688	EFO_0004611	low density lipoprotein cholesterol measurement	low density lipoprotein cholesterol measurement	EFO_0004611	Cardiovascular measurement	Cardiovascular	PGS000672	EFO_0004615	apolipoprotein B measurement	apolipoprotein B measurement	EFO_0004615	Lipid or lipoprotein measurement	Metabolic
0.9565	1.15E-245	1.10E-241	PGS002540	EFO_0000400	diabetes mellitus	diabetes mellitus	EFO_0000400	Digestive system disorder	Gastrointestinal	PGS002396	EFO_0001379	endocrine system disease|diabetes mellitus	endocrine system disease	EFO_0001379	Other disease	Metabolic
0.9565	1.15E-245	1.10E-241	PGS002540	EFO_0000400	diabetes mellitus	diabetes mellitus	EFO_0000400	Metabolic disorder	Metabolic	PGS002396	EFO_0001379	endocrine system disease|diabetes mellitus	endocrine system disease	EFO_0001379	Other disease	Metabolic
0.9565	1.33E-245	1.26E-241	PGS002839	EFO_0007878	alcohol consumption measurement	alcohol consumption measurement	EFO_0007878	Other measurement	Psychiatric	PGS000203	EFO_0007878	alcohol consumption measurement	alcohol consumption measurement	EFO_0007878	Other measurement	Psychiatric
0.9565	1.40E-245	1.32E-241	PGS002147	EFO_0009253	Immature Reticulocyte Fraction Measurement	Immature Reticulocyte Fraction Measurement	EFO_0009253	Hematological measurement	Hematological	PGS001930	EFO_0009253	Immature Reticulocyte Fraction Measurement	Measurement	EFO_0009253	Hematological measurement	Hematological
0.9564	1.93E-245	1.83E-241	PGS001146	EFO_0005409	fat body mass	fat body mass	EFO_0005409	Other measurement	Body measurement	PGS001165	EFO_0004995	lean body mass	lean body mass	EFO_0004995	Other measurement	Skeletal
0.9564	2.69E-245	2.53E-241	PGS001347	EFO_0009268	family history of Alzheimer’s disease	family history of Alzheimer’s disease	EFO_0009268	Other measurement	Neurological	PGS001348	MONDO_0004975	Alzheimer disease	Alzheimer disease	MONDO_0004975	Neurological disorder	Neurological
0.9562	6.83E-245	6.43E-241	PGS002547	EFO_0004468	glucose measurement	glucose measurement	EFO_0004468	Other measurement	Metabolic	PGS002400	EFO_0004468	glucose measurement	glucose measurement	EFO_0004468	Other measurement	Metabolic
0.9561	9.48E-245	8.90E-241	PGS002839	EFO_0007878	alcohol consumption measurement	alcohol consumption measurement	EFO_0007878	Other measurement	Psychiatric	PGS002834	EFO_0007878	alcohol consumption measurement	alcohol consumption measurement	EFO_0007878	Other measurement	Psychiatric
0.9559	3.44E-244	3.22E-240	PGS002834	EFO_0007878	alcohol consumption measurement	alcohol consumption measurement	EFO_0007878	Other measurement	Psychiatric	PGS000203	EFO_0007878	alcohol consumption measurement	alcohol consumption measurement	EFO_0007878	Other measurement	Psychiatric
0.9557	9.44E-244	8.84E-240	PGS001234	EFO_0004324	body weights and measures	body weights and measures	EFO_0004324	Body measurement	Body measurement	PGS001002	EFO_0009805	whole body water mass	whole body water mass	EFO_0009805	Other measurement	Body measurement
0.9556	1.67E-243	1.56E-239	PGS002167	EFO_0007991	eosinophil percentage of leukocytes	eosinophil percentage of leukocytes	EFO_0007991	Hematological measurement	Hematological	PGS001949	EFO_0007991	eosinophil percentage of leukocytes	eosinophil percentage of leukocytes	EFO_0007991	Hematological measurement	Hematological
0.9555	1.87E-243	1.74E-239	PGS000030	EFO_0001663	prostate carcinoma	prostate carcinoma	EFO_0001663	Cancer	Neoplasms	PGS000719	EFO_0001663	prostate carcinoma	prostate carcinoma	EFO_0001663	Cancer	Neoplasms
0.9553	5.31E-243	4.94E-239	PGS003108	MONDO_0005148	type 2 diabetes mellitus	type 2 diabetes mellitus	MONDO_0005148	Digestive system disorder	Gastrointestinal	PGS001781	MONDO_0005148	type 2 diabetes mellitus	type 2 diabetes mellitus	MONDO_0005148	Digestive system disorder	Gastrointestinal
0.9553	5.31E-243	4.94E-239	PGS003108	MONDO_0005148	type 2 diabetes mellitus	type 2 diabetes mellitus	MONDO_0005148	Digestive system disorder	Gastrointestinal	PGS001781	MONDO_0005148	type 2 diabetes mellitus	type 2 diabetes mellitus	MONDO_0005148	Metabolic disorder	Metabolic
0.9553	5.31E-243	4.94E-239	PGS003108	MONDO_0005148	type 2 diabetes mellitus	type 2 diabetes mellitus	MONDO_0005148	Metabolic disorder	Metabolic	PGS001781	MONDO_0005148	type 2 diabetes mellitus	type 2 diabetes mellitus	MONDO_0005148	Metabolic disorder	Metabolic
0.9553	5.31E-243	4.94E-239	PGS003108	MONDO_0005148	type 2 diabetes mellitus	type 2 diabetes mellitus	MONDO_0005148	Metabolic disorder	Metabolic	PGS001781	MONDO_0005148	type 2 diabetes mellitus	type 2 diabetes mellitus	MONDO_0005148	Digestive system disorder	Gastrointestinal
0.9551	1.82E-242	1.69E-238	PGS002834	EFO_0007878	alcohol consumption measurement	alcohol consumption measurement	EFO_0007878	Other measurement	Psychiatric	PGS000204	EFO_0007878	alcohol consumption measurement	alcohol consumption measurement	EFO_0007878	Other measurement	Psychiatric
0.9549	4.84E-242	4.49E-238	PGS002396	EFO_0001379	endocrine system disease|diabetes mellitus	endocrine system disease	EFO_0001379	Other disease	Metabolic	PGS002622	MONDO_0005148	type 2 diabetes mellitus	type 2 diabetes mellitus	MONDO_0005148	Metabolic disorder	Metabolic
0.9549	4.84E-242	4.49E-238	PGS002396	EFO_0001379	endocrine system disease|diabetes mellitus	endocrine system disease	EFO_0001379	Other disease	Metabolic	PGS002622	MONDO_0005148	type 2 diabetes mellitus	type 2 diabetes mellitus	MONDO_0005148	Digestive system disorder	Gastrointestinal
0.9548	8.43E-242	7.80E-238	PGS001340	EFO_0000275	atrial fibrillation	atrial fibrillation	EFO_0000275	Cardiovascular disease	Cardiovascular	PGS001339	EFO_0000275	atrial fibrillation|atrial flutter	atrial fibrillation	EFO_0000275	Cardiovascular disease	Cardiovascular
0.9548	8.92E-242	8.23E-238	PGS001826	MONDO_0015722	deficiency	deficiency	MONDO_0015722	Other disease	Hematological	PGS002034	MONDO_0015722	factors deficiency	coagulation factors deficiency	MONDO_0015722	Other disease	Hematological
0.9546	2.01E-241	1.85E-237	PGS002826	EFO_0007878	alcohol consumption measurement	alcohol consumption measurement	EFO_0007878	Other measurement	Psychiatric	PGS002827	EFO_0007878	alcohol consumption measurement	alcohol consumption measurement	EFO_0007878	Other measurement	Psychiatric
0.9545	3.47E-241	3.19E-237	PGS002540	EFO_0000400	diabetes mellitus	diabetes mellitus	EFO_0000400	Metabolic disorder	Metabolic	PGS002589	EFO_0000400	diabetes mellitus	diabetes mellitus	EFO_0000400	Digestive system disorder	Gastrointestinal
0.9545	3.47E-241	3.19E-237	PGS002540	EFO_0000400	diabetes mellitus	diabetes mellitus	EFO_0000400	Metabolic disorder	Metabolic	PGS002589	EFO_0000400	diabetes mellitus	diabetes mellitus	EFO_0000400	Metabolic disorder	Metabolic
0.9545	3.47E-241	3.19E-237	PGS002540	EFO_0000400	diabetes mellitus	diabetes mellitus	EFO_0000400	Digestive system disorder	Gastrointestinal	PGS002589	EFO_0000400	diabetes mellitus	diabetes mellitus	EFO_0000400	Metabolic disorder	Metabolic
0.9545	3.47E-241	3.19E-237	PGS002540	EFO_0000400	diabetes mellitus	diabetes mellitus	EFO_0000400	Digestive system disorder	Gastrointestinal	PGS002589	EFO_0000400	diabetes mellitus	diabetes mellitus	EFO_0000400	Digestive system disorder	Gastrointestinal
0.9544	4.89E-241	4.49E-237	PGS002581	EFO_0004340	body mass index	body mass index	EFO_0004340	Body measurement	Body measurement	PGS002532	EFO_0004340	body mass index	body mass index	EFO_0004340	Body measurement	Body measurement
0.9543	9.15E-241	8.39E-237	PGS003226	EFO_0005208	glomerular filtration rate	glomerular filtration rate	EFO_0005208	Other measurement	Genitourinary	PGS003220	EFO_0005208	glomerular filtration rate	glomerular filtration rate	EFO_0005208	Other measurement	Genitourinary
0.9538	8.30E-240	7.59E-236	PGS000203	EFO_0007878	alcohol consumption measurement	alcohol consumption measurement	EFO_0007878	Other measurement	Psychiatric	PGS000204	EFO_0007878	alcohol consumption measurement	alcohol consumption measurement	EFO_0007878	Other measurement	Psychiatric
0.9538	9.52E-240	8.69E-236	PGS003278	EFO_0010472	creatine measurement|glomerular filtration rate	creatine measurement	EFO_0010472	Other measurement	Metabolic	PGS003263	EFO_0010472	creatine measurement|glomerular filtration rate	creatine measurement	EFO_0010472	Other measurement	Metabolic
0.9537	1.31E-239	1.20E-235	PGS000748	EFO_0001645	coronary artery disease	coronary artery disease	EFO_0001645	Cardiovascular disease	Cardiovascular	PGS000749	EFO_0001645	coronary artery disease	coronary artery disease	EFO_0001645	Cardiovascular disease	Cardiovascular
0.9537	1.41E-239	1.29E-235	PGS001347	EFO_0009268	family history of Alzheimer’s disease	family history of Alzheimer’s disease	EFO_0009268	Other measurement	Neurological	PGS001827	MONDO_0001627	dementia	dementia	MONDO_0001627	Neurological disorder	Neurological
0.9533	1.03E-238	9.35E-235	PGS002186	EFO_0005091	monocyte count	monocyte count	EFO_0005091	Hematological measurement	Hematological	PGS001968	EFO_0005091	monocyte count	monocyte count	EFO_0005091	Hematological measurement	Hematological
0.953	4.02E-238	3.64E-234	PGS002035	MONDO_0001627	dementia	dementia	MONDO_0001627	Neurological disorder	Neurological	PGS001347	EFO_0009268	family history of Alzheimer’s disease	family history of Alzheimer’s disease	EFO_0009268	Other measurement	Neurological
0.9529	5.46E-238	4.93E-234	PGS001955	EFO_0009270	heel bone mineral density	heel bone mineral density	EFO_0009270	Other measurement	Skeletal	PGS001956	EFO_0007772	measurement	measurement	EFO_0007772	Other measurement	Skeletal
0.9529	8.37E-238	7.55E-234	PGS001169	EFO_0004995	lean body mass	lean body mass	EFO_0004995	Other measurement	Skeletal	PGS001234	EFO_0004324	body weights and measures	body weights and measures	EFO_0004324	Body measurement	Body measurement
0.9527	1.56E-237	1.40E-233	PGS002442	EFO_0000400	diabetes mellitus	diabetes mellitus	EFO_0000400	Digestive system disorder	Gastrointestinal	PGS002573	MONDO_0005148	type 2 diabetes mellitus	type 2 diabetes mellitus	MONDO_0005148	Digestive system disorder	Gastrointestinal
0.9527	1.56E-237	1.40E-233	PGS002442	EFO_0000400	diabetes mellitus	diabetes mellitus	EFO_0000400	Metabolic disorder	Metabolic	PGS002573	MONDO_0005148	type 2 diabetes mellitus	type 2 diabetes mellitus	MONDO_0005148	Metabolic disorder	Metabolic
0.9527	1.56E-237	1.40E-233	PGS002442	EFO_0000400	diabetes mellitus	diabetes mellitus	EFO_0000400	Digestive system disorder	Gastrointestinal	PGS002573	MONDO_0005148	type 2 diabetes mellitus	type 2 diabetes mellitus	MONDO_0005148	Metabolic disorder	Metabolic
0.9527	1.56E-237	1.40E-233	PGS002442	EFO_0000400	diabetes mellitus	diabetes mellitus	EFO_0000400	Metabolic disorder	Metabolic	PGS002573	MONDO_0005148	type 2 diabetes mellitus	type 2 diabetes mellitus	MONDO_0005148	Digestive system disorder	Gastrointestinal
0.9527	1.87E-237	1.68E-233	PGS002396	EFO_0001379	endocrine system disease|diabetes mellitus	endocrine system disease	EFO_0001379	Other disease	Metabolic	PGS002442	EFO_0000400	diabetes mellitus	diabetes mellitus	EFO_0000400	Digestive system disorder	Gastrointestinal
0.9527	1.87E-237	1.68E-233	PGS002396	EFO_0001379	endocrine system disease|diabetes mellitus	endocrine system disease	EFO_0001379	Other disease	Metabolic	PGS002442	EFO_0000400	diabetes mellitus	diabetes mellitus	EFO_0000400	Metabolic disorder	Metabolic
0.9526	2.60E-237	2.33E-233	PGS003268	EFO_0010472	creatine measurement|glomerular filtration rate	creatine measurement	EFO_0010472	Other measurement	Metabolic	PGS003253	EFO_0010472	creatine measurement|glomerular filtration rate	creatine measurement	EFO_0010472	Other measurement	Metabolic
0.9526	2.76E-237	2.47E-233	PGS002543	EFO_0001379	endocrine system disease|diabetes mellitus	endocrine system disease	EFO_0001379	Other disease	Metabolic	PGS002393	EFO_0000400	diabetes mellitus	diabetes mellitus	EFO_0000400	Digestive system disorder	Gastrointestinal
0.9526	2.76E-237	2.47E-233	PGS002543	EFO_0001379	endocrine system disease|diabetes mellitus	endocrine system disease	EFO_0001379	Other disease	Metabolic	PGS002393	EFO_0000400	diabetes mellitus	diabetes mellitus	EFO_0000400	Metabolic disorder	Metabolic
0.9523	1.41E-236	1.26E-232	PGS002396	EFO_0001379	endocrine system disease|diabetes mellitus	endocrine system disease	EFO_0001379	Other disease	Metabolic	PGS002573	MONDO_0005148	type 2 diabetes mellitus	type 2 diabetes mellitus	MONDO_0005148	Digestive system disorder	Gastrointestinal
0.9523	1.41E-236	1.26E-232	PGS002396	EFO_0001379	endocrine system disease|diabetes mellitus	endocrine system disease	EFO_0001379	Other disease	Metabolic	PGS002573	MONDO_0005148	type 2 diabetes mellitus	type 2 diabetes mellitus	MONDO_0005148	Metabolic disorder	Metabolic
0.9522	1.71E-236	1.53E-232	PGS001037	EFO_0009270	heel bone mineral density	heel bone mineral density	EFO_0009270	Other measurement	Skeletal	PGS000951	EFO_0004514	bone quantitative ultrasound measurement	measurement	EFO_0004514	Other measurement	Skeletal
0.9522	1.94E-236	1.72E-232	PGS002540	EFO_0000400	diabetes mellitus	diabetes mellitus	EFO_0000400	Digestive system disorder	Gastrointestinal	PGS002393	EFO_0000400	diabetes mellitus	diabetes mellitus	EFO_0000400	Digestive system disorder	Gastrointestinal
0.9522	1.94E-236	1.72E-232	PGS002540	EFO_0000400	diabetes mellitus	diabetes mellitus	EFO_0000400	Metabolic disorder	Metabolic	PGS002393	EFO_0000400	diabetes mellitus	diabetes mellitus	EFO_0000400	Digestive system disorder	Gastrointestinal
0.9522	1.94E-236	1.72E-232	PGS002540	EFO_0000400	diabetes mellitus	diabetes mellitus	EFO_0000400	Metabolic disorder	Metabolic	PGS002393	EFO_0000400	diabetes mellitus	diabetes mellitus	EFO_0000400	Metabolic disorder	Metabolic
0.9522	1.94E-236	1.72E-232	PGS002540	EFO_0000400	diabetes mellitus	diabetes mellitus	EFO_0000400	Digestive system disorder	Gastrointestinal	PGS002393	EFO_0000400	diabetes mellitus	diabetes mellitus	EFO_0000400	Metabolic disorder	Metabolic
0.9522	1.97E-236	1.75E-232	PGS000952	EFO_0004514	bone quantitative ultrasound measurement	bone quantitative ultrasound measurement	EFO_0004514	Other measurement	Skeletal	PGS000951	EFO_0004514	bone quantitative ultrasound measurement	measurement	EFO_0004514	Other measurement	Skeletal
0.9521	2.47E-236	2.19E-232	PGS001174	EFO_0004799	cholelithiasis	cholelithiasis	EFO_0004799	Digestive system disorder	Gastrointestinal	PGS001256	EFO_0004210	gallstones	gallstones	EFO_0004210	Digestive system disorder	Gastrointestinal
0.9521	3.31E-236	2.93E-232	PGS003110	MONDO_0005148	type 2 diabetes mellitus	type 2 diabetes mellitus	MONDO_0005148	Metabolic disorder	Metabolic	PGS003111	MONDO_0005148	type 2 diabetes mellitus	type 2 diabetes mellitus	MONDO_0005148	Metabolic disorder	Metabolic
0.9521	3.31E-236	2.93E-232	PGS003110	MONDO_0005148	type 2 diabetes mellitus	type 2 diabetes mellitus	MONDO_0005148	Metabolic disorder	Metabolic	PGS003111	MONDO_0005148	type 2 diabetes mellitus	type 2 diabetes mellitus	MONDO_0005148	Digestive system disorder	Gastrointestinal
0.9521	3.31E-236	2.93E-232	PGS003110	MONDO_0005148	type 2 diabetes mellitus	type 2 diabetes mellitus	MONDO_0005148	Digestive system disorder	Gastrointestinal	PGS003111	MONDO_0005148	type 2 diabetes mellitus	type 2 diabetes mellitus	MONDO_0005148	Digestive system disorder	Gastrointestinal
0.9521	3.31E-236	2.93E-232	PGS003110	MONDO_0005148	type 2 diabetes mellitus	type 2 diabetes mellitus	MONDO_0005148	Digestive system disorder	Gastrointestinal	PGS003111	MONDO_0005148	type 2 diabetes mellitus	type 2 diabetes mellitus	MONDO_0005148	Metabolic disorder	Metabolic
0.952	5.31E-236	4.70E-232	PGS001528	EFO_0007986	reticulocyte count	reticulocyte count	EFO_0007986	Hematological measurement	Hematological	PGS000989	EFO_0010700	reticulocyte measurement	reticulocyte measurement	EFO_0010700	Hematological measurement	Hematological
0.9518	1.14E-235	1.01E-231	PGS001234	EFO_0004324	body weights and measures	body weights and measures	EFO_0004324	Body measurement	Body measurement	PGS001108	EFO_0007777	base metabolic rate measurement	base metabolic rate measurement	EFO_0007777	Other measurement	Metabolic
0.9517	2.20E-235	1.93E-231	PGS001925	EFO_0004348	hematocrit	hematocrit	EFO_0004348	Hematological measurement	Hematological	PGS002141	EFO_0004348	hematocrit	hematocrit	EFO_0004348	Hematological measurement	Hematological
0.9516	2.45E-235	2.16E-231	PGS002445	EFO_0001379	endocrine system disease|diabetes mellitus	endocrine system disease	EFO_0001379	Other disease	Metabolic	PGS002442	EFO_0000400	diabetes mellitus	diabetes mellitus	EFO_0000400	Digestive system disorder	Gastrointestinal
0.9516	2.45E-235	2.16E-231	PGS002445	EFO_0001379	endocrine system disease|diabetes mellitus	endocrine system disease	EFO_0001379	Other disease	Metabolic	PGS002442	EFO_0000400	diabetes mellitus	diabetes mellitus	EFO_0000400	Metabolic disorder	Metabolic
0.9516	2.77E-235	2.43E-231	PGS000946	HP_0000726	Dementia	Dementia	HP_0000726	Other trait	Psychiatric	PGS001347	EFO_0009268	family history of Alzheimer’s disease	family history of Alzheimer’s disease	EFO_0009268	Other measurement	Neurological
0.9516	2.97E-235	2.60E-231	PGS000135	MONDO_0005090	schizophrenia	schizophrenia	MONDO_0005090	Neurological disorder	Neurological	PGS000134	MONDO_0005090	schizophrenia	schizophrenia	MONDO_0005090	Neurological disorder	Neurological
0.9516	3.37E-235	2.94E-231	PGS001840	MONDO_0004596	cor pulmonale	cor pulmonale	MONDO_0004596	Cardiovascular disease	Cardiovascular	PGS002049	MONDO_0004596	cor pulmonale	cor pulmonale	MONDO_0004596	Cardiovascular disease	Cardiovascular
0.9515	4.19E-235	3.66E-231	PGS002579	MONDO_0004979	asthma	asthma	MONDO_0004979	Other disease	Respiratory	PGS002615	EFO_0000684	respiratory system disease	respiratory system disease	EFO_0000684	Other disease	Skeletal
0.9515	4.87E-235	4.24E-231	PGS002592	EFO_0001379	endocrine system disease|diabetes mellitus	endocrine system disease	EFO_0001379	Other disease	Metabolic	PGS002596	EFO_0004468	glucose measurement	glucose measurement	EFO_0004468	Other measurement	Metabolic
0.9514	7.22E-235	6.28E-231	PGS001341	MONDO_0004979	asthma	asthma	MONDO_0004979	Other disease	Respiratory	PGS001346	MONDO_0004979	asthma	asthma	MONDO_0004979	Other disease	Respiratory
0.9514	7.63E-235	6.62E-231	PGS002543	EFO_0001379	endocrine system disease|diabetes mellitus	endocrine system disease	EFO_0001379	Other disease	Metabolic	PGS002426	MONDO_0005148	type 2 diabetes mellitus	type 2 diabetes mellitus	MONDO_0005148	Metabolic disorder	Metabolic
0.9514	7.63E-235	6.62E-231	PGS002543	EFO_0001379	endocrine system disease|diabetes mellitus	endocrine system disease	EFO_0001379	Other disease	Metabolic	PGS002426	MONDO_0005148	type 2 diabetes mellitus	type 2 diabetes mellitus	MONDO_0005148	Digestive system disorder	Gastrointestinal
0.9514	8.39E-235	7.27E-231	PGS000492	EFO_0000305	breast carcinoma	breast carcinoma	EFO_0000305	Cancer	Neoplasms	PGS000491	EFO_0000305	breast carcinoma	breast carcinoma	EFO_0000305	Cancer	Neoplasms

0.9512	1.70E-234	1.47E-230	PGS002410	EFO_0004587	lymphocyte count	lymphocyte count	EFO_0004587	Hematological measurement	Hematological	PGS002557	EFO_0004587	lymphocyte count	lymphocyte count	EFO_0004587	Hematological measurement	Hematological
0.9507	1.38E-233	1.19E-229	PGS000352	EFO_1001958	high grade ovarian serous adenocarcinoma	high grade ovarian serous adenocarcinoma	EFO_1001958	Cancer	Neoplasms	PGS000351	EFO_1001075	ovarian carcinoma	ovarian carcinoma	EFO_1001075	Cancer	Neoplasms
0.9507	1.63E-233	1.40E-229	PGS000467	EFO_0000707	squamous cell carcinoma	squamous cell carcinoma	EFO_0000707	Cancer	Neoplasms	PGS000466	EFO_0000707	squamous cell carcinoma	squamous cell carcinoma	EFO_0000707	Cancer	Neoplasms
0.9507	1.63E-233	1.40E-229	PGS000467	EFO_0000707	squamous cell carcinoma	squamous cell carcinoma	EFO_0000707	Cancer	Neoplasms	PGS000465	EFO_0000707	squamous cell carcinoma	squamous cell carcinoma	EFO_0000707	Cancer	Neoplasms
0.9507	1.76E-233	1.51E-229	PGS000945	HP_0000726	Dementia|Alzheimer disease	Dementia	HP_0000726	Other trait	Psychiatric	PGS002035	MONDO_0001627	dementia	dementia	MONDO_0001627	Neurological disorder	Neurological
0.9505	4.00E-233	3.43E-229	PGS001970	EFO_0007985	platelet crit	platelet crit	EFO_0007985	Hematological measurement	Hematological	PGS002188	EFO_0007985	platelet crit	platelet crit	EFO_0007985	Hematological measurement	Hematological
-0.9505	4.08E-233	3.49E-229	PGS000682	EFO_0005208	glomerular filtration rate	glomerular filtration rate	EFO_0005208	Other measurement	Genitourinary	PGS000678	EFO_0004518	creatinine measurement	creatinine measurement	EFO_0004518	Other measurement	Metabolic
0.9505	4.88E-233	4.17E-229	PGS000136	MONDO_0005090	schizophrenia	schizophrenia	MONDO_0005090	Neurological disorder	Neurological	PGS000134	MONDO_0005090	schizophrenia	schizophrenia	MONDO_0005090	Neurological disorder	Neurological
0.9503	9.39E-233	8.00E-229	PGS002589	EFO_0000400	diabetes mellitus	diabetes mellitus	EFO_0000400	Metabolic disorder	Metabolic	PGS002592	EFO_0001379	endocrine system disease|diabetes mellitus	endocrine system disease	EFO_0001379	Other disease	Metabolic
0.9503	9.39E-233	8.00E-229	PGS002589	EFO_0000400	diabetes mellitus	diabetes mellitus	EFO_0000400	Digestive system disorder	Gastrointestinal	PGS002592	EFO_0001379	endocrine system disease|diabetes mellitus	endocrine system disease	EFO_0001379	Other disease	Metabolic
0.9503	1.10E-232	9.32E-229	PGS001817	MONDO_0005147	type 1 diabetes mellitus	type 1 diabetes mellitus	MONDO_0005147	Digestive system disorder	Gastrointestinal	PGS002025	MONDO_0005147	type 1 diabetes mellitus	type 1 diabetes mellitus	MONDO_0005147	Immune system disorder	Immunological
0.9503	1.10E-232	9.32E-229	PGS001817	MONDO_0005147	type 1 diabetes mellitus	type 1 diabetes mellitus	MONDO_0005147	Immune system disorder	Immunological	PGS002025	MONDO_0005147	type 1 diabetes mellitus	type 1 diabetes mellitus	MONDO_0005147	Metabolic disorder	Metabolic
0.9503	1.10E-232	9.32E-229	PGS001817	MONDO_0005147	type 1 diabetes mellitus	type 1 diabetes mellitus	MONDO_0005147	Immune system disorder	Immunological	PGS002025	MONDO_0005147	type 1 diabetes mellitus	type 1 diabetes mellitus	MONDO_0005147	Digestive system disorder	Gastrointestinal
0.9503	1.10E-232	9.32E-229	PGS001817	MONDO_0005147	type 1 diabetes mellitus	type 1 diabetes mellitus	MONDO_0005147	Metabolic disorder	Metabolic	PGS002025	MONDO_0005147	type 1 diabetes mellitus	type 1 diabetes mellitus	MONDO_0005147	Digestive system disorder	Gastrointestinal
0.9503	1.10E-232	9.32E-229	PGS001817	MONDO_0005147	type 1 diabetes mellitus	type 1 diabetes mellitus	MONDO_0005147	Metabolic disorder	Metabolic	PGS002025	MONDO_0005147	type 1 diabetes mellitus	type 1 diabetes mellitus	MONDO_0005147	Metabolic disorder	Metabolic
0.9503	1.10E-232	9.32E-229	PGS001817	MONDO_0005147	type 1 diabetes mellitus	type 1 diabetes mellitus	MONDO_0005147	Metabolic disorder	Metabolic	PGS002025	MONDO_0005147	type 1 diabetes mellitus	type 1 diabetes mellitus	MONDO_0005147	Immune system disorder	Immunological
0.9503	1.10E-232	9.32E-229	PGS001817	MONDO_0005147	type 1 diabetes mellitus	type 1 diabetes mellitus	MONDO_0005147	Digestive system disorder	Gastrointestinal	PGS002025	MONDO_0005147	type 1 diabetes mellitus	type 1 diabetes mellitus	MONDO_0005147	Metabolic disorder	Metabolic
0.9503	1.10E-232	9.32E-229	PGS001817	MONDO_0005147	type 1 diabetes mellitus	type 1 diabetes mellitus	MONDO_0005147	Immune system disorder	Immunological	PGS002025	MONDO_0005147	type 1 diabetes mellitus	type 1 diabetes mellitus	MONDO_0005147	Immune system disorder	Immunological
0.9503	1.10E-232	9.32E-229	PGS001817	MONDO_0005147	type 1 diabetes mellitus	type 1 diabetes mellitus	MONDO_0005147	Digestive system disorder	Gastrointestinal	PGS002025	MONDO_0005147	type 1 diabetes mellitus	type 1 diabetes mellitus	MONDO_0005147	Digestive system disorder	Gastrointestinal
0.9503	1.25E-232	1.06E-228	PGS002187	EFO_0004833	neutrophil count	neutrophil count	EFO_0004833	Hematological measurement	Hematological	PGS001969	EFO_0004833	neutrophil count	neutrophil count	EFO_0004833	Hematological measurement	Hematological
0.9502	1.33E-232	1.13E-228	PGS002752	EFO_0007878	alcohol consumption measurement	alcohol consumption measurement	EFO_0007878	Other measurement	Psychiatric	PGS002839	EFO_0007878	alcohol consumption measurement	alcohol consumption measurement	EFO_0007878	Other measurement	Psychiatric
0.9502	1.49E-232	1.26E-228	PGS002190	EFO_0007984	platelet component distribution width	platelet component distribution width	EFO_0007984	Hematological measurement	Hematological	PGS001972	EFO_0007984	platelet component distribution width	platelet component distribution width	EFO_0007984	Hematological measurement	Hematological
0.9502	1.65E-232	1.39E-228	PGS002540	EFO_0000400	diabetes mellitus	diabetes mellitus	EFO_0000400	Metabolic disorder	Metabolic	PGS002592	EFO_0001379	endocrine system disease|diabetes mellitus	endocrine system disease	EFO_0001379	Other disease	Metabolic
0.9502	1.65E-232	1.39E-228	PGS002540	EFO_0000400	diabetes mellitus	diabetes mellitus	EFO_0000400	Digestive system disorder	Gastrointestinal	PGS002592	EFO_0001379	endocrine system disease|diabetes mellitus	endocrine system disease	EFO_0001379	Other disease	Metabolic
0.9502	1.83E-232	1.54E-228	PGS001975	EFO_0004326	heart rate	heart rate	EFO_0004326	Cardiovascular measurement	Cardiovascular	PGS002193	EFO_0004326	heart rate	heart rate	EFO_0004326	Cardiovascular measurement	Cardiovascular
0.9501	2.03E-232	1.71E-228	PGS001402	EFO_0009270	heel bone mineral density	heel bone mineral density	EFO_0009270	Other measurement	Skeletal	PGS000951	EFO_0004514	bone quantitative ultrasound measurement	measurement	EFO_0004514	Other measurement	Skeletal
0.9501	2.27E-232	1.91E-228	PGS002540	EFO_0000400	diabetes mellitus	diabetes mellitus	EFO_0000400	Digestive system disorder	Gastrointestinal	PGS002622	MONDO_0005148	type 2 diabetes mellitus	type 2 diabetes mellitus	MONDO_0005148	Digestive system disorder	Gastrointestinal
0.9501	2.27E-232	1.91E-228	PGS002540	EFO_0000400	diabetes mellitus	diabetes mellitus	EFO_0000400	Metabolic disorder	Metabolic	PGS002622	MONDO_0005148	type 2 diabetes mellitus	type 2 diabetes mellitus	MONDO_0005148	Metabolic disorder	Metabolic
0.9501	2.27E-232	1.91E-228	PGS002540	EFO_0000400	diabetes mellitus	diabetes mellitus	EFO_0000400	Metabolic disorder	Metabolic	PGS002622	MONDO_0005148	type 2 diabetes mellitus	type 2 diabetes mellitus	MONDO_0005148	Digestive system disorder	Gastrointestinal
0.9501	2.27E-232	1.91E-228	PGS002540	EFO_0000400	diabetes mellitus	diabetes mellitus	EFO_0000400	Digestive system disorder	Gastrointestinal	PGS002622	MONDO_0005148	type 2 diabetes mellitus	type 2 diabetes mellitus	MONDO_0005148	Metabolic disorder	Metabolic
0.9501	2.93E-232	2.46E-228	PGS002566	EFO_0000684	respiratory system disease	respiratory system disease	EFO_0000684	Other disease	Skeletal	PGS002615	EFO_0000684	respiratory system disease	respiratory system disease	EFO_0000684	Other disease	Skeletal
0.9501	2.97E-232	2.49E-228	PGS000697	EFO_0004570	bilirubin measurement	bilirubin measurement	EFO_0004570	Other measurement	Metabolic	PGS002032	Orphanet_309816	Disorder of bilirubin metabolism and excretion	excretion	Orphanet_309816	Metabolic disorder	Metabolic
0.9498	7.77E-232	6.49E-228	PGS002449	EFO_0004468	glucose measurement	glucose measurement	EFO_0004468	Other measurement	Metabolic	PGS002400	EFO_0004468	glucose measurement	glucose measurement	EFO_0004468	Other measurement	Metabolic
0.9498	1.05E-231	8.77E-228	PGS002181	EFO_0006925	lipoprotein A measurement	lipoprotein A measurement	EFO_0006925	Lipid or lipoprotein measurement	Metabolic	PGS001963	EFO_0006925	lipoprotein A measurement	lipoprotein A measurement	EFO_0006925	Lipid or lipoprotein measurement	Metabolic
0.9498	1.06E-231	8.81E-228	PGS003172	EFO_0004620	vitamin B12 measurement	vitamin B12 measurement	EFO_0004620	Other measurement	Metabolic	PGS003167	EFO_0004620	vitamin B12 measurement	vitamin B12 measurement	EFO_0004620	Other measurement	Metabolic
0.9497	1.31E-231	1.09E-227	PGS002200	EFO_0004631	vitamin D measurement	vitamin D measurement	EFO_0004631	Lipid or lipoprotein measurement	Metabolic	PGS001982	EFO_0004631	vitamin D measurement	vitamin D measurement	EFO_0004631	Lipid or lipoprotein measurement	Metabolic
0.9496	1.91E-231	1.59E-227	PGS002858	EFO_0004340	body mass index	body mass index	EFO_0004340	Body measurement	Body measurement	PGS002848	EFO_0004340	body mass index	body mass index	EFO_0004340	Body measurement	Body measurement
0.9494	6.00E-231	4.97E-227	PGS002195	EFO_0004696	sex hormone-binding globulin measurement	sex hormone-binding globulin measurement	EFO_0004696	Other measurement	Metabolic	PGS001977	EFO_0004696	sex hormone-binding globulin measurement	measurement	EFO_0004696	Other measurement	Metabolic
0.9489	3.97E-230	3.29E-226	PGS002336	EFO_0004705	hypothyroidism	hypothyroidism	EFO_0004705	Other disease	Endocrine	PGS002351	EFO_1000627	thyroid disease	thyroid disease	EFO_1000627	Other disease	Endocrine
0.9488	6.17E-230	5.09E-226	PGS001165	EFO_0004995	lean body mass	lean body mass	EFO_0004995	Other measurement	Skeletal	PGS001002	EFO_0009805	whole body water mass	whole body water mass	EFO_0009805	Other measurement	Body measurement
0.9487	9.50E-230	7.83E-226	PGS001805	EFO_0001663	prostate carcinoma	prostate carcinoma	EFO_0001663	Cancer	Neoplasms	PGS002016	EFO_0001663	prostate carcinoma	prostate carcinoma	EFO_0001663	Cancer	Neoplasms
0.9487	1.18E-229	9.74E-226	PGS000071	MONDO_0004986	urinary bladder carcinoma	urinary bladder carcinoma	MONDO_0004986	Cancer	Neoplasms	PGS000782	MONDO_0004986	urinary bladder carcinoma	urinary bladder carcinoma	MONDO_0004986	Cancer	Neoplasms
0.9486	2.13E-229	1.75E-225	PGS002871	EFO_0004458	C-reactive protein measurement	C-reactive protein measurement	EFO_0004458	Inflammatory measurement	Immunological	PGS002870	EFO_0004458	C-reactive protein measurement	C-reactive protein measurement	EFO_0004458	Inflammatory measurement	Immunological
0.9485	2.71E-229	2.22E-225	PGS002549	EFO_0007822	hair colour measurement	hair colour measurement	EFO_0007822	Other measurement	Dermatological	PGS002451	EFO_0007822	hair colour measurement	hair colour measurement	EFO_0007822	Other measurement	Dermatological
0.9484	3.88E-229	3.17E-225	PGS001980	EFO_0011005	urea measurement	urea measurement	EFO_0011005	Other measurement	Metabolic	PGS002198	EFO_0011005	urea measurement	urea measurement	EFO_0011005	Other measurement	Metabolic
0.9484	4.85E-229	3.96E-225	PGS001149	EFO_0005409	fat body mass	fat body mass	EFO_0005409	Other measurement	Body measurement	PGS001147	EFO_0005409	fat body mass	fat body mass	EFO_0005409	Other measurement	Body measurement
0.9483	6.84E-229	5.58E-225	PGS001824	Orphanet_309816	Disorder of bilirubin metabolism and excretion	Disorder of bilirubin metabolism and excretion	Orphanet_309816	Metabolic disorder	Metabolic	PGS000681	EFO_0004570	bilirubin measurement	bilirubin measurement	EFO_0004570	Other measurement	Metabolic
0.9482	1.16E-228	9.43E-225	PGS003077	EFO_0006335	systolic blood pressure	systolic blood pressure	EFO_0006335	Other measurement	Cardiovascular	PGS003072	EFO_0006335	systolic blood pressure	systolic blood pressure	EFO_0006335	Other measurement	Cardiovascular
0.948	2.40E-228	1.95E-224	PGS002415	EFO_0004309	platelet count	platelet count	EFO_0004309	Hematological measurement	Hematological	PGS002464	EFO_0004309	platelet count	platelet count	EFO_0004309	Hematological measurement	Hematological
0.9478	5.13E-228	4.17E-224	PGS001179	EFO_0004718	vascular dementia	vascular dementia	EFO_0004718	Cardiovascular disease	Cardiovascular	PGS001347	EFO_0009268	family history of Alzheimer's disease	family history of Alzheimer's disease	EFO_0009268	Other measurement	Neurological
0.9478	5.13E-228	4.17E-224	PGS001179	EFO_0004718	vascular dementia	vascular dementia	EFO_0004718	Neurological disorder	Neurological	PGS001347	EFO_0009268	family history of Alzheimer's disease	family history of Alzheimer's disease	EFO_0009268	Other measurement	Neurological
0.9474	2.47E-227	2.00E-223	PGS002584	EFO_0000319	cardiovascular disease	cardiovascular disease	EFO_0000319	Cardiovascular disease	Cardiovascular	PGS002535	EFO_0000319	cardiovascular disease	cardiovascular disease	EFO_0000319	Cardiovascular disease	Cardiovascular
0.9474	2.89E-227	2.34E-223	PGS002173	EFO_0009270	heel bone mineral density	heel bone mineral density	EFO_0009270	Other measurement	Skeletal	PGS002174	EFO_0007772	measurement	measurement	EFO_0007772	Other measurement	Skeletal
0.9474	3.29E-227	2.65E-223	PGS002611	EFO_0004309	platelet count	platelet count	EFO_0004309	Hematological measurement	Hematological	PGS002415	EFO_0004309	platelet count	platelet count	EFO_0004309	Hematological measurement	Hematological
0.9473	3.83E-227	3.09E-223	PGS002752	EFO_0007878	alcohol consumption measurement	alcohol consumption measurement	EFO_0007878	Other measurement	Psychiatric	PGS000204	EFO_0007878	alcohol consumption measurement	alcohol consumption measurement	EFO_0007878	Other measurement	Psychiatric
0.947	1.56E-226	1.26E-222	PGS002159	EFO_0004736	aspartate aminotransferase measurement	aspartate aminotransferase measurement	EFO_0004736	Liver enzyme measurement	Gastrointestinal	PGS001941	EFO_0004736	aspartate aminotransferase measurement	measurement	EFO_0004736	Liver enzyme measurement	Gastrointestinal
0.9469	2.17E-226	1.75E-222	PGS000782	MONDO_0004986	urinary bladder carcinoma	urinary bladder carcinoma	MONDO_0004986	Cancer	Neoplasms	PGS003335	MONDO_0004986	urinary bladder carcinoma	urinary bladder carcinoma	MONDO_0004986	Cancer	Neoplasms
0.9468	3.08E-226	2.47E-222	PGS001165	EFO_0004995	lean body mass	lean body mass	EFO_0004995	Other measurement	Skeletal	PGS001108	EFO_0007777	base metabolic rate measurement	base metabolic rate measurement	EFO_0007777	Other measurement	Metabolic
0.9468	3.51E-226	2.81E-222	PGS000930	MONDO_0000831	thrombotic disease	thrombotic disease	MONDO_0000831	Cardiovascular disease	Cardiovascular	PGS001277	EFO_0003827	pulmonary embolism|deep vein thrombosis	pulmonary embolism	EFO_0003827	Cardiovascular disease	Cardiovascular
0.9467	6.28E-226	5.02E-222	PGS002851	EFO_0004340	body mass index	body mass index	EFO_0004340	Body measurement	Body measurement	PGS002841	EFO_0004340	body mass index	body mass index	EFO_0004340	Body measurement	Body measurement
0.9466	7.65E-226	6.10E-222	PGS002397	EFO_0004842	eosinophil count	eosinophil count	EFO_0004842	Hematological measurement	Hematological	PGS002593	EFO_0004842	eosinophil count	eosinophil count	EFO_0004842	Hematological measurement	Hematological
0.9465	1.13E-225	9.00E-222	PGS001909	EFO_0004305	erythrocyte count	erythrocyte count	EFO_0004305	Hematological measurement	Hematological	PGS002123	EFO_0004305	erythrocyte count	erythrocyte count	EFO_0004305	Hematological measurement	Hematological
0.9465	1.30E-225	1.04E-221	PGS002142	EFO_0004509	hemoglobin measurement	hemoglobin measurement	EFO_0004509	Hematological measurement	Hematological	PGS001926	EFO_0004509	hemoglobin measurement	hemoglobin measurement	EFO_0004509	Hematological measurement	Hematological
0.9465	1.48E-225	1.17E-221	PGS001169	EFO_0004995	lean body mass	lean body mass	EFO_0004995	Other measurement	Skeletal	PGS001165	EFO_0004995	lean body mass	lean body mass	EFO_0004995	Other measurement	Skeletal
0.9463	3.17E-225	2.51E-221	PGS003318	EFO_0004617	cystatin C measurement|glomerular filtration rate	cystatin C measurement	EFO_0004617	Other measurement	Metabolic	PGS003303	EFO_0004617	cystatin C measurement|glomerular filtration rate	cystatin C measurement	EFO_0004617	Other measurement	Metabolic
0.9462	3.97E-225	3.14E-221	PGS002752	EFO_0007878	alcohol consumption measurement	alcohol consumption measurement	EFO_0007878	Other measurement	Psychiatric	PGS002834	EFO_0007878	alcohol consumption measurement	alcohol consumption measurement	EFO_0007878	Other measurement	Psychiatric
0.9461	6.60E-225	5.21E-221	PGS002831	EFO_0007878	alcohol consumption measurement	alcohol consumption measurement	EFO_0007878	Other measurement	Psychiatric	PGS002836	EFO_0007878	alcohol consumption measurement	alcohol consumption measurement	EFO_0007878	Other measurement	Psychiatric
0.946	1.02E-224	8.01E-221	PGS001715	EFO_0006930	brain volume measurement|neuroimaging measurement	brain volume measurement	EFO_0006930	Other measurement	Neurological	PGS001730	EFO_0006930	measurement	brain volume measurement	EFO_0006930	Other measurement	Neurological
0.9459	1.39E-224	1.09E-220	PGS002005	EFO_0011011	sitting height measurement	sitting height measurement	EFO_0011011	Other measurement	Skeletal	PGS002224	EFO_0011011	sitting height measurement	sitting height measurement	EFO_0011011	Other measurement	Skeletal
0.9458	2.33E-224	1.83E-220	PGS002445	EFO_0001379	endocrine system disease|diabetes mellitus	endocrine system disease	EFO_0001379	Other disease	Metabolic	PGS002475	MONDO_0005148	type 2 diabetes mellitus	type 2 diabetes mellitus	MONDO_0005148	Digestive system disorder	Gastrointestinal
0.9458	2.33E-224	1.83E-220	PGS002445	EFO_0001379	endocrine system disease|diabetes mellitus	endocrine system disease	EFO_0001379	Other disease	Metabolic	PGS002475	MONDO_0005148	type 2 diabetes mellitus	type 2 diabetes mellitus	MONDO_0005148	Metabolic disorder	Metabolic
0.9457	3.50E-224	2.75E-220	PGS002592	EFO_0001379	endocrine system disease|diabetes mellitus	endocrine system disease	EFO_0001379	Other disease	Metabolic	PGS002573	MONDO_0005148	type 2 diabetes mellitus	type 2 diabetes mellitus	MONDO_0005148	Digestive system disorder	Gastrointestinal
0.9457	3.50E-224	2.75E-220	PGS002592	EFO_0001379	endocrine system disease|diabetes mellitus	endocrine system disease	EFO_0001379	Other disease	Metabolic	PGS002573	MONDO_0005148	type 2 diabetes mellitus	type 2 diabetes mellitus	MONDO_0005148	Metabolic disorder	Metabolic
0.9457	3.91E-224	3.06E-220	PGS001279	EFO_0003827	pulmonary embolism|deep vein thrombosis	pulmonary embolism	EFO_0003827	Cardiovascular disease	Cardiovascular	PGS001277	EFO_0003827	pulmonary embolism|deep vein thrombosis	pulmonary embolism	EFO_0003827	Cardiovascular disease	Cardiovascular
0.9457	4.01E-224	3.14E-220	PGS002158	EFO_0004735	serum alanine aminotransferase measurement	serum alanine aminotransferase measurement	EFO_0004735	Liver enzyme measurement	Gastrointestinal	PGS001940	EFO_0004735	serum alanine aminotransferase measurement	measurement	EFO_0004735	Liver enzyme measurement	Gastrointestinal
0.9456	4.76E-224	3.72E-220	PGS002404	EFO_0004339	body height	body height	EFO_0004339	Body measurement	Body measurement	PGS002600	EFO_0004339	body height	body height	EFO_0004339	Body measurement	Body measurement
0.9455	8.34E-224	6.50E-220	PGS002559	EFO_0004584	mean platelet volume	mean platelet volume	EFO_0004584	Hematological measurement	Hematological	PGS002461	EFO_0004584	mean platelet volume	mean platelet volume	EFO_0004584	Hematological measurement	Hematological
0.9454	1.24E-223	9.69E-220	PGS001998	EFO_0010968	phosphate measurement	phosphate measurement	EFO_0010968	Other measurement	Metabolic	PGS002216	EFO_0010968	phosphate measurement	phosphate measurement	EFO_0010968	Other measurement	Metabolic
0.9454	1.32E-223	1.02E-219	PGS002083	EFO_0000676	psoriasis	psoriasis	EFO_0000676	Immune system disorder	Immunological	PGS001871	EFO_0000676	psoriasis	psoriasis	EFO_0000676	Immune system disorder	Immunological
0.9452	2.94E-223	2.28E-219	PGS001964	EFO_0004532	serum gamma-glutamyl transferase measurement	serum gamma-glutamyl transferase measurement	EFO_0004532	Liver enzyme measurement	Gastrointestinal	PGS002182	EFO_0004532	serum gamma-glutamyl transferase measurement	measurement	EFO_0004532	Liver enzyme measurement	Gastrointestinal
0.945	5.07E-223	3.92E-219	PGS000518	EFO_0000305	breast carcinoma	breast carcinoma	EFO_0000305	Cancer	Neoplasms	PGS000522	EFO_0000305	breast carcinoma	breast carcinoma	EFO_0000305	Cancer	Neoplasms
0.945	5.07E-223	3.92E-219	PGS000518	EFO_0000305	breast carcinoma	breast carcinoma	EFO_0000305	Cancer	Neoplasms	PGS000522	EFO_0000305	breast carcinoma	breast carcinoma	EFO_0000305	Cancer	Neoplasms
0.9448	1.17E-222	9.05E-219	PGS001986	EFO_0007993	lymphocyte percentage of leukocytes	lymphocyte percentage of leukocytes	EFO_0007993	Hematological measurement	Hematological	PGS002203	EFO_0007993	lymphocyte percentage of leukocytes	lymphocyte percentage of leukocytes	EFO_0007993	Hematological measurement	Hematological
0.9448	1.25E-222	9.64E-219	PGS001145	EFO_0005409	fat body mass	fat body mass	EFO_0005409	Other measurement	Body measurement	PGS001147	EFO_0005409	fat body mass	fat body mass	EFO_0005409	Other measurement	Body measurement
0.9448	1.28E-222	9.89E-219	PGS002601	EFO_0007986	reticulocyte count	reticulocyte count	EFO_0007986	Hematological measurement	Hematological	PGS002405	EFO_0007986	reticulocyte count	reticulocyte count	EFO_0007986	Hematological measurement	Hematological
0.9448	1.43E-222	1.10E-218	PGS000133	MONDO_0005090	schizophrenia	schizophrenia	MONDO_0005090	Neurological disorder	Neurological	PGS000136	MONDO_0005090	schizophrenia	schizophrenia	MONDO_0005090	Neurological disorder	Neurological
0.9447	1.67E-222	1.28E-218	PGS000954	EFO_0004514	bone quantitative ultrasound measurement	bone quantitative ultrasound measurement	EFO_0004514	Other measurement	Skeletal	PGS000953	EFO_0004514	bone quantitative ultrasound measurement	measurement	EFO_0004514	Other measurement	Skeletal
0.9447	1.74E-222	1.33E-218	PGS001038	EFO_0009270	heel bone mineral density	heel bone mineral density	EFO_0009270	Other measurement	Skeletal	PGS000953	EFO_0004514	bone quantitative ultrasound measurement	measurement	EFO_0004514	Other measurement	Skeletal
0.9445	4.90E-222	3.75E-218	PGS000719	EFO_0001663	prostate carcinoma	prostate carcinoma	EFO_0001663	Cancer	Neoplasms	PGS000580	EFO_0001663	prostate carcinoma	prostate carcinoma	EFO_0001663	Cancer	Neoplasms
0.9442	1.18E-221	8.98E-218	PGS001933	EFO_0004611	low density lipoprotein cholesterol measurement	low density lipoprotein cholesterol measurement	EFO_0004611	Cardiovascular measurement	Cardiovascular	PGS001889	EFO_0004615	apolipoprotein B measurement	apolipoprotein B measurement	EFO_0004615	Lipid or lipoprotein measurement	Metabolic
0.9442	1.18E-221	8.98E-218	PGS001933	EFO_0004611	low density lipoprotein cholesterol measurement	low density lipoprotein cholesterol measurement	EFO_0004611	Lipid or lipoprotein measurement	Metabolic	PGS001889	EFO_0004615	apolipoprotein B measurement	apolipoprotein B measurement	EFO_0004615	Lipid or lipoprotein measurement	Metabolic
0.9442	1.50E-221	1.14E-217	PGS003191	EFO_0004631	vitamin D measurement	vitamin D measurement	EFO_0004631	Lipid or lipoprotein measurement	Metabolic	PGS003190	EFO_0004631	vitamin D measurement	vitamin D measurement	EFO_0004631	Lipid or lipoprotein measurement	Metabolic
0.944	3.09E-221	2.36E-217	PGS001108	EFO_0007777	base metabolic rate measurement	base metabolic rate measurement	EFO_0007777	Other measurement	Metabolic	PGS001167	EFO_0004995	lean body mass	lean body mass	EFO_0004995	Other measurement	Skeletal
0.944	3.46E-221	2.63E-217	PGS001108	EFO_0007777	base metabolic rate measurement	base metabolic rate measurement	EFO_0007777	Other measurement	Metabolic	PGS001159	EFO_0005106	body composition measurement	body composition measurement	EFO_0005106	Body measurement	Body measurement
0.9439	4.60E-221	3.49E-217	PGS001893	EFO_0004838	calcium measurement	calcium measurement	EFO_0004838	Other measurement	Metabolic	PGS002106	EFO_0004838	calcium measurement	calcium measurement	EFO_0004838	Other measurement	Metabolic
0.9439	4.74E-221	3.59E-217	PGS002537	EFO_0008328	chronotype measurement	chronotype measurement	EFO_0008328	Other measurement	Psychiatric	PGS002586	EFO_0008328	chronotype measurement	chronotype measurement	EFO_0008328	Other measurement	Psychiatric
0.9439	5.35E-221	4.04E-217	PGS002229	EFO_0004531	urate measurement	urate measurement	EFO_0004531	Other measurement	Metabolic	PGS002010	EFO_0004531	urate measurement	urate measurement	EFO_0004531	Other measurement	Metabolic
0.9438	6.85E-221	5.17E-217	PGS003031	EFO_0004611	low density lipoprotein cholesterol measurement	low density lipoprotein cholesterol measurement	EFO_0004611	Cardiovascular measurement	Cardiovascular	PGS003030	EFO_0004611	low density lipoprotein cholesterol measurement	measurement	EFO_0004611	Cardiovascular measurement	Cardiovascular
0.9438	6.85E-221	5.17E-217	PGS003031	EFO_0004611	low density lipoprotein cholesterol measurement	low density lipoprotein cholesterol measurement	EFO_0004611	Cardiovascular measurement	Cardiovascular	PGS003030	EFO_0004611	low density lipoprotein cholesterol measurement	measurement	EFO_0004611	Lipid or lipoprotein measurement	Metabolic
0.9438	6.85E-221	5.17E-217	PGS003031	EFO_0004611	low density lipoprotein cholesterol measurement	low density lipoprotein cholesterol measurement	EFO_0004611	Lipid or lipoprotein measurement	Metabolic	PGS003030	EFO_0004611	low density lipoprotein cholesterol measurement	measurement	EFO_0004611	Lipid or lipoprotein measurement	Metabolic
0.9438	6.85E-221	5.17E-217	PGS003031	EFO_0004611	low density lipoprotein cholesterol measurement	low density lipoprotein cholesterol measurement	EFO_0004611	Lipid or lipoprotein measurement	Metabolic	PGS003030	EFO_0004611	low density lipoprotein cholesterol measurement	measurement	EFO_0004611	Cardiovascular measurement	Cardiovascular
0.9438	7.60E-221	5.73E-217	PGS002410	EFO_0004587	lymphocyte count	lymphocyte count	EFO_0004587	Hematological measurement	Hematological	PGS002459	EFO_0004587	lymphocyte count	lymphocyte count	EFO_0004587	Hematological measurement	Hematological
0.9437	1.14E-220	8.55E-217	PGS002321	EFO_0000400	diabetes mellitus	diabetes mellitus	EFO_0000400	Digestive system disorder	Gastrointestinal	PGS002354	MONDO_0005148	type 2 diabetes mellitus	type 2 diabetes mellitus	MONDO_0005148	Digestive system disorder	Gastrointestinal

0.9437	1.14E-220	8.55E-217	PGS002321	EFO_0000400	diabetes mellitus	diabetes mellitus	EFO_0000400	Digestive system disorder	Gastrointestinal	PGS002354	MONDO_0005148	type 2 diabetes mellitus	type 2 diabetes mellitus	MONDO_0005148	Metabolic disorder	Metabolic
0.9437	1.14E-220	8.55E-217	PGS002321	EFO_0000400	diabetes mellitus	diabetes mellitus	EFO_0000400	Metabolic disorder	Metabolic	PGS002354	MONDO_0005148	type 2 diabetes mellitus	type 2 diabetes mellitus	MONDO_0005148	Digestive system disorder	Gastrointestinal
0.9437	1.14E-220	8.55E-217	PGS002321	EFO_0000400	diabetes mellitus	diabetes mellitus	EFO_0000400	Metabolic disorder	Metabolic	PGS002354	MONDO_0005148	type 2 diabetes mellitus	type 2 diabetes mellitus	MONDO_0005148	Metabolic disorder	Metabolic
0.9436	1.50E-220	1.12E-216	PGS000930	MONDO_0000831	thrombotic disease	thrombotic disease	MONDO_0000831	Cardiovascular disease	Cardiovascular	PGS001280	EFO_0003827	pulmonary embolism	pulmonary embolism	EFO_0003827	Cardiovascular disease	Cardiovascular
0.9436	1.69E-220	1.27E-216	PGS001948	EFO_0005055	QRS duration	QRS duration	EFO_0005055	Cardiovascular measurement	Cardiovascular	PGS002166	EFO_0005055	QRS duration	QRS duration	EFO_0005055	Cardiovascular measurement	Cardiovascular
0.9435	2.02E-220	1.51E-216	PGS002425	EFO_0004530	triglyceride measurement	triglyceride measurement	EFO_0004530	Lipid or lipoprotein measurement	Metabolic	PGS002621	EFO_0004530	triglyceride measurement	triglyceride measurement	EFO_0004530	Lipid or lipoprotein measurement	Metabolic
0.9435	2.48E-220	1.85E-216	PGS003283	EFO_0004617	cystatin C measurement	cystatin C measurement	EFO_0004617	Other measurement	Metabolic	PGS003288	EFO_0004617	cystatin C measurement	cystatin C measurement	EFO_0004617	Other measurement	Metabolic
0.9434	2.81E-220	2.10E-216	PGS002475	MONDO_0005148	type 2 diabetes mellitus	type 2 diabetes mellitus	MONDO_0005148	Digestive system disorder	Gastrointestinal	PGS002396	EFO_0001379	endocrine system disease|diabetes mellitus	endocrine system disease	EFO_0001379	Other disease	Metabolic
0.9434	2.81E-220	2.10E-216	PGS002475	MONDO_0005148	type 2 diabetes mellitus	type 2 diabetes mellitus	MONDO_0005148	Metabolic disorder	Metabolic	PGS002396	EFO_0001379	endocrine system disease|diabetes mellitus	endocrine system disease	EFO_0001379	Other disease	Metabolic
0.9431	1.04E-219	7.77E-216	PGS001403	EFO_0009270	heel bone mineral density	heel bone mineral density	EFO_0009270	Other measurement	Skeletal	PGS000953	EFO_0004514	bone quantitative ultrasound measurement	measurement	EFO_0004514	Other measurement	Skeletal
0.9428	2.77E-219	2.06E-215	PGS000488	EFO_0000305	breast carcinoma	breast carcinoma	EFO_0000305	Cancer	Neoplasms	PGS000485	EFO_0000305	breast carcinoma	breast carcinoma	EFO_0000305	Cancer	Neoplasms
0.9428	2.77E-219	2.06E-215	PGS000488	EFO_0000305	breast carcinoma	breast carcinoma	EFO_0000305	Cancer	Neoplasms	PGS000487	EFO_0000305	breast carcinoma	breast carcinoma	EFO_0000305	Cancer	Neoplasms
0.9428	2.98E-219	2.21E-215	PGS000578	EFO_0001663	prostate carcinoma	prostate carcinoma	EFO_0001663	Cancer	Neoplasms	PGS000719	EFO_0001663	prostate carcinoma	prostate carcinoma	EFO_0001663	Cancer	Neoplasms
0.9428	2.98E-219	2.21E-215	PGS000577	EFO_0001663	prostate carcinoma	prostate carcinoma	EFO_0001663	Cancer	Neoplasms	PGS000719	EFO_0001663	prostate carcinoma	prostate carcinoma	EFO_0001663	Cancer	Neoplasms
0.9428	3.19E-219	2.36E-215	PGS002426	MONDO_0005148	type 2 diabetes mellitus	type 2 diabetes mellitus	MONDO_0005148	Digestive system disorder	Gastrointestinal	PGS002592	EFO_0001379	endocrine system disease|diabetes mellitus	endocrine system disease	EFO_0001379	Other disease	Metabolic
0.9428	3.19E-219	2.36E-215	PGS002426	MONDO_0005148	type 2 diabetes mellitus	type 2 diabetes mellitus	MONDO_0005148	Metabolic disorder	Metabolic	PGS002592	EFO_0001379	endocrine system disease|diabetes mellitus	endocrine system disease	EFO_0001379	Other disease	Metabolic
0.9426	6.51E-219	4.81E-215	PGS002564	EFO_0004305	erythrocyte count	erythrocyte count	EFO_0004305	Hematological measurement	Hematological	PGS002417	EFO_0004305	erythrocyte count	erythrocyte count	EFO_0004305	Hematological measurement	Hematological
0.9425	1.09E-218	8.01E-215	PGS002622	MONDO_0005148	type 2 diabetes mellitus	type 2 diabetes mellitus	MONDO_0005148	Digestive system disorder	Gastrointestinal	PGS002573	MONDO_0005148	type 2 diabetes mellitus	type 2 diabetes mellitus	MONDO_0005148	Digestive system disorder	Gastrointestinal
0.9425	1.09E-218	8.01E-215	PGS002622	MONDO_0005148	type 2 diabetes mellitus	type 2 diabetes mellitus	MONDO_0005148	Metabolic disorder	Metabolic	PGS002573	MONDO_0005148	type 2 diabetes mellitus	type 2 diabetes mellitus	MONDO_0005148	Digestive system disorder	Gastrointestinal
0.9425	1.09E-218	8.01E-215	PGS002622	MONDO_0005148	type 2 diabetes mellitus	type 2 diabetes mellitus	MONDO_0005148	Digestive system disorder	Gastrointestinal	PGS002573	MONDO_0005148	type 2 diabetes mellitus	type 2 diabetes mellitus	MONDO_0005148	Metabolic disorder	Metabolic
0.9425	1.09E-218	8.01E-215	PGS002622	MONDO_0005148	type 2 diabetes mellitus	type 2 diabetes mellitus	MONDO_0005148	Metabolic disorder	Metabolic	PGS002573	MONDO_0005148	type 2 diabetes mellitus	type 2 diabetes mellitus	MONDO_0005148	Metabolic disorder	Metabolic
0.9425	1.18E-218	8.72E-215	PGS002543	EFO_0001379	endocrine system disease|diabetes mellitus	endocrine system disease	EFO_0001379	Other disease	Metabolic	PGS002403	EFO_0004541	HbA1c measurement	HbA1c measurement	EFO_0004541	Hematological measurement	Hematological
0.9424	1.59E-218	1.17E-214	PGS000577	EFO_0001663	prostate carcinoma	prostate carcinoma	EFO_0001663	Cancer	Neoplasms	PGS000579	EFO_0001663	prostate carcinoma	prostate carcinoma	EFO_0001663	Cancer	Neoplasms
0.9424	1.59E-218	1.17E-214	PGS000578	EFO_0001663	prostate carcinoma	prostate carcinoma	EFO_0001663	Cancer	Neoplasms	PGS000579	EFO_0001663	prostate carcinoma	prostate carcinoma	EFO_0001663	Cancer	Neoplasms
0.9423	2.18E-218	1.60E-214	PGS003189	EFO_0004631	vitamin D measurement	vitamin D measurement	EFO_0004631	Lipid or lipoprotein measurement	Metabolic	PGS003194	EFO_0004631	vitamin D measurement	vitamin D measurement	EFO_0004631	Lipid or lipoprotein measurement	Metabolic
0.9422	2.93E-218	2.15E-214	PGS001280	EFO_0003827	pulmonary embolism	pulmonary embolism	EFO_0003827	Cardiovascular disease	Cardiovascular	PGS001279	EFO_0003827	pulmonary embolism|deep vein thrombosis	pulmonary embolism	EFO_0003827	Cardiovascular disease	Cardiovascular
0.9422	3.09E-218	2.26E-214	PGS002426	MONDO_0005148	type 2 diabetes mellitus	type 2 diabetes mellitus	MONDO_0005148	Digestive system disorder	Gastrointestinal	PGS002622	MONDO_0005148	type 2 diabetes mellitus	type 2 diabetes mellitus	MONDO_0005148	Metabolic disorder	Metabolic
0.9422	3.09E-218	2.26E-214	PGS002426	MONDO_0005148	type 2 diabetes mellitus	type 2 diabetes mellitus	MONDO_0005148	Metabolic disorder	Metabolic	PGS002622	MONDO_0005148	type 2 diabetes mellitus	type 2 diabetes mellitus	MONDO_0005148	Metabolic disorder	Metabolic
0.9422	3.09E-218	2.26E-214	PGS002426	MONDO_0005148	type 2 diabetes mellitus	type 2 diabetes mellitus	MONDO_0005148	Digestive system disorder	Gastrointestinal	PGS002622	MONDO_0005148	type 2 diabetes mellitus	type 2 diabetes mellitus	MONDO_0005148	Digestive system disorder	Gastrointestinal
0.9422	3.09E-218	2.26E-214	PGS002426	MONDO_0005148	type 2 diabetes mellitus	type 2 diabetes mellitus	MONDO_0005148	Metabolic disorder	Metabolic	PGS002622	MONDO_0005148	type 2 diabetes mellitus	type 2 diabetes mellitus	MONDO_0005148	Digestive system disorder	Gastrointestinal
0.9422	3.34E-218	2.44E-214	PGS002598	EFO_0007822	hair colour measurement	hair colour measurement	EFO_0007822	Other measurement	Dermatological	PGS002451	EFO_0007822	hair colour measurement	hair colour measurement	EFO_0007822	Other measurement	Dermatological
0.9421	5.10E-218	3.72E-214	PGS002543	EFO_0001379	endocrine system disease|diabetes mellitus	endocrine system disease	EFO_0001379	Other disease	Metabolic	PGS002400	EFO_0004468	glucose measurement	glucose measurement	EFO_0004468	Other measurement	Metabolic
0.9421	5.56E-218	4.04E-214	PGS000051	EFO_0000305	breast carcinoma	breast carcinoma	EFO_0000305	Cancer	Neoplasms	PGS000001	EFO_0000305	breast carcinoma	breast carcinoma	EFO_0000305	Cancer	Neoplasms
0.942	6.30E-218	4.58E-214	PGS000579	EFO_0001663	prostate carcinoma	prostate carcinoma	EFO_0001663	Cancer	Neoplasms	PGS000580	EFO_0001663	prostate carcinoma	prostate carcinoma	EFO_0001663	Cancer	Neoplasms
0.942	7.24E-218	5.25E-214	PGS002540	EFO_0000400	diabetes mellitus	diabetes mellitus	EFO_0000400	Metabolic disorder	Metabolic	PGS002573	MONDO_0005148	type 2 diabetes mellitus	type 2 diabetes mellitus	MONDO_0005148	Metabolic disorder	Metabolic
0.942	7.24E-218	5.25E-214	PGS002540	EFO_0000400	diabetes mellitus	diabetes mellitus	EFO_0000400	Metabolic disorder	Metabolic	PGS002573	MONDO_0005148	type 2 diabetes mellitus	type 2 diabetes mellitus	MONDO_0005148	Digestive system disorder	Gastrointestinal
0.942	7.24E-218	5.25E-214	PGS002540	EFO_0000400	diabetes mellitus	diabetes mellitus	EFO_0000400	Digestive system disorder	Gastrointestinal	PGS002573	MONDO_0005148	type 2 diabetes mellitus	type 2 diabetes mellitus	MONDO_0005148	Metabolic disorder	Metabolic
0.942	7.24E-218	5.25E-214	PGS002540	EFO_0000400	diabetes mellitus	diabetes mellitus	EFO_0000400	Digestive system disorder	Gastrointestinal	PGS002573	MONDO_0005148	type 2 diabetes mellitus	type 2 diabetes mellitus	MONDO_0005148	Digestive system disorder	Gastrointestinal
0.9419	9.64E-218	6.98E-214	PGS001828	MONDO_0004975	Alzheimer disease	Alzheimer disease	MONDO_0004975	Neurological disorder	Neurological	PGS001347	EFO_0009268	family history of Alzheimer's disease	family history of Alzheimer's disease	EFO_0009268	Other measurement	Neurological
0.9419	1.04E-217	7.49E-214	PGS001971	EFO_0004584	mean platelet volume	mean platelet volume	EFO_0004584	Hematological measurement	Hematological	PGS002340	EFO_0004584	mean platelet volume	mean platelet volume	EFO_0004584	Hematological measurement	Hematological
0.9418	1.27E-217	9.18E-214	PGS001957	EFO_0005654	velocity of sound measurement	velocity of sound measurement	EFO_0005654	Other measurement	Others	PGS002175	EFO_0005654	velocity of sound measurement	velocity of sound measurement	EFO_0005654	Other measurement	Others
0.9418	1.61E-217	1.16E-213	PGS001343	MONDO_0004979	asthma	asthma	MONDO_0004979	Other disease	Respiratory	PGS001346	MONDO_0004979	asthma	asthma	MONDO_0004979	Other disease	Respiratory
0.9418	1.83E-217	1.32E-213	PGS001965	EFO_0004587	lymphocyte count	lymphocyte count	EFO_0004587	Hematological measurement	Hematological	PGS002183	EFO_0004587	lymphocyte count	lymphocyte count	EFO_0004587	Hematological measurement	Hematological
0.9417	2.31E-217	1.66E-213	PGS001997	EFO_0007990	neutrophil percentage of leukocytes	neutrophil percentage of leukocytes	EFO_0007990	Hematological measurement	Hematological	PGS002214	EFO_0007990	neutrophil percentage of leukocytes	neutrophil percentage of leukocytes	EFO_0007990	Hematological measurement	Hematological
0.9416	3.05E-217	2.19E-213	PGS003113	MONDO_0005148	type 2 diabetes mellitus	type 2 diabetes mellitus	MONDO_0005148	Metabolic disorder	Metabolic	PGS003098	MONDO_0005148	type 2 diabetes mellitus	type 2 diabetes mellitus	MONDO_0005148	Metabolic disorder	Metabolic
0.9416	3.05E-217	2.19E-213	PGS003113	MONDO_0005148	type 2 diabetes mellitus	type 2 diabetes mellitus	MONDO_0005148	Digestive system disorder	Gastrointestinal	PGS003098	MONDO_0005148	type 2 diabetes mellitus	type 2 diabetes mellitus	MONDO_0005148	Metabolic disorder	Metabolic
0.9416	3.05E-217	2.19E-213	PGS003113	MONDO_0005148	type 2 diabetes mellitus	type 2 diabetes mellitus	MONDO_0005148	Metabolic disorder	Metabolic	PGS003098	MONDO_0005148	type 2 diabetes mellitus	type 2 diabetes mellitus	MONDO_0005148	Digestive system disorder	Gastrointestinal
0.9416	3.05E-217	2.19E-213	PGS003113	MONDO_0005148	type 2 diabetes mellitus	type 2 diabetes mellitus	MONDO_0005148	Digestive system disorder	Gastrointestinal	PGS003098	MONDO_0005148	type 2 diabetes mellitus	type 2 diabetes mellitus	MONDO_0005148	Digestive system disorder	Gastrointestinal
0.9416	3.27E-217	2.33E-213	PGS002947	EFO_0004465	fasting blood glucose measurement	fasting blood glucose measurement	EFO_0004465	Other measurement	Metabolic	PGS002925	EFO_0004465	fasting blood glucose measurement	fasting blood glucose measurement	EFO_0004465	Other measurement	Metabolic
0.9416	3.27E-217	2.33E-213	PGS002947	EFO_0004465	fasting blood glucose measurement	fasting blood glucose measurement	EFO_0004465	Other measurement	Metabolic	PGS002926	EFO_0004465	fasting blood glucose measurement	fasting blood glucose measurement	EFO_0004465	Other measurement	Metabolic
0.9416	3.27E-217	2.33E-213	PGS002952	EFO_0004465	fasting blood glucose measurement	fasting blood glucose measurement	EFO_0004465	Other measurement	Metabolic	PGS002926	EFO_0004465	fasting blood glucose measurement	fasting blood glucose measurement	EFO_0004465	Other measurement	Metabolic
0.9416	3.27E-217	2.33E-213	PGS002952	EFO_0004465	fasting blood glucose measurement	fasting blood glucose measurement	EFO_0004465	Other measurement	Metabolic	PGS002925	EFO_0004465	fasting blood glucose measurement	fasting blood glucose measurement	EFO_0004465	Other measurement	Metabolic
0.9415	3.99E-217	2.84E-213	PGS001235	EFO_0004324	body weights and measures	body weights and measures	EFO_0004324	Body measurement	Body measurement	PGS001002	EFO_0009805	whole body water mass	whole body water mass	EFO_0009805	Other measurement	Body measurement
0.9415	4.46E-217	3.17E-213	PGS001979	EFO_0004530	triglyceride measurement	triglyceride measurement	EFO_0004530	Lipid or lipoprotein measurement	Metabolic	PGS002197	EFO_0004530	triglyceride measurement	triglyceride measurement	EFO_0004530	Lipid or lipoprotein measurement	Metabolic
0.9414	8.31E-217	5.90E-213	PGS001946	EFO_0004458	C-reactive protein measurement	C-reactive protein measurement	EFO_0004458	Inflammatory measurement	Immunological	PGS002164	EFO_0004458	C-reactive protein measurement	C-reactive protein measurement	EFO_0004458	Inflammatory measurement	Immunological
0.9413	9.99E-217	7.08E-213	PGS001147	EFO_0005409	fat body mass	fat body mass	EFO_0005409	Other measurement	Body measurement	PGS001144	EFO_0005409	fat body mass	fat body mass	EFO_0005409	Other measurement	Body measurement
0.9413	1.15E-216	8.11E-213	PGS001926	EFO_0004509	hemoglobin measurement	hemoglobin measurement	EFO_0004509	Hematological measurement	Hematological	PGS001925	EFO_0004348	hematocrit	hematocrit	EFO_0004348	Hematological measurement	Hematological
0.9412	1.33E-216	9.42E-213	PGS000633	EFO_0002892	thyroid carcinoma	thyroid carcinoma	EFO_0002892	Cancer	Neoplasms	PGS000208	EFO_0002892	thyroid carcinoma	thyroid carcinoma	EFO_0002892	Cancer	Neoplasms
0.941	2.62E-216	1.85E-212	PGS002150	EFO_0004611	low density lipoprotein cholesterol measurement	low density lipoprotein cholesterol measurement	EFO_0004611	Lipid or lipoprotein measurement	Metabolic	PGS002102	EFO_0004615	apolipoprotein B measurement	apolipoprotein B measurement	EFO_0004615	Lipid or lipoprotein measurement	Metabolic
0.941	2.62E-216	1.85E-212	PGS002150	EFO_0004611	low density lipoprotein cholesterol measurement	low density lipoprotein cholesterol measurement	EFO_0004611	Cardiovascular measurement	Cardiovascular	PGS002102	EFO_0004615	apolipoprotein B measurement	apolipoprotein B measurement	EFO_0004615	Lipid or lipoprotein measurement	Metabolic
0.941	3.65E-216	2.57E-212	PGS001861	EFO_0004799	cholelithiasis|Cholecystitis	cholelithiasis	EFO_0004799	Digestive system disorder	Gastrointestinal	PGS002072	EFO_0004799	cholelithiasis|Cholecystitis	cholelithiasis	EFO_0004799	Digestive system disorder	Gastrointestinal
0.9409	4.03E-216	2.84E-212	PGS002752	EFO_0007878	alcohol consumption measurement	alcohol consumption measurement	EFO_0007878	Other measurement	Psychiatric	PGS000203	EFO_0007878	alcohol consumption measurement	alcohol consumption measurement	EFO_0007878	Other measurement	Psychiatric
0.9409	5.27E-216	3.70E-212	PGS002138	EFO_0004312	vital capacity	vital capacity	EFO_0004312	Other measurement	Activities	PGS001922	EFO_0004312	vital capacity	vital capacity	EFO_0004312	Other measurement	Activities
0.9408	6.28E-216	4.41E-212	PGS000955	EFO_0004514	bone quantitative ultrasound measurement	bone quantitative ultrasound measurement	EFO_0004514	Other measurement	Skeletal	PGS000956	EFO_0004514	bone quantitative ultrasound measurement	measurement	EFO_0004514	Other measurement	Skeletal
0.9408	6.41E-216	4.49E-212	PGS000955	EFO_0004514	bone quantitative ultrasound measurement	bone quantitative ultrasound measurement	EFO_0004514	Other measurement	Skeletal	PGS001039	EFO_0009270	heel bone mineral density	heel bone mineral density	EFO_0009270	Other measurement	Skeletal
0.9408	6.61E-216	4.63E-212	PGS002219	EFO_0004536	total blood protein measurement	total blood protein measurement	EFO_0004536	Hematological measurement	Hematological	PGS002001	EFO_0004536	total blood protein measurement	total blood protein measurement	EFO_0004536	Hematological measurement	Hematological
0.9407	9.00E-216	6.29E-212	PGS001169	EFO_0004995	lean body mass	lean body mass	EFO_0004995	Other measurement	Skeletal	PGS001235	EFO_0004324	body weights and measures	body weights and measures	EFO_0004324	Body measurement	Body measurement
0.9407	9.48E-216	6.61E-212	PGS003262	EFO_0010472	creatine measurement|glomerular filtration rate	creatine measurement	EFO_0010472	Other measurement	Metabolic	PGS003277	EFO_0010472	creatine measurement|glomerular filtration rate	creatine measurement	EFO_0010472	Other measurement	Metabolic
0.9407	1.01E-215	7.07E-212	PGS000302	EFO_0006336	diastolic blood pressure	diastolic blood pressure	EFO_0006336	Other measurement	Cardiovascular	PGS002258	EFO_0006336	diastolic blood pressure	diastolic blood pressure	EFO_0006336	Other measurement	Cardiovascular
0.9406	1.56E-215	1.08E-211	PGS001962	EFO_0004308	leukocyte count	leukocyte count	EFO_0004308	Hematological measurement	Hematological	PGS002180	EFO_0004308	leukocyte count	leukocyte count	EFO_0004308	Hematological measurement	Hematological
0.9405	1.92E-215	1.33E-211	PGS001841	EFO_0003911	atrial flutter|atrial fibrillation	atrial flutter	EFO_0003911	Cardiovascular disease	Cardiovascular	PGS002050	EFO_0003911	atrial flutter|atrial fibrillation	atrial flutter	EFO_0003911	Cardiovascular disease	Cardiovascular
0.9403	3.59E-215	2.49E-211	PGS002393	EFO_0000400	diabetes mellitus	diabetes mellitus	EFO_0000400	Digestive system disorder	Gastrointestinal	PGS002592	EFO_0001379	endocrine system disease|diabetes mellitus	endocrine system disease	EFO_0001379	Other disease	Metabolic
0.9403	3.59E-215	2.49E-211	PGS002393	EFO_0000400	diabetes mellitus	diabetes mellitus	EFO_0000400	Metabolic disorder	Metabolic	PGS002592	EFO_0001379	endocrine system disease|diabetes mellitus	endocrine system disease	EFO_0001379	Other disease	Metabolic
0.9403	4.22E-215	2.92E-211	PGS001745	EFO_0006930	brain volume measurement|neuroimaging measurement	brain volume measurement	EFO_0006930	Other measurement	Neurological	PGS001715	EFO_0006930	measurement	brain volume measurement	EFO_0006930	Other measurement	Neurological
0.9403	4.36E-215	3.01E-211	PGS001146	EFO_0005409	fat body mass	fat body mass	EFO_0005409	Other measurement	Body measurement	PGS001002	EFO_0009805	whole body water mass	whole body water mass	EFO_0009805	Other measurement	Body measurement
0.9402	5.47E-215	3.77E-211	PGS002543	EFO_0001379	endocrine system disease|diabetes mellitus	endocrine system disease	EFO_0001379	Other disease	Metabolic	PGS002596	EFO_0004468	glucose measurement	glucose measurement	EFO_0004468	Other measurement	Metabolic
0.9402	5.83E-215	4.01E-211	PGS002543	EFO_0001379	endocrine system disease|diabetes mellitus	endocrine system disease	EFO_0001379	Other disease	Metabolic	PGS002589	EFO_0000400	diabetes mellitus	diabetes mellitus	EFO_0000400	Digestive system disorder	Gastrointestinal
0.9402	5.83E-215	4.01E-211	PGS002543	EFO_0001379	endocrine system disease|diabetes mellitus	endocrine system disease	EFO_0001379	Other disease	Metabolic	PGS002589	EFO_0000400	diabetes mellitus	diabetes mellitus	EFO_0000400	Metabolic disorder	Metabolic
0.94	1.18E-214	8.12E-211	PGS001990	EFO_0004526	mean corpuscular volume	mean corpuscular volume	EFO_0004526	Hematological measurement	Hematological	PGS001989	EFO_0004527	mean corpuscular hemoglobin	mean corpuscular hemoglobin	EFO_0004527	Hematological measurement	Hematological
0.9399	1.72E-214	1.18E-210	PGS001745	EFO_0006930	brain volume measurement|neuroimaging measurement	brain volume measurement	EFO_0006930	Other measurement	Neurological	PGS001730	EFO_0006930	measurement	brain volume measurement	EFO_0006930	Other measurement	Neurological
0.9398	2.21E-214	1.51E-210	PGS000955	EFO_0004514	bone quantitative ultrasound measurement	bone quantitative ultrasound measurement	EFO_0004514	Other measurement	Skeletal	PGS001404	EFO_0009270	heel bone mineral density	heel bone mineral density	EFO_0009270	Other measurement	Skeletal
0.9398	2.22E-214	1.52E-210	PGS002194	EFO_0007986	reticulocyte count	reticulocyte count	EFO_0007986	Hematological measurement	Hematological	PGS001976	EFO_0007986	reticulocyte count	reticulocyte count	EFO_0007986	Hematological measurement	Hematological
0.9398	2.93E-214	2.00E-210	PGS002846	EFO_0004340	body mass index	body mass index	EFO_0004340	Body measurement	Body measurement	PGS002856	EFO_0004340	body mass index	body mass index	EFO_0004340	Body measurement	Body measurement
0.9396	4.91E-214	3.35E-210	PGS001275	EFO_0003839	retinopathy	retinopathy	EFO_0003839	Neurological disorder	Neurological	PGS001013	EFO_0009606	macular degeneration	macular degeneration	EFO_0009606	Neurological disorder	Neurological
0.9395	7.05E-214	4.80E-210	PGS002311	MONDO_0004979	asthma	asthma	MONDO_0004979	Other disease	Respiratory	PGS002347	EFO_0000684	respiratory system disease	respiratory system disease	EFO_0000684	Other disease	Skeletal
0.9394	1.01E-213	6.85E-210	PGS001235	EFO_0004324	body weights and measures	body weights and measures	EFO_0004324	Body measurement	Body measurement	PGS001108	EFO_0007777	base metabolic rate measurement	base metabolic rate measurement	EFO_0007777	Other measurement	Metabolic
0.9393	1.63E-213	1.10E-209	PGS001169	EFO_0004995	lean body mass	lean body mass	EFO_0004995	Other measurement	Skeletal	PGS001146	EFO_0005409	fat body mass	fat body mass	EFO_0005409	Other measurement	Body measurement
0.9393	1.84E-213	1.25E-209	PGS002142	EFO_0004509	hemoglobin measurement	hemoglobin measurement	EFO_0004509	Hematological measurement	Hematological	PGS002141	EFO_0004348	hematocrit	hematocrit	EFO_0004348	Hematological measurement	Hematological
0.9392	1.99E-213	1.34E-209	PGS001265	EFO_0003907	deep vein thrombosis	deep vein thrombosis	EFO_0003907	Other trait	Cardiovascular	PGS001264	EFO_0003907	deep vein thrombosis	deep vein thrombosis	EFO_0003907	Other trait	Cardiovascular
0.9392	2.09E-213	1.41E-209	PGS002393	EFO_0000400	diabetes mellitus	diabetes mellitus	EFO_0000400	Digestive system disorder	Gastrointestinal	PGS002622	MONDO_0005148	type 2 diabetes mellitus	type 2 diabetes mellitus	MONDO_0005148	Digestive system disorder	Gastrointestinal
0.9392	2.09E-213	1.41E-209	PGS002393	EFO_0000400	diabetes mellitus	diabetes mellitus	EFO_0000400	Metabolic disorder	Metabolic	PGS002622	MONDO_0005148	type 2 diabetes mellitus	type 2 diabetes mellitus	MONDO_0005148	Metabolic disorder	Metabolic
0.9392	2.09E-213	1.41E-209	PGS002393	EFO_0000400	diabetes mellitus	diabetes mellitus	EFO_0000400	Digestive system disorder	Gastrointestinal	PGS002622	MONDO_0005148	type 2 diabetes mellitus	type 2 diabetes mellitus	MONDO_0005148	Metabolic disorder	Metabolic
0.9392	2.09E-213	1.41E-209	PGS002393	EFO_0000400	diabetes mellitus	diabetes mellitus	EFO_0000400	Metabolic disorder	Metabolic	PGS002622	MONDO_0005148	type 2 diabetes mellitus	type 2 diabetes mellitus	MONDO_0005148	Digestive system disorder	Gastrointestinal
0.9391	3.10E-213	2.09E-209	PGS001947	EFO_0004617	cystatin C measurement	cystatin C measurement	EFO_0004617	Other measurement	Metabolic	PGS002165	EFO_0004617	cystatin C measurement	cystatin C measurement	EFO_0004617	Other measurement	Metabolic
0.9391	3.43E-213	2.31E-209	PGS000681	EFO_0004570	bilirubin measurement	bilirubin measurement	EFO_0004570	Other measurement	Metabolic	PGS002032	Orphanet_309816	Disorder of bilirubin metabolism and excretion	excretion	Orphanet_309816	Metabolic disorder	Metabolic
0.939	5.49E-213	3.69E-209	PGS001961	EFO_0005106	body composition measurement	body composition measurement	EFO_0005106	Body measurement	Body measurement	PGS002179	EFO_0005106	body composition measurement	body composition measurement	EFO_0005106	Body measurement	Body measurement
0.939	5.74E-213	3.86E-209	PGS002178	EFO_0004627	IGF-1 measurement	IGF-1 measurement	EFO_0004627	Other measurement	Metabolic	PGS001960	EFO_0004627	IGF-1 measurement	IGF-1 measurement	EFO_0004627	Other measurement	Metabolic
0.9388	9.32E-213	6.25E-209	PGS001929	EFO_0004339	body height	body height	EFO_0004339	Body measurement	Body measurement	PGS002146	EFO_0004339	body height	body height	EFO_0004339	Body measurement	Body measurement
0.9388	9.47E-213	6.34E-209	PGS001987	EFO_0007825	balding measurement	balding measurement	EFO_0007825	Other measurement	Activities	PGS002204	EFO_0007825	balding measurement	balding measurement	EFO_0007825	Other measurement	Activities
0.9387	1.28E-212	8.59E-209	PGS000511	EFO_0000305	breast carcinoma	breast carcinoma	EFO_0000305	Cancer	Neoplasms	PGS000512	EFO_0000305	breast carcinoma	breast carcinoma	EFO_0000305	Cancer	Neoplasms
0.9386	1.82E-212	1.22E-208	PGS003293	EFO_0004617	cystatin C measurement|glomerular filtration rate	cystatin C measurement	EFO_0004617	Other measurement	Metabolic	PGS003308	EFO_0004617	cystatin C measurement|glomerular filtration rate	cystatin C measurement	EFO_0004617	Other measurement	Metabolic
0.9386	2.43E-212	1.62E-208	PGS002852	EFO_0004340	body mass index	body mass index	EFO_0004340	Body measurement	Body measurement	PGS002842	EFO_0004340	body mass index	body mass index	EFO_0004340	Body measurement	Body measurement
0.9384	4.82E-212	3.21E-208	PGS003140	EFO_0004574	total cholesterol measurement	total cholesterol measurement	EFO_0004574	Lipid or lipoprotein measurement	Metabolic	PGS003141	EFO_0004574	total cholesterol measurement	total cholesterol measurement	EFO_0004574	Lipid or lipoprotein measurement	Metabolic

0.9383	5.80E-212	3.86E-208	PGS002550	EFO_0004541	HbA1c measurement	HbA1c measurement	EFO_0004541	Hematological measurement	Hematological	PGS002403	EFO_0004541	HbA1c measurement	HbA1c measurement	EFO_0004541	Hematological measurement	Hematological
0.9383	6.04E-212	4.02E-208	PGS002547	EFO_0004468	glucose measurement	glucose measurement	EFO_0004468	Other measurement	Metabolic	PGS002396	EFO_0001379	endocrine system disease|diabetes mellitus	endocrine system disease	EFO_0001379	Other disease	Metabolic
0.9382	8.18E-212	5.43E-208	PGS002009	EFO_0006335	systolic blood pressure	systolic blood pressure	EFO_0006335	Other measurement	Cardiovascular	PGS002228	EFO_0006335	systolic blood pressure	systolic blood pressure	EFO_0006335	Other measurement	Cardiovascular
0.9381	1.07E-211	7.08E-208	PGS000495	EFO_0000305	breast carcinoma	breast carcinoma	EFO_0000305	Cancer	Neoplasms	PGS000496	EFO_0000305	breast carcinoma	breast carcinoma	EFO_0000305	Cancer	Neoplasms
0.9381	1.19E-211	7.90E-208	PGS001146	EFO_0005409	fat body mass	fat body mass	EFO_0005409	Other measurement	Body measurement	PGS001108	EFO_0007777	base metabolic rate measurement	base metabolic rate measurement	EFO_0007777	Other measurement	Metabolic
0.938	1.91E-211	1.26E-207	PGS002540	EFO_0000400	diabetes mellitus	diabetes mellitus	EFO_0000400	Digestive system disorder	Gastrointestinal	PGS002426	MONDO_0005148	type 2 diabetes mellitus	type 2 diabetes mellitus	MONDO_0005148	Metabolic disorder	Metabolic
0.938	1.91E-211	1.26E-207	PGS002540	EFO_0000400	diabetes mellitus	diabetes mellitus	EFO_0000400	Metabolic disorder	Metabolic	PGS002426	MONDO_0005148	type 2 diabetes mellitus	type 2 diabetes mellitus	MONDO_0005148	Metabolic disorder	Metabolic
0.938	1.91E-211	1.26E-207	PGS002540	EFO_0000400	diabetes mellitus	diabetes mellitus	EFO_0000400	Metabolic disorder	Metabolic	PGS002426	MONDO_0005148	type 2 diabetes mellitus	type 2 diabetes mellitus	MONDO_0005148	Digestive system disorder	Gastrointestinal
0.938	1.91E-211	1.26E-207	PGS002540	EFO_0000400	diabetes mellitus	diabetes mellitus	EFO_0000400	Digestive system disorder	Gastrointestinal	PGS002426	MONDO_0005148	type 2 diabetes mellitus	type 2 diabetes mellitus	MONDO_0005148	Digestive system disorder	Gastrointestinal
0.9378	3.99E-211	2.64E-207	PGS003007	EFO_0000537	hypertension	hypertension	EFO_0000537	Cardiovascular disease	Cardiovascular	PGS003012	EFO_0000537	hypertension	hypertension	EFO_0000537	Cardiovascular disease	Cardiovascular
0.9377	5.06E-211	3.33E-207	PGS002206	EFO_0004527	mean corpuscular hemoglobin	mean corpuscular hemoglobin	EFO_0004527	Hematological measurement	Hematological	PGS002207	EFO_0004526	mean corpuscular volume	mean corpuscular volume	EFO_0004526	Hematological measurement	Hematological
0.9376	8.33E-211	5.48E-207	PGS002953	EFO_0004465	fasting blood glucose measurement	fasting blood glucose measurement	EFO_0004465	Other measurement	Metabolic	PGS002933	EFO_0004465	fasting blood glucose measurement	fasting blood glucose measurement	EFO_0004465	Other measurement	Metabolic
0.9374	1.27E-210	8.38E-207	PGS002092	EFO_0004229	Dupuytren Contracture	Dupuytren Contracture	EFO_0004229	Other disease	Others	PGS001880	EFO_0004229	Dupuytren Contracture	Dupuytren Contracture	EFO_0004229	Other disease	Others
0.9374	1.38E-210	9.07E-207	PGS002471	EFO_0004279	suntan	suntan	EFO_0004279	Other trait	Dermatological	PGS002569	EFO_0004279	suntan	suntan	EFO_0004279	Other trait	Dermatological
0.9371	3.91E-210	2.57E-206	PGS003135	EFO_0004574	total cholesterol measurement	total cholesterol measurement	EFO_0004574	Lipid or lipoprotein measurement	Metabolic	PGS003136	EFO_0004574	total cholesterol measurement	total cholesterol measurement	EFO_0004574	Lipid or lipoprotein measurement	Metabolic
0.9369	7.91E-210	5.18E-206	PGS002596	EFO_0004468	glucose measurement	glucose measurement	EFO_0004468	Other measurement	Metabolic	PGS002400	EFO_0004468	glucose measurement	glucose measurement	EFO_0004468	Other measurement	Metabolic
0.9366	2.07E-209	1.35E-205	PGS000014	MONDO_0005148	type 2 diabetes mellitus	type 2 diabetes mellitus	MONDO_0005148	Metabolic disorder	Metabolic	PGS002243	MONDO_0005148	type 2 diabetes mellitus	type 2 diabetes mellitus	MONDO_0005148	Metabolic disorder	Metabolic
0.9366	2.07E-209	1.35E-205	PGS000014	MONDO_0005148	type 2 diabetes mellitus	type 2 diabetes mellitus	MONDO_0005148	Digestive system disorder	Gastrointestinal	PGS002243	MONDO_0005148	type 2 diabetes mellitus	type 2 diabetes mellitus	MONDO_0005148	Metabolic disorder	Metabolic
0.9366	2.07E-209	1.35E-205	PGS000014	MONDO_0005148	type 2 diabetes mellitus	type 2 diabetes mellitus	MONDO_0005148	Metabolic disorder	Metabolic	PGS002243	MONDO_0005148	type 2 diabetes mellitus	type 2 diabetes mellitus	MONDO_0005148	Digestive system disorder	Gastrointestinal
0.9366	2.07E-209	1.35E-205	PGS000014	MONDO_0005148	type 2 diabetes mellitus	type 2 diabetes mellitus	MONDO_0005148	Digestive system disorder	Gastrointestinal	PGS002243	MONDO_0005148	type 2 diabetes mellitus	type 2 diabetes mellitus	MONDO_0005148	Digestive system disorder	Gastrointestinal
0.9364	5.18E-209	3.38E-205	PGS001860	EFO_0001421	liver disease	liver disease	EFO_0001421	Digestive system disorder	Gastrointestinal	PGS002071	EFO_0001421	liver disease	liver disease	EFO_0001421	Digestive system disorder	Gastrointestinal
0.9363	5.88E-209	3.84E-205	PGS001983	EFO_0004342	waist circumference	waist circumference	EFO_0004342	Body measurement	Body measurement	PGS002201	EFO_0004342	waist circumference	waist circumference	EFO_0004342	Body measurement	Body measurement
0.9362	8.25E-209	5.37E-205	PGS001030	EFO_0009448	pulmonary fibrosis	pulmonary fibrosis	EFO_0009448	Cancer	Neoplasms	PGS001251	EFO_0004244	interstitial lung disease	interstitial lung disease	EFO_0004244	Other disease	Respiratory
-0.9362	9.00E-209	5.85E-205	PGS001095	EFO_0007822	hair colour measurement	hair colour measurement	EFO_0007822	Other measurement	Dermatological	PGS001093	EFO_0007822	hair colour measurement	hair colour measurement	EFO_0007822	Other measurement	Dermatological
0.9362	9.36E-209	6.08E-205	PGS003302	EFO_0004617	cystatin C measurement|glomerular filtration rate	cystatin C measurement	EFO_0004617	Other measurement	Metabolic	PGS003317	EFO_0004617	cystatin C measurement|glomerular filtration rate	cystatin C measurement	EFO_0004617	Other measurement	Metabolic
0.9361	1.25E-208	8.11E-205	PGS002530	MONDO_0004979	asthma	asthma	MONDO_0004979	Other disease	Respiratory	PGS002579	MONDO_0004979	asthma	asthma	MONDO_0004979	Other disease	Respiratory
0.9359	2.52E-208	1.63E-204	PGS002177	EFO_0010700	reticulocyte measurement	reticulocyte measurement	EFO_0010700	Hematological measurement	Hematological	PGS001959	EFO_0010700	reticulocyte measurement	reticulocyte measurement	EFO_0010700	Hematological measurement	Hematological
0.9358	3.63E-208	2.35E-204	PGS002610	EFO_0007660	neuroticism measurement	neuroticism measurement	EFO_0007660	Other measurement	Psychiatric	PGS002561	EFO_0007660	neuroticism measurement	neuroticism measurement	EFO_0007660	Other measurement	Psychiatric
0.9357	4.65E-208	3.00E-204	PGS001639	EFO_0008080	measurement	cerebrospinal fluid volume measurement	EFO_0008080	Other measurement	Neurological	PGS001070	EFO_0008080	cerebrospinal fluid volume measurement	measurement	EFO_0008080	Other measurement	Neurological
0.9357	5.09E-208	3.28E-204	PGS003035	EFO_0004611	low density lipoprotein cholesterol measurement	low density lipoprotein cholesterol measurement	EFO_0004611	Lipid or lipoprotein measurement	Metabolic	PGS003036	EFO_0004611	low density lipoprotein cholesterol measurement	measurement	EFO_0004611	Cardiovascular measurement	Cardiovascular
0.9357	5.09E-208	3.28E-204	PGS003035	EFO_0004611	low density lipoprotein cholesterol measurement	low density lipoprotein cholesterol measurement	EFO_0004611	Lipid or lipoprotein measurement	Metabolic	PGS003036	EFO_0004611	low density lipoprotein cholesterol measurement	measurement	EFO_0004611	Lipid or lipoprotein measurement	Metabolic
0.9357	5.09E-208	3.28E-204	PGS003035	EFO_0004611	low density lipoprotein cholesterol measurement	low density lipoprotein cholesterol measurement	EFO_0004611	Cardiovascular measurement	Cardiovascular	PGS003036	EFO_0004611	low density lipoprotein cholesterol measurement	measurement	EFO_0004611	Cardiovascular measurement	Cardiovascular
0.9357	5.09E-208	3.28E-204	PGS003035	EFO_0004611	low density lipoprotein cholesterol measurement	low density lipoprotein cholesterol measurement	EFO_0004611	Cardiovascular measurement	Cardiovascular	PGS003036	EFO_0004611	low density lipoprotein cholesterol measurement	measurement	EFO_0004611	Lipid or lipoprotein measurement	Metabolic
0.9356	6.42E-208	4.14E-204	PGS002246	EFO_0000756	melanoma	melanoma	EFO_0000756	Cancer	Neoplasms	PGS002247	EFO_0000756	melanoma	melanoma	EFO_0000756	Cancer	Neoplasms
0.9356	7.99E-208	5.14E-204	PGS000888	EFO_0004611	low density lipoprotein cholesterol measurement	low density lipoprotein cholesterol measurement	EFO_0004611	Cardiovascular measurement	Cardiovascular	PGS000892	EFO_0004611	low density lipoprotein cholesterol measurement	measurement	EFO_0004611	Lipid or lipoprotein measurement	Metabolic
0.9356	7.99E-208	5.14E-204	PGS000888	EFO_0004611	low density lipoprotein cholesterol measurement	low density lipoprotein cholesterol measurement	EFO_0004611	Lipid or lipoprotein measurement	Metabolic	PGS000892	EFO_0004611	low density lipoprotein cholesterol measurement	measurement	EFO_0004611	Cardiovascular measurement	Cardiovascular
0.9356	7.99E-208	5.14E-204	PGS000888	EFO_0004611	low density lipoprotein cholesterol measurement	low density lipoprotein cholesterol measurement	EFO_0004611	Lipid or lipoprotein measurement	Metabolic	PGS000892	EFO_0004611	low density lipoprotein cholesterol measurement	measurement	EFO_0004611	Lipid or lipoprotein measurement	Metabolic
0.9356	7.99E-208	5.14E-204	PGS000888	EFO_0004611	low density lipoprotein cholesterol measurement	low density lipoprotein cholesterol measurement	EFO_0004611	Cardiovascular measurement	Cardiovascular	PGS000892	EFO_0004611	low density lipoprotein cholesterol measurement	measurement	EFO_0004611	Cardiovascular measurement	Cardiovascular
0.9354	1.63E-207	1.05E-203	PGS000344	EFO_0000305	breast carcinoma	breast carcinoma	EFO_0000305	Cancer	Neoplasms	PGS000345	EFO_0000305	breast carcinoma	breast carcinoma	EFO_0000305	Cancer	Neoplasms
0.9353	2.35E-207	1.51E-203	PGS000618	EFO_0000326	central nervous system cancer	central nervous system cancer	EFO_0000326	Neurological disorder	Neurological	PGS000619	EFO_0000326	central nervous system cancer	central nervous system cancer	EFO_0000326	Cancer	Neoplasms
0.9353	2.35E-207	1.51E-203	PGS000618	EFO_0000326	central nervous system cancer	central nervous system cancer	EFO_0000326	Cancer	Neoplasms	PGS000619	EFO_0000326	central nervous system cancer	central nervous system cancer	EFO_0000326	Cancer	Neoplasms
0.9353	2.35E-207	1.51E-203	PGS000618	EFO_0000326	central nervous system cancer	central nervous system cancer	EFO_0000326	Cancer	Neoplasms	PGS000619	EFO_0000326	central nervous system cancer	central nervous system cancer	EFO_0000326	Neurological disorder	Neurological
0.9353	2.35E-207	1.51E-203	PGS000618	EFO_0000326	central nervous system cancer	central nervous system cancer	EFO_0000326	Neurological disorder	Neurological	PGS000619	EFO_0000326	central nervous system cancer	central nervous system cancer	EFO_0000326	Neurological disorder	Neurological
0.9353	2.40E-207	1.54E-203	PGS001943	EFO_0004340	body mass index	body mass index	EFO_0004340	Body measurement	Body measurement	PGS002161	EFO_0004340	body mass index	body mass index	EFO_0004340	Body measurement	Body measurement
0.9351	3.89E-207	2.49E-203	PGS000504	EFO_0000305	breast carcinoma	breast carcinoma	EFO_0000305	Cancer	Neoplasms	PGS000503	EFO_0000305	breast carcinoma	breast carcinoma	EFO_0000305	Cancer	Neoplasms
0.9351	4.16E-207	2.66E-203	PGS001984	EFO_0009805	whole body water mass	whole body water mass	EFO_0009805	Other measurement	Body measurement	PGS002202	EFO_0009805	whole body water mass	whole body water mass	EFO_0009805	Other measurement	Body measurement
0.935	6.30E-207	4.02E-203	PGS002848	EFO_0004340	body mass index	body mass index	EFO_0004340	Other measurement	Body measurement	PGS002843	EFO_0004340	body mass index	body mass index	EFO_0004340	Body measurement	Body measurement
0.9349	9.33E-207	5.95E-203	PGS002099	EFO_0004535	serum albumin measurement	serum albumin measurement	EFO_0004535	Other measurement	Metabolic	PGS001886	EFO_0004535	serum albumin measurement	serum albumin measurement	EFO_0004535	Other measurement	Metabolic
0.9348	1.03E-206	6.57E-203	PGS000501	EFO_0000305	breast carcinoma	breast carcinoma	EFO_0000305	Cancer	Neoplasms	PGS000502	EFO_0000305	breast carcinoma	breast carcinoma	EFO_0000305	Cancer	Neoplasms
0.9347	1.39E-206	8.82E-203	PGS002609	EFO_0005091	monocyte count	monocyte count	EFO_0005091	Hematological measurement	Hematological	PGS002413	EFO_0005091	monocyte count	monocyte count	EFO_0005091	Hematological measurement	Hematological
0.9347	1.49E-206	9.47E-203	PGS001169	EFO_0004995	lean body mass	lean body mass	EFO_0004995	Other measurement	Skeletal	PGS001158	EFO_0005106	body composition measurement	body composition measurement	EFO_0005106	Body measurement	Body measurement
0.9346	2.15E-206	1.36E-202	PGS001929	EFO_0004339	body height	body height	EFO_0004339	Body measurement	Body measurement	PGS002332	EFO_0004339	body height	body height	EFO_0004339	Body measurement	Body measurement
0.9345	3.48E-206	2.20E-202	PGS001169	EFO_0004995	lean body mass	lean body mass	EFO_0004995	Other measurement	Skeletal	PGS001166	EFO_0004995	lean body mass	lean body mass	EFO_0004995	Other measurement	Skeletal
0.9344	4.66E-206	2.95E-202	PGS003013	EFO_0000537	hypertension	hypertension	EFO_0000537	Cardiovascular disease	Cardiovascular	PGS003028	EFO_0000537	hypertension	hypertension	EFO_0000537	Cardiovascular disease	Cardiovascular
0.9343	7.15E-206	4.51E-202	PGS002553	HP_0003124	Hypercholesterolemia	Hypercholesterolemia	HP_0003124	Other trait	Metabolic	PGS002602	HP_0003124	Hypercholesterolemia	Hypercholesterolemia	HP_0003124	Other trait	Metabolic
0.9341	1.14E-205	7.19E-202	PGS000697	EFO_0004570	bilirubin measurement	bilirubin measurement	EFO_0004570	Other measurement	Metabolic	PGS000924	MONDO_0037821	disease	porphyrin metabolism disease	MONDO_0037821	Metabolic disorder	Metabolic
0.9341	1.32E-205	8.33E-202	PGS000638	EFO_0000183	Hodgkins lymphoma	Hodgkins lymphoma	EFO_0000183	Cancer	Neoplasms	PGS000639	EFO_0000183	Hodgkins lymphoma	Hodgkins lymphoma	EFO_0000183	Cancer	Neoplasms
0.9339	2.55E-205	1.60E-201	PGS001145	EFO_0005409	fat body mass	fat body mass	EFO_0005409	Other measurement	Body measurement	PGS001149	EFO_0005409	fat body mass	fat body mass	EFO_0005409	Other measurement	Body measurement
0.9338	3.14E-205	1.97E-201	PGS002970	EFO_0004339	body height	body height	EFO_0004339	Body measurement	Body measurement	PGS002971	EFO_0004339	body height	body height	EFO_0004339	Body measurement	Body measurement
0.9338	3.78E-205	2.37E-201	PGS002571	EFO_0004574	total cholesterol measurement	total cholesterol measurement	EFO_0004574	Lipid or lipoprotein measurement	Metabolic	PGS002424	EFO_0004574	total cholesterol measurement	total cholesterol measurement	EFO_0004574	Lipid or lipoprotein measurement	Metabolic
0.9337	4.19E-205	2.62E-201	PGS001984	EFO_0009805	whole body water mass	whole body water mass	EFO_0009805	Other measurement	Body measurement	PGS002168	EFO_0004995	lean body mass	lean body mass	EFO_0004995	Other measurement	Skeletal
0.9335	7.69E-205	4.81E-201	PGS001169	EFO_0004995	lean body mass	lean body mass	EFO_0004995	Other measurement	Skeletal	PGS001159	EFO_0005106	body composition measurement	body composition measurement	EFO_0005106	Body measurement	Body measurement
0.9335	8.95E-205	5.59E-201	PGS002173	EFO_0009270	heel bone mineral density	heel bone mineral density	EFO_0009270	Other measurement	Skeletal	PGS001955	EFO_0009270	heel bone mineral density	heel bone mineral density	EFO_0009270	Other measurement	Skeletal
0.9334	1.16E-204	7.23E-201	PGS001950	EFO_0004995	lean body mass	lean body mass	EFO_0004995	Other measurement	Skeletal	PGS002168	EFO_0004995	lean body mass	lean body mass	EFO_0004995	Other measurement	Skeletal
0.9334	1.21E-204	7.53E-201	PGS003190	EFO_0004631	vitamin D measurement	vitamin D measurement	EFO_0004631	Lipid or lipoprotein measurement	Metabolic	PGS003196	EFO_0004631	vitamin D measurement	vitamin D measurement	EFO_0004631	Lipid or lipoprotein measurement	Metabolic
0.9334	1.27E-204	7.91E-201	PGS001489	EFO_0006930	brain volume measurement|neuroimaging measurement	brain volume measurement	EFO_0006930	Other measurement	Neurological	PGS001683	EFO_0006930	measurement	brain volume measurement	EFO_0006930	Other measurement	Neurological
0.9334	1.32E-204	8.20E-201	PGS001954	EFO_0004612	high density lipoprotein cholesterol measurement	high density lipoprotein cholesterol measurement	EFO_0004612	Lipid or lipoprotein measurement	Metabolic	PGS002172	EFO_0004612	high density lipoprotein cholesterol measurement	measurement	EFO_0004612	Lipid or lipoprotein measurement	Metabolic
0.9331	3.12E-204	1.93E-200	PGS000509	EFO_0000305	breast carcinoma	breast carcinoma	EFO_0000305	Cancer	Neoplasms	PGS000540	EFO_0000305	breast carcinoma	breast carcinoma	EFO_0000305	Cancer	Neoplasms
0.9326	1.58E-203	9.82E-200	PGS002143	EFO_0006941	grip strength measurement	grip strength measurement	EFO_0006941	Other measurement	Muscular	PGS001927	EFO_0006941	grip strength measurement	grip strength measurement	EFO_0006941	Other measurement	Muscular
0.9326	1.80E-203	1.11E-199	PGS001950	EFO_0004995	lean body mass	lean body mass	EFO_0004995	Other measurement	Skeletal	PGS002202	EFO_0009805	whole body water mass	whole body water mass	EFO_0009805	Other measurement	Body measurement
0.9326	1.89E-203	1.17E-199	PGS001169	EFO_0004995	lean body mass	lean body mass	EFO_0004995	Other measurement	Skeletal	PGS001167	EFO_0004995	lean body mass	lean body mass	EFO_0004995	Other measurement	Skeletal
0.9325	1.98E-203	1.22E-199	PGS002445	EFO_0001379	endocrine system disease|diabetes mellitus	endocrine system disease	EFO_0001379	Other disease	Metabolic	PGS002393	EFO_0000400	diabetes mellitus	diabetes mellitus	EFO_0000400	Metabolic disorder	Metabolic
0.9325	1.98E-203	1.22E-199	PGS002445	EFO_0001379	endocrine system disease|diabetes mellitus	endocrine system disease	EFO_0001379	Other disease	Metabolic	PGS002393	EFO_0000400	diabetes mellitus	diabetes mellitus	EFO_0000400	Digestive system disorder	Gastrointestinal
0.9325	2.64E-203	1.63E-199	PGS000071	MONDO_0004986	urinary bladder carcinoma	urinary bladder carcinoma	MONDO_0004986	Cancer	Neoplasms	PGS003335	MONDO_0004986	urinary bladder carcinoma	urinary bladder carcinoma	MONDO_0004986	Cancer	Neoplasms
0.9323	4.81E-203	2.96E-199	PGS001158	EFO_0005106	body composition measurement	body composition measurement	EFO_0005106	Body measurement	Body measurement	PGS001002	EFO_0009805	whole body water mass	whole body water mass	EFO_0009805	Other measurement	Body measurement
0.9322	5.49E-203	3.37E-199	PGS002393	EFO_0000400	diabetes mellitus	diabetes mellitus	EFO_0000400	Digestive system disorder	Gastrointestinal	PGS002589	EFO_0000400	diabetes mellitus	diabetes mellitus	EFO_0000400	Digestive system disorder	Gastrointestinal
0.9322	5.49E-203	3.37E-199	PGS002393	EFO_0000400	diabetes mellitus	diabetes mellitus	EFO_0000400	Metabolic disorder	Metabolic	PGS002589	EFO_0000400	diabetes mellitus	diabetes mellitus	EFO_0000400	Metabolic disorder	Metabolic
0.9322	5.49E-203	3.37E-199	PGS002393	EFO_0000400	diabetes mellitus	diabetes mellitus	EFO_0000400	Digestive system disorder	Gastrointestinal	PGS002589	EFO_0000400	diabetes mellitus	diabetes mellitus	EFO_0000400	Metabolic disorder	Metabolic
0.9322	5.49E-203	3.37E-199	PGS002393	EFO_0000400	diabetes mellitus	diabetes mellitus	EFO_0000400	Metabolic disorder	Metabolic	PGS002589	EFO_0000400	diabetes mellitus	diabetes mellitus	EFO_0000400	Digestive system disorder	Gastrointestinal
0.9322	5.84E-203	3.58E-199	PGS000945	HP_0000726	Dementia|Alzheimer disease	Dementia	HP_0000726	Other trait	Psychiatric	PGS001347	EFO_0009268	family history of Alzheimer's disease	family history of Alzheimer's disease	EFO_0009268	Other measurement	Neurological
0.9321	7.42E-203	4.54E-199	PGS001900	EFO_0006336	diastolic blood pressure	diastolic blood pressure	EFO_0006336	Other measurement	Cardiovascular	PGS002114	EFO_0006336	diastolic blood pressure	diastolic blood pressure	EFO_0006336	Other measurement	Cardiovascular
0.9321	8.23E-203	5.03E-199	PGS001956	EFO_0007772	calcaneal bone quantitative ultrasound measurement	calcaneal bone quantitative ultrasound measurement	EFO_0007772	Other measurement	Skeletal	PGS002174	EFO_0007772	measurement	measurement	EFO_0007772	Other measurement	Skeletal
0.932	1.05E-202	6.43E-199	PGS002339	EFO_0004527	mean corpuscular hemoglobin	mean corpuscular hemoglobin	EFO_0004527	Hematological measurement	Hematological	PGS001989	EFO_0004527	mean corpuscular hemoglobin	mean corpuscular hemoglobin	EFO_0004527	Hematological measurement	Hematological
0.932	1.14E-202	6.95E-199	PGS003313	EFO_0004617	cystatin C measurement|glomerular filtration rate	cystatin C measurement	EFO_0004617	Other measurement	Metabolic	PGS003309	EFO_0004617	cystatin C measurement|glomerular filtration rate	cystatin C measurement	EFO_0004617	Other measurement	Metabolic
0.932	1.33E-202	8.11E-199	PGS003191	EFO_0004631	vitamin D measurement	vitamin D measurement	EFO_0004631	Lipid or lipoprotein measurement	Metabolic	PGS003195	EFO_0004631	vitamin D measurement	vitamin D measurement	EFO_0004631	Lipid or lipoprotein measurement	Metabolic
0.932	1.35E-202	8.19E-199	PGS001169	EFO_0004995	lean body mass	lean body mass	EFO_0004995	Other measurement	Skeletal	PGS001002	EFO_0009805	whole body water mass	whole body water mass	EFO_0009805	Other measurement	Body measurement
0.9317	3.49E-202	2.12E-198	PGS002543	EFO_0001379	endocrine system disease|diabetes mellitus	endocrine system disease	EFO_0001379	Other disease	Metabolic	PGS002445	EFO_0001379	endocrine system disease|diabetes mellitus	endocrine system disease	EFO_0001379	Other disease	Metabolic
0.9315	5.17E-202	3.13E-198	PGS001149	EFO_0005409	fat body mass	fat body mass	EFO_0005409	Other measurement	Body measurement	PGS001144	EFO_0005409	fat body mass	fat body mass	EFO_0005409	Other measurement	Body measurement
0.9315	5.92E-202	3.58E-198	PGS002955	EFO_0004612	high density lipoprotein cholesterol measurement	high density lipoprotein cholesterol measurement	EFO_0004612	Lipid or lipoprotein measurement	Metabolic	PGS002960	EFO_0004612	high density lipoprotein cholesterol measurement	measurement	EFO_0004612	Lipid or lipoprotein measurement	Metabolic
0.9315	6.79E-202	4.11E-198	PGS001159	EFO_0005106	body composition measurement	body composition measurement	EFO_0005106	Body measurement	Body measurement	PGS001002	EFO_0009805	whole body water mass	whole body water mass	EFO_0009805	Other measurement	Body measurement
0.9314	6.87E-202	4.15E-198	PGS002589	EFO_0000400	diabetes mellitus	diabetes mellitus	EFO_0000400	Digestive system disorder	Gastrointestinal	PGS002396	EFO_0001379	endocrine system disease|diabetes mellitus	endocrine system disease	EFO_0001379	Other disease	Metabolic
0.9314	6.87E-202	4.15E-198	PGS002589	EFO_0000400	diabetes mellitus	diabetes mellitus	EFO_0000400	Metabolic disorder	Metabolic	PGS002396	EFO_0001379	endocrine system disease|diabetes mellitus	endocrine system disease	EFO_0001379	Other disease	Metabolic
0.9314	8.82E-202	5.32E-198	PGS001594	EFO_0005420	measurement	grey matter volume measurement	EFO_0005420	Other measurement	Neurological	PGS001593	EFO_0005420	measurement	grey matter volume measurement	EFO_0005420	Other measurement	Neurological
0.9311	2.02E-201	1.22E-197	PGS001838	EFO_0000537	hypertension	hypertension	EFO_0000537	Cardiovascular disease	Cardiovascular	PGS002047	EFO_0000537	hypertension	hypertension	EFO_0000537	Cardiovascular disease	Cardiovascular
0.931	2.72E-201	1.64E-197	PGS000541	MONDO_0002715	uterine cancer	uterine cancer	MONDO_0002715	Cancer	Neoplasms	PGS002735	EFO_1001512	endometrial carcinoma	endometrial carcinoma	EFO_1001512	Cancer	Neoplasms
0.931	3.08E-201	1.85E-197	PGS001160	EFO_0005106	body composition measurement	body composition measurement	EFO_0005106	Body measurement	Body measurement	PGS001108	EFO_0007777	base metabolic rate measurement	base metabolic rate measurement	EFO_0007777	Other measurement	Metabolic
0.931	3.34E-201	2.00E-197	PGS002169	EFO_0005409	fat body mass	fat body mass	EFO_0005409	Other measurement	Body measurement	PGS002133	EFO_0007800	body fat percentage	body fat percentage	EFO_0007800	Body measurement	Body measurement
0.9309	4.14E-201	2.48E-197	PGS001168	EFO_0004995	lean body mass	lean body mass	EFO_0004995	Other measurement	Skeletal	PGS001108	EFO_0007777	base metabolic rate measurement	base metabolic rate measurement	EFO_0007777	Other measurement	Metabolic
0.9308	4.87E-201	2.92E-197	PGS003411	EFO_0003870	brain aneurysm|female	brain aneurysm	EFO_0003870	Neurological disorder	Neurological	PGS003410	EFO_0003870	brain aneurysm|male	brain aneurysm	EFO_0003870	Neurological disorder	Neurological
0.9308	4.87E-201	2.92E-197	PGS003411	EFO_0003870	brain aneurysm|female	brain aneurysm	EFO_0003870	Cardiovascular disease	Cardiovascular	PGS003410	EFO_0003870	brain aneurysm|male	brain aneurysm	EFO_0003870	Neurological disorder	Neurological
0.9308	4.87E-201	2.92E-197	PGS003411	EFO_0003870	brain aneurysm|female	brain aneurysm	EFO_0003870	Neurological disorder	Neurological	PGS003410	EFO_0003870	brain aneurysm|male	brain aneurysm	EFO_0003870	Cardiovascular disease	Cardiovascular
0.9308	4.87E-201	2.92E-197	PGS003411	EFO_0003870	brain aneurysm|female	brain aneurysm	EFO_0003870	Cardiovascular disease	Cardiovascular	PGS003410	EFO_0003870	brain aneurysm|male	brain aneurysm	EFO_0003870	Cardiovascular disease	Cardiovascular
0.9308	5.26E-201	3.14E-197	PGS002471	EFO_0004279	suntan	suntan	EFO_0004279	Other trait	Dermatological	PGS002618	EFO_0004279	suntan	suntan	EFO_0004279	Other trait	Dermatological

0.9307	6.55E-201	3.91E-197	PGS002088	EFO_0000685	rheumatoid arthritis	rheumatoid arthritis	EFO_0000685	Immune system disorder	Immunological	PGS001875	EFO_0000685	rheumatoid arthritis	rheumatoid arthritis	EFO_0000685	Immune system disorder	Immunological
0.9306	1.10E-200	6.58E-197	PGS001488	EFO_0006930	brain volume measurement|neuroimaging measurement	brain volume measurement	EFO_0006930	Other measurement	Neurological	PGS001682	EFO_0006930	measurement	brain volume measurement	EFO_0006930	Other measurement	Neurological
0.9306	1.14E-200	6.78E-197	PGS003309	EFO_0004617	cystatin C measurement|glomerular filtration rate	cystatin C measurement	EFO_0004617	Other measurement	Metabolic	PGS003298	EFO_0004617	cystatin C measurement|glomerular filtration rate	cystatin C measurement	EFO_0004617	Other measurement	Metabolic
0.9304	2.22E-200	1.32E-196	PGS001167	EFO_0004995	lean body mass	lean body mass	EFO_0004995	Other measurement	Skeletal	PGS001002	EFO_0009805	whole body water mass	whole body water mass	EFO_0009805	Other measurement	Body measurement
0.9304	2.24E-200	1.33E-196	PGS001160	EFO_0005106	body composition measurement	body composition measurement	EFO_0005106	Body measurement	Body measurement	PGS001234	EFO_0004324	body weights and measures	body weights and measures	EFO_0004324	Body measurement	Body measurement
-0.9303	2.28E-200	1.35E-196	PGS001297	MONDO_0005147	type 1 diabetes mellitus	type 1 diabetes mellitus	MONDO_0005147	Digestive system disorder	Gastrointestinal	PGS001371	EFO_0004918	age at diagnosis|diabetes mellitus	age at diagnosis	EFO_0004918	Other measurement	Metabolic
-0.9303	2.28E-200	1.35E-196	PGS001297	MONDO_0005147	type 1 diabetes mellitus	type 1 diabetes mellitus	MONDO_0005147	Immune system disorder	Immunological	PGS001371	EFO_0004918	age at diagnosis|diabetes mellitus	age at diagnosis	EFO_0004918	Other measurement	Metabolic
-0.9303	2.28E-200	1.35E-196	PGS001297	MONDO_0005147	type 1 diabetes mellitus	type 1 diabetes mellitus	MONDO_0005147	Metabolic disorder	Metabolic	PGS001371	EFO_0004918	age at diagnosis|diabetes mellitus	age at diagnosis	EFO_0004918	Other measurement	Metabolic
0.9303	2.28E-200	1.35E-196	PGS001310	EFO_0000685	rheumatoid arthritis	rheumatoid arthritis	EFO_0000685	Immune system disorder	Immunological	PGS001309	EFO_0000685	rheumatoid arthritis	rheumatoid arthritis	EFO_0000685	Immune system disorder	Immunological
0.9303	2.62E-200	1.55E-196	PGS003035	EFO_0004611	low density lipoprotein cholesterol measurement	low density lipoprotein cholesterol measurement	EFO_0004611	Cardiovascular measurement	Cardiovascular	PGS003031	EFO_0004611	low density lipoprotein cholesterol measurement	measurement	EFO_0004611	Lipid or lipoprotein measurement	Metabolic
0.9303	2.62E-200	1.55E-196	PGS003035	EFO_0004611	low density lipoprotein cholesterol measurement	low density lipoprotein cholesterol measurement	EFO_0004611	Lipid or lipoprotein measurement	Metabolic	PGS003031	EFO_0004611	low density lipoprotein cholesterol measurement	measurement	EFO_0004611	Cardiovascular measurement	Cardiovascular
0.9303	2.62E-200	1.55E-196	PGS003035	EFO_0004611	low density lipoprotein cholesterol measurement	low density lipoprotein cholesterol measurement	EFO_0004611	Lipid or lipoprotein measurement	Metabolic	PGS003031	EFO_0004611	low density lipoprotein cholesterol measurement	measurement	EFO_0004611	Lipid or lipoprotein measurement	Metabolic
0.9303	2.62E-200	1.55E-196	PGS003035	EFO_0004611	low density lipoprotein cholesterol measurement	low density lipoprotein cholesterol measurement	EFO_0004611	Cardiovascular measurement	Cardiovascular	PGS003031	EFO_0004611	low density lipoprotein cholesterol measurement	measurement	EFO_0004611	Cardiovascular measurement	Cardiovascular
0.9303	2.66E-200	1.58E-196	PGS002556	EFO_0004611	low density lipoprotein cholesterol measurement	low density lipoprotein cholesterol measurement	EFO_0004611	Cardiovascular measurement	Cardiovascular	PGS002620	EFO_0004574	total cholesterol measurement	total cholesterol measurement	EFO_0004574	Lipid or lipoprotein measurement	Metabolic
0.9303	2.66E-200	1.58E-196	PGS002556	EFO_0004611	low density lipoprotein cholesterol measurement	low density lipoprotein cholesterol measurement	EFO_0004611	Lipid or lipoprotein measurement	Metabolic	PGS002620	EFO_0004574	total cholesterol measurement	total cholesterol measurement	EFO_0004574	Lipid or lipoprotein measurement	Metabolic
0.9303	2.85E-200	1.68E-196	PGS001856	EFO_0001060	celiac disease	celiac disease	EFO_0001060	Immune system disorder	Immunological	PGS002067	EFO_0001060	celiac disease	celiac disease	EFO_0001060	Digestive system disorder	Gastrointestinal
0.9303	2.85E-200	1.68E-196	PGS001856	EFO_0001060	celiac disease	celiac disease	EFO_0001060	Digestive system disorder	Gastrointestinal	PGS002067	EFO_0001060	celiac disease	celiac disease	EFO_0001060	Digestive system disorder	Gastrointestinal
0.9303	2.85E-200	1.68E-196	PGS001856	EFO_0001060	celiac disease	celiac disease	EFO_0001060	Immune system disorder	Immunological	PGS002067	EFO_0001060	celiac disease	celiac disease	EFO_0001060	Immune system disorder	Immunological
0.9303	2.85E-200	1.68E-196	PGS001856	EFO_0001060	celiac disease	celiac disease	EFO_0001060	Digestive system disorder	Gastrointestinal	PGS002067	EFO_0001060	celiac disease	celiac disease	EFO_0001060	Immune system disorder	Immunological
0.9299	7.97E-200	4.70E-196	PGS001157	EFO_0005106	body composition measurement	body composition measurement	EFO_0005106	Body measurement	Body measurement	PGS001155	EFO_0005106	body composition measurement	body composition measurement	EFO_0005106	Body measurement	Body measurement
0.9299	8.46E-200	4.98E-196	PGS002588	EFO_0000701	skin disease	skin disease	EFO_0000701	Other disease	Dermatological	PGS002539	EFO_0000701	skin disease	skin disease	EFO_0000701	Other disease	Dermatological
0.9294	4.19E-199	2.47E-195	PGS003248	EFO_0010472	creatine measurement	creatine measurement	EFO_0010472	Other measurement	Metabolic	PGS003243	EFO_0010472	creatine measurement	creatine measurement	EFO_0010472	Other measurement	Metabolic
0.9294	4.25E-199	2.50E-195	PGS000317	EFO_0000305	breast carcinoma	breast carcinoma	EFO_0000305	Cancer	Neoplasms	PGS000052	EFO_0000305	breast carcinoma	breast carcinoma	EFO_0000305	Cancer	Neoplasms
0.9294	5.08E-199	2.98E-195	PGS001945	EFO_0004518	creatinine measurement	creatinine measurement	EFO_0004518	Other measurement	Metabolic	PGS002163	EFO_0004518	creatinine measurement	creatinine measurement	EFO_0004518	Other measurement	Metabolic
0.9293	5.41E-199	3.17E-195	PGS002608	EFO_0004584	mean platelet volume	mean platelet volume	EFO_0004584	Hematological measurement	Hematological	PGS002461	EFO_0004584	mean platelet volume	mean platelet volume	EFO_0004584	Hematological measurement	Hematological
0.9293	6.06E-199	3.55E-195	PGS002273	MONDO_0100096	COVID-19	COVID-19	MONDO_0100096	Other disease	Infection	PGS002272	MONDO_0100096	COVID-19	COVID-19	MONDO_0100096	Other disease	Infection
0.9292	7.82E-199	4.58E-195	PGS001168	EFO_0004995	lean body mass	lean body mass	EFO_0004995	Other measurement	Skeletal	PGS001234	EFO_0004324	body weights and measures	body weights and measures	EFO_0004324	Body measurement	Body measurement
-0.9291	9.54E-199	5.57E-195	PGS001117	EFO_0600082	insulin use measurement	insulin use measurement	EFO_0600082	Other measurement	Metabolic	PGS001371	EFO_0004918	age at diagnosis|diabetes mellitus	age at diagnosis	EFO_0004918	Other measurement	Metabolic
0.9289	2.23E-198	1.30E-194	PGS003140	EFO_0004574	total cholesterol measurement	total cholesterol measurement	EFO_0004574	Lipid or lipoprotein measurement	Metabolic	PGS003136	EFO_0004574	total cholesterol measurement	total cholesterol measurement	EFO_0004574	Lipid or lipoprotein measurement	Metabolic
0.9286	5.45E-198	3.18E-194	PGS001330	EFO_0000384	Crohn's disease	Crohn's disease	EFO_0000384	Digestive system disorder	Gastrointestinal	PGS001331	EFO_0000384	Crohn's disease	Crohn's disease	EFO_0000384	Digestive system disorder	Gastrointestinal
0.9286	5.45E-198	3.18E-194	PGS001330	EFO_0000384	Crohn's disease	Crohn's disease	EFO_0000384	Digestive system disorder	Gastrointestinal	PGS001331	EFO_0000384	Crohn's disease	Crohn's disease	EFO_0000384	Immune system disorder	Immunological
0.9286	5.45E-198	3.18E-194	PGS001330	EFO_0000384	Crohn's disease	Crohn's disease	EFO_0000384	Immune system disorder	Immunological	PGS001331	EFO_0000384	Crohn's disease	Crohn's disease	EFO_0000384	Immune system disorder	Immunological
0.9286	5.45E-198	3.18E-194	PGS001330	EFO_0000384	Crohn's disease	Crohn's disease	EFO_0000384	Immune system disorder	Immunological	PGS001331	EFO_0000384	Crohn's disease	Crohn's disease	EFO_0000384	Digestive system disorder	Gastrointestinal
0.9286	6.03E-198	3.51E-194	PGS001363	EFO_0004731	eye measurement	eye measurement	EFO_0004731	Other measurement	Ophthalmological	PGS001362	EFO_0004731	eye measurement	eye measurement	EFO_0004731	Other measurement	Ophthalmological
0.9285	6.93E-198	4.03E-194	PGS000317	EFO_0000305	breast carcinoma	breast carcinoma	EFO_0000305	Cancer	Neoplasms	PGS000773	EFO_0000305	breast carcinoma	breast carcinoma	EFO_0000305	Cancer	Neoplasms
0.9285	7.40E-198	4.30E-194	PGS002257	EFO_0006335	systolic blood pressure	systolic blood pressure	EFO_0006335	Other measurement	Cardiovascular	PGS000301	EFO_0006335	systolic blood pressure	systolic blood pressure	EFO_0006335	Other measurement	Cardiovascular
0.928	2.92E-197	1.69E-193	PGS002566	EFO_0000684	respiratory system disease	respiratory system disease	EFO_0000684	Other disease	Skeletal	PGS002579	MONDO_0004979	asthma	asthma	MONDO_0004979	Other disease	Respiratory
0.928	3.74E-197	2.17E-193	PGS003008	EFO_0000537	hypertension	hypertension	EFO_0000537	Cardiovascular disease	Cardiovascular	PGS003023	EFO_0000537	hypertension	hypertension	EFO_0000537	Cardiovascular disease	Cardiovascular
0.9279	4.38E-197	2.54E-193	PGS001918	EFO_0004314	forced expiratory volume	forced expiratory volume	EFO_0004314	Other measurement	Respiratory	PGS002134	EFO_0004314	forced expiratory volume	forced expiratory volume	EFO_0004314	Other measurement	Respiratory
0.9278	6.00E-197	3.47E-193	PGS000561	EFO_0003893	ovarian neoplasm	ovarian neoplasm	EFO_0003893	Cancer	Neoplasms	PGS000562	EFO_0003893	ovarian neoplasm	ovarian neoplasm	EFO_0003893	Cancer	Neoplasms
0.9277	6.99E-197	4.03E-193	PGS003285	EFO_0004617	cystatin C measurement	cystatin C measurement	EFO_0004617	Other measurement	Metabolic	PGS003286	EFO_0004617	cystatin C measurement	cystatin C measurement	EFO_0004617	Other measurement	Metabolic
0.9277	8.67E-197	5.00E-193	PGS003249	EFO_0010472	creatine measurement|glomerular filtration rate	creatine measurement	EFO_0010472	Other measurement	Metabolic	PGS003264	EFO_0010472	creatine measurement|glomerular filtration rate	creatine measurement	EFO_0010472	Other measurement	Metabolic
0.9276	1.06E-196	6.08E-193	PGS001369	EFO_0007149	appendicitis	appendicitis	EFO_0007149	Immune system disorder	Immunological	PGS001853	EFO_0009542	disorder of appendix	disorder of appendix	EFO_0009542	Digestive system disorder	Gastrointestinal
0.9276	1.06E-196	6.08E-193	PGS001369	EFO_0007149	appendicitis	appendicitis	EFO_0007149	Digestive system disorder	Gastrointestinal	PGS001853	EFO_0009542	disorder of appendix	disorder of appendix	EFO_0009542	Digestive system disorder	Gastrointestinal
0.9276	1.22E-196	6.99E-193	PGS002900	EFO_0006336	diastolic blood pressure	diastolic blood pressure	EFO_0006336	Other measurement	Cardiovascular	PGS002905	EFO_0006336	diastolic blood pressure	diastolic blood pressure	EFO_0006336	Other measurement	Cardiovascular
0.9275	1.33E-196	7.65E-193	PGS001958	EFO_0005093	hip circumference	hip circumference	EFO_0005093	Body measurement	Body measurement	PGS002176	EFO_0005093	hip circumference	hip circumference	EFO_0005093	Body measurement	Body measurement
0.9275	1.46E-196	8.35E-193	PGS002605	EFO_0004611	low density lipoprotein cholesterol measurement	low density lipoprotein cholesterol measurement	EFO_0004611	Lipid or lipoprotein measurement	Metabolic	PGS002620	EFO_0004574	total cholesterol measurement	total cholesterol measurement	EFO_0004574	Lipid or lipoprotein measurement	Metabolic
0.9275	1.46E-196	8.35E-193	PGS002605	EFO_0004611	low density lipoprotein cholesterol measurement	low density lipoprotein cholesterol measurement	EFO_0004611	Cardiovascular measurement	Cardiovascular	PGS002620	EFO_0004574	total cholesterol measurement	total cholesterol measurement	EFO_0004574	Lipid or lipoprotein measurement	Metabolic
0.9274	1.84E-196	1.05E-192	PGS002592	EFO_0001379	endocrine system disease|diabetes mellitus	endocrine system disease	EFO_0001379	Other disease	Metabolic	PGS002400	EFO_0004468	glucose measurement	glucose measurement	EFO_0004468	Other measurement	Metabolic
0.9273	2.48E-196	1.42E-192	PGS002540	EFO_0000400	diabetes mellitus	diabetes mellitus	EFO_0000400	Metabolic disorder	Metabolic	PGS002403	EFO_0004541	HbA1c measurement	HbA1c measurement	EFO_0004541	Hematological measurement	Hematological
0.9273	2.48E-196	1.42E-192	PGS002540	EFO_0000400	diabetes mellitus	diabetes mellitus	EFO_0000400	Digestive system disorder	Gastrointestinal	PGS002403	EFO_0004541	HbA1c measurement	HbA1c measurement	EFO_0004541	Hematological measurement	Hematological
0.9273	2.86E-196	1.64E-192	PGS000133	MONDO_0005090	schizophrenia	schizophrenia	MONDO_0005090	Neurological disorder	Neurological	PGS000135	MONDO_0005090	schizophrenia	schizophrenia	MONDO_0005090	Neurological disorder	Neurological
0.9272	3.53E-196	2.01E-192	PGS000463	EFO_0000707	squamous cell carcinoma	squamous cell carcinoma	EFO_0000707	Cancer	Neoplasms	PGS000464	EFO_0000707	squamous cell carcinoma	squamous cell carcinoma	EFO_0000707	Cancer	Neoplasms
0.9272	3.53E-196	2.01E-192	PGS000463	EFO_0000707	squamous cell carcinoma	squamous cell carcinoma	EFO_0000707	Cancer	Neoplasms	PGS000461	EFO_0000707	squamous cell carcinoma	squamous cell carcinoma	EFO_0000707	Cancer	Neoplasms
0.9272	3.53E-196	2.01E-192	PGS000463	EFO_0000707	squamous cell carcinoma	squamous cell carcinoma	EFO_0000707	Cancer	Neoplasms	PGS000462	EFO_0000707	squamous cell carcinoma	squamous cell carcinoma	EFO_0000707	Cancer	Neoplasms
0.9272	3.78E-196	2.15E-192	PGS002957	EFO_0004612	high density lipoprotein cholesterol measurement	high density lipoprotein cholesterol measurement	EFO_0004612	Lipid or lipoprotein measurement	Metabolic	PGS002962	EFO_0004612	high density lipoprotein cholesterol measurement	measurement	EFO_0004612	Lipid or lipoprotein measurement	Metabolic
0.9271	5.52E-196	3.13E-192	PGS000051	EFO_0000305	breast carcinoma	breast carcinoma	EFO_0000305	Cancer	Neoplasms	PGS000002	EFO_1000649	estrogen-receptor positive breast cancer	cancer	EFO_1000649	Cancer	Neoplasms
0.9268	1.17E-195	6.61E-192	PGS002541	EFO_0006336	diastolic blood pressure	diastolic blood pressure	EFO_0006336	Other measurement	Cardiovascular	PGS002394	EFO_0006336	diastolic blood pressure	diastolic blood pressure	EFO_0006336	Other measurement	Cardiovascular
0.9267	1.53E-195	8.65E-192	PGS000347	EFO_1000649	estrogen-receptor positive breast cancer	estrogen-receptor positive breast cancer	EFO_1000649	Cancer	Neoplasms	PGS000345	EFO_0000305	breast carcinoma	breast carcinoma	EFO_0000305	Cancer	Neoplasms
0.9267	1.66E-195	9.39E-192	PGS003135	EFO_0004574	total cholesterol measurement	total cholesterol measurement	EFO_0004574	Lipid or lipoprotein measurement	Metabolic	PGS003141	EFO_0004574	total cholesterol measurement	total cholesterol measurement	EFO_0004574	Lipid or lipoprotein measurement	Metabolic
0.9265	2.86E-195	1.62E-191	PGS002556	EFO_0004611	low density lipoprotein cholesterol measurement	low density lipoprotein cholesterol measurement	EFO_0004611	Lipid or lipoprotein measurement	Metabolic	PGS002571	EFO_0004574	total cholesterol measurement	total cholesterol measurement	EFO_0004574	Lipid or lipoprotein measurement	Metabolic
0.9265	2.86E-195	1.62E-191	PGS002556	EFO_0004611	low density lipoprotein cholesterol measurement	low density lipoprotein cholesterol measurement	EFO_0004611	Cardiovascular measurement	Cardiovascular	PGS002571	EFO_0004574	total cholesterol measurement	total cholesterol measurement	EFO_0004574	Lipid or lipoprotein measurement	Metabolic
0.9265	3.16E-195	1.78E-191	PGS002340	EFO_0004584	mean platelet volume	mean platelet volume	EFO_0004584	Hematological measurement	Hematological	PGS002189	EFO_0004584	mean platelet volume	mean platelet volume	EFO_0004584	Hematological measurement	Hematological
0.9263	5.31E-195	2.99E-191	PGS002339	EFO_0004527	mean corpuscular hemoglobin	mean corpuscular hemoglobin	EFO_0004527	Hematological measurement	Hematological	PGS002206	EFO_0004527	mean corpuscular hemoglobin	mean corpuscular hemoglobin	EFO_0004527	Hematological measurement	Hematological
0.9263	6.19E-195	3.48E-191	PGS003398	EFO_0000305	breast carcinoma	breast carcinoma	EFO_0000305	Cancer	Neoplasms	PGS000773	EFO_0000305	breast carcinoma	breast carcinoma	EFO_0000305	Cancer	Neoplasms
0.9262	6.35E-195	3.57E-191	PGS000296	EFO_0001645	coronary artery disease	coronary artery disease	EFO_0001645	Cardiovascular disease	Cardiovascular	PGS000013	EFO_0001645	coronary artery disease	coronary artery disease	EFO_0001645	Cardiovascular disease	Cardiovascular
0.9262	7.42E-195	4.17E-191	PGS000575	EFO_0001663	prostate carcinoma	prostate carcinoma	EFO_0001663	Cancer	Neoplasms	PGS000573	EFO_0001663	prostate carcinoma	prostate carcinoma	EFO_0001663	Cancer	Neoplasms
0.9261	1.02E-194	5.74E-191	PGS001874	EFO_1000243	Epidermal Inclusion Cyst	Epidermal Inclusion Cyst	EFO_1000243	Cancer	Neoplasms	PGS001873	MONDO_0002917	disorder of pilosebaceous unit	disorder of pilosebaceous unit	MONDO_0002917	Other disease	Dermatological
0.926	1.39E-194	7.81E-191	PGS002101	EFO_0004614	apolipoprotein A 1 measurement	apolipoprotein A 1 measurement	EFO_0004614	Cardiovascular measurement	Cardiovascular	PGS001888	EFO_0004614	apolipoprotein A 1 measurement	apolipoprotein A 1 measurement	EFO_0004614	Lipid or lipoprotein measurement	Metabolic
0.926	1.39E-194	7.81E-191	PGS002101	EFO_0004614	apolipoprotein A 1 measurement	apolipoprotein A 1 measurement	EFO_0004614	Lipid or lipoprotein measurement	Metabolic	PGS001888	EFO_0004614	apolipoprotein A 1 measurement	apolipoprotein A 1 measurement	EFO_0004614	Lipid or lipoprotein measurement	Metabolic
0.926	1.39E-194	7.81E-191	PGS002101	EFO_0004614	apolipoprotein A 1 measurement	apolipoprotein A 1 measurement	EFO_0004614	Cardiovascular measurement	Cardiovascular	PGS001888	EFO_0004614	apolipoprotein A 1 measurement	apolipoprotein A 1 measurement	EFO_0004614	Inflammatory measurement	Immunological
0.926	1.39E-194	7.81E-191	PGS002101	EFO_0004614	apolipoprotein A 1 measurement	apolipoprotein A 1 measurement	EFO_0004614	Lipid or lipoprotein measurement	Metabolic	PGS001888	EFO_0004614	apolipoprotein A 1 measurement	apolipoprotein A 1 measurement	EFO_0004614	Inflammatory measurement	Immunological
0.926	1.39E-194	7.81E-191	PGS002101	EFO_0004614	apolipoprotein A 1 measurement	apolipoprotein A 1 measurement	EFO_0004614	Lipid or lipoprotein measurement	Metabolic	PGS001888	EFO_0004614	apolipoprotein A 1 measurement	apolipoprotein A 1 measurement	EFO_0004614	Cardiovascular measurement	Cardiovascular
0.926	1.39E-194	7.81E-191	PGS002101	EFO_0004614	apolipoprotein A 1 measurement	apolipoprotein A 1 measurement	EFO_0004614	Cardiovascular measurement	Cardiovascular	PGS001888	EFO_0004614	apolipoprotein A 1 measurement	apolipoprotein A 1 measurement	EFO_0004614	Cardiovascular measurement	Cardiovascular
0.926	1.39E-194	7.81E-191	PGS002101	EFO_0004614	apolipoprotein A 1 measurement	apolipoprotein A 1 measurement	EFO_0004614	Inflammatory measurement	Immunological	PGS001888	EFO_0004614	apolipoprotein A 1 measurement	apolipoprotein A 1 measurement	EFO_0004614	Inflammatory measurement	Immunological
0.926	1.39E-194	7.81E-191	PGS002101	EFO_0004614	apolipoprotein A 1 measurement	apolipoprotein A 1 measurement	EFO_0004614	Inflammatory measurement	Immunological	PGS001888	EFO_0004614	apolipoprotein A 1 measurement	apolipoprotein A 1 measurement	EFO_0004614	Cardiovascular measurement	Cardiovascular
0.926	1.39E-194	7.81E-191	PGS002101	EFO_0004614	apolipoprotein A 1 measurement	apolipoprotein A 1 measurement	EFO_0004614	Inflammatory measurement	Immunological	PGS001888	EFO_0004614	apolipoprotein A 1 measurement	apolipoprotein A 1 measurement	EFO_0004614	Lipid or lipoprotein measurement	Metabolic
0.9259	1.82E-194	1.02E-190	PGS003271	EFO_0010472	creatine measurement|glomerular filtration rate	creatine measurement	EFO_0010472	Other measurement	Metabolic	PGS003256	EFO_0010472	creatine measurement|glomerular filtration rate	creatine measurement	EFO_0010472	Other measurement	Metabolic
0.9258	2.17E-194	1.22E-190	PGS003281	EFO_0004617	cystatin C measurement	cystatin C measurement	EFO_0004617	Other measurement	Metabolic	PGS003280	EFO_0004617	cystatin C measurement	cystatin C measurement	EFO_0004617	Other measurement	Metabolic
0.9257	3.01E-194	1.68E-190	PGS002478	EFO_0004308	leukocyte count	leukocyte count	EFO_0004308	Hematological measurement	Hematological	PGS002429	EFO_0004308	leukocyte count	leukocyte count	EFO_0004308	Hematological measurement	Hematological
0.9256	3.85E-194	2.15E-190	PGS002576	EFO_0004308	leukocyte count	leukocyte count	EFO_0004308	Hematological measurement	Hematological	PGS002429	EFO_0004308	leukocyte count	leukocyte count	EFO_0004308	Hematological measurement	Hematological
0.9255	5.25E-194	2.92E-190	PGS002205	EFO_0004908	testosterone measurement|male	testosterone measurement	EFO_0004908	Lipid or lipoprotein measurement	Metabolic	PGS001988	EFO_0004908	testosterone measurement|male	testosterone measurement	EFO_0004908	Lipid or lipoprotein measurement	Metabolic
0.9255	5.89E-194	3.28E-190	PGS002965	EFO_0004339	body height	body height	EFO_0004339	Body measurement	Body measurement	PGS002966	EFO_0004339	body height	body height	EFO_0004339	Body measurement	Body measurement
0.9255	6.34E-194	3.52E-190	PGS002169	EFO_0005409	fat body mass	fat body mass	EFO_0005409	Other measurement	Body measurement	PGS001951	EFO_0005409	fat body mass	fat body mass	EFO_0005409	Other measurement	Body measurement
0.9254	8.22E-194	4.56E-190	PGS002432	MONDO_0004979	asthma	asthma	MONDO_0004979	Other disease	Respiratory	PGS002383	MONDO_0004979	asthma	asthma	MONDO_0004979	Other disease	Respiratory
0.9254	8.59E-194	4.76E-190	PGS002975	EFO_0004339	body height	body height	EFO_0004339	Body measurement	Body measurement	PGS002985	EFO_0004339	body height	body height	EFO_0004339	Body measurement	Body measurement
0.9251	1.74E-193	9.66E-190	PGS001822	EFO_0004274	gout	gout	EFO_0004274	Metabolic disorder	Metabolic	PGS002030	EFO_0004274	gout	gout	EFO_0004274	Metabolic disorder	Metabolic
0.925	2.57E-193	1.42E-189	PGS002986	EFO_0004339	body height	body height	EFO_0004339	Body measurement	Body measurement	PGS002976	EFO_0004339	body height	body height	EFO_0004339	Body measurement	Body measurement
0.9249	3.27E-193	1.81E-189	PGS000553	EFO_0003893	ovarian neoplasm	ovarian neoplasm	EFO_0003893	Cancer	Neoplasms	PGS000555	EFO_0003893	ovarian neoplasm	ovarian neoplasm	EFO_0003893	Cancer	Neoplasms
0.9249	3.52E-193	1.94E-189	PGS003032	EFO_0004611	low density lipoprotein cholesterol measurement	low density lipoprotein cholesterol measurement	EFO_0004611	Cardiovascular measurement	Cardiovascular	PGS003037	EFO_0004611	low density lipoprotein cholesterol measurement	measurement	EFO_0004611	Lipid or lipoprotein measurement	Metabolic
0.9249	3.52E-193	1.94E-189	PGS003032	EFO_0004611	low density lipoprotein cholesterol measurement	low density lipoprotein cholesterol measurement	EFO_0004611	Lipid or lipoprotein measurement	Metabolic	PGS003037	EFO_0004611	low density lipoprotein cholesterol measurement	measurement	EFO_0004611	Cardiovascular measurement	Cardiovascular
0.9249	3.52E-193	1.94E-189	PGS003032	EFO_0004611	low density lipoprotein cholesterol measurement	low density lipoprotein cholesterol measurement	EFO_0004611	Lipid or lipoprotein measurement	Metabolic	PGS003037	EFO_0004611	low density lipoprotein cholesterol measurement	measurement	EFO_0004611	Lipid or lipoprotein measurement	Metabolic
0.9249	3.52E-193	1.94E-189	PGS003032	EFO_0004611	low density lipoprotein cholesterol measurement	low density lipoprotein cholesterol measurement	EFO_0004611	Cardiovascular measurement	Cardiovascular	PGS003037	EFO_0004611	low density lipoprotein cholesterol measurement	measurement	EFO_0004611	Cardiovascular measurement	Cardiovascular
0.9248	4.09E-193	2.25E-189	PGS002466	EFO_0004305	erythrocyte count	erythrocyte count	EFO_0004305	Hematological measurement	Hematological	PGS002417	EFO_0004305	erythrocyte count	erythrocyte count	EFO_0004305	Hematological measurement	Hematological
0.9248	4.28E-193	2.35E-189	PGS001892	EFO_0004344	birth weight	birth weight	EFO_0004344	Body measurement	Body measurement	PGS002105	EFO_0004344	birth weight	birth weight	EFO_0004344	Body measurement	Body measurement
0.9248	4.65E-193	2.56E-189	PGS000688	EFO_0004611	low density lipoprotein cholesterol measurement	low density lipoprotein cholesterol measurement	EFO_0004611	Cardiovascular measurement	Cardiovascular	PGS000677	EFO_0004574	total cholesterol measurement	total cholesterol measurement	EFO_0004574	Lipid or lipoprotein measurement	Metabolic
0.9248	4.65E-193	2.56E-189	PGS000688	EFO_0004611	low density lipoprotein cholesterol measurement	low density lipoprotein cholesterol measurement	EFO_0004611	Lipid or lipoprotein measurement	Metabolic	PGS000677	EFO_0004574	total cholesterol measurement	total cholesterol measurement	EFO_0004574	Lipid or lipoprotein measurement	Metabolic
0.9247	6.45E-193	3.54E-189	PGS002403	EFO_0004541	HbA1c measurement	HbA1c measurement	EFO_0004541	Hematological measurement	Hematological	PGS002592	EFO_0001379	endocrine system disease|diabetes mellitus	endocrine system disease	EFO_0001379	Other disease	Metabolic
0.9247	6.77E-193	3.72E-189	PGS002538	EFO_0011015	educational attainment	educational attainment	EFO_0011015	Other measurement	Activities	PGS002587	EFO_0011015	educational attainment	educational attainment	EFO_0011015	Other measurement	Activities
0.9246	8.12E-193	4.45E-189	PGS002791	EFO_0001663	prostate carcinoma	prostate carcinoma	EFO_0001663	Cancer	Neoplasms	PGS000719	EFO_0001663	prostate carcinoma	prostate carcinoma	EFO_0001663	Cancer	Neoplasms
0.9245	1.19E-192	6.53E-189	PGS001663	EFO_0006930	brain volume measurement|neuroimaging measurement	brain volume measurement	EFO_0006930	Other measurement	Neurological	PGS001664	EFO_0006930	measurement	brain volume measurement	EFO_0006930	Other measurement	Neurological
0.9245	1.20E-192	6.58E-189	PGS002547	EFO_0004468	glucose measurement	glucose measurement	EFO_0004468	Other measurement	Metabolic	PGS002622	MONDO_0005148	type 2 diabetes mellitus	type 2 diabetes mellitus	MONDO_0005148	Digestive system disorder	Gastrointestinal
0.9245	1.20E-192	6.58E-189	PGS002547	EFO_0004468	glucose measurement	glucose measurement	EFO_0004468	Other measurement	Metabolic	PGS002622	MONDO_0005148	type 2 diabetes mellitus	type 2 diabetes mellitus	MONDO_0005148	Metabolic disorder	Metabolic

0.9244	1.48E-192	8.09E-189	PGS002543	EFO_0001379	endocrine system disease|diabetes mellitus	endocrine system disease	EFO_0001379	Other disease	Metabolic	PGS002475	MONDO_0005148	type 2 diabetes mellitus	type 2 diabetes mellitus	MONDO_0005148	Metabolic disorder	Metabolic
0.9244	1.48E-192	8.09E-189	PGS002543	EFO_0001379	endocrine system disease|diabetes mellitus	endocrine system disease	EFO_0001379	Other disease	Metabolic	PGS002475	MONDO_0005148	type 2 diabetes mellitus	type 2 diabetes mellitus	MONDO_0005148	Digestive system disorder	Gastrointestinal
0.9243	1.84E-192	1.01E-188	PGS002853	EFO_0004340	body mass index	body mass index	EFO_0004340	Body measurement	Body measurement	PGS002848	EFO_0004340	body mass index	body mass index	EFO_0004340	Body measurement	Body measurement
0.9242	2.35E-192	1.28E-188	PGS000504	EFO_0000305	breast carcinoma	breast carcinoma	EFO_0000305	Cancer	Neoplasms	PGS000512	EFO_0000305	breast carcinoma	breast carcinoma	EFO_0000305	Cancer	Neoplasms
0.9239	5.28E-192	2.87E-188	PGS001160	EFO_0005106	body composition measurement	body composition measurement	EFO_0005106	Body measurement	Body measurement	PGS001165	EFO_0004995	lean body mass	lean body mass	EFO_0004995	Other measurement	Skeletal
0.9239	6.48E-192	3.52E-188	PGS002410	EFO_0004587	lymphocyte count	lymphocyte count	EFO_0004587	Hematological measurement	Hematological	PGS002606	EFO_0004587	lymphocyte count	lymphocyte count	EFO_0004587	Hematological measurement	Hematological
0.9238	7.22E-192	3.92E-188	PGS002403	EFO_0004541	HbA1c measurement	HbA1c measurement	EFO_0004541	Hematological measurement	Hematological	PGS002396	EFO_0001379	endocrine system disease|diabetes mellitus	endocrine system disease	EFO_0001379	Other disease	Metabolic
0.9237	9.56E-192	5.19E-188	PGS002445	EFO_0001379	endocrine system disease|diabetes mellitus	endocrine system disease	EFO_0001379	Other disease	Metabolic	PGS002426	MONDO_0005148	type 2 diabetes mellitus	type 2 diabetes mellitus	MONDO_0005148	Digestive system disorder	Gastrointestinal
0.9237	9.56E-192	5.19E-188	PGS002445	EFO_0001379	endocrine system disease|diabetes mellitus	endocrine system disease	EFO_0001379	Other disease	Metabolic	PGS002426	MONDO_0005148	type 2 diabetes mellitus	type 2 diabetes mellitus	MONDO_0005148	Metabolic disorder	Metabolic
0.9236	1.29E-191	7.01E-188	PGS000681	EFO_0004570	bilirubin measurement	bilirubin measurement	EFO_0004570	Other measurement	Metabolic	PGS000924	MONDO_0037821	disease	porphyrin metabolism disease	MONDO_0037821	Metabolic disorder	Metabolic
0.9233	3.63E-191	1.97E-187	PGS000532	EFO_0000305	breast carcinoma	breast carcinoma	EFO_0000305	Cancer	Neoplasms	PGS000540	EFO_0000305	breast carcinoma	breast carcinoma	EFO_0000305	Cancer	Neoplasms
0.9232	4.90E-191	2.65E-187	PGS001168	EFO_0004995	lean body mass	lean body mass	EFO_0004995	Other measurement	Skeletal	PGS001165	EFO_0004995	lean body mass	lean body mass	EFO_0004995	Other measurement	Skeletal
0.9231	5.58E-191	3.01E-187	PGS003198	EFO_0004631	vitamin D measurement	vitamin D measurement	EFO_0004631	Lipid or lipoprotein measurement	Metabolic	PGS002355	EFO_0004631	vitamin D measurement	vitamin D measurement	EFO_0004631	Lipid or lipoprotein measurement	Metabolic
0.9229	8.98E-191	4.82E-187	PGS002837	EFO_0007878	alcohol consumption measurement	alcohol consumption measurement	EFO_0007878	Other measurement	Psychiatric	PGS002832	EFO_0007878	alcohol consumption measurement	alcohol consumption measurement	EFO_0007878	Other measurement	Psychiatric
0.9229	1.15E-190	6.16E-187	PGS003133	EFO_0004340	body mass index	body mass index	EFO_0004340	Body measurement	Body measurement	PGS003128	EFO_0004340	body mass index	body mass index	EFO_0004340	Body measurement	Body measurement
0.9228	1.21E-190	6.50E-187	PGS002428	EFO_0004343	waist-hip ratio	waist-hip ratio	EFO_0004343	Body measurement	Body measurement	PGS002575	EFO_0004343	waist-hip ratio	waist-hip ratio	EFO_0004343	Body measurement	Body measurement
0.9227	1.85E-190	9.92E-187	PGS002853	EFO_0004340	body mass index	body mass index	EFO_0004340	Body measurement	Body measurement	PGS002858	EFO_0004340	body mass index	body mass index	EFO_0004340	Body measurement	Body measurement
0.9225	3.46E-190	1.85E-186	PGS002024	EFO_0004705	hypothyroidism	hypothyroidism	EFO_0004705	Other disease	Endocrine	PGS001816	EFO_0004705	hypothyroidism	hypothyroidism	EFO_0004705	Other disease	Endocrine
0.9222	6.84E-190	3.65E-186	PGS003146	EFO_0004530	triglyceride measurement	triglyceride measurement	EFO_0004530	Lipid or lipoprotein measurement	Metabolic	PGS003145	EFO_0004530	triglyceride measurement	triglyceride measurement	EFO_0004530	Lipid or lipoprotein measurement	Metabolic
0.9222	8.49E-190	4.53E-186	PGS001451	EFO_0006930	brain volume measurement|neuroimaging measurement	brain volume measurement	EFO_0006930	Other measurement	Neurological	PGS001450	EFO_0006930	measurement	brain volume measurement	EFO_0006930	Other measurement	Neurological
0.9221	9.71E-190	5.17E-186	PGS001225	EFO_0004348	hematocrit	hematocrit	EFO_0004348	Hematological measurement	Hematological	PGS001400	EFO_0004509	hemoglobin measurement	hemoglobin measurement	EFO_0004509	Hematological measurement	Hematological
0.9221	1.07E-189	5.69E-186	PGS002453	EFO_0004339	body height	body height	EFO_0004339	Body measurement	Body measurement	PGS002551	EFO_0004339	body height	body height	EFO_0004339	Body measurement	Body measurement
0.9217	2.64E-189	1.40E-185	PGS003284	EFO_0004617	cystatin C measurement	cystatin C measurement	EFO_0004617	Other measurement	Metabolic	PGS003279	EFO_0004617	cystatin C measurement	cystatin C measurement	EFO_0004617	Other measurement	Metabolic
0.9217	3.36E-189	1.78E-185	PGS003150	EFO_0004530	triglyceride measurement	triglyceride measurement	EFO_0004530	Lipid or lipoprotein measurement	Metabolic	PGS003151	EFO_0004530	triglyceride measurement	triglyceride measurement	EFO_0004530	Lipid or lipoprotein measurement	Metabolic
0.9216	4.16E-189	2.21E-185	PGS002445	EFO_0001379	endocrine system disease|diabetes mellitus	endocrine system disease	EFO_0001379	Other disease	Metabolic	PGS002592	EFO_0001379	endocrine system disease|diabetes mellitus	endocrine system disease	EFO_0001379	Other disease	Metabolic
0.9215	5.15E-189	2.73E-185	PGS001917	EFO_0007800	body fat percentage	body fat percentage	EFO_0007800	Body measurement	Body measurement	PGS001951	EFO_0005409	fat body mass	fat body mass	EFO_0005409	Other measurement	Body measurement
0.9214	7.85E-189	4.15E-185	PGS002156	EFO_0009749	age at first sexual intercourse measurement	age at first sexual intercourse measurement	EFO_0009749	Other measurement	Genitourinary	PGS001938	EFO_0009749	age at first sexual intercourse measurement	measurement	EFO_0009749	Other measurement	Genitourinary
0.9213	8.35E-189	4.41E-185	PGS002550	EFO_0004541	HbA1c measurement	HbA1c measurement	EFO_0004541	Hematological measurement	Hematological	PGS002599	EFO_0004541	HbA1c measurement	HbA1c measurement	EFO_0004541	Hematological measurement	Hematological
0.9213	9.27E-189	4.89E-185	PGS002976	EFO_0004339	body height	body height	EFO_0004339	Body measurement	Body measurement	PGS002985	EFO_0004339	body height	body height	EFO_0004339	Body measurement	Body measurement
0.9208	3.17E-188	1.67E-184	PGS001955	EFO_0009270	heel bone mineral density	heel bone mineral density	EFO_0009270	Other measurement	Skeletal	PGS002175	EFO_0005654	velocity of sound measurement	velocity of sound measurement	EFO_0005654	Other measurement	Others
0.9208	3.23E-188	1.70E-184	PGS000030	EFO_0001663	prostate carcinoma	prostate carcinoma	EFO_0001663	Cancer	Neoplasms	PGS000580	EFO_0001663	prostate carcinoma	prostate carcinoma	EFO_0001663	Cancer	Neoplasms
0.9206	6.04E-188	3.18E-184	PGS001917	EFO_0007800	body fat percentage	body fat percentage	EFO_0007800	Body measurement	Body measurement	PGS002133	EFO_0007800	body fat percentage	body fat percentage	EFO_0007800	Body measurement	Body measurement
-0.9204	1.02E-187	5.39E-184	PGS001246	EFO_0004279	suntan	suntan	EFO_0004279	Other trait	Dermatological	PGS001244	EFO_0004279	suntan	suntan	EFO_0004279	Other trait	Dermatological
0.9203	1.56E-187	8.18E-184	PGS000030	EFO_0001663	prostate carcinoma	prostate carcinoma	EFO_0001663	Cancer	Neoplasms	PGS000795	EFO_0001663	prostate carcinoma	prostate carcinoma	EFO_0001663	Cancer	Neoplasms
0.9202	1.78E-187	9.35E-184	PGS002908	EFO_0006336	diastolic blood pressure	diastolic blood pressure	EFO_0006336	Other measurement	Cardiovascular	PGS002903	EFO_0006336	diastolic blood pressure	diastolic blood pressure	EFO_0006336	Other measurement	Cardiovascular
0.9202	1.88E-187	9.86E-184	PGS002906	EFO_0006336	diastolic blood pressure	diastolic blood pressure	EFO_0006336	Other measurement	Cardiovascular	PGS002901	EFO_0006336	diastolic blood pressure	diastolic blood pressure	EFO_0006336	Other measurement	Cardiovascular
0.92	3.16E-187	1.65E-183	PGS002426	MONDO_0005148	type 2 diabetes mellitus	type 2 diabetes mellitus	MONDO_0005148	Metabolic disorder	Metabolic	PGS002589	EFO_0000400	diabetes mellitus	diabetes mellitus	EFO_0000400	Metabolic disorder	Metabolic
0.92	3.16E-187	1.65E-183	PGS002426	MONDO_0005148	type 2 diabetes mellitus	type 2 diabetes mellitus	MONDO_0005148	Metabolic disorder	Metabolic	PGS002589	EFO_0000400	diabetes mellitus	diabetes mellitus	EFO_0000400	Digestive system disorder	Gastrointestinal
0.92	3.16E-187	1.65E-183	PGS002426	MONDO_0005148	type 2 diabetes mellitus	type 2 diabetes mellitus	MONDO_0005148	Digestive system disorder	Gastrointestinal	PGS002589	EFO_0000400	diabetes mellitus	diabetes mellitus	EFO_0000400	Metabolic disorder	Metabolic
0.92	3.16E-187	1.65E-183	PGS002426	MONDO_0005148	type 2 diabetes mellitus	type 2 diabetes mellitus	MONDO_0005148	Digestive system disorder	Gastrointestinal	PGS002589	EFO_0000400	diabetes mellitus	diabetes mellitus	EFO_0000400	Digestive system disorder	Gastrointestinal
0.92	3.57E-187	1.87E-183	PGS000577	EFO_0001663	prostate carcinoma	prostate carcinoma	EFO_0001663	Cancer	Neoplasms	PGS000030	EFO_0001663	prostate carcinoma	prostate carcinoma	EFO_0001663	Cancer	Neoplasms
0.92	3.57E-187	1.87E-183	PGS000578	EFO_0001663	prostate carcinoma	prostate carcinoma	EFO_0001663	Cancer	Neoplasms	PGS000030	EFO_0001663	prostate carcinoma	prostate carcinoma	EFO_0001663	Cancer	Neoplasms
0.9199	3.77E-187	1.97E-183	PGS002396	EFO_0001379	endocrine system disease|diabetes mellitus	endocrine system disease	EFO_0001379	Other disease	Metabolic	PGS002400	EFO_0004468	glucose measurement	glucose measurement	EFO_0004468	Other measurement	Metabolic
0.9199	3.99E-187	2.08E-183	PGS002524	MONDO_0005148	type 2 diabetes mellitus	type 2 diabetes mellitus	MONDO_0005148	Metabolic disorder	Metabolic	PGS002475	MONDO_0005148	type 2 diabetes mellitus	type 2 diabetes mellitus	MONDO_0005148	Metabolic disorder	Metabolic
0.9199	3.99E-187	2.08E-183	PGS002524	MONDO_0005148	type 2 diabetes mellitus	type 2 diabetes mellitus	MONDO_0005148	Digestive system disorder	Gastrointestinal	PGS002475	MONDO_0005148	type 2 diabetes mellitus	type 2 diabetes mellitus	MONDO_0005148	Digestive system disorder	Gastrointestinal
0.9199	3.99E-187	2.08E-183	PGS002524	MONDO_0005148	type 2 diabetes mellitus	type 2 diabetes mellitus	MONDO_0005148	Digestive system disorder	Gastrointestinal	PGS002475	MONDO_0005148	type 2 diabetes mellitus	type 2 diabetes mellitus	MONDO_0005148	Metabolic disorder	Metabolic
0.9199	3.99E-187	2.08E-183	PGS002524	MONDO_0005148	type 2 diabetes mellitus	type 2 diabetes mellitus	MONDO_0005148	Metabolic disorder	Metabolic	PGS002475	MONDO_0005148	type 2 diabetes mellitus	type 2 diabetes mellitus	MONDO_0005148	Digestive system disorder	Gastrointestinal
0.9198	6.01E-187	3.13E-183	PGS000588	EFO_0001663	prostate carcinoma	prostate carcinoma	EFO_0001663	Cancer	Neoplasms	PGS003383	EFO_0000673	prostate adenocarcinoma	prostate adenocarcinoma	EFO_0000673	Cancer	Neoplasms
0.9198	6.35E-187	3.30E-183	PGS002984	EFO_0004339	body height	body height	EFO_0004339	Body measurement	Body measurement	PGS002974	EFO_0004339	body height	body height	EFO_0004339	Body measurement	Body measurement
0.9198	6.36E-187	3.30E-183	PGS000519	EFO_0000305	breast carcinoma	breast carcinoma	EFO_0000305	Cancer	Neoplasms	PGS000520	EFO_0000305	breast carcinoma	breast carcinoma	EFO_0000305	Cancer	Neoplasms
0.9197	8.16E-187	4.23E-183	PGS000133	MONDO_0005090	schizophrenia	schizophrenia	MONDO_0005090	Neurological disorder	Neurological	PGS000134	MONDO_0005090	schizophrenia	schizophrenia	MONDO_0005090	Neurological disorder	Neurological
0.9195	1.28E-186	6.63E-183	PGS002029	HP_0003119	Abnormal circulating lipid concentration	Abnormal circulating lipid concentration	HP_0003119	Other trait	Metabolic	PGS001821	HP_0003119	Abnormal circulating lipid concentration	Abnormal circulating lipid concentration	HP_0003119	Other trait	Metabolic
0.9195	1.29E-186	6.68E-183	PGS002764	HP_0003124	Hypercholesterolemia	Hypercholesterolemia	HP_0003124	Other trait	Metabolic	PGS003038	EFO_0004611	low density lipoprotein cholesterol measurement	measurement	EFO_0004611	Lipid or lipoprotein measurement	Metabolic
0.9195	1.29E-186	6.68E-183	PGS002764	HP_0003124	Hypercholesterolemia	Hypercholesterolemia	HP_0003124	Other trait	Metabolic	PGS003038	EFO_0004611	low density lipoprotein cholesterol measurement	measurement	EFO_0004611	Cardiovascular measurement	Cardiovascular
0.919	4.30E-186	2.22E-182	PGS002337	EFO_0004611	low density lipoprotein cholesterol measurement	low density lipoprotein cholesterol measurement	EFO_0004611	Cardiovascular measurement	Cardiovascular	PGS001933	EFO_0004611	low density lipoprotein cholesterol measurement	measurement	EFO_0004611	Cardiovascular measurement	Cardiovascular
0.919	4.30E-186	2.22E-182	PGS002337	EFO_0004611	low density lipoprotein cholesterol measurement	low density lipoprotein cholesterol measurement	EFO_0004611	Cardiovascular measurement	Cardiovascular	PGS001933	EFO_0004611	low density lipoprotein cholesterol measurement	measurement	EFO_0004611	Lipid or lipoprotein measurement	Metabolic
0.919	4.30E-186	2.22E-182	PGS002337	EFO_0004611	low density lipoprotein cholesterol measurement	low density lipoprotein cholesterol measurement	EFO_0004611	Lipid or lipoprotein measurement	Metabolic	PGS001933	EFO_0004611	low density lipoprotein cholesterol measurement	measurement	EFO_0004611	Lipid or lipoprotein measurement	Metabolic
0.919	4.30E-186	2.22E-182	PGS002337	EFO_0004611	low density lipoprotein cholesterol measurement	low density lipoprotein cholesterol measurement	EFO_0004611	Lipid or lipoprotein measurement	Metabolic	PGS001933	EFO_0004611	low density lipoprotein cholesterol measurement	measurement	EFO_0004611	Cardiovascular measurement	Cardiovascular
0.919	4.89E-186	2.53E-182	PGS002530	MONDO_0004979	asthma	asthma	MONDO_0004979	Other disease	Respiratory	PGS002566	EFO_0000684	respiratory system disease	respiratory system disease	EFO_0000684	Other disease	Skeletal
0.9188	7.59E-186	3.91E-182	PGS002409	EFO_0004611	low density lipoprotein cholesterol measurement	low density lipoprotein cholesterol measurement	EFO_0004611	Lipid or lipoprotein measurement	Metabolic	PGS002458	EFO_0004611	low density lipoprotein cholesterol measurement	measurement	EFO_0004611	Lipid or lipoprotein measurement	Metabolic
0.9188	7.59E-186	3.91E-182	PGS002409	EFO_0004611	low density lipoprotein cholesterol measurement	low density lipoprotein cholesterol measurement	EFO_0004611	Lipid or lipoprotein measurement	Metabolic	PGS002458	EFO_0004611	low density lipoprotein cholesterol measurement	measurement	EFO_0004611	Cardiovascular measurement	Cardiovascular
0.9188	7.59E-186	3.91E-182	PGS002409	EFO_0004611	low density lipoprotein cholesterol measurement	low density lipoprotein cholesterol measurement	EFO_0004611	Cardiovascular measurement	Cardiovascular	PGS002458	EFO_0004611	low density lipoprotein cholesterol measurement	measurement	EFO_0004611	Lipid or lipoprotein measurement	Metabolic
0.9188	7.59E-186	3.91E-182	PGS002409	EFO_0004611	low density lipoprotein cholesterol measurement	low density lipoprotein cholesterol measurement	EFO_0004611	Cardiovascular measurement	Cardiovascular	PGS002458	EFO_0004611	low density lipoprotein cholesterol measurement	measurement	EFO_0004611	Cardiovascular measurement	Cardiovascular
0.9188	8.51E-186	4.39E-182	PGS002107	EFO_0001060	celiac disease	celiac disease	EFO_0001060	Immune system disorder	Immunological	PGS000040	EFO_0001060	celiac disease	celiac disease	EFO_0001060	Digestive system disorder	Gastrointestinal
0.9188	8.51E-186	4.39E-182	PGS002107	EFO_0001060	celiac disease	celiac disease	EFO_0001060	Digestive system disorder	Gastrointestinal	PGS000040	EFO_0001060	celiac disease	celiac disease	EFO_0001060	Digestive system disorder	Gastrointestinal
0.9188	8.51E-186	4.39E-182	PGS002107	EFO_0001060	celiac disease	celiac disease	EFO_0001060	Immune system disorder	Immunological	PGS000040	EFO_0001060	celiac disease	celiac disease	EFO_0001060	Immune system disorder	Immunological
0.9188	8.51E-186	4.39E-182	PGS002107	EFO_0001060	celiac disease	celiac disease	EFO_0001060	Digestive system disorder	Gastrointestinal	PGS000040	EFO_0001060	celiac disease	celiac disease	EFO_0001060	Immune system disorder	Immunological
0.9187	1.10E-185	5.68E-182	PGS002897	EFO_0006336	diastolic blood pressure	diastolic blood pressure	EFO_0006336	Other measurement	Cardiovascular	PGS002892	EFO_0006336	diastolic blood pressure	diastolic blood pressure	EFO_0006336	Other measurement	Cardiovascular
0.9186	1.32E-185	6.77E-182	PGS003036	EFO_0004611	low density lipoprotein cholesterol measurement	low density lipoprotein cholesterol measurement	EFO_0004611	Cardiovascular measurement	Cardiovascular	PGS003030	EFO_0004611	low density lipoprotein cholesterol measurement	measurement	EFO_0004611	Lipid or lipoprotein measurement	Metabolic
0.9186	1.32E-185	6.77E-182	PGS003036	EFO_0004611	low density lipoprotein cholesterol measurement	low density lipoprotein cholesterol measurement	EFO_0004611	Lipid or lipoprotein measurement	Metabolic	PGS003030	EFO_0004611	low density lipoprotein cholesterol measurement	measurement	EFO_0004611	Lipid or lipoprotein measurement	Metabolic
0.9186	1.32E-185	6.77E-182	PGS003036	EFO_0004611	low density lipoprotein cholesterol measurement	low density lipoprotein cholesterol measurement	EFO_0004611	Lipid or lipoprotein measurement	Metabolic	PGS003030	EFO_0004611	low density lipoprotein cholesterol measurement	measurement	EFO_0004611	Cardiovascular measurement	Cardiovascular
0.9186	1.32E-185	6.77E-182	PGS003036	EFO_0004611	low density lipoprotein cholesterol measurement	low density lipoprotein cholesterol measurement	EFO_0004611	Cardiovascular measurement	Cardiovascular	PGS003030	EFO_0004611	low density lipoprotein cholesterol measurement	measurement	EFO_0004611	Cardiovascular measurement	Cardiovascular
0.9185	1.66E-185	8.54E-182	PGS002396	EFO_0001379	endocrine system disease|diabetes mellitus	endocrine system disease	EFO_0001379	Other disease	Metabolic	PGS002596	EFO_0004468	glucose measurement	glucose measurement	EFO_0004468	Other measurement	Metabolic
0.9185	1.82E-185	9.34E-182	PGS002543	EFO_0001379	endocrine system disease|diabetes mellitus	endocrine system disease	EFO_0001379	Other disease	Metabolic	PGS002442	EFO_0000400	diabetes mellitus	diabetes mellitus	EFO_0000400	Metabolic disorder	Metabolic
0.9185	1.82E-185	9.34E-182	PGS002543	EFO_0001379	endocrine system disease|diabetes mellitus	endocrine system disease	EFO_0001379	Other disease	Metabolic	PGS002442	EFO_0000400	diabetes mellitus	diabetes mellitus	EFO_0000400	Digestive system disorder	Gastrointestinal
0.9184	2.25E-185	1.15E-181	PGS000401	EFO_0009259	skin carcinoma	skin carcinoma	EFO_0009259	Cancer	Neoplasms	PGS000434	MONDO_0002898	skin cancer	skin cancer	MONDO_0002898	Cancer	Neoplasms
0.9184	2.35E-185	1.20E-181	PGS002883	EFO_0004458	C-reactive protein measurement	C-reactive protein measurement	EFO_0004458	Inflammatory measurement	Immunological	PGS002863	EFO_0004458	C-reactive protein measurement	C-reactive protein measurement	EFO_0004458	Inflammatory measurement	Immunological
0.9183	3.05E-185	1.56E-181	PGS002589	EFO_0000400	diabetes mellitus	diabetes mellitus	EFO_0000400	Metabolic disorder	Metabolic	PGS002573	MONDO_0005148	type 2 diabetes mellitus	type 2 diabetes mellitus	MONDO_0005148	Metabolic disorder	Metabolic
0.9183	3.05E-185	1.56E-181	PGS002589	EFO_0000400	diabetes mellitus	diabetes mellitus	EFO_0000400	Metabolic disorder	Metabolic	PGS002573	MONDO_0005148	type 2 diabetes mellitus	type 2 diabetes mellitus	MONDO_0005148	Digestive system disorder	Gastrointestinal
0.9183	3.05E-185	1.56E-181	PGS002589	EFO_0000400	diabetes mellitus	diabetes mellitus	EFO_0000400	Digestive system disorder	Gastrointestinal	PGS002573	MONDO_0005148	type 2 diabetes mellitus	type 2 diabetes mellitus	MONDO_0005148	Digestive system disorder	Gastrointestinal
0.9183	3.05E-185	1.56E-181	PGS002589	EFO_0000400	diabetes mellitus	diabetes mellitus	EFO_0000400	Digestive system disorder	Gastrointestinal	PGS002573	MONDO_0005148	type 2 diabetes mellitus	type 2 diabetes mellitus	MONDO_0005148	Metabolic disorder	Metabolic
0.9183	3.18E-185	1.62E-181	PGS002150	EFO_0004611	low density lipoprotein cholesterol measurement	low density lipoprotein cholesterol measurement	EFO_0004611	Cardiovascular measurement	Cardiovascular	PGS001889	EFO_0004615	apolipoprotein B measurement	apolipoprotein B measurement	EFO_0004615	Lipid or lipoprotein measurement	Metabolic
0.9183	3.18E-185	1.62E-181	PGS002150	EFO_0004611	low density lipoprotein cholesterol measurement	low density lipoprotein cholesterol measurement	EFO_0004611	Lipid or lipoprotein measurement	Metabolic	PGS001889	EFO_0004615	apolipoprotein B measurement	apolipoprotein B measurement	EFO_0004615	Lipid or lipoprotein measurement	Metabolic
0.9183	3.57E-185	1.82E-181	PGS000169	EFO_0007986	reticulocyte count	reticulocyte count	EFO_0007986	Hematological measurement	Hematological	PGS000170	EFO_0007986	reticulocyte count	reticulocyte count	EFO_0007986	Hematological measurement	Hematological
0.9181	4.92E-185	2.51E-181	PGS002406	HP_0003124	Hypercholesterolemia	Hypercholesterolemia	HP_0003124	Other trait	Metabolic	PGS002455	HP_0003124	Hypercholesterolemia	Hypercholesterolemia	HP_0003124	Other trait	Metabolic
0.9179	9.19E-185	4.68E-181	PGS002547	EFO_0004468	glucose measurement	glucose measurement	EFO_0004468	Other measurement	Metabolic	PGS002403	EFO_0004541	HbA1c measurement	HbA1c measurement	EFO_0004541	Hematological measurement	Hematological
0.9179	9.36E-185	4.76E-181	PGS000424	EFO_0000756	melanoma	melanoma	EFO_0000756	Cancer	Neoplasms	PGS000422	EFO_0000756	melanoma	melanoma	EFO_0000756	Cancer	Neoplasms
0.9179	9.36E-185	4.76E-181	PGS000424	EFO_0000756	melanoma	melanoma	EFO_0000756	Cancer	Neoplasms	PGS000421	EFO_0000756	melanoma	melanoma	EFO_0000756	Cancer	Neoplasms
0.9178	1.19E-184	6.04E-181	PGS003088	EFO_0006335	systolic blood pressure	systolic blood pressure	EFO_0006335	Other measurement	Cardiovascular	PGS003083	EFO_0006335	systolic blood pressure	systolic blood pressure	EFO_0006335	Other measurement	Cardiovascular
0.9177	1.44E-184	7.31E-181	PGS002847	EFO_0004340	body mass index	body mass index	EFO_0004340	Body measurement	Body measurement	PGS002857	EFO_0004340	body mass index	body mass index	EFO_0004340	Body measurement	Body measurement
0.9177	1.56E-184	7.93E-181	PGS000507	EFO_0000305	breast carcinoma	breast carcinoma	EFO_0000305	Cancer	Neoplasms	PGS000500	EFO_0000305	breast carcinoma	breast carcinoma	EFO_0000305	Cancer	Neoplasms
0.9174	3.48E-184	1.76E-180	PGS001757	EFO_0006930	brain volume measurement|neuroimaging measurement	brain volume measurement	EFO_0006930	Other measurement	Neurological	PGS001727	EFO_0006930	measurement	brain volume measurement	EFO_0006930	Other measurement	Neurological
0.9172	5.28E-184	2.67E-180	PGS001915	EFO_0004703	age at menarche	age at menarche	EFO_0004703	Other measurement	Genitourinary	PGS002131	EFO_0004703	age at menarche	age at menarche	EFO_0004703	Other measurement	Genitourinary
0.9168	1.85E-183	9.34E-180	PGS001887	EFO_0004302	anthropometric measurement	anthropometric measurement	EFO_0004302	Other measurement	Body measurement	PGS002100	EFO_0004302	anthropometric measurement	anthropometric measurement	EFO_0004302	Other measurement	Body measurement
0.9165	3.84E-183	1.94E-179	PGS001990	EFO_0004526	mean corpuscular volume	mean corpuscular volume	EFO_0004526	Hematological measurement	Hematological	PGS002206	EFO_0004527	mean corpuscular hemoglobin	mean corpuscular hemoglobin	EFO_0004527	Hematological measurement	Hematological
0.9163	5.81E-183	2.93E-179	PGS003398	EFO_0000305	breast carcinoma	breast carcinoma	EFO_0000305	Cancer	Neoplasms	PGS000052	EFO_0000305	breast carcinoma	breast carcinoma	EFO_0000305	Cancer	Neoplasms
0.9163	6.32E-183	3.18E-179	PGS002102	EFO_0004615	apolipoprotein B measurement	apolipoprotein B measurement	EFO_0004615	Lipid or lipoprotein measurement	Metabolic	PGS001933	EFO_0004611	low density lipoprotein cholesterol measurement	measurement	EFO_0004611	Lipid or lipoprotein measurement	Metabolic
0.9163	6.32E-183	3.18E-179	PGS002102	EFO_0004615	apolipoprotein B measurement	apolipoprotein B measurement	EFO_0004615	Lipid or lipoprotein measurement	Metabolic	PGS001933	EFO_0004611	low density lipoprotein cholesterol measurement	measurement	EFO_0004611	Cardiovascular measurement	Cardiovascular
0.9163	6.59E-183	3.31E-179	PGS000499	EFO_0000305	breast carcinoma	breast carcinoma	EFO_0000305	Cancer	Neoplasms	PGS000507	EFO_0000305	breast carcinoma	breast carcinoma	EFO_0000305	Cancer	Neoplasms
0.9162	7.82E-183	3.93E-179	PGS002764	HP_0003124	Hypercholesterolemia	Hypercholesterolemia	HP_0003124	Other trait	Metabolic	PGS003033	EFO_0004611	low density lipoprotein cholesterol measurement	measurement	EFO_0004611	Cardiovascular measurement	Cardiovascular
0.9162	7.82E-183	3.93E-179	PGS002764	HP_0003124	Hypercholesterolemia	Hypercholesterolemia	HP_0003124	Other trait	Metabolic	PGS003033	EFO_0004611	low density lipoprotein cholesterol measurement	measurement	EFO_0004611	Lipid or lipoprotein measurement	Metabolic
0.916	1.24E-182	6.24E-179	PGS001992	EFO_0008328	chronotype measurement	chronotype measurement	EFO_0008328	Other measurement	Psychiatric	PGS002209	EFO_0008328	chronotype measurement	chronotype measurement	EFO_0008328	Other measurement	Psychiatric
0.9159	1.79E-182	8.98E-179	PGS000058	EFO_0001645	coronary artery disease	coronary artery disease	EFO_0001645	Cardiovascular disease	Cardiovascular	PGS000899	EFO_0001645	coronary artery disease	coronary artery disease	EFO_0001645	Cardiovascular disease	Cardiovascular

0.9158	2.23E-182	1.12E-178	PGS002547	EFO_0004468	glucose measurement	glucose measurement	EFO_0004468	Other measurement	Metabolic	PGS002573	MONDO_0005148	type 2 diabetes mellitus	type 2 diabetes mellitus	MONDO_0005148	Digestive system disorder	Gastrointestinal
0.9158	2.23E-182	1.12E-178	PGS002547	EFO_0004468	glucose measurement	glucose measurement	EFO_0004468	Other measurement	Metabolic	PGS002573	MONDO_0005148	type 2 diabetes mellitus	type 2 diabetes mellitus	MONDO_0005148	Metabolic disorder	Metabolic
0.9157	2.59E-182	1.29E-178	PGS002475	MONDO_0005148	type 2 diabetes mellitus	type 2 diabetes mellitus	MONDO_0005148	Digestive system disorder	Gastrointestinal	PGS002592	EFO_0001379	endocrine system disease|diabetes mellitus	endocrine system disease	EFO_0001379	Other disease	Metabolic
0.9157	2.59E-182	1.29E-178	PGS002475	MONDO_0005148	type 2 diabetes mellitus	type 2 diabetes mellitus	MONDO_0005148	Metabolic disorder	Metabolic	PGS002592	EFO_0001379	endocrine system disease|diabetes mellitus	endocrine system disease	EFO_0001379	Other disease	Metabolic
0.9157	2.66E-182	1.33E-178	PGS003312	EFO_0004617	cystatin C measurement|glomerular filtration rate	cystatin C measurement	EFO_0004617	Other measurement	Metabolic	PGS003310	EFO_0004617	cystatin C measurement|glomerular filtration rate	cystatin C measurement	EFO_0004617	Other measurement	Metabolic
0.9155	5.02E-182	2.51E-178	PGS001220	EFO_0004526	mean corpuscular volume	mean corpuscular volume	EFO_0004526	Hematological measurement	Hematological	PGS001219	EFO_0004527	mean corpuscular hemoglobin	mean corpuscular hemoglobin	EFO_0004527	Hematological measurement	Hematological
0.9154	6.06E-182	3.02E-178	PGS001360	EFO_0004731	eye measurement	eye measurement	EFO_0004731	Other measurement	Ophthalmological	PGS001359	EFO_0004731	eye measurement	eye measurement	EFO_0004731	Other measurement	Ophthalmological
0.9154	7.12E-182	3.55E-178	PGS002605	EFO_0004611	low density lipoprotein cholesterol measurement	low density lipoprotein cholesterol measurement	EFO_0004611	Cardiovascular measurement	Cardiovascular	PGS002571	EFO_0004574	total cholesterol measurement	total cholesterol measurement	EFO_0004574	Lipid or lipoprotein measurement	Metabolic
0.9154	7.12E-182	3.55E-178	PGS002605	EFO_0004611	low density lipoprotein cholesterol measurement	low density lipoprotein cholesterol measurement	EFO_0004611	Lipid or lipoprotein measurement	Metabolic	PGS002571	EFO_0004574	total cholesterol measurement	total cholesterol measurement	EFO_0004574	Lipid or lipoprotein measurement	Metabolic
0.9153	8.01E-182	3.99E-178	PGS002409	EFO_0004611	low density lipoprotein cholesterol measurement	low density lipoprotein cholesterol measurement	EFO_0004611	Cardiovascular measurement	Cardiovascular	PGS002424	EFO_0004574	total cholesterol measurement	total cholesterol measurement	EFO_0004574	Lipid or lipoprotein measurement	Metabolic
0.9153	8.01E-182	3.99E-178	PGS002409	EFO_0004611	low density lipoprotein cholesterol measurement	low density lipoprotein cholesterol measurement	EFO_0004611	Lipid or lipoprotein measurement	Metabolic	PGS002424	EFO_0004574	total cholesterol measurement	total cholesterol measurement	EFO_0004574	Lipid or lipoprotein measurement	Metabolic
0.9152	9.75E-182	4.85E-178	PGS003297	EFO_0004617	cystatin C measurement|glomerular filtration rate	cystatin C measurement	EFO_0004617	Other measurement	Metabolic	PGS003310	EFO_0004617	cystatin C measurement|glomerular filtration rate	cystatin C measurement	EFO_0004617	Other measurement	Metabolic
0.9152	1.03E-181	5.09E-178	PGS001264	EFO_0003907	deep vein thrombosis	deep vein thrombosis	EFO_0003907	Other trait	Cardiovascular	PGS001266	EFO_0003907	deep vein thrombosis	deep vein thrombosis	EFO_0003907	Other trait	Cardiovascular
0.9152	1.02E-181	5.09E-178	PGS001102	EFO_0007800	body fat percentage	body fat percentage	EFO_0007800	Body measurement	Body measurement	PGS001101	EFO_0007800	body fat percentage	body fat percentage	EFO_0007800	Body measurement	Body measurement
0.915	1.67E-181	8.28E-178	PGS002530	MONDO_0004979	asthma	asthma	MONDO_0004979	Other disease	Respiratory	PGS002615	EFO_0000684	respiratory system disease	respiratory system disease	EFO_0000684	Other disease	Skeletal
0.9149	2.27E-181	1.12E-177	PGS000774	EFO_1000649	estrogen-receptor positive breast cancer	estrogen-receptor positive breast cancer	EFO_1000649	Cancer	Neoplasms	PGS003398	EFO_0000305	breast carcinoma	breast carcinoma	EFO_0000305	Cancer	Neoplasms
0.9149	2.43E-181	1.20E-177	PGS002545	EFO_0004713	FEV|FEC ratio	FEV|FEC ratio	EFO_0004713	Other measurement	Respiratory	PGS002398	EFO_0004713	FEV|FEC ratio	FEV|FEC ratio	EFO_0004713	Other measurement	Respiratory
0.9147	3.40E-181	1.68E-177	PGS000508	EFO_0000305	breast carcinoma	breast carcinoma	EFO_0000305	Cancer	Neoplasms	PGS000500	EFO_0000305	breast carcinoma	breast carcinoma	EFO_0000305	Cancer	Neoplasms
0.9147	3.63E-181	1.79E-177	PGS001803	EFO_0009259	skin carcinoma	skin carcinoma	EFO_0009259	Cancer	Neoplasms	PGS002014	EFO_0009259	skin carcinoma	skin carcinoma	EFO_0009259	Cancer	Neoplasms
-0.9145	6.26E-181	3.08E-177	PGS001092	EFO_0007822	hair colour measurement	hair colour measurement	EFO_0007822	Other measurement	Dermatological	PGS001093	EFO_0007822	hair colour measurement	hair colour measurement	EFO_0007822	Other measurement	Dermatological
0.9144	7.76E-181	3.82E-177	PGS000508	EFO_0000305	breast carcinoma	breast carcinoma	EFO_0000305	Cancer	Neoplasms	PGS000499	EFO_0000305	breast carcinoma	breast carcinoma	EFO_0000305	Cancer	Neoplasms
0.9144	8.56E-181	4.21E-177	PGS001042	EFO_0009190	Thyrotoxicosis	Thyrotoxicosis	EFO_0009190	Immune system disorder	Immunological	PGS001043	EFO_0009189	hyperthyroidism|Thyrotoxicosis	hyperthyroidism	EFO_0009189	Other disease	Endocrine
0.9144	8.69E-181	4.27E-177	PGS001297	MONDO_0005147	type 1 diabetes mellitus	type 1 diabetes mellitus	MONDO_0005147	Immune system disorder	Immunological	PGS001117	EFO_0600082	insulin use measurement	insulin use measurement	EFO_0600082	Other measurement	Metabolic
0.9144	8.69E-181	4.27E-177	PGS001297	MONDO_0005147	type 1 diabetes mellitus	type 1 diabetes mellitus	MONDO_0005147	Digestive system disorder	Gastrointestinal	PGS001117	EFO_0600082	insulin use measurement	insulin use measurement	EFO_0600082	Other measurement	Metabolic
0.9144	8.69E-181	4.27E-177	PGS001297	MONDO_0005147	type 1 diabetes mellitus	type 1 diabetes mellitus	MONDO_0005147	Metabolic disorder	Metabolic	PGS001117	EFO_0600082	insulin use measurement	insulin use measurement	EFO_0600082	Other measurement	Metabolic
0.9142	1.47E-180	7.24E-177	PGS001849	MONDO_0004979	asthma	asthma	MONDO_0004979	Other disease	Respiratory	PGS002061	MONDO_0004979	asthma	asthma	MONDO_0004979	Other disease	Respiratory
0.9141	1.60E-180	7.85E-177	PGS002540	EFO_0000400	diabetes mellitus	diabetes mellitus	EFO_0000400	Digestive system disorder	Gastrointestinal	PGS002547	EFO_0004468	glucose measurement	glucose measurement	EFO_0004468	Other measurement	Metabolic
0.9141	1.60E-180	7.85E-177	PGS002540	EFO_0000400	diabetes mellitus	diabetes mellitus	EFO_0000400	Metabolic disorder	Metabolic	PGS002547	EFO_0004468	glucose measurement	glucose measurement	EFO_0004468	Other measurement	Metabolic
0.9141	1.82E-180	8.94E-177	PGS001724	EFO_0006930	brain volume measurement|neuroimaging measurement	brain volume measurement	EFO_0006930	Other measurement	Neurological	PGS001753	EFO_0006930	measurement	brain volume measurement	EFO_0006930	Other measurement	Neurological
0.9139	2.59E-180	1.27E-176	PGS003238	EFO_0010472	creatine measurement	creatine measurement	EFO_0010472	Other measurement	Metabolic	PGS003233	EFO_0010472	creatine measurement	creatine measurement	EFO_0010472	Other measurement	Metabolic
0.9139	2.82E-180	1.38E-176	PGS000499	EFO_0000305	breast carcinoma	breast carcinoma	EFO_0000305	Cancer	Neoplasms	PGS000335	EFO_0000305	breast carcinoma	breast carcinoma	EFO_0000305	Cancer	Neoplasms
0.9138	3.87E-180	1.89E-176	PGS002475	MONDO_0005148	type 2 diabetes mellitus	type 2 diabetes mellitus	MONDO_0005148	Digestive system disorder	Gastrointestinal	PGS002491	EFO_0000400	diabetes mellitus	diabetes mellitus	EFO_0000400	Digestive system disorder	Gastrointestinal
0.9138	3.87E-180	1.89E-176	PGS002475	MONDO_0005148	type 2 diabetes mellitus	type 2 diabetes mellitus	MONDO_0005148	Digestive system disorder	Gastrointestinal	PGS002491	EFO_0000400	diabetes mellitus	diabetes mellitus	EFO_0000400	Metabolic disorder	Metabolic
0.9138	3.87E-180	1.89E-176	PGS002475	MONDO_0005148	type 2 diabetes mellitus	type 2 diabetes mellitus	MONDO_0005148	Metabolic disorder	Metabolic	PGS002491	EFO_0000400	diabetes mellitus	diabetes mellitus	EFO_0000400	Digestive system disorder	Gastrointestinal
0.9138	3.87E-180	1.89E-176	PGS002475	MONDO_0005148	type 2 diabetes mellitus	type 2 diabetes mellitus	MONDO_0005148	Metabolic disorder	Metabolic	PGS002491	EFO_0000400	diabetes mellitus	diabetes mellitus	EFO_0000400	Metabolic disorder	Metabolic
0.9137	4.31E-180	2.10E-176	PGS000596	EFO_0005088	testicular carcinoma	testicular carcinoma	EFO_0005088	Cancer	Neoplasms	PGS000598	EFO_0005088	testicular carcinoma	testicular carcinoma	EFO_0005088	Cancer	Neoplasms
0.9137	4.91E-180	2.40E-176	PGS001488	EFO_0006930	brain volume measurement|neuroimaging measurement	brain volume measurement	EFO_0006930	Other measurement	Neurological	PGS001489	EFO_0006930	measurement	brain volume measurement	EFO_0006930	Other measurement	Neurological
0.9136	5.61E-180	2.73E-176	PGS003193	EFO_0004631	vitamin D measurement	vitamin D measurement	EFO_0004631	Lipid or lipoprotein measurement	Metabolic	PGS002355	EFO_0004631	vitamin D measurement	vitamin D measurement	EFO_0004631	Lipid or lipoprotein measurement	Metabolic
0.9136	5.70E-180	2.77E-176	PGS001994	HP_0000545	Myopia	Myopia	HP_0000545	Other trait	Ophthalmological	PGS002211	HP_0000545	Myopia	Myopia	HP_0000545	Other trait	Ophthalmological
0.9135	7.46E-180	3.63E-176	PGS002442	EFO_0000400	diabetes mellitus	diabetes mellitus	EFO_0000400	Digestive system disorder	Gastrointestinal	PGS002491	EFO_0000400	diabetes mellitus	diabetes mellitus	EFO_0000400	Metabolic disorder	Metabolic
0.9135	7.46E-180	3.63E-176	PGS002442	EFO_0000400	diabetes mellitus	diabetes mellitus	EFO_0000400	Metabolic disorder	Metabolic	PGS002491	EFO_0000400	diabetes mellitus	diabetes mellitus	EFO_0000400	Digestive system disorder	Gastrointestinal
0.9135	7.46E-180	3.63E-176	PGS002442	EFO_0000400	diabetes mellitus	diabetes mellitus	EFO_0000400	Metabolic disorder	Metabolic	PGS002491	EFO_0000400	diabetes mellitus	diabetes mellitus	EFO_0000400	Metabolic disorder	Metabolic
0.9135	7.46E-180	3.63E-176	PGS002442	EFO_0000400	diabetes mellitus	diabetes mellitus	EFO_0000400	Digestive system disorder	Gastrointestinal	PGS002491	EFO_0000400	diabetes mellitus	diabetes mellitus	EFO_0000400	Digestive system disorder	Gastrointestinal
0.9134	1.06E-179	5.14E-176	PGS002150	EFO_0004611	low density lipoprotein cholesterol measurement	low density lipoprotein cholesterol measurement	EFO_0004611	Lipid or lipoprotein measurement	Metabolic	PGS002108	EFO_0004574	total cholesterol measurement	total cholesterol measurement	EFO_0004574	Lipid or lipoprotein measurement	Metabolic
0.9134	1.06E-179	5.14E-176	PGS002150	EFO_0004611	low density lipoprotein cholesterol measurement	low density lipoprotein cholesterol measurement	EFO_0004611	Cardiovascular measurement	Cardiovascular	PGS002108	EFO_0004574	total cholesterol measurement	total cholesterol measurement	EFO_0004574	Lipid or lipoprotein measurement	Metabolic
0.9132	1.77E-179	8.56E-176	PGS000335	EFO_0000305	breast carcinoma	breast carcinoma	EFO_0000305	Cancer	Neoplasms	PGS000500	EFO_0000305	breast carcinoma	breast carcinoma	EFO_0000305	Cancer	Neoplasms
0.9131	2.36E-179	1.14E-175	PGS002986	EFO_0004339	body height	body height	EFO_0004339	Body measurement	Body measurement	PGS002975	EFO_0004339	body height	body height	EFO_0004339	Body measurement	Body measurement
0.9131	2.43E-179	1.18E-175	PGS002534	EFO_0003923	bone density	bone density	EFO_0003923	Other measurement	Skeletal	PGS002387	EFO_0003923	bone density	bone density	EFO_0003923	Other measurement	Skeletal
0.9131	2.45E-179	1.18E-175	PGS003310	EFO_0004617	cystatin C measurement|glomerular filtration rate	cystatin C measurement	EFO_0004617	Other measurement	Metabolic	PGS003298	EFO_0004617	cystatin C measurement|glomerular filtration rate	cystatin C measurement	EFO_0004617	Other measurement	Metabolic
0.913	2.70E-179	1.30E-175	PGS002475	MONDO_0005148	type 2 diabetes mellitus	type 2 diabetes mellitus	MONDO_0005148	Metabolic disorder	Metabolic	PGS002622	MONDO_0005148	type 2 diabetes mellitus	type 2 diabetes mellitus	MONDO_0005148	Digestive system disorder	Gastrointestinal
0.913	2.70E-179	1.30E-175	PGS002475	MONDO_0005148	type 2 diabetes mellitus	type 2 diabetes mellitus	MONDO_0005148	Metabolic disorder	Metabolic	PGS002622	MONDO_0005148	type 2 diabetes mellitus	type 2 diabetes mellitus	MONDO_0005148	Metabolic disorder	Metabolic
0.913	2.70E-179	1.30E-175	PGS002475	MONDO_0005148	type 2 diabetes mellitus	type 2 diabetes mellitus	MONDO_0005148	Digestive system disorder	Gastrointestinal	PGS002622	MONDO_0005148	type 2 diabetes mellitus	type 2 diabetes mellitus	MONDO_0005148	Metabolic disorder	Metabolic
0.913	2.70E-179	1.30E-175	PGS002475	MONDO_0005148	type 2 diabetes mellitus	type 2 diabetes mellitus	MONDO_0005148	Digestive system disorder	Gastrointestinal	PGS002622	MONDO_0005148	type 2 diabetes mellitus	type 2 diabetes mellitus	MONDO_0005148	Digestive system disorder	Gastrointestinal
0.9129	3.66E-179	1.77E-175	PGS002118	EFO_0004462	PR interval	PR interval	EFO_0004462	Cardiovascular measurement	Cardiovascular	PGS001904	EFO_0004462	PR interval	PR interval	EFO_0004462	Cardiovascular measurement	Cardiovascular
0.9129	3.99E-179	1.92E-175	PGS002337	EFO_0004611	low density lipoprotein cholesterol measurement	low density lipoprotein cholesterol measurement	EFO_0004611	Lipid or lipoprotein measurement	Metabolic	PGS002352	EFO_0004574	total cholesterol measurement	total cholesterol measurement	EFO_0004574	Lipid or lipoprotein measurement	Metabolic
0.9129	3.99E-179	1.92E-175	PGS002337	EFO_0004611	low density lipoprotein cholesterol measurement	low density lipoprotein cholesterol measurement	EFO_0004611	Cardiovascular measurement	Cardiovascular	PGS002352	EFO_0004574	total cholesterol measurement	total cholesterol measurement	EFO_0004574	Lipid or lipoprotein measurement	Metabolic
0.9128	4.33E-179	2.09E-175	PGS002540	EFO_0000400	diabetes mellitus	diabetes mellitus	EFO_0000400	Metabolic disorder	Metabolic	PGS002442	EFO_0000400	diabetes mellitus	diabetes mellitus	EFO_0000400	Digestive system disorder	Gastrointestinal
0.9128	4.33E-179	2.09E-175	PGS002540	EFO_0000400	diabetes mellitus	diabetes mellitus	EFO_0000400	Digestive system disorder	Gastrointestinal	PGS002442	EFO_0000400	diabetes mellitus	diabetes mellitus	EFO_0000400	Metabolic disorder	Metabolic
0.9128	4.33E-179	2.09E-175	PGS002540	EFO_0000400	diabetes mellitus	diabetes mellitus	EFO_0000400	Digestive system disorder	Gastrointestinal	PGS002442	EFO_0000400	diabetes mellitus	diabetes mellitus	EFO_0000400	Digestive system disorder	Gastrointestinal
0.9128	4.33E-179	2.09E-175	PGS002540	EFO_0000400	diabetes mellitus	diabetes mellitus	EFO_0000400	Metabolic disorder	Metabolic	PGS002442	EFO_0000400	diabetes mellitus	diabetes mellitus	EFO_0000400	Metabolic disorder	Metabolic
0.9127	5.41E-179	2.60E-175	PGS000587	EFO_0001663	prostate carcinoma	prostate carcinoma	EFO_0001663	Cancer	Neoplasms	PGS000588	EFO_0001663	prostate carcinoma	prostate carcinoma	EFO_0001663	Cancer	Neoplasms
0.9127	5.88E-179	2.83E-175	PGS002426	MONDO_0005148	type 2 diabetes mellitus	type 2 diabetes mellitus	MONDO_0005148	Digestive system disorder	Gastrointestinal	PGS002547	EFO_0004468	glucose measurement	glucose measurement	EFO_0004468	Other measurement	Metabolic
0.9127	5.88E-179	2.83E-175	PGS002426	MONDO_0005148	type 2 diabetes mellitus	type 2 diabetes mellitus	MONDO_0005148	Metabolic disorder	Metabolic	PGS002547	EFO_0004468	glucose measurement	glucose measurement	EFO_0004468	Other measurement	Metabolic
0.9126	6.79E-179	3.26E-175	PGS002895	EFO_0006336	diastolic blood pressure	diastolic blood pressure	EFO_0006336	Other measurement	Cardiovascular	PGS002890	EFO_0006336	diastolic blood pressure	diastolic blood pressure	EFO_0006336	Other measurement	Cardiovascular
0.9126	6.92E-179	3.32E-175	PGS001959	EFO_0010700	reticulocyte measurement	reticulocyte measurement	EFO_0010700	Hematological measurement	Hematological	PGS001976	EFO_0007986	reticulocyte count	reticulocyte count	EFO_0007986	Hematological measurement	Hematological
0.9126	7.14E-179	3.42E-175	PGS001839	MONDO_0021661	coronary atherosclerosis	coronary atherosclerosis	MONDO_0021661	Cardiovascular disease	Cardiovascular	PGS002048	MONDO_0021661	coronary atherosclerosis	coronary atherosclerosis	MONDO_0021661	Cardiovascular disease	Cardiovascular
0.9121	2.39E-178	1.14E-174	PGS000429	EFO_0000756	melanoma	melanoma	EFO_0000756	Cancer	Neoplasms	PGS000431	EFO_0000756	melanoma	melanoma	EFO_0000756	Cancer	Neoplasms
0.912	3.52E-178	1.68E-174	PGS001824	Orphanet_309816	Disorder of bilirubin metabolism and excretion	Disorder of bilirubin metabolism and excretion	Orphanet_309816	Metabolic disorder	Metabolic	PGS002160	EFO_0004570	bilirubin measurement	bilirubin measurement	EFO_0004570	Other measurement	Metabolic
0.9118	5.01E-178	2.40E-174	PGS002146	EFO_0004339	body height	body height	EFO_0004339	Body measurement	Body measurement	PGS002332	EFO_0004339	body height	body height	EFO_0004339	Body measurement	Body measurement
0.9118	5.04E-178	2.41E-174	PGS000521	EFO_0000305	breast carcinoma	breast carcinoma	EFO_0000305	Cancer	Neoplasms	PGS000522	EFO_0000305	breast carcinoma	breast carcinoma	EFO_0000305	Cancer	Neoplasms
0.9118	5.09E-178	2.43E-174	PGS001989	EFO_0004527	mean corpuscular hemoglobin	mean corpuscular hemoglobin	EFO_0004527	Hematological measurement	Hematological	PGS002207	EFO_0004526	mean corpuscular volume	mean corpuscular volume	EFO_0004526	Hematological measurement	Hematological
0.9118	5.91E-178	2.81E-174	PGS002173	EFO_0009270	heel bone mineral density	heel bone mineral density	EFO_0009270	Other measurement	Skeletal	PGS001957	EFO_0005654	velocity of sound measurement	velocity of sound measurement	EFO_0005654	Other measurement	Others
0.9117	6.19E-178	2.95E-174	PGS001724	EFO_0006930	brain volume measurement|neuroimaging measurement	brain volume measurement	EFO_0006930	Other measurement	Neurological	PGS001738	EFO_0006930	measurement	brain volume measurement	EFO_0006930	Other measurement	Neurological
0.9116	8.24E-178	3.92E-174	PGS002721	EFO_0004631	vitamin D measurement	vitamin D measurement	EFO_0004631	Lipid or lipoprotein measurement	Metabolic	PGS003198	EFO_0004631	vitamin D measurement	vitamin D measurement	EFO_0004631	Lipid or lipoprotein measurement	Metabolic
0.9116	9.96E-178	4.73E-174	PGS003123	MONDO_0005148	type 2 diabetes mellitus	type 2 diabetes mellitus	MONDO_0005148	Digestive system disorder	Gastrointestinal	PGS000014	MONDO_0005148	type 2 diabetes mellitus	type 2 diabetes mellitus	MONDO_0005148	Metabolic disorder	Metabolic
0.9116	9.96E-178	4.73E-174	PGS003123	MONDO_0005148	type 2 diabetes mellitus	type 2 diabetes mellitus	MONDO_0005148	Metabolic disorder	Metabolic	PGS000014	MONDO_0005148	type 2 diabetes mellitus	type 2 diabetes mellitus	MONDO_0005148	Digestive system disorder	Gastrointestinal
0.9116	9.96E-178	4.73E-174	PGS003123	MONDO_0005148	type 2 diabetes mellitus	type 2 diabetes mellitus	MONDO_0005148	Metabolic disorder	Metabolic	PGS000014	MONDO_0005148	type 2 diabetes mellitus	type 2 diabetes mellitus	MONDO_0005148	Metabolic disorder	Metabolic
0.9116	9.96E-178	4.73E-174	PGS003123	MONDO_0005148	type 2 diabetes mellitus	type 2 diabetes mellitus	MONDO_0005148	Digestive system disorder	Gastrointestinal	PGS000014	MONDO_0005148	type 2 diabetes mellitus	type 2 diabetes mellitus	MONDO_0005148	Digestive system disorder	Gastrointestinal
0.9114	1.51E-177	7.15E-174	PGS002540	EFO_0000400	diabetes mellitus	diabetes mellitus	EFO_0000400	Metabolic disorder	Metabolic	PGS002400	EFO_0004468	glucose measurement	glucose measurement	EFO_0004468	Other measurement	Metabolic
0.9114	1.51E-177	7.15E-174	PGS002540	EFO_0000400	diabetes mellitus	diabetes mellitus	EFO_0000400	Digestive system disorder	Gastrointestinal	PGS002400	EFO_0004468	glucose measurement	glucose measurement	EFO_0004468	Other measurement	Metabolic
0.9114	1.52E-177	7.19E-174	PGS002444	HP_0000964	Eczema	Eczema	HP_0000964	Other trait	Dermatological	PGS002493	HP_0000964	Eczema	Eczema	HP_0000964	Other trait	Dermatological
0.9113	1.77E-177	8.36E-174	PGS000662	EFO_0001663	prostate carcinoma	prostate carcinoma	EFO_0001663	Cancer	Neoplasms	PGS003331	EFO_0001663	prostate carcinoma	prostate carcinoma	EFO_0001663	Cancer	Neoplasms
0.9113	1.77E-177	8.36E-174	PGS003331	EFO_0001663	prostate carcinoma	prostate carcinoma	EFO_0001663	Cancer	Neoplasms	PGS003415	EFO_0001663	prostate carcinoma	prostate carcinoma	EFO_0001663	Cancer	Neoplasms
0.9111	2.96E-177	1.40E-173	PGS001996	EFO_0007660	neuroticism measurement	neuroticism measurement	EFO_0007660	Other measurement	Psychiatric	PGS002213	EFO_0007660	neuroticism measurement	neuroticism measurement	EFO_0007660	Other measurement	Psychiatric
0.9111	3.04E-177	1.43E-173	PGS001682	EFO_0006930	brain volume measurement|neuroimaging measurement	brain volume measurement	EFO_0006930	Other measurement	Neurological	PGS001683	EFO_0006930	measurement	brain volume measurement	EFO_0006930	Other measurement	Neurological
0.9111	3.19E-177	1.50E-173	PGS003143	EFO_0004574	total cholesterol measurement	total cholesterol measurement	EFO_0004574	Lipid or lipoprotein measurement	Metabolic	PGS003033	EFO_0004611	low density lipoprotein cholesterol measurement	measurement	EFO_0004611	Cardiovascular measurement	Cardiovascular
0.9111	3.19E-177	1.50E-173	PGS003143	EFO_0004574	total cholesterol measurement	total cholesterol measurement	EFO_0004574	Lipid or lipoprotein measurement	Metabolic	PGS003033	EFO_0004611	low density lipoprotein cholesterol measurement	measurement	EFO_0004611	Lipid or lipoprotein measurement	Metabolic
0.9109	5.11E-177	2.41E-173	PGS002251	EFO_0004340	body mass index	body mass index	EFO_0004340	Body measurement	Body measurement	PGS003400	EFO_0001073	obesity	obesity	EFO_0001073	Metabolic disorder	Metabolic
0.9109	5.30E-177	2.50E-173	PGS000317	EFO_0000305	breast carcinoma	breast carcinoma	EFO_0000305	Cancer	Neoplasms	PGS000774	EFO_1000649	estrogen-receptor positive breast cancer	cancer	EFO_1000649	Cancer	Neoplasms
0.9108	5.91E-177	2.78E-173	PGS002403	EFO_0004541	HbA1c measurement	HbA1c measurement	EFO_0004541	Hematological measurement	Hematological	PGS002622	MONDO_0005148	type 2 diabetes mellitus	type 2 diabetes mellitus	MONDO_0005148	Metabolic disorder	Metabolic
0.9108	5.91E-177	2.78E-173	PGS002403	EFO_0004541	HbA1c measurement	HbA1c measurement	EFO_0004541	Hematological measurement	Hematological	PGS002622	MONDO_0005148	type 2 diabetes mellitus	type 2 diabetes mellitus	MONDO_0005148	Digestive system disorder	Gastrointestinal
0.9108	6.48E-177	3.04E-173	PGS001968	EFO_0005091	monocyte count	monocyte count	EFO_0005091	Hematological measurement	Hematological	PGS002341	EFO_0005091	monocyte count	monocyte count	EFO_0005091	Hematological measurement	Hematological
0.9108	6.78E-177	3.18E-173	PGS002540	EFO_0000400	diabetes mellitus	diabetes mellitus	EFO_0000400	Digestive system disorder	Gastrointestinal	PGS002445	EFO_0001379	endocrine system disease|diabetes mellitus	endocrine system disease	EFO_0001379	Other disease	Metabolic
0.9108	6.78E-177	3.18E-173	PGS002540	EFO_0000400	diabetes mellitus	diabetes mellitus	EFO_0000400	Metabolic disorder	Metabolic	PGS002445	EFO_0001379	endocrine system disease|diabetes mellitus	endocrine system disease	EFO_0001379	Other disease	Metabolic
0.9106	9.30E-177	4.36E-173	PGS001792	MONDO_0005041	glaucoma	glaucoma	MONDO_0005041	Other disease	Ophthalmological	PGS001797	EFO_0004190	open-angle glaucoma	open-angle glaucoma	EFO_0004190	Other disease	Ophthalmological
0.9106	1.04E-176	4.89E-173	PGS000633	EFO_0002892	thyroid carcinoma	thyroid carcinoma	EFO_0002892	Cancer	Neoplasms	PGS000207	EFO_0002892	thyroid carcinoma	thyroid carcinoma	EFO_0002892	Cancer	Neoplasms
0.9105	1.45E-176	6.76E-173	PGS001895	EFO_0004574	total cholesterol measurement	total cholesterol measurement	EFO_0004574	Lipid or lipoprotein measurement	Metabolic	PGS001933	EFO_0004611	low density lipoprotein cholesterol measurement	measurement	EFO_0004611	Cardiovascular measurement	Cardiovascular
0.9105	1.45E-176	6.76E-173	PGS001895	EFO_0004574	total cholesterol measurement	total cholesterol measurement	EFO_0004574	Lipid or lipoprotein measurement	Metabolic	PGS001933	EFO_0004611	low density lipoprotein cholesterol measurement	measurement	EFO_0004611	Lipid or lipoprotein measurement	Metabolic
0.9103	2.30E-176	1.08E-172	PGS002961	EFO_0004612	high density lipoprotein cholesterol measurement	high density lipoprotein cholesterol measurement	EFO_0004612	Lipid or lipoprotein measurement	Metabolic	PGS002956	EFO_0004612	high density lipoprotein cholesterol measurement	measurement	EFO_0004612	Lipid or lipoprotein measurement	Metabolic
0.9102	2.87E-176	1.34E-172	PGS002177	EFO_0010700	reticulocyte measurement	reticulocyte measurement	EFO_0010700	Hematological measurement	Hematological	PGS002194	EFO_0007986	reticulocyte count	reticulocyte count	EFO_0007986	Hematological measurement	Hematological
0.9101	3.47E-176	1.62E-172	PGS003123	MONDO_0005148	type 2 diabetes mellitus	type 2 diabetes mellitus	MONDO_0005148	Digestive system disorder	Gastrointestinal	PGS002243	MONDO_0005148	type 2 diabetes mellitus	type 2 diabetes mellitus	MONDO_0005148	Digestive system disorder	Gastrointestinal
0.9101	3.47E-176	1.62E-172	PGS003123	MONDO_0005148	type 2 diabetes mellitus	type 2 diabetes mellitus	MONDO_0005148	Metabolic disorder	Metabolic	PGS002243	MONDO_0005148	type 2 diabetes mellitus	type 2 diabetes mellitus	MONDO_0005148	Metabolic disorder	Metabolic
0.9101	3.47E-176	1.62E-172	PGS003123	MONDO_0005148	type 2 diabetes mellitus	type 2 diabetes mellitus	MONDO_0005148	Metabolic disorder	Metabolic	PGS002243	MONDO_0005148	type 2 diabetes mellitus	type 2 diabetes mellitus	MONDO_0005148	Digestive system disorder	Gastrointestinal

0.9101	3.47E-176	1.62E-172	PGS003123	MONDO_0005148	type 2 diabetes mellitus	type 2 diabetes mellitus	MONDO_0005148	Digestive system disorder	Gastrointestinal	PGS002243	MONDO_0005148	type 2 diabetes mellitus	type 2 diabetes mellitus	MONDO_0005148	Metabolic disorder	Metabolic
0.91	4.73E-176	2.21E-172	PGS002393	EFO_0000400	diabetes mellitus	diabetes mellitus	EFO_0000400	Metabolic disorder	Metabolic	PGS002547	EFO_0004468	glucose measurement	glucose measurement	EFO_0004468	Other measurement	Metabolic
0.91	4.73E-176	2.21E-172	PGS002393	EFO_0000400	diabetes mellitus	diabetes mellitus	EFO_0000400	Digestive system disorder	Gastrointestinal	PGS002547	EFO_0004468	glucose measurement	glucose measurement	EFO_0004468	Other measurement	Metabolic
0.9099	5.46E-176	2.54E-172	PGS001154	EFO_0005106	body composition measurement	body composition measurement	EFO_0005106	Body measurement	Body measurement	PGS001156	EFO_0005106	body composition measurement	body composition measurement	EFO_0005106	Body measurement	Body measurement
0.9098	6.68E-176	3.11E-172	PGS002540	EFO_0000400	diabetes mellitus	diabetes mellitus	EFO_0000400	Metabolic disorder	Metabolic	PGS002475	MONDO_0005148	type 2 diabetes mellitus	type 2 diabetes mellitus	MONDO_0005148	Metabolic disorder	Metabolic
0.9098	6.68E-176	3.11E-172	PGS002540	EFO_0000400	diabetes mellitus	diabetes mellitus	EFO_0000400	Metabolic disorder	Metabolic	PGS002475	MONDO_0005148	type 2 diabetes mellitus	type 2 diabetes mellitus	MONDO_0005148	Digestive system disorder	Gastrointestinal
0.9098	6.68E-176	3.11E-172	PGS002540	EFO_0000400	diabetes mellitus	diabetes mellitus	EFO_0000400	Digestive system disorder	Gastrointestinal	PGS002475	MONDO_0005148	type 2 diabetes mellitus	type 2 diabetes mellitus	MONDO_0005148	Metabolic disorder	Metabolic
0.9098	6.68E-176	3.11E-172	PGS002540	EFO_0000400	diabetes mellitus	diabetes mellitus	EFO_0000400	Digestive system disorder	Gastrointestinal	PGS002475	MONDO_0005148	type 2 diabetes mellitus	type 2 diabetes mellitus	MONDO_0005148	Digestive system disorder	Gastrointestinal
0.9098	7.64E-176	3.55E-172	PGS002965	EFO_0004339	body height	body height	EFO_0004339	Body measurement	Body measurement	PGS002971	EFO_0004339	body height	body height	EFO_0004339	Body measurement	Body measurement
0.9096	1.02E-175	4.75E-172	PGS002445	EFO_0001379	endocrine system disease|diabetes mellitus	endocrine system disease	EFO_0001379	Other disease	Metabolic	PGS002622	MONDO_0005148	type 2 diabetes mellitus	type 2 diabetes mellitus	MONDO_0005148	Metabolic disorder	Metabolic
0.9096	1.02E-175	4.75E-172	PGS002445	EFO_0001379	endocrine system disease|diabetes mellitus	endocrine system disease	EFO_0001379	Other disease	Metabolic	PGS002622	MONDO_0005148	type 2 diabetes mellitus	type 2 diabetes mellitus	MONDO_0005148	Digestive system disorder	Gastrointestinal
0.9095	1.55E-175	7.21E-172	PGS002858	EFO_0004340	body mass index	body mass index	EFO_0004340	Body measurement	Body measurement	PGS002843	EFO_0004340	body mass index	body mass index	EFO_0004340	Body measurement	Body measurement
0.9094	1.64E-175	7.62E-172	PGS001787	MONDO_0004979	asthma	asthma	MONDO_0004979	Other disease	Respiratory	PGS001782	MONDO_0004979	asthma	asthma	MONDO_0004979	Other disease	Respiratory
0.9093	2.07E-175	9.59E-172	PGS002978	EFO_0004339	body height	body height	EFO_0004339	Body measurement	Body measurement	PGS002988	EFO_0004339	body height	body height	EFO_0004339	Body measurement	Body measurement
0.9092	3.16E-175	1.46E-171	PGS002432	MONDO_0004979	asthma	asthma	MONDO_0004979	Other disease	Respiratory	PGS002468	EFO_0000684	respiratory system disease	respiratory system disease	EFO_0000684	Other disease	Skeletal
0.9091	3.38E-175	1.56E-171	PGS002403	EFO_0004541	HbA1c measurement	HbA1c measurement	EFO_0004541	Hematological measurement	Hematological	PGS002596	EFO_0004468	glucose measurement	glucose measurement	EFO_0004468	Other measurement	Metabolic
0.909	5.26E-175	2.43E-171	PGS002567	EFO_0006527	smoking status measurement	smoking status measurement	EFO_0006527	Other measurement	Psychiatric	PGS002616	EFO_0006527	smoking status measurement	smoking status measurement	EFO_0006527	Other measurement	Psychiatric
0.9089	6.14E-175	2.83E-171	PGS000595	EFO_0005088	testicular carcinoma	testicular carcinoma	EFO_0005088	Cancer	Neoplasms	PGS000596	EFO_0005088	testicular carcinoma	testicular carcinoma	EFO_0005088	Cancer	Neoplasms
0.9088	7.26E-175	3.35E-171	PGS000957	MONDO_0001134	essential hypertension	essential hypertension	MONDO_0001134	Cardiovascular disease	Cardiovascular	PGS000958	MONDO_0001134	essential hypertension	essential hypertension	MONDO_0001134	Cardiovascular disease	Cardiovascular
0.9084	1.95E-174	8.96E-171	PGS001264	EFO_0003907	deep vein thrombosis	deep vein thrombosis	EFO_0003907	Other trait	Cardiovascular	PGS001278	EFO_0003827	pulmonary embolism|deep vein thrombosis	pulmonary embolism	EFO_0003827	Cardiovascular disease	Cardiovascular
0.9082	3.29E-174	1.51E-170	PGS003084	EFO_0006335	systolic blood pressure	systolic blood pressure	EFO_0006335	Other measurement	Cardiovascular	PGS003079	EFO_0006335	systolic blood pressure	systolic blood pressure	EFO_0006335	Other measurement	Cardiovascular
0.9081	4.12E-174	1.89E-170	PGS002403	EFO_0004541	HbA1c measurement	HbA1c measurement	EFO_0004541	Hematological measurement	Hematological	PGS002573	MONDO_0005148	type 2 diabetes mellitus	type 2 diabetes mellitus	MONDO_0005148	Digestive system disorder	Gastrointestinal
0.9081	4.12E-174	1.89E-170	PGS002403	EFO_0004541	HbA1c measurement	HbA1c measurement	EFO_0004541	Hematological measurement	Hematological	PGS002573	MONDO_0005148	type 2 diabetes mellitus	type 2 diabetes mellitus	MONDO_0005148	Metabolic disorder	Metabolic
0.9079	5.88E-174	2.70E-170	PGS002329	EFO_0004612	high density lipoprotein cholesterol measurement	high density lipoprotein cholesterol measurement	EFO_0004612	Lipid or lipoprotein measurement	Metabolic	PGS001954	EFO_0004612	high density lipoprotein cholesterol measurement	measurement	EFO_0004612	Lipid or lipoprotein measurement	Metabolic
0.9079	6.21E-174	2.85E-170	PGS001823	MONDO_0002279	iron metabolism disease	iron metabolism disease	MONDO_0002279	Metabolic disorder	Metabolic	PGS002031	MONDO_0002279	iron metabolism disease	iron metabolism disease	MONDO_0002279	Metabolic disorder	Metabolic
0.9079	6.56E-174	3.01E-170	PGS001633	EFO_0005420	measurement	grey matter volume measurement	EFO_0005420	Other measurement	Neurological	PGS001544	EFO_0005420	measurement	grey matter volume measurement	EFO_0005420	Other measurement	Neurological
0.9075	1.71E-173	7.80E-170	PGS001812	MONDO_0000385	benign digestive system neoplasm	benign digestive system neoplasm	MONDO_0000385	Cancer	Neoplasms	PGS002020	MONDO_0000385	benign digestive system neoplasm	benign digestive system neoplasm	MONDO_0000385	Cancer	Neoplasms
0.9075	1.71E-173	7.80E-170	PGS001812	MONDO_0000385	benign digestive system neoplasm	benign digestive system neoplasm	MONDO_0000385	Digestive system disorder	Gastrointestinal	PGS002020	MONDO_0000385	benign digestive system neoplasm	benign digestive system neoplasm	MONDO_0000385	Cancer	Neoplasms
0.9075	1.71E-173	7.80E-170	PGS001812	MONDO_0000385	benign digestive system neoplasm	benign digestive system neoplasm	MONDO_0000385	Digestive system disorder	Gastrointestinal	PGS002020	MONDO_0000385	benign digestive system neoplasm	benign digestive system neoplasm	MONDO_0000385	Digestive system disorder	Gastrointestinal
0.9075	1.71E-173	7.80E-170	PGS001812	MONDO_0000385	benign digestive system neoplasm	benign digestive system neoplasm	MONDO_0000385	Cancer	Neoplasms	PGS002020	MONDO_0000385	benign digestive system neoplasm	benign digestive system neoplasm	MONDO_0000385	Digestive system disorder	Gastrointestinal
0.9075	1.78E-173	8.15E-170	PGS002038	MONDO_0005301	multiple sclerosis	multiple sclerosis	MONDO_0005301	Immune system disorder	Immunological	PGS001831	MONDO_0005301	multiple sclerosis	multiple sclerosis	MONDO_0005301	Neurological disorder	Neurological
0.9075	1.78E-173	8.15E-170	PGS002038	MONDO_0005301	multiple sclerosis	multiple sclerosis	MONDO_0005301	Neurological disorder	Neurological	PGS001831	MONDO_0005301	multiple sclerosis	multiple sclerosis	MONDO_0005301	Neurological disorder	Neurological
0.9075	1.78E-173	8.15E-170	PGS002038	MONDO_0005301	multiple sclerosis	multiple sclerosis	MONDO_0005301	Immune system disorder	Immunological	PGS001831	MONDO_0005301	multiple sclerosis	multiple sclerosis	MONDO_0005301	Immune system disorder	Immunological
0.9075	1.78E-173	8.15E-170	PGS002038	MONDO_0005301	multiple sclerosis	multiple sclerosis	MONDO_0005301	Neurological disorder	Neurological	PGS001831	MONDO_0005301	multiple sclerosis	multiple sclerosis	MONDO_0005301	Immune system disorder	Immunological
0.9074	2.08E-173	9.51E-170	PGS000030	EFO_0001663	prostate carcinoma	prostate carcinoma	EFO_0001663	Cancer	Neoplasms	PGS002791	EFO_0001663	prostate carcinoma	prostate carcinoma	EFO_0001663	Cancer	Neoplasms
0.9071	4.09E-173	1.87E-169	PGS002620	EFO_0004574	total cholesterol measurement	total cholesterol measurement	EFO_0004574	Lipid or lipoprotein measurement	Metabolic	PGS002424	EFO_0004574	total cholesterol measurement	total cholesterol measurement	EFO_0004574	Lipid or lipoprotein measurement	Metabolic
0.9071	4.16E-173	1.90E-169	PGS001105	EFO_0007800	body fat percentage	body fat percentage	EFO_0007800	Body measurement	Body measurement	PGS001148	EFO_0005409	fat body mass	fat body mass	EFO_0005409	Other measurement	Body measurement
0.907	4.93E-173	2.25E-169	PGS001908	EFO_0005192	red blood cell distribution width	red blood cell distribution width	EFO_0005192	Hematological measurement	Hematological	PGS002346	EFO_0005192	red blood cell distribution width	red blood cell distribution width	EFO_0005192	Hematological measurement	Hematological
0.907	4.97E-173	2.26E-169	PGS002622	MONDO_0005148	type 2 diabetes mellitus	type 2 diabetes mellitus	MONDO_0005148	Digestive system disorder	Gastrointestinal	PGS002596	EFO_0004468	glucose measurement	glucose measurement	EFO_0004468	Other measurement	Metabolic
0.907	4.97E-173	2.26E-169	PGS002622	MONDO_0005148	type 2 diabetes mellitus	type 2 diabetes mellitus	MONDO_0005148	Metabolic disorder	Metabolic	PGS002596	EFO_0004468	glucose measurement	glucose measurement	EFO_0004468	Other measurement	Metabolic
0.907	5.06E-173	2.30E-169	PGS002846	EFO_0004340	body mass index	body mass index	EFO_0004340	Body measurement	Body measurement	PGS002841	EFO_0004340	body mass index	body mass index	EFO_0004340	Body measurement	Body measurement
0.907	5.67E-173	2.57E-169	PGS001160	EFO_0005106	body composition measurement	body composition measurement	EFO_0005106	Body measurement	Body measurement	PGS001146	EFO_0005409	fat body mass	fat body mass	EFO_0005409	Other measurement	Body measurement
0.9069	6.80E-173	3.09E-169	PGS002409	EFO_0004611	low density lipoprotein cholesterol measurement	low density lipoprotein cholesterol measurement	EFO_0004611	Lipid or lipoprotein measurement	Metabolic	PGS002556	EFO_0004611	low density lipoprotein cholesterol measurement	measurement	EFO_0004611	Lipid or lipoprotein measurement	Metabolic
0.9069	6.80E-173	3.09E-169	PGS002409	EFO_0004611	low density lipoprotein cholesterol measurement	low density lipoprotein cholesterol measurement	EFO_0004611	Lipid or lipoprotein measurement	Metabolic	PGS002556	EFO_0004611	low density lipoprotein cholesterol measurement	measurement	EFO_0004611	Cardiovascular measurement	Cardiovascular
0.9069	6.80E-173	3.09E-169	PGS002409	EFO_0004611	low density lipoprotein cholesterol measurement	low density lipoprotein cholesterol measurement	EFO_0004611	Cardiovascular measurement	Cardiovascular	PGS002556	EFO_0004611	low density lipoprotein cholesterol measurement	measurement	EFO_0004611	Cardiovascular measurement	Cardiovascular
0.9069	6.80E-173	3.09E-169	PGS002409	EFO_0004611	low density lipoprotein cholesterol measurement	low density lipoprotein cholesterol measurement	EFO_0004611	Cardiovascular measurement	Cardiovascular	PGS002556	EFO_0004611	low density lipoprotein cholesterol measurement	measurement	EFO_0004611	Lipid or lipoprotein measurement	Metabolic
0.9069	7.41E-173	3.36E-169	PGS002592	EFO_0001379	endocrine system disease|diabetes mellitus	endocrine system disease	EFO_0001379	Other disease	Metabolic	PGS002442	EFO_0000400	diabetes mellitus	diabetes mellitus	EFO_0000400	Digestive system disorder	Gastrointestinal
0.9069	7.41E-173	3.36E-169	PGS002592	EFO_0001379	endocrine system disease|diabetes mellitus	endocrine system disease	EFO_0001379	Other disease	Metabolic	PGS002442	EFO_0000400	diabetes mellitus	diabetes mellitus	EFO_0000400	Metabolic disorder	Metabolic
0.9068	7.84E-173	3.55E-169	PGS003033	EFO_0004611	low density lipoprotein cholesterol measurement	low density lipoprotein cholesterol measurement	EFO_0004611	Lipid or lipoprotein measurement	Metabolic	PGS003138	EFO_0004574	total cholesterol measurement	total cholesterol measurement	EFO_0004574	Lipid or lipoprotein measurement	Metabolic
0.9068	7.84E-173	3.55E-169	PGS003033	EFO_0004611	low density lipoprotein cholesterol measurement	low density lipoprotein cholesterol measurement	EFO_0004611	Cardiovascular measurement	Cardiovascular	PGS003138	EFO_0004574	total cholesterol measurement	total cholesterol measurement	EFO_0004574	Lipid or lipoprotein measurement	Metabolic
0.9067	9.75E-173	4.41E-169	PGS002445	EFO_0001379	endocrine system disease|diabetes mellitus	endocrine system disease	EFO_0001379	Other disease	Metabolic	PGS002491	EFO_0000400	diabetes mellitus	diabetes mellitus	EFO_0000400	Metabolic disorder	Metabolic
0.9067	9.75E-173	4.41E-169	PGS002445	EFO_0001379	endocrine system disease|diabetes mellitus	endocrine system disease	EFO_0001379	Other disease	Metabolic	PGS002491	EFO_0000400	diabetes mellitus	diabetes mellitus	EFO_0000400	Digestive system disorder	Gastrointestinal
0.9066	1.25E-172	5.64E-169	PGS002445	EFO_0001379	endocrine system disease|diabetes mellitus	endocrine system disease	EFO_0001379	Other disease	Metabolic	PGS002573	MONDO_0005148	type 2 diabetes mellitus	type 2 diabetes mellitus	MONDO_0005148	Digestive system disorder	Gastrointestinal
0.9066	1.25E-172	5.64E-169	PGS002445	EFO_0001379	endocrine system disease|diabetes mellitus	endocrine system disease	EFO_0001379	Other disease	Metabolic	PGS002573	MONDO_0005148	type 2 diabetes mellitus	type 2 diabetes mellitus	MONDO_0005148	Metabolic disorder	Metabolic
0.9065	1.51E-172	6.81E-169	PGS001894	EFO_0001060	celiac disease	celiac disease	EFO_0001060	Immune system disorder	Immunological	PGS001856	EFO_0001060	celiac disease	celiac disease	EFO_0001060	Immune system disorder	Immunological
0.9065	1.51E-172	6.81E-169	PGS001894	EFO_0001060	celiac disease	celiac disease	EFO_0001060	Immune system disorder	Immunological	PGS001856	EFO_0001060	celiac disease	celiac disease	EFO_0001060	Digestive system disorder	Gastrointestinal
0.9065	1.51E-172	6.81E-169	PGS001894	EFO_0001060	celiac disease	celiac disease	EFO_0001060	Digestive system disorder	Gastrointestinal	PGS001856	EFO_0001060	celiac disease	celiac disease	EFO_0001060	Digestive system disorder	Gastrointestinal
0.9065	1.51E-172	6.81E-169	PGS001894	EFO_0001060	celiac disease	celiac disease	EFO_0001060	Digestive system disorder	Gastrointestinal	PGS001856	EFO_0001060	celiac disease	celiac disease	EFO_0001060	Immune system disorder	Immunological
0.9065	1.60E-172	7.22E-169	PGS001160	EFO_0005106	body composition measurement	body composition measurement	EFO_0005106	Body measurement	Body measurement	PGS001235	EFO_0004324	body weights and measures	body weights and measures	EFO_0004324	Body measurement	Body measurement
0.9065	1.81E-172	8.15E-169	PGS000508	EFO_0000305	breast carcinoma	breast carcinoma	EFO_0000305	Cancer	Neoplasms	PGS000332	EFO_0000305	breast carcinoma	breast carcinoma	EFO_0000305	Cancer	Neoplasms
0.9063	2.48E-172	1.12E-168	PGS002543	EFO_0001379	endocrine system disease|diabetes mellitus	endocrine system disease	EFO_0001379	Other disease	Metabolic	PGS002452	EFO_0004541	HbA1c measurement	HbA1c measurement	EFO_0004541	Hematological measurement	Hematological
0.9063	2.50E-172	1.13E-168	PGS000423	EFO_0000756	melanoma	melanoma	EFO_0000756	Cancer	Neoplasms	PGS000422	EFO_0000756	melanoma	melanoma	EFO_0000756	Cancer	Neoplasms
0.9063	2.50E-172	1.13E-168	PGS000423	EFO_0000756	melanoma	melanoma	EFO_0000756	Cancer	Neoplasms	PGS000421	EFO_0000756	melanoma	melanoma	EFO_0000756	Cancer	Neoplasms
0.9062	3.38E-172	1.52E-168	PGS002514	EFO_0000676	psoriasis	psoriasis	EFO_0000676	Immune system disorder	Immunological	PGS002465	EFO_0000676	psoriasis	psoriasis	EFO_0000676	Immune system disorder	Immunological
0.9061	3.86E-172	1.73E-168	PGS001979	EFO_0004530	triglyceride measurement	triglyceride measurement	EFO_0004530	Lipid or lipoprotein measurement	Metabolic	PGS002353	EFO_0004530	triglyceride measurement	triglyceride measurement	EFO_0004530	Lipid or lipoprotein measurement	Metabolic
0.906	4.79E-172	2.14E-168	PGS000603	EFO_0005088	testicular carcinoma	testicular carcinoma	EFO_0005088	Cancer	Neoplasms	PGS000601	EFO_0005088	testicular carcinoma	testicular carcinoma	EFO_0005088	Cancer	Neoplasms
0.906	4.79E-172	2.14E-168	PGS000603	EFO_0005088	testicular carcinoma	testicular carcinoma	EFO_0005088	Cancer	Neoplasms	PGS000602	EFO_0005088	testicular carcinoma	testicular carcinoma	EFO_0005088	Cancer	Neoplasms
0.906	5.07E-172	2.27E-168	PGS000201	EFO_0007835	alcohol dependence measurement	alcohol dependence measurement	EFO_0007835	Other measurement	Psychiatric	PGS000202	EFO_0007835	alcohol dependence measurement	alcohol dependence measurement	EFO_0007835	Other measurement	Psychiatric
0.9059	6.98E-172	3.12E-168	PGS001942	EFO_0004570	bilirubin measurement	bilirubin measurement	EFO_0004570	Other measurement	Metabolic	PGS001824	Orphanet_309816	Disorder of bilirubin metabolism and excretion	excretion	Orphanet_309816	Metabolic disorder	Metabolic
0.9057	9.55E-172	4.26E-168	PGS002160	EFO_0004570	bilirubin measurement	bilirubin measurement	EFO_0004570	Other measurement	Metabolic	PGS002032	Orphanet_309816	Disorder of bilirubin metabolism and excretion	excretion	Orphanet_309816	Metabolic disorder	Metabolic
0.9057	1.01E-171	4.48E-168	PGS001793	EFO_0000712	stroke	stroke	EFO_0000712	Cardiovascular disease	Cardiovascular	PGS001798	EFO_0000712	stroke	stroke	EFO_0000712	Neurological disorder	Neurological
0.9057	1.01E-171	4.48E-168	PGS001793	EFO_0000712	stroke	stroke	EFO_0000712	Cardiovascular disease	Cardiovascular	PGS001798	EFO_0000712	stroke	stroke	EFO_0000712	Cardiovascular disease	Cardiovascular
0.9057	1.01E-171	4.48E-168	PGS001793	EFO_0000712	stroke	stroke	EFO_0000712	Neurological disorder	Neurological	PGS001798	EFO_0000712	stroke	stroke	EFO_0000712	Cardiovascular disease	Cardiovascular
0.9057	1.01E-171	4.48E-168	PGS001793	EFO_0000712	stroke	stroke	EFO_0000712	Neurological disorder	Neurological	PGS001798	EFO_0000712	stroke	stroke	EFO_0000712	Neurological disorder	Neurological
0.9057	1.18E-171	5.26E-168	PGS001926	EFO_0004509	hemoglobin measurement	hemoglobin measurement	EFO_0004509	Hematological measurement	Hematological	PGS002141	EFO_0004348	hematocrit	hematocrit	EFO_0004348	Hematological measurement	Hematological
0.9056	1.49E-171	6.63E-168	PGS003139	EFO_0004574	total cholesterol measurement	total cholesterol measurement	EFO_0004574	Lipid or lipoprotein measurement	Metabolic	PGS003029	EFO_0004611	low density lipoprotein cholesterol measurement	measurement	EFO_0004611	Cardiovascular measurement	Cardiovascular
0.9056	1.49E-171	6.63E-168	PGS003139	EFO_0004574	total cholesterol measurement	total cholesterol measurement	EFO_0004574	Lipid or lipoprotein measurement	Metabolic	PGS003029	EFO_0004611	low density lipoprotein cholesterol measurement	measurement	EFO_0004611	Lipid or lipoprotein measurement	Metabolic
0.9055	1.58E-171	7.01E-168	PGS001813	MONDO_0000632	uterine benign neoplasm	uterine benign neoplasm	MONDO_0000632	Cancer	Neoplasms	PGS002021	MONDO_0000632	uterine benign neoplasm	uterine benign neoplasm	MONDO_0000632	Cancer	Neoplasms
0.9055	1.59E-171	7.05E-168	PGS003313	EFO_0004617	cystatin C measurement|glomerular filtration rate	cystatin C measurement	EFO_0004617	Other measurement	Metabolic	PGS003312	EFO_0004617	cystatin C measurement|glomerular filtration rate	cystatin C measurement	EFO_0004617	Other measurement	Metabolic
0.9055	1.83E-171	8.10E-168	PGS001799	EFO_0002892	thyroid carcinoma	thyroid carcinoma	EFO_0002892	Cancer	Neoplasms	PGS001794	EFO_0002892	thyroid carcinoma	thyroid carcinoma	EFO_0002892	Cancer	Neoplasms
0.9054	2.08E-171	9.22E-168	PGS003143	EFO_0004574	total cholesterol measurement	total cholesterol measurement	EFO_0004574	Lipid or lipoprotein measurement	Metabolic	PGS003038	EFO_0004611	low density lipoprotein cholesterol measurement	measurement	EFO_0004611	Lipid or lipoprotein measurement	Metabolic
0.9054	2.08E-171	9.22E-168	PGS003143	EFO_0004574	total cholesterol measurement	total cholesterol measurement	EFO_0004574	Lipid or lipoprotein measurement	Metabolic	PGS003038	EFO_0004611	low density lipoprotein cholesterol measurement	measurement	EFO_0004611	Cardiovascular measurement	Cardiovascular
0.9054	2.27E-171	1.01E-167	PGS003139	EFO_0004574	total cholesterol measurement	total cholesterol measurement	EFO_0004574	Lipid or lipoprotein measurement	Metabolic	PGS003034	EFO_0004611	low density lipoprotein cholesterol measurement	measurement	EFO_0004611	Lipid or lipoprotein measurement	Metabolic
0.9054	2.27E-171	1.01E-167	PGS003139	EFO_0004574	total cholesterol measurement	total cholesterol measurement	EFO_0004574	Lipid or lipoprotein measurement	Metabolic	PGS003034	EFO_0004611	low density lipoprotein cholesterol measurement	measurement	EFO_0004611	Cardiovascular measurement	Cardiovascular
0.9053	2.55E-171	1.13E-167	PGS001734	EFO_0006930	brain volume measurement|neuroimaging measurement	brain volume measurement	EFO_0006930	Other measurement	Neurological	PGS001720	EFO_0006930	measurement	brain volume measurement	EFO_0006930	Other measurement	Neurological
0.9052	3.10E-171	1.37E-167	PGS003297	EFO_0004617	cystatin C measurement|glomerular filtration rate	cystatin C measurement	EFO_0004617	Other measurement	Metabolic	PGS003313	EFO_0004617	cystatin C measurement|glomerular filtration rate	cystatin C measurement	EFO_0004617	Other measurement	Metabolic
0.9052	3.25E-171	1.44E-167	PGS001753	EFO_0006930	brain volume measurement|neuroimaging measurement	brain volume measurement	EFO_0006930	Other measurement	Neurological	PGS001738	EFO_0006930	measurement	brain volume measurement	EFO_0006930	Other measurement	Neurological
0.9051	3.98E-171	1.76E-167	PGS001076	EFO_0007991	eosinophil percentage of leukocytes	eosinophil percentage of leukocytes	EFO_0007991	Hematological measurement	Hematological	PGS001172	EFO_0004842	eosinophil count	eosinophil count	EFO_0004842	Hematological measurement	Hematological
0.905	5.89E-171	2.60E-167	PGS002403	EFO_0004541	HbA1c measurement	HbA1c measurement	EFO_0004541	Hematological measurement	Hematological	PGS002400	EFO_0004468	glucose measurement	glucose measurement	EFO_0004468	Other measurement	Metabolic
0.9049	6.74E-171	2.97E-167	PGS001145	EFO_0005409	fat body mass	fat body mass	EFO_0005409	Other measurement	Body measurement	PGS001228	EFO_0004340	body mass index	body mass index	EFO_0004340	Body measurement	Body measurement
0.9049	7.33E-171	3.23E-167	PGS000077	EFO_0004289	lymphoid leukemia	lymphoid leukemia	EFO_0004289	Immune system disorder	Immunological	PGS000788	EFO_0004289	lymphoid leukemia	lymphoid leukemia	EFO_0004289	Immune system disorder	Immunological
0.9049	7.33E-171	3.23E-167	PGS000077	EFO_0004289	lymphoid leukemia	lymphoid leukemia	EFO_0004289	Cancer	Neoplasms	PGS000788	EFO_0004289	lymphoid leukemia	lymphoid leukemia	EFO_0004289	Immune system disorder	Immunological
0.9049	7.33E-171	3.23E-167	PGS000077	EFO_0004289	lymphoid leukemia	lymphoid leukemia	EFO_0004289	Immune system disorder	Immunological	PGS000788	EFO_0004289	lymphoid leukemia	lymphoid leukemia	EFO_0004289	Cancer	Neoplasms
0.9049	7.33E-171	3.23E-167	PGS000077	EFO_0004289	lymphoid leukemia	lymphoid leukemia	EFO_0004289	Cancer	Neoplasms	PGS000788	EFO_0004289	lymphoid leukemia	lymphoid leukemia	EFO_0004289	Cancer	Neoplasms
0.9048	7.87E-171	3.46E-167	PGS001168	EFO_0004995	lean body mass	lean body mass	EFO_0004995	Other measurement	Skeletal	PGS001146	EFO_0005409	fat body mass	fat body mass	EFO_0005409	Other measurement	Body measurement
0.9048	8.58E-171	3.77E-167	PGS002501	EFO_0004541	HbA1c measurement	HbA1c measurement	EFO_0004541	Hematological measurement	Hematological	PGS002452	EFO_0004541	HbA1c measurement	HbA1c measurement	EFO_0004541	Hematological measurement	Hematological
0.9047	1.02E-170	4.47E-167	PGS001855	EFO_0000729	ulcerative colitis	ulcerative colitis	EFO_0000729	Immune system disorder	Immunological	PGS002066	EFO_0000729	ulcerative colitis	ulcerative colitis	EFO_0000729	Digestive system disorder	Gastrointestinal
0.9047	1.02E-170	4.47E-167	PGS001855	EFO_0000729	ulcerative colitis	ulcerative colitis	EFO_0000729	Immune system disorder	Immunological	PGS002066	EFO_0000729	ulcerative colitis	ulcerative colitis	EFO_0000729	Immune system disorder	Immunological
0.9047	1.02E-170	4.47E-167	PGS001855	EFO_0000729	ulcerative colitis	ulcerative colitis	EFO_0000729	Digestive system disorder	Gastrointestinal	PGS002066	EFO_0000729	ulcerative colitis	ulcerative colitis	EFO_0000729	Digestive system disorder	Gastrointestinal
0.9047	1.02E-170	4.47E-167	PGS001855	EFO_0000729	ulcerative colitis	ulcerative colitis	EFO_0000729	Digestive system disorder	Gastrointestinal	PGS002066	EFO_0000729	ulcerative colitis	ulcerative colitis	EFO_0000729	Immune system disorder	Immunological
0.9047	1.04E-170	4.56E-167	PGS000797	EFO_0002892	thyroid carcinoma	thyroid carcinoma	EFO_0002892	Cancer	Neoplasms	PGS000087	EFO_0002892	thyroid carcinoma	thyroid carcinoma	EFO_0002892	Cancer	Neoplasms
0.9046	1.33E-170	5.82E-167	PGS002851	EFO_0004340	body mass index	body mass index	EFO_0004340	Body measurement	Body measurement	PGS002856	EFO_0004340	body mass index	body mass index	EFO_0004340	Body measurement	Body measurement
0.9046	1.46E-170	6.39E-167	PGS002150	EFO_0004611	low density lipoprotein cholesterol measurement	low density lipoprotein cholesterol measurement	EFO_0004611	Lipid or lipoprotein measurement	Metabolic	PGS002337	EFO_0004611	low density lipoprotein cholesterol measurement	measurement	EFO_0004611	Cardiovascular measurement	Cardiovascular

0.9046	1.46E-170	6.39E-167	PGS002150	EFO_0004611	low density lipoprotein cholesterol measurement	low density lipoprotein cholesterol measurement	EFO_0004611	Cardiovascular measurement	Cardiovascular	PGS002337	EFO_0004611	low density lipoprotein cholesterol measurement	measurement	EFO_0004611	Lipid or lipoprotein measurement	Metabolic
0.9046	1.46E-170	6.39E-167	PGS002150	EFO_0004611	low density lipoprotein cholesterol measurement	low density lipoprotein cholesterol measurement	EFO_0004611	Cardiovascular measurement	Cardiovascular	PGS002337	EFO_0004611	low density lipoprotein cholesterol measurement	measurement	EFO_0004611	Cardiovascular measurement	Cardiovascular
0.9046	1.46E-170	6.39E-167	PGS002150	EFO_0004611	low density lipoprotein cholesterol measurement	low density lipoprotein cholesterol measurement	EFO_0004611	Lipid or lipoprotein measurement	Metabolic	PGS002337	EFO_0004611	low density lipoprotein cholesterol measurement	measurement	EFO_0004611	Lipid or lipoprotein measurement	Metabolic
0.9045	1.74E-170	7.60E-167	PGS001093	EFO_0007822	hair colour measurement	hair colour measurement	EFO_0007822	Other measurement	Dermatological	PGS001096	EFO_0007822	hair colour measurement	hair colour measurement	EFO_0007822	Other measurement	Dermatological
0.9044	1.95E-170	8.54E-167	PGS001168	EFO_0004995	lean body mass	lean body mass	EFO_0004995	Other measurement	Skeletal	PGS001235	EFO_0004324	body weights and measures	body weights and measures	EFO_0004324	Body measurement	Body measurement
0.9044	2.04E-170	8.92E-167	PGS001736	EFO_0006930	brain volume measurement|neuroimaging measurement	brain volume measurement	EFO_0006930	Other measurement	Neurological	PGS001722	EFO_0006930	measurement	brain volume measurement	EFO_0006930	Other measurement	Neurological
0.9043	2.58E-170	1.12E-166	PGS003029	EFO_0004611	low density lipoprotein cholesterol measurement	low density lipoprotein cholesterol measurement	EFO_0004611	Cardiovascular measurement	Cardiovascular	PGS003134	EFO_0004574	total cholesterol measurement	total cholesterol measurement	EFO_0004574	Lipid or lipoprotein measurement	Metabolic
0.9043	2.58E-170	1.12E-166	PGS003029	EFO_0004611	low density lipoprotein cholesterol measurement	low density lipoprotein cholesterol measurement	EFO_0004611	Lipid or lipoprotein measurement	Metabolic	PGS003134	EFO_0004574	total cholesterol measurement	total cholesterol measurement	EFO_0004574	Lipid or lipoprotein measurement	Metabolic
0.9043	2.80E-170	1.22E-166	PGS000510	EFO_0000305	breast carcinoma	breast carcinoma	EFO_0000305	Cancer	Neoplasms	PGS000509	EFO_0000305	breast carcinoma	breast carcinoma	EFO_0000305	Cancer	Neoplasms
0.904	4.73E-170	2.06E-166	PGS002426	MONDO_0005148	type 2 diabetes mellitus	type 2 diabetes mellitus	MONDO_0005148	Metabolic disorder	Metabolic	PGS002403	EFO_0004541	HbA1c measurement	HbA1c measurement	EFO_0004541	Hematological measurement	Hematological
0.904	4.73E-170	2.06E-166	PGS002426	MONDO_0005148	type 2 diabetes mellitus	type 2 diabetes mellitus	MONDO_0005148	Digestive system disorder	Gastrointestinal	PGS002403	EFO_0004541	HbA1c measurement	HbA1c measurement	EFO_0004541	Hematological measurement	Hematological
0.9039	6.19E-170	2.69E-166	PGS002103	EFO_0008385	visual acuity measurement	visual acuity measurement	EFO_0008385	Other measurement	Ophthalmological	PGS001890	EFO_0008385	visual acuity measurement	visual acuity measurement	EFO_0008385	Other measurement	Ophthalmological
0.9039	6.31E-170	2.74E-166	PGS000391	MONDO_0001672	bronchus cancer|lung cancer|tracheal cancer	bronchus cancer	MONDO_0001672	Cancer	Neoplasms	PGS000392	MONDO_0001672	bronchus cancer|lung cancer|tracheal cancer	bronchus cancer	MONDO_0001672	Cancer	Neoplasms
0.9038	7.45E-170	3.24E-166	PGS001529	EFO_0007772	calcaneal bone quantitative ultrasound measurement	calcaneal bone quantitative ultrasound measurement	EFO_0007772	Other measurement	Skeletal	PGS000951	EFO_0004514	bone quantitative ultrasound measurement	measurement	EFO_0004514	Other measurement	Skeletal
0.9037	1.01E-169	4.40E-166	PGS002865	EFO_0004458	C-reactive protein measurement	C-reactive protein measurement	EFO_0004458	Inflammatory measurement	Immunological	PGS002866	EFO_0004458	C-reactive protein measurement	C-reactive protein measurement	EFO_0004458	Inflammatory measurement	Immunological
0.9036	1.17E-169	5.09E-166	PGS001103	EFO_0007800	body fat percentage	body fat percentage	EFO_0007800	Body measurement	Body measurement	PGS001101	EFO_0007800	body fat percentage	body fat percentage	EFO_0007800	Body measurement	Body measurement
0.9035	1.62E-169	7.00E-166	PGS003241	EFO_0010472	creatine measurement	creatine measurement	EFO_0010472	Other measurement	Metabolic	PGS003240	EFO_0010472	creatine measurement	creatine measurement	EFO_0010472	Other measurement	Metabolic
0.9031	3.44E-169	1.49E-165	PGS003070	EFO_0006335	systolic blood pressure	systolic blood pressure	EFO_0006335	Other measurement	Cardiovascular	PGS003075	EFO_0006335	systolic blood pressure	systolic blood pressure	EFO_0006335	Other measurement	Cardiovascular
0.903	4.65E-169	2.01E-165	PGS001742	EFO_0006930	brain volume measurement|neuroimaging measurement	brain volume measurement	EFO_0006930	Other measurement	Neurological	PGS001727	EFO_0006930	measurement	brain volume measurement	EFO_0006930	Other measurement	Neurological
0.903	5.29E-169	2.29E-165	PGS002613	EFO_0004305	erythrocyte count	erythrocyte count	EFO_0004305	Hematological measurement	Hematological	PGS002417	EFO_0004305	erythrocyte count	erythrocyte count	EFO_0004305	Hematological measurement	Hematological
0.9029	5.42E-169	2.34E-165	PGS001264	EFO_0003907	deep vein thrombosis	deep vein thrombosis	EFO_0003907	Other trait	Cardiovascular	PGS000931	MONDO_0000831	thrombotic disease|deep vein thrombosis	thrombotic disease	MONDO_0000831	Cardiovascular disease	Cardiovascular
0.9029	5.95E-169	2.56E-165	PGS000542	MONDO_0002715	uterine cancer	uterine cancer	MONDO_0002715	Cancer	Neoplasms	PGS002735	EFO_1001512	endometrial carcinoma	endometrial carcinoma	EFO_1001512	Cancer	Neoplasms
0.9029	6.05E-169	2.61E-165	PGS002970	EFO_0004339	body height	body height	EFO_0004339	Body measurement	Body measurement	PGS002966	EFO_0004339	body height	body height	EFO_0004339	Body measurement	Body measurement
0.9029	6.60E-169	2.84E-165	PGS001848	HP_0100582	Nasal polyposis	Nasal polyposis	HP_0100582	Other trait	Ear, Nose, Throat	PGS002060	HP_0100582	Nasal polyposis	Nasal polyposis	HP_0100582	Other trait	Ear, Nose, Throat
0.9028	7.75E-169	3.33E-165	PGS002460	EFO_0004527	mean corpuscular hemoglobin	mean corpuscular hemoglobin	EFO_0004527	Hematological measurement	Hematological	PGS002411	EFO_0004527	mean corpuscular hemoglobin	mean corpuscular hemoglobin	EFO_0004527	Hematological measurement	Hematological
0.9027	9.33E-169	4.01E-165	PGS001484	EFO_0006930	brain volume measurement|neuroimaging measurement	brain volume measurement	EFO_0006930	Other measurement	Neurological	PGS001485	EFO_0006930	measurement	brain volume measurement	EFO_0006930	Other measurement	Neurological
0.9026	1.08E-168	4.64E-165	PGS002339	EFO_0004527	mean corpuscular hemoglobin	mean corpuscular hemoglobin	EFO_0004527	Hematological measurement	Hematological	PGS001990	EFO_0004526	mean corpuscular volume	mean corpuscular volume	EFO_0004526	Hematological measurement	Hematological
0.9026	1.12E-168	4.79E-165	PGS000014	MONDO_0005148	type 2 diabetes mellitus	type 2 diabetes mellitus	MONDO_0005148	Digestive system disorder	Gastrointestinal	PGS001781	MONDO_0005148	type 2 diabetes mellitus	type 2 diabetes mellitus	MONDO_0005148	Digestive system disorder	Gastrointestinal
0.9026	1.12E-168	4.79E-165	PGS000014	MONDO_0005148	type 2 diabetes mellitus	type 2 diabetes mellitus	MONDO_0005148	Digestive system disorder	Gastrointestinal	PGS001781	MONDO_0005148	type 2 diabetes mellitus	type 2 diabetes mellitus	MONDO_0005148	Metabolic disorder	Metabolic
0.9026	1.12E-168	4.79E-165	PGS000014	MONDO_0005148	type 2 diabetes mellitus	type 2 diabetes mellitus	MONDO_0005148	Metabolic disorder	Metabolic	PGS001781	MONDO_0005148	type 2 diabetes mellitus	type 2 diabetes mellitus	MONDO_0005148	Digestive system disorder	Gastrointestinal
0.9026	1.12E-168	4.79E-165	PGS000014	MONDO_0005148	type 2 diabetes mellitus	type 2 diabetes mellitus	MONDO_0005148	Metabolic disorder	Metabolic	PGS001781	MONDO_0005148	type 2 diabetes mellitus	type 2 diabetes mellitus	MONDO_0005148	Metabolic disorder	Metabolic
0.9026	1.13E-168	4.83E-165	PGS003108	MONDO_0005148	type 2 diabetes mellitus	type 2 diabetes mellitus	MONDO_0005148	Metabolic disorder	Metabolic	PGS002243	MONDO_0005148	type 2 diabetes mellitus	type 2 diabetes mellitus	MONDO_0005148	Digestive system disorder	Gastrointestinal
0.9026	1.13E-168	4.83E-165	PGS003108	MONDO_0005148	type 2 diabetes mellitus	type 2 diabetes mellitus	MONDO_0005148	Digestive system disorder	Gastrointestinal	PGS002243	MONDO_0005148	type 2 diabetes mellitus	type 2 diabetes mellitus	MONDO_0005148	Metabolic disorder	Metabolic
0.9026	1.13E-168	4.83E-165	PGS003108	MONDO_0005148	type 2 diabetes mellitus	type 2 diabetes mellitus	MONDO_0005148	Digestive system disorder	Gastrointestinal	PGS002243	MONDO_0005148	type 2 diabetes mellitus	type 2 diabetes mellitus	MONDO_0005148	Digestive system disorder	Gastrointestinal
0.9026	1.13E-168	4.83E-165	PGS003108	MONDO_0005148	type 2 diabetes mellitus	type 2 diabetes mellitus	MONDO_0005148	Metabolic disorder	Metabolic	PGS002243	MONDO_0005148	type 2 diabetes mellitus	type 2 diabetes mellitus	MONDO_0005148	Metabolic disorder	Metabolic
0.9026	1.20E-168	5.16E-165	PGS002393	EFO_0000400	diabetes mellitus	diabetes mellitus	EFO_0000400	Metabolic disorder	Metabolic	PGS002403	EFO_0004541	HbA1c measurement	HbA1c measurement	EFO_0004541	Hematological measurement	Hematological
0.9026	1.20E-168	5.16E-165	PGS002393	EFO_0000400	diabetes mellitus	diabetes mellitus	EFO_0000400	Digestive system disorder	Gastrointestinal	PGS002403	EFO_0004541	HbA1c measurement	HbA1c measurement	EFO_0004541	Hematological measurement	Hematological
0.9025	1.38E-168	5.89E-165	PGS001781	MONDO_0005148	type 2 diabetes mellitus	type 2 diabetes mellitus	MONDO_0005148	Metabolic disorder	Metabolic	PGS002243	MONDO_0005148	type 2 diabetes mellitus	type 2 diabetes mellitus	MONDO_0005148	Metabolic disorder	Metabolic
0.9025	1.38E-168	5.89E-165	PGS001781	MONDO_0005148	type 2 diabetes mellitus	type 2 diabetes mellitus	MONDO_0005148	Digestive system disorder	Gastrointestinal	PGS002243	MONDO_0005148	type 2 diabetes mellitus	type 2 diabetes mellitus	MONDO_0005148	Digestive system disorder	Gastrointestinal
0.9025	1.38E-168	5.89E-165	PGS001781	MONDO_0005148	type 2 diabetes mellitus	type 2 diabetes mellitus	MONDO_0005148	Digestive system disorder	Gastrointestinal	PGS002243	MONDO_0005148	type 2 diabetes mellitus	type 2 diabetes mellitus	MONDO_0005148	Metabolic disorder	Metabolic
0.9025	1.38E-168	5.89E-165	PGS001781	MONDO_0005148	type 2 diabetes mellitus	type 2 diabetes mellitus	MONDO_0005148	Metabolic disorder	Metabolic	PGS002243	MONDO_0005148	type 2 diabetes mellitus	type 2 diabetes mellitus	MONDO_0005148	Digestive system disorder	Gastrointestinal
0.9024	1.70E-168	7.27E-165	PGS002345	EFO_0004305	erythrocyte count	erythrocyte count	EFO_0004305	Hematological measurement	Hematological	PGS001909	EFO_0004305	erythrocyte count	erythrocyte count	EFO_0004305	Hematological measurement	Hematological
0.9024	1.72E-168	7.35E-165	PGS002622	MONDO_0005148	type 2 diabetes mellitus	type 2 diabetes mellitus	MONDO_0005148	Digestive system disorder	Gastrointestinal	PGS002442	EFO_0000400	diabetes mellitus	diabetes mellitus	EFO_0000400	Digestive system disorder	Gastrointestinal
0.9024	1.72E-168	7.35E-165	PGS002622	MONDO_0005148	type 2 diabetes mellitus	type 2 diabetes mellitus	MONDO_0005148	Digestive system disorder	Gastrointestinal	PGS002442	EFO_0000400	diabetes mellitus	diabetes mellitus	EFO_0000400	Metabolic disorder	Metabolic
0.9024	1.72E-168	7.35E-165	PGS002622	MONDO_0005148	type 2 diabetes mellitus	type 2 diabetes mellitus	MONDO_0005148	Metabolic disorder	Metabolic	PGS002442	EFO_0000400	diabetes mellitus	diabetes mellitus	EFO_0000400	Digestive system disorder	Gastrointestinal
0.9024	1.72E-168	7.35E-165	PGS002622	MONDO_0005148	type 2 diabetes mellitus	type 2 diabetes mellitus	MONDO_0005148	Metabolic disorder	Metabolic	PGS002442	EFO_0000400	diabetes mellitus	diabetes mellitus	EFO_0000400	Metabolic disorder	Metabolic
0.9023	2.35E-168	1.00E-164	PGS001894	EFO_0001060	celiac disease	celiac disease	EFO_0001060	Digestive system disorder	Gastrointestinal	PGS000040	EFO_0001060	celiac disease	celiac disease	EFO_0001060	Digestive system disorder	Gastrointestinal
0.9023	2.35E-168	1.00E-164	PGS001894	EFO_0001060	celiac disease	celiac disease	EFO_0001060	Immune system disorder	Immunological	PGS000040	EFO_0001060	celiac disease	celiac disease	EFO_0001060	Digestive system disorder	Gastrointestinal
0.9023	2.35E-168	1.00E-164	PGS001894	EFO_0001060	celiac disease	celiac disease	EFO_0001060	Digestive system disorder	Gastrointestinal	PGS000040	EFO_0001060	celiac disease	celiac disease	EFO_0001060	Immune system disorder	Immunological
0.9023	2.35E-168	1.00E-164	PGS001894	EFO_0001060	celiac disease	celiac disease	EFO_0001060	Immune system disorder	Immunological	PGS000040	EFO_0001060	celiac disease	celiac disease	EFO_0001060	Immune system disorder	Immunological
0.9021	3.33E-168	1.42E-164	PGS001671	EFO_0006930	brain volume measurement|neuroimaging measurement	brain volume measurement	EFO_0006930	Other measurement	Neurological	PGS001672	EFO_0006930	measurement	brain volume measurement	EFO_0006930	Other measurement	Neurological
0.902	4.50E-168	1.92E-164	PGS002628	MONDO_0004979	asthma	asthma	MONDO_0004979	Other disease	Respiratory	PGS002664	EFO_0000684	respiratory system disease	respiratory system disease	EFO_0000684	Other disease	Skeletal
0.902	4.66E-168	1.98E-164	PGS001973	EFO_0004309	platelet count	platelet count	EFO_0004309	Hematological measurement	Hematological	PGS002343	EFO_0004309	platelet count	platelet count	EFO_0004309	Hematological measurement	Hematological
-0.9019	5.20E-168	2.21E-164	PGS001095	EFO_0007822	hair colour measurement	hair colour measurement	EFO_0007822	Other measurement	Dermatological	PGS001096	EFO_0007822	hair colour measurement	hair colour measurement	EFO_0007822	Other measurement	Dermatological
0.9018	7.37E-168	3.13E-164	PGS002721	EFO_0004631	vitamin D measurement	vitamin D measurement	EFO_0004631	Lipid or lipoprotein measurement	Metabolic	PGS003193	EFO_0004631	vitamin D measurement	vitamin D measurement	EFO_0004631	Lipid or lipoprotein measurement	Metabolic
0.9017	8.08E-168	3.43E-164	PGS003134	EFO_0004574	total cholesterol measurement	total cholesterol measurement	EFO_0004574	Lipid or lipoprotein measurement	Metabolic	PGS003034	EFO_0004611	low density lipoprotein cholesterol measurement	measurement	EFO_0004611	Lipid or lipoprotein measurement	Metabolic
0.9017	8.08E-168	3.43E-164	PGS003134	EFO_0004574	total cholesterol measurement	total cholesterol measurement	EFO_0004574	Lipid or lipoprotein measurement	Metabolic	PGS003034	EFO_0004611	low density lipoprotein cholesterol measurement	measurement	EFO_0004611	Cardiovascular measurement	Cardiovascular
0.9017	8.94E-168	3.79E-164	PGS001301	EFO_0001060	celiac disease	celiac disease	EFO_0001060	Digestive system disorder	Gastrointestinal	PGS001856	EFO_0001060	celiac disease	celiac disease	EFO_0001060	Digestive system disorder	Gastrointestinal
0.9017	8.94E-168	3.79E-164	PGS001301	EFO_0001060	celiac disease	celiac disease	EFO_0001060	Immune system disorder	Immunological	PGS001856	EFO_0001060	celiac disease	celiac disease	EFO_0001060	Immune system disorder	Immunological
0.9017	8.94E-168	3.79E-164	PGS001301	EFO_0001060	celiac disease	celiac disease	EFO_0001060	Immune system disorder	Immunological	PGS001856	EFO_0001060	celiac disease	celiac disease	EFO_0001060	Digestive system disorder	Gastrointestinal
0.9017	8.94E-168	3.79E-164	PGS001301	EFO_0001060	celiac disease	celiac disease	EFO_0001060	Digestive system disorder	Gastrointestinal	PGS001856	EFO_0001060	celiac disease	celiac disease	EFO_0001060	Immune system disorder	Immunological
0.9016	9.74E-168	4.12E-164	PGS001716	EFO_0006930	brain volume measurement|neuroimaging measurement	brain volume measurement	EFO_0006930	Other measurement	Neurological	PGS001731	EFO_0006930	measurement	brain volume measurement	EFO_0006930	Other measurement	Neurological
0.9016	1.07E-167	4.53E-164	PGS002433	EFO_0005140	autoimmune disease	autoimmune disease	EFO_0005140	Immune system disorder	Immunological	PGS002482	EFO_0005140	autoimmune disease	autoimmune disease	EFO_0005140	Immune system disorder	Immunological
0.9016	1.10E-167	4.63E-164	PGS000888	EFO_0004611	low density lipoprotein cholesterol measurement	low density lipoprotein cholesterol measurement	EFO_0004611	Lipid or lipoprotein measurement	Metabolic	PGS002782	EFO_0005689	measurement	measurement	EFO_0005689	Lipid or lipoprotein measurement	Metabolic
0.9016	1.10E-167	4.63E-164	PGS000888	EFO_0004611	low density lipoprotein cholesterol measurement	low density lipoprotein cholesterol measurement	EFO_0004611	Lipid or lipoprotein measurement	Metabolic	PGS002782	EFO_0005689	measurement	measurement	EFO_0005689	Cardiovascular measurement	Cardiovascular
0.9016	1.10E-167	4.63E-164	PGS000888	EFO_0004611	low density lipoprotein cholesterol measurement	low density lipoprotein cholesterol measurement	EFO_0004611	Cardiovascular measurement	Cardiovascular	PGS002782	EFO_0005689	measurement	measurement	EFO_0005689	Lipid or lipoprotein measurement	Metabolic
0.9016	1.10E-167	4.63E-164	PGS000888	EFO_0004611	low density lipoprotein cholesterol measurement	low density lipoprotein cholesterol measurement	EFO_0004611	Cardiovascular measurement	Cardiovascular	PGS002782	EFO_0005689	measurement	measurement	EFO_0005689	Cardiovascular measurement	Cardiovascular
0.9015	1.35E-167	5.69E-164	PGS003038	EFO_0004611	low density lipoprotein cholesterol measurement	low density lipoprotein cholesterol measurement	EFO_0004611	Lipid or lipoprotein measurement	Metabolic	PGS003138	EFO_0004574	total cholesterol measurement	total cholesterol measurement	EFO_0004574	Lipid or lipoprotein measurement	Metabolic
0.9015	1.35E-167	5.69E-164	PGS003038	EFO_0004611	low density lipoprotein cholesterol measurement	low density lipoprotein cholesterol measurement	EFO_0004611	Cardiovascular measurement	Cardiovascular	PGS003138	EFO_0004574	total cholesterol measurement	total cholesterol measurement	EFO_0004574	Lipid or lipoprotein measurement	Metabolic
0.9014	1.51E-167	6.37E-164	PGS000586	EFO_0001663	prostate carcinoma	prostate carcinoma	EFO_0001663	Cancer	Neoplasms	PGS000582	EFO_0001663	prostate carcinoma	prostate carcinoma	EFO_0001663	Cancer	Neoplasms
0.9014	1.51E-167	6.37E-164	PGS000586	EFO_0001663	prostate carcinoma	prostate carcinoma	EFO_0001663	Cancer	Neoplasms	PGS000581	EFO_0001663	prostate carcinoma	prostate carcinoma	EFO_0001663	Cancer	Neoplasms
0.9012	2.68E-167	1.13E-163	PGS001613	EFO_0005420	measurement	grey matter volume measurement	EFO_0005420	Other measurement	Neurological	PGS001612	EFO_0005420	measurement	grey matter volume measurement	EFO_0005420	Other measurement	Neurological
0.9012	2.72E-167	1.15E-163	PGS002251	EFO_0004340	body mass index	body mass index	EFO_0004340	Body measurement	Body measurement	PGS000034	EFO_0004340	body mass index	body mass index	EFO_0004340	Body measurement	Body measurement
0.901	3.54E-167	1.49E-163	PGS003196	EFO_0004631	vitamin D measurement	vitamin D measurement	EFO_0004631	Lipid or lipoprotein measurement	Metabolic	PGS002574	EFO_0004631	vitamin D measurement	vitamin D measurement	EFO_0004631	Lipid or lipoprotein measurement	Metabolic
0.901	3.58E-167	1.50E-163	PGS000479	EFO_0000305	breast carcinoma	breast carcinoma	EFO_0000305	Cancer	Neoplasms	PGS000483	EFO_0000305	breast carcinoma	breast carcinoma	EFO_0000305	Cancer	Neoplasms
0.9008	6.47E-167	2.72E-163	PGS000332	EFO_0000305	breast carcinoma	breast carcinoma	EFO_0000305	Cancer	Neoplasms	PGS000335	EFO_0000305	breast carcinoma	breast carcinoma	EFO_0000305	Cancer	Neoplasms
0.9007	8.10E-167	3.40E-163	PGS003147	EFO_0004530	triglyceride measurement	triglyceride measurement	EFO_0004530	Lipid or lipoprotein measurement	Metabolic	PGS003152	EFO_0004530	triglyceride measurement	triglyceride measurement	EFO_0004530	Lipid or lipoprotein measurement	Metabolic
0.9005	1.06E-166	4.46E-163	PGS002386	EFO_0007825	balding measurement	balding measurement	EFO_0007825	Other measurement	Activities	PGS002435	EFO_0007825	balding measurement	balding measurement	EFO_0007825	Other measurement	Activities
-0.9004	1.53E-166	6.39E-163	PGS001357	MONDO_0005148	type 2 diabetes mellitus	type 2 diabetes mellitus	MONDO_0005148	Metabolic disorder	Metabolic	PGS002243	MONDO_0005148	type 2 diabetes mellitus	type 2 diabetes mellitus	MONDO_0005148	Metabolic disorder	Metabolic
-0.9004	1.53E-166	6.39E-163	PGS001357	MONDO_0005148	type 2 diabetes mellitus	type 2 diabetes mellitus	MONDO_0005148	Digestive system disorder	Gastrointestinal	PGS002243	MONDO_0005148	type 2 diabetes mellitus	type 2 diabetes mellitus	MONDO_0005148	Digestive system disorder	Gastrointestinal
-0.9004	1.53E-166	6.39E-163	PGS001357	MONDO_0005148	type 2 diabetes mellitus	type 2 diabetes mellitus	MONDO_0005148	Digestive system disorder	Gastrointestinal	PGS002243	MONDO_0005148	type 2 diabetes mellitus	type 2 diabetes mellitus	MONDO_0005148	Metabolic disorder	Metabolic
-0.9004	1.53E-166	6.39E-163	PGS001357	MONDO_0005148	type 2 diabetes mellitus	type 2 diabetes mellitus	MONDO_0005148	Metabolic disorder	Metabolic	PGS002243	MONDO_0005148	type 2 diabetes mellitus	type 2 diabetes mellitus	MONDO_0005148	Digestive system disorder	Gastrointestinal
0.9003	1.65E-166	6.91E-163	PGS002142	EFO_0004509	hemoglobin measurement	hemoglobin measurement	EFO_0004509	Hematological measurement	Hematological	PGS001925	EFO_0004348	hematocrit	hematocrit	EFO_0004348	Hematological measurement	Hematological
0.9002	2.31E-166	9.67E-163	PGS003312	EFO_0004617	cystatin C measurement|glomerular filtration rate	cystatin C measurement	EFO_0004617	Other measurement	Metabolic	PGS003298	EFO_0004617	cystatin C measurement|glomerular filtration rate	cystatin C measurement	EFO_0004617	Other measurement	Metabolic
0.9001	2.52E-166	1.05E-162	PGS003297	EFO_0004617	cystatin C measurement|glomerular filtration rate	cystatin C measurement	EFO_0004617	Other measurement	Metabolic	PGS003298	EFO_0004617	cystatin C measurement|glomerular filtration rate	cystatin C measurement	EFO_0004617	Other measurement	Metabolic
0.9	3.13E-166	1.31E-162	PGS001818	MONDO_0005148	type 2 diabetes mellitus	type 2 diabetes mellitus	MONDO_0005148	Digestive system disorder	Gastrointestinal	PGS002026	MONDO_0005148	type 2 diabetes mellitus	type 2 diabetes mellitus	MONDO_0005148	Digestive system disorder	Gastrointestinal
0.9	3.13E-166	1.31E-162	PGS001818	MONDO_0005148	type 2 diabetes mellitus	type 2 diabetes mellitus	MONDO_0005148	Digestive system disorder	Gastrointestinal	PGS002026	MONDO_0005148	type 2 diabetes mellitus	type 2 diabetes mellitus	MONDO_0005148	Metabolic disorder	Metabolic
0.9	3.13E-166	1.31E-162	PGS001818	MONDO_0005148	type 2 diabetes mellitus	type 2 diabetes mellitus	MONDO_0005148	Metabolic disorder	Metabolic	PGS002026	MONDO_0005148	type 2 diabetes mellitus	type 2 diabetes mellitus	MONDO_0005148	Digestive system disorder	Gastrointestinal
0.9	3.13E-166	1.31E-162	PGS001818	MONDO_0005148	type 2 diabetes mellitus	type 2 diabetes mellitus	MONDO_0005148	Metabolic disorder	Metabolic	PGS002026	MONDO_0005148	type 2 diabetes mellitus	type 2 diabetes mellitus	MONDO_0005148	Metabolic disorder	Metabolic
0.9	3.50E-166	1.46E-162	PGS000476	EFO_0000305	breast carcinoma	breast carcinoma	EFO_0000305	Cancer	Neoplasms	PGS000480	EFO_0000305	breast carcinoma	breast carcinoma	EFO_0000305	Cancer	Neoplasms
0.9	3.77E-166	1.57E-162	PGS000482	EFO_0000305	breast carcinoma	breast carcinoma	EFO_0000305	Cancer	Neoplasms	PGS000476	EFO_0000305	breast carcinoma	breast carcinoma	EFO_0000305	Cancer	Neoplasms
0.8998	4.99E-166	2.08E-162	PGS000482	EFO_0000305	breast carcinoma	breast carcinoma	EFO_0000305	Cancer	Neoplasms	PGS000480	EFO_0000305	breast carcinoma	breast carcinoma	EFO_0000305	Cancer	Neoplasms
0.8996	7.37E-166	3.06E-162	PGS002421	EFO_0006335	systolic blood pressure	systolic blood pressure	EFO_0006335	Other measurement	Cardiovascular	PGS002568	EFO_0006335	systolic blood pressure	systolic blood pressure	EFO_0006335	Other measurement	Cardiovascular
0.8992	1.74E-165	7.24E-162	PGS000332	EFO_0000305	breast carcinoma	breast carcinoma	EFO_0000305	Cancer	Neoplasms	PGS000507	EFO_0000305	breast carcinoma	breast carcinoma	EFO_0000305	Cancer	Neoplasms
0.8992	1.87E-165	7.74E-162	PGS000457	EFO_0004193	basal cell carcinoma	basal cell carcinoma	EFO_0004193	Cancer	Neoplasms	PGS000453	EFO_0004193	basal cell carcinoma	basal cell carcinoma	EFO_0004193	Cancer	Neoplasms
0.8992	1.87E-165	7.74E-162	PGS000456	EFO_0004193	basal cell carcinoma	basal cell carcinoma	EFO_0004193	Cancer	Neoplasms	PGS000453	EFO_0004193	basal cell carcinoma	basal cell carcinoma	EFO_0004193	Cancer	Neoplasms
0.8992	1.87E-165	7.74E-162	PGS000455	EFO_0004193	basal cell carcinoma	basal cell carcinoma	EFO_0004193	Cancer	Neoplasms	PGS000456	EFO_0004193	basal cell carcinoma	basal cell carcinoma	EFO_0004193	Cancer	Neoplasms
0.8992	1.87E-165	7.74E-162	PGS000457	EFO_0004193	basal cell carcinoma	basal cell carcinoma	EFO_0004193	Cancer	Neoplasms	PGS000452	EFO_0004193	basal cell carcinoma	basal cell carcinoma	EFO_0004193	Cancer	Neoplasms
0.8992	1.87E-165	7.74E-162	PGS000456	EFO_0004193	basal cell carcinoma	basal cell carcinoma	EFO_0004193	Cancer	Neoplasms	PGS000452	EFO_0004193	basal cell carcinoma	basal cell carcinoma	EFO_0004193	Cancer	Neoplasms
0.8992	1.87E-165	7.74E-162	PGS000455	EFO_0004193	basal cell carcinoma	basal cell carcinoma	EFO_0004193	Cancer	Neoplasms	PGS000457	EFO_0004193	basal cell carcinoma	basal cell carcinoma	EFO_0004193	Cancer	Neoplasms
0.8992	1.99E-165	8.22E-162	PGS000773	EFO_0000305	breast carcinoma	breast carcinoma	EFO_0000305	Cancer	Neoplasms	PGS000873	EFO_0000305	breast carcinoma	breast carcinoma	EFO_0000305	Cancer	Neoplasms
0.8992	2.10E-165	8.65E-162	PGS003311	EFO_0004617	cystatin C measurement|glomerular filtration rate	cystatin C measurement	EFO_0004617	Other measurement	Metabolic	PGS003298	EFO_0004617	cystatin C measurement|glomerular filtration rate	cystatin C measurement	EFO_0004617	Other measurement	Metabolic
0.8991	2.26E-165	9.31E-162	PGS000014	MONDO_0005148	type 2 diabetes mellitus	type 2 diabetes mellitus	MONDO_0005148	Metabolic disorder	Metabolic	PGS003108	MONDO_0005148	type 2 diabetes mellitus	type 2 diabetes mellitus	MONDO_0005148	Metabolic disorder	Metabolic

0.8991	2.26E-165	9.31E-162	PGS000014	MONDO_0005148	type 2 diabetes mellitus	type 2 diabetes mellitus	MONDO_0005148	Digestive system disorder	Gastrointestinal	PGS003108	MONDO_0005148	type 2 diabetes mellitus	type 2 diabetes mellitus	MONDO_0005148	Digestive system disorder	Gastrointestinal
0.8991	2.26E-165	9.31E-162	PGS000014	MONDO_0005148	type 2 diabetes mellitus	type 2 diabetes mellitus	MONDO_0005148	Digestive system disorder	Gastrointestinal	PGS003108	MONDO_0005148	type 2 diabetes mellitus	type 2 diabetes mellitus	MONDO_0005148	Metabolic disorder	Metabolic
0.8991	2.26E-165	9.31E-162	PGS000014	MONDO_0005148	type 2 diabetes mellitus	type 2 diabetes mellitus	MONDO_0005148	Metabolic disorder	Metabolic	PGS003108	MONDO_0005148	type 2 diabetes mellitus	type 2 diabetes mellitus	MONDO_0005148	Digestive system disorder	Gastrointestinal
0.8991	2.39E-165	9.84E-162	PGS001735	EFO_0006930	brain volume measurement|neuroimaging measurement	brain volume measurement	EFO_0006930	Other measurement	Neurological	PGS001721	EFO_0006930	measurement	brain volume measurement	EFO_0006930	Other measurement	Neurological
0.8991	2.58E-165	1.06E-161	PGS001914	EFO_0004908	testosterone measurement|female	testosterone measurement	EFO_0004908	Lipid or lipoprotein measurement	Metabolic	PGS002130	EFO_0004908	testosterone measurement|female	testosterone measurement	EFO_0004908	Lipid or lipoprotein measurement	Metabolic
0.8991	2.61E-165	1.07E-161	PGS000515	EFO_0000305	breast carcinoma	breast carcinoma	EFO_0000305	Cancer	Neoplasms	PGS000513	EFO_0000305	breast carcinoma	breast carcinoma	EFO_0000305	Cancer	Neoplasms
0.899	2.65E-165	1.09E-161	PGS002573	MONDO_0005148	type 2 diabetes mellitus	type 2 diabetes mellitus	MONDO_0005148	Metabolic disorder	Metabolic	PGS002400	EFO_0004468	glucose measurement	glucose measurement	EFO_0004468	Other measurement	Metabolic
0.899	2.65E-165	1.09E-161	PGS002573	MONDO_0005148	type 2 diabetes mellitus	type 2 diabetes mellitus	MONDO_0005148	Digestive system disorder	Gastrointestinal	PGS002400	EFO_0004468	glucose measurement	glucose measurement	EFO_0004468	Other measurement	Metabolic
0.8989	3.93E-165	1.61E-161	PGS002173	EFO_0009270	heel bone mineral density	heel bone mineral density	EFO_0009270	Other measurement	Skeletal	PGS001956	EFO_0007772	measurement	measurement	EFO_0007772	Other measurement	Skeletal
0.8987	5.52E-165	2.26E-161	PGS002152	EFO_0007878	alcohol consumption measurement	alcohol consumption measurement	EFO_0007878	Other measurement	Psychiatric	PGS001934	EFO_0007878	alcohol consumption measurement	alcohol consumption measurement	EFO_0007878	Other measurement	Psychiatric
0.8987	5.58E-165	2.28E-161	PGS000347	EFO_1000649	estrogen-receptor positive breast cancer	estrogen-receptor positive breast cancer	EFO_1000649	Cancer	Neoplasms	PGS000212	MONDO_0021116	luminal A breast carcinoma	luminal A breast carcinoma	MONDO_0021116	Cancer	Neoplasms
0.8986	6.83E-165	2.79E-161	PGS001942	EFO_0004570	bilirubin measurement	bilirubin measurement	EFO_0004570	Other measurement	Metabolic	PGS002032	Orphanet_309816	Disorder of bilirubin metabolism and excretion	excretion	Orphanet_309816	Metabolic disorder	Metabolic
0.8985	9.12E-165	3.73E-161	PGS002623	EFO_0004631	vitamin D measurement	vitamin D measurement	EFO_0004631	Lipid or lipoprotein measurement	Metabolic	PGS003196	EFO_0004631	vitamin D measurement	vitamin D measurement	EFO_0004631	Lipid or lipoprotein measurement	Metabolic
0.8984	1.02E-164	4.15E-161	PGS000213	EFO_0009780	HER2 negative breast carcinoma	HER2 negative breast carcinoma	EFO_0009780	Cancer	Neoplasms	PGS000212	MONDO_0021116	luminal A breast carcinoma	luminal A breast carcinoma	MONDO_0021116	Cancer	Neoplasms
0.8983	1.26E-164	5.16E-161	PGS003312	EFO_0004617	cystatin C measurement|glomerular filtration rate	cystatin C measurement	EFO_0004617	Other measurement	Metabolic	PGS003311	EFO_0004617	cystatin C measurement|glomerular filtration rate	cystatin C measurement	EFO_0004617	Other measurement	Metabolic
0.898	2.66E-164	1.09E-160	PGS003297	EFO_0004617	cystatin C measurement|glomerular filtration rate	cystatin C measurement	EFO_0004617	Other measurement	Metabolic	PGS003311	EFO_0004617	cystatin C measurement|glomerular filtration rate	cystatin C measurement	EFO_0004617	Other measurement	Metabolic
0.898	2.70E-164	1.10E-160	PGS002547	EFO_0004468	glucose measurement	glucose measurement	EFO_0004468	Other measurement	Metabolic	PGS002589	EFO_0000400	diabetes mellitus	diabetes mellitus	EFO_0000400	Digestive system disorder	Gastrointestinal
0.898	2.70E-164	1.10E-160	PGS002547	EFO_0004468	glucose measurement	glucose measurement	EFO_0004468	Other measurement	Metabolic	PGS002589	EFO_0000400	diabetes mellitus	diabetes mellitus	EFO_0000400	Metabolic disorder	Metabolic
0.8979	3.18E-164	1.29E-160	PGS002488	EFO_0008328	chronotype measurement	chronotype measurement	EFO_0008328	Other measurement	Psychiatric	PGS002439	EFO_0008328	chronotype measurement	chronotype measurement	EFO_0008328	Other measurement	Psychiatric
0.8976	5.31E-164	2.16E-160	PGS002186	EFO_0005091	monocyte count	monocyte count	EFO_0005091	Hematological measurement	Hematological	PGS002341	EFO_0005091	monocyte count	monocyte count	EFO_0005091	Hematological measurement	Hematological
0.8974	8.11E-164	3.30E-160	PGS003313	EFO_0004617	cystatin C measurement|glomerular filtration rate	cystatin C measurement	EFO_0004617	Other measurement	Metabolic	PGS003310	EFO_0004617	cystatin C measurement|glomerular filtration rate	cystatin C measurement	EFO_0004617	Other measurement	Metabolic
0.8974	8.36E-164	3.39E-160	PGS001752	EFO_0006930	brain volume measurement|neuroimaging measurement	brain volume measurement	EFO_0006930	Other measurement	Neurological	PGS001723	EFO_0006930	measurement	brain volume measurement	EFO_0006930	Other measurement	Neurological
0.8974	9.47E-164	3.84E-160	PGS002146	EFO_0004339	body height	body height	EFO_0004339	Body measurement	Body measurement	PGS002973	EFO_0004339	body height	body height	EFO_0004339	Body measurement	Body measurement
0.8973	1.16E-163	4.71E-160	PGS002842	EFO_0004340	body mass index	body mass index	EFO_0004340	Body measurement	Body measurement	PGS002841	EFO_0004340	body mass index	body mass index	EFO_0004340	Body measurement	Body measurement
0.8972	1.38E-163	5.59E-160	PGS001267	EFO_0003898	ankylosing spondylitis	ankylosing spondylitis	EFO_0003898	Immune system disorder	Immunological	PGS001876	EFO_0003898	ankylosing spondylitis	ankylosing spondylitis	EFO_0003898	Immune system disorder	Immunological
0.8972	1.41E-163	5.69E-160	PGS001367	EFO_0004731	eye measurement	eye measurement	EFO_0004731	Other measurement	Ophthalmological	PGS001365	EFO_0004731	eye measurement	eye measurement	EFO_0004731	Other measurement	Ophthalmological
0.8971	1.58E-163	6.38E-160	PGS001228	EFO_0004340	body mass index	body mass index	EFO_0004340	Body measurement	Body measurement	PGS001144	EFO_0005409	fat body mass	fat body mass	EFO_0005409	Other measurement	Body measurement
0.897	1.97E-163	7.94E-160	PGS000190	EFO_0007986	reticulocyte count	reticulocyte count	EFO_0007986	Hematological measurement	Hematological	PGS000189	EFO_0007986	reticulocyte count	reticulocyte count	EFO_0007986	Hematological measurement	Hematological
0.897	1.99E-163	8.03E-160	PGS002619	EFO_1000627	thyroid disease	thyroid disease	EFO_1000627	Other disease	Endocrine	PGS002604	EFO_0004705	hypothyroidism	hypothyroidism	EFO_0004705	Other disease	Endocrine
0.897	2.01E-163	8.10E-160	PGS001956	EFO_0007772	calcaneal bone quantitative ultrasound measurement	calcaneal bone quantitative ultrasound measurement	EFO_0007772	Other measurement	Skeletal	PGS001957	EFO_0005654	velocity of sound measurement	velocity of sound measurement	EFO_0005654	Other measurement	Others
0.897	2.12E-163	8.55E-160	PGS000095	EFO_0007986	reticulocyte count	reticulocyte count	EFO_0007986	Hematological measurement	Hematological	PGS000094	EFO_0007986	reticulocyte count	reticulocyte count	EFO_0007986	Hematological measurement	Hematological
0.8968	3.05E-163	1.23E-159	PGS002107	EFO_0001060	celiac disease	celiac disease	EFO_0001060	Digestive system disorder	Gastrointestinal	PGS002067	EFO_0001060	celiac disease	celiac disease	EFO_0001060	Digestive system disorder	Gastrointestinal
0.8968	3.05E-163	1.23E-159	PGS002107	EFO_0001060	celiac disease	celiac disease	EFO_0001060	Digestive system disorder	Gastrointestinal	PGS002067	EFO_0001060	celiac disease	celiac disease	EFO_0001060	Immune system disorder	Immunological
0.8968	3.05E-163	1.23E-159	PGS002107	EFO_0001060	celiac disease	celiac disease	EFO_0001060	Immune system disorder	Immunological	PGS002067	EFO_0001060	celiac disease	celiac disease	EFO_0001060	Digestive system disorder	Gastrointestinal
0.8968	3.05E-163	1.23E-159	PGS002107	EFO_0001060	celiac disease	celiac disease	EFO_0001060	Immune system disorder	Immunological	PGS002067	EFO_0001060	celiac disease	celiac disease	EFO_0001060	Immune system disorder	Immunological
0.8966	4.52E-163	1.82E-159	PGS000532	EFO_0000305	breast carcinoma	breast carcinoma	EFO_0000305	Cancer	Neoplasms	PGS000531	EFO_0000305	breast carcinoma	breast carcinoma	EFO_0000305	Cancer	Neoplasms
0.8965	5.26E-163	2.11E-159	PGS002540	EFO_0000400	diabetes mellitus	diabetes mellitus	EFO_0000400	Metabolic disorder	Metabolic	PGS002596	EFO_0004468	glucose measurement	glucose measurement	EFO_0004468	Other measurement	Metabolic
0.8965	5.26E-163	2.11E-159	PGS002540	EFO_0000400	diabetes mellitus	diabetes mellitus	EFO_0000400	Digestive system disorder	Gastrointestinal	PGS002596	EFO_0004468	glucose measurement	glucose measurement	EFO_0004468	Other measurement	Metabolic
0.8965	5.31E-163	2.13E-159	PGS001876	EFO_0003898	ankylosing spondylitis	ankylosing spondylitis	EFO_0003898	Immune system disorder	Immunological	PGS001268	EFO_0003898	ankylosing spondylitis	ankylosing spondylitis	EFO_0003898	Immune system disorder	Immunological
0.8965	6.13E-163	2.46E-159	PGS001106	EFO_0007800	body fat percentage	body fat percentage	EFO_0007800	Body measurement	Body measurement	PGS001101	EFO_0007800	body fat percentage	body fat percentage	EFO_0007800	Body measurement	Body measurement
0.8964	6.39E-163	2.56E-159	PGS002321	EFO_0000400	diabetes mellitus	diabetes mellitus	EFO_0000400	Digestive system disorder	Gastrointestinal	PGS002324	EFO_0001379	endocrine system disease|diabetes mellitus	endocrine system disease	EFO_0001379	Other disease	Metabolic
0.8964	6.39E-163	2.56E-159	PGS002321	EFO_0000400	diabetes mellitus	diabetes mellitus	EFO_0000400	Metabolic disorder	Metabolic	PGS002324	EFO_0001379	endocrine system disease|diabetes mellitus	endocrine system disease	EFO_0001379	Other disease	Metabolic
0.8964	7.36E-163	2.95E-159	PGS001758	EFO_0006930	brain volume measurement|neuroimaging measurement	brain volume measurement	EFO_0006930	Other measurement	Neurological	PGS001728	EFO_0006930	measurement	brain volume measurement	EFO_0006930	Other measurement	Neurological
0.8962	1.04E-162	4.18E-159	PGS001814	MONDO_0000334	multinodular goiter|nontoxic goiter	multinodular goiter	MONDO_0000334	Other disease	Endocrine	PGS002022	MONDO_0000334	multinodular goiter|nontoxic goiter	multinodular goiter	MONDO_0000334	Other disease	Endocrine
0.896	1.44E-162	5.77E-159	PGS000783	EFO_0000305	breast carcinoma	breast carcinoma	EFO_0000305	Cancer	Neoplasms	PGS000317	EFO_0000305	breast carcinoma	breast carcinoma	EFO_0000305	Cancer	Neoplasms
0.896	1.50E-162	5.98E-159	PGS003385	EFO_1001516	ovarian serous carcinoma	ovarian serous carcinoma	EFO_1001516	Cancer	Neoplasms	PGS000564	EFO_0003893	ovarian neoplasm	ovarian neoplasm	EFO_0003893	Cancer	Neoplasms
0.896	1.52E-162	6.06E-159	PGS002445	EFO_0001379	endocrine system disease|diabetes mellitus	endocrine system disease	EFO_0001379	Other disease	Metabolic	PGS002547	EFO_0004468	glucose measurement	glucose measurement	EFO_0004468	Other measurement	Metabolic
0.8959	1.82E-162	7.28E-159	PGS001815	EFO_0009190	Thyrotoxicosis	Thyrotoxicosis	EFO_0009190	Immune system disorder	Immunological	PGS002023	EFO_0009190	Thyrotoxicosis	Thyrotoxicosis	EFO_0009190	Immune system disorder	Immunological
0.8959	2.08E-162	8.29E-159	PGS001099	EFO_0007814	refractive error measurement	refractive error measurement	EFO_0007814	Other measurement	Ophthalmological	PGS001100	EFO_0007814	refractive error measurement	refractive error measurement	EFO_0007814	Other measurement	Ophthalmological
0.8958	2.67E-162	1.06E-158	PGS002345	EFO_0004305	erythrocyte count	erythrocyte count	EFO_0004305	Hematological measurement	Hematological	PGS002123	EFO_0004305	erythrocyte count	erythrocyte count	EFO_0004305	Hematological measurement	Hematological
0.8954	5.92E-162	2.35E-158	PGS002426	MONDO_0005148	type 2 diabetes mellitus	type 2 diabetes mellitus	MONDO_0005148	Digestive system disorder	Gastrointestinal	PGS002400	EFO_0004468	glucose measurement	glucose measurement	EFO_0004468	Other measurement	Metabolic
0.8954	5.92E-162	2.35E-158	PGS002426	MONDO_0005148	type 2 diabetes mellitus	type 2 diabetes mellitus	MONDO_0005148	Metabolic disorder	Metabolic	PGS002400	EFO_0004468	glucose measurement	glucose measurement	EFO_0004468	Other measurement	Metabolic
0.8953	7.40E-162	2.94E-158	PGS000612	MONDO_0004986	urinary bladder carcinoma	urinary bladder carcinoma	MONDO_0004986	Cancer	Neoplasms	PGS000782	MONDO_0004986	urinary bladder carcinoma	urinary bladder carcinoma	MONDO_0004986	Cancer	Neoplasms
0.8953	7.40E-162	2.94E-158	PGS000611	MONDO_0004986	urinary bladder carcinoma	urinary bladder carcinoma	MONDO_0004986	Cancer	Neoplasms	PGS000782	MONDO_0004986	urinary bladder carcinoma	urinary bladder carcinoma	MONDO_0004986	Cancer	Neoplasms
0.8953	7.40E-162	2.94E-158	PGS000610	MONDO_0004986	urinary bladder carcinoma	urinary bladder carcinoma	MONDO_0004986	Cancer	Neoplasms	PGS000782	MONDO_0004986	urinary bladder carcinoma	urinary bladder carcinoma	MONDO_0004986	Cancer	Neoplasms
0.8952	7.88E-162	3.12E-158	PGS002851	EFO_0004340	body mass index	body mass index	EFO_0004340	Body measurement	Body measurement	PGS002846	EFO_0004340	body mass index	body mass index	EFO_0004340	Body measurement	Body measurement
0.8952	8.67E-162	3.43E-158	PGS000006	EFO_1000650	estrogen-receptor negative breast cancer	estrogen-receptor negative breast cancer	EFO_1000650	Cancer	Neoplasms	PGS000346	EFO_1000650	estrogen-receptor negative breast cancer	cancer	EFO_1000650	Cancer	Neoplasms
0.8952	9.18E-162	3.63E-158	PGS002393	EFO_0000400	diabetes mellitus	diabetes mellitus	EFO_0000400	Metabolic disorder	Metabolic	PGS002400	EFO_0004468	glucose measurement	glucose measurement	EFO_0004468	Other measurement	Metabolic
0.8952	9.18E-162	3.63E-158	PGS002393	EFO_0000400	diabetes mellitus	diabetes mellitus	EFO_0000400	Digestive system disorder	Gastrointestinal	PGS002400	EFO_0004468	glucose measurement	glucose measurement	EFO_0004468	Other measurement	Metabolic
0.8951	9.86E-162	3.90E-158	PGS002540	EFO_0000400	diabetes mellitus	diabetes mellitus	EFO_0000400	Metabolic disorder	Metabolic	PGS002452	EFO_0004541	HbA1c measurement	HbA1c measurement	EFO_0004541	Hematological measurement	Hematological
0.8951	9.86E-162	3.90E-158	PGS002540	EFO_0000400	diabetes mellitus	diabetes mellitus	EFO_0000400	Digestive system disorder	Gastrointestinal	PGS002452	EFO_0004541	HbA1c measurement	HbA1c measurement	EFO_0004541	Hematological measurement	Hematological
0.895	1.17E-161	4.62E-158	PGS002120	EFO_0004682	QT interval	QT interval	EFO_0004682	Cardiovascular measurement	Cardiovascular	PGS001906	EFO_0004682	QT interval	QT interval	EFO_0004682	Cardiovascular measurement	Cardiovascular
0.8949	1.67E-161	6.61E-158	PGS002065	HP_0000023	Inguinal hernia	Inguinal hernia	HP_0000023	Other trait	Gastrointestinal	PGS001854	HP_0000023	Inguinal hernia	Inguinal hernia	HP_0000023	Other trait	Gastrointestinal
0.8948	1.78E-161	7.03E-158	PGS002721	EFO_0004631	vitamin D measurement	vitamin D measurement	EFO_0004631	Lipid or lipoprotein measurement	Metabolic	PGS001982	EFO_0004631	vitamin D measurement	vitamin D measurement	EFO_0004631	Lipid or lipoprotein measurement	Metabolic
0.8946	2.70E-161	1.06E-157	PGS002383	MONDO_0004979	asthma	asthma	MONDO_0004979	Other disease	Respiratory	PGS002468	EFO_0000684	respiratory system disease	respiratory system disease	EFO_0000684	Other disease	Skeletal
0.8945	3.51E-161	1.38E-157	PGS001954	EFO_0004612	high density lipoprotein cholesterol measurement	high density lipoprotein cholesterol measurement	EFO_0004612	Lipid or lipoprotein measurement	Metabolic	PGS001888	EFO_0004614	apolipoprotein A 1 measurement	apolipoprotein A 1 measurement	EFO_0004614	Lipid or lipoprotein measurement	Metabolic
0.8945	3.51E-161	1.38E-157	PGS001954	EFO_0004612	high density lipoprotein cholesterol measurement	high density lipoprotein cholesterol measurement	EFO_0004612	Lipid or lipoprotein measurement	Metabolic	PGS001888	EFO_0004614	apolipoprotein A 1 measurement	apolipoprotein A 1 measurement	EFO_0004614	Cardiovascular measurement	Cardiovascular
0.8945	3.51E-161	1.38E-157	PGS001954	EFO_0004612	high density lipoprotein cholesterol measurement	high density lipoprotein cholesterol measurement	EFO_0004612	Lipid or lipoprotein measurement	Metabolic	PGS001888	EFO_0004614	apolipoprotein A 1 measurement	apolipoprotein A 1 measurement	EFO_0004614	Inflammatory measurement	Immunological
0.8943	4.88E-161	1.92E-157	PGS002612	EFO_0000676	psoriasis	psoriasis	EFO_0000676	Immune system disorder	Immunological	PGS002563	EFO_0000676	psoriasis	psoriasis	EFO_0000676	Immune system disorder	Immunological
0.8943	5.16E-161	2.03E-157	PGS002108	EFO_0004574	total cholesterol measurement	total cholesterol measurement	EFO_0004574	Lipid or lipoprotein measurement	Metabolic	PGS002352	EFO_0004574	total cholesterol measurement	total cholesterol measurement	EFO_0004574	Lipid or lipoprotein measurement	Metabolic
0.8942	5.86E-161	2.30E-157	PGS002405	EFO_0007986	reticulocyte count	reticulocyte count	EFO_0007986	Hematological measurement	Hematological	PGS002454	EFO_0007986	reticulocyte count	reticulocyte count	EFO_0007986	Hematological measurement	Hematological
0.8942	6.33E-161	2.48E-157	PGS002672	EFO_0004631	vitamin D measurement	vitamin D measurement	EFO_0004631	Lipid or lipoprotein measurement	Metabolic	PGS002721	EFO_0004631	vitamin D measurement	vitamin D measurement	EFO_0004631	Lipid or lipoprotein measurement	Metabolic
0.8942	6.55E-161	2.57E-157	PGS000391	MONDO_0001672	bronchus cancer|lung cancer|tracheal cancer	bronchus cancer	MONDO_0001672	Cancer	Neoplasms	PGS000390	MONDO_0001672	bronchus cancer|lung cancer|tracheal cancer	bronchus cancer	MONDO_0001672	Cancer	Neoplasms
0.8942	6.55E-161	2.57E-157	PGS001366	EFO_0004731	eye measurement	eye measurement	EFO_0004731	Other measurement	Ophthalmological	PGS001368	EFO_0004731	eye measurement	eye measurement	EFO_0004731	Other measurement	Ophthalmological
0.8942	6.55E-161	2.57E-157	PGS000391	MONDO_0001672	bronchus cancer|lung cancer|tracheal cancer	bronchus cancer	MONDO_0001672	Cancer	Neoplasms	PGS000389	MONDO_0001672	bronchus cancer|lung cancer|tracheal cancer	bronchus cancer	MONDO_0001672	Cancer	Neoplasms
0.894	8.73E-161	3.42E-157	PGS002146	EFO_0004339	body height	body height	EFO_0004339	Body measurement	Body measurement	PGS002968	EFO_0004339	body height	body height	EFO_0004339	Body measurement	Body measurement
0.894	9.18E-161	3.59E-157	PGS002452	EFO_0004541	HbA1c measurement	HbA1c measurement	EFO_0004541	Hematological measurement	Hematological	PGS002396	EFO_0001379	endocrine system disease|diabetes mellitus	endocrine system disease	EFO_0001379	Other disease	Metabolic
0.8939	1.25E-160	4.87E-157	PGS001671	EFO_0006930	brain volume measurement|neuroimaging measurement	brain volume measurement	EFO_0006930	Other measurement	Neurological	PGS001673	EFO_0006930	measurement	brain volume measurement	EFO_0006930	Other measurement	Neurological
0.8938	1.47E-160	5.75E-157	PGS002169	EFO_0005409	fat body mass	fat body mass	EFO_0005409	Other measurement	Body measurement	PGS002201	EFO_0004342	waist circumference	waist circumference	EFO_0004342	Body measurement	Body measurement
0.8935	2.46E-160	9.59E-157	PGS001966	EFO_0007117	carotid artery intima media thickness	carotid artery intima media thickness	EFO_0007117	Other measurement	Cardiovascular	PGS002184	EFO_0007117	carotid artery intima media thickness	carotid artery intima media thickness	EFO_0007117	Other measurement	Cardiovascular
0.8935	2.71E-160	1.06E-156	PGS002403	EFO_0004541	HbA1c measurement	HbA1c measurement	EFO_0004541	Hematological measurement	Hematological	PGS002599	EFO_0004541	HbA1c measurement	HbA1c measurement	EFO_0004541	Hematological measurement	Hematological
-0.8935	2.73E-160	1.06E-156	PGS001246	EFO_0004279	suntan	suntan	EFO_0004279	Other trait	Dermatological	PGS001247	EFO_0004279	suntan	suntan	EFO_0004279	Other trait	Dermatological
0.8934	3.01E-160	1.17E-156	PGS002596	EFO_0004468	glucose measurement	glucose measurement	EFO_0004468	Other measurement	Metabolic	PGS002573	MONDO_0005148	type 2 diabetes mellitus	type 2 diabetes mellitus	MONDO_0005148	Digestive system disorder	Gastrointestinal
0.8934	3.01E-160	1.17E-156	PGS002596	EFO_0004468	glucose measurement	glucose measurement	EFO_0004468	Other measurement	Metabolic	PGS002573	MONDO_0005148	type 2 diabetes mellitus	type 2 diabetes mellitus	MONDO_0005148	Metabolic disorder	Metabolic
0.8932	4.37E-160	1.70E-156	PGS002619	EFO_1000627	thyroid disease	thyroid disease	EFO_1000627	Other disease	Endocrine	PGS002580	EFO_0005140	autoimmune disease	autoimmune disease	EFO_0005140	Immune system disorder	Immunological
0.8931	5.84E-160	2.27E-156	PGS000101	EFO_0004526	mean corpuscular volume	mean corpuscular volume	EFO_0004526	Hematological measurement	Hematological	PGS000099	EFO_0004527	mean corpuscular hemoglobin	mean corpuscular hemoglobin	EFO_0004527	Hematological measurement	Hematological
0.893	6.60E-160	2.56E-156	PGS000642	EFO_0005952	non-Hodgkins lymphoma	non-Hodgkins lymphoma	EFO_0005952	Cancer	Neoplasms	PGS000641	EFO_0005952	non-Hodgkins lymphoma	non-Hodgkins lymphoma	EFO_0005952	Cancer	Neoplasms
0.893	6.64E-160	2.57E-156	PGS001905	EFO_0004682	QT interval	QT interval	EFO_0004682	Cardiovascular measurement	Cardiovascular	PGS002119	EFO_0004682	QT interval	QT interval	EFO_0004682	Cardiovascular measurement	Cardiovascular
0.8928	1.00E-159	3.89E-156	PGS002321	EFO_0000400	diabetes mellitus	diabetes mellitus	EFO_0000400	Digestive system disorder	Gastrointestinal	PGS003093	MONDO_0005148	type 2 diabetes mellitus	type 2 diabetes mellitus	MONDO_0005148	Digestive system disorder	Gastrointestinal
0.8928	1.00E-159	3.89E-156	PGS002321	EFO_0000400	diabetes mellitus	diabetes mellitus	EFO_0000400	Digestive system disorder	Gastrointestinal	PGS003093	MONDO_0005148	type 2 diabetes mellitus	type 2 diabetes mellitus	MONDO_0005148	Metabolic disorder	Metabolic
0.8928	1.00E-159	3.89E-156	PGS002321	EFO_0000400	diabetes mellitus	diabetes mellitus	EFO_0000400	Metabolic disorder	Metabolic	PGS003093	MONDO_0005148	type 2 diabetes mellitus	type 2 diabetes mellitus	MONDO_0005148	Digestive system disorder	Gastrointestinal
0.8928	1.00E-159	3.89E-156	PGS002321	EFO_0000400	diabetes mellitus	diabetes mellitus	EFO_0000400	Metabolic disorder	Metabolic	PGS003093	MONDO_0005148	type 2 diabetes mellitus	type 2 diabetes mellitus	MONDO_0005148	Metabolic disorder	Metabolic
0.8928	1.03E-159	3.99E-156	PGS002355	EFO_0004631	vitamin D measurement	vitamin D measurement	EFO_0004631	Lipid or lipoprotein measurement	Metabolic	PGS001982	EFO_0004631	vitamin D measurement	vitamin D measurement	EFO_0004631	Lipid or lipoprotein measurement	Metabolic
0.8927	1.24E-159	4.81E-156	PGS002759	EFO_0003761	unipolar depression	unipolar depression	EFO_0003761	Neurological disorder	Neurological	PGS002789	MONDO_0002009	major depressive disorder	major depressive disorder	MONDO_0002009	Neurological disorder	Neurological
-0.8927	1.36E-159	5.25E-156	PGS002919	EFO_0004697	estradiol measurement	estradiol measurement	EFO_0004697	Other measurement	Endocrine	PGS001913	EFO_0007786	menstrual cycle measurement	menstrual cycle measurement	EFO_0007786	Other measurement	Genitourinary
0.8925	1.87E-159	7.22E-156	PGS000457	EFO_0004193	basal cell carcinoma	basal cell carcinoma	EFO_0004193	Cancer	Neoplasms	PGS000454	EFO_0004193	basal cell carcinoma	basal cell carcinoma	EFO_0004193	Cancer	Neoplasms
0.8925	1.87E-159	7.22E-156	PGS000456	EFO_0004193	basal cell carcinoma	basal cell carcinoma	EFO_0004193	Cancer	Neoplasms	PGS000454	EFO_0004193	basal cell carcinoma	basal cell carcinoma	EFO_0004193	Cancer	Neoplasms
0.8924	2.31E-159	8.90E-156	PGS002332	EFO_0004339	body height	body height	EFO_0004339	Body measurement	Body measurement	PGS002968	EFO_0004339	body height	body height	EFO_0004339	Body measurement	Body measurement
0.8924	2.49E-159	9.57E-156	PGS002589	EFO_0000400	diabetes mellitus	diabetes mellitus	EFO_0000400	Digestive system disorder	Gastrointestinal	PGS002442	EFO_0000400	diabetes mellitus	diabetes mellitus	EFO_0000400	Digestive system disorder	Gastrointestinal
0.8924	2.49E-159	9.57E-156	PGS002589	EFO_0000400	diabetes mellitus	diabetes mellitus	EFO_0000400	Digestive system disorder	Gastrointestinal	PGS002442	EFO_0000400	diabetes mellitus	diabetes mellitus	EFO_0000400	Metabolic disorder	Metabolic
0.8924	2.49E-159	9.57E-156	PGS002589	EFO_0000400	diabetes mellitus	diabetes mellitus	EFO_0000400	Metabolic disorder	Metabolic	PGS002442	EFO_0000400	diabetes mellitus	diabetes mellitus	EFO_0000400	Metabolic disorder	Metabolic
0.8924	2.49E-159	9.57E-156	PGS002589	EFO_0000400	diabetes mellitus	diabetes mellitus	EFO_0000400	Metabolic disorder	Metabolic	PGS002442	EFO_0000400	diabetes mellitus	diabetes mellitus	EFO_0000400	Digestive system disorder	Gastrointestinal
0.8923	2.70E-159	1.04E-155	PGS002550	EFO_0004541	HbA1c measurement	HbA1c measurement	EFO_0004541	Hematological measurement	Hematological	PGS002452	EFO_0004541	HbA1c measurement	HbA1c measurement	EFO_0004541	Hematological measurement	Hematological

0.8923	3.04E-159	1.17E-155	PGS000790	EFO_0000756	melanoma	melanoma	EFO_0000756	Cancer	Neoplasms	PGS000079	EFO_0000756	melanoma	melanoma	EFO_0000756	Cancer	Neoplasms
0.8922	3.73E-159	1.43E-155	PGS002453	EFO_0004339	body height	body height	EFO_0004339	Body measurement	Body measurement	PGS002600	EFO_0004339	body height	body height	EFO_0004339	Body measurement	Body measurement
0.892	5.53E-159	2.12E-155	PGS002592	EFO_0001379	endocrine system disease|diabetes mellitus	endocrine system disease	EFO_0001379	Other disease	Metabolic	PGS002452	EFO_0004541	HbA1c measurement	HbA1c measurement	EFO_0004541	Hematological measurement	Hematological
0.892	5.60E-159	2.15E-155	PGS003244	EFO_0010472	creatine measurement	creatine measurement	EFO_0010472	Other measurement	Metabolic	PGS003239	EFO_0010472	creatine measurement	creatine measurement	EFO_0010472	Other measurement	Metabolic
0.892	5.63E-159	2.16E-155	PGS002842	EFO_0004340	body mass index	body mass index	EFO_0004340	Body measurement	Body measurement	PGS002847	EFO_0004340	body mass index	body mass index	EFO_0004340	Body measurement	Body measurement
0.8919	5.90E-159	2.26E-155	PGS002500	EFO_0007822	hair colour measurement	hair colour measurement	EFO_0007822	Other measurement	Dermatological	PGS002451	EFO_0007822	hair colour measurement	hair colour measurement	EFO_0007822	Other measurement	Dermatological
0.8919	6.39E-159	2.44E-155	PGS001895	EFO_0004574	total cholesterol measurement	total cholesterol measurement	EFO_0004574	Lipid or lipoprotein measurement	Metabolic	PGS002352	EFO_0004574	total cholesterol measurement	total cholesterol measurement	EFO_0004574	Lipid or lipoprotein measurement	Metabolic
0.8916	1.29E-158	4.93E-155	PGS002562	EFO_0004309	platelet count	platelet count	EFO_0004309	Hematological measurement	Hematological	PGS002464	EFO_0004309	platelet count	platelet count	EFO_0004309	Hematological measurement	Hematological
0.8915	1.33E-158	5.09E-155	PGS002332	EFO_0004339	body height	body height	EFO_0004339	Body measurement	Body measurement	PGS002973	EFO_0004339	body height	body height	EFO_0004339	Body measurement	Body measurement
0.8914	1.89E-158	7.22E-155	PGS000486	EFO_0000305	breast carcinoma	breast carcinoma	EFO_0000305	Cancer	Neoplasms	PGS000488	EFO_0000305	breast carcinoma	breast carcinoma	EFO_0000305	Cancer	Neoplasms
0.8913	2.12E-158	8.08E-155	PGS002339	EFO_0004527	mean corpuscular hemoglobin	mean corpuscular hemoglobin	EFO_0004527	Hematological measurement	Hematological	PGS002207	EFO_0004526	mean corpuscular volume	mean corpuscular volume	EFO_0004526	Hematological measurement	Hematological
0.8913	2.16E-158	8.23E-155	PGS000795	EFO_0001663	prostate carcinoma	prostate carcinoma	EFO_0001663	Cancer	Neoplasms	PGS000719	EFO_0001663	prostate carcinoma	prostate carcinoma	EFO_0001663	Cancer	Neoplasms
0.8912	2.80E-158	1.07E-154	PGS002589	EFO_0000400	diabetes mellitus	diabetes mellitus	EFO_0000400	Digestive system disorder	Gastrointestinal	PGS002475	MONDO_0005148	type 2 diabetes mellitus	type 2 diabetes mellitus	MONDO_0005148	Digestive system disorder	Gastrointestinal
0.8912	2.80E-158	1.07E-154	PGS002589	EFO_0000400	diabetes mellitus	diabetes mellitus	EFO_0000400	Metabolic disorder	Metabolic	PGS002475	MONDO_0005148	type 2 diabetes mellitus	type 2 diabetes mellitus	MONDO_0005148	Digestive system disorder	Gastrointestinal
0.8912	2.80E-158	1.07E-154	PGS002589	EFO_0000400	diabetes mellitus	diabetes mellitus	EFO_0000400	Digestive system disorder	Gastrointestinal	PGS002475	MONDO_0005148	type 2 diabetes mellitus	type 2 diabetes mellitus	MONDO_0005148	Metabolic disorder	Metabolic
0.8912	2.80E-158	1.07E-154	PGS002589	EFO_0000400	diabetes mellitus	diabetes mellitus	EFO_0000400	Metabolic disorder	Metabolic	PGS002475	MONDO_0005148	type 2 diabetes mellitus	type 2 diabetes mellitus	MONDO_0005148	Metabolic disorder	Metabolic
0.8911	3.25E-158	1.24E-154	PGS002452	EFO_0004541	HbA1c measurement	HbA1c measurement	EFO_0004541	Hematological measurement	Hematological	PGS002622	MONDO_0005148	type 2 diabetes mellitus	type 2 diabetes mellitus	MONDO_0005148	Metabolic disorder	Metabolic
0.8911	3.25E-158	1.24E-154	PGS002452	EFO_0004541	HbA1c measurement	HbA1c measurement	EFO_0004541	Hematological measurement	Hematological	PGS002622	MONDO_0005148	type 2 diabetes mellitus	type 2 diabetes mellitus	MONDO_0005148	Digestive system disorder	Gastrointestinal
0.8911	3.33E-158	1.27E-154	PGS000213	EFO_0009780	HER2 negative breast carcinoma	HER2 negative breast carcinoma	EFO_0009780	Cancer	Neoplasms	PGS000214	MONDO_0021115	luminal B breast carcinoma	luminal B breast carcinoma	MONDO_0021115	Cancer	Neoplasms
-0.8909	4.43E-158	1.68E-154	PGS001997	EFO_0007990	neutrophil percentage of leukocytes	neutrophil percentage of leukocytes	EFO_0007990	Hematological measurement	Hematological	PGS001986	EFO_0007993	lymphocyte percentage of leukocytes	lymphocyte percentage of leukocytes	EFO_0007993	Hematological measurement	Hematological
0.8909	4.87E-158	1.85E-154	PGS002852	EFO_0004340	body mass index	body mass index	EFO_0004340	Body measurement	Body measurement	PGS002857	EFO_0004340	body mass index	body mass index	EFO_0004340	Body measurement	Body measurement
0.8909	4.91E-158	1.86E-154	PGS001682	EFO_0006930	brain volume measurement|neuroimaging measurement	brain volume measurement	EFO_0006930	Other measurement	Neurological	PGS001489	EFO_0006930	measurement	brain volume measurement	EFO_0006930	Other measurement	Neurological
0.8907	7.37E-158	2.79E-154	PGS003200	EFO_0004631	vitamin D measurement	vitamin D measurement	EFO_0004631	Lipid or lipoprotein measurement	Metabolic	PGS003184	EFO_0004631	vitamin D measurement	vitamin D measurement	EFO_0004631	Lipid or lipoprotein measurement	Metabolic
0.8906	8.08E-158	3.06E-154	PGS001674	EFO_0006930	brain volume measurement|neuroimaging measurement	brain volume measurement	EFO_0006930	Other measurement	Neurological	PGS001673	EFO_0006930	measurement	brain volume measurement	EFO_0006930	Other measurement	Neurological
0.8906	8.46E-158	3.20E-154	PGS002419	EFO_0000684	respiratory system disease	respiratory system disease	EFO_0000684	Other disease	Skeletal	PGS002468	EFO_0000684	respiratory system disease	respiratory system disease	EFO_0000684	Other disease	Skeletal
0.8906	8.92E-158	3.37E-154	PGS002426	MONDO_0005148	type 2 diabetes mellitus	type 2 diabetes mellitus	MONDO_0005148	Digestive system disorder	Gastrointestinal	PGS002596	EFO_0004468	glucose measurement	glucose measurement	EFO_0004468	Other measurement	Metabolic
0.8906	8.92E-158	3.37E-154	PGS002426	MONDO_0005148	type 2 diabetes mellitus	type 2 diabetes mellitus	MONDO_0005148	Metabolic disorder	Metabolic	PGS002596	EFO_0004468	glucose measurement	glucose measurement	EFO_0004468	Other measurement	Metabolic
0.8904	1.37E-157	5.17E-154	PGS003199	EFO_0004631	vitamin D measurement	vitamin D measurement	EFO_0004631	Lipid or lipoprotein measurement	Metabolic	PGS003200	EFO_0004631	vitamin D measurement	vitamin D measurement	EFO_0004631	Lipid or lipoprotein measurement	Metabolic
0.8903	1.48E-157	5.59E-154	PGS003312	EFO_0004617	cystatin C measurement|glomerular filtration rate	cystatin C measurement	EFO_0004617	Other measurement	Metabolic	PGS003309	EFO_0004617	cystatin C measurement|glomerular filtration rate	cystatin C measurement	EFO_0004617	Other measurement	Metabolic
0.8902	1.76E-157	6.63E-154	PGS000633	EFO_0002892	thyroid carcinoma	thyroid carcinoma	EFO_0002892	Cancer	Neoplasms	PGS000632	EFO_0002892	thyroid carcinoma	thyroid carcinoma	EFO_0002892	Cancer	Neoplasms
0.8902	1.98E-157	7.45E-154	PGS001148	EFO_0005409	fat body mass	fat body mass	EFO_0005409	Other measurement	Body measurement	PGS001101	EFO_0007800	body fat percentage	body fat percentage	EFO_0007800	Body measurement	Body measurement
0.8901	2.36E-157	8.89E-154	PGS002138	EFO_0004312	vital capacity	vital capacity	EFO_0004312	Other measurement	Activities	PGS002134	EFO_0004314	forced expiratory volume	forced expiratory volume	EFO_0004314	Other measurement	Respiratory
0.89	2.69E-157	1.01E-153	PGS001366	EFO_0004731	eye measurement	eye measurement	EFO_0004731	Other measurement	Ophthalmological	PGS001360	EFO_0004731	eye measurement	eye measurement	EFO_0004731	Other measurement	Ophthalmological
0.8899	3.64E-157	1.37E-153	PGS002122	EFO_0005192	red blood cell distribution width	red blood cell distribution width	EFO_0005192	Hematological measurement	Hematological	PGS002346	EFO_0005192	red blood cell distribution width	red blood cell distribution width	EFO_0005192	Hematological measurement	Hematological
0.8898	4.36E-157	1.64E-153	PGS003297	EFO_0004617	cystatin C measurement|glomerular filtration rate	cystatin C measurement	EFO_0004617	Other measurement	Metabolic	PGS003309	EFO_0004617	cystatin C measurement|glomerular filtration rate	cystatin C measurement	EFO_0004617	Other measurement	Metabolic
0.8896	5.52E-157	2.07E-153	PGS001955	EFO_0009270	heel bone mineral density	heel bone mineral density	EFO_0009270	Other measurement	Skeletal	PGS002174	EFO_0007772	measurement	measurement	EFO_0007772	Other measurement	Skeletal
0.8895	6.57E-157	2.47E-153	PGS001095	EFO_0007822	hair colour measurement	hair colour measurement	EFO_0007822	Other measurement	Dermatological	PGS001092	EFO_0007822	hair colour measurement	hair colour measurement	EFO_0007822	Other measurement	Dermatological
0.8895	7.84E-157	2.94E-153	PGS002161	EFO_0004340	body mass index	body mass index	EFO_0004340	Body measurement	Body measurement	PGS002313	EFO_0004340	body mass index	body mass index	EFO_0004340	Body measurement	Body measurement
0.8894	8.50E-157	3.18E-153	PGS001363	EFO_0004731	eye measurement	eye measurement	EFO_0004731	Other measurement	Ophthalmological	PGS001360	EFO_0004731	eye measurement	eye measurement	EFO_0004731	Other measurement	Ophthalmological
0.8892	1.26E-156	4.70E-153	PGS000465	EFO_0000707	squamous cell carcinoma	squamous cell carcinoma	EFO_0000707	Cancer	Neoplasms	PGS000462	EFO_0000707	squamous cell carcinoma	squamous cell carcinoma	EFO_0000707	Cancer	Neoplasms
0.8892	1.26E-156	4.70E-153	PGS000464	EFO_0000707	squamous cell carcinoma	squamous cell carcinoma	EFO_0000707	Cancer	Neoplasms	PGS000465	EFO_0000707	squamous cell carcinoma	squamous cell carcinoma	EFO_0000707	Cancer	Neoplasms
0.8892	1.26E-156	4.70E-153	PGS000466	EFO_0000707	squamous cell carcinoma	squamous cell carcinoma	EFO_0000707	Cancer	Neoplasms	PGS000462	EFO_0000707	squamous cell carcinoma	squamous cell carcinoma	EFO_0000707	Cancer	Neoplasms
0.8892	1.26E-156	4.70E-153	PGS000465	EFO_0000707	squamous cell carcinoma	squamous cell carcinoma	EFO_0000707	Cancer	Neoplasms	PGS000461	EFO_0000707	squamous cell carcinoma	squamous cell carcinoma	EFO_0000707	Cancer	Neoplasms
0.8892	1.26E-156	4.70E-153	PGS000464	EFO_0000707	squamous cell carcinoma	squamous cell carcinoma	EFO_0000707	Cancer	Neoplasms	PGS000466	EFO_0000707	squamous cell carcinoma	squamous cell carcinoma	EFO_0000707	Cancer	Neoplasms
0.8892	1.26E-156	4.70E-153	PGS000466	EFO_0000707	squamous cell carcinoma	squamous cell carcinoma	EFO_0000707	Cancer	Neoplasms	PGS000461	EFO_0000707	squamous cell carcinoma	squamous cell carcinoma	EFO_0000707	Cancer	Neoplasms
0.8892	1.32E-156	4.93E-153	PGS002308	MONDO_0005148	type 2 diabetes mellitus	type 2 diabetes mellitus	MONDO_0005148	Metabolic disorder	Metabolic	PGS002771	MONDO_0005148	type 2 diabetes mellitus	type 2 diabetes mellitus	MONDO_0005148	Metabolic disorder	Metabolic
0.8892	1.32E-156	4.93E-153	PGS002308	MONDO_0005148	type 2 diabetes mellitus	type 2 diabetes mellitus	MONDO_0005148	Digestive system disorder	Gastrointestinal	PGS002771	MONDO_0005148	type 2 diabetes mellitus	type 2 diabetes mellitus	MONDO_0005148	Digestive system disorder	Gastrointestinal
0.8892	1.32E-156	4.93E-153	PGS002308	MONDO_0005148	type 2 diabetes mellitus	type 2 diabetes mellitus	MONDO_0005148	Digestive system disorder	Gastrointestinal	PGS002771	MONDO_0005148	type 2 diabetes mellitus	type 2 diabetes mellitus	MONDO_0005148	Metabolic disorder	Metabolic
0.8892	1.32E-156	4.93E-153	PGS002308	MONDO_0005148	type 2 diabetes mellitus	type 2 diabetes mellitus	MONDO_0005148	Metabolic disorder	Metabolic	PGS002771	MONDO_0005148	type 2 diabetes mellitus	type 2 diabetes mellitus	MONDO_0005148	Digestive system disorder	Gastrointestinal
0.8891	1.62E-156	6.04E-153	PGS001737	EFO_0006930	brain volume measurement|neuroimaging measurement	brain volume measurement	EFO_0006930	Other measurement	Neurological	PGS001723	EFO_0006930	measurement	brain volume measurement	EFO_0006930	Other measurement	Neurological
0.889	1.77E-156	6.58E-153	PGS002791	EFO_0001663	prostate carcinoma	prostate carcinoma	EFO_0001663	Cancer	Neoplasms	PGS000580	EFO_0001663	prostate carcinoma	prostate carcinoma	EFO_0001663	Cancer	Neoplasms
0.8888	2.57E-156	9.57E-153	PGS000296	EFO_0001645	coronary artery disease	coronary artery disease	EFO_0001645	Cardiovascular disease	Cardiovascular	PGS002244	EFO_0001645	coronary artery disease	coronary artery disease	EFO_0001645	Cardiovascular disease	Cardiovascular
0.8888	2.81E-156	1.04E-152	PGS000062	EFO_0004574	total cholesterol measurement	total cholesterol measurement	EFO_0004574	Lipid or lipoprotein measurement	Metabolic	PGS000061	EFO_0004611	low density lipoprotein cholesterol measurement	measurement	EFO_0004611	Cardiovascular measurement	Cardiovascular
0.8888	2.81E-156	1.04E-152	PGS000062	EFO_0004574	total cholesterol measurement	total cholesterol measurement	EFO_0004574	Lipid or lipoprotein measurement	Metabolic	PGS000061	EFO_0004611	low density lipoprotein cholesterol measurement	measurement	EFO_0004611	Lipid or lipoprotein measurement	Metabolic
0.8888	2.84E-156	1.05E-152	PGS001530	EFO_0007772	calcaneal bone quantitative ultrasound measurement	calcaneal bone quantitative ultrasound measurement	EFO_0007772	Other measurement	Skeletal	PGS000953	EFO_0004514	bone quantitative ultrasound measurement	measurement	EFO_0004514	Other measurement	Skeletal
0.8888	3.02E-156	1.12E-152	PGS000436	MONDO_0002898	skin cancer	skin cancer	MONDO_0002898	Cancer	Neoplasms	PGS000403	EFO_0009259	skin carcinoma	skin carcinoma	EFO_0009259	Cancer	Neoplasms
0.8887	3.18E-156	1.18E-152	PGS003310	EFO_0004617	cystatin C measurement|glomerular filtration rate	cystatin C measurement	EFO_0004617	Other measurement	Metabolic	PGS003309	EFO_0004617	cystatin C measurement|glomerular filtration rate	cystatin C measurement	EFO_0004617	Other measurement	Metabolic
0.8886	3.92E-156	1.45E-152	PGS002524	MONDO_0005148	type 2 diabetes mellitus	type 2 diabetes mellitus	MONDO_0005148	Digestive system disorder	Gastrointestinal	PGS002445	EFO_0001379	endocrine system disease|diabetes mellitus	endocrine system disease	EFO_0001379	Other disease	Metabolic
0.8886	3.92E-156	1.45E-152	PGS002524	MONDO_0005148	type 2 diabetes mellitus	type 2 diabetes mellitus	MONDO_0005148	Metabolic disorder	Metabolic	PGS002445	EFO_0001379	endocrine system disease|diabetes mellitus	endocrine system disease	EFO_0001379	Other disease	Metabolic
0.8886	3.97E-156	1.47E-152	PGS002409	EFO_0004611	low density lipoprotein cholesterol measurement	low density lipoprotein cholesterol measurement	EFO_0004611	Cardiovascular measurement	Cardiovascular	PGS002605	EFO_0004611	low density lipoprotein cholesterol measurement	measurement	EFO_0004611	Cardiovascular measurement	Cardiovascular
0.8886	3.97E-156	1.47E-152	PGS002409	EFO_0004611	low density lipoprotein cholesterol measurement	low density lipoprotein cholesterol measurement	EFO_0004611	Cardiovascular measurement	Cardiovascular	PGS002605	EFO_0004611	low density lipoprotein cholesterol measurement	measurement	EFO_0004611	Lipid or lipoprotein measurement	Metabolic
0.8886	3.97E-156	1.47E-152	PGS002409	EFO_0004611	low density lipoprotein cholesterol measurement	low density lipoprotein cholesterol measurement	EFO_0004611	Lipid or lipoprotein measurement	Metabolic	PGS002605	EFO_0004611	low density lipoprotein cholesterol measurement	measurement	EFO_0004611	Cardiovascular measurement	Cardiovascular
0.8886	3.97E-156	1.47E-152	PGS002409	EFO_0004611	low density lipoprotein cholesterol measurement	low density lipoprotein cholesterol measurement	EFO_0004611	Lipid or lipoprotein measurement	Metabolic	PGS002605	EFO_0004611	low density lipoprotein cholesterol measurement	measurement	EFO_0004611	Lipid or lipoprotein measurement	Metabolic
0.8886	4.09E-156	1.51E-152	PGS002108	EFO_0004574	total cholesterol measurement	total cholesterol measurement	EFO_0004574	Lipid or lipoprotein measurement	Metabolic	PGS001933	EFO_0004611	low density lipoprotein cholesterol measurement	measurement	EFO_0004611	Lipid or lipoprotein measurement	Metabolic
0.8886	4.09E-156	1.51E-152	PGS002108	EFO_0004574	total cholesterol measurement	total cholesterol measurement	EFO_0004574	Lipid or lipoprotein measurement	Metabolic	PGS001933	EFO_0004611	low density lipoprotein cholesterol measurement	measurement	EFO_0004611	Cardiovascular measurement	Cardiovascular
0.8886	4.30E-156	1.59E-152	PGS001993	EFO_0010729	sun exposure measurement	sun exposure measurement	EFO_0010729	Other measurement	Activities	PGS002210	EFO_0010729	sun exposure measurement	sun exposure measurement	EFO_0010729	Other measurement	Activities
0.8885	5.23E-156	1.93E-152	PGS000577	EFO_0001663	prostate carcinoma	prostate carcinoma	EFO_0001663	Cancer	Neoplasms	PGS002791	EFO_0001663	prostate carcinoma	prostate carcinoma	EFO_0001663	Cancer	Neoplasms
0.8885	5.23E-156	1.93E-152	PGS000578	EFO_0001663	prostate carcinoma	prostate carcinoma	EFO_0001663	Cancer	Neoplasms	PGS002791	EFO_0001663	prostate carcinoma	prostate carcinoma	EFO_0001663	Cancer	Neoplasms
0.8885	5.45E-156	2.01E-152	PGS003224	EFO_0005208	glomerular filtration rate	glomerular filtration rate	EFO_0005208	Other measurement	Genitourinary	PGS002757	EFO_0003884	chronic kidney disease	chronic kidney disease	EFO_0003884	Other disease	Metabolic
0.8884	5.57E-156	2.05E-152	PGS000603	EFO_0005088	testicular carcinoma	testicular carcinoma	EFO_0005088	Cancer	Neoplasms	PGS000604	EFO_0005088	testicular carcinoma	testicular carcinoma	EFO_0005088	Cancer	Neoplasms
0.8884	6.27E-156	2.31E-152	PGS000444	MONDO_0002898	skin cancer	skin cancer	MONDO_0002898	Cancer	Neoplasms	PGS000407	EFO_0009259	skin carcinoma	skin carcinoma	EFO_0009259	Cancer	Neoplasms
0.8882	9.02E-156	3.32E-152	PGS002524	MONDO_0005148	type 2 diabetes mellitus	type 2 diabetes mellitus	MONDO_0005148	Metabolic disorder	Metabolic	PGS002442	EFO_0000400	diabetes mellitus	diabetes mellitus	EFO_0000400	Digestive system disorder	Gastrointestinal
0.8882	9.02E-156	3.32E-152	PGS002524	MONDO_0005148	type 2 diabetes mellitus	type 2 diabetes mellitus	MONDO_0005148	Digestive system disorder	Gastrointestinal	PGS002442	EFO_0000400	diabetes mellitus	diabetes mellitus	EFO_0000400	Digestive system disorder	Gastrointestinal
0.8882	9.02E-156	3.32E-152	PGS002524	MONDO_0005148	type 2 diabetes mellitus	type 2 diabetes mellitus	MONDO_0005148	Metabolic disorder	Metabolic	PGS002442	EFO_0000400	diabetes mellitus	diabetes mellitus	EFO_0000400	Metabolic disorder	Metabolic
0.8882	9.02E-156	3.32E-152	PGS002524	MONDO_0005148	type 2 diabetes mellitus	type 2 diabetes mellitus	MONDO_0005148	Digestive system disorder	Gastrointestinal	PGS002442	EFO_0000400	diabetes mellitus	diabetes mellitus	EFO_0000400	Metabolic disorder	Metabolic
0.8882	9.33E-156	3.43E-152	PGS000530	EFO_0000305	breast carcinoma	breast carcinoma	EFO_0000305	Cancer	Neoplasms	PGS000529	EFO_0000305	breast carcinoma	breast carcinoma	EFO_0000305	Cancer	Neoplasms
0.8882	9.66E-156	3.55E-152	PGS001228	EFO_0004340	body mass index	body mass index	EFO_0004340	Body measurement	Body measurement	PGS001147	EFO_0005409	fat body mass	fat body mass	EFO_0005409	Other measurement	Body measurement
0.8881	1.03E-155	3.77E-152	PGS001739	EFO_0006930	brain volume measurement|neuroimaging measurement	brain volume measurement	EFO_0006930	Other measurement	Neurological	PGS001754	EFO_0006930	measurement	brain volume measurement	EFO_0006930	Other measurement	Neurological
0.8881	1.12E-155	4.10E-152	PGS002547	EFO_0004468	glucose measurement	glucose measurement	EFO_0004468	Other measurement	Metabolic	PGS002475	MONDO_0005148	type 2 diabetes mellitus	type 2 diabetes mellitus	MONDO_0005148	Digestive system disorder	Gastrointestinal
0.8881	1.12E-155	4.10E-152	PGS002547	EFO_0004468	glucose measurement	glucose measurement	EFO_0004468	Other measurement	Metabolic	PGS002475	MONDO_0005148	type 2 diabetes mellitus	type 2 diabetes mellitus	MONDO_0005148	Metabolic disorder	Metabolic
0.888	1.25E-155	4.58E-152	PGS002554	EFO_0000537	hypertension	hypertension	EFO_0000537	Cardiovascular disease	Cardiovascular	PGS002407	EFO_0000537	hypertension	hypertension	EFO_0000537	Cardiovascular disease	Cardiovascular
0.8879	1.55E-155	5.66E-152	PGS003201	EFO_0004631	vitamin D measurement	vitamin D measurement	EFO_0004631	Lipid or lipoprotein measurement	Metabolic	PGS003184	EFO_0004631	vitamin D measurement	vitamin D measurement	EFO_0004631	Lipid or lipoprotein measurement	Metabolic
0.8879	1.68E-155	6.13E-152	PGS003185	EFO_0004631	vitamin D measurement	vitamin D measurement	EFO_0004631	Lipid or lipoprotein measurement	Metabolic	PGS003184	EFO_0004631	vitamin D measurement	vitamin D measurement	EFO_0004631	Lipid or lipoprotein measurement	Metabolic
0.8878	1.96E-155	7.18E-152	PGS002343	EFO_0004309	platelet count	platelet count	EFO_0004309	Hematological measurement	Hematological	PGS002191	EFO_0004309	platelet count	platelet count	EFO_0004309	Hematological measurement	Hematological
0.8877	2.10E-155	7.67E-152	PGS000771	MONDO_0007915	systemic lupus erythematosus	systemic lupus erythematosus	MONDO_0007915	Immune system disorder	Immunological	PGS000772	MONDO_0007915	systemic lupus erythematosus	systemic lupus erythematosus	MONDO_0007915	Immune system disorder	Immunological
0.8877	2.28E-155	8.33E-152	PGS003199	EFO_0004631	vitamin D measurement	vitamin D measurement	EFO_0004631	Lipid or lipoprotein measurement	Metabolic	PGS003185	EFO_0004631	vitamin D measurement	vitamin D measurement	EFO_0004631	Lipid or lipoprotein measurement	Metabolic
0.8877	2.29E-155	8.34E-152	PGS002418	EFO_0005192	red blood cell distribution width	red blood cell distribution width	EFO_0005192	Hematological measurement	Hematological	PGS002467	EFO_0005192	red blood cell distribution width	red blood cell distribution width	EFO_0005192	Hematological measurement	Hematological
0.8877	2.52E-155	9.18E-152	PGS001751	EFO_0006930	brain volume measurement|neuroimaging measurement	brain volume measurement	EFO_0006930	Other measurement	Neurological	PGS001722	EFO_0006930	measurement	brain volume measurement	EFO_0006930	Other measurement	Neurological
0.8875	3.27E-155	1.19E-151	PGS003201	EFO_0004631	vitamin D measurement	vitamin D measurement	EFO_0004631	Lipid or lipoprotein measurement	Metabolic	PGS003199	EFO_0004631	vitamin D measurement	vitamin D measurement	EFO_0004631	Lipid or lipoprotein measurement	Metabolic
0.8873	5.28E-155	1.92E-151	PGS002086	MONDO_0002917	disorder of pilosebaceous unit	disorder of pilosebaceous unit	MONDO_0002917	Other disease	Dermatological	PGS001873	MONDO_0002917	disorder of pilosebaceous unit	disorder of pilosebaceous unit	MONDO_0002917	Other disease	Dermatological
0.8872	6.47E-155	2.35E-151	PGS002851	EFO_0004340	body mass index	body mass index	EFO_0004340	Body measurement	Body measurement	PGS002852	EFO_0004340	body mass index	body mass index	EFO_0004340	Body measurement	Body measurement
0.8871	6.75E-155	2.45E-151	PGS002244	EFO_0001645	coronary artery disease	coronary artery disease	EFO_0001645	Cardiovascular disease	Cardiovascular	PGS000013	EFO_0001645	coronary artery disease	coronary artery disease	EFO_0001645	Cardiovascular disease	Cardiovascular
0.8871	6.78E-155	2.46E-151	PGS002856	EFO_0004340	body mass index	body mass index	EFO_0004340	Body measurement	Body measurement	PGS002841	EFO_0004340	body mass index	body mass index	EFO_0004340	Body measurement	Body measurement
0.887	8.10E-155	2.94E-151	PGS002393	EFO_0000400	diabetes mellitus	diabetes mellitus	EFO_0000400	Digestive system disorder	Gastrointestinal	PGS002596	EFO_0004468	glucose measurement	glucose measurement	EFO_0004468	Other measurement	Metabolic
0.887	8.10E-155	2.94E-151	PGS002393	EFO_0000400	diabetes mellitus	diabetes mellitus	EFO_0000400	Metabolic disorder	Metabolic	PGS002596	EFO_0004468	glucose measurement	glucose measurement	EFO_0004468	Other measurement	Metabolic
0.8869	1.02E-154	3.71E-151	PGS001367	EFO_0004731	eye measurement	eye measurement	EFO_0004731	Other measurement	Ophthalmological	PGS001362	EFO_0004731	eye measurement	eye measurement	EFO_0004731	Other measurement	Ophthalmological
0.8868	1.17E-154	4.23E-151	PGS001344	MONDO_0004979	asthma	asthma	MONDO_0004979	Other disease	Respiratory	PGS001341	MONDO_0004979	asthma	asthma	MONDO_0004979	Other disease	Respiratory
0.8868	1.21E-154	4.39E-151	PGS000442	MONDO_0002898	skin cancer	skin cancer	MONDO_0002898	Cancer	Neoplasms	PGS000405	EFO_0009259	skin carcinoma	skin carcinoma	EFO_0009259	Cancer	Neoplasms
0.8867	1.40E-154	5.05E-151	PGS000579	EFO_0001663	prostate carcinoma	prostate carcinoma	EFO_0001663	Cancer	Neoplasms	PGS000719	EFO_0001663	prostate carcinoma	prostate carcinoma	EFO_0001663	Cancer	Neoplasms
-0.8867	1.67E-154	6.04E-151	PGS003312	EFO_0004617	cystatin C measurement|glomerular filtration rate	cystatin C measurement	EFO_0004617	Other measurement	Metabolic	PGS003304	EFO_0004617	cystatin C measurement|glomerular filtration rate	cystatin C measurement	EFO_0004617	Other measurement	Metabolic
0.8867	1.68E-154	6.05E-151	PGS002445	EFO_0001379	endocrine system disease|diabetes mellitus	endocrine system disease	EFO_0001379	Other disease	Metabolic	PGS002589	EFO_0000400	diabetes mellitus	diabetes mellitus	EFO_0000400	Digestive system disorder	Gastrointestinal
0.8867	1.68E-154	6.05E-151	PGS002445	EFO_0001379	endocrine system disease|diabetes mellitus	endocrine system disease	EFO_0001379	Other disease	Metabolic	PGS002589	EFO_0000400	diabetes mellitus	diabetes mellitus	EFO_0000400	Metabolic disorder	Metabolic
0.8866	1.75E-154	6.31E-151	PGS002174	EFO_0007772	calcaneal bone quantitative ultrasound measurement	calcaneal bone quantitative ultrasound measurement	EFO_0007772	Other measurement	Skeletal	PGS002175	EFO_0005654	velocity of sound measurement	velocity of sound measurement	EFO_0005654	Other measurement	Others

-0.8866	1.76E-154	6.34E-151	PGS003297	EFO_0004617	cystatin C measurement|glomerular filtration rate	cystatin C measurement	EFO_0004617	Other measurement	Metabolic	PGS003304	EFO_0004617	cystatin C measurement|glomerular filtration rate	cystatin C measurement	EFO_0004617	Other measurement	Metabolic
0.8865	2.04E-154	7.36E-151	PGS002427	EFO_0004631	vitamin D measurement	vitamin D measurement	EFO_0004631	Lipid or lipoprotein measurement	Metabolic	PGS003196	EFO_0004631	vitamin D measurement	vitamin D measurement	EFO_0004631	Lipid or lipoprotein measurement	Metabolic
0.8865	2.09E-154	7.51E-151	PGS002403	EFO_0004541	HbA1c measurement	HbA1c measurement	EFO_0004541	Hematological measurement	Hematological	PGS002589	EFO_0000400	diabetes mellitus	diabetes mellitus	EFO_0000400	Metabolic disorder	Metabolic
0.8865	2.09E-154	7.51E-151	PGS002403	EFO_0004541	HbA1c measurement	HbA1c measurement	EFO_0004541	Hematological measurement	Hematological	PGS002589	EFO_0000400	diabetes mellitus	diabetes mellitus	EFO_0000400	Digestive system disorder	Gastrointestinal
0.8865	2.23E-154	8.02E-151	PGS002353	EFO_0004530	triglyceride measurement	triglyceride measurement	EFO_0004530	Lipid or lipoprotein measurement	Metabolic	PGS002197	EFO_0004530	triglyceride measurement	triglyceride measurement	EFO_0004530	Lipid or lipoprotein measurement	Metabolic
0.8864	2.50E-154	8.99E-151	PGS001681	EFO_0006930	brain volume measurement|neuroimaging measurement	brain volume measurement	EFO_0006930	Other measurement	Neurological	PGS001680	EFO_0006930	measurement	brain volume measurement	EFO_0006930	Other measurement	Neurological
0.8863	3.45E-154	1.24E-150	PGS002338	EFO_0004587	lymphocyte count	lymphocyte count	EFO_0004587	Hematological measurement	Hematological	PGS001965	EFO_0004587	lymphocyte count	lymphocyte count	EFO_0004587	Hematological measurement	Hematological
0.8862	3.84E-154	1.38E-150	PGS002160	EFO_0004570	bilirubin measurement	bilirubin measurement	EFO_0004570	Other measurement	Metabolic	PGS000924	MONDO_0037821	disease	porphyrin metabolism disease	MONDO_0037821	Metabolic disorder	Metabolic
0.8862	4.00E-154	1.43E-150	PGS003186	EFO_0004631	vitamin D measurement	vitamin D measurement	EFO_0004631	Lipid or lipoprotein measurement	Metabolic	PGS003184	EFO_0004631	vitamin D measurement	vitamin D measurement	EFO_0004631	Lipid or lipoprotein measurement	Metabolic
0.886	6.24E-154	2.23E-150	PGS003186	EFO_0004631	vitamin D measurement	vitamin D measurement	EFO_0004631	Lipid or lipoprotein measurement	Metabolic	PGS003199	EFO_0004631	vitamin D measurement	vitamin D measurement	EFO_0004631	Lipid or lipoprotein measurement	Metabolic
0.8859	7.24E-154	2.59E-150	PGS003228	EFO_0005208	glomerular filtration rate	glomerular filtration rate	EFO_0005208	Other measurement	Genitourinary	PGS003224	EFO_0005208	glomerular filtration rate	glomerular filtration rate	EFO_0005208	Other measurement	Genitourinary
0.8858	8.40E-154	3.01E-150	PGS002011	EFO_0004315	drinking behavior	drinking behavior	EFO_0004315	Biological process	Psychiatric	PGS002230	EFO_0004315	drinking behavior	drinking behavior	EFO_0004315	Biological process	Psychiatric
0.8857	1.03E-153	3.67E-150	PGS003118	MONDO_0005148	type 2 diabetes mellitus	type 2 diabetes mellitus	MONDO_0005148	Digestive system disorder	Gastrointestinal	PGS003099	MONDO_0005148	type 2 diabetes mellitus	type 2 diabetes mellitus	MONDO_0005148	Digestive system disorder	Gastrointestinal
0.8857	1.03E-153	3.67E-150	PGS003118	MONDO_0005148	type 2 diabetes mellitus	type 2 diabetes mellitus	MONDO_0005148	Digestive system disorder	Gastrointestinal	PGS003099	MONDO_0005148	type 2 diabetes mellitus	type 2 diabetes mellitus	MONDO_0005148	Metabolic disorder	Metabolic
0.8857	1.03E-153	3.67E-150	PGS003118	MONDO_0005148	type 2 diabetes mellitus	type 2 diabetes mellitus	MONDO_0005148	Metabolic disorder	Metabolic	PGS003099	MONDO_0005148	type 2 diabetes mellitus	type 2 diabetes mellitus	MONDO_0005148	Digestive system disorder	Gastrointestinal
0.8857	1.03E-153	3.67E-150	PGS003118	MONDO_0005148	type 2 diabetes mellitus	type 2 diabetes mellitus	MONDO_0005148	Metabolic disorder	Metabolic	PGS003099	MONDO_0005148	type 2 diabetes mellitus	type 2 diabetes mellitus	MONDO_0005148	Metabolic disorder	Metabolic
0.8857	1.05E-153	3.75E-150	PGS001154	EFO_0005106	body composition measurement	body composition measurement	EFO_0005106	Body measurement	Body measurement	PGS001161	EFO_0005106	body composition measurement	body composition measurement	EFO_0005106	Body measurement	Body measurement
0.8856	1.18E-153	4.22E-150	PGS002445	EFO_0001379	endocrine system disease|diabetes mellitus	endocrine system disease	EFO_0001379	Other disease	Metabolic	PGS002400	EFO_0004468	glucose measurement	glucose measurement	EFO_0004468	Other measurement	Metabolic
0.8855	1.38E-153	4.92E-150	PGS000482	EFO_0000305	breast carcinoma	breast carcinoma	EFO_0000305	Cancer	Neoplasms	PGS000513	EFO_0000305	breast carcinoma	breast carcinoma	EFO_0000305	Cancer	Neoplasms
0.8854	1.63E-153	5.81E-150	PGS002150	EFO_0004611	low density lipoprotein cholesterol measurement	low density lipoprotein cholesterol measurement	EFO_0004611	Cardiovascular measurement	Cardiovascular	PGS001895	EFO_0004574	total cholesterol measurement	total cholesterol measurement	EFO_0004574	Lipid or lipoprotein measurement	Metabolic
0.8854	1.63E-153	5.81E-150	PGS002150	EFO_0004611	low density lipoprotein cholesterol measurement	low density lipoprotein cholesterol measurement	EFO_0004611	Lipid or lipoprotein measurement	Metabolic	PGS001895	EFO_0004574	total cholesterol measurement	total cholesterol measurement	EFO_0004574	Lipid or lipoprotein measurement	Metabolic
0.8852	2.36E-153	8.40E-150	PGS003218	EFO_0003877	sleep apnea	sleep apnea	EFO_0003877	Neurological disorder	Neurological	PGS003208	EFO_0003877	sleep apnea	sleep apnea	EFO_0003877	Neurological disorder	Neurological
-0.8852	2.51E-153	8.93E-150	PGS001371	EFO_0004918	age at diagnosis|diabetes mellitus	age at diagnosis	EFO_0004918	Other measurement	Metabolic	PGS002028	HP_0001943	Hypoglycemia	Hypoglycemia	HP_0001943	Other trait	Metabolic
0.8852	2.60E-153	9.25E-150	PGS002583	EFO_0003923	bone density	bone density	EFO_0003923	Other measurement	Skeletal	PGS002387	EFO_0003923	bone density	bone density	EFO_0003923	Other measurement	Skeletal
0.8852	2.63E-153	9.34E-150	PGS000326	EFO_1002020	negative	negative	EFO_1002020	Immune system disorder	Immunological	PGS000325	EFO_1002019	oligoarticular juvenile idiopathic arthritis	oligoarticular juvenile idiopathic arthritis	EFO_1002019	Immune system disorder	Immunological
0.8852	2.80E-153	9.93E-150	PGS000112	EFO_0007986	reticulocyte count	reticulocyte count	EFO_0007986	Hematological measurement	Hematological	PGS000111	EFO_0007986	reticulocyte count	reticulocyte count	EFO_0007986	Hematological measurement	Hematological
0.8851	2.80E-153	9.93E-150	PGS002619	EFO_1000627	thyroid disease	thyroid disease	EFO_1000627	Other disease	Endocrine	PGS002570	EFO_1000627	thyroid disease	thyroid disease	EFO_1000627	Other disease	Endocrine
0.8851	2.99E-153	1.06E-149	PGS000530	EFO_0000305	breast carcinoma	breast carcinoma	EFO_0000305	Cancer	Neoplasms	PGS000526	EFO_0000305	breast carcinoma	breast carcinoma	EFO_0000305	Cancer	Neoplasms
0.8851	2.99E-153	1.06E-149	PGS000525	EFO_0000305	breast carcinoma	breast carcinoma	EFO_0000305	Cancer	Neoplasms	PGS000530	EFO_0000305	breast carcinoma	breast carcinoma	EFO_0000305	Cancer	Neoplasms
0.8851	3.23E-153	1.14E-149	PGS001363	EFO_0004731	eye measurement	eye measurement	EFO_0004731	Other measurement	Ophthalmological	PGS001368	EFO_0004731	eye measurement	eye measurement	EFO_0004731	Other measurement	Ophthalmological
0.8848	5.24E-153	1.85E-149	PGS003313	EFO_0004617	cystatin C measurement|glomerular filtration rate	cystatin C measurement	EFO_0004617	Other measurement	Metabolic	PGS003311	EFO_0004617	cystatin C measurement|glomerular filtration rate	cystatin C measurement	EFO_0004617	Other measurement	Metabolic
0.8848	5.49E-153	1.94E-149	PGS003023	EFO_0000537	hypertension	hypertension	EFO_0000537	Cardiovascular disease	Cardiovascular	PGS003028	EFO_0000537	hypertension	hypertension	EFO_0000537	Cardiovascular disease	Cardiovascular
0.8847	6.49E-153	2.29E-149	PGS003224	EFO_0005208	glomerular filtration rate	glomerular filtration rate	EFO_0005208	Other measurement	Genitourinary	PGS003223	EFO_0005208	glomerular filtration rate	glomerular filtration rate	EFO_0005208	Other measurement	Genitourinary
0.8847	6.95E-153	2.45E-149	PGS000955	EFO_0004514	bone quantitative ultrasound measurement	bone quantitative ultrasound measurement	EFO_0004514	Other measurement	Skeletal	PGS001531	EFO_0007772	measurement	measurement	EFO_0007772	Other measurement	Skeletal
0.8846	7.86E-153	2.77E-149	PGS002042	MONDO_0018102	corneal dystrophy	corneal dystrophy	MONDO_0018102	Other disease	Ophthalmological	PGS001835	MONDO_0018102	corneal dystrophy	corneal dystrophy	MONDO_0018102	Other disease	Ophthalmological
0.8845	8.53E-153	3.01E-149	PGS001104	EFO_0007800	body fat percentage	body fat percentage	EFO_0007800	Body measurement	Body measurement	PGS001101	EFO_0007800	body fat percentage	body fat percentage	EFO_0007800	Body measurement	Body measurement
0.8844	1.04E-152	3.67E-149	PGS000913	EFO_0006335	systolic blood pressure	systolic blood pressure	EFO_0006335	Other measurement	Cardiovascular	PGS002807	EFO_0006335	systolic blood pressure	systolic blood pressure	EFO_0006335	Other measurement	Cardiovascular
0.8844	1.05E-152	3.70E-149	PGS001242	EFO_0004302	anthropometric measurement	anthropometric measurement	EFO_0004302	Other measurement	Body measurement	PGS001243	EFO_0004302	anthropometric measurement	anthropometric measurement	EFO_0004302	Other measurement	Body measurement
0.8844	1.17E-152	4.10E-149	PGS000433	MONDO_0002898	skin cancer	skin cancer	MONDO_0002898	Cancer	Neoplasms	PGS000400	EFO_0009259	skin carcinoma	skin carcinoma	EFO_0009259	Cancer	Neoplasms
0.8844	1.17E-152	4.12E-149	PGS001149	EFO_0005409	fat body mass	fat body mass	EFO_0005409	Other measurement	Body measurement	PGS001230	EFO_0004338	body weight	body weight	EFO_0004338	Body measurement	Body measurement
0.8843	1.36E-152	4.78E-149	PGS000040	EFO_0001060	celiac disease	celiac disease	EFO_0001060	Immune system disorder	Immunological	PGS002067	EFO_0001060	celiac disease	celiac disease	EFO_0001060	Immune system disorder	Immunological
0.8843	1.36E-152	4.78E-149	PGS000040	EFO_0001060	celiac disease	celiac disease	EFO_0001060	Digestive system disorder	Gastrointestinal	PGS002067	EFO_0001060	celiac disease	celiac disease	EFO_0001060	Digestive system disorder	Gastrointestinal
0.8843	1.36E-152	4.78E-149	PGS000040	EFO_0001060	celiac disease	celiac disease	EFO_0001060	Immune system disorder	Immunological	PGS002067	EFO_0001060	celiac disease	celiac disease	EFO_0001060	Digestive system disorder	Gastrointestinal
0.8843	1.36E-152	4.78E-149	PGS000040	EFO_0001060	celiac disease	celiac disease	EFO_0001060	Digestive system disorder	Gastrointestinal	PGS002067	EFO_0001060	celiac disease	celiac disease	EFO_0001060	Immune system disorder	Immunological
0.8843	1.42E-152	5.00E-149	PGS000774	EFO_1000649	estrogen-receptor positive breast cancer	estrogen-receptor positive breast cancer	EFO_1000649	Cancer	Neoplasms	PGS000873	EFO_0000305	breast carcinoma	breast carcinoma	EFO_0000305	Cancer	Neoplasms
0.8842	1.77E-152	6.21E-149	PGS003191	EFO_0004631	vitamin D measurement	vitamin D measurement	EFO_0004631	Lipid or lipoprotein measurement	Metabolic	PGS002574	EFO_0004631	vitamin D measurement	vitamin D measurement	EFO_0004631	Lipid or lipoprotein measurement	Metabolic
0.8839	2.90E-152	1.02E-148	PGS001359	EFO_0004731	eye measurement	eye measurement	EFO_0004731	Other measurement	Ophthalmological	PGS001362	EFO_0004731	eye measurement	eye measurement	EFO_0004731	Other measurement	Ophthalmological
0.8838	3.37E-152	1.18E-148	PGS002672	EFO_0004631	vitamin D measurement	vitamin D measurement	EFO_0004631	Lipid or lipoprotein measurement	Metabolic	PGS002355	EFO_0004631	vitamin D measurement	vitamin D measurement	EFO_0004631	Lipid or lipoprotein measurement	Metabolic
0.8838	3.59E-152	1.26E-148	PGS001359	EFO_0004731	eye measurement	eye measurement	EFO_0004731	Other measurement	Ophthalmological	PGS001365	EFO_0004731	eye measurement	eye measurement	EFO_0004731	Other measurement	Ophthalmological
0.8837	3.91E-152	1.37E-148	PGS002342	EFO_0007660	neuroticism measurement	neuroticism measurement	EFO_0007660	Other measurement	Psychiatric	PGS002213	EFO_0007660	neuroticism measurement	neuroticism measurement	EFO_0007660	Other measurement	Psychiatric
0.8837	3.93E-152	1.37E-148	PGS000013	EFO_0001645	coronary artery disease	coronary artery disease	EFO_0001645	Cardiovascular disease	Cardiovascular	PGS001355	EFO_0001645	coronary artery disease	coronary artery disease	EFO_0001645	Cardiovascular disease	Cardiovascular
0.8837	4.32E-152	1.51E-148	PGS001857	HP_0002253	Colonic diverticula	Colonic diverticula	HP_0002253	Other trait	Gastrointestinal	PGS002068	HP_0002253	Colonic diverticula	Colonic diverticula	HP_0002253	Other trait	Gastrointestinal
0.8837	4.44E-152	1.55E-148	PGS000344	EFO_0000305	breast carcinoma	breast carcinoma	EFO_0000305	Cancer	Neoplasms	PGS000212	MONDO_0021116	luminal A breast carcinoma	luminal A breast carcinoma	MONDO_0021116	Cancer	Neoplasms
0.8836	4.75E-152	1.66E-148	PGS002547	EFO_0004468	glucose measurement	glucose measurement	EFO_0004468	Other measurement	Metabolic	PGS002449	EFO_0004468	glucose measurement	glucose measurement	EFO_0004468	Other measurement	Metabolic
0.8835	6.04E-152	2.10E-148	PGS002429	EFO_0004308	leukocyte count	leukocyte count	EFO_0004308	Hematological measurement	Hematological	PGS002625	EFO_0004308	leukocyte count	leukocyte count	EFO_0004308	Hematological measurement	Hematological
0.8835	6.09E-152	2.12E-148	PGS002200	EFO_0004631	vitamin D measurement	vitamin D measurement	EFO_0004631	Lipid or lipoprotein measurement	Metabolic	PGS002721	EFO_0004631	vitamin D measurement	vitamin D measurement	EFO_0004631	Lipid or lipoprotein measurement	Metabolic
0.8835	6.44E-152	2.24E-148	PGS002998	EFO_0000537	hypertension	hypertension	EFO_0000537	Cardiovascular disease	Cardiovascular	PGS002335	EFO_0000537	hypertension	hypertension	EFO_0000537	Cardiovascular disease	Cardiovascular
0.8834	6.73E-152	2.34E-148	PGS000741	EFO_0001663	prostate carcinoma	prostate carcinoma	EFO_0001663	Cancer	Neoplasms	PGS000733	EFO_0001663	prostate carcinoma	prostate carcinoma	EFO_0001663	Cancer	Neoplasms
0.8832	9.47E-152	3.29E-148	PGS002547	EFO_0004468	glucose measurement	glucose measurement	EFO_0004468	Other measurement	Metabolic	PGS002442	EFO_0000400	diabetes mellitus	diabetes mellitus	EFO_0000400	Digestive system disorder	Gastrointestinal
0.8832	9.47E-152	3.29E-148	PGS002547	EFO_0004468	glucose measurement	glucose measurement	EFO_0004468	Other measurement	Metabolic	PGS002442	EFO_0000400	diabetes mellitus	diabetes mellitus	EFO_0000400	Metabolic disorder	Metabolic
0.8832	9.92E-152	3.44E-148	PGS002590	EFO_0006336	diastolic blood pressure	diastolic blood pressure	EFO_0006336	Other measurement	Cardiovascular	PGS002394	EFO_0006336	diastolic blood pressure	diastolic blood pressure	EFO_0006336	Other measurement	Cardiovascular
0.8831	1.12E-151	3.89E-148	PGS000511	EFO_0000305	breast carcinoma	breast carcinoma	EFO_0000305	Cancer	Neoplasms	PGS000503	EFO_0000305	breast carcinoma	breast carcinoma	EFO_0000305	Cancer	Neoplasms
0.8831	1.26E-151	4.37E-148	PGS001297	MONDO_0005147	type 1 diabetes mellitus	type 1 diabetes mellitus	MONDO_0005147	Metabolic disorder	Metabolic	PGS002028	HP_0001943	Hypoglycemia	Hypoglycemia	HP_0001943	Other trait	Metabolic
0.8831	1.26E-151	4.37E-148	PGS001297	MONDO_0005147	type 1 diabetes mellitus	type 1 diabetes mellitus	MONDO_0005147	Immune system disorder	Immunological	PGS002028	HP_0001943	Hypoglycemia	Hypoglycemia	HP_0001943	Other trait	Metabolic
0.8831	1.26E-151	4.37E-148	PGS001297	MONDO_0005147	type 1 diabetes mellitus	type 1 diabetes mellitus	MONDO_0005147	Digestive system disorder	Gastrointestinal	PGS002028	HP_0001943	Hypoglycemia	Hypoglycemia	HP_0001943	Other trait	Metabolic
0.8829	1.71E-151	5.92E-148	PGS000988	EFO_0010700	reticulocyte measurement	reticulocyte measurement	EFO_0010700	Hematological measurement	Hematological	PGS001406	EFO_0010700	reticulocyte measurement	reticulocyte measurement	EFO_0010700	Hematological measurement	Hematological
0.8829	1.81E-151	6.25E-148	PGS000468	EFO_0000707	squamous cell carcinoma	squamous cell carcinoma	EFO_0000707	Cancer	Neoplasms	PGS000461	EFO_0000707	squamous cell carcinoma	squamous cell carcinoma	EFO_0000707	Cancer	Neoplasms
0.8829	1.81E-151	6.25E-148	PGS000468	EFO_0000707	squamous cell carcinoma	squamous cell carcinoma	EFO_0000707	Cancer	Neoplasms	PGS000464	EFO_0000707	squamous cell carcinoma	squamous cell carcinoma	EFO_0000707	Cancer	Neoplasms
0.8829	1.81E-151	6.25E-148	PGS000468	EFO_0000707	squamous cell carcinoma	squamous cell carcinoma	EFO_0000707	Cancer	Neoplasms	PGS000462	EFO_0000707	squamous cell carcinoma	squamous cell carcinoma	EFO_0000707	Cancer	Neoplasms
0.8827	2.41E-151	8.31E-148	PGS000176	EFO_0004526	mean corpuscular volume	mean corpuscular volume	EFO_0004526	Hematological measurement	Hematological	PGS000174	EFO_0004527	mean corpuscular hemoglobin	mean corpuscular hemoglobin	EFO_0004527	Hematological measurement	Hematological
0.8826	3.18E-151	1.10E-147	PGS000613	MONDO_0004986	urinary bladder carcinoma	urinary bladder carcinoma	MONDO_0004986	Cancer	Neoplasms	PGS000782	MONDO_0004986	urinary bladder carcinoma	urinary bladder carcinoma	MONDO_0004986	Cancer	Neoplasms
0.8824	4.24E-151	1.46E-147	PGS001149	EFO_0005409	fat body mass	fat body mass	EFO_0005409	Other measurement	Body measurement	PGS001101	EFO_0007800	body fat percentage	body fat percentage	EFO_0007800	Body measurement	Body measurement
0.8823	5.21E-151	1.79E-147	PGS000441	MONDO_0002898	skin cancer	skin cancer	MONDO_0002898	Cancer	Neoplasms	PGS000404	EFO_0009259	skin carcinoma	skin carcinoma	EFO_0009259	Cancer	Neoplasms
-0.8823	5.32E-151	1.83E-147	PGS002768	EFO_0003882	osteoporosis	osteoporosis	EFO_0003882	Other disease	Skeletal	PGS002315	EFO_0003923	bone density	bone density	EFO_0003923	Other measurement	Skeletal
0.8823	5.61E-151	1.93E-147	PGS000873	EFO_0000305	breast carcinoma	breast carcinoma	EFO_0000305	Cancer	Neoplasms	PGS000052	EFO_0000305	breast carcinoma	breast carcinoma	EFO_0000305	Cancer	Neoplasms
0.8822	6.13E-151	2.11E-147	PGS001978	EFO_0005271	sleep duration	sleep duration	EFO_0005271	Other measurement	Psychiatric	PGS002196	EFO_0005271	sleep duration	sleep duration	EFO_0005271	Other measurement	Psychiatric
0.8822	6.17E-151	2.12E-147	PGS002107	EFO_0001060	celiac disease	celiac disease	EFO_0001060	Digestive system disorder	Gastrointestinal	PGS001856	EFO_0001060	celiac disease	celiac disease	EFO_0001060	Digestive system disorder	Gastrointestinal
0.8822	6.17E-151	2.12E-147	PGS002107	EFO_0001060	celiac disease	celiac disease	EFO_0001060	Immune system disorder	Immunological	PGS001856	EFO_0001060	celiac disease	celiac disease	EFO_0001060	Immune system disorder	Immunological
0.8822	6.17E-151	2.12E-147	PGS002107	EFO_0001060	celiac disease	celiac disease	EFO_0001060	Immune system disorder	Immunological	PGS001856	EFO_0001060	celiac disease	celiac disease	EFO_0001060	Digestive system disorder	Gastrointestinal
0.8822	6.17E-151	2.12E-147	PGS002107	EFO_0001060	celiac disease	celiac disease	EFO_0001060	Digestive system disorder	Gastrointestinal	PGS001856	EFO_0001060	celiac disease	celiac disease	EFO_0001060	Immune system disorder	Immunological
0.8821	7.90E-151	2.71E-147	PGS003008	EFO_0000537	hypertension	hypertension	EFO_0000537	Cardiovascular disease	Cardiovascular	PGS003013	EFO_0000537	hypertension	hypertension	EFO_0000537	Cardiovascular disease	Cardiovascular
0.882	8.82E-151	3.02E-147	PGS002574	EFO_0004631	vitamin D measurement	vitamin D measurement	EFO_0004631	Lipid or lipoprotein measurement	Metabolic	PGS003195	EFO_0004631	vitamin D measurement	vitamin D measurement	EFO_0004631	Lipid or lipoprotein measurement	Metabolic
0.882	9.01E-151	3.09E-147	PGS002101	EFO_0004614	apolipoprotein A 1 measurement	apolipoprotein A 1 measurement	EFO_0004614	Lipid or lipoprotein measurement	Metabolic	PGS002172	EFO_0004612	high density lipoprotein cholesterol measurement	measurement	EFO_0004612	Lipid or lipoprotein measurement	Metabolic
0.882	9.01E-151	3.09E-147	PGS002101	EFO_0004614	apolipoprotein A 1 measurement	apolipoprotein A 1 measurement	EFO_0004614	Cardiovascular measurement	Cardiovascular	PGS002172	EFO_0004612	high density lipoprotein cholesterol measurement	measurement	EFO_0004612	Lipid or lipoprotein measurement	Metabolic
0.882	9.01E-151	3.09E-147	PGS002101	EFO_0004614	apolipoprotein A 1 measurement	apolipoprotein A 1 measurement	EFO_0004614	Inflammatory measurement	Immunological	PGS002172	EFO_0004612	high density lipoprotein cholesterol measurement	measurement	EFO_0004612	Lipid or lipoprotein measurement	Metabolic
0.8818	1.32E-150	4.52E-147	PGS002354	MONDO_0005148	type 2 diabetes mellitus	type 2 diabetes mellitus	MONDO_0005148	Metabolic disorder	Metabolic	PGS002324	EFO_0001379	endocrine system disease|diabetes mellitus	endocrine system disease	EFO_0001379	Other disease	Metabolic
0.8818	1.32E-150	4.52E-147	PGS002354	MONDO_0005148	type 2 diabetes mellitus	type 2 diabetes mellitus	MONDO_0005148	Digestive system disorder	Gastrointestinal	PGS002324	EFO_0001379	endocrine system disease|diabetes mellitus	endocrine system disease	EFO_0001379	Other disease	Metabolic
0.8818	1.33E-150	4.55E-147	PGS002844	EFO_0004340	body mass index	body mass index	EFO_0004340	Body measurement	Body measurement	PGS002313	EFO_0004340	body mass index	body mass index	EFO_0004340	Body measurement	Body measurement
0.8815	2.26E-150	7.72E-147	PGS002589	EFO_0000400	diabetes mellitus	diabetes mellitus	EFO_0000400	Digestive system disorder	Gastrointestinal	PGS002596	EFO_0004468	glucose measurement	glucose measurement	EFO_0004468	Other measurement	Metabolic
0.8815	2.26E-150	7.72E-147	PGS002589	EFO_0000400	diabetes mellitus	diabetes mellitus	EFO_0000400	Metabolic disorder	Metabolic	PGS002596	EFO_0004468	glucose measurement	glucose measurement	EFO_0004468	Other measurement	Metabolic
0.8814	2.53E-150	8.64E-147	PGS001955	EFO_0009270	heel bone mineral density	heel bone mineral density	EFO_0009270	Other measurement	Skeletal	PGS002315	EFO_0003923	bone density	bone density	EFO_0003923	Other measurement	Skeletal
0.8814	2.86E-150	9.77E-147	PGS002547	EFO_0004468	glucose measurement	glucose measurement	EFO_0004468	Other measurement	Metabolic	PGS002452	EFO_0004541	HbA1c measurement	HbA1c measurement	EFO_0004541	Hematological measurement	Hematological
0.8813	3.40E-150	1.16E-146	PGS000525	EFO_0000305	breast carcinoma	breast carcinoma	EFO_0000305	Cancer	Neoplasms	PGS000538	EFO_0000305	breast carcinoma	breast carcinoma	EFO_0000305	Cancer	Neoplasms
0.8813	3.40E-150	1.16E-146	PGS000538	EFO_0000305	breast carcinoma	breast carcinoma	EFO_0000305	Cancer	Neoplasms	PGS000526	EFO_0000305	breast carcinoma	breast carcinoma	EFO_0000305	Cancer	Neoplasms
0.8812	3.70E-150	1.26E-146	PGS000454	EFO_0004193	basal cell carcinoma	basal cell carcinoma	EFO_0004193	Cancer	Neoplasms	PGS000459	EFO_0004193	basal cell carcinoma	basal cell carcinoma	EFO_0004193	Cancer	Neoplasms
0.8811	4.25E-150	1.45E-146	PGS002445	EFO_0001379	endocrine system disease|diabetes mellitus	endocrine system disease	EFO_0001379	Other disease	Metabolic	PGS002403	EFO_0004541	HbA1c measurement	HbA1c measurement	EFO_0004541	Hematological measurement	Hematological
0.8811	4.58E-150	1.56E-146	PGS001856	EFO_0001060	celiac disease	celiac disease	EFO_0001060	Digestive system disorder	Gastrointestinal	PGS000040	EFO_0001060	celiac disease	celiac disease	EFO_0001060	Digestive system disorder	Gastrointestinal
0.8811	4.58E-150	1.56E-146	PGS001856	EFO_0001060	celiac disease	celiac disease	EFO_0001060	Immune system disorder	Immunological	PGS000040	EFO_0001060	celiac disease	celiac disease	EFO_0001060	Digestive system disorder	Gastrointestinal
0.8811	4.58E-150	1.56E-146	PGS001856	EFO_0001060	celiac disease	celiac disease	EFO_0001060	Immune system disorder	Immunological	PGS000040	EFO_0001060	celiac disease	celiac disease	EFO_0001060	Immune system disorder	Immunological
0.8811	4.58E-150	1.56E-146	PGS001856	EFO_0001060	celiac disease	celiac disease	EFO_0001060	Digestive system disorder	Gastrointestinal	PGS000040	EFO_0001060	celiac disease	celiac disease	EFO_0001060	Immune system disorder	Immunological
0.881	5.34E-150	1.82E-146	PGS001876	EFO_0003898	ankylosing spondylitis	ankylosing spondylitis	EFO_0003898	Immune system disorder	Immunological	PGS002089	EFO_0003898	ankylosing spondylitis	ankylosing spondylitis	EFO_0003898	Immune system disorder	Immunological
0.881	5.85E-150	1.99E-146	PGS003103	MONDO_0005148	type 2 diabetes mellitus	type 2 diabetes mellitus	MONDO_0005148	Metabolic disorder	Metabolic	PGS003099	MONDO_0005148	type 2 diabetes mellitus	type 2 diabetes mellitus	MONDO_0005148	Digestive system disorder	Gastrointestinal
0.881	5.85E-150	1.99E-146	PGS003103	MONDO_0005148	type 2 diabetes mellitus	type 2 diabetes mellitus	MONDO_0005148	Digestive system disorder	Gastrointestinal	PGS003099	MONDO_0005148	type 2 diabetes mellitus	type 2 diabetes mellitus	MONDO_0005148	Digestive system disorder	Gastrointestinal

0.881	5.85E-150	1.99E-146	PGS003103	MONDO_0005148	type 2 diabetes mellitus	type 2 diabetes mellitus	MONDO_0005148	Metabolic disorder	Metabolic	PGS003099	MONDO_0005148	type 2 diabetes mellitus	type 2 diabetes mellitus	MONDO_0005148	Metabolic disorder	Metabolic
0.881	5.85E-150	1.99E-146	PGS003103	MONDO_0005148	type 2 diabetes mellitus	type 2 diabetes mellitus	MONDO_0005148	Digestive system disorder	Gastrointestinal	PGS003099	MONDO_0005148	type 2 diabetes mellitus	type 2 diabetes mellitus	MONDO_0005148	Metabolic disorder	Metabolic
0.881	5.96E-150	2.02E-146	PGS001368	EFO_0004731	eye measurement	eye measurement	EFO_0004731	Other measurement	Ophthalmological	PGS001367	EFO_0004731	eye measurement	eye measurement	EFO_0004731	Other measurement	Ophthalmological
0.8809	6.51E-150	2.21E-146	PGS001749	EFO_0006930	brain volume measurement|neuroimaging measurement	brain volume measurement	EFO_0006930	Other measurement	Neurological	PGS001734	EFO_0006930	measurement	brain volume measurement	EFO_0006930	Other measurement	Neurological
0.8809	6.98E-150	2.37E-146	PGS002623	EFO_0004631	vitamin D measurement	vitamin D measurement	EFO_0004631	Lipid or lipoprotein measurement	Metabolic	PGS003191	EFO_0004631	vitamin D measurement	vitamin D measurement	EFO_0004631	Lipid or lipoprotein measurement	Metabolic
0.8808	7.18E-150	2.43E-146	PGS003219	EFO_0005208	glomerular filtration rate	glomerular filtration rate	EFO_0005208	Other measurement	Genitourinary	PGS002757	EFO_0003884	chronic kidney disease	chronic kidney disease	EFO_0003884	Other disease	Metabolic
0.8808	8.26E-150	2.80E-146	PGS003073	EFO_0006335	systolic blood pressure	systolic blood pressure	EFO_0006335	Other measurement	Cardiovascular	PGS002807	EFO_0006335	systolic blood pressure	systolic blood pressure	EFO_0006335	Other measurement	Cardiovascular
-0.8808	8.42E-150	2.85E-146	PGS001077	EFO_0007990	neutrophil percentage of leukocytes	neutrophil percentage of leukocytes	EFO_0007990	Hematological measurement	Hematological	PGS001414	EFO_0007993	lymphocyte percentage of leukocytes	lymphocyte percentage of leukocytes	EFO_0007993	Hematological measurement	Hematological
0.8807	9.69E-150	3.28E-146	PGS001332	EFO_0000341	chronic obstructive pulmonary disease	chronic obstructive pulmonary disease	EFO_0000341	Other disease	Respiratory	PGS001334	EFO_0000341	chronic obstructive pulmonary disease	chronic obstructive pulmonary disease	EFO_0000341	Other disease	Respiratory
0.8805	1.31E-149	4.41E-146	PGS000648	EFO_0000095	chronic lymphocytic leukemia	chronic lymphocytic leukemia	EFO_0000095	Cancer	Neoplasms	PGS000647	EFO_0000095	chronic lymphocytic leukemia	chronic lymphocytic leukemia	EFO_0000095	Immune system disorder	Immunological
0.8805	1.31E-149	4.41E-146	PGS000648	EFO_0000095	chronic lymphocytic leukemia	chronic lymphocytic leukemia	EFO_0000095	Immune system disorder	Immunological	PGS000647	EFO_0000095	chronic lymphocytic leukemia	chronic lymphocytic leukemia	EFO_0000095	Cancer	Neoplasms
0.8805	1.31E-149	4.41E-146	PGS000648	EFO_0000095	chronic lymphocytic leukemia	chronic lymphocytic leukemia	EFO_0000095	Cancer	Neoplasms	PGS000646	EFO_0000095	chronic lymphocytic leukemia	chronic lymphocytic leukemia	EFO_0000095	Immune system disorder	Immunological
0.8805	1.31E-149	4.41E-146	PGS000648	EFO_0000095	chronic lymphocytic leukemia	chronic lymphocytic leukemia	EFO_0000095	Cancer	Neoplasms	PGS000646	EFO_0000095	chronic lymphocytic leukemia	chronic lymphocytic leukemia	EFO_0000095	Cancer	Neoplasms
0.8805	1.31E-149	4.41E-146	PGS000648	EFO_0000095	chronic lymphocytic leukemia	chronic lymphocytic leukemia	EFO_0000095	Immune system disorder	Immunological	PGS000646	EFO_0000095	chronic lymphocytic leukemia	chronic lymphocytic leukemia	EFO_0000095	Cancer	Neoplasms
0.8805	1.31E-149	4.41E-146	PGS000648	EFO_0000095	chronic lymphocytic leukemia	chronic lymphocytic leukemia	EFO_0000095	Immune system disorder	Immunological	PGS000646	EFO_0000095	chronic lymphocytic leukemia	chronic lymphocytic leukemia	EFO_0000095	Immune system disorder	Immunological
0.8805	1.31E-149	4.41E-146	PGS000648	EFO_0000095	chronic lymphocytic leukemia	chronic lymphocytic leukemia	EFO_0000095	Cancer	Neoplasms	PGS000647	EFO_0000095	chronic lymphocytic leukemia	chronic lymphocytic leukemia	EFO_0000095	Cancer	Neoplasms
0.8805	1.31E-149	4.41E-146	PGS000648	EFO_0000095	chronic lymphocytic leukemia	chronic lymphocytic leukemia	EFO_0000095	Immune system disorder	Immunological	PGS000647	EFO_0000095	chronic lymphocytic leukemia	chronic lymphocytic leukemia	EFO_0000095	Immune system disorder	Immunological
0.8804	1.66E-149	5.61E-146	PGS000309	EFO_0004612	high density lipoprotein cholesterol measurement	high density lipoprotein cholesterol measurement	EFO_0004612	Lipid or lipoprotein measurement	Metabolic	PGS000064	EFO_0004612	high density lipoprotein cholesterol measurement	measurement	EFO_0004612	Lipid or lipoprotein measurement	Metabolic
0.8802	2.14E-149	7.23E-146	PGS000795	EFO_0001663	prostate carcinoma	prostate carcinoma	EFO_0001663	Cancer	Neoplasms	PGS000742	EFO_0001663	prostate carcinoma	prostate carcinoma	EFO_0001663	Cancer	Neoplasms
0.8802	2.15E-149	7.23E-146	PGS000551	EFO_0003893	ovarian neoplasm	ovarian neoplasm	EFO_0003893	Cancer	Neoplasms	PGS000549	EFO_0003893	ovarian neoplasm	ovarian neoplasm	EFO_0003893	Cancer	Neoplasms
0.8801	2.53E-149	8.51E-146	PGS001942	EFO_0004570	bilirubin measurement	bilirubin measurement	EFO_0004570	Other measurement	Metabolic	PGS000924	MONDO_0037821	disease	porphyrin metabolism disease	MONDO_0037821	Metabolic disorder	Metabolic
0.8799	3.75E-149	1.26E-145	PGS001230	EFO_0004338	body weight	body weight	EFO_0004338	Body measurement	Body measurement	PGS001158	EFO_0005106	body composition measurement	body composition measurement	EFO_0005106	Body measurement	Body measurement
-0.8799	4.07E-149	1.37E-145	PGS002203	EFO_0007993	lymphocyte percentage of leukocytes	lymphocyte percentage of leukocytes	EFO_0007993	Hematological measurement	Hematological	PGS002214	EFO_0007990	neutrophil percentage of leukocytes	neutrophil percentage of leukocytes	EFO_0007990	Hematological measurement	Hematological
0.8799	4.19E-149	1.41E-145	PGS002623	EFO_0004631	vitamin D measurement	vitamin D measurement	EFO_0004631	Lipid or lipoprotein measurement	Metabolic	PGS003195	EFO_0004631	vitamin D measurement	vitamin D measurement	EFO_0004631	Lipid or lipoprotein measurement	Metabolic
0.8795	8.15E-149	2.73E-145	PGS002622	MONDO_0005148	type 2 diabetes mellitus	type 2 diabetes mellitus	MONDO_0005148	Digestive system disorder	Gastrointestinal	PGS002400	EFO_0004468	glucose measurement	glucose measurement	EFO_0004468	Other measurement	Metabolic
0.8795	8.15E-149	2.73E-145	PGS002622	MONDO_0005148	type 2 diabetes mellitus	type 2 diabetes mellitus	MONDO_0005148	Metabolic disorder	Metabolic	PGS002400	EFO_0004468	glucose measurement	glucose measurement	EFO_0004468	Other measurement	Metabolic
0.8795	8.24E-149	2.76E-145	PGS001874	EFO_1000243	Epidermal Inclusion Cyst	Epidermal Inclusion Cyst	EFO_1000243	Cancer	Neoplasms	PGS002087	EFO_1000243	Epidermal Inclusion Cyst	Epidermal Inclusion Cyst	EFO_1000243	Cancer	Neoplasms
0.8795	8.59E-149	2.88E-145	PGS002624	EFO_0004343	waist-hip ratio	waist-hip ratio	EFO_0004343	Body measurement	Body measurement	PGS002428	EFO_0004343	waist-hip ratio	waist-hip ratio	EFO_0004343	Body measurement	Body measurement
0.8794	9.99E-149	3.35E-145	PGS001230	EFO_0004338	body weight	body weight	EFO_0004338	Body measurement	Body measurement	PGS001166	EFO_0004995	lean body mass	lean body mass	EFO_0004995	Other measurement	Skeletal
0.8793	1.09E-148	3.66E-145	PGS000502	EFO_0000305	breast carcinoma	breast carcinoma	EFO_0000305	Cancer	Neoplasms	PGS000497	EFO_0000305	breast carcinoma	breast carcinoma	EFO_0000305	Cancer	Neoplasms
0.8793	1.09E-148	3.66E-145	PGS000502	EFO_0000305	breast carcinoma	breast carcinoma	EFO_0000305	Cancer	Neoplasms	PGS000498	EFO_0000305	breast carcinoma	breast carcinoma	EFO_0000305	Cancer	Neoplasms
0.8792	1.27E-148	4.23E-145	PGS002856	EFO_0004340	body mass index	body mass index	EFO_0004340	Body measurement	Body measurement	PGS002857	EFO_0004340	body mass index	body mass index	EFO_0004340	Body measurement	Body measurement
0.8791	1.56E-148	5.22E-145	PGS000893	EFO_0004611	low density lipoprotein cholesterol measurement	low density lipoprotein cholesterol measurement	EFO_0004611	Lipid or lipoprotein measurement	Metabolic	PGS000889	EFO_0004611	low density lipoprotein cholesterol measurement	measurement	EFO_0004611	Lipid or lipoprotein measurement	Metabolic
0.8791	1.56E-148	5.22E-145	PGS000893	EFO_0004611	low density lipoprotein cholesterol measurement	low density lipoprotein cholesterol measurement	EFO_0004611	Cardiovascular measurement	Cardiovascular	PGS000889	EFO_0004611	low density lipoprotein cholesterol measurement	measurement	EFO_0004611	Lipid or lipoprotein measurement	Metabolic
0.8791	1.56E-148	5.22E-145	PGS000893	EFO_0004611	low density lipoprotein cholesterol measurement	low density lipoprotein cholesterol measurement	EFO_0004611	Lipid or lipoprotein measurement	Metabolic	PGS000889	EFO_0004611	low density lipoprotein cholesterol measurement	measurement	EFO_0004611	Cardiovascular measurement	Cardiovascular
0.8791	1.56E-148	5.22E-145	PGS000893	EFO_0004611	low density lipoprotein cholesterol measurement	low density lipoprotein cholesterol measurement	EFO_0004611	Cardiovascular measurement	Cardiovascular	PGS000889	EFO_0004611	low density lipoprotein cholesterol measurement	measurement	EFO_0004611	Cardiovascular measurement	Cardiovascular
0.8789	2.20E-148	7.35E-145	PGS001230	EFO_0004338	body weight	body weight	EFO_0004338	Body measurement	Body measurement	PGS001108	EFO_0007777	base metabolic rate measurement	base metabolic rate measurement	EFO_0007777	Other measurement	Metabolic
0.8789	2.45E-148	8.16E-145	PGS002891	EFO_0006336	diastolic blood pressure	diastolic blood pressure	EFO_0006336	Other measurement	Cardiovascular	PGS002896	EFO_0006336	diastolic blood pressure	diastolic blood pressure	EFO_0006336	Other measurement	Cardiovascular
0.8789	2.46E-148	8.19E-145	PGS001894	EFO_0001060	celiac disease	celiac disease	EFO_0001060	Immune system disorder	Immunological	PGS002067	EFO_0001060	celiac disease	celiac disease	EFO_0001060	Digestive system disorder	Gastrointestinal
0.8789	2.46E-148	8.19E-145	PGS001894	EFO_0001060	celiac disease	celiac disease	EFO_0001060	Digestive system disorder	Gastrointestinal	PGS002067	EFO_0001060	celiac disease	celiac disease	EFO_0001060	Immune system disorder	Immunological
0.8789	2.46E-148	8.19E-145	PGS001894	EFO_0001060	celiac disease	celiac disease	EFO_0001060	Digestive system disorder	Gastrointestinal	PGS002067	EFO_0001060	celiac disease	celiac disease	EFO_0001060	Digestive system disorder	Gastrointestinal
0.8789	2.46E-148	8.19E-145	PGS001894	EFO_0001060	celiac disease	celiac disease	EFO_0001060	Immune system disorder	Immunological	PGS002067	EFO_0001060	celiac disease	celiac disease	EFO_0001060	Immune system disorder	Immunological
0.8786	3.98E-148	1.32E-144	PGS000441	MONDO_0002898	skin cancer	skin cancer	MONDO_0002898	Cancer	Neoplasms	PGS000443	MONDO_0002898	skin cancer	skin cancer	MONDO_0002898	Cancer	Neoplasms
0.8785	4.60E-148	1.53E-144	PGS002502	EFO_0004339	body height	body height	EFO_0004339	Body measurement	Body measurement	PGS002453	EFO_0004339	body height	body height	EFO_0004339	Body measurement	Body measurement
0.8785	4.88E-148	1.62E-144	PGS001749	EFO_0006930	brain volume measurement|neuroimaging measurement	brain volume measurement	EFO_0006930	Other measurement	Neurological	PGS001720	EFO_0006930	measurement	brain volume measurement	EFO_0006930	Other measurement	Neurological
0.8784	5.69E-148	1.89E-144	PGS000569	EFO_0001663	prostate carcinoma	prostate carcinoma	EFO_0001663	Cancer	Neoplasms	PGS000593	EFO_0007355	male reproductive organ cancer	male reproductive organ cancer	EFO_0007355	Cancer	Neoplasms
0.8783	6.11E-148	2.03E-144	PGS002329	EFO_0004612	high density lipoprotein cholesterol measurement	high density lipoprotein cholesterol measurement	EFO_0004612	Lipid or lipoprotein measurement	Metabolic	PGS002172	EFO_0004612	high density lipoprotein cholesterol measurement	measurement	EFO_0004612	Lipid or lipoprotein measurement	Metabolic
0.8782	7.72E-148	2.56E-144	PGS001404	EFO_0009270	heel bone mineral density	heel bone mineral density	EFO_0009270	Other measurement	Skeletal	PGS001403	EFO_0009270	heel bone mineral density	heel bone mineral density	EFO_0009270	Other measurement	Skeletal
0.8781	8.80E-148	2.92E-144	PGS002546	EFO_0004312	vital capacity	vital capacity	EFO_0004312	Other measurement	Activities	PGS002399	EFO_0004312	vital capacity	vital capacity	EFO_0004312	Other measurement	Activities
0.878	1.03E-147	3.42E-144	PGS002854	EFO_0004340	body mass index	body mass index	EFO_0004340	Body measurement	Body measurement	PGS002313	EFO_0004340	body mass index	body mass index	EFO_0004340	Body measurement	Body measurement
0.878	1.03E-147	3.42E-144	PGS002531	EFO_0005140	autoimmune disease	autoimmune disease	EFO_0005140	Immune system disorder	Immunological	PGS002619	EFO_1000627	thyroid disease	thyroid disease	EFO_1000627	Other disease	Endocrine
0.878	1.18E-147	3.91E-144	PGS002393	EFO_0000400	diabetes mellitus	diabetes mellitus	EFO_0000400	Metabolic disorder	Metabolic	PGS002452	EFO_0004541	HbA1c measurement	HbA1c measurement	EFO_0004541	Hematological measurement	Hematological
0.878	1.18E-147	3.91E-144	PGS002393	EFO_0000400	diabetes mellitus	diabetes mellitus	EFO_0000400	Digestive system disorder	Gastrointestinal	PGS002452	EFO_0004541	HbA1c measurement	HbA1c measurement	EFO_0004541	Hematological measurement	Hematological
0.8778	1.59E-147	5.26E-144	PGS002218	EFO_0007652	health trait	health trait	EFO_0007652	Other trait	Others	PGS002000	EFO_0007652	health trait	health trait	EFO_0007652	Other trait	Others
0.8778	1.65E-147	5.47E-144	PGS002335	EFO_0000537	hypertension	hypertension	EFO_0000537	Cardiovascular disease	Cardiovascular	PGS002316	EFO_0000319	cardiovascular disease	cardiovascular disease	EFO_0000319	Cardiovascular disease	Cardiovascular
0.8775	2.81E-147	9.29E-144	PGS001728	EFO_0006930	brain volume measurement|neuroimaging measurement	brain volume measurement	EFO_0006930	Other measurement	Neurological	PGS001743	EFO_0006930	measurement	brain volume measurement	EFO_0006930	Other measurement	Neurological
0.8774	3.15E-147	1.04E-143	PGS000847	EFO_0004530	triglyceride measurement	triglyceride measurement	EFO_0004530	Lipid or lipoprotein measurement	Metabolic	PGS000826	EFO_0004530	triglyceride measurement	triglyceride measurement	EFO_0004530	Lipid or lipoprotein measurement	Metabolic
0.8773	3.79E-147	1.25E-143	PGS000452	EFO_0004193	basal cell carcinoma	basal cell carcinoma	EFO_0004193	Cancer	Neoplasms	PGS000459	EFO_0004193	basal cell carcinoma	basal cell carcinoma	EFO_0004193	Cancer	Neoplasms
0.8773	3.79E-147	1.25E-143	PGS000455	EFO_0004193	basal cell carcinoma	basal cell carcinoma	EFO_0004193	Cancer	Neoplasms	PGS000459	EFO_0004193	basal cell carcinoma	basal cell carcinoma	EFO_0004193	Cancer	Neoplasms
0.8773	3.79E-147	1.25E-143	PGS000453	EFO_0004193	basal cell carcinoma	basal cell carcinoma	EFO_0004193	Cancer	Neoplasms	PGS000459	EFO_0004193	basal cell carcinoma	basal cell carcinoma	EFO_0004193	Cancer	Neoplasms
0.8773	3.80E-147	1.25E-143	PGS003228	EFO_0005208	glomerular filtration rate	glomerular filtration rate	EFO_0005208	Other measurement	Genitourinary	PGS003219	EFO_0005208	glomerular filtration rate	glomerular filtration rate	EFO_0005208	Other measurement	Genitourinary
0.8772	4.26E-147	1.40E-143	PGS002426	MONDO_0005148	type 2 diabetes mellitus	type 2 diabetes mellitus	MONDO_0005148	Metabolic disorder	Metabolic	PGS002452	EFO_0004541	HbA1c measurement	HbA1c measurement	EFO_0004541	Hematological measurement	Hematological
0.8772	4.26E-147	1.40E-143	PGS002426	MONDO_0005148	type 2 diabetes mellitus	type 2 diabetes mellitus	MONDO_0005148	Digestive system disorder	Gastrointestinal	PGS002452	EFO_0004541	HbA1c measurement	HbA1c measurement	EFO_0004541	Hematological measurement	Hematological
0.8771	5.09E-147	1.67E-143	PGS003071	EFO_0006335	systolic blood pressure	systolic blood pressure	EFO_0006335	Other measurement	Cardiovascular	PGS003076	EFO_0006335	systolic blood pressure	systolic blood pressure	EFO_0006335	Other measurement	Cardiovascular
0.8769	7.15E-147	2.35E-143	PGS002200	EFO_0004631	vitamin D measurement	vitamin D measurement	EFO_0004631	Lipid or lipoprotein measurement	Metabolic	PGS002355	EFO_0004631	vitamin D measurement	vitamin D measurement	EFO_0004631	Lipid or lipoprotein measurement	Metabolic
0.8769	7.30E-147	2.40E-143	PGS000344	EFO_0000305	breast carcinoma	breast carcinoma	EFO_0000305	Cancer	Neoplasms	PGS000004	EFO_0000305	breast carcinoma	breast carcinoma	EFO_0000305	Cancer	Neoplasms
0.8768	9.46E-147	3.10E-143	PGS002445	EFO_0001379	endocrine system disease|diabetes mellitus	endocrine system disease	EFO_0001379	Other disease	Metabolic	PGS002596	EFO_0004468	glucose measurement	glucose measurement	EFO_0004468	Other measurement	Metabolic
0.8767	1.00E-146	3.29E-143	PGS000536	EFO_0000305	breast carcinoma	breast carcinoma	EFO_0000305	Cancer	Neoplasms	PGS000527	EFO_0000305	breast carcinoma	breast carcinoma	EFO_0000305	Cancer	Neoplasms
0.8766	1.31E-146	4.28E-143	PGS001929	EFO_0004339	body height	body height	EFO_0004339	Body measurement	Body measurement	PGS002968	EFO_0004339	body height	body height	EFO_0004339	Body measurement	Body measurement
0.8765	1.54E-146	5.05E-143	PGS001343	MONDO_0004979	asthma	asthma	MONDO_0004979	Other disease	Respiratory	PGS001344	MONDO_0004979	asthma	asthma	MONDO_0004979	Other disease	Respiratory
0.8763	2.15E-146	7.02E-143	PGS001472	EFO_0006930	brain volume measurement|neuroimaging measurement	brain volume measurement	EFO_0006930	Other measurement	Neurological	PGS001473	EFO_0006930	measurement	brain volume measurement	EFO_0006930	Other measurement	Neurological
0.8763	2.25E-146	7.36E-143	PGS002333	EFO_0007986	reticulocyte count	reticulocyte count	EFO_0007986	Hematological measurement	Hematological	PGS001959	EFO_0010700	reticulocyte measurement	reticulocyte measurement	EFO_0010700	Hematological measurement	Hematological
0.8759	4.28E-146	1.40E-142	PGS003219	EFO_0005208	glomerular filtration rate	glomerular filtration rate	EFO_0005208	Other measurement	Genitourinary	PGS003223	EFO_0005208	glomerular filtration rate	glomerular filtration rate	EFO_0005208	Other measurement	Genitourinary
0.8756	7.15E-146	2.33E-142	PGS002337	EFO_0004611	low density lipoprotein cholesterol measurement	low density lipoprotein cholesterol measurement	EFO_0004611	Lipid or lipoprotein measurement	Metabolic	PGS001889	EFO_0004615	apolipoprotein B measurement	apolipoprotein B measurement	EFO_0004615	Lipid or lipoprotein measurement	Metabolic
0.8756	7.15E-146	2.33E-142	PGS002337	EFO_0004611	low density lipoprotein cholesterol measurement	low density lipoprotein cholesterol measurement	EFO_0004611	Cardiovascular measurement	Cardiovascular	PGS001889	EFO_0004615	apolipoprotein B measurement	apolipoprotein B measurement	EFO_0004615	Lipid or lipoprotein measurement	Metabolic
0.8755	8.18E-146	2.67E-142	PGS000572	EFO_0001663	prostate carcinoma	prostate carcinoma	EFO_0001663	Cancer	Neoplasms	PGS000576	EFO_0001663	prostate carcinoma	prostate carcinoma	EFO_0001663	Cancer	Neoplasms
0.8755	8.65E-146	2.82E-142	PGS002475	MONDO_0005148	type 2 diabetes mellitus	type 2 diabetes mellitus	MONDO_0005148	Digestive system disorder	Gastrointestinal	PGS002400	EFO_0004468	glucose measurement	glucose measurement	EFO_0004468	Other measurement	Metabolic
0.8755	8.65E-146	2.82E-142	PGS002475	MONDO_0005148	type 2 diabetes mellitus	type 2 diabetes mellitus	MONDO_0005148	Metabolic disorder	Metabolic	PGS002400	EFO_0004468	glucose measurement	glucose measurement	EFO_0004468	Other measurement	Metabolic
0.8754	8.99E-146	2.93E-142	PGS002073	EFO_0009534	biliary tract disease	biliary tract disease	EFO_0009534	Digestive system disorder	Gastrointestinal	PGS001862	EFO_0009534	biliary tract disease	biliary tract disease	EFO_0009534	Digestive system disorder	Gastrointestinal
0.8754	1.02E-145	3.31E-142	PGS000454	EFO_0004193	basal cell carcinoma	basal cell carcinoma	EFO_0004193	Cancer	Neoplasms	PGS000458	EFO_0004193	basal cell carcinoma	basal cell carcinoma	EFO_0004193	Cancer	Neoplasms
0.8754	1.06E-145	3.44E-142	PGS001929	EFO_0004339	body height	body height	EFO_0004339	Body measurement	Body measurement	PGS002973	EFO_0004339	body height	body height	EFO_0004339	Body measurement	Body measurement
-0.875	1.81E-145	5.89E-142	PGS001092	EFO_0007822	hair colour measurement	hair colour measurement	EFO_0007822	Other measurement	Dermatological	PGS001096	EFO_0007822	hair colour measurement	hair colour measurement	EFO_0007822	Other measurement	Dermatological
0.8747	3.33E-145	1.08E-141	PGS001929	EFO_0004339	body height	body height	EFO_0004339	Body measurement	Body measurement	PGS000758	EFO_0004339	body height	body height	EFO_0004339	Body measurement	Body measurement
0.8747	3.36E-145	1.09E-141	PGS001244	EFO_0004279	suntan	suntan	EFO_0004279	Other trait	Dermatological	PGS001247	EFO_0004279	suntan	suntan	EFO_0004279	Other trait	Dermatological
0.8746	3.89E-145	1.26E-141	PGS000212	MONDO_0021116	luminal A breast carcinoma	luminal A breast carcinoma	MONDO_0021116	Cancer	Neoplasms	PGS000214	MONDO_0021115	luminal B breast carcinoma	luminal B breast carcinoma	MONDO_0021115	Cancer	Neoplasms
0.8745	4.88E-145	1.58E-141	PGS001876	EFO_0003898	ankylosing spondylitis	ankylosing spondylitis	EFO_0003898	Immune system disorder	Immunological	PGS000324	EFO_0009732	enthesitis-related juvenile idiopathic arthritis	arthritis	EFO_0009732	Immune system disorder	Immunological
0.8744	5.17E-145	1.67E-141	PGS001672	EFO_0006930	brain volume measurement|neuroimaging measurement	brain volume measurement	EFO_0006930	Other measurement	Neurological	PGS001673	EFO_0006930	measurement	brain volume measurement	EFO_0006930	Other measurement	Neurological
0.8744	5.20E-145	1.68E-141	PGS002842	EFO_0004340	body mass index	body mass index	EFO_0004340	Body measurement	Body measurement	PGS002857	EFO_0004340	body mass index	body mass index	EFO_0004340	Body measurement	Body measurement
0.8744	5.41E-145	1.75E-141	PGS001531	EFO_0007772	calcaneal bone quantitative ultrasound measurement	calcaneal bone quantitative ultrasound measurement	EFO_0007772	Other measurement	Skeletal	PGS001530	EFO_0007772	measurement	measurement	EFO_0007772	Other measurement	Skeletal
0.8744	5.42E-145	1.75E-141	PGS002989	EFO_0004339	body height	body height	EFO_0004339	Body measurement	Body measurement	PGS002983	EFO_0004339	body height	body height	EFO_0004339	Body measurement	Body measurement
-0.8743	6.19E-145	2.00E-141	PGS003304	EFO_0004617	cystatin C measurement|glomerular filtration rate	cystatin C measurement	EFO_0004617	Other measurement	Metabolic	PGS003314	EFO_0004617	cystatin C measurement|glomerular filtration rate	cystatin C measurement	EFO_0004617	Other measurement	Metabolic
0.8742	7.84E-145	2.53E-141	PGS000534	EFO_0000305	breast carcinoma	breast carcinoma	EFO_0000305	Cancer	Neoplasms	PGS000526	EFO_0000305	breast carcinoma	breast carcinoma	EFO_0000305	Cancer	Neoplasms
0.8742	7.84E-145	2.53E-141	PGS000534	EFO_0000305	breast carcinoma	breast carcinoma	EFO_0000305	Cancer	Neoplasms	PGS000525	EFO_0000305	breast carcinoma	breast carcinoma	EFO_0000305	Cancer	Neoplasms
0.8742	7.84E-145	2.53E-141	PGS000533	EFO_0000305	breast carcinoma	breast carcinoma	EFO_0000305	Cancer	Neoplasms	PGS000526	EFO_0000305	breast carcinoma	breast carcinoma	EFO_0000305	Cancer	Neoplasms
0.8742	7.84E-145	2.53E-141	PGS000525	EFO_0000305	breast carcinoma	breast carcinoma	EFO_0000305	Cancer	Neoplasms	PGS000533	EFO_0000305	breast carcinoma	breast carcinoma	EFO_0000305	Cancer	Neoplasms
0.8742	8.14E-145	2.62E-141	PGS003080	EFO_0006335	systolic blood pressure	systolic blood pressure	EFO_0006335	Other measurement	Cardiovascular	PGS003085	EFO_0006335	systolic blood pressure	systolic blood pressure	EFO_0006335	Other measurement	Cardiovascular
0.874	9.89E-145	3.18E-141	PGS001166	EFO_0004995	lean body mass	lean body mass	EFO_0004995	Other measurement	Skeletal	PGS001235	EFO_0004324	body weights and measures	body weights and measures	EFO_0004324	Body measurement	Body measurement
0.874	9.96E-145	3.20E-141	PGS002452	EFO_0004541	HbA1c measurement	HbA1c measurement	EFO_0004541	Hematological measurement	Hematological	PGS002573	MONDO_0005148	type 2 diabetes mellitus	type 2 diabetes mellitus	MONDO_0005148	Metabolic disorder	Metabolic
0.874	9.96E-145	3.20E-141	PGS002452	EFO_0004541	HbA1c measurement	HbA1c measurement	EFO_0004541	Hematological measurement	Hematological	PGS002573	MONDO_0005148	type 2 diabetes mellitus	type 2 diabetes mellitus	MONDO_0005148	Digestive system disorder	Gastrointestinal
0.874	1.03E-144	3.30E-141	PGS002409	EFO_0004611	low density lipoprotein cholesterol measurement	low density lipoprotein cholesterol measurement	EFO_0004611	Cardiovascular measurement	Cardiovascular	PGS002571	EFO_0004574	total cholesterol measurement	total cholesterol measurement	EFO_0004574	Lipid or lipoprotein measurement	Metabolic
0.874	1.03E-144	3.30E-141	PGS002409	EFO_0004611	low density lipoprotein cholesterol measurement	low density lipoprotein cholesterol measurement	EFO_0004611	Lipid or lipoprotein measurement	Metabolic	PGS002571	EFO_0004574	total cholesterol measurement	total cholesterol measurement	EFO_0004574	Lipid or lipoprotein measurement	Metabolic
0.8738	1.50E-144	4.82E-141	PGS002126	EFO_0006527	smoking status measurement	smoking status measurement	EFO_0006527	Other measurement	Psychiatric	PGS003058	EFO_0006527	smoking status measurement	smoking status measurement	EFO_0006527	Other measurement	Psychiatric
0.8738	1.50E-144	4.82E-141	PGS001923	EFO_0010724	lifestyle measurement	lifestyle measurement	EFO_0010724	Other measurement	Activities	PGS002139	EFO_0010724	lifestyle measurement	lifestyle measurement	EFO_0010724	Other measurement	Activities

0.8738	1.58E-144	5.07E-141	PGS001297	MONDO_0005147	type 1 diabetes mellitus	type 1 diabetes mellitus	MONDO_0005147	Immune system disorder	Immunological	PGS001820	HP_0001943	Hypoglycemia	Hypoglycemia	HP_0001943	Other trait	Metabolic
0.8738	1.58E-144	5.07E-141	PGS001297	MONDO_0005147	type 1 diabetes mellitus	type 1 diabetes mellitus	MONDO_0005147	Digestive system disorder	Gastrointestinal	PGS001820	HP_0001943	Hypoglycemia	Hypoglycemia	HP_0001943	Other trait	Metabolic
0.8738	1.58E-144	5.07E-141	PGS001297	MONDO_0005147	type 1 diabetes mellitus	type 1 diabetes mellitus	MONDO_0005147	Metabolic disorder	Metabolic	PGS001820	HP_0001943	Hypoglycemia	Hypoglycemia	HP_0001943	Other trait	Metabolic
0.8737	1.67E-144	5.37E-141	PGS001039	EFO_0009270	heel bone mineral density	heel bone mineral density	EFO_0009270	Other measurement	Skeletal	PGS001403	EFO_0009270	heel bone mineral density	heel bone mineral density	EFO_0009270	Other measurement	Skeletal
0.8737	1.68E-144	5.37E-141	PGS000956	EFO_0004514	bone quantitative ultrasound measurement	bone quantitative ultrasound measurement	EFO_0004514	Other measurement	Skeletal	PGS001403	EFO_0009270	heel bone mineral density	heel bone mineral density	EFO_0009270	Other measurement	Skeletal
0.8737	1.86E-144	5.96E-141	PGS001158	EFO_0005106	body composition measurement	body composition measurement	EFO_0005106	Body measurement	Body measurement	PGS001235	EFO_0004324	body weights and measures	body weights and measures	EFO_0004324	Body measurement	Body measurement
0.8735	2.24E-144	7.17E-141	PGS002161	EFO_0004340	body mass index	body mass index	EFO_0004340	Body measurement	Body measurement	PGS002854	EFO_0004340	body mass index	body mass index	EFO_0004340	Body measurement	Body measurement
0.8735	2.25E-144	7.19E-141	PGS001750	EFO_0006930	brain volume measurement|neuroimaging measurement	brain volume measurement	EFO_0006930	Other measurement	Neurological	PGS001721	EFO_0006930	measurement	brain volume measurement	EFO_0006930	Other measurement	Neurological
0.8735	2.28E-144	7.28E-141	PGS002427	EFO_0004631	vitamin D measurement	vitamin D measurement	EFO_0004631	Lipid or lipoprotein measurement	Metabolic	PGS003191	EFO_0004631	vitamin D measurement	vitamin D measurement	EFO_0004631	Lipid or lipoprotein measurement	Metabolic
0.8735	2.61E-144	8.34E-141	PGS003078	EFO_0006335	systolic blood pressure	systolic blood pressure	EFO_0006335	Other measurement	Cardiovascular	PGS002807	EFO_0006335	systolic blood pressure	systolic blood pressure	EFO_0006335	Other measurement	Cardiovascular
0.8734	2.75E-144	8.79E-141	PGS002604	EFO_0004705	hypothyroidism	hypothyroidism	EFO_0004705	Other disease	Endocrine	PGS002580	EFO_0005140	autoimmune disease	autoimmune disease	EFO_0005140	Immune system disorder	Immunological
0.8734	3.10E-144	9.90E-141	PGS002403	EFO_0004541	HbA1c measurement	HbA1c measurement	EFO_0004541	Hematological measurement	Hematological	PGS002475	MONDO_0005148	type 2 diabetes mellitus	type 2 diabetes mellitus	MONDO_0005148	Metabolic disorder	Metabolic
0.8734	3.10E-144	9.90E-141	PGS002403	EFO_0004541	HbA1c measurement	HbA1c measurement	EFO_0004541	Hematological measurement	Hematological	PGS002475	MONDO_0005148	type 2 diabetes mellitus	type 2 diabetes mellitus	MONDO_0005148	Digestive system disorder	Gastrointestinal
0.8733	3.24E-144	1.03E-140	PGS002452	EFO_0004541	HbA1c measurement	HbA1c measurement	EFO_0004541	Hematological measurement	Hematological	PGS002596	EFO_0004468	glucose measurement	glucose measurement	EFO_0004468	Other measurement	Metabolic
0.8733	3.31E-144	1.05E-140	PGS002557	EFO_0004587	lymphocyte count	lymphocyte count	EFO_0004587	Hematological measurement	Hematological	PGS002459	EFO_0004587	lymphocyte count	lymphocyte count	EFO_0004587	Hematological measurement	Hematological
0.8733	3.52E-144	1.12E-140	PGS001404	EFO_0009270	heel bone mineral density	heel bone mineral density	EFO_0009270	Other measurement	Skeletal	PGS000954	EFO_0004514	bone quantitative ultrasound measurement	measurement	EFO_0004514	Other measurement	Skeletal
0.8733	3.65E-144	1.16E-140	PGS001404	EFO_0009270	heel bone mineral density	heel bone mineral density	EFO_0009270	Other measurement	Skeletal	PGS001038	EFO_0009270	heel bone mineral density	heel bone mineral density	EFO_0009270	Other measurement	Skeletal
0.8732	3.83E-144	1.22E-140	PGS001531	EFO_0007772	calcaneal bone quantitative ultrasound measurement	calcaneal bone quantitative ultrasound measurement	EFO_0007772	Other measurement	Skeletal	PGS001403	EFO_0009270	heel bone mineral density	heel bone mineral density	EFO_0009270	Other measurement	Skeletal
0.8732	4.05E-144	1.29E-140	PGS002993	EFO_0004339	body height	body height	EFO_0004339	Body measurement	Body measurement	PGS002989	EFO_0004339	body height	body height	EFO_0004339	Body measurement	Body measurement
0.873	5.48E-144	1.74E-140	PGS000041	EFO_0001060	celiac disease	celiac disease	EFO_0001060	Immune system disorder	Immunological	PGS000042	EFO_0001060	celiac disease	celiac disease	EFO_0001060	Digestive system disorder	Gastrointestinal
0.873	5.48E-144	1.74E-140	PGS000041	EFO_0001060	celiac disease	celiac disease	EFO_0001060	Immune system disorder	Immunological	PGS000042	EFO_0001060	celiac disease	celiac disease	EFO_0001060	Immune system disorder	Immunological
0.873	5.48E-144	1.74E-140	PGS000041	EFO_0001060	celiac disease	celiac disease	EFO_0001060	Digestive system disorder	Gastrointestinal	PGS000042	EFO_0001060	celiac disease	celiac disease	EFO_0001060	Immune system disorder	Immunological
0.873	5.48E-144	1.74E-140	PGS000041	EFO_0001060	celiac disease	celiac disease	EFO_0001060	Digestive system disorder	Gastrointestinal	PGS000042	EFO_0001060	celiac disease	celiac disease	EFO_0001060	Digestive system disorder	Gastrointestinal
0.873	5.58E-144	1.77E-140	PGS003311	EFO_0004617	cystatin C measurement|glomerular filtration rate	cystatin C measurement	EFO_0004617	Other measurement	Metabolic	PGS003309	EFO_0004617	cystatin C measurement|glomerular filtration rate	cystatin C measurement	EFO_0004617	Other measurement	Metabolic
0.8729	6.79E-144	2.15E-140	PGS000507	EFO_0000305	breast carcinoma	breast carcinoma	EFO_0000305	Cancer	Neoplasms	PGS000512	EFO_0000305	breast carcinoma	breast carcinoma	EFO_0000305	Cancer	Neoplasms
0.8727	8.86E-144	2.81E-140	PGS002442	EFO_0000400	diabetes mellitus	diabetes mellitus	EFO_0000400	Digestive system disorder	Gastrointestinal	PGS002400	EFO_0004468	glucose measurement	glucose measurement	EFO_0004468	Other measurement	Metabolic
0.8727	8.86E-144	2.81E-140	PGS002442	EFO_0000400	diabetes mellitus	diabetes mellitus	EFO_0000400	Metabolic disorder	Metabolic	PGS002400	EFO_0004468	glucose measurement	glucose measurement	EFO_0004468	Other measurement	Metabolic
0.8726	1.08E-143	3.43E-140	PGS003064	EFO_0006527	smoking status measurement	smoking status measurement	EFO_0006527	Other measurement	Psychiatric	PGS003059	EFO_0006527	smoking status measurement	smoking status measurement	EFO_0006527	Other measurement	Psychiatric
0.8725	1.39E-143	4.41E-140	PGS002403	EFO_0004541	HbA1c measurement	HbA1c measurement	EFO_0004541	Hematological measurement	Hematological	PGS002442	EFO_0000400	diabetes mellitus	diabetes mellitus	EFO_0000400	Digestive system disorder	Gastrointestinal
0.8725	1.39E-143	4.41E-140	PGS002403	EFO_0004541	HbA1c measurement	HbA1c measurement	EFO_0004541	Hematological measurement	Hematological	PGS002442	EFO_0000400	diabetes mellitus	diabetes mellitus	EFO_0000400	Metabolic disorder	Metabolic
0.8724	1.49E-143	4.69E-140	PGS000611	MONDO_0004986	urinary bladder carcinoma	urinary bladder carcinoma	MONDO_0004986	Cancer	Neoplasms	PGS003335	MONDO_0004986	urinary bladder carcinoma	urinary bladder carcinoma	MONDO_0004986	Cancer	Neoplasms
0.8724	1.49E-143	4.69E-140	PGS000612	MONDO_0004986	urinary bladder carcinoma	urinary bladder carcinoma	MONDO_0004986	Cancer	Neoplasms	PGS003335	MONDO_0004986	urinary bladder carcinoma	urinary bladder carcinoma	MONDO_0004986	Cancer	Neoplasms
0.8724	1.49E-143	4.69E-140	PGS000610	MONDO_0004986	urinary bladder carcinoma	urinary bladder carcinoma	MONDO_0004986	Cancer	Neoplasms	PGS003335	MONDO_0004986	urinary bladder carcinoma	urinary bladder carcinoma	MONDO_0004986	Cancer	Neoplasms
0.8722	2.08E-143	6.56E-140	PGS000585	EFO_0001663	prostate carcinoma	prostate carcinoma	EFO_0001663	Cancer	Neoplasms	PGS000581	EFO_0001663	prostate carcinoma	prostate carcinoma	EFO_0001663	Cancer	Neoplasms
0.8722	2.08E-143	6.56E-140	PGS000585	EFO_0001663	prostate carcinoma	prostate carcinoma	EFO_0001663	Cancer	Neoplasms	PGS000582	EFO_0001663	prostate carcinoma	prostate carcinoma	EFO_0001663	Cancer	Neoplasms
0.8722	2.19E-143	6.90E-140	PGS003008	EFO_0000537	hypertension	hypertension	EFO_0000537	Cardiovascular disease	Cardiovascular	PGS003028	EFO_0000537	hypertension	hypertension	EFO_0000537	Cardiovascular disease	Cardiovascular
0.8721	2.42E-143	7.62E-140	PGS002328	EFO_0004468	glucose measurement	glucose measurement	EFO_0004468	Other measurement	Metabolic	PGS002938	EFO_0004468	glucose measurement	glucose measurement	EFO_0004468	Other measurement	Metabolic
0.8721	2.49E-143	7.84E-140	PGS000758	EFO_0004339	body height	body height	EFO_0004339	Body measurement	Body measurement	PGS002332	EFO_0004339	body height	body height	EFO_0004339	Body measurement	Body measurement
0.872	3.01E-143	9.46E-140	PGS001725	EFO_0006930	brain volume measurement|neuroimaging measurement	brain volume measurement	EFO_0006930	Other measurement	Neurological	PGS001754	EFO_0006930	measurement	brain volume measurement	EFO_0006930	Other measurement	Neurological
0.8719	3.47E-143	1.09E-139	PGS000453	EFO_0004193	basal cell carcinoma	basal cell carcinoma	EFO_0004193	Cancer	Neoplasms	PGS000458	EFO_0004193	basal cell carcinoma	basal cell carcinoma	EFO_0004193	Cancer	Neoplasms
0.8719	3.47E-143	1.09E-139	PGS000452	EFO_0004193	basal cell carcinoma	basal cell carcinoma	EFO_0004193	Cancer	Neoplasms	PGS000458	EFO_0004193	basal cell carcinoma	basal cell carcinoma	EFO_0004193	Cancer	Neoplasms
0.8719	3.47E-143	1.09E-139	PGS000455	EFO_0004193	basal cell carcinoma	basal cell carcinoma	EFO_0004193	Cancer	Neoplasms	PGS000458	EFO_0004193	basal cell carcinoma	basal cell carcinoma	EFO_0004193	Cancer	Neoplasms
0.8719	3.65E-143	1.15E-139	PGS000783	EFO_0000305	breast carcinoma	breast carcinoma	EFO_0000305	Cancer	Neoplasms	PGS000773	EFO_0000305	breast carcinoma	breast carcinoma	EFO_0000305	Cancer	Neoplasms
0.8719	3.75E-143	1.18E-139	PGS002161	EFO_0004340	body mass index	body mass index	EFO_0004340	Body measurement	Body measurement	PGS002844	EFO_0004340	body mass index	body mass index	EFO_0004340	Body measurement	Body measurement
0.8717	4.55E-143	1.42E-139	PGS000783	EFO_0000305	breast carcinoma	breast carcinoma	EFO_0000305	Cancer	Neoplasms	PGS000052	EFO_0000305	breast carcinoma	breast carcinoma	EFO_0000305	Cancer	Neoplasms
0.8717	4.87E-143	1.53E-139	PGS000516	EFO_0000305	breast carcinoma	breast carcinoma	EFO_0000305	Cancer	Neoplasms	PGS000479	EFO_0000305	breast carcinoma	breast carcinoma	EFO_0000305	Cancer	Neoplasms
0.8715	6.39E-143	2.00E-139	PGS001146	EFO_0005409	fat body mass	fat body mass	EFO_0005409	Other measurement	Body measurement	PGS001166	EFO_0004995	lean body mass	lean body mass	EFO_0004995	Other measurement	Skeletal
0.8714	7.36E-143	2.30E-139	PGS001462	EFO_0006930	brain volume measurement|neuroimaging measurement	brain volume measurement	EFO_0006930	Other measurement	Neurological	PGS001461	EFO_0006930	measurement	brain volume measurement	EFO_0006930	Other measurement	Neurological
0.8714	7.57E-143	2.36E-139	PGS001117	EFO_0600082	insulin use measurement	insulin use measurement	EFO_0600082	Other measurement	Metabolic	PGS001296	MONDO_0005147	type 1 diabetes mellitus	type 1 diabetes mellitus	MONDO_0005147	Metabolic disorder	Metabolic
0.8714	7.57E-143	2.36E-139	PGS001117	EFO_0600082	insulin use measurement	insulin use measurement	EFO_0600082	Other measurement	Metabolic	PGS001296	MONDO_0005147	type 1 diabetes mellitus	type 1 diabetes mellitus	MONDO_0005147	Digestive system disorder	Gastrointestinal
0.8714	7.57E-143	2.36E-139	PGS001117	EFO_0600082	insulin use measurement	insulin use measurement	EFO_0600082	Other measurement	Metabolic	PGS001296	MONDO_0005147	type 1 diabetes mellitus	type 1 diabetes mellitus	MONDO_0005147	Immune system disorder	Immunological
0.8713	9.11E-143	2.84E-139	PGS001149	EFO_0005409	fat body mass	fat body mass	EFO_0005409	Other measurement	Body measurement	PGS001162	EFO_0005093	hip circumference	hip circumference	EFO_0005093	Body measurement	Body measurement
0.8713	9.21E-143	2.87E-139	PGS001158	EFO_0005106	body composition measurement	body composition measurement	EFO_0005106	Body measurement	Body measurement	PGS001146	EFO_0005409	fat body mass	fat body mass	EFO_0005409	Other measurement	Body measurement
-0.8713	1.01E-142	3.13E-139	PGS002155	EFO_0004279	suntan	suntan	EFO_0004279	Other trait	Dermatological	PGS002110	EFO_0003784	skin pigmentation	skin pigmentation	EFO_0003784	Other trait	Dermatological
0.8712	1.04E-142	3.24E-139	PGS002594	EFO_0004713	FEV|FEC ratio	FEV|FEC ratio	EFO_0004713	Other measurement	Respiratory	PGS002398	EFO_0004713	FEV|FEC ratio	FEV|FEC ratio	EFO_0004713	Other measurement	Respiratory
0.8711	1.24E-142	3.86E-139	PGS001166	EFO_0004995	lean body mass	lean body mass	EFO_0004995	Other measurement	Skeletal	PGS001234	EFO_0004324	body weights and measures	body weights and measures	EFO_0004324	Body measurement	Body measurement
0.8711	1.40E-142	4.37E-139	PGS001158	EFO_0005106	body composition measurement	body composition measurement	EFO_0005106	Body measurement	Body measurement	PGS001234	EFO_0004324	body weights and measures	body weights and measures	EFO_0004324	Body measurement	Body measurement
0.871	1.43E-142	4.43E-139	PGS003013	EFO_0000537	hypertension	hypertension	EFO_0000537	Cardiovascular disease	Cardiovascular	PGS003023	EFO_0000537	hypertension	hypertension	EFO_0000537	Cardiovascular disease	Cardiovascular
0.8709	1.88E-142	5.84E-139	PGS001488	EFO_0006930	brain volume measurement|neuroimaging measurement	brain volume measurement	EFO_0006930	Other measurement	Neurological	PGS001683	EFO_0006930	measurement	brain volume measurement	EFO_0006930	Other measurement	Neurological
0.8708	2.03E-142	6.29E-139	PGS002955	EFO_0004612	high density lipoprotein cholesterol measurement	high density lipoprotein cholesterol measurement	EFO_0004612	Lipid or lipoprotein measurement	Metabolic	PGS002956	EFO_0004612	high density lipoprotein cholesterol measurement	measurement	EFO_0004612	Lipid or lipoprotein measurement	Metabolic
0.8708	2.12E-142	6.58E-139	PGS000503	EFO_0000305	breast carcinoma	breast carcinoma	EFO_0000305	Cancer	Neoplasms	PGS000512	EFO_0000305	breast carcinoma	breast carcinoma	EFO_0000305	Cancer	Neoplasms
0.8707	2.56E-142	7.94E-139	PGS002846	EFO_0004340	body mass index	body mass index	EFO_0004340	Body measurement	Body measurement	PGS002847	EFO_0004340	body mass index	body mass index	EFO_0004340	Body measurement	Body measurement
0.8706	3.05E-142	9.44E-139	PGS003295	EFO_0004617	cystatin C measurement|glomerular filtration rate	cystatin C measurement	EFO_0004617	Other measurement	Metabolic	PGS003309	EFO_0004617	cystatin C measurement|glomerular filtration rate	cystatin C measurement	EFO_0004617	Other measurement	Metabolic
0.8704	3.80E-142	1.18E-138	PGS000463	EFO_0000707	squamous cell carcinoma	squamous cell carcinoma	EFO_0000707	Cancer	Neoplasms	PGS000467	EFO_0000707	squamous cell carcinoma	squamous cell carcinoma	EFO_0000707	Cancer	Neoplasms
0.8704	4.46E-142	1.38E-138	PGS000480	EFO_0000305	breast carcinoma	breast carcinoma	EFO_0000305	Cancer	Neoplasms	PGS000472	EFO_0000305	breast carcinoma	breast carcinoma	EFO_0000305	Cancer	Neoplasms
0.8703	4.54E-142	1.41E-138	PGS001480	EFO_0006930	brain volume measurement|neuroimaging measurement	brain volume measurement	EFO_0006930	Other measurement	Neurological	PGS001481	EFO_0006930	measurement	brain volume measurement	EFO_0006930	Other measurement	Neurological
0.8703	4.57E-142	1.41E-138	PGS000523	EFO_0000305	breast carcinoma	breast carcinoma	EFO_0000305	Cancer	Neoplasms	PGS000524	EFO_0000305	breast carcinoma	breast carcinoma	EFO_0000305	Cancer	Neoplasms
-0.8702	5.89E-142	1.82E-138	PGS000016	EFO_0000275	atrial fibrillation	atrial fibrillation	EFO_0000275	Cardiovascular disease	Cardiovascular	PGS001356	EFO_0000275	atrial fibrillation	atrial fibrillation	EFO_0000275	Cardiovascular disease	Cardiovascular
0.8701	7.24E-142	2.24E-138	PGS001147	EFO_0005409	fat body mass	fat body mass	EFO_0005409	Other measurement	Body measurement	PGS001148	EFO_0005409	fat body mass	fat body mass	EFO_0005409	Other measurement	Body measurement
0.87	8.05E-142	2.48E-138	PGS001943	EFO_0004340	body mass index	body mass index	EFO_0004340	Body measurement	Body measurement	PGS002313	EFO_0004340	body mass index	body mass index	EFO_0004340	Body measurement	Body measurement
0.8699	8.75E-142	2.70E-138	PGS000594	EFO_0007355	male reproductive organ cancer	male reproductive organ cancer	EFO_0007355	Cancer	Neoplasms	PGS000593	EFO_0007355	male reproductive organ cancer	male reproductive organ cancer	EFO_0007355	Cancer	Neoplasms
0.8699	9.86E-142	3.04E-138	PGS000955	EFO_0004514	bone quantitative ultrasound measurement	bone quantitative ultrasound measurement	EFO_0004514	Other measurement	Skeletal	PGS000953	EFO_0004514	bone quantitative ultrasound measurement	measurement	EFO_0004514	Other measurement	Skeletal
0.8698	1.16E-141	3.59E-138	PGS000347	EFO_1000649	estrogen-receptor positive breast cancer	estrogen-receptor positive breast cancer	EFO_1000649	Cancer	Neoplasms	PGS000004	EFO_0000305	breast carcinoma	breast carcinoma	EFO_0000305	Cancer	Neoplasms
0.8697	1.27E-141	3.90E-138	PGS001939	EFO_0004533	alkaline phosphatase measurement	alkaline phosphatase measurement	EFO_0004533	Liver enzyme measurement	Gastrointestinal	PGS000670	EFO_0004533	alkaline phosphatase measurement	alkaline phosphatase measurement	EFO_0004533	Liver enzyme measurement	Gastrointestinal
0.8697	1.32E-141	4.04E-138	PGS000956	EFO_0004514	bone quantitative ultrasound measurement	bone quantitative ultrasound measurement	EFO_0004514	Other measurement	Skeletal	PGS000954	EFO_0004514	bone quantitative ultrasound measurement	measurement	EFO_0004514	Other measurement	Skeletal
0.8697	1.32E-141	4.04E-138	PGS001039	EFO_0009270	heel bone mineral density	heel bone mineral density	EFO_0009270	Other measurement	Skeletal	PGS000954	EFO_0004514	bone quantitative ultrasound measurement	measurement	EFO_0004514	Other measurement	Skeletal
0.8697	1.35E-141	4.16E-138	PGS000956	EFO_0004514	bone quantitative ultrasound measurement	bone quantitative ultrasound measurement	EFO_0004514	Other measurement	Skeletal	PGS001038	EFO_0009270	heel bone mineral density	heel bone mineral density	EFO_0009270	Other measurement	Skeletal
0.8697	1.35E-141	4.16E-138	PGS001039	EFO_0009270	heel bone mineral density	heel bone mineral density	EFO_0009270	Other measurement	Skeletal	PGS001038	EFO_0009270	heel bone mineral density	heel bone mineral density	EFO_0009270	Other measurement	Skeletal
0.8696	1.53E-141	4.70E-138	PGS002611	EFO_0004309	platelet count	platelet count	EFO_0004309	Hematological measurement	Hematological	PGS002464	EFO_0004309	platelet count	platelet count	EFO_0004309	Hematological measurement	Hematological
0.8695	1.80E-141	5.52E-138	PGS003328	EFO_0004698	insomnia	insomnia	EFO_0004698	Neurological disorder	Neurological	PGS002149	EFO_0004698	insomnia	insomnia	EFO_0004698	Neurological disorder	Neurological
0.8694	2.05E-141	6.27E-138	PGS000030	EFO_0001663	prostate carcinoma	prostate carcinoma	EFO_0001663	Cancer	Neoplasms	PGS000742	EFO_0001663	prostate carcinoma	prostate carcinoma	EFO_0001663	Cancer	Neoplasms
0.8694	2.08E-141	6.36E-138	PGS002161	EFO_0004340	body mass index	body mass index	EFO_0004340	Body measurement	Body measurement	PGS002859	EFO_0004340	body mass index	body mass index	EFO_0004340	Body measurement	Body measurement
0.8694	2.21E-141	6.76E-138	PGS001114	EFO_0007012	NSAID use measurement|self-reported trait	NSAID use measurement	EFO_0007012	Other measurement	Activities	PGS001116	EFO_0007012	NSAID use measurement	NSAID use measurement	EFO_0007012	Other measurement	Activities
0.8693	2.35E-141	7.17E-138	PGS002543	EFO_0001379	endocrine system disease|diabetes mellitus	endocrine system disease	EFO_0001379	Other disease	Metabolic	PGS002449	EFO_0004468	glucose measurement	glucose measurement	EFO_0004468	Other measurement	Metabolic
0.8693	2.36E-141	7.20E-138	PGS000671	EFO_0004614	apolipoprotein A 1 measurement	apolipoprotein A 1 measurement	EFO_0004614	Lipid or lipoprotein measurement	Metabolic	PGS000686	EFO_0004612	high density lipoprotein cholesterol measurement	measurement	EFO_0004612	Lipid or lipoprotein measurement	Metabolic
0.8693	2.36E-141	7.20E-138	PGS000671	EFO_0004614	apolipoprotein A 1 measurement	apolipoprotein A 1 measurement	EFO_0004614	Inflammatory measurement	Immunological	PGS000686	EFO_0004612	high density lipoprotein cholesterol measurement	measurement	EFO_0004612	Lipid or lipoprotein measurement	Metabolic
0.8693	2.36E-141	7.20E-138	PGS000671	EFO_0004614	apolipoprotein A 1 measurement	apolipoprotein A 1 measurement	EFO_0004614	Cardiovascular measurement	Cardiovascular	PGS000686	EFO_0004612	high density lipoprotein cholesterol measurement	measurement	EFO_0004612	Lipid or lipoprotein measurement	Metabolic
0.8693	2.39E-141	7.30E-138	PGS001531	EFO_0007772	calcaneal bone quantitative ultrasound measurement	calcaneal bone quantitative ultrasound measurement	EFO_0007772	Other measurement	Skeletal	PGS000954	EFO_0004514	bone quantitative ultrasound measurement	measurement	EFO_0004514	Other measurement	Skeletal
0.8693	2.41E-141	7.36E-138	PGS001531	EFO_0007772	calcaneal bone quantitative ultrasound measurement	calcaneal bone quantitative ultrasound measurement	EFO_0007772	Other measurement	Skeletal	PGS001038	EFO_0009270	heel bone mineral density	heel bone mineral density	EFO_0009270	Other measurement	Skeletal
0.8692	3.01E-141	9.19E-138	PGS001918	EFO_0004314	forced expiratory volume	forced expiratory volume	EFO_0004314	Other measurement	Respiratory	PGS001922	EFO_0004312	vital capacity	vital capacity	EFO_0004312	Other measurement	Activities
0.8691	3.38E-141	1.03E-137	PGS002589	EFO_0000400	diabetes mellitus	diabetes mellitus	EFO_0000400	Metabolic disorder	Metabolic	PGS002452	EFO_0004541	HbA1c measurement	HbA1c measurement	EFO_0004541	Hematological measurement	Hematological
0.8691	3.38E-141	1.03E-137	PGS002589	EFO_0000400	diabetes mellitus	diabetes mellitus	EFO_0000400	Digestive system disorder	Gastrointestinal	PGS002452	EFO_0004541	HbA1c measurement	HbA1c measurement	EFO_0004541	Hematological measurement	Hematological
0.8689	4.78E-141	1.46E-137	PGS003169	EFO_0004620	vitamin B12 measurement	vitamin B12 measurement	EFO_0004620	Other measurement	Metabolic	PGS003164	EFO_0004620	vitamin B12 measurement	vitamin B12 measurement	EFO_0004620	Other measurement	Metabolic
0.8687	6.20E-141	1.89E-137	PGS002427	EFO_0004631	vitamin D measurement	vitamin D measurement	EFO_0004631	Lipid or lipoprotein measurement	Metabolic	PGS003195	EFO_0004631	vitamin D measurement	vitamin D measurement	EFO_0004631	Lipid or lipoprotein measurement	Metabolic
0.8686	7.17E-141	2.18E-137	PGS001229	EFO_0004339	body height	body height	EFO_0004339	Body measurement	Body measurement	PGS002332	EFO_0004339	body height	body height	EFO_0004339	Body measurement	Body measurement
0.8686	7.66E-141	2.33E-137	PGS002452	EFO_0004541	HbA1c measurement	HbA1c measurement	EFO_0004541	Hematological measurement	Hematological	PGS002400	EFO_0004468	glucose measurement	glucose measurement	EFO_0004468	Other measurement	Metabolic
0.8685	8.59E-141	2.61E-137	PGS001522	HP_0003040	Arthropathy	Arthropathy	HP_0003040	Other trait	Skeletal	PGS001287	EFO_0003778	psoriatic arthritis	psoriatic arthritis	EFO_0003778	Immune system disorder	Immunological
0.8685	8.63E-141	2.62E-137	PGS002044	MONDO_0005129	cataract	cataract	MONDO_0005129	Other disease	Ophthalmological	PGS001837	MONDO_0005129	cataract	cataract	MONDO_0005129	Other disease	Ophthalmological
0.8685	9.60E-141	2.91E-137	PGS000482	EFO_0000305	breast carcinoma	breast carcinoma	EFO_0000305	Cancer	Neoplasms	PGS000515	EFO_0000305	breast carcinoma	breast carcinoma	EFO_0000305	Cancer	Neoplasms
0.8684	1.03E-140	3.13E-137	PGS000589	EFO_0001663	prostate carcinoma	prostate carcinoma	EFO_0001663	Cancer	Neoplasms	PGS000591	EFO_0001663	prostate carcinoma	prostate carcinoma	EFO_0001663	Cancer	Neoplasms
0.8684	1.10E-140	3.32E-137	PGS002177	EFO_0010700	reticulocyte measurement	reticulocyte measurement	EFO_0010700	Hematological measurement	Hematological	PGS001976	EFO_0007986	reticulocyte count	reticulocyte count	EFO_0007986	Hematological measurement	Hematological
0.8682	1.35E-140	4.10E-137	PGS000613	MONDO_0004986	urinary bladder carcinoma	urinary bladder carcinoma	MONDO_0004986	Cancer	Neoplasms	PGS003335	MONDO_0004986	urinary bladder carcinoma	urinary bladder carcinoma	MONDO_0004986	Cancer	Neoplasms
0.8682	1.38E-140	4.19E-137	PGS000795	EFO_0001663	prostate carcinoma	prostate carcinoma	EFO_0001663	Cancer	Neoplasms	PGS000580	EFO_0001663	prostate carcinoma	prostate carcinoma	EFO_0001663	Cancer	Neoplasms

0.8682	1.51E-140	4.55E-137	PGS002315	EFO_0003923	bone density	bone density	EFO_0003923	Other measurement	Skeletal	PGS001957	EFO_0005654	velocity of sound measurement	velocity of sound measurement	EFO_0005654	Other measurement	Others
0.868	1.96E-140	5.93E-137	PGS002313	EFO_0004340	body mass index	body mass index	EFO_0004340	Body measurement	Body measurement	PGS002859	EFO_0004340	body mass index	body mass index	EFO_0004340	Body measurement	Body measurement
0.8679	2.18E-140	6.58E-137	PGS002338	EFO_0004587	lymphocyte count	lymphocyte count	EFO_0004587	Hematological measurement	Hematological	PGS002183	EFO_0004587	lymphocyte count	lymphocyte count	EFO_0004587	Hematological measurement	Hematological
0.8679	2.18E-140	6.59E-137	PGS000535	EFO_0000305	breast carcinoma	breast carcinoma	EFO_0000305	Cancer	Neoplasms	PGS000527	EFO_0000305	breast carcinoma	breast carcinoma	EFO_0000305	Cancer	Neoplasms
0.8679	2.38E-140	7.18E-137	PGS001640	EFO_0008320	measurement	white matter volume measurement	EFO_0008320	Other measurement	Neurological	PGS001540	EFO_0008320	volume measurement|neuroimaging measurement	white matter volume measurement	EFO_0008320	Other measurement	Neurological
0.8679	2.38E-140	7.19E-137	PGS002473	EFO_0004574	total cholesterol measurement	total cholesterol measurement	EFO_0004574	Lipid or lipoprotein measurement	Metabolic	PGS002424	EFO_0004574	total cholesterol measurement	total cholesterol measurement	EFO_0004574	Lipid or lipoprotein measurement	Metabolic
0.8679	2.48E-140	7.48E-137	PGS003399	EFO_0000305	breast carcinoma	breast carcinoma	EFO_0000305	Cancer	Neoplasms	PGS000345	EFO_0000305	breast carcinoma	breast carcinoma	EFO_0000305	Cancer	Neoplasms
0.8678	2.97E-140	8.94E-137	PGS002313	EFO_0004340	body mass index	body mass index	EFO_0004340	Body measurement	Body measurement	PGS002849	EFO_0004340	body mass index	body mass index	EFO_0004340	Body measurement	Body measurement
0.8677	3.11E-140	9.36E-137	PGS002007	EFO_0009282	sodium measurement	sodium measurement	EFO_0009282	Other measurement	Metabolic	PGS002226	EFO_0009282	sodium measurement	sodium measurement	EFO_0009282	Other measurement	Metabolic
0.8677	3.48E-140	1.05E-136	PGS002161	EFO_0004340	body mass index	body mass index	EFO_0004340	Body measurement	Body measurement	PGS002849	EFO_0004340	body mass index	body mass index	EFO_0004340	Body measurement	Body measurement
0.8677	3.48E-140	1.05E-136	PGS001166	EFO_0004995	lean body mass	lean body mass	EFO_0004995	Other measurement	Skeletal	PGS001165	EFO_0004995	lean body mass	lean body mass	EFO_0004995	Other measurement	Skeletal
0.8676	3.56E-140	1.07E-136	PGS000347	EFO_1000649	estrogen-receptor positive breast cancer	estrogen-receptor positive breast cancer	EFO_1000649	Cancer	Neoplasms	PGS000005	EFO_1000649	estrogen-receptor positive breast cancer	cancer	EFO_1000649	Cancer	Neoplasms
0.8676	3.81E-140	1.14E-136	PGS001158	EFO_0005106	body composition measurement	body composition measurement	EFO_0005106	Body measurement	Body measurement	PGS001165	EFO_0004995	lean body mass	lean body mass	EFO_0004995	Other measurement	Skeletal
0.8675	4.34E-140	1.30E-136	PGS000476	EFO_0000305	breast carcinoma	breast carcinoma	EFO_0000305	Cancer	Neoplasms	PGS000513	EFO_0000305	breast carcinoma	breast carcinoma	EFO_0000305	Cancer	Neoplasms
0.8675	4.68E-140	1.40E-136	PGS002577	EFO_0009102	number of children ever born measurement	number of children ever born measurement	EFO_0009102	Other measurement	Genitourinary	PGS002528	EFO_0009102	number of children ever born measurement	measurement	EFO_0009102	Other measurement	Genitourinary
0.8674	5.51E-140	1.65E-136	PGS000577	EFO_0001663	prostate carcinoma	prostate carcinoma	EFO_0001663	Cancer	Neoplasms	PGS000795	EFO_0001663	prostate carcinoma	prostate carcinoma	EFO_0001663	Cancer	Neoplasms
0.8674	5.51E-140	1.65E-136	PGS000578	EFO_0001663	prostate carcinoma	prostate carcinoma	EFO_0001663	Cancer	Neoplasms	PGS000795	EFO_0001663	prostate carcinoma	prostate carcinoma	EFO_0001663	Cancer	Neoplasms
0.8674	5.51E-140	1.65E-136	PGS002161	EFO_0004340	body mass index	body mass index	EFO_0004340	Body measurement	Body measurement	PGS002201	EFO_0004342	waist circumference	waist circumference	EFO_0004342	Body measurement	Body measurement
0.8673	5.75E-140	1.72E-136	PGS000795	EFO_0001663	prostate carcinoma	prostate carcinoma	EFO_0001663	Cancer	Neoplasms	PGS002791	EFO_0001663	prostate carcinoma	prostate carcinoma	EFO_0001663	Cancer	Neoplasms
0.8673	6.40E-140	1.91E-136	PGS002852	EFO_0004340	body mass index	body mass index	EFO_0004340	Body measurement	Body measurement	PGS002847	EFO_0004340	body mass index	body mass index	EFO_0004340	Body measurement	Body measurement
0.8672	7.14E-140	2.13E-136	PGS000528	EFO_0000305	breast carcinoma	breast carcinoma	EFO_0000305	Cancer	Neoplasms	PGS000536	EFO_0000305	breast carcinoma	breast carcinoma	EFO_0000305	Cancer	Neoplasms
0.867	9.58E-140	2.86E-136	PGS000913	EFO_0006335	systolic blood pressure	systolic blood pressure	EFO_0006335	Other measurement	Cardiovascular	PGS000900	EFO_0006335	systolic blood pressure|female	systolic blood pressure	EFO_0006335	Other measurement	Cardiovascular
0.867	1.02E-139	3.05E-136	PGS003027	EFO_0000537	hypertension	hypertension	EFO_0000537	Cardiovascular disease	Cardiovascular	PGS003022	EFO_0000537	hypertension	hypertension	EFO_0000537	Cardiovascular disease	Cardiovascular
0.8669	1.11E-139	3.31E-136	PGS000758	EFO_0004339	body height	body height	EFO_0004339	Body measurement	Body measurement	PGS002146	EFO_0004339	body height	body height	EFO_0004339	Body measurement	Body measurement
0.8668	1.35E-139	4.04E-136	PGS000335	EFO_0000305	breast carcinoma	breast carcinoma	EFO_0000305	Cancer	Neoplasms	PGS000512	EFO_0000305	breast carcinoma	breast carcinoma	EFO_0000305	Cancer	Neoplasms
0.8667	1.66E-139	4.94E-136	PGS002460	EFO_0004527	mean corpuscular hemoglobin	mean corpuscular hemoglobin	EFO_0004527	Hematological measurement	Hematological	PGS002558	EFO_0004527	mean corpuscular hemoglobin	mean corpuscular hemoglobin	EFO_0004527	Hematological measurement	Hematological
0.8667	1.67E-139	4.96E-136	PGS001953	EFO_0004541	HbA1c measurement	HbA1c measurement	EFO_0004541	Hematological measurement	Hematological	PGS002331	EFO_0004541	HbA1c measurement	HbA1c measurement	EFO_0004541	Hematological measurement	Hematological
0.8667	1.72E-139	5.10E-136	PGS001903	EFO_0600085	PP interval	PP interval	EFO_0600085	Cardiovascular measurement	Cardiovascular	PGS001907	EFO_0004831	RR interval	RR interval	EFO_0004831	Cardiovascular measurement	Cardiovascular
0.8666	1.80E-139	5.36E-136	PGS001149	EFO_0005409	fat body mass	fat body mass	EFO_0005409	Other measurement	Body measurement	PGS001102	EFO_0007800	body fat percentage	body fat percentage	EFO_0007800	Body measurement	Body measurement
0.8665	2.05E-139	6.08E-136	PGS000613	MONDO_0004986	urinary bladder carcinoma	urinary bladder carcinoma	MONDO_0004986	Cancer	Neoplasms	PGS000071	MONDO_0004986	urinary bladder carcinoma	urinary bladder carcinoma	MONDO_0004986	Cancer	Neoplasms
0.8663	2.81E-139	8.35E-136	PGS002057	HP_0002619	Varicose veins	Varicose veins	HP_0002619	Other trait	Cardiovascular	PGS001845	HP_0002619	Varicose veins	Varicose veins	HP_0002619	Other trait	Cardiovascular
0.8661	3.96E-139	1.17E-135	PGS001737	EFO_0006930	brain volume measurement|neuroimaging measurement	brain volume measurement	EFO_0006930	Other measurement	Neurological	PGS001752	EFO_0006930	measurement	brain volume measurement	EFO_0006930	Other measurement	Neurological
0.866	5.10E-139	1.51E-135	PGS000579	EFO_0001663	prostate carcinoma	prostate carcinoma	EFO_0001663	Cancer	Neoplasms	PGS000030	EFO_0001663	prostate carcinoma	prostate carcinoma	EFO_0001663	Cancer	Neoplasms
0.8659	5.26E-139	1.56E-135	PGS001366	EFO_0004731	eye measurement	eye measurement	EFO_0004731	Other measurement	Ophthalmological	PGS001365	EFO_0004731	eye measurement	eye measurement	EFO_0004731	Other measurement	Ophthalmological
0.8658	6.53E-139	1.93E-135	PGS002169	EFO_0005409	fat body mass	fat body mass	EFO_0005409	Other measurement	Body measurement	PGS002176	EFO_0005093	hip circumference	hip circumference	EFO_0005093	Body measurement	Body measurement
0.8656	8.43E-139	2.49E-135	PGS002475	MONDO_0005148	type 2 diabetes mellitus	type 2 diabetes mellitus	MONDO_0005148	Digestive system disorder	Gastrointestinal	PGS002596	EFO_0004468	glucose measurement	glucose measurement	EFO_0004468	Other measurement	Metabolic
0.8656	8.43E-139	2.49E-135	PGS002475	MONDO_0005148	type 2 diabetes mellitus	type 2 diabetes mellitus	MONDO_0005148	Metabolic disorder	Metabolic	PGS002596	EFO_0004468	glucose measurement	glucose measurement	EFO_0004468	Other measurement	Metabolic
0.8656	8.89E-139	2.63E-135	PGS001145	EFO_0005409	fat body mass	fat body mass	EFO_0005409	Other measurement	Body measurement	PGS001102	EFO_0007800	body fat percentage	body fat percentage	EFO_0007800	Body measurement	Body measurement
0.8656	9.08E-139	2.68E-135	PGS002854	EFO_0004340	body mass index	body mass index	EFO_0004340	Body measurement	Body measurement	PGS002850	EFO_0004340	body mass index	body mass index	EFO_0004340	Body measurement	Body measurement
0.8654	1.27E-138	3.73E-135	PGS000497	EFO_0000305	breast carcinoma	breast carcinoma	EFO_0000305	Cancer	Neoplasms	PGS000505	EFO_0000305	breast carcinoma	breast carcinoma	EFO_0000305	Cancer	Neoplasms
0.8654	1.27E-138	3.73E-135	PGS000498	EFO_0000305	breast carcinoma	breast carcinoma	EFO_0000305	Cancer	Neoplasms	PGS000505	EFO_0000305	breast carcinoma	breast carcinoma	EFO_0000305	Cancer	Neoplasms
0.8654	1.27E-138	3.73E-135	PGS000506	EFO_0000305	breast carcinoma	breast carcinoma	EFO_0000305	Cancer	Neoplasms	PGS000497	EFO_0000305	breast carcinoma	breast carcinoma	EFO_0000305	Cancer	Neoplasms
0.8654	1.27E-138	3.73E-135	PGS000506	EFO_0000305	breast carcinoma	breast carcinoma	EFO_0000305	Cancer	Neoplasms	PGS000498	EFO_0000305	breast carcinoma	breast carcinoma	EFO_0000305	Cancer	Neoplasms
0.8654	1.29E-138	3.80E-135	PGS000438	MONDO_0002898	skin cancer	skin cancer	MONDO_0002898	Cancer	Neoplasms	PGS000442	MONDO_0002898	skin cancer	skin cancer	MONDO_0002898	Cancer	Neoplasms
0.8652	1.70E-138	5.00E-135	PGS002177	EFO_0010700	reticulocyte measurement	reticulocyte measurement	EFO_0010700	Hematological measurement	Hematological	PGS002333	EFO_0007986	reticulocyte count	reticulocyte count	EFO_0007986	Hematological measurement	Hematological
0.8652	1.72E-138	5.07E-135	PGS002764	HP_0003124	Hypercholesterolemia	Hypercholesterolemia	HP_0003124	Other trait	Metabolic	PGS000892	EFO_0004611	low density lipoprotein cholesterol measurement	measurement	EFO_0004611	Cardiovascular measurement	Cardiovascular
0.8652	1.72E-138	5.07E-135	PGS002764	HP_0003124	Hypercholesterolemia	Hypercholesterolemia	HP_0003124	Other trait	Metabolic	PGS000892	EFO_0004611	low density lipoprotein cholesterol measurement	measurement	EFO_0004611	Lipid or lipoprotein measurement	Metabolic
0.8651	1.84E-138	5.41E-135	PGS001145	EFO_0005409	fat body mass	fat body mass	EFO_0005409	Other measurement	Body measurement	PGS001106	EFO_0007800	body fat percentage	body fat percentage	EFO_0007800	Body measurement	Body measurement
0.8651	1.96E-138	5.75E-135	PGS002840	EFO_0004340	body mass index	body mass index	EFO_0004340	Body measurement	Body measurement	PGS002854	EFO_0004340	body mass index	body mass index	EFO_0004340	Body measurement	Body measurement
0.865	2.20E-138	6.47E-135	PGS001235	EFO_0004324	body weights and measures	body weights and measures	EFO_0004324	Body measurement	Body measurement	PGS001159	EFO_0005106	body composition measurement	body composition measurement	EFO_0005106	Body measurement	Body measurement
0.865	2.40E-138	7.04E-135	PGS001265	EFO_0003907	deep vein thrombosis	deep vein thrombosis	EFO_0003907	Other trait	Cardiovascular	PGS001278	EFO_0003827	pulmonary embolism|deep vein thrombosis	pulmonary embolism	EFO_0003827	Cardiovascular disease	Cardiovascular
0.865	2.46E-138	7.22E-135	PGS000648	EFO_0000095	chronic lymphocytic leukemia	chronic lymphocytic leukemia	EFO_0000095	Immune system disorder	Immunological	PGS000649	EFO_0000095	chronic lymphocytic leukemia	chronic lymphocytic leukemia	EFO_0000095	Immune system disorder	Immunological
0.865	2.46E-138	7.22E-135	PGS000648	EFO_0000095	chronic lymphocytic leukemia	chronic lymphocytic leukemia	EFO_0000095	Cancer	Neoplasms	PGS000649	EFO_0000095	chronic lymphocytic leukemia	chronic lymphocytic leukemia	EFO_0000095	Cancer	Neoplasms
0.865	2.46E-138	7.22E-135	PGS000648	EFO_0000095	chronic lymphocytic leukemia	chronic lymphocytic leukemia	EFO_0000095	Immune system disorder	Immunological	PGS000649	EFO_0000095	chronic lymphocytic leukemia	chronic lymphocytic leukemia	EFO_0000095	Cancer	Neoplasms
0.865	2.46E-138	7.22E-135	PGS000648	EFO_0000095	chronic lymphocytic leukemia	chronic lymphocytic leukemia	EFO_0000095	Cancer	Neoplasms	PGS000649	EFO_0000095	chronic lymphocytic leukemia	chronic lymphocytic leukemia	EFO_0000095	Immune system disorder	Immunological
0.865	2.51E-138	7.36E-135	PGS001320	EFO_0000537	hypertension	hypertension	EFO_0000537	Cardiovascular disease	Cardiovascular	PGS000706	EFO_0000537	hypertension	hypertension	EFO_0000537	Cardiovascular disease	Cardiovascular
0.8649	2.83E-138	8.28E-135	PGS001102	EFO_0007800	body fat percentage	body fat percentage	EFO_0007800	Body measurement	Body measurement	PGS001144	EFO_0005409	fat body mass	fat body mass	EFO_0005409	Other measurement	Body measurement
0.8648	2.98E-138	8.71E-135	PGS002161	EFO_0004340	body mass index	body mass index	EFO_0004340	Body measurement	Body measurement	PGS002169	EFO_0005409	fat body mass	fat body mass	EFO_0005409	Other measurement	Body measurement
0.8648	3.24E-138	9.47E-135	PGS002337	EFO_0004611	low density lipoprotein cholesterol measurement	low density lipoprotein cholesterol measurement	EFO_0004611	Cardiovascular measurement	Cardiovascular	PGS002102	EFO_0004615	apolipoprotein B measurement	apolipoprotein B measurement	EFO_0004615	Lipid or lipoprotein measurement	Metabolic
0.8648	3.24E-138	9.47E-135	PGS002337	EFO_0004611	low density lipoprotein cholesterol measurement	low density lipoprotein cholesterol measurement	EFO_0004611	Lipid or lipoprotein measurement	Metabolic	PGS002102	EFO_0004615	apolipoprotein B measurement	apolipoprotein B measurement	EFO_0004615	Lipid or lipoprotein measurement	Metabolic
0.8648	3.31E-138	9.67E-135	PGS001836	MONDO_0005041	glaucoma	glaucoma	MONDO_0005041	Other disease	Ophthalmological	PGS002043	MONDO_0005041	glaucoma	glaucoma	MONDO_0005041	Other disease	Ophthalmological
-0.8647	3.65E-138	1.07E-134	PGS003304	EFO_0004617	cystatin C measurement|glomerular filtration rate	cystatin C measurement	EFO_0004617	Other measurement	Metabolic	PGS003298	EFO_0004617	cystatin C measurement|glomerular filtration rate	cystatin C measurement	EFO_0004617	Other measurement	Metabolic
0.8646	4.36E-138	1.27E-134	PGS001917	EFO_0007800	body fat percentage	body fat percentage	EFO_0007800	Body measurement	Body measurement	PGS002169	EFO_0005409	fat body mass	fat body mass	EFO_0005409	Other measurement	Body measurement
0.8645	5.09E-138	1.49E-134	PGS003383	EFO_0000673	prostate adenocarcinoma	prostate adenocarcinoma	EFO_0000673	Cancer	Neoplasms	PGS000583	EFO_0001663	prostate carcinoma	prostate carcinoma	EFO_0001663	Cancer	Neoplasms
0.8645	5.14E-138	1.50E-134	PGS002979	EFO_0004339	body height	body height	EFO_0004339	Body measurement	Body measurement	PGS002983	EFO_0004339	body height	body height	EFO_0004339	Body measurement	Body measurement
0.8644	5.64E-138	1.64E-134	PGS002221	EFO_0010701	mean reticulocyte volume	mean reticulocyte volume	EFO_0010701	Hematological measurement	Hematological	PGS002227	EFO_0004503	hematological measurement	hematological measurement	EFO_0004503	Hematological measurement	Hematological
0.8643	6.89E-138	2.01E-134	PGS001235	EFO_0004324	body weights and measures	body weights and measures	EFO_0004324	Body measurement	Body measurement	PGS001167	EFO_0004995	lean body mass	lean body mass	EFO_0004995	Other measurement	Skeletal
0.8643	7.30E-138	2.12E-134	PGS000592	EFO_0001663	prostate carcinoma	prostate carcinoma	EFO_0001663	Cancer	Neoplasms	PGS000572	EFO_0001663	prostate carcinoma	prostate carcinoma	EFO_0001663	Cancer	Neoplasms
0.8642	7.62E-138	2.22E-134	PGS001811	MONDO_0002278	benign colon neoplasm	benign colon neoplasm	MONDO_0002278	Cancer	Neoplasms	PGS002019	MONDO_0002278	benign colon neoplasm	benign colon neoplasm	MONDO_0002278	Cancer	Neoplasms
0.8642	7.62E-138	2.22E-134	PGS001811	MONDO_0002278	benign colon neoplasm	benign colon neoplasm	MONDO_0002278	Digestive system disorder	Gastrointestinal	PGS002019	MONDO_0002278	benign colon neoplasm	benign colon neoplasm	MONDO_0002278	Cancer	Neoplasms
0.8642	7.62E-138	2.22E-134	PGS001811	MONDO_0002278	benign colon neoplasm	benign colon neoplasm	MONDO_0002278	Digestive system disorder	Gastrointestinal	PGS002019	MONDO_0002278	benign colon neoplasm	benign colon neoplasm	MONDO_0002278	Digestive system disorder	Gastrointestinal
0.8642	7.62E-138	2.22E-134	PGS001811	MONDO_0002278	benign colon neoplasm	benign colon neoplasm	MONDO_0002278	Cancer	Neoplasms	PGS002019	MONDO_0002278	benign colon neoplasm	benign colon neoplasm	MONDO_0002278	Digestive system disorder	Gastrointestinal
0.8642	7.73E-138	2.25E-134	PGS002531	EFO_0005140	autoimmune disease	autoimmune disease	EFO_0005140	Immune system disorder	Immunological	PGS002604	EFO_0004705	hypothyroidism	hypothyroidism	EFO_0004705	Other disease	Endocrine
0.864	1.10E-137	3.19E-134	PGS002672	EFO_0004631	vitamin D measurement	vitamin D measurement	EFO_0004631	Lipid or lipoprotein measurement	Metabolic	PGS001982	EFO_0004631	vitamin D measurement	vitamin D measurement	EFO_0004631	Lipid or lipoprotein measurement	Metabolic
0.864	1.11E-137	3.22E-134	PGS000482	EFO_0000305	breast carcinoma	breast carcinoma	EFO_0000305	Cancer	Neoplasms	PGS000478	EFO_0000305	breast carcinoma	breast carcinoma	EFO_0000305	Cancer	Neoplasms
0.8639	1.28E-137	3.72E-134	PGS000900	EFO_0006335	systolic blood pressure|female	systolic blood pressure	EFO_0006335	Other measurement	Cardiovascular	PGS003073	EFO_0006335	systolic blood pressure	systolic blood pressure	EFO_0006335	Other measurement	Cardiovascular
0.8639	1.30E-137	3.78E-134	PGS002993	EFO_0004339	body height	body height	EFO_0004339	Body measurement	Body measurement	PGS002979	EFO_0004339	body height	body height	EFO_0004339	Body measurement	Body measurement
0.8639	1.32E-137	3.82E-134	PGS002171	EFO_0004541	HbA1c measurement	HbA1c measurement	EFO_0004541	Hematological measurement	Hematological	PGS002331	EFO_0004541	HbA1c measurement	HbA1c measurement	EFO_0004541	Hematological measurement	Hematological
0.8638	1.60E-137	4.62E-134	PGS001983	EFO_0004342	waist circumference	waist circumference	EFO_0004342	Body measurement	Body measurement	PGS001951	EFO_0005409	fat body mass	fat body mass	EFO_0005409	Other measurement	Body measurement
0.8636	1.98E-137	5.74E-134	PGS001301	EFO_0001060	celiac disease	celiac disease	EFO_0001060	Digestive system disorder	Gastrointestinal	PGS001065	EFO_0008111	diet measurement	diet measurement	EFO_0008111	Other measurement	Activities
0.8636	1.98E-137	5.74E-134	PGS001301	EFO_0001060	celiac disease	celiac disease	EFO_0001060	Immune system disorder	Immunological	PGS001065	EFO_0008111	diet measurement	diet measurement	EFO_0008111	Other measurement	Activities
0.8636	2.05E-137	5.93E-134	PGS001476	EFO_0006930	brain volume measurement|neuroimaging measurement	brain volume measurement	EFO_0006930	Other measurement	Neurological	PGS001477	EFO_0006930	measurement	brain volume measurement	EFO_0006930	Other measurement	Neurological
0.8636	2.07E-137	6.00E-134	PGS002840	EFO_0004340	body mass index	body mass index	EFO_0004340	Body measurement	Body measurement	PGS002844	EFO_0004340	body mass index	body mass index	EFO_0004340	Body measurement	Body measurement
0.8635	2.41E-137	6.96E-134	PGS001368	EFO_0004731	eye measurement	eye measurement	EFO_0004731	Other measurement	Ophthalmological	PGS001362	EFO_0004731	eye measurement	eye measurement	EFO_0004731	Other measurement	Ophthalmological
0.8634	2.70E-137	7.80E-134	PGS002596	EFO_0004468	glucose measurement	glucose measurement	EFO_0004468	Other measurement	Metabolic	PGS002449	EFO_0004468	glucose measurement	glucose measurement	EFO_0004468	Other measurement	Metabolic
0.8634	2.70E-137	7.80E-134	PGS000508	EFO_0000305	breast carcinoma	breast carcinoma	EFO_0000305	Cancer	Neoplasms	PGS000512	EFO_0000305	breast carcinoma	breast carcinoma	EFO_0000305	Cancer	Neoplasms
0.8632	3.61E-137	1.04E-133	PGS001360	EFO_0004731	eye measurement	eye measurement	EFO_0004731	Other measurement	Ophthalmological	PGS001362	EFO_0004731	eye measurement	eye measurement	EFO_0004731	Other measurement	Ophthalmological
0.8632	3.94E-137	1.14E-133	PGS002008	EFO_0004503	hematological measurement	hematological measurement	EFO_0004503	Hematological measurement	Hematological	PGS002003	EFO_0010701	mean reticulocyte volume	mean reticulocyte volume	EFO_0010701	Hematological measurement	Hematological
0.8631	4.59E-137	1.32E-133	PGS002506	EFO_0004705	hypothyroidism	hypothyroidism	EFO_0004705	Other disease	Endocrine	PGS002521	EFO_1000627	thyroid disease	thyroid disease	EFO_1000627	Other disease	Endocrine
0.863	4.81E-137	1.38E-133	PGS001751	EFO_0006930	brain volume measurement|neuroimaging measurement	brain volume measurement	EFO_0006930	Other measurement	Neurological	PGS001736	EFO_0006930	measurement	brain volume measurement	EFO_0006930	Other measurement	Neurological
0.8629	6.07E-137	1.74E-133	PGS001146	EFO_0005409	fat body mass	fat body mass	EFO_0005409	Other measurement	Body measurement	PGS001159	EFO_0005106	body composition measurement	body composition measurement	EFO_0005106	Body measurement	Body measurement
0.8628	7.04E-137	2.02E-133	PGS002428	EFO_0004343	waist-hip ratio	waist-hip ratio	EFO_0004343	Body measurement	Body measurement	PGS002477	EFO_0004343	waist-hip ratio	waist-hip ratio	EFO_0004343	Body measurement	Body measurement
0.8627	8.71E-137	2.50E-133	PGS001155	EFO_0005106	body composition measurement	body composition measurement	EFO_0005106	Body measurement	Body measurement	PGS001161	EFO_0005106	body composition measurement	body composition measurement	EFO_0005106	Body measurement	Body measurement
0.8626	9.04E-137	2.59E-133	PGS000344	EFO_0000305	breast carcinoma	breast carcinoma	EFO_0000305	Cancer	Neoplasms	PGS000214	MONDO_0021115	luminal B breast carcinoma	luminal B breast carcinoma	MONDO_0021115	Cancer	Neoplasms
0.8626	9.33E-137	2.68E-133	PGS002357	EFO_0004308	leukocyte count	leukocyte count	EFO_0004308	Hematological measurement	Hematological	PGS002180	EFO_0004308	leukocyte count	leukocyte count	EFO_0004308	Hematological measurement	Hematological
0.8626	9.82E-137	2.82E-133	PGS000569	EFO_0001663	prostate carcinoma	prostate carcinoma	EFO_0001663	Cancer	Neoplasms	PGS000571	EFO_0001663	prostate carcinoma	prostate carcinoma	EFO_0001663	Cancer	Neoplasms
0.8624	1.31E-136	3.77E-133	PGS002419	EFO_0000684	respiratory system disease	respiratory system disease	EFO_0000684	Other disease	Skeletal	PGS002383	MONDO_0004979	asthma	asthma	MONDO_0004979	Other disease	Respiratory
0.8623	1.45E-136	4.17E-133	PGS001275	EFO_0003839	retinopathy	retinopathy	EFO_0003839	Neurological disorder	Neurological	PGS001834	EFO_0001365	age-related macular degeneration	age-related macular degeneration	EFO_0001365	Neurological disorder	Neurological
0.8623	1.58E-136	4.53E-133	PGS001297	MONDO_0005147	type 1 diabetes mellitus	type 1 diabetes mellitus	MONDO_0005147	Digestive system disorder	Gastrointestinal	PGS001296	MONDO_0005147	type 1 diabetes mellitus	type 1 diabetes mellitus	MONDO_0005147	Immune system disorder	Immunological
0.8623	1.58E-136	4.53E-133	PGS001297	MONDO_0005147	type 1 diabetes mellitus	type 1 diabetes mellitus	MONDO_0005147	Immune system disorder	Immunological	PGS001296	MONDO_0005147	type 1 diabetes mellitus	type 1 diabetes mellitus	MONDO_0005147	Digestive system disorder	Gastrointestinal
0.8623	1.58E-136	4.53E-133	PGS001297	MONDO_0005147	type 1 diabetes mellitus	type 1 diabetes mellitus	MONDO_0005147	Metabolic disorder	Metabolic	PGS001296	MONDO_0005147	type 1 diabetes mellitus	type 1 diabetes mellitus	MONDO_0005147	Metabolic disorder	Metabolic
0.8623	1.58E-136	4.53E-133	PGS001297	MONDO_0005147	type 1 diabetes mellitus	type 1 diabetes mellitus	MONDO_0005147	Digestive system disorder	Gastrointestinal	PGS001296	MONDO_0005147	type 1 diabetes mellitus	type 1 diabetes mellitus	MONDO_0005147	Metabolic disorder	Metabolic
0.8623	1.58E-136	4.53E-133	PGS001297	MONDO_0005147	type 1 diabetes mellitus	type 1 diabetes mellitus	MONDO_0005147	Digestive system disorder	Gastrointestinal	PGS001296	MONDO_0005147	type 1 diabetes mellitus	type 1 diabetes mellitus	MONDO_0005147	Digestive system disorder	Gastrointestinal

0.8623	1.58E-136	4.53E-133	PGS001297	MONDO_0005147	type 1 diabetes mellitus	type 1 diabetes mellitus	MONDO_0005147	Metabolic disorder	Metabolic	PGS001296	MONDO_0005147	type 1 diabetes mellitus	type 1 diabetes mellitus	MONDO_0005147	Digestive system disorder	Gastrointestinal
0.8623	1.58E-136	4.53E-133	PGS001297	MONDO_0005147	type 1 diabetes mellitus	type 1 diabetes mellitus	MONDO_0005147	Metabolic disorder	Metabolic	PGS001296	MONDO_0005147	type 1 diabetes mellitus	type 1 diabetes mellitus	MONDO_0005147	Immune system disorder	Immunological
0.8623	1.58E-136	4.53E-133	PGS001297	MONDO_0005147	type 1 diabetes mellitus	type 1 diabetes mellitus	MONDO_0005147	Immune system disorder	Immunological	PGS001296	MONDO_0005147	type 1 diabetes mellitus	type 1 diabetes mellitus	MONDO_0005147	Immune system disorder	Immunological
0.8623	1.58E-136	4.53E-133	PGS001297	MONDO_0005147	type 1 diabetes mellitus	type 1 diabetes mellitus	MONDO_0005147	Immune system disorder	Immunological	PGS001296	MONDO_0005147	type 1 diabetes mellitus	type 1 diabetes mellitus	MONDO_0005147	Metabolic disorder	Metabolic
0.8622	1.73E-136	4.95E-133	PGS001530	EFO_0007772	calcaneal bone quantitative ultrasound measurement	calcaneal bone quantitative ultrasound measurement	EFO_0007772	Other measurement	Skeletal	PGS001404	EFO_0009270	heel bone mineral density	heel bone mineral density	EFO_0009270	Other measurement	Skeletal
0.8622	1.79E-136	5.11E-133	PGS001146	EFO_0005409	fat body mass	fat body mass	EFO_0005409	Other measurement	Body measurement	PGS001167	EFO_0004995	lean body mass	lean body mass	EFO_0004995	Other measurement	Skeletal
0.862	2.24E-136	6.41E-133	PGS000539	EFO_0000305	breast carcinoma	breast carcinoma	EFO_0000305	Cancer	Neoplasms	PGS000532	EFO_0000305	breast carcinoma	breast carcinoma	EFO_0000305	Cancer	Neoplasms
0.8619	2.62E-136	7.49E-133	PGS002382	EFO_0004703	age at menarche	age at menarche	EFO_0004703	Other measurement	Genitourinary	PGS002529	EFO_0004703	age at menarche	age at menarche	EFO_0004703	Other measurement	Genitourinary
0.8619	3.01E-136	8.58E-133	PGS001366	EFO_0004731	eye measurement	eye measurement	EFO_0004731	Other measurement	Ophthalmological	PGS001359	EFO_0004731	eye measurement	eye measurement	EFO_0004731	Other measurement	Ophthalmological
0.8618	3.06E-136	8.74E-133	PGS002445	EFO_0001379	endocrine system disease|diabetes mellitus	endocrine system disease	EFO_0001379	Other disease	Metabolic	PGS002452	EFO_0004541	HbA1c measurement	HbA1c measurement	EFO_0004541	Hematological measurement	Hematological
0.8618	3.27E-136	9.33E-133	PGS001234	EFO_0004324	body weights and measures	body weights and measures	EFO_0004324	Body measurement	Body measurement	PGS001159	EFO_0005106	body composition measurement	body composition measurement	EFO_0005106	Body measurement	Body measurement
0.8617	3.65E-136	1.04E-132	PGS000480	EFO_0000305	breast carcinoma	breast carcinoma	EFO_0000305	Cancer	Neoplasms	PGS000513	EFO_0000305	breast carcinoma	breast carcinoma	EFO_0000305	Cancer	Neoplasms
0.8616	4.36E-136	1.24E-132	PGS001788	EFO_0000341	chronic obstructive pulmonary disease	chronic obstructive pulmonary disease	EFO_0000341	Other disease	Respiratory	PGS001783	EFO_0000341	chronic obstructive pulmonary disease	chronic obstructive pulmonary disease	EFO_0000341	Other disease	Respiratory
0.8616	4.42E-136	1.26E-132	PGS000592	EFO_0001663	prostate carcinoma	prostate carcinoma	EFO_0001663	Cancer	Neoplasms	PGS000576	EFO_0001663	prostate carcinoma	prostate carcinoma	EFO_0001663	Cancer	Neoplasms
0.8616	4.71E-136	1.34E-132	PGS001301	EFO_0001060	celiac disease	celiac disease	EFO_0001060	Digestive system disorder	Gastrointestinal	PGS000940	HP_0002024	Malabsorption	Malabsorption	HP_0002024	Other trait	Gastrointestinal
0.8616	4.71E-136	1.34E-132	PGS001301	EFO_0001060	celiac disease	celiac disease	EFO_0001060	Immune system disorder	Immunological	PGS000940	HP_0002024	Malabsorption	Malabsorption	HP_0002024	Other trait	Gastrointestinal
0.8616	4.73E-136	1.35E-132	PGS002194	EFO_0007986	reticulocyte count	reticulocyte count	EFO_0007986	Hematological measurement	Hematological	PGS001959	EFO_0010700	reticulocyte measurement	reticulocyte measurement	EFO_0010700	Hematological measurement	Hematological
0.8614	5.80E-136	1.65E-132	PGS000560	EFO_0003893	ovarian neoplasm	ovarian neoplasm	EFO_0003893	Cancer	Neoplasms	PGS000563	EFO_0003893	ovarian neoplasm	ovarian neoplasm	EFO_0003893	Cancer	Neoplasms
0.8614	5.80E-136	1.65E-132	PGS000563	EFO_0003893	ovarian neoplasm	ovarian neoplasm	EFO_0003893	Cancer	Neoplasms	PGS000559	EFO_0003893	ovarian neoplasm	ovarian neoplasm	EFO_0003893	Cancer	Neoplasms
0.8613	6.66E-136	1.89E-132	PGS001094	EFO_0007822	hair colour measurement	hair colour measurement	EFO_0007822	Other measurement	Dermatological	PGS001095	EFO_0007822	hair colour measurement	hair colour measurement	EFO_0007822	Other measurement	Dermatological
0.8611	9.71E-136	2.75E-132	PGS002855	EFO_0004340	body mass index	body mass index	EFO_0004340	Body measurement	Body measurement	PGS002859	EFO_0004340	body mass index	body mass index	EFO_0004340	Body measurement	Body measurement
0.861	1.12E-135	3.16E-132	PGS000621	MONDO_0001657	brain cancer	brain cancer	MONDO_0001657	Cancer	Neoplasms	PGS000624	MONDO_0001657	brain cancer	brain cancer	MONDO_0001657	Neurological disorder	Neurological
0.861	1.12E-135	3.16E-132	PGS000624	MONDO_0001657	brain cancer	brain cancer	MONDO_0001657	Neurological disorder	Neurological	PGS000622	MONDO_0001657	brain cancer	brain cancer	MONDO_0001657	Neurological disorder	Neurological
0.861	1.12E-135	3.16E-132	PGS000621	MONDO_0001657	brain cancer	brain cancer	MONDO_0001657	Cancer	Neoplasms	PGS000624	MONDO_0001657	brain cancer	brain cancer	MONDO_0001657	Cancer	Neoplasms
0.861	1.12E-135	3.16E-132	PGS000624	MONDO_0001657	brain cancer	brain cancer	MONDO_0001657	Cancer	Neoplasms	PGS000622	MONDO_0001657	brain cancer	brain cancer	MONDO_0001657	Neurological disorder	Neurological
0.861	1.12E-135	3.16E-132	PGS000621	MONDO_0001657	brain cancer	brain cancer	MONDO_0001657	Neurological disorder	Neurological	PGS000624	MONDO_0001657	brain cancer	brain cancer	MONDO_0001657	Neurological disorder	Neurological
0.861	1.12E-135	3.16E-132	PGS000624	MONDO_0001657	brain cancer	brain cancer	MONDO_0001657	Cancer	Neoplasms	PGS000622	MONDO_0001657	brain cancer	brain cancer	MONDO_0001657	Cancer	Neoplasms
0.861	1.12E-135	3.16E-132	PGS000621	MONDO_0001657	brain cancer	brain cancer	MONDO_0001657	Neurological disorder	Neurological	PGS000624	MONDO_0001657	brain cancer	brain cancer	MONDO_0001657	Cancer	Neoplasms
0.861	1.12E-135	3.16E-132	PGS000624	MONDO_0001657	brain cancer	brain cancer	MONDO_0001657	Neurological disorder	Neurological	PGS000622	MONDO_0001657	brain cancer	brain cancer	MONDO_0001657	Cancer	Neoplasms
0.861	1.12E-135	3.16E-132	PGS000623	MONDO_0001657	brain cancer	brain cancer	MONDO_0001657	Neurological disorder	Neurological	PGS000624	MONDO_0001657	brain cancer	brain cancer	MONDO_0001657	Cancer	Neoplasms
0.861	1.12E-135	3.16E-132	PGS000623	MONDO_0001657	brain cancer	brain cancer	MONDO_0001657	Cancer	Neoplasms	PGS000624	MONDO_0001657	brain cancer	brain cancer	MONDO_0001657	Neurological disorder	Neurological
0.861	1.12E-135	3.16E-132	PGS000623	MONDO_0001657	brain cancer	brain cancer	MONDO_0001657	Cancer	Neoplasms	PGS000624	MONDO_0001657	brain cancer	brain cancer	MONDO_0001657	Cancer	Neoplasms
0.861	1.12E-135	3.16E-132	PGS000623	MONDO_0001657	brain cancer	brain cancer	MONDO_0001657	Neurological disorder	Neurological	PGS000624	MONDO_0001657	brain cancer	brain cancer	MONDO_0001657	Neurological disorder	Neurological
0.8609	1.24E-135	3.50E-132	PGS001234	EFO_0004324	body weights and measures	body weights and measures	EFO_0004324	Body measurement	Body measurement	PGS001167	EFO_0004995	lean body mass	lean body mass	EFO_0004995	Other measurement	Skeletal
0.8609	1.37E-135	3.88E-132	PGS000594	EFO_0007355	male reproductive organ cancer	male reproductive organ cancer	EFO_0007355	Cancer	Neoplasms	PGS000571	EFO_0001663	prostate carcinoma	prostate carcinoma	EFO_0001663	Cancer	Neoplasms
0.8608	1.56E-135	4.40E-132	PGS003398	EFO_0000305	breast carcinoma	breast carcinoma	EFO_0000305	Cancer	Neoplasms	PGS000873	EFO_0000305	breast carcinoma	breast carcinoma	EFO_0000305	Cancer	Neoplasms
0.8604	2.61E-135	7.37E-132	PGS002596	EFO_0004468	glucose measurement	glucose measurement	EFO_0004468	Other measurement	Metabolic	PGS002442	EFO_0000400	diabetes mellitus	diabetes mellitus	EFO_0000400	Metabolic disorder	Metabolic
0.8604	2.61E-135	7.37E-132	PGS002596	EFO_0004468	glucose measurement	glucose measurement	EFO_0004468	Other measurement	Metabolic	PGS002442	EFO_0000400	diabetes mellitus	diabetes mellitus	EFO_0000400	Digestive system disorder	Gastrointestinal
0.8603	3.04E-135	8.57E-132	PGS003258	EFO_0010472	creatine measurement|glomerular filtration rate	creatine measurement	EFO_0010472	Other measurement	Metabolic	PGS003269	EFO_0010472	creatine measurement|glomerular filtration rate	creatine measurement	EFO_0010472	Other measurement	Metabolic
0.8602	3.49E-135	9.86E-132	PGS003287	EFO_0004617	cystatin C measurement	cystatin C measurement	EFO_0004617	Other measurement	Metabolic	PGS003282	EFO_0004617	cystatin C measurement	cystatin C measurement	EFO_0004617	Other measurement	Metabolic
0.8602	3.80E-135	1.07E-131	PGS001758	EFO_0006930	brain volume measurement|neuroimaging measurement	brain volume measurement	EFO_0006930	Other measurement	Neurological	PGS001743	EFO_0006930	measurement	brain volume measurement	EFO_0006930	Other measurement	Neurological
-0.8602	3.93E-135	1.11E-131	PGS003313	EFO_0004617	cystatin C measurement|glomerular filtration rate	cystatin C measurement	EFO_0004617	Other measurement	Metabolic	PGS003304	EFO_0004617	cystatin C measurement|glomerular filtration rate	cystatin C measurement	EFO_0004617	Other measurement	Metabolic
0.86	4.66E-135	1.31E-131	PGS002845	EFO_0004340	body mass index	body mass index	EFO_0004340	Body measurement	Body measurement	PGS002859	EFO_0004340	body mass index	body mass index	EFO_0004340	Body measurement	Body measurement
0.86	5.04E-135	1.42E-131	PGS002393	EFO_0000400	diabetes mellitus	diabetes mellitus	EFO_0000400	Metabolic disorder	Metabolic	PGS002491	EFO_0000400	diabetes mellitus	diabetes mellitus	EFO_0000400	Metabolic disorder	Metabolic
0.86	5.04E-135	1.42E-131	PGS002393	EFO_0000400	diabetes mellitus	diabetes mellitus	EFO_0000400	Digestive system disorder	Gastrointestinal	PGS002491	EFO_0000400	diabetes mellitus	diabetes mellitus	EFO_0000400	Digestive system disorder	Gastrointestinal
0.86	5.04E-135	1.42E-131	PGS002393	EFO_0000400	diabetes mellitus	diabetes mellitus	EFO_0000400	Digestive system disorder	Gastrointestinal	PGS002491	EFO_0000400	diabetes mellitus	diabetes mellitus	EFO_0000400	Metabolic disorder	Metabolic
0.86	5.04E-135	1.42E-131	PGS002393	EFO_0000400	diabetes mellitus	diabetes mellitus	EFO_0000400	Metabolic disorder	Metabolic	PGS002491	EFO_0000400	diabetes mellitus	diabetes mellitus	EFO_0000400	Digestive system disorder	Gastrointestinal
-0.86	5.23E-135	1.47E-131	PGS002155	EFO_0004279	suntan	suntan	EFO_0004279	Other trait	Dermatological	PGS001897	EFO_0003784	skin pigmentation	skin pigmentation	EFO_0003784	Other trait	Dermatological
0.8598	6.49E-135	1.82E-131	PGS003091	MONDO_0005148	type 2 diabetes mellitus	type 2 diabetes mellitus	MONDO_0005148	Metabolic disorder	Metabolic	PGS003090	MONDO_0005148	type 2 diabetes mellitus	type 2 diabetes mellitus	MONDO_0005148	Digestive system disorder	Gastrointestinal
0.8598	6.49E-135	1.82E-131	PGS003091	MONDO_0005148	type 2 diabetes mellitus	type 2 diabetes mellitus	MONDO_0005148	Metabolic disorder	Metabolic	PGS003090	MONDO_0005148	type 2 diabetes mellitus	type 2 diabetes mellitus	MONDO_0005148	Metabolic disorder	Metabolic
0.8598	6.49E-135	1.82E-131	PGS003091	MONDO_0005148	type 2 diabetes mellitus	type 2 diabetes mellitus	MONDO_0005148	Digestive system disorder	Gastrointestinal	PGS003090	MONDO_0005148	type 2 diabetes mellitus	type 2 diabetes mellitus	MONDO_0005148	Metabolic disorder	Metabolic
0.8598	6.49E-135	1.82E-131	PGS003091	MONDO_0005148	type 2 diabetes mellitus	type 2 diabetes mellitus	MONDO_0005148	Digestive system disorder	Gastrointestinal	PGS003090	MONDO_0005148	type 2 diabetes mellitus	type 2 diabetes mellitus	MONDO_0005148	Digestive system disorder	Gastrointestinal
0.8598	7.22E-135	2.03E-131	PGS003078	EFO_0006335	systolic blood pressure	systolic blood pressure	EFO_0006335	Other measurement	Cardiovascular	PGS000900	EFO_0006335	systolic blood pressure|female	systolic blood pressure	EFO_0006335	Other measurement	Cardiovascular
0.8597	7.25E-135	2.04E-131	PGS002126	EFO_0006527	smoking status measurement	smoking status measurement	EFO_0006527	Other measurement	Psychiatric	PGS001911	EFO_0006527	smoking status measurement	smoking status measurement	EFO_0006527	Other measurement	Psychiatric
0.8597	7.52E-135	2.11E-131	PGS002844	EFO_0004340	body mass index	body mass index	EFO_0004340	Body measurement	Body measurement	PGS002850	EFO_0004340	body mass index	body mass index	EFO_0004340	Body measurement	Body measurement
0.8597	7.62E-135	2.14E-131	PGS001145	EFO_0005409	fat body mass	fat body mass	EFO_0005409	Other measurement	Body measurement	PGS001148	EFO_0005409	fat body mass	fat body mass	EFO_0005409	Other measurement	Body measurement
0.8596	8.69E-135	2.44E-131	PGS002225	EFO_0008341	snoring measurement	snoring measurement	EFO_0008341	Other measurement	Psychiatric	PGS002006	EFO_0008341	snoring measurement	snoring measurement	EFO_0008341	Other measurement	Psychiatric
0.8595	9.90E-135	2.77E-131	PGS001674	EFO_0006930	brain volume measurement|neuroimaging measurement	brain volume measurement	EFO_0006930	Other measurement	Neurological	PGS001672	EFO_0006930	measurement	brain volume measurement	EFO_0006930	Other measurement	Neurological
0.8595	1.06E-134	2.96E-131	PGS000539	EFO_0000305	breast carcinoma	breast carcinoma	EFO_0000305	Cancer	Neoplasms	PGS000531	EFO_0000305	breast carcinoma	breast carcinoma	EFO_0000305	Cancer	Neoplasms
-0.8593	1.44E-134	4.03E-131	PGS003310	EFO_0004617	cystatin C measurement|glomerular filtration rate	cystatin C measurement	EFO_0004617	Other measurement	Metabolic	PGS003304	EFO_0004617	cystatin C measurement|glomerular filtration rate	cystatin C measurement	EFO_0004617	Other measurement	Metabolic
-0.8592	1.62E-134	4.53E-131	PGS003123	MONDO_0005148	type 2 diabetes mellitus	type 2 diabetes mellitus	MONDO_0005148	Metabolic disorder	Metabolic	PGS001357	MONDO_0005148	type 2 diabetes mellitus	type 2 diabetes mellitus	MONDO_0005148	Metabolic disorder	Metabolic
-0.8592	1.62E-134	4.53E-131	PGS003123	MONDO_0005148	type 2 diabetes mellitus	type 2 diabetes mellitus	MONDO_0005148	Metabolic disorder	Metabolic	PGS001357	MONDO_0005148	type 2 diabetes mellitus	type 2 diabetes mellitus	MONDO_0005148	Digestive system disorder	Gastrointestinal
-0.8592	1.62E-134	4.53E-131	PGS003123	MONDO_0005148	type 2 diabetes mellitus	type 2 diabetes mellitus	MONDO_0005148	Digestive system disorder	Gastrointestinal	PGS001357	MONDO_0005148	type 2 diabetes mellitus	type 2 diabetes mellitus	MONDO_0005148	Metabolic disorder	Metabolic
-0.8592	1.62E-134	4.53E-131	PGS003123	MONDO_0005148	type 2 diabetes mellitus	type 2 diabetes mellitus	MONDO_0005148	Digestive system disorder	Gastrointestinal	PGS001357	MONDO_0005148	type 2 diabetes mellitus	type 2 diabetes mellitus	MONDO_0005148	Digestive system disorder	Gastrointestinal
0.8591	1.84E-134	5.14E-131	PGS000296	EFO_0001645	coronary artery disease	coronary artery disease	EFO_0001645	Cardiovascular disease	Cardiovascular	PGS001355	EFO_0001645	coronary artery disease	coronary artery disease	EFO_0001645	Cardiovascular disease	Cardiovascular
-0.8591	1.86E-134	5.19E-131	PGS003304	EFO_0004617	cystatin C measurement|glomerular filtration rate	cystatin C measurement	EFO_0004617	Other measurement	Metabolic	PGS003309	EFO_0004617	cystatin C measurement|glomerular filtration rate	cystatin C measurement	EFO_0004617	Other measurement	Metabolic
0.859	2.11E-134	5.89E-131	PGS001956	EFO_0007772	calcaneal bone quantitative ultrasound measurement	calcaneal bone quantitative ultrasound measurement	EFO_0007772	Other measurement	Skeletal	PGS002175	EFO_0005654	velocity of sound measurement	velocity of sound measurement	EFO_0005654	Other measurement	Others
0.859	2.17E-134	6.05E-131	PGS001149	EFO_0005409	fat body mass	fat body mass	EFO_0005409	Other measurement	Body measurement	PGS001227	EFO_0004342	waist circumference	waist circumference	EFO_0004342	Body measurement	Body measurement
0.859	2.21E-134	6.15E-131	PGS001314	EFO_0000612	myocardial infarction	myocardial infarction	EFO_0000612	Cardiovascular disease	Cardiovascular	PGS001315	EFO_0000612	myocardial infarction	myocardial infarction	EFO_0000612	Cardiovascular disease	Cardiovascular
0.8589	2.40E-134	6.69E-131	PGS001039	EFO_0009270	heel bone mineral density	heel bone mineral density	EFO_0009270	Other measurement	Skeletal	PGS001530	EFO_0007772	measurement	measurement	EFO_0007772	Other measurement	Skeletal
0.8589	2.41E-134	6.71E-131	PGS000956	EFO_0004514	bone quantitative ultrasound measurement	bone quantitative ultrasound measurement	EFO_0004514	Other measurement	Skeletal	PGS001530	EFO_0007772	measurement	measurement	EFO_0007772	Other measurement	Skeletal
0.8583	6.51E-134	1.81E-130	PGS002592	EFO_0001379	endocrine system disease|diabetes mellitus	endocrine system disease	EFO_0001379	Other disease	Metabolic	PGS002449	EFO_0004468	glucose measurement	glucose measurement	EFO_0004468	Other measurement	Metabolic
0.8583	6.64E-134	1.85E-130	PGS000633	EFO_0002892	thyroid carcinoma	thyroid carcinoma	EFO_0002892	Cancer	Neoplasms	PGS000630	EFO_0002892	thyroid carcinoma	thyroid carcinoma	EFO_0002892	Cancer	Neoplasms
0.8583	6.64E-134	1.85E-130	PGS000633	EFO_0002892	thyroid carcinoma	thyroid carcinoma	EFO_0002892	Cancer	Neoplasms	PGS000631	EFO_0002892	thyroid carcinoma	thyroid carcinoma	EFO_0002892	Cancer	Neoplasms
0.8582	7.63E-134	2.12E-130	PGS003190	EFO_0004631	vitamin D measurement	vitamin D measurement	EFO_0004631	Lipid or lipoprotein measurement	Metabolic	PGS002574	EFO_0004631	vitamin D measurement	vitamin D measurement	EFO_0004631	Lipid or lipoprotein measurement	Metabolic
0.8581	7.98E-134	2.22E-130	PGS002961	EFO_0004612	high density lipoprotein cholesterol measurement	high density lipoprotein cholesterol measurement	EFO_0004612	Lipid or lipoprotein measurement	Metabolic	PGS002960	EFO_0004612	high density lipoprotein cholesterol measurement	measurement	EFO_0004612	Lipid or lipoprotein measurement	Metabolic
0.8581	7.99E-134	2.22E-130	PGS000310	EFO_0004611	low density lipoprotein cholesterol measurement	low density lipoprotein cholesterol measurement	EFO_0004611	Lipid or lipoprotein measurement	Metabolic	PGS000065	EFO_0004611	low density lipoprotein cholesterol measurement	measurement	EFO_0004611	Cardiovascular measurement	Cardiovascular
0.8581	7.99E-134	2.22E-130	PGS000310	EFO_0004611	low density lipoprotein cholesterol measurement	low density lipoprotein cholesterol measurement	EFO_0004611	Cardiovascular measurement	Cardiovascular	PGS000065	EFO_0004611	low density lipoprotein cholesterol measurement	measurement	EFO_0004611	Cardiovascular measurement	Cardiovascular
0.8581	7.99E-134	2.22E-130	PGS000310	EFO_0004611	low density lipoprotein cholesterol measurement	low density lipoprotein cholesterol measurement	EFO_0004611	Lipid or lipoprotein measurement	Metabolic	PGS000065	EFO_0004611	low density lipoprotein cholesterol measurement	measurement	EFO_0004611	Lipid or lipoprotein measurement	Metabolic
0.8581	7.99E-134	2.22E-130	PGS000310	EFO_0004611	low density lipoprotein cholesterol measurement	low density lipoprotein cholesterol measurement	EFO_0004611	Cardiovascular measurement	Cardiovascular	PGS000065	EFO_0004611	low density lipoprotein cholesterol measurement	measurement	EFO_0004611	Lipid or lipoprotein measurement	Metabolic
0.8579	1.08E-133	2.99E-130	PGS002421	EFO_0006335	systolic blood pressure	systolic blood pressure	EFO_0006335	Other measurement	Cardiovascular	PGS002617	EFO_0006335	systolic blood pressure	systolic blood pressure	EFO_0006335	Other measurement	Cardiovascular
0.8579	1.17E-133	3.25E-130	PGS001230	EFO_0004338	body weight	body weight	EFO_0004338	Body measurement	Body measurement	PGS001162	EFO_0005093	hip circumference	hip circumference	EFO_0005093	Body measurement	Body measurement
0.8579	1.22E-133	3.38E-130	PGS002173	EFO_0009270	heel bone mineral density	heel bone mineral density	EFO_0009270	Other measurement	Skeletal	PGS002315	EFO_0003923	bone density	bone density	EFO_0003923	Other measurement	Skeletal
0.8578	1.28E-133	3.53E-130	PGS000783	EFO_0000305	breast carcinoma	breast carcinoma	EFO_0000305	Cancer	Neoplasms	PGS000774	EFO_1000649	estrogen-receptor positive breast cancer	cancer	EFO_1000649	Cancer	Neoplasms
0.8578	1.42E-133	3.93E-130	PGS002852	EFO_0004340	body mass index	body mass index	EFO_0004340	Body measurement	Body measurement	PGS002841	EFO_0004340	body mass index	body mass index	EFO_0004340	Body measurement	Body measurement
0.8577	1.56E-133	4.32E-130	PGS001363	EFO_0004731	eye measurement	eye measurement	EFO_0004731	Other measurement	Ophthalmological	PGS001359	EFO_0004731	eye measurement	eye measurement	EFO_0004731	Other measurement	Ophthalmological
0.8577	1.58E-133	4.35E-130	PGS003150	EFO_0004530	triglyceride measurement	triglyceride measurement	EFO_0004530	Lipid or lipoprotein measurement	Metabolic	PGS003145	EFO_0004530	triglyceride measurement	triglyceride measurement	EFO_0004530	Lipid or lipoprotein measurement	Metabolic
-0.8576	1.71E-133	4.73E-130	PGS002834	EFO_0007878	alcohol consumption measurement	alcohol consumption measurement	EFO_0007878	Other measurement	Psychiatric	PGS003376	EFO_0007878	alcohol consumption measurement	alcohol consumption measurement	EFO_0007878	Other measurement	Psychiatric
0.8576	1.85E-133	5.10E-130	PGS002840	EFO_0004340	body mass index	body mass index	EFO_0004340	Body measurement	Body measurement	PGS002859	EFO_0004340	body mass index	body mass index	EFO_0004340	Body measurement	Body measurement
0.8575	1.93E-133	5.32E-130	PGS002385	EFO_0004340	body mass index	body mass index	EFO_0004340	Body measurement	Body measurement	PGS002532	EFO_0004340	body mass index	body mass index	EFO_0004340	Body measurement	Body measurement
-0.8575	1.95E-133	5.38E-130	PGS001141	EFO_0005422	skin aging	skin aging	EFO_0005422	Biological process	Dermatological	PGS001071	EFO_0008006	skin aging measurement	skin aging measurement	EFO_0008006	Other measurement	Dermatological
0.8575	1.99E-133	5.49E-130	PGS002473	EFO_0004574	total cholesterol measurement	total cholesterol measurement	EFO_0004574	Lipid or lipoprotein measurement	Metabolic	PGS002458	EFO_0004611	low density lipoprotein cholesterol measurement	measurement	EFO_0004611	Cardiovascular measurement	Cardiovascular
0.8575	1.99E-133	5.49E-130	PGS002473	EFO_0004574	total cholesterol measurement	total cholesterol measurement	EFO_0004574	Lipid or lipoprotein measurement	Metabolic	PGS002458	EFO_0004611	low density lipoprotein cholesterol measurement	measurement	EFO_0004611	Lipid or lipoprotein measurement	Metabolic
0.8575	2.01E-133	5.55E-130	PGS001944	EFO_0007934	creatinine clearance measurement	creatinine clearance measurement	EFO_0007934	Other measurement	Metabolic	PGS002162	EFO_0007934	creatinine clearance measurement	creatinine clearance measurement	EFO_0007934	Other measurement	Metabolic
-0.8574	2.27E-133	6.24E-130	PGS001357	MONDO_0005148	type 2 diabetes mellitus	type 2 diabetes mellitus	MONDO_0005148	Metabolic disorder	Metabolic	PGS003108	MONDO_0005148	type 2 diabetes mellitus	type 2 diabetes mellitus	MONDO_0005148	Metabolic disorder	Metabolic
-0.8574	2.27E-133	6.24E-130	PGS001357	MONDO_0005148	type 2 diabetes mellitus	type 2 diabetes mellitus	MONDO_0005148	Digestive system disorder	Gastrointestinal	PGS003108	MONDO_0005148	type 2 diabetes mellitus	type 2 diabetes mellitus	MONDO_0005148	Digestive system disorder	Gastrointestinal
-0.8574	2.27E-133	6.24E-130	PGS001357	MONDO_0005148	type 2 diabetes mellitus	type 2 diabetes mellitus	MONDO_0005148	Metabolic disorder	Metabolic	PGS003108	MONDO_0005148	type 2 diabetes mellitus	type 2 diabetes mellitus	MONDO_0005148	Digestive system disorder	Gastrointestinal
-0.8574	2.27E-133	6.24E-130	PGS001357	MONDO_0005148	type 2 diabetes mellitus	type 2 diabetes mellitus	MONDO_0005148	Digestive system disorder	Gastrointestinal	PGS003108	MONDO_0005148	type 2 diabetes mellitus	type 2 diabetes mellitus	MONDO_0005148	Metabolic disorder	Metabolic
-0.8574	2.27E-133	6.25E-130	PGS001937	EFO_0004279	suntan	suntan	EFO_0004279	Other trait	Dermatological	PGS001897	EFO_0003784	skin pigmentation	skin pigmentation	EFO_0003784	Other trait	Dermatological
0.8574	2.37E-133	6.51E-130	PGS001165	EFO_0004995	lean body mass	lean body mass	EFO_0004995	Other measurement	Skeletal	PGS001159	EFO_0005106	body composition measurement	body composition measurement	EFO_0005106	Body measurement	Body measurement
0.8574	2.39E-133	6.55E-130	PGS000347	EFO_1000649	estrogen-receptor positive breast cancer	estrogen-receptor positive breast cancer	EFO_1000649	Cancer	Neoplasms	PGS000214	MONDO_0021115	luminal B breast carcinoma	luminal B breast carcinoma	MONDO_0021115	Cancer	Neoplasms
0.8573	2.88E-133	7.89E-130	PGS002623	EFO_0004631	vitamin D measurement	vitamin D measurement	EFO_0004631	Lipid or lipoprotein measurement	Metabolic	PGS003190	EFO_0004631	vitamin D measurement	vitamin D measurement	EFO_0004631	Lipid or lipoprotein measurement	Metabolic
0.8571	3.82E-133	1.05E-129	PGS002475	MONDO_0005148	type 2 diabetes mellitus	type 2 diabetes mellitus	MONDO_0005148	Digestive system disorder	Gastrointestinal	PGS002452	EFO_0004541	HbA1c measurement	HbA1c measurement	EFO_0004541	Hematological measurement	Hematological

0.8571	3.82E-133	1.05E-129	PGS002475	MONDO_0005148	type 2 diabetes mellitus	type 2 diabetes mellitus	MONDO_0005148	Metabolic disorder	Metabolic	PGS002452	EFO_0004541	HbA1c measurement	HbA1c measurement	EFO_0004541	Hematological measurement	Hematological
0.857	4.53E-133	1.24E-129	PGS002409	EFO_0004611	low density lipoprotein cholesterol measurement	low density lipoprotein cholesterol measurement	EFO_0004611	Lipid or lipoprotein measurement	Metabolic	PGS002620	EFO_0004574	total cholesterol measurement	total cholesterol measurement	EFO_0004574	Lipid or lipoprotein measurement	Metabolic
0.857	4.53E-133	1.24E-129	PGS002409	EFO_0004611	low density lipoprotein cholesterol measurement	low density lipoprotein cholesterol measurement	EFO_0004611	Cardiovascular measurement	Cardiovascular	PGS002620	EFO_0004574	total cholesterol measurement	total cholesterol measurement	EFO_0004574	Lipid or lipoprotein measurement	Metabolic
-0.8568	5.57E-133	1.53E-129	PGS001357	MONDO_0005148	type 2 diabetes mellitus	type 2 diabetes mellitus	MONDO_0005148	Metabolic disorder	Metabolic	PGS001781	MONDO_0005148	type 2 diabetes mellitus	type 2 diabetes mellitus	MONDO_0005148	Metabolic disorder	Metabolic
-0.8568	5.57E-133	1.53E-129	PGS001357	MONDO_0005148	type 2 diabetes mellitus	type 2 diabetes mellitus	MONDO_0005148	Digestive system disorder	Gastrointestinal	PGS001781	MONDO_0005148	type 2 diabetes mellitus	type 2 diabetes mellitus	MONDO_0005148	Digestive system disorder	Gastrointestinal
-0.8568	5.57E-133	1.53E-129	PGS001357	MONDO_0005148	type 2 diabetes mellitus	type 2 diabetes mellitus	MONDO_0005148	Metabolic disorder	Metabolic	PGS001781	MONDO_0005148	type 2 diabetes mellitus	type 2 diabetes mellitus	MONDO_0005148	Digestive system disorder	Gastrointestinal
-0.8568	5.57E-133	1.53E-129	PGS001357	MONDO_0005148	type 2 diabetes mellitus	type 2 diabetes mellitus	MONDO_0005148	Digestive system disorder	Gastrointestinal	PGS001781	MONDO_0005148	type 2 diabetes mellitus	type 2 diabetes mellitus	MONDO_0005148	Metabolic disorder	Metabolic
0.8568	6.12E-133	1.68E-129	PGS002854	EFO_0004340	body mass index	body mass index	EFO_0004340	Body measurement	Body measurement	PGS002855	EFO_0004340	body mass index	body mass index	EFO_0004340	Body measurement	Body measurement
0.8567	6.47E-133	1.77E-129	PGS000528	EFO_0000305	breast carcinoma	breast carcinoma	EFO_0000305	Cancer	Neoplasms	PGS000535	EFO_0000305	breast carcinoma	breast carcinoma	EFO_0000305	Cancer	Neoplasms
0.8567	6.98E-133	1.91E-129	PGS001165	EFO_0004995	lean body mass	lean body mass	EFO_0004995	Other measurement	Skeletal	PGS001167	EFO_0004995	lean body mass	lean body mass	EFO_0004995	Other measurement	Skeletal
0.8566	7.27E-133	1.99E-129	PGS002221	EFO_0010701	mean reticulocyte volume	mean reticulocyte volume	EFO_0010701	Hematological measurement	Hematological	PGS002008	EFO_0004503	hematological measurement	hematological measurement	EFO_0004503	Hematological measurement	Hematological
0.8566	7.80E-133	2.13E-129	PGS000331	EFO_0000275	atrial fibrillation	atrial fibrillation	EFO_0000275	Cardiovascular disease	Cardiovascular	PGS002756	EFO_0000275	atrial fibrillation	atrial fibrillation	EFO_0000275	Cardiovascular disease	Cardiovascular
0.8566	8.08E-133	2.21E-129	PGS000633	EFO_0002892	thyroid carcinoma	thyroid carcinoma	EFO_0002892	Cancer	Neoplasms	PGS000209	EFO_0002892	thyroid carcinoma	thyroid carcinoma	EFO_0002892	Cancer	Neoplasms
0.8566	8.24E-133	2.25E-129	PGS003142	EFO_0004574	total cholesterol measurement	total cholesterol measurement	EFO_0004574	Lipid or lipoprotein measurement	Metabolic	PGS003032	EFO_0004611	low density lipoprotein cholesterol measurement	measurement	EFO_0004611	Lipid or lipoprotein measurement	Metabolic
0.8566	8.24E-133	2.25E-129	PGS003142	EFO_0004574	total cholesterol measurement	total cholesterol measurement	EFO_0004574	Lipid or lipoprotein measurement	Metabolic	PGS003032	EFO_0004611	low density lipoprotein cholesterol measurement	measurement	EFO_0004611	Cardiovascular measurement	Cardiovascular
0.8565	9.09E-133	2.48E-129	PGS000344	EFO_0000305	breast carcinoma	breast carcinoma	EFO_0000305	Cancer	Neoplasms	PGS000005	EFO_1000649	estrogen-receptor positive breast cancer	cancer	EFO_1000649	Cancer	Neoplasms
0.8565	9.50E-133	2.59E-129	PGS002086	MONDO_0002917	disorder of pilosebaceous unit	disorder of pilosebaceous unit	MONDO_0002917	Other disease	Dermatological	PGS001874	EFO_1000243	Epidermal Inclusion Cyst	Epidermal Inclusion Cyst	EFO_1000243	Cancer	Neoplasms
0.8561	1.61E-132	4.39E-129	PGS003119	MONDO_0005148	type 2 diabetes mellitus	type 2 diabetes mellitus	MONDO_0005148	Metabolic disorder	Metabolic	PGS002243	MONDO_0005148	type 2 diabetes mellitus	type 2 diabetes mellitus	MONDO_0005148	Metabolic disorder	Metabolic
0.8561	1.61E-132	4.39E-129	PGS003119	MONDO_0005148	type 2 diabetes mellitus	type 2 diabetes mellitus	MONDO_0005148	Digestive system disorder	Gastrointestinal	PGS002243	MONDO_0005148	type 2 diabetes mellitus	type 2 diabetes mellitus	MONDO_0005148	Digestive system disorder	Gastrointestinal
0.8561	1.61E-132	4.39E-129	PGS003119	MONDO_0005148	type 2 diabetes mellitus	type 2 diabetes mellitus	MONDO_0005148	Digestive system disorder	Gastrointestinal	PGS002243	MONDO_0005148	type 2 diabetes mellitus	type 2 diabetes mellitus	MONDO_0005148	Metabolic disorder	Metabolic
0.8561	1.61E-132	4.39E-129	PGS003119	MONDO_0005148	type 2 diabetes mellitus	type 2 diabetes mellitus	MONDO_0005148	Metabolic disorder	Metabolic	PGS002243	MONDO_0005148	type 2 diabetes mellitus	type 2 diabetes mellitus	MONDO_0005148	Digestive system disorder	Gastrointestinal
0.8559	2.04E-132	5.57E-129	PGS002452	EFO_0004541	HbA1c measurement	HbA1c measurement	EFO_0004541	Hematological measurement	Hematological	PGS002599	EFO_0004541	HbA1c measurement	HbA1c measurement	EFO_0004541	Hematological measurement	Hematological
0.8558	2.64E-132	7.18E-129	PGS001105	EFO_0007800	body fat percentage	body fat percentage	EFO_0007800	Body measurement	Body measurement	PGS001102	EFO_0007800	body fat percentage	body fat percentage	EFO_0007800	Body measurement	Body measurement
0.8557	3.00E-132	8.17E-129	PGS002850	EFO_0004340	body mass index	body mass index	EFO_0004340	Body measurement	Body measurement	PGS002859	EFO_0004340	body mass index	body mass index	EFO_0004340	Body measurement	Body measurement
0.8554	4.16E-132	1.13E-128	PGS001148	EFO_0005409	fat body mass	fat body mass	EFO_0005409	Other measurement	Body measurement	PGS001144	EFO_0005409	fat body mass	fat body mass	EFO_0005409	Other measurement	Body measurement
0.8553	4.84E-132	1.32E-128	PGS001230	EFO_0004338	body weight	body weight	EFO_0004338	Body measurement	Body measurement	PGS001148	EFO_0005409	fat body mass	fat body mass	EFO_0005409	Other measurement	Body measurement
0.8553	5.47E-132	1.49E-128	PGS002896	EFO_0006336	diastolic blood pressure	diastolic blood pressure	EFO_0006336	Other measurement	Cardiovascular	PGS002895	EFO_0006336	diastolic blood pressure	diastolic blood pressure	EFO_0006336	Other measurement	Cardiovascular
0.8552	5.66E-132	1.54E-128	PGS003104	MONDO_0005148	type 2 diabetes mellitus	type 2 diabetes mellitus	MONDO_0005148	Metabolic disorder	Metabolic	PGS002243	MONDO_0005148	type 2 diabetes mellitus	type 2 diabetes mellitus	MONDO_0005148	Digestive system disorder	Gastrointestinal
0.8552	5.66E-132	1.54E-128	PGS003104	MONDO_0005148	type 2 diabetes mellitus	type 2 diabetes mellitus	MONDO_0005148	Digestive system disorder	Gastrointestinal	PGS002243	MONDO_0005148	type 2 diabetes mellitus	type 2 diabetes mellitus	MONDO_0005148	Digestive system disorder	Gastrointestinal
0.8552	5.66E-132	1.54E-128	PGS003104	MONDO_0005148	type 2 diabetes mellitus	type 2 diabetes mellitus	MONDO_0005148	Metabolic disorder	Metabolic	PGS002243	MONDO_0005148	type 2 diabetes mellitus	type 2 diabetes mellitus	MONDO_0005148	Metabolic disorder	Metabolic
0.8552	5.66E-132	1.54E-128	PGS003104	MONDO_0005148	type 2 diabetes mellitus	type 2 diabetes mellitus	MONDO_0005148	Digestive system disorder	Gastrointestinal	PGS002243	MONDO_0005148	type 2 diabetes mellitus	type 2 diabetes mellitus	MONDO_0005148	Metabolic disorder	Metabolic
0.855	8.17E-132	2.22E-128	PGS001265	EFO_0003907	deep vein thrombosis	deep vein thrombosis	EFO_0003907	Other trait	Cardiovascular	PGS000931	MONDO_0000831	thrombotic disease|deep vein thrombosis	thrombotic disease	MONDO_0000831	Cardiovascular disease	Cardiovascular
0.8549	8.80E-132	2.38E-128	PGS002425	EFO_0004530	triglyceride measurement	triglyceride measurement	EFO_0004530	Lipid or lipoprotein measurement	Metabolic	PGS002474	EFO_0004530	triglyceride measurement	triglyceride measurement	EFO_0004530	Lipid or lipoprotein measurement	Metabolic
0.8547	1.25E-131	3.39E-128	PGS003315	EFO_0004617	cystatin C measurement|glomerular filtration rate	cystatin C measurement	EFO_0004617	Other measurement	Metabolic	PGS003297	EFO_0004617	cystatin C measurement|glomerular filtration rate	cystatin C measurement	EFO_0004617	Other measurement	Metabolic
0.8547	1.25E-131	3.39E-128	PGS003297	EFO_0004617	cystatin C measurement|glomerular filtration rate	cystatin C measurement	EFO_0004617	Other measurement	Metabolic	PGS003316	EFO_0004617	cystatin C measurement|glomerular filtration rate	cystatin C measurement	EFO_0004617	Other measurement	Metabolic
0.8547	1.30E-131	3.52E-128	PGS000892	EFO_0004611	low density lipoprotein cholesterol measurement	low density lipoprotein cholesterol measurement	EFO_0004611	Lipid or lipoprotein measurement	Metabolic	PGS003038	EFO_0004611	low density lipoprotein cholesterol measurement	measurement	EFO_0004611	Cardiovascular measurement	Cardiovascular
0.8547	1.30E-131	3.52E-128	PGS000892	EFO_0004611	low density lipoprotein cholesterol measurement	low density lipoprotein cholesterol measurement	EFO_0004611	Cardiovascular measurement	Cardiovascular	PGS003038	EFO_0004611	low density lipoprotein cholesterol measurement	measurement	EFO_0004611	Lipid or lipoprotein measurement	Metabolic
0.8547	1.30E-131	3.52E-128	PGS000892	EFO_0004611	low density lipoprotein cholesterol measurement	low density lipoprotein cholesterol measurement	EFO_0004611	Cardiovascular measurement	Cardiovascular	PGS003038	EFO_0004611	low density lipoprotein cholesterol measurement	measurement	EFO_0004611	Cardiovascular measurement	Cardiovascular
0.8547	1.30E-131	3.52E-128	PGS000892	EFO_0004611	low density lipoprotein cholesterol measurement	low density lipoprotein cholesterol measurement	EFO_0004611	Lipid or lipoprotein measurement	Metabolic	PGS003038	EFO_0004611	low density lipoprotein cholesterol measurement	measurement	EFO_0004611	Lipid or lipoprotein measurement	Metabolic
0.8546	1.33E-131	3.59E-128	PGS001478	EFO_0006930	brain volume measurement|neuroimaging measurement	brain volume measurement	EFO_0006930	Other measurement	Neurological	PGS001479	EFO_0006930	measurement	brain volume measurement	EFO_0006930	Other measurement	Neurological
0.8545	1.60E-131	4.33E-128	PGS000948	HP_0000023	Inguinal hernia	Inguinal hernia	HP_0000023	Other trait	Gastrointestinal	PGS000949	HP_0000023	Inguinal hernia	Inguinal hernia	HP_0000023	Other trait	Gastrointestinal
0.8545	1.63E-131	4.39E-128	PGS002157	EFO_0004533	alkaline phosphatase measurement	alkaline phosphatase measurement	EFO_0004533	Liver enzyme measurement	Gastrointestinal	PGS000670	EFO_0004533	alkaline phosphatase measurement	alkaline phosphatase measurement	EFO_0004533	Liver enzyme measurement	Gastrointestinal
0.8545	1.64E-131	4.42E-128	PGS003383	EFO_0000673	prostate adenocarcinoma	prostate adenocarcinoma	EFO_0000673	Cancer	Neoplasms	PGS000584	EFO_0001663	prostate carcinoma	prostate carcinoma	EFO_0001663	Cancer	Neoplasms
0.8545	1.70E-131	4.60E-128	PGS003315	EFO_0004617	cystatin C measurement|glomerular filtration rate	cystatin C measurement	EFO_0004617	Other measurement	Metabolic	PGS003312	EFO_0004617	cystatin C measurement|glomerular filtration rate	cystatin C measurement	EFO_0004617	Other measurement	Metabolic
0.8545	1.70E-131	4.60E-128	PGS003316	EFO_0004617	cystatin C measurement|glomerular filtration rate	cystatin C measurement	EFO_0004617	Other measurement	Metabolic	PGS003312	EFO_0004617	cystatin C measurement|glomerular filtration rate	cystatin C measurement	EFO_0004617	Other measurement	Metabolic
0.8543	2.21E-131	5.97E-128	PGS002854	EFO_0004340	body mass index	body mass index	EFO_0004340	Body measurement	Body measurement	PGS002845	EFO_0004340	body mass index	body mass index	EFO_0004340	Body measurement	Body measurement
0.8543	2.29E-131	6.17E-128	PGS001289	EFO_0002892	thyroid carcinoma	thyroid carcinoma	EFO_0002892	Cancer	Neoplasms	PGS001809	EFO_0002892	thyroid carcinoma	thyroid carcinoma	EFO_0002892	Cancer	Neoplasms
0.8543	2.33E-131	6.26E-128	PGS001275	EFO_0003839	retinopathy	retinopathy	EFO_0003839	Neurological disorder	Neurological	PGS002041	EFO_0001365	age-related macular degeneration	age-related macular degeneration	EFO_0001365	Neurological disorder	Neurological
0.854	3.51E-131	9.44E-128	PGS000007	EFO_0000305	breast carcinoma	breast carcinoma	EFO_0000305	Cancer	Neoplasms	PGS000009	EFO_1000650	estrogen-receptor negative breast cancer	cancer	EFO_1000650	Cancer	Neoplasms
0.8538	4.75E-131	1.28E-127	PGS002315	EFO_0003923	bone density	bone density	EFO_0003923	Other measurement	Skeletal	PGS002175	EFO_0005654	velocity of sound measurement	velocity of sound measurement	EFO_0005654	Other measurement	Others
0.8537	4.85E-131	1.30E-127	PGS002396	EFO_0001379	endocrine system disease|diabetes mellitus	endocrine system disease	EFO_0001379	Other disease	Metabolic	PGS002449	EFO_0004468	glucose measurement	glucose measurement	EFO_0004468	Other measurement	Metabolic
0.8537	4.92E-131	1.32E-127	PGS002003	EFO_0010701	mean reticulocyte volume	mean reticulocyte volume	EFO_0010701	Hematological measurement	Hematological	PGS002227	EFO_0004503	hematological measurement	hematological measurement	EFO_0004503	Hematological measurement	Hematological
0.8537	5.41E-131	1.45E-127	PGS001300	EFO_0001060	celiac disease	celiac disease	EFO_0001060	Immune system disorder	Immunological	PGS001399	EFO_0009340	Varicella zoster virus seropositivity	Varicella zoster virus seropositivity	EFO_0009340	Other measurement	Infection
0.8537	5.41E-131	1.45E-127	PGS001300	EFO_0001060	celiac disease	celiac disease	EFO_0001060	Digestive system disorder	Gastrointestinal	PGS001399	EFO_0009340	Varicella zoster virus seropositivity	Varicella zoster virus seropositivity	EFO_0009340	Other measurement	Infection
0.8536	5.78E-131	1.55E-127	PGS002589	EFO_0000400	diabetes mellitus	diabetes mellitus	EFO_0000400	Digestive system disorder	Gastrointestinal	PGS002400	EFO_0004468	glucose measurement	glucose measurement	EFO_0004468	Other measurement	Metabolic
0.8536	5.78E-131	1.55E-127	PGS002589	EFO_0000400	diabetes mellitus	diabetes mellitus	EFO_0000400	Metabolic disorder	Metabolic	PGS002400	EFO_0004468	glucose measurement	glucose measurement	EFO_0004468	Other measurement	Metabolic
0.8536	5.88E-131	1.58E-127	PGS002844	EFO_0004340	body mass index	body mass index	EFO_0004340	Body measurement	Body measurement	PGS002845	EFO_0004340	body mass index	body mass index	EFO_0004340	Body measurement	Body measurement
0.8536	6.34E-131	1.70E-127	PGS001674	EFO_0006930	brain volume measurement|neuroimaging measurement	brain volume measurement	EFO_0006930	Other measurement	Neurological	PGS001481	EFO_0006930	measurement	brain volume measurement	EFO_0006930	Other measurement	Neurological
0.8535	6.65E-131	1.78E-127	PGS002452	EFO_0004541	HbA1c measurement	HbA1c measurement	EFO_0004541	Hematological measurement	Hematological	PGS002442	EFO_0000400	diabetes mellitus	diabetes mellitus	EFO_0000400	Digestive system disorder	Gastrointestinal
0.8535	6.65E-131	1.78E-127	PGS002452	EFO_0004541	HbA1c measurement	HbA1c measurement	EFO_0004541	Hematological measurement	Hematological	PGS002442	EFO_0000400	diabetes mellitus	diabetes mellitus	EFO_0000400	Metabolic disorder	Metabolic
0.8533	9.23E-131	2.47E-127	PGS002283	EFO_0003095	non-alcoholic fatty liver disease	non-alcoholic fatty liver disease	EFO_0003095	Digestive system disorder	Gastrointestinal	PGS002282	EFO_0003095	non-alcoholic fatty liver disease	non-alcoholic fatty liver disease	EFO_0003095	Digestive system disorder	Gastrointestinal
0.8533	9.81E-131	2.62E-127	PGS000672	EFO_0004615	apolipoprotein B measurement	apolipoprotein B measurement	EFO_0004615	Lipid or lipoprotein measurement	Metabolic	PGS000677	EFO_0004574	total cholesterol measurement	total cholesterol measurement	EFO_0004574	Lipid or lipoprotein measurement	Metabolic
0.853	1.33E-130	3.55E-127	PGS001112	EFO_0007013	aspirin use measurement	aspirin use measurement	EFO_0007013	Other measurement	Activities	PGS001113	EFO_0007013	aspirin use measurement	aspirin use measurement	EFO_0007013	Other measurement	Activities
-0.853	1.41E-130	3.77E-127	PGS003376	EFO_0007878	alcohol consumption measurement	alcohol consumption measurement	EFO_0007878	Other measurement	Psychiatric	PGS000204	EFO_0007878	alcohol consumption measurement	alcohol consumption measurement	EFO_0007878	Other measurement	Psychiatric
0.8528	1.92E-130	5.12E-127	PGS000588	EFO_0001663	prostate carcinoma	prostate carcinoma	EFO_0001663	Cancer	Neoplasms	PGS000583	EFO_0001663	prostate carcinoma	prostate carcinoma	EFO_0001663	Cancer	Neoplasms
0.8528	1.94E-130	5.18E-127	PGS002851	EFO_0004340	body mass index	body mass index	EFO_0004340	Body measurement	Body measurement	PGS002842	EFO_0004340	body mass index	body mass index	EFO_0004340	Body measurement	Body measurement
0.8527	2.14E-130	5.71E-127	PGS000587	EFO_0001663	prostate carcinoma	prostate carcinoma	EFO_0001663	Cancer	Neoplasms	PGS003383	EFO_0000673	prostate adenocarcinoma	prostate adenocarcinoma	EFO_0000673	Cancer	Neoplasms
0.8527	2.18E-130	5.82E-127	PGS002509	EFO_0004527	mean corpuscular hemoglobin	mean corpuscular hemoglobin	EFO_0004527	Hematological measurement	Hematological	PGS002460	EFO_0004527	mean corpuscular hemoglobin	mean corpuscular hemoglobin	EFO_0004527	Hematological measurement	Hematological
0.8526	2.32E-130	6.18E-127	PGS001742	EFO_0006930	brain volume measurement|neuroimaging measurement	brain volume measurement	EFO_0006930	Other measurement	Neurological	PGS001757	EFO_0006930	measurement	brain volume measurement	EFO_0006930	Other measurement	Neurological
0.8526	2.37E-130	6.31E-127	PGS000707	HP_0001082	Cholecystitis	Cholecystitis	HP_0001082	Other trait	Gastrointestinal	PGS000705	EFO_0004210	gallstones	gallstones	EFO_0004210	Digestive system disorder	Gastrointestinal
0.8526	2.43E-130	6.47E-127	PGS001145	EFO_0005409	fat body mass	fat body mass	EFO_0005409	Other measurement	Body measurement	PGS001162	EFO_0005093	hip circumference	hip circumference	EFO_0005093	Body measurement	Body measurement
0.8526	2.52E-130	6.71E-127	PGS003380	EFO_0000305	breast carcinoma	breast carcinoma	EFO_0000305	Cancer	Neoplasms	PGS000507	EFO_0000305	breast carcinoma	breast carcinoma	EFO_0000305	Cancer	Neoplasms
0.8526	2.57E-130	6.82E-127	PGS000389	MONDO_0001672	bronchus cancer|lung cancer|tracheal cancer	bronchus cancer	MONDO_0001672	Cancer	Neoplasms	PGS000880	EFO_0001071	lung carcinoma	lung carcinoma	EFO_0001071	Cancer	Neoplasms
0.8526	2.57E-130	6.82E-127	PGS000390	MONDO_0001672	bronchus cancer|lung cancer|tracheal cancer	bronchus cancer	MONDO_0001672	Cancer	Neoplasms	PGS000880	EFO_0001071	lung carcinoma	lung carcinoma	EFO_0001071	Cancer	Neoplasms
0.8525	2.70E-130	7.17E-127	PGS002462	EFO_0005091	monocyte count	monocyte count	EFO_0005091	Hematological measurement	Hematological	PGS002413	EFO_0005091	monocyte count	monocyte count	EFO_0005091	Hematological measurement	Hematological
-0.8522	4.46E-130	1.18E-126	PGS003315	EFO_0004617	cystatin C measurement|glomerular filtration rate	cystatin C measurement	EFO_0004617	Other measurement	Metabolic	PGS003304	EFO_0004617	cystatin C measurement|glomerular filtration rate	cystatin C measurement	EFO_0004617	Other measurement	Metabolic
-0.8522	4.46E-130	1.18E-126	PGS003316	EFO_0004617	cystatin C measurement|glomerular filtration rate	cystatin C measurement	EFO_0004617	Other measurement	Metabolic	PGS003304	EFO_0004617	cystatin C measurement|glomerular filtration rate	cystatin C measurement	EFO_0004617	Other measurement	Metabolic
0.8521	4.93E-130	1.31E-126	PGS002187	EFO_0004833	neutrophil count	neutrophil count	EFO_0004833	Hematological measurement	Hematological	PGS002180	EFO_0004308	leukocyte count	leukocyte count	EFO_0004308	Hematological measurement	Hematological
0.8521	5.34E-130	1.41E-126	PGS000560	EFO_0003893	ovarian neoplasm	ovarian neoplasm	EFO_0003893	Cancer	Neoplasms	PGS000549	EFO_0003893	ovarian neoplasm	ovarian neoplasm	EFO_0003893	Cancer	Neoplasms
0.8521	5.34E-130	1.41E-126	PGS000559	EFO_0003893	ovarian neoplasm	ovarian neoplasm	EFO_0003893	Cancer	Neoplasms	PGS000549	EFO_0003893	ovarian neoplasm	ovarian neoplasm	EFO_0003893	Cancer	Neoplasms
0.852	5.53E-130	1.46E-126	PGS000474	EFO_0000305	breast carcinoma	breast carcinoma	EFO_0000305	Cancer	Neoplasms	PGS000472	EFO_0000305	breast carcinoma	breast carcinoma	EFO_0000305	Cancer	Neoplasms
0.852	5.66E-130	1.50E-126	PGS003125	EFO_0004340	body mass index	body mass index	EFO_0004340	Body measurement	Body measurement	PGS003126	EFO_0004340	body mass index	body mass index	EFO_0004340	Body measurement	Body measurement
0.8519	6.37E-130	1.68E-126	PGS001106	EFO_0007800	body fat percentage	body fat percentage	EFO_0007800	Body measurement	Body measurement	PGS001144	EFO_0005409	fat body mass	fat body mass	EFO_0005409	Other measurement	Body measurement
0.8519	6.55E-130	1.73E-126	PGS001065	EFO_0008111	diet measurement	diet measurement	EFO_0008111	Other measurement	Activities	PGS001894	EFO_0001060	celiac disease	celiac disease	EFO_0001060	Immune system disorder	Immunological
0.8519	6.55E-130	1.73E-126	PGS001065	EFO_0008111	diet measurement	diet measurement	EFO_0008111	Other measurement	Activities	PGS001894	EFO_0001060	celiac disease	celiac disease	EFO_0001060	Digestive system disorder	Gastrointestinal
0.8518	7.45E-130	1.97E-126	PGS002458	EFO_0004611	low density lipoprotein cholesterol measurement	low density lipoprotein cholesterol measurement	EFO_0004611	Lipid or lipoprotein measurement	Metabolic	PGS002424	EFO_0004574	total cholesterol measurement	total cholesterol measurement	EFO_0004574	Lipid or lipoprotein measurement	Metabolic
0.8518	7.45E-130	1.97E-126	PGS002458	EFO_0004611	low density lipoprotein cholesterol measurement	low density lipoprotein cholesterol measurement	EFO_0004611	Cardiovascular measurement	Cardiovascular	PGS002424	EFO_0004574	total cholesterol measurement	total cholesterol measurement	EFO_0004574	Lipid or lipoprotein measurement	Metabolic
0.8518	8.02E-130	2.12E-126	PGS001951	EFO_0005409	fat body mass	fat body mass	EFO_0005409	Other measurement	Body measurement	PGS002133	EFO_0007800	body fat percentage	body fat percentage	EFO_0007800	Body measurement	Body measurement
0.8517	9.47E-130	2.50E-126	PGS001962	EFO_0004308	leukocyte count	leukocyte count	EFO_0004308	Hematological measurement	Hematological	PGS002357	EFO_0004308	leukocyte count	leukocyte count	EFO_0004308	Hematological measurement	Hematological
0.8516	1.09E-129	2.88E-126	PGS001149	EFO_0005409	fat body mass	fat body mass	EFO_0005409	Other measurement	Body measurement	PGS001106	EFO_0007800	body fat percentage	body fat percentage	EFO_0007800	Body measurement	Body measurement
0.8515	1.14E-129	3.00E-126	PGS001145	EFO_0005409	fat body mass	fat body mass	EFO_0005409	Other measurement	Body measurement	PGS001230	EFO_0004338	body weight	body weight	EFO_0004338	Body measurement	Body measurement
0.8514	1.34E-129	3.53E-126	PGS001079	EFO_0007984	platelet component distribution width	platelet component distribution width	EFO_0007984	Hematological measurement	Hematological	PGS001972	EFO_0007984	platelet component distribution width	platelet component distribution width	EFO_0007984	Hematological measurement	Hematological
0.8514	1.38E-129	3.64E-126	PGS002844	EFO_0004340	body mass index	body mass index	EFO_0004340	Body measurement	Body measurement	PGS002855	EFO_0004340	body mass index	body mass index	EFO_0004340	Body measurement	Body measurement
0.8514	1.45E-129	3.81E-126	PGS000095	EFO_0007986	reticulocyte count	reticulocyte count	EFO_0007986	Hematological measurement	Hematological	PGS000112	EFO_0007986	reticulocyte count	reticulocyte count	EFO_0007986	Hematological measurement	Hematological
0.8514	1.45E-129	3.82E-126	PGS002002	EFO_0007822	hair colour measurement	hair colour measurement	EFO_0007822	Other measurement	Dermatological	PGS002220	EFO_0007822	hair colour measurement	hair colour measurement	EFO_0007822	Other measurement	Dermatological
0.8512	1.74E-129	4.57E-126	PGS003198	EFO_0004631	vitamin D measurement	vitamin D measurement	EFO_0004631	Lipid or lipoprotein measurement	Metabolic	PGS001982	EFO_0004631	vitamin D measurement	vitamin D measurement	EFO_0004631	Lipid or lipoprotein measurement	Metabolic
0.851	2.26E-129	5.94E-126	PGS001228	EFO_0004340	body mass index	body mass index	EFO_0004340	Body measurement	Body measurement	PGS001102	EFO_0007800	body fat percentage	body fat percentage	EFO_0007800	Body measurement	Body measurement
0.8508	2.94E-129	7.73E-126	PGS002200	EFO_0004631	vitamin D measurement	vitamin D measurement	EFO_0004631	Lipid or lipoprotein measurement	Metabolic	PGS002672	EFO_0004631	vitamin D measurement	vitamin D measurement	EFO_0004631	Lipid or lipoprotein measurement	Metabolic
0.8508	2.96E-129	7.77E-126	PGS000588	EFO_0001663	prostate carcinoma	prostate carcinoma	EFO_0001663	Cancer	Neoplasms	PGS000584	EFO_0001663	prostate carcinoma	prostate carcinoma	EFO_0001663	Cancer	Neoplasms
0.8507	3.59E-129	9.41E-126	PGS003067	EFO_0006527	smoking status measurement	smoking status measurement	EFO_0006527	Other measurement	Psychiatric	PGS003062	EFO_0006527	smoking status measurement	smoking status measurement	EFO_0006527	Other measurement	Psychiatric
0.8506	4.01E-129	1.05E-125	PGS002845	EFO_0004340	body mass index	body mass index	EFO_0004340	Body measurement	Body measurement	PGS002849	EFO_0004340	body mass index	body mass index	EFO_0004340	Body measurement	Body measurement
0.8506	4.06E-129	1.06E-125	PGS003295	EFO_0004617	cystatin C measurement|glomerular filtration rate	cystatin C measurement	EFO_0004617	Other measurement	Metabolic	PGS003296	EFO_0004617	cystatin C measurement|glomerular filtration rate	cystatin C measurement	EFO_0004617	Other measurement	Metabolic
0.8506	4.13E-129	1.08E-125	PGS002397	EFO_0004842	eosinophil count	eosinophil count	EFO_0004842	Hematological measurement	Hematological	PGS002446	EFO_0004842	eosinophil count	eosinophil count	EFO_0004842	Hematological measurement	Hematological

0.8506	4.41E-129	1.15E-125	PGS002603	EFO_0000537	hypertension	hypertension	EFO_0000537	Cardiovascular disease	Cardiovascular	PGS002407	EFO_0000537	hypertension	hypertension	EFO_0000537	Cardiovascular disease	Cardiovascular
0.8504	5.91E-129	1.54E-125	PGS000179	EFO_0004584	mean platelet volume	mean platelet volume	EFO_0004584	Hematological measurement	Hematological	PGS000104	EFO_0004584	mean platelet volume	mean platelet volume	EFO_0004584	Hematological measurement	Hematological
0.8503	6.18E-129	1.61E-125	PGS000516	EFO_0000305	breast carcinoma	breast carcinoma	EFO_0000305	Cancer	Neoplasms	PGS000483	EFO_0000305	breast carcinoma	breast carcinoma	EFO_0000305	Cancer	Neoplasms
0.8502	7.14E-129	1.87E-125	PGS002516	EFO_0005192	red blood cell distribution width	red blood cell distribution width	EFO_0005192	Hematological measurement	Hematological	PGS002467	EFO_0005192	red blood cell distribution width	red blood cell distribution width	EFO_0005192	Hematological measurement	Hematological
0.8502	7.44E-129	1.94E-125	PGS003232	EFO_0010472	creatine measurement	creatine measurement	EFO_0010472	Other measurement	Metabolic	PGS003237	EFO_0010472	creatine measurement	creatine measurement	EFO_0010472	Other measurement	Metabolic
0.8498	1.24E-128	3.24E-125	PGS002418	EFO_0005192	red blood cell distribution width	red blood cell distribution width	EFO_0005192	Hematological measurement	Hematological	PGS002565	EFO_0005192	red blood cell distribution width	red blood cell distribution width	EFO_0005192	Hematological measurement	Hematological
0.8497	1.44E-128	3.76E-125	PGS002427	EFO_0004631	vitamin D measurement	vitamin D measurement	EFO_0004631	Lipid or lipoprotein measurement	Metabolic	PGS003190	EFO_0004631	vitamin D measurement	vitamin D measurement	EFO_0004631	Lipid or lipoprotein measurement	Metabolic
0.8496	1.79E-128	4.67E-125	PGS000892	EFO_0004611	low density lipoprotein cholesterol measurement	low density lipoprotein cholesterol measurement	EFO_0004611	Cardiovascular measurement	Cardiovascular	PGS003033	EFO_0004611	low density lipoprotein cholesterol measurement	measurement	EFO_0004611	Lipid or lipoprotein measurement	Metabolic
0.8496	1.79E-128	4.67E-125	PGS000892	EFO_0004611	low density lipoprotein cholesterol measurement	low density lipoprotein cholesterol measurement	EFO_0004611	Lipid or lipoprotein measurement	Metabolic	PGS003033	EFO_0004611	low density lipoprotein cholesterol measurement	measurement	EFO_0004611	Cardiovascular measurement	Cardiovascular
0.8496	1.79E-128	4.67E-125	PGS000892	EFO_0004611	low density lipoprotein cholesterol measurement	low density lipoprotein cholesterol measurement	EFO_0004611	Lipid or lipoprotein measurement	Metabolic	PGS003033	EFO_0004611	low density lipoprotein cholesterol measurement	measurement	EFO_0004611	Lipid or lipoprotein measurement	Metabolic
0.8496	1.79E-128	4.67E-125	PGS000892	EFO_0004611	low density lipoprotein cholesterol measurement	low density lipoprotein cholesterol measurement	EFO_0004611	Cardiovascular measurement	Cardiovascular	PGS003033	EFO_0004611	low density lipoprotein cholesterol measurement	measurement	EFO_0004611	Cardiovascular measurement	Cardiovascular
0.8494	2.11E-128	5.50E-125	PGS003140	EFO_0004574	total cholesterol measurement	total cholesterol measurement	EFO_0004574	Lipid or lipoprotein measurement	Metabolic	PGS003035	EFO_0004611	low density lipoprotein cholesterol measurement	measurement	EFO_0004611	Lipid or lipoprotein measurement	Metabolic
0.8494	2.11E-128	5.50E-125	PGS003140	EFO_0004574	total cholesterol measurement	total cholesterol measurement	EFO_0004574	Lipid or lipoprotein measurement	Metabolic	PGS003035	EFO_0004611	low density lipoprotein cholesterol measurement	measurement	EFO_0004611	Cardiovascular measurement	Cardiovascular
0.8492	2.95E-128	7.67E-125	PGS001200	EFO_0004584	mean platelet volume	mean platelet volume	EFO_0004584	Hematological measurement	Hematological	PGS001971	EFO_0004584	mean platelet volume	mean platelet volume	EFO_0004584	Hematological measurement	Hematological
0.8492	3.11E-128	8.10E-125	PGS003032	EFO_0004611	low density lipoprotein cholesterol measurement	low density lipoprotein cholesterol measurement	EFO_0004611	Lipid or lipoprotein measurement	Metabolic	PGS003137	EFO_0004574	total cholesterol measurement	total cholesterol measurement	EFO_0004574	Lipid or lipoprotein measurement	Metabolic
0.8492	3.11E-128	8.10E-125	PGS003032	EFO_0004611	low density lipoprotein cholesterol measurement	low density lipoprotein cholesterol measurement	EFO_0004611	Cardiovascular measurement	Cardiovascular	PGS003137	EFO_0004574	total cholesterol measurement	total cholesterol measurement	EFO_0004574	Lipid or lipoprotein measurement	Metabolic
0.8491	3.20E-128	8.31E-125	PGS000467	EFO_0000707	squamous cell carcinoma	squamous cell carcinoma	EFO_0000707	Cancer	Neoplasms	PGS000462	EFO_0000707	squamous cell carcinoma	squamous cell carcinoma	EFO_0000707	Cancer	Neoplasms
0.8491	3.20E-128	8.31E-125	PGS000467	EFO_0000707	squamous cell carcinoma	squamous cell carcinoma	EFO_0000707	Cancer	Neoplasms	PGS000461	EFO_0000707	squamous cell carcinoma	squamous cell carcinoma	EFO_0000707	Cancer	Neoplasms
0.8491	3.20E-128	8.31E-125	PGS000467	EFO_0000707	squamous cell carcinoma	squamous cell carcinoma	EFO_0000707	Cancer	Neoplasms	PGS000464	EFO_0000707	squamous cell carcinoma	squamous cell carcinoma	EFO_0000707	Cancer	Neoplasms
0.8491	3.47E-128	9.00E-125	PGS000486	EFO_0000305	breast carcinoma	breast carcinoma	EFO_0000305	Cancer	Neoplasms	PGS000485	EFO_0000305	breast carcinoma	breast carcinoma	EFO_0000305	Cancer	Neoplasms
0.8491	3.47E-128	9.00E-125	PGS000486	EFO_0000305	breast carcinoma	breast carcinoma	EFO_0000305	Cancer	Neoplasms	PGS000487	EFO_0000305	breast carcinoma	breast carcinoma	EFO_0000305	Cancer	Neoplasms
0.849	3.65E-128	9.47E-125	PGS000515	EFO_0000305	breast carcinoma	breast carcinoma	EFO_0000305	Cancer	Neoplasms	PGS000478	EFO_0000305	breast carcinoma	breast carcinoma	EFO_0000305	Cancer	Neoplasms
0.849	3.85E-128	9.97E-125	PGS001161	EFO_0005106	body composition measurement	body composition measurement	EFO_0005106	Body measurement	Body measurement	PGS001156	EFO_0005106	body composition measurement	body composition measurement	EFO_0005106	Body measurement	Body measurement
0.8489	4.68E-128	1.21E-124	PGS001750	EFO_0006930	brain volume measurement|neuroimaging measurement	brain volume measurement	EFO_0006930	Other measurement	Neurological	PGS001735	EFO_0006930	measurement	brain volume measurement	EFO_0006930	Other measurement	Neurological
0.8489	4.69E-128	1.21E-124	PGS002426	MONDO_0005148	type 2 diabetes mellitus	type 2 diabetes mellitus	MONDO_0005148	Metabolic disorder	Metabolic	PGS002491	EFO_0000400	diabetes mellitus	diabetes mellitus	EFO_0000400	Metabolic disorder	Metabolic
0.8489	4.69E-128	1.21E-124	PGS002426	MONDO_0005148	type 2 diabetes mellitus	type 2 diabetes mellitus	MONDO_0005148	Digestive system disorder	Gastrointestinal	PGS002491	EFO_0000400	diabetes mellitus	diabetes mellitus	EFO_0000400	Digestive system disorder	Gastrointestinal
0.8489	4.69E-128	1.21E-124	PGS002426	MONDO_0005148	type 2 diabetes mellitus	type 2 diabetes mellitus	MONDO_0005148	Digestive system disorder	Gastrointestinal	PGS002491	EFO_0000400	diabetes mellitus	diabetes mellitus	EFO_0000400	Metabolic disorder	Metabolic
0.8489	4.69E-128	1.21E-124	PGS002426	MONDO_0005148	type 2 diabetes mellitus	type 2 diabetes mellitus	MONDO_0005148	Metabolic disorder	Metabolic	PGS002491	EFO_0000400	diabetes mellitus	diabetes mellitus	EFO_0000400	Digestive system disorder	Gastrointestinal
0.8487	5.59E-128	1.45E-124	PGS001404	EFO_0009270	heel bone mineral density	heel bone mineral density	EFO_0009270	Other measurement	Skeletal	PGS000953	EFO_0004514	bone quantitative ultrasound measurement	measurement	EFO_0004514	Other measurement	Skeletal
0.8487	6.04E-128	1.56E-124	PGS000332	EFO_0000305	breast carcinoma	breast carcinoma	EFO_0000305	Cancer	Neoplasms	PGS000500	EFO_0000305	breast carcinoma	breast carcinoma	EFO_0000305	Cancer	Neoplasms
0.8486	6.88E-128	1.78E-124	PGS001817	MONDO_0005147	type 1 diabetes mellitus	type 1 diabetes mellitus	MONDO_0005147	Immune system disorder	Immunological	PGS001296	MONDO_0005147	type 1 diabetes mellitus	type 1 diabetes mellitus	MONDO_0005147	Metabolic disorder	Metabolic
0.8486	6.88E-128	1.78E-124	PGS001817	MONDO_0005147	type 1 diabetes mellitus	type 1 diabetes mellitus	MONDO_0005147	Metabolic disorder	Metabolic	PGS001296	MONDO_0005147	type 1 diabetes mellitus	type 1 diabetes mellitus	MONDO_0005147	Immune system disorder	Immunological
0.8486	6.88E-128	1.78E-124	PGS001817	MONDO_0005147	type 1 diabetes mellitus	type 1 diabetes mellitus	MONDO_0005147	Metabolic disorder	Metabolic	PGS001296	MONDO_0005147	type 1 diabetes mellitus	type 1 diabetes mellitus	MONDO_0005147	Digestive system disorder	Gastrointestinal
0.8486	6.88E-128	1.78E-124	PGS001817	MONDO_0005147	type 1 diabetes mellitus	type 1 diabetes mellitus	MONDO_0005147	Digestive system disorder	Gastrointestinal	PGS001296	MONDO_0005147	type 1 diabetes mellitus	type 1 diabetes mellitus	MONDO_0005147	Immune system disorder	Immunological
0.8486	6.88E-128	1.78E-124	PGS001817	MONDO_0005147	type 1 diabetes mellitus	type 1 diabetes mellitus	MONDO_0005147	Digestive system disorder	Gastrointestinal	PGS001296	MONDO_0005147	type 1 diabetes mellitus	type 1 diabetes mellitus	MONDO_0005147	Metabolic disorder	Metabolic
0.8486	6.88E-128	1.78E-124	PGS001817	MONDO_0005147	type 1 diabetes mellitus	type 1 diabetes mellitus	MONDO_0005147	Immune system disorder	Immunological	PGS001296	MONDO_0005147	type 1 diabetes mellitus	type 1 diabetes mellitus	MONDO_0005147	Immune system disorder	Immunological
0.8486	6.88E-128	1.78E-124	PGS001817	MONDO_0005147	type 1 diabetes mellitus	type 1 diabetes mellitus	MONDO_0005147	Immune system disorder	Immunological	PGS001296	MONDO_0005147	type 1 diabetes mellitus	type 1 diabetes mellitus	MONDO_0005147	Digestive system disorder	Gastrointestinal
0.8486	6.88E-128	1.78E-124	PGS001817	MONDO_0005147	type 1 diabetes mellitus	type 1 diabetes mellitus	MONDO_0005147	Metabolic disorder	Metabolic	PGS001296	MONDO_0005147	type 1 diabetes mellitus	type 1 diabetes mellitus	MONDO_0005147	Metabolic disorder	Metabolic
0.8486	6.88E-128	1.78E-124	PGS001817	MONDO_0005147	type 1 diabetes mellitus	type 1 diabetes mellitus	MONDO_0005147	Digestive system disorder	Gastrointestinal	PGS001296	MONDO_0005147	type 1 diabetes mellitus	type 1 diabetes mellitus	MONDO_0005147	Digestive system disorder	Gastrointestinal
0.8485	7.33E-128	1.89E-124	PGS000212	MONDO_0021116	luminal A breast carcinoma	luminal A breast carcinoma	MONDO_0021116	Cancer	Neoplasms	PGS000345	EFO_0000305	breast carcinoma	breast carcinoma	EFO_0000305	Cancer	Neoplasms
0.8483	1.09E-127	2.82E-124	PGS002162	EFO_0007934	creatinine clearance measurement	creatinine clearance measurement	EFO_0007934	Other measurement	Metabolic	PGS003233	EFO_0010472	creatine measurement	creatine measurement	EFO_0010472	Other measurement	Metabolic
0.8479	1.74E-127	4.49E-124	PGS002506	EFO_0004705	hypothyroidism	hypothyroidism	EFO_0004705	Other disease	Endocrine	PGS002457	EFO_0004705	hypothyroidism	hypothyroidism	EFO_0004705	Other disease	Endocrine
0.8478	2.05E-127	5.30E-124	PGS002840	EFO_0004340	body mass index	body mass index	EFO_0004340	Body measurement	Body measurement	PGS002849	EFO_0004340	body mass index	body mass index	EFO_0004340	Body measurement	Body measurement
0.8478	2.08E-127	5.35E-124	PGS002086	MONDO_0002917	disorder of pilosebaceous unit	disorder of pilosebaceous unit	MONDO_0002917	Other disease	Dermatological	PGS002087	EFO_1000243	Epidermal Inclusion Cyst	Epidermal Inclusion Cyst	EFO_1000243	Cancer	Neoplasms
0.8478	2.15E-127	5.55E-124	PGS001149	EFO_0005409	fat body mass	fat body mass	EFO_0005409	Other measurement	Body measurement	PGS001105	EFO_0007800	body fat percentage	body fat percentage	EFO_0007800	Body measurement	Body measurement
0.8477	2.17E-127	5.60E-124	PGS002070	MONDO_0060766	anal polyp|polyp of rectum	anal polyp	MONDO_0060766	Digestive system disorder	Gastrointestinal	PGS001859	MONDO_0060766	anal polyp|polyp of rectum	anal polyp	MONDO_0060766	Digestive system disorder	Gastrointestinal
-0.8477	2.23E-127	5.74E-124	PGS003311	EFO_0004617	cystatin C measurement|glomerular filtration rate	cystatin C measurement	EFO_0004617	Other measurement	Metabolic	PGS003304	EFO_0004617	cystatin C measurement|glomerular filtration rate	cystatin C measurement	EFO_0004617	Other measurement	Metabolic
-0.8476	2.54E-127	6.54E-124	PGS002839	EFO_0007878	alcohol consumption measurement	alcohol consumption measurement	EFO_0007878	Other measurement	Psychiatric	PGS003376	EFO_0007878	alcohol consumption measurement	alcohol consumption measurement	EFO_0007878	Other measurement	Psychiatric
0.8476	2.73E-127	7.03E-124	PGS001229	EFO_0004339	body height	body height	EFO_0004339	Body measurement	Body measurement	PGS001929	EFO_0004339	body height	body height	EFO_0004339	Body measurement	Body measurement
0.8475	2.96E-127	7.60E-124	PGS001458	EFO_0006930	brain volume measurement|neuroimaging measurement	brain volume measurement	EFO_0006930	Other measurement	Neurological	PGS001457	EFO_0006930	measurement	brain volume measurement	EFO_0006930	Other measurement	Neurological
0.8474	3.51E-127	9.02E-124	PGS001230	EFO_0004338	body weight	body weight	EFO_0004338	Body measurement	Body measurement	PGS001144	EFO_0005409	fat body mass	fat body mass	EFO_0005409	Other measurement	Body measurement
0.8474	3.63E-127	9.33E-124	PGS000942	HP_0001082	Cholecystitis	Cholecystitis	HP_0001082	Other trait	Gastrointestinal	PGS001256	EFO_0004210	gallstones	gallstones	EFO_0004210	Digestive system disorder	Gastrointestinal
0.8473	3.82E-127	9.79E-124	PGS001362	EFO_0004731	eye measurement	eye measurement	EFO_0004731	Other measurement	Ophthalmological	PGS001365	EFO_0004731	eye measurement	eye measurement	EFO_0004731	Other measurement	Ophthalmological
0.8473	4.08E-127	1.05E-123	PGS001847	EFO_0004264	vascular disease	vascular disease	EFO_0004264	Cardiovascular disease	Cardiovascular	PGS001844	EFO_1001395	Phlebitis|Thrombophlebitis	Phlebitis	EFO_1001395	Cardiovascular disease	Cardiovascular
0.8472	4.43E-127	1.14E-123	PGS003149	EFO_0004530	triglyceride measurement	triglyceride measurement	EFO_0004530	Lipid or lipoprotein measurement	Metabolic	PGS003153	EFO_0004530	triglyceride measurement	triglyceride measurement	EFO_0004530	Lipid or lipoprotein measurement	Metabolic
0.8472	4.71E-127	1.21E-123	PGS001363	EFO_0004731	eye measurement	eye measurement	EFO_0004731	Other measurement	Ophthalmological	PGS001367	EFO_0004731	eye measurement	eye measurement	EFO_0004731	Other measurement	Ophthalmological
0.8472	4.93E-127	1.26E-123	PGS001344	MONDO_0004979	asthma	asthma	MONDO_0004979	Other disease	Respiratory	PGS001346	MONDO_0004979	asthma	asthma	MONDO_0004979	Other disease	Respiratory
0.8471	5.15E-127	1.32E-123	PGS000476	EFO_0000305	breast carcinoma	breast carcinoma	EFO_0000305	Cancer	Neoplasms	PGS000478	EFO_0000305	breast carcinoma	breast carcinoma	EFO_0000305	Cancer	Neoplasms
0.847	5.72E-127	1.46E-123	PGS000702	EFO_0004631	vitamin D measurement	vitamin D measurement	EFO_0004631	Lipid or lipoprotein measurement	Metabolic	PGS001982	EFO_0004631	vitamin D measurement	vitamin D measurement	EFO_0004631	Lipid or lipoprotein measurement	Metabolic
0.847	6.00E-127	1.53E-123	PGS002352	EFO_0004574	total cholesterol measurement	total cholesterol measurement	EFO_0004574	Lipid or lipoprotein measurement	Metabolic	PGS001933	EFO_0004611	low density lipoprotein cholesterol measurement	measurement	EFO_0004611	Cardiovascular measurement	Cardiovascular
0.847	6.00E-127	1.53E-123	PGS002352	EFO_0004574	total cholesterol measurement	total cholesterol measurement	EFO_0004574	Lipid or lipoprotein measurement	Metabolic	PGS001933	EFO_0004611	low density lipoprotein cholesterol measurement	measurement	EFO_0004611	Lipid or lipoprotein measurement	Metabolic
0.847	6.39E-127	1.63E-123	PGS002765	EFO_0000537	hypertension	hypertension	EFO_0000537	Cardiovascular disease	Cardiovascular	PGS002807	EFO_0006335	systolic blood pressure	systolic blood pressure	EFO_0006335	Other measurement	Cardiovascular
0.8469	6.57E-127	1.68E-123	PGS002460	EFO_0004527	mean corpuscular hemoglobin	mean corpuscular hemoglobin	EFO_0004527	Hematological measurement	Hematological	PGS002607	EFO_0004527	mean corpuscular hemoglobin	mean corpuscular hemoglobin	EFO_0004527	Hematological measurement	Hematological
0.8468	7.81E-127	1.99E-123	PGS001149	EFO_0005409	fat body mass	fat body mass	EFO_0005409	Other measurement	Body measurement	PGS001228	EFO_0004340	body mass index	body mass index	EFO_0004340	Body measurement	Body measurement
0.8468	8.03E-127	2.05E-123	PGS001943	EFO_0004340	body mass index	body mass index	EFO_0004340	Body measurement	Body measurement	PGS001951	EFO_0005409	fat body mass	fat body mass	EFO_0005409	Other measurement	Body measurement
0.8466	9.92E-127	2.53E-123	PGS001162	EFO_0005093	hip circumference	hip circumference	EFO_0005093	Body measurement	Body measurement	PGS001144	EFO_0005409	fat body mass	fat body mass	EFO_0005409	Other measurement	Body measurement
0.8465	1.22E-126	3.11E-123	PGS001360	EFO_0004731	eye measurement	eye measurement	EFO_0004731	Other measurement	Ophthalmological	PGS001365	EFO_0004731	eye measurement	eye measurement	EFO_0004731	Other measurement	Ophthalmological
0.8465	1.25E-126	3.20E-123	PGS002987	EFO_0004339	body height	body height	EFO_0004339	Body measurement	Body measurement	PGS002977	EFO_0004339	body height	body height	EFO_0004339	Body measurement	Body measurement
0.8464	1.32E-126	3.35E-123	PGS000112	EFO_0007986	reticulocyte count	reticulocyte count	EFO_0007986	Hematological measurement	Hematological	PGS000094	EFO_0007986	reticulocyte count	reticulocyte count	EFO_0007986	Hematological measurement	Hematological
0.8464	1.40E-126	3.56E-123	PGS002855	EFO_0004340	body mass index	body mass index	EFO_0004340	Body measurement	Body measurement	PGS002849	EFO_0004340	body mass index	body mass index	EFO_0004340	Body measurement	Body measurement
0.8463	1.61E-126	4.09E-123	PGS001230	EFO_0004338	body weight	body weight	EFO_0004338	Body measurement	Body measurement	PGS001147	EFO_0005409	fat body mass	fat body mass	EFO_0005409	Other measurement	Body measurement
0.8463	1.65E-126	4.19E-123	PGS003380	EFO_0000305	breast carcinoma	breast carcinoma	EFO_0000305	Cancer	Neoplasms	PGS000335	EFO_0000305	breast carcinoma	breast carcinoma	EFO_0000305	Cancer	Neoplasms
0.8463	1.67E-126	4.25E-123	PGS000783	EFO_0000305	breast carcinoma	breast carcinoma	EFO_0000305	Cancer	Neoplasms	PGS000873	EFO_0000305	breast carcinoma	breast carcinoma	EFO_0000305	Cancer	Neoplasms
0.8462	1.70E-126	4.32E-123	PGS000499	EFO_0000305	breast carcinoma	breast carcinoma	EFO_0000305	Cancer	Neoplasms	PGS000332	EFO_0000305	breast carcinoma	breast carcinoma	EFO_0000305	Cancer	Neoplasms
0.8462	1.71E-126	4.35E-123	PGS001174	EFO_0004799	cholelithiasis	cholelithiasis	EFO_0004799	Digestive system disorder	Gastrointestinal	PGS000942	HP_0001082	Cholecystitis	Cholecystitis	HP_0001082	Other trait	Gastrointestinal
0.8462	1.92E-126	4.86E-123	PGS002337	EFO_0004611	low density lipoprotein cholesterol measurement	low density lipoprotein cholesterol measurement	EFO_0004611	Lipid or lipoprotein measurement	Metabolic	PGS002654	EFO_0004611	low density lipoprotein cholesterol measurement	measurement	EFO_0004611	Lipid or lipoprotein measurement	Metabolic
0.8462	1.92E-126	4.86E-123	PGS002337	EFO_0004611	low density lipoprotein cholesterol measurement	low density lipoprotein cholesterol measurement	EFO_0004611	Cardiovascular measurement	Cardiovascular	PGS002654	EFO_0004611	low density lipoprotein cholesterol measurement	measurement	EFO_0004611	Lipid or lipoprotein measurement	Metabolic
0.8462	1.92E-126	4.86E-123	PGS002337	EFO_0004611	low density lipoprotein cholesterol measurement	low density lipoprotein cholesterol measurement	EFO_0004611	Lipid or lipoprotein measurement	Metabolic	PGS002654	EFO_0004611	low density lipoprotein cholesterol measurement	measurement	EFO_0004611	Cardiovascular measurement	Cardiovascular
0.8462	1.92E-126	4.86E-123	PGS002337	EFO_0004611	low density lipoprotein cholesterol measurement	low density lipoprotein cholesterol measurement	EFO_0004611	Cardiovascular measurement	Cardiovascular	PGS002654	EFO_0004611	low density lipoprotein cholesterol measurement	measurement	EFO_0004611	Cardiovascular measurement	Cardiovascular
0.8461	2.03E-126	5.15E-123	PGS002501	EFO_0004541	HbA1c measurement	HbA1c measurement	EFO_0004541	Hematological measurement	Hematological	PGS002403	EFO_0004541	HbA1c measurement	HbA1c measurement	EFO_0004541	Hematological measurement	Hematological
0.846	2.40E-126	6.07E-123	PGS002012	EFO_0011015	educational attainment	educational attainment	EFO_0011015	Other measurement	Activities	PGS002231	EFO_0011015	educational attainment	educational attainment	EFO_0011015	Other measurement	Activities
0.846	2.43E-126	6.14E-123	PGS001943	EFO_0004340	body mass index	body mass index	EFO_0004340	Body measurement	Body measurement	PGS001983	EFO_0004342	waist circumference	waist circumference	EFO_0004342	Body measurement	Body measurement
0.8459	2.59E-126	6.55E-123	PGS001327	EFO_0000400	diabetes mellitus	diabetes mellitus	EFO_0000400	Metabolic disorder	Metabolic	PGS001294	MONDO_0005148	type 2 diabetes mellitus	type 2 diabetes mellitus	MONDO_0005148	Digestive system disorder	Gastrointestinal
0.8459	2.59E-126	6.55E-123	PGS001327	EFO_0000400	diabetes mellitus	diabetes mellitus	EFO_0000400	Digestive system disorder	Gastrointestinal	PGS001294	MONDO_0005148	type 2 diabetes mellitus	type 2 diabetes mellitus	MONDO_0005148	Metabolic disorder	Metabolic
0.8459	2.59E-126	6.55E-123	PGS001327	EFO_0000400	diabetes mellitus	diabetes mellitus	EFO_0000400	Metabolic disorder	Metabolic	PGS001294	MONDO_0005148	type 2 diabetes mellitus	type 2 diabetes mellitus	MONDO_0005148	Metabolic disorder	Metabolic
0.8459	2.59E-126	6.55E-123	PGS001327	EFO_0000400	diabetes mellitus	diabetes mellitus	EFO_0000400	Digestive system disorder	Gastrointestinal	PGS001294	MONDO_0005148	type 2 diabetes mellitus	type 2 diabetes mellitus	MONDO_0005148	Digestive system disorder	Gastrointestinal
0.8458	3.23E-126	8.15E-123	PGS002396	EFO_0001379	endocrine system disease|diabetes mellitus	endocrine system disease	EFO_0001379	Other disease	Metabolic	PGS002491	EFO_0000400	diabetes mellitus	diabetes mellitus	EFO_0000400	Digestive system disorder	Gastrointestinal
0.8458	3.23E-126	8.15E-123	PGS002396	EFO_0001379	endocrine system disease|diabetes mellitus	endocrine system disease	EFO_0001379	Other disease	Metabolic	PGS002491	EFO_0000400	diabetes mellitus	diabetes mellitus	EFO_0000400	Metabolic disorder	Metabolic
0.8457	3.66E-126	9.24E-123	PGS002556	EFO_0004611	low density lipoprotein cholesterol measurement	low density lipoprotein cholesterol measurement	EFO_0004611	Cardiovascular measurement	Cardiovascular	PGS002424	EFO_0004574	total cholesterol measurement	total cholesterol measurement	EFO_0004574	Lipid or lipoprotein measurement	Metabolic
0.8457	3.66E-126	9.24E-123	PGS002556	EFO_0004611	low density lipoprotein cholesterol measurement	low density lipoprotein cholesterol measurement	EFO_0004611	Lipid or lipoprotein measurement	Metabolic	PGS002424	EFO_0004574	total cholesterol measurement	total cholesterol measurement	EFO_0004574	Lipid or lipoprotein measurement	Metabolic
0.8457	3.67E-126	9.26E-123	PGS001106	EFO_0007800	body fat percentage	body fat percentage	EFO_0007800	Body measurement	Body measurement	PGS001228	EFO_0004340	body mass index	body mass index	EFO_0004340	Body measurement	Body measurement
-0.8456	4.15E-126	1.05E-122	PGS001371	EFO_0004918	age at diagnosis|diabetes mellitus	age at diagnosis	EFO_0004918	Other measurement	Metabolic	PGS001296	MONDO_0005147	type 1 diabetes mellitus	type 1 diabetes mellitus	MONDO_0005147	Immune system disorder	Immunological
-0.8456	4.15E-126	1.05E-122	PGS001371	EFO_0004918	age at diagnosis|diabetes mellitus	age at diagnosis	EFO_0004918	Other measurement	Metabolic	PGS001296	MONDO_0005147	type 1 diabetes mellitus	type 1 diabetes mellitus	MONDO_0005147	Digestive system disorder	Gastrointestinal
-0.8456	4.15E-126	1.05E-122	PGS001371	EFO_0004918	age at diagnosis|diabetes mellitus	age at diagnosis	EFO_0004918	Other measurement	Metabolic	PGS001296	MONDO_0005147	type 1 diabetes mellitus	type 1 diabetes mellitus	MONDO_0005147	Metabolic disorder	Metabolic
0.8456	4.31E-126	1.09E-122	PGS003193	EFO_0004631	vitamin D measurement	vitamin D measurement	EFO_0004631	Lipid or lipoprotein measurement	Metabolic	PGS001982	EFO_0004631	vitamin D measurement	vitamin D measurement	EFO_0004631	Lipid or lipoprotein measurement	Metabolic
0.8455	4.37E-126	1.10E-122	PGS001363	EFO_0004731	eye measurement	eye measurement	EFO_0004731	Other measurement	Ophthalmological	PGS001366	EFO_0004731	eye measurement	eye measurement	EFO_0004731	Other measurement	Ophthalmological
-0.8455	4.68E-126	1.18E-122	PGS001986	EFO_0007993	lymphocyte percentage of leukocytes	lymphocyte percentage of leukocytes	EFO_0007993	Hematological measurement	Hematological	PGS002214	EFO_0007990	neutrophil percentage of leukocytes	neutrophil percentage of leukocytes	EFO_0007990	Hematological measurement	Hematological
0.8455	4.71E-126	1.19E-122	PGS003140	EFO_0004574	total cholesterol measurement	total cholesterol measurement	EFO_0004574	Lipid or lipoprotein measurement	Metabolic	PGS003030	EFO_0004611	low density lipoprotein cholesterol measurement	measurement	EFO_0004611	Lipid or lipoprotein measurement	Metabolic
0.8455	4.71E-126	1.19E-122	PGS003140	EFO_0004574	total cholesterol measurement	total cholesterol measurement	EFO_0004574	Lipid or lipoprotein measurement	Metabolic	PGS003030	EFO_0004611	low density lipoprotein cholesterol measurement	measurement	EFO_0004611	Cardiovascular measurement	Cardiovascular
0.8455	4.75E-126	1.20E-122	PGS003036	EFO_0004611	low density lipoprotein cholesterol measurement	low density lipoprotein cholesterol measurement	EFO_0004611	Cardiovascular measurement	Cardiovascular	PGS003141	EFO_0004574	total cholesterol measurement	total cholesterol measurement	EFO_0004574	Lipid or lipoprotein measurement	Metabolic
0.8455	4.75E-126	1.20E-122	PGS003036	EFO_0004611	low density lipoprotein cholesterol measurement	low density lipoprotein cholesterol measurement	EFO_0004611	Lipid or lipoprotein measurement	Metabolic	PGS003141	EFO_0004574	total cholesterol measurement	total cholesterol measurement	EFO_0004574	Lipid or lipoprotein measurement	Metabolic
0.8455	4.92E-126	1.24E-122	PGS000956	EFO_0004514	bone quantitative ultrasound measurement	bone quantitative ultrasound measurement	EFO_0004514	Other measurement	Skeletal	PGS000953	EFO_0004514	bone quantitative ultrasound measurement	measurement	EFO_0004514	Other measurement	Skeletal
0.8455	4.93E-126	1.24E-122	PGS001039	EFO_0009270	heel bone mineral density	heel bone mineral density	EFO_0009270	Other measurement	Skeletal	PGS000953	EFO_0004514	bone quantitative ultrasound measurement	measurement	EFO_0004514	Other measurement	Skeletal

0.8455	4.93E-126	1.24E-122	PGS001230	EFO_0004338	body weight	body weight	EFO_0004338	Body measurement	Body measurement	PGS001167	EFO_0004995	lean body mass	lean body mass	EFO_0004995	Other measurement	Skeletal
0.8455	4.95E-126	1.24E-122	PGS000111	EFO_0007986	reticulocyte count	reticulocyte count	EFO_0007986	Hematological measurement	Hematological	PGS000094	EFO_0007986	reticulocyte count	reticulocyte count	EFO_0007986	Hematological measurement	Hematological
0.8454	5.58E-126	1.40E-122	PGS001834	EFO_0001365	age-related macular degeneration	age-related macular degeneration	EFO_0001365	Neurological disorder	Neurological	PGS001013	EFO_0009606	macular degeneration	macular degeneration	EFO_0009606	Neurological disorder	Neurological
-0.8453	6.13E-126	1.54E-122	PGS001997	EFO_0007990	neutrophil percentage of leukocytes	neutrophil percentage of leukocytes	EFO_0007990	Hematological measurement	Hematological	PGS002203	EFO_0007993	lymphocyte percentage of leukocytes	lymphocyte percentage of leukocytes	EFO_0007993	Hematological measurement	Hematological
0.8452	7.07E-126	1.77E-122	PGS002116	EFO_0005094	P wave duration	P wave duration	EFO_0005094	Cardiovascular measurement	Cardiovascular	PGS001902	EFO_0005094	P wave duration	P wave duration	EFO_0005094	Cardiovascular measurement	Cardiovascular
-0.8452	7.21E-126	1.81E-122	PGS001371	EFO_0004918	age at diagnosis|diabetes mellitus	age at diagnosis	EFO_0004918	Other measurement	Metabolic	PGS001014	EFO_0009605	pancreas disease	pancreas disease	EFO_0009605	Digestive system disorder	Gastrointestinal
0.8451	7.84E-126	1.96E-122	PGS003146	EFO_0004530	triglyceride measurement	triglyceride measurement	EFO_0004530	Lipid or lipoprotein measurement	Metabolic	PGS003151	EFO_0004530	triglyceride measurement	triglyceride measurement	EFO_0004530	Lipid or lipoprotein measurement	Metabolic
0.8451	8.51E-126	2.13E-122	PGS001946	EFO_0004458	C-reactive protein measurement	C-reactive protein measurement	EFO_0004458	Inflammatory measurement	Immunological	PGS000675	EFO_0004458	C-reactive protein measurement	C-reactive protein measurement	EFO_0004458	Inflammatory measurement	Immunological
0.845	8.84E-126	2.21E-122	PGS001956	EFO_0007772	calcaneal bone quantitative ultrasound measurement	calcaneal bone quantitative ultrasound measurement	EFO_0007772	Other measurement	Skeletal	PGS002315	EFO_0003923	bone density	bone density	EFO_0003923	Other measurement	Skeletal
0.845	9.70E-126	2.43E-122	PGS002386	EFO_0007825	balding measurement	balding measurement	EFO_0007825	Other measurement	Activities	PGS002533	EFO_0007825	balding measurement	balding measurement	EFO_0007825	Other measurement	Activities
0.8449	1.08E-125	2.70E-122	PGS001147	EFO_0005409	fat body mass	fat body mass	EFO_0005409	Other measurement	Body measurement	PGS001102	EFO_0007800	body fat percentage	body fat percentage	EFO_0007800	Body measurement	Body measurement
0.8448	1.15E-125	2.87E-122	PGS001809	EFO_0002892	thyroid carcinoma	thyroid carcinoma	EFO_0002892	Cancer	Neoplasms	PGS002018	EFO_0002892	thyroid carcinoma	thyroid carcinoma	EFO_0002892	Cancer	Neoplasms
-0.8448	1.18E-125	2.95E-122	PGS000014	MONDO_0005148	type 2 diabetes mellitus	type 2 diabetes mellitus	MONDO_0005148	Metabolic disorder	Metabolic	PGS001357	MONDO_0005148	type 2 diabetes mellitus	type 2 diabetes mellitus	MONDO_0005148	Metabolic disorder	Metabolic
-0.8448	1.18E-125	2.95E-122	PGS000014	MONDO_0005148	type 2 diabetes mellitus	type 2 diabetes mellitus	MONDO_0005148	Digestive system disorder	Gastrointestinal	PGS001357	MONDO_0005148	type 2 diabetes mellitus	type 2 diabetes mellitus	MONDO_0005148	Digestive system disorder	Gastrointestinal
-0.8448	1.18E-125	2.95E-122	PGS000014	MONDO_0005148	type 2 diabetes mellitus	type 2 diabetes mellitus	MONDO_0005148	Digestive system disorder	Gastrointestinal	PGS001357	MONDO_0005148	type 2 diabetes mellitus	type 2 diabetes mellitus	MONDO_0005148	Metabolic disorder	Metabolic
-0.8448	1.18E-125	2.95E-122	PGS000014	MONDO_0005148	type 2 diabetes mellitus	type 2 diabetes mellitus	MONDO_0005148	Metabolic disorder	Metabolic	PGS001357	MONDO_0005148	type 2 diabetes mellitus	type 2 diabetes mellitus	MONDO_0005148	Digestive system disorder	Gastrointestinal
0.8447	1.45E-125	3.61E-122	PGS000207	EFO_0002892	thyroid carcinoma	thyroid carcinoma	EFO_0002892	Cancer	Neoplasms	PGS000797	EFO_0002892	thyroid carcinoma	thyroid carcinoma	EFO_0002892	Cancer	Neoplasms
0.8446	1.60E-125	3.99E-122	PGS003076	EFO_0006335	systolic blood pressure	systolic blood pressure	EFO_0006335	Other measurement	Cardiovascular	PGS003075	EFO_0006335	systolic blood pressure	systolic blood pressure	EFO_0006335	Other measurement	Cardiovascular
0.8445	1.78E-125	4.43E-122	PGS003383	EFO_0000673	prostate adenocarcinoma	prostate adenocarcinoma	EFO_0000673	Cancer	Neoplasms	PGS002240	EFO_0001663	prostate carcinoma	prostate carcinoma	EFO_0001663	Cancer	Neoplasms
0.8444	2.08E-125	5.19E-122	PGS001147	EFO_0005409	fat body mass	fat body mass	EFO_0005409	Other measurement	Body measurement	PGS001227	EFO_0004342	waist circumference	waist circumference	EFO_0004342	Body measurement	Body measurement
0.8443	2.34E-125	5.82E-122	PGS002524	MONDO_0005148	type 2 diabetes mellitus	type 2 diabetes mellitus	MONDO_0005148	Metabolic disorder	Metabolic	PGS002426	MONDO_0005148	type 2 diabetes mellitus	type 2 diabetes mellitus	MONDO_0005148	Digestive system disorder	Gastrointestinal
0.8443	2.34E-125	5.82E-122	PGS002524	MONDO_0005148	type 2 diabetes mellitus	type 2 diabetes mellitus	MONDO_0005148	Digestive system disorder	Gastrointestinal	PGS002426	MONDO_0005148	type 2 diabetes mellitus	type 2 diabetes mellitus	MONDO_0005148	Metabolic disorder	Metabolic
0.8443	2.34E-125	5.82E-122	PGS002524	MONDO_0005148	type 2 diabetes mellitus	type 2 diabetes mellitus	MONDO_0005148	Digestive system disorder	Gastrointestinal	PGS002426	MONDO_0005148	type 2 diabetes mellitus	type 2 diabetes mellitus	MONDO_0005148	Digestive system disorder	Gastrointestinal
0.8443	2.34E-125	5.82E-122	PGS002524	MONDO_0005148	type 2 diabetes mellitus	type 2 diabetes mellitus	MONDO_0005148	Metabolic disorder	Metabolic	PGS002426	MONDO_0005148	type 2 diabetes mellitus	type 2 diabetes mellitus	MONDO_0005148	Metabolic disorder	Metabolic
0.8441	2.93E-125	7.28E-122	PGS001160	EFO_0005106	body composition measurement	body composition measurement	EFO_0005106	Body measurement	Body measurement	PGS001159	EFO_0005106	body composition measurement	body composition measurement	EFO_0005106	Body measurement	Body measurement
0.8441	3.20E-125	7.95E-122	PGS002283	EFO_0003095	non-alcoholic fatty liver disease	non-alcoholic fatty liver disease	EFO_0003095	Digestive system disorder	Gastrointestinal	PGS002732	EFO_0004735	serum alanine aminotransferase measurement	measurement	EFO_0004735	Liver enzyme measurement	Gastrointestinal
0.8439	3.99E-125	9.92E-122	PGS002200	EFO_0004631	vitamin D measurement	vitamin D measurement	EFO_0004631	Lipid or lipoprotein measurement	Metabolic	PGS003193	EFO_0004631	vitamin D measurement	vitamin D measurement	EFO_0004631	Lipid or lipoprotein measurement	Metabolic
-0.8438	4.65E-125	1.16E-121	PGS001955	EFO_0009270	heel bone mineral density	heel bone mineral density	EFO_0009270	Other measurement	Skeletal	PGS002768	EFO_0003882	osteoporosis	osteoporosis	EFO_0003882	Other disease	Skeletal
0.8437	5.53E-125	1.37E-121	PGS002782	EFO_0005689	non-high density lipoprotein cholesterol measurement	non-high density lipoprotein cholesterol measurement	EFO_0005689	Cardiovascular measurement	Cardiovascular	PGS000892	EFO_0004611	low density lipoprotein cholesterol measurement	measurement	EFO_0004611	Cardiovascular measurement	Cardiovascular
0.8437	5.53E-125	1.37E-121	PGS002782	EFO_0005689	non-high density lipoprotein cholesterol measurement	non-high density lipoprotein cholesterol measurement	EFO_0005689	Cardiovascular measurement	Cardiovascular	PGS000892	EFO_0004611	low density lipoprotein cholesterol measurement	measurement	EFO_0004611	Lipid or lipoprotein measurement	Metabolic
0.8437	5.53E-125	1.37E-121	PGS002782	EFO_0005689	non-high density lipoprotein cholesterol measurement	non-high density lipoprotein cholesterol measurement	EFO_0005689	Lipid or lipoprotein measurement	Metabolic	PGS000892	EFO_0004611	low density lipoprotein cholesterol measurement	measurement	EFO_0004611	Cardiovascular measurement	Cardiovascular
0.8437	5.53E-125	1.37E-121	PGS002782	EFO_0005689	non-high density lipoprotein cholesterol measurement	non-high density lipoprotein cholesterol measurement	EFO_0005689	Lipid or lipoprotein measurement	Metabolic	PGS000892	EFO_0004611	low density lipoprotein cholesterol measurement	measurement	EFO_0004611	Lipid or lipoprotein measurement	Metabolic
0.8435	6.60E-125	1.64E-121	PGS002540	EFO_0000400	diabetes mellitus	diabetes mellitus	EFO_0000400	Metabolic disorder	Metabolic	PGS002449	EFO_0004468	glucose measurement	glucose measurement	EFO_0004468	Other measurement	Metabolic
0.8435	6.60E-125	1.64E-121	PGS002540	EFO_0000400	diabetes mellitus	diabetes mellitus	EFO_0000400	Digestive system disorder	Gastrointestinal	PGS002449	EFO_0004468	glucose measurement	glucose measurement	EFO_0004468	Other measurement	Metabolic
0.8435	7.21E-125	1.79E-121	PGS001236	EFO_0004314	forced expiratory volume	forced expiratory volume	EFO_0004314	Other measurement	Respiratory	PGS001237	EFO_0004312	vital capacity	vital capacity	EFO_0004312	Other measurement	Activities
0.8434	7.52E-125	1.87E-121	PGS002200	EFO_0004631	vitamin D measurement	vitamin D measurement	EFO_0004631	Lipid or lipoprotein measurement	Metabolic	PGS003198	EFO_0004631	vitamin D measurement	vitamin D measurement	EFO_0004631	Lipid or lipoprotein measurement	Metabolic
0.8433	8.54E-125	2.12E-121	PGS000001	EFO_0000305	breast carcinoma	breast carcinoma	EFO_0000305	Cancer	Neoplasms	PGS000046	EFO_1000649	estrogen-receptor positive breast cancer	cancer	EFO_1000649	Cancer	Neoplasms
0.8432	9.55E-125	2.37E-121	PGS002174	EFO_0007772	calcaneal bone quantitative ultrasound measurement	calcaneal bone quantitative ultrasound measurement	EFO_0007772	Other measurement	Skeletal	PGS001957	EFO_0005654	velocity of sound measurement	velocity of sound measurement	EFO_0005654	Other measurement	Others
0.843	1.41E-124	3.49E-121	PGS002524	MONDO_0005148	type 2 diabetes mellitus	type 2 diabetes mellitus	MONDO_0005148	Metabolic disorder	Metabolic	PGS002393	EFO_0000400	diabetes mellitus	diabetes mellitus	EFO_0000400	Metabolic disorder	Metabolic
0.843	1.41E-124	3.49E-121	PGS002524	MONDO_0005148	type 2 diabetes mellitus	type 2 diabetes mellitus	MONDO_0005148	Digestive system disorder	Gastrointestinal	PGS002393	EFO_0000400	diabetes mellitus	diabetes mellitus	EFO_0000400	Digestive system disorder	Gastrointestinal
0.843	1.41E-124	3.49E-121	PGS002524	MONDO_0005148	type 2 diabetes mellitus	type 2 diabetes mellitus	MONDO_0005148	Metabolic disorder	Metabolic	PGS002393	EFO_0000400	diabetes mellitus	diabetes mellitus	EFO_0000400	Digestive system disorder	Gastrointestinal
0.843	1.41E-124	3.49E-121	PGS002524	MONDO_0005148	type 2 diabetes mellitus	type 2 diabetes mellitus	MONDO_0005148	Digestive system disorder	Gastrointestinal	PGS002393	EFO_0000400	diabetes mellitus	diabetes mellitus	EFO_0000400	Metabolic disorder	Metabolic
0.8429	1.58E-124	3.92E-121	PGS003135	EFO_0004574	total cholesterol measurement	total cholesterol measurement	EFO_0004574	Lipid or lipoprotein measurement	Metabolic	PGS003035	EFO_0004611	low density lipoprotein cholesterol measurement	measurement	EFO_0004611	Cardiovascular measurement	Cardiovascular
0.8429	1.58E-124	3.92E-121	PGS003135	EFO_0004574	total cholesterol measurement	total cholesterol measurement	EFO_0004574	Lipid or lipoprotein measurement	Metabolic	PGS003035	EFO_0004611	low density lipoprotein cholesterol measurement	measurement	EFO_0004611	Lipid or lipoprotein measurement	Metabolic
0.8428	1.63E-124	4.04E-121	PGS001106	EFO_0007800	body fat percentage	body fat percentage	EFO_0007800	Body measurement	Body measurement	PGS001147	EFO_0005409	fat body mass	fat body mass	EFO_0005409	Other measurement	Body measurement
0.8428	1.68E-124	4.16E-121	PGS001888	EFO_0004614	apolipoprotein A 1 measurement	apolipoprotein A 1 measurement	EFO_0004614	Cardiovascular measurement	Cardiovascular	PGS002172	EFO_0004612	high density lipoprotein cholesterol measurement	measurement	EFO_0004612	Lipid or lipoprotein measurement	Metabolic
0.8428	1.68E-124	4.16E-121	PGS001888	EFO_0004614	apolipoprotein A 1 measurement	apolipoprotein A 1 measurement	EFO_0004614	Lipid or lipoprotein measurement	Metabolic	PGS002172	EFO_0004612	high density lipoprotein cholesterol measurement	measurement	EFO_0004612	Lipid or lipoprotein measurement	Metabolic
0.8428	1.68E-124	4.16E-121	PGS001888	EFO_0004614	apolipoprotein A 1 measurement	apolipoprotein A 1 measurement	EFO_0004614	Inflammatory measurement	Immunological	PGS002172	EFO_0004612	high density lipoprotein cholesterol measurement	measurement	EFO_0004612	Lipid or lipoprotein measurement	Metabolic
0.8427	1.86E-124	4.60E-121	PGS001168	EFO_0004995	lean body mass	lean body mass	EFO_0004995	Other measurement	Skeletal	PGS001159	EFO_0005106	body composition measurement	body composition measurement	EFO_0005106	Body measurement	Body measurement
0.8427	1.93E-124	4.78E-121	PGS003273	EFO_0010472	creatine measurement|glomerular filtration rate	creatine measurement	EFO_0010472	Other measurement	Metabolic	PGS003269	EFO_0010472	creatine measurement|glomerular filtration rate	creatine measurement	EFO_0010472	Other measurement	Metabolic
0.8426	2.21E-124	5.45E-121	PGS001230	EFO_0004338	body weight	body weight	EFO_0004338	Body measurement	Body measurement	PGS001159	EFO_0005106	body composition measurement	body composition measurement	EFO_0005106	Body measurement	Body measurement
-0.8424	2.80E-124	6.90E-121	PGS003376	EFO_0007878	alcohol consumption measurement	alcohol consumption measurement	EFO_0007878	Other measurement	Psychiatric	PGS000203	EFO_0007878	alcohol consumption measurement	alcohol consumption measurement	EFO_0007878	Other measurement	Psychiatric
0.8423	3.13E-124	7.71E-121	PGS002894	EFO_0006336	diastolic blood pressure	diastolic blood pressure	EFO_0006336	Other measurement	Cardiovascular	PGS002893	EFO_0006336	diastolic blood pressure	diastolic blood pressure	EFO_0006336	Other measurement	Cardiovascular
0.8423	3.22E-124	7.95E-121	PGS001160	EFO_0005106	body composition measurement	body composition measurement	EFO_0005106	Body measurement	Body measurement	PGS001166	EFO_0004995	lean body mass	lean body mass	EFO_0004995	Other measurement	Skeletal
0.8423	3.38E-124	8.32E-121	PGS003251	EFO_0010472	creatine measurement|glomerular filtration rate	creatine measurement	EFO_0010472	Other measurement	Metabolic	PGS003265	EFO_0010472	creatine measurement|glomerular filtration rate	creatine measurement	EFO_0010472	Other measurement	Metabolic
0.8423	3.38E-124	8.32E-121	PGS003250	EFO_0010472	creatine measurement|glomerular filtration rate	creatine measurement	EFO_0010472	Other measurement	Metabolic	PGS003265	EFO_0010472	creatine measurement|glomerular filtration rate	creatine measurement	EFO_0010472	Other measurement	Metabolic
0.8422	3.83E-124	9.43E-121	PGS001145	EFO_0005409	fat body mass	fat body mass	EFO_0005409	Other measurement	Body measurement	PGS001227	EFO_0004342	waist circumference	waist circumference	EFO_0004342	Body measurement	Body measurement
0.8422	3.96E-124	9.75E-121	PGS001160	EFO_0005106	body composition measurement	body composition measurement	EFO_0005106	Body measurement	Body measurement	PGS001167	EFO_0004995	lean body mass	lean body mass	EFO_0004995	Other measurement	Skeletal
0.8421	4.59E-124	1.13E-120	PGS000783	EFO_0000305	breast carcinoma	breast carcinoma	EFO_0000305	Cancer	Neoplasms	PGS003398	EFO_0000305	breast carcinoma	breast carcinoma	EFO_0000305	Cancer	Neoplasms
0.842	4.66E-124	1.14E-120	PGS001160	EFO_0005106	body composition measurement	body composition measurement	EFO_0005106	Body measurement	Body measurement	PGS001158	EFO_0005106	body composition measurement	body composition measurement	EFO_0005106	Body measurement	Body measurement
-0.842	4.78E-124	1.17E-120	PGS001371	EFO_0004918	age at diagnosis|diabetes mellitus	age at diagnosis	EFO_0004918	Other measurement	Metabolic	PGS001820	HP_0001943	Hypoglycemia	Hypoglycemia	HP_0001943	Other trait	Metabolic
0.8419	5.44E-124	1.33E-120	PGS000076	EFO_0000681	renal cell carcinoma	renal cell carcinoma	EFO_0000681	Cancer	Neoplasms	PGS000722	EFO_0002890	renal carcinoma	renal carcinoma	EFO_0002890	Cancer	Neoplasms
0.8419	5.47E-124	1.34E-120	PGS001314	EFO_0000612	myocardial infarction	myocardial infarction	EFO_0000612	Cardiovascular disease	Cardiovascular	PGS001316	EFO_0000612	myocardial infarction	myocardial infarction	EFO_0000612	Cardiovascular disease	Cardiovascular
0.8419	5.79E-124	1.42E-120	PGS003266	EFO_0010472	creatine measurement|glomerular filtration rate	creatine measurement	EFO_0010472	Other measurement	Metabolic	PGS003251	EFO_0010472	creatine measurement|glomerular filtration rate	creatine measurement	EFO_0010472	Other measurement	Metabolic
0.8419	5.79E-124	1.42E-120	PGS003266	EFO_0010472	creatine measurement|glomerular filtration rate	creatine measurement	EFO_0010472	Other measurement	Metabolic	PGS003250	EFO_0010472	creatine measurement|glomerular filtration rate	creatine measurement	EFO_0010472	Other measurement	Metabolic
0.8419	5.99E-124	1.47E-120	PGS003065	EFO_0006527	smoking status measurement	smoking status measurement	EFO_0006527	Other measurement	Psychiatric	PGS003060	EFO_0006527	smoking status measurement	smoking status measurement	EFO_0006527	Other measurement	Psychiatric
0.8418	6.84E-124	1.67E-120	PGS000955	EFO_0004514	bone quantitative ultrasound measurement	bone quantitative ultrasound measurement	EFO_0004514	Other measurement	Skeletal	PGS001403	EFO_0009270	heel bone mineral density	heel bone mineral density	EFO_0009270	Other measurement	Skeletal
0.8417	6.92E-124	1.69E-120	PGS003380	EFO_0000305	breast carcinoma	breast carcinoma	EFO_0000305	Cancer	Neoplasms	PGS000508	EFO_0000305	breast carcinoma	breast carcinoma	EFO_0000305	Cancer	Neoplasms
0.8417	7.40E-124	1.81E-120	PGS002850	EFO_0004340	body mass index	body mass index	EFO_0004340	Body measurement	Body measurement	PGS002849	EFO_0004340	body mass index	body mass index	EFO_0004340	Body measurement	Body measurement
0.8416	7.94E-124	1.94E-120	PGS003144	EFO_0004530	triglyceride measurement	triglyceride measurement	EFO_0004530	Lipid or lipoprotein measurement	Metabolic	PGS003148	EFO_0004530	triglyceride measurement	triglyceride measurement	EFO_0004530	Lipid or lipoprotein measurement	Metabolic
0.8415	9.90E-124	2.42E-120	PGS002973	EFO_0004339	body height	body height	EFO_0004339	Body measurement	Body measurement	PGS002969	EFO_0004339	body height	body height	EFO_0004339	Body measurement	Body measurement
0.8415	9.95E-124	2.43E-120	PGS000987	EFO_0010701	mean reticulocyte volume	mean reticulocyte volume	EFO_0010701	Hematological measurement	Hematological	PGS002003	EFO_0010701	mean reticulocyte volume	mean reticulocyte volume	EFO_0010701	Hematological measurement	Hematological
0.8415	1.00E-123	2.44E-120	PGS003239	EFO_0010472	creatine measurement	creatine measurement	EFO_0010472	Other measurement	Metabolic	PGS003243	EFO_0010472	creatine measurement	creatine measurement	EFO_0010472	Other measurement	Metabolic
0.8414	1.03E-123	2.50E-120	PGS000347	EFO_1000649	estrogen-receptor positive breast cancer	estrogen-receptor positive breast cancer	EFO_1000649	Cancer	Neoplasms	PGS000213	EFO_0009780	HER2 negative breast carcinoma	HER2 negative breast carcinoma	EFO_0009780	Cancer	Neoplasms
0.8414	1.09E-123	2.65E-120	PGS001278	EFO_0003827	pulmonary embolism|deep vein thrombosis	pulmonary embolism	EFO_0003827	Cardiovascular disease	Cardiovascular	PGS001266	EFO_0003907	deep vein thrombosis	deep vein thrombosis	EFO_0003907	Other trait	Cardiovascular
0.8413	1.21E-123	2.94E-120	PGS001168	EFO_0004995	lean body mass	lean body mass	EFO_0004995	Other measurement	Skeletal	PGS001166	EFO_0004995	lean body mass	lean body mass	EFO_0004995	Other measurement	Skeletal
0.8411	1.54E-123	3.75E-120	PGS001544	EFO_0005420	measurement	grey matter volume measurement	EFO_0005420	Other measurement	Neurological	PGS001540	EFO_0008320	volume measurement|neuroimaging measurement	white matter volume measurement	EFO_0008320	Other measurement	Neurological
0.841	1.78E-123	4.32E-120	PGS001158	EFO_0005106	body composition measurement	body composition measurement	EFO_0005106	Body measurement	Body measurement	PGS001168	EFO_0004995	lean body mass	lean body mass	EFO_0004995	Other measurement	Skeletal
0.841	1.80E-123	4.38E-120	PGS002353	EFO_0004530	triglyceride measurement	triglyceride measurement	EFO_0004530	Lipid or lipoprotein measurement	Metabolic	PGS003153	EFO_0004530	triglyceride measurement	triglyceride measurement	EFO_0004530	Lipid or lipoprotein measurement	Metabolic
0.841	1.93E-123	4.70E-120	PGS001168	EFO_0004995	lean body mass	lean body mass	EFO_0004995	Other measurement	Skeletal	PGS001167	EFO_0004995	lean body mass	lean body mass	EFO_0004995	Other measurement	Skeletal
0.841	1.94E-123	4.72E-120	PGS002164	EFO_0004458	C-reactive protein measurement	C-reactive protein measurement	EFO_0004458	Inflammatory measurement	Immunological	PGS002868	EFO_0004458	C-reactive protein measurement	C-reactive protein measurement	EFO_0004458	Inflammatory measurement	Immunological
0.8409	1.98E-123	4.81E-120	PGS001819	EFO_0003770	diabetic retinopathy	diabetic retinopathy	EFO_0003770	Neurological disorder	Neurological	PGS002027	EFO_0003770	diabetic retinopathy	diabetic retinopathy	EFO_0003770	Cardiovascular disease	Cardiovascular
0.8409	1.98E-123	4.81E-120	PGS001819	EFO_0003770	diabetic retinopathy	diabetic retinopathy	EFO_0003770	Neurological disorder	Neurological	PGS002027	EFO_0003770	diabetic retinopathy	diabetic retinopathy	EFO_0003770	Neurological disorder	Neurological
0.8409	1.98E-123	4.81E-120	PGS001819	EFO_0003770	diabetic retinopathy	diabetic retinopathy	EFO_0003770	Neurological disorder	Neurological	PGS002027	EFO_0003770	diabetic retinopathy	diabetic retinopathy	EFO_0003770	Metabolic disorder	Metabolic
0.8409	1.98E-123	4.81E-120	PGS001819	EFO_0003770	diabetic retinopathy	diabetic retinopathy	EFO_0003770	Cardiovascular disease	Cardiovascular	PGS002027	EFO_0003770	diabetic retinopathy	diabetic retinopathy	EFO_0003770	Cardiovascular disease	Cardiovascular
0.8409	1.98E-123	4.81E-120	PGS001819	EFO_0003770	diabetic retinopathy	diabetic retinopathy	EFO_0003770	Metabolic disorder	Metabolic	PGS002027	EFO_0003770	diabetic retinopathy	diabetic retinopathy	EFO_0003770	Cardiovascular disease	Cardiovascular
0.8409	1.98E-123	4.81E-120	PGS001819	EFO_0003770	diabetic retinopathy	diabetic retinopathy	EFO_0003770	Metabolic disorder	Metabolic	PGS002027	EFO_0003770	diabetic retinopathy	diabetic retinopathy	EFO_0003770	Metabolic disorder	Metabolic
0.8409	1.98E-123	4.81E-120	PGS001819	EFO_0003770	diabetic retinopathy	diabetic retinopathy	EFO_0003770	Cardiovascular disease	Cardiovascular	PGS002027	EFO_0003770	diabetic retinopathy	diabetic retinopathy	EFO_0003770	Neurological disorder	Neurological
0.8409	1.98E-123	4.81E-120	PGS001819	EFO_0003770	diabetic retinopathy	diabetic retinopathy	EFO_0003770	Metabolic disorder	Metabolic	PGS002027	EFO_0003770	diabetic retinopathy	diabetic retinopathy	EFO_0003770	Neurological disorder	Neurological
0.8409	1.98E-123	4.81E-120	PGS001819	EFO_0003770	diabetic retinopathy	diabetic retinopathy	EFO_0003770	Cardiovascular disease	Cardiovascular	PGS002027	EFO_0003770	diabetic retinopathy	diabetic retinopathy	EFO_0003770	Metabolic disorder	Metabolic
0.8407	2.60E-123	6.32E-120	PGS002454	EFO_0007986	reticulocyte count	reticulocyte count	EFO_0007986	Hematological measurement	Hematological	PGS002552	EFO_0007986	reticulocyte count	reticulocyte count	EFO_0007986	Hematological measurement	Hematological
0.8407	2.87E-123	6.98E-120	PGS002337	EFO_0004611	low density lipoprotein cholesterol measurement	low density lipoprotein cholesterol measurement	EFO_0004611	Lipid or lipoprotein measurement	Metabolic	PGS000688	EFO_0004611	low density lipoprotein cholesterol measurement	measurement	EFO_0004611	Cardiovascular measurement	Cardiovascular
0.8407	2.87E-123	6.98E-120	PGS002337	EFO_0004611	low density lipoprotein cholesterol measurement	low density lipoprotein cholesterol measurement	EFO_0004611	Cardiovascular measurement	Cardiovascular	PGS000688	EFO_0004611	low density lipoprotein cholesterol measurement	measurement	EFO_0004611	Cardiovascular measurement	Cardiovascular
0.8407	2.87E-123	6.98E-120	PGS002337	EFO_0004611	low density lipoprotein cholesterol measurement	low density lipoprotein cholesterol measurement	EFO_0004611	Cardiovascular measurement	Cardiovascular	PGS000688	EFO_0004611	low density lipoprotein cholesterol measurement	measurement	EFO_0004611	Lipid or lipoprotein measurement	Metabolic
0.8407	2.87E-123	6.98E-120	PGS002337	EFO_0004611	low density lipoprotein cholesterol measurement	low density lipoprotein cholesterol measurement	EFO_0004611	Lipid or lipoprotein measurement	Metabolic	PGS000688	EFO_0004611	low density lipoprotein cholesterol measurement	measurement	EFO_0004611	Lipid or lipoprotein measurement	Metabolic
0.8406	3.14E-123	7.61E-120	PGS002137	EFO_0003931	bone fracture	bone fracture	EFO_0003931	Other disease	Skeletal	PGS001921	EFO_0003931	bone fracture	bone fracture	EFO_0003931	Other disease	Skeletal
0.8406	3.31E-123	8.03E-120	PGS002217	EFO_0010724	lifestyle measurement	lifestyle measurement	EFO_0010724	Other measurement	Activities	PGS001999	EFO_0010724	lifestyle measurement	lifestyle measurement	EFO_0010724	Other measurement	Activities
0.8405	3.35E-123	8.11E-120	PGS003312	EFO_0004617	cystatin C measurement|glomerular filtration rate	cystatin C measurement	EFO_0004617	Other measurement	Metabolic	PGS003314	EFO_0004617	cystatin C measurement|glomerular filtration rate	cystatin C measurement	EFO_0004617	Other measurement	Metabolic
0.8405	3.45E-123	8.36E-120	PGS001817	MONDO_0005147	type 1 diabetes mellitus	type 1 diabetes mellitus	MONDO_0005147	Metabolic disorder	Metabolic	PGS001820	HP_0001943	Hypoglycemia	Hypoglycemia	HP_0001943	Other trait	Metabolic
0.8405	3.45E-123	8.36E-120	PGS001817	MONDO_0005147	type 1 diabetes mellitus	type 1 diabetes mellitus	MONDO_0005147	Immune system disorder	Immunological	PGS001820	HP_0001943	Hypoglycemia	Hypoglycemia	HP_0001943	Other trait	Metabolic
0.8405	3.45E-123	8.36E-120	PGS001817	MONDO_0005147	type 1 diabetes mellitus	type 1 diabetes mellitus	MONDO_0005147	Digestive system disorder	Gastrointestinal	PGS001820	HP_0001943	Hypoglycemia	Hypoglycemia	HP_0001943	Other trait	Metabolic
0.8404	3.83E-123	9.27E-120	PGS001200	EFO_0004584	mean platelet volume	mean platelet volume	EFO_0004584	Hematological measurement	Hematological	PGS002340	EFO_0004584	mean platelet volume	mean platelet volume	EFO_0004584	Hematological measurement	Hematological
0.8404	3.93E-123	9.50E-120	PGS002041	EFO_0001365	age-related macular degeneration	age-related macular degeneration	EFO_0001365	Neurological disorder	Neurological	PGS001013	EFO_0009606	macular degeneration	macular degeneration	EFO_0009606	Neurological disorder	Neurological

0.8404	3.98E-123	9.62E-120	PGS003297	EFO_0004617	cystatin C measurement|glomerular filtration rate	cystatin C measurement	EFO_0004617	Other measurement	Metabolic	PGS003314	EFO_0004617	cystatin C measurement|glomerular filtration rate	cystatin C measurement	EFO_0004617	Other measurement	Metabolic
0.8404	4.22E-123	1.02E-119	PGS002392	EFO_0000701	skin disease	skin disease	EFO_0000701	Other disease	Dermatological	PGS002441	EFO_0000701	skin disease	skin disease	EFO_0000701	Other disease	Dermatological
0.8403	4.42E-123	1.07E-119	PGS002466	EFO_0004305	erythrocyte count	erythrocyte count	EFO_0004305	Hematological measurement	Hematological	PGS002564	EFO_0004305	erythrocyte count	erythrocyte count	EFO_0004305	Hematological measurement	Hematological
0.8403	4.69E-123	1.13E-119	PGS001817	MONDO_0005147	type 1 diabetes mellitus	type 1 diabetes mellitus	MONDO_0005147	Metabolic disorder	Metabolic	PGS001297	MONDO_0005147	type 1 diabetes mellitus	type 1 diabetes mellitus	MONDO_0005147	Metabolic disorder	Metabolic
0.8403	4.69E-123	1.13E-119	PGS001817	MONDO_0005147	type 1 diabetes mellitus	type 1 diabetes mellitus	MONDO_0005147	Metabolic disorder	Metabolic	PGS001297	MONDO_0005147	type 1 diabetes mellitus	type 1 diabetes mellitus	MONDO_0005147	Digestive system disorder	Gastrointestinal
0.8403	4.69E-123	1.13E-119	PGS001817	MONDO_0005147	type 1 diabetes mellitus	type 1 diabetes mellitus	MONDO_0005147	Immune system disorder	Immunological	PGS001297	MONDO_0005147	type 1 diabetes mellitus	type 1 diabetes mellitus	MONDO_0005147	Metabolic disorder	Metabolic
0.8403	4.69E-123	1.13E-119	PGS001817	MONDO_0005147	type 1 diabetes mellitus	type 1 diabetes mellitus	MONDO_0005147	Immune system disorder	Immunological	PGS001297	MONDO_0005147	type 1 diabetes mellitus	type 1 diabetes mellitus	MONDO_0005147	Immune system disorder	Immunological
0.8403	4.69E-123	1.13E-119	PGS001817	MONDO_0005147	type 1 diabetes mellitus	type 1 diabetes mellitus	MONDO_0005147	Immune system disorder	Immunological	PGS001297	MONDO_0005147	type 1 diabetes mellitus	type 1 diabetes mellitus	MONDO_0005147	Digestive system disorder	Gastrointestinal
0.8403	4.69E-123	1.13E-119	PGS001817	MONDO_0005147	type 1 diabetes mellitus	type 1 diabetes mellitus	MONDO_0005147	Metabolic disorder	Metabolic	PGS001297	MONDO_0005147	type 1 diabetes mellitus	type 1 diabetes mellitus	MONDO_0005147	Immune system disorder	Immunological
0.8403	4.69E-123	1.13E-119	PGS001817	MONDO_0005147	type 1 diabetes mellitus	type 1 diabetes mellitus	MONDO_0005147	Digestive system disorder	Gastrointestinal	PGS001297	MONDO_0005147	type 1 diabetes mellitus	type 1 diabetes mellitus	MONDO_0005147	Metabolic disorder	Metabolic
0.8403	4.69E-123	1.13E-119	PGS001817	MONDO_0005147	type 1 diabetes mellitus	type 1 diabetes mellitus	MONDO_0005147	Digestive system disorder	Gastrointestinal	PGS001297	MONDO_0005147	type 1 diabetes mellitus	type 1 diabetes mellitus	MONDO_0005147	Digestive system disorder	Gastrointestinal
0.8403	4.69E-123	1.13E-119	PGS001817	MONDO_0005147	type 1 diabetes mellitus	type 1 diabetes mellitus	MONDO_0005147	Digestive system disorder	Gastrointestinal	PGS001297	MONDO_0005147	type 1 diabetes mellitus	type 1 diabetes mellitus	MONDO_0005147	Immune system disorder	Immunological
0.8403	4.81E-123	1.16E-119	PGS000797	EFO_0002892	thyroid carcinoma	thyroid carcinoma	EFO_0002892	Cancer	Neoplasms	PGS000208	EFO_0002892	thyroid carcinoma	thyroid carcinoma	EFO_0002892	Cancer	Neoplasms
0.8402	4.98E-123	1.20E-119	PGS002764	HP_0003124	Hypercholesterolemia	Hypercholesterolemia	HP_0003124	Other trait	Metabolic	PGS003143	EFO_0004574	total cholesterol measurement	total cholesterol measurement	EFO_0004574	Lipid or lipoprotein measurement	Metabolic
0.84	7.13E-123	1.72E-119	PGS001529	EFO_0007772	calcaneal bone quantitative ultrasound measurement	calcaneal bone quantitative ultrasound measurement	EFO_0007772	Other measurement	Skeletal	PGS001957	EFO_0005654	velocity of sound measurement	velocity of sound measurement	EFO_0005654	Other measurement	Others
-0.8399	7.53E-123	1.82E-119	PGS000884	EFO_0005208	glomerular filtration rate	glomerular filtration rate	EFO_0005208	Other measurement	Genitourinary	PGS000883	EFO_0005208	glomerular filtration rate	glomerular filtration rate	EFO_0005208	Other measurement	Genitourinary
0.8398	8.42E-123	2.03E-119	PGS002998	EFO_0000537	hypertension	hypertension	EFO_0000537	Cardiovascular disease	Cardiovascular	PGS002316	EFO_0000319	cardiovascular disease	cardiovascular disease	EFO_0000319	Cardiovascular disease	Cardiovascular
0.8398	9.08E-123	2.19E-119	PGS000214	MONDO_0021115	luminal B breast carcinoma	luminal B breast carcinoma	MONDO_0021115	Cancer	Neoplasms	PGS000345	EFO_0000305	breast carcinoma	breast carcinoma	EFO_0000305	Cancer	Neoplasms
0.8397	9.70E-123	2.34E-119	PGS002445	EFO_0001379	endocrine system disease|diabetes mellitus	endocrine system disease	EFO_0001379	Other disease	Metabolic	PGS002449	EFO_0004468	glucose measurement	glucose measurement	EFO_0004468	Other measurement	Metabolic
0.8397	1.04E-122	2.51E-119	PGS000797	EFO_0002892	thyroid carcinoma	thyroid carcinoma	EFO_0002892	Cancer	Neoplasms	PGS000209	EFO_0002892	thyroid carcinoma	thyroid carcinoma	EFO_0002892	Cancer	Neoplasms
0.8396	1.08E-122	2.59E-119	PGS000499	EFO_0000305	breast carcinoma	breast carcinoma	EFO_0000305	Cancer	Neoplasms	PGS000504	EFO_0000305	breast carcinoma	breast carcinoma	EFO_0000305	Cancer	Neoplasms
0.8396	1.15E-122	2.77E-119	PGS000931	MONDO_0000831	thrombotic disease|deep vein thrombosis	thrombotic disease	MONDO_0000831	Cardiovascular disease	Cardiovascular	PGS001266	EFO_0003907	deep vein thrombosis	deep vein thrombosis	EFO_0003907	Other trait	Cardiovascular
0.8394	1.43E-122	3.42E-119	PGS003036	EFO_0004611	low density lipoprotein cholesterol measurement	low density lipoprotein cholesterol measurement	EFO_0004611	Cardiovascular measurement	Cardiovascular	PGS003136	EFO_0004574	total cholesterol measurement	total cholesterol measurement	EFO_0004574	Lipid or lipoprotein measurement	Metabolic
0.8394	1.43E-122	3.42E-119	PGS003036	EFO_0004611	low density lipoprotein cholesterol measurement	low density lipoprotein cholesterol measurement	EFO_0004611	Lipid or lipoprotein measurement	Metabolic	PGS003136	EFO_0004574	total cholesterol measurement	total cholesterol measurement	EFO_0004574	Lipid or lipoprotein measurement	Metabolic
0.8394	1.53E-122	3.68E-119	PGS001162	EFO_0005093	hip circumference	hip circumference	EFO_0005093	Body measurement	Body measurement	PGS001148	EFO_0005409	fat body mass	fat body mass	EFO_0005409	Other measurement	Body measurement
0.8393	1.69E-122	4.05E-119	PGS000913	EFO_0006335	systolic blood pressure	systolic blood pressure	EFO_0006335	Other measurement	Cardiovascular	PGS002228	EFO_0006335	systolic blood pressure	systolic blood pressure	EFO_0006335	Other measurement	Cardiovascular
0.8391	2.29E-122	5.49E-119	PGS002403	EFO_0004541	HbA1c measurement	HbA1c measurement	EFO_0004541	Hematological measurement	Hematological	PGS002449	EFO_0004468	glucose measurement	glucose measurement	EFO_0004468	Other measurement	Metabolic
0.8389	2.80E-122	6.72E-119	PGS002228	EFO_0006335	systolic blood pressure	systolic blood pressure	EFO_0006335	Other measurement	Cardiovascular	PGS003073	EFO_0006335	systolic blood pressure	systolic blood pressure	EFO_0006335	Other measurement	Cardiovascular
0.8388	3.13E-122	7.51E-119	PGS000408	EFO_0000756	melanoma	melanoma	EFO_0000756	Cancer	Neoplasms	PGS000410	EFO_0000756	melanoma	melanoma	EFO_0000756	Cancer	Neoplasms
0.8388	3.14E-122	7.52E-119	PGS001932	EFO_0004698	insomnia	insomnia	EFO_0004698	Neurological disorder	Neurological	PGS002149	EFO_0004698	insomnia	insomnia	EFO_0004698	Neurological disorder	Neurological
-0.8386	3.96E-122	9.48E-119	PGS001937	EFO_0004279	suntan	suntan	EFO_0004279	Other trait	Dermatological	PGS002110	EFO_0003784	skin pigmentation	skin pigmentation	EFO_0003784	Other trait	Dermatological
0.8384	5.08E-122	1.22E-118	PGS001946	EFO_0004458	C-reactive protein measurement	C-reactive protein measurement	EFO_0004458	Inflammatory measurement	Immunological	PGS002868	EFO_0004458	C-reactive protein measurement	C-reactive protein measurement	EFO_0004458	Inflammatory measurement	Immunological
0.8384	5.17E-122	1.24E-118	PGS002964	EFO_0004339	body height	body height	EFO_0004339	Body measurement	Body measurement	PGS002973	EFO_0004339	body height	body height	EFO_0004339	Body measurement	Body measurement
0.8384	5.58E-122	1.33E-118	PGS003063	EFO_0006527	smoking status measurement	smoking status measurement	EFO_0006527	Other measurement	Psychiatric	PGS003059	EFO_0006527	smoking status measurement	smoking status measurement	EFO_0006527	Other measurement	Psychiatric
0.8383	6.19E-122	1.48E-118	PGS001157	EFO_0005106	body composition measurement	body composition measurement	EFO_0005106	Body measurement	Body measurement	PGS001161	EFO_0005106	body composition measurement	body composition measurement	EFO_0005106	Body measurement	Body measurement
0.8381	7.52E-122	1.80E-118	PGS000797	EFO_0002892	thyroid carcinoma	thyroid carcinoma	EFO_0002892	Cancer	Neoplasms	PGS000630	EFO_0002892	thyroid carcinoma	thyroid carcinoma	EFO_0002892	Cancer	Neoplasms
0.8381	7.52E-122	1.80E-118	PGS000797	EFO_0002892	thyroid carcinoma	thyroid carcinoma	EFO_0002892	Cancer	Neoplasms	PGS000631	EFO_0002892	thyroid carcinoma	thyroid carcinoma	EFO_0002892	Cancer	Neoplasms
0.8381	7.91E-122	1.89E-118	PGS000216	EFO_0005537	triple-negative breast cancer	triple-negative breast cancer	EFO_0005537	Cancer	Neoplasms	PGS000346	EFO_1000650	estrogen-receptor negative breast cancer	cancer	EFO_1000650	Cancer	Neoplasms
0.8381	8.29E-122	1.98E-118	PGS002459	EFO_0004587	lymphocyte count	lymphocyte count	EFO_0004587	Hematological measurement	Hematological	PGS002606	EFO_0004587	lymphocyte count	lymphocyte count	EFO_0004587	Hematological measurement	Hematological
0.838	9.11E-122	2.17E-118	PGS002968	EFO_0004339	body height	body height	EFO_0004339	Body measurement	Body measurement	PGS002969	EFO_0004339	body height	body height	EFO_0004339	Body measurement	Body measurement
0.838	9.28E-122	2.21E-118	PGS002441	EFO_0000701	skin disease	skin disease	EFO_0000701	Other disease	Dermatological	PGS002490	EFO_0000701	skin disease	skin disease	EFO_0000701	Other disease	Dermatological
0.8379	9.67E-122	2.30E-118	PGS002134	EFO_0004314	forced expiratory volume	forced expiratory volume	EFO_0004314	Other measurement	Respiratory	PGS001922	EFO_0004312	vital capacity	vital capacity	EFO_0004312	Other measurement	Activities
0.8378	1.16E-121	2.75E-118	PGS002846	EFO_0004340	body mass index	body mass index	EFO_0004340	Body measurement	Body measurement	PGS002857	EFO_0004340	body mass index	body mass index	EFO_0004340	Body measurement	Body measurement
0.8377	1.30E-121	3.09E-118	PGS003135	EFO_0004574	total cholesterol measurement	total cholesterol measurement	EFO_0004574	Lipid or lipoprotein measurement	Metabolic	PGS003030	EFO_0004611	low density lipoprotein cholesterol measurement	measurement	EFO_0004611	Cardiovascular measurement	Cardiovascular
0.8377	1.30E-121	3.09E-118	PGS003135	EFO_0004574	total cholesterol measurement	total cholesterol measurement	EFO_0004574	Lipid or lipoprotein measurement	Metabolic	PGS003030	EFO_0004611	low density lipoprotein cholesterol measurement	measurement	EFO_0004611	Lipid or lipoprotein measurement	Metabolic
0.8377	1.34E-121	3.17E-118	PGS002894	EFO_0006336	diastolic blood pressure	diastolic blood pressure	EFO_0006336	Other measurement	Cardiovascular	PGS002898	EFO_0006336	diastolic blood pressure	diastolic blood pressure	EFO_0006336	Other measurement	Cardiovascular
0.8376	1.45E-121	3.44E-118	PGS000511	EFO_0000305	breast carcinoma	breast carcinoma	EFO_0000305	Cancer	Neoplasms	PGS000335	EFO_0000305	breast carcinoma	breast carcinoma	EFO_0000305	Cancer	Neoplasms
0.8376	1.52E-121	3.62E-118	PGS000344	EFO_0000305	breast carcinoma	breast carcinoma	EFO_0000305	Cancer	Neoplasms	PGS000213	EFO_0009780	HER2 negative breast carcinoma	HER2 negative breast carcinoma	EFO_0009780	Cancer	Neoplasms
0.8374	2.05E-121	4.87E-118	PGS000921	EFO_0004340	body mass index	body mass index	EFO_0004340	Body measurement	Body measurement	PGS002313	EFO_0004340	body mass index	body mass index	EFO_0004340	Body measurement	Body measurement
0.8373	2.07E-121	4.91E-118	PGS001079	EFO_0007984	platelet component distribution width	platelet component distribution width	EFO_0007984	Hematological measurement	Hematological	PGS002190	EFO_0007984	platelet component distribution width	platelet component distribution width	EFO_0007984	Hematological measurement	Hematological
0.8373	2.32E-121	5.50E-118	PGS001958	EFO_0005093	hip circumference	hip circumference	EFO_0005093	Body measurement	Body measurement	PGS001951	EFO_0005409	fat body mass	fat body mass	EFO_0005409	Other measurement	Body measurement
0.8372	2.46E-121	5.84E-118	PGS000511	EFO_0000305	breast carcinoma	breast carcinoma	EFO_0000305	Cancer	Neoplasms	PGS000504	EFO_0000305	breast carcinoma	breast carcinoma	EFO_0000305	Cancer	Neoplasms
0.8371	2.77E-121	6.55E-118	PGS002025	MONDO_0005147	type 1 diabetes mellitus	type 1 diabetes mellitus	MONDO_0005147	Digestive system disorder	Gastrointestinal	PGS001296	MONDO_0005147	type 1 diabetes mellitus	type 1 diabetes mellitus	MONDO_0005147	Metabolic disorder	Metabolic
0.8371	2.77E-121	6.55E-118	PGS002025	MONDO_0005147	type 1 diabetes mellitus	type 1 diabetes mellitus	MONDO_0005147	Metabolic disorder	Metabolic	PGS001296	MONDO_0005147	type 1 diabetes mellitus	type 1 diabetes mellitus	MONDO_0005147	Metabolic disorder	Metabolic
0.8371	2.77E-121	6.55E-118	PGS002025	MONDO_0005147	type 1 diabetes mellitus	type 1 diabetes mellitus	MONDO_0005147	Immune system disorder	Immunological	PGS001296	MONDO_0005147	type 1 diabetes mellitus	type 1 diabetes mellitus	MONDO_0005147	Digestive system disorder	Gastrointestinal
0.8371	2.77E-121	6.55E-118	PGS002025	MONDO_0005147	type 1 diabetes mellitus	type 1 diabetes mellitus	MONDO_0005147	Digestive system disorder	Gastrointestinal	PGS001296	MONDO_0005147	type 1 diabetes mellitus	type 1 diabetes mellitus	MONDO_0005147	Digestive system disorder	Gastrointestinal
0.8371	2.77E-121	6.55E-118	PGS002025	MONDO_0005147	type 1 diabetes mellitus	type 1 diabetes mellitus	MONDO_0005147	Immune system disorder	Immunological	PGS001296	MONDO_0005147	type 1 diabetes mellitus	type 1 diabetes mellitus	MONDO_0005147	Immune system disorder	Immunological
0.8371	2.77E-121	6.55E-118	PGS002025	MONDO_0005147	type 1 diabetes mellitus	type 1 diabetes mellitus	MONDO_0005147	Immune system disorder	Immunological	PGS001296	MONDO_0005147	type 1 diabetes mellitus	type 1 diabetes mellitus	MONDO_0005147	Metabolic disorder	Metabolic
0.8371	2.77E-121	6.55E-118	PGS002025	MONDO_0005147	type 1 diabetes mellitus	type 1 diabetes mellitus	MONDO_0005147	Metabolic disorder	Metabolic	PGS001296	MONDO_0005147	type 1 diabetes mellitus	type 1 diabetes mellitus	MONDO_0005147	Immune system disorder	Immunological
0.8371	2.77E-121	6.55E-118	PGS002025	MONDO_0005147	type 1 diabetes mellitus	type 1 diabetes mellitus	MONDO_0005147	Metabolic disorder	Metabolic	PGS001296	MONDO_0005147	type 1 diabetes mellitus	type 1 diabetes mellitus	MONDO_0005147	Digestive system disorder	Gastrointestinal
0.8371	2.77E-121	6.55E-118	PGS002025	MONDO_0005147	type 1 diabetes mellitus	type 1 diabetes mellitus	MONDO_0005147	Digestive system disorder	Gastrointestinal	PGS001296	MONDO_0005147	type 1 diabetes mellitus	type 1 diabetes mellitus	MONDO_0005147	Immune system disorder	Immunological
0.8368	3.99E-121	9.46E-118	PGS003078	EFO_0006335	systolic blood pressure	systolic blood pressure	EFO_0006335	Other measurement	Cardiovascular	PGS002228	EFO_0006335	systolic blood pressure	systolic blood pressure	EFO_0006335	Other measurement	Cardiovascular
0.8367	4.75E-121	1.13E-117	PGS000847	EFO_0004530	triglyceride measurement	triglyceride measurement	EFO_0004530	Lipid or lipoprotein measurement	Metabolic	PGS002621	EFO_0004530	triglyceride measurement	triglyceride measurement	EFO_0004530	Lipid or lipoprotein measurement	Metabolic
0.8366	5.18E-121	1.22E-117	PGS000001	EFO_0000305	breast carcinoma	breast carcinoma	EFO_0000305	Cancer	Neoplasms	PGS000045	EFO_0000305	breast carcinoma	breast carcinoma	EFO_0000305	Cancer	Neoplasms
0.8366	5.65E-121	1.33E-117	PGS002964	EFO_0004339	body height	body height	EFO_0004339	Body measurement	Body measurement	PGS002968	EFO_0004339	body height	body height	EFO_0004339	Body measurement	Body measurement
0.8366	5.68E-121	1.34E-117	PGS003086	EFO_0006335	systolic blood pressure	systolic blood pressure	EFO_0006335	Other measurement	Cardiovascular	PGS003081	EFO_0006335	systolic blood pressure	systolic blood pressure	EFO_0006335	Other measurement	Cardiovascular
0.8363	7.42E-121	1.75E-117	PGS002721	EFO_0004631	vitamin D measurement	vitamin D measurement	EFO_0004631	Lipid or lipoprotein measurement	Metabolic	PGS000702	EFO_0004631	vitamin D measurement	vitamin D measurement	EFO_0004631	Lipid or lipoprotein measurement	Metabolic
0.8362	8.60E-121	2.03E-117	PGS001366	EFO_0004731	eye measurement	eye measurement	EFO_0004731	Other measurement	Ophthalmological	PGS001362	EFO_0004731	eye measurement	eye measurement	EFO_0004731	Other measurement	Ophthalmological
0.8361	1.00E-120	2.36E-117	PGS000002	EFO_1000649	estrogen-receptor positive breast cancer	estrogen-receptor positive breast cancer	EFO_1000649	Cancer	Neoplasms	PGS000046	EFO_1000649	estrogen-receptor positive breast cancer	cancer	EFO_1000649	Cancer	Neoplasms
0.8361	1.06E-120	2.51E-117	PGS000662	EFO_0001663	prostate carcinoma	prostate carcinoma	EFO_0001663	Cancer	Neoplasms	PGS002796	EFO_0001663	prostate carcinoma	prostate carcinoma	EFO_0001663	Cancer	Neoplasms
-0.836	1.10E-120	2.58E-117	PGS002752	EFO_0007878	alcohol consumption measurement	alcohol consumption measurement	EFO_0007878	Other measurement	Psychiatric	PGS003376	EFO_0007878	alcohol consumption measurement	alcohol consumption measurement	EFO_0007878	Other measurement	Psychiatric
0.836	1.15E-120	2.72E-117	PGS000463	EFO_0000707	squamous cell carcinoma	squamous cell carcinoma	EFO_0000707	Cancer	Neoplasms	PGS000468	EFO_0000707	squamous cell carcinoma	squamous cell carcinoma	EFO_0000707	Cancer	Neoplasms
0.8359	1.37E-120	3.22E-117	PGS001147	EFO_0005409	fat body mass	fat body mass	EFO_0005409	Other measurement	Body measurement	PGS001162	EFO_0005093	hip circumference	hip circumference	EFO_0005093	Body measurement	Body measurement
0.8357	1.57E-120	3.70E-117	PGS003392	EFO_0000708	squamous cell lung carcinoma	squamous cell lung carcinoma	EFO_0000708	Cancer	Neoplasms	PGS003391	EFO_0001071	lung carcinoma	lung carcinoma	EFO_0001071	Cancer	Neoplasms
0.8357	1.65E-120	3.89E-117	PGS000702	EFO_0004631	vitamin D measurement	vitamin D measurement	EFO_0004631	Lipid or lipoprotein measurement	Metabolic	PGS002355	EFO_0004631	vitamin D measurement	vitamin D measurement	EFO_0004631	Lipid or lipoprotein measurement	Metabolic
0.8357	1.73E-120	4.08E-117	PGS000579	EFO_0001663	prostate carcinoma	prostate carcinoma	EFO_0001663	Cancer	Neoplasms	PGS000795	EFO_0001663	prostate carcinoma	prostate carcinoma	EFO_0001663	Cancer	Neoplasms
0.8356	1.90E-120	4.48E-117	PGS000955	EFO_0004514	bone quantitative ultrasound measurement	bone quantitative ultrasound measurement	EFO_0004514	Other measurement	Skeletal	PGS000954	EFO_0004514	bone quantitative ultrasound measurement	measurement	EFO_0004514	Other measurement	Skeletal
0.8356	1.93E-120	4.54E-117	PGS000955	EFO_0004514	bone quantitative ultrasound measurement	bone quantitative ultrasound measurement	EFO_0004514	Other measurement	Skeletal	PGS001038	EFO_0009270	heel bone mineral density	heel bone mineral density	EFO_0009270	Other measurement	Skeletal
0.8356	1.95E-120	4.57E-117	PGS000952	EFO_0004514	bone quantitative ultrasound measurement	bone quantitative ultrasound measurement	EFO_0004514	Other measurement	Skeletal	PGS001957	EFO_0005654	velocity of sound measurement	velocity of sound measurement	EFO_0005654	Other measurement	Others
0.8356	2.00E-120	4.70E-117	PGS002353	EFO_0004530	triglyceride measurement	triglyceride measurement	EFO_0004530	Lipid or lipoprotein measurement	Metabolic	PGS003148	EFO_0004530	triglyceride measurement	triglyceride measurement	EFO_0004530	Lipid or lipoprotein measurement	Metabolic
0.8356	2.01E-120	4.71E-117	PGS001037	EFO_0009270	heel bone mineral density	heel bone mineral density	EFO_0009270	Other measurement	Skeletal	PGS001957	EFO_0005654	velocity of sound measurement	velocity of sound measurement	EFO_0005654	Other measurement	Others
0.8355	2.15E-120	5.04E-117	PGS000504	EFO_0000305	breast carcinoma	breast carcinoma	EFO_0000305	Cancer	Neoplasms	PGS000500	EFO_0000305	breast carcinoma	breast carcinoma	EFO_0000305	Cancer	Neoplasms
-0.8355	2.29E-120	5.35E-117	PGS001532	EFO_0005671	smoking behaviour measurement	smoking behaviour measurement	EFO_0005671	Other measurement	Psychiatric	PGS001515	EFO_0006525	cigarettes per day measurement	cigarettes per day measurement	EFO_0006525	Other measurement	Psychiatric
0.8354	2.41E-120	5.64E-117	PGS000511	EFO_0000305	breast carcinoma	breast carcinoma	EFO_0000305	Cancer	Neoplasms	PGS000507	EFO_0000305	breast carcinoma	breast carcinoma	EFO_0000305	Cancer	Neoplasms
0.8353	2.77E-120	6.48E-117	PGS000579	EFO_0001663	prostate carcinoma	prostate carcinoma	EFO_0001663	Cancer	Neoplasms	PGS002791	EFO_0001663	prostate carcinoma	prostate carcinoma	EFO_0001663	Cancer	Neoplasms
0.8352	3.14E-120	7.36E-117	PGS002150	EFO_0004611	low density lipoprotein cholesterol measurement	low density lipoprotein cholesterol measurement	EFO_0004611	Lipid or lipoprotein measurement	Metabolic	PGS002352	EFO_0004574	total cholesterol measurement	total cholesterol measurement	EFO_0004574	Lipid or lipoprotein measurement	Metabolic
0.8352	3.14E-120	7.36E-117	PGS002150	EFO_0004611	low density lipoprotein cholesterol measurement	low density lipoprotein cholesterol measurement	EFO_0004611	Cardiovascular measurement	Cardiovascular	PGS002352	EFO_0004574	total cholesterol measurement	total cholesterol measurement	EFO_0004574	Lipid or lipoprotein measurement	Metabolic
-0.8351	3.38E-120	7.90E-117	PGS002768	EFO_0003882	osteoporosis	osteoporosis	EFO_0003882	Other disease	Skeletal	PGS001957	EFO_0005654	velocity of sound measurement	velocity of sound measurement	EFO_0005654	Other measurement	Others
0.8351	3.58E-120	8.36E-117	PGS001947	EFO_0004617	cystatin C measurement	cystatin C measurement	EFO_0004617	Other measurement	Metabolic	PGS000680	EFO_0004617	cystatin C measurement	cystatin C measurement	EFO_0004617	Other measurement	Metabolic
0.835	3.82E-120	8.93E-117	PGS000758	EFO_0004339	body height	body height	EFO_0004339	Body measurement	Body measurement	PGS002973	EFO_0004339	body height	body height	EFO_0004339	Body measurement	Body measurement
-0.835	3.83E-120	8.94E-117	PGS002173	EFO_0009270	heel bone mineral density	heel bone mineral density	EFO_0009270	Other measurement	Skeletal	PGS002768	EFO_0003882	osteoporosis	osteoporosis	EFO_0003882	Other disease	Skeletal
0.8349	4.51E-120	1.05E-116	PGS002101	EFO_0004614	apolipoprotein A 1 measurement	apolipoprotein A 1 measurement	EFO_0004614	Inflammatory measurement	Immunological	PGS001954	EFO_0004612	high density lipoprotein cholesterol measurement	measurement	EFO_0004612	Lipid or lipoprotein measurement	Metabolic
0.8349	4.51E-120	1.05E-116	PGS002101	EFO_0004614	apolipoprotein A 1 measurement	apolipoprotein A 1 measurement	EFO_0004614	Cardiovascular measurement	Cardiovascular	PGS001954	EFO_0004612	high density lipoprotein cholesterol measurement	measurement	EFO_0004612	Lipid or lipoprotein measurement	Metabolic
0.8349	4.51E-120	1.05E-116	PGS002101	EFO_0004614	apolipoprotein A 1 measurement	apolipoprotein A 1 measurement	EFO_0004614	Lipid or lipoprotein measurement	Metabolic	PGS001954	EFO_0004612	high density lipoprotein cholesterol measurement	measurement	EFO_0004612	Lipid or lipoprotein measurement	Metabolic
0.8349	4.76E-120	1.11E-116	PGS002840	EFO_0004340	body mass index	body mass index	EFO_0004340	Body measurement	Body measurement	PGS002313	EFO_0004340	body mass index	body mass index	EFO_0004340	Body measurement	Body measurement
0.8348	4.89E-120	1.14E-116	PGS003071	EFO_0006335	systolic blood pressure	systolic blood pressure	EFO_0006335	Other measurement	Cardiovascular	PGS003070	EFO_0006335	systolic blood pressure	systolic blood pressure	EFO_0006335	Other measurement	Cardiovascular
0.8348	5.06E-120	1.18E-116	PGS000353	MONDO_0004992	cancer	cancer	MONDO_0004992	Cancer	Neoplasms	PGS000356	MONDO_0004992	cancer	cancer	MONDO_0004992	Cancer	Neoplasms
0.8348	5.19E-120	1.21E-116	PGS000575	EFO_0001663	prostate carcinoma	prostate carcinoma	EFO_0001663	Cancer	Neoplasms	PGS000569	EFO_0001663	prostate carcinoma	prostate carcinoma	EFO_0001663	Cancer	Neoplasms
0.8348	5.41E-120	1.26E-116	PGS001402	EFO_0009270	heel bone mineral density	heel bone mineral density	EFO_0009270	Other measurement	Skeletal	PGS001957	EFO_0005654	velocity of sound measurement	velocity of sound measurement	EFO_0005654	Other measurement	Others
0.8347	5.53E-120	1.29E-116	PGS001264	EFO_0003907	deep vein thrombosis	deep vein thrombosis	EFO_0003907	Other trait	Cardiovascular	PGS000961	EFO_1001395	Phlebitis|Thrombophlebitis	Phlebitis	EFO_1001395	Cardiovascular disease	Cardiovascular
0.8347	5.98E-120	1.39E-116	PGS000588	EFO_0001663	prostate carcinoma	prostate carcinoma	EFO_0001663	Cancer	Neoplasms	PGS002240	EFO_0001663	prostate carcinoma	prostate carcinoma	EFO_0001663	Cancer	Neoplasms
0.8346	7.00E-120	1.62E-116	PGS003399	EFO_0000305	breast carcinoma	breast carcinoma	EFO_0000305	Cancer	Neoplasms	PGS000212	MONDO_0021116	luminal A breast carcinoma	luminal A breast carcinoma	MONDO_0021116	Cancer	Neoplasms
-0.8344	8.04E-120	1.87E-116	PGS003119	MONDO_0005148	type 2 diabetes mellitus	type 2 diabetes mellitus	MONDO_0005148	Metabolic disorder	Metabolic	PGS001357	MONDO_0005148	type 2 diabetes mellitus	type 2 diabetes mellitus	MONDO_0005148	Metabolic disorder	Metabolic

-0.8344	8.04E-120	1.87E-116	PGS003119	MONDO_0005148	type 2 diabetes mellitus	type 2 diabetes mellitus	MONDO_0005148	Metabolic disorder	Metabolic	PGS001357	MONDO_0005148	type 2 diabetes mellitus	type 2 diabetes mellitus	MONDO_0005148	Digestive system disorder	Gastrointestinal
-0.8344	8.04E-120	1.87E-116	PGS003119	MONDO_0005148	type 2 diabetes mellitus	type 2 diabetes mellitus	MONDO_0005148	Digestive system disorder	Gastrointestinal	PGS001357	MONDO_0005148	type 2 diabetes mellitus	type 2 diabetes mellitus	MONDO_0005148	Metabolic disorder	Metabolic
-0.8344	8.04E-120	1.87E-116	PGS003119	MONDO_0005148	type 2 diabetes mellitus	type 2 diabetes mellitus	MONDO_0005148	Digestive system disorder	Gastrointestinal	PGS001357	MONDO_0005148	type 2 diabetes mellitus	type 2 diabetes mellitus	MONDO_0005148	Digestive system disorder	Gastrointestinal
0.8344	8.81E-120	2.04E-116	PGS001227	EFO_0004342	waist circumference	waist circumference	EFO_0004342	Body measurement	Body measurement	PGS001144	EFO_0005409	fat body mass	fat body mass	EFO_0005409	Other measurement	Body measurement
0.8344	8.94E-120	2.07E-116	PGS000317	EFO_0000305	breast carcinoma	breast carcinoma	EFO_0000305	Cancer	Neoplasms	PGS003398	EFO_0000305	breast carcinoma	breast carcinoma	EFO_0000305	Cancer	Neoplasms
0.8344	8.95E-120	2.07E-116	PGS002796	EFO_0001663	prostate carcinoma	prostate carcinoma	EFO_0001663	Cancer	Neoplasms	PGS003415	EFO_0001663	prostate carcinoma	prostate carcinoma	EFO_0001663	Cancer	Neoplasms
0.8344	8.97E-120	2.08E-116	PGS001065	EFO_0008111	diet measurement	diet measurement	EFO_0008111	Other measurement	Activities	PGS001856	EFO_0001060	celiac disease	celiac disease	EFO_0001060	Immune system disorder	Immunological
0.8344	8.97E-120	2.08E-116	PGS001065	EFO_0008111	diet measurement	diet measurement	EFO_0008111	Other measurement	Activities	PGS001856	EFO_0001060	celiac disease	celiac disease	EFO_0001060	Digestive system disorder	Gastrointestinal
0.8344	9.10E-120	2.10E-116	PGS000499	EFO_0000305	breast carcinoma	breast carcinoma	EFO_0000305	Cancer	Neoplasms	PGS000512	EFO_0000305	breast carcinoma	breast carcinoma	EFO_0000305	Cancer	Neoplasms
0.8343	9.51E-120	2.20E-116	PGS002614	EFO_0005192	red blood cell distribution width	red blood cell distribution width	EFO_0005192	Hematological measurement	Hematological	PGS002418	EFO_0005192	red blood cell distribution width	red blood cell distribution width	EFO_0005192	Hematological measurement	Hematological
0.8341	1.20E-119	2.77E-116	PGS000672	EFO_0004615	apolipoprotein B measurement	apolipoprotein B measurement	EFO_0004615	Lipid or lipoprotein measurement	Metabolic	PGS001889	EFO_0004615	apolipoprotein B measurement	apolipoprotein B measurement	EFO_0004615	Lipid or lipoprotein measurement	Metabolic
0.8337	1.96E-119	4.53E-116	PGS002773	EFO_0000275	atrial fibrillation	atrial fibrillation	EFO_0000275	Cardiovascular disease	Cardiovascular	PGS000035	EFO_0000275	atrial fibrillation	atrial fibrillation	EFO_0000275	Cardiovascular disease	Cardiovascular
0.8337	2.12E-119	4.90E-116	PGS002163	EFO_0004518	creatinine measurement	creatinine measurement	EFO_0004518	Other measurement	Metabolic	PGS003243	EFO_0010472	creatine measurement	creatine measurement	EFO_0010472	Other measurement	Metabolic
0.8336	2.45E-119	5.65E-116	PGS001297	MONDO_0005147	type 1 diabetes mellitus	type 1 diabetes mellitus	MONDO_0005147	Metabolic disorder	Metabolic	PGS002025	MONDO_0005147	type 1 diabetes mellitus	type 1 diabetes mellitus	MONDO_0005147	Metabolic disorder	Metabolic
0.8336	2.45E-119	5.65E-116	PGS001297	MONDO_0005147	type 1 diabetes mellitus	type 1 diabetes mellitus	MONDO_0005147	Digestive system disorder	Gastrointestinal	PGS002025	MONDO_0005147	type 1 diabetes mellitus	type 1 diabetes mellitus	MONDO_0005147	Metabolic disorder	Metabolic
0.8336	2.45E-119	5.65E-116	PGS001297	MONDO_0005147	type 1 diabetes mellitus	type 1 diabetes mellitus	MONDO_0005147	Metabolic disorder	Metabolic	PGS002025	MONDO_0005147	type 1 diabetes mellitus	type 1 diabetes mellitus	MONDO_0005147	Digestive system disorder	Gastrointestinal
0.8336	2.45E-119	5.65E-116	PGS001297	MONDO_0005147	type 1 diabetes mellitus	type 1 diabetes mellitus	MONDO_0005147	Immune system disorder	Immunological	PGS002025	MONDO_0005147	type 1 diabetes mellitus	type 1 diabetes mellitus	MONDO_0005147	Metabolic disorder	Metabolic
0.8336	2.45E-119	5.65E-116	PGS001297	MONDO_0005147	type 1 diabetes mellitus	type 1 diabetes mellitus	MONDO_0005147	Digestive system disorder	Gastrointestinal	PGS002025	MONDO_0005147	type 1 diabetes mellitus	type 1 diabetes mellitus	MONDO_0005147	Digestive system disorder	Gastrointestinal
0.8336	2.45E-119	5.65E-116	PGS001297	MONDO_0005147	type 1 diabetes mellitus	type 1 diabetes mellitus	MONDO_0005147	Digestive system disorder	Gastrointestinal	PGS002025	MONDO_0005147	type 1 diabetes mellitus	type 1 diabetes mellitus	MONDO_0005147	Immune system disorder	Immunological
0.8336	2.45E-119	5.65E-116	PGS001297	MONDO_0005147	type 1 diabetes mellitus	type 1 diabetes mellitus	MONDO_0005147	Metabolic disorder	Metabolic	PGS002025	MONDO_0005147	type 1 diabetes mellitus	type 1 diabetes mellitus	MONDO_0005147	Immune system disorder	Immunological
0.8336	2.45E-119	5.65E-116	PGS001297	MONDO_0005147	type 1 diabetes mellitus	type 1 diabetes mellitus	MONDO_0005147	Immune system disorder	Immunological	PGS002025	MONDO_0005147	type 1 diabetes mellitus	type 1 diabetes mellitus	MONDO_0005147	Digestive system disorder	Gastrointestinal
0.8336	2.45E-119	5.65E-116	PGS001297	MONDO_0005147	type 1 diabetes mellitus	type 1 diabetes mellitus	MONDO_0005147	Immune system disorder	Immunological	PGS002025	MONDO_0005147	type 1 diabetes mellitus	type 1 diabetes mellitus	MONDO_0005147	Immune system disorder	Immunological
-0.8335	2.47E-119	5.70E-116	PGS001357	MONDO_0005148	type 2 diabetes mellitus	type 2 diabetes mellitus	MONDO_0005148	Metabolic disorder	Metabolic	PGS003104	MONDO_0005148	type 2 diabetes mellitus	type 2 diabetes mellitus	MONDO_0005148	Digestive system disorder	Gastrointestinal
-0.8335	2.47E-119	5.70E-116	PGS001357	MONDO_0005148	type 2 diabetes mellitus	type 2 diabetes mellitus	MONDO_0005148	Digestive system disorder	Gastrointestinal	PGS003104	MONDO_0005148	type 2 diabetes mellitus	type 2 diabetes mellitus	MONDO_0005148	Metabolic disorder	Metabolic
-0.8335	2.47E-119	5.70E-116	PGS001357	MONDO_0005148	type 2 diabetes mellitus	type 2 diabetes mellitus	MONDO_0005148	Metabolic disorder	Metabolic	PGS003104	MONDO_0005148	type 2 diabetes mellitus	type 2 diabetes mellitus	MONDO_0005148	Metabolic disorder	Metabolic
-0.8335	2.47E-119	5.70E-116	PGS001357	MONDO_0005148	type 2 diabetes mellitus	type 2 diabetes mellitus	MONDO_0005148	Digestive system disorder	Gastrointestinal	PGS003104	MONDO_0005148	type 2 diabetes mellitus	type 2 diabetes mellitus	MONDO_0005148	Digestive system disorder	Gastrointestinal
0.8335	2.55E-119	5.87E-116	PGS000476	EFO_0000305	breast carcinoma	breast carcinoma	EFO_0000305	Cancer	Neoplasms	PGS000515	EFO_0000305	breast carcinoma	breast carcinoma	EFO_0000305	Cancer	Neoplasms
0.8335	2.71E-119	6.25E-116	PGS001106	EFO_0007800	body fat percentage	body fat percentage	EFO_0007800	Body measurement	Body measurement	PGS001105	EFO_0007800	body fat percentage	body fat percentage	EFO_0007800	Body measurement	Body measurement
0.8334	3.14E-119	7.24E-116	PGS000758	EFO_0004339	body height	body height	EFO_0004339	Body measurement	Body measurement	PGS002968	EFO_0004339	body height	body height	EFO_0004339	Body measurement	Body measurement
0.8333	3.43E-119	7.90E-116	PGS001725	EFO_0006930	brain volume measurement|neuroimaging measurement	brain volume measurement	EFO_0006930	Other measurement	Neurological	PGS001739	EFO_0006930	measurement	brain volume measurement	EFO_0006930	Other measurement	Neurological
0.8332	3.61E-119	8.31E-116	PGS002393	EFO_0000400	diabetes mellitus	diabetes mellitus	EFO_0000400	Digestive system disorder	Gastrointestinal	PGS002449	EFO_0004468	glucose measurement	glucose measurement	EFO_0004468	Other measurement	Metabolic
0.8332	3.61E-119	8.31E-116	PGS002393	EFO_0000400	diabetes mellitus	diabetes mellitus	EFO_0000400	Metabolic disorder	Metabolic	PGS002449	EFO_0004468	glucose measurement	glucose measurement	EFO_0004468	Other measurement	Metabolic
0.8332	3.95E-119	9.07E-116	PGS000742	EFO_0001663	prostate carcinoma	prostate carcinoma	EFO_0001663	Cancer	Neoplasms	PGS000719	EFO_0001663	prostate carcinoma	prostate carcinoma	EFO_0001663	Cancer	Neoplasms
0.8332	3.98E-119	9.16E-116	PGS002398	EFO_0004713	FEV|FEC ratio	FEV|FEC ratio	EFO_0004713	Other measurement	Respiratory	PGS002447	EFO_0004713	FEV|FEC ratio	FEV|FEC ratio	EFO_0004713	Other measurement	Respiratory
0.8331	4.12E-119	9.47E-116	PGS002603	EFO_0000537	hypertension	hypertension	EFO_0000537	Cardiovascular disease	Cardiovascular	PGS002535	EFO_0000319	cardiovascular disease	cardiovascular disease	EFO_0000319	Cardiovascular disease	Cardiovascular
0.8331	4.31E-119	9.89E-116	PGS003399	EFO_0000305	breast carcinoma	breast carcinoma	EFO_0000305	Cancer	Neoplasms	PGS000214	MONDO_0021115	luminal B breast carcinoma	luminal B breast carcinoma	MONDO_0021115	Cancer	Neoplasms
0.833	4.72E-119	1.08E-115	PGS001366	EFO_0004731	eye measurement	eye measurement	EFO_0004731	Other measurement	Ophthalmological	PGS001367	EFO_0004731	eye measurement	eye measurement	EFO_0004731	Other measurement	Ophthalmological
0.833	5.09E-119	1.17E-115	PGS000933	HP_0100582	Nasal polyposis	Nasal polyposis	HP_0100582	Other trait	Ear, Nose, Throat	PGS000934	HP_0100582	Nasal polyposis	Nasal polyposis	HP_0100582	Other trait	Ear, Nose, Throat
0.8329	5.71E-119	1.31E-115	PGS001301	EFO_0001060	celiac disease	celiac disease	EFO_0001060	Immune system disorder	Immunological	PGS001894	EFO_0001060	celiac disease	celiac disease	EFO_0001060	Immune system disorder	Immunological
0.8329	5.71E-119	1.31E-115	PGS001301	EFO_0001060	celiac disease	celiac disease	EFO_0001060	Immune system disorder	Immunological	PGS001894	EFO_0001060	celiac disease	celiac disease	EFO_0001060	Digestive system disorder	Gastrointestinal
0.8329	5.71E-119	1.31E-115	PGS001301	EFO_0001060	celiac disease	celiac disease	EFO_0001060	Digestive system disorder	Gastrointestinal	PGS001894	EFO_0001060	celiac disease	celiac disease	EFO_0001060	Immune system disorder	Immunological
0.8329	5.71E-119	1.31E-115	PGS001301	EFO_0001060	celiac disease	celiac disease	EFO_0001060	Digestive system disorder	Gastrointestinal	PGS001894	EFO_0001060	celiac disease	celiac disease	EFO_0001060	Digestive system disorder	Gastrointestinal
0.8329	5.77E-119	1.32E-115	PGS002894	EFO_0006336	diastolic blood pressure	diastolic blood pressure	EFO_0006336	Other measurement	Cardiovascular	PGS000912	EFO_0006336	diastolic blood pressure	diastolic blood pressure	EFO_0006336	Other measurement	Cardiovascular
0.8328	5.89E-119	1.35E-115	PGS000090	EFO_0004842	eosinophil count	eosinophil count	EFO_0004842	Hematological measurement	Hematological	PGS000091	EFO_0007991	eosinophil percentage of leukocytes	eosinophil percentage of leukocytes	EFO_0007991	Hematological measurement	Hematological
0.8328	5.93E-119	1.36E-115	PGS000694	EFO_0004696	sex hormone-binding globulin measurement	sex hormone-binding globulin measurement	EFO_0004696	Other measurement	Metabolic	PGS001977	EFO_0004696	sex hormone-binding globulin measurement	measurement	EFO_0004696	Other measurement	Metabolic
0.8327	6.96E-119	1.59E-115	PGS002889	EFO_0006336	diastolic blood pressure	diastolic blood pressure	EFO_0006336	Other measurement	Cardiovascular	PGS002893	EFO_0006336	diastolic blood pressure	diastolic blood pressure	EFO_0006336	Other measurement	Cardiovascular
0.8327	7.48E-119	1.71E-115	PGS003149	EFO_0004530	triglyceride measurement	triglyceride measurement	EFO_0004530	Lipid or lipoprotein measurement	Metabolic	PGS003148	EFO_0004530	triglyceride measurement	triglyceride measurement	EFO_0004530	Lipid or lipoprotein measurement	Metabolic
0.8326	7.79E-119	1.78E-115	PGS001117	EFO_0600082	insulin use measurement	insulin use measurement	EFO_0600082	Other measurement	Metabolic	PGS002028	HP_0001943	Hypoglycemia	Hypoglycemia	HP_0001943	Other trait	Metabolic
0.8324	9.70E-119	2.22E-115	PGS001674	EFO_0006930	brain volume measurement|neuroimaging measurement	brain volume measurement	EFO_0006930	Other measurement	Neurological	PGS001480	EFO_0006930	measurement	brain volume measurement	EFO_0006930	Other measurement	Neurological
0.8324	1.01E-118	2.31E-115	PGS002500	EFO_0007822	hair colour measurement	hair colour measurement	EFO_0007822	Other measurement	Dermatological	PGS002402	EFO_0007822	hair colour measurement	hair colour measurement	EFO_0007822	Other measurement	Dermatological
0.8324	1.01E-118	2.31E-115	PGS002774	EFO_0000275	atrial fibrillation	atrial fibrillation	EFO_0000275	Cardiovascular disease	Cardiovascular	PGS002773	EFO_0000275	atrial fibrillation	atrial fibrillation	EFO_0000275	Cardiovascular disease	Cardiovascular
-0.8324	1.03E-118	2.35E-115	PGS002768	EFO_0003882	osteoporosis	osteoporosis	EFO_0003882	Other disease	Skeletal	PGS002175	EFO_0005654	velocity of sound measurement	velocity of sound measurement	EFO_0005654	Other measurement	Others
0.8322	1.27E-118	2.90E-115	PGS000831	EFO_0004574	total cholesterol measurement	total cholesterol measurement	EFO_0004574	Lipid or lipoprotein measurement	Metabolic	PGS000824	EFO_0004611	low density lipoprotein cholesterol measurement	measurement	EFO_0004611	Cardiovascular measurement	Cardiovascular
0.8322	1.27E-118	2.90E-115	PGS000831	EFO_0004574	total cholesterol measurement	total cholesterol measurement	EFO_0004574	Lipid or lipoprotein measurement	Metabolic	PGS000824	EFO_0004611	low density lipoprotein cholesterol measurement	measurement	EFO_0004611	Lipid or lipoprotein measurement	Metabolic
0.8322	1.30E-118	2.98E-115	PGS001301	EFO_0001060	celiac disease	celiac disease	EFO_0001060	Digestive system disorder	Gastrointestinal	PGS002067	EFO_0001060	celiac disease	celiac disease	EFO_0001060	Digestive system disorder	Gastrointestinal
0.8322	1.30E-118	2.98E-115	PGS001301	EFO_0001060	celiac disease	celiac disease	EFO_0001060	Digestive system disorder	Gastrointestinal	PGS002067	EFO_0001060	celiac disease	celiac disease	EFO_0001060	Immune system disorder	Immunological
0.8322	1.30E-118	2.98E-115	PGS001301	EFO_0001060	celiac disease	celiac disease	EFO_0001060	Immune system disorder	Immunological	PGS002067	EFO_0001060	celiac disease	celiac disease	EFO_0001060	Digestive system disorder	Gastrointestinal
0.8322	1.30E-118	2.98E-115	PGS001301	EFO_0001060	celiac disease	celiac disease	EFO_0001060	Immune system disorder	Immunological	PGS002067	EFO_0001060	celiac disease	celiac disease	EFO_0001060	Immune system disorder	Immunological
0.8321	1.53E-118	3.48E-115	PGS002891	EFO_0006336	diastolic blood pressure	diastolic blood pressure	EFO_0006336	Other measurement	Cardiovascular	PGS002890	EFO_0006336	diastolic blood pressure	diastolic blood pressure	EFO_0006336	Other measurement	Cardiovascular
0.8321	1.54E-118	3.50E-115	PGS002764	HP_0003124	Hypercholesterolemia	Hypercholesterolemia	HP_0003124	Other trait	Metabolic	PGS003138	EFO_0004574	total cholesterol measurement	total cholesterol measurement	EFO_0004574	Lipid or lipoprotein measurement	Metabolic
0.8321	1.58E-118	3.60E-115	PGS002960	EFO_0004612	high density lipoprotein cholesterol measurement	high density lipoprotein cholesterol measurement	EFO_0004612	Lipid or lipoprotein measurement	Metabolic	PGS002956	EFO_0004612	high density lipoprotein cholesterol measurement	measurement	EFO_0004612	Lipid or lipoprotein measurement	Metabolic
0.832	1.72E-118	3.91E-115	PGS001895	EFO_0004574	total cholesterol measurement	total cholesterol measurement	EFO_0004574	Lipid or lipoprotein measurement	Metabolic	PGS001889	EFO_0004615	apolipoprotein B measurement	apolipoprotein B measurement	EFO_0004615	Lipid or lipoprotein measurement	Metabolic
0.8319	1.81E-118	4.13E-115	PGS002764	HP_0003124	Hypercholesterolemia	Hypercholesterolemia	HP_0003124	Other trait	Metabolic	PGS000888	EFO_0004611	low density lipoprotein cholesterol measurement	measurement	EFO_0004611	Lipid or lipoprotein measurement	Metabolic
0.8319	1.81E-118	4.13E-115	PGS002764	HP_0003124	Hypercholesterolemia	Hypercholesterolemia	HP_0003124	Other trait	Metabolic	PGS000888	EFO_0004611	low density lipoprotein cholesterol measurement	measurement	EFO_0004611	Cardiovascular measurement	Cardiovascular
0.8318	2.08E-118	4.74E-115	PGS001951	EFO_0005409	fat body mass	fat body mass	EFO_0005409	Other measurement	Body measurement	PGS002201	EFO_0004342	waist circumference	waist circumference	EFO_0004342	Body measurement	Body measurement
0.8318	2.09E-118	4.76E-115	PGS002343	EFO_0004309	platelet count	platelet count	EFO_0004309	Hematological measurement	Hematological	PGS001238	EFO_0004309	platelet count	platelet count	EFO_0004309	Hematological measurement	Hematological
0.8318	2.15E-118	4.89E-115	PGS002426	MONDO_0005148	type 2 diabetes mellitus	type 2 diabetes mellitus	MONDO_0005148	Digestive system disorder	Gastrointestinal	PGS002449	EFO_0004468	glucose measurement	glucose measurement	EFO_0004468	Other measurement	Metabolic
0.8318	2.15E-118	4.89E-115	PGS002426	MONDO_0005148	type 2 diabetes mellitus	type 2 diabetes mellitus	MONDO_0005148	Metabolic disorder	Metabolic	PGS002449	EFO_0004468	glucose measurement	glucose measurement	EFO_0004468	Other measurement	Metabolic
0.8318	2.18E-118	4.94E-115	PGS000575	EFO_0001663	prostate carcinoma	prostate carcinoma	EFO_0001663	Cancer	Neoplasms	PGS000593	EFO_0007355	male reproductive organ cancer	male reproductive organ cancer	EFO_0007355	Cancer	Neoplasms
0.8317	2.31E-118	5.25E-115	PGS001964	EFO_0004532	serum gamma-glutamyl transferase measurement	serum gamma-glutamyl transferase measurement	EFO_0004532	Liver enzyme measurement	Gastrointestinal	PGS000683	EFO_0004532	serum gamma-glutamyl transferase measurement	measurement	EFO_0004532	Liver enzyme measurement	Gastrointestinal
0.8317	2.41E-118	5.47E-115	PGS002554	EFO_0000537	hypertension	hypertension	EFO_0000537	Cardiovascular disease	Cardiovascular	PGS002535	EFO_0000319	cardiovascular disease	cardiovascular disease	EFO_0000319	Cardiovascular disease	Cardiovascular
0.8316	2.87E-118	6.50E-115	PGS002513	EFO_0004309	platelet count	platelet count	EFO_0004309	Hematological measurement	Hematological	PGS002464	EFO_0004309	platelet count	platelet count	EFO_0004309	Hematological measurement	Hematological
0.8315	2.95E-118	6.68E-115	PGS003261	EFO_0010472	creatine measurement|glomerular filtration rate	creatine measurement	EFO_0010472	Other measurement	Metabolic	PGS003276	EFO_0010472	creatine measurement|glomerular filtration rate	creatine measurement	EFO_0010472	Other measurement	Metabolic
0.8315	2.95E-118	6.68E-115	PGS003261	EFO_0010472	creatine measurement|glomerular filtration rate	creatine measurement	EFO_0010472	Other measurement	Metabolic	PGS003275	EFO_0010472	creatine measurement|glomerular filtration rate	creatine measurement	EFO_0010472	Other measurement	Metabolic
0.8315	2.95E-118	6.68E-115	PGS003260	EFO_0010472	creatine measurement|glomerular filtration rate	creatine measurement	EFO_0010472	Other measurement	Metabolic	PGS003275	EFO_0010472	creatine measurement|glomerular filtration rate	creatine measurement	EFO_0010472	Other measurement	Metabolic
0.8315	2.95E-118	6.68E-115	PGS003260	EFO_0010472	creatine measurement|glomerular filtration rate	creatine measurement	EFO_0010472	Other measurement	Metabolic	PGS003276	EFO_0010472	creatine measurement|glomerular filtration rate	creatine measurement	EFO_0010472	Other measurement	Metabolic
0.8315	3.30E-118	7.47E-115	PGS000930	MONDO_0000831	thrombotic disease	thrombotic disease	MONDO_0000831	Cardiovascular disease	Cardiovascular	PGS000931	MONDO_0000831	thrombotic disease|deep vein thrombosis	thrombotic disease	MONDO_0000831	Cardiovascular disease	Cardiovascular
0.8314	3.59E-118	8.12E-115	PGS002475	MONDO_0005148	type 2 diabetes mellitus	type 2 diabetes mellitus	MONDO_0005148	Digestive system disorder	Gastrointestinal	PGS002449	EFO_0004468	glucose measurement	glucose measurement	EFO_0004468	Other measurement	Metabolic
0.8314	3.59E-118	8.12E-115	PGS002475	MONDO_0005148	type 2 diabetes mellitus	type 2 diabetes mellitus	MONDO_0005148	Metabolic disorder	Metabolic	PGS002449	EFO_0004468	glucose measurement	glucose measurement	EFO_0004468	Other measurement	Metabolic
0.8313	4.07E-118	9.20E-115	PGS001684	EFO_0006930	brain volume measurement|neuroimaging measurement	brain volume measurement	EFO_0006930	Other measurement	Neurological	PGS001685	EFO_0006930	measurement	brain volume measurement	EFO_0006930	Other measurement	Neurological
0.8312	4.40E-118	9.94E-115	PGS003056	EFO_0006527	smoking status measurement	smoking status measurement	EFO_0006527	Other measurement	Psychiatric	PGS003055	EFO_0006527	smoking status measurement	smoking status measurement	EFO_0006527	Other measurement	Psychiatric
0.8311	4.99E-118	1.13E-114	PGS003050	EFO_0006527	smoking status measurement	smoking status measurement	EFO_0006527	Other measurement	Psychiatric	PGS003051	EFO_0006527	smoking status measurement	smoking status measurement	EFO_0006527	Other measurement	Psychiatric
0.8311	5.25E-118	1.19E-114	PGS002354	MONDO_0005148	type 2 diabetes mellitus	type 2 diabetes mellitus	MONDO_0005148	Digestive system disorder	Gastrointestinal	PGS003093	MONDO_0005148	type 2 diabetes mellitus	type 2 diabetes mellitus	MONDO_0005148	Metabolic disorder	Metabolic
0.8311	5.25E-118	1.19E-114	PGS002354	MONDO_0005148	type 2 diabetes mellitus	type 2 diabetes mellitus	MONDO_0005148	Metabolic disorder	Metabolic	PGS003093	MONDO_0005148	type 2 diabetes mellitus	type 2 diabetes mellitus	MONDO_0005148	Metabolic disorder	Metabolic
0.8311	5.25E-118	1.19E-114	PGS002354	MONDO_0005148	type 2 diabetes mellitus	type 2 diabetes mellitus	MONDO_0005148	Digestive system disorder	Gastrointestinal	PGS003093	MONDO_0005148	type 2 diabetes mellitus	type 2 diabetes mellitus	MONDO_0005148	Digestive system disorder	Gastrointestinal
0.8311	5.25E-118	1.19E-114	PGS002354	MONDO_0005148	type 2 diabetes mellitus	type 2 diabetes mellitus	MONDO_0005148	Metabolic disorder	Metabolic	PGS003093	MONDO_0005148	type 2 diabetes mellitus	type 2 diabetes mellitus	MONDO_0005148	Digestive system disorder	Gastrointestinal
0.831	5.86E-118	1.32E-114	PGS000345	EFO_0000305	breast carcinoma	breast carcinoma	EFO_0000305	Cancer	Neoplasms	PGS000004	EFO_0000305	breast carcinoma	breast carcinoma	EFO_0000305	Cancer	Neoplasms
0.8308	7.28E-118	1.64E-114	PGS001327	EFO_0000400	diabetes mellitus	diabetes mellitus	EFO_0000400	Metabolic disorder	Metabolic	PGS001329	EFO_0000400	diabetes mellitus	diabetes mellitus	EFO_0000400	Metabolic disorder	Metabolic
0.8308	7.28E-118	1.64E-114	PGS001327	EFO_0000400	diabetes mellitus	diabetes mellitus	EFO_0000400	Digestive system disorder	Gastrointestinal	PGS001329	EFO_0000400	diabetes mellitus	diabetes mellitus	EFO_0000400	Digestive system disorder	Gastrointestinal
0.8308	7.28E-118	1.64E-114	PGS001327	EFO_0000400	diabetes mellitus	diabetes mellitus	EFO_0000400	Digestive system disorder	Gastrointestinal	PGS001329	EFO_0000400	diabetes mellitus	diabetes mellitus	EFO_0000400	Metabolic disorder	Metabolic
0.8308	7.28E-118	1.64E-114	PGS001327	EFO_0000400	diabetes mellitus	diabetes mellitus	EFO_0000400	Metabolic disorder	Metabolic	PGS001329	EFO_0000400	diabetes mellitus	diabetes mellitus	EFO_0000400	Digestive system disorder	Gastrointestinal
0.8308	7.40E-118	1.67E-114	PGS002850	EFO_0004340	body mass index	body mass index	EFO_0004340	Body measurement	Body measurement	PGS002313	EFO_0004340	body mass index	body mass index	EFO_0004340	Body measurement	Body measurement
0.8306	9.08E-118	2.04E-114	PGS001229	EFO_0004339	body height	body height	EFO_0004339	Body measurement	Body measurement	PGS002146	EFO_0004339	body height	body height	EFO_0004339	Body measurement	Body measurement
0.8305	1.02E-117	2.29E-114	PGS002786	MONDO_0004985	bipolar disorder	bipolar disorder	MONDO_0004985	Neurological disorder	Neurological	PGS002787	EFO_0009963	bipolar I disorder	bipolar I disorder	EFO_0009963	Neurological disorder	Neurological
0.8304	1.13E-117	2.55E-114	PGS001382	EFO_0010067	corneal resistance factor	corneal resistance factor	EFO_0010067	Other measurement	Ophthalmological	PGS001380	EFO_0010066	corneal hysteresis	corneal hysteresis	EFO_0010066	Other measurement	Ophthalmological
0.8304	1.21E-117	2.73E-114	PGS001973	EFO_0004309	platelet count	platelet count	EFO_0004309	Hematological measurement	Hematological	PGS001238	EFO_0004309	platelet count	platelet count	EFO_0004309	Hematological measurement	Hematological
0.8303	1.28E-117	2.88E-114	PGS001995	MONDO_0021107	narcolepsy	narcolepsy	MONDO_0021107	Neurological disorder	Neurological	PGS002212	MONDO_0021107	narcolepsy	narcolepsy	MONDO_0021107	Neurological disorder	Neurological
-0.8302	1.54E-117	3.47E-114	PGS001388	EFO_0005671	smoking behaviour measurement	smoking behaviour measurement	EFO_0005671	Other measurement	Psychiatric	PGS001532	EFO_0005671	smoking behaviour measurement	smoking behaviour measurement	EFO_0005671	Other measurement	Psychiatric
0.8302	1.61E-117	3.61E-114	PGS001454	EFO_0006930	brain volume measurement|neuroimaging measurement	brain volume measurement	EFO_0006930	Other measurement	Neurological	PGS001466	EFO_0006930	measurement	brain volume measurement	EFO_0006930	Other measurement	Neurological
0.8301	1.69E-117	3.79E-114	PGS000540	EFO_0000305	breast carcinoma	breast carcinoma	EFO_0000305	Cancer	Neoplasms	PGS000531	EFO_0000305	breast carcinoma	breast carcinoma	EFO_0000305	Cancer	Neoplasms
0.8301	1.80E-117	4.05E-114	PGS000672	EFO_0004615	apolipoprotein B measurement	apolipoprotein B measurement	EFO_0004615	Lipid or lipoprotein measurement	Metabolic	PGS002102	EFO_0004615	apolipoprotein B measurement	apolipoprotein B measurement	EFO_0004615	Lipid or lipoprotein measurement	Metabolic
0.83	1.99E-117	4.46E-114	PGS002958	EFO_0004612	high density lipoprotein cholesterol measurement	high density lipoprotein cholesterol measurement	EFO_0004612	Lipid or lipoprotein measurement	Metabolic	PGS002781	EFO_0004612	high density lipoprotein cholesterol measurement	measurement	EFO_0004612	Lipid or lipoprotein measurement	Metabolic
0.8298	2.41E-117	5.41E-114	PGS000500	EFO_0000305	breast carcinoma	breast carcinoma	EFO_0000305	Cancer	Neoplasms	PGS000512	EFO_0000305	breast carcinoma	breast carcinoma	EFO_0000305	Cancer	Neoplasms

0.8298	2.42E-117	5.42E-114	PGS002104	MONDO_0021945	hearing disorder	hearing disorder	MONDO_0021945	Neurological disorder	Neurological	PGS001891	MONDO_0021945	hearing disorder	hearing disorder	MONDO_0021945	Neurological disorder	Neurological
0.8298	2.43E-117	5.45E-114	PGS001148	EFO_0005409	fat body mass	fat body mass	EFO_0005409	Other measurement	Body measurement	PGS001102	EFO_0007800	body fat percentage	body fat percentage	EFO_0007800	Body measurement	Body measurement
0.8298	2.44E-117	5.47E-114	PGS002005	EFO_0011011	sitting height measurement	sitting height measurement	EFO_0011011	Other measurement	Skeletal	PGS000969	EFO_0011011	sitting height measurement	sitting height measurement	EFO_0011011	Other measurement	Skeletal
0.8298	2.62E-117	5.87E-114	PGS001200	EFO_0004584	mean platelet volume	mean platelet volume	EFO_0004584	Hematological measurement	Hematological	PGS000104	EFO_0004584	mean platelet volume	mean platelet volume	EFO_0004584	Hematological measurement	Hematological
0.8297	2.65E-117	5.92E-114	PGS000102	EFO_0005091	monocyte count	monocyte count	EFO_0005091	Hematological measurement	Hematological	PGS002658	EFO_0005091	monocyte count	monocyte count	EFO_0005091	Hematological measurement	Hematological
0.8296	3.16E-117	7.07E-114	PGS002337	EFO_0004611	low density lipoprotein cholesterol measurement	low density lipoprotein cholesterol measurement	EFO_0004611	Cardiovascular measurement	Cardiovascular	PGS002108	EFO_0004574	total cholesterol measurement	total cholesterol measurement	EFO_0004574	Lipid or lipoprotein measurement	Metabolic
0.8296	3.16E-117	7.07E-114	PGS002337	EFO_0004611	low density lipoprotein cholesterol measurement	low density lipoprotein cholesterol measurement	EFO_0004611	Lipid or lipoprotein measurement	Metabolic	PGS002108	EFO_0004574	total cholesterol measurement	total cholesterol measurement	EFO_0004574	Lipid or lipoprotein measurement	Metabolic
0.8295	3.64E-117	8.14E-114	PGS000013	EFO_0001645	coronary artery disease	coronary artery disease	EFO_0001645	Cardiovascular disease	Cardiovascular	PGS003355	EFO_0001645	coronary artery disease	coronary artery disease	EFO_0001645	Cardiovascular disease	Cardiovascular
0.8295	3.69E-117	8.24E-114	PGS000095	EFO_0007986	reticulocyte count	reticulocyte count	EFO_0007986	Hematological measurement	Hematological	PGS000111	EFO_0007986	reticulocyte count	reticulocyte count	EFO_0007986	Hematological measurement	Hematological
0.8295	3.78E-117	8.42E-114	PGS000611	MONDO_0004986	urinary bladder carcinoma	urinary bladder carcinoma	MONDO_0004986	Cancer	Neoplasms	PGS000071	MONDO_0004986	urinary bladder carcinoma	urinary bladder carcinoma	MONDO_0004986	Cancer	Neoplasms
0.8295	3.78E-117	8.42E-114	PGS000612	MONDO_0004986	urinary bladder carcinoma	urinary bladder carcinoma	MONDO_0004986	Cancer	Neoplasms	PGS000071	MONDO_0004986	urinary bladder carcinoma	urinary bladder carcinoma	MONDO_0004986	Cancer	Neoplasms
0.8295	3.78E-117	8.42E-114	PGS000071	MONDO_0004986	urinary bladder carcinoma	urinary bladder carcinoma	MONDO_0004986	Cancer	Neoplasms	PGS000610	MONDO_0004986	urinary bladder carcinoma	urinary bladder carcinoma	MONDO_0004986	Cancer	Neoplasms
0.8294	3.91E-117	8.72E-114	PGS000888	EFO_0004611	low density lipoprotein cholesterol measurement	low density lipoprotein cholesterol measurement	EFO_0004611	Lipid or lipoprotein measurement	Metabolic	PGS003038	EFO_0004611	low density lipoprotein cholesterol measurement	measurement	EFO_0004611	Cardiovascular measurement	Cardiovascular
0.8294	3.91E-117	8.72E-114	PGS000888	EFO_0004611	low density lipoprotein cholesterol measurement	low density lipoprotein cholesterol measurement	EFO_0004611	Lipid or lipoprotein measurement	Metabolic	PGS003038	EFO_0004611	low density lipoprotein cholesterol measurement	measurement	EFO_0004611	Lipid or lipoprotein measurement	Metabolic
0.8294	3.91E-117	8.72E-114	PGS000888	EFO_0004611	low density lipoprotein cholesterol measurement	low density lipoprotein cholesterol measurement	EFO_0004611	Cardiovascular measurement	Cardiovascular	PGS003038	EFO_0004611	low density lipoprotein cholesterol measurement	measurement	EFO_0004611	Lipid or lipoprotein measurement	Metabolic
0.8294	3.91E-117	8.72E-114	PGS000888	EFO_0004611	low density lipoprotein cholesterol measurement	low density lipoprotein cholesterol measurement	EFO_0004611	Cardiovascular measurement	Cardiovascular	PGS003038	EFO_0004611	low density lipoprotein cholesterol measurement	measurement	EFO_0004611	Cardiovascular measurement	Cardiovascular
0.8293	4.63E-117	1.03E-113	PGS000477	EFO_0000305	breast carcinoma	breast carcinoma	EFO_0000305	Cancer	Neoplasms	PGS000481	EFO_0000305	breast carcinoma	breast carcinoma	EFO_0000305	Cancer	Neoplasms
0.8292	4.84E-117	1.08E-113	PGS002221	EFO_0010701	mean reticulocyte volume	mean reticulocyte volume	EFO_0010701	Hematological measurement	Hematological	PGS000987	EFO_0010701	mean reticulocyte volume	mean reticulocyte volume	EFO_0010701	Hematological measurement	Hematological
0.8292	5.14E-117	1.14E-113	PGS002781	EFO_0004612	high density lipoprotein cholesterol measurement	high density lipoprotein cholesterol measurement	EFO_0004612	Lipid or lipoprotein measurement	Metabolic	PGS002963	EFO_0004612	high density lipoprotein cholesterol measurement	measurement	EFO_0004612	Lipid or lipoprotein measurement	Metabolic
0.829	6.32E-117	1.41E-113	PGS001737	EFO_0006930	brain volume measurement|neuroimaging measurement	brain volume measurement	EFO_0006930	Other measurement	Neurological	PGS001735	EFO_0006930	measurement	brain volume measurement	EFO_0006930	Other measurement	Neurological
0.8289	7.29E-117	1.62E-113	PGS000595	EFO_0005088	testicular carcinoma	testicular carcinoma	EFO_0005088	Cancer	Neoplasms	PGS000598	EFO_0005088	testicular carcinoma	testicular carcinoma	EFO_0005088	Cancer	Neoplasms
0.8288	7.91E-117	1.76E-113	PGS000990	EFO_0010698	retinal break|retinal detachment	retinal break	EFO_0010698	Neurological disorder	Neurological	PGS001137	EFO_0005773	retinal detachment	retinal detachment	EFO_0005773	Neurological disorder	Neurological
0.8288	8.15E-117	1.81E-113	PGS001681	EFO_0006930	brain volume measurement|neuroimaging measurement	brain volume measurement	EFO_0006930	Other measurement	Neurological	PGS001672	EFO_0006930	measurement	brain volume measurement	EFO_0006930	Other measurement	Neurological
0.8288	8.21E-117	1.82E-113	PGS000093	EFO_0004509	hemoglobin measurement	hemoglobin measurement	EFO_0004509	Hematological measurement	Hematological	PGS000092	EFO_0004348	hematocrit	hematocrit	EFO_0004348	Hematological measurement	Hematological
0.8288	8.48E-117	1.88E-113	PGS002845	EFO_0004340	body mass index	body mass index	EFO_0004340	Body measurement	Body measurement	PGS002313	EFO_0004340	body mass index	body mass index	EFO_0004340	Body measurement	Body measurement
0.8288	8.52E-117	1.89E-113	PGS001673	EFO_0006930	brain volume measurement|neuroimaging measurement	brain volume measurement	EFO_0006930	Other measurement	Neurological	PGS001680	EFO_0006930	measurement	brain volume measurement	EFO_0006930	Other measurement	Neurological
0.8287	9.39E-117	2.08E-113	PGS000476	EFO_0000305	breast carcinoma	breast carcinoma	EFO_0000305	Cancer	Neoplasms	PGS000472	EFO_0000305	breast carcinoma	breast carcinoma	EFO_0000305	Cancer	Neoplasms
0.8286	1.06E-116	2.35E-113	PGS003059	EFO_0006527	smoking status measurement	smoking status measurement	EFO_0006527	Other measurement	Psychiatric	PGS003068	EFO_0006527	smoking status measurement	smoking status measurement	EFO_0006527	Other measurement	Psychiatric
0.8286	1.08E-116	2.39E-113	PGS003143	EFO_0004574	total cholesterol measurement	total cholesterol measurement	EFO_0004574	Lipid or lipoprotein measurement	Metabolic	PGS003134	EFO_0004574	total cholesterol measurement	total cholesterol measurement	EFO_0004574	Lipid or lipoprotein measurement	Metabolic
0.8286	1.12E-116	2.48E-113	PGS003314	EFO_0004617	cystatin C measurement|glomerular filtration rate	cystatin C measurement	EFO_0004617	Other measurement	Metabolic	PGS003298	EFO_0004617	cystatin C measurement|glomerular filtration rate	cystatin C measurement	EFO_0004617	Other measurement	Metabolic
0.8285	1.14E-116	2.53E-113	PGS001480	EFO_0006930	brain volume measurement|neuroimaging measurement	brain volume measurement	EFO_0006930	Other measurement	Neurological	PGS001673	EFO_0006930	measurement	brain volume measurement	EFO_0006930	Other measurement	Neurological
0.8285	1.20E-116	2.64E-113	PGS001368	EFO_0004731	eye measurement	eye measurement	EFO_0004731	Other measurement	Ophthalmological	PGS001365	EFO_0004731	eye measurement	eye measurement	EFO_0004731	Other measurement	Ophthalmological
0.8284	1.39E-116	3.07E-113	PGS000388	MONDO_0001672	bronchus cancer|lung cancer|tracheal cancer	bronchus cancer	MONDO_0001672	Cancer	Neoplasms	PGS000394	MONDO_0001672	bronchus cancer|lung cancer|tracheal cancer	bronchus cancer	MONDO_0001672	Cancer	Neoplasms
0.8284	1.39E-116	3.07E-113	PGS000388	MONDO_0001672	bronchus cancer|lung cancer|tracheal cancer	bronchus cancer	MONDO_0001672	Cancer	Neoplasms	PGS000393	MONDO_0001672	bronchus cancer|lung cancer|tracheal cancer	bronchus cancer	MONDO_0001672	Cancer	Neoplasms
0.8283	1.51E-116	3.33E-113	PGS002449	EFO_0004468	glucose measurement	glucose measurement	EFO_0004468	Other measurement	Metabolic	PGS002573	MONDO_0005148	type 2 diabetes mellitus	type 2 diabetes mellitus	MONDO_0005148	Digestive system disorder	Gastrointestinal
0.8283	1.51E-116	3.33E-113	PGS002449	EFO_0004468	glucose measurement	glucose measurement	EFO_0004468	Other measurement	Metabolic	PGS002573	MONDO_0005148	type 2 diabetes mellitus	type 2 diabetes mellitus	MONDO_0005148	Metabolic disorder	Metabolic
0.8282	1.63E-116	3.59E-113	PGS001230	EFO_0004338	body weight	body weight	EFO_0004338	Body measurement	Body measurement	PGS001227	EFO_0004342	waist circumference	waist circumference	EFO_0004342	Body measurement	Body measurement
0.8282	1.66E-116	3.65E-113	PGS002337	EFO_0004611	low density lipoprotein cholesterol measurement	low density lipoprotein cholesterol measurement	EFO_0004611	Cardiovascular measurement	Cardiovascular	PGS001895	EFO_0004574	total cholesterol measurement	total cholesterol measurement	EFO_0004574	Lipid or lipoprotein measurement	Metabolic
0.8282	1.66E-116	3.65E-113	PGS002337	EFO_0004611	low density lipoprotein cholesterol measurement	low density lipoprotein cholesterol measurement	EFO_0004611	Lipid or lipoprotein measurement	Metabolic	PGS001895	EFO_0004574	total cholesterol measurement	total cholesterol measurement	EFO_0004574	Lipid or lipoprotein measurement	Metabolic
0.8282	1.80E-116	3.98E-113	PGS002311	MONDO_0004979	asthma	asthma	MONDO_0004979	Other disease	Respiratory	PGS002754	MONDO_0004979	asthma	asthma	MONDO_0004979	Other disease	Respiratory
0.828	2.16E-116	4.76E-113	PGS000501	EFO_0000305	breast carcinoma	breast carcinoma	EFO_0000305	Cancer	Neoplasms	PGS000498	EFO_0000305	breast carcinoma	breast carcinoma	EFO_0000305	Cancer	Neoplasms
0.828	2.16E-116	4.76E-113	PGS000501	EFO_0000305	breast carcinoma	breast carcinoma	EFO_0000305	Cancer	Neoplasms	PGS000497	EFO_0000305	breast carcinoma	breast carcinoma	EFO_0000305	Cancer	Neoplasms
0.8279	2.35E-116	5.17E-113	PGS000385	EFO_0002618	pancreatic carcinoma	pancreatic carcinoma	EFO_0002618	Cancer	Neoplasms	PGS000083	EFO_0002618	pancreatic carcinoma	pancreatic carcinoma	EFO_0002618	Digestive system disorder	Gastrointestinal
0.8279	2.35E-116	5.17E-113	PGS000385	EFO_0002618	pancreatic carcinoma	pancreatic carcinoma	EFO_0002618	Digestive system disorder	Gastrointestinal	PGS000083	EFO_0002618	pancreatic carcinoma	pancreatic carcinoma	EFO_0002618	Cancer	Neoplasms
0.8279	2.35E-116	5.17E-113	PGS000385	EFO_0002618	pancreatic carcinoma	pancreatic carcinoma	EFO_0002618	Digestive system disorder	Gastrointestinal	PGS000083	EFO_0002618	pancreatic carcinoma	pancreatic carcinoma	EFO_0002618	Digestive system disorder	Gastrointestinal
0.8279	2.35E-116	5.17E-113	PGS000385	EFO_0002618	pancreatic carcinoma	pancreatic carcinoma	EFO_0002618	Cancer	Neoplasms	PGS000083	EFO_0002618	pancreatic carcinoma	pancreatic carcinoma	EFO_0002618	Cancer	Neoplasms
0.8279	2.36E-116	5.19E-113	PGS002846	EFO_0004340	body mass index	body mass index	EFO_0004340	Body measurement	Body measurement	PGS002842	EFO_0004340	body mass index	body mass index	EFO_0004340	Body measurement	Body measurement
0.8279	2.36E-116	5.20E-113	PGS002645	EFO_0004468	glucose measurement	glucose measurement	EFO_0004468	Other measurement	Metabolic	PGS002328	EFO_0004468	glucose measurement	glucose measurement	EFO_0004468	Other measurement	Metabolic
0.8279	2.60E-116	5.71E-113	PGS002576	EFO_0004308	leukocyte count	leukocyte count	EFO_0004308	Hematological measurement	Hematological	PGS002478	EFO_0004308	leukocyte count	leukocyte count	EFO_0004308	Hematological measurement	Hematological
0.8278	2.69E-116	5.91E-113	PGS003314	EFO_0004617	cystatin C measurement|glomerular filtration rate	cystatin C measurement	EFO_0004617	Other measurement	Metabolic	PGS003309	EFO_0004617	cystatin C measurement|glomerular filtration rate	cystatin C measurement	EFO_0004617	Other measurement	Metabolic
0.8278	2.72E-116	5.97E-113	PGS002946	EFO_0004465	fasting blood glucose measurement	fasting blood glucose measurement	EFO_0004465	Other measurement	Metabolic	PGS002945	EFO_0004465	fasting blood glucose measurement	fasting blood glucose measurement	EFO_0004465	Other measurement	Metabolic
0.8278	2.80E-116	6.16E-113	PGS001674	EFO_0006930	brain volume measurement|neuroimaging measurement	brain volume measurement	EFO_0006930	Other measurement	Neurological	PGS001477	EFO_0006930	measurement	brain volume measurement	EFO_0006930	Other measurement	Neurological
0.8274	4.27E-116	9.37E-113	PGS000355	MONDO_0004992	cancer	cancer	MONDO_0004992	Cancer	Neoplasms	PGS000356	MONDO_0004992	cancer	cancer	MONDO_0004992	Cancer	Neoplasms
0.8271	6.03E-116	1.32E-112	PGS002524	MONDO_0005148	type 2 diabetes mellitus	type 2 diabetes mellitus	MONDO_0005148	Metabolic disorder	Metabolic	PGS002396	EFO_0001379	endocrine system disease|diabetes mellitus	endocrine system disease	EFO_0001379	Other disease	Metabolic
0.8271	6.03E-116	1.32E-112	PGS002524	MONDO_0005148	type 2 diabetes mellitus	type 2 diabetes mellitus	MONDO_0005148	Digestive system disorder	Gastrointestinal	PGS002396	EFO_0001379	endocrine system disease|diabetes mellitus	endocrine system disease	EFO_0001379	Other disease	Metabolic
0.8271	6.26E-116	1.37E-112	PGS002889	EFO_0006336	diastolic blood pressure	diastolic blood pressure	EFO_0006336	Other measurement	Cardiovascular	PGS002898	EFO_0006336	diastolic blood pressure	diastolic blood pressure	EFO_0006336	Other measurement	Cardiovascular
0.8271	6.32E-116	1.38E-112	PGS002244	EFO_0001645	coronary artery disease	coronary artery disease	EFO_0001645	Cardiovascular disease	Cardiovascular	PGS001355	EFO_0001645	coronary artery disease	coronary artery disease	EFO_0001645	Cardiovascular disease	Cardiovascular
0.8271	6.51E-116	1.43E-112	PGS003310	EFO_0004617	cystatin C measurement|glomerular filtration rate	cystatin C measurement	EFO_0004617	Other measurement	Metabolic	PGS003314	EFO_0004617	cystatin C measurement|glomerular filtration rate	cystatin C measurement	EFO_0004617	Other measurement	Metabolic
0.8269	7.99E-116	1.75E-112	PGS002169	EFO_0005409	fat body mass	fat body mass	EFO_0005409	Other measurement	Body measurement	PGS001983	EFO_0004342	waist circumference	waist circumference	EFO_0004342	Body measurement	Body measurement
0.8269	8.03E-116	1.76E-112	PGS001945	EFO_0004518	creatinine measurement	creatinine measurement	EFO_0004518	Other measurement	Metabolic	PGS003243	EFO_0010472	creatine measurement	creatine measurement	EFO_0010472	Other measurement	Metabolic
0.8269	8.40E-116	1.84E-112	PGS000288	EFO_0007937	blood protein measurement	blood protein measurement	EFO_0007937	Other measurement	Hematological	PGS000287	EFO_0007937	blood protein measurement	blood protein measurement	EFO_0007937	Other measurement	Hematological
0.8268	9.38E-116	2.05E-112	PGS001200	EFO_0004584	mean platelet volume	mean platelet volume	EFO_0004584	Hematological measurement	Hematological	PGS002189	EFO_0004584	mean platelet volume	mean platelet volume	EFO_0004584	Hematological measurement	Hematological
0.8266	1.16E-115	2.53E-112	PGS001508	EFO_0004346	neuroimaging measurement	neuroimaging measurement	EFO_0004346	Other measurement	Neurological	PGS001507	EFO_0004346	neuroimaging measurement	neuroimaging measurement	EFO_0004346	Other measurement	Neurological
0.8264	1.48E-115	3.23E-112	PGS003031	EFO_0004611	low density lipoprotein cholesterol measurement	low density lipoprotein cholesterol measurement	EFO_0004611	Cardiovascular measurement	Cardiovascular	PGS003141	EFO_0004574	total cholesterol measurement	total cholesterol measurement	EFO_0004574	Lipid or lipoprotein measurement	Metabolic
0.8264	1.48E-115	3.23E-112	PGS003031	EFO_0004611	low density lipoprotein cholesterol measurement	low density lipoprotein cholesterol measurement	EFO_0004611	Lipid or lipoprotein measurement	Metabolic	PGS003141	EFO_0004574	total cholesterol measurement	total cholesterol measurement	EFO_0004574	Lipid or lipoprotein measurement	Metabolic
0.8263	1.68E-115	3.67E-112	PGS003078	EFO_0006335	systolic blood pressure	systolic blood pressure	EFO_0006335	Other measurement	Cardiovascular	PGS002349	EFO_0006335	systolic blood pressure	systolic blood pressure	EFO_0006335	Other measurement	Cardiovascular
0.8262	1.88E-115	4.09E-112	PGS000313	EFO_0006925	lipoprotein A measurement	lipoprotein A measurement	EFO_0006925	Lipid or lipoprotein measurement	Metabolic	PGS002297	EFO_0006925	lipoprotein A measurement	lipoprotein A measurement	EFO_0006925	Lipid or lipoprotein measurement	Metabolic
0.8259	2.50E-115	5.45E-112	PGS001377	EFO_0007992	basophil percentage of leukocytes	basophil percentage of leukocytes	EFO_0007992	Inflammatory measurement	Immunological	PGS001378	EFO_0005090	basophil count	basophil count	EFO_0005090	Inflammatory measurement	Immunological
0.8259	2.50E-115	5.45E-112	PGS001377	EFO_0007992	basophil percentage of leukocytes	basophil percentage of leukocytes	EFO_0007992	Hematological measurement	Hematological	PGS001378	EFO_0005090	basophil count	basophil count	EFO_0005090	Inflammatory measurement	Immunological
0.8259	2.50E-115	5.45E-112	PGS001377	EFO_0007992	basophil percentage of leukocytes	basophil percentage of leukocytes	EFO_0007992	Hematological measurement	Hematological	PGS001378	EFO_0005090	basophil count	basophil count	EFO_0005090	Hematological measurement	Hematological
0.8259	2.50E-115	5.45E-112	PGS001377	EFO_0007992	basophil percentage of leukocytes	basophil percentage of leukocytes	EFO_0007992	Inflammatory measurement	Immunological	PGS001378	EFO_0005090	basophil count	basophil count	EFO_0005090	Hematological measurement	Hematological
0.8259	2.61E-115	5.68E-112	PGS003034	EFO_0004611	low density lipoprotein cholesterol measurement	low density lipoprotein cholesterol measurement	EFO_0004611	Cardiovascular measurement	Cardiovascular	PGS003038	EFO_0004611	low density lipoprotein cholesterol measurement	measurement	EFO_0004611	Lipid or lipoprotein measurement	Metabolic
0.8259	2.61E-115	5.68E-112	PGS003034	EFO_0004611	low density lipoprotein cholesterol measurement	low density lipoprotein cholesterol measurement	EFO_0004611	Lipid or lipoprotein measurement	Metabolic	PGS003038	EFO_0004611	low density lipoprotein cholesterol measurement	measurement	EFO_0004611	Cardiovascular measurement	Cardiovascular
0.8259	2.61E-115	5.68E-112	PGS003034	EFO_0004611	low density lipoprotein cholesterol measurement	low density lipoprotein cholesterol measurement	EFO_0004611	Lipid or lipoprotein measurement	Metabolic	PGS003038	EFO_0004611	low density lipoprotein cholesterol measurement	measurement	EFO_0004611	Lipid or lipoprotein measurement	Metabolic
0.8259	2.61E-115	5.68E-112	PGS003034	EFO_0004611	low density lipoprotein cholesterol measurement	low density lipoprotein cholesterol measurement	EFO_0004611	Cardiovascular measurement	Cardiovascular	PGS003038	EFO_0004611	low density lipoprotein cholesterol measurement	measurement	EFO_0004611	Cardiovascular measurement	Cardiovascular
0.8258	2.81E-115	6.12E-112	PGS002672	EFO_0004631	vitamin D measurement	vitamin D measurement	EFO_0004631	Lipid or lipoprotein measurement	Metabolic	PGS000702	EFO_0004631	vitamin D measurement	vitamin D measurement	EFO_0004631	Lipid or lipoprotein measurement	Metabolic
0.8257	3.34E-115	7.28E-112	PGS002076	EFO_0000284	benign prostatic hyperplasia	benign prostatic hyperplasia	EFO_0000284	Other disease	Genitourinary	PGS001865	EFO_0000284	benign prostatic hyperplasia	benign prostatic hyperplasia	EFO_0000284	Other disease	Genitourinary
0.8256	3.65E-115	7.95E-112	PGS002228	EFO_0006335	systolic blood pressure	systolic blood pressure	EFO_0006335	Other measurement	Cardiovascular	PGS002807	EFO_0006335	systolic blood pressure	systolic blood pressure	EFO_0006335	Other measurement	Cardiovascular
0.8256	4.01E-115	8.72E-112	PGS001986	EFO_0007993	lymphocyte percentage of leukocytes	lymphocyte percentage of leukocytes	EFO_0007993	Hematological measurement	Hematological	PGS001414	EFO_0007993	lymphocyte percentage of leukocytes	lymphocyte percentage of leukocytes	EFO_0007993	Hematological measurement	Hematological
0.8255	4.51E-115	9.80E-112	PGS001103	EFO_0007800	body fat percentage	body fat percentage	EFO_0007800	Body measurement	Body measurement	PGS001147	EFO_0005409	fat body mass	fat body mass	EFO_0005409	Other measurement	Body measurement
0.8254	4.87E-115	1.06E-111	PGS002241	EFO_0001663	prostate carcinoma	prostate carcinoma	EFO_0001663	Cancer	Neoplasms	PGS000584	EFO_0001663	prostate carcinoma	prostate carcinoma	EFO_0001663	Cancer	Neoplasms
0.8254	5.00E-115	1.09E-111	PGS002448	EFO_0004312	vital capacity	vital capacity	EFO_0004312	Other measurement	Activities	PGS002399	EFO_0004312	vital capacity	vital capacity	EFO_0004312	Other measurement	Activities
0.8254	5.02E-115	1.09E-111	PGS000931	MONDO_0000831	thrombotic disease|deep vein thrombosis	thrombotic disease	MONDO_0000831	Cardiovascular disease	Cardiovascular	PGS001279	EFO_0003827	pulmonary embolism|deep vein thrombosis	pulmonary embolism	EFO_0003827	Cardiovascular disease	Cardiovascular
0.8253	5.09E-115	1.11E-111	PGS003246	EFO_0010472	creatine measurement	creatine measurement	EFO_0010472	Other measurement	Metabolic	PGS003245	EFO_0010472	creatine measurement	creatine measurement	EFO_0010472	Other measurement	Metabolic
0.8253	5.46E-115	1.19E-111	PGS001955	EFO_0009270	heel bone mineral density	heel bone mineral density	EFO_0009270	Other measurement	Skeletal	PGS001402	EFO_0009270	heel bone mineral density	heel bone mineral density	EFO_0009270	Other measurement	Skeletal
0.8253	5.48E-115	1.19E-111	PGS000700	EFO_0004531	urate measurement	urate measurement	EFO_0004531	Other measurement	Metabolic	PGS002010	EFO_0004531	urate measurement	urate measurement	EFO_0004531	Other measurement	Metabolic
0.8252	5.82E-115	1.26E-111	PGS002443	EFO_0006336	diastolic blood pressure	diastolic blood pressure	EFO_0006336	Other measurement	Cardiovascular	PGS002394	EFO_0006336	diastolic blood pressure	diastolic blood pressure	EFO_0006336	Other measurement	Cardiovascular
0.8252	5.91E-115	1.28E-111	PGS003134	EFO_0004574	total cholesterol measurement	total cholesterol measurement	EFO_0004574	Lipid or lipoprotein measurement	Metabolic	PGS003138	EFO_0004574	total cholesterol measurement	total cholesterol measurement	EFO_0004574	Lipid or lipoprotein measurement	Metabolic
-0.8251	6.46E-115	1.40E-111	PGS002309	EFO_0009102	number of children ever born measurement	number of children ever born measurement	EFO_0009102	Other measurement	Genitourinary	PGS002317	EFO_0007862	reproductive behaviour measurement	reproductive behaviour measurement	EFO_0007862	Other measurement	Activities
0.8251	6.62E-115	1.43E-111	PGS000952	EFO_0004514	bone quantitative ultrasound measurement	bone quantitative ultrasound measurement	EFO_0004514	Other measurement	Skeletal	PGS001955	EFO_0009270	heel bone mineral density	heel bone mineral density	EFO_0009270	Other measurement	Skeletal
0.8251	6.73E-115	1.46E-111	PGS001955	EFO_0009270	heel bone mineral density	heel bone mineral density	EFO_0009270	Other measurement	Skeletal	PGS001037	EFO_0009270	heel bone mineral density	heel bone mineral density	EFO_0009270	Other measurement	Skeletal
0.8251	6.77E-115	1.46E-111	PGS001103	EFO_0007800	body fat percentage	body fat percentage	EFO_0007800	Body measurement	Body measurement	PGS001102	EFO_0007800	body fat percentage	body fat percentage	EFO_0007800	Body measurement	Body measurement
0.8248	9.59E-115	2.07E-111	PGS003029	EFO_0004611	low density lipoprotein cholesterol measurement	low density lipoprotein cholesterol measurement	EFO_0004611	Cardiovascular measurement	Cardiovascular	PGS003033	EFO_0004611	low density lipoprotein cholesterol measurement	measurement	EFO_0004611	Cardiovascular measurement	Cardiovascular
0.8248	9.59E-115	2.07E-111	PGS003029	EFO_0004611	low density lipoprotein cholesterol measurement	low density lipoprotein cholesterol measurement	EFO_0004611	Cardiovascular measurement	Cardiovascular	PGS003033	EFO_0004611	low density lipoprotein cholesterol measurement	measurement	EFO_0004611	Lipid or lipoprotein measurement	Metabolic
0.8248	9.59E-115	2.07E-111	PGS003029	EFO_0004611	low density lipoprotein cholesterol measurement	low density lipoprotein cholesterol measurement	EFO_0004611	Lipid or lipoprotein measurement	Metabolic	PGS003033	EFO_0004611	low density lipoprotein cholesterol measurement	measurement	EFO_0004611	Cardiovascular measurement	Cardiovascular
0.8248	9.59E-115	2.07E-111	PGS003029	EFO_0004611	low density lipoprotein cholesterol measurement	low density lipoprotein cholesterol measurement	EFO_0004611	Lipid or lipoprotein measurement	Metabolic	PGS003033	EFO_0004611	low density lipoprotein cholesterol measurement	measurement	EFO_0004611	Lipid or lipoprotein measurement	Metabolic
0.8247	1.03E-114	2.23E-111	PGS000743	EFO_0000756	melanoma	melanoma	EFO_0000756	Cancer	Neoplasms	PGS000744	EFO_0000756	melanoma	melanoma	EFO_0000756	Cancer	Neoplasms
0.8247	1.06E-114	2.29E-111	PGS003029	EFO_0004611	low density lipoprotein cholesterol measurement	low density lipoprotein cholesterol measurement	EFO_0004611	Cardiovascular measurement	Cardiovascular	PGS003038	EFO_0004611	low density lipoprotein cholesterol measurement	measurement	EFO_0004611	Cardiovascular measurement	Cardiovascular
0.8247	1.06E-114	2.29E-111	PGS003029	EFO_0004611	low density lipoprotein cholesterol measurement	low density lipoprotein cholesterol measurement	EFO_0004611	Lipid or lipoprotein measurement	Metabolic	PGS003038	EFO_0004611	low density lipoprotein cholesterol measurement	measurement	EFO_0004611	Cardiovascular measurement	Cardiovascular
0.8247	1.06E-114	2.29E-111	PGS003029	EFO_0004611	low density lipoprotein cholesterol measurement	low density lipoprotein cholesterol measurement	EFO_0004611	Lipid or lipoprotein measurement	Metabolic	PGS003038	EFO_0004611	low density lipoprotein cholesterol measurement	measurement	EFO_0004611	Lipid or lipoprotein measurement	Metabolic
0.8247	1.06E-114	2.29E-111	PGS003029	EFO_0004611	low density lipoprotein cholesterol measurement	low density lipoprotein cholesterol measurement	EFO_0004611	Cardiovascular measurement	Cardiovascular	PGS003038	EFO_0004611	low density lipoprotein cholesterol measurement	measurement	EFO_0004611	Lipid or lipoprotein measurement	Metabolic
0.8247	1.10E-114	2.38E-111	PGS002672	EFO_0004631	vitamin D measurement	vitamin D measurement	EFO_0004631	Lipid or lipoprotein measurement	Metabolic	PGS003198	EFO_0004631	vitamin D measurement	vitamin D measurement	EFO_0004631	Lipid or lipoprotein measurement	Metabolic
0.8247	1.14E-114	2.46E-111	PGS003034	EFO_0004611	low density lipoprotein cholesterol measurement	low density lipoprotein cholesterol measurement	EFO_0004611	Cardiovascular measurement	Cardiovascular	PGS003033	EFO_0004611	low density lipoprotein cholesterol measurement	measurement	EFO_0004611	Cardiovascular measurement	Cardiovascular

0.8247	1.14E-114	2.46E-111	PGS003034	EFO_0004611	low density lipoprotein cholesterol measurement	low density lipoprotein cholesterol measurement	EFO_0004611	Lipid or lipoprotein measurement	Metabolic	PGS003033	EFO_0004611	low density lipoprotein cholesterol measurement	measurement	EFO_0004611	Lipid or lipoprotein measurement	Metabolic
0.8247	1.14E-114	2.46E-111	PGS003034	EFO_0004611	low density lipoprotein cholesterol measurement	low density lipoprotein cholesterol measurement	EFO_0004611	Lipid or lipoprotein measurement	Metabolic	PGS003033	EFO_0004611	low density lipoprotein cholesterol measurement	measurement	EFO_0004611	Cardiovascular measurement	Cardiovascular
0.8247	1.14E-114	2.46E-111	PGS003034	EFO_0004611	low density lipoprotein cholesterol measurement	low density lipoprotein cholesterol measurement	EFO_0004611	Cardiovascular measurement	Cardiovascular	PGS003033	EFO_0004611	low density lipoprotein cholesterol measurement	measurement	EFO_0004611	Lipid or lipoprotein measurement	Metabolic
0.8246	1.18E-114	2.54E-111	PGS001918	EFO_0004314	forced expiratory volume	forced expiratory volume	EFO_0004314	Other measurement	Respiratory	PGS002138	EFO_0004312	vital capacity	vital capacity	EFO_0004312	Other measurement	Activities
0.8246	1.27E-114	2.74E-111	PGS001230	EFO_0004338	body weight	body weight	EFO_0004338	Body measurement	Body measurement	PGS001002	EFO_0009805	whole body water mass	whole body water mass	EFO_0009805	Other measurement	Body measurement
0.8245	1.37E-114	2.95E-111	PGS000317	EFO_0000305	breast carcinoma	breast carcinoma	EFO_0000305	Cancer	Neoplasms	PGS000873	EFO_0000305	breast carcinoma	breast carcinoma	EFO_0000305	Cancer	Neoplasms
0.8245	1.45E-114	3.13E-111	PGS001147	EFO_0005409	fat body mass	fat body mass	EFO_0005409	Other measurement	Body measurement	PGS001101	EFO_0007800	body fat percentage	body fat percentage	EFO_0007800	Body measurement	Body measurement
0.8244	1.55E-114	3.34E-111	PGS000392	MONDO_0001672	bronchus cancer|lung cancer|tracheal cancer	bronchus cancer	MONDO_0001672	Cancer	Neoplasms	PGS000880	EFO_0001071	lung carcinoma	lung carcinoma	EFO_0001071	Cancer	Neoplasms
0.8243	1.68E-114	3.61E-111	PGS000534	EFO_0000305	breast carcinoma	breast carcinoma	EFO_0000305	Cancer	Neoplasms	PGS000530	EFO_0000305	breast carcinoma	breast carcinoma	EFO_0000305	Cancer	Neoplasms
0.8243	1.68E-114	3.61E-111	PGS000533	EFO_0000305	breast carcinoma	breast carcinoma	EFO_0000305	Cancer	Neoplasms	PGS000530	EFO_0000305	breast carcinoma	breast carcinoma	EFO_0000305	Cancer	Neoplasms
0.8242	1.94E-114	4.17E-111	PGS001363	EFO_0004731	eye measurement	eye measurement	EFO_0004731	Other measurement	Ophthalmological	PGS001365	EFO_0004731	eye measurement	eye measurement	EFO_0004731	Other measurement	Ophthalmological
0.8242	2.00E-114	4.31E-111	PGS000435	MONDO_0002898	skin cancer	skin cancer	MONDO_0002898	Cancer	Neoplasms	PGS000402	EFO_0009259	skin carcinoma	skin carcinoma	EFO_0009259	Cancer	Neoplasms
0.8241	2.09E-114	4.49E-111	PGS001278	EFO_0003827	pulmonary embolism|deep vein thrombosis	pulmonary embolism	EFO_0003827	Cardiovascular disease	Cardiovascular	PGS000961	EFO_1001395	Phlebitis|Thrombophlebitis	Phlebitis	EFO_1001395	Cardiovascular disease	Cardiovascular
0.8241	2.24E-114	4.81E-111	PGS003073	EFO_0006335	systolic blood pressure	systolic blood pressure	EFO_0006335	Other measurement	Cardiovascular	PGS002349	EFO_0006335	systolic blood pressure	systolic blood pressure	EFO_0006335	Other measurement	Cardiovascular
0.824	2.34E-114	5.03E-111	PGS003069	EFO_0006335	systolic blood pressure	systolic blood pressure	EFO_0006335	Other measurement	Cardiovascular	PGS003073	EFO_0006335	systolic blood pressure	systolic blood pressure	EFO_0006335	Other measurement	Cardiovascular
0.8239	2.89E-114	6.20E-111	PGS002150	EFO_0004611	low density lipoprotein cholesterol measurement	low density lipoprotein cholesterol measurement	EFO_0004611	Lipid or lipoprotein measurement	Metabolic	PGS002654	EFO_0004611	low density lipoprotein cholesterol measurement	measurement	EFO_0004611	Cardiovascular measurement	Cardiovascular
0.8239	2.89E-114	6.20E-111	PGS002150	EFO_0004611	low density lipoprotein cholesterol measurement	low density lipoprotein cholesterol measurement	EFO_0004611	Cardiovascular measurement	Cardiovascular	PGS002654	EFO_0004611	low density lipoprotein cholesterol measurement	measurement	EFO_0004611	Cardiovascular measurement	Cardiovascular
0.8239	2.89E-114	6.20E-111	PGS002150	EFO_0004611	low density lipoprotein cholesterol measurement	low density lipoprotein cholesterol measurement	EFO_0004611	Cardiovascular measurement	Cardiovascular	PGS002654	EFO_0004611	low density lipoprotein cholesterol measurement	measurement	EFO_0004611	Lipid or lipoprotein measurement	Metabolic
0.8239	2.89E-114	6.20E-111	PGS002150	EFO_0004611	low density lipoprotein cholesterol measurement	low density lipoprotein cholesterol measurement	EFO_0004611	Lipid or lipoprotein measurement	Metabolic	PGS002654	EFO_0004611	low density lipoprotein cholesterol measurement	measurement	EFO_0004611	Lipid or lipoprotein measurement	Metabolic
0.8238	2.98E-114	6.40E-111	PGS001529	EFO_0007772	calcaneal bone quantitative ultrasound measurement	calcaneal bone quantitative ultrasound measurement	EFO_0007772	Other measurement	Skeletal	PGS002175	EFO_0005654	velocity of sound measurement	velocity of sound measurement	EFO_0005654	Other measurement	Others
0.8238	3.25E-114	6.97E-111	PGS002241	EFO_0001663	prostate carcinoma	prostate carcinoma	EFO_0001663	Cancer	Neoplasms	PGS000583	EFO_0001663	prostate carcinoma	prostate carcinoma	EFO_0001663	Cancer	Neoplasms
0.8236	4.02E-114	8.60E-111	PGS001962	EFO_0004308	leukocyte count	leukocyte count	EFO_0004308	Hematological measurement	Hematological	PGS001969	EFO_0004833	neutrophil count	neutrophil count	EFO_0004833	Hematological measurement	Hematological
0.8236	4.07E-114	8.71E-111	PGS000931	MONDO_0000831	thrombotic disease|deep vein thrombosis	thrombotic disease	MONDO_0000831	Cardiovascular disease	Cardiovascular	PGS001280	EFO_0003827	pulmonary embolism	pulmonary embolism	EFO_0003827	Cardiovascular disease	Cardiovascular
0.8234	4.96E-114	1.06E-110	PGS000511	EFO_0000305	breast carcinoma	breast carcinoma	EFO_0000305	Cancer	Neoplasms	PGS000508	EFO_0000305	breast carcinoma	breast carcinoma	EFO_0000305	Cancer	Neoplasms
0.8233	5.34E-114	1.14E-110	PGS001081	EFO_0007937	blood protein measurement	blood protein measurement	EFO_0007937	Other measurement	Hematological	PGS000976	EFO_0010907	polyomavirus 2 seropositivity	polyomavirus 2 seropositivity	EFO_0010907	Other measurement	Infection
0.8233	5.45E-114	1.17E-110	PGS003103	MONDO_0005148	type 2 diabetes mellitus	type 2 diabetes mellitus	MONDO_0005148	Metabolic disorder	Metabolic	PGS002771	MONDO_0005148	type 2 diabetes mellitus	type 2 diabetes mellitus	MONDO_0005148	Metabolic disorder	Metabolic
0.8233	5.45E-114	1.17E-110	PGS003103	MONDO_0005148	type 2 diabetes mellitus	type 2 diabetes mellitus	MONDO_0005148	Metabolic disorder	Metabolic	PGS002771	MONDO_0005148	type 2 diabetes mellitus	type 2 diabetes mellitus	MONDO_0005148	Digestive system disorder	Gastrointestinal
0.8233	5.45E-114	1.17E-110	PGS003103	MONDO_0005148	type 2 diabetes mellitus	type 2 diabetes mellitus	MONDO_0005148	Digestive system disorder	Gastrointestinal	PGS002771	MONDO_0005148	type 2 diabetes mellitus	type 2 diabetes mellitus	MONDO_0005148	Metabolic disorder	Metabolic
0.8233	5.45E-114	1.17E-110	PGS003103	MONDO_0005148	type 2 diabetes mellitus	type 2 diabetes mellitus	MONDO_0005148	Digestive system disorder	Gastrointestinal	PGS002771	MONDO_0005148	type 2 diabetes mellitus	type 2 diabetes mellitus	MONDO_0005148	Digestive system disorder	Gastrointestinal
0.8233	5.54E-114	1.18E-110	PGS001229	EFO_0004339	body height	body height	EFO_0004339	Body measurement	Body measurement	PGS002968	EFO_0004339	body height	body height	EFO_0004339	Body measurement	Body measurement
0.823	8.40E-114	1.79E-110	PGS001169	EFO_0004995	lean body mass	lean body mass	EFO_0004995	Other measurement	Skeletal	PGS001230	EFO_0004338	body weight	body weight	EFO_0004338	Body measurement	Body measurement
0.8229	8.49E-114	1.81E-110	PGS001823	MONDO_0002279	iron metabolism disease	iron metabolism disease	MONDO_0002279	Metabolic disorder	Metabolic	PGS001023	EFO_0009556	mineral metabolism disease	mineral metabolism disease	EFO_0009556	Metabolic disorder	Metabolic
0.8228	9.51E-114	2.03E-110	PGS002934	EFO_0004468	glucose measurement	glucose measurement	EFO_0004468	Other measurement	Metabolic	PGS002938	EFO_0004468	glucose measurement	glucose measurement	EFO_0004468	Other measurement	Metabolic
0.8227	1.08E-113	2.31E-110	PGS000888	EFO_0004611	low density lipoprotein cholesterol measurement	low density lipoprotein cholesterol measurement	EFO_0004611	Cardiovascular measurement	Cardiovascular	PGS003033	EFO_0004611	low density lipoprotein cholesterol measurement	measurement	EFO_0004611	Cardiovascular measurement	Cardiovascular
0.8227	1.08E-113	2.31E-110	PGS000888	EFO_0004611	low density lipoprotein cholesterol measurement	low density lipoprotein cholesterol measurement	EFO_0004611	Lipid or lipoprotein measurement	Metabolic	PGS003033	EFO_0004611	low density lipoprotein cholesterol measurement	measurement	EFO_0004611	Cardiovascular measurement	Cardiovascular
0.8227	1.08E-113	2.31E-110	PGS000888	EFO_0004611	low density lipoprotein cholesterol measurement	low density lipoprotein cholesterol measurement	EFO_0004611	Cardiovascular measurement	Cardiovascular	PGS003033	EFO_0004611	low density lipoprotein cholesterol measurement	measurement	EFO_0004611	Lipid or lipoprotein measurement	Metabolic
0.8227	1.08E-113	2.31E-110	PGS000888	EFO_0004611	low density lipoprotein cholesterol measurement	low density lipoprotein cholesterol measurement	EFO_0004611	Lipid or lipoprotein measurement	Metabolic	PGS003033	EFO_0004611	low density lipoprotein cholesterol measurement	measurement	EFO_0004611	Lipid or lipoprotein measurement	Metabolic
0.8227	1.18E-113	2.53E-110	PGS000478	EFO_0000305	breast carcinoma	breast carcinoma	EFO_0000305	Cancer	Neoplasms	PGS000513	EFO_0000305	breast carcinoma	breast carcinoma	EFO_0000305	Cancer	Neoplasms
0.8226	1.30E-113	2.77E-110	PGS000931	MONDO_0000831	thrombotic disease|deep vein thrombosis	thrombotic disease	MONDO_0000831	Cardiovascular disease	Cardiovascular	PGS000961	EFO_1001395	Phlebitis|Thrombophlebitis	Phlebitis	EFO_1001395	Cardiovascular disease	Cardiovascular
-0.8226	1.32E-113	2.82E-110	PGS000884	EFO_0005208	glomerular filtration rate	glomerular filtration rate	EFO_0005208	Other measurement	Genitourinary	PGS003377	EFO_0005208	glomerular filtration rate	glomerular filtration rate	EFO_0005208	Other measurement	Genitourinary
0.8224	1.52E-113	3.23E-110	PGS002161	EFO_0004340	body mass index	body mass index	EFO_0004340	Body measurement	Body measurement	PGS002133	EFO_0007800	body fat percentage	body fat percentage	EFO_0007800	Body measurement	Body measurement
0.8223	1.75E-113	3.72E-110	PGS003313	EFO_0004617	cystatin C measurement|glomerular filtration rate	cystatin C measurement	EFO_0004617	Other measurement	Metabolic	PGS003314	EFO_0004617	cystatin C measurement|glomerular filtration rate	cystatin C measurement	EFO_0004617	Other measurement	Metabolic
0.8223	1.76E-113	3.75E-110	PGS003145	EFO_0004530	triglyceride measurement	triglyceride measurement	EFO_0004530	Lipid or lipoprotein measurement	Metabolic	PGS003151	EFO_0004530	triglyceride measurement	triglyceride measurement	EFO_0004530	Lipid or lipoprotein measurement	Metabolic
0.8222	1.90E-113	4.03E-110	PGS001227	EFO_0004342	waist circumference	waist circumference	EFO_0004342	Body measurement	Body measurement	PGS001148	EFO_0005409	fat body mass	fat body mass	EFO_0005409	Other measurement	Body measurement
0.8222	1.95E-113	4.14E-110	PGS001453	EFO_0006930	brain volume measurement|neuroimaging measurement	brain volume measurement	EFO_0006930	Other measurement	Neurological	PGS001452	EFO_0006930	measurement	brain volume measurement	EFO_0006930	Other measurement	Neurological
0.8222	2.01E-113	4.26E-110	PGS000694	EFO_0004696	sex hormone-binding globulin measurement	sex hormone-binding globulin measurement	EFO_0004696	Other measurement	Metabolic	PGS002195	EFO_0004696	sex hormone-binding globulin measurement	measurement	EFO_0004696	Other measurement	Metabolic
0.8221	2.17E-113	4.62E-110	PGS002313	EFO_0004340	body mass index	body mass index	EFO_0004340	Body measurement	Body measurement	PGS002855	EFO_0004340	body mass index	body mass index	EFO_0004340	Body measurement	Body measurement
0.8221	2.24E-113	4.77E-110	PGS002386	EFO_0007825	balding measurement	balding measurement	EFO_0007825	Other measurement	Activities	PGS002582	EFO_0007825	balding measurement	balding measurement	EFO_0007825	Other measurement	Activities
0.8221	2.30E-113	4.88E-110	PGS001820	HP_0001943	Hypoglycemia	Hypoglycemia	HP_0001943	Other trait	Metabolic	PGS002025	MONDO_0005147	type 1 diabetes mellitus	type 1 diabetes mellitus	MONDO_0005147	Immune system disorder	Immunological
0.8221	2.30E-113	4.88E-110	PGS001820	HP_0001943	Hypoglycemia	Hypoglycemia	HP_0001943	Other trait	Metabolic	PGS002025	MONDO_0005147	type 1 diabetes mellitus	type 1 diabetes mellitus	MONDO_0005147	Metabolic disorder	Metabolic
0.8221	2.30E-113	4.88E-110	PGS001820	HP_0001943	Hypoglycemia	Hypoglycemia	HP_0001943	Other trait	Metabolic	PGS002025	MONDO_0005147	type 1 diabetes mellitus	type 1 diabetes mellitus	MONDO_0005147	Digestive system disorder	Gastrointestinal
0.8221	2.38E-113	5.05E-110	PGS002349	EFO_0006335	systolic blood pressure	systolic blood pressure	EFO_0006335	Other measurement	Cardiovascular	PGS002807	EFO_0006335	systolic blood pressure	systolic blood pressure	EFO_0006335	Other measurement	Cardiovascular
0.8218	3.02E-113	6.40E-110	PGS000310	EFO_0004611	low density lipoprotein cholesterol measurement	low density lipoprotein cholesterol measurement	EFO_0004611	Lipid or lipoprotein measurement	Metabolic	PGS000311	EFO_0004574	total cholesterol measurement	total cholesterol measurement	EFO_0004574	Lipid or lipoprotein measurement	Metabolic
0.8218	3.02E-113	6.40E-110	PGS000310	EFO_0004611	low density lipoprotein cholesterol measurement	low density lipoprotein cholesterol measurement	EFO_0004611	Cardiovascular measurement	Cardiovascular	PGS000311	EFO_0004574	total cholesterol measurement	total cholesterol measurement	EFO_0004574	Lipid or lipoprotein measurement	Metabolic
0.8218	3.06E-113	6.50E-110	PGS002201	EFO_0004342	waist circumference	waist circumference	EFO_0004342	Body measurement	Body measurement	PGS002133	EFO_0007800	body fat percentage	body fat percentage	EFO_0007800	Body measurement	Body measurement
0.8218	3.25E-113	6.88E-110	PGS000404	EFO_0009259	skin carcinoma	skin carcinoma	EFO_0009259	Cancer	Neoplasms	PGS000443	MONDO_0002898	skin cancer	skin cancer	MONDO_0002898	Cancer	Neoplasms
0.8217	3.58E-113	7.58E-110	PGS002876	EFO_0004458	C-reactive protein measurement	C-reactive protein measurement	EFO_0004458	Inflammatory measurement	Immunological	PGS002886	EFO_0004458	C-reactive protein measurement	C-reactive protein measurement	EFO_0004458	Inflammatory measurement	Immunological
0.8217	3.58E-113	7.58E-110	PGS002875	EFO_0004458	C-reactive protein measurement	C-reactive protein measurement	EFO_0004458	Inflammatory measurement	Immunological	PGS002885	EFO_0004458	C-reactive protein measurement	C-reactive protein measurement	EFO_0004458	Inflammatory measurement	Immunological
0.8217	3.58E-113	7.58E-110	PGS002886	EFO_0004458	C-reactive protein measurement	C-reactive protein measurement	EFO_0004458	Inflammatory measurement	Immunological	PGS002875	EFO_0004458	C-reactive protein measurement	C-reactive protein measurement	EFO_0004458	Inflammatory measurement	Immunological
0.8217	3.58E-113	7.58E-110	PGS002876	EFO_0004458	C-reactive protein measurement	C-reactive protein measurement	EFO_0004458	Inflammatory measurement	Immunological	PGS002885	EFO_0004458	C-reactive protein measurement	C-reactive protein measurement	EFO_0004458	Inflammatory measurement	Immunological
0.8217	3.70E-113	7.82E-110	PGS003031	EFO_0004611	low density lipoprotein cholesterol measurement	low density lipoprotein cholesterol measurement	EFO_0004611	Cardiovascular measurement	Cardiovascular	PGS003136	EFO_0004574	total cholesterol measurement	total cholesterol measurement	EFO_0004574	Lipid or lipoprotein measurement	Metabolic
0.8217	3.70E-113	7.82E-110	PGS003031	EFO_0004611	low density lipoprotein cholesterol measurement	low density lipoprotein cholesterol measurement	EFO_0004611	Lipid or lipoprotein measurement	Metabolic	PGS003136	EFO_0004574	total cholesterol measurement	total cholesterol measurement	EFO_0004574	Lipid or lipoprotein measurement	Metabolic
0.8217	3.78E-113	7.99E-110	PGS002873	EFO_0004458	C-reactive protein measurement	C-reactive protein measurement	EFO_0004458	Inflammatory measurement	Immunological	PGS002868	EFO_0004458	C-reactive protein measurement	C-reactive protein measurement	EFO_0004458	Inflammatory measurement	Immunological
0.8216	3.96E-113	8.36E-110	PGS001248	EFO_0004274	gout	gout	EFO_0004274	Metabolic disorder	Metabolic	PGS001249	EFO_0004274	gout	gout	EFO_0004274	Metabolic disorder	Metabolic
0.8216	4.21E-113	8.89E-110	PGS003069	EFO_0006335	systolic blood pressure	systolic blood pressure	EFO_0006335	Other measurement	Cardiovascular	PGS000913	EFO_0006335	systolic blood pressure	systolic blood pressure	EFO_0006335	Other measurement	Cardiovascular
0.8215	4.51E-113	9.51E-110	PGS001278	EFO_0003827	pulmonary embolism|deep vein thrombosis	pulmonary embolism	EFO_0003827	Cardiovascular disease	Cardiovascular	PGS000930	MONDO_0000831	thrombotic disease	thrombotic disease	MONDO_0000831	Cardiovascular disease	Cardiovascular
0.8214	4.86E-113	1.03E-109	PGS001726	EFO_0006930	brain volume measurement|neuroimaging measurement	brain volume measurement	EFO_0006930	Other measurement	Neurological	PGS001740	EFO_0006930	measurement	brain volume measurement	EFO_0006930	Other measurement	Neurological
0.8214	5.19E-113	1.09E-109	PGS001819	EFO_0003770	diabetic retinopathy	diabetic retinopathy	EFO_0003770	Metabolic disorder	Metabolic	PGS002028	HP_0001943	Hypoglycemia	Hypoglycemia	HP_0001943	Other trait	Metabolic
0.8214	5.19E-113	1.09E-109	PGS001819	EFO_0003770	diabetic retinopathy	diabetic retinopathy	EFO_0003770	Neurological disorder	Neurological	PGS002028	HP_0001943	Hypoglycemia	Hypoglycemia	HP_0001943	Other trait	Metabolic
0.8214	5.19E-113	1.09E-109	PGS001819	EFO_0003770	diabetic retinopathy	diabetic retinopathy	EFO_0003770	Cardiovascular disease	Cardiovascular	PGS002028	HP_0001943	Hypoglycemia	Hypoglycemia	HP_0001943	Other trait	Metabolic
0.8214	5.19E-113	1.09E-109	PGS003244	EFO_0010472	creatine measurement	creatine measurement	EFO_0010472	Other measurement	Metabolic	PGS003248	EFO_0010472	creatine measurement	creatine measurement	EFO_0010472	Other measurement	Metabolic
0.8213	5.47E-113	1.15E-109	PGS000312	EFO_0004530	triglyceride measurement	triglyceride measurement	EFO_0004530	Lipid or lipoprotein measurement	Metabolic	PGS003401	EFO_0004530	triglyceride measurement	triglyceride measurement	EFO_0004530	Lipid or lipoprotein measurement	Metabolic
0.8213	5.67E-113	1.19E-109	PGS001402	EFO_0009270	heel bone mineral density	heel bone mineral density	EFO_0009270	Other measurement	Skeletal	PGS002175	EFO_0005654	velocity of sound measurement	velocity of sound measurement	EFO_0005654	Other measurement	Others
0.8213	5.72E-113	1.20E-109	PGS002199	EFO_0007928	ventricular rate measurement	ventricular rate measurement	EFO_0007928	Cardiovascular measurement	Cardiovascular	PGS001981	EFO_0007928	ventricular rate measurement	ventricular rate measurement	EFO_0007928	Cardiovascular measurement	Cardiovascular
0.8213	5.74E-113	1.21E-109	PGS001228	EFO_0004340	body mass index	body mass index	EFO_0004340	Body measurement	Body measurement	PGS001227	EFO_0004342	waist circumference	waist circumference	EFO_0004342	Body measurement	Body measurement
0.8212	6.59E-113	1.39E-109	PGS000913	EFO_0006335	systolic blood pressure	systolic blood pressure	EFO_0006335	Other measurement	Cardiovascular	PGS002349	EFO_0006335	systolic blood pressure	systolic blood pressure	EFO_0006335	Other measurement	Cardiovascular
0.8211	7.19E-113	1.51E-109	PGS002161	EFO_0004340	body mass index	body mass index	EFO_0004340	Body measurement	Body measurement	PGS000921	EFO_0004340	body mass index	body mass index	EFO_0004340	Body measurement	Body measurement
0.821	7.65E-113	1.61E-109	PGS002201	EFO_0004342	waist circumference	waist circumference	EFO_0004342	Body measurement	Body measurement	PGS002176	EFO_0005093	hip circumference	hip circumference	EFO_0005093	Body measurement	Body measurement
0.821	7.82E-113	1.64E-109	PGS000412	EFO_0000756	melanoma	melanoma	EFO_0000756	Cancer	Neoplasms	PGS000416	EFO_0000756	melanoma	melanoma	EFO_0000756	Cancer	Neoplasms
0.821	7.82E-113	1.64E-109	PGS000418	EFO_0000756	melanoma	melanoma	EFO_0000756	Cancer	Neoplasms	PGS000412	EFO_0000756	melanoma	melanoma	EFO_0000756	Cancer	Neoplasms
0.8209	8.60E-113	1.80E-109	PGS002442	EFO_0000400	diabetes mellitus	diabetes mellitus	EFO_0000400	Metabolic disorder	Metabolic	PGS002449	EFO_0004468	glucose measurement	glucose measurement	EFO_0004468	Other measurement	Metabolic
0.8209	8.60E-113	1.80E-109	PGS002442	EFO_0000400	diabetes mellitus	diabetes mellitus	EFO_0000400	Digestive system disorder	Gastrointestinal	PGS002449	EFO_0004468	glucose measurement	glucose measurement	EFO_0004468	Other measurement	Metabolic
0.8207	1.07E-112	2.23E-109	PGS001817	MONDO_0005147	type 1 diabetes mellitus	type 1 diabetes mellitus	MONDO_0005147	Immune system disorder	Immunological	PGS001117	EFO_0600082	insulin use measurement	insulin use measurement	EFO_0600082	Other measurement	Metabolic
0.8207	1.07E-112	2.23E-109	PGS001817	MONDO_0005147	type 1 diabetes mellitus	type 1 diabetes mellitus	MONDO_0005147	Metabolic disorder	Metabolic	PGS001117	EFO_0600082	insulin use measurement	insulin use measurement	EFO_0600082	Other measurement	Metabolic
0.8207	1.07E-112	2.23E-109	PGS001817	MONDO_0005147	type 1 diabetes mellitus	type 1 diabetes mellitus	MONDO_0005147	Digestive system disorder	Gastrointestinal	PGS001117	EFO_0600082	insulin use measurement	insulin use measurement	EFO_0600082	Other measurement	Metabolic
0.8207	1.08E-112	2.27E-109	PGS002584	EFO_0000319	cardiovascular disease	cardiovascular disease	EFO_0000319	Cardiovascular disease	Cardiovascular	PGS002603	EFO_0000537	hypertension	hypertension	EFO_0000537	Cardiovascular disease	Cardiovascular
0.8207	1.10E-112	2.31E-109	PGS001674	EFO_0006930	brain volume measurement|neuroimaging measurement	brain volume measurement	EFO_0006930	Other measurement	Neurological	PGS001681	EFO_0006930	measurement	brain volume measurement	EFO_0006930	Other measurement	Neurological
0.8207	1.12E-112	2.35E-109	PGS002253	EFO_0000756	melanoma	melanoma	EFO_0000756	Cancer	Neoplasms	PGS000744	EFO_0000756	melanoma	melanoma	EFO_0000756	Cancer	Neoplasms
0.8207	1.14E-112	2.39E-109	PGS000952	EFO_0004514	bone quantitative ultrasound measurement	bone quantitative ultrasound measurement	EFO_0004514	Other measurement	Skeletal	PGS002175	EFO_0005654	velocity of sound measurement	velocity of sound measurement	EFO_0005654	Other measurement	Others
0.8207	1.15E-112	2.42E-109	PGS000688	EFO_0004611	low density lipoprotein cholesterol measurement	low density lipoprotein cholesterol measurement	EFO_0004611	Lipid or lipoprotein measurement	Metabolic	PGS001933	EFO_0004611	low density lipoprotein cholesterol measurement	measurement	EFO_0004611	Cardiovascular measurement	Cardiovascular
0.8207	1.15E-112	2.42E-109	PGS000688	EFO_0004611	low density lipoprotein cholesterol measurement	low density lipoprotein cholesterol measurement	EFO_0004611	Cardiovascular measurement	Cardiovascular	PGS001933	EFO_0004611	low density lipoprotein cholesterol measurement	measurement	EFO_0004611	Lipid or lipoprotein measurement	Metabolic
0.8207	1.15E-112	2.42E-109	PGS000688	EFO_0004611	low density lipoprotein cholesterol measurement	low density lipoprotein cholesterol measurement	EFO_0004611	Cardiovascular measurement	Cardiovascular	PGS001933	EFO_0004611	low density lipoprotein cholesterol measurement	measurement	EFO_0004611	Cardiovascular measurement	Cardiovascular
0.8207	1.15E-112	2.42E-109	PGS000688	EFO_0004611	low density lipoprotein cholesterol measurement	low density lipoprotein cholesterol measurement	EFO_0004611	Lipid or lipoprotein measurement	Metabolic	PGS001933	EFO_0004611	low density lipoprotein cholesterol measurement	measurement	EFO_0004611	Lipid or lipoprotein measurement	Metabolic
0.8207	1.16E-112	2.43E-109	PGS001037	EFO_0009270	heel bone mineral density	heel bone mineral density	EFO_0009270	Other measurement	Skeletal	PGS002175	EFO_0005654	velocity of sound measurement	velocity of sound measurement	EFO_0005654	Other measurement	Others
0.8207	1.17E-112	2.44E-109	PGS002170	EFO_0004468	glucose measurement	glucose measurement	EFO_0004468	Other measurement	Metabolic	PGS002328	EFO_0004468	glucose measurement	glucose measurement	EFO_0004468	Other measurement	Metabolic
0.8206	1.20E-112	2.51E-109	PGS002314	EFO_0007825	balding measurement	balding measurement	EFO_0007825	Other measurement	Activities	PGS002631	EFO_0007825	balding measurement	balding measurement	EFO_0007825	Other measurement	Activities
0.8206	1.30E-112	2.71E-109	PGS000931	MONDO_0000831	thrombotic disease|deep vein thrombosis	thrombotic disease	MONDO_0000831	Cardiovascular disease	Cardiovascular	PGS001277	EFO_0003827	pulmonary embolism|deep vein thrombosis	pulmonary embolism	EFO_0003827	Cardiovascular disease	Cardiovascular
0.8205	1.44E-112	3.00E-109	PGS001320	EFO_0000537	hypertension	hypertension	EFO_0000537	Cardiovascular disease	Cardiovascular	PGS001335	EFO_0000319	cardiovascular disease	cardiovascular disease	EFO_0000319	Cardiovascular disease	Cardiovascular
0.8205	1.49E-112	3.10E-109	PGS001103	EFO_0007800	body fat percentage	body fat percentage	EFO_0007800	Body measurement	Body measurement	PGS001106	EFO_0007800	body fat percentage	body fat percentage	EFO_0007800	Body measurement	Body measurement
0.8205	1.50E-112	3.13E-109	PGS002352	EFO_0004574	total cholesterol measurement	total cholesterol measurement	EFO_0004574	Lipid or lipoprotein measurement	Metabolic	PGS003138	EFO_0004574	total cholesterol measurement	total cholesterol measurement	EFO_0004574	Lipid or lipoprotein measurement	Metabolic
0.8204	1.66E-112	3.47E-109	PGS001661	EFO_0006930	brain volume measurement|neuroimaging measurement	brain volume measurement	EFO_0006930	Other measurement	Neurological	PGS001662	EFO_0006930	measurement	brain volume measurement	EFO_0006930	Other measurement	Neurological
0.8203	1.76E-112	3.67E-109	PGS002856	EFO_0004340	body mass index	body mass index	EFO_0004340	Body measurement	Body measurement	PGS002847	EFO_0004340	body mass index	body mass index	EFO_0004340	Body measurement	Body measurement
0.8202	1.92E-112	4.00E-109	PGS001737	EFO_0006930	brain volume measurement|neuroimaging measurement	brain volume measurement	EFO_0006930	Other measurement	Neurological	PGS001738	EFO_0006930	measurement	brain volume measurement	EFO_0006930	Other measurement	Neurological

0.8202	1.99E-112	4.14E-109	PGS002889	EFO_0006336	diastolic blood pressure	diastolic blood pressure	EFO_0006336	Other measurement	Cardiovascular	PGS000912	EFO_0006336	diastolic blood pressure	diastolic blood pressure	EFO_0006336	Other measurement	Cardiovascular
0.82	2.44E-112	5.08E-109	PGS001573	EFO_0005420	measurement	grey matter volume measurement	EFO_0005420	Other measurement	Neurological	PGS001572	EFO_0005420	measurement	grey matter volume measurement	EFO_0005420	Other measurement	Neurological
0.82	2.51E-112	5.23E-109	PGS003009	EFO_0000537	hypertension	hypertension	EFO_0000537	Cardiovascular disease	Cardiovascular	PGS003025	EFO_0000537	hypertension	hypertension	EFO_0000537	Cardiovascular disease	Cardiovascular
0.82	2.51E-112	5.23E-109	PGS003009	EFO_0000537	hypertension	hypertension	EFO_0000537	Cardiovascular disease	Cardiovascular	PGS003026	EFO_0000537	hypertension	hypertension	EFO_0000537	Cardiovascular disease	Cardiovascular
0.8199	2.69E-112	5.60E-109	PGS001360	EFO_0004731	eye measurement	eye measurement	EFO_0004731	Other measurement	Ophthalmological	PGS001368	EFO_0004731	eye measurement	eye measurement	EFO_0004731	Other measurement	Ophthalmological
0.8199	2.77E-112	5.76E-109	PGS000569	EFO_0001663	prostate carcinoma	prostate carcinoma	EFO_0001663	Cancer	Neoplasms	PGS000594	EFO_0007355	male reproductive organ cancer	male reproductive organ cancer	EFO_0007355	Cancer	Neoplasms
0.8196	3.85E-112	7.99E-109	PGS000463	EFO_0000707	squamous cell carcinoma	squamous cell carcinoma	EFO_0000707	Cancer	Neoplasms	PGS000466	EFO_0000707	squamous cell carcinoma	squamous cell carcinoma	EFO_0000707	Cancer	Neoplasms
0.8196	3.85E-112	7.99E-109	PGS000463	EFO_0000707	squamous cell carcinoma	squamous cell carcinoma	EFO_0000707	Cancer	Neoplasms	PGS000465	EFO_0000707	squamous cell carcinoma	squamous cell carcinoma	EFO_0000707	Cancer	Neoplasms
0.8195	4.28E-112	8.89E-109	PGS001956	EFO_0007772	calcaneal bone quantitative ultrasound measurement	calcaneal bone quantitative ultrasound measurement	EFO_0007772	Other measurement	Skeletal	PGS000951	EFO_0004514	bone quantitative ultrasound measurement	measurement	EFO_0004514	Other measurement	Skeletal
0.8195	4.41E-112	9.15E-109	PGS002846	EFO_0004340	body mass index	body mass index	EFO_0004340	Body measurement	Body measurement	PGS002852	EFO_0004340	body mass index	body mass index	EFO_0004340	Body measurement	Body measurement
0.8195	4.53E-112	9.39E-109	PGS001817	MONDO_0005147	type 1 diabetes mellitus	type 1 diabetes mellitus	MONDO_0005147	Immune system disorder	Immunological	PGS002028	HP_0001943	Hypoglycemia	Hypoglycemia	HP_0001943	Other trait	Metabolic
0.8195	4.53E-112	9.39E-109	PGS001817	MONDO_0005147	type 1 diabetes mellitus	type 1 diabetes mellitus	MONDO_0005147	Digestive system disorder	Gastrointestinal	PGS002028	HP_0001943	Hypoglycemia	Hypoglycemia	HP_0001943	Other trait	Metabolic
0.8195	4.53E-112	9.39E-109	PGS001817	MONDO_0005147	type 1 diabetes mellitus	type 1 diabetes mellitus	MONDO_0005147	Metabolic disorder	Metabolic	PGS002028	HP_0001943	Hypoglycemia	Hypoglycemia	HP_0001943	Other trait	Metabolic
0.8194	4.81E-112	9.96E-109	PGS002605	EFO_0004611	low density lipoprotein cholesterol measurement	low density lipoprotein cholesterol measurement	EFO_0004611	Cardiovascular measurement	Cardiovascular	PGS002424	EFO_0004574	total cholesterol measurement	total cholesterol measurement	EFO_0004574	Lipid or lipoprotein measurement	Metabolic
0.8194	4.81E-112	9.96E-109	PGS002605	EFO_0004611	low density lipoprotein cholesterol measurement	low density lipoprotein cholesterol measurement	EFO_0004611	Lipid or lipoprotein measurement	Metabolic	PGS002424	EFO_0004574	total cholesterol measurement	total cholesterol measurement	EFO_0004574	Lipid or lipoprotein measurement	Metabolic
0.8194	5.03E-112	1.04E-108	PGS003069	EFO_0006335	systolic blood pressure	systolic blood pressure	EFO_0006335	Other measurement	Cardiovascular	PGS003078	EFO_0006335	systolic blood pressure	systolic blood pressure	EFO_0006335	Other measurement	Cardiovascular
0.8193	5.42E-112	1.12E-108	PGS001933	EFO_0004611	low density lipoprotein cholesterol measurement	low density lipoprotein cholesterol measurement	EFO_0004611	Lipid or lipoprotein measurement	Metabolic	PGS002654	EFO_0004611	low density lipoprotein cholesterol measurement	measurement	EFO_0004611	Cardiovascular measurement	Cardiovascular
0.8193	5.42E-112	1.12E-108	PGS001933	EFO_0004611	low density lipoprotein cholesterol measurement	low density lipoprotein cholesterol measurement	EFO_0004611	Cardiovascular measurement	Cardiovascular	PGS002654	EFO_0004611	low density lipoprotein cholesterol measurement	measurement	EFO_0004611	Lipid or lipoprotein measurement	Metabolic
0.8193	5.42E-112	1.12E-108	PGS001933	EFO_0004611	low density lipoprotein cholesterol measurement	low density lipoprotein cholesterol measurement	EFO_0004611	Lipid or lipoprotein measurement	Metabolic	PGS002654	EFO_0004611	low density lipoprotein cholesterol measurement	measurement	EFO_0004611	Lipid or lipoprotein measurement	Metabolic
0.8193	5.42E-112	1.12E-108	PGS001933	EFO_0004611	low density lipoprotein cholesterol measurement	low density lipoprotein cholesterol measurement	EFO_0004611	Cardiovascular measurement	Cardiovascular	PGS002654	EFO_0004611	low density lipoprotein cholesterol measurement	measurement	EFO_0004611	Cardiovascular measurement	Cardiovascular
0.8193	5.60E-112	1.16E-108	PGS001637	EFO_0006935	thalamus volume|neuroimaging measurement	thalamus volume	EFO_0006935	Other measurement	Neurological	PGS001638	EFO_0006935	thalamus volume|neuroimaging measurement	thalamus volume	EFO_0006935	Other measurement	Neurological
0.8192	6.05E-112	1.25E-108	PGS002550	EFO_0004541	HbA1c measurement	HbA1c measurement	EFO_0004541	Hematological measurement	Hematological	PGS002540	EFO_0000400	diabetes mellitus	diabetes mellitus	EFO_0000400	Metabolic disorder	Metabolic
0.8192	6.05E-112	1.25E-108	PGS002550	EFO_0004541	HbA1c measurement	HbA1c measurement	EFO_0004541	Hematological measurement	Hematological	PGS002540	EFO_0000400	diabetes mellitus	diabetes mellitus	EFO_0000400	Digestive system disorder	Gastrointestinal
0.819	8.25E-112	1.71E-108	PGS001230	EFO_0004338	body weight	body weight	EFO_0004338	Body measurement	Body measurement	PGS001165	EFO_0004995	lean body mass	lean body mass	EFO_0004995	Other measurement	Skeletal
0.8189	8.56E-112	1.77E-108	PGS001229	EFO_0004339	body height	body height	EFO_0004339	Body measurement	Body measurement	PGS002973	EFO_0004339	body height	body height	EFO_0004339	Body measurement	Body measurement
0.8188	9.29E-112	1.92E-108	PGS001819	EFO_0003770	diabetic retinopathy	diabetic retinopathy	EFO_0003770	Neurological disorder	Neurological	PGS002025	MONDO_0005147	type 1 diabetes mellitus	type 1 diabetes mellitus	MONDO_0005147	Metabolic disorder	Metabolic
0.8188	9.29E-112	1.92E-108	PGS001819	EFO_0003770	diabetic retinopathy	diabetic retinopathy	EFO_0003770	Metabolic disorder	Metabolic	PGS002025	MONDO_0005147	type 1 diabetes mellitus	type 1 diabetes mellitus	MONDO_0005147	Metabolic disorder	Metabolic
0.8188	9.29E-112	1.92E-108	PGS001819	EFO_0003770	diabetic retinopathy	diabetic retinopathy	EFO_0003770	Cardiovascular disease	Cardiovascular	PGS002025	MONDO_0005147	type 1 diabetes mellitus	type 1 diabetes mellitus	MONDO_0005147	Digestive system disorder	Gastrointestinal
0.8188	9.29E-112	1.92E-108	PGS001819	EFO_0003770	diabetic retinopathy	diabetic retinopathy	EFO_0003770	Metabolic disorder	Metabolic	PGS002025	MONDO_0005147	type 1 diabetes mellitus	type 1 diabetes mellitus	MONDO_0005147	Immune system disorder	Immunological
0.8188	9.29E-112	1.92E-108	PGS001819	EFO_0003770	diabetic retinopathy	diabetic retinopathy	EFO_0003770	Neurological disorder	Neurological	PGS002025	MONDO_0005147	type 1 diabetes mellitus	type 1 diabetes mellitus	MONDO_0005147	Digestive system disorder	Gastrointestinal
0.8188	9.29E-112	1.92E-108	PGS001819	EFO_0003770	diabetic retinopathy	diabetic retinopathy	EFO_0003770	Cardiovascular disease	Cardiovascular	PGS002025	MONDO_0005147	type 1 diabetes mellitus	type 1 diabetes mellitus	MONDO_0005147	Immune system disorder	Immunological
0.8188	9.29E-112	1.92E-108	PGS001819	EFO_0003770	diabetic retinopathy	diabetic retinopathy	EFO_0003770	Neurological disorder	Neurological	PGS002025	MONDO_0005147	type 1 diabetes mellitus	type 1 diabetes mellitus	MONDO_0005147	Immune system disorder	Immunological
0.8188	9.29E-112	1.92E-108	PGS001819	EFO_0003770	diabetic retinopathy	diabetic retinopathy	EFO_0003770	Metabolic disorder	Metabolic	PGS002025	MONDO_0005147	type 1 diabetes mellitus	type 1 diabetes mellitus	MONDO_0005147	Digestive system disorder	Gastrointestinal
0.8188	9.29E-112	1.92E-108	PGS001819	EFO_0003770	diabetic retinopathy	diabetic retinopathy	EFO_0003770	Cardiovascular disease	Cardiovascular	PGS002025	MONDO_0005147	type 1 diabetes mellitus	type 1 diabetes mellitus	MONDO_0005147	Metabolic disorder	Metabolic
0.8188	9.50E-112	1.96E-108	PGS001990	EFO_0004526	mean corpuscular volume	mean corpuscular volume	EFO_0004526	Hematological measurement	Hematological	PGS000101	EFO_0004526	mean corpuscular volume	mean corpuscular volume	EFO_0004526	Hematological measurement	Hematological
0.8187	1.06E-111	2.18E-108	PGS001531	EFO_0007772	calcaneal bone quantitative ultrasound measurement	calcaneal bone quantitative ultrasound measurement	EFO_0007772	Other measurement	Skeletal	PGS000953	EFO_0004514	bone quantitative ultrasound measurement	measurement	EFO_0004514	Other measurement	Skeletal
0.8187	1.10E-111	2.27E-108	PGS003118	MONDO_0005148	type 2 diabetes mellitus	type 2 diabetes mellitus	MONDO_0005148	Digestive system disorder	Gastrointestinal	PGS002771	MONDO_0005148	type 2 diabetes mellitus	type 2 diabetes mellitus	MONDO_0005148	Digestive system disorder	Gastrointestinal
0.8187	1.10E-111	2.27E-108	PGS003118	MONDO_0005148	type 2 diabetes mellitus	type 2 diabetes mellitus	MONDO_0005148	Metabolic disorder	Metabolic	PGS002771	MONDO_0005148	type 2 diabetes mellitus	type 2 diabetes mellitus	MONDO_0005148	Metabolic disorder	Metabolic
0.8187	1.10E-111	2.27E-108	PGS003118	MONDO_0005148	type 2 diabetes mellitus	type 2 diabetes mellitus	MONDO_0005148	Digestive system disorder	Gastrointestinal	PGS002771	MONDO_0005148	type 2 diabetes mellitus	type 2 diabetes mellitus	MONDO_0005148	Metabolic disorder	Metabolic
0.8187	1.10E-111	2.27E-108	PGS003118	MONDO_0005148	type 2 diabetes mellitus	type 2 diabetes mellitus	MONDO_0005148	Metabolic disorder	Metabolic	PGS002771	MONDO_0005148	type 2 diabetes mellitus	type 2 diabetes mellitus	MONDO_0005148	Digestive system disorder	Gastrointestinal
0.8185	1.44E-111	2.97E-108	PGS002990	EFO_0004339	body height	body height	EFO_0004339	Body measurement	Body measurement	PGS002980	EFO_0004339	body height	body height	EFO_0004339	Body measurement	Body measurement
0.8184	1.48E-111	3.05E-108	PGS002432	MONDO_0004979	asthma	asthma	MONDO_0004979	Other disease	Respiratory	PGS002419	EFO_0000684	respiratory system disease	respiratory system disease	EFO_0000684	Other disease	Skeletal
-0.8184	1.62E-111	3.34E-108	PGS001245	EFO_0004279	suntan	suntan	EFO_0004279	Other trait	Dermatological	PGS001257	EFO_0003958	sunburn	sunburn	EFO_0003958	Other disease	Dermatological
0.8183	1.68E-111	3.46E-108	PGS003110	MONDO_0005148	type 2 diabetes mellitus	type 2 diabetes mellitus	MONDO_0005148	Digestive system disorder	Gastrointestinal	PGS003095	MONDO_0005148	type 2 diabetes mellitus	type 2 diabetes mellitus	MONDO_0005148	Metabolic disorder	Metabolic
0.8183	1.68E-111	3.46E-108	PGS003110	MONDO_0005148	type 2 diabetes mellitus	type 2 diabetes mellitus	MONDO_0005148	Digestive system disorder	Gastrointestinal	PGS003095	MONDO_0005148	type 2 diabetes mellitus	type 2 diabetes mellitus	MONDO_0005148	Digestive system disorder	Gastrointestinal
0.8183	1.68E-111	3.46E-108	PGS003110	MONDO_0005148	type 2 diabetes mellitus	type 2 diabetes mellitus	MONDO_0005148	Metabolic disorder	Metabolic	PGS003095	MONDO_0005148	type 2 diabetes mellitus	type 2 diabetes mellitus	MONDO_0005148	Metabolic disorder	Metabolic
0.8183	1.68E-111	3.46E-108	PGS003110	MONDO_0005148	type 2 diabetes mellitus	type 2 diabetes mellitus	MONDO_0005148	Metabolic disorder	Metabolic	PGS003095	MONDO_0005148	type 2 diabetes mellitus	type 2 diabetes mellitus	MONDO_0005148	Digestive system disorder	Gastrointestinal
0.8182	1.92E-111	3.96E-108	PGS000479	EFO_0000305	breast carcinoma	breast carcinoma	EFO_0000305	Cancer	Neoplasms	PGS001336	EFO_0000305	breast carcinoma	breast carcinoma	EFO_0000305	Cancer	Neoplasms
0.8182	1.93E-111	3.97E-108	PGS001297	MONDO_0005147	type 1 diabetes mellitus	type 1 diabetes mellitus	MONDO_0005147	Immune system disorder	Immunological	PGS001014	EFO_0009605	pancreas disease	pancreas disease	EFO_0009605	Digestive system disorder	Gastrointestinal
0.8182	1.93E-111	3.97E-108	PGS001297	MONDO_0005147	type 1 diabetes mellitus	type 1 diabetes mellitus	MONDO_0005147	Metabolic disorder	Metabolic	PGS001014	EFO_0009605	pancreas disease	pancreas disease	EFO_0009605	Digestive system disorder	Gastrointestinal
0.8182	1.93E-111	3.97E-108	PGS001297	MONDO_0005147	type 1 diabetes mellitus	type 1 diabetes mellitus	MONDO_0005147	Digestive system disorder	Gastrointestinal	PGS001014	EFO_0009605	pancreas disease	pancreas disease	EFO_0009605	Digestive system disorder	Gastrointestinal
0.8182	2.00E-111	4.12E-108	PGS001819	EFO_0003770	diabetic retinopathy	diabetic retinopathy	EFO_0003770	Neurological disorder	Neurological	PGS001817	MONDO_0005147	type 1 diabetes mellitus	type 1 diabetes mellitus	MONDO_0005147	Immune system disorder	Immunological
0.8182	2.00E-111	4.12E-108	PGS001819	EFO_0003770	diabetic retinopathy	diabetic retinopathy	EFO_0003770	Cardiovascular disease	Cardiovascular	PGS001817	MONDO_0005147	type 1 diabetes mellitus	type 1 diabetes mellitus	MONDO_0005147	Digestive system disorder	Gastrointestinal
0.8182	2.00E-111	4.12E-108	PGS001819	EFO_0003770	diabetic retinopathy	diabetic retinopathy	EFO_0003770	Neurological disorder	Neurological	PGS001817	MONDO_0005147	type 1 diabetes mellitus	type 1 diabetes mellitus	MONDO_0005147	Metabolic disorder	Metabolic
0.8182	2.00E-111	4.12E-108	PGS001819	EFO_0003770	diabetic retinopathy	diabetic retinopathy	EFO_0003770	Metabolic disorder	Metabolic	PGS001817	MONDO_0005147	type 1 diabetes mellitus	type 1 diabetes mellitus	MONDO_0005147	Metabolic disorder	Metabolic
0.8182	2.00E-111	4.12E-108	PGS001819	EFO_0003770	diabetic retinopathy	diabetic retinopathy	EFO_0003770	Cardiovascular disease	Cardiovascular	PGS001817	MONDO_0005147	type 1 diabetes mellitus	type 1 diabetes mellitus	MONDO_0005147	Immune system disorder	Immunological
0.8182	2.00E-111	4.12E-108	PGS001819	EFO_0003770	diabetic retinopathy	diabetic retinopathy	EFO_0003770	Cardiovascular disease	Cardiovascular	PGS001817	MONDO_0005147	type 1 diabetes mellitus	type 1 diabetes mellitus	MONDO_0005147	Metabolic disorder	Metabolic
0.8182	2.00E-111	4.12E-108	PGS001819	EFO_0003770	diabetic retinopathy	diabetic retinopathy	EFO_0003770	Neurological disorder	Neurological	PGS001817	MONDO_0005147	type 1 diabetes mellitus	type 1 diabetes mellitus	MONDO_0005147	Digestive system disorder	Gastrointestinal
0.8182	2.00E-111	4.12E-108	PGS001819	EFO_0003770	diabetic retinopathy	diabetic retinopathy	EFO_0003770	Metabolic disorder	Metabolic	PGS001817	MONDO_0005147	type 1 diabetes mellitus	type 1 diabetes mellitus	MONDO_0005147	Immune system disorder	Immunological
0.8182	2.00E-111	4.12E-108	PGS001819	EFO_0003770	diabetic retinopathy	diabetic retinopathy	EFO_0003770	Metabolic disorder	Metabolic	PGS001817	MONDO_0005147	type 1 diabetes mellitus	type 1 diabetes mellitus	MONDO_0005147	Digestive system disorder	Gastrointestinal
0.818	2.33E-111	4.80E-108	PGS000589	EFO_0001663	prostate carcinoma	prostate carcinoma	EFO_0001663	Cancer	Neoplasms	PGS000573	EFO_0001663	prostate carcinoma	prostate carcinoma	EFO_0001663	Cancer	Neoplasms
0.818	2.38E-111	4.90E-108	PGS001952	EFO_0004468	glucose measurement	glucose measurement	EFO_0004468	Other measurement	Metabolic	PGS002328	EFO_0004468	glucose measurement	glucose measurement	EFO_0004468	Other measurement	Metabolic
0.8179	2.76E-111	5.68E-108	PGS002108	EFO_0004574	total cholesterol measurement	total cholesterol measurement	EFO_0004574	Lipid or lipoprotein measurement	Metabolic	PGS002102	EFO_0004615	apolipoprotein B measurement	apolipoprotein B measurement	EFO_0004615	Lipid or lipoprotein measurement	Metabolic
0.8176	3.92E-111	8.06E-108	PGS003311	EFO_0004617	cystatin C measurement|glomerular filtration rate	cystatin C measurement	EFO_0004617	Other measurement	Metabolic	PGS003314	EFO_0004617	cystatin C measurement|glomerular filtration rate	cystatin C measurement	EFO_0004617	Other measurement	Metabolic
0.8173	5.10E-111	1.05E-107	PGS003274	EFO_0010472	creatine measurement|glomerular filtration rate	creatine measurement	EFO_0010472	Other measurement	Metabolic	PGS003263	EFO_0010472	creatine measurement|glomerular filtration rate	creatine measurement	EFO_0010472	Other measurement	Metabolic
0.8173	5.50E-111	1.13E-107	PGS002491	EFO_0000400	diabetes mellitus	diabetes mellitus	EFO_0000400	Metabolic disorder	Metabolic	PGS002573	MONDO_0005148	type 2 diabetes mellitus	type 2 diabetes mellitus	MONDO_0005148	Metabolic disorder	Metabolic
0.8173	5.50E-111	1.13E-107	PGS002491	EFO_0000400	diabetes mellitus	diabetes mellitus	EFO_0000400	Digestive system disorder	Gastrointestinal	PGS002573	MONDO_0005148	type 2 diabetes mellitus	type 2 diabetes mellitus	MONDO_0005148	Digestive system disorder	Gastrointestinal
0.8173	5.50E-111	1.13E-107	PGS002491	EFO_0000400	diabetes mellitus	diabetes mellitus	EFO_0000400	Metabolic disorder	Metabolic	PGS002573	MONDO_0005148	type 2 diabetes mellitus	type 2 diabetes mellitus	MONDO_0005148	Digestive system disorder	Gastrointestinal
0.8173	5.50E-111	1.13E-107	PGS002491	EFO_0000400	diabetes mellitus	diabetes mellitus	EFO_0000400	Digestive system disorder	Gastrointestinal	PGS002573	MONDO_0005148	type 2 diabetes mellitus	type 2 diabetes mellitus	MONDO_0005148	Metabolic disorder	Metabolic
0.8172	5.73E-111	1.18E-107	PGS003139	EFO_0004574	total cholesterol measurement	total cholesterol measurement	EFO_0004574	Lipid or lipoprotein measurement	Metabolic	PGS003143	EFO_0004574	total cholesterol measurement	total cholesterol measurement	EFO_0004574	Lipid or lipoprotein measurement	Metabolic
0.8172	5.93E-111	1.22E-107	PGS000002	EFO_1000649	estrogen-receptor positive breast cancer	estrogen-receptor positive breast cancer	EFO_1000649	Cancer	Neoplasms	PGS000045	EFO_0000305	breast carcinoma	breast carcinoma	EFO_0000305	Cancer	Neoplasms
0.817	7.36E-111	1.51E-107	PGS000587	EFO_0001663	prostate carcinoma	prostate carcinoma	EFO_0001663	Cancer	Neoplasms	PGS000583	EFO_0001663	prostate carcinoma	prostate carcinoma	EFO_0001663	Cancer	Neoplasms
0.8168	9.21E-111	1.89E-107	PGS003259	EFO_0010472	creatine measurement|glomerular filtration rate	creatine measurement	EFO_0010472	Other measurement	Metabolic	PGS003263	EFO_0010472	creatine measurement|glomerular filtration rate	creatine measurement	EFO_0010472	Other measurement	Metabolic
0.8168	9.69E-111	1.99E-107	PGS002224	EFO_0011011	sitting height measurement	sitting height measurement	EFO_0011011	Other measurement	Skeletal	PGS002146	EFO_0004339	body height	body height	EFO_0004339	Body measurement	Body measurement
0.8167	1.00E-110	2.06E-107	PGS002025	MONDO_0005147	type 1 diabetes mellitus	type 1 diabetes mellitus	MONDO_0005147	Metabolic disorder	Metabolic	PGS002028	HP_0001943	Hypoglycemia	Hypoglycemia	HP_0001943	Other trait	Metabolic
0.8167	1.00E-110	2.06E-107	PGS002025	MONDO_0005147	type 1 diabetes mellitus	type 1 diabetes mellitus	MONDO_0005147	Immune system disorder	Immunological	PGS002028	HP_0001943	Hypoglycemia	Hypoglycemia	HP_0001943	Other trait	Metabolic
0.8167	1.00E-110	2.06E-107	PGS002025	MONDO_0005147	type 1 diabetes mellitus	type 1 diabetes mellitus	MONDO_0005147	Digestive system disorder	Gastrointestinal	PGS002028	HP_0001943	Hypoglycemia	Hypoglycemia	HP_0001943	Other trait	Metabolic
0.8166	1.21E-110	2.47E-107	PGS001278	EFO_0003827	pulmonary embolism|deep vein thrombosis	pulmonary embolism	EFO_0003827	Cardiovascular disease	Cardiovascular	PGS001279	EFO_0003827	pulmonary embolism|deep vein thrombosis	pulmonary embolism	EFO_0003827	Cardiovascular disease	Cardiovascular
0.8165	1.24E-110	2.53E-107	PGS000847	EFO_0004530	triglyceride measurement	triglyceride measurement	EFO_0004530	Lipid or lipoprotein measurement	Metabolic	PGS002572	EFO_0004530	triglyceride measurement	triglyceride measurement	EFO_0004530	Lipid or lipoprotein measurement	Metabolic
0.8164	1.46E-110	2.98E-107	PGS003281	EFO_0004617	cystatin C measurement	cystatin C measurement	EFO_0004617	Other measurement	Metabolic	PGS003286	EFO_0004617	cystatin C measurement	cystatin C measurement	EFO_0004617	Other measurement	Metabolic
0.8164	1.48E-110	3.02E-107	PGS003140	EFO_0004574	total cholesterol measurement	total cholesterol measurement	EFO_0004574	Lipid or lipoprotein measurement	Metabolic	PGS003036	EFO_0004611	low density lipoprotein cholesterol measurement	measurement	EFO_0004611	Cardiovascular measurement	Cardiovascular
0.8164	1.48E-110	3.02E-107	PGS003140	EFO_0004574	total cholesterol measurement	total cholesterol measurement	EFO_0004574	Lipid or lipoprotein measurement	Metabolic	PGS003036	EFO_0004611	low density lipoprotein cholesterol measurement	measurement	EFO_0004611	Lipid or lipoprotein measurement	Metabolic
0.8162	1.81E-110	3.70E-107	PGS002544	EFO_0004842	eosinophil count	eosinophil count	EFO_0004842	Hematological measurement	Hematological	PGS002446	EFO_0004842	eosinophil count	eosinophil count	EFO_0004842	Hematological measurement	Hematological
0.8162	1.89E-110	3.85E-107	PGS001674	EFO_0006930	brain volume measurement|neuroimaging measurement	brain volume measurement	EFO_0006930	Other measurement	Neurological	PGS001671	EFO_0006930	measurement	brain volume measurement	EFO_0006930	Other measurement	Neurological
0.8161	1.95E-110	3.98E-107	PGS002337	EFO_0004611	low density lipoprotein cholesterol measurement	low density lipoprotein cholesterol measurement	EFO_0004611	Lipid or lipoprotein measurement	Metabolic	PGS000672	EFO_0004615	apolipoprotein B measurement	apolipoprotein B measurement	EFO_0004615	Lipid or lipoprotein measurement	Metabolic
0.8161	1.95E-110	3.98E-107	PGS002337	EFO_0004611	low density lipoprotein cholesterol measurement	low density lipoprotein cholesterol measurement	EFO_0004611	Cardiovascular measurement	Cardiovascular	PGS000672	EFO_0004615	apolipoprotein B measurement	apolipoprotein B measurement	EFO_0004615	Lipid or lipoprotein measurement	Metabolic
0.8161	1.99E-110	4.06E-107	PGS002852	EFO_0004340	body mass index	body mass index	EFO_0004340	Body measurement	Body measurement	PGS002856	EFO_0004340	body mass index	body mass index	EFO_0004340	Body measurement	Body measurement
0.8161	2.03E-110	4.13E-107	PGS002543	EFO_0001379	endocrine system disease|diabetes mellitus	endocrine system disease	EFO_0001379	Other disease	Metabolic	PGS002550	EFO_0004541	HbA1c measurement	HbA1c measurement	EFO_0004541	Hematological measurement	Hematological
0.8161	2.06E-110	4.20E-107	PGS000344	EFO_0000305	breast carcinoma	breast carcinoma	EFO_0000305	Cancer	Neoplasms	PGS003399	EFO_0000305	breast carcinoma	breast carcinoma	EFO_0000305	Cancer	Neoplasms
0.816	2.28E-110	4.65E-107	PGS002203	EFO_0007993	lymphocyte percentage of leukocytes	lymphocyte percentage of leukocytes	EFO_0007993	Hematological measurement	Hematological	PGS001414	EFO_0007993	lymphocyte percentage of leukocytes	lymphocyte percentage of leukocytes	EFO_0007993	Hematological measurement	Hematological
0.816	2.30E-110	4.69E-107	PGS003078	EFO_0006335	systolic blood pressure	systolic blood pressure	EFO_0006335	Other measurement	Cardiovascular	PGS003074	EFO_0006335	systolic blood pressure	systolic blood pressure	EFO_0006335	Other measurement	Cardiovascular
0.8159	2.57E-110	5.22E-107	PGS001840	MONDO_0004596	cor pulmonale	cor pulmonale	MONDO_0004596	Cardiovascular disease	Cardiovascular	PGS001280	EFO_0003827	pulmonary embolism	pulmonary embolism	EFO_0003827	Cardiovascular disease	Cardiovascular
-0.8158	2.77E-110	5.63E-107	PGS001045	EFO_0009116	vitamin supplement exposure measurement	vitamin supplement exposure measurement	EFO_0009116	Other measurement	Activities	PGS001271	MONDO_0005301	multiple sclerosis	multiple sclerosis	MONDO_0005301	Immune system disorder	Immunological
-0.8158	2.77E-110	5.63E-107	PGS001045	EFO_0009116	vitamin supplement exposure measurement	vitamin supplement exposure measurement	EFO_0009116	Other measurement	Activities	PGS001271	MONDO_0005301	multiple sclerosis	multiple sclerosis	MONDO_0005301	Neurological disorder	Neurological
0.8158	2.79E-110	5.68E-107	PGS001955	EFO_0009270	heel bone mineral density	heel bone mineral density	EFO_0009270	Other measurement	Skeletal	PGS001529	EFO_0007772	measurement	measurement	EFO_0007772	Other measurement	Skeletal
0.8157	3.12E-110	6.34E-107	PGS003074	EFO_0006335	systolic blood pressure	systolic blood pressure	EFO_0006335	Other measurement	Cardiovascular	PGS003073	EFO_0006335	systolic blood pressure	systolic blood pressure	EFO_0006335	Other measurement	Cardiovascular
0.8157	3.23E-110	6.56E-107	PGS002981	EFO_0004339	body height	body height	EFO_0004339	Body measurement	Body measurement	PGS002990	EFO_0004339	body height	body height	EFO_0004339	Body measurement	Body measurement
0.8155	4.12E-110	8.37E-107	PGS002286	EFO_0004574	total cholesterol measurement	total cholesterol measurement	EFO_0004574	Lipid or lipoprotein measurement	Metabolic	PGS002287	EFO_0004530	triglyceride measurement	triglyceride measurement	EFO_0004530	Lipid or lipoprotein measurement	Metabolic
0.8154	4.31E-110	8.76E-107	PGS000729	MONDO_0005148	type 2 diabetes mellitus	type 2 diabetes mellitus	MONDO_0005148	Metabolic disorder	Metabolic	PGS000330	MONDO_0005148	type 2 diabetes mellitus	type 2 diabetes mellitus	MONDO_0005148	Metabolic disorder	Metabolic
0.8154	4.31E-110	8.76E-107	PGS000729	MONDO_0005148	type 2 diabetes mellitus	type 2 diabetes mellitus	MONDO_0005148	Metabolic disorder	Metabolic	PGS000330	MONDO_0005148	type 2 diabetes mellitus	type 2 diabetes mellitus	MONDO_0005148	Digestive system disorder	Gastrointestinal
0.8154	4.31E-110	8.76E-107	PGS000729	MONDO_0005148	type 2 diabetes mellitus	type 2 diabetes mellitus	MONDO_0005148	Digestive system disorder	Gastrointestinal	PGS000330	MONDO_0005148	type 2 diabetes mellitus	type 2 diabetes mellitus	MONDO_0005148	Metabolic disorder	Metabolic
0.8154	4.31E-110	8.76E-107	PGS000729	MONDO_0005148	type 2 diabetes mellitus	type 2 diabetes mellitus	MONDO_0005148	Digestive system disorder	Gastrointestinal	PGS000330	MONDO_0005148	type 2 diabetes mellitus	type 2 diabetes mellitus	MONDO_0005148	Digestive system disorder	Gastrointestinal
0.8154	4.49E-110	9.12E-107	PGS002207	EFO_0004526	mean corpuscular volume	mean corpuscular volume	EFO_0004526	Hematological measurement	Hematological	PGS000101	EFO_0004526	mean corpuscular volume	mean corpuscular volume	EFO_0004526	Hematological measurement	Hematological

0.8153	4.72E-110	9.58E-107	PGS003142	EFO_0004574	total cholesterol measurement	total cholesterol measurement	EFO_0004574	Lipid or lipoprotein measurement	Metabolic	PGS003037	EFO_0004611	low density lipoprotein cholesterol measurement	measurement	EFO_0004611	Cardiovascular measurement	Cardiovascular
0.8153	4.72E-110	9.58E-107	PGS003142	EFO_0004574	total cholesterol measurement	total cholesterol measurement	EFO_0004574	Lipid or lipoprotein measurement	Metabolic	PGS003037	EFO_0004611	low density lipoprotein cholesterol measurement	measurement	EFO_0004611	Lipid or lipoprotein measurement	Metabolic
0.8153	4.83E-110	9.80E-107	PGS001822	EFO_0004274	gout	gout	EFO_0004274	Metabolic disorder	Metabolic	PGS003329	EFO_0004274	gout	gout	EFO_0004274	Metabolic disorder	Metabolic
-0.8153	5.04E-110	1.02E-106	PGS001956	EFO_0007772	calcaneal bone quantitative ultrasound measurement	calcaneal bone quantitative ultrasound measurement	EFO_0007772	Other measurement	Skeletal	PGS002768	EFO_0003882	osteoporosis	osteoporosis	EFO_0003882	Other disease	Skeletal
0.815	6.53E-110	1.32E-106	PGS003143	EFO_0004574	total cholesterol measurement	total cholesterol measurement	EFO_0004574	Lipid or lipoprotein measurement	Metabolic	PGS002352	EFO_0004574	total cholesterol measurement	total cholesterol measurement	EFO_0004574	Lipid or lipoprotein measurement	Metabolic
0.815	7.14E-110	1.45E-106	PGS001532	EFO_0005671	smoking behaviour measurement	smoking behaviour measurement	EFO_0005671	Other measurement	Psychiatric	PGS001139	EFO_0005671	smoking behaviour measurement	smoking behaviour measurement	EFO_0005671	Other measurement	Psychiatric
0.8149	7.56E-110	1.53E-106	PGS000168	EFO_0004509	hemoglobin measurement	hemoglobin measurement	EFO_0004509	Hematological measurement	Hematological	PGS000167	EFO_0004348	hematocrit	hematocrit	EFO_0004348	Hematological measurement	Hematological
0.8149	7.86E-110	1.59E-106	PGS002028	HP_0001943	Hypoglycemia	Hypoglycemia	HP_0001943	Other trait	Metabolic	PGS001014	EFO_0009605	pancreas disease	pancreas disease	EFO_0009605	Digestive system disorder	Gastrointestinal
-0.8148	8.79E-110	1.78E-106	PGS001271	MONDO_0005301	multiple sclerosis	multiple sclerosis	MONDO_0005301	Neurological disorder	Neurological	PGS001153	EFO_0005111	folic acid measurement	folic acid measurement	EFO_0005111	Other measurement	Metabolic
-0.8148	8.79E-110	1.78E-106	PGS001271	MONDO_0005301	multiple sclerosis	multiple sclerosis	MONDO_0005301	Immune system disorder	Immunological	PGS001153	EFO_0005111	folic acid measurement	folic acid measurement	EFO_0005111	Other measurement	Metabolic
0.8147	9.08E-110	1.84E-106	PGS000048	EFO_0001075	ovarian carcinoma	ovarian carcinoma	EFO_0001075	Cancer	Neoplasms	PGS000069	EFO_1001516	ovarian serous carcinoma	ovarian serous carcinoma	EFO_1001516	Cancer	Neoplasms
0.8147	9.11E-110	1.84E-106	PGS000018	EFO_0001645	coronary artery disease	coronary artery disease	EFO_0001645	Cardiovascular disease	Cardiovascular	PGS003355	EFO_0001645	coronary artery disease	coronary artery disease	EFO_0001645	Cardiovascular disease	Cardiovascular
0.8146	1.10E-109	2.22E-106	PGS000213	EFO_0009780	HER2 negative breast carcinoma	HER2 negative breast carcinoma	EFO_0009780	Cancer	Neoplasms	PGS000345	EFO_0000305	breast carcinoma	breast carcinoma	EFO_0000305	Cancer	Neoplasms
0.8145	1.19E-109	2.40E-106	PGS001840	MONDO_0004596	cor pulmonale	cor pulmonale	MONDO_0004596	Cardiovascular disease	Cardiovascular	PGS001277	EFO_0003827	pulmonary embolism|deep vein thrombosis	pulmonary embolism	EFO_0003827	Cardiovascular disease	Cardiovascular
0.8144	1.30E-109	2.63E-106	PGS002333	EFO_0007986	reticulocyte count	reticulocyte count	EFO_0007986	Hematological measurement	Hematological	PGS001976	EFO_0007986	reticulocyte count	reticulocyte count	EFO_0007986	Hematological measurement	Hematological
0.8143	1.47E-109	2.98E-106	PGS002840	EFO_0004340	body mass index	body mass index	EFO_0004340	Body measurement	Body measurement	PGS002161	EFO_0004340	body mass index	body mass index	EFO_0004340	Body measurement	Body measurement
0.814	2.00E-109	4.03E-106	PGS000913	EFO_0006335	systolic blood pressure	systolic blood pressure	EFO_0006335	Other measurement	Cardiovascular	PGS003074	EFO_0006335	systolic blood pressure	systolic blood pressure	EFO_0006335	Other measurement	Cardiovascular
0.814	2.14E-109	4.32E-106	PGS001740	EFO_0006930	brain volume measurement|neuroimaging measurement	brain volume measurement	EFO_0006930	Other measurement	Neurological	PGS001755	EFO_0006930	measurement	brain volume measurement	EFO_0006930	Other measurement	Neurological
0.8138	2.45E-109	4.94E-106	PGS000517	EFO_0000305	breast carcinoma	breast carcinoma	EFO_0000305	Cancer	Neoplasms	PGS000523	EFO_0000305	breast carcinoma	breast carcinoma	EFO_0000305	Cancer	Neoplasms
0.8138	2.45E-109	4.94E-106	PGS000518	EFO_0000305	breast carcinoma	breast carcinoma	EFO_0000305	Cancer	Neoplasms	PGS000523	EFO_0000305	breast carcinoma	breast carcinoma	EFO_0000305	Cancer	Neoplasms
0.8138	2.57E-109	5.17E-106	PGS002200	EFO_0004631	vitamin D measurement	vitamin D measurement	EFO_0004631	Lipid or lipoprotein measurement	Metabolic	PGS000702	EFO_0004631	vitamin D measurement	vitamin D measurement	EFO_0004631	Lipid or lipoprotein measurement	Metabolic
0.8137	2.85E-109	5.74E-106	PGS002409	EFO_0004611	low density lipoprotein cholesterol measurement	low density lipoprotein cholesterol measurement	EFO_0004611	Lipid or lipoprotein measurement	Metabolic	PGS002473	EFO_0004574	total cholesterol measurement	total cholesterol measurement	EFO_0004574	Lipid or lipoprotein measurement	Metabolic
0.8137	2.85E-109	5.74E-106	PGS002409	EFO_0004611	low density lipoprotein cholesterol measurement	low density lipoprotein cholesterol measurement	EFO_0004611	Cardiovascular measurement	Cardiovascular	PGS002473	EFO_0004574	total cholesterol measurement	total cholesterol measurement	EFO_0004574	Lipid or lipoprotein measurement	Metabolic
0.8137	2.86E-109	5.75E-106	PGS003318	EFO_0004617	cystatin C measurement|glomerular filtration rate	cystatin C measurement	EFO_0004617	Other measurement	Metabolic	PGS003299	EFO_0004617	cystatin C measurement|glomerular filtration rate	cystatin C measurement	EFO_0004617	Other measurement	Metabolic
0.8137	3.02E-109	6.07E-106	PGS002601	EFO_0007986	reticulocyte count	reticulocyte count	EFO_0007986	Hematological measurement	Hematological	PGS002454	EFO_0007986	reticulocyte count	reticulocyte count	EFO_0007986	Hematological measurement	Hematological
0.8136	3.24E-109	6.52E-106	PGS002329	EFO_0004612	high density lipoprotein cholesterol measurement	high density lipoprotein cholesterol measurement	EFO_0004612	Lipid or lipoprotein measurement	Metabolic	PGS001888	EFO_0004614	apolipoprotein A 1 measurement	apolipoprotein A 1 measurement	EFO_0004614	Inflammatory measurement	Immunological
0.8136	3.24E-109	6.52E-106	PGS002329	EFO_0004612	high density lipoprotein cholesterol measurement	high density lipoprotein cholesterol measurement	EFO_0004612	Lipid or lipoprotein measurement	Metabolic	PGS001888	EFO_0004614	apolipoprotein A 1 measurement	apolipoprotein A 1 measurement	EFO_0004614	Cardiovascular measurement	Cardiovascular
0.8136	3.24E-109	6.52E-106	PGS002329	EFO_0004612	high density lipoprotein cholesterol measurement	high density lipoprotein cholesterol measurement	EFO_0004612	Lipid or lipoprotein measurement	Metabolic	PGS001888	EFO_0004614	apolipoprotein A 1 measurement	apolipoprotein A 1 measurement	EFO_0004614	Lipid or lipoprotein measurement	Metabolic
0.8136	3.28E-109	6.59E-106	PGS002534	EFO_0003923	bone density	bone density	EFO_0003923	Other measurement	Skeletal	PGS002436	EFO_0003923	bone density	bone density	EFO_0003923	Other measurement	Skeletal
0.8135	3.71E-109	7.45E-106	PGS001306	EFO_0000729	ulcerative colitis	ulcerative colitis	EFO_0000729	Digestive system disorder	Gastrointestinal	PGS001307	EFO_0000729	ulcerative colitis	ulcerative colitis	EFO_0000729	Digestive system disorder	Gastrointestinal
0.8135	3.71E-109	7.45E-106	PGS001306	EFO_0000729	ulcerative colitis	ulcerative colitis	EFO_0000729	Immune system disorder	Immunological	PGS001307	EFO_0000729	ulcerative colitis	ulcerative colitis	EFO_0000729	Immune system disorder	Immunological
0.8135	3.71E-109	7.45E-106	PGS001306	EFO_0000729	ulcerative colitis	ulcerative colitis	EFO_0000729	Immune system disorder	Immunological	PGS001307	EFO_0000729	ulcerative colitis	ulcerative colitis	EFO_0000729	Digestive system disorder	Gastrointestinal
0.8135	3.71E-109	7.45E-106	PGS001306	EFO_0000729	ulcerative colitis	ulcerative colitis	EFO_0000729	Digestive system disorder	Gastrointestinal	PGS001307	EFO_0000729	ulcerative colitis	ulcerative colitis	EFO_0000729	Immune system disorder	Immunological
0.8134	3.91E-109	7.85E-106	PGS002672	EFO_0004631	vitamin D measurement	vitamin D measurement	EFO_0004631	Lipid or lipoprotein measurement	Metabolic	PGS003193	EFO_0004631	vitamin D measurement	vitamin D measurement	EFO_0004631	Lipid or lipoprotein measurement	Metabolic
0.8133	4.30E-109	8.64E-106	PGS000051	EFO_0000305	breast carcinoma	breast carcinoma	EFO_0000305	Cancer	Neoplasms	PGS000045	EFO_0000305	breast carcinoma	breast carcinoma	EFO_0000305	Cancer	Neoplasms
0.8133	4.32E-109	8.67E-106	PGS000530	EFO_0000305	breast carcinoma	breast carcinoma	EFO_0000305	Cancer	Neoplasms	PGS000497	EFO_0000305	breast carcinoma	breast carcinoma	EFO_0000305	Cancer	Neoplasms
0.8133	4.32E-109	8.67E-106	PGS000530	EFO_0000305	breast carcinoma	breast carcinoma	EFO_0000305	Cancer	Neoplasms	PGS000498	EFO_0000305	breast carcinoma	breast carcinoma	EFO_0000305	Cancer	Neoplasms
0.8133	4.37E-109	8.77E-106	PGS002470	EFO_0006335	systolic blood pressure	systolic blood pressure	EFO_0006335	Other measurement	Cardiovascular	PGS002421	EFO_0006335	systolic blood pressure	systolic blood pressure	EFO_0006335	Other measurement	Cardiovascular
-0.8133	4.46E-109	8.93E-106	PGS002757	EFO_0003884	chronic kidney disease	chronic kidney disease	EFO_0003884	Other disease	Metabolic	PGS003377	EFO_0005208	glomerular filtration rate	glomerular filtration rate	EFO_0005208	Other measurement	Genitourinary
0.8133	4.52E-109	9.05E-106	PGS000633	EFO_0002892	thyroid carcinoma	thyroid carcinoma	EFO_0002892	Cancer	Neoplasms	PGS001809	EFO_0002892	thyroid carcinoma	thyroid carcinoma	EFO_0002892	Cancer	Neoplasms
0.8132	4.89E-109	9.78E-106	PGS000532	EFO_0000305	breast carcinoma	breast carcinoma	EFO_0000305	Cancer	Neoplasms	PGS000504	EFO_0000305	breast carcinoma	breast carcinoma	EFO_0000305	Cancer	Neoplasms
0.8132	4.94E-109	9.88E-106	PGS000005	EFO_1000649	estrogen-receptor positive breast cancer	estrogen-receptor positive breast cancer	EFO_1000649	Cancer	Neoplasms	PGS000345	EFO_0000305	breast carcinoma	breast carcinoma	EFO_0000305	Cancer	Neoplasms
0.8132	4.94E-109	9.88E-106	PGS002791	EFO_0001663	prostate carcinoma	prostate carcinoma	EFO_0001663	Cancer	Neoplasms	PGS000742	EFO_0001663	prostate carcinoma	prostate carcinoma	EFO_0001663	Cancer	Neoplasms
0.8132	5.05E-109	1.01E-105	PGS000404	EFO_0009259	skin carcinoma	skin carcinoma	EFO_0009259	Cancer	Neoplasms	PGS000406	EFO_0009259	skin carcinoma	skin carcinoma	EFO_0009259	Cancer	Neoplasms
0.8132	5.20E-109	1.04E-105	PGS000507	EFO_0000305	breast carcinoma	breast carcinoma	EFO_0000305	Cancer	Neoplasms	PGS000536	EFO_0000305	breast carcinoma	breast carcinoma	EFO_0000305	Cancer	Neoplasms
0.8132	5.25E-109	1.05E-105	PGS003258	EFO_0010472	creatine measurement|glomerular filtration rate	creatine measurement	EFO_0010472	Other measurement	Metabolic	PGS003254	EFO_0010472	creatine measurement|glomerular filtration rate	creatine measurement	EFO_0010472	Other measurement	Metabolic
0.8131	5.59E-109	1.12E-105	PGS000482	EFO_0000305	breast carcinoma	breast carcinoma	EFO_0000305	Cancer	Neoplasms	PGS000472	EFO_0000305	breast carcinoma	breast carcinoma	EFO_0000305	Cancer	Neoplasms
0.8131	5.66E-109	1.13E-105	PGS000200	EFO_0001645	coronary artery disease	coronary artery disease	EFO_0001645	Cardiovascular disease	Cardiovascular	PGS000011	EFO_0001645	coronary artery disease	coronary artery disease	EFO_0001645	Cardiovascular disease	Cardiovascular
0.813	5.94E-109	1.18E-105	PGS002161	EFO_0004340	body mass index	body mass index	EFO_0004340	Body measurement	Body measurement	PGS001983	EFO_0004342	waist circumference	waist circumference	EFO_0004342	Body measurement	Body measurement
0.813	6.13E-109	1.22E-105	PGS003144	EFO_0004530	triglyceride measurement	triglyceride measurement	EFO_0004530	Lipid or lipoprotein measurement	Metabolic	PGS003153	EFO_0004530	triglyceride measurement	triglyceride measurement	EFO_0004530	Lipid or lipoprotein measurement	Metabolic
0.8129	6.65E-109	1.33E-105	PGS002524	MONDO_0005148	type 2 diabetes mellitus	type 2 diabetes mellitus	MONDO_0005148	Metabolic disorder	Metabolic	PGS002573	MONDO_0005148	type 2 diabetes mellitus	type 2 diabetes mellitus	MONDO_0005148	Metabolic disorder	Metabolic
0.8129	6.65E-109	1.33E-105	PGS002524	MONDO_0005148	type 2 diabetes mellitus	type 2 diabetes mellitus	MONDO_0005148	Digestive system disorder	Gastrointestinal	PGS002573	MONDO_0005148	type 2 diabetes mellitus	type 2 diabetes mellitus	MONDO_0005148	Digestive system disorder	Gastrointestinal
0.8129	6.65E-109	1.33E-105	PGS002524	MONDO_0005148	type 2 diabetes mellitus	type 2 diabetes mellitus	MONDO_0005148	Metabolic disorder	Metabolic	PGS002573	MONDO_0005148	type 2 diabetes mellitus	type 2 diabetes mellitus	MONDO_0005148	Digestive system disorder	Gastrointestinal
0.8129	6.65E-109	1.33E-105	PGS002524	MONDO_0005148	type 2 diabetes mellitus	type 2 diabetes mellitus	MONDO_0005148	Digestive system disorder	Gastrointestinal	PGS002573	MONDO_0005148	type 2 diabetes mellitus	type 2 diabetes mellitus	MONDO_0005148	Metabolic disorder	Metabolic
0.8129	6.72E-109	1.34E-105	PGS001230	EFO_0004338	body weight	body weight	EFO_0004338	Body measurement	Body measurement	PGS001234	EFO_0004324	body weights and measures	body weights and measures	EFO_0004324	Body measurement	Body measurement
0.8129	7.24E-109	1.44E-105	PGS000587	EFO_0001663	prostate carcinoma	prostate carcinoma	EFO_0001663	Cancer	Neoplasms	PGS000584	EFO_0001663	prostate carcinoma	prostate carcinoma	EFO_0001663	Cancer	Neoplasms
0.8129	7.27E-109	1.45E-105	PGS003139	EFO_0004574	total cholesterol measurement	total cholesterol measurement	EFO_0004574	Lipid or lipoprotein measurement	Metabolic	PGS003138	EFO_0004574	total cholesterol measurement	total cholesterol measurement	EFO_0004574	Lipid or lipoprotein measurement	Metabolic
0.8128	7.92E-109	1.58E-105	PGS000688	EFO_0004611	low density lipoprotein cholesterol measurement	low density lipoprotein cholesterol measurement	EFO_0004611	Cardiovascular measurement	Cardiovascular	PGS002654	EFO_0004611	low density lipoprotein cholesterol measurement	measurement	EFO_0004611	Cardiovascular measurement	Cardiovascular
0.8128	7.92E-109	1.58E-105	PGS000688	EFO_0004611	low density lipoprotein cholesterol measurement	low density lipoprotein cholesterol measurement	EFO_0004611	Cardiovascular measurement	Cardiovascular	PGS002654	EFO_0004611	low density lipoprotein cholesterol measurement	measurement	EFO_0004611	Lipid or lipoprotein measurement	Metabolic
0.8128	7.92E-109	1.58E-105	PGS000688	EFO_0004611	low density lipoprotein cholesterol measurement	low density lipoprotein cholesterol measurement	EFO_0004611	Lipid or lipoprotein measurement	Metabolic	PGS002654	EFO_0004611	low density lipoprotein cholesterol measurement	measurement	EFO_0004611	Lipid or lipoprotein measurement	Metabolic
0.8128	7.92E-109	1.58E-105	PGS000688	EFO_0004611	low density lipoprotein cholesterol measurement	low density lipoprotein cholesterol measurement	EFO_0004611	Lipid or lipoprotein measurement	Metabolic	PGS002654	EFO_0004611	low density lipoprotein cholesterol measurement	measurement	EFO_0004611	Cardiovascular measurement	Cardiovascular
0.8125	1.05E-108	2.10E-105	PGS001278	EFO_0003827	pulmonary embolism|deep vein thrombosis	pulmonary embolism	EFO_0003827	Cardiovascular disease	Cardiovascular	PGS001280	EFO_0003827	pulmonary embolism	pulmonary embolism	EFO_0003827	Cardiovascular disease	Cardiovascular
0.8124	1.14E-108	2.26E-105	PGS000482	EFO_0000305	breast carcinoma	breast carcinoma	EFO_0000305	Cancer	Neoplasms	PGS000474	EFO_0000305	breast carcinoma	breast carcinoma	EFO_0000305	Cancer	Neoplasms
0.8123	1.30E-108	2.59E-105	PGS000591	EFO_0001663	prostate carcinoma	prostate carcinoma	EFO_0001663	Cancer	Neoplasms	PGS000571	EFO_0001663	prostate carcinoma	prostate carcinoma	EFO_0001663	Cancer	Neoplasms
0.8122	1.56E-108	3.09E-105	PGS001962	EFO_0004308	leukocyte count	leukocyte count	EFO_0004308	Hematological measurement	Hematological	PGS002187	EFO_0004833	neutrophil count	neutrophil count	EFO_0004833	Hematological measurement	Hematological
0.8121	1.57E-108	3.11E-105	PGS003140	EFO_0004574	total cholesterol measurement	total cholesterol measurement	EFO_0004574	Lipid or lipoprotein measurement	Metabolic	PGS003031	EFO_0004611	low density lipoprotein cholesterol measurement	measurement	EFO_0004611	Cardiovascular measurement	Cardiovascular
0.8121	1.57E-108	3.11E-105	PGS003140	EFO_0004574	total cholesterol measurement	total cholesterol measurement	EFO_0004574	Lipid or lipoprotein measurement	Metabolic	PGS003031	EFO_0004611	low density lipoprotein cholesterol measurement	measurement	EFO_0004611	Lipid or lipoprotein measurement	Metabolic
0.8121	1.61E-108	3.19E-105	PGS002622	MONDO_0005148	type 2 diabetes mellitus	type 2 diabetes mellitus	MONDO_0005148	Digestive system disorder	Gastrointestinal	PGS002449	EFO_0004468	glucose measurement	glucose measurement	EFO_0004468	Other measurement	Metabolic
0.8121	1.61E-108	3.19E-105	PGS002622	MONDO_0005148	type 2 diabetes mellitus	type 2 diabetes mellitus	MONDO_0005148	Metabolic disorder	Metabolic	PGS002449	EFO_0004468	glucose measurement	glucose measurement	EFO_0004468	Other measurement	Metabolic
0.8121	1.64E-108	3.26E-105	PGS002502	EFO_0004339	body height	body height	EFO_0004339	Body measurement	Body measurement	PGS002404	EFO_0004339	body height	body height	EFO_0004339	Body measurement	Body measurement
-0.8119	1.95E-108	3.86E-105	PGS002103	EFO_0008385	visual acuity measurement	visual acuity measurement	EFO_0008385	Other measurement	Ophthalmological	PGS002211	HP_0000545	Myopia	Myopia	HP_0000545	Other trait	Ophthalmological
0.8119	2.15E-108	4.27E-105	PGS002257	EFO_0006335	systolic blood pressure	systolic blood pressure	EFO_0006335	Other measurement	Cardiovascular	PGS002258	EFO_0006336	diastolic blood pressure	diastolic blood pressure	EFO_0006336	Other measurement	Cardiovascular
0.8117	2.57E-108	5.08E-105	PGS000471	MONDO_0004641	skin carcinoma in situ	skin carcinoma in situ	MONDO_0004641	Cancer	Neoplasms	PGS000950	GO_0009314	response to radiation	response to radiation	GO_0009314	Biological process	Others
0.8116	2.84E-108	5.62E-105	PGS002981	EFO_0004339	body height	body height	EFO_0004339	Body measurement	Body measurement	PGS002991	EFO_0004339	body height	body height	EFO_0004339	Body measurement	Body measurement
0.8115	3.24E-108	6.42E-105	PGS000513	EFO_0000305	breast carcinoma	breast carcinoma	EFO_0000305	Cancer	Neoplasms	PGS000472	EFO_0000305	breast carcinoma	breast carcinoma	EFO_0000305	Cancer	Neoplasms
-0.8114	3.36E-108	6.65E-105	PGS002330	EFO_0007822	hair colour measurement	hair colour measurement	EFO_0007822	Other measurement	Dermatological	PGS001095	EFO_0007822	hair colour measurement	hair colour measurement	EFO_0007822	Other measurement	Dermatological
0.8114	3.39E-108	6.72E-105	PGS000675	EFO_0004458	C-reactive protein measurement	C-reactive protein measurement	EFO_0004458	Inflammatory measurement	Immunological	PGS002164	EFO_0004458	C-reactive protein measurement	C-reactive protein measurement	EFO_0004458	Inflammatory measurement	Immunological
0.8113	3.74E-108	7.40E-105	PGS002604	EFO_0004705	hypothyroidism	hypothyroidism	EFO_0004705	Other disease	Endocrine	PGS002570	EFO_1000627	thyroid disease	thyroid disease	EFO_1000627	Other disease	Endocrine
0.8113	3.94E-108	7.79E-105	PGS001359	EFO_0004731	eye measurement	eye measurement	EFO_0004731	Other measurement	Ophthalmological	PGS001367	EFO_0004731	eye measurement	eye measurement	EFO_0004731	Other measurement	Ophthalmological
0.8111	4.64E-108	9.17E-105	PGS000169	EFO_0007986	reticulocyte count	reticulocyte count	EFO_0007986	Hematological measurement	Hematological	PGS000189	EFO_0007986	reticulocyte count	reticulocyte count	EFO_0007986	Hematological measurement	Hematological
0.8111	4.71E-108	9.31E-105	PGS001780	EFO_0001645	coronary artery disease	coronary artery disease	EFO_0001645	Cardiovascular disease	Cardiovascular	PGS001355	EFO_0001645	coronary artery disease	coronary artery disease	EFO_0001645	Cardiovascular disease	Cardiovascular
0.8111	4.99E-108	9.86E-105	PGS000313	EFO_0006925	lipoprotein A measurement	lipoprotein A measurement	EFO_0006925	Lipid or lipoprotein measurement	Metabolic	PGS000689	EFO_0006925	lipoprotein A measurement	lipoprotein A measurement	EFO_0006925	Lipid or lipoprotein measurement	Metabolic
0.811	5.36E-108	1.06E-104	PGS001106	EFO_0007800	body fat percentage	body fat percentage	EFO_0007800	Body measurement	Body measurement	PGS001148	EFO_0005409	fat body mass	fat body mass	EFO_0005409	Other measurement	Body measurement
0.811	5.46E-108	1.08E-104	PGS002778	EFO_0001645	coronary artery disease	coronary artery disease	EFO_0001645	Cardiovascular disease	Cardiovascular	PGS002777	EFO_0001645	coronary artery disease	coronary artery disease	EFO_0001645	Cardiovascular disease	Cardiovascular
0.811	5.68E-108	1.12E-104	PGS002954	EFO_0004612	high density lipoprotein cholesterol measurement	high density lipoprotein cholesterol measurement	EFO_0004612	Lipid or lipoprotein measurement	Metabolic	PGS002958	EFO_0004612	high density lipoprotein cholesterol measurement	measurement	EFO_0004612	Lipid or lipoprotein measurement	Metabolic
0.811	5.73E-108	1.13E-104	PGS000181	EFO_0004526	mean corpuscular volume	mean corpuscular volume	EFO_0004526	Hematological measurement	Hematological	PGS000180	EFO_0010701	mean reticulocyte volume	mean reticulocyte volume	EFO_0010701	Hematological measurement	Hematological
0.8109	5.79E-108	1.14E-104	PGS001117	EFO_0600082	insulin use measurement	insulin use measurement	EFO_0600082	Other measurement	Metabolic	PGS001820	HP_0001943	Hypoglycemia	Hypoglycemia	HP_0001943	Other trait	Metabolic
0.8109	6.10E-108	1.20E-104	PGS001806	EFO_0004281	testicular neoplasm	testicular neoplasm	EFO_0004281	Other disease	Neoplasms	PGS000598	EFO_0005088	testicular carcinoma	testicular carcinoma	EFO_0005088	Cancer	Neoplasms
0.8108	7.06E-108	1.39E-104	PGS002898	EFO_0006336	diastolic blood pressure	diastolic blood pressure	EFO_0006336	Other measurement	Cardiovascular	PGS002114	EFO_0006336	diastolic blood pressure	diastolic blood pressure	EFO_0006336	Other measurement	Cardiovascular
0.8107	7.65E-108	1.51E-104	PGS002764	HP_0003124	Hypercholesterolemia	Hypercholesterolemia	HP_0003124	Other trait	Metabolic	PGS002782	EFO_0005689	measurement	measurement	EFO_0005689	Cardiovascular measurement	Cardiovascular
0.8107	7.65E-108	1.51E-104	PGS002764	HP_0003124	Hypercholesterolemia	Hypercholesterolemia	HP_0003124	Other trait	Metabolic	PGS002782	EFO_0005689	measurement	measurement	EFO_0005689	Lipid or lipoprotein measurement	Metabolic
0.8106	8.51E-108	1.67E-104	PGS003150	EFO_0004530	triglyceride measurement	triglyceride measurement	EFO_0004530	Lipid or lipoprotein measurement	Metabolic	PGS003146	EFO_0004530	triglyceride measurement	triglyceride measurement	EFO_0004530	Lipid or lipoprotein measurement	Metabolic
0.8105	9.33E-108	1.84E-104	PGS002958	EFO_0004612	high density lipoprotein cholesterol measurement	high density lipoprotein cholesterol measurement	EFO_0004612	Lipid or lipoprotein measurement	Metabolic	PGS002959	EFO_0004612	high density lipoprotein cholesterol measurement	measurement	EFO_0004612	Lipid or lipoprotein measurement	Metabolic
0.8105	9.45E-108	1.86E-104	PGS000347	EFO_1000649	estrogen-receptor positive breast cancer	estrogen-receptor positive breast cancer	EFO_1000649	Cancer	Neoplasms	PGS003399	EFO_0000305	breast carcinoma	breast carcinoma	EFO_0000305	Cancer	Neoplasms
-0.8105	9.51E-108	1.87E-104	PGS003228	EFO_0005208	glomerular filtration rate	glomerular filtration rate	EFO_0005208	Other measurement	Genitourinary	PGS003377	EFO_0005208	glomerular filtration rate	glomerular filtration rate	EFO_0005208	Other measurement	Genitourinary
0.8105	9.64E-108	1.89E-104	PGS002382	EFO_0004703	age at menarche	age at menarche	EFO_0004703	Other measurement	Genitourinary	PGS002578	EFO_0004703	age at menarche	age at menarche	EFO_0004703	Other measurement	Genitourinary
0.8105	9.69E-108	1.90E-104	PGS000499	EFO_0000305	breast carcinoma	breast carcinoma	EFO_0000305	Cancer	Neoplasms	PGS000503	EFO_0000305	breast carcinoma	breast carcinoma	EFO_0000305	Cancer	Neoplasms
0.8104	1.02E-107	2.00E-104	PGS001979	EFO_0004530	triglyceride measurement	triglyceride measurement	EFO_0004530	Lipid or lipoprotein measurement	Metabolic	PGS003148	EFO_0004530	triglyceride measurement	triglyceride measurement	EFO_0004530	Lipid or lipoprotein measurement	Metabolic
0.8104	1.02E-107	2.00E-104	PGS003253	EFO_0010472	creatine measurement|glomerular filtration rate	creatine measurement	EFO_0010472	Other measurement	Metabolic	PGS003264	EFO_0010472	creatine measurement|glomerular filtration rate	creatine measurement	EFO_0010472	Other measurement	Metabolic
0.8102	1.28E-107	2.51E-104	PGS002672	EFO_0004631	vitamin D measurement	vitamin D measurement	EFO_0004631	Lipid or lipoprotein measurement	Metabolic	PGS002427	EFO_0004631	vitamin D measurement	vitamin D measurement	EFO_0004631	Lipid or lipoprotein measurement	Metabolic
0.8101	1.46E-107	2.86E-104	PGS001278	EFO_0003827	pulmonary embolism|deep vein thrombosis	pulmonary embolism	EFO_0003827	Cardiovascular disease	Cardiovascular	PGS001277	EFO_0003827	pulmonary embolism|deep vein thrombosis	pulmonary embolism	EFO_0003827	Cardiovascular disease	Cardiovascular
0.8099	1.68E-107	3.29E-104	PGS003110	MONDO_0005148	type 2 diabetes mellitus	type 2 diabetes mellitus	MONDO_0005148	Metabolic disorder	Metabolic	PGS003096	MONDO_0005148	type 2 diabetes mellitus	type 2 diabetes mellitus	MONDO_0005148	Metabolic disorder	Metabolic
0.8099	1.68E-107	3.29E-104	PGS003110	MONDO_0005148	type 2 diabetes mellitus	type 2 diabetes mellitus	MONDO_0005148	Digestive system disorder	Gastrointestinal	PGS003096	MONDO_0005148	type 2 diabetes mellitus	type 2 diabetes mellitus	MONDO_0005148	Metabolic disorder	Metabolic

0.8099	1.68E-107	3.29E-104	PGS003110	MONDO_0005148	type 2 diabetes mellitus	type 2 diabetes mellitus	MONDO_0005148	Digestive system disorder	Gastrointestinal	PGS003096	MONDO_0005148	type 2 diabetes mellitus	type 2 diabetes mellitus	MONDO_0005148	Digestive system disorder	Gastrointestinal
0.8099	1.68E-107	3.29E-104	PGS003110	MONDO_0005148	type 2 diabetes mellitus	type 2 diabetes mellitus	MONDO_0005148	Metabolic disorder	Metabolic	PGS003096	MONDO_0005148	type 2 diabetes mellitus	type 2 diabetes mellitus	MONDO_0005148	Digestive system disorder	Gastrointestinal
0.8099	1.71E-107	3.36E-104	PGS002161	EFO_0004340	body mass index	body mass index	EFO_0004340	Body measurement	Body measurement	PGS002850	EFO_0004340	body mass index	body mass index	EFO_0004340	Body measurement	Body measurement
0.8099	1.73E-107	3.39E-104	PGS002170	EFO_0004468	glucose measurement	glucose measurement	EFO_0004468	Other measurement	Metabolic	PGS002938	EFO_0004468	glucose measurement	glucose measurement	EFO_0004468	Other measurement	Metabolic
0.8099	1.74E-107	3.41E-104	PGS000575	EFO_0001663	prostate carcinoma	prostate carcinoma	EFO_0001663	Cancer	Neoplasms	PGS000589	EFO_0001663	prostate carcinoma	prostate carcinoma	EFO_0001663	Cancer	Neoplasms
0.8099	1.82E-107	3.56E-104	PGS002771	MONDO_0005148	type 2 diabetes mellitus	type 2 diabetes mellitus	MONDO_0005148	Digestive system disorder	Gastrointestinal	PGS000330	MONDO_0005148	type 2 diabetes mellitus	type 2 diabetes mellitus	MONDO_0005148	Digestive system disorder	Gastrointestinal
0.8099	1.82E-107	3.56E-104	PGS002771	MONDO_0005148	type 2 diabetes mellitus	type 2 diabetes mellitus	MONDO_0005148	Digestive system disorder	Gastrointestinal	PGS000330	MONDO_0005148	type 2 diabetes mellitus	type 2 diabetes mellitus	MONDO_0005148	Metabolic disorder	Metabolic
0.8099	1.82E-107	3.56E-104	PGS002771	MONDO_0005148	type 2 diabetes mellitus	type 2 diabetes mellitus	MONDO_0005148	Metabolic disorder	Metabolic	PGS000330	MONDO_0005148	type 2 diabetes mellitus	type 2 diabetes mellitus	MONDO_0005148	Metabolic disorder	Metabolic
0.8099	1.82E-107	3.56E-104	PGS002771	MONDO_0005148	type 2 diabetes mellitus	type 2 diabetes mellitus	MONDO_0005148	Metabolic disorder	Metabolic	PGS000330	MONDO_0005148	type 2 diabetes mellitus	type 2 diabetes mellitus	MONDO_0005148	Digestive system disorder	Gastrointestinal
0.8097	2.20E-107	4.31E-104	PGS002893	EFO_0006336	diastolic blood pressure	diastolic blood pressure	EFO_0006336	Other measurement	Cardiovascular	PGS002114	EFO_0006336	diastolic blood pressure	diastolic blood pressure	EFO_0006336	Other measurement	Cardiovascular
0.8097	2.22E-107	4.34E-104	PGS000573	EFO_0001663	prostate carcinoma	prostate carcinoma	EFO_0001663	Cancer	Neoplasms	PGS000593	EFO_0007355	male reproductive organ cancer	male reproductive organ cancer	EFO_0007355	Cancer	Neoplasms
0.8095	2.75E-107	5.38E-104	PGS000212	MONDO_0021116	luminal A breast carcinoma	luminal A breast carcinoma	MONDO_0021116	Cancer	Neoplasms	PGS000005	EFO_1000649	estrogen-receptor positive breast cancer	cancer	EFO_1000649	Cancer	Neoplasms
0.8095	2.81E-107	5.48E-104	PGS000742	EFO_0001663	prostate carcinoma	prostate carcinoma	EFO_0001663	Cancer	Neoplasms	PGS000580	EFO_0001663	prostate carcinoma	prostate carcinoma	EFO_0001663	Cancer	Neoplasms
0.8094	3.06E-107	5.96E-104	PGS001951	EFO_0005409	fat body mass	fat body mass	EFO_0005409	Other measurement	Body measurement	PGS002176	EFO_0005093	hip circumference	hip circumference	EFO_0005093	Body measurement	Body measurement
-0.8092	3.65E-107	7.12E-104	PGS003223	EFO_0005208	glomerular filtration rate	glomerular filtration rate	EFO_0005208	Other measurement	Genitourinary	PGS003377	EFO_0005208	glomerular filtration rate	glomerular filtration rate	EFO_0005208	Other measurement	Genitourinary
0.8092	3.87E-107	7.54E-104	PGS002161	EFO_0004340	body mass index	body mass index	EFO_0004340	Body measurement	Body measurement	PGS001951	EFO_0005409	fat body mass	fat body mass	EFO_0005409	Other measurement	Body measurement
0.8091	4.20E-107	8.18E-104	PGS002452	EFO_0004541	HbA1c measurement	HbA1c measurement	EFO_0004541	Hematological measurement	Hematological	PGS002449	EFO_0004468	glucose measurement	glucose measurement	EFO_0004468	Other measurement	Metabolic
0.809	4.52E-107	8.80E-104	PGS003148	EFO_0004530	triglyceride measurement	triglyceride measurement	EFO_0004530	Lipid or lipoprotein measurement	Metabolic	PGS002197	EFO_0004530	triglyceride measurement	triglyceride measurement	EFO_0004530	Lipid or lipoprotein measurement	Metabolic
0.8089	5.11E-107	9.96E-104	PGS002182	EFO_0004532	serum gamma-glutamyl transferase measurement	serum gamma-glutamyl transferase measurement	EFO_0004532	Liver enzyme measurement	Gastrointestinal	PGS000683	EFO_0004532	serum gamma-glutamyl transferase measurement	measurement	EFO_0004532	Liver enzyme measurement	Gastrointestinal
0.8088	5.72E-107	1.11E-103	PGS003093	MONDO_0005148	type 2 diabetes mellitus	type 2 diabetes mellitus	MONDO_0005148	Metabolic disorder	Metabolic	PGS002324	EFO_0001379	endocrine system disease|diabetes mellitus	endocrine system disease	EFO_0001379	Other disease	Metabolic
0.8088	5.72E-107	1.11E-103	PGS003093	MONDO_0005148	type 2 diabetes mellitus	type 2 diabetes mellitus	MONDO_0005148	Digestive system disorder	Gastrointestinal	PGS002324	EFO_0001379	endocrine system disease|diabetes mellitus	endocrine system disease	EFO_0001379	Other disease	Metabolic
0.8088	6.02E-107	1.17E-103	PGS001943	EFO_0004340	body mass index	body mass index	EFO_0004340	Body measurement	Body measurement	PGS002854	EFO_0004340	body mass index	body mass index	EFO_0004340	Body measurement	Body measurement
-0.8087	6.07E-107	1.18E-103	PGS001045	EFO_0009116	vitamin supplement exposure measurement	vitamin supplement exposure measurement	EFO_0009116	Other measurement	Activities	PGS001270	MONDO_0005301	multiple sclerosis	multiple sclerosis	MONDO_0005301	Neurological disorder	Neurological
-0.8087	6.07E-107	1.18E-103	PGS001045	EFO_0009116	vitamin supplement exposure measurement	vitamin supplement exposure measurement	EFO_0009116	Other measurement	Activities	PGS001270	MONDO_0005301	multiple sclerosis	multiple sclerosis	MONDO_0005301	Immune system disorder	Immunological
0.8087	6.24E-107	1.21E-103	PGS000684	EFO_0004468	glucose measurement	glucose measurement	EFO_0004468	Other measurement	Metabolic	PGS001952	EFO_0004468	glucose measurement	glucose measurement	EFO_0004468	Other measurement	Metabolic
0.8087	6.28E-107	1.22E-103	PGS002150	EFO_0004611	low density lipoprotein cholesterol measurement	low density lipoprotein cholesterol measurement	EFO_0004611	Cardiovascular measurement	Cardiovascular	PGS000688	EFO_0004611	low density lipoprotein cholesterol measurement	measurement	EFO_0004611	Lipid or lipoprotein measurement	Metabolic
0.8087	6.28E-107	1.22E-103	PGS002150	EFO_0004611	low density lipoprotein cholesterol measurement	low density lipoprotein cholesterol measurement	EFO_0004611	Lipid or lipoprotein measurement	Metabolic	PGS000688	EFO_0004611	low density lipoprotein cholesterol measurement	measurement	EFO_0004611	Lipid or lipoprotein measurement	Metabolic
0.8087	6.28E-107	1.22E-103	PGS002150	EFO_0004611	low density lipoprotein cholesterol measurement	low density lipoprotein cholesterol measurement	EFO_0004611	Lipid or lipoprotein measurement	Metabolic	PGS000688	EFO_0004611	low density lipoprotein cholesterol measurement	measurement	EFO_0004611	Cardiovascular measurement	Cardiovascular
0.8087	6.28E-107	1.22E-103	PGS002150	EFO_0004611	low density lipoprotein cholesterol measurement	low density lipoprotein cholesterol measurement	EFO_0004611	Cardiovascular measurement	Cardiovascular	PGS000688	EFO_0004611	low density lipoprotein cholesterol measurement	measurement	EFO_0004611	Cardiovascular measurement	Cardiovascular
0.8087	6.33E-107	1.23E-103	PGS002224	EFO_0011011	sitting height measurement	sitting height measurement	EFO_0011011	Other measurement	Skeletal	PGS000969	EFO_0011011	sitting height measurement	sitting height measurement	EFO_0011011	Other measurement	Skeletal
0.8087	6.33E-107	1.23E-103	PGS002391	EFO_0011015	educational attainment	educational attainment	EFO_0011015	Other measurement	Activities	PGS002538	EFO_0011015	educational attainment	educational attainment	EFO_0011015	Other measurement	Activities
0.8086	6.75E-107	1.31E-103	PGS002180	EFO_0004308	leukocyte count	leukocyte count	EFO_0004308	Hematological measurement	Hematological	PGS001969	EFO_0004833	neutrophil count	neutrophil count	EFO_0004833	Hematological measurement	Hematological
0.8086	6.78E-107	1.32E-103	PGS000700	EFO_0004531	urate measurement	urate measurement	EFO_0004531	Other measurement	Metabolic	PGS002229	EFO_0004531	urate measurement	urate measurement	EFO_0004531	Other measurement	Metabolic
0.8086	7.46E-107	1.45E-103	PGS002174	EFO_0007772	calcaneal bone quantitative ultrasound measurement	calcaneal bone quantitative ultrasound measurement	EFO_0007772	Other measurement	Skeletal	PGS002315	EFO_0003923	bone density	bone density	EFO_0003923	Other measurement	Skeletal
0.8085	7.65E-107	1.48E-103	PGS001147	EFO_0005409	fat body mass	fat body mass	EFO_0005409	Other measurement	Body measurement	PGS001104	EFO_0007800	body fat percentage	body fat percentage	EFO_0007800	Body measurement	Body measurement
0.8085	7.92E-107	1.54E-103	PGS001943	EFO_0004340	body mass index	body mass index	EFO_0004340	Body measurement	Body measurement	PGS002201	EFO_0004342	waist circumference	waist circumference	EFO_0004342	Body measurement	Body measurement
0.8085	8.15E-107	1.58E-103	PGS000577	EFO_0001663	prostate carcinoma	prostate carcinoma	EFO_0001663	Cancer	Neoplasms	PGS000742	EFO_0001663	prostate carcinoma	prostate carcinoma	EFO_0001663	Cancer	Neoplasms
0.8085	8.15E-107	1.58E-103	PGS000578	EFO_0001663	prostate carcinoma	prostate carcinoma	EFO_0001663	Cancer	Neoplasms	PGS000742	EFO_0001663	prostate carcinoma	prostate carcinoma	EFO_0001663	Cancer	Neoplasms
0.8085	8.29E-107	1.60E-103	PGS001943	EFO_0004340	body mass index	body mass index	EFO_0004340	Body measurement	Body measurement	PGS002859	EFO_0004340	body mass index	body mass index	EFO_0004340	Body measurement	Body measurement
0.8083	9.46E-107	1.83E-103	PGS002581	EFO_0004340	body mass index	body mass index	EFO_0004340	Body measurement	Body measurement	PGS002385	EFO_0004340	body mass index	body mass index	EFO_0004340	Body measurement	Body measurement
0.8082	1.05E-106	2.02E-103	PGS002550	EFO_0004541	HbA1c measurement	HbA1c measurement	EFO_0004541	Hematological measurement	Hematological	PGS002400	EFO_0004468	glucose measurement	glucose measurement	EFO_0004468	Other measurement	Metabolic
0.8082	1.06E-106	2.05E-103	PGS002584	EFO_0000319	cardiovascular disease	cardiovascular disease	EFO_0000319	Cardiovascular disease	Cardiovascular	PGS002554	EFO_0000537	hypertension	hypertension	EFO_0000537	Cardiovascular disease	Cardiovascular
0.8082	1.08E-106	2.08E-103	PGS000027	EFO_0004340	body mass index	body mass index	EFO_0004340	Body measurement	Body measurement	PGS000320	EFO_0004340	body mass index	body mass index	EFO_0004340	Body measurement	Body measurement
0.8081	1.16E-106	2.25E-103	PGS002173	EFO_0009270	heel bone mineral density	heel bone mineral density	EFO_0009270	Other measurement	Skeletal	PGS001402	EFO_0009270	heel bone mineral density	heel bone mineral density	EFO_0009270	Other measurement	Skeletal
0.8081	1.21E-106	2.33E-103	PGS001674	EFO_0006930	brain volume measurement|neuroimaging measurement	brain volume measurement	EFO_0006930	Other measurement	Neurological	PGS001683	EFO_0006930	measurement	brain volume measurement	EFO_0006930	Other measurement	Neurological
0.8081	1.24E-106	2.39E-103	PGS002191	EFO_0004309	platelet count	platelet count	EFO_0004309	Hematological measurement	Hematological	PGS001238	EFO_0004309	platelet count	platelet count	EFO_0004309	Hematological measurement	Hematological
0.808	1.28E-106	2.47E-103	PGS000332	EFO_0000305	breast carcinoma	breast carcinoma	EFO_0000305	Cancer	Neoplasms	PGS000512	EFO_0000305	breast carcinoma	breast carcinoma	EFO_0000305	Cancer	Neoplasms
0.808	1.37E-106	2.64E-103	PGS002108	EFO_0004574	total cholesterol measurement	total cholesterol measurement	EFO_0004574	Lipid or lipoprotein measurement	Metabolic	PGS001889	EFO_0004615	apolipoprotein B measurement	apolipoprotein B measurement	EFO_0004615	Lipid or lipoprotein measurement	Metabolic
0.8079	1.51E-106	2.91E-103	PGS000437	MONDO_0002898	skin cancer	skin cancer	MONDO_0002898	Cancer	Neoplasms	PGS000441	MONDO_0002898	skin cancer	skin cancer	MONDO_0002898	Cancer	Neoplasms
0.8079	1.53E-106	2.94E-103	PGS003135	EFO_0004574	total cholesterol measurement	total cholesterol measurement	EFO_0004574	Lipid or lipoprotein measurement	Metabolic	PGS003036	EFO_0004611	low density lipoprotein cholesterol measurement	measurement	EFO_0004611	Lipid or lipoprotein measurement	Metabolic
0.8079	1.53E-106	2.94E-103	PGS003135	EFO_0004574	total cholesterol measurement	total cholesterol measurement	EFO_0004574	Lipid or lipoprotein measurement	Metabolic	PGS003036	EFO_0004611	low density lipoprotein cholesterol measurement	measurement	EFO_0004611	Cardiovascular measurement	Cardiovascular
0.8078	1.68E-106	3.23E-103	PGS002173	EFO_0009270	heel bone mineral density	heel bone mineral density	EFO_0009270	Other measurement	Skeletal	PGS001037	EFO_0009270	heel bone mineral density	heel bone mineral density	EFO_0009270	Other measurement	Skeletal
0.8078	1.70E-106	3.27E-103	PGS000952	EFO_0004514	bone quantitative ultrasound measurement	bone quantitative ultrasound measurement	EFO_0004514	Other measurement	Skeletal	PGS002173	EFO_0009270	heel bone mineral density	heel bone mineral density	EFO_0009270	Other measurement	Skeletal
0.8078	1.74E-106	3.36E-103	PGS000569	EFO_0001663	prostate carcinoma	prostate carcinoma	EFO_0001663	Cancer	Neoplasms	PGS000589	EFO_0001663	prostate carcinoma	prostate carcinoma	EFO_0001663	Cancer	Neoplasms
0.8077	1.78E-106	3.43E-103	PGS000377	EFO_1001950	colon carcinoma	colon carcinoma	EFO_1001950	Cancer	Neoplasms	PGS000379	EFO_1001950	colon carcinoma	colon carcinoma	EFO_1001950	Cancer	Neoplasms
-0.8077	1.79E-106	3.43E-103	PGS001153	EFO_0005111	folic acid measurement	folic acid measurement	EFO_0005111	Other measurement	Metabolic	PGS001270	MONDO_0005301	multiple sclerosis	multiple sclerosis	MONDO_0005301	Neurological disorder	Neurological
0.8077	1.78E-106	3.43E-103	PGS000377	EFO_1001950	colon carcinoma	colon carcinoma	EFO_1001950	Cancer	Neoplasms	PGS000379	EFO_1001950	colon carcinoma	colon carcinoma	EFO_1001950	Digestive system disorder	Gastrointestinal
0.8077	1.78E-106	3.43E-103	PGS000377	EFO_1001950	colon carcinoma	colon carcinoma	EFO_1001950	Digestive system disorder	Gastrointestinal	PGS000379	EFO_1001950	colon carcinoma	colon carcinoma	EFO_1001950	Digestive system disorder	Gastrointestinal
0.8077	1.78E-106	3.43E-103	PGS000377	EFO_1001950	colon carcinoma	colon carcinoma	EFO_1001950	Digestive system disorder	Gastrointestinal	PGS000379	EFO_1001950	colon carcinoma	colon carcinoma	EFO_1001950	Cancer	Neoplasms
-0.8077	1.79E-106	3.43E-103	PGS001153	EFO_0005111	folic acid measurement	folic acid measurement	EFO_0005111	Other measurement	Metabolic	PGS001270	MONDO_0005301	multiple sclerosis	multiple sclerosis	MONDO_0005301	Immune system disorder	Immunological
0.8076	1.96E-106	3.76E-103	PGS000832	MONDO_0005148	type 2 diabetes mellitus	type 2 diabetes mellitus	MONDO_0005148	Metabolic disorder	Metabolic	PGS000868	MONDO_0005148	type 2 diabetes mellitus	type 2 diabetes mellitus	MONDO_0005148	Digestive system disorder	Gastrointestinal
0.8076	1.96E-106	3.76E-103	PGS000832	MONDO_0005148	type 2 diabetes mellitus	type 2 diabetes mellitus	MONDO_0005148	Digestive system disorder	Gastrointestinal	PGS000868	MONDO_0005148	type 2 diabetes mellitus	type 2 diabetes mellitus	MONDO_0005148	Digestive system disorder	Gastrointestinal
0.8076	1.96E-106	3.76E-103	PGS000832	MONDO_0005148	type 2 diabetes mellitus	type 2 diabetes mellitus	MONDO_0005148	Metabolic disorder	Metabolic	PGS000868	MONDO_0005148	type 2 diabetes mellitus	type 2 diabetes mellitus	MONDO_0005148	Metabolic disorder	Metabolic
0.8076	1.96E-106	3.76E-103	PGS000832	MONDO_0005148	type 2 diabetes mellitus	type 2 diabetes mellitus	MONDO_0005148	Digestive system disorder	Gastrointestinal	PGS000868	MONDO_0005148	type 2 diabetes mellitus	type 2 diabetes mellitus	MONDO_0005148	Metabolic disorder	Metabolic
0.8075	2.17E-106	4.16E-103	PGS001672	EFO_0006930	brain volume measurement|neuroimaging measurement	brain volume measurement	EFO_0006930	Other measurement	Neurological	PGS001680	EFO_0006930	measurement	brain volume measurement	EFO_0006930	Other measurement	Neurological
0.8075	2.27E-106	4.37E-103	PGS003009	EFO_0000537	hypertension	hypertension	EFO_0000537	Cardiovascular disease	Cardiovascular	PGS003020	EFO_0000537	hypertension	hypertension	EFO_0000537	Cardiovascular disease	Cardiovascular
0.8075	2.27E-106	4.37E-103	PGS003021	EFO_0000537	hypertension	hypertension	EFO_0000537	Cardiovascular disease	Cardiovascular	PGS003009	EFO_0000537	hypertension	hypertension	EFO_0000537	Cardiovascular disease	Cardiovascular
0.8073	2.82E-106	5.41E-103	PGS003123	MONDO_0005148	type 2 diabetes mellitus	type 2 diabetes mellitus	MONDO_0005148	Digestive system disorder	Gastrointestinal	PGS003104	MONDO_0005148	type 2 diabetes mellitus	type 2 diabetes mellitus	MONDO_0005148	Digestive system disorder	Gastrointestinal
0.8073	2.82E-106	5.41E-103	PGS003123	MONDO_0005148	type 2 diabetes mellitus	type 2 diabetes mellitus	MONDO_0005148	Metabolic disorder	Metabolic	PGS003104	MONDO_0005148	type 2 diabetes mellitus	type 2 diabetes mellitus	MONDO_0005148	Metabolic disorder	Metabolic
0.8073	2.82E-106	5.41E-103	PGS003123	MONDO_0005148	type 2 diabetes mellitus	type 2 diabetes mellitus	MONDO_0005148	Digestive system disorder	Gastrointestinal	PGS003104	MONDO_0005148	type 2 diabetes mellitus	type 2 diabetes mellitus	MONDO_0005148	Metabolic disorder	Metabolic
0.8073	2.82E-106	5.41E-103	PGS003123	MONDO_0005148	type 2 diabetes mellitus	type 2 diabetes mellitus	MONDO_0005148	Metabolic disorder	Metabolic	PGS003104	MONDO_0005148	type 2 diabetes mellitus	type 2 diabetes mellitus	MONDO_0005148	Digestive system disorder	Gastrointestinal
0.8072	3.14E-106	6.02E-103	PGS002339	EFO_0004527	mean corpuscular hemoglobin	mean corpuscular hemoglobin	EFO_0004527	Hematological measurement	Hematological	PGS000099	EFO_0004527	mean corpuscular hemoglobin	mean corpuscular hemoglobin	EFO_0004527	Hematological measurement	Hematological
0.8072	3.22E-106	6.18E-103	PGS000023	MONDO_0005147	type 1 diabetes mellitus	type 1 diabetes mellitus	MONDO_0005147	Digestive system disorder	Gastrointestinal	PGS002028	HP_0001943	Hypoglycemia	Hypoglycemia	HP_0001943	Other trait	Metabolic
0.8072	3.22E-106	6.18E-103	PGS000023	MONDO_0005147	type 1 diabetes mellitus	type 1 diabetes mellitus	MONDO_0005147	Immune system disorder	Immunological	PGS002028	HP_0001943	Hypoglycemia	Hypoglycemia	HP_0001943	Other trait	Metabolic
0.8072	3.22E-106	6.18E-103	PGS000023	MONDO_0005147	type 1 diabetes mellitus	type 1 diabetes mellitus	MONDO_0005147	Metabolic disorder	Metabolic	PGS002028	HP_0001943	Hypoglycemia	Hypoglycemia	HP_0001943	Other trait	Metabolic
0.8071	3.39E-106	6.49E-103	PGS003103	MONDO_0005148	type 2 diabetes mellitus	type 2 diabetes mellitus	MONDO_0005148	Digestive system disorder	Gastrointestinal	PGS003117	MONDO_0005148	type 2 diabetes mellitus	type 2 diabetes mellitus	MONDO_0005148	Digestive system disorder	Gastrointestinal
0.8071	3.39E-106	6.49E-103	PGS003103	MONDO_0005148	type 2 diabetes mellitus	type 2 diabetes mellitus	MONDO_0005148	Digestive system disorder	Gastrointestinal	PGS003117	MONDO_0005148	type 2 diabetes mellitus	type 2 diabetes mellitus	MONDO_0005148	Metabolic disorder	Metabolic
0.8071	3.39E-106	6.49E-103	PGS003103	MONDO_0005148	type 2 diabetes mellitus	type 2 diabetes mellitus	MONDO_0005148	Metabolic disorder	Metabolic	PGS003117	MONDO_0005148	type 2 diabetes mellitus	type 2 diabetes mellitus	MONDO_0005148	Metabolic disorder	Metabolic
0.8071	3.39E-106	6.49E-103	PGS003103	MONDO_0005148	type 2 diabetes mellitus	type 2 diabetes mellitus	MONDO_0005148	Metabolic disorder	Metabolic	PGS003117	MONDO_0005148	type 2 diabetes mellitus	type 2 diabetes mellitus	MONDO_0005148	Digestive system disorder	Gastrointestinal
0.8071	3.48E-106	6.67E-103	PGS003108	MONDO_0005148	type 2 diabetes mellitus	type 2 diabetes mellitus	MONDO_0005148	Digestive system disorder	Gastrointestinal	PGS003104	MONDO_0005148	type 2 diabetes mellitus	type 2 diabetes mellitus	MONDO_0005148	Digestive system disorder	Gastrointestinal
0.8071	3.48E-106	6.67E-103	PGS003108	MONDO_0005148	type 2 diabetes mellitus	type 2 diabetes mellitus	MONDO_0005148	Metabolic disorder	Metabolic	PGS003104	MONDO_0005148	type 2 diabetes mellitus	type 2 diabetes mellitus	MONDO_0005148	Metabolic disorder	Metabolic
0.8071	3.48E-106	6.67E-103	PGS003108	MONDO_0005148	type 2 diabetes mellitus	type 2 diabetes mellitus	MONDO_0005148	Digestive system disorder	Gastrointestinal	PGS003104	MONDO_0005148	type 2 diabetes mellitus	type 2 diabetes mellitus	MONDO_0005148	Metabolic disorder	Metabolic
0.8071	3.48E-106	6.67E-103	PGS003108	MONDO_0005148	type 2 diabetes mellitus	type 2 diabetes mellitus	MONDO_0005148	Metabolic disorder	Metabolic	PGS003104	MONDO_0005148	type 2 diabetes mellitus	type 2 diabetes mellitus	MONDO_0005148	Digestive system disorder	Gastrointestinal
0.8071	3.57E-106	6.84E-103	PGS002325	EFO_0004842	eosinophil count	eosinophil count	EFO_0004842	Hematological measurement	Hematological	PGS002642	EFO_0004842	eosinophil count	eosinophil count	EFO_0004842	Hematological measurement	Hematological
-0.8071	3.60E-106	6.89E-103	PGS002326	EFO_0004713	FEV|FEC ratio	FEV|FEC ratio	EFO_0004713	Other measurement	Respiratory	PGS001801	EFO_0004713	FEV|FEC ratio	FEV|FEC ratio	EFO_0004713	Other measurement	Respiratory
0.8071	3.67E-106	7.02E-103	PGS003103	MONDO_0005148	type 2 diabetes mellitus	type 2 diabetes mellitus	MONDO_0005148	Digestive system disorder	Gastrointestinal	PGS003102	MONDO_0005148	type 2 diabetes mellitus	type 2 diabetes mellitus	MONDO_0005148	Digestive system disorder	Gastrointestinal
0.8071	3.67E-106	7.02E-103	PGS003103	MONDO_0005148	type 2 diabetes mellitus	type 2 diabetes mellitus	MONDO_0005148	Metabolic disorder	Metabolic	PGS003102	MONDO_0005148	type 2 diabetes mellitus	type 2 diabetes mellitus	MONDO_0005148	Digestive system disorder	Gastrointestinal
0.8071	3.67E-106	7.02E-103	PGS003103	MONDO_0005148	type 2 diabetes mellitus	type 2 diabetes mellitus	MONDO_0005148	Metabolic disorder	Metabolic	PGS003102	MONDO_0005148	type 2 diabetes mellitus	type 2 diabetes mellitus	MONDO_0005148	Metabolic disorder	Metabolic
0.8071	3.67E-106	7.02E-103	PGS003103	MONDO_0005148	type 2 diabetes mellitus	type 2 diabetes mellitus	MONDO_0005148	Digestive system disorder	Gastrointestinal	PGS003102	MONDO_0005148	type 2 diabetes mellitus	type 2 diabetes mellitus	MONDO_0005148	Metabolic disorder	Metabolic
0.807	3.73E-106	7.14E-103	PGS002197	EFO_0004530	triglyceride measurement	triglyceride measurement	EFO_0004530	Lipid or lipoprotein measurement	Metabolic	PGS003153	EFO_0004530	triglyceride measurement	triglyceride measurement	EFO_0004530	Lipid or lipoprotein measurement	Metabolic
0.8066	5.62E-106	1.07E-102	PGS000504	EFO_0000305	breast carcinoma	breast carcinoma	EFO_0000305	Cancer	Neoplasms	PGS000540	EFO_0000305	breast carcinoma	breast carcinoma	EFO_0000305	Cancer	Neoplasms
0.8066	5.93E-106	1.13E-102	PGS001819	EFO_0003770	diabetic retinopathy	diabetic retinopathy	EFO_0003770	Cardiovascular disease	Cardiovascular	PGS001820	HP_0001943	Hypoglycemia	Hypoglycemia	HP_0001943	Other trait	Metabolic
0.8066	5.93E-106	1.13E-102	PGS001819	EFO_0003770	diabetic retinopathy	diabetic retinopathy	EFO_0003770	Metabolic disorder	Metabolic	PGS001820	HP_0001943	Hypoglycemia	Hypoglycemia	HP_0001943	Other trait	Metabolic
0.8066	5.93E-106	1.13E-102	PGS001819	EFO_0003770	diabetic retinopathy	diabetic retinopathy	EFO_0003770	Neurological disorder	Neurological	PGS001820	HP_0001943	Hypoglycemia	Hypoglycemia	HP_0001943	Other trait	Metabolic
0.8066	5.97E-106	1.14E-102	PGS003123	MONDO_0005148	type 2 diabetes mellitus	type 2 diabetes mellitus	MONDO_0005148	Digestive system disorder	Gastrointestinal	PGS003119	MONDO_0005148	type 2 diabetes mellitus	type 2 diabetes mellitus	MONDO_0005148	Metabolic disorder	Metabolic
0.8066	5.97E-106	1.14E-102	PGS003123	MONDO_0005148	type 2 diabetes mellitus	type 2 diabetes mellitus	MONDO_0005148	Digestive system disorder	Gastrointestinal	PGS003119	MONDO_0005148	type 2 diabetes mellitus	type 2 diabetes mellitus	MONDO_0005148	Digestive system disorder	Gastrointestinal
0.8066	5.97E-106	1.14E-102	PGS003123	MONDO_0005148	type 2 diabetes mellitus	type 2 diabetes mellitus	MONDO_0005148	Metabolic disorder	Metabolic	PGS003119	MONDO_0005148	type 2 diabetes mellitus	type 2 diabetes mellitus	MONDO_0005148	Metabolic disorder	Metabolic
0.8066	5.97E-106	1.14E-102	PGS003123	MONDO_0005148	type 2 diabetes mellitus	type 2 diabetes mellitus	MONDO_0005148	Metabolic disorder	Metabolic	PGS003119	MONDO_0005148	type 2 diabetes mellitus	type 2 diabetes mellitus	MONDO_0005148	Digestive system disorder	Gastrointestinal
0.8065	6.33E-106	1.21E-102	PGS001990	EFO_0004526	mean corpuscular volume	mean corpuscular volume	EFO_0004526	Hematological measurement	Hematological	PGS001220	EFO_0004526	mean corpuscular volume	mean corpuscular volume	EFO_0004526	Hematological measurement	Hematological
0.8065	6.47E-106	1.23E-102	PGS000912	EFO_0006336	diastolic blood pressure	diastolic blood pressure	EFO_0006336	Other measurement	Cardiovascular	PGS002114	EFO_0006336	diastolic blood pressure	diastolic blood pressure	EFO_0006336	Other measurement	Cardiovascular
0.8065	6.50E-106	1.24E-102	PGS000514	EFO_0000305	breast carcinoma	breast carcinoma	EFO_0000305	Cancer	Neoplasms	PGS000481	EFO_0000305	breast carcinoma	breast carcinoma	EFO_0000305	Cancer	Neoplasms
0.8065	6.53E-106	1.24E-102	PGS000440	MONDO_0002898	skin cancer	skin cancer	MONDO_0002898	Cancer	Neoplasms	PGS000444	MONDO_0002898	skin cancer	skin cancer	MONDO_0002898	Cancer	Neoplasms
0.8065	6.76E-106	1.29E-102	PGS001943	EFO_0004340	body mass index	body mass index	EFO_0004340	Body measurement	Body measurement	PGS002844	EFO_0004340	body mass index	body mass index	EFO_0004340	Body measurement	Body measurement

0.8064	6.97E-106	1.33E-102	PGS001406	EFO_0010700	reticulocyte measurement	reticulocyte measurement	EFO_0010700	Hematological measurement	Hematological	PGS001959	EFO_0010700	reticulocyte measurement	reticulocyte measurement	EFO_0010700	Hematological measurement	Hematological
0.8064	7.56E-106	1.44E-102	PGS000672	EFO_0004615	apolipoprotein B measurement	apolipoprotein B measurement	EFO_0004615	Lipid or lipoprotein measurement	Metabolic	PGS001933	EFO_0004611	low density lipoprotein cholesterol measurement	measurement	EFO_0004611	Lipid or lipoprotein measurement	Metabolic
0.8064	7.56E-106	1.44E-102	PGS000672	EFO_0004615	apolipoprotein B measurement	apolipoprotein B measurement	EFO_0004615	Lipid or lipoprotein measurement	Metabolic	PGS001933	EFO_0004611	low density lipoprotein cholesterol measurement	measurement	EFO_0004611	Cardiovascular measurement	Cardiovascular
0.8063	7.78E-106	1.48E-102	PGS000008	EFO_1000649	estrogen-receptor positive breast cancer	estrogen-receptor positive breast cancer	EFO_1000649	Cancer	Neoplasms	PGS000005	EFO_1000649	estrogen-receptor positive breast cancer	cancer	EFO_1000649	Cancer	Neoplasms
0.8063	8.27E-106	1.57E-102	PGS002056	EFO_1001395	Phlebitis|Thrombophlebitis	Phlebitis	EFO_1001395	Cardiovascular disease	Cardiovascular	PGS001847	EFO_0004264	vascular disease	vascular disease	EFO_0004264	Cardiovascular disease	Cardiovascular
0.8062	8.54E-106	1.63E-102	PGS003078	EFO_0006335	systolic blood pressure	systolic blood pressure	EFO_0006335	Other measurement	Cardiovascular	PGS002765	EFO_0000537	hypertension	hypertension	EFO_0000537	Cardiovascular disease	Cardiovascular
0.8062	9.13E-106	1.74E-102	PGS002851	EFO_0004340	body mass index	body mass index	EFO_0004340	Body measurement	Body measurement	PGS002857	EFO_0004340	body mass index	body mass index	EFO_0004340	Body measurement	Body measurement
0.8061	9.60E-106	1.83E-102	PGS002500	EFO_0007822	hair colour measurement	hair colour measurement	EFO_0007822	Other measurement	Dermatological	PGS002549	EFO_0007822	hair colour measurement	hair colour measurement	EFO_0007822	Other measurement	Dermatological
0.8061	1.01E-105	1.92E-102	PGS000564	EFO_0003893	ovarian neoplasm	ovarian neoplasm	EFO_0003893	Cancer	Neoplasms	PGS000552	EFO_0003893	ovarian neoplasm	ovarian neoplasm	EFO_0003893	Cancer	Neoplasms
0.8061	1.04E-105	1.98E-102	PGS001240	EFO_0004305	erythrocyte count	erythrocyte count	EFO_0004305	Hematological measurement	Hematological	PGS002345	EFO_0004305	erythrocyte count	erythrocyte count	EFO_0004305	Hematological measurement	Hematological
0.806	1.06E-105	2.02E-102	PGS000165	EFO_0004842	eosinophil count	eosinophil count	EFO_0004842	Hematological measurement	Hematological	PGS000166	EFO_0007991	eosinophil percentage of leukocytes	eosinophil percentage of leukocytes	EFO_0007991	Hematological measurement	Hematological
0.806	1.10E-105	2.08E-102	PGS000913	EFO_0006335	systolic blood pressure	systolic blood pressure	EFO_0006335	Other measurement	Cardiovascular	PGS002765	EFO_0000537	hypertension	hypertension	EFO_0000537	Cardiovascular disease	Cardiovascular
0.8059	1.17E-105	2.22E-102	PGS003119	MONDO_0005148	type 2 diabetes mellitus	type 2 diabetes mellitus	MONDO_0005148	Digestive system disorder	Gastrointestinal	PGS003108	MONDO_0005148	type 2 diabetes mellitus	type 2 diabetes mellitus	MONDO_0005148	Digestive system disorder	Gastrointestinal
0.8059	1.17E-105	2.22E-102	PGS003119	MONDO_0005148	type 2 diabetes mellitus	type 2 diabetes mellitus	MONDO_0005148	Digestive system disorder	Gastrointestinal	PGS003108	MONDO_0005148	type 2 diabetes mellitus	type 2 diabetes mellitus	MONDO_0005148	Metabolic disorder	Metabolic
0.8059	1.17E-105	2.22E-102	PGS003119	MONDO_0005148	type 2 diabetes mellitus	type 2 diabetes mellitus	MONDO_0005148	Metabolic disorder	Metabolic	PGS003108	MONDO_0005148	type 2 diabetes mellitus	type 2 diabetes mellitus	MONDO_0005148	Digestive system disorder	Gastrointestinal
0.8059	1.17E-105	2.22E-102	PGS003119	MONDO_0005148	type 2 diabetes mellitus	type 2 diabetes mellitus	MONDO_0005148	Metabolic disorder	Metabolic	PGS003108	MONDO_0005148	type 2 diabetes mellitus	type 2 diabetes mellitus	MONDO_0005148	Metabolic disorder	Metabolic
0.8059	1.21E-105	2.29E-102	PGS000538	EFO_0000305	breast carcinoma	breast carcinoma	EFO_0000305	Cancer	Neoplasms	PGS000530	EFO_0000305	breast carcinoma	breast carcinoma	EFO_0000305	Cancer	Neoplasms
0.8059	1.26E-105	2.38E-102	PGS001955	EFO_0009270	heel bone mineral density	heel bone mineral density	EFO_0009270	Other measurement	Skeletal	PGS000951	EFO_0004514	bone quantitative ultrasound measurement	measurement	EFO_0004514	Other measurement	Skeletal
0.8058	1.40E-105	2.66E-102	PGS001634	EFO_0005420	measurement	grey matter volume measurement	EFO_0005420	Other measurement	Neurological	PGS001545	EFO_0005420	measurement	grey matter volume measurement	EFO_0005420	Other measurement	Neurological
0.8058	1.42E-105	2.68E-102	PGS001864	MONDO_0024647	urolithiasis	urolithiasis	MONDO_0024647	Other disease	Genitourinary	PGS002075	MONDO_0024647	urolithiasis	urolithiasis	MONDO_0024647	Other disease	Genitourinary
0.8057	1.46E-105	2.76E-102	PGS000797	EFO_0002892	thyroid carcinoma	thyroid carcinoma	EFO_0002892	Cancer	Neoplasms	PGS000632	EFO_0002892	thyroid carcinoma	thyroid carcinoma	EFO_0002892	Cancer	Neoplasms
0.8057	1.46E-105	2.76E-102	PGS002835	EFO_0007878	alcohol consumption measurement	alcohol consumption measurement	EFO_0007878	Other measurement	Psychiatric	PGS000204	EFO_0007878	alcohol consumption measurement	alcohol consumption measurement	EFO_0007878	Other measurement	Psychiatric
0.8056	1.71E-105	3.23E-102	PGS003303	EFO_0004617	cystatin C measurement|glomerular filtration rate	cystatin C measurement	EFO_0004617	Other measurement	Metabolic	PGS003299	EFO_0004617	cystatin C measurement|glomerular filtration rate	cystatin C measurement	EFO_0004617	Other measurement	Metabolic
0.8055	1.86E-105	3.52E-102	PGS001388	EFO_0005671	smoking behaviour measurement	smoking behaviour measurement	EFO_0005671	Other measurement	Psychiatric	PGS001326	EFO_0000464	emphysema	emphysema	EFO_0000464	Other disease	Respiratory
0.8055	1.94E-105	3.67E-102	PGS000333	EFO_0001663	prostate carcinoma	prostate carcinoma	EFO_0001663	Cancer	Neoplasms	PGS000583	EFO_0001663	prostate carcinoma	prostate carcinoma	EFO_0001663	Cancer	Neoplasms
0.8054	1.98E-105	3.74E-102	PGS001781	MONDO_0005148	type 2 diabetes mellitus	type 2 diabetes mellitus	MONDO_0005148	Digestive system disorder	Gastrointestinal	PGS003104	MONDO_0005148	type 2 diabetes mellitus	type 2 diabetes mellitus	MONDO_0005148	Metabolic disorder	Metabolic
0.8054	1.98E-105	3.74E-102	PGS001781	MONDO_0005148	type 2 diabetes mellitus	type 2 diabetes mellitus	MONDO_0005148	Digestive system disorder	Gastrointestinal	PGS003104	MONDO_0005148	type 2 diabetes mellitus	type 2 diabetes mellitus	MONDO_0005148	Digestive system disorder	Gastrointestinal
0.8054	1.98E-105	3.74E-102	PGS001781	MONDO_0005148	type 2 diabetes mellitus	type 2 diabetes mellitus	MONDO_0005148	Metabolic disorder	Metabolic	PGS003104	MONDO_0005148	type 2 diabetes mellitus	type 2 diabetes mellitus	MONDO_0005148	Metabolic disorder	Metabolic
0.8054	1.98E-105	3.74E-102	PGS001781	MONDO_0005148	type 2 diabetes mellitus	type 2 diabetes mellitus	MONDO_0005148	Metabolic disorder	Metabolic	PGS003104	MONDO_0005148	type 2 diabetes mellitus	type 2 diabetes mellitus	MONDO_0005148	Digestive system disorder	Gastrointestinal
0.8054	2.05E-105	3.87E-102	PGS002802	EFO_0004339	body height	body height	EFO_0004339	Body measurement	Body measurement	PGS002332	EFO_0004339	body height	body height	EFO_0004339	Body measurement	Body measurement
0.8053	2.19E-105	4.13E-102	PGS002537	EFO_0008328	chronotype measurement	chronotype measurement	EFO_0008328	Other measurement	Psychiatric	PGS002390	EFO_0008328	chronotype measurement	chronotype measurement	EFO_0008328	Other measurement	Psychiatric
0.8053	2.24E-105	4.23E-102	PGS002765	EFO_0000537	hypertension	hypertension	EFO_0000537	Cardiovascular disease	Cardiovascular	PGS003073	EFO_0006335	systolic blood pressure	systolic blood pressure	EFO_0006335	Other measurement	Cardiovascular
0.8053	2.25E-105	4.25E-102	PGS000688	EFO_0004611	low density lipoprotein cholesterol measurement	low density lipoprotein cholesterol measurement	EFO_0004611	Lipid or lipoprotein measurement	Metabolic	PGS001889	EFO_0004615	apolipoprotein B measurement	apolipoprotein B measurement	EFO_0004615	Lipid or lipoprotein measurement	Metabolic
0.8053	2.25E-105	4.25E-102	PGS000688	EFO_0004611	low density lipoprotein cholesterol measurement	low density lipoprotein cholesterol measurement	EFO_0004611	Cardiovascular measurement	Cardiovascular	PGS001889	EFO_0004615	apolipoprotein B measurement	apolipoprotein B measurement	EFO_0004615	Lipid or lipoprotein measurement	Metabolic
0.8053	2.36E-105	4.46E-102	PGS002632	EFO_0003923	bone density	bone density	EFO_0003923	Other measurement	Skeletal	PGS002315	EFO_0003923	bone density	bone density	EFO_0003923	Other measurement	Skeletal
0.8052	2.64E-105	4.98E-102	PGS000114	EFO_0002609	juvenile idiopathic arthritis	juvenile idiopathic arthritis	EFO_0002609	Immune system disorder	Immunological	PGS000325	EFO_1002019	oligoarticular juvenile idiopathic arthritis	oligoarticular juvenile idiopathic arthritis	EFO_1002019	Immune system disorder	Immunological
0.8052	2.66E-105	5.02E-102	PGS002206	EFO_0004527	mean corpuscular hemoglobin	mean corpuscular hemoglobin	EFO_0004527	Hematological measurement	Hematological	PGS000099	EFO_0004527	mean corpuscular hemoglobin	mean corpuscular hemoglobin	EFO_0004527	Hematological measurement	Hematological
0.8051	2.74E-105	5.17E-102	PGS003119	MONDO_0005148	type 2 diabetes mellitus	type 2 diabetes mellitus	MONDO_0005148	Digestive system disorder	Gastrointestinal	PGS001781	MONDO_0005148	type 2 diabetes mellitus	type 2 diabetes mellitus	MONDO_0005148	Digestive system disorder	Gastrointestinal
0.8051	2.74E-105	5.17E-102	PGS003119	MONDO_0005148	type 2 diabetes mellitus	type 2 diabetes mellitus	MONDO_0005148	Metabolic disorder	Metabolic	PGS001781	MONDO_0005148	type 2 diabetes mellitus	type 2 diabetes mellitus	MONDO_0005148	Digestive system disorder	Gastrointestinal
0.8051	2.74E-105	5.17E-102	PGS003119	MONDO_0005148	type 2 diabetes mellitus	type 2 diabetes mellitus	MONDO_0005148	Metabolic disorder	Metabolic	PGS001781	MONDO_0005148	type 2 diabetes mellitus	type 2 diabetes mellitus	MONDO_0005148	Metabolic disorder	Metabolic
0.8051	2.74E-105	5.17E-102	PGS003119	MONDO_0005148	type 2 diabetes mellitus	type 2 diabetes mellitus	MONDO_0005148	Digestive system disorder	Gastrointestinal	PGS001781	MONDO_0005148	type 2 diabetes mellitus	type 2 diabetes mellitus	MONDO_0005148	Metabolic disorder	Metabolic
0.8051	2.81E-105	5.30E-102	PGS002005	EFO_0011011	sitting height measurement	sitting height measurement	EFO_0011011	Other measurement	Skeletal	PGS001929	EFO_0004339	body height	body height	EFO_0004339	Body measurement	Body measurement
0.8051	2.91E-105	5.47E-102	PGS000333	EFO_0001663	prostate carcinoma	prostate carcinoma	EFO_0001663	Cancer	Neoplasms	PGS000584	EFO_0001663	prostate carcinoma	prostate carcinoma	EFO_0001663	Cancer	Neoplasms
0.8051	2.93E-105	5.52E-102	PGS002938	EFO_0004468	glucose measurement	glucose measurement	EFO_0004468	Other measurement	Metabolic	PGS002943	EFO_0004468	glucose measurement	glucose measurement	EFO_0004468	Other measurement	Metabolic
0.805	2.99E-105	5.62E-102	PGS002194	EFO_0007986	reticulocyte count	reticulocyte count	EFO_0007986	Hematological measurement	Hematological	PGS002333	EFO_0007986	reticulocyte count	reticulocyte count	EFO_0007986	Hematological measurement	Hematological
0.805	3.18E-105	5.98E-102	PGS001671	EFO_0006930	brain volume measurement|neuroimaging measurement	brain volume measurement	EFO_0006930	Other measurement	Neurological	PGS001680	EFO_0006930	measurement	brain volume measurement	EFO_0006930	Other measurement	Neurological
0.8049	3.40E-105	6.38E-102	PGS003331	EFO_0001663	prostate carcinoma	prostate carcinoma	EFO_0001663	Cancer	Neoplasms	PGS002796	EFO_0001663	prostate carcinoma	prostate carcinoma	EFO_0001663	Cancer	Neoplasms
0.8049	3.42E-105	6.43E-102	PGS002963	EFO_0004612	high density lipoprotein cholesterol measurement	high density lipoprotein cholesterol measurement	EFO_0004612	Lipid or lipoprotein measurement	Metabolic	PGS002959	EFO_0004612	high density lipoprotein cholesterol measurement	measurement	EFO_0004612	Lipid or lipoprotein measurement	Metabolic
0.8047	4.24E-105	7.97E-102	PGS000731	EFO_0000707	squamous cell carcinoma	squamous cell carcinoma	EFO_0000707	Cancer	Neoplasms	PGS000950	GO_0009314	response to radiation	response to radiation	GO_0009314	Biological process	Others
0.8047	4.38E-105	8.22E-102	PGS003131	EFO_0004340	body mass index	body mass index	EFO_0004340	Body measurement	Body measurement	PGS003130	EFO_0004340	body mass index	body mass index	EFO_0004340	Body measurement	Body measurement
0.8047	4.44E-105	8.33E-102	PGS002485	EFO_0003923	bone density	bone density	EFO_0003923	Other measurement	Skeletal	PGS002436	EFO_0003923	bone density	bone density	EFO_0003923	Other measurement	Skeletal
0.8047	4.49E-105	8.41E-102	PGS002432	MONDO_0004979	asthma	asthma	MONDO_0004979	Other disease	Respiratory	PGS002481	MONDO_0004979	asthma	asthma	MONDO_0004979	Other disease	Respiratory
0.8046	4.74E-105	8.88E-102	PGS003189	EFO_0004631	vitamin D measurement	vitamin D measurement	EFO_0004631	Lipid or lipoprotein measurement	Metabolic	PGS002427	EFO_0004631	vitamin D measurement	vitamin D measurement	EFO_0004631	Lipid or lipoprotein measurement	Metabolic
0.8045	5.09E-105	9.54E-102	PGS001943	EFO_0004340	body mass index	body mass index	EFO_0004340	Body measurement	Body measurement	PGS002849	EFO_0004340	body mass index	body mass index	EFO_0004340	Body measurement	Body measurement
0.8045	5.45E-105	1.02E-101	PGS000212	MONDO_0021116	luminal A breast carcinoma	luminal A breast carcinoma	MONDO_0021116	Cancer	Neoplasms	PGS000004	EFO_0000305	breast carcinoma	breast carcinoma	EFO_0000305	Cancer	Neoplasms
0.8043	6.84E-105	1.28E-101	PGS000950	GO_0009314	response to radiation	response to radiation	GO_0009314	Biological process	Others	PGS001257	EFO_0003958	sunburn	sunburn	EFO_0003958	Other disease	Dermatological
0.8042	7.33E-105	1.37E-101	PGS002654	EFO_0004611	low density lipoprotein cholesterol measurement	low density lipoprotein cholesterol measurement	EFO_0004611	Lipid or lipoprotein measurement	Metabolic	PGS001889	EFO_0004615	apolipoprotein B measurement	apolipoprotein B measurement	EFO_0004615	Lipid or lipoprotein measurement	Metabolic
0.8042	7.33E-105	1.37E-101	PGS002654	EFO_0004611	low density lipoprotein cholesterol measurement	low density lipoprotein cholesterol measurement	EFO_0004611	Cardiovascular measurement	Cardiovascular	PGS001889	EFO_0004615	apolipoprotein B measurement	apolipoprotein B measurement	EFO_0004615	Lipid or lipoprotein measurement	Metabolic
0.8042	7.42E-105	1.39E-101	PGS002329	EFO_0004612	high density lipoprotein cholesterol measurement	high density lipoprotein cholesterol measurement	EFO_0004612	Lipid or lipoprotein measurement	Metabolic	PGS002963	EFO_0004612	high density lipoprotein cholesterol measurement	measurement	EFO_0004612	Lipid or lipoprotein measurement	Metabolic
0.8041	8.36E-105	1.56E-101	PGS000510	EFO_0000305	breast carcinoma	breast carcinoma	EFO_0000305	Cancer	Neoplasms	PGS000505	EFO_0000305	breast carcinoma	breast carcinoma	EFO_0000305	Cancer	Neoplasms
0.8041	8.36E-105	1.56E-101	PGS000510	EFO_0000305	breast carcinoma	breast carcinoma	EFO_0000305	Cancer	Neoplasms	PGS000506	EFO_0000305	breast carcinoma	breast carcinoma	EFO_0000305	Cancer	Neoplasms
0.804	8.76E-105	1.64E-101	PGS000213	EFO_0009780	HER2 negative breast carcinoma	HER2 negative breast carcinoma	EFO_0009780	Cancer	Neoplasms	PGS003399	EFO_0000305	breast carcinoma	breast carcinoma	EFO_0000305	Cancer	Neoplasms
0.804	9.07E-105	1.69E-101	PGS001780	EFO_0001645	coronary artery disease	coronary artery disease	EFO_0001645	Cardiovascular disease	Cardiovascular	PGS003355	EFO_0001645	coronary artery disease	coronary artery disease	EFO_0001645	Cardiovascular disease	Cardiovascular
0.8039	9.51E-105	1.78E-101	PGS002875	EFO_0004458	C-reactive protein measurement	C-reactive protein measurement	EFO_0004458	Inflammatory measurement	Immunological	PGS002884	EFO_0004458	C-reactive protein measurement	C-reactive protein measurement	EFO_0004458	Inflammatory measurement	Immunological
0.8039	9.51E-105	1.78E-101	PGS002876	EFO_0004458	C-reactive protein measurement	C-reactive protein measurement	EFO_0004458	Inflammatory measurement	Immunological	PGS002884	EFO_0004458	C-reactive protein measurement	C-reactive protein measurement	EFO_0004458	Inflammatory measurement	Immunological
0.8039	1.03E-104	1.92E-101	PGS000433	MONDO_0002898	skin cancer	skin cancer	MONDO_0002898	Cancer	Neoplasms	PGS000435	MONDO_0002898	skin cancer	skin cancer	MONDO_0002898	Cancer	Neoplasms
0.8038	1.05E-104	1.96E-101	PGS000344	EFO_0000305	breast carcinoma	breast carcinoma	EFO_0000305	Cancer	Neoplasms	PGS000346	EFO_1000650	estrogen-receptor negative breast cancer	cancer	EFO_1000650	Cancer	Neoplasms
0.8038	1.06E-104	1.98E-101	PGS002765	EFO_0000537	hypertension	hypertension	EFO_0000537	Cardiovascular disease	Cardiovascular	PGS002349	EFO_0006335	systolic blood pressure	systolic blood pressure	EFO_0006335	Other measurement	Cardiovascular
0.8037	1.25E-104	2.34E-101	PGS001989	EFO_0004527	mean corpuscular hemoglobin	mean corpuscular hemoglobin	EFO_0004527	Hematological measurement	Hematological	PGS000099	EFO_0004527	mean corpuscular hemoglobin	mean corpuscular hemoglobin	EFO_0004527	Hematological measurement	Hematological
0.8036	1.34E-104	2.50E-101	PGS000048	EFO_0001075	ovarian carcinoma	ovarian carcinoma	EFO_0001075	Cancer	Neoplasms	PGS000068	EFO_0001075	ovarian carcinoma	ovarian carcinoma	EFO_0001075	Cancer	Neoplasms
0.8036	1.39E-104	2.59E-101	PGS002161	EFO_0004340	body mass index	body mass index	EFO_0004340	Body measurement	Body measurement	PGS002845	EFO_0004340	body mass index	body mass index	EFO_0004340	Body measurement	Body measurement
0.8035	1.43E-104	2.67E-101	PGS001079	EFO_0007984	platelet component distribution width	platelet component distribution width	EFO_0007984	Hematological measurement	Hematological	PGS000108	EFO_0007984	platelet component distribution width	platelet component distribution width	EFO_0007984	Hematological measurement	Hematological
0.8034	1.64E-104	3.06E-101	PGS002521	EFO_1000627	thyroid disease	thyroid disease	EFO_1000627	Other disease	Endocrine	PGS002472	EFO_1000627	thyroid disease	thyroid disease	EFO_1000627	Other disease	Endocrine
0.8034	1.65E-104	3.06E-101	PGS003268	EFO_0010472	creatine measurement|glomerular filtration rate	creatine measurement	EFO_0010472	Other measurement	Metabolic	PGS003264	EFO_0010472	creatine measurement|glomerular filtration rate	creatine measurement	EFO_0010472	Other measurement	Metabolic
0.8033	1.83E-104	3.40E-101	PGS001467	EFO_0006930	brain volume measurement|neuroimaging measurement	brain volume measurement	EFO_0006930	Other measurement	Neurological	PGS001468	EFO_0006930	measurement	brain volume measurement	EFO_0006930	Other measurement	Neurological
0.8032	2.02E-104	3.74E-101	PGS000783	EFO_0000305	breast carcinoma	breast carcinoma	EFO_0000305	Cancer	Neoplasms	PGS000045	EFO_0000305	breast carcinoma	breast carcinoma	EFO_0000305	Cancer	Neoplasms
0.8032	2.04E-104	3.78E-101	PGS002991	EFO_0004339	body height	body height	EFO_0004339	Body measurement	Body measurement	PGS002980	EFO_0004339	body height	body height	EFO_0004339	Body measurement	Body measurement
0.8032	2.06E-104	3.83E-101	PGS001729	EFO_0006930	brain volume measurement|neuroimaging measurement	brain volume measurement	EFO_0006930	Other measurement	Neurological	PGS001714	EFO_0006930	measurement	brain volume measurement	EFO_0006930	Other measurement	Neurological
0.8032	2.12E-104	3.93E-101	PGS000569	EFO_0001663	prostate carcinoma	prostate carcinoma	EFO_0001663	Cancer	Neoplasms	PGS000591	EFO_0001663	prostate carcinoma	prostate carcinoma	EFO_0001663	Cancer	Neoplasms
0.8031	2.20E-104	4.07E-101	PGS002815	EFO_0004703	age at menarche	age at menarche	EFO_0004703	Other measurement	Genitourinary	PGS002819	EFO_0004703	age at menarche	age at menarche	EFO_0004703	Other measurement	Genitourinary
0.8031	2.30E-104	4.26E-101	PGS002783	EFO_0004574	total cholesterol measurement	total cholesterol measurement	EFO_0004574	Lipid or lipoprotein measurement	Metabolic	PGS000889	EFO_0004611	low density lipoprotein cholesterol measurement	measurement	EFO_0004611	Cardiovascular measurement	Cardiovascular
0.8031	2.30E-104	4.26E-101	PGS002783	EFO_0004574	total cholesterol measurement	total cholesterol measurement	EFO_0004574	Lipid or lipoprotein measurement	Metabolic	PGS000889	EFO_0004611	low density lipoprotein cholesterol measurement	measurement	EFO_0004611	Lipid or lipoprotein measurement	Metabolic
0.8029	2.73E-104	5.06E-101	PGS003274	EFO_0010472	creatine measurement|glomerular filtration rate	creatine measurement	EFO_0010472	Other measurement	Metabolic	PGS003278	EFO_0010472	creatine measurement|glomerular filtration rate	creatine measurement	EFO_0010472	Other measurement	Metabolic
0.8028	3.23E-104	5.98E-101	PGS003035	EFO_0004611	low density lipoprotein cholesterol measurement	low density lipoprotein cholesterol measurement	EFO_0004611	Cardiovascular measurement	Cardiovascular	PGS003141	EFO_0004574	total cholesterol measurement	total cholesterol measurement	EFO_0004574	Lipid or lipoprotein measurement	Metabolic
0.8028	3.23E-104	5.98E-101	PGS003035	EFO_0004611	low density lipoprotein cholesterol measurement	low density lipoprotein cholesterol measurement	EFO_0004611	Lipid or lipoprotein measurement	Metabolic	PGS003141	EFO_0004574	total cholesterol measurement	total cholesterol measurement	EFO_0004574	Lipid or lipoprotein measurement	Metabolic
0.8027	3.31E-104	6.12E-101	PGS001979	EFO_0004530	triglyceride measurement	triglyceride measurement	EFO_0004530	Lipid or lipoprotein measurement	Metabolic	PGS003153	EFO_0004530	triglyceride measurement	triglyceride measurement	EFO_0004530	Lipid or lipoprotein measurement	Metabolic
0.8027	3.43E-104	6.35E-101	PGS002501	EFO_0004541	HbA1c measurement	HbA1c measurement	EFO_0004541	Hematological measurement	Hematological	PGS002475	MONDO_0005148	type 2 diabetes mellitus	type 2 diabetes mellitus	MONDO_0005148	Metabolic disorder	Metabolic
0.8027	3.43E-104	6.35E-101	PGS002501	EFO_0004541	HbA1c measurement	HbA1c measurement	EFO_0004541	Hematological measurement	Hematological	PGS002475	MONDO_0005148	type 2 diabetes mellitus	type 2 diabetes mellitus	MONDO_0005148	Digestive system disorder	Gastrointestinal
0.8026	3.67E-104	6.79E-101	PGS003278	EFO_0010472	creatine measurement|glomerular filtration rate	creatine measurement	EFO_0010472	Other measurement	Metabolic	PGS003259	EFO_0010472	creatine measurement|glomerular filtration rate	creatine measurement	EFO_0010472	Other measurement	Metabolic
0.8026	3.83E-104	7.08E-101	PGS001360	EFO_0004731	eye measurement	eye measurement	EFO_0004731	Other measurement	Ophthalmological	PGS001367	EFO_0004731	eye measurement	eye measurement	EFO_0004731	Other measurement	Ophthalmological
-0.8025	4.19E-104	7.75E-101	PGS000061	EFO_0004611	low density lipoprotein cholesterol measurement	low density lipoprotein cholesterol measurement	EFO_0004611	Lipid or lipoprotein measurement	Metabolic	PGS000115	EFO_0004611	low density lipoprotein cholesterol measurement	measurement	EFO_0004611	Cardiovascular measurement	Cardiovascular
-0.8025	4.19E-104	7.75E-101	PGS000061	EFO_0004611	low density lipoprotein cholesterol measurement	low density lipoprotein cholesterol measurement	EFO_0004611	Cardiovascular measurement	Cardiovascular	PGS000115	EFO_0004611	low density lipoprotein cholesterol measurement	measurement	EFO_0004611	Lipid or lipoprotein measurement	Metabolic
-0.8025	4.19E-104	7.75E-101	PGS000061	EFO_0004611	low density lipoprotein cholesterol measurement	low density lipoprotein cholesterol measurement	EFO_0004611	Cardiovascular measurement	Cardiovascular	PGS000115	EFO_0004611	low density lipoprotein cholesterol measurement	measurement	EFO_0004611	Cardiovascular measurement	Cardiovascular
-0.8025	4.19E-104	7.75E-101	PGS000061	EFO_0004611	low density lipoprotein cholesterol measurement	low density lipoprotein cholesterol measurement	EFO_0004611	Lipid or lipoprotein measurement	Metabolic	PGS000115	EFO_0004611	low density lipoprotein cholesterol measurement	measurement	EFO_0004611	Lipid or lipoprotein measurement	Metabolic
0.8025	4.20E-104	7.76E-101	PGS003295	EFO_0004617	cystatin C measurement|glomerular filtration rate	cystatin C measurement	EFO_0004617	Other measurement	Metabolic	PGS003310	EFO_0004617	cystatin C measurement|glomerular filtration rate	cystatin C measurement	EFO_0004617	Other measurement	Metabolic
0.8025	4.29E-104	7.93E-101	PGS003035	EFO_0004611	low density lipoprotein cholesterol measurement	low density lipoprotein cholesterol measurement	EFO_0004611	Lipid or lipoprotein measurement	Metabolic	PGS003136	EFO_0004574	total cholesterol measurement	total cholesterol measurement	EFO_0004574	Lipid or lipoprotein measurement	Metabolic
0.8025	4.29E-104	7.93E-101	PGS003035	EFO_0004611	low density lipoprotein cholesterol measurement	low density lipoprotein cholesterol measurement	EFO_0004611	Cardiovascular measurement	Cardiovascular	PGS003136	EFO_0004574	total cholesterol measurement	total cholesterol measurement	EFO_0004574	Lipid or lipoprotein measurement	Metabolic
0.8023	5.12E-104	9.46E-101	PGS002835	EFO_0007878	alcohol consumption measurement	alcohol consumption measurement	EFO_0007878	Other measurement	Psychiatric	PGS002839	EFO_0007878	alcohol consumption measurement	alcohol consumption measurement	EFO_0007878	Other measurement	Psychiatric
0.8022	5.49E-104	1.01E-100	PGS000677	EFO_0004574	total cholesterol measurement	total cholesterol measurement	EFO_0004574	Lipid or lipoprotein measurement	Metabolic	PGS002352	EFO_0004574	total cholesterol measurement	total cholesterol measurement	EFO_0004574	Lipid or lipoprotein measurement	Metabolic
0.8021	6.03E-104	1.11E-100	PGS000955	EFO_0004514	bone quantitative ultrasound measurement	bone quantitative ultrasound measurement	EFO_0004514	Other measurement	Skeletal	PGS001530	EFO_0007772	measurement	measurement	EFO_0007772	Other measurement	Skeletal
0.8021	6.21E-104	1.15E-100	PGS003235	EFO_0010472	creatine measurement	creatine measurement	EFO_0010472	Other measurement	Metabolic	PGS003236	EFO_0010472	creatine measurement	creatine measurement	EFO_0010472	Other measurement	Metabolic
0.802	7.16E-104	1.32E-100	PGS002241	EFO_0001663	prostate carcinoma	prostate carcinoma	EFO_0001663	Cancer	Neoplasms	PGS002240	EFO_0001663	prostate carcinoma	prostate carcinoma	EFO_0001663	Cancer	Neoplasms
0.8019	7.80E-104	1.44E-100	PGS000569	EFO_0001663	prostate carcinoma	prostate carcinoma	EFO_0001663	Cancer	Neoplasms	PGS000573	EFO_0001663	prostate carcinoma	prostate carcinoma	EFO_0001663	Cancer	Neoplasms

0.8018	8.32E-104	1.53E-100	PGS001149	EFO_0005409	fat body mass	fat body mass	EFO_0005409	Other measurement	Body measurement	PGS001103	EFO_0007800	body fat percentage	body fat percentage	EFO_0007800	Body measurement	Body measurement
0.8018	8.57E-104	1.58E-100	PGS001301	EFO_0001060	celiac disease	celiac disease	EFO_0001060	Immune system disorder	Immunological	PGS000040	EFO_0001060	celiac disease	celiac disease	EFO_0001060	Immune system disorder	Immunological
0.8018	8.57E-104	1.58E-100	PGS001301	EFO_0001060	celiac disease	celiac disease	EFO_0001060	Immune system disorder	Immunological	PGS000040	EFO_0001060	celiac disease	celiac disease	EFO_0001060	Digestive system disorder	Gastrointestinal
0.8018	8.57E-104	1.58E-100	PGS001301	EFO_0001060	celiac disease	celiac disease	EFO_0001060	Digestive system disorder	Gastrointestinal	PGS000040	EFO_0001060	celiac disease	celiac disease	EFO_0001060	Immune system disorder	Immunological
0.8018	8.57E-104	1.58E-100	PGS001301	EFO_0001060	celiac disease	celiac disease	EFO_0001060	Digestive system disorder	Gastrointestinal	PGS000040	EFO_0001060	celiac disease	celiac disease	EFO_0001060	Digestive system disorder	Gastrointestinal
0.8018	9.01E-104	1.66E-100	PGS002471	EFO_0004279	suntan	suntan	EFO_0004279	Other trait	Dermatological	PGS002520	EFO_0004279	suntan	suntan	EFO_0004279	Other trait	Dermatological
0.8017	9.15E-104	1.68E-100	PGS002165	EFO_0004617	cystatin C measurement	cystatin C measurement	EFO_0004617	Other measurement	Metabolic	PGS000680	EFO_0004617	cystatin C measurement	cystatin C measurement	EFO_0004617	Other measurement	Metabolic
-0.8017	9.29E-104	1.71E-100	PGS002318	EFO_0008328	chronotype measurement	chronotype measurement	EFO_0008328	Other measurement	Psychiatric	PGS002209	EFO_0008328	chronotype measurement	chronotype measurement	EFO_0008328	Other measurement	Psychiatric
0.8017	9.34E-104	1.72E-100	PGS000385	EFO_0002618	pancreatic carcinoma	pancreatic carcinoma	EFO_0002618	Cancer	Neoplasms	PGS000794	EFO_0002618	pancreatic carcinoma	pancreatic carcinoma	EFO_0002618	Cancer	Neoplasms
0.8017	9.34E-104	1.72E-100	PGS000385	EFO_0002618	pancreatic carcinoma	pancreatic carcinoma	EFO_0002618	Digestive system disorder	Gastrointestinal	PGS000794	EFO_0002618	pancreatic carcinoma	pancreatic carcinoma	EFO_0002618	Cancer	Neoplasms
0.8017	9.34E-104	1.72E-100	PGS000385	EFO_0002618	pancreatic carcinoma	pancreatic carcinoma	EFO_0002618	Digestive system disorder	Gastrointestinal	PGS000794	EFO_0002618	pancreatic carcinoma	pancreatic carcinoma	EFO_0002618	Digestive system disorder	Gastrointestinal
0.8017	9.34E-104	1.72E-100	PGS000385	EFO_0002618	pancreatic carcinoma	pancreatic carcinoma	EFO_0002618	Cancer	Neoplasms	PGS000794	EFO_0002618	pancreatic carcinoma	pancreatic carcinoma	EFO_0002618	Digestive system disorder	Gastrointestinal
0.8017	9.50E-104	1.75E-100	PGS002970	EFO_0004339	body height	body height	EFO_0004339	Body measurement	Body measurement	PGS002968	EFO_0004339	body height	body height	EFO_0004339	Body measurement	Body measurement
0.8016	1.07E-103	1.96E-100	PGS001521	EFO_0004462	PR interval	PR interval	EFO_0004462	Cardiovascular measurement	Cardiovascular	PGS001904	EFO_0004462	PR interval	PR interval	EFO_0004462	Cardiovascular measurement	Cardiovascular
0.8016	1.07E-103	1.97E-100	PGS001847	EFO_0004264	vascular disease	vascular disease	EFO_0004264	Cardiovascular disease	Cardiovascular	PGS001280	EFO_0003827	pulmonary embolism	pulmonary embolism	EFO_0003827	Cardiovascular disease	Cardiovascular
0.8015	1.11E-103	2.04E-100	PGS003137	EFO_0004574	total cholesterol measurement	total cholesterol measurement	EFO_0004574	Lipid or lipoprotein measurement	Metabolic	PGS003037	EFO_0004611	low density lipoprotein cholesterol measurement	measurement	EFO_0004611	Lipid or lipoprotein measurement	Metabolic
0.8015	1.11E-103	2.04E-100	PGS003137	EFO_0004574	total cholesterol measurement	total cholesterol measurement	EFO_0004574	Lipid or lipoprotein measurement	Metabolic	PGS003037	EFO_0004611	low density lipoprotein cholesterol measurement	measurement	EFO_0004611	Cardiovascular measurement	Cardiovascular
0.8015	1.15E-103	2.10E-100	PGS000783	EFO_0000305	breast carcinoma	breast carcinoma	EFO_0000305	Cancer	Neoplasms	PGS000046	EFO_1000649	estrogen-receptor positive breast cancer	cancer	EFO_1000649	Cancer	Neoplasms
0.8015	1.21E-103	2.22E-100	PGS000474	EFO_0000305	breast carcinoma	breast carcinoma	EFO_0000305	Cancer	Neoplasms	PGS000480	EFO_0000305	breast carcinoma	breast carcinoma	EFO_0000305	Cancer	Neoplasms
0.8014	1.29E-103	2.36E-100	PGS000433	MONDO_0002898	skin cancer	skin cancer	MONDO_0002898	Cancer	Neoplasms	PGS000441	MONDO_0002898	skin cancer	skin cancer	MONDO_0002898	Cancer	Neoplasms
0.8014	1.29E-103	2.36E-100	PGS000988	EFO_0010700	reticulocyte measurement	reticulocyte measurement	EFO_0010700	Hematological measurement	Hematological	PGS000989	EFO_0010700	reticulocyte measurement	reticulocyte measurement	EFO_0010700	Hematological measurement	Hematological
0.8013	1.40E-103	2.56E-100	PGS002841	EFO_0004340	body mass index	body mass index	EFO_0004340	Body measurement	Body measurement	PGS002857	EFO_0004340	body mass index	body mass index	EFO_0004340	Body measurement	Body measurement
0.8013	1.46E-103	2.68E-100	PGS002970	EFO_0004339	body height	body height	EFO_0004339	Body measurement	Body measurement	PGS002973	EFO_0004339	body height	body height	EFO_0004339	Body measurement	Body measurement
0.8013	1.49E-103	2.73E-100	PGS001895	EFO_0004574	total cholesterol measurement	total cholesterol measurement	EFO_0004574	Lipid or lipoprotein measurement	Metabolic	PGS002102	EFO_0004615	apolipoprotein B measurement	apolipoprotein B measurement	EFO_0004615	Lipid or lipoprotein measurement	Metabolic
0.8012	1.51E-103	2.76E-100	PGS002500	EFO_0007822	hair colour measurement	hair colour measurement	EFO_0007822	Other measurement	Dermatological	PGS002598	EFO_0007822	hair colour measurement	hair colour measurement	EFO_0007822	Other measurement	Dermatological
0.8012	1.55E-103	2.84E-100	PGS002754	MONDO_0004979	asthma	asthma	MONDO_0004979	Other disease	Respiratory	PGS002347	EFO_0000684	respiratory system disease	respiratory system disease	EFO_0000684	Other disease	Skeletal
0.801	1.85E-103	3.39E-100	PGS000001	EFO_0000305	breast carcinoma	breast carcinoma	EFO_0000305	Cancer	Neoplasms	PGS000873	EFO_0000305	breast carcinoma	breast carcinoma	EFO_0000305	Cancer	Neoplasms
0.801	2.00E-103	3.67E-100	PGS000507	EFO_0000305	breast carcinoma	breast carcinoma	EFO_0000305	Cancer	Neoplasms	PGS000535	EFO_0000305	breast carcinoma	breast carcinoma	EFO_0000305	Cancer	Neoplasms
0.8009	2.23E-103	4.08E-100	PGS001484	EFO_0006930	brain volume measurement|neuroimaging measurement	brain volume measurement	EFO_0006930	Other measurement	Neurological	PGS001451	EFO_0006930	measurement	brain volume measurement	EFO_0006930	Other measurement	Neurological
0.8008	2.34E-103	4.28E-100	PGS001103	EFO_0007800	body fat percentage	body fat percentage	EFO_0007800	Body measurement	Body measurement	PGS001105	EFO_0007800	body fat percentage	body fat percentage	EFO_0007800	Body measurement	Body measurement
0.8008	2.37E-103	4.32E-100	PGS002596	EFO_0004468	glucose measurement	glucose measurement	EFO_0004468	Other measurement	Metabolic	PGS002599	EFO_0004541	HbA1c measurement	HbA1c measurement	EFO_0004541	Hematological measurement	Hematological
0.8008	2.43E-103	4.44E-100	PGS001687	EFO_0006930	brain volume measurement|neuroimaging measurement	brain volume measurement	EFO_0006930	Other measurement	Neurological	PGS001686	EFO_0006930	measurement	brain volume measurement	EFO_0006930	Other measurement	Neurological
0.8008	2.47E-103	4.51E-100	PGS000196	MONDO_0007915	systemic lupus erythematosus	systemic lupus erythematosus	MONDO_0007915	Immune system disorder	Immunological	PGS000803	MONDO_0007915	systemic lupus erythematosus	systemic lupus erythematosus	MONDO_0007915	Immune system disorder	Immunological
0.8008	2.49E-103	4.54E-100	PGS000900	EFO_0006335	systolic blood pressure|female	systolic blood pressure	EFO_0006335	Other measurement	Cardiovascular	PGS002807	EFO_0006335	systolic blood pressure	systolic blood pressure	EFO_0006335	Other measurement	Cardiovascular
0.8007	2.72E-103	4.97E-100	PGS001117	EFO_0600082	insulin use measurement	insulin use measurement	EFO_0600082	Other measurement	Metabolic	PGS002025	MONDO_0005147	type 1 diabetes mellitus	type 1 diabetes mellitus	MONDO_0005147	Digestive system disorder	Gastrointestinal
0.8007	2.72E-103	4.97E-100	PGS001117	EFO_0600082	insulin use measurement	insulin use measurement	EFO_0600082	Other measurement	Metabolic	PGS002025	MONDO_0005147	type 1 diabetes mellitus	type 1 diabetes mellitus	MONDO_0005147	Metabolic disorder	Metabolic
0.8007	2.72E-103	4.97E-100	PGS001117	EFO_0600082	insulin use measurement	insulin use measurement	EFO_0600082	Other measurement	Metabolic	PGS002025	MONDO_0005147	type 1 diabetes mellitus	type 1 diabetes mellitus	MONDO_0005147	Immune system disorder	Immunological
0.8006	2.99E-103	5.45E-100	PGS002835	EFO_0007878	alcohol consumption measurement	alcohol consumption measurement	EFO_0007878	Other measurement	Psychiatric	PGS000203	EFO_0007878	alcohol consumption measurement	alcohol consumption measurement	EFO_0007878	Other measurement	Psychiatric
0.8005	3.18E-103	5.80E-100	PGS001484	EFO_0006930	brain volume measurement|neuroimaging measurement	brain volume measurement	EFO_0006930	Other measurement	Neurological	PGS001450	EFO_0006930	measurement	brain volume measurement	EFO_0006930	Other measurement	Neurological
0.8005	3.24E-103	5.89E-100	PGS002856	EFO_0004340	body mass index	body mass index	EFO_0004340	Body measurement	Body measurement	PGS002842	EFO_0004340	body mass index	body mass index	EFO_0004340	Body measurement	Body measurement
0.8005	3.30E-103	6.01E-100	PGS002869	EFO_0004458	C-reactive protein measurement	C-reactive protein measurement	EFO_0004458	Inflammatory measurement	Immunological	PGS002873	EFO_0004458	C-reactive protein measurement	C-reactive protein measurement	EFO_0004458	Inflammatory measurement	Immunological
0.8005	3.36E-103	6.12E-100	PGS000190	EFO_0007986	reticulocyte count	reticulocyte count	EFO_0007986	Hematological measurement	Hematological	PGS000170	EFO_0007986	reticulocyte count	reticulocyte count	EFO_0007986	Hematological measurement	Hematological
0.8005	3.37E-103	6.13E-100	PGS001956	EFO_0007772	calcaneal bone quantitative ultrasound measurement	calcaneal bone quantitative ultrasound measurement	EFO_0007772	Other measurement	Skeletal	PGS001402	EFO_0009270	heel bone mineral density	heel bone mineral density	EFO_0009270	Other measurement	Skeletal
0.8004	3.47E-103	6.31E-100	PGS003054	EFO_0006527	smoking status measurement	smoking status measurement	EFO_0006527	Other measurement	Psychiatric	PGS003058	EFO_0006527	smoking status measurement	smoking status measurement	EFO_0006527	Other measurement	Psychiatric
0.8004	3.70E-103	6.73E-100	PGS000335	EFO_0000305	breast carcinoma	breast carcinoma	EFO_0000305	Cancer	Neoplasms	PGS000536	EFO_0000305	breast carcinoma	breast carcinoma	EFO_0000305	Cancer	Neoplasms
0.8003	3.94E-103	7.15E-100	PGS002337	EFO_0004611	low density lipoprotein cholesterol measurement	low density lipoprotein cholesterol measurement	EFO_0004611	Cardiovascular measurement	Cardiovascular	PGS003033	EFO_0004611	low density lipoprotein cholesterol measurement	measurement	EFO_0004611	Cardiovascular measurement	Cardiovascular
0.8003	3.94E-103	7.15E-100	PGS002337	EFO_0004611	low density lipoprotein cholesterol measurement	low density lipoprotein cholesterol measurement	EFO_0004611	Lipid or lipoprotein measurement	Metabolic	PGS003033	EFO_0004611	low density lipoprotein cholesterol measurement	measurement	EFO_0004611	Lipid or lipoprotein measurement	Metabolic
0.8003	3.94E-103	7.15E-100	PGS002337	EFO_0004611	low density lipoprotein cholesterol measurement	low density lipoprotein cholesterol measurement	EFO_0004611	Lipid or lipoprotein measurement	Metabolic	PGS003033	EFO_0004611	low density lipoprotein cholesterol measurement	measurement	EFO_0004611	Cardiovascular measurement	Cardiovascular
0.8003	3.94E-103	7.15E-100	PGS002337	EFO_0004611	low density lipoprotein cholesterol measurement	low density lipoprotein cholesterol measurement	EFO_0004611	Cardiovascular measurement	Cardiovascular	PGS003033	EFO_0004611	low density lipoprotein cholesterol measurement	measurement	EFO_0004611	Lipid or lipoprotein measurement	Metabolic
0.8001	4.93E-103	8.95E-100	PGS002595	EFO_0004312	vital capacity	vital capacity	EFO_0004312	Other measurement	Activities	PGS002399	EFO_0004312	vital capacity	vital capacity	EFO_0004312	Other measurement	Activities
0.8001	5.01E-103	9.09E-100	PGS002339	EFO_0004527	mean corpuscular hemoglobin	mean corpuscular hemoglobin	EFO_0004527	Hematological measurement	Hematological	PGS000101	EFO_0004526	mean corpuscular volume	mean corpuscular volume	EFO_0004526	Hematological measurement	Hematological
0.8	5.28E-103	9.58E-100	PGS002961	EFO_0004612	high density lipoprotein cholesterol measurement	high density lipoprotein cholesterol measurement	EFO_0004612	Lipid or lipoprotein measurement	Metabolic	PGS002955	EFO_0004612	high density lipoprotein cholesterol measurement	measurement	EFO_0004612	Lipid or lipoprotein measurement	Metabolic
-0.8	5.34E-103	9.70E-100	PGS001371	EFO_0004918	age at diagnosis|diabetes mellitus	age at diagnosis	EFO_0004918	Other measurement	Metabolic	PGS002025	MONDO_0005147	type 1 diabetes mellitus	type 1 diabetes mellitus	MONDO_0005147	Immune system disorder	Immunological
-0.8	5.34E-103	9.70E-100	PGS001371	EFO_0004918	age at diagnosis|diabetes mellitus	age at diagnosis	EFO_0004918	Other measurement	Metabolic	PGS002025	MONDO_0005147	type 1 diabetes mellitus	type 1 diabetes mellitus	MONDO_0005147	Metabolic disorder	Metabolic
-0.8	5.34E-103	9.70E-100	PGS001371	EFO_0004918	age at diagnosis|diabetes mellitus	age at diagnosis	EFO_0004918	Other measurement	Metabolic	PGS002025	MONDO_0005147	type 1 diabetes mellitus	type 1 diabetes mellitus	MONDO_0005147	Digestive system disorder	Gastrointestinal
0.8	5.45E-103	9.88E-100	PGS001485	EFO_0006930	brain volume measurement|neuroimaging measurement	brain volume measurement	EFO_0006930	Other measurement	Neurological	PGS001451	EFO_0006930	measurement	brain volume measurement	EFO_0006930	Other measurement	Neurological
0.7999	5.65E-103	1.02E-99	PGS001485	EFO_0006930	brain volume measurement|neuroimaging measurement	brain volume measurement	EFO_0006930	Other measurement	Neurological	PGS001450	EFO_0006930	measurement	brain volume measurement	EFO_0006930	Other measurement	Neurological
0.7999	5.93E-103	1.08E-99	PGS001037	EFO_0009270	heel bone mineral density	heel bone mineral density	EFO_0009270	Other measurement	Skeletal	PGS001956	EFO_0007772	measurement	measurement	EFO_0007772	Other measurement	Skeletal
0.7999	5.94E-103	1.08E-99	PGS000952	EFO_0004514	bone quantitative ultrasound measurement	bone quantitative ultrasound measurement	EFO_0004514	Other measurement	Skeletal	PGS001956	EFO_0007772	measurement	measurement	EFO_0007772	Other measurement	Skeletal
0.7997	7.04E-103	1.27E-99	PGS002965	EFO_0004339	body height	body height	EFO_0004339	Body measurement	Body measurement	PGS002968	EFO_0004339	body height	body height	EFO_0004339	Body measurement	Body measurement
-0.7997	7.10E-103	1.29E-99	PGS003219	EFO_0005208	glomerular filtration rate	glomerular filtration rate	EFO_0005208	Other measurement	Genitourinary	PGS002237	EFO_0003884	chronic kidney disease	chronic kidney disease	EFO_0003884	Other disease	Metabolic
0.7996	8.30E-103	1.50E-99	PGS000515	EFO_0000305	breast carcinoma	breast carcinoma	EFO_0000305	Cancer	Neoplasms	PGS000480	EFO_0000305	breast carcinoma	breast carcinoma	EFO_0000305	Cancer	Neoplasms
0.7995	8.63E-103	1.56E-99	PGS003135	EFO_0004574	total cholesterol measurement	total cholesterol measurement	EFO_0004574	Lipid or lipoprotein measurement	Metabolic	PGS003031	EFO_0004611	low density lipoprotein cholesterol measurement	measurement	EFO_0004611	Lipid or lipoprotein measurement	Metabolic
0.7995	8.63E-103	1.56E-99	PGS003135	EFO_0004574	total cholesterol measurement	total cholesterol measurement	EFO_0004574	Lipid or lipoprotein measurement	Metabolic	PGS003031	EFO_0004611	low density lipoprotein cholesterol measurement	measurement	EFO_0004611	Cardiovascular measurement	Cardiovascular
0.7994	9.37E-103	1.69E-99	PGS000044	EFO_0001663	prostate carcinoma	prostate carcinoma	EFO_0001663	Cancer	Neoplasms	PGS000030	EFO_0001663	prostate carcinoma	prostate carcinoma	EFO_0001663	Cancer	Neoplasms
0.7994	9.85E-103	1.78E-99	PGS002633	EFO_0000319	cardiovascular disease	cardiovascular disease	EFO_0000319	Cardiovascular disease	Cardiovascular	PGS002652	EFO_0000537	hypertension	hypertension	EFO_0000537	Cardiovascular disease	Cardiovascular
0.7994	9.96E-103	1.80E-99	PGS000744	EFO_0000756	melanoma	melanoma	EFO_0000756	Cancer	Neoplasms	PGS000813	EFO_0000756	melanoma	melanoma	EFO_0000756	Cancer	Neoplasms
0.7992	1.18E-102	2.14E-99	PGS000562	EFO_0003893	ovarian neoplasm	ovarian neoplasm	EFO_0003893	Cancer	Neoplasms	PGS000550	EFO_0003893	ovarian neoplasm	ovarian neoplasm	EFO_0003893	Cancer	Neoplasms
0.7991	1.33E-102	2.40E-99	PGS003230	EFO_0010472	creatine measurement	creatine measurement	EFO_0010472	Other measurement	Metabolic	PGS003231	EFO_0010472	creatine measurement	creatine measurement	EFO_0010472	Other measurement	Metabolic
0.7991	1.37E-102	2.47E-99	PGS002228	EFO_0006335	systolic blood pressure	systolic blood pressure	EFO_0006335	Other measurement	Cardiovascular	PGS002349	EFO_0006335	systolic blood pressure	systolic blood pressure	EFO_0006335	Other measurement	Cardiovascular
0.7991	1.37E-102	2.48E-99	PGS002629	EFO_0005140	autoimmune disease	autoimmune disease	EFO_0005140	Immune system disorder	Immunological	PGS002668	EFO_1000627	thyroid disease	thyroid disease	EFO_1000627	Other disease	Endocrine
0.7991	1.38E-102	2.50E-99	PGS002337	EFO_0004611	low density lipoprotein cholesterol measurement	low density lipoprotein cholesterol measurement	EFO_0004611	Lipid or lipoprotein measurement	Metabolic	PGS003038	EFO_0004611	low density lipoprotein cholesterol measurement	measurement	EFO_0004611	Cardiovascular measurement	Cardiovascular
0.7991	1.38E-102	2.50E-99	PGS002337	EFO_0004611	low density lipoprotein cholesterol measurement	low density lipoprotein cholesterol measurement	EFO_0004611	Cardiovascular measurement	Cardiovascular	PGS003038	EFO_0004611	low density lipoprotein cholesterol measurement	measurement	EFO_0004611	Lipid or lipoprotein measurement	Metabolic
0.7991	1.38E-102	2.50E-99	PGS002337	EFO_0004611	low density lipoprotein cholesterol measurement	low density lipoprotein cholesterol measurement	EFO_0004611	Cardiovascular measurement	Cardiovascular	PGS003038	EFO_0004611	low density lipoprotein cholesterol measurement	measurement	EFO_0004611	Cardiovascular measurement	Cardiovascular
0.7991	1.38E-102	2.50E-99	PGS002337	EFO_0004611	low density lipoprotein cholesterol measurement	low density lipoprotein cholesterol measurement	EFO_0004611	Lipid or lipoprotein measurement	Metabolic	PGS003038	EFO_0004611	low density lipoprotein cholesterol measurement	measurement	EFO_0004611	Lipid or lipoprotein measurement	Metabolic
0.799	1.44E-102	2.59E-99	PGS000500	EFO_0000305	breast carcinoma	breast carcinoma	EFO_0000305	Cancer	Neoplasms	PGS000503	EFO_0000305	breast carcinoma	breast carcinoma	EFO_0000305	Cancer	Neoplasms
0.799	1.47E-102	2.64E-99	PGS001106	EFO_0007800	body fat percentage	body fat percentage	EFO_0007800	Body measurement	Body measurement	PGS001104	EFO_0007800	body fat percentage	body fat percentage	EFO_0007800	Body measurement	Body measurement
0.7989	1.61E-102	2.91E-99	PGS001477	EFO_0006930	brain volume measurement|neuroimaging measurement	brain volume measurement	EFO_0006930	Other measurement	Neurological	PGS001681	EFO_0006930	measurement	brain volume measurement	EFO_0006930	Other measurement	Neurological
0.7988	1.81E-102	3.26E-99	PGS001847	EFO_0004264	vascular disease	vascular disease	EFO_0004264	Cardiovascular disease	Cardiovascular	PGS001277	EFO_0003827	pulmonary embolism|deep vein thrombosis	pulmonary embolism	EFO_0003827	Cardiovascular disease	Cardiovascular
0.7988	1.81E-102	3.26E-99	PGS003048	EFO_0006527	smoking status measurement	smoking status measurement	EFO_0006527	Other measurement	Psychiatric	PGS003044	EFO_0006527	smoking status measurement	smoking status measurement	EFO_0006527	Other measurement	Psychiatric
0.7987	1.89E-102	3.41E-99	PGS000561	EFO_0003893	ovarian neoplasm	ovarian neoplasm	EFO_0003893	Cancer	Neoplasms	PGS000550	EFO_0003893	ovarian neoplasm	ovarian neoplasm	EFO_0003893	Cancer	Neoplasms
0.7987	2.08E-102	3.74E-99	PGS003248	EFO_0010472	creatine measurement	creatine measurement	EFO_0010472	Other measurement	Metabolic	PGS003239	EFO_0010472	creatine measurement	creatine measurement	EFO_0010472	Other measurement	Metabolic
0.7986	2.17E-102	3.91E-99	PGS001477	EFO_0006930	brain volume measurement|neuroimaging measurement	brain volume measurement	EFO_0006930	Other measurement	Neurological	PGS001480	EFO_0006930	measurement	brain volume measurement	EFO_0006930	Other measurement	Neurological
0.7985	2.45E-102	4.41E-99	PGS000594	EFO_0007355	male reproductive organ cancer	male reproductive organ cancer	EFO_0007355	Cancer	Neoplasms	PGS000591	EFO_0001663	prostate carcinoma	prostate carcinoma	EFO_0001663	Cancer	Neoplasms
0.7985	2.52E-102	4.54E-99	PGS002466	EFO_0004305	erythrocyte count	erythrocyte count	EFO_0004305	Hematological measurement	Hematological	PGS002613	EFO_0004305	erythrocyte count	erythrocyte count	EFO_0004305	Hematological measurement	Hematological
0.7985	2.53E-102	4.55E-99	PGS002173	EFO_0009270	heel bone mineral density	heel bone mineral density	EFO_0009270	Other measurement	Skeletal	PGS001529	EFO_0007772	measurement	measurement	EFO_0007772	Other measurement	Skeletal
0.7984	2.63E-102	4.73E-99	PGS002550	EFO_0004541	HbA1c measurement	HbA1c measurement	EFO_0004541	Hematological measurement	Hematological	PGS002596	EFO_0004468	glucose measurement	glucose measurement	EFO_0004468	Other measurement	Metabolic
0.7984	2.76E-102	4.95E-99	PGS002965	EFO_0004339	body height	body height	EFO_0004339	Body measurement	Body measurement	PGS002973	EFO_0004339	body height	body height	EFO_0004339	Body measurement	Body measurement
0.7984	2.76E-102	4.95E-99	PGS002135	EFO_0004337	intelligence	intelligence	EFO_0004337	Biological process	Cognitive	PGS001919	EFO_0004337	intelligence	intelligence	EFO_0004337	Biological process	Cognitive
-0.7984	2.78E-102	4.99E-99	PGS001817	MONDO_0005147	type 1 diabetes mellitus	type 1 diabetes mellitus	MONDO_0005147	Metabolic disorder	Metabolic	PGS001371	EFO_0004918	age at diagnosis|diabetes mellitus	age at diagnosis	EFO_0004918	Other measurement	Metabolic
-0.7984	2.78E-102	4.99E-99	PGS001817	MONDO_0005147	type 1 diabetes mellitus	type 1 diabetes mellitus	MONDO_0005147	Immune system disorder	Immunological	PGS001371	EFO_0004918	age at diagnosis|diabetes mellitus	age at diagnosis	EFO_0004918	Other measurement	Metabolic
-0.7984	2.78E-102	4.99E-99	PGS001817	MONDO_0005147	type 1 diabetes mellitus	type 1 diabetes mellitus	MONDO_0005147	Digestive system disorder	Gastrointestinal	PGS001371	EFO_0004918	age at diagnosis|diabetes mellitus	age at diagnosis	EFO_0004918	Other measurement	Metabolic
0.7983	2.94E-102	5.27E-99	PGS000508	EFO_0000305	breast carcinoma	breast carcinoma	EFO_0000305	Cancer	Neoplasms	PGS000536	EFO_0000305	breast carcinoma	breast carcinoma	EFO_0000305	Cancer	Neoplasms
0.7983	3.04E-102	5.45E-99	PGS000006	EFO_1000650	estrogen-receptor negative breast cancer	estrogen-receptor negative breast cancer	EFO_1000650	Cancer	Neoplasms	PGS000216	EFO_0005537	triple-negative breast cancer	triple-negative breast cancer	EFO_0005537	Cancer	Neoplasms
0.7983	3.05E-102	5.47E-99	PGS001065	EFO_0008111	diet measurement	diet measurement	EFO_0008111	Other measurement	Activities	PGS000040	EFO_0001060	celiac disease	celiac disease	EFO_0001060	Digestive system disorder	Gastrointestinal
0.7983	3.05E-102	5.47E-99	PGS001065	EFO_0008111	diet measurement	diet measurement	EFO_0008111	Other measurement	Activities	PGS000040	EFO_0001060	celiac disease	celiac disease	EFO_0001060	Immune system disorder	Immunological
0.7983	3.07E-102	5.51E-99	PGS002501	EFO_0004541	HbA1c measurement	HbA1c measurement	EFO_0004541	Hematological measurement	Hematological	PGS002442	EFO_0000400	diabetes mellitus	diabetes mellitus	EFO_0000400	Digestive system disorder	Gastrointestinal
0.7983	3.07E-102	5.51E-99	PGS002501	EFO_0004541	HbA1c measurement	HbA1c measurement	EFO_0004541	Hematological measurement	Hematological	PGS002442	EFO_0000400	diabetes mellitus	diabetes mellitus	EFO_0000400	Metabolic disorder	Metabolic
0.7982	3.27E-102	5.85E-99	PGS001102	EFO_0007800	body fat percentage	body fat percentage	EFO_0007800	Body measurement	Body measurement	PGS001104	EFO_0007800	body fat percentage	body fat percentage	EFO_0007800	Body measurement	Body measurement
0.7982	3.36E-102	6.01E-99	PGS002672	EFO_0004631	vitamin D measurement	vitamin D measurement	EFO_0004631	Lipid or lipoprotein measurement	Metabolic	PGS002574	EFO_0004631	vitamin D measurement	vitamin D measurement	EFO_0004631	Lipid or lipoprotein measurement	Metabolic
0.7981	3.63E-102	6.49E-99	PGS000675	EFO_0004458	C-reactive protein measurement	C-reactive protein measurement	EFO_0004458	Inflammatory measurement	Immunological	PGS002868	EFO_0004458	C-reactive protein measurement	C-reactive protein measurement	EFO_0004458	Inflammatory measurement	Immunological
0.798	3.85E-102	6.88E-99	PGS000108	EFO_0007984	platelet component distribution width	platelet component distribution width	EFO_0007984	Hematological measurement	Hematological	PGS001972	EFO_0007984	platelet component distribution width	platelet component distribution width	EFO_0007984	Hematological measurement	Hematological
0.798	3.94E-102	7.04E-99	PGS000688	EFO_0004611	low density lipoprotein cholesterol measurement	low density lipoprotein cholesterol measurement	EFO_0004611	Cardiovascular measurement	Cardiovascular	PGS002102	EFO_0004615	apolipoprotein B measurement	apolipoprotein B measurement	EFO_0004615	Lipid or lipoprotein measurement	Metabolic

0.798	3.94E-102	7.04E-99	PGS000688	EFO_0004611	low density lipoprotein cholesterol measurement	low density lipoprotein cholesterol measurement	EFO_0004611	Lipid or lipoprotein measurement	Metabolic	PGS002102	EFO_0004615	apolipoprotein B measurement	apolipoprotein B measurement	EFO_0004615	Lipid or lipoprotein measurement	Metabolic
0.798	3.99E-102	7.13E-99	PGS002161	EFO_0004340	body mass index	body mass index	EFO_0004340	Body measurement	Body measurement	PGS002855	EFO_0004340	body mass index	body mass index	EFO_0004340	Body measurement	Body measurement
0.798	4.02E-102	7.18E-99	PGS002161	EFO_0004340	body mass index	body mass index	EFO_0004340	Body measurement	Body measurement	PGS002176	EFO_0005093	hip circumference	hip circumference	EFO_0005093	Body measurement	Body measurement
0.7978	4.68E-102	8.35E-99	PGS001220	EFO_0004526	mean corpuscular volume	mean corpuscular volume	EFO_0004526	Hematological measurement	Hematological	PGS002207	EFO_0004526	mean corpuscular volume	mean corpuscular volume	EFO_0004526	Hematological measurement	Hematological
0.7978	4.79E-102	8.55E-99	PGS000378	EFO_1001950	colon carcinoma	colon carcinoma	EFO_1001950	Digestive system disorder	Gastrointestinal	PGS000379	EFO_1001950	colon carcinoma	colon carcinoma	EFO_1001950	Cancer	Neoplasms
0.7978	4.79E-102	8.55E-99	PGS000378	EFO_1001950	colon carcinoma	colon carcinoma	EFO_1001950	Cancer	Neoplasms	PGS000379	EFO_1001950	colon carcinoma	colon carcinoma	EFO_1001950	Cancer	Neoplasms
0.7978	4.79E-102	8.55E-99	PGS000378	EFO_1001950	colon carcinoma	colon carcinoma	EFO_1001950	Cancer	Neoplasms	PGS000379	EFO_1001950	colon carcinoma	colon carcinoma	EFO_1001950	Digestive system disorder	Gastrointestinal
0.7978	4.79E-102	8.55E-99	PGS000378	EFO_1001950	colon carcinoma	colon carcinoma	EFO_1001950	Digestive system disorder	Gastrointestinal	PGS000379	EFO_1001950	colon carcinoma	colon carcinoma	EFO_1001950	Digestive system disorder	Gastrointestinal
0.7976	5.84E-102	1.04E-98	PGS000502	EFO_0000305	breast carcinoma	breast carcinoma	EFO_0000305	Cancer	Neoplasms	PGS000505	EFO_0000305	breast carcinoma	breast carcinoma	EFO_0000305	Cancer	Neoplasms
0.7976	5.84E-102	1.04E-98	PGS000502	EFO_0000305	breast carcinoma	breast carcinoma	EFO_0000305	Cancer	Neoplasms	PGS000506	EFO_0000305	breast carcinoma	breast carcinoma	EFO_0000305	Cancer	Neoplasms
0.7976	5.86E-102	1.04E-98	PGS000504	EFO_0000305	breast carcinoma	breast carcinoma	EFO_0000305	Cancer	Neoplasms	PGS000531	EFO_0000305	breast carcinoma	breast carcinoma	EFO_0000305	Cancer	Neoplasms
0.7976	6.05E-102	1.08E-98	PGS001945	EFO_0004518	creatinine measurement	creatinine measurement	EFO_0004518	Other measurement	Metabolic	PGS003248	EFO_0010472	creatine measurement	creatine measurement	EFO_0010472	Other measurement	Metabolic
0.7976	6.18E-102	1.10E-98	PGS000587	EFO_0001663	prostate carcinoma	prostate carcinoma	EFO_0001663	Cancer	Neoplasms	PGS002240	EFO_0001663	prostate carcinoma	prostate carcinoma	EFO_0001663	Cancer	Neoplasms
0.7976	6.22E-102	1.11E-98	PGS002954	EFO_0004612	high density lipoprotein cholesterol measurement	high density lipoprotein cholesterol measurement	EFO_0004612	Lipid or lipoprotein measurement	Metabolic	PGS002963	EFO_0004612	high density lipoprotein cholesterol measurement	measurement	EFO_0004612	Lipid or lipoprotein measurement	Metabolic
0.7975	6.80E-102	1.21E-98	PGS001528	EFO_0007986	reticulocyte count	reticulocyte count	EFO_0007986	Hematological measurement	Hematological	PGS001406	EFO_0010700	reticulocyte measurement	reticulocyte measurement	EFO_0010700	Hematological measurement	Hematological
0.7972	8.97E-102	1.60E-98	PGS002589	EFO_0000400	diabetes mellitus	diabetes mellitus	EFO_0000400	Metabolic disorder	Metabolic	PGS002449	EFO_0004468	glucose measurement	glucose measurement	EFO_0004468	Other measurement	Metabolic
0.7972	8.97E-102	1.60E-98	PGS002589	EFO_0000400	diabetes mellitus	diabetes mellitus	EFO_0000400	Digestive system disorder	Gastrointestinal	PGS002449	EFO_0004468	glucose measurement	glucose measurement	EFO_0004468	Other measurement	Metabolic
0.7971	9.97E-102	1.77E-98	PGS000521	EFO_0000305	breast carcinoma	breast carcinoma	EFO_0000305	Cancer	Neoplasms	PGS000523	EFO_0000305	breast carcinoma	breast carcinoma	EFO_0000305	Cancer	Neoplasms
0.797	1.05E-101	1.86E-98	PGS002146	EFO_0004339	body height	body height	EFO_0004339	Body measurement	Body measurement	PGS002969	EFO_0004339	body height	body height	EFO_0004339	Body measurement	Body measurement
0.7969	1.18E-101	2.09E-98	PGS002605	EFO_0004611	low density lipoprotein cholesterol measurement	low density lipoprotein cholesterol measurement	EFO_0004611	Cardiovascular measurement	Cardiovascular	PGS002602	HP_0003124	Hypercholesterolemia	Hypercholesterolemia	HP_0003124	Other trait	Metabolic
0.7969	1.18E-101	2.09E-98	PGS002605	EFO_0004611	low density lipoprotein cholesterol measurement	low density lipoprotein cholesterol measurement	EFO_0004611	Lipid or lipoprotein measurement	Metabolic	PGS002602	HP_0003124	Hypercholesterolemia	Hypercholesterolemia	HP_0003124	Other trait	Metabolic
-0.7968	1.32E-101	2.35E-98	PGS002155	EFO_0004279	suntan	suntan	EFO_0004279	Other trait	Dermatological	PGS002350	EFO_0004279	suntan	suntan	EFO_0004279	Other trait	Dermatological
-0.7968	1.33E-101	2.35E-98	PGS001326	EFO_0000464	emphysema	emphysema	EFO_0000464	Other disease	Respiratory	PGS001139	EFO_0005671	smoking behaviour measurement	smoking behaviour measurement	EFO_0005671	Other measurement	Psychiatric
0.7967	1.46E-101	2.58E-98	PGS000814	EFO_0004611	low density lipoprotein cholesterol measurement	low density lipoprotein cholesterol measurement	EFO_0004611	Lipid or lipoprotein measurement	Metabolic	PGS000340	EFO_0004611	low density lipoprotein cholesterol measurement	measurement	EFO_0004611	Cardiovascular measurement	Cardiovascular
0.7967	1.46E-101	2.58E-98	PGS000814	EFO_0004611	low density lipoprotein cholesterol measurement	low density lipoprotein cholesterol measurement	EFO_0004611	Lipid or lipoprotein measurement	Metabolic	PGS000340	EFO_0004611	low density lipoprotein cholesterol measurement	measurement	EFO_0004611	Lipid or lipoprotein measurement	Metabolic
0.7967	1.46E-101	2.58E-98	PGS000814	EFO_0004611	low density lipoprotein cholesterol measurement	low density lipoprotein cholesterol measurement	EFO_0004611	Cardiovascular measurement	Cardiovascular	PGS003403	EFO_0004611	low density lipoprotein cholesterol measurement	measurement	EFO_0004611	Cardiovascular measurement	Cardiovascular
0.7967	1.46E-101	2.58E-98	PGS000814	EFO_0004611	low density lipoprotein cholesterol measurement	low density lipoprotein cholesterol measurement	EFO_0004611	Lipid or lipoprotein measurement	Metabolic	PGS003403	EFO_0004611	low density lipoprotein cholesterol measurement	measurement	EFO_0004611	Cardiovascular measurement	Cardiovascular
0.7967	1.46E-101	2.58E-98	PGS000814	EFO_0004611	low density lipoprotein cholesterol measurement	low density lipoprotein cholesterol measurement	EFO_0004611	Cardiovascular measurement	Cardiovascular	PGS003403	EFO_0004611	low density lipoprotein cholesterol measurement	measurement	EFO_0004611	Lipid or lipoprotein measurement	Metabolic
0.7967	1.46E-101	2.58E-98	PGS000814	EFO_0004611	low density lipoprotein cholesterol measurement	low density lipoprotein cholesterol measurement	EFO_0004611	Cardiovascular measurement	Cardiovascular	PGS000340	EFO_0004611	low density lipoprotein cholesterol measurement	measurement	EFO_0004611	Cardiovascular measurement	Cardiovascular
0.7967	1.46E-101	2.58E-98	PGS000814	EFO_0004611	low density lipoprotein cholesterol measurement	low density lipoprotein cholesterol measurement	EFO_0004611	Cardiovascular measurement	Cardiovascular	PGS000340	EFO_0004611	low density lipoprotein cholesterol measurement	measurement	EFO_0004611	Lipid or lipoprotein measurement	Metabolic
0.7967	1.46E-101	2.58E-98	PGS000814	EFO_0004611	low density lipoprotein cholesterol measurement	low density lipoprotein cholesterol measurement	EFO_0004611	Lipid or lipoprotein measurement	Metabolic	PGS003403	EFO_0004611	low density lipoprotein cholesterol measurement	measurement	EFO_0004611	Lipid or lipoprotein measurement	Metabolic
0.7967	1.49E-101	2.65E-98	PGS002517	EFO_0000684	respiratory system disease	respiratory system disease	EFO_0000684	Other disease	Skeletal	PGS002481	MONDO_0004979	asthma	asthma	MONDO_0004979	Other disease	Respiratory
0.7967	1.51E-101	2.68E-98	PGS001943	EFO_0004340	body mass index	body mass index	EFO_0004340	Body measurement	Body measurement	PGS002169	EFO_0005409	fat body mass	fat body mass	EFO_0005409	Other measurement	Body measurement
0.7964	1.90E-101	3.37E-98	PGS001674	EFO_0006930	brain volume measurement|neuroimaging measurement	brain volume measurement	EFO_0006930	Other measurement	Neurological	PGS001479	EFO_0006930	measurement	brain volume measurement	EFO_0006930	Other measurement	Neurological
0.7964	1.95E-101	3.45E-98	PGS003118	MONDO_0005148	type 2 diabetes mellitus	type 2 diabetes mellitus	MONDO_0005148	Digestive system disorder	Gastrointestinal	PGS003117	MONDO_0005148	type 2 diabetes mellitus	type 2 diabetes mellitus	MONDO_0005148	Digestive system disorder	Gastrointestinal
0.7964	1.95E-101	3.45E-98	PGS003118	MONDO_0005148	type 2 diabetes mellitus	type 2 diabetes mellitus	MONDO_0005148	Metabolic disorder	Metabolic	PGS003117	MONDO_0005148	type 2 diabetes mellitus	type 2 diabetes mellitus	MONDO_0005148	Metabolic disorder	Metabolic
0.7964	1.95E-101	3.45E-98	PGS003118	MONDO_0005148	type 2 diabetes mellitus	type 2 diabetes mellitus	MONDO_0005148	Metabolic disorder	Metabolic	PGS003117	MONDO_0005148	type 2 diabetes mellitus	type 2 diabetes mellitus	MONDO_0005148	Digestive system disorder	Gastrointestinal
0.7964	1.95E-101	3.45E-98	PGS003118	MONDO_0005148	type 2 diabetes mellitus	type 2 diabetes mellitus	MONDO_0005148	Digestive system disorder	Gastrointestinal	PGS003117	MONDO_0005148	type 2 diabetes mellitus	type 2 diabetes mellitus	MONDO_0005148	Metabolic disorder	Metabolic
0.7964	2.01E-101	3.56E-98	PGS001930	EFO_0009253	Immature Reticulocyte Fraction Measurement	Immature Reticulocyte Fraction Measurement	EFO_0009253	Hematological measurement	Hematological	PGS001408	EFO_0009253	Immature Reticulocyte Fraction Measurement	Measurement	EFO_0009253	Hematological measurement	Hematological
0.7963	2.14E-101	3.80E-98	PGS003118	MONDO_0005148	type 2 diabetes mellitus	type 2 diabetes mellitus	MONDO_0005148	Digestive system disorder	Gastrointestinal	PGS003102	MONDO_0005148	type 2 diabetes mellitus	type 2 diabetes mellitus	MONDO_0005148	Digestive system disorder	Gastrointestinal
0.7963	2.14E-101	3.80E-98	PGS003118	MONDO_0005148	type 2 diabetes mellitus	type 2 diabetes mellitus	MONDO_0005148	Metabolic disorder	Metabolic	PGS003102	MONDO_0005148	type 2 diabetes mellitus	type 2 diabetes mellitus	MONDO_0005148	Metabolic disorder	Metabolic
0.7963	2.14E-101	3.80E-98	PGS003118	MONDO_0005148	type 2 diabetes mellitus	type 2 diabetes mellitus	MONDO_0005148	Digestive system disorder	Gastrointestinal	PGS003102	MONDO_0005148	type 2 diabetes mellitus	type 2 diabetes mellitus	MONDO_0005148	Metabolic disorder	Metabolic
0.7963	2.14E-101	3.80E-98	PGS003118	MONDO_0005148	type 2 diabetes mellitus	type 2 diabetes mellitus	MONDO_0005148	Metabolic disorder	Metabolic	PGS003102	MONDO_0005148	type 2 diabetes mellitus	type 2 diabetes mellitus	MONDO_0005148	Digestive system disorder	Gastrointestinal
0.7963	2.19E-101	3.87E-98	PGS001971	EFO_0004584	mean platelet volume	mean platelet volume	EFO_0004584	Hematological measurement	Hematological	PGS000104	EFO_0004584	mean platelet volume	mean platelet volume	EFO_0004584	Hematological measurement	Hematological
0.7963	2.19E-101	3.88E-98	PGS001958	EFO_0005093	hip circumference	hip circumference	EFO_0005093	Body measurement	Body measurement	PGS001983	EFO_0004342	waist circumference	waist circumference	EFO_0004342	Body measurement	Body measurement
0.7962	2.39E-101	4.23E-98	PGS000376	EFO_1001950	colon carcinoma	colon carcinoma	EFO_1001950	Cancer	Neoplasms	PGS000377	EFO_1001950	colon carcinoma	colon carcinoma	EFO_1001950	Cancer	Neoplasms
0.7962	2.39E-101	4.23E-98	PGS000376	EFO_1001950	colon carcinoma	colon carcinoma	EFO_1001950	Cancer	Neoplasms	PGS000377	EFO_1001950	colon carcinoma	colon carcinoma	EFO_1001950	Digestive system disorder	Gastrointestinal
0.7962	2.39E-101	4.23E-98	PGS000376	EFO_1001950	colon carcinoma	colon carcinoma	EFO_1001950	Digestive system disorder	Gastrointestinal	PGS000377	EFO_1001950	colon carcinoma	colon carcinoma	EFO_1001950	Cancer	Neoplasms
0.7962	2.39E-101	4.23E-98	PGS000376	EFO_1001950	colon carcinoma	colon carcinoma	EFO_1001950	Digestive system disorder	Gastrointestinal	PGS000377	EFO_1001950	colon carcinoma	colon carcinoma	EFO_1001950	Digestive system disorder	Gastrointestinal
0.7962	2.46E-101	4.35E-98	PGS001230	EFO_0004338	body weight	body weight	EFO_0004338	Body measurement	Body measurement	PGS001235	EFO_0004324	body weights and measures	body weights and measures	EFO_0004324	Body measurement	Body measurement
0.796	2.86E-101	5.06E-98	PGS000214	MONDO_0021115	luminal B breast carcinoma	luminal B breast carcinoma	MONDO_0021115	Cancer	Neoplasms	PGS000004	EFO_0000305	breast carcinoma	breast carcinoma	EFO_0000305	Cancer	Neoplasms
0.796	3.03E-101	5.35E-98	PGS000706	EFO_0000537	hypertension	hypertension	EFO_0000537	Cardiovascular disease	Cardiovascular	PGS001335	EFO_0000319	cardiovascular disease	cardiovascular disease	EFO_0000319	Cardiovascular disease	Cardiovascular
0.7958	3.46E-101	6.11E-98	PGS001681	EFO_0006930	brain volume measurement|neuroimaging measurement	brain volume measurement	EFO_0006930	Other measurement	Neurological	PGS001673	EFO_0006930	measurement	brain volume measurement	EFO_0006930	Other measurement	Neurological
0.7958	3.59E-101	6.34E-98	PGS001254	EFO_0004229	Dupuytren Contracture	Dupuytren Contracture	EFO_0004229	Other disease	Others	PGS001031	EFO_0009432	fibroblastic disorder	fibroblastic disorder	EFO_0009432	Other disease	Connective Tissue
0.7957	3.89E-101	6.87E-98	PGS000005	EFO_1000649	estrogen-receptor positive breast cancer	estrogen-receptor positive breast cancer	EFO_1000649	Cancer	Neoplasms	PGS000007	EFO_0000305	breast carcinoma	breast carcinoma	EFO_0000305	Cancer	Neoplasms
0.7957	4.11E-101	7.26E-98	PGS001943	EFO_0004340	body mass index	body mass index	EFO_0004340	Body measurement	Body measurement	PGS000921	EFO_0004340	body mass index	body mass index	EFO_0004340	Body measurement	Body measurement
0.7956	4.28E-101	7.55E-98	PGS000438	MONDO_0002898	skin cancer	skin cancer	MONDO_0002898	Cancer	Neoplasms	PGS000405	EFO_0009259	skin carcinoma	skin carcinoma	EFO_0009259	Cancer	Neoplasms
0.7956	4.47E-101	7.88E-98	PGS000579	EFO_0001663	prostate carcinoma	prostate carcinoma	EFO_0001663	Cancer	Neoplasms	PGS000742	EFO_0001663	prostate carcinoma	prostate carcinoma	EFO_0001663	Cancer	Neoplasms
0.7956	4.49E-101	7.92E-98	PGS000563	EFO_0003893	ovarian neoplasm	ovarian neoplasm	EFO_0003893	Cancer	Neoplasms	PGS000549	EFO_0003893	ovarian neoplasm	ovarian neoplasm	EFO_0003893	Cancer	Neoplasms
0.7956	4.54E-101	8.00E-98	PGS002835	EFO_0007878	alcohol consumption measurement	alcohol consumption measurement	EFO_0007878	Other measurement	Psychiatric	PGS002834	EFO_0007878	alcohol consumption measurement	alcohol consumption measurement	EFO_0007878	Other measurement	Psychiatric
0.7955	4.59E-101	8.09E-98	PGS001359	EFO_0004731	eye measurement	eye measurement	EFO_0004731	Other measurement	Ophthalmological	PGS001368	EFO_0004731	eye measurement	eye measurement	EFO_0004731	Other measurement	Ophthalmological
0.7955	4.74E-101	8.35E-98	PGS002752	EFO_0007878	alcohol consumption measurement	alcohol consumption measurement	EFO_0007878	Other measurement	Psychiatric	PGS002835	EFO_0007878	alcohol consumption measurement	alcohol consumption measurement	EFO_0007878	Other measurement	Psychiatric
0.7955	4.88E-101	8.58E-98	PGS000047	EFO_1000650	estrogen-receptor negative breast cancer	estrogen-receptor negative breast cancer	EFO_1000650	Cancer	Neoplasms	PGS000003	EFO_1000650	estrogen-receptor negative breast cancer	cancer	EFO_1000650	Cancer	Neoplasms
0.7954	5.49E-101	9.66E-98	PGS003030	EFO_0004611	low density lipoprotein cholesterol measurement	low density lipoprotein cholesterol measurement	EFO_0004611	Cardiovascular measurement	Cardiovascular	PGS003141	EFO_0004574	total cholesterol measurement	total cholesterol measurement	EFO_0004574	Lipid or lipoprotein measurement	Metabolic
0.7954	5.49E-101	9.66E-98	PGS003030	EFO_0004611	low density lipoprotein cholesterol measurement	low density lipoprotein cholesterol measurement	EFO_0004611	Lipid or lipoprotein measurement	Metabolic	PGS003141	EFO_0004574	total cholesterol measurement	total cholesterol measurement	EFO_0004574	Lipid or lipoprotein measurement	Metabolic
0.7953	5.95E-101	1.05E-97	PGS002556	EFO_0004611	low density lipoprotein cholesterol measurement	low density lipoprotein cholesterol measurement	EFO_0004611	Cardiovascular measurement	Cardiovascular	PGS000889	EFO_0004611	low density lipoprotein cholesterol measurement	measurement	EFO_0004611	Cardiovascular measurement	Cardiovascular
0.7953	5.95E-101	1.05E-97	PGS002556	EFO_0004611	low density lipoprotein cholesterol measurement	low density lipoprotein cholesterol measurement	EFO_0004611	Cardiovascular measurement	Cardiovascular	PGS000889	EFO_0004611	low density lipoprotein cholesterol measurement	measurement	EFO_0004611	Lipid or lipoprotein measurement	Metabolic
0.7953	5.95E-101	1.05E-97	PGS002556	EFO_0004611	low density lipoprotein cholesterol measurement	low density lipoprotein cholesterol measurement	EFO_0004611	Lipid or lipoprotein measurement	Metabolic	PGS000889	EFO_0004611	low density lipoprotein cholesterol measurement	measurement	EFO_0004611	Cardiovascular measurement	Cardiovascular
0.7953	5.95E-101	1.05E-97	PGS002556	EFO_0004611	low density lipoprotein cholesterol measurement	low density lipoprotein cholesterol measurement	EFO_0004611	Lipid or lipoprotein measurement	Metabolic	PGS000889	EFO_0004611	low density lipoprotein cholesterol measurement	measurement	EFO_0004611	Lipid or lipoprotein measurement	Metabolic
0.7953	6.12E-101	1.08E-97	PGS002356	EFO_0004343	waist-hip ratio	waist-hip ratio	EFO_0004343	Body measurement	Body measurement	PGS002673	EFO_0004343	waist-hip ratio	waist-hip ratio	EFO_0004343	Body measurement	Body measurement
0.7952	6.19E-101	1.09E-97	PGS001336	EFO_0000305	breast carcinoma	breast carcinoma	EFO_0000305	Cancer	Neoplasms	PGS001337	EFO_0005606	family history of breast cancer	family history of breast cancer	EFO_0005606	Other trait	Neoplasms
0.7952	6.25E-101	1.10E-97	PGS002445	EFO_0001379	endocrine system disease|diabetes mellitus	endocrine system disease	EFO_0001379	Other disease	Metabolic	PGS002501	EFO_0004541	HbA1c measurement	HbA1c measurement	EFO_0004541	Hematological measurement	Hematological
-0.7952	6.28E-101	1.10E-97	PGS000023	MONDO_0005147	type 1 diabetes mellitus	type 1 diabetes mellitus	MONDO_0005147	Metabolic disorder	Metabolic	PGS001371	EFO_0004918	age at diagnosis|diabetes mellitus	age at diagnosis	EFO_0004918	Other measurement	Metabolic
-0.7952	6.28E-101	1.10E-97	PGS000023	MONDO_0005147	type 1 diabetes mellitus	type 1 diabetes mellitus	MONDO_0005147	Immune system disorder	Immunological	PGS001371	EFO_0004918	age at diagnosis|diabetes mellitus	age at diagnosis	EFO_0004918	Other measurement	Metabolic
-0.7952	6.28E-101	1.10E-97	PGS000023	MONDO_0005147	type 1 diabetes mellitus	type 1 diabetes mellitus	MONDO_0005147	Digestive system disorder	Gastrointestinal	PGS001371	EFO_0004918	age at diagnosis|diabetes mellitus	age at diagnosis	EFO_0004918	Other measurement	Metabolic
0.7952	6.29E-101	1.10E-97	PGS001929	EFO_0004339	body height	body height	EFO_0004339	Body measurement	Body measurement	PGS002969	EFO_0004339	body height	body height	EFO_0004339	Body measurement	Body measurement
0.7952	6.59E-101	1.16E-97	PGS001674	EFO_0006930	brain volume measurement|neuroimaging measurement	brain volume measurement	EFO_0006930	Other measurement	Neurological	PGS001682	EFO_0006930	measurement	brain volume measurement	EFO_0006930	Other measurement	Neurological
0.7952	6.69E-101	1.17E-97	PGS002998	EFO_0000537	hypertension	hypertension	EFO_0000537	Cardiovascular disease	Cardiovascular	PGS002994	EFO_0000537	hypertension	hypertension	EFO_0000537	Cardiovascular disease	Cardiovascular
0.7952	6.78E-101	1.19E-97	PGS002964	EFO_0004339	body height	body height	EFO_0004339	Body measurement	Body measurement	PGS002146	EFO_0004339	body height	body height	EFO_0004339	Body measurement	Body measurement
0.795	7.81E-101	1.37E-97	PGS001230	EFO_0004338	body weight	body weight	EFO_0004338	Body measurement	Body measurement	PGS001146	EFO_0005409	fat body mass	fat body mass	EFO_0005409	Other measurement	Body measurement
0.795	7.94E-101	1.39E-97	PGS001699	EFO_0006930	brain volume measurement|neuroimaging measurement	brain volume measurement	EFO_0006930	Other measurement	Neurological	PGS001698	EFO_0006930	measurement	brain volume measurement	EFO_0006930	Other measurement	Neurological
0.7948	9.17E-101	1.61E-97	PGS001991	EFO_0007989	monocyte percentage of leukocytes	monocyte percentage of leukocytes	EFO_0007989	Hematological measurement	Hematological	PGS001078	EFO_0007989	monocyte percentage of leukocytes	monocyte percentage of leukocytes	EFO_0007989	Hematological measurement	Hematological
0.7948	9.69E-101	1.70E-97	PGS002408	EFO_0004705	hypothyroidism	hypothyroidism	EFO_0004705	Other disease	Endocrine	PGS002457	EFO_0004705	hypothyroidism	hypothyroidism	EFO_0004705	Other disease	Endocrine
0.7948	9.74E-101	1.71E-97	PGS001952	EFO_0004468	glucose measurement	glucose measurement	EFO_0004468	Other measurement	Metabolic	PGS002938	EFO_0004468	glucose measurement	glucose measurement	EFO_0004468	Other measurement	Metabolic
0.7945	1.27E-100	2.21E-97	PGS000589	EFO_0001663	prostate carcinoma	prostate carcinoma	EFO_0001663	Cancer	Neoplasms	PGS000593	EFO_0007355	male reproductive organ cancer	male reproductive organ cancer	EFO_0007355	Cancer	Neoplasms
0.7944	1.36E-100	2.38E-97	PGS002663	EFO_0005192	red blood cell distribution width	red blood cell distribution width	EFO_0005192	Hematological measurement	Hematological	PGS002346	EFO_0005192	red blood cell distribution width	red blood cell distribution width	EFO_0005192	Hematological measurement	Hematological
0.7944	1.43E-100	2.49E-97	PGS001997	EFO_0007990	neutrophil percentage of leukocytes	neutrophil percentage of leukocytes	EFO_0007990	Hematological measurement	Hematological	PGS001077	EFO_0007990	neutrophil percentage of leukocytes	neutrophil percentage of leukocytes	EFO_0007990	Hematological measurement	Hematological
0.7943	1.60E-100	2.79E-97	PGS000015	EFO_0000305	breast carcinoma	breast carcinoma	EFO_0000305	Cancer	Neoplasms	PGS000509	EFO_0000305	breast carcinoma	breast carcinoma	EFO_0000305	Cancer	Neoplasms
-0.7942	1.65E-100	2.88E-97	PGS000883	EFO_0005208	glomerular filtration rate	glomerular filtration rate	EFO_0005208	Other measurement	Genitourinary	PGS002757	EFO_0003884	chronic kidney disease	chronic kidney disease	EFO_0003884	Other disease	Metabolic
0.7942	1.67E-100	2.91E-97	PGS000051	EFO_0000305	breast carcinoma	breast carcinoma	EFO_0000305	Cancer	Neoplasms	PGS000046	EFO_1000649	estrogen-receptor positive breast cancer	cancer	EFO_1000649	Cancer	Neoplasms
0.794	2.01E-100	3.51E-97	PGS000600	EFO_0005088	testicular carcinoma	testicular carcinoma	EFO_0005088	Cancer	Neoplasms	PGS000597	EFO_0005088	testicular carcinoma	testicular carcinoma	EFO_0005088	Cancer	Neoplasms
0.7939	2.34E-100	4.08E-97	PGS002330	EFO_0007822	hair colour measurement	hair colour measurement	EFO_0007822	Other measurement	Dermatological	PGS001093	EFO_0007822	hair colour measurement	hair colour measurement	EFO_0007822	Other measurement	Dermatological
-0.7938	2.50E-100	4.36E-97	PGS002768	EFO_0003882	osteoporosis	osteoporosis	EFO_0003882	Other disease	Skeletal	PGS002174	EFO_0007772	measurement	measurement	EFO_0007772	Other measurement	Skeletal
0.7938	2.55E-100	4.44E-97	PGS002339	EFO_0004527	mean corpuscular hemoglobin	mean corpuscular hemoglobin	EFO_0004527	Hematological measurement	Hematological	PGS001219	EFO_0004527	mean corpuscular hemoglobin	mean corpuscular hemoglobin	EFO_0004527	Hematological measurement	Hematological
0.7938	2.57E-100	4.47E-97	PGS001326	EFO_0000464	emphysema	emphysema	EFO_0000464	Other disease	Respiratory	PGS001515	EFO_0006525	cigarettes per day measurement	cigarettes per day measurement	EFO_0006525	Other measurement	Psychiatric
0.7938	2.64E-100	4.60E-97	PGS001672	EFO_0006930	brain volume measurement|neuroimaging measurement	brain volume measurement	EFO_0006930	Other measurement	Neurological	PGS001481	EFO_0006930	measurement	brain volume measurement	EFO_0006930	Other measurement	Neurological
0.7938	2.65E-100	4.61E-97	PGS001961	EFO_0005106	body composition measurement	body composition measurement	EFO_0005106	Body measurement	Body measurement	PGS001161	EFO_0005106	body composition measurement	body composition measurement	EFO_0005106	Body measurement	Body measurement
0.7937	2.71E-100	4.72E-97	PGS001806	EFO_0004281	testicular neoplasm	testicular neoplasm	EFO_0004281	Other disease	Neoplasms	PGS001164	EFO_0005088	testicular carcinoma	testicular carcinoma	EFO_0005088	Cancer	Neoplasms
0.7937	2.86E-100	4.97E-97	PGS001318	EFO_0000589	metabolic disease	metabolic disease	EFO_0000589	Metabolic disorder	Metabolic	PGS000936	HP_0003124	Hypercholesterolemia	Hypercholesterolemia	HP_0003124	Other trait	Metabolic
0.7936	2.99E-100	5.21E-97	PGS003189	EFO_0004631	vitamin D measurement	vitamin D measurement	EFO_0004631	Lipid or lipoprotein measurement	Metabolic	PGS002574	EFO_0004631	vitamin D measurement	vitamin D measurement	EFO_0004631	Lipid or lipoprotein measurement	Metabolic
0.7936	3.16E-100	5.50E-97	PGS003323	EFO_0004698	insomnia	insomnia	EFO_0004698	Neurological disorder	Neurological	PGS002196	EFO_0005271	sleep duration	sleep duration	EFO_0005271	Other measurement	Psychiatric
0.7936	3.22E-100	5.60E-97	PGS003136	EFO_0004574	total cholesterol measurement	total cholesterol measurement	EFO_0004574	Lipid or lipoprotein measurement	Metabolic	PGS003030	EFO_0004611	low density lipoprotein cholesterol measurement	measurement	EFO_0004611	Cardiovascular measurement	Cardiovascular
0.7936	3.22E-100	5.60E-97	PGS003136	EFO_0004574	total cholesterol measurement	total cholesterol measurement	EFO_0004574	Lipid or lipoprotein measurement	Metabolic	PGS003030	EFO_0004611	low density lipoprotein cholesterol measurement	measurement	EFO_0004611	Lipid or lipoprotein measurement	Metabolic

0.7935	3.26E-100	5.67E-97	PGS000930	MONDO_0000831	thrombotic disease	thrombotic disease	MONDO_0000831	Cardiovascular disease	Cardiovascular	PGS001847	EFO_0004264	vascular disease	vascular disease	EFO_0004264	Cardiovascular disease	Cardiovascular
0.7935	3.28E-100	5.69E-97	PGS001958	EFO_0005093	hip circumference	hip circumference	EFO_0005093	Body measurement	Body measurement	PGS002169	EFO_0005409	fat body mass	fat body mass	EFO_0005409	Other measurement	Body measurement
0.7934	3.63E-100	6.31E-97	PGS001474	EFO_0006930	brain volume measurement|neuroimaging measurement	brain volume measurement	EFO_0006930	Other measurement	Neurological	PGS001475	EFO_0006930	measurement	brain volume measurement	EFO_0006930	Other measurement	Neurological
-0.7932	4.52E-100	7.85E-97	PGS001094	EFO_0007822	hair colour measurement	hair colour measurement	EFO_0007822	Other measurement	Dermatological	PGS001093	EFO_0007822	hair colour measurement	hair colour measurement	EFO_0007822	Other measurement	Dermatological
0.793	5.43E-100	9.43E-97	PGS002621	EFO_0004530	triglyceride measurement	triglyceride measurement	EFO_0004530	Lipid or lipoprotein measurement	Metabolic	PGS000826	EFO_0004530	triglyceride measurement	triglyceride measurement	EFO_0004530	Lipid or lipoprotein measurement	Metabolic
0.793	5.80E-100	1.01E-96	PGS003316	EFO_0004617	cystatin C measurement|glomerular filtration rate	cystatin C measurement	EFO_0004617	Other measurement	Metabolic	PGS003314	EFO_0004617	cystatin C measurement|glomerular filtration rate	cystatin C measurement	EFO_0004617	Other measurement	Metabolic
0.793	5.80E-100	1.01E-96	PGS003315	EFO_0004617	cystatin C measurement|glomerular filtration rate	cystatin C measurement	EFO_0004617	Other measurement	Metabolic	PGS003314	EFO_0004617	cystatin C measurement|glomerular filtration rate	cystatin C measurement	EFO_0004617	Other measurement	Metabolic
0.7929	6.16E-100	1.07E-96	PGS002841	EFO_0004340	body mass index	body mass index	EFO_0004340	Body measurement	Body measurement	PGS002847	EFO_0004340	body mass index	body mass index	EFO_0004340	Body measurement	Body measurement
0.7928	6.66E-100	1.15E-96	PGS001674	EFO_0006930	brain volume measurement|neuroimaging measurement	brain volume measurement	EFO_0006930	Other measurement	Neurological	PGS001489	EFO_0006930	measurement	brain volume measurement	EFO_0006930	Other measurement	Neurological
0.7927	7.52E-100	1.30E-96	PGS001738	EFO_0006930	brain volume measurement|neuroimaging measurement	brain volume measurement	EFO_0006930	Other measurement	Neurological	PGS001736	EFO_0006930	measurement	brain volume measurement	EFO_0006930	Other measurement	Neurological
0.7927	7.54E-100	1.31E-96	PGS002958	EFO_0004612	high density lipoprotein cholesterol measurement	high density lipoprotein cholesterol measurement	EFO_0004612	Lipid or lipoprotein measurement	Metabolic	PGS002329	EFO_0004612	high density lipoprotein cholesterol measurement	measurement	EFO_0004612	Lipid or lipoprotein measurement	Metabolic
0.7927	7.64E-100	1.32E-96	PGS002830	EFO_0007878	alcohol consumption measurement	alcohol consumption measurement	EFO_0007878	Other measurement	Psychiatric	PGS000204	EFO_0007878	alcohol consumption measurement	alcohol consumption measurement	EFO_0007878	Other measurement	Psychiatric
0.7927	7.66E-100	1.33E-96	PGS003243	EFO_0010472	creatine measurement	creatine measurement	EFO_0010472	Other measurement	Metabolic	PGS003377	EFO_0005208	glomerular filtration rate	glomerular filtration rate	EFO_0005208	Other measurement	Genitourinary
0.7926	7.88E-100	1.36E-96	PGS003331	EFO_0001663	prostate carcinoma	prostate carcinoma	EFO_0001663	Cancer	Neoplasms	PGS000742	EFO_0001663	prostate carcinoma	prostate carcinoma	EFO_0001663	Cancer	Neoplasms
0.7926	8.26E-100	1.43E-96	PGS002174	EFO_0007772	calcaneal bone quantitative ultrasound measurement	calcaneal bone quantitative ultrasound measurement	EFO_0007772	Other measurement	Skeletal	PGS000951	EFO_0004514	bone quantitative ultrasound measurement	measurement	EFO_0004514	Other measurement	Skeletal
0.7926	8.57E-100	1.48E-96	PGS000476	EFO_0000305	breast carcinoma	breast carcinoma	EFO_0000305	Cancer	Neoplasms	PGS000474	EFO_0000305	breast carcinoma	breast carcinoma	EFO_0000305	Cancer	Neoplasms
0.7926	8.58E-100	1.48E-96	PGS002388	EFO_0000319	cardiovascular disease	cardiovascular disease	EFO_0000319	Cardiovascular disease	Cardiovascular	PGS002535	EFO_0000319	cardiovascular disease	cardiovascular disease	EFO_0000319	Cardiovascular disease	Cardiovascular
0.7925	8.82E-100	1.52E-96	PGS001477	EFO_0006930	brain volume measurement|neuroimaging measurement	brain volume measurement	EFO_0006930	Other measurement	Neurological	PGS001481	EFO_0006930	measurement	brain volume measurement	EFO_0006930	Other measurement	Neurological
0.7924	9.80E-100	1.69E-96	PGS001917	EFO_0007800	body fat percentage	body fat percentage	EFO_0007800	Body measurement	Body measurement	PGS001943	EFO_0004340	body mass index	body mass index	EFO_0004340	Body measurement	Body measurement
0.7924	1.02E-99	1.77E-96	PGS002672	EFO_0004631	vitamin D measurement	vitamin D measurement	EFO_0004631	Lipid or lipoprotein measurement	Metabolic	PGS003191	EFO_0004631	vitamin D measurement	vitamin D measurement	EFO_0004631	Lipid or lipoprotein measurement	Metabolic
0.7923	1.09E-99	1.89E-96	PGS002672	EFO_0004631	vitamin D measurement	vitamin D measurement	EFO_0004631	Lipid or lipoprotein measurement	Metabolic	PGS002623	EFO_0004631	vitamin D measurement	vitamin D measurement	EFO_0004631	Lipid or lipoprotein measurement	Metabolic
0.7922	1.20E-99	2.06E-96	PGS002964	EFO_0004339	body height	body height	EFO_0004339	Body measurement	Body measurement	PGS001929	EFO_0004339	body height	body height	EFO_0004339	Body measurement	Body measurement
0.7922	1.22E-99	2.11E-96	PGS002287	EFO_0004530	triglyceride measurement	triglyceride measurement	EFO_0004530	Lipid or lipoprotein measurement	Metabolic	PGS002285	EFO_0004611	low density lipoprotein cholesterol measurement	measurement	EFO_0004611	Cardiovascular measurement	Cardiovascular
0.7922	1.22E-99	2.11E-96	PGS002287	EFO_0004530	triglyceride measurement	triglyceride measurement	EFO_0004530	Lipid or lipoprotein measurement	Metabolic	PGS002285	EFO_0004611	low density lipoprotein cholesterol measurement	measurement	EFO_0004611	Lipid or lipoprotein measurement	Metabolic
0.7921	1.27E-99	2.19E-96	PGS001297	MONDO_0005147	type 1 diabetes mellitus	type 1 diabetes mellitus	MONDO_0005147	Metabolic disorder	Metabolic	PGS000023	MONDO_0005147	type 1 diabetes mellitus	type 1 diabetes mellitus	MONDO_0005147	Immune system disorder	Immunological
0.7921	1.27E-99	2.19E-96	PGS001297	MONDO_0005147	type 1 diabetes mellitus	type 1 diabetes mellitus	MONDO_0005147	Immune system disorder	Immunological	PGS000023	MONDO_0005147	type 1 diabetes mellitus	type 1 diabetes mellitus	MONDO_0005147	Digestive system disorder	Gastrointestinal
0.7921	1.27E-99	2.19E-96	PGS001297	MONDO_0005147	type 1 diabetes mellitus	type 1 diabetes mellitus	MONDO_0005147	Metabolic disorder	Metabolic	PGS000023	MONDO_0005147	type 1 diabetes mellitus	type 1 diabetes mellitus	MONDO_0005147	Metabolic disorder	Metabolic
0.7921	1.27E-99	2.19E-96	PGS001297	MONDO_0005147	type 1 diabetes mellitus	type 1 diabetes mellitus	MONDO_0005147	Digestive system disorder	Gastrointestinal	PGS000023	MONDO_0005147	type 1 diabetes mellitus	type 1 diabetes mellitus	MONDO_0005147	Immune system disorder	Immunological
0.7921	1.27E-99	2.19E-96	PGS001297	MONDO_0005147	type 1 diabetes mellitus	type 1 diabetes mellitus	MONDO_0005147	Metabolic disorder	Metabolic	PGS000023	MONDO_0005147	type 1 diabetes mellitus	type 1 diabetes mellitus	MONDO_0005147	Digestive system disorder	Gastrointestinal
0.7921	1.27E-99	2.19E-96	PGS001297	MONDO_0005147	type 1 diabetes mellitus	type 1 diabetes mellitus	MONDO_0005147	Immune system disorder	Immunological	PGS000023	MONDO_0005147	type 1 diabetes mellitus	type 1 diabetes mellitus	MONDO_0005147	Metabolic disorder	Metabolic
0.7921	1.27E-99	2.19E-96	PGS001297	MONDO_0005147	type 1 diabetes mellitus	type 1 diabetes mellitus	MONDO_0005147	Digestive system disorder	Gastrointestinal	PGS000023	MONDO_0005147	type 1 diabetes mellitus	type 1 diabetes mellitus	MONDO_0005147	Digestive system disorder	Gastrointestinal
0.7921	1.27E-99	2.19E-96	PGS001297	MONDO_0005147	type 1 diabetes mellitus	type 1 diabetes mellitus	MONDO_0005147	Digestive system disorder	Gastrointestinal	PGS000023	MONDO_0005147	type 1 diabetes mellitus	type 1 diabetes mellitus	MONDO_0005147	Metabolic disorder	Metabolic
0.7921	1.27E-99	2.19E-96	PGS001297	MONDO_0005147	type 1 diabetes mellitus	type 1 diabetes mellitus	MONDO_0005147	Immune system disorder	Immunological	PGS000023	MONDO_0005147	type 1 diabetes mellitus	type 1 diabetes mellitus	MONDO_0005147	Immune system disorder	Immunological
0.7921	1.33E-99	2.29E-96	PGS001957	EFO_0005654	velocity of sound measurement	velocity of sound measurement	EFO_0005654	Other measurement	Others	PGS000951	EFO_0004514	bone quantitative ultrasound measurement	measurement	EFO_0004514	Other measurement	Skeletal
0.7921	1.38E-99	2.38E-96	PGS001673	EFO_0006930	brain volume measurement|neuroimaging measurement	brain volume measurement	EFO_0006930	Other measurement	Neurological	PGS001481	EFO_0006930	measurement	brain volume measurement	EFO_0006930	Other measurement	Neurological
0.792	1.43E-99	2.46E-96	PGS002332	EFO_0004339	body height	body height	EFO_0004339	Body measurement	Body measurement	PGS002969	EFO_0004339	body height	body height	EFO_0004339	Body measurement	Body measurement
0.792	1.50E-99	2.58E-96	PGS000516	EFO_0000305	breast carcinoma	breast carcinoma	EFO_0000305	Cancer	Neoplasms	PGS001336	EFO_0000305	breast carcinoma	breast carcinoma	EFO_0000305	Cancer	Neoplasms
0.7918	1.72E-99	2.96E-96	PGS001840	MONDO_0004596	cor pulmonale	cor pulmonale	MONDO_0004596	Cardiovascular disease	Cardiovascular	PGS001279	EFO_0003827	pulmonary embolism|deep vein thrombosis	pulmonary embolism	EFO_0003827	Cardiovascular disease	Cardiovascular
0.7918	1.73E-99	2.97E-96	PGS002426	MONDO_0005148	type 2 diabetes mellitus	type 2 diabetes mellitus	MONDO_0005148	Metabolic disorder	Metabolic	PGS002501	EFO_0004541	HbA1c measurement	HbA1c measurement	EFO_0004541	Hematological measurement	Hematological
0.7918	1.73E-99	2.97E-96	PGS002426	MONDO_0005148	type 2 diabetes mellitus	type 2 diabetes mellitus	MONDO_0005148	Digestive system disorder	Gastrointestinal	PGS002501	EFO_0004541	HbA1c measurement	HbA1c measurement	EFO_0004541	Hematological measurement	Hematological
0.7918	1.77E-99	3.05E-96	PGS002393	EFO_0000400	diabetes mellitus	diabetes mellitus	EFO_0000400	Metabolic disorder	Metabolic	PGS002501	EFO_0004541	HbA1c measurement	HbA1c measurement	EFO_0004541	Hematological measurement	Hematological
0.7918	1.77E-99	3.05E-96	PGS002393	EFO_0000400	diabetes mellitus	diabetes mellitus	EFO_0000400	Digestive system disorder	Gastrointestinal	PGS002501	EFO_0004541	HbA1c measurement	HbA1c measurement	EFO_0004541	Hematological measurement	Hematological
0.7918	1.79E-99	3.07E-96	PGS001670	EFO_0006930	brain volume measurement|neuroimaging measurement	brain volume measurement	EFO_0006930	Other measurement	Neurological	PGS001671	EFO_0006930	measurement	brain volume measurement	EFO_0006930	Other measurement	Neurological
0.7918	1.80E-99	3.08E-96	PGS001847	EFO_0004264	vascular disease	vascular disease	EFO_0004264	Cardiovascular disease	Cardiovascular	PGS001279	EFO_0003827	pulmonary embolism|deep vein thrombosis	pulmonary embolism	EFO_0003827	Cardiovascular disease	Cardiovascular
0.7917	1.92E-99	3.30E-96	PGS000002	EFO_1000649	estrogen-receptor positive breast cancer	estrogen-receptor positive breast cancer	EFO_1000649	Cancer	Neoplasms	PGS000873	EFO_0000305	breast carcinoma	breast carcinoma	EFO_0000305	Cancer	Neoplasms
0.7917	1.93E-99	3.32E-96	PGS002148	EFO_0009695	household income	household income	EFO_0009695	Other trait	Others	PGS001931	EFO_0009695	household income	household income	EFO_0009695	Other trait	Others
0.7916	2.12E-99	3.63E-96	PGS002550	EFO_0004541	HbA1c measurement	HbA1c measurement	EFO_0004541	Hematological measurement	Hematological	PGS002547	EFO_0004468	glucose measurement	glucose measurement	EFO_0004468	Other measurement	Metabolic
0.7916	2.13E-99	3.66E-96	PGS002466	EFO_0004305	erythrocyte count	erythrocyte count	EFO_0004305	Hematological measurement	Hematological	PGS002515	EFO_0004305	erythrocyte count	erythrocyte count	EFO_0004305	Hematological measurement	Hematological
0.7916	2.26E-99	3.88E-96	PGS000169	EFO_0007986	reticulocyte count	reticulocyte count	EFO_0007986	Hematological measurement	Hematological	PGS000190	EFO_0007986	reticulocyte count	reticulocyte count	EFO_0007986	Hematological measurement	Hematological
0.7915	2.46E-99	4.22E-96	PGS001229	EFO_0004339	body height	body height	EFO_0004339	Body measurement	Body measurement	PGS000758	EFO_0004339	body height	body height	EFO_0004339	Body measurement	Body measurement
0.7915	2.48E-99	4.24E-96	PGS000591	EFO_0001663	prostate carcinoma	prostate carcinoma	EFO_0001663	Cancer	Neoplasms	PGS000593	EFO_0007355	male reproductive organ cancer	male reproductive organ cancer	EFO_0007355	Cancer	Neoplasms
0.7914	2.52E-99	4.32E-96	PGS002163	EFO_0004518	creatinine measurement	creatinine measurement	EFO_0004518	Other measurement	Metabolic	PGS003248	EFO_0010472	creatine measurement	creatine measurement	EFO_0010472	Other measurement	Metabolic
0.7912	3.14E-99	5.38E-96	PGS002839	EFO_0007878	alcohol consumption measurement	alcohol consumption measurement	EFO_0007878	Other measurement	Psychiatric	PGS002830	EFO_0007878	alcohol consumption measurement	alcohol consumption measurement	EFO_0007878	Other measurement	Psychiatric
0.7911	3.47E-99	5.94E-96	PGS000333	EFO_0001663	prostate carcinoma	prostate carcinoma	EFO_0001663	Cancer	Neoplasms	PGS002240	EFO_0001663	prostate carcinoma	prostate carcinoma	EFO_0001663	Cancer	Neoplasms
0.7911	3.57E-99	6.10E-96	PGS003020	EFO_0000537	hypertension	hypertension	EFO_0000537	Cardiovascular disease	Cardiovascular	PGS003004	EFO_0000537	hypertension	hypertension	EFO_0000537	Cardiovascular disease	Cardiovascular
0.7911	3.57E-99	6.10E-96	PGS003021	EFO_0000537	hypertension	hypertension	EFO_0000537	Cardiovascular disease	Cardiovascular	PGS003004	EFO_0000537	hypertension	hypertension	EFO_0000537	Cardiovascular disease	Cardiovascular
0.7911	3.62E-99	6.19E-96	PGS000525	EFO_0000305	breast carcinoma	breast carcinoma	EFO_0000305	Cancer	Neoplasms	PGS000498	EFO_0000305	breast carcinoma	breast carcinoma	EFO_0000305	Cancer	Neoplasms
0.7911	3.62E-99	6.19E-96	PGS000525	EFO_0000305	breast carcinoma	breast carcinoma	EFO_0000305	Cancer	Neoplasms	PGS000497	EFO_0000305	breast carcinoma	breast carcinoma	EFO_0000305	Cancer	Neoplasms
0.7911	3.62E-99	6.19E-96	PGS000526	EFO_0000305	breast carcinoma	breast carcinoma	EFO_0000305	Cancer	Neoplasms	PGS000497	EFO_0000305	breast carcinoma	breast carcinoma	EFO_0000305	Cancer	Neoplasms
0.7911	3.62E-99	6.19E-96	PGS000526	EFO_0000305	breast carcinoma	breast carcinoma	EFO_0000305	Cancer	Neoplasms	PGS000498	EFO_0000305	breast carcinoma	breast carcinoma	EFO_0000305	Cancer	Neoplasms
0.791	3.85E-99	6.58E-96	PGS003313	EFO_0004617	cystatin C measurement|glomerular filtration rate	cystatin C measurement	EFO_0004617	Other measurement	Metabolic	PGS003295	EFO_0004617	cystatin C measurement|glomerular filtration rate	cystatin C measurement	EFO_0004617	Other measurement	Metabolic
0.791	3.88E-99	6.62E-96	PGS003380	EFO_0000305	breast carcinoma	breast carcinoma	EFO_0000305	Cancer	Neoplasms	PGS000499	EFO_0000305	breast carcinoma	breast carcinoma	EFO_0000305	Cancer	Neoplasms
0.791	3.93E-99	6.70E-96	PGS001477	EFO_0006930	brain volume measurement|neuroimaging measurement	brain volume measurement	EFO_0006930	Other measurement	Neurological	PGS001673	EFO_0006930	measurement	brain volume measurement	EFO_0006930	Other measurement	Neurological
-0.791	3.98E-99	6.79E-96	PGS000883	EFO_0005208	glomerular filtration rate	glomerular filtration rate	EFO_0005208	Other measurement	Genitourinary	PGS003223	EFO_0005208	glomerular filtration rate	glomerular filtration rate	EFO_0005208	Other measurement	Genitourinary
0.7909	4.07E-99	6.94E-96	PGS002102	EFO_0004615	apolipoprotein B measurement	apolipoprotein B measurement	EFO_0004615	Lipid or lipoprotein measurement	Metabolic	PGS002654	EFO_0004611	low density lipoprotein cholesterol measurement	measurement	EFO_0004611	Cardiovascular measurement	Cardiovascular
0.7909	4.07E-99	6.94E-96	PGS002102	EFO_0004615	apolipoprotein B measurement	apolipoprotein B measurement	EFO_0004615	Lipid or lipoprotein measurement	Metabolic	PGS002654	EFO_0004611	low density lipoprotein cholesterol measurement	measurement	EFO_0004611	Lipid or lipoprotein measurement	Metabolic
0.7908	4.54E-99	7.75E-96	PGS002543	EFO_0001379	endocrine system disease|diabetes mellitus	endocrine system disease	EFO_0001379	Other disease	Metabolic	PGS002491	EFO_0000400	diabetes mellitus	diabetes mellitus	EFO_0000400	Digestive system disorder	Gastrointestinal
0.7908	4.54E-99	7.75E-96	PGS002543	EFO_0001379	endocrine system disease|diabetes mellitus	endocrine system disease	EFO_0001379	Other disease	Metabolic	PGS002491	EFO_0000400	diabetes mellitus	diabetes mellitus	EFO_0000400	Metabolic disorder	Metabolic
0.7907	4.92E-99	8.39E-96	PGS002462	EFO_0005091	monocyte count	monocyte count	EFO_0005091	Hematological measurement	Hematological	PGS002560	EFO_0005091	monocyte count	monocyte count	EFO_0005091	Hematological measurement	Hematological
0.7907	5.24E-99	8.91E-96	PGS000534	EFO_0000305	breast carcinoma	breast carcinoma	EFO_0000305	Cancer	Neoplasms	PGS000505	EFO_0000305	breast carcinoma	breast carcinoma	EFO_0000305	Cancer	Neoplasms
0.7907	5.24E-99	8.91E-96	PGS000533	EFO_0000305	breast carcinoma	breast carcinoma	EFO_0000305	Cancer	Neoplasms	PGS000506	EFO_0000305	breast carcinoma	breast carcinoma	EFO_0000305	Cancer	Neoplasms
0.7907	5.24E-99	8.91E-96	PGS000533	EFO_0000305	breast carcinoma	breast carcinoma	EFO_0000305	Cancer	Neoplasms	PGS000505	EFO_0000305	breast carcinoma	breast carcinoma	EFO_0000305	Cancer	Neoplasms
0.7907	5.24E-99	8.91E-96	PGS000534	EFO_0000305	breast carcinoma	breast carcinoma	EFO_0000305	Cancer	Neoplasms	PGS000506	EFO_0000305	breast carcinoma	breast carcinoma	EFO_0000305	Cancer	Neoplasms
0.7907	5.36E-99	9.11E-96	PGS000296	EFO_0001645	coronary artery disease	coronary artery disease	EFO_0001645	Cardiovascular disease	Cardiovascular	PGS001780	EFO_0001645	coronary artery disease	coronary artery disease	EFO_0001645	Cardiovascular disease	Cardiovascular
0.7905	6.32E-99	1.07E-95	PGS002173	EFO_0009270	heel bone mineral density	heel bone mineral density	EFO_0009270	Other measurement	Skeletal	PGS002632	EFO_0003923	bone density	bone density	EFO_0003923	Other measurement	Skeletal
0.7905	6.46E-99	1.10E-95	PGS001247	EFO_0004279	suntan	suntan	EFO_0004279	Other trait	Dermatological	PGS001257	EFO_0003958	sunburn	sunburn	EFO_0003958	Other disease	Dermatological
0.7904	6.60E-99	1.12E-95	PGS000400	EFO_0009259	skin carcinoma	skin carcinoma	EFO_0009259	Cancer	Neoplasms	PGS000402	EFO_0009259	skin carcinoma	skin carcinoma	EFO_0009259	Cancer	Neoplasms
0.7903	7.41E-99	1.26E-95	PGS002325	EFO_0004842	eosinophil count	eosinophil count	EFO_0004842	Hematological measurement	Hematological	PGS001172	EFO_0004842	eosinophil count	eosinophil count	EFO_0004842	Hematological measurement	Hematological
0.7903	7.44E-99	1.26E-95	PGS002206	EFO_0004527	mean corpuscular hemoglobin	mean corpuscular hemoglobin	EFO_0004527	Hematological measurement	Hematological	PGS001219	EFO_0004527	mean corpuscular hemoglobin	mean corpuscular hemoglobin	EFO_0004527	Hematological measurement	Hematological
0.7903	7.68E-99	1.30E-95	PGS002107	EFO_0001060	celiac disease	celiac disease	EFO_0001060	Digestive system disorder	Gastrointestinal	PGS001065	EFO_0008111	diet measurement	diet measurement	EFO_0008111	Other measurement	Activities
0.7903	7.68E-99	1.30E-95	PGS002107	EFO_0001060	celiac disease	celiac disease	EFO_0001060	Immune system disorder	Immunological	PGS001065	EFO_0008111	diet measurement	diet measurement	EFO_0008111	Other measurement	Activities
0.7902	8.55E-99	1.45E-95	PGS001169	EFO_0004995	lean body mass	lean body mass	EFO_0004995	Other measurement	Skeletal	PGS001984	EFO_0009805	whole body water mass	whole body water mass	EFO_0009805	Other measurement	Body measurement
0.7902	8.60E-99	1.46E-95	PGS001840	MONDO_0004596	cor pulmonale	cor pulmonale	MONDO_0004596	Cardiovascular disease	Cardiovascular	PGS000930	MONDO_0000831	thrombotic disease	thrombotic disease	MONDO_0000831	Cardiovascular disease	Cardiovascular
0.7902	8.72E-99	1.48E-95	PGS000841	EFO_0004340	body mass index	body mass index	EFO_0004340	Body measurement	Body measurement	PGS003400	EFO_0001073	obesity	obesity	EFO_0001073	Metabolic disorder	Metabolic
0.7901	9.47E-99	1.60E-95	PGS000433	MONDO_0002898	skin cancer	skin cancer	MONDO_0002898	Cancer	Neoplasms	PGS000404	EFO_0009259	skin carcinoma	skin carcinoma	EFO_0009259	Cancer	Neoplasms
0.7899	1.07E-98	1.82E-95	PGS000504	EFO_0000305	breast carcinoma	breast carcinoma	EFO_0000305	Cancer	Neoplasms	PGS000507	EFO_0000305	breast carcinoma	breast carcinoma	EFO_0000305	Cancer	Neoplasms
0.7899	1.10E-98	1.87E-95	PGS001476	EFO_0006930	brain volume measurement|neuroimaging measurement	brain volume measurement	EFO_0006930	Other measurement	Neurological	PGS001480	EFO_0006930	measurement	brain volume measurement	EFO_0006930	Other measurement	Neurological
0.7897	1.29E-98	2.19E-95	PGS000117	EFO_0000319	cardiovascular disease	cardiovascular disease	EFO_0000319	Cardiovascular disease	Cardiovascular	PGS000116	EFO_0001645	coronary artery disease	coronary artery disease	EFO_0001645	Cardiovascular disease	Cardiovascular
0.7897	1.35E-98	2.29E-95	PGS002483	EFO_0004340	body mass index	body mass index	EFO_0004340	Body measurement	Body measurement	PGS002434	EFO_0004340	body mass index	body mass index	EFO_0004340	Body measurement	Body measurement
0.7897	1.40E-98	2.36E-95	PGS002643	EFO_0004713	FEV|FEC ratio	FEV|FEC ratio	EFO_0004713	Other measurement	Respiratory	PGS002326	EFO_0004713	FEV|FEC ratio	FEV|FEC ratio	EFO_0004713	Other measurement	Respiratory
0.7896	1.41E-98	2.39E-95	PGS001169	EFO_0004995	lean body mass	lean body mass	EFO_0004995	Other measurement	Skeletal	PGS001950	EFO_0004995	lean body mass	lean body mass	EFO_0004995	Other measurement	Skeletal
0.7895	1.56E-98	2.63E-95	PGS000523	EFO_0000305	breast carcinoma	breast carcinoma	EFO_0000305	Cancer	Neoplasms	PGS000522	EFO_0000305	breast carcinoma	breast carcinoma	EFO_0000305	Cancer	Neoplasms
0.7895	1.68E-98	2.84E-95	PGS001729	EFO_0006930	brain volume measurement|neuroimaging measurement	brain volume measurement	EFO_0006930	Other measurement	Neurological	PGS001744	EFO_0006930	measurement	brain volume measurement	EFO_0006930	Other measurement	Neurological
0.7894	1.71E-98	2.88E-95	PGS002550	EFO_0004541	HbA1c measurement	HbA1c measurement	EFO_0004541	Hematological measurement	Hematological	PGS002396	EFO_0001379	endocrine system disease|diabetes mellitus	endocrine system disease	EFO_0001379	Other disease	Metabolic
0.7894	1.72E-98	2.91E-95	PGS000400	EFO_0009259	skin carcinoma	skin carcinoma	EFO_0009259	Cancer	Neoplasms	PGS000404	EFO_0009259	skin carcinoma	skin carcinoma	EFO_0009259	Cancer	Neoplasms
0.7894	1.84E-98	3.10E-95	PGS003103	MONDO_0005148	type 2 diabetes mellitus	type 2 diabetes mellitus	MONDO_0005148	Metabolic disorder	Metabolic	PGS002308	MONDO_0005148	type 2 diabetes mellitus	type 2 diabetes mellitus	MONDO_0005148	Metabolic disorder	Metabolic
0.7894	1.84E-98	3.10E-95	PGS003103	MONDO_0005148	type 2 diabetes mellitus	type 2 diabetes mellitus	MONDO_0005148	Digestive system disorder	Gastrointestinal	PGS002308	MONDO_0005148	type 2 diabetes mellitus	type 2 diabetes mellitus	MONDO_0005148	Metabolic disorder	Metabolic
0.7894	1.84E-98	3.10E-95	PGS003103	MONDO_0005148	type 2 diabetes mellitus	type 2 diabetes mellitus	MONDO_0005148	Digestive system disorder	Gastrointestinal	PGS002308	MONDO_0005148	type 2 diabetes mellitus	type 2 diabetes mellitus	MONDO_0005148	Digestive system disorder	Gastrointestinal
0.7894	1.84E-98	3.10E-95	PGS003103	MONDO_0005148	type 2 diabetes mellitus	type 2 diabetes mellitus	MONDO_0005148	Metabolic disorder	Metabolic	PGS002308	MONDO_0005148	type 2 diabetes mellitus	type 2 diabetes mellitus	MONDO_0005148	Digestive system disorder	Gastrointestinal
0.7894	1.85E-98	3.13E-95	PGS001026	EFO_0009514	upper extremity fracture	upper extremity fracture	EFO_0009514	Other disease	Skeletal	PGS001258	EFO_0003957	radius fracture	radius fracture	EFO_0003957	Other disease	Skeletal
0.7893	1.93E-98	3.25E-95	PGS000511	EFO_0000305	breast carcinoma	breast carcinoma	EFO_0000305	Cancer	Neoplasms	PGS000499	EFO_0000305	breast carcinoma	breast carcinoma	EFO_0000305	Cancer	Neoplasms
0.7893	2.06E-98	3.47E-95	PGS000203	EFO_0007878	alcohol consumption measurement	alcohol consumption measurement	EFO_0007878	Other measurement	Psychiatric	PGS002830	EFO_0007878	alcohol consumption measurement	alcohol consumption measurement	EFO_0007878	Other measurement	Psychiatric
0.7891	2.45E-98	4.13E-95	PGS003273	EFO_0010472	creatine measurement|glomerular filtration rate	creatine measurement	EFO_0010472	Other measurement	Metabolic	PGS003254	EFO_0010472	creatine measurement|glomerular filtration rate	creatine measurement	EFO_0010472	Other measurement	Metabolic

0.789	2.67E-98	4.49E-95	PGS001989	EFO_0004527	mean corpuscular hemoglobin	mean corpuscular hemoglobin	EFO_0004527	Hematological measurement	Hematological	PGS001219	EFO_0004527	mean corpuscular hemoglobin	mean corpuscular hemoglobin	EFO_0004527	Hematological measurement	Hematological
0.789	2.72E-98	4.58E-95	PGS002150	EFO_0004611	low density lipoprotein cholesterol measurement	low density lipoprotein cholesterol measurement	EFO_0004611	Lipid or lipoprotein measurement	Metabolic	PGS000672	EFO_0004615	apolipoprotein B measurement	apolipoprotein B measurement	EFO_0004615	Lipid or lipoprotein measurement	Metabolic
0.789	2.72E-98	4.58E-95	PGS002150	EFO_0004611	low density lipoprotein cholesterol measurement	low density lipoprotein cholesterol measurement	EFO_0004611	Cardiovascular measurement	Cardiovascular	PGS000672	EFO_0004615	apolipoprotein B measurement	apolipoprotein B measurement	EFO_0004615	Lipid or lipoprotein measurement	Metabolic
0.789	2.74E-98	4.60E-95	PGS002107	EFO_0001060	celiac disease	celiac disease	EFO_0001060	Digestive system disorder	Gastrointestinal	PGS001301	EFO_0001060	celiac disease	celiac disease	EFO_0001060	Digestive system disorder	Gastrointestinal
0.789	2.74E-98	4.60E-95	PGS002107	EFO_0001060	celiac disease	celiac disease	EFO_0001060	Digestive system disorder	Gastrointestinal	PGS001301	EFO_0001060	celiac disease	celiac disease	EFO_0001060	Immune system disorder	Immunological
0.789	2.74E-98	4.60E-95	PGS002107	EFO_0001060	celiac disease	celiac disease	EFO_0001060	Immune system disorder	Immunological	PGS001301	EFO_0001060	celiac disease	celiac disease	EFO_0001060	Immune system disorder	Immunological
0.789	2.74E-98	4.60E-95	PGS002107	EFO_0001060	celiac disease	celiac disease	EFO_0001060	Immune system disorder	Immunological	PGS001301	EFO_0001060	celiac disease	celiac disease	EFO_0001060	Digestive system disorder	Gastrointestinal
0.789	2.75E-98	4.62E-95	PGS001511	EFO_0004346	neuroimaging measurement	neuroimaging measurement	EFO_0004346	Other measurement	Neurological	PGS001512	EFO_0004346	neuroimaging measurement	neuroimaging measurement	EFO_0004346	Other measurement	Neurological
0.7889	2.76E-98	4.64E-95	PGS000274	EFO_0007937	blood protein measurement	blood protein measurement	EFO_0007937	Other measurement	Hematological	PGS000233	EFO_0007937	blood protein measurement	blood protein measurement	EFO_0007937	Other measurement	Hematological
0.7889	2.78E-98	4.67E-95	PGS001683	EFO_0006930	brain volume measurement|neuroimaging measurement	brain volume measurement	EFO_0006930	Other measurement	Neurological	PGS001450	EFO_0006930	measurement	brain volume measurement	EFO_0006930	Other measurement	Neurological
0.7889	2.96E-98	4.97E-95	PGS001671	EFO_0006930	brain volume measurement|neuroimaging measurement	brain volume measurement	EFO_0006930	Other measurement	Neurological	PGS001480	EFO_0006930	measurement	brain volume measurement	EFO_0006930	Other measurement	Neurological
0.7888	3.20E-98	5.37E-95	PGS002993	EFO_0004339	body height	body height	EFO_0004339	Body measurement	Body measurement	PGS002973	EFO_0004339	body height	body height	EFO_0004339	Body measurement	Body measurement
0.7887	3.43E-98	5.77E-95	PGS000296	EFO_0001645	coronary artery disease	coronary artery disease	EFO_0001645	Cardiovascular disease	Cardiovascular	PGS003355	EFO_0001645	coronary artery disease	coronary artery disease	EFO_0001645	Cardiovascular disease	Cardiovascular
0.7886	3.75E-98	6.30E-95	PGS002456	EFO_0000537	hypertension	hypertension	EFO_0000537	Cardiovascular disease	Cardiovascular	PGS002407	EFO_0000537	hypertension	hypertension	EFO_0000537	Cardiovascular disease	Cardiovascular
0.7886	3.90E-98	6.54E-95	PGS002983	EFO_0004339	body height	body height	EFO_0004339	Body measurement	Body measurement	PGS002973	EFO_0004339	body height	body height	EFO_0004339	Body measurement	Body measurement
0.7885	4.29E-98	7.19E-95	PGS002351	EFO_1000627	thyroid disease	thyroid disease	EFO_1000627	Other disease	Endocrine	PGS002312	EFO_0005140	autoimmune disease	autoimmune disease	EFO_0005140	Immune system disorder	Immunological
0.7884	4.75E-98	7.97E-95	PGS001998	EFO_0010968	phosphate measurement	phosphate measurement	EFO_0010968	Other measurement	Metabolic	PGS000692	EFO_0010968	phosphate measurement	phosphate measurement	EFO_0010968	Other measurement	Metabolic
0.7883	4.95E-98	8.29E-95	PGS002802	EFO_0004339	body height	body height	EFO_0004339	Body measurement	Body measurement	PGS001929	EFO_0004339	body height	body height	EFO_0004339	Body measurement	Body measurement
0.788	6.61E-98	1.11E-94	PGS002501	EFO_0004541	HbA1c measurement	HbA1c measurement	EFO_0004541	Hematological measurement	Hematological	PGS002396	EFO_0001379	endocrine system disease|diabetes mellitus	endocrine system disease	EFO_0001379	Other disease	Metabolic
0.788	6.87E-98	1.15E-94	PGS001670	EFO_0006930	brain volume measurement|neuroimaging measurement	brain volume measurement	EFO_0006930	Other measurement	Neurological	PGS001672	EFO_0006930	measurement	brain volume measurement	EFO_0006930	Other measurement	Neurological
0.7879	7.32E-98	1.23E-94	PGS003189	EFO_0004631	vitamin D measurement	vitamin D measurement	EFO_0004631	Lipid or lipoprotein measurement	Metabolic	PGS002623	EFO_0004631	vitamin D measurement	vitamin D measurement	EFO_0004631	Lipid or lipoprotein measurement	Metabolic
0.7879	7.63E-98	1.28E-94	PGS002964	EFO_0004339	body height	body height	EFO_0004339	Body measurement	Body measurement	PGS002332	EFO_0004339	body height	body height	EFO_0004339	Body measurement	Body measurement
0.7878	7.91E-98	1.32E-94	PGS002122	EFO_0005192	red blood cell distribution width	red blood cell distribution width	EFO_0005192	Hematological measurement	Hematological	PGS002663	EFO_0005192	red blood cell distribution width	red blood cell distribution width	EFO_0005192	Hematological measurement	Hematological
0.7878	8.50E-98	1.42E-94	PGS002648	EFO_0004541	HbA1c measurement	HbA1c measurement	EFO_0004541	Hematological measurement	Hematological	PGS002331	EFO_0004541	HbA1c measurement	HbA1c measurement	EFO_0004541	Hematological measurement	Hematological
0.7877	8.88E-98	1.49E-94	PGS000007	EFO_0000305	breast carcinoma	breast carcinoma	EFO_0000305	Cancer	Neoplasms	PGS000004	EFO_0000305	breast carcinoma	breast carcinoma	EFO_0000305	Cancer	Neoplasms
0.7877	8.98E-98	1.50E-94	PGS003238	EFO_0010472	creatine measurement	creatine measurement	EFO_0010472	Other measurement	Metabolic	PGS002162	EFO_0007934	creatinine clearance measurement	creatinine clearance measurement	EFO_0007934	Other measurement	Metabolic
0.7877	9.02E-98	1.51E-94	PGS000530	EFO_0000305	breast carcinoma	breast carcinoma	EFO_0000305	Cancer	Neoplasms	PGS000506	EFO_0000305	breast carcinoma	breast carcinoma	EFO_0000305	Cancer	Neoplasms
0.7877	9.02E-98	1.51E-94	PGS000530	EFO_0000305	breast carcinoma	breast carcinoma	EFO_0000305	Cancer	Neoplasms	PGS000505	EFO_0000305	breast carcinoma	breast carcinoma	EFO_0000305	Cancer	Neoplasms
0.7876	9.60E-98	1.60E-94	PGS001682	EFO_0006930	brain volume measurement|neuroimaging measurement	brain volume measurement	EFO_0006930	Other measurement	Neurological	PGS001450	EFO_0006930	measurement	brain volume measurement	EFO_0006930	Other measurement	Neurological
0.7875	1.04E-97	1.74E-94	PGS001929	EFO_0004339	body height	body height	EFO_0004339	Body measurement	Body measurement	PGS002224	EFO_0011011	sitting height measurement	sitting height measurement	EFO_0011011	Other measurement	Skeletal
0.7875	1.05E-97	1.75E-94	PGS002547	EFO_0004468	glucose measurement	glucose measurement	EFO_0004468	Other measurement	Metabolic	PGS002599	EFO_0004541	HbA1c measurement	HbA1c measurement	EFO_0004541	Hematological measurement	Hematological
0.7875	1.06E-97	1.76E-94	PGS002192	EFO_0010952	urinary potassium measurement	urinary potassium measurement	EFO_0010952	Other measurement	Metabolic	PGS001974	EFO_0010952	urinary potassium measurement	urinary potassium measurement	EFO_0010952	Other measurement	Metabolic
0.7875	1.06E-97	1.77E-94	PGS002540	EFO_0000400	diabetes mellitus	diabetes mellitus	EFO_0000400	Metabolic disorder	Metabolic	PGS002491	EFO_0000400	diabetes mellitus	diabetes mellitus	EFO_0000400	Metabolic disorder	Metabolic
0.7875	1.06E-97	1.77E-94	PGS002540	EFO_0000400	diabetes mellitus	diabetes mellitus	EFO_0000400	Digestive system disorder	Gastrointestinal	PGS002491	EFO_0000400	diabetes mellitus	diabetes mellitus	EFO_0000400	Metabolic disorder	Metabolic
0.7875	1.06E-97	1.77E-94	PGS002540	EFO_0000400	diabetes mellitus	diabetes mellitus	EFO_0000400	Digestive system disorder	Gastrointestinal	PGS002491	EFO_0000400	diabetes mellitus	diabetes mellitus	EFO_0000400	Digestive system disorder	Gastrointestinal
0.7875	1.06E-97	1.77E-94	PGS002540	EFO_0000400	diabetes mellitus	diabetes mellitus	EFO_0000400	Metabolic disorder	Metabolic	PGS002491	EFO_0000400	diabetes mellitus	diabetes mellitus	EFO_0000400	Digestive system disorder	Gastrointestinal
0.7875	1.06E-97	1.77E-94	PGS001220	EFO_0004526	mean corpuscular volume	mean corpuscular volume	EFO_0004526	Hematological measurement	Hematological	PGS000101	EFO_0004526	mean corpuscular volume	mean corpuscular volume	EFO_0004526	Hematological measurement	Hematological
0.7875	1.12E-97	1.87E-94	PGS003111	MONDO_0005148	type 2 diabetes mellitus	type 2 diabetes mellitus	MONDO_0005148	Digestive system disorder	Gastrointestinal	PGS003095	MONDO_0005148	type 2 diabetes mellitus	type 2 diabetes mellitus	MONDO_0005148	Digestive system disorder	Gastrointestinal
0.7875	1.12E-97	1.87E-94	PGS003111	MONDO_0005148	type 2 diabetes mellitus	type 2 diabetes mellitus	MONDO_0005148	Metabolic disorder	Metabolic	PGS003095	MONDO_0005148	type 2 diabetes mellitus	type 2 diabetes mellitus	MONDO_0005148	Metabolic disorder	Metabolic
0.7875	1.12E-97	1.87E-94	PGS003111	MONDO_0005148	type 2 diabetes mellitus	type 2 diabetes mellitus	MONDO_0005148	Digestive system disorder	Gastrointestinal	PGS003095	MONDO_0005148	type 2 diabetes mellitus	type 2 diabetes mellitus	MONDO_0005148	Metabolic disorder	Metabolic
0.7875	1.12E-97	1.87E-94	PGS003111	MONDO_0005148	type 2 diabetes mellitus	type 2 diabetes mellitus	MONDO_0005148	Metabolic disorder	Metabolic	PGS003095	MONDO_0005148	type 2 diabetes mellitus	type 2 diabetes mellitus	MONDO_0005148	Digestive system disorder	Gastrointestinal
0.7874	1.20E-97	2.00E-94	PGS000440	MONDO_0002898	skin cancer	skin cancer	MONDO_0002898	Cancer	Neoplasms	PGS000407	EFO_0009259	skin carcinoma	skin carcinoma	EFO_0009259	Cancer	Neoplasms
0.7874	1.22E-97	2.03E-94	PGS002028	HP_0001943	Hypoglycemia	Hypoglycemia	HP_0001943	Other trait	Metabolic	PGS001296	MONDO_0005147	type 1 diabetes mellitus	type 1 diabetes mellitus	MONDO_0005147	Digestive system disorder	Gastrointestinal
0.7874	1.22E-97	2.03E-94	PGS002028	HP_0001943	Hypoglycemia	Hypoglycemia	HP_0001943	Other trait	Metabolic	PGS001296	MONDO_0005147	type 1 diabetes mellitus	type 1 diabetes mellitus	MONDO_0005147	Immune system disorder	Immunological
0.7874	1.22E-97	2.03E-94	PGS002028	HP_0001943	Hypoglycemia	Hypoglycemia	HP_0001943	Other trait	Metabolic	PGS001296	MONDO_0005147	type 1 diabetes mellitus	type 1 diabetes mellitus	MONDO_0005147	Metabolic disorder	Metabolic
0.7874	1.23E-97	2.04E-94	PGS002990	EFO_0004339	body height	body height	EFO_0004339	Body measurement	Body measurement	PGS002983	EFO_0004339	body height	body height	EFO_0004339	Body measurement	Body measurement
0.7873	1.26E-97	2.09E-94	PGS002337	EFO_0004611	low density lipoprotein cholesterol measurement	low density lipoprotein cholesterol measurement	EFO_0004611	Cardiovascular measurement	Cardiovascular	PGS002764	HP_0003124	Hypercholesterolemia	Hypercholesterolemia	HP_0003124	Other trait	Metabolic
0.7873	1.26E-97	2.09E-94	PGS002337	EFO_0004611	low density lipoprotein cholesterol measurement	low density lipoprotein cholesterol measurement	EFO_0004611	Lipid or lipoprotein measurement	Metabolic	PGS002764	HP_0003124	Hypercholesterolemia	Hypercholesterolemia	HP_0003124	Other trait	Metabolic
0.7873	1.28E-97	2.13E-94	PGS002593	EFO_0004842	eosinophil count	eosinophil count	EFO_0004842	Hematological measurement	Hematological	PGS002446	EFO_0004842	eosinophil count	eosinophil count	EFO_0004842	Hematological measurement	Hematological
0.7872	1.43E-97	2.37E-94	PGS001312	EFO_0000676	psoriasis	psoriasis	EFO_0000676	Immune system disorder	Immunological	PGS001313	EFO_0000676	psoriasis	psoriasis	EFO_0000676	Immune system disorder	Immunological
0.7872	1.44E-97	2.40E-94	PGS000787	EFO_0000681	renal cell carcinoma	renal cell carcinoma	EFO_0000681	Cancer	Neoplasms	PGS000722	EFO_0002890	renal carcinoma	renal carcinoma	EFO_0002890	Cancer	Neoplasms
0.787	1.73E-97	2.88E-94	PGS001173	EFO_0004833	neutrophil count	neutrophil count	EFO_0004833	Hematological measurement	Hematological	PGS001239	EFO_0004308	leukocyte count	leukocyte count	EFO_0004308	Hematological measurement	Hematological
0.7869	1.84E-97	3.05E-94	PGS000058	EFO_0001645	coronary artery disease	coronary artery disease	EFO_0001645	Cardiovascular disease	Cardiovascular	PGS000798	EFO_0001645	coronary artery disease	coronary artery disease	EFO_0001645	Cardiovascular disease	Cardiovascular
0.7868	2.01E-97	3.34E-94	PGS000684	EFO_0004468	glucose measurement	glucose measurement	EFO_0004468	Other measurement	Metabolic	PGS002170	EFO_0004468	glucose measurement	glucose measurement	EFO_0004468	Other measurement	Metabolic
-0.7868	2.13E-97	3.53E-94	PGS000752	EFO_0006925	lipoprotein A measurement	lipoprotein A measurement	EFO_0006925	Lipid or lipoprotein measurement	Metabolic	PGS001963	EFO_0006925	lipoprotein A measurement	lipoprotein A measurement	EFO_0006925	Lipid or lipoprotein measurement	Metabolic
0.7868	2.16E-97	3.58E-94	PGS003243	EFO_0010472	creatine measurement	creatine measurement	EFO_0010472	Other measurement	Metabolic	PGS000883	EFO_0005208	glomerular filtration rate	glomerular filtration rate	EFO_0005208	Other measurement	Genitourinary
-0.7867	2.24E-97	3.72E-94	PGS001095	EFO_0007822	hair colour measurement	hair colour measurement	EFO_0007822	Other measurement	Dermatological	PGS002647	EFO_0007822	hair colour measurement	hair colour measurement	EFO_0007822	Other measurement	Dermatological
0.7867	2.31E-97	3.83E-94	PGS003078	EFO_0006335	systolic blood pressure	systolic blood pressure	EFO_0006335	Other measurement	Cardiovascular	PGS000901	EFO_0006335	systolic blood pressure|male	systolic blood pressure	EFO_0006335	Other measurement	Cardiovascular
0.7865	2.73E-97	4.52E-94	PGS000335	EFO_0000305	breast carcinoma	breast carcinoma	EFO_0000305	Cancer	Neoplasms	PGS000535	EFO_0000305	breast carcinoma	breast carcinoma	EFO_0000305	Cancer	Neoplasms
0.7865	2.86E-97	4.74E-94	PGS001570	EFO_0005420	measurement	grey matter volume measurement	EFO_0005420	Other measurement	Neurological	PGS001569	EFO_0005420	measurement	grey matter volume measurement	EFO_0005420	Other measurement	Neurological
0.7865	2.92E-97	4.83E-94	PGS001953	EFO_0004541	HbA1c measurement	HbA1c measurement	EFO_0004541	Hematological measurement	Hematological	PGS000685	EFO_0004541	HbA1c measurement	HbA1c measurement	EFO_0004541	Hematological measurement	Hematological
0.7864	2.99E-97	4.96E-94	PGS003073	EFO_0006335	systolic blood pressure	systolic blood pressure	EFO_0006335	Other measurement	Cardiovascular	PGS000901	EFO_0006335	systolic blood pressure|male	systolic blood pressure	EFO_0006335	Other measurement	Cardiovascular
0.7864	3.08E-97	5.10E-94	PGS001984	EFO_0009805	whole body water mass	whole body water mass	EFO_0009805	Other measurement	Body measurement	PGS001002	EFO_0009805	whole body water mass	whole body water mass	EFO_0009805	Other measurement	Body measurement
0.7864	3.18E-97	5.26E-94	PGS003118	MONDO_0005148	type 2 diabetes mellitus	type 2 diabetes mellitus	MONDO_0005148	Metabolic disorder	Metabolic	PGS002308	MONDO_0005148	type 2 diabetes mellitus	type 2 diabetes mellitus	MONDO_0005148	Metabolic disorder	Metabolic
0.7864	3.18E-97	5.26E-94	PGS003118	MONDO_0005148	type 2 diabetes mellitus	type 2 diabetes mellitus	MONDO_0005148	Metabolic disorder	Metabolic	PGS002308	MONDO_0005148	type 2 diabetes mellitus	type 2 diabetes mellitus	MONDO_0005148	Digestive system disorder	Gastrointestinal
0.7864	3.18E-97	5.26E-94	PGS003118	MONDO_0005148	type 2 diabetes mellitus	type 2 diabetes mellitus	MONDO_0005148	Digestive system disorder	Gastrointestinal	PGS002308	MONDO_0005148	type 2 diabetes mellitus	type 2 diabetes mellitus	MONDO_0005148	Metabolic disorder	Metabolic
0.7864	3.18E-97	5.26E-94	PGS003118	MONDO_0005148	type 2 diabetes mellitus	type 2 diabetes mellitus	MONDO_0005148	Digestive system disorder	Gastrointestinal	PGS002308	MONDO_0005148	type 2 diabetes mellitus	type 2 diabetes mellitus	MONDO_0005148	Digestive system disorder	Gastrointestinal
0.7863	3.34E-97	5.53E-94	PGS002206	EFO_0004527	mean corpuscular hemoglobin	mean corpuscular hemoglobin	EFO_0004527	Hematological measurement	Hematological	PGS000101	EFO_0004526	mean corpuscular volume	mean corpuscular volume	EFO_0004526	Hematological measurement	Hematological
0.7862	3.68E-97	6.08E-94	PGS002084	EFO_1000767	skin sarcoidosis	skin sarcoidosis	EFO_1000767	Other disease	Others	PGS001872	EFO_1000767	skin sarcoidosis	skin sarcoidosis	EFO_1000767	Other disease	Others
0.7862	3.82E-97	6.32E-94	PGS000575	EFO_0001663	prostate carcinoma	prostate carcinoma	EFO_0001663	Cancer	Neoplasms	PGS000591	EFO_0001663	prostate carcinoma	prostate carcinoma	EFO_0001663	Cancer	Neoplasms
0.7859	4.95E-97	8.18E-94	PGS001917	EFO_0007800	body fat percentage	body fat percentage	EFO_0007800	Body measurement	Body measurement	PGS001983	EFO_0004342	waist circumference	waist circumference	EFO_0004342	Body measurement	Body measurement
-0.7858	5.15E-97	8.51E-94	PGS003228	EFO_0005208	glomerular filtration rate	glomerular filtration rate	EFO_0005208	Other measurement	Genitourinary	PGS000883	EFO_0005208	glomerular filtration rate	glomerular filtration rate	EFO_0005208	Other measurement	Genitourinary
0.7858	5.26E-97	8.68E-94	PGS001970	EFO_0007985	platelet crit	platelet crit	EFO_0007985	Hematological measurement	Hematological	PGS001517	EFO_0007985	platelet crit	platelet crit	EFO_0007985	Hematological measurement	Hematological
0.7858	5.52E-97	9.11E-94	PGS000560	EFO_0003893	ovarian neoplasm	ovarian neoplasm	EFO_0003893	Cancer	Neoplasms	PGS000068	EFO_0001075	ovarian carcinoma	ovarian carcinoma	EFO_0001075	Cancer	Neoplasms
0.7858	5.52E-97	9.11E-94	PGS000559	EFO_0003893	ovarian neoplasm	ovarian neoplasm	EFO_0003893	Cancer	Neoplasms	PGS000068	EFO_0001075	ovarian carcinoma	ovarian carcinoma	EFO_0001075	Cancer	Neoplasms
0.7857	5.67E-97	9.35E-94	PGS003380	EFO_0000305	breast carcinoma	breast carcinoma	EFO_0000305	Cancer	Neoplasms	PGS000500	EFO_0000305	breast carcinoma	breast carcinoma	EFO_0000305	Cancer	Neoplasms
0.7857	5.93E-97	9.77E-94	PGS002474	EFO_0004530	triglyceride measurement	triglyceride measurement	EFO_0004530	Lipid or lipoprotein measurement	Metabolic	PGS002572	EFO_0004530	triglyceride measurement	triglyceride measurement	EFO_0004530	Lipid or lipoprotein measurement	Metabolic
0.7856	6.37E-97	1.05E-93	PGS003199	EFO_0004631	vitamin D measurement	vitamin D measurement	EFO_0004631	Lipid or lipoprotein measurement	Metabolic	PGS002623	EFO_0004631	vitamin D measurement	vitamin D measurement	EFO_0004631	Lipid or lipoprotein measurement	Metabolic
0.7856	6.52E-97	1.08E-93	PGS003189	EFO_0004631	vitamin D measurement	vitamin D measurement	EFO_0004631	Lipid or lipoprotein measurement	Metabolic	PGS002672	EFO_0004631	vitamin D measurement	vitamin D measurement	EFO_0004631	Lipid or lipoprotein measurement	Metabolic
0.7855	7.10E-97	1.17E-93	PGS002605	EFO_0004611	low density lipoprotein cholesterol measurement	low density lipoprotein cholesterol measurement	EFO_0004611	Lipid or lipoprotein measurement	Metabolic	PGS000889	EFO_0004611	low density lipoprotein cholesterol measurement	measurement	EFO_0004611	Lipid or lipoprotein measurement	Metabolic
0.7855	7.10E-97	1.17E-93	PGS002605	EFO_0004611	low density lipoprotein cholesterol measurement	low density lipoprotein cholesterol measurement	EFO_0004611	Lipid or lipoprotein measurement	Metabolic	PGS000889	EFO_0004611	low density lipoprotein cholesterol measurement	measurement	EFO_0004611	Cardiovascular measurement	Cardiovascular
0.7855	7.10E-97	1.17E-93	PGS002605	EFO_0004611	low density lipoprotein cholesterol measurement	low density lipoprotein cholesterol measurement	EFO_0004611	Cardiovascular measurement	Cardiovascular	PGS000889	EFO_0004611	low density lipoprotein cholesterol measurement	measurement	EFO_0004611	Lipid or lipoprotein measurement	Metabolic
0.7855	7.10E-97	1.17E-93	PGS002605	EFO_0004611	low density lipoprotein cholesterol measurement	low density lipoprotein cholesterol measurement	EFO_0004611	Cardiovascular measurement	Cardiovascular	PGS000889	EFO_0004611	low density lipoprotein cholesterol measurement	measurement	EFO_0004611	Cardiovascular measurement	Cardiovascular
0.7855	7.14E-97	1.18E-93	PGS000741	EFO_0001663	prostate carcinoma	prostate carcinoma	EFO_0001663	Cancer	Neoplasms	PGS000742	EFO_0001663	prostate carcinoma	prostate carcinoma	EFO_0001663	Cancer	Neoplasms
0.7854	7.52E-97	1.24E-93	PGS002382	EFO_0004703	age at menarche	age at menarche	EFO_0004703	Other measurement	Genitourinary	PGS002431	EFO_0004703	age at menarche	age at menarche	EFO_0004703	Other measurement	Genitourinary
0.7854	7.57E-97	1.25E-93	PGS001229	EFO_0004339	body height	body height	EFO_0004339	Body measurement	Body measurement	PGS000969	EFO_0011011	sitting height measurement	sitting height measurement	EFO_0011011	Other measurement	Skeletal
0.7854	7.98E-97	1.31E-93	PGS002605	EFO_0004611	low density lipoprotein cholesterol measurement	low density lipoprotein cholesterol measurement	EFO_0004611	Lipid or lipoprotein measurement	Metabolic	PGS000824	EFO_0004611	low density lipoprotein cholesterol measurement	measurement	EFO_0004611	Lipid or lipoprotein measurement	Metabolic
0.7854	7.98E-97	1.31E-93	PGS002605	EFO_0004611	low density lipoprotein cholesterol measurement	low density lipoprotein cholesterol measurement	EFO_0004611	Cardiovascular measurement	Cardiovascular	PGS000824	EFO_0004611	low density lipoprotein cholesterol measurement	measurement	EFO_0004611	Cardiovascular measurement	Cardiovascular
0.7854	7.98E-97	1.31E-93	PGS002605	EFO_0004611	low density lipoprotein cholesterol measurement	low density lipoprotein cholesterol measurement	EFO_0004611	Cardiovascular measurement	Cardiovascular	PGS000824	EFO_0004611	low density lipoprotein cholesterol measurement	measurement	EFO_0004611	Lipid or lipoprotein measurement	Metabolic
0.7854	7.98E-97	1.31E-93	PGS002605	EFO_0004611	low density lipoprotein cholesterol measurement	low density lipoprotein cholesterol measurement	EFO_0004611	Lipid or lipoprotein measurement	Metabolic	PGS000824	EFO_0004611	low density lipoprotein cholesterol measurement	measurement	EFO_0004611	Cardiovascular measurement	Cardiovascular
0.7854	8.00E-97	1.32E-93	PGS002082	MONDO_0004670	lupus erythematosus	lupus erythematosus	MONDO_0004670	Immune system disorder	Immunological	PGS001870	MONDO_0004670	lupus erythematosus	lupus erythematosus	MONDO_0004670	Immune system disorder	Immunological
0.7853	8.29E-97	1.36E-93	PGS002340	EFO_0004584	mean platelet volume	mean platelet volume	EFO_0004584	Hematological measurement	Hematological	PGS000104	EFO_0004584	mean platelet volume	mean platelet volume	EFO_0004584	Hematological measurement	Hematological
0.7853	8.37E-97	1.38E-93	PGS003149	EFO_0004530	triglyceride measurement	triglyceride measurement	EFO_0004530	Lipid or lipoprotein measurement	Metabolic	PGS002197	EFO_0004530	triglyceride measurement	triglyceride measurement	EFO_0004530	Lipid or lipoprotein measurement	Metabolic
0.7853	8.77E-97	1.44E-93	PGS003295	EFO_0004617	cystatin C measurement|glomerular filtration rate	cystatin C measurement	EFO_0004617	Other measurement	Metabolic	PGS003298	EFO_0004617	cystatin C measurement|glomerular filtration rate	cystatin C measurement	EFO_0004617	Other measurement	Metabolic
0.7852	9.37E-97	1.54E-93	PGS000213	EFO_0009780	HER2 negative breast carcinoma	HER2 negative breast carcinoma	EFO_0009780	Cancer	Neoplasms	PGS000004	EFO_0000305	breast carcinoma	breast carcinoma	EFO_0000305	Cancer	Neoplasms
0.7852	9.62E-97	1.58E-93	PGS002337	EFO_0004611	low density lipoprotein cholesterol measurement	low density lipoprotein cholesterol measurement	EFO_0004611	Lipid or lipoprotein measurement	Metabolic	PGS000677	EFO_0004574	total cholesterol measurement	total cholesterol measurement	EFO_0004574	Lipid or lipoprotein measurement	Metabolic
0.7852	9.62E-97	1.58E-93	PGS002337	EFO_0004611	low density lipoprotein cholesterol measurement	low density lipoprotein cholesterol measurement	EFO_0004611	Cardiovascular measurement	Cardiovascular	PGS000677	EFO_0004574	total cholesterol measurement	total cholesterol measurement	EFO_0004574	Lipid or lipoprotein measurement	Metabolic
0.7852	9.65E-97	1.58E-93	PGS001655	EFO_0006930	brain volume measurement|neuroimaging measurement	brain volume measurement	EFO_0006930	Other measurement	Neurological	PGS001654	EFO_0006930	measurement	brain volume measurement	EFO_0006930	Other measurement	Neurological
0.7851	1.01E-96	1.65E-93	PGS002623	EFO_0004631	vitamin D measurement	vitamin D measurement	EFO_0004631	Lipid or lipoprotein measurement	Metabolic	PGS003184	EFO_0004631	vitamin D measurement	vitamin D measurement	EFO_0004631	Lipid or lipoprotein measurement	Metabolic
0.7851	1.01E-96	1.66E-93	PGS002173	EFO_0009270	heel bone mineral density	heel bone mineral density	EFO_0009270	Other measurement	Skeletal	PGS000951	EFO_0004514	bone quantitative ultrasound measurement	measurement	EFO_0004514	Other measurement	Skeletal
0.785	1.10E-96	1.80E-93	PGS000913	EFO_0006335	systolic blood pressure	systolic blood pressure	EFO_0006335	Other measurement	Cardiovascular	PGS000901	EFO_0006335	systolic blood pressure|male	systolic blood pressure	EFO_0006335	Other measurement	Cardiovascular
0.785	1.16E-96	1.89E-93	PGS003295	EFO_0004617	cystatin C measurement|glomerular filtration rate	cystatin C measurement	EFO_0004617	Other measurement	Metabolic	PGS003311	EFO_0004617	cystatin C measurement|glomerular filtration rate	cystatin C measurement	EFO_0004617	Other measurement	Metabolic
0.785	1.19E-96	1.95E-93	PGS003111	MONDO_0005148	type 2 diabetes mellitus	type 2 diabetes mellitus	MONDO_0005148	Metabolic disorder	Metabolic	PGS003096	MONDO_0005148	type 2 diabetes mellitus	type 2 diabetes mellitus	MONDO_0005148	Digestive system disorder	Gastrointestinal

0.785	1.19E-96	1.95E-93	PGS003111	MONDO_0005148	type 2 diabetes mellitus	type 2 diabetes mellitus	MONDO_0005148	Digestive system disorder	Gastrointestinal	PGS003096	MONDO_0005148	type 2 diabetes mellitus	type 2 diabetes mellitus	MONDO_0005148	Metabolic disorder	Metabolic
0.785	1.19E-96	1.95E-93	PGS003111	MONDO_0005148	type 2 diabetes mellitus	type 2 diabetes mellitus	MONDO_0005148	Digestive system disorder	Gastrointestinal	PGS003096	MONDO_0005148	type 2 diabetes mellitus	type 2 diabetes mellitus	MONDO_0005148	Digestive system disorder	Gastrointestinal
0.785	1.19E-96	1.95E-93	PGS003111	MONDO_0005148	type 2 diabetes mellitus	type 2 diabetes mellitus	MONDO_0005148	Metabolic disorder	Metabolic	PGS003096	MONDO_0005148	type 2 diabetes mellitus	type 2 diabetes mellitus	MONDO_0005148	Metabolic disorder	Metabolic
0.7849	1.27E-96	2.08E-93	PGS001950	EFO_0004995	lean body mass	lean body mass	EFO_0004995	Other measurement	Skeletal	PGS001002	EFO_0009805	whole body water mass	whole body water mass	EFO_0009805	Other measurement	Body measurement
0.7848	1.33E-96	2.17E-93	PGS000795	EFO_0001663	prostate carcinoma	prostate carcinoma	EFO_0001663	Cancer	Neoplasms	PGS000049	EFO_0001663	prostate carcinoma	prostate carcinoma	EFO_0001663	Cancer	Neoplasms
0.7848	1.35E-96	2.20E-93	PGS000540	EFO_0000305	breast carcinoma	breast carcinoma	EFO_0000305	Cancer	Neoplasms	PGS000512	EFO_0000305	breast carcinoma	breast carcinoma	EFO_0000305	Cancer	Neoplasms
0.7848	1.38E-96	2.26E-93	PGS003194	EFO_0004631	vitamin D measurement	vitamin D measurement	EFO_0004631	Lipid or lipoprotein measurement	Metabolic	PGS003198	EFO_0004631	vitamin D measurement	vitamin D measurement	EFO_0004631	Lipid or lipoprotein measurement	Metabolic
0.7848	1.40E-96	2.29E-93	PGS000051	EFO_0000305	breast carcinoma	breast carcinoma	EFO_0000305	Cancer	Neoplasms	PGS003398	EFO_0000305	breast carcinoma	breast carcinoma	EFO_0000305	Cancer	Neoplasms
0.7848	1.41E-96	2.30E-93	PGS000499	EFO_0000305	breast carcinoma	breast carcinoma	EFO_0000305	Cancer	Neoplasms	PGS000527	EFO_0000305	breast carcinoma	breast carcinoma	EFO_0000305	Cancer	Neoplasms
0.7848	1.41E-96	2.30E-93	PGS000534	EFO_0000305	breast carcinoma	breast carcinoma	EFO_0000305	Cancer	Neoplasms	PGS000537	EFO_0000305	breast carcinoma	breast carcinoma	EFO_0000305	Cancer	Neoplasms
0.7848	1.41E-96	2.30E-93	PGS000533	EFO_0000305	breast carcinoma	breast carcinoma	EFO_0000305	Cancer	Neoplasms	PGS000537	EFO_0000305	breast carcinoma	breast carcinoma	EFO_0000305	Cancer	Neoplasms
0.7847	1.50E-96	2.44E-93	PGS001744	EFO_0006930	brain volume measurement|neuroimaging measurement	brain volume measurement	EFO_0006930	Other measurement	Neurological	PGS001714	EFO_0006930	measurement	brain volume measurement	EFO_0006930	Other measurement	Neurological
0.7847	1.52E-96	2.49E-93	PGS000508	EFO_0000305	breast carcinoma	breast carcinoma	EFO_0000305	Cancer	Neoplasms	PGS000535	EFO_0000305	breast carcinoma	breast carcinoma	EFO_0000305	Cancer	Neoplasms
0.7847	1.53E-96	2.49E-93	PGS003248	EFO_0010472	creatine measurement	creatine measurement	EFO_0010472	Other measurement	Metabolic	PGS003377	EFO_0005208	glomerular filtration rate	glomerular filtration rate	EFO_0005208	Other measurement	Genitourinary
0.7845	1.89E-96	3.08E-93	PGS003380	EFO_0000305	breast carcinoma	breast carcinoma	EFO_0000305	Cancer	Neoplasms	PGS000512	EFO_0000305	breast carcinoma	breast carcinoma	EFO_0000305	Cancer	Neoplasms
0.7844	1.95E-96	3.17E-93	PGS000672	EFO_0004615	apolipoprotein B measurement	apolipoprotein B measurement	EFO_0004615	Lipid or lipoprotein measurement	Metabolic	PGS002654	EFO_0004611	low density lipoprotein cholesterol measurement	measurement	EFO_0004611	Cardiovascular measurement	Cardiovascular
0.7844	1.95E-96	3.17E-93	PGS000672	EFO_0004615	apolipoprotein B measurement	apolipoprotein B measurement	EFO_0004615	Lipid or lipoprotein measurement	Metabolic	PGS002654	EFO_0004611	low density lipoprotein cholesterol measurement	measurement	EFO_0004611	Lipid or lipoprotein measurement	Metabolic
0.7844	1.98E-96	3.23E-93	PGS001240	EFO_0004305	erythrocyte count	erythrocyte count	EFO_0004305	Hematological measurement	Hematological	PGS001909	EFO_0004305	erythrocyte count	erythrocyte count	EFO_0004305	Hematological measurement	Hematological
0.7844	2.03E-96	3.30E-93	PGS000508	EFO_0000305	breast carcinoma	breast carcinoma	EFO_0000305	Cancer	Neoplasms	PGS000504	EFO_0000305	breast carcinoma	breast carcinoma	EFO_0000305	Cancer	Neoplasms
0.7844	2.05E-96	3.34E-93	PGS000504	EFO_0000305	breast carcinoma	breast carcinoma	EFO_0000305	Cancer	Neoplasms	PGS000335	EFO_0000305	breast carcinoma	breast carcinoma	EFO_0000305	Cancer	Neoplasms
0.7843	2.25E-96	3.67E-93	PGS003124	EFO_0004340	body mass index	body mass index	EFO_0004340	Body measurement	Body measurement	PGS003129	EFO_0004340	body mass index	body mass index	EFO_0004340	Body measurement	Body measurement
0.7842	2.29E-96	3.72E-93	PGS001989	EFO_0004527	mean corpuscular hemoglobin	mean corpuscular hemoglobin	EFO_0004527	Hematological measurement	Hematological	PGS000101	EFO_0004526	mean corpuscular volume	mean corpuscular volume	EFO_0004526	Hematological measurement	Hematological
0.7842	2.47E-96	4.03E-93	PGS002190	EFO_0007984	platelet component distribution width	platelet component distribution width	EFO_0007984	Hematological measurement	Hematological	PGS000108	EFO_0007984	platelet component distribution width	platelet component distribution width	EFO_0007984	Hematological measurement	Hematological
0.7841	2.60E-96	4.24E-93	PGS001109	EFO_0007533	vasomotor rhinitis|allergic rhinitis	vasomotor rhinitis	EFO_0007533	Immune system disorder	Immunological	PGS001259	EFO_0003956	seasonal allergic rhinitis	seasonal allergic rhinitis	EFO_0003956	Immune system disorder	Immunological
0.784	2.78E-96	4.52E-93	PGS000899	EFO_0001645	coronary artery disease	coronary artery disease	EFO_0001645	Cardiovascular disease	Cardiovascular	PGS000798	EFO_0001645	coronary artery disease	coronary artery disease	EFO_0001645	Cardiovascular disease	Cardiovascular
0.784	2.90E-96	4.72E-93	PGS003189	EFO_0004631	vitamin D measurement	vitamin D measurement	EFO_0004631	Lipid or lipoprotein measurement	Metabolic	PGS003199	EFO_0004631	vitamin D measurement	vitamin D measurement	EFO_0004631	Lipid or lipoprotein measurement	Metabolic
0.7839	3.08E-96	5.00E-93	PGS000001	EFO_0000305	breast carcinoma	breast carcinoma	EFO_0000305	Cancer	Neoplasms	PGS003398	EFO_0000305	breast carcinoma	breast carcinoma	EFO_0000305	Cancer	Neoplasms
0.7839	3.11E-96	5.06E-93	PGS001336	EFO_0000305	breast carcinoma	breast carcinoma	EFO_0000305	Cancer	Neoplasms	PGS001804	EFO_0000305	breast carcinoma	breast carcinoma	EFO_0000305	Cancer	Neoplasms
0.7839	3.13E-96	5.08E-93	PGS000516	EFO_0000305	breast carcinoma	breast carcinoma	EFO_0000305	Cancer	Neoplasms	PGS001804	EFO_0000305	breast carcinoma	breast carcinoma	EFO_0000305	Cancer	Neoplasms
0.7838	3.51E-96	5.69E-93	PGS002009	EFO_0006335	systolic blood pressure	systolic blood pressure	EFO_0006335	Other measurement	Cardiovascular	PGS000913	EFO_0006335	systolic blood pressure	systolic blood pressure	EFO_0006335	Other measurement	Cardiovascular
0.7838	3.61E-96	5.86E-93	PGS002672	EFO_0004631	vitamin D measurement	vitamin D measurement	EFO_0004631	Lipid or lipoprotein measurement	Metabolic	PGS003196	EFO_0004631	vitamin D measurement	vitamin D measurement	EFO_0004631	Lipid or lipoprotein measurement	Metabolic
0.7837	3.92E-96	6.35E-93	PGS001161	EFO_0005106	body composition measurement	body composition measurement	EFO_0005106	Body measurement	Body measurement	PGS002179	EFO_0005106	body composition measurement	body composition measurement	EFO_0005106	Body measurement	Body measurement
0.7836	4.20E-96	6.82E-93	PGS003189	EFO_0004631	vitamin D measurement	vitamin D measurement	EFO_0004631	Lipid or lipoprotein measurement	Metabolic	PGS003184	EFO_0004631	vitamin D measurement	vitamin D measurement	EFO_0004631	Lipid or lipoprotein measurement	Metabolic
0.7835	4.52E-96	7.33E-93	PGS002501	EFO_0004541	HbA1c measurement	HbA1c measurement	EFO_0004541	Hematological measurement	Hematological	PGS002491	EFO_0000400	diabetes mellitus	diabetes mellitus	EFO_0000400	Metabolic disorder	Metabolic
0.7835	4.52E-96	7.33E-93	PGS002501	EFO_0004541	HbA1c measurement	HbA1c measurement	EFO_0004541	Hematological measurement	Hematological	PGS002491	EFO_0000400	diabetes mellitus	diabetes mellitus	EFO_0000400	Digestive system disorder	Gastrointestinal
0.7835	4.57E-96	7.41E-93	PGS002009	EFO_0006335	systolic blood pressure	systolic blood pressure	EFO_0006335	Other measurement	Cardiovascular	PGS003073	EFO_0006335	systolic blood pressure	systolic blood pressure	EFO_0006335	Other measurement	Cardiovascular
0.7834	4.88E-96	7.91E-93	PGS003288	EFO_0004617	cystatin C measurement	cystatin C measurement	EFO_0004617	Other measurement	Metabolic	PGS003284	EFO_0004617	cystatin C measurement	cystatin C measurement	EFO_0004617	Other measurement	Metabolic
0.7833	5.44E-96	8.82E-93	PGS002030	EFO_0004274	gout	gout	EFO_0004274	Metabolic disorder	Metabolic	PGS003329	EFO_0004274	gout	gout	EFO_0004274	Metabolic disorder	Metabolic
0.7833	5.73E-96	9.28E-93	PGS002993	EFO_0004339	body height	body height	EFO_0004339	Body measurement	Body measurement	PGS002990	EFO_0004339	body height	body height	EFO_0004339	Body measurement	Body measurement
0.7832	5.76E-96	9.32E-93	PGS000044	EFO_0001663	prostate carcinoma	prostate carcinoma	EFO_0001663	Cancer	Neoplasms	PGS000719	EFO_0001663	prostate carcinoma	prostate carcinoma	EFO_0001663	Cancer	Neoplasms
0.7832	5.87E-96	9.49E-93	PGS001979	EFO_0004530	triglyceride measurement	triglyceride measurement	EFO_0004530	Lipid or lipoprotein measurement	Metabolic	PGS003149	EFO_0004530	triglyceride measurement	triglyceride measurement	EFO_0004530	Lipid or lipoprotein measurement	Metabolic
0.7832	6.07E-96	9.82E-93	PGS002458	EFO_0004611	low density lipoprotein cholesterol measurement	low density lipoprotein cholesterol measurement	EFO_0004611	Cardiovascular measurement	Cardiovascular	PGS002556	EFO_0004611	low density lipoprotein cholesterol measurement	measurement	EFO_0004611	Lipid or lipoprotein measurement	Metabolic
0.7832	6.07E-96	9.82E-93	PGS002458	EFO_0004611	low density lipoprotein cholesterol measurement	low density lipoprotein cholesterol measurement	EFO_0004611	Lipid or lipoprotein measurement	Metabolic	PGS002556	EFO_0004611	low density lipoprotein cholesterol measurement	measurement	EFO_0004611	Cardiovascular measurement	Cardiovascular
0.7832	6.07E-96	9.82E-93	PGS002458	EFO_0004611	low density lipoprotein cholesterol measurement	low density lipoprotein cholesterol measurement	EFO_0004611	Cardiovascular measurement	Cardiovascular	PGS002556	EFO_0004611	low density lipoprotein cholesterol measurement	measurement	EFO_0004611	Cardiovascular measurement	Cardiovascular
0.7832	6.07E-96	9.82E-93	PGS002458	EFO_0004611	low density lipoprotein cholesterol measurement	low density lipoprotein cholesterol measurement	EFO_0004611	Lipid or lipoprotein measurement	Metabolic	PGS002556	EFO_0004611	low density lipoprotein cholesterol measurement	measurement	EFO_0004611	Lipid or lipoprotein measurement	Metabolic
0.7832	6.14E-96	9.93E-93	PGS002353	EFO_0004530	triglyceride measurement	triglyceride measurement	EFO_0004530	Lipid or lipoprotein measurement	Metabolic	PGS003149	EFO_0004530	triglyceride measurement	triglyceride measurement	EFO_0004530	Lipid or lipoprotein measurement	Metabolic
0.7832	6.15E-96	9.95E-93	PGS001406	EFO_0010700	reticulocyte measurement	reticulocyte measurement	EFO_0010700	Hematological measurement	Hematological	PGS002333	EFO_0007986	reticulocyte count	reticulocyte count	EFO_0007986	Hematological measurement	Hematological
0.7832	6.20E-96	1.00E-92	PGS000940	HP_0002024	Malabsorption	Malabsorption	HP_0002024	Other trait	Gastrointestinal	PGS001856	EFO_0001060	celiac disease	celiac disease	EFO_0001060	Immune system disorder	Immunological
0.7832	6.20E-96	1.00E-92	PGS000940	HP_0002024	Malabsorption	Malabsorption	HP_0002024	Other trait	Gastrointestinal	PGS001856	EFO_0001060	celiac disease	celiac disease	EFO_0001060	Digestive system disorder	Gastrointestinal
0.7832	6.29E-96	1.02E-92	PGS002752	EFO_0007878	alcohol consumption measurement	alcohol consumption measurement	EFO_0007878	Other measurement	Psychiatric	PGS002830	EFO_0007878	alcohol consumption measurement	alcohol consumption measurement	EFO_0007878	Other measurement	Psychiatric
0.7831	6.45E-96	1.04E-92	PGS002782	EFO_0005689	non-high density lipoprotein cholesterol measurement	non-high density lipoprotein cholesterol measurement	EFO_0005689	Lipid or lipoprotein measurement	Metabolic	PGS003038	EFO_0004611	low density lipoprotein cholesterol measurement	measurement	EFO_0004611	Lipid or lipoprotein measurement	Metabolic
0.7831	6.45E-96	1.04E-92	PGS002782	EFO_0005689	non-high density lipoprotein cholesterol measurement	non-high density lipoprotein cholesterol measurement	EFO_0005689	Lipid or lipoprotein measurement	Metabolic	PGS003038	EFO_0004611	low density lipoprotein cholesterol measurement	measurement	EFO_0004611	Cardiovascular measurement	Cardiovascular
0.7831	6.45E-96	1.04E-92	PGS002782	EFO_0005689	non-high density lipoprotein cholesterol measurement	non-high density lipoprotein cholesterol measurement	EFO_0005689	Cardiovascular measurement	Cardiovascular	PGS003038	EFO_0004611	low density lipoprotein cholesterol measurement	measurement	EFO_0004611	Lipid or lipoprotein measurement	Metabolic
0.7831	6.45E-96	1.04E-92	PGS002782	EFO_0005689	non-high density lipoprotein cholesterol measurement	non-high density lipoprotein cholesterol measurement	EFO_0005689	Cardiovascular measurement	Cardiovascular	PGS003038	EFO_0004611	low density lipoprotein cholesterol measurement	measurement	EFO_0004611	Cardiovascular measurement	Cardiovascular
0.7831	6.57E-96	1.06E-92	PGS000989	EFO_0010700	reticulocyte measurement	reticulocyte measurement	EFO_0010700	Hematological measurement	Hematological	PGS001406	EFO_0010700	reticulocyte measurement	reticulocyte measurement	EFO_0010700	Hematological measurement	Hematological
0.783	7.16E-96	1.16E-92	PGS000069	EFO_1001516	ovarian serous carcinoma	ovarian serous carcinoma	EFO_1001516	Cancer	Neoplasms	PGS000563	EFO_0003893	ovarian neoplasm	ovarian neoplasm	EFO_0003893	Cancer	Neoplasms
0.783	7.17E-96	1.16E-92	PGS002231	EFO_0011015	educational attainment	educational attainment	EFO_0011015	Other measurement	Activities	PGS002319	EFO_0011015	educational attainment	educational attainment	EFO_0011015	Other measurement	Activities
0.7829	8.12E-96	1.31E-92	PGS001840	MONDO_0004596	cor pulmonale	cor pulmonale	MONDO_0004596	Cardiovascular disease	Cardiovascular	PGS001847	EFO_0004264	vascular disease	vascular disease	EFO_0004264	Cardiovascular disease	Cardiovascular
0.7829	8.26E-96	1.33E-92	PGS003025	EFO_0000537	hypertension	hypertension	EFO_0000537	Cardiovascular disease	Cardiovascular	PGS003004	EFO_0000537	hypertension	hypertension	EFO_0000537	Cardiovascular disease	Cardiovascular
0.7829	8.26E-96	1.33E-92	PGS003026	EFO_0000537	hypertension	hypertension	EFO_0000537	Cardiovascular disease	Cardiovascular	PGS003004	EFO_0000537	hypertension	hypertension	EFO_0000537	Cardiovascular disease	Cardiovascular
0.7828	8.36E-96	1.35E-92	PGS000008	EFO_1000649	estrogen-receptor positive breast cancer	estrogen-receptor positive breast cancer	EFO_1000649	Cancer	Neoplasms	PGS000004	EFO_0000305	breast carcinoma	breast carcinoma	EFO_0000305	Cancer	Neoplasms
0.7827	9.28E-96	1.49E-92	PGS001946	EFO_0004458	C-reactive protein measurement	C-reactive protein measurement	EFO_0004458	Inflammatory measurement	Immunological	PGS002873	EFO_0004458	C-reactive protein measurement	C-reactive protein measurement	EFO_0004458	Inflammatory measurement	Immunological
0.7827	9.43E-96	1.52E-92	PGS002009	EFO_0006335	systolic blood pressure	systolic blood pressure	EFO_0006335	Other measurement	Cardiovascular	PGS002807	EFO_0006335	systolic blood pressure	systolic blood pressure	EFO_0006335	Other measurement	Cardiovascular
0.7827	9.48E-96	1.53E-92	PGS000414	EFO_0000756	melanoma	melanoma	EFO_0000756	Cancer	Neoplasms	PGS000418	EFO_0000756	melanoma	melanoma	EFO_0000756	Cancer	Neoplasms
0.7827	9.48E-96	1.53E-92	PGS000414	EFO_0000756	melanoma	melanoma	EFO_0000756	Cancer	Neoplasms	PGS000416	EFO_0000756	melanoma	melanoma	EFO_0000756	Cancer	Neoplasms
0.7825	1.10E-95	1.77E-92	PGS002583	EFO_0003923	bone density	bone density	EFO_0003923	Other measurement	Skeletal	PGS002436	EFO_0003923	bone density	bone density	EFO_0003923	Other measurement	Skeletal
0.7825	1.11E-95	1.78E-92	PGS002983	EFO_0004339	body height	body height	EFO_0004339	Body measurement	Body measurement	PGS002968	EFO_0004339	body height	body height	EFO_0004339	Body measurement	Body measurement
0.7825	1.13E-95	1.82E-92	PGS000507	EFO_0000305	breast carcinoma	breast carcinoma	EFO_0000305	Cancer	Neoplasms	PGS000008	EFO_1000649	estrogen-receptor positive breast cancer	cancer	EFO_1000649	Cancer	Neoplasms
0.7825	1.14E-95	1.84E-92	PGS000563	EFO_0003893	ovarian neoplasm	ovarian neoplasm	EFO_0003893	Cancer	Neoplasms	PGS000068	EFO_0001075	ovarian carcinoma	ovarian carcinoma	EFO_0001075	Cancer	Neoplasms
0.7825	1.16E-95	1.86E-92	PGS003069	EFO_0006335	systolic blood pressure	systolic blood pressure	EFO_0006335	Other measurement	Cardiovascular	PGS002228	EFO_0006335	systolic blood pressure	systolic blood pressure	EFO_0006335	Other measurement	Cardiovascular
0.7824	1.23E-95	1.98E-92	PGS001248	EFO_0004274	gout	gout	EFO_0004274	Metabolic disorder	Metabolic	PGS003329	EFO_0004274	gout	gout	EFO_0004274	Metabolic disorder	Metabolic
0.7824	1.29E-95	2.08E-92	PGS003101	MONDO_0005148	type 2 diabetes mellitus	type 2 diabetes mellitus	MONDO_0005148	Metabolic disorder	Metabolic	PGS003099	MONDO_0005148	type 2 diabetes mellitus	type 2 diabetes mellitus	MONDO_0005148	Metabolic disorder	Metabolic
0.7824	1.29E-95	2.08E-92	PGS003099	MONDO_0005148	type 2 diabetes mellitus	type 2 diabetes mellitus	MONDO_0005148	Digestive system disorder	Gastrointestinal	PGS003100	MONDO_0005148	type 2 diabetes mellitus	type 2 diabetes mellitus	MONDO_0005148	Metabolic disorder	Metabolic
0.7824	1.29E-95	2.08E-92	PGS003099	MONDO_0005148	type 2 diabetes mellitus	type 2 diabetes mellitus	MONDO_0005148	Metabolic disorder	Metabolic	PGS003100	MONDO_0005148	type 2 diabetes mellitus	type 2 diabetes mellitus	MONDO_0005148	Digestive system disorder	Gastrointestinal
0.7824	1.29E-95	2.08E-92	PGS003099	MONDO_0005148	type 2 diabetes mellitus	type 2 diabetes mellitus	MONDO_0005148	Metabolic disorder	Metabolic	PGS003100	MONDO_0005148	type 2 diabetes mellitus	type 2 diabetes mellitus	MONDO_0005148	Metabolic disorder	Metabolic
0.7824	1.29E-95	2.08E-92	PGS003101	MONDO_0005148	type 2 diabetes mellitus	type 2 diabetes mellitus	MONDO_0005148	Digestive system disorder	Gastrointestinal	PGS003099	MONDO_0005148	type 2 diabetes mellitus	type 2 diabetes mellitus	MONDO_0005148	Digestive system disorder	Gastrointestinal
0.7824	1.29E-95	2.08E-92	PGS003101	MONDO_0005148	type 2 diabetes mellitus	type 2 diabetes mellitus	MONDO_0005148	Metabolic disorder	Metabolic	PGS003099	MONDO_0005148	type 2 diabetes mellitus	type 2 diabetes mellitus	MONDO_0005148	Digestive system disorder	Gastrointestinal
0.7824	1.29E-95	2.08E-92	PGS003101	MONDO_0005148	type 2 diabetes mellitus	type 2 diabetes mellitus	MONDO_0005148	Digestive system disorder	Gastrointestinal	PGS003099	MONDO_0005148	type 2 diabetes mellitus	type 2 diabetes mellitus	MONDO_0005148	Metabolic disorder	Metabolic
0.7824	1.29E-95	2.08E-92	PGS003099	MONDO_0005148	type 2 diabetes mellitus	type 2 diabetes mellitus	MONDO_0005148	Digestive system disorder	Gastrointestinal	PGS003100	MONDO_0005148	type 2 diabetes mellitus	type 2 diabetes mellitus	MONDO_0005148	Digestive system disorder	Gastrointestinal
0.7823	1.34E-95	2.15E-92	PGS002005	EFO_0011011	sitting height measurement	sitting height measurement	EFO_0011011	Other measurement	Skeletal	PGS002332	EFO_0004339	body height	body height	EFO_0004339	Body measurement	Body measurement
0.7823	1.43E-95	2.29E-92	PGS002009	EFO_0006335	systolic blood pressure	systolic blood pressure	EFO_0006335	Other measurement	Cardiovascular	PGS003078	EFO_0006335	systolic blood pressure	systolic blood pressure	EFO_0006335	Other measurement	Cardiovascular
0.7822	1.48E-95	2.37E-92	PGS001984	EFO_0009805	whole body water mass	whole body water mass	EFO_0009805	Other measurement	Body measurement	PGS001168	EFO_0004995	lean body mass	lean body mass	EFO_0004995	Other measurement	Skeletal
0.7822	1.55E-95	2.48E-92	PGS001975	EFO_0004326	heart rate	heart rate	EFO_0004326	Cardiovascular measurement	Cardiovascular	PGS001233	EFO_0004326	heart rate	heart rate	EFO_0004326	Cardiovascular measurement	Cardiovascular
0.7821	1.69E-95	2.71E-92	PGS002851	EFO_0004340	body mass index	body mass index	EFO_0004340	Body measurement	Body measurement	PGS002847	EFO_0004340	body mass index	body mass index	EFO_0004340	Body measurement	Body measurement
0.7821	1.70E-95	2.72E-92	PGS001451	EFO_0006930	brain volume measurement|neuroimaging measurement	brain volume measurement	EFO_0006930	Other measurement	Neurological	PGS001683	EFO_0006930	measurement	brain volume measurement	EFO_0006930	Other measurement	Neurological
0.7821	1.70E-95	2.72E-92	PGS001480	EFO_0006930	brain volume measurement|neuroimaging measurement	brain volume measurement	EFO_0006930	Other measurement	Neurological	PGS001672	EFO_0006930	measurement	brain volume measurement	EFO_0006930	Other measurement	Neurological
0.782	1.78E-95	2.85E-92	PGS001595	EFO_0005420	measurement	grey matter volume measurement	EFO_0005420	Other measurement	Neurological	PGS001596	EFO_0005420	measurement	grey matter volume measurement	EFO_0005420	Other measurement	Neurological
-0.782	1.79E-95	2.86E-92	PGS003224	EFO_0005208	glomerular filtration rate	glomerular filtration rate	EFO_0005208	Other measurement	Genitourinary	PGS002237	EFO_0003884	chronic kidney disease	chronic kidney disease	EFO_0003884	Other disease	Metabolic
0.782	1.88E-95	3.01E-92	PGS001145	EFO_0005409	fat body mass	fat body mass	EFO_0005409	Other measurement	Body measurement	PGS001101	EFO_0007800	body fat percentage	body fat percentage	EFO_0007800	Body measurement	Body measurement
0.7819	2.06E-95	3.30E-92	PGS002660	EFO_0004309	platelet count	platelet count	EFO_0004309	Hematological measurement	Hematological	PGS001238	EFO_0004309	platelet count	platelet count	EFO_0004309	Hematological measurement	Hematological
0.7819	2.07E-95	3.30E-92	PGS000528	EFO_0000305	breast carcinoma	breast carcinoma	EFO_0000305	Cancer	Neoplasms	PGS000531	EFO_0000305	breast carcinoma	breast carcinoma	EFO_0000305	Cancer	Neoplasms
0.7818	2.09E-95	3.33E-92	PGS002934	EFO_0004468	glucose measurement	glucose measurement	EFO_0004468	Other measurement	Metabolic	PGS002328	EFO_0004468	glucose measurement	glucose measurement	EFO_0004468	Other measurement	Metabolic
0.7817	2.44E-95	3.90E-92	PGS002993	EFO_0004339	body height	body height	EFO_0004339	Body measurement	Body measurement	PGS002968	EFO_0004339	body height	body height	EFO_0004339	Body measurement	Body measurement
0.7815	2.99E-95	4.78E-92	PGS002776	EFO_0001645	coronary artery disease	coronary artery disease	EFO_0001645	Cardiovascular disease	Cardiovascular	PGS001355	EFO_0001645	coronary artery disease	coronary artery disease	EFO_0001645	Cardiovascular disease	Cardiovascular
0.7814	3.03E-95	4.84E-92	PGS001943	EFO_0004340	body mass index	body mass index	EFO_0004340	Body measurement	Body measurement	PGS001958	EFO_0005093	hip circumference	hip circumference	EFO_0005093	Body measurement	Body measurement
0.7814	3.05E-95	4.87E-92	PGS001895	EFO_0004574	total cholesterol measurement	total cholesterol measurement	EFO_0004574	Lipid or lipoprotein measurement	Metabolic	PGS003138	EFO_0004574	total cholesterol measurement	total cholesterol measurement	EFO_0004574	Lipid or lipoprotein measurement	Metabolic
0.7813	3.54E-95	5.64E-92	PGS000214	MONDO_0021115	luminal B breast carcinoma	luminal B breast carcinoma	MONDO_0021115	Cancer	Neoplasms	PGS000005	EFO_1000649	estrogen-receptor positive breast cancer	cancer	EFO_1000649	Cancer	Neoplasms
0.7813	3.59E-95	5.72E-92	PGS001509	EFO_0004346	neuroimaging measurement	neuroimaging measurement	EFO_0004346	Other measurement	Neurological	PGS001510	EFO_0004346	neuroimaging measurement	neuroimaging measurement	EFO_0004346	Other measurement	Neurological
0.7812	3.70E-95	5.90E-92	PGS000437	MONDO_0002898	skin cancer	skin cancer	MONDO_0002898	Cancer	Neoplasms	PGS000400	EFO_0009259	skin carcinoma	skin carcinoma	EFO_0009259	Cancer	Neoplasms
0.7812	3.79E-95	6.03E-92	PGS001991	EFO_0007989	monocyte percentage of leukocytes	monocyte percentage of leukocytes	EFO_0007989	Hematological measurement	Hematological	PGS000103	EFO_0007989	monocyte percentage of leukocytes	monocyte percentage of leukocytes	EFO_0007989	Hematological measurement	Hematological
0.7812	3.85E-95	6.13E-92	PGS003114	MONDO_0005148	type 2 diabetes mellitus	type 2 diabetes mellitus	MONDO_0005148	Metabolic disorder	Metabolic	PGS003115	MONDO_0005148	type 2 diabetes mellitus	type 2 diabetes mellitus	MONDO_0005148	Digestive system disorder	Gastrointestinal
0.7812	3.85E-95	6.13E-92	PGS003116	MONDO_0005148	type 2 diabetes mellitus	type 2 diabetes mellitus	MONDO_0005148	Metabolic disorder	Metabolic	PGS003114	MONDO_0005148	type 2 diabetes mellitus	type 2 diabetes mellitus	MONDO_0005148	Metabolic disorder	Metabolic
0.7812	3.85E-95	6.13E-92	PGS003114	MONDO_0005148	type 2 diabetes mellitus	type 2 diabetes mellitus	MONDO_0005148	Digestive system disorder	Gastrointestinal	PGS003115	MONDO_0005148	type 2 diabetes mellitus	type 2 diabetes mellitus	MONDO_0005148	Digestive system disorder	Gastrointestinal

0.7812	3.85E-95	6.13E-92	PGS003114	MONDO_0005148	type 2 diabetes mellitus	type 2 diabetes mellitus	MONDO_0005148	Digestive system disorder	Gastrointestinal	PGS003115	MONDO_0005148	type 2 diabetes mellitus	type 2 diabetes mellitus	MONDO_0005148	Metabolic disorder	Metabolic
0.7812	3.85E-95	6.13E-92	PGS003114	MONDO_0005148	type 2 diabetes mellitus	type 2 diabetes mellitus	MONDO_0005148	Metabolic disorder	Metabolic	PGS003115	MONDO_0005148	type 2 diabetes mellitus	type 2 diabetes mellitus	MONDO_0005148	Metabolic disorder	Metabolic
0.7812	3.85E-95	6.13E-92	PGS003116	MONDO_0005148	type 2 diabetes mellitus	type 2 diabetes mellitus	MONDO_0005148	Digestive system disorder	Gastrointestinal	PGS003114	MONDO_0005148	type 2 diabetes mellitus	type 2 diabetes mellitus	MONDO_0005148	Digestive system disorder	Gastrointestinal
0.7812	3.85E-95	6.13E-92	PGS003116	MONDO_0005148	type 2 diabetes mellitus	type 2 diabetes mellitus	MONDO_0005148	Digestive system disorder	Gastrointestinal	PGS003114	MONDO_0005148	type 2 diabetes mellitus	type 2 diabetes mellitus	MONDO_0005148	Metabolic disorder	Metabolic
0.7812	3.85E-95	6.13E-92	PGS003116	MONDO_0005148	type 2 diabetes mellitus	type 2 diabetes mellitus	MONDO_0005148	Metabolic disorder	Metabolic	PGS003114	MONDO_0005148	type 2 diabetes mellitus	type 2 diabetes mellitus	MONDO_0005148	Digestive system disorder	Gastrointestinal
0.781	4.45E-95	7.09E-92	PGS002840	EFO_0004340	body mass index	body mass index	EFO_0004340	Body measurement	Body measurement	PGS001943	EFO_0004340	body mass index	body mass index	EFO_0004340	Body measurement	Body measurement
0.781	4.71E-95	7.50E-92	PGS001908	EFO_0005192	red blood cell distribution width	red blood cell distribution width	EFO_0005192	Hematological measurement	Hematological	PGS002663	EFO_0005192	red blood cell distribution width	red blood cell distribution width	EFO_0005192	Hematological measurement	Hematological
0.7809	4.87E-95	7.75E-92	PGS000537	EFO_0000305	breast carcinoma	breast carcinoma	EFO_0000305	Cancer	Neoplasms	PGS000530	EFO_0000305	breast carcinoma	breast carcinoma	EFO_0000305	Cancer	Neoplasms
0.7808	5.21E-95	8.29E-92	PGS002556	EFO_0004611	low density lipoprotein cholesterol measurement	low density lipoprotein cholesterol measurement	EFO_0004611	Lipid or lipoprotein measurement	Metabolic	PGS000824	EFO_0004611	low density lipoprotein cholesterol measurement	measurement	EFO_0004611	Lipid or lipoprotein measurement	Metabolic
0.7808	5.21E-95	8.29E-92	PGS002556	EFO_0004611	low density lipoprotein cholesterol measurement	low density lipoprotein cholesterol measurement	EFO_0004611	Cardiovascular measurement	Cardiovascular	PGS000824	EFO_0004611	low density lipoprotein cholesterol measurement	measurement	EFO_0004611	Cardiovascular measurement	Cardiovascular
0.7808	5.21E-95	8.29E-92	PGS002556	EFO_0004611	low density lipoprotein cholesterol measurement	low density lipoprotein cholesterol measurement	EFO_0004611	Lipid or lipoprotein measurement	Metabolic	PGS000824	EFO_0004611	low density lipoprotein cholesterol measurement	measurement	EFO_0004611	Cardiovascular measurement	Cardiovascular
0.7808	5.21E-95	8.29E-92	PGS002556	EFO_0004611	low density lipoprotein cholesterol measurement	low density lipoprotein cholesterol measurement	EFO_0004611	Cardiovascular measurement	Cardiovascular	PGS000824	EFO_0004611	low density lipoprotein cholesterol measurement	measurement	EFO_0004611	Lipid or lipoprotein measurement	Metabolic
0.7808	5.26E-95	8.37E-92	PGS003163	EFO_0004620	vitamin B12 measurement	vitamin B12 measurement	EFO_0004620	Other measurement	Metabolic	PGS003158	EFO_0004620	vitamin B12 measurement	vitamin B12 measurement	EFO_0004620	Other measurement	Metabolic
0.7808	5.40E-95	8.59E-92	PGS001950	EFO_0004995	lean body mass	lean body mass	EFO_0004995	Other measurement	Skeletal	PGS001168	EFO_0004995	lean body mass	lean body mass	EFO_0004995	Other measurement	Skeletal
0.7808	5.66E-95	8.99E-92	PGS000406	EFO_0009259	skin carcinoma	skin carcinoma	EFO_0009259	Cancer	Neoplasms	PGS000443	MONDO_0002898	skin cancer	skin cancer	MONDO_0002898	Cancer	Neoplasms
0.7806	6.58E-95	1.04E-91	PGS001484	EFO_0006930	brain volume measurement|neuroimaging measurement	brain volume measurement	EFO_0006930	Other measurement	Neurological	PGS001472	EFO_0006930	measurement	brain volume measurement	EFO_0006930	Other measurement	Neurological
0.7805	6.95E-95	1.10E-91	PGS002543	EFO_0001379	endocrine system disease|diabetes mellitus	endocrine system disease	EFO_0001379	Other disease	Metabolic	PGS002524	MONDO_0005148	type 2 diabetes mellitus	type 2 diabetes mellitus	MONDO_0005148	Digestive system disorder	Gastrointestinal
0.7805	6.95E-95	1.10E-91	PGS002543	EFO_0001379	endocrine system disease|diabetes mellitus	endocrine system disease	EFO_0001379	Other disease	Metabolic	PGS002524	MONDO_0005148	type 2 diabetes mellitus	type 2 diabetes mellitus	MONDO_0005148	Metabolic disorder	Metabolic
0.7805	7.21E-95	1.14E-91	PGS002550	EFO_0004541	HbA1c measurement	HbA1c measurement	EFO_0004541	Hematological measurement	Hematological	PGS002573	MONDO_0005148	type 2 diabetes mellitus	type 2 diabetes mellitus	MONDO_0005148	Digestive system disorder	Gastrointestinal
0.7805	7.21E-95	1.14E-91	PGS002550	EFO_0004541	HbA1c measurement	HbA1c measurement	EFO_0004541	Hematological measurement	Hematological	PGS002573	MONDO_0005148	type 2 diabetes mellitus	type 2 diabetes mellitus	MONDO_0005148	Metabolic disorder	Metabolic
0.7804	7.69E-95	1.22E-91	PGS000826	EFO_0004530	triglyceride measurement	triglyceride measurement	EFO_0004530	Lipid or lipoprotein measurement	Metabolic	PGS002572	EFO_0004530	triglyceride measurement	triglyceride measurement	EFO_0004530	Lipid or lipoprotein measurement	Metabolic
0.7804	7.81E-95	1.24E-91	PGS003016	EFO_0000537	hypertension	hypertension	EFO_0000537	Cardiovascular disease	Cardiovascular	PGS003014	EFO_0000537	hypertension	hypertension	EFO_0000537	Cardiovascular disease	Cardiovascular
0.7803	8.46E-95	1.34E-91	PGS001682	EFO_0006930	brain volume measurement|neuroimaging measurement	brain volume measurement	EFO_0006930	Other measurement	Neurological	PGS001451	EFO_0006930	measurement	brain volume measurement	EFO_0006930	Other measurement	Neurological
0.7803	8.54E-95	1.35E-91	PGS001543	EFO_0004830	caudate nucleus volume|neuroimaging measurement	caudate nucleus volume	EFO_0004830	Other measurement	Neurological	PGS001542	EFO_0004830	measurement	caudate nucleus volume	EFO_0004830	Other measurement	Neurological
0.7803	8.81E-95	1.40E-91	PGS003015	EFO_0000537	hypertension	hypertension	EFO_0000537	Cardiovascular disease	Cardiovascular	PGS003014	EFO_0000537	hypertension	hypertension	EFO_0000537	Cardiovascular disease	Cardiovascular
0.7802	9.01E-95	1.43E-91	PGS002854	EFO_0004340	body mass index	body mass index	EFO_0004340	Body measurement	Body measurement	PGS000921	EFO_0004340	body mass index	body mass index	EFO_0004340	Body measurement	Body measurement
0.7802	9.20E-95	1.46E-91	PGS000478	EFO_0000305	breast carcinoma	breast carcinoma	EFO_0000305	Cancer	Neoplasms	PGS000480	EFO_0000305	breast carcinoma	breast carcinoma	EFO_0000305	Cancer	Neoplasms
0.7802	9.25E-95	1.46E-91	PGS002896	EFO_0006336	diastolic blood pressure	diastolic blood pressure	EFO_0006336	Other measurement	Cardiovascular	PGS002890	EFO_0006336	diastolic blood pressure	diastolic blood pressure	EFO_0006336	Other measurement	Cardiovascular
0.7802	9.51E-95	1.51E-91	PGS002844	EFO_0004340	body mass index	body mass index	EFO_0004340	Body measurement	Body measurement	PGS000921	EFO_0004340	body mass index	body mass index	EFO_0004340	Body measurement	Body measurement
0.7801	1.03E-94	1.63E-91	PGS003244	EFO_0010472	creatine measurement	creatine measurement	EFO_0010472	Other measurement	Metabolic	PGS003243	EFO_0010472	creatine measurement	creatine measurement	EFO_0010472	Other measurement	Metabolic
0.78	1.08E-94	1.70E-91	PGS000593	EFO_0007355	male reproductive organ cancer	male reproductive organ cancer	EFO_0007355	Cancer	Neoplasms	PGS000571	EFO_0001663	prostate carcinoma	prostate carcinoma	EFO_0001663	Cancer	Neoplasms
0.78	1.10E-94	1.74E-91	PGS002189	EFO_0004584	mean platelet volume	mean platelet volume	EFO_0004584	Hematological measurement	Hematological	PGS000104	EFO_0004584	mean platelet volume	mean platelet volume	EFO_0004584	Hematological measurement	Hematological
0.78	1.10E-94	1.74E-91	PGS000771	MONDO_0007915	systemic lupus erythematosus	systemic lupus erythematosus	MONDO_0007915	Immune system disorder	Immunological	PGS000803	MONDO_0007915	systemic lupus erythematosus	systemic lupus erythematosus	MONDO_0007915	Immune system disorder	Immunological
0.78	1.12E-94	1.77E-91	PGS000504	EFO_0000305	breast carcinoma	breast carcinoma	EFO_0000305	Cancer	Neoplasms	PGS000008	EFO_1000649	estrogen-receptor positive breast cancer	cancer	EFO_1000649	Cancer	Neoplasms
0.78	1.13E-94	1.78E-91	PGS000873	EFO_0000305	breast carcinoma	breast carcinoma	EFO_0000305	Cancer	Neoplasms	PGS000045	EFO_0000305	breast carcinoma	breast carcinoma	EFO_0000305	Cancer	Neoplasms
0.7799	1.19E-94	1.88E-91	PGS002501	EFO_0004541	HbA1c measurement	HbA1c measurement	EFO_0004541	Hematological measurement	Hematological	PGS002573	MONDO_0005148	type 2 diabetes mellitus	type 2 diabetes mellitus	MONDO_0005148	Metabolic disorder	Metabolic
0.7799	1.19E-94	1.88E-91	PGS002501	EFO_0004541	HbA1c measurement	HbA1c measurement	EFO_0004541	Hematological measurement	Hematological	PGS002573	MONDO_0005148	type 2 diabetes mellitus	type 2 diabetes mellitus	MONDO_0005148	Digestive system disorder	Gastrointestinal
0.7798	1.40E-94	2.22E-91	PGS002474	EFO_0004530	triglyceride measurement	triglyceride measurement	EFO_0004530	Lipid or lipoprotein measurement	Metabolic	PGS002523	EFO_0004530	triglyceride measurement	triglyceride measurement	EFO_0004530	Lipid or lipoprotein measurement	Metabolic
0.7797	1.42E-94	2.24E-91	PGS002592	EFO_0001379	endocrine system disease|diabetes mellitus	endocrine system disease	EFO_0001379	Other disease	Metabolic	PGS002491	EFO_0000400	diabetes mellitus	diabetes mellitus	EFO_0000400	Digestive system disorder	Gastrointestinal
0.7797	1.42E-94	2.24E-91	PGS002592	EFO_0001379	endocrine system disease|diabetes mellitus	endocrine system disease	EFO_0001379	Other disease	Metabolic	PGS002491	EFO_0000400	diabetes mellitus	diabetes mellitus	EFO_0000400	Metabolic disorder	Metabolic
0.7797	1.43E-94	2.25E-91	PGS001160	EFO_0005106	body composition measurement	body composition measurement	EFO_0005106	Body measurement	Body measurement	PGS001984	EFO_0009805	whole body water mass	whole body water mass	EFO_0009805	Other measurement	Body measurement
0.7797	1.47E-94	2.31E-91	PGS002536	EFO_0007862	reproductive behaviour measurement	reproductive behaviour measurement	EFO_0007862	Other measurement	Activities	PGS002585	EFO_0007862	reproductive behaviour measurement	reproductive behaviour measurement	EFO_0007862	Other measurement	Activities
0.7797	1.53E-94	2.41E-91	PGS003355	EFO_0001645	coronary artery disease	coronary artery disease	EFO_0001645	Cardiovascular disease	Cardiovascular	PGS001355	EFO_0001645	coronary artery disease	coronary artery disease	EFO_0001645	Cardiovascular disease	Cardiovascular
0.7796	1.55E-94	2.44E-91	PGS003183	EFO_0004631	vitamin D measurement	vitamin D measurement	EFO_0004631	Lipid or lipoprotein measurement	Metabolic	PGS003178	EFO_0004631	vitamin D measurement	vitamin D measurement	EFO_0004631	Lipid or lipoprotein measurement	Metabolic
0.7796	1.57E-94	2.47E-91	PGS001264	EFO_0003907	deep vein thrombosis	deep vein thrombosis	EFO_0003907	Other trait	Cardiovascular	PGS000930	MONDO_0000831	thrombotic disease	thrombotic disease	MONDO_0000831	Cardiovascular disease	Cardiovascular
0.7796	1.63E-94	2.57E-91	PGS001105	EFO_0007800	body fat percentage	body fat percentage	EFO_0007800	Body measurement	Body measurement	PGS001104	EFO_0007800	body fat percentage	body fat percentage	EFO_0007800	Body measurement	Body measurement
0.7795	1.72E-94	2.71E-91	PGS000507	EFO_0000305	breast carcinoma	breast carcinoma	EFO_0000305	Cancer	Neoplasms	PGS000007	EFO_0000305	breast carcinoma	breast carcinoma	EFO_0000305	Cancer	Neoplasms
0.7795	1.78E-94	2.80E-91	PGS002005	EFO_0011011	sitting height measurement	sitting height measurement	EFO_0011011	Other measurement	Skeletal	PGS002146	EFO_0004339	body height	body height	EFO_0004339	Body measurement	Body measurement
0.7795	1.80E-94	2.83E-91	PGS000538	EFO_0000305	breast carcinoma	breast carcinoma	EFO_0000305	Cancer	Neoplasms	PGS000506	EFO_0000305	breast carcinoma	breast carcinoma	EFO_0000305	Cancer	Neoplasms
0.7795	1.80E-94	2.83E-91	PGS000538	EFO_0000305	breast carcinoma	breast carcinoma	EFO_0000305	Cancer	Neoplasms	PGS000505	EFO_0000305	breast carcinoma	breast carcinoma	EFO_0000305	Cancer	Neoplasms
0.7795	1.85E-94	2.91E-91	PGS003199	EFO_0004631	vitamin D measurement	vitamin D measurement	EFO_0004631	Lipid or lipoprotein measurement	Metabolic	PGS002574	EFO_0004631	vitamin D measurement	vitamin D measurement	EFO_0004631	Lipid or lipoprotein measurement	Metabolic
0.7794	1.86E-94	2.92E-91	PGS001682	EFO_0006930	brain volume measurement|neuroimaging measurement	brain volume measurement	EFO_0006930	Other measurement	Neurological	PGS001673	EFO_0006930	measurement	brain volume measurement	EFO_0006930	Other measurement	Neurological
-0.7794	1.89E-94	2.97E-91	PGS003403	EFO_0004611	low density lipoprotein cholesterol measurement	low density lipoprotein cholesterol measurement	EFO_0004611	Lipid or lipoprotein measurement	Metabolic	PGS000061	EFO_0004611	low density lipoprotein cholesterol measurement	measurement	EFO_0004611	Lipid or lipoprotein measurement	Metabolic
-0.7794	1.89E-94	2.97E-91	PGS000061	EFO_0004611	low density lipoprotein cholesterol measurement	low density lipoprotein cholesterol measurement	EFO_0004611	Cardiovascular measurement	Cardiovascular	PGS000340	EFO_0004611	low density lipoprotein cholesterol measurement	measurement	EFO_0004611	Cardiovascular measurement	Cardiovascular
-0.7794	1.89E-94	2.97E-91	PGS000061	EFO_0004611	low density lipoprotein cholesterol measurement	low density lipoprotein cholesterol measurement	EFO_0004611	Cardiovascular measurement	Cardiovascular	PGS000340	EFO_0004611	low density lipoprotein cholesterol measurement	measurement	EFO_0004611	Lipid or lipoprotein measurement	Metabolic
-0.7794	1.89E-94	2.97E-91	PGS003403	EFO_0004611	low density lipoprotein cholesterol measurement	low density lipoprotein cholesterol measurement	EFO_0004611	Cardiovascular measurement	Cardiovascular	PGS000061	EFO_0004611	low density lipoprotein cholesterol measurement	measurement	EFO_0004611	Cardiovascular measurement	Cardiovascular
-0.7794	1.89E-94	2.97E-91	PGS003403	EFO_0004611	low density lipoprotein cholesterol measurement	low density lipoprotein cholesterol measurement	EFO_0004611	Cardiovascular measurement	Cardiovascular	PGS000061	EFO_0004611	low density lipoprotein cholesterol measurement	measurement	EFO_0004611	Lipid or lipoprotein measurement	Metabolic
-0.7794	1.89E-94	2.97E-91	PGS000061	EFO_0004611	low density lipoprotein cholesterol measurement	low density lipoprotein cholesterol measurement	EFO_0004611	Lipid or lipoprotein measurement	Metabolic	PGS000340	EFO_0004611	low density lipoprotein cholesterol measurement	measurement	EFO_0004611	Cardiovascular measurement	Cardiovascular
-0.7794	1.89E-94	2.97E-91	PGS003403	EFO_0004611	low density lipoprotein cholesterol measurement	low density lipoprotein cholesterol measurement	EFO_0004611	Lipid or lipoprotein measurement	Metabolic	PGS000061	EFO_0004611	low density lipoprotein cholesterol measurement	measurement	EFO_0004611	Cardiovascular measurement	Cardiovascular
-0.7794	1.89E-94	2.97E-91	PGS000061	EFO_0004611	low density lipoprotein cholesterol measurement	low density lipoprotein cholesterol measurement	EFO_0004611	Lipid or lipoprotein measurement	Metabolic	PGS000340	EFO_0004611	low density lipoprotein cholesterol measurement	measurement	EFO_0004611	Lipid or lipoprotein measurement	Metabolic
0.7794	1.94E-94	3.05E-91	PGS002147	EFO_0009253	Immature Reticulocyte Fraction Measurement	Immature Reticulocyte Fraction Measurement	EFO_0009253	Hematological measurement	Hematological	PGS001408	EFO_0009253	Immature Reticulocyte Fraction Measurement	Measurement	EFO_0009253	Hematological measurement	Hematological
-0.7794	1.99E-94	3.12E-91	PGS003228	EFO_0005208	glomerular filtration rate	glomerular filtration rate	EFO_0005208	Other measurement	Genitourinary	PGS002237	EFO_0003884	chronic kidney disease	chronic kidney disease	EFO_0003884	Other disease	Metabolic
0.7794	2.00E-94	3.14E-91	PGS001162	EFO_0005093	hip circumference	hip circumference	EFO_0005093	Body measurement	Body measurement	PGS001227	EFO_0004342	waist circumference	waist circumference	EFO_0004342	Body measurement	Body measurement
-0.7793	2.09E-94	3.28E-91	PGS003293	EFO_0004617	cystatin C measurement|glomerular filtration rate	cystatin C measurement	EFO_0004617	Other measurement	Metabolic	PGS003303	EFO_0004617	cystatin C measurement|glomerular filtration rate	cystatin C measurement	EFO_0004617	Other measurement	Metabolic
0.7792	2.25E-94	3.53E-91	PGS002802	EFO_0004339	body height	body height	EFO_0004339	Body measurement	Body measurement	PGS002146	EFO_0004339	body height	body height	EFO_0004339	Body measurement	Body measurement
0.7792	2.27E-94	3.55E-91	PGS000574	EFO_0001663	prostate carcinoma	prostate carcinoma	EFO_0001663	Cancer	Neoplasms	PGS000570	EFO_0001663	prostate carcinoma	prostate carcinoma	EFO_0001663	Cancer	Neoplasms
-0.7791	2.48E-94	3.89E-91	PGS001937	EFO_0004279	suntan	suntan	EFO_0004279	Other trait	Dermatological	PGS002350	EFO_0004279	suntan	suntan	EFO_0004279	Other trait	Dermatological
0.779	2.70E-94	4.23E-91	PGS001841	EFO_0003911	atrial flutter|atrial fibrillation	atrial flutter	EFO_0003911	Cardiovascular disease	Cardiovascular	PGS001339	EFO_0000275	atrial fibrillation|atrial flutter	atrial fibrillation	EFO_0000275	Cardiovascular disease	Cardiovascular
0.779	2.88E-94	4.52E-91	PGS003184	EFO_0004631	vitamin D measurement	vitamin D measurement	EFO_0004631	Lipid or lipoprotein measurement	Metabolic	PGS002574	EFO_0004631	vitamin D measurement	vitamin D measurement	EFO_0004631	Lipid or lipoprotein measurement	Metabolic
0.7789	3.04E-94	4.76E-91	PGS001704	EFO_0006930	brain volume measurement|neuroimaging measurement	brain volume measurement	EFO_0006930	Other measurement	Neurological	PGS001749	EFO_0006930	measurement	brain volume measurement	EFO_0006930	Other measurement	Neurological
0.7788	3.30E-94	5.17E-91	PGS000511	EFO_0000305	breast carcinoma	breast carcinoma	EFO_0000305	Cancer	Neoplasms	PGS000332	EFO_0000305	breast carcinoma	breast carcinoma	EFO_0000305	Cancer	Neoplasms
0.7788	3.44E-94	5.38E-91	PGS002776	EFO_0001645	coronary artery disease	coronary artery disease	EFO_0001645	Cardiovascular disease	Cardiovascular	PGS000013	EFO_0001645	coronary artery disease	coronary artery disease	EFO_0001645	Cardiovascular disease	Cardiovascular
0.7786	3.91E-94	6.11E-91	PGS000008	EFO_1000649	estrogen-receptor positive breast cancer	estrogen-receptor positive breast cancer	EFO_1000649	Cancer	Neoplasms	PGS000512	EFO_0000305	breast carcinoma	breast carcinoma	EFO_0000305	Cancer	Neoplasms
0.7786	4.05E-94	6.32E-91	PGS001144	EFO_0005409	fat body mass	fat body mass	EFO_0005409	Other measurement	Body measurement	PGS001101	EFO_0007800	body fat percentage	body fat percentage	EFO_0007800	Body measurement	Body measurement
0.7786	4.06E-94	6.35E-91	PGS001671	EFO_0006930	brain volume measurement|neuroimaging measurement	brain volume measurement	EFO_0006930	Other measurement	Neurological	PGS001681	EFO_0006930	measurement	brain volume measurement	EFO_0006930	Other measurement	Neurological
-0.7785	4.20E-94	6.56E-91	PGS000098	EFO_0007993	lymphocyte percentage of leukocytes	lymphocyte percentage of leukocytes	EFO_0007993	Hematological measurement	Hematological	PGS000106	EFO_0007990	neutrophil percentage of leukocytes	neutrophil percentage of leukocytes	EFO_0007990	Hematological measurement	Hematological
0.7785	4.32E-94	6.75E-91	PGS001381	EFO_0010066	corneal hysteresis	corneal hysteresis	EFO_0010066	Other measurement	Ophthalmological	PGS001383	EFO_0010067	corneal resistance factor	corneal resistance factor	EFO_0010067	Other measurement	Ophthalmological
0.7785	4.39E-94	6.85E-91	PGS000509	EFO_0000305	breast carcinoma	breast carcinoma	EFO_0000305	Cancer	Neoplasms	PGS000512	EFO_0000305	breast carcinoma	breast carcinoma	EFO_0000305	Cancer	Neoplasms
0.7785	4.50E-94	7.02E-91	PGS001731	EFO_0006930	brain volume measurement|neuroimaging measurement	brain volume measurement	EFO_0006930	Other measurement	Neurological	PGS001730	EFO_0006930	measurement	brain volume measurement	EFO_0006930	Other measurement	Neurological
0.7782	5.49E-94	8.57E-91	PGS000511	EFO_0000305	breast carcinoma	breast carcinoma	EFO_0000305	Cancer	Neoplasms	PGS000500	EFO_0000305	breast carcinoma	breast carcinoma	EFO_0000305	Cancer	Neoplasms
0.7782	5.63E-94	8.78E-91	PGS003189	EFO_0004631	vitamin D measurement	vitamin D measurement	EFO_0004631	Lipid or lipoprotein measurement	Metabolic	PGS003198	EFO_0004631	vitamin D measurement	vitamin D measurement	EFO_0004631	Lipid or lipoprotein measurement	Metabolic
0.7782	5.76E-94	8.98E-91	PGS001160	EFO_0005106	body composition measurement	body composition measurement	EFO_0005106	Body measurement	Body measurement	PGS001950	EFO_0004995	lean body mass	lean body mass	EFO_0004995	Other measurement	Skeletal
0.7782	5.82E-94	9.07E-91	PGS000046	EFO_1000649	estrogen-receptor positive breast cancer	estrogen-receptor positive breast cancer	EFO_1000649	Cancer	Neoplasms	PGS000873	EFO_0000305	breast carcinoma	breast carcinoma	EFO_0000305	Cancer	Neoplasms
0.7782	5.90E-94	9.19E-91	PGS002343	EFO_0004309	platelet count	platelet count	EFO_0004309	Hematological measurement	Hematological	PGS002660	EFO_0004309	platelet count	platelet count	EFO_0004309	Hematological measurement	Hematological
0.7781	6.00E-94	9.35E-91	PGS002632	EFO_0003923	bone density	bone density	EFO_0003923	Other measurement	Skeletal	PGS002175	EFO_0005654	velocity of sound measurement	velocity of sound measurement	EFO_0005654	Other measurement	Others
0.7781	6.27E-94	9.77E-91	PGS002087	EFO_1000243	Epidermal Inclusion Cyst	Epidermal Inclusion Cyst	EFO_1000243	Cancer	Neoplasms	PGS001873	MONDO_0002917	disorder of pilosebaceous unit	disorder of pilosebaceous unit	MONDO_0002917	Other disease	Dermatological
0.7779	7.22E-94	1.12E-90	PGS001943	EFO_0004340	body mass index	body mass index	EFO_0004340	Body measurement	Body measurement	PGS002845	EFO_0004340	body mass index	body mass index	EFO_0004340	Body measurement	Body measurement
0.7779	7.56E-94	1.18E-90	PGS000023	MONDO_0005147	type 1 diabetes mellitus	type 1 diabetes mellitus	MONDO_0005147	Digestive system disorder	Gastrointestinal	PGS001820	HP_0001943	Hypoglycemia	Hypoglycemia	HP_0001943	Other trait	Metabolic
0.7779	7.56E-94	1.18E-90	PGS000023	MONDO_0005147	type 1 diabetes mellitus	type 1 diabetes mellitus	MONDO_0005147	Immune system disorder	Immunological	PGS001820	HP_0001943	Hypoglycemia	Hypoglycemia	HP_0001943	Other trait	Metabolic
0.7779	7.56E-94	1.18E-90	PGS000023	MONDO_0005147	type 1 diabetes mellitus	type 1 diabetes mellitus	MONDO_0005147	Metabolic disorder	Metabolic	PGS001820	HP_0001943	Hypoglycemia	Hypoglycemia	HP_0001943	Other trait	Metabolic
0.7779	7.61E-94	1.18E-90	PGS001670	EFO_0006930	brain volume measurement|neuroimaging measurement	brain volume measurement	EFO_0006930	Other measurement	Neurological	PGS001451	EFO_0006930	measurement	brain volume measurement	EFO_0006930	Other measurement	Neurological
0.7779	7.80E-94	1.21E-90	PGS000069	EFO_1001516	ovarian serous carcinoma	ovarian serous carcinoma	EFO_1001516	Cancer	Neoplasms	PGS000559	EFO_0003893	ovarian neoplasm	ovarian neoplasm	EFO_0003893	Cancer	Neoplasms
0.7779	7.79E-94	1.21E-90	PGS002996	EFO_0000537	hypertension	hypertension	EFO_0000537	Cardiovascular disease	Cardiovascular	PGS002994	EFO_0000537	hypertension	hypertension	EFO_0000537	Cardiovascular disease	Cardiovascular
0.7779	7.80E-94	1.21E-90	PGS000069	EFO_1001516	ovarian serous carcinoma	ovarian serous carcinoma	EFO_1001516	Cancer	Neoplasms	PGS000560	EFO_0003893	ovarian neoplasm	ovarian neoplasm	EFO_0003893	Cancer	Neoplasms
0.7778	7.88E-94	1.22E-90	PGS000008	EFO_1000649	estrogen-receptor positive breast cancer	estrogen-receptor positive breast cancer	EFO_1000649	Cancer	Neoplasms	PGS000335	EFO_0000305	breast carcinoma	breast carcinoma	EFO_0000305	Cancer	Neoplasms
0.7777	9.10E-94	1.41E-90	PGS002834	EFO_0007878	alcohol consumption measurement	alcohol consumption measurement	EFO_0007878	Other measurement	Psychiatric	PGS002830	EFO_0007878	alcohol consumption measurement	alcohol consumption measurement	EFO_0007878	Other measurement	Psychiatric
0.7777	9.17E-94	1.42E-90	PGS001952	EFO_0004468	glucose measurement	glucose measurement	EFO_0004468	Other measurement	Metabolic	PGS002645	EFO_0004468	glucose measurement	glucose measurement	EFO_0004468	Other measurement	Metabolic
0.7776	9.44E-94	1.46E-90	PGS000529	EFO_0000305	breast carcinoma	breast carcinoma	EFO_0000305	Cancer	Neoplasms	PGS000526	EFO_0000305	breast carcinoma	breast carcinoma	EFO_0000305	Cancer	Neoplasms
0.7776	9.44E-94	1.46E-90	PGS000525	EFO_0000305	breast carcinoma	breast carcinoma	EFO_0000305	Cancer	Neoplasms	PGS000529	EFO_0000305	breast carcinoma	breast carcinoma	EFO_0000305	Cancer	Neoplasms
0.7776	1.01E-93	1.57E-90	PGS002171	EFO_0004541	HbA1c measurement	HbA1c measurement	EFO_0004541	Hematological measurement	Hematological	PGS000685	EFO_0004541	HbA1c measurement	HbA1c measurement	EFO_0004541	Hematological measurement	Hematological
0.7775	1.06E-93	1.65E-90	PGS002782	EFO_0005689	non-high density lipoprotein cholesterol measurement	non-high density lipoprotein cholesterol measurement	EFO_0005689	Cardiovascular measurement	Cardiovascular	PGS003033	EFO_0004611	low density lipoprotein cholesterol measurement	measurement	EFO_0004611	Cardiovascular measurement	Cardiovascular
0.7775	1.06E-93	1.65E-90	PGS002782	EFO_0005689	non-high density lipoprotein cholesterol measurement	non-high density lipoprotein cholesterol measurement	EFO_0005689	Cardiovascular measurement	Cardiovascular	PGS003033	EFO_0004611	low density lipoprotein cholesterol measurement	measurement	EFO_0004611	Lipid or lipoprotein measurement	Metabolic
0.7775	1.06E-93	1.65E-90	PGS002782	EFO_0005689	non-high density lipoprotein cholesterol measurement	non-high density lipoprotein cholesterol measurement	EFO_0005689	Lipid or lipoprotein measurement	Metabolic	PGS003033	EFO_0004611	low density lipoprotein cholesterol measurement	measurement	EFO_0004611	Cardiovascular measurement	Cardiovascular

0.7775	1.06E-93	1.65E-90	PGS002782	EFO_0005689	non-high density lipoprotein cholesterol measurement	non-high density lipoprotein cholesterol measurement	EFO_0005689	Lipid or lipoprotein measurement	Metabolic	PGS003033	EFO_0004611	low density lipoprotein cholesterol measurement	measurement	EFO_0004611	Lipid or lipoprotein measurement	Metabolic
-0.7772	1.38E-93	2.14E-90	PGS003293	EFO_0004617	cystatin C measurement|glomerular filtration rate	cystatin C measurement	EFO_0004617	Other measurement	Metabolic	PGS003318	EFO_0004617	cystatin C measurement|glomerular filtration rate	cystatin C measurement	EFO_0004617	Other measurement	Metabolic
0.7772	1.39E-93	2.16E-90	PGS002329	EFO_0004612	high density lipoprotein cholesterol measurement	high density lipoprotein cholesterol measurement	EFO_0004612	Lipid or lipoprotein measurement	Metabolic	PGS002101	EFO_0004614	apolipoprotein A 1 measurement	apolipoprotein A 1 measurement	EFO_0004614	Lipid or lipoprotein measurement	Metabolic
0.7772	1.39E-93	2.16E-90	PGS002329	EFO_0004612	high density lipoprotein cholesterol measurement	high density lipoprotein cholesterol measurement	EFO_0004612	Lipid or lipoprotein measurement	Metabolic	PGS002101	EFO_0004614	apolipoprotein A 1 measurement	apolipoprotein A 1 measurement	EFO_0004614	Cardiovascular measurement	Cardiovascular
0.7772	1.39E-93	2.16E-90	PGS002329	EFO_0004612	high density lipoprotein cholesterol measurement	high density lipoprotein cholesterol measurement	EFO_0004612	Lipid or lipoprotein measurement	Metabolic	PGS002101	EFO_0004614	apolipoprotein A 1 measurement	apolipoprotein A 1 measurement	EFO_0004614	Inflammatory measurement	Immunological
0.7772	1.41E-93	2.18E-90	PGS001820	HP_0001943	Hypoglycemia	Hypoglycemia	HP_0001943	Other trait	Metabolic	PGS001296	MONDO_0005147	type 1 diabetes mellitus	type 1 diabetes mellitus	MONDO_0005147	Immune system disorder	Immunological
0.7772	1.41E-93	2.18E-90	PGS001820	HP_0001943	Hypoglycemia	Hypoglycemia	HP_0001943	Other trait	Metabolic	PGS001296	MONDO_0005147	type 1 diabetes mellitus	type 1 diabetes mellitus	MONDO_0005147	Digestive system disorder	Gastrointestinal
0.7772	1.41E-93	2.18E-90	PGS001820	HP_0001943	Hypoglycemia	Hypoglycemia	HP_0001943	Other trait	Metabolic	PGS001296	MONDO_0005147	type 1 diabetes mellitus	type 1 diabetes mellitus	MONDO_0005147	Metabolic disorder	Metabolic
0.777	1.72E-93	2.67E-90	PGS000312	EFO_0004530	triglyceride measurement	triglyceride measurement	EFO_0004530	Lipid or lipoprotein measurement	Metabolic	PGS002621	EFO_0004530	triglyceride measurement	triglyceride measurement	EFO_0004530	Lipid or lipoprotein measurement	Metabolic
0.7769	1.84E-93	2.84E-90	PGS003143	EFO_0004574	total cholesterol measurement	total cholesterol measurement	EFO_0004574	Lipid or lipoprotein measurement	Metabolic	PGS001895	EFO_0004574	total cholesterol measurement	total cholesterol measurement	EFO_0004574	Lipid or lipoprotein measurement	Metabolic
0.7768	1.95E-93	3.01E-90	PGS001077	EFO_0007990	neutrophil percentage of leukocytes	neutrophil percentage of leukocytes	EFO_0007990	Hematological measurement	Hematological	PGS002214	EFO_0007990	neutrophil percentage of leukocytes	neutrophil percentage of leukocytes	EFO_0007990	Hematological measurement	Hematological
0.7768	1.96E-93	3.03E-90	PGS002658	EFO_0005091	monocyte count	monocyte count	EFO_0005091	Hematological measurement	Hematological	PGS002341	EFO_0005091	monocyte count	monocyte count	EFO_0005091	Hematological measurement	Hematological
0.7768	1.97E-93	3.05E-90	PGS001736	EFO_0006930	brain volume measurement|neuroimaging measurement	brain volume measurement	EFO_0006930	Other measurement	Neurological	PGS001735	EFO_0006930	measurement	brain volume measurement	EFO_0006930	Other measurement	Neurological
0.7768	2.03E-93	3.13E-90	PGS000213	EFO_0009780	HER2 negative breast carcinoma	HER2 negative breast carcinoma	EFO_0009780	Cancer	Neoplasms	PGS000005	EFO_1000649	estrogen-receptor positive breast cancer	cancer	EFO_1000649	Cancer	Neoplasms
0.7768	2.05E-93	3.17E-90	PGS001670	EFO_0006930	brain volume measurement|neuroimaging measurement	brain volume measurement	EFO_0006930	Other measurement	Neurological	PGS001466	EFO_0006930	measurement	brain volume measurement	EFO_0006930	Other measurement	Neurological
0.7767	2.25E-93	3.48E-90	PGS002208	EFO_0007989	monocyte percentage of leukocytes	monocyte percentage of leukocytes	EFO_0007989	Hematological measurement	Hematological	PGS001078	EFO_0007989	monocyte percentage of leukocytes	monocyte percentage of leukocytes	EFO_0007989	Hematological measurement	Hematological
0.7767	2.26E-93	3.49E-90	PGS000044	EFO_0001663	prostate carcinoma	prostate carcinoma	EFO_0001663	Cancer	Neoplasms	PGS000795	EFO_0001663	prostate carcinoma	prostate carcinoma	EFO_0001663	Cancer	Neoplasms
0.7766	2.35E-93	3.62E-90	PGS001264	EFO_0003907	deep vein thrombosis	deep vein thrombosis	EFO_0003907	Other trait	Cardiovascular	PGS001279	EFO_0003827	pulmonary embolism|deep vein thrombosis	pulmonary embolism	EFO_0003827	Cardiovascular disease	Cardiovascular
0.7765	2.60E-93	4.01E-90	PGS000774	EFO_1000649	estrogen-receptor positive breast cancer	estrogen-receptor positive breast cancer	EFO_1000649	Cancer	Neoplasms	PGS000002	EFO_1000649	estrogen-receptor positive breast cancer	cancer	EFO_1000649	Cancer	Neoplasms
0.7765	2.73E-93	4.20E-90	PGS000533	EFO_0000305	breast carcinoma	breast carcinoma	EFO_0000305	Cancer	Neoplasms	PGS000540	EFO_0000305	breast carcinoma	breast carcinoma	EFO_0000305	Cancer	Neoplasms
0.7765	2.73E-93	4.20E-90	PGS000534	EFO_0000305	breast carcinoma	breast carcinoma	EFO_0000305	Cancer	Neoplasms	PGS000540	EFO_0000305	breast carcinoma	breast carcinoma	EFO_0000305	Cancer	Neoplasms
0.7764	2.81E-93	4.33E-90	PGS000480	EFO_0000305	breast carcinoma	breast carcinoma	EFO_0000305	Cancer	Neoplasms	PGS001337	EFO_0005606	family history of breast cancer	family history of breast cancer	EFO_0005606	Other trait	Neoplasms
0.7764	2.89E-93	4.45E-90	PGS000551	EFO_0003893	ovarian neoplasm	ovarian neoplasm	EFO_0003893	Cancer	Neoplasms	PGS000563	EFO_0003893	ovarian neoplasm	ovarian neoplasm	EFO_0003893	Cancer	Neoplasms
0.7763	3.04E-93	4.68E-90	PGS003119	MONDO_0005148	type 2 diabetes mellitus	type 2 diabetes mellitus	MONDO_0005148	Metabolic disorder	Metabolic	PGS000014	MONDO_0005148	type 2 diabetes mellitus	type 2 diabetes mellitus	MONDO_0005148	Metabolic disorder	Metabolic
0.7763	3.04E-93	4.68E-90	PGS003119	MONDO_0005148	type 2 diabetes mellitus	type 2 diabetes mellitus	MONDO_0005148	Digestive system disorder	Gastrointestinal	PGS000014	MONDO_0005148	type 2 diabetes mellitus	type 2 diabetes mellitus	MONDO_0005148	Digestive system disorder	Gastrointestinal
0.7763	3.04E-93	4.68E-90	PGS003119	MONDO_0005148	type 2 diabetes mellitus	type 2 diabetes mellitus	MONDO_0005148	Digestive system disorder	Gastrointestinal	PGS000014	MONDO_0005148	type 2 diabetes mellitus	type 2 diabetes mellitus	MONDO_0005148	Metabolic disorder	Metabolic
0.7763	3.04E-93	4.68E-90	PGS003119	MONDO_0005148	type 2 diabetes mellitus	type 2 diabetes mellitus	MONDO_0005148	Metabolic disorder	Metabolic	PGS000014	MONDO_0005148	type 2 diabetes mellitus	type 2 diabetes mellitus	MONDO_0005148	Digestive system disorder	Gastrointestinal
0.7763	3.08E-93	4.74E-90	PGS003194	EFO_0004631	vitamin D measurement	vitamin D measurement	EFO_0004631	Lipid or lipoprotein measurement	Metabolic	PGS003193	EFO_0004631	vitamin D measurement	vitamin D measurement	EFO_0004631	Lipid or lipoprotein measurement	Metabolic
0.7763	3.17E-93	4.88E-90	PGS001485	EFO_0006930	brain volume measurement|neuroimaging measurement	brain volume measurement	EFO_0006930	Other measurement	Neurological	PGS001489	EFO_0006930	measurement	brain volume measurement	EFO_0006930	Other measurement	Neurological
0.7763	3.21E-93	4.94E-90	PGS000008	EFO_1000649	estrogen-receptor positive breast cancer	estrogen-receptor positive breast cancer	EFO_1000649	Cancer	Neoplasms	PGS000503	EFO_0000305	breast carcinoma	breast carcinoma	EFO_0000305	Cancer	Neoplasms
0.7763	3.22E-93	4.95E-90	PGS003411	EFO_0003870	brain aneurysm|female	brain aneurysm	EFO_0003870	Neurological disorder	Neurological	PGS003408	EFO_0003870	brain aneurysm|female	brain aneurysm	EFO_0003870	Neurological disorder	Neurological
0.7763	3.22E-93	4.95E-90	PGS003411	EFO_0003870	brain aneurysm|female	brain aneurysm	EFO_0003870	Neurological disorder	Neurological	PGS003408	EFO_0003870	brain aneurysm|female	brain aneurysm	EFO_0003870	Cardiovascular disease	Cardiovascular
0.7763	3.22E-93	4.95E-90	PGS003411	EFO_0003870	brain aneurysm|female	brain aneurysm	EFO_0003870	Cardiovascular disease	Cardiovascular	PGS003408	EFO_0003870	brain aneurysm|female	brain aneurysm	EFO_0003870	Cardiovascular disease	Cardiovascular
0.7763	3.22E-93	4.95E-90	PGS003411	EFO_0003870	brain aneurysm|female	brain aneurysm	EFO_0003870	Cardiovascular disease	Cardiovascular	PGS003408	EFO_0003870	brain aneurysm|female	brain aneurysm	EFO_0003870	Neurological disorder	Neurological
0.7762	3.53E-93	5.42E-90	PGS003181	EFO_0004631	vitamin D measurement	vitamin D measurement	EFO_0004631	Lipid or lipoprotein measurement	Metabolic	PGS003182	EFO_0004631	vitamin D measurement	vitamin D measurement	EFO_0004631	Lipid or lipoprotein measurement	Metabolic
0.7761	3.62E-93	5.56E-90	PGS002321	EFO_0000400	diabetes mellitus	diabetes mellitus	EFO_0000400	Digestive system disorder	Gastrointestinal	PGS003089	MONDO_0005148	type 2 diabetes mellitus	type 2 diabetes mellitus	MONDO_0005148	Metabolic disorder	Metabolic
0.7761	3.62E-93	5.56E-90	PGS002321	EFO_0000400	diabetes mellitus	diabetes mellitus	EFO_0000400	Digestive system disorder	Gastrointestinal	PGS003089	MONDO_0005148	type 2 diabetes mellitus	type 2 diabetes mellitus	MONDO_0005148	Digestive system disorder	Gastrointestinal
0.7761	3.62E-93	5.56E-90	PGS002321	EFO_0000400	diabetes mellitus	diabetes mellitus	EFO_0000400	Metabolic disorder	Metabolic	PGS003089	MONDO_0005148	type 2 diabetes mellitus	type 2 diabetes mellitus	MONDO_0005148	Digestive system disorder	Gastrointestinal
0.7761	3.62E-93	5.56E-90	PGS002321	EFO_0000400	diabetes mellitus	diabetes mellitus	EFO_0000400	Metabolic disorder	Metabolic	PGS003089	MONDO_0005148	type 2 diabetes mellitus	type 2 diabetes mellitus	MONDO_0005148	Metabolic disorder	Metabolic
0.7761	3.67E-93	5.64E-90	PGS001488	EFO_0006930	brain volume measurement|neuroimaging measurement	brain volume measurement	EFO_0006930	Other measurement	Neurological	PGS001450	EFO_0006930	measurement	brain volume measurement	EFO_0006930	Other measurement	Neurological
0.7761	3.74E-93	5.75E-90	PGS000302	EFO_0006336	diastolic blood pressure	diastolic blood pressure	EFO_0006336	Other measurement	Cardiovascular	PGS000301	EFO_0006335	systolic blood pressure	systolic blood pressure	EFO_0006335	Other measurement	Cardiovascular
0.776	4.17E-93	6.40E-90	PGS002995	EFO_0000537	hypertension	hypertension	EFO_0000537	Cardiovascular disease	Cardiovascular	PGS002994	EFO_0000537	hypertension	hypertension	EFO_0000537	Cardiovascular disease	Cardiovascular
0.7759	4.42E-93	6.79E-90	PGS000474	EFO_0000305	breast carcinoma	breast carcinoma	EFO_0000305	Cancer	Neoplasms	PGS000513	EFO_0000305	breast carcinoma	breast carcinoma	EFO_0000305	Cancer	Neoplasms
0.7759	4.56E-93	6.99E-90	PGS000090	EFO_0004842	eosinophil count	eosinophil count	EFO_0004842	Hematological measurement	Hematological	PGS002325	EFO_0004842	eosinophil count	eosinophil count	EFO_0004842	Hematological measurement	Hematological
0.7758	5.01E-93	7.69E-90	PGS000775	EFO_1000650	estrogen-receptor negative breast cancer	estrogen-receptor negative breast cancer	EFO_1000650	Cancer	Neoplasms	PGS000773	EFO_0000305	breast carcinoma	breast carcinoma	EFO_0000305	Cancer	Neoplasms
0.7757	5.32E-93	8.16E-90	PGS001716	EFO_0006930	brain volume measurement|neuroimaging measurement	brain volume measurement	EFO_0006930	Other measurement	Neurological	PGS001745	EFO_0006930	measurement	brain volume measurement	EFO_0006930	Other measurement	Neurological
0.7756	5.72E-93	8.76E-90	PGS002776	EFO_0001645	coronary artery disease	coronary artery disease	EFO_0001645	Cardiovascular disease	Cardiovascular	PGS003355	EFO_0001645	coronary artery disease	coronary artery disease	EFO_0001645	Cardiovascular disease	Cardiovascular
0.7756	5.89E-93	9.03E-90	PGS003197	EFO_0004631	vitamin D measurement	vitamin D measurement	EFO_0004631	Lipid or lipoprotein measurement	Metabolic	PGS003192	EFO_0004631	vitamin D measurement	vitamin D measurement	EFO_0004631	Lipid or lipoprotein measurement	Metabolic
0.7755	6.15E-93	9.42E-90	PGS002164	EFO_0004458	C-reactive protein measurement	C-reactive protein measurement	EFO_0004458	Inflammatory measurement	Immunological	PGS002873	EFO_0004458	C-reactive protein measurement	C-reactive protein measurement	EFO_0004458	Inflammatory measurement	Immunological
0.7755	6.34E-93	9.70E-90	PGS001477	EFO_0006930	brain volume measurement|neuroimaging measurement	brain volume measurement	EFO_0006930	Other measurement	Neurological	PGS001672	EFO_0006930	measurement	brain volume measurement	EFO_0006930	Other measurement	Neurological
0.7755	6.56E-93	1.00E-89	PGS003289	EFO_0004617	cystatin C measurement|glomerular filtration rate	cystatin C measurement	EFO_0004617	Other measurement	Metabolic	PGS003304	EFO_0004617	cystatin C measurement|glomerular filtration rate	cystatin C measurement	EFO_0004617	Other measurement	Metabolic
0.7755	6.57E-93	1.01E-89	PGS002640	HP_0000964	Eczema	Eczema	HP_0000964	Other trait	Dermatological	PGS001285	MONDO_0005271	allergic disease	allergic disease	MONDO_0005271	Immune system disorder	Immunological
0.7754	6.75E-93	1.03E-89	PGS002224	EFO_0011011	sitting height measurement	sitting height measurement	EFO_0011011	Other measurement	Skeletal	PGS002973	EFO_0004339	body height	body height	EFO_0004339	Body measurement	Body measurement
0.7754	7.12E-93	1.09E-89	PGS001169	EFO_0004995	lean body mass	lean body mass	EFO_0004995	Other measurement	Skeletal	PGS002202	EFO_0009805	whole body water mass	whole body water mass	EFO_0009805	Other measurement	Body measurement
0.7753	7.34E-93	1.12E-89	PGS002936	EFO_0004468	glucose measurement	glucose measurement	EFO_0004468	Other measurement	Metabolic	PGS002934	EFO_0004468	glucose measurement	glucose measurement	EFO_0004468	Other measurement	Metabolic
0.7753	7.40E-93	1.13E-89	PGS002531	EFO_0005140	autoimmune disease	autoimmune disease	EFO_0005140	Immune system disorder	Immunological	PGS002570	EFO_1000627	thyroid disease	thyroid disease	EFO_1000627	Other disease	Endocrine
0.7753	7.41E-93	1.13E-89	PGS003069	EFO_0006335	systolic blood pressure	systolic blood pressure	EFO_0006335	Other measurement	Cardiovascular	PGS002807	EFO_0006335	systolic blood pressure	systolic blood pressure	EFO_0006335	Other measurement	Cardiovascular
0.7752	8.49E-93	1.30E-89	PGS000951	EFO_0004514	bone quantitative ultrasound measurement	bone quantitative ultrasound measurement	EFO_0004514	Other measurement	Skeletal	PGS002175	EFO_0005654	velocity of sound measurement	velocity of sound measurement	EFO_0005654	Other measurement	Others
0.7752	8.61E-93	1.31E-89	PGS003089	MONDO_0005148	type 2 diabetes mellitus	type 2 diabetes mellitus	MONDO_0005148	Digestive system disorder	Gastrointestinal	PGS003093	MONDO_0005148	type 2 diabetes mellitus	type 2 diabetes mellitus	MONDO_0005148	Digestive system disorder	Gastrointestinal
0.7752	8.61E-93	1.31E-89	PGS003089	MONDO_0005148	type 2 diabetes mellitus	type 2 diabetes mellitus	MONDO_0005148	Metabolic disorder	Metabolic	PGS003093	MONDO_0005148	type 2 diabetes mellitus	type 2 diabetes mellitus	MONDO_0005148	Digestive system disorder	Gastrointestinal
0.7752	8.61E-93	1.31E-89	PGS003089	MONDO_0005148	type 2 diabetes mellitus	type 2 diabetes mellitus	MONDO_0005148	Metabolic disorder	Metabolic	PGS003093	MONDO_0005148	type 2 diabetes mellitus	type 2 diabetes mellitus	MONDO_0005148	Metabolic disorder	Metabolic
0.7752	8.61E-93	1.31E-89	PGS003089	MONDO_0005148	type 2 diabetes mellitus	type 2 diabetes mellitus	MONDO_0005148	Digestive system disorder	Gastrointestinal	PGS003093	MONDO_0005148	type 2 diabetes mellitus	type 2 diabetes mellitus	MONDO_0005148	Metabolic disorder	Metabolic
0.7751	8.92E-93	1.36E-89	PGS002311	MONDO_0004979	asthma	asthma	MONDO_0004979	Other disease	Respiratory	PGS001344	MONDO_0004979	asthma	asthma	MONDO_0004979	Other disease	Respiratory
0.7751	9.40E-93	1.43E-89	PGS002108	EFO_0004574	total cholesterol measurement	total cholesterol measurement	EFO_0004574	Lipid or lipoprotein measurement	Metabolic	PGS003138	EFO_0004574	total cholesterol measurement	total cholesterol measurement	EFO_0004574	Lipid or lipoprotein measurement	Metabolic
0.775	9.53E-93	1.45E-89	PGS001956	EFO_0007772	calcaneal bone quantitative ultrasound measurement	calcaneal bone quantitative ultrasound measurement	EFO_0007772	Other measurement	Skeletal	PGS001529	EFO_0007772	measurement	measurement	EFO_0007772	Other measurement	Skeletal
0.775	9.61E-93	1.47E-89	PGS001488	EFO_0006930	brain volume measurement|neuroimaging measurement	brain volume measurement	EFO_0006930	Other measurement	Neurological	PGS001674	EFO_0006930	measurement	brain volume measurement	EFO_0006930	Other measurement	Neurological
0.775	1.00E-92	1.53E-89	PGS000538	EFO_0000305	breast carcinoma	breast carcinoma	EFO_0000305	Cancer	Neoplasms	PGS000498	EFO_0000305	breast carcinoma	breast carcinoma	EFO_0000305	Cancer	Neoplasms
0.775	1.00E-92	1.53E-89	PGS000538	EFO_0000305	breast carcinoma	breast carcinoma	EFO_0000305	Cancer	Neoplasms	PGS000497	EFO_0000305	breast carcinoma	breast carcinoma	EFO_0000305	Cancer	Neoplasms
0.775	1.03E-92	1.56E-89	PGS003398	EFO_0000305	breast carcinoma	breast carcinoma	EFO_0000305	Cancer	Neoplasms	PGS000045	EFO_0000305	breast carcinoma	breast carcinoma	EFO_0000305	Cancer	Neoplasms
0.775	1.04E-92	1.58E-89	PGS002478	EFO_0004308	leukocyte count	leukocyte count	EFO_0004308	Hematological measurement	Hematological	PGS002625	EFO_0004308	leukocyte count	leukocyte count	EFO_0004308	Hematological measurement	Hematological
0.7749	1.07E-92	1.62E-89	PGS001402	EFO_0009270	heel bone mineral density	heel bone mineral density	EFO_0009270	Other measurement	Skeletal	PGS002174	EFO_0007772	measurement	measurement	EFO_0007772	Other measurement	Skeletal
0.7749	1.12E-92	1.70E-89	PGS001076	EFO_0007991	eosinophil percentage of leukocytes	eosinophil percentage of leukocytes	EFO_0007991	Hematological measurement	Hematological	PGS002325	EFO_0004842	eosinophil count	eosinophil count	EFO_0004842	Hematological measurement	Hematological
0.7749	1.13E-92	1.72E-89	PGS000189	EFO_0007986	reticulocyte count	reticulocyte count	EFO_0007986	Hematological measurement	Hematological	PGS000170	EFO_0007986	reticulocyte count	reticulocyte count	EFO_0007986	Hematological measurement	Hematological
0.7748	1.16E-92	1.76E-89	PGS001716	EFO_0006930	brain volume measurement|neuroimaging measurement	brain volume measurement	EFO_0006930	Other measurement	Neurological	PGS001730	EFO_0006930	measurement	brain volume measurement	EFO_0006930	Other measurement	Neurological
0.7748	1.17E-92	1.79E-89	PGS001674	EFO_0006930	brain volume measurement|neuroimaging measurement	brain volume measurement	EFO_0006930	Other measurement	Neurological	PGS001478	EFO_0006930	measurement	brain volume measurement	EFO_0006930	Other measurement	Neurological
0.7748	1.19E-92	1.81E-89	PGS001037	EFO_0009270	heel bone mineral density	heel bone mineral density	EFO_0009270	Other measurement	Skeletal	PGS002174	EFO_0007772	measurement	measurement	EFO_0007772	Other measurement	Skeletal
0.7748	1.22E-92	1.85E-89	PGS000952	EFO_0004514	bone quantitative ultrasound measurement	bone quantitative ultrasound measurement	EFO_0004514	Other measurement	Skeletal	PGS002174	EFO_0007772	measurement	measurement	EFO_0007772	Other measurement	Skeletal
0.7747	1.30E-92	1.97E-89	PGS003248	EFO_0010472	creatine measurement	creatine measurement	EFO_0010472	Other measurement	Metabolic	PGS000883	EFO_0005208	glomerular filtration rate	glomerular filtration rate	EFO_0005208	Other measurement	Genitourinary
0.7747	1.34E-92	2.03E-89	PGS002408	EFO_0004705	hypothyroidism	hypothyroidism	EFO_0004705	Other disease	Endocrine	PGS002472	EFO_1000627	thyroid disease	thyroid disease	EFO_1000627	Other disease	Endocrine
0.7746	1.35E-92	2.05E-89	PGS000531	EFO_0000305	breast carcinoma	breast carcinoma	EFO_0000305	Cancer	Neoplasms	PGS000527	EFO_0000305	breast carcinoma	breast carcinoma	EFO_0000305	Cancer	Neoplasms
0.7746	1.38E-92	2.10E-89	PGS001633	EFO_0005420	measurement	grey matter volume measurement	EFO_0005420	Other measurement	Neurological	PGS001540	EFO_0008320	volume measurement|neuroimaging measurement	white matter volume measurement	EFO_0008320	Other measurement	Neurological
0.7746	1.41E-92	2.14E-89	PGS003143	EFO_0004574	total cholesterol measurement	total cholesterol measurement	EFO_0004574	Lipid or lipoprotein measurement	Metabolic	PGS003029	EFO_0004611	low density lipoprotein cholesterol measurement	measurement	EFO_0004611	Cardiovascular measurement	Cardiovascular
0.7746	1.41E-92	2.14E-89	PGS003143	EFO_0004574	total cholesterol measurement	total cholesterol measurement	EFO_0004574	Lipid or lipoprotein measurement	Metabolic	PGS003029	EFO_0004611	low density lipoprotein cholesterol measurement	measurement	EFO_0004611	Lipid or lipoprotein measurement	Metabolic
0.7745	1.53E-92	2.32E-89	PGS001943	EFO_0004340	body mass index	body mass index	EFO_0004340	Body measurement	Body measurement	PGS002850	EFO_0004340	body mass index	body mass index	EFO_0004340	Body measurement	Body measurement
0.7744	1.62E-92	2.46E-89	PGS000001	EFO_0000305	breast carcinoma	breast carcinoma	EFO_0000305	Cancer	Neoplasms	PGS000774	EFO_1000649	estrogen-receptor positive breast cancer	cancer	EFO_1000649	Cancer	Neoplasms
0.7744	1.64E-92	2.48E-89	PGS002543	EFO_0001379	endocrine system disease|diabetes mellitus	endocrine system disease	EFO_0001379	Other disease	Metabolic	PGS002501	EFO_0004541	HbA1c measurement	HbA1c measurement	EFO_0004541	Hematological measurement	Hematological
-0.7744	1.71E-92	2.59E-89	PGS003318	EFO_0004617	cystatin C measurement|glomerular filtration rate	cystatin C measurement	EFO_0004617	Other measurement	Metabolic	PGS003308	EFO_0004617	cystatin C measurement|glomerular filtration rate	cystatin C measurement	EFO_0004617	Other measurement	Metabolic
0.7742	1.97E-92	2.98E-89	PGS001478	EFO_0006930	brain volume measurement|neuroimaging measurement	brain volume measurement	EFO_0006930	Other measurement	Neurological	PGS001673	EFO_0006930	measurement	brain volume measurement	EFO_0006930	Other measurement	Neurological
0.7742	2.03E-92	3.08E-89	PGS002622	MONDO_0005148	type 2 diabetes mellitus	type 2 diabetes mellitus	MONDO_0005148	Digestive system disorder	Gastrointestinal	PGS002491	EFO_0000400	diabetes mellitus	diabetes mellitus	EFO_0000400	Digestive system disorder	Gastrointestinal
0.7742	2.03E-92	3.08E-89	PGS002622	MONDO_0005148	type 2 diabetes mellitus	type 2 diabetes mellitus	MONDO_0005148	Digestive system disorder	Gastrointestinal	PGS002491	EFO_0000400	diabetes mellitus	diabetes mellitus	EFO_0000400	Metabolic disorder	Metabolic
0.7742	2.03E-92	3.08E-89	PGS002622	MONDO_0005148	type 2 diabetes mellitus	type 2 diabetes mellitus	MONDO_0005148	Metabolic disorder	Metabolic	PGS002491	EFO_0000400	diabetes mellitus	diabetes mellitus	EFO_0000400	Digestive system disorder	Gastrointestinal
0.7742	2.03E-92	3.08E-89	PGS002622	MONDO_0005148	type 2 diabetes mellitus	type 2 diabetes mellitus	MONDO_0005148	Metabolic disorder	Metabolic	PGS002491	EFO_0000400	diabetes mellitus	diabetes mellitus	EFO_0000400	Metabolic disorder	Metabolic
0.7742	2.10E-92	3.18E-89	PGS003046	EFO_0006527	smoking status measurement	smoking status measurement	EFO_0006527	Other measurement	Psychiatric	PGS003045	EFO_0006527	smoking status measurement	smoking status measurement	EFO_0006527	Other measurement	Psychiatric
0.7741	2.12E-92	3.20E-89	PGS000479	EFO_0000305	breast carcinoma	breast carcinoma	EFO_0000305	Cancer	Neoplasms	PGS001337	EFO_0005606	family history of breast cancer	family history of breast cancer	EFO_0005606	Other trait	Neoplasms
0.7741	2.17E-92	3.28E-89	PGS001149	EFO_0005409	fat body mass	fat body mass	EFO_0005409	Other measurement	Body measurement	PGS001104	EFO_0007800	body fat percentage	body fat percentage	EFO_0007800	Body measurement	Body measurement
0.7741	2.22E-92	3.36E-89	PGS002965	EFO_0004339	body height	body height	EFO_0004339	Body measurement	Body measurement	PGS002969	EFO_0004339	body height	body height	EFO_0004339	Body measurement	Body measurement
0.7741	2.25E-92	3.40E-89	PGS001805	EFO_0001663	prostate carcinoma	prostate carcinoma	EFO_0001663	Cancer	Neoplasms	PGS001291	EFO_0001663	prostate carcinoma	prostate carcinoma	EFO_0001663	Cancer	Neoplasms
0.774	2.43E-92	3.68E-89	PGS002556	EFO_0004611	low density lipoprotein cholesterol measurement	low density lipoprotein cholesterol measurement	EFO_0004611	Lipid or lipoprotein measurement	Metabolic	PGS002602	HP_0003124	Hypercholesterolemia	Hypercholesterolemia	HP_0003124	Other trait	Metabolic
0.774	2.43E-92	3.68E-89	PGS002556	EFO_0004611	low density lipoprotein cholesterol measurement	low density lipoprotein cholesterol measurement	EFO_0004611	Cardiovascular measurement	Cardiovascular	PGS002602	HP_0003124	Hypercholesterolemia	Hypercholesterolemia	HP_0003124	Other trait	Metabolic
0.774	2.49E-92	3.76E-89	PGS000301	EFO_0006335	systolic blood pressure	systolic blood pressure	EFO_0006335	Other measurement	Cardiovascular	PGS002258	EFO_0006336	diastolic blood pressure	diastolic blood pressure	EFO_0006336	Other measurement	Cardiovascular
0.7739	2.59E-92	3.91E-89	PGS003143	EFO_0004574	total cholesterol measurement	total cholesterol measurement	EFO_0004574	Lipid or lipoprotein measurement	Metabolic	PGS003034	EFO_0004611	low density lipoprotein cholesterol measurement	measurement	EFO_0004611	Lipid or lipoprotein measurement	Metabolic
0.7739	2.59E-92	3.91E-89	PGS003143	EFO_0004574	total cholesterol measurement	total cholesterol measurement	EFO_0004574	Lipid or lipoprotein measurement	Metabolic	PGS003034	EFO_0004611	low density lipoprotein cholesterol measurement	measurement	EFO_0004611	Cardiovascular measurement	Cardiovascular
-0.7739	2.66E-92	4.01E-89	PGS003223	EFO_0005208	glomerular filtration rate	glomerular filtration rate	EFO_0005208	Other measurement	Genitourinary	PGS002237	EFO_0003884	chronic kidney disease	chronic kidney disease	EFO_0003884	Other disease	Metabolic

0.7738	2.85E-92	4.31E-89	PGS001990	EFO_0004526	mean corpuscular volume	mean corpuscular volume	EFO_0004526	Hematological measurement	Hematological	PGS000099	EFO_0004527	mean corpuscular hemoglobin	mean corpuscular hemoglobin	EFO_0004527	Hematological measurement	Hematological
0.7738	2.95E-92	4.45E-89	PGS000477	EFO_0000305	breast carcinoma	breast carcinoma	EFO_0000305	Cancer	Neoplasms	PGS000514	EFO_0000305	breast carcinoma	breast carcinoma	EFO_0000305	Cancer	Neoplasms
0.7737	3.00E-92	4.53E-89	PGS002550	EFO_0004541	HbA1c measurement	HbA1c measurement	EFO_0004541	Hematological measurement	Hematological	PGS002592	EFO_0001379	endocrine system disease|diabetes mellitus	endocrine system disease	EFO_0001379	Other disease	Metabolic
0.7737	3.04E-92	4.58E-89	PGS000500	EFO_0000305	breast carcinoma	breast carcinoma	EFO_0000305	Cancer	Neoplasms	PGS000527	EFO_0000305	breast carcinoma	breast carcinoma	EFO_0000305	Cancer	Neoplasms
0.7737	3.15E-92	4.76E-89	PGS003290	EFO_0004617	cystatin C measurement|glomerular filtration rate	cystatin C measurement	EFO_0004617	Other measurement	Metabolic	PGS003306	EFO_0004617	cystatin C measurement|glomerular filtration rate	cystatin C measurement	EFO_0004617	Other measurement	Metabolic
0.7736	3.29E-92	4.97E-89	PGS002339	EFO_0004527	mean corpuscular hemoglobin	mean corpuscular hemoglobin	EFO_0004527	Hematological measurement	Hematological	PGS001220	EFO_0004526	mean corpuscular volume	mean corpuscular volume	EFO_0004526	Hematological measurement	Hematological
0.7736	3.34E-92	5.03E-89	PGS002630	EFO_0004340	body mass index	body mass index	EFO_0004340	Body measurement	Body measurement	PGS002859	EFO_0004340	body mass index	body mass index	EFO_0004340	Body measurement	Body measurement
0.7736	3.53E-92	5.32E-89	PGS000014	MONDO_0005148	type 2 diabetes mellitus	type 2 diabetes mellitus	MONDO_0005148	Digestive system disorder	Gastrointestinal	PGS003104	MONDO_0005148	type 2 diabetes mellitus	type 2 diabetes mellitus	MONDO_0005148	Metabolic disorder	Metabolic
0.7736	3.53E-92	5.32E-89	PGS000014	MONDO_0005148	type 2 diabetes mellitus	type 2 diabetes mellitus	MONDO_0005148	Metabolic disorder	Metabolic	PGS003104	MONDO_0005148	type 2 diabetes mellitus	type 2 diabetes mellitus	MONDO_0005148	Digestive system disorder	Gastrointestinal
0.7736	3.53E-92	5.32E-89	PGS000014	MONDO_0005148	type 2 diabetes mellitus	type 2 diabetes mellitus	MONDO_0005148	Metabolic disorder	Metabolic	PGS003104	MONDO_0005148	type 2 diabetes mellitus	type 2 diabetes mellitus	MONDO_0005148	Metabolic disorder	Metabolic
0.7736	3.53E-92	5.32E-89	PGS000014	MONDO_0005148	type 2 diabetes mellitus	type 2 diabetes mellitus	MONDO_0005148	Digestive system disorder	Gastrointestinal	PGS003104	MONDO_0005148	type 2 diabetes mellitus	type 2 diabetes mellitus	MONDO_0005148	Digestive system disorder	Gastrointestinal
0.7735	3.62E-92	5.44E-89	PGS001169	EFO_0004995	lean body mass	lean body mass	EFO_0004995	Other measurement	Skeletal	PGS002168	EFO_0004995	lean body mass	lean body mass	EFO_0004995	Other measurement	Skeletal
0.7734	3.94E-92	5.93E-89	PGS000534	EFO_0000305	breast carcinoma	breast carcinoma	EFO_0000305	Cancer	Neoplasms	PGS000498	EFO_0000305	breast carcinoma	breast carcinoma	EFO_0000305	Cancer	Neoplasms
0.7734	3.94E-92	5.93E-89	PGS000533	EFO_0000305	breast carcinoma	breast carcinoma	EFO_0000305	Cancer	Neoplasms	PGS000497	EFO_0000305	breast carcinoma	breast carcinoma	EFO_0000305	Cancer	Neoplasms
0.7734	3.94E-92	5.93E-89	PGS000533	EFO_0000305	breast carcinoma	breast carcinoma	EFO_0000305	Cancer	Neoplasms	PGS000498	EFO_0000305	breast carcinoma	breast carcinoma	EFO_0000305	Cancer	Neoplasms
0.7734	3.94E-92	5.93E-89	PGS000534	EFO_0000305	breast carcinoma	breast carcinoma	EFO_0000305	Cancer	Neoplasms	PGS000497	EFO_0000305	breast carcinoma	breast carcinoma	EFO_0000305	Cancer	Neoplasms
-0.7734	3.98E-92	5.99E-89	PGS003303	EFO_0004617	cystatin C measurement|glomerular filtration rate	cystatin C measurement	EFO_0004617	Other measurement	Metabolic	PGS003308	EFO_0004617	cystatin C measurement|glomerular filtration rate	cystatin C measurement	EFO_0004617	Other measurement	Metabolic
0.7733	4.30E-92	6.46E-89	PGS000538	EFO_0000305	breast carcinoma	breast carcinoma	EFO_0000305	Cancer	Neoplasms	PGS000540	EFO_0000305	breast carcinoma	breast carcinoma	EFO_0000305	Cancer	Neoplasms
0.7733	4.35E-92	6.53E-89	PGS002991	EFO_0004339	body height	body height	EFO_0004339	Body measurement	Body measurement	PGS002983	EFO_0004339	body height	body height	EFO_0004339	Body measurement	Body measurement
0.7733	4.42E-92	6.64E-89	PGS002220	EFO_0007822	hair colour measurement	hair colour measurement	EFO_0007822	Other measurement	Dermatological	PGS001098	EFO_0007822	hair colour measurement	hair colour measurement	EFO_0007822	Other measurement	Dermatological
0.7732	4.67E-92	7.02E-89	PGS001968	EFO_0005091	monocyte count	monocyte count	EFO_0005091	Hematological measurement	Hematological	PGS001163	EFO_0005091	monocyte count	monocyte count	EFO_0005091	Hematological measurement	Hematological
0.7732	4.69E-92	7.04E-89	PGS002078	MONDO_0001592	prolapse of female genital organ	prolapse of female genital organ	MONDO_0001592	Other disease	Others	PGS001867	MONDO_0001592	prolapse of female genital organ	prolapse of female genital organ	MONDO_0001592	Other disease	Others
0.7732	4.83E-92	7.25E-89	PGS002313	EFO_0004340	body mass index	body mass index	EFO_0004340	Body measurement	Body measurement	PGS002201	EFO_0004342	waist circumference	waist circumference	EFO_0004342	Body measurement	Body measurement
0.7732	4.94E-92	7.42E-89	PGS002964	EFO_0004339	body height	body height	EFO_0004339	Body measurement	Body measurement	PGS002965	EFO_0004339	body height	body height	EFO_0004339	Body measurement	Body measurement
-0.7732	5.00E-92	7.49E-89	PGS001994	HP_0000545	Myopia	Myopia	HP_0000545	Other trait	Ophthalmological	PGS001890	EFO_0008385	visual acuity measurement	visual acuity measurement	EFO_0008385	Other measurement	Ophthalmological
0.7731	5.36E-92	8.04E-89	PGS002491	EFO_0000400	diabetes mellitus	diabetes mellitus	EFO_0000400	Metabolic disorder	Metabolic	PGS002400	EFO_0004468	glucose measurement	glucose measurement	EFO_0004468	Other measurement	Metabolic
0.7731	5.36E-92	8.04E-89	PGS002491	EFO_0000400	diabetes mellitus	diabetes mellitus	EFO_0000400	Digestive system disorder	Gastrointestinal	PGS002400	EFO_0004468	glucose measurement	glucose measurement	EFO_0004468	Other measurement	Metabolic
0.7731	5.50E-92	8.24E-89	PGS002224	EFO_0011011	sitting height measurement	sitting height measurement	EFO_0011011	Other measurement	Skeletal	PGS002332	EFO_0004339	body height	body height	EFO_0004339	Body measurement	Body measurement
0.773	5.54E-92	8.30E-89	PGS003152	EFO_0004530	triglyceride measurement	triglyceride measurement	EFO_0004530	Lipid or lipoprotein measurement	Metabolic	PGS003151	EFO_0004530	triglyceride measurement	triglyceride measurement	EFO_0004530	Lipid or lipoprotein measurement	Metabolic
0.773	5.66E-92	8.48E-89	PGS001488	EFO_0006930	brain volume measurement|neuroimaging measurement	brain volume measurement	EFO_0006930	Other measurement	Neurological	PGS001451	EFO_0006930	measurement	brain volume measurement	EFO_0006930	Other measurement	Neurological
0.773	5.94E-92	8.90E-89	PGS001736	EFO_0006930	brain volume measurement|neuroimaging measurement	brain volume measurement	EFO_0006930	Other measurement	Neurological	PGS001721	EFO_0006930	measurement	brain volume measurement	EFO_0006930	Other measurement	Neurological
0.7729	6.18E-92	9.25E-89	PGS002630	EFO_0004340	body mass index	body mass index	EFO_0004340	Body measurement	Body measurement	PGS002849	EFO_0004340	body mass index	body mass index	EFO_0004340	Body measurement	Body measurement
0.7729	6.39E-92	9.56E-89	PGS000472	EFO_0000305	breast carcinoma	breast carcinoma	EFO_0000305	Cancer	Neoplasms	PGS001337	EFO_0005606	family history of breast cancer	family history of breast cancer	EFO_0005606	Other trait	Neoplasms
0.7729	6.44E-92	9.64E-89	PGS000008	EFO_1000649	estrogen-receptor positive breast cancer	estrogen-receptor positive breast cancer	EFO_1000649	Cancer	Neoplasms	PGS000500	EFO_0000305	breast carcinoma	breast carcinoma	EFO_0000305	Cancer	Neoplasms
0.7727	7.34E-92	1.10E-88	PGS003290	EFO_0004617	cystatin C measurement|glomerular filtration rate	cystatin C measurement	EFO_0004617	Other measurement	Metabolic	PGS003305	EFO_0004617	cystatin C measurement|glomerular filtration rate	cystatin C measurement	EFO_0004617	Other measurement	Metabolic
0.7727	7.61E-92	1.14E-88	PGS002632	EFO_0003923	bone density	bone density	EFO_0003923	Other measurement	Skeletal	PGS002174	EFO_0007772	measurement	measurement	EFO_0007772	Other measurement	Skeletal
0.7727	7.65E-92	1.14E-88	PGS002971	EFO_0004339	body height	body height	EFO_0004339	Body measurement	Body measurement	PGS002968	EFO_0004339	body height	body height	EFO_0004339	Body measurement	Body measurement
0.7726	8.35E-92	1.25E-88	PGS002550	EFO_0004541	HbA1c measurement	HbA1c measurement	EFO_0004541	Hematological measurement	Hematological	PGS002393	EFO_0000400	diabetes mellitus	diabetes mellitus	EFO_0000400	Digestive system disorder	Gastrointestinal
0.7726	8.35E-92	1.25E-88	PGS002550	EFO_0004541	HbA1c measurement	HbA1c measurement	EFO_0004541	Hematological measurement	Hematological	PGS002393	EFO_0000400	diabetes mellitus	diabetes mellitus	EFO_0000400	Metabolic disorder	Metabolic
0.7725	8.59E-92	1.28E-88	PGS002352	EFO_0004574	total cholesterol measurement	total cholesterol measurement	EFO_0004574	Lipid or lipoprotein measurement	Metabolic	PGS001889	EFO_0004615	apolipoprotein B measurement	apolipoprotein B measurement	EFO_0004615	Lipid or lipoprotein measurement	Metabolic
0.7725	8.70E-92	1.30E-88	PGS003198	EFO_0004631	vitamin D measurement	vitamin D measurement	EFO_0004631	Lipid or lipoprotein measurement	Metabolic	PGS000702	EFO_0004631	vitamin D measurement	vitamin D measurement	EFO_0004631	Lipid or lipoprotein measurement	Metabolic
0.7725	8.86E-92	1.32E-88	PGS001997	EFO_0007990	neutrophil percentage of leukocytes	neutrophil percentage of leukocytes	EFO_0007990	Hematological measurement	Hematological	PGS000106	EFO_0007990	neutrophil percentage of leukocytes	neutrophil percentage of leukocytes	EFO_0007990	Hematological measurement	Hematological
0.7724	9.37E-92	1.40E-88	PGS003280	EFO_0004617	cystatin C measurement	cystatin C measurement	EFO_0004617	Other measurement	Metabolic	PGS003286	EFO_0004617	cystatin C measurement	cystatin C measurement	EFO_0004617	Other measurement	Metabolic
0.7724	9.57E-92	1.43E-88	PGS001489	EFO_0006930	brain volume measurement|neuroimaging measurement	brain volume measurement	EFO_0006930	Other measurement	Neurological	PGS001450	EFO_0006930	measurement	brain volume measurement	EFO_0006930	Other measurement	Neurological
0.7724	9.84E-92	1.47E-88	PGS002202	EFO_0009805	whole body water mass	whole body water mass	EFO_0009805	Other measurement	Body measurement	PGS001002	EFO_0009805	whole body water mass	whole body water mass	EFO_0009805	Other measurement	Body measurement
0.7722	1.15E-91	1.71E-88	PGS002628	MONDO_0004979	asthma	asthma	MONDO_0004979	Other disease	Respiratory	PGS002311	MONDO_0004979	asthma	asthma	MONDO_0004979	Other disease	Respiratory
0.7722	1.16E-91	1.73E-88	PGS000006	EFO_1000650	estrogen-receptor negative breast cancer	estrogen-receptor negative breast cancer	EFO_1000650	Cancer	Neoplasms	PGS000004	EFO_0000305	breast carcinoma	breast carcinoma	EFO_0000305	Cancer	Neoplasms
0.7721	1.22E-91	1.82E-88	PGS001168	EFO_0004995	lean body mass	lean body mass	EFO_0004995	Other measurement	Skeletal	PGS002202	EFO_0009805	whole body water mass	whole body water mass	EFO_0009805	Other measurement	Body measurement
0.7721	1.24E-91	1.84E-88	PGS002411	EFO_0004527	mean corpuscular hemoglobin	mean corpuscular hemoglobin	EFO_0004527	Hematological measurement	Hematological	PGS001989	EFO_0004527	mean corpuscular hemoglobin	mean corpuscular hemoglobin	EFO_0004527	Hematological measurement	Hematological
0.772	1.33E-91	1.98E-88	PGS000110	EFO_0004305	erythrocyte count	erythrocyte count	EFO_0004305	Hematological measurement	Hematological	PGS001909	EFO_0004305	erythrocyte count	erythrocyte count	EFO_0004305	Hematological measurement	Hematological
0.7719	1.45E-91	2.16E-88	PGS000642	EFO_0005952	non-Hodgkins lymphoma	non-Hodgkins lymphoma	EFO_0005952	Cancer	Neoplasms	PGS000791	EFO_0005952	non-Hodgkins lymphoma	non-Hodgkins lymphoma	EFO_0005952	Cancer	Neoplasms
0.7718	1.59E-91	2.36E-88	PGS003074	EFO_0006335	systolic blood pressure	systolic blood pressure	EFO_0006335	Other measurement	Cardiovascular	PGS002228	EFO_0006335	systolic blood pressure	systolic blood pressure	EFO_0006335	Other measurement	Cardiovascular
0.7718	1.64E-91	2.44E-88	PGS001228	EFO_0004340	body mass index	body mass index	EFO_0004340	Body measurement	Body measurement	PGS001162	EFO_0005093	hip circumference	hip circumference	EFO_0005093	Body measurement	Body measurement
0.7717	1.74E-91	2.59E-88	PGS002187	EFO_0004833	neutrophil count	neutrophil count	EFO_0004833	Hematological measurement	Hematological	PGS002357	EFO_0004308	leukocyte count	leukocyte count	EFO_0004308	Hematological measurement	Hematological
0.7717	1.79E-91	2.66E-88	PGS003316	EFO_0004617	cystatin C measurement|glomerular filtration rate	cystatin C measurement	EFO_0004617	Other measurement	Metabolic	PGS003310	EFO_0004617	cystatin C measurement|glomerular filtration rate	cystatin C measurement	EFO_0004617	Other measurement	Metabolic
0.7717	1.79E-91	2.66E-88	PGS003315	EFO_0004617	cystatin C measurement|glomerular filtration rate	cystatin C measurement	EFO_0004617	Other measurement	Metabolic	PGS003310	EFO_0004617	cystatin C measurement|glomerular filtration rate	cystatin C measurement	EFO_0004617	Other measurement	Metabolic
0.7717	1.84E-91	2.74E-88	PGS002524	MONDO_0005148	type 2 diabetes mellitus	type 2 diabetes mellitus	MONDO_0005148	Metabolic disorder	Metabolic	PGS002592	EFO_0001379	endocrine system disease|diabetes mellitus	endocrine system disease	EFO_0001379	Other disease	Metabolic
0.7717	1.84E-91	2.74E-88	PGS002524	MONDO_0005148	type 2 diabetes mellitus	type 2 diabetes mellitus	MONDO_0005148	Digestive system disorder	Gastrointestinal	PGS002592	EFO_0001379	endocrine system disease|diabetes mellitus	endocrine system disease	EFO_0001379	Other disease	Metabolic
0.7716	1.90E-91	2.82E-88	PGS001996	EFO_0007660	neuroticism measurement	neuroticism measurement	EFO_0007660	Other measurement	Psychiatric	PGS002342	EFO_0007660	neuroticism measurement	neuroticism measurement	EFO_0007660	Other measurement	Psychiatric
0.7716	1.92E-91	2.86E-88	PGS002059	EFO_0004264	vascular disease	vascular disease	EFO_0004264	Cardiovascular disease	Cardiovascular	PGS001847	EFO_0004264	vascular disease	vascular disease	EFO_0004264	Cardiovascular disease	Cardiovascular
0.7716	2.02E-91	2.99E-88	PGS001029	EFO_0009464	corneal disease	corneal disease	EFO_0009464	Other disease	Ophthalmological	PGS001835	MONDO_0018102	corneal dystrophy	corneal dystrophy	MONDO_0018102	Other disease	Ophthalmological
0.7715	2.17E-91	3.22E-88	PGS000510	EFO_0000305	breast carcinoma	breast carcinoma	EFO_0000305	Cancer	Neoplasms	PGS000501	EFO_0000305	breast carcinoma	breast carcinoma	EFO_0000305	Cancer	Neoplasms
0.7714	2.32E-91	3.45E-88	PGS000297	EFO_0004339	body height	body height	EFO_0004339	Body measurement	Body measurement	PGS002973	EFO_0004339	body height	body height	EFO_0004339	Body measurement	Body measurement
0.7714	2.35E-91	3.48E-88	PGS000110	EFO_0004305	erythrocyte count	erythrocyte count	EFO_0004305	Hematological measurement	Hematological	PGS002662	EFO_0004305	erythrocyte count	erythrocyte count	EFO_0004305	Hematological measurement	Hematological
0.7714	2.42E-91	3.59E-88	PGS000751	EFO_0001663	prostate carcinoma	prostate carcinoma	EFO_0001663	Cancer	Neoplasms	PGS000742	EFO_0001663	prostate carcinoma	prostate carcinoma	EFO_0001663	Cancer	Neoplasms
0.7713	2.43E-91	3.61E-88	PGS001265	EFO_0003907	deep vein thrombosis	deep vein thrombosis	EFO_0003907	Other trait	Cardiovascular	PGS000961	EFO_1001395	Phlebitis|Thrombophlebitis	Phlebitis	EFO_1001395	Cardiovascular disease	Cardiovascular
0.7712	2.74E-91	4.06E-88	PGS001478	EFO_0006930	brain volume measurement|neuroimaging measurement	brain volume measurement	EFO_0006930	Other measurement	Neurological	PGS001480	EFO_0006930	measurement	brain volume measurement	EFO_0006930	Other measurement	Neurological
0.7712	2.76E-91	4.08E-88	PGS000499	EFO_0000305	breast carcinoma	breast carcinoma	EFO_0000305	Cancer	Neoplasms	PGS000008	EFO_1000649	estrogen-receptor positive breast cancer	cancer	EFO_1000649	Cancer	Neoplasms
0.7712	2.85E-91	4.22E-88	PGS001460	EFO_0006930	brain volume measurement|neuroimaging measurement	brain volume measurement	EFO_0006930	Other measurement	Neurological	PGS001459	EFO_0006930	measurement	brain volume measurement	EFO_0006930	Other measurement	Neurological
0.7712	2.87E-91	4.24E-88	PGS001402	EFO_0009270	heel bone mineral density	heel bone mineral density	EFO_0009270	Other measurement	Skeletal	PGS002315	EFO_0003923	bone density	bone density	EFO_0003923	Other measurement	Skeletal
0.7711	3.01E-91	4.46E-88	PGS000528	EFO_0000305	breast carcinoma	breast carcinoma	EFO_0000305	Cancer	Neoplasms	PGS000499	EFO_0000305	breast carcinoma	breast carcinoma	EFO_0000305	Cancer	Neoplasms
0.7711	3.02E-91	4.46E-88	PGS003030	EFO_0004611	low density lipoprotein cholesterol measurement	low density lipoprotein cholesterol measurement	EFO_0004611	Lipid or lipoprotein measurement	Metabolic	PGS002556	EFO_0004611	low density lipoprotein cholesterol measurement	measurement	EFO_0004611	Lipid or lipoprotein measurement	Metabolic
0.7711	3.02E-91	4.46E-88	PGS003030	EFO_0004611	low density lipoprotein cholesterol measurement	low density lipoprotein cholesterol measurement	EFO_0004611	Cardiovascular measurement	Cardiovascular	PGS002556	EFO_0004611	low density lipoprotein cholesterol measurement	measurement	EFO_0004611	Cardiovascular measurement	Cardiovascular
0.7711	3.02E-91	4.46E-88	PGS003030	EFO_0004611	low density lipoprotein cholesterol measurement	low density lipoprotein cholesterol measurement	EFO_0004611	Cardiovascular measurement	Cardiovascular	PGS002556	EFO_0004611	low density lipoprotein cholesterol measurement	measurement	EFO_0004611	Lipid or lipoprotein measurement	Metabolic
0.7711	3.02E-91	4.46E-88	PGS003030	EFO_0004611	low density lipoprotein cholesterol measurement	low density lipoprotein cholesterol measurement	EFO_0004611	Lipid or lipoprotein measurement	Metabolic	PGS002556	EFO_0004611	low density lipoprotein cholesterol measurement	measurement	EFO_0004611	Cardiovascular measurement	Cardiovascular
-0.771	3.23E-91	4.78E-88	PGS001097	EFO_0007822	hair colour measurement	hair colour measurement	EFO_0007822	Other measurement	Dermatological	PGS001094	EFO_0007822	hair colour measurement	hair colour measurement	EFO_0007822	Other measurement	Dermatological
0.771	3.27E-91	4.83E-88	PGS001984	EFO_0009805	whole body water mass	whole body water mass	EFO_0009805	Other measurement	Body measurement	PGS001108	EFO_0007777	base metabolic rate measurement	base metabolic rate measurement	EFO_0007777	Other measurement	Metabolic
0.771	3.28E-91	4.85E-88	PGS002570	EFO_1000627	thyroid disease	thyroid disease	EFO_1000627	Other disease	Endocrine	PGS002580	EFO_0005140	autoimmune disease	autoimmune disease	EFO_0005140	Immune system disorder	Immunological
0.771	3.30E-91	4.88E-88	PGS000515	EFO_0000305	breast carcinoma	breast carcinoma	EFO_0000305	Cancer	Neoplasms	PGS000474	EFO_0000305	breast carcinoma	breast carcinoma	EFO_0000305	Cancer	Neoplasms
0.771	3.32E-91	4.91E-88	PGS001950	EFO_0004995	lean body mass	lean body mass	EFO_0004995	Other measurement	Skeletal	PGS001108	EFO_0007777	base metabolic rate measurement	base metabolic rate measurement	EFO_0007777	Other measurement	Metabolic
0.771	3.36E-91	4.96E-88	PGS001750	EFO_0006930	brain volume measurement|neuroimaging measurement	brain volume measurement	EFO_0006930	Other measurement	Neurological	PGS001752	EFO_0006930	measurement	brain volume measurement	EFO_0006930	Other measurement	Neurological
0.7709	3.45E-91	5.09E-88	PGS002474	EFO_0004530	triglyceride measurement	triglyceride measurement	EFO_0004530	Lipid or lipoprotein measurement	Metabolic	PGS002621	EFO_0004530	triglyceride measurement	triglyceride measurement	EFO_0004530	Lipid or lipoprotein measurement	Metabolic
0.7708	3.83E-91	5.65E-88	PGS001037	EFO_0009270	heel bone mineral density	heel bone mineral density	EFO_0009270	Other measurement	Skeletal	PGS002315	EFO_0003923	bone density	bone density	EFO_0003923	Other measurement	Skeletal
0.7708	3.85E-91	5.68E-88	PGS000952	EFO_0004514	bone quantitative ultrasound measurement	bone quantitative ultrasound measurement	EFO_0004514	Other measurement	Skeletal	PGS002315	EFO_0003923	bone density	bone density	EFO_0003923	Other measurement	Skeletal
0.7708	3.87E-91	5.71E-88	PGS003143	EFO_0004574	total cholesterol measurement	total cholesterol measurement	EFO_0004574	Lipid or lipoprotein measurement	Metabolic	PGS002108	EFO_0004574	total cholesterol measurement	total cholesterol measurement	EFO_0004574	Lipid or lipoprotein measurement	Metabolic
0.7708	3.89E-91	5.74E-88	PGS002207	EFO_0004526	mean corpuscular volume	mean corpuscular volume	EFO_0004526	Hematological measurement	Hematological	PGS000099	EFO_0004527	mean corpuscular hemoglobin	mean corpuscular hemoglobin	EFO_0004527	Hematological measurement	Hematological
0.7707	4.34E-91	6.40E-88	PGS000385	EFO_0002618	pancreatic carcinoma	pancreatic carcinoma	EFO_0002618	Digestive system disorder	Gastrointestinal	PGS000386	EFO_0002618	pancreatic carcinoma	pancreatic carcinoma	EFO_0002618	Digestive system disorder	Gastrointestinal
0.7707	4.34E-91	6.40E-88	PGS000385	EFO_0002618	pancreatic carcinoma	pancreatic carcinoma	EFO_0002618	Digestive system disorder	Gastrointestinal	PGS000386	EFO_0002618	pancreatic carcinoma	pancreatic carcinoma	EFO_0002618	Cancer	Neoplasms
0.7707	4.34E-91	6.40E-88	PGS000385	EFO_0002618	pancreatic carcinoma	pancreatic carcinoma	EFO_0002618	Cancer	Neoplasms	PGS000386	EFO_0002618	pancreatic carcinoma	pancreatic carcinoma	EFO_0002618	Cancer	Neoplasms
0.7707	4.34E-91	6.40E-88	PGS000385	EFO_0002618	pancreatic carcinoma	pancreatic carcinoma	EFO_0002618	Cancer	Neoplasms	PGS000386	EFO_0002618	pancreatic carcinoma	pancreatic carcinoma	EFO_0002618	Digestive system disorder	Gastrointestinal
-0.7707	4.34E-91	6.40E-88	PGS002768	EFO_0003882	osteoporosis	osteoporosis	EFO_0003882	Other disease	Skeletal	PGS001402	EFO_0009270	heel bone mineral density	heel bone mineral density	EFO_0009270	Other measurement	Skeletal
0.7707	4.43E-91	6.53E-88	PGS000539	EFO_0000305	breast carcinoma	breast carcinoma	EFO_0000305	Cancer	Neoplasms	PGS000504	EFO_0000305	breast carcinoma	breast carcinoma	EFO_0000305	Cancer	Neoplasms
0.7706	4.55E-91	6.70E-88	PGS000335	EFO_0000305	breast carcinoma	breast carcinoma	EFO_0000305	Cancer	Neoplasms	PGS000007	EFO_0000305	breast carcinoma	breast carcinoma	EFO_0000305	Cancer	Neoplasms
0.7706	4.65E-91	6.84E-88	PGS001160	EFO_0005106	body composition measurement	body composition measurement	EFO_0005106	Body measurement	Body measurement	PGS002202	EFO_0009805	whole body water mass	whole body water mass	EFO_0009805	Other measurement	Body measurement
0.7706	4.75E-91	6.99E-88	PGS001973	EFO_0004309	platelet count	platelet count	EFO_0004309	Hematological measurement	Hematological	PGS001970	EFO_0007985	platelet crit	platelet crit	EFO_0007985	Hematological measurement	Hematological
-0.7706	4.86E-91	7.14E-88	PGS001997	EFO_0007990	neutrophil percentage of leukocytes	neutrophil percentage of leukocytes	EFO_0007990	Hematological measurement	Hematological	PGS001414	EFO_0007993	lymphocyte percentage of leukocytes	lymphocyte percentage of leukocytes	EFO_0007993	Hematological measurement	Hematological
0.7705	5.03E-91	7.40E-88	PGS003143	EFO_0004574	total cholesterol measurement	total cholesterol measurement	EFO_0004574	Lipid or lipoprotein measurement	Metabolic	PGS000892	EFO_0004611	low density lipoprotein cholesterol measurement	measurement	EFO_0004611	Lipid or lipoprotein measurement	Metabolic
0.7705	5.03E-91	7.40E-88	PGS003143	EFO_0004574	total cholesterol measurement	total cholesterol measurement	EFO_0004574	Lipid or lipoprotein measurement	Metabolic	PGS000892	EFO_0004611	low density lipoprotein cholesterol measurement	measurement	EFO_0004611	Cardiovascular measurement	Cardiovascular
0.7705	5.05E-91	7.43E-88	PGS000508	EFO_0000305	breast carcinoma	breast carcinoma	EFO_0000305	Cancer	Neoplasms	PGS000008	EFO_1000649	estrogen-receptor positive breast cancer	cancer	EFO_1000649	Cancer	Neoplasms
0.7705	5.12E-91	7.53E-88	PGS002550	EFO_0004541	HbA1c measurement	HbA1c measurement	EFO_0004541	Hematological measurement	Hematological	PGS002426	MONDO_0005148	type 2 diabetes mellitus	type 2 diabetes mellitus	MONDO_0005148	Metabolic disorder	Metabolic
0.7705	5.12E-91	7.53E-88	PGS002550	EFO_0004541	HbA1c measurement	HbA1c measurement	EFO_0004541	Hematological measurement	Hematological	PGS002426	MONDO_0005148	type 2 diabetes mellitus	type 2 diabetes mellitus	MONDO_0005148	Digestive system disorder	Gastrointestinal
0.7705	5.23E-91	7.69E-88	PGS001943	EFO_0004340	body mass index	body mass index	EFO_0004340	Body measurement	Body measurement	PGS002855	EFO_0004340	body mass index	body mass index	EFO_0004340	Body measurement	Body measurement
0.7703	5.90E-91	8.66E-88	PGS003144	EFO_0004530	triglyceride measurement	triglyceride measurement	EFO_0004530	Lipid or lipoprotein measurement	Metabolic	PGS002197	EFO_0004530	triglyceride measurement	triglyceride measurement	EFO_0004530	Lipid or lipoprotein measurement	Metabolic

0.7703	6.22E-91	9.13E-88	PGS002971	EFO_0004339	body height	body height	EFO_0004339	Body measurement	Body measurement	PGS002973	EFO_0004339	body height	body height	EFO_0004339	Body measurement	Body measurement
0.7703	6.24E-91	9.16E-88	PGS000007	EFO_0000305	breast carcinoma	breast carcinoma	EFO_0000305	Cancer	Neoplasms	PGS000512	EFO_0000305	breast carcinoma	breast carcinoma	EFO_0000305	Cancer	Neoplasms
0.7702	6.61E-91	9.70E-88	PGS003199	EFO_0004631	vitamin D measurement	vitamin D measurement	EFO_0004631	Lipid or lipoprotein measurement	Metabolic	PGS003196	EFO_0004631	vitamin D measurement	vitamin D measurement	EFO_0004631	Lipid or lipoprotein measurement	Metabolic
0.7701	6.97E-91	1.02E-87	PGS003193	EFO_0004631	vitamin D measurement	vitamin D measurement	EFO_0004631	Lipid or lipoprotein measurement	Metabolic	PGS000702	EFO_0004631	vitamin D measurement	vitamin D measurement	EFO_0004631	Lipid or lipoprotein measurement	Metabolic
0.7701	7.03E-91	1.03E-87	PGS002672	EFO_0004631	vitamin D measurement	vitamin D measurement	EFO_0004631	Lipid or lipoprotein measurement	Metabolic	PGS003195	EFO_0004631	vitamin D measurement	vitamin D measurement	EFO_0004631	Lipid or lipoprotein measurement	Metabolic
0.7701	7.10E-91	1.04E-87	PGS003184	EFO_0004631	vitamin D measurement	vitamin D measurement	EFO_0004631	Lipid or lipoprotein measurement	Metabolic	PGS003196	EFO_0004631	vitamin D measurement	vitamin D measurement	EFO_0004631	Lipid or lipoprotein measurement	Metabolic
0.7701	7.36E-91	1.08E-87	PGS001489	EFO_0006930	brain volume measurement|neuroimaging measurement	brain volume measurement	EFO_0006930	Other measurement	Neurological	PGS001451	EFO_0006930	measurement	brain volume measurement	EFO_0006930	Other measurement	Neurological
0.77	7.76E-91	1.14E-87	PGS000814	EFO_0004611	low density lipoprotein cholesterol measurement	low density lipoprotein cholesterol measurement	EFO_0004611	Lipid or lipoprotein measurement	Metabolic	PGS003404	EFO_0004611	low density lipoprotein cholesterol measurement	measurement	EFO_0004611	Lipid or lipoprotein measurement	Metabolic
0.77	7.76E-91	1.14E-87	PGS000814	EFO_0004611	low density lipoprotein cholesterol measurement	low density lipoprotein cholesterol measurement	EFO_0004611	Cardiovascular measurement	Cardiovascular	PGS003404	EFO_0004611	low density lipoprotein cholesterol measurement	measurement	EFO_0004611	Lipid or lipoprotein measurement	Metabolic
0.77	7.76E-91	1.14E-87	PGS000814	EFO_0004611	low density lipoprotein cholesterol measurement	low density lipoprotein cholesterol measurement	EFO_0004611	Cardiovascular measurement	Cardiovascular	PGS003404	EFO_0004611	low density lipoprotein cholesterol measurement	measurement	EFO_0004611	Cardiovascular measurement	Cardiovascular
0.77	7.76E-91	1.14E-87	PGS000814	EFO_0004611	low density lipoprotein cholesterol measurement	low density lipoprotein cholesterol measurement	EFO_0004611	Lipid or lipoprotein measurement	Metabolic	PGS003404	EFO_0004611	low density lipoprotein cholesterol measurement	measurement	EFO_0004611	Cardiovascular measurement	Cardiovascular
0.77	7.98E-91	1.17E-87	PGS002970	EFO_0004339	body height	body height	EFO_0004339	Body measurement	Body measurement	PGS002969	EFO_0004339	body height	body height	EFO_0004339	Body measurement	Body measurement
0.77	7.98E-91	1.17E-87	PGS002177	EFO_0010700	reticulocyte measurement	reticulocyte measurement	EFO_0010700	Hematological measurement	Hematological	PGS001406	EFO_0010700	reticulocyte measurement	reticulocyte measurement	EFO_0010700	Hematological measurement	Hematological
0.7699	8.55E-91	1.25E-87	PGS001168	EFO_0004995	lean body mass	lean body mass	EFO_0004995	Other measurement	Skeletal	PGS002168	EFO_0004995	lean body mass	lean body mass	EFO_0004995	Other measurement	Skeletal
0.7697	1.03E-90	1.50E-87	PGS000575	EFO_0001663	prostate carcinoma	prostate carcinoma	EFO_0001663	Cancer	Neoplasms	PGS000571	EFO_0001663	prostate carcinoma	prostate carcinoma	EFO_0001663	Cancer	Neoplasms
0.7695	1.19E-90	1.75E-87	PGS001613	EFO_0005420	measurement	grey matter volume measurement	EFO_0005420	Other measurement	Neurological	PGS001593	EFO_0005420	measurement	grey matter volume measurement	EFO_0005420	Other measurement	Neurological
0.7695	1.25E-90	1.83E-87	PGS003071	EFO_0006335	systolic blood pressure	systolic blood pressure	EFO_0006335	Other measurement	Cardiovascular	PGS003075	EFO_0006335	systolic blood pressure	systolic blood pressure	EFO_0006335	Other measurement	Cardiovascular
0.7693	1.43E-90	2.09E-87	PGS001983	EFO_0004342	waist circumference	waist circumference	EFO_0004342	Body measurement	Body measurement	PGS002176	EFO_0005093	hip circumference	hip circumference	EFO_0005093	Body measurement	Body measurement
0.7693	1.46E-90	2.13E-87	PGS001979	EFO_0004530	triglyceride measurement	triglyceride measurement	EFO_0004530	Lipid or lipoprotein measurement	Metabolic	PGS003144	EFO_0004530	triglyceride measurement	triglyceride measurement	EFO_0004530	Lipid or lipoprotein measurement	Metabolic
0.7692	1.51E-90	2.21E-87	PGS001002	EFO_0009805	whole body water mass	whole body water mass	EFO_0009805	Other measurement	Body measurement	PGS002168	EFO_0004995	lean body mass	lean body mass	EFO_0004995	Other measurement	Skeletal
0.7692	1.54E-90	2.26E-87	PGS000001	EFO_0000305	breast carcinoma	breast carcinoma	EFO_0000305	Cancer	Neoplasms	PGS000773	EFO_0000305	breast carcinoma	breast carcinoma	EFO_0000305	Cancer	Neoplasms
0.7691	1.64E-90	2.40E-87	PGS002640	HP_0000964	Eczema	Eczema	HP_0000964	Other trait	Dermatological	PGS001284	MONDO_0005271	allergic disease	allergic disease	MONDO_0005271	Immune system disorder	Immunological
-0.7691	1.65E-90	2.41E-87	PGS000952	EFO_0004514	bone quantitative ultrasound measurement	bone quantitative ultrasound measurement	EFO_0004514	Other measurement	Skeletal	PGS002768	EFO_0003882	osteoporosis	osteoporosis	EFO_0003882	Other disease	Skeletal
-0.7691	1.65E-90	2.41E-87	PGS001037	EFO_0009270	heel bone mineral density	heel bone mineral density	EFO_0009270	Other measurement	Skeletal	PGS002768	EFO_0003882	osteoporosis	osteoporosis	EFO_0003882	Other disease	Skeletal
0.7691	1.71E-90	2.50E-87	PGS000433	MONDO_0002898	skin cancer	skin cancer	MONDO_0002898	Cancer	Neoplasms	PGS000437	MONDO_0002898	skin cancer	skin cancer	MONDO_0002898	Cancer	Neoplasms
0.7691	1.76E-90	2.57E-87	PGS001723	EFO_0006930	brain volume measurement|neuroimaging measurement	brain volume measurement	EFO_0006930	Other measurement	Neurological	PGS001721	EFO_0006930	measurement	brain volume measurement	EFO_0006930	Other measurement	Neurological
0.7688	2.11E-90	3.08E-87	PGS001120	EFO_0006941	grip strength measurement	grip strength measurement	EFO_0006941	Other measurement	Muscular	PGS001119	EFO_0006941	grip strength measurement	grip strength measurement	EFO_0006941	Other measurement	Muscular
0.7688	2.26E-90	3.29E-87	PGS002620	EFO_0004574	total cholesterol measurement	total cholesterol measurement	EFO_0004574	Lipid or lipoprotein measurement	Metabolic	PGS002602	HP_0003124	Hypercholesterolemia	Hypercholesterolemia	HP_0003124	Other trait	Metabolic
0.7688	2.27E-90	3.31E-87	PGS000002	EFO_1000649	estrogen-receptor positive breast cancer	estrogen-receptor positive breast cancer	EFO_1000649	Cancer	Neoplasms	PGS003398	EFO_0000305	breast carcinoma	breast carcinoma	EFO_0000305	Cancer	Neoplasms
0.7687	2.40E-90	3.49E-87	PGS001841	EFO_0003911	atrial flutter|atrial fibrillation	atrial flutter	EFO_0003911	Cardiovascular disease	Cardiovascular	PGS001263	EFO_0003911	atrial flutter	atrial flutter	EFO_0003911	Cardiovascular disease	Cardiovascular
0.7686	2.55E-90	3.71E-87	PGS001517	EFO_0007985	platelet crit	platelet crit	EFO_0007985	Hematological measurement	Hematological	PGS002188	EFO_0007985	platelet crit	platelet crit	EFO_0007985	Hematological measurement	Hematological
0.7686	2.65E-90	3.86E-87	PGS002934	EFO_0004468	glucose measurement	glucose measurement	EFO_0004468	Other measurement	Metabolic	PGS002935	EFO_0004468	glucose measurement	glucose measurement	EFO_0004468	Other measurement	Metabolic
0.7685	2.76E-90	4.01E-87	PGS002672	EFO_0004631	vitamin D measurement	vitamin D measurement	EFO_0004631	Lipid or lipoprotein measurement	Metabolic	PGS003184	EFO_0004631	vitamin D measurement	vitamin D measurement	EFO_0004631	Lipid or lipoprotein measurement	Metabolic
-0.7685	2.79E-90	4.06E-87	PGS000825	EFO_0004612	high density lipoprotein cholesterol measurement	high density lipoprotein cholesterol measurement	EFO_0004612	Lipid or lipoprotein measurement	Metabolic	PGS002597	EFO_0004612	high density lipoprotein cholesterol measurement	measurement	EFO_0004612	Lipid or lipoprotein measurement	Metabolic
0.7685	2.81E-90	4.09E-87	PGS002672	EFO_0004631	vitamin D measurement	vitamin D measurement	EFO_0004631	Lipid or lipoprotein measurement	Metabolic	PGS003199	EFO_0004631	vitamin D measurement	vitamin D measurement	EFO_0004631	Lipid or lipoprotein measurement	Metabolic
0.7685	2.82E-90	4.10E-87	PGS002524	MONDO_0005148	type 2 diabetes mellitus	type 2 diabetes mellitus	MONDO_0005148	Digestive system disorder	Gastrointestinal	PGS002622	MONDO_0005148	type 2 diabetes mellitus	type 2 diabetes mellitus	MONDO_0005148	Metabolic disorder	Metabolic
0.7685	2.82E-90	4.10E-87	PGS002524	MONDO_0005148	type 2 diabetes mellitus	type 2 diabetes mellitus	MONDO_0005148	Metabolic disorder	Metabolic	PGS002622	MONDO_0005148	type 2 diabetes mellitus	type 2 diabetes mellitus	MONDO_0005148	Digestive system disorder	Gastrointestinal
0.7685	2.82E-90	4.10E-87	PGS002524	MONDO_0005148	type 2 diabetes mellitus	type 2 diabetes mellitus	MONDO_0005148	Metabolic disorder	Metabolic	PGS002622	MONDO_0005148	type 2 diabetes mellitus	type 2 diabetes mellitus	MONDO_0005148	Metabolic disorder	Metabolic
0.7685	2.82E-90	4.10E-87	PGS002524	MONDO_0005148	type 2 diabetes mellitus	type 2 diabetes mellitus	MONDO_0005148	Digestive system disorder	Gastrointestinal	PGS002622	MONDO_0005148	type 2 diabetes mellitus	type 2 diabetes mellitus	MONDO_0005148	Digestive system disorder	Gastrointestinal
0.7685	2.87E-90	4.17E-87	PGS003189	EFO_0004631	vitamin D measurement	vitamin D measurement	EFO_0004631	Lipid or lipoprotein measurement	Metabolic	PGS003193	EFO_0004631	vitamin D measurement	vitamin D measurement	EFO_0004631	Lipid or lipoprotein measurement	Metabolic
0.7684	3.12E-90	4.54E-87	PGS000297	EFO_0004339	body height	body height	EFO_0004339	Body measurement	Body measurement	PGS002968	EFO_0004339	body height	body height	EFO_0004339	Body measurement	Body measurement
0.7683	3.27E-90	4.76E-87	PGS001900	EFO_0006336	diastolic blood pressure	diastolic blood pressure	EFO_0006336	Other measurement	Cardiovascular	PGS002898	EFO_0006336	diastolic blood pressure	diastolic blood pressure	EFO_0006336	Other measurement	Cardiovascular
0.7683	3.36E-90	4.87E-87	PGS000380	EFO_1001950	colon carcinoma	colon carcinoma	EFO_1001950	Cancer	Neoplasms	PGS000376	EFO_1001950	colon carcinoma	colon carcinoma	EFO_1001950	Digestive system disorder	Gastrointestinal
0.7683	3.36E-90	4.87E-87	PGS000380	EFO_1001950	colon carcinoma	colon carcinoma	EFO_1001950	Digestive system disorder	Gastrointestinal	PGS000376	EFO_1001950	colon carcinoma	colon carcinoma	EFO_1001950	Cancer	Neoplasms
0.7683	3.36E-90	4.87E-87	PGS000380	EFO_1001950	colon carcinoma	colon carcinoma	EFO_1001950	Digestive system disorder	Gastrointestinal	PGS000376	EFO_1001950	colon carcinoma	colon carcinoma	EFO_1001950	Digestive system disorder	Gastrointestinal
0.7683	3.36E-90	4.87E-87	PGS000380	EFO_1001950	colon carcinoma	colon carcinoma	EFO_1001950	Cancer	Neoplasms	PGS000376	EFO_1001950	colon carcinoma	colon carcinoma	EFO_1001950	Cancer	Neoplasms
0.7683	3.39E-90	4.91E-87	PGS002672	EFO_0004631	vitamin D measurement	vitamin D measurement	EFO_0004631	Lipid or lipoprotein measurement	Metabolic	PGS003190	EFO_0004631	vitamin D measurement	vitamin D measurement	EFO_0004631	Lipid or lipoprotein measurement	Metabolic
0.7683	3.41E-90	4.94E-87	PGS000506	EFO_0000305	breast carcinoma	breast carcinoma	EFO_0000305	Cancer	Neoplasms	PGS000873	EFO_0000305	breast carcinoma	breast carcinoma	EFO_0000305	Cancer	Neoplasms
0.7683	3.41E-90	4.94E-87	PGS000873	EFO_0000305	breast carcinoma	breast carcinoma	EFO_0000305	Cancer	Neoplasms	PGS000505	EFO_0000305	breast carcinoma	breast carcinoma	EFO_0000305	Cancer	Neoplasms
0.7683	3.43E-90	4.97E-87	PGS002540	EFO_0000400	diabetes mellitus	diabetes mellitus	EFO_0000400	Metabolic disorder	Metabolic	PGS002501	EFO_0004541	HbA1c measurement	HbA1c measurement	EFO_0004541	Hematological measurement	Hematological
0.7683	3.43E-90	4.97E-87	PGS002540	EFO_0000400	diabetes mellitus	diabetes mellitus	EFO_0000400	Digestive system disorder	Gastrointestinal	PGS002501	EFO_0004541	HbA1c measurement	HbA1c measurement	EFO_0004541	Hematological measurement	Hematological
0.7682	3.66E-90	5.31E-87	PGS001160	EFO_0005106	body composition measurement	body composition measurement	EFO_0005106	Body measurement	Body measurement	PGS002168	EFO_0004995	lean body mass	lean body mass	EFO_0004995	Other measurement	Skeletal
0.7682	3.73E-90	5.40E-87	PGS002993	EFO_0004339	body height	body height	EFO_0004339	Body measurement	Body measurement	PGS002991	EFO_0004339	body height	body height	EFO_0004339	Body measurement	Body measurement
0.7681	3.82E-90	5.54E-87	PGS002963	EFO_0004612	high density lipoprotein cholesterol measurement	high density lipoprotein cholesterol measurement	EFO_0004612	Lipid or lipoprotein measurement	Metabolic	PGS001954	EFO_0004612	high density lipoprotein cholesterol measurement	measurement	EFO_0004612	Lipid or lipoprotein measurement	Metabolic
0.7681	3.93E-90	5.70E-87	PGS000366	MONDO_0007576	esophageal cancer	esophageal cancer	MONDO_0007576	Digestive system disorder	Gastrointestinal	PGS000363	MONDO_0007576	esophageal cancer	esophageal cancer	MONDO_0007576	Digestive system disorder	Gastrointestinal
0.7681	3.93E-90	5.70E-87	PGS000366	MONDO_0007576	esophageal cancer	esophageal cancer	MONDO_0007576	Cancer	Neoplasms	PGS000363	MONDO_0007576	esophageal cancer	esophageal cancer	MONDO_0007576	Digestive system disorder	Gastrointestinal
0.7681	3.93E-90	5.70E-87	PGS000366	MONDO_0007576	esophageal cancer	esophageal cancer	MONDO_0007576	Digestive system disorder	Gastrointestinal	PGS000363	MONDO_0007576	esophageal cancer	esophageal cancer	MONDO_0007576	Cancer	Neoplasms
0.7681	3.93E-90	5.70E-87	PGS000366	MONDO_0007576	esophageal cancer	esophageal cancer	MONDO_0007576	Cancer	Neoplasms	PGS000363	MONDO_0007576	esophageal cancer	esophageal cancer	MONDO_0007576	Cancer	Neoplasms
0.7681	4.01E-90	5.81E-87	PGS002844	EFO_0004340	body mass index	body mass index	EFO_0004340	Body measurement	Body measurement	PGS002630	EFO_0004340	body mass index	body mass index	EFO_0004340	Body measurement	Body measurement
0.7681	4.10E-90	5.93E-87	PGS002540	EFO_0000400	diabetes mellitus	diabetes mellitus	EFO_0000400	Metabolic disorder	Metabolic	PGS002524	MONDO_0005148	type 2 diabetes mellitus	type 2 diabetes mellitus	MONDO_0005148	Metabolic disorder	Metabolic
0.7681	4.10E-90	5.93E-87	PGS002540	EFO_0000400	diabetes mellitus	diabetes mellitus	EFO_0000400	Metabolic disorder	Metabolic	PGS002524	MONDO_0005148	type 2 diabetes mellitus	type 2 diabetes mellitus	MONDO_0005148	Digestive system disorder	Gastrointestinal
0.7681	4.10E-90	5.93E-87	PGS002540	EFO_0000400	diabetes mellitus	diabetes mellitus	EFO_0000400	Digestive system disorder	Gastrointestinal	PGS002524	MONDO_0005148	type 2 diabetes mellitus	type 2 diabetes mellitus	MONDO_0005148	Metabolic disorder	Metabolic
0.7681	4.10E-90	5.93E-87	PGS002540	EFO_0000400	diabetes mellitus	diabetes mellitus	EFO_0000400	Digestive system disorder	Gastrointestinal	PGS002524	MONDO_0005148	type 2 diabetes mellitus	type 2 diabetes mellitus	MONDO_0005148	Digestive system disorder	Gastrointestinal
0.7681	4.11E-90	5.95E-87	PGS002009	EFO_0006335	systolic blood pressure	systolic blood pressure	EFO_0006335	Other measurement	Cardiovascular	PGS002349	EFO_0006335	systolic blood pressure	systolic blood pressure	EFO_0006335	Other measurement	Cardiovascular
0.768	4.19E-90	6.06E-87	PGS000574	EFO_0001663	prostate carcinoma	prostate carcinoma	EFO_0001663	Cancer	Neoplasms	PGS000590	EFO_0001663	prostate carcinoma	prostate carcinoma	EFO_0001663	Cancer	Neoplasms
0.768	4.19E-90	6.06E-87	PGS000847	EFO_0004530	triglyceride measurement	triglyceride measurement	EFO_0004530	Lipid or lipoprotein measurement	Metabolic	PGS002425	EFO_0004530	triglyceride measurement	triglyceride measurement	EFO_0004530	Lipid or lipoprotein measurement	Metabolic
0.768	4.22E-90	6.11E-87	PGS001230	EFO_0004338	body weight	body weight	EFO_0004338	Body measurement	Body measurement	PGS001228	EFO_0004340	body mass index	body mass index	EFO_0004340	Body measurement	Body measurement
0.768	4.52E-90	6.54E-87	PGS000113	EFO_0004308	leukocyte count	leukocyte count	EFO_0004308	Hematological measurement	Hematological	PGS000105	EFO_0004833	neutrophil count	neutrophil count	EFO_0004833	Hematological measurement	Hematological
0.7679	4.65E-90	6.72E-87	PGS002169	EFO_0005409	fat body mass	fat body mass	EFO_0005409	Other measurement	Body measurement	PGS002313	EFO_0004340	body mass index	body mass index	EFO_0004340	Body measurement	Body measurement
0.7679	4.75E-90	6.86E-87	PGS002554	EFO_0000537	hypertension	hypertension	EFO_0000537	Cardiovascular disease	Cardiovascular	PGS002617	EFO_0006335	systolic blood pressure	systolic blood pressure	EFO_0006335	Other measurement	Cardiovascular
0.7679	4.75E-90	6.86E-87	PGS000841	EFO_0004340	body mass index	body mass index	EFO_0004340	Body measurement	Body measurement	PGS000034	EFO_0004340	body mass index	body mass index	EFO_0004340	Body measurement	Body measurement
0.7678	5.08E-90	7.34E-87	PGS003053	EFO_0006527	smoking status measurement	smoking status measurement	EFO_0006527	Other measurement	Psychiatric	PGS003049	EFO_0006527	smoking status measurement	smoking status measurement	EFO_0006527	Other measurement	Psychiatric
0.7677	5.45E-90	7.87E-87	PGS003074	EFO_0006335	systolic blood pressure	systolic blood pressure	EFO_0006335	Other measurement	Cardiovascular	PGS002807	EFO_0006335	systolic blood pressure	systolic blood pressure	EFO_0006335	Other measurement	Cardiovascular
0.7677	5.80E-90	8.37E-87	PGS002547	EFO_0004468	glucose measurement	glucose measurement	EFO_0004468	Other measurement	Metabolic	PGS002491	EFO_0000400	diabetes mellitus	diabetes mellitus	EFO_0000400	Digestive system disorder	Gastrointestinal
0.7677	5.80E-90	8.37E-87	PGS002547	EFO_0004468	glucose measurement	glucose measurement	EFO_0004468	Other measurement	Metabolic	PGS002491	EFO_0000400	diabetes mellitus	diabetes mellitus	EFO_0000400	Metabolic disorder	Metabolic
0.7676	5.97E-90	8.62E-87	PGS001926	EFO_0004509	hemoglobin measurement	hemoglobin measurement	EFO_0004509	Hematological measurement	Hematological	PGS001400	EFO_0004509	hemoglobin measurement	hemoglobin measurement	EFO_0004509	Hematological measurement	Hematological
-0.7676	6.06E-90	8.74E-87	PGS001247	EFO_0004279	suntan	suntan	EFO_0004279	Other trait	Dermatological	PGS001245	EFO_0004279	suntan	suntan	EFO_0004279	Other trait	Dermatological
0.7676	6.21E-90	8.95E-87	PGS001917	EFO_0007800	body fat percentage	body fat percentage	EFO_0007800	Body measurement	Body measurement	PGS002161	EFO_0004340	body mass index	body mass index	EFO_0004340	Body measurement	Body measurement
0.7676	6.35E-90	9.16E-87	PGS000539	EFO_0000305	breast carcinoma	breast carcinoma	EFO_0000305	Cancer	Neoplasms	PGS000503	EFO_0000305	breast carcinoma	breast carcinoma	EFO_0000305	Cancer	Neoplasms
0.7675	6.69E-90	9.64E-87	PGS002550	EFO_0004541	HbA1c measurement	HbA1c measurement	EFO_0004541	Hematological measurement	Hematological	PGS002501	EFO_0004541	HbA1c measurement	HbA1c measurement	EFO_0004541	Hematological measurement	Hematological
0.7675	6.71E-90	9.66E-87	PGS003184	EFO_0004631	vitamin D measurement	vitamin D measurement	EFO_0004631	Lipid or lipoprotein measurement	Metabolic	PGS003191	EFO_0004631	vitamin D measurement	vitamin D measurement	EFO_0004631	Lipid or lipoprotein measurement	Metabolic
0.7674	6.95E-90	1.00E-86	PGS003199	EFO_0004631	vitamin D measurement	vitamin D measurement	EFO_0004631	Lipid or lipoprotein measurement	Metabolic	PGS003191	EFO_0004631	vitamin D measurement	vitamin D measurement	EFO_0004631	Lipid or lipoprotein measurement	Metabolic
0.7674	7.05E-90	1.02E-86	PGS002224	EFO_0011011	sitting height measurement	sitting height measurement	EFO_0011011	Other measurement	Skeletal	PGS002968	EFO_0004339	body height	body height	EFO_0004339	Body measurement	Body measurement
0.7674	7.52E-90	1.08E-86	PGS001809	EFO_0002892	thyroid carcinoma	thyroid carcinoma	EFO_0002892	Cancer	Neoplasms	PGS000208	EFO_0002892	thyroid carcinoma	thyroid carcinoma	EFO_0002892	Cancer	Neoplasms
0.7673	7.68E-90	1.10E-86	PGS000030	EFO_0001663	prostate carcinoma	prostate carcinoma	EFO_0001663	Cancer	Neoplasms	PGS000049	EFO_0001663	prostate carcinoma	prostate carcinoma	EFO_0001663	Cancer	Neoplasms
0.7673	7.85E-90	1.13E-86	PGS002423	EFO_1000627	thyroid disease	thyroid disease	EFO_1000627	Other disease	Endocrine	PGS002472	EFO_1000627	thyroid disease	thyroid disease	EFO_1000627	Other disease	Endocrine
0.7673	8.00E-90	1.15E-86	PGS003255	EFO_0010472	creatine measurement|glomerular filtration rate	creatine measurement	EFO_0010472	Other measurement	Metabolic	PGS003269	EFO_0010472	creatine measurement|glomerular filtration rate	creatine measurement	EFO_0010472	Other measurement	Metabolic
0.7672	8.26E-90	1.19E-86	PGS001778	EFO_0000305	breast carcinoma	breast carcinoma	EFO_0000305	Cancer	Neoplasms	PGS001337	EFO_0005606	family history of breast cancer	family history of breast cancer	EFO_0005606	Other trait	Neoplasms
0.7672	8.57E-90	1.23E-86	PGS001721	EFO_0006930	brain volume measurement|neuroimaging measurement	brain volume measurement	EFO_0006930	Other measurement	Neurological	PGS001722	EFO_0006930	measurement	brain volume measurement	EFO_0006930	Other measurement	Neurological
0.7672	8.84E-90	1.27E-86	PGS002764	HP_0003124	Hypercholesterolemia	Hypercholesterolemia	HP_0003124	Other trait	Metabolic	PGS003034	EFO_0004611	low density lipoprotein cholesterol measurement	measurement	EFO_0004611	Lipid or lipoprotein measurement	Metabolic
0.7672	8.84E-90	1.27E-86	PGS002764	HP_0003124	Hypercholesterolemia	Hypercholesterolemia	HP_0003124	Other trait	Metabolic	PGS003034	EFO_0004611	low density lipoprotein cholesterol measurement	measurement	EFO_0004611	Cardiovascular measurement	Cardiovascular
0.7672	8.87E-90	1.27E-86	PGS002603	EFO_0000537	hypertension	hypertension	EFO_0000537	Cardiovascular disease	Cardiovascular	PGS002617	EFO_0006335	systolic blood pressure	systolic blood pressure	EFO_0006335	Other measurement	Cardiovascular
0.7672	8.94E-90	1.28E-86	PGS001289	EFO_0002892	thyroid carcinoma	thyroid carcinoma	EFO_0002892	Cancer	Neoplasms	PGS000633	EFO_0002892	thyroid carcinoma	thyroid carcinoma	EFO_0002892	Cancer	Neoplasms
0.7671	9.17E-90	1.32E-86	PGS002974	EFO_0004339	body height	body height	EFO_0004339	Body measurement	Body measurement	PGS002978	EFO_0004339	body height	body height	EFO_0004339	Body measurement	Body measurement
0.7671	9.20E-90	1.32E-86	PGS001683	EFO_0006930	brain volume measurement|neuroimaging measurement	brain volume measurement	EFO_0006930	Other measurement	Neurological	PGS001673	EFO_0006930	measurement	brain volume measurement	EFO_0006930	Other measurement	Neurological
0.7671	9.67E-90	1.39E-86	PGS002391	EFO_0011015	educational attainment	educational attainment	EFO_0011015	Other measurement	Activities	PGS002440	EFO_0011015	educational attainment	educational attainment	EFO_0011015	Other measurement	Activities
0.767	1.01E-89	1.44E-86	PGS000508	EFO_0000305	breast carcinoma	breast carcinoma	EFO_0000305	Cancer	Neoplasms	PGS000007	EFO_0000305	breast carcinoma	breast carcinoma	EFO_0000305	Cancer	Neoplasms
0.7669	1.09E-89	1.56E-86	PGS002632	EFO_0003923	bone density	bone density	EFO_0003923	Other measurement	Skeletal	PGS001955	EFO_0009270	heel bone mineral density	heel bone mineral density	EFO_0009270	Other measurement	Skeletal
0.7668	1.18E-89	1.69E-86	PGS002315	EFO_0003923	bone density	bone density	EFO_0003923	Other measurement	Skeletal	PGS001529	EFO_0007772	measurement	measurement	EFO_0007772	Other measurement	Skeletal
0.7667	1.30E-89	1.87E-86	PGS003061	EFO_0006527	smoking status measurement	smoking status measurement	EFO_0006527	Other measurement	Psychiatric	PGS003066	EFO_0006527	smoking status measurement	smoking status measurement	EFO_0006527	Other measurement	Psychiatric
-0.7666	1.46E-89	2.09E-86	PGS002181	EFO_0006925	lipoprotein A measurement	lipoprotein A measurement	EFO_0006925	Lipid or lipoprotein measurement	Metabolic	PGS000752	EFO_0006925	lipoprotein A measurement	lipoprotein A measurement	EFO_0006925	Lipid or lipoprotein measurement	Metabolic
0.7666	1.48E-89	2.12E-86	PGS000538	EFO_0000305	breast carcinoma	breast carcinoma	EFO_0000305	Cancer	Neoplasms	PGS000537	EFO_0000305	breast carcinoma	breast carcinoma	EFO_0000305	Cancer	Neoplasms
0.7665	1.50E-89	2.15E-86	PGS001674	EFO_0006930	brain volume measurement|neuroimaging measurement	brain volume measurement	EFO_0006930	Other measurement	Neurological	PGS001680	EFO_0006930	measurement	brain volume measurement	EFO_0006930	Other measurement	Neurological

0.7665	1.50E-89	2.15E-86	PGS002854	EFO_0004340	body mass index	body mass index	EFO_0004340	Body measurement	Body measurement	PGS002630	EFO_0004340	body mass index	body mass index	EFO_0004340	Body measurement	Body measurement
0.7664	1.62E-89	2.32E-86	PGS002341	EFO_0005091	monocyte count	monocyte count	EFO_0005091	Hematological measurement	Hematological	PGS001163	EFO_0005091	monocyte count	monocyte count	EFO_0005091	Hematological measurement	Hematological
0.7664	1.63E-89	2.33E-86	PGS002589	EFO_0000400	diabetes mellitus	diabetes mellitus	EFO_0000400	Metabolic disorder	Metabolic	PGS002491	EFO_0000400	diabetes mellitus	diabetes mellitus	EFO_0000400	Metabolic disorder	Metabolic
0.7664	1.63E-89	2.33E-86	PGS002589	EFO_0000400	diabetes mellitus	diabetes mellitus	EFO_0000400	Digestive system disorder	Gastrointestinal	PGS002491	EFO_0000400	diabetes mellitus	diabetes mellitus	EFO_0000400	Metabolic disorder	Metabolic
0.7664	1.63E-89	2.33E-86	PGS002589	EFO_0000400	diabetes mellitus	diabetes mellitus	EFO_0000400	Digestive system disorder	Gastrointestinal	PGS002491	EFO_0000400	diabetes mellitus	diabetes mellitus	EFO_0000400	Digestive system disorder	Gastrointestinal
0.7664	1.63E-89	2.33E-86	PGS002589	EFO_0000400	diabetes mellitus	diabetes mellitus	EFO_0000400	Metabolic disorder	Metabolic	PGS002491	EFO_0000400	diabetes mellitus	diabetes mellitus	EFO_0000400	Digestive system disorder	Gastrointestinal
0.7663	1.79E-89	2.56E-86	PGS003029	EFO_0004611	low density lipoprotein cholesterol measurement	low density lipoprotein cholesterol measurement	EFO_0004611	Lipid or lipoprotein measurement	Metabolic	PGS003138	EFO_0004574	total cholesterol measurement	total cholesterol measurement	EFO_0004574	Lipid or lipoprotein measurement	Metabolic
0.7663	1.79E-89	2.56E-86	PGS003029	EFO_0004611	low density lipoprotein cholesterol measurement	low density lipoprotein cholesterol measurement	EFO_0004611	Cardiovascular measurement	Cardiovascular	PGS003138	EFO_0004574	total cholesterol measurement	total cholesterol measurement	EFO_0004574	Lipid or lipoprotein measurement	Metabolic
0.7663	1.87E-89	2.67E-86	PGS000065	EFO_0004611	low density lipoprotein cholesterol measurement	low density lipoprotein cholesterol measurement	EFO_0004611	Cardiovascular measurement	Cardiovascular	PGS001933	EFO_0004611	low density lipoprotein cholesterol measurement	measurement	EFO_0004611	Cardiovascular measurement	Cardiovascular
0.7663	1.87E-89	2.67E-86	PGS000065	EFO_0004611	low density lipoprotein cholesterol measurement	low density lipoprotein cholesterol measurement	EFO_0004611	Lipid or lipoprotein measurement	Metabolic	PGS001933	EFO_0004611	low density lipoprotein cholesterol measurement	measurement	EFO_0004611	Cardiovascular measurement	Cardiovascular
0.7663	1.87E-89	2.67E-86	PGS000065	EFO_0004611	low density lipoprotein cholesterol measurement	low density lipoprotein cholesterol measurement	EFO_0004611	Cardiovascular measurement	Cardiovascular	PGS001933	EFO_0004611	low density lipoprotein cholesterol measurement	measurement	EFO_0004611	Lipid or lipoprotein measurement	Metabolic
0.7663	1.87E-89	2.67E-86	PGS000065	EFO_0004611	low density lipoprotein cholesterol measurement	low density lipoprotein cholesterol measurement	EFO_0004611	Lipid or lipoprotein measurement	Metabolic	PGS001933	EFO_0004611	low density lipoprotein cholesterol measurement	measurement	EFO_0004611	Lipid or lipoprotein measurement	Metabolic
0.7663	1.92E-89	2.74E-86	PGS001479	EFO_0006930	brain volume measurement|neuroimaging measurement	brain volume measurement	EFO_0006930	Other measurement	Neurological	PGS001480	EFO_0006930	measurement	brain volume measurement	EFO_0006930	Other measurement	Neurological
0.7662	1.92E-89	2.74E-86	PGS000511	EFO_0000305	breast carcinoma	breast carcinoma	EFO_0000305	Cancer	Neoplasms	PGS003380	EFO_0000305	breast carcinoma	breast carcinoma	EFO_0000305	Cancer	Neoplasms
0.7662	1.96E-89	2.80E-86	PGS001724	EFO_0006930	brain volume measurement|neuroimaging measurement	brain volume measurement	EFO_0006930	Other measurement	Neurological	PGS001737	EFO_0006930	measurement	brain volume measurement	EFO_0006930	Other measurement	Neurological
0.7662	1.96E-89	2.80E-86	PGS001753	EFO_0006930	brain volume measurement|neuroimaging measurement	brain volume measurement	EFO_0006930	Other measurement	Neurological	PGS001752	EFO_0006930	measurement	brain volume measurement	EFO_0006930	Other measurement	Neurological
0.7662	2.01E-89	2.87E-86	PGS002458	EFO_0004611	low density lipoprotein cholesterol measurement	low density lipoprotein cholesterol measurement	EFO_0004611	Lipid or lipoprotein measurement	Metabolic	PGS002571	EFO_0004574	total cholesterol measurement	total cholesterol measurement	EFO_0004574	Lipid or lipoprotein measurement	Metabolic
0.7662	2.01E-89	2.87E-86	PGS002458	EFO_0004611	low density lipoprotein cholesterol measurement	low density lipoprotein cholesterol measurement	EFO_0004611	Cardiovascular measurement	Cardiovascular	PGS002571	EFO_0004574	total cholesterol measurement	total cholesterol measurement	EFO_0004574	Lipid or lipoprotein measurement	Metabolic
0.7661	2.10E-89	3.00E-86	PGS002126	EFO_0006527	smoking status measurement	smoking status measurement	EFO_0006527	Other measurement	Psychiatric	PGS003054	EFO_0006527	smoking status measurement	smoking status measurement	EFO_0006527	Other measurement	Psychiatric
0.7661	2.13E-89	3.03E-86	PGS001674	EFO_0006930	brain volume measurement|neuroimaging measurement	brain volume measurement	EFO_0006930	Other measurement	Neurological	PGS001451	EFO_0006930	measurement	brain volume measurement	EFO_0006930	Other measurement	Neurological
0.7661	2.16E-89	3.08E-86	PGS002521	EFO_1000627	thyroid disease	thyroid disease	EFO_1000627	Other disease	Endocrine	PGS002457	EFO_0004705	hypothyroidism	hypothyroidism	EFO_0004705	Other disease	Endocrine
0.7661	2.22E-89	3.16E-86	PGS003291	EFO_0004617	cystatin C measurement|glomerular filtration rate	cystatin C measurement	EFO_0004617	Other measurement	Metabolic	PGS003306	EFO_0004617	cystatin C measurement|glomerular filtration rate	cystatin C measurement	EFO_0004617	Other measurement	Metabolic
0.766	2.34E-89	3.33E-86	PGS003031	EFO_0004611	low density lipoprotein cholesterol measurement	low density lipoprotein cholesterol measurement	EFO_0004611	Lipid or lipoprotein measurement	Metabolic	PGS002556	EFO_0004611	low density lipoprotein cholesterol measurement	measurement	EFO_0004611	Lipid or lipoprotein measurement	Metabolic
0.766	2.34E-89	3.33E-86	PGS003031	EFO_0004611	low density lipoprotein cholesterol measurement	low density lipoprotein cholesterol measurement	EFO_0004611	Cardiovascular measurement	Cardiovascular	PGS002556	EFO_0004611	low density lipoprotein cholesterol measurement	measurement	EFO_0004611	Lipid or lipoprotein measurement	Metabolic
0.766	2.34E-89	3.33E-86	PGS003031	EFO_0004611	low density lipoprotein cholesterol measurement	low density lipoprotein cholesterol measurement	EFO_0004611	Cardiovascular measurement	Cardiovascular	PGS002556	EFO_0004611	low density lipoprotein cholesterol measurement	measurement	EFO_0004611	Cardiovascular measurement	Cardiovascular
0.766	2.34E-89	3.33E-86	PGS003031	EFO_0004611	low density lipoprotein cholesterol measurement	low density lipoprotein cholesterol measurement	EFO_0004611	Lipid or lipoprotein measurement	Metabolic	PGS002556	EFO_0004611	low density lipoprotein cholesterol measurement	measurement	EFO_0004611	Cardiovascular measurement	Cardiovascular
0.766	2.40E-89	3.42E-86	PGS001106	EFO_0007800	body fat percentage	body fat percentage	EFO_0007800	Body measurement	Body measurement	PGS001227	EFO_0004342	waist circumference	waist circumference	EFO_0004342	Body measurement	Body measurement
0.7659	2.54E-89	3.62E-86	PGS001973	EFO_0004309	platelet count	platelet count	EFO_0004309	Hematological measurement	Hematological	PGS002660	EFO_0004309	platelet count	platelet count	EFO_0004309	Hematological measurement	Hematological
0.7659	2.58E-89	3.67E-86	PGS000921	EFO_0004340	body mass index	body mass index	EFO_0004340	Body measurement	Body measurement	PGS002859	EFO_0004340	body mass index	body mass index	EFO_0004340	Body measurement	Body measurement
0.7659	2.61E-89	3.72E-86	PGS000595	EFO_0005088	testicular carcinoma	testicular carcinoma	EFO_0005088	Cancer	Neoplasms	PGS001806	EFO_0004281	testicular neoplasm	testicular neoplasm	EFO_0004281	Other disease	Neoplasms
0.7658	2.74E-89	3.90E-86	PGS000516	EFO_0000305	breast carcinoma	breast carcinoma	EFO_0000305	Cancer	Neoplasms	PGS000540	EFO_0000305	breast carcinoma	breast carcinoma	EFO_0000305	Cancer	Neoplasms
0.7657	3.08E-89	4.38E-86	PGS000479	EFO_0000305	breast carcinoma	breast carcinoma	EFO_0000305	Cancer	Neoplasms	PGS001804	EFO_0000305	breast carcinoma	breast carcinoma	EFO_0000305	Cancer	Neoplasms
0.7657	3.12E-89	4.43E-86	PGS000015	EFO_0000305	breast carcinoma	breast carcinoma	EFO_0000305	Cancer	Neoplasms	PGS000512	EFO_0000305	breast carcinoma	breast carcinoma	EFO_0000305	Cancer	Neoplasms
0.7656	3.22E-89	4.57E-86	PGS001820	HP_0001943	Hypoglycemia	Hypoglycemia	HP_0001943	Other trait	Metabolic	PGS001014	EFO_0009605	pancreas disease	pancreas disease	EFO_0009605	Digestive system disorder	Gastrointestinal
0.7656	3.30E-89	4.69E-86	PGS000779	MONDO_0004975	Alzheimer disease	Alzheimer disease	MONDO_0004975	Neurological disorder	Neurological	PGS002035	MONDO_0001627	dementia	dementia	MONDO_0001627	Neurological disorder	Neurological
0.7656	3.40E-89	4.82E-86	PGS000687	EFO_0004627	IGF-1 measurement	IGF-1 measurement	EFO_0004627	Other measurement	Metabolic	PGS001960	EFO_0004627	IGF-1 measurement	IGF-1 measurement	EFO_0004627	Other measurement	Metabolic
0.7655	3.59E-89	5.10E-86	PGS002491	EFO_0000400	diabetes mellitus	diabetes mellitus	EFO_0000400	Digestive system disorder	Gastrointestinal	PGS002449	EFO_0004468	glucose measurement	glucose measurement	EFO_0004468	Other measurement	Metabolic
0.7655	3.59E-89	5.10E-86	PGS002491	EFO_0000400	diabetes mellitus	diabetes mellitus	EFO_0000400	Metabolic disorder	Metabolic	PGS002449	EFO_0004468	glucose measurement	glucose measurement	EFO_0004468	Other measurement	Metabolic
0.7655	3.65E-89	5.19E-86	PGS002966	EFO_0004339	body height	body height	EFO_0004339	Body measurement	Body measurement	PGS002968	EFO_0004339	body height	body height	EFO_0004339	Body measurement	Body measurement
0.7654	3.92E-89	5.56E-86	PGS000051	EFO_0000305	breast carcinoma	breast carcinoma	EFO_0000305	Cancer	Neoplasms	PGS000873	EFO_0000305	breast carcinoma	breast carcinoma	EFO_0000305	Cancer	Neoplasms
0.7653	4.41E-89	6.26E-86	PGS002452	EFO_0004541	HbA1c measurement	HbA1c measurement	EFO_0004541	Hematological measurement	Hematological	PGS002491	EFO_0000400	diabetes mellitus	diabetes mellitus	EFO_0000400	Metabolic disorder	Metabolic
0.7653	4.41E-89	6.26E-86	PGS002452	EFO_0004541	HbA1c measurement	HbA1c measurement	EFO_0004541	Hematological measurement	Hematological	PGS002491	EFO_0000400	diabetes mellitus	diabetes mellitus	EFO_0000400	Digestive system disorder	Gastrointestinal
0.7652	4.50E-89	6.38E-86	PGS002590	EFO_0006336	diastolic blood pressure	diastolic blood pressure	EFO_0006336	Other measurement	Cardiovascular	PGS002617	EFO_0006335	systolic blood pressure	systolic blood pressure	EFO_0006335	Other measurement	Cardiovascular
0.7652	4.60E-89	6.52E-86	PGS003143	EFO_0004574	total cholesterol measurement	total cholesterol measurement	EFO_0004574	Lipid or lipoprotein measurement	Metabolic	PGS000888	EFO_0004611	low density lipoprotein cholesterol measurement	measurement	EFO_0004611	Lipid or lipoprotein measurement	Metabolic
0.7652	4.60E-89	6.52E-86	PGS003143	EFO_0004574	total cholesterol measurement	total cholesterol measurement	EFO_0004574	Lipid or lipoprotein measurement	Metabolic	PGS000888	EFO_0004611	low density lipoprotein cholesterol measurement	measurement	EFO_0004611	Cardiovascular measurement	Cardiovascular
0.7652	4.61E-89	6.53E-86	PGS001227	EFO_0004342	waist circumference	waist circumference	EFO_0004342	Body measurement	Body measurement	PGS001102	EFO_0007800	body fat percentage	body fat percentage	EFO_0007800	Body measurement	Body measurement
0.7652	4.69E-89	6.64E-86	PGS000893	EFO_0004611	low density lipoprotein cholesterol measurement	low density lipoprotein cholesterol measurement	EFO_0004611	Lipid or lipoprotein measurement	Metabolic	PGS002556	EFO_0004611	low density lipoprotein cholesterol measurement	measurement	EFO_0004611	Cardiovascular measurement	Cardiovascular
0.7652	4.69E-89	6.64E-86	PGS000893	EFO_0004611	low density lipoprotein cholesterol measurement	low density lipoprotein cholesterol measurement	EFO_0004611	Cardiovascular measurement	Cardiovascular	PGS002556	EFO_0004611	low density lipoprotein cholesterol measurement	measurement	EFO_0004611	Lipid or lipoprotein measurement	Metabolic
0.7652	4.69E-89	6.64E-86	PGS000893	EFO_0004611	low density lipoprotein cholesterol measurement	low density lipoprotein cholesterol measurement	EFO_0004611	Lipid or lipoprotein measurement	Metabolic	PGS002556	EFO_0004611	low density lipoprotein cholesterol measurement	measurement	EFO_0004611	Lipid or lipoprotein measurement	Metabolic
0.7652	4.69E-89	6.64E-86	PGS000893	EFO_0004611	low density lipoprotein cholesterol measurement	low density lipoprotein cholesterol measurement	EFO_0004611	Cardiovascular measurement	Cardiovascular	PGS002556	EFO_0004611	low density lipoprotein cholesterol measurement	measurement	EFO_0004611	Cardiovascular measurement	Cardiovascular
0.7651	5.05E-89	7.15E-86	PGS000642	EFO_0005952	non-Hodgkins lymphoma	non-Hodgkins lymphoma	EFO_0005952	Cancer	Neoplasms	PGS000080	EFO_0005952	non-Hodgkins lymphoma	non-Hodgkins lymphoma	EFO_0005952	Cancer	Neoplasms
0.7649	5.80E-89	8.22E-86	PGS002331	EFO_0004541	HbA1c measurement	HbA1c measurement	EFO_0004541	Hematological measurement	Hematological	PGS000685	EFO_0004541	HbA1c measurement	HbA1c measurement	EFO_0004541	Hematological measurement	Hematological
0.7649	5.95E-89	8.42E-86	PGS001517	EFO_0007985	platelet crit	platelet crit	EFO_0007985	Hematological measurement	Hematological	PGS001238	EFO_0004309	platelet count	platelet count	EFO_0004309	Hematological measurement	Hematological
0.7649	6.14E-89	8.69E-86	PGS000892	EFO_0004611	low density lipoprotein cholesterol measurement	low density lipoprotein cholesterol measurement	EFO_0004611	Lipid or lipoprotein measurement	Metabolic	PGS003138	EFO_0004574	total cholesterol measurement	total cholesterol measurement	EFO_0004574	Lipid or lipoprotein measurement	Metabolic
0.7649	6.14E-89	8.69E-86	PGS000892	EFO_0004611	low density lipoprotein cholesterol measurement	low density lipoprotein cholesterol measurement	EFO_0004611	Cardiovascular measurement	Cardiovascular	PGS003138	EFO_0004574	total cholesterol measurement	total cholesterol measurement	EFO_0004574	Lipid or lipoprotein measurement	Metabolic
0.7648	6.36E-89	9.00E-86	PGS002970	EFO_0004339	body height	body height	EFO_0004339	Body measurement	Body measurement	PGS002964	EFO_0004339	body height	body height	EFO_0004339	Body measurement	Body measurement
0.7648	6.59E-89	9.33E-86	PGS000531	EFO_0000305	breast carcinoma	breast carcinoma	EFO_0000305	Cancer	Neoplasms	PGS000512	EFO_0000305	breast carcinoma	breast carcinoma	EFO_0000305	Cancer	Neoplasms
0.7648	6.70E-89	9.47E-86	PGS002322	EFO_0006336	diastolic blood pressure	diastolic blood pressure	EFO_0006336	Other measurement	Cardiovascular	PGS002898	EFO_0006336	diastolic blood pressure	diastolic blood pressure	EFO_0006336	Other measurement	Cardiovascular
0.7647	7.18E-89	1.01E-85	PGS001674	EFO_0006930	brain volume measurement|neuroimaging measurement	brain volume measurement	EFO_0006930	Other measurement	Neurological	PGS001450	EFO_0006930	measurement	brain volume measurement	EFO_0006930	Other measurement	Neurological
0.7646	7.82E-89	1.11E-85	PGS003201	EFO_0004631	vitamin D measurement	vitamin D measurement	EFO_0004631	Lipid or lipoprotein measurement	Metabolic	PGS003191	EFO_0004631	vitamin D measurement	vitamin D measurement	EFO_0004631	Lipid or lipoprotein measurement	Metabolic
0.7644	9.28E-89	1.31E-85	PGS002656	EFO_0004527	mean corpuscular hemoglobin	mean corpuscular hemoglobin	EFO_0004527	Hematological measurement	Hematological	PGS000101	EFO_0004526	mean corpuscular volume	mean corpuscular volume	EFO_0004526	Hematological measurement	Hematological
0.7644	9.29E-89	1.31E-85	PGS002958	EFO_0004612	high density lipoprotein cholesterol measurement	high density lipoprotein cholesterol measurement	EFO_0004612	Lipid or lipoprotein measurement	Metabolic	PGS001954	EFO_0004612	high density lipoprotein cholesterol measurement	measurement	EFO_0004612	Lipid or lipoprotein measurement	Metabolic
0.7644	9.45E-89	1.33E-85	PGS000708	EFO_1002048	kidney failure	kidney failure	EFO_1002048	Other disease	Genitourinary	PGS000680	EFO_0004617	cystatin C measurement	cystatin C measurement	EFO_0004617	Other measurement	Metabolic
0.7643	9.99E-89	1.41E-85	PGS002660	EFO_0004309	platelet count	platelet count	EFO_0004309	Hematological measurement	Hematological	PGS002191	EFO_0004309	platelet count	platelet count	EFO_0004309	Hematological measurement	Hematological
0.7642	1.05E-88	1.48E-85	PGS002037	EFO_0003768	nicotine dependence	nicotine dependence	EFO_0003768	Neurological disorder	Neurological	PGS001830	EFO_0003768	nicotine dependence	nicotine dependence	EFO_0003768	Neurological disorder	Neurological
0.7642	1.06E-88	1.50E-85	PGS003034	EFO_0004611	low density lipoprotein cholesterol measurement	low density lipoprotein cholesterol measurement	EFO_0004611	Lipid or lipoprotein measurement	Metabolic	PGS003138	EFO_0004574	total cholesterol measurement	total cholesterol measurement	EFO_0004574	Lipid or lipoprotein measurement	Metabolic
0.7642	1.06E-88	1.50E-85	PGS003034	EFO_0004611	low density lipoprotein cholesterol measurement	low density lipoprotein cholesterol measurement	EFO_0004611	Cardiovascular measurement	Cardiovascular	PGS003138	EFO_0004574	total cholesterol measurement	total cholesterol measurement	EFO_0004574	Lipid or lipoprotein measurement	Metabolic
0.7642	1.08E-88	1.52E-85	PGS000532	EFO_0000305	breast carcinoma	breast carcinoma	EFO_0000305	Cancer	Neoplasms	PGS000512	EFO_0000305	breast carcinoma	breast carcinoma	EFO_0000305	Cancer	Neoplasms
0.7641	1.14E-88	1.61E-85	PGS000539	EFO_0000305	breast carcinoma	breast carcinoma	EFO_0000305	Cancer	Neoplasms	PGS000008	EFO_1000649	estrogen-receptor positive breast cancer	cancer	EFO_1000649	Cancer	Neoplasms
0.764	1.29E-88	1.82E-85	PGS001724	EFO_0006930	brain volume measurement|neuroimaging measurement	brain volume measurement	EFO_0006930	Other measurement	Neurological	PGS001723	EFO_0006930	measurement	brain volume measurement	EFO_0006930	Other measurement	Neurological
0.764	1.29E-88	1.82E-85	PGS001476	EFO_0006930	brain volume measurement|neuroimaging measurement	brain volume measurement	EFO_0006930	Other measurement	Neurological	PGS001673	EFO_0006930	measurement	brain volume measurement	EFO_0006930	Other measurement	Neurological
0.7639	1.34E-88	1.88E-85	PGS001456	EFO_0006930	brain volume measurement|neuroimaging measurement	brain volume measurement	EFO_0006930	Other measurement	Neurological	PGS001455	EFO_0006930	measurement	brain volume measurement	EFO_0006930	Other measurement	Neurological
0.7638	1.46E-88	2.06E-85	PGS000215	EFO_1000294	HER2 Positive Breast Carcinoma	HER2 Positive Breast Carcinoma	EFO_1000294	Cancer	Neoplasms	PGS000346	EFO_1000650	estrogen-receptor negative breast cancer	cancer	EFO_1000650	Cancer	Neoplasms
0.7638	1.48E-88	2.08E-85	PGS000790	EFO_0000756	melanoma	melanoma	EFO_0000756	Cancer	Neoplasms	PGS000426	EFO_0000756	melanoma	melanoma	EFO_0000756	Cancer	Neoplasms
0.7638	1.48E-88	2.08E-85	PGS000790	EFO_0000756	melanoma	melanoma	EFO_0000756	Cancer	Neoplasms	PGS000428	EFO_0000756	melanoma	melanoma	EFO_0000756	Cancer	Neoplasms
0.7638	1.48E-88	2.08E-85	PGS000790	EFO_0000756	melanoma	melanoma	EFO_0000756	Cancer	Neoplasms	PGS000427	EFO_0000756	melanoma	melanoma	EFO_0000756	Cancer	Neoplasms
0.7638	1.48E-88	2.08E-85	PGS000790	EFO_0000756	melanoma	melanoma	EFO_0000756	Cancer	Neoplasms	PGS000425	EFO_0000756	melanoma	melanoma	EFO_0000756	Cancer	Neoplasms
0.7638	1.48E-88	2.08E-85	PGS002403	EFO_0004541	HbA1c measurement	HbA1c measurement	EFO_0004541	Hematological measurement	Hematological	PGS002491	EFO_0000400	diabetes mellitus	diabetes mellitus	EFO_0000400	Digestive system disorder	Gastrointestinal
0.7638	1.48E-88	2.08E-85	PGS002403	EFO_0004541	HbA1c measurement	HbA1c measurement	EFO_0004541	Hematological measurement	Hematological	PGS002491	EFO_0000400	diabetes mellitus	diabetes mellitus	EFO_0000400	Metabolic disorder	Metabolic
0.7637	1.60E-88	2.25E-85	PGS002592	EFO_0001379	endocrine system disease|diabetes mellitus	endocrine system disease	EFO_0001379	Other disease	Metabolic	PGS002599	EFO_0004541	HbA1c measurement	HbA1c measurement	EFO_0004541	Hematological measurement	Hematological
0.7637	1.64E-88	2.31E-85	PGS003035	EFO_0004611	low density lipoprotein cholesterol measurement	low density lipoprotein cholesterol measurement	EFO_0004611	Lipid or lipoprotein measurement	Metabolic	PGS002556	EFO_0004611	low density lipoprotein cholesterol measurement	measurement	EFO_0004611	Cardiovascular measurement	Cardiovascular
0.7637	1.64E-88	2.31E-85	PGS003035	EFO_0004611	low density lipoprotein cholesterol measurement	low density lipoprotein cholesterol measurement	EFO_0004611	Lipid or lipoprotein measurement	Metabolic	PGS002556	EFO_0004611	low density lipoprotein cholesterol measurement	measurement	EFO_0004611	Lipid or lipoprotein measurement	Metabolic
0.7637	1.64E-88	2.31E-85	PGS003035	EFO_0004611	low density lipoprotein cholesterol measurement	low density lipoprotein cholesterol measurement	EFO_0004611	Cardiovascular measurement	Cardiovascular	PGS002556	EFO_0004611	low density lipoprotein cholesterol measurement	measurement	EFO_0004611	Cardiovascular measurement	Cardiovascular
0.7637	1.64E-88	2.31E-85	PGS003035	EFO_0004611	low density lipoprotein cholesterol measurement	low density lipoprotein cholesterol measurement	EFO_0004611	Cardiovascular measurement	Cardiovascular	PGS002556	EFO_0004611	low density lipoprotein cholesterol measurement	measurement	EFO_0004611	Lipid or lipoprotein measurement	Metabolic
0.7637	1.65E-88	2.32E-85	PGS002764	HP_0003124	Hypercholesterolemia	Hypercholesterolemia	HP_0003124	Other trait	Metabolic	PGS001933	EFO_0004611	low density lipoprotein cholesterol measurement	measurement	EFO_0004611	Lipid or lipoprotein measurement	Metabolic
0.7637	1.65E-88	2.32E-85	PGS002764	HP_0003124	Hypercholesterolemia	Hypercholesterolemia	HP_0003124	Other trait	Metabolic	PGS001933	EFO_0004611	low density lipoprotein cholesterol measurement	measurement	EFO_0004611	Cardiovascular measurement	Cardiovascular
0.7637	1.66E-88	2.33E-85	PGS000510	EFO_0000305	breast carcinoma	breast carcinoma	EFO_0000305	Cancer	Neoplasms	PGS000502	EFO_0000305	breast carcinoma	breast carcinoma	EFO_0000305	Cancer	Neoplasms
0.7637	1.68E-88	2.36E-85	PGS002891	EFO_0006336	diastolic blood pressure	diastolic blood pressure	EFO_0006336	Other measurement	Cardiovascular	PGS002895	EFO_0006336	diastolic blood pressure	diastolic blood pressure	EFO_0006336	Other measurement	Cardiovascular
0.7637	1.70E-88	2.38E-85	PGS002501	EFO_0004541	HbA1c measurement	HbA1c measurement	EFO_0004541	Hematological measurement	Hematological	PGS002592	EFO_0001379	endocrine system disease|diabetes mellitus	endocrine system disease	EFO_0001379	Other disease	Metabolic
0.7636	1.72E-88	2.42E-85	PGS000103	EFO_0007989	monocyte percentage of leukocytes	monocyte percentage of leukocytes	EFO_0007989	Hematological measurement	Hematological	PGS002208	EFO_0007989	monocyte percentage of leukocytes	monocyte percentage of leukocytes	EFO_0007989	Hematological measurement	Hematological
0.7635	1.86E-88	2.61E-85	PGS000888	EFO_0004611	low density lipoprotein cholesterol measurement	low density lipoprotein cholesterol measurement	EFO_0004611	Lipid or lipoprotein measurement	Metabolic	PGS003138	EFO_0004574	total cholesterol measurement	total cholesterol measurement	EFO_0004574	Lipid or lipoprotein measurement	Metabolic
0.7635	1.86E-88	2.61E-85	PGS000888	EFO_0004611	low density lipoprotein cholesterol measurement	low density lipoprotein cholesterol measurement	EFO_0004611	Cardiovascular measurement	Cardiovascular	PGS003138	EFO_0004574	total cholesterol measurement	total cholesterol measurement	EFO_0004574	Lipid or lipoprotein measurement	Metabolic
0.7635	1.99E-88	2.79E-85	PGS002501	EFO_0004541	HbA1c measurement	HbA1c measurement	EFO_0004541	Hematological measurement	Hematological	PGS002622	MONDO_0005148	type 2 diabetes mellitus	type 2 diabetes mellitus	MONDO_0005148	Digestive system disorder	Gastrointestinal
0.7635	1.99E-88	2.79E-85	PGS002501	EFO_0004541	HbA1c measurement	HbA1c measurement	EFO_0004541	Hematological measurement	Hematological	PGS002622	MONDO_0005148	type 2 diabetes mellitus	type 2 diabetes mellitus	MONDO_0005148	Metabolic disorder	Metabolic
-0.7635	1.99E-88	2.79E-85	PGS002774	EFO_0000275	atrial fibrillation	atrial fibrillation	EFO_0000275	Cardiovascular disease	Cardiovascular	PGS001356	EFO_0000275	atrial fibrillation	atrial fibrillation	EFO_0000275	Cardiovascular disease	Cardiovascular
0.7634	2.02E-88	2.84E-85	PGS003291	EFO_0004617	cystatin C measurement|glomerular filtration rate	cystatin C measurement	EFO_0004617	Other measurement	Metabolic	PGS003305	EFO_0004617	cystatin C measurement|glomerular filtration rate	cystatin C measurement	EFO_0004617	Other measurement	Metabolic
0.7634	2.04E-88	2.86E-85	PGS001479	EFO_0006930	brain volume measurement|neuroimaging measurement	brain volume measurement	EFO_0006930	Other measurement	Neurological	PGS001489	EFO_0006930	measurement	brain volume measurement	EFO_0006930	Other measurement	Neurological
-0.7634	2.05E-88	2.86E-85	PGS002768	EFO_0003882	osteoporosis	osteoporosis	EFO_0003882	Other disease	Skeletal	PGS001529	EFO_0007772	measurement	measurement	EFO_0007772	Other measurement	Skeletal
0.7634	2.07E-88	2.89E-85	PGS000082	EFO_0001075	ovarian carcinoma	ovarian carcinoma	EFO_0001075	Cancer	Neoplasms	PGS000793	EFO_0001075	ovarian carcinoma	ovarian carcinoma	EFO_0001075	Cancer	Neoplasms
0.7634	2.08E-88	2.91E-85	PGS003194	EFO_0004631	vitamin D measurement	vitamin D measurement	EFO_0004631	Lipid or lipoprotein measurement	Metabolic	PGS002355	EFO_0004631	vitamin D measurement	vitamin D measurement	EFO_0004631	Lipid or lipoprotein measurement	Metabolic
0.7634	2.08E-88	2.91E-85	PGS002764	HP_0003124	Hypercholesterolemia	Hypercholesterolemia	HP_0003124	Other trait	Metabolic	PGS003029	EFO_0004611	low density lipoprotein cholesterol measurement	measurement	EFO_0004611	Cardiovascular measurement	Cardiovascular
0.7634	2.08E-88	2.91E-85	PGS002764	HP_0003124	Hypercholesterolemia	Hypercholesterolemia	HP_0003124	Other trait	Metabolic	PGS003029	EFO_0004611	low density lipoprotein cholesterol measurement	measurement	EFO_0004611	Lipid or lipoprotein measurement	Metabolic
0.7634	2.12E-88	2.96E-85	PGS002517	EFO_0000684	respiratory system disease	respiratory system disease	EFO_0000684	Other disease	Skeletal	PGS002468	EFO_0000684	respiratory system disease	respiratory system disease	EFO_0000684	Other disease	Skeletal

0.7633	2.20E-88	3.07E-85	PGS001652	EFO_0006930	brain volume measurement|neuroimaging measurement	brain volume measurement	EFO_0006930	Other measurement	Neurological	PGS001654	EFO_0006930	measurement	brain volume measurement	EFO_0006930	Other measurement	Neurological
0.7633	2.21E-88	3.09E-85	PGS001037	EFO_0009270	heel bone mineral density	heel bone mineral density	EFO_0009270	Other measurement	Skeletal	PGS001530	EFO_0007772	measurement	measurement	EFO_0007772	Other measurement	Skeletal
0.7633	2.22E-88	3.10E-85	PGS000952	EFO_0004514	bone quantitative ultrasound measurement	bone quantitative ultrasound measurement	EFO_0004514	Other measurement	Skeletal	PGS001530	EFO_0007772	measurement	measurement	EFO_0007772	Other measurement	Skeletal
0.7633	2.28E-88	3.18E-85	PGS001900	EFO_0006336	diastolic blood pressure	diastolic blood pressure	EFO_0006336	Other measurement	Cardiovascular	PGS002893	EFO_0006336	diastolic blood pressure	diastolic blood pressure	EFO_0006336	Other measurement	Cardiovascular
0.7632	2.41E-88	3.37E-85	PGS001037	EFO_0009270	heel bone mineral density	heel bone mineral density	EFO_0009270	Other measurement	Skeletal	PGS001403	EFO_0009270	heel bone mineral density	heel bone mineral density	EFO_0009270	Other measurement	Skeletal
0.7632	2.45E-88	3.42E-85	PGS000952	EFO_0004514	bone quantitative ultrasound measurement	bone quantitative ultrasound measurement	EFO_0004514	Other measurement	Skeletal	PGS001403	EFO_0009270	heel bone mineral density	heel bone mineral density	EFO_0009270	Other measurement	Skeletal
0.7631	2.64E-88	3.68E-85	PGS002963	EFO_0004612	high density lipoprotein cholesterol measurement	high density lipoprotein cholesterol measurement	EFO_0004612	Lipid or lipoprotein measurement	Metabolic	PGS002172	EFO_0004612	high density lipoprotein cholesterol measurement	measurement	EFO_0004612	Lipid or lipoprotein measurement	Metabolic
0.7631	2.68E-88	3.74E-85	PGS000515	EFO_0000305	breast carcinoma	breast carcinoma	EFO_0000305	Cancer	Neoplasms	PGS001804	EFO_0000305	breast carcinoma	breast carcinoma	EFO_0000305	Cancer	Neoplasms
0.7629	3.12E-88	4.35E-85	PGS001840	MONDO_0004596	cor pulmonale	cor pulmonale	MONDO_0004596	Cardiovascular disease	Cardiovascular	PGS001844	EFO_1001395	Phlebitis|Thrombophlebitis	Phlebitis	EFO_1001395	Cardiovascular disease	Cardiovascular
0.7628	3.40E-88	4.74E-85	PGS002191	EFO_0004309	platelet count	platelet count	EFO_0004309	Hematological measurement	Hematological	PGS002188	EFO_0007985	platelet crit	platelet crit	EFO_0007985	Hematological measurement	Hematological
0.7627	3.71E-88	5.17E-85	PGS003186	EFO_0004631	vitamin D measurement	vitamin D measurement	EFO_0004631	Lipid or lipoprotein measurement	Metabolic	PGS003191	EFO_0004631	vitamin D measurement	vitamin D measurement	EFO_0004631	Lipid or lipoprotein measurement	Metabolic
0.7627	3.82E-88	5.33E-85	PGS003270	EFO_0010472	creatine measurement|glomerular filtration rate	creatine measurement	EFO_0010472	Other measurement	Metabolic	PGS003269	EFO_0010472	creatine measurement|glomerular filtration rate	creatine measurement	EFO_0010472	Other measurement	Metabolic
0.7627	3.90E-88	5.44E-85	PGS002337	EFO_0004611	low density lipoprotein cholesterol measurement	low density lipoprotein cholesterol measurement	EFO_0004611	Cardiovascular measurement	Cardiovascular	PGS000892	EFO_0004611	low density lipoprotein cholesterol measurement	measurement	EFO_0004611	Cardiovascular measurement	Cardiovascular
0.7627	3.90E-88	5.44E-85	PGS002337	EFO_0004611	low density lipoprotein cholesterol measurement	low density lipoprotein cholesterol measurement	EFO_0004611	Lipid or lipoprotein measurement	Metabolic	PGS000892	EFO_0004611	low density lipoprotein cholesterol measurement	measurement	EFO_0004611	Cardiovascular measurement	Cardiovascular
0.7627	3.90E-88	5.44E-85	PGS002337	EFO_0004611	low density lipoprotein cholesterol measurement	low density lipoprotein cholesterol measurement	EFO_0004611	Lipid or lipoprotein measurement	Metabolic	PGS000892	EFO_0004611	low density lipoprotein cholesterol measurement	measurement	EFO_0004611	Lipid or lipoprotein measurement	Metabolic
0.7627	3.90E-88	5.44E-85	PGS002337	EFO_0004611	low density lipoprotein cholesterol measurement	low density lipoprotein cholesterol measurement	EFO_0004611	Cardiovascular measurement	Cardiovascular	PGS000892	EFO_0004611	low density lipoprotein cholesterol measurement	measurement	EFO_0004611	Lipid or lipoprotein measurement	Metabolic
0.7626	3.98E-88	5.54E-85	PGS001117	EFO_0600082	insulin use measurement	insulin use measurement	EFO_0600082	Other measurement	Metabolic	PGS001014	EFO_0009605	pancreas disease	pancreas disease	EFO_0009605	Digestive system disorder	Gastrointestinal
0.7626	4.15E-88	5.77E-85	PGS001990	EFO_0004526	mean corpuscular volume	mean corpuscular volume	EFO_0004526	Hematological measurement	Hematological	PGS001219	EFO_0004527	mean corpuscular hemoglobin	mean corpuscular hemoglobin	EFO_0004527	Hematological measurement	Hematological
-0.7626	4.21E-88	5.86E-85	PGS002757	EFO_0003884	chronic kidney disease	chronic kidney disease	EFO_0003884	Other disease	Metabolic	PGS002237	EFO_0003884	chronic kidney disease	chronic kidney disease	EFO_0003884	Other disease	Metabolic
0.7625	4.53E-88	6.31E-85	PGS000532	EFO_0000305	breast carcinoma	breast carcinoma	EFO_0000305	Cancer	Neoplasms	PGS000503	EFO_0000305	breast carcinoma	breast carcinoma	EFO_0000305	Cancer	Neoplasms
0.7624	4.85E-88	6.75E-85	PGS003076	EFO_0006335	systolic blood pressure	systolic blood pressure	EFO_0006335	Other measurement	Cardiovascular	PGS003070	EFO_0006335	systolic blood pressure	systolic blood pressure	EFO_0006335	Other measurement	Cardiovascular
0.7623	5.07E-88	7.05E-85	PGS001917	EFO_0007800	body fat percentage	body fat percentage	EFO_0007800	Body measurement	Body measurement	PGS002201	EFO_0004342	waist circumference	waist circumference	EFO_0004342	Body measurement	Body measurement
0.7623	5.31E-88	7.38E-85	PGS001716	EFO_0006930	brain volume measurement|neuroimaging measurement	brain volume measurement	EFO_0006930	Other measurement	Neurological	PGS001715	EFO_0006930	measurement	brain volume measurement	EFO_0006930	Other measurement	Neurological
0.7623	5.40E-88	7.51E-85	PGS001103	EFO_0007800	body fat percentage	body fat percentage	EFO_0007800	Body measurement	Body measurement	PGS001228	EFO_0004340	body mass index	body mass index	EFO_0004340	Body measurement	Body measurement
0.7623	5.41E-88	7.52E-85	PGS000332	EFO_0000305	breast carcinoma	breast carcinoma	EFO_0000305	Cancer	Neoplasms	PGS000536	EFO_0000305	breast carcinoma	breast carcinoma	EFO_0000305	Cancer	Neoplasms
0.762	6.48E-88	9.00E-85	PGS002966	EFO_0004339	body height	body height	EFO_0004339	Body measurement	Body measurement	PGS002973	EFO_0004339	body height	body height	EFO_0004339	Body measurement	Body measurement
0.762	6.62E-88	9.19E-85	PGS000110	EFO_0004305	erythrocyte count	erythrocyte count	EFO_0004305	Hematological measurement	Hematological	PGS002345	EFO_0004305	erythrocyte count	erythrocyte count	EFO_0004305	Hematological measurement	Hematological
0.762	6.66E-88	9.24E-85	PGS003118	MONDO_0005148	type 2 diabetes mellitus	type 2 diabetes mellitus	MONDO_0005148	Digestive system disorder	Gastrointestinal	PGS003114	MONDO_0005148	type 2 diabetes mellitus	type 2 diabetes mellitus	MONDO_0005148	Digestive system disorder	Gastrointestinal
0.762	6.66E-88	9.24E-85	PGS003118	MONDO_0005148	type 2 diabetes mellitus	type 2 diabetes mellitus	MONDO_0005148	Metabolic disorder	Metabolic	PGS003114	MONDO_0005148	type 2 diabetes mellitus	type 2 diabetes mellitus	MONDO_0005148	Digestive system disorder	Gastrointestinal
0.762	6.66E-88	9.24E-85	PGS003118	MONDO_0005148	type 2 diabetes mellitus	type 2 diabetes mellitus	MONDO_0005148	Digestive system disorder	Gastrointestinal	PGS003114	MONDO_0005148	type 2 diabetes mellitus	type 2 diabetes mellitus	MONDO_0005148	Metabolic disorder	Metabolic
0.762	6.66E-88	9.24E-85	PGS003118	MONDO_0005148	type 2 diabetes mellitus	type 2 diabetes mellitus	MONDO_0005148	Metabolic disorder	Metabolic	PGS003114	MONDO_0005148	type 2 diabetes mellitus	type 2 diabetes mellitus	MONDO_0005148	Metabolic disorder	Metabolic
0.762	6.91E-88	9.59E-85	PGS002605	EFO_0004611	low density lipoprotein cholesterol measurement	low density lipoprotein cholesterol measurement	EFO_0004611	Cardiovascular measurement	Cardiovascular	PGS002458	EFO_0004611	low density lipoprotein cholesterol measurement	measurement	EFO_0004611	Cardiovascular measurement	Cardiovascular
0.762	6.91E-88	9.59E-85	PGS002605	EFO_0004611	low density lipoprotein cholesterol measurement	low density lipoprotein cholesterol measurement	EFO_0004611	Cardiovascular measurement	Cardiovascular	PGS002458	EFO_0004611	low density lipoprotein cholesterol measurement	measurement	EFO_0004611	Lipid or lipoprotein measurement	Metabolic
0.762	6.91E-88	9.59E-85	PGS002605	EFO_0004611	low density lipoprotein cholesterol measurement	low density lipoprotein cholesterol measurement	EFO_0004611	Lipid or lipoprotein measurement	Metabolic	PGS002458	EFO_0004611	low density lipoprotein cholesterol measurement	measurement	EFO_0004611	Cardiovascular measurement	Cardiovascular
0.762	6.91E-88	9.59E-85	PGS002605	EFO_0004611	low density lipoprotein cholesterol measurement	low density lipoprotein cholesterol measurement	EFO_0004611	Lipid or lipoprotein measurement	Metabolic	PGS002458	EFO_0004611	low density lipoprotein cholesterol measurement	measurement	EFO_0004611	Lipid or lipoprotein measurement	Metabolic
0.762	6.95E-88	9.65E-85	PGS001670	EFO_0006930	brain volume measurement|neuroimaging measurement	brain volume measurement	EFO_0006930	Other measurement	Neurological	PGS001673	EFO_0006930	measurement	brain volume measurement	EFO_0006930	Other measurement	Neurological
0.762	7.00E-88	9.71E-85	PGS002329	EFO_0004612	high density lipoprotein cholesterol measurement	high density lipoprotein cholesterol measurement	EFO_0004612	Lipid or lipoprotein measurement	Metabolic	PGS000686	EFO_0004612	high density lipoprotein cholesterol measurement	measurement	EFO_0004612	Lipid or lipoprotein measurement	Metabolic
0.7618	7.79E-88	1.08E-84	PGS002257	EFO_0006335	systolic blood pressure	systolic blood pressure	EFO_0006335	Other measurement	Cardiovascular	PGS000302	EFO_0006336	diastolic blood pressure	diastolic blood pressure	EFO_0006336	Other measurement	Cardiovascular
0.7618	7.95E-88	1.10E-84	PGS001108	EFO_0007777	base metabolic rate measurement	base metabolic rate measurement	EFO_0007777	Other measurement	Metabolic	PGS002202	EFO_0009805	whole body water mass	whole body water mass	EFO_0009805	Other measurement	Body measurement
0.7617	8.91E-88	1.23E-84	PGS001117	EFO_0600082	insulin use measurement	insulin use measurement	EFO_0600082	Other measurement	Metabolic	PGS000023	MONDO_0005147	type 1 diabetes mellitus	type 1 diabetes mellitus	MONDO_0005147	Immune system disorder	Immunological
0.7617	8.91E-88	1.23E-84	PGS001117	EFO_0600082	insulin use measurement	insulin use measurement	EFO_0600082	Other measurement	Metabolic	PGS000023	MONDO_0005147	type 1 diabetes mellitus	type 1 diabetes mellitus	MONDO_0005147	Metabolic disorder	Metabolic
0.7617	8.91E-88	1.23E-84	PGS001117	EFO_0600082	insulin use measurement	insulin use measurement	EFO_0600082	Other measurement	Metabolic	PGS000023	MONDO_0005147	type 1 diabetes mellitus	type 1 diabetes mellitus	MONDO_0005147	Digestive system disorder	Gastrointestinal
0.7616	9.01E-88	1.25E-84	PGS001402	EFO_0009270	heel bone mineral density	heel bone mineral density	EFO_0009270	Other measurement	Skeletal	PGS001403	EFO_0009270	heel bone mineral density	heel bone mineral density	EFO_0009270	Other measurement	Skeletal
0.7616	9.02E-88	1.25E-84	PGS001402	EFO_0009270	heel bone mineral density	heel bone mineral density	EFO_0009270	Other measurement	Skeletal	PGS001530	EFO_0007772	measurement	measurement	EFO_0007772	Other measurement	Skeletal
0.7616	9.22E-88	1.28E-84	PGS000529	EFO_0000305	breast carcinoma	breast carcinoma	EFO_0000305	Cancer	Neoplasms	PGS000497	EFO_0000305	breast carcinoma	breast carcinoma	EFO_0000305	Cancer	Neoplasms
0.7616	9.22E-88	1.28E-84	PGS000529	EFO_0000305	breast carcinoma	breast carcinoma	EFO_0000305	Cancer	Neoplasms	PGS000498	EFO_0000305	breast carcinoma	breast carcinoma	EFO_0000305	Cancer	Neoplasms
0.7616	9.22E-88	1.28E-84	PGS002854	EFO_0004340	body mass index	body mass index	EFO_0004340	Body measurement	Body measurement	PGS002169	EFO_0005409	fat body mass	fat body mass	EFO_0005409	Other measurement	Body measurement
0.7616	9.49E-88	1.31E-84	PGS000575	EFO_0001663	prostate carcinoma	prostate carcinoma	EFO_0001663	Cancer	Neoplasms	PGS000594	EFO_0007355	male reproductive organ cancer	male reproductive organ cancer	EFO_0007355	Cancer	Neoplasms
-0.7615	1.01E-87	1.39E-84	PGS001890	EFO_0008385	visual acuity measurement	visual acuity measurement	EFO_0008385	Other measurement	Ophthalmological	PGS002211	HP_0000545	Myopia	Myopia	HP_0000545	Other trait	Ophthalmological
0.7615	1.01E-87	1.40E-84	PGS001488	EFO_0006930	brain volume measurement|neuroimaging measurement	brain volume measurement	EFO_0006930	Other measurement	Neurological	PGS001484	EFO_0006930	measurement	brain volume measurement	EFO_0006930	Other measurement	Neurological
0.7614	1.08E-87	1.49E-84	PGS001594	EFO_0005420	measurement	grey matter volume measurement	EFO_0005420	Other measurement	Neurological	PGS001613	EFO_0005420	measurement	grey matter volume measurement	EFO_0005420	Other measurement	Neurological
0.7614	1.09E-87	1.51E-84	PGS000633	EFO_0002892	thyroid carcinoma	thyroid carcinoma	EFO_0002892	Cancer	Neoplasms	PGS000797	EFO_0002892	thyroid carcinoma	thyroid carcinoma	EFO_0002892	Cancer	Neoplasms
0.7614	1.11E-87	1.54E-84	PGS000537	EFO_0000305	breast carcinoma	breast carcinoma	EFO_0000305	Cancer	Neoplasms	PGS000540	EFO_0000305	breast carcinoma	breast carcinoma	EFO_0000305	Cancer	Neoplasms
0.7614	1.12E-87	1.55E-84	PGS002802	EFO_0004339	body height	body height	EFO_0004339	Body measurement	Body measurement	PGS000758	EFO_0004339	body height	body height	EFO_0004339	Body measurement	Body measurement
0.7614	1.13E-87	1.56E-84	PGS003033	EFO_0004611	low density lipoprotein cholesterol measurement	low density lipoprotein cholesterol measurement	EFO_0004611	Lipid or lipoprotein measurement	Metabolic	PGS001933	EFO_0004611	low density lipoprotein cholesterol measurement	measurement	EFO_0004611	Cardiovascular measurement	Cardiovascular
0.7614	1.13E-87	1.56E-84	PGS003033	EFO_0004611	low density lipoprotein cholesterol measurement	low density lipoprotein cholesterol measurement	EFO_0004611	Cardiovascular measurement	Cardiovascular	PGS001933	EFO_0004611	low density lipoprotein cholesterol measurement	measurement	EFO_0004611	Cardiovascular measurement	Cardiovascular
0.7614	1.13E-87	1.56E-84	PGS003033	EFO_0004611	low density lipoprotein cholesterol measurement	low density lipoprotein cholesterol measurement	EFO_0004611	Lipid or lipoprotein measurement	Metabolic	PGS001933	EFO_0004611	low density lipoprotein cholesterol measurement	measurement	EFO_0004611	Lipid or lipoprotein measurement	Metabolic
0.7614	1.13E-87	1.56E-84	PGS003033	EFO_0004611	low density lipoprotein cholesterol measurement	low density lipoprotein cholesterol measurement	EFO_0004611	Cardiovascular measurement	Cardiovascular	PGS001933	EFO_0004611	low density lipoprotein cholesterol measurement	measurement	EFO_0004611	Lipid or lipoprotein measurement	Metabolic
0.7613	1.16E-87	1.60E-84	PGS001479	EFO_0006930	brain volume measurement|neuroimaging measurement	brain volume measurement	EFO_0006930	Other measurement	Neurological	PGS001481	EFO_0006930	measurement	brain volume measurement	EFO_0006930	Other measurement	Neurological
0.7612	1.27E-87	1.76E-84	PGS002170	EFO_0004468	glucose measurement	glucose measurement	EFO_0004468	Other measurement	Metabolic	PGS002645	EFO_0004468	glucose measurement	glucose measurement	EFO_0004468	Other measurement	Metabolic
0.7611	1.37E-87	1.90E-84	PGS000310	EFO_0004611	low density lipoprotein cholesterol measurement	low density lipoprotein cholesterol measurement	EFO_0004611	Cardiovascular measurement	Cardiovascular	PGS000672	EFO_0004615	apolipoprotein B measurement	apolipoprotein B measurement	EFO_0004615	Lipid or lipoprotein measurement	Metabolic
0.7611	1.37E-87	1.90E-84	PGS000310	EFO_0004611	low density lipoprotein cholesterol measurement	low density lipoprotein cholesterol measurement	EFO_0004611	Lipid or lipoprotein measurement	Metabolic	PGS000672	EFO_0004615	apolipoprotein B measurement	apolipoprotein B measurement	EFO_0004615	Lipid or lipoprotein measurement	Metabolic
0.7611	1.42E-87	1.96E-84	PGS000317	EFO_0000305	breast carcinoma	breast carcinoma	EFO_0000305	Cancer	Neoplasms	PGS000045	EFO_0000305	breast carcinoma	breast carcinoma	EFO_0000305	Cancer	Neoplasms
0.7611	1.43E-87	1.97E-84	PGS002524	MONDO_0005148	type 2 diabetes mellitus	type 2 diabetes mellitus	MONDO_0005148	Digestive system disorder	Gastrointestinal	PGS002400	EFO_0004468	glucose measurement	glucose measurement	EFO_0004468	Other measurement	Metabolic
0.7611	1.43E-87	1.97E-84	PGS002524	MONDO_0005148	type 2 diabetes mellitus	type 2 diabetes mellitus	MONDO_0005148	Metabolic disorder	Metabolic	PGS002400	EFO_0004468	glucose measurement	glucose measurement	EFO_0004468	Other measurement	Metabolic
0.7611	1.44E-87	1.99E-84	PGS000001	EFO_0000305	breast carcinoma	breast carcinoma	EFO_0000305	Cancer	Neoplasms	PGS000488	EFO_0000305	breast carcinoma	breast carcinoma	EFO_0000305	Cancer	Neoplasms
0.761	1.49E-87	2.05E-84	PGS000312	EFO_0004530	triglyceride measurement	triglyceride measurement	EFO_0004530	Lipid or lipoprotein measurement	Metabolic	PGS002572	EFO_0004530	triglyceride measurement	triglyceride measurement	EFO_0004530	Lipid or lipoprotein measurement	Metabolic
0.761	1.53E-87	2.10E-84	PGS001778	EFO_0000305	breast carcinoma	breast carcinoma	EFO_0000305	Cancer	Neoplasms	PGS000479	EFO_0000305	breast carcinoma	breast carcinoma	EFO_0000305	Cancer	Neoplasms
0.761	1.55E-87	2.13E-84	PGS000888	EFO_0004611	low density lipoprotein cholesterol measurement	low density lipoprotein cholesterol measurement	EFO_0004611	Cardiovascular measurement	Cardiovascular	PGS000889	EFO_0004611	low density lipoprotein cholesterol measurement	measurement	EFO_0004611	Cardiovascular measurement	Cardiovascular
0.761	1.55E-87	2.13E-84	PGS000888	EFO_0004611	low density lipoprotein cholesterol measurement	low density lipoprotein cholesterol measurement	EFO_0004611	Lipid or lipoprotein measurement	Metabolic	PGS000889	EFO_0004611	low density lipoprotein cholesterol measurement	measurement	EFO_0004611	Cardiovascular measurement	Cardiovascular
0.761	1.55E-87	2.13E-84	PGS000888	EFO_0004611	low density lipoprotein cholesterol measurement	low density lipoprotein cholesterol measurement	EFO_0004611	Cardiovascular measurement	Cardiovascular	PGS000889	EFO_0004611	low density lipoprotein cholesterol measurement	measurement	EFO_0004611	Lipid or lipoprotein measurement	Metabolic
0.761	1.55E-87	2.13E-84	PGS000888	EFO_0004611	low density lipoprotein cholesterol measurement	low density lipoprotein cholesterol measurement	EFO_0004611	Lipid or lipoprotein measurement	Metabolic	PGS000889	EFO_0004611	low density lipoprotein cholesterol measurement	measurement	EFO_0004611	Lipid or lipoprotein measurement	Metabolic
0.761	1.58E-87	2.17E-84	PGS000525	EFO_0000305	breast carcinoma	breast carcinoma	EFO_0000305	Cancer	Neoplasms	PGS000505	EFO_0000305	breast carcinoma	breast carcinoma	EFO_0000305	Cancer	Neoplasms
0.761	1.58E-87	2.17E-84	PGS000526	EFO_0000305	breast carcinoma	breast carcinoma	EFO_0000305	Cancer	Neoplasms	PGS000505	EFO_0000305	breast carcinoma	breast carcinoma	EFO_0000305	Cancer	Neoplasms
0.761	1.58E-87	2.17E-84	PGS000525	EFO_0000305	breast carcinoma	breast carcinoma	EFO_0000305	Cancer	Neoplasms	PGS000506	EFO_0000305	breast carcinoma	breast carcinoma	EFO_0000305	Cancer	Neoplasms
0.761	1.58E-87	2.17E-84	PGS000506	EFO_0000305	breast carcinoma	breast carcinoma	EFO_0000305	Cancer	Neoplasms	PGS000526	EFO_0000305	breast carcinoma	breast carcinoma	EFO_0000305	Cancer	Neoplasms
0.761	1.60E-87	2.19E-84	PGS001484	EFO_0006930	brain volume measurement|neuroimaging measurement	brain volume measurement	EFO_0006930	Other measurement	Neurological	PGS001473	EFO_0006930	measurement	brain volume measurement	EFO_0006930	Other measurement	Neurological
0.7609	1.61E-87	2.22E-84	PGS002575	EFO_0004343	waist-hip ratio	waist-hip ratio	EFO_0004343	Body measurement	Body measurement	PGS002477	EFO_0004343	waist-hip ratio	waist-hip ratio	EFO_0004343	Body measurement	Body measurement
0.7609	1.64E-87	2.25E-84	PGS001665	EFO_0006930	brain volume measurement|neuroimaging measurement	brain volume measurement	EFO_0006930	Other measurement	Neurological	PGS001666	EFO_0006930	measurement	brain volume measurement	EFO_0006930	Other measurement	Neurological
-0.7608	1.80E-87	2.48E-84	PGS002548	EFO_0004612	high density lipoprotein cholesterol measurement	high density lipoprotein cholesterol measurement	EFO_0004612	Lipid or lipoprotein measurement	Metabolic	PGS000825	EFO_0004612	high density lipoprotein cholesterol measurement	measurement	EFO_0004612	Lipid or lipoprotein measurement	Metabolic
0.7607	1.92E-87	2.64E-84	PGS001219	EFO_0004527	mean corpuscular hemoglobin	mean corpuscular hemoglobin	EFO_0004527	Hematological measurement	Hematological	PGS002207	EFO_0004526	mean corpuscular volume	mean corpuscular volume	EFO_0004526	Hematological measurement	Hematological
0.7607	1.95E-87	2.68E-84	PGS002462	EFO_0005091	monocyte count	monocyte count	EFO_0005091	Hematological measurement	Hematological	PGS002609	EFO_0005091	monocyte count	monocyte count	EFO_0005091	Hematological measurement	Hematological
0.7607	1.97E-87	2.70E-84	PGS001488	EFO_0006930	brain volume measurement|neuroimaging measurement	brain volume measurement	EFO_0006930	Other measurement	Neurological	PGS001673	EFO_0006930	measurement	brain volume measurement	EFO_0006930	Other measurement	Neurological
0.7606	2.05E-87	2.81E-84	PGS001958	EFO_0005093	hip circumference	hip circumference	EFO_0005093	Body measurement	Body measurement	PGS002201	EFO_0004342	waist circumference	waist circumference	EFO_0004342	Body measurement	Body measurement
0.7606	2.07E-87	2.84E-84	PGS002844	EFO_0004340	body mass index	body mass index	EFO_0004340	Body measurement	Body measurement	PGS002169	EFO_0005409	fat body mass	fat body mass	EFO_0005409	Other measurement	Body measurement
0.7606	2.18E-87	2.98E-84	PGS002501	EFO_0004541	HbA1c measurement	HbA1c measurement	EFO_0004541	Hematological measurement	Hematological	PGS002547	EFO_0004468	glucose measurement	glucose measurement	EFO_0004468	Other measurement	Metabolic
0.7605	2.29E-87	3.14E-84	PGS000643	MONDO_0018906	follicular lymphoma	follicular lymphoma	MONDO_0018906	Immune system disorder	Immunological	PGS000644	MONDO_0018906	follicular lymphoma	follicular lymphoma	MONDO_0018906	Cancer	Neoplasms
0.7605	2.29E-87	3.14E-84	PGS000643	MONDO_0018906	follicular lymphoma	follicular lymphoma	MONDO_0018906	Cancer	Neoplasms	PGS000644	MONDO_0018906	follicular lymphoma	follicular lymphoma	MONDO_0018906	Cancer	Neoplasms
0.7605	2.29E-87	3.14E-84	PGS000643	MONDO_0018906	follicular lymphoma	follicular lymphoma	MONDO_0018906	Immune system disorder	Immunological	PGS000644	MONDO_0018906	follicular lymphoma	follicular lymphoma	MONDO_0018906	Immune system disorder	Immunological
0.7605	2.29E-87	3.14E-84	PGS000643	MONDO_0018906	follicular lymphoma	follicular lymphoma	MONDO_0018906	Cancer	Neoplasms	PGS000644	MONDO_0018906	follicular lymphoma	follicular lymphoma	MONDO_0018906	Immune system disorder	Immunological
0.7605	2.31E-87	3.16E-84	PGS001878	EFO_0008518	polymyalgia rheumatica	polymyalgia rheumatica	EFO_0008518	Other disease	Immunological	PGS001875	EFO_0000685	rheumatoid arthritis	rheumatoid arthritis	EFO_0000685	Immune system disorder	Immunological
0.7604	2.47E-87	3.39E-84	PGS002501	EFO_0004541	HbA1c measurement	HbA1c measurement	EFO_0004541	Hematological measurement	Hematological	PGS002400	EFO_0004468	glucose measurement	glucose measurement	EFO_0004468	Other measurement	Metabolic
0.7604	2.51E-87	3.44E-84	PGS002555	EFO_0004705	hypothyroidism	hypothyroidism	EFO_0004705	Other disease	Endocrine	PGS002604	EFO_0004705	hypothyroidism	hypothyroidism	EFO_0004705	Other disease	Endocrine
0.7604	2.54E-87	3.48E-84	PGS001065	EFO_0008111	diet measurement	diet measurement	EFO_0008111	Other measurement	Activities	PGS002067	EFO_0001060	celiac disease	celiac disease	EFO_0001060	Digestive system disorder	Gastrointestinal
0.7604	2.54E-87	3.48E-84	PGS001065	EFO_0008111	diet measurement	diet measurement	EFO_0008111	Other measurement	Activities	PGS002067	EFO_0001060	celiac disease	celiac disease	EFO_0001060	Immune system disorder	Immunological
0.7604	2.56E-87	3.50E-84	PGS000335	EFO_0000305	breast carcinoma	breast carcinoma	EFO_0000305	Cancer	Neoplasms	PGS000503	EFO_0000305	breast carcinoma	breast carcinoma	EFO_0000305	Cancer	Neoplasms
0.7604	2.57E-87	3.52E-84	PGS000537	EFO_0000305	breast carcinoma	breast carcinoma	EFO_0000305	Cancer	Neoplasms	PGS000529	EFO_0000305	breast carcinoma	breast carcinoma	EFO_0000305	Cancer	Neoplasms
0.7604	2.59E-87	3.55E-84	PGS002206	EFO_0004527	mean corpuscular hemoglobin	mean corpuscular hemoglobin	EFO_0004527	Hematological measurement	Hematological	PGS002656	EFO_0004527	mean corpuscular hemoglobin	mean corpuscular hemoglobin	EFO_0004527	Hematological measurement	Hematological
0.7603	2.64E-87	3.61E-84	PGS000065	EFO_0004611	low density lipoprotein cholesterol measurement	low density lipoprotein cholesterol measurement	EFO_0004611	Lipid or lipoprotein measurement	Metabolic	PGS001889	EFO_0004615	apolipoprotein B measurement	apolipoprotein B measurement	EFO_0004615	Lipid or lipoprotein measurement	Metabolic
0.7603	2.64E-87	3.61E-84	PGS000065	EFO_0004611	low density lipoprotein cholesterol measurement	low density lipoprotein cholesterol measurement	EFO_0004611	Cardiovascular measurement	Cardiovascular	PGS001889	EFO_0004615	apolipoprotein B measurement	apolipoprotein B measurement	EFO_0004615	Lipid or lipoprotein measurement	Metabolic
0.7602	2.92E-87	3.99E-84	PGS002632	EFO_0003923	bone density	bone density	EFO_0003923	Other measurement	Skeletal	PGS001957	EFO_0005654	velocity of sound measurement	velocity of sound measurement	EFO_0005654	Other measurement	Others
0.7601	3.22E-87	4.40E-84	PGS000503	EFO_0000305	breast carcinoma	breast carcinoma	EFO_0000305	Cancer	Neoplasms	PGS000531	EFO_0000305	breast carcinoma	breast carcinoma	EFO_0000305	Cancer	Neoplasms
0.76	3.48E-87	4.76E-84	PGS000539	EFO_0000305	breast carcinoma	breast carcinoma	EFO_0000305	Cancer	Neoplasms	PGS000537	EFO_0000305	breast carcinoma	breast carcinoma	EFO_0000305	Cancer	Neoplasms

0.76	3.59E-87	4.90E-84	PGS001108	EFO_0007777	base metabolic rate measurement	base metabolic rate measurement	EFO_0007777	Other measurement	Metabolic	PGS002168	EFO_0004995	lean body mass	lean body mass	EFO_0004995	Other measurement	Skeletal
0.7599	3.74E-87	5.11E-84	PGS000540	EFO_0000305	breast carcinoma	breast carcinoma	EFO_0000305	Cancer	Neoplasms	PGS000506	EFO_0000305	breast carcinoma	breast carcinoma	EFO_0000305	Cancer	Neoplasms
0.7599	3.74E-87	5.11E-84	PGS002502	EFO_0004339	body height	body height	EFO_0004339	Body measurement	Body measurement	PGS002551	EFO_0004339	body height	body height	EFO_0004339	Body measurement	Body measurement
0.7599	3.74E-87	5.11E-84	PGS000540	EFO_0000305	breast carcinoma	breast carcinoma	EFO_0000305	Cancer	Neoplasms	PGS000505	EFO_0000305	breast carcinoma	breast carcinoma	EFO_0000305	Cancer	Neoplasms
0.7599	3.86E-87	5.26E-84	PGS001406	EFO_0010700	reticulocyte measurement	reticulocyte measurement	EFO_0010700	Hematological measurement	Hematological	PGS001976	EFO_0007986	reticulocyte count	reticulocyte count	EFO_0007986	Hematological measurement	Hematological
0.7598	4.04E-87	5.51E-84	PGS000528	EFO_0000305	breast carcinoma	breast carcinoma	EFO_0000305	Cancer	Neoplasms	PGS000500	EFO_0000305	breast carcinoma	breast carcinoma	EFO_0000305	Cancer	Neoplasms
0.7598	4.09E-87	5.58E-84	PGS001219	EFO_0004527	mean corpuscular hemoglobin	mean corpuscular hemoglobin	EFO_0004527	Hematological measurement	Hematological	PGS000101	EFO_0004526	mean corpuscular volume	mean corpuscular volume	EFO_0004526	Hematological measurement	Hematological
0.7598	4.13E-87	5.63E-84	PGS001529	EFO_0007772	calcaneal bone quantitative ultrasound measurement	calcaneal bone quantitative ultrasound measurement	EFO_0007772	Other measurement	Skeletal	PGS001530	EFO_0007772	measurement	measurement	EFO_0007772	Other measurement	Skeletal
0.7597	4.43E-87	6.03E-84	PGS000008	EFO_1000649	estrogen-receptor positive breast cancer	estrogen-receptor positive breast cancer	EFO_1000649	Cancer	Neoplasms	PGS000009	EFO_1000650	estrogen-receptor negative breast cancer	cancer	EFO_1000650	Cancer	Neoplasms
0.7597	4.52E-87	6.15E-84	PGS000773	EFO_0000305	breast carcinoma	breast carcinoma	EFO_0000305	Cancer	Neoplasms	PGS000045	EFO_0000305	breast carcinoma	breast carcinoma	EFO_0000305	Cancer	Neoplasms
0.7596	4.75E-87	6.48E-84	PGS003249	EFO_0010472	creatine measurement|glomerular filtration rate	creatine measurement	EFO_0010472	Other measurement	Metabolic	PGS003253	EFO_0010472	creatine measurement|glomerular filtration rate	creatine measurement	EFO_0010472	Other measurement	Metabolic
0.7596	5.02E-87	6.83E-84	PGS002336	EFO_0004705	hypothyroidism	hypothyroidism	EFO_0004705	Other disease	Endocrine	PGS000761	EFO_0004705	hypothyroidism	hypothyroidism	EFO_0004705	Other disease	Endocrine
0.7595	5.36E-87	7.30E-84	PGS000549	EFO_0003893	ovarian neoplasm	ovarian neoplasm	EFO_0003893	Cancer	Neoplasms	PGS000068	EFO_0001075	ovarian carcinoma	ovarian carcinoma	EFO_0001075	Cancer	Neoplasms
0.7594	5.64E-87	7.68E-84	PGS002958	EFO_0004612	high density lipoprotein cholesterol measurement	high density lipoprotein cholesterol measurement	EFO_0004612	Lipid or lipoprotein measurement	Metabolic	PGS002172	EFO_0004612	high density lipoprotein cholesterol measurement	measurement	EFO_0004612	Lipid or lipoprotein measurement	Metabolic
0.7594	5.65E-87	7.69E-84	PGS002524	MONDO_0005148	type 2 diabetes mellitus	type 2 diabetes mellitus	MONDO_0005148	Metabolic disorder	Metabolic	PGS002449	EFO_0004468	glucose measurement	glucose measurement	EFO_0004468	Other measurement	Metabolic
0.7594	5.65E-87	7.69E-84	PGS002524	MONDO_0005148	type 2 diabetes mellitus	type 2 diabetes mellitus	MONDO_0005148	Digestive system disorder	Gastrointestinal	PGS002449	EFO_0004468	glucose measurement	glucose measurement	EFO_0004468	Other measurement	Metabolic
0.7594	5.80E-87	7.89E-84	PGS003184	EFO_0004631	vitamin D measurement	vitamin D measurement	EFO_0004631	Lipid or lipoprotein measurement	Metabolic	PGS003195	EFO_0004631	vitamin D measurement	vitamin D measurement	EFO_0004631	Lipid or lipoprotein measurement	Metabolic
0.7593	6.01E-87	8.18E-84	PGS003038	EFO_0004611	low density lipoprotein cholesterol measurement	low density lipoprotein cholesterol measurement	EFO_0004611	Cardiovascular measurement	Cardiovascular	PGS001933	EFO_0004611	low density lipoprotein cholesterol measurement	measurement	EFO_0004611	Cardiovascular measurement	Cardiovascular
0.7593	6.01E-87	8.18E-84	PGS003038	EFO_0004611	low density lipoprotein cholesterol measurement	low density lipoprotein cholesterol measurement	EFO_0004611	Lipid or lipoprotein measurement	Metabolic	PGS001933	EFO_0004611	low density lipoprotein cholesterol measurement	measurement	EFO_0004611	Cardiovascular measurement	Cardiovascular
0.7593	6.01E-87	8.18E-84	PGS003038	EFO_0004611	low density lipoprotein cholesterol measurement	low density lipoprotein cholesterol measurement	EFO_0004611	Cardiovascular measurement	Cardiovascular	PGS001933	EFO_0004611	low density lipoprotein cholesterol measurement	measurement	EFO_0004611	Lipid or lipoprotein measurement	Metabolic
0.7593	6.01E-87	8.18E-84	PGS003038	EFO_0004611	low density lipoprotein cholesterol measurement	low density lipoprotein cholesterol measurement	EFO_0004611	Lipid or lipoprotein measurement	Metabolic	PGS001933	EFO_0004611	low density lipoprotein cholesterol measurement	measurement	EFO_0004611	Lipid or lipoprotein measurement	Metabolic
0.7593	6.03E-87	8.20E-84	PGS003199	EFO_0004631	vitamin D measurement	vitamin D measurement	EFO_0004631	Lipid or lipoprotein measurement	Metabolic	PGS003195	EFO_0004631	vitamin D measurement	vitamin D measurement	EFO_0004631	Lipid or lipoprotein measurement	Metabolic
0.7593	6.20E-87	8.42E-84	PGS001893	EFO_0004838	calcium measurement	calcium measurement	EFO_0004838	Other measurement	Metabolic	PGS000676	EFO_0004838	calcium measurement	calcium measurement	EFO_0004838	Other measurement	Metabolic
0.7593	6.20E-87	8.42E-84	PGS002322	EFO_0006336	diastolic blood pressure	diastolic blood pressure	EFO_0006336	Other measurement	Cardiovascular	PGS000912	EFO_0006336	diastolic blood pressure	diastolic blood pressure	EFO_0006336	Other measurement	Cardiovascular
0.7593	6.44E-87	8.75E-84	PGS001266	EFO_0003907	deep vein thrombosis	deep vein thrombosis	EFO_0003907	Other trait	Cardiovascular	PGS000961	EFO_1001395	Phlebitis|Thrombophlebitis	Phlebitis	EFO_1001395	Cardiovascular disease	Cardiovascular
0.7591	7.53E-87	1.02E-83	PGS000044	EFO_0001663	prostate carcinoma	prostate carcinoma	EFO_0001663	Cancer	Neoplasms	PGS002791	EFO_0001663	prostate carcinoma	prostate carcinoma	EFO_0001663	Cancer	Neoplasms
-0.759	7.90E-87	1.07E-83	PGS000062	EFO_0004574	total cholesterol measurement	total cholesterol measurement	EFO_0004574	Lipid or lipoprotein measurement	Metabolic	PGS000115	EFO_0004611	low density lipoprotein cholesterol measurement	measurement	EFO_0004611	Cardiovascular measurement	Cardiovascular
-0.759	7.90E-87	1.07E-83	PGS000062	EFO_0004574	total cholesterol measurement	total cholesterol measurement	EFO_0004574	Lipid or lipoprotein measurement	Metabolic	PGS000115	EFO_0004611	low density lipoprotein cholesterol measurement	measurement	EFO_0004611	Lipid or lipoprotein measurement	Metabolic
0.7589	8.71E-87	1.18E-83	PGS000317	EFO_0000305	breast carcinoma	breast carcinoma	EFO_0000305	Cancer	Neoplasms	PGS000775	EFO_1000650	estrogen-receptor negative breast cancer	cancer	EFO_1000650	Cancer	Neoplasms
0.7588	9.61E-87	1.30E-83	PGS002074	HP_0000790	Hematuria	Hematuria	HP_0000790	Other trait	Others	PGS001863	HP_0000790	Hematuria	Hematuria	HP_0000790	Other trait	Others
0.7587	1.01E-86	1.37E-83	PGS000065	EFO_0004611	low density lipoprotein cholesterol measurement	low density lipoprotein cholesterol measurement	EFO_0004611	Lipid or lipoprotein measurement	Metabolic	PGS002654	EFO_0004611	low density lipoprotein cholesterol measurement	measurement	EFO_0004611	Lipid or lipoprotein measurement	Metabolic
0.7587	1.01E-86	1.37E-83	PGS000065	EFO_0004611	low density lipoprotein cholesterol measurement	low density lipoprotein cholesterol measurement	EFO_0004611	Lipid or lipoprotein measurement	Metabolic	PGS002654	EFO_0004611	low density lipoprotein cholesterol measurement	measurement	EFO_0004611	Cardiovascular measurement	Cardiovascular
0.7587	1.01E-86	1.37E-83	PGS000065	EFO_0004611	low density lipoprotein cholesterol measurement	low density lipoprotein cholesterol measurement	EFO_0004611	Cardiovascular measurement	Cardiovascular	PGS002654	EFO_0004611	low density lipoprotein cholesterol measurement	measurement	EFO_0004611	Cardiovascular measurement	Cardiovascular
0.7587	1.01E-86	1.37E-83	PGS000065	EFO_0004611	low density lipoprotein cholesterol measurement	low density lipoprotein cholesterol measurement	EFO_0004611	Cardiovascular measurement	Cardiovascular	PGS002654	EFO_0004611	low density lipoprotein cholesterol measurement	measurement	EFO_0004611	Lipid or lipoprotein measurement	Metabolic
0.7586	1.12E-86	1.52E-83	PGS002874	EFO_0004458	C-reactive protein measurement	C-reactive protein measurement	EFO_0004458	Inflammatory measurement	Immunological	PGS002884	EFO_0004458	C-reactive protein measurement	C-reactive protein measurement	EFO_0004458	Inflammatory measurement	Immunological
0.7586	1.13E-86	1.53E-83	PGS003030	EFO_0004611	low density lipoprotein cholesterol measurement	low density lipoprotein cholesterol measurement	EFO_0004611	Cardiovascular measurement	Cardiovascular	PGS002605	EFO_0004611	low density lipoprotein cholesterol measurement	measurement	EFO_0004611	Cardiovascular measurement	Cardiovascular
0.7586	1.13E-86	1.53E-83	PGS003030	EFO_0004611	low density lipoprotein cholesterol measurement	low density lipoprotein cholesterol measurement	EFO_0004611	Lipid or lipoprotein measurement	Metabolic	PGS002605	EFO_0004611	low density lipoprotein cholesterol measurement	measurement	EFO_0004611	Lipid or lipoprotein measurement	Metabolic
0.7586	1.13E-86	1.53E-83	PGS003030	EFO_0004611	low density lipoprotein cholesterol measurement	low density lipoprotein cholesterol measurement	EFO_0004611	Cardiovascular measurement	Cardiovascular	PGS002605	EFO_0004611	low density lipoprotein cholesterol measurement	measurement	EFO_0004611	Lipid or lipoprotein measurement	Metabolic
0.7586	1.13E-86	1.53E-83	PGS003030	EFO_0004611	low density lipoprotein cholesterol measurement	low density lipoprotein cholesterol measurement	EFO_0004611	Lipid or lipoprotein measurement	Metabolic	PGS002605	EFO_0004611	low density lipoprotein cholesterol measurement	measurement	EFO_0004611	Cardiovascular measurement	Cardiovascular
0.7586	1.14E-86	1.55E-83	PGS002008	EFO_0004503	hematological measurement	hematological measurement	EFO_0004503	Hematological measurement	Hematological	PGS001990	EFO_0004526	mean corpuscular volume	mean corpuscular volume	EFO_0004526	Hematological measurement	Hematological
0.7585	1.19E-86	1.61E-83	PGS001103	EFO_0007800	body fat percentage	body fat percentage	EFO_0007800	Body measurement	Body measurement	PGS001148	EFO_0005409	fat body mass	fat body mass	EFO_0005409	Other measurement	Body measurement
0.7585	1.20E-86	1.63E-83	PGS001738	EFO_0006930	brain volume measurement|neuroimaging measurement	brain volume measurement	EFO_0006930	Other measurement	Neurological	PGS001723	EFO_0006930	measurement	brain volume measurement	EFO_0006930	Other measurement	Neurological
0.7585	1.23E-86	1.67E-83	PGS000773	EFO_0000305	breast carcinoma	breast carcinoma	EFO_0000305	Cancer	Neoplasms	PGS000488	EFO_0000305	breast carcinoma	breast carcinoma	EFO_0000305	Cancer	Neoplasms
0.7584	1.29E-86	1.74E-83	PGS000888	EFO_0004611	low density lipoprotein cholesterol measurement	low density lipoprotein cholesterol measurement	EFO_0004611	Cardiovascular measurement	Cardiovascular	PGS001933	EFO_0004611	low density lipoprotein cholesterol measurement	measurement	EFO_0004611	Lipid or lipoprotein measurement	Metabolic
0.7584	1.29E-86	1.74E-83	PGS000888	EFO_0004611	low density lipoprotein cholesterol measurement	low density lipoprotein cholesterol measurement	EFO_0004611	Lipid or lipoprotein measurement	Metabolic	PGS001933	EFO_0004611	low density lipoprotein cholesterol measurement	measurement	EFO_0004611	Cardiovascular measurement	Cardiovascular
0.7584	1.29E-86	1.74E-83	PGS000888	EFO_0004611	low density lipoprotein cholesterol measurement	low density lipoprotein cholesterol measurement	EFO_0004611	Lipid or lipoprotein measurement	Metabolic	PGS001933	EFO_0004611	low density lipoprotein cholesterol measurement	measurement	EFO_0004611	Lipid or lipoprotein measurement	Metabolic
0.7584	1.29E-86	1.74E-83	PGS000888	EFO_0004611	low density lipoprotein cholesterol measurement	low density lipoprotein cholesterol measurement	EFO_0004611	Cardiovascular measurement	Cardiovascular	PGS001933	EFO_0004611	low density lipoprotein cholesterol measurement	measurement	EFO_0004611	Cardiovascular measurement	Cardiovascular
0.7584	1.29E-86	1.74E-83	PGS000095	EFO_0007986	reticulocyte count	reticulocyte count	EFO_0007986	Hematological measurement	Hematological	PGS000169	EFO_0007986	reticulocyte count	reticulocyte count	EFO_0007986	Hematological measurement	Hematological
0.7584	1.31E-86	1.77E-83	PGS000951	EFO_0004514	bone quantitative ultrasound measurement	bone quantitative ultrasound measurement	EFO_0004514	Other measurement	Skeletal	PGS001403	EFO_0009270	heel bone mineral density	heel bone mineral density	EFO_0009270	Other measurement	Skeletal
0.7583	1.35E-86	1.83E-83	PGS002206	EFO_0004527	mean corpuscular hemoglobin	mean corpuscular hemoglobin	EFO_0004527	Hematological measurement	Hematological	PGS002411	EFO_0004527	mean corpuscular hemoglobin	mean corpuscular hemoglobin	EFO_0004527	Hematological measurement	Hematological
0.7583	1.36E-86	1.84E-83	PGS001954	EFO_0004612	high density lipoprotein cholesterol measurement	high density lipoprotein cholesterol measurement	EFO_0004612	Lipid or lipoprotein measurement	Metabolic	PGS000686	EFO_0004612	high density lipoprotein cholesterol measurement	measurement	EFO_0004612	Lipid or lipoprotein measurement	Metabolic
0.7583	1.43E-86	1.93E-83	PGS002831	EFO_0007878	alcohol consumption measurement	alcohol consumption measurement	EFO_0007878	Other measurement	Psychiatric	PGS002832	EFO_0007878	alcohol consumption measurement	alcohol consumption measurement	EFO_0007878	Other measurement	Psychiatric
0.7583	1.46E-86	1.97E-83	PGS003114	MONDO_0005148	type 2 diabetes mellitus	type 2 diabetes mellitus	MONDO_0005148	Metabolic disorder	Metabolic	PGS003099	MONDO_0005148	type 2 diabetes mellitus	type 2 diabetes mellitus	MONDO_0005148	Metabolic disorder	Metabolic
0.7583	1.46E-86	1.97E-83	PGS003114	MONDO_0005148	type 2 diabetes mellitus	type 2 diabetes mellitus	MONDO_0005148	Digestive system disorder	Gastrointestinal	PGS003099	MONDO_0005148	type 2 diabetes mellitus	type 2 diabetes mellitus	MONDO_0005148	Digestive system disorder	Gastrointestinal
0.7583	1.46E-86	1.97E-83	PGS003114	MONDO_0005148	type 2 diabetes mellitus	type 2 diabetes mellitus	MONDO_0005148	Digestive system disorder	Gastrointestinal	PGS003099	MONDO_0005148	type 2 diabetes mellitus	type 2 diabetes mellitus	MONDO_0005148	Metabolic disorder	Metabolic
0.7583	1.46E-86	1.97E-83	PGS003114	MONDO_0005148	type 2 diabetes mellitus	type 2 diabetes mellitus	MONDO_0005148	Metabolic disorder	Metabolic	PGS003099	MONDO_0005148	type 2 diabetes mellitus	type 2 diabetes mellitus	MONDO_0005148	Digestive system disorder	Gastrointestinal
0.7582	1.50E-86	2.02E-83	PGS001200	EFO_0004584	mean platelet volume	mean platelet volume	EFO_0004584	Hematological measurement	Hematological	PGS000179	EFO_0004584	mean platelet volume	mean platelet volume	EFO_0004584	Hematological measurement	Hematological
0.7582	1.57E-86	2.12E-83	PGS000310	EFO_0004611	low density lipoprotein cholesterol measurement	low density lipoprotein cholesterol measurement	EFO_0004611	Cardiovascular measurement	Cardiovascular	PGS000688	EFO_0004611	low density lipoprotein cholesterol measurement	measurement	EFO_0004611	Lipid or lipoprotein measurement	Metabolic
0.7582	1.57E-86	2.12E-83	PGS000310	EFO_0004611	low density lipoprotein cholesterol measurement	low density lipoprotein cholesterol measurement	EFO_0004611	Lipid or lipoprotein measurement	Metabolic	PGS000688	EFO_0004611	low density lipoprotein cholesterol measurement	measurement	EFO_0004611	Cardiovascular measurement	Cardiovascular
0.7582	1.57E-86	2.12E-83	PGS000310	EFO_0004611	low density lipoprotein cholesterol measurement	low density lipoprotein cholesterol measurement	EFO_0004611	Cardiovascular measurement	Cardiovascular	PGS000688	EFO_0004611	low density lipoprotein cholesterol measurement	measurement	EFO_0004611	Cardiovascular measurement	Cardiovascular
0.7582	1.57E-86	2.12E-83	PGS000310	EFO_0004611	low density lipoprotein cholesterol measurement	low density lipoprotein cholesterol measurement	EFO_0004611	Lipid or lipoprotein measurement	Metabolic	PGS000688	EFO_0004611	low density lipoprotein cholesterol measurement	measurement	EFO_0004611	Lipid or lipoprotein measurement	Metabolic
0.7581	1.63E-86	2.20E-83	PGS000102	EFO_0005091	monocyte count	monocyte count	EFO_0005091	Hematological measurement	Hematological	PGS001968	EFO_0005091	monocyte count	monocyte count	EFO_0005091	Hematological measurement	Hematological
0.7581	1.64E-86	2.21E-83	PGS001240	EFO_0004305	erythrocyte count	erythrocyte count	EFO_0004305	Hematological measurement	Hematological	PGS002123	EFO_0004305	erythrocyte count	erythrocyte count	EFO_0004305	Hematological measurement	Hematological
0.7581	1.65E-86	2.23E-83	PGS001220	EFO_0004526	mean corpuscular volume	mean corpuscular volume	EFO_0004526	Hematological measurement	Hematological	PGS001989	EFO_0004527	mean corpuscular hemoglobin	mean corpuscular hemoglobin	EFO_0004527	Hematological measurement	Hematological
0.7581	1.67E-86	2.25E-83	PGS001968	EFO_0005091	monocyte count	monocyte count	EFO_0005091	Hematological measurement	Hematological	PGS002658	EFO_0005091	monocyte count	monocyte count	EFO_0005091	Hematological measurement	Hematological
0.7581	1.69E-86	2.28E-83	PGS000921	EFO_0004340	body mass index	body mass index	EFO_0004340	Body measurement	Body measurement	PGS002849	EFO_0004340	body mass index	body mass index	EFO_0004340	Body measurement	Body measurement
0.758	1.75E-86	2.35E-83	PGS001228	EFO_0004340	body mass index	body mass index	EFO_0004340	Body measurement	Body measurement	PGS001148	EFO_0005409	fat body mass	fat body mass	EFO_0005409	Other measurement	Body measurement
0.758	1.76E-86	2.38E-83	PGS000504	EFO_0000305	breast carcinoma	breast carcinoma	EFO_0000305	Cancer	Neoplasms	PGS000007	EFO_0000305	breast carcinoma	breast carcinoma	EFO_0000305	Cancer	Neoplasms
0.758	1.84E-86	2.48E-83	PGS002524	MONDO_0005148	type 2 diabetes mellitus	type 2 diabetes mellitus	MONDO_0005148	Metabolic disorder	Metabolic	PGS002547	EFO_0004468	glucose measurement	glucose measurement	EFO_0004468	Other measurement	Metabolic
0.758	1.84E-86	2.48E-83	PGS002524	MONDO_0005148	type 2 diabetes mellitus	type 2 diabetes mellitus	MONDO_0005148	Digestive system disorder	Gastrointestinal	PGS002547	EFO_0004468	glucose measurement	glucose measurement	EFO_0004468	Other measurement	Metabolic
0.7579	1.93E-86	2.60E-83	PGS002150	EFO_0004611	low density lipoprotein cholesterol measurement	low density lipoprotein cholesterol measurement	EFO_0004611	Cardiovascular measurement	Cardiovascular	PGS002764	HP_0003124	Hypercholesterolemia	Hypercholesterolemia	HP_0003124	Other trait	Metabolic
0.7579	1.93E-86	2.60E-83	PGS002150	EFO_0004611	low density lipoprotein cholesterol measurement	low density lipoprotein cholesterol measurement	EFO_0004611	Lipid or lipoprotein measurement	Metabolic	PGS002764	HP_0003124	Hypercholesterolemia	Hypercholesterolemia	HP_0003124	Other trait	Metabolic
0.7579	1.97E-86	2.65E-83	PGS000400	EFO_0009259	skin carcinoma	skin carcinoma	EFO_0009259	Cancer	Neoplasms	PGS000441	MONDO_0002898	skin cancer	skin cancer	MONDO_0002898	Cancer	Neoplasms
0.7578	2.06E-86	2.77E-83	PGS003200	EFO_0004631	vitamin D measurement	vitamin D measurement	EFO_0004631	Lipid or lipoprotein measurement	Metabolic	PGS003191	EFO_0004631	vitamin D measurement	vitamin D measurement	EFO_0004631	Lipid or lipoprotein measurement	Metabolic
0.7578	2.09E-86	2.81E-83	PGS000893	EFO_0004611	low density lipoprotein cholesterol measurement	low density lipoprotein cholesterol measurement	EFO_0004611	Lipid or lipoprotein measurement	Metabolic	PGS002605	EFO_0004611	low density lipoprotein cholesterol measurement	measurement	EFO_0004611	Cardiovascular measurement	Cardiovascular
0.7578	2.09E-86	2.81E-83	PGS000893	EFO_0004611	low density lipoprotein cholesterol measurement	low density lipoprotein cholesterol measurement	EFO_0004611	Lipid or lipoprotein measurement	Metabolic	PGS002605	EFO_0004611	low density lipoprotein cholesterol measurement	measurement	EFO_0004611	Lipid or lipoprotein measurement	Metabolic
0.7578	2.09E-86	2.81E-83	PGS000893	EFO_0004611	low density lipoprotein cholesterol measurement	low density lipoprotein cholesterol measurement	EFO_0004611	Cardiovascular measurement	Cardiovascular	PGS002605	EFO_0004611	low density lipoprotein cholesterol measurement	measurement	EFO_0004611	Lipid or lipoprotein measurement	Metabolic
0.7578	2.09E-86	2.81E-83	PGS000893	EFO_0004611	low density lipoprotein cholesterol measurement	low density lipoprotein cholesterol measurement	EFO_0004611	Cardiovascular measurement	Cardiovascular	PGS002605	EFO_0004611	low density lipoprotein cholesterol measurement	measurement	EFO_0004611	Cardiovascular measurement	Cardiovascular
0.7576	2.43E-86	3.27E-83	PGS002894	EFO_0006336	diastolic blood pressure	diastolic blood pressure	EFO_0006336	Other measurement	Cardiovascular	PGS002114	EFO_0006336	diastolic blood pressure	diastolic blood pressure	EFO_0006336	Other measurement	Cardiovascular
0.7576	2.54E-86	3.42E-83	PGS000692	EFO_0010968	phosphate measurement	phosphate measurement	EFO_0010968	Other measurement	Metabolic	PGS002216	EFO_0010968	phosphate measurement	phosphate measurement	EFO_0010968	Other measurement	Metabolic
0.7575	2.63E-86	3.54E-83	PGS003315	EFO_0004617	cystatin C measurement|glomerular filtration rate	cystatin C measurement	EFO_0004617	Other measurement	Metabolic	PGS003313	EFO_0004617	cystatin C measurement|glomerular filtration rate	cystatin C measurement	EFO_0004617	Other measurement	Metabolic
0.7575	2.63E-86	3.54E-83	PGS003316	EFO_0004617	cystatin C measurement|glomerular filtration rate	cystatin C measurement	EFO_0004617	Other measurement	Metabolic	PGS003313	EFO_0004617	cystatin C measurement|glomerular filtration rate	cystatin C measurement	EFO_0004617	Other measurement	Metabolic
0.7575	2.65E-86	3.56E-83	PGS002322	EFO_0006336	diastolic blood pressure	diastolic blood pressure	EFO_0006336	Other measurement	Cardiovascular	PGS002893	EFO_0006336	diastolic blood pressure	diastolic blood pressure	EFO_0006336	Other measurement	Cardiovascular
0.7575	2.79E-86	3.75E-83	PGS000551	EFO_0003893	ovarian neoplasm	ovarian neoplasm	EFO_0003893	Cancer	Neoplasms	PGS000559	EFO_0003893	ovarian neoplasm	ovarian neoplasm	EFO_0003893	Cancer	Neoplasms
0.7575	2.79E-86	3.75E-83	PGS000551	EFO_0003893	ovarian neoplasm	ovarian neoplasm	EFO_0003893	Cancer	Neoplasms	PGS000560	EFO_0003893	ovarian neoplasm	ovarian neoplasm	EFO_0003893	Cancer	Neoplasms
0.7574	2.80E-86	3.75E-83	PGS002781	EFO_0004612	high density lipoprotein cholesterol measurement	high density lipoprotein cholesterol measurement	EFO_0004612	Lipid or lipoprotein measurement	Metabolic	PGS001954	EFO_0004612	high density lipoprotein cholesterol measurement	measurement	EFO_0004612	Lipid or lipoprotein measurement	Metabolic
0.7574	2.91E-86	3.91E-83	PGS000871	EFO_0008001	insulin secretion measurement	insulin secretion measurement	EFO_0008001	Other measurement	Metabolic	PGS002733	MONDO_0005148	type 2 diabetes mellitus	type 2 diabetes mellitus	MONDO_0005148	Metabolic disorder	Metabolic
0.7574	2.91E-86	3.91E-83	PGS000871	EFO_0008001	insulin secretion measurement	insulin secretion measurement	EFO_0008001	Other measurement	Metabolic	PGS002733	MONDO_0005148	type 2 diabetes mellitus	type 2 diabetes mellitus	MONDO_0005148	Digestive system disorder	Gastrointestinal
0.7573	3.16E-86	4.24E-83	PGS000479	EFO_0000305	breast carcinoma	breast carcinoma	EFO_0000305	Cancer	Neoplasms	PGS000540	EFO_0000305	breast carcinoma	breast carcinoma	EFO_0000305	Cancer	Neoplasms
0.7573	3.16E-86	4.24E-83	PGS000471	MONDO_0004641	skin carcinoma in situ	skin carcinoma in situ	MONDO_0004641	Cancer	Neoplasms	PGS001072	EFO_0008006	skin aging measurement	skin aging measurement	EFO_0008006	Other measurement	Dermatological
0.7572	3.34E-86	4.47E-83	PGS001528	EFO_0007986	reticulocyte count	reticulocyte count	EFO_0007986	Hematological measurement	Hematological	PGS000988	EFO_0010700	reticulocyte measurement	reticulocyte measurement	EFO_0010700	Hematological measurement	Hematological
-0.7572	3.35E-86	4.49E-83	PGS002632	EFO_0003923	bone density	bone density	EFO_0003923	Other measurement	Skeletal	PGS002768	EFO_0003882	osteoporosis	osteoporosis	EFO_0003882	Other disease	Skeletal
0.7572	3.37E-86	4.52E-83	PGS000677	EFO_0004574	total cholesterol measurement	total cholesterol measurement	EFO_0004574	Lipid or lipoprotein measurement	Metabolic	PGS001933	EFO_0004611	low density lipoprotein cholesterol measurement	measurement	EFO_0004611	Cardiovascular measurement	Cardiovascular
0.7572	3.37E-86	4.52E-83	PGS000677	EFO_0004574	total cholesterol measurement	total cholesterol measurement	EFO_0004574	Lipid or lipoprotein measurement	Metabolic	PGS001933	EFO_0004611	low density lipoprotein cholesterol measurement	measurement	EFO_0004611	Lipid or lipoprotein measurement	Metabolic
0.7572	3.39E-86	4.55E-83	PGS000522	EFO_0000305	breast carcinoma	breast carcinoma	EFO_0000305	Cancer	Neoplasms	PGS000524	EFO_0000305	breast carcinoma	breast carcinoma	EFO_0000305	Cancer	Neoplasms
0.7571	3.74E-86	5.01E-83	PGS001900	EFO_0006336	diastolic blood pressure	diastolic blood pressure	EFO_0006336	Other measurement	Cardiovascular	PGS000912	EFO_0006336	diastolic blood pressure	diastolic blood pressure	EFO_0006336	Other measurement	Cardiovascular
-0.7571	3.78E-86	5.06E-83	PGS001987	EFO_0007825	balding measurement	balding measurement	EFO_0007825	Other measurement	Activities	PGS002314	EFO_0007825	balding measurement	balding measurement	EFO_0007825	Other measurement	Activities
0.757	3.89E-86	5.20E-83	PGS001478	EFO_0006930	brain volume measurement|neuroimaging measurement	brain volume measurement	EFO_0006930	Other measurement	Neurological	PGS001489	EFO_0006930	measurement	brain volume measurement	EFO_0006930	Other measurement	Neurological
0.7569	4.47E-86	5.98E-83	PGS001670	EFO_0006930	brain volume measurement|neuroimaging measurement	brain volume measurement	EFO_0006930	Other measurement	Neurological	PGS001450	EFO_0006930	measurement	brain volume measurement	EFO_0006930	Other measurement	Neurological
0.7569	4.52E-86	6.04E-83	PGS000102	EFO_0005091	monocyte count	monocyte count	EFO_0005091	Hematological measurement	Hematological	PGS002341	EFO_0005091	monocyte count	monocyte count	EFO_0005091	Hematological measurement	Hematological
0.7569	4.53E-86	6.06E-83	PGS002541	EFO_0006336	diastolic blood pressure	diastolic blood pressure	EFO_0006336	Other measurement	Cardiovascular	PGS002617	EFO_0006335	systolic blood pressure	systolic blood pressure	EFO_0006335	Other measurement	Cardiovascular
0.7568	4.58E-86	6.13E-83	PGS002102	EFO_0004615	apolipoprotein B measurement	apolipoprotein B measurement	EFO_0004615	Lipid or lipoprotein measurement	Metabolic	PGS002352	EFO_0004574	total cholesterol measurement	total cholesterol measurement	EFO_0004574	Lipid or lipoprotein measurement	Metabolic
0.7568	4.72E-86	6.32E-83	PGS001488	EFO_0006930	brain volume measurement|neuroimaging measurement	brain volume measurement	EFO_0006930	Other measurement	Neurological	PGS001485	EFO_0006930	measurement	brain volume measurement	EFO_0006930	Other measurement	Neurological

0.7568	4.83E-86	6.45E-83	PGS002186	EFO_0005091	monocyte count	monocyte count	EFO_0005091	Hematological measurement	Hematological	PGS002658	EFO_0005091	monocyte count	monocyte count	EFO_0005091	Hematological measurement	Hematological
0.7568	4.86E-86	6.50E-83	PGS002206	EFO_0004527	mean corpuscular hemoglobin	mean corpuscular hemoglobin	EFO_0004527	Hematological measurement	Hematological	PGS001220	EFO_0004526	mean corpuscular volume	mean corpuscular volume	EFO_0004526	Hematological measurement	Hematological
0.7567	4.95E-86	6.62E-83	PGS002889	EFO_0006336	diastolic blood pressure	diastolic blood pressure	EFO_0006336	Other measurement	Cardiovascular	PGS002114	EFO_0006336	diastolic blood pressure	diastolic blood pressure	EFO_0006336	Other measurement	Cardiovascular
0.7567	5.09E-86	6.80E-83	PGS002658	EFO_0005091	monocyte count	monocyte count	EFO_0005091	Hematological measurement	Hematological	PGS001163	EFO_0005091	monocyte count	monocyte count	EFO_0005091	Hematological measurement	Hematological
0.7567	5.17E-86	6.91E-83	PGS001094	EFO_0007822	hair colour measurement	hair colour measurement	EFO_0007822	Other measurement	Dermatological	PGS001092	EFO_0007822	hair colour measurement	hair colour measurement	EFO_0007822	Other measurement	Dermatological
0.7567	5.18E-86	6.92E-83	PGS000758	EFO_0004339	body height	body height	EFO_0004339	Body measurement	Body measurement	PGS002224	EFO_0011011	sitting height measurement	sitting height measurement	EFO_0011011	Other measurement	Skeletal
0.7565	5.80E-86	7.74E-83	PGS000511	EFO_0000305	breast carcinoma	breast carcinoma	EFO_0000305	Cancer	Neoplasms	PGS000008	EFO_1000649	estrogen-receptor positive breast cancer	cancer	EFO_1000649	Cancer	Neoplasms
0.7565	5.85E-86	7.81E-83	PGS000317	EFO_0000305	breast carcinoma	breast carcinoma	EFO_0000305	Cancer	Neoplasms	PGS000046	EFO_1000649	estrogen-receptor positive breast cancer	cancer	EFO_1000649	Cancer	Neoplasms
0.7565	6.14E-86	8.18E-83	PGS002407	EFO_0000537	hypertension	hypertension	EFO_0000537	Cardiovascular disease	Cardiovascular	PGS002535	EFO_0000319	cardiovascular disease	cardiovascular disease	EFO_0000319	Cardiovascular disease	Cardiovascular
-0.7565	6.17E-86	8.22E-83	PGS001414	EFO_0007993	lymphocyte percentage of leukocytes	lymphocyte percentage of leukocytes	EFO_0007993	Hematological measurement	Hematological	PGS002214	EFO_0007990	neutrophil percentage of leukocytes	neutrophil percentage of leukocytes	EFO_0007990	Hematological measurement	Hematological
0.7565	6.22E-86	8.30E-83	PGS000433	MONDO_0002898	skin cancer	skin cancer	MONDO_0002898	Cancer	Neoplasms	PGS000443	MONDO_0002898	skin cancer	skin cancer	MONDO_0002898	Cancer	Neoplasms
0.7564	6.40E-86	8.53E-83	PGS002336	EFO_0004705	hypothyroidism	hypothyroidism	EFO_0004705	Other disease	Endocrine	PGS002312	EFO_0005140	autoimmune disease	autoimmune disease	EFO_0005140	Immune system disorder	Immunological
0.7563	7.02E-86	9.35E-83	PGS000478	EFO_0000305	breast carcinoma	breast carcinoma	EFO_0000305	Cancer	Neoplasms	PGS000479	EFO_0000305	breast carcinoma	breast carcinoma	EFO_0000305	Cancer	Neoplasms
0.7563	7.07E-86	9.42E-83	PGS001485	EFO_0006930	brain volume measurement|neuroimaging measurement	brain volume measurement	EFO_0006930	Other measurement	Neurological	PGS001473	EFO_0006930	measurement	brain volume measurement	EFO_0006930	Other measurement	Neurological
0.7563	7.23E-86	9.62E-83	PGS002541	EFO_0006336	diastolic blood pressure	diastolic blood pressure	EFO_0006336	Other measurement	Cardiovascular	PGS002568	EFO_0006335	systolic blood pressure	systolic blood pressure	EFO_0006335	Other measurement	Cardiovascular
0.7563	7.30E-86	9.72E-83	PGS000511	EFO_0000305	breast carcinoma	breast carcinoma	EFO_0000305	Cancer	Neoplasms	PGS000509	EFO_0000305	breast carcinoma	breast carcinoma	EFO_0000305	Cancer	Neoplasms
0.7562	7.76E-86	1.03E-82	PGS001983	EFO_0004342	waist circumference	waist circumference	EFO_0004342	Body measurement	Body measurement	PGS002133	EFO_0007800	body fat percentage	body fat percentage	EFO_0007800	Body measurement	Body measurement
0.7561	8.37E-86	1.11E-82	PGS003194	EFO_0004631	vitamin D measurement	vitamin D measurement	EFO_0004631	Lipid or lipoprotein measurement	Metabolic	PGS002427	EFO_0004631	vitamin D measurement	vitamin D measurement	EFO_0004631	Lipid or lipoprotein measurement	Metabolic
0.7559	9.49E-86	1.26E-82	PGS003150	EFO_0004530	triglyceride measurement	triglyceride measurement	EFO_0004530	Lipid or lipoprotein measurement	Metabolic	PGS003152	EFO_0004530	triglyceride measurement	triglyceride measurement	EFO_0004530	Lipid or lipoprotein measurement	Metabolic
0.7559	9.70E-86	1.29E-82	PGS002339	EFO_0004527	mean corpuscular hemoglobin	mean corpuscular hemoglobin	EFO_0004527	Hematological measurement	Hematological	PGS002656	EFO_0004527	mean corpuscular hemoglobin	mean corpuscular hemoglobin	EFO_0004527	Hematological measurement	Hematological
0.7558	1.02E-85	1.36E-82	PGS002632	EFO_0003923	bone density	bone density	EFO_0003923	Other measurement	Skeletal	PGS001956	EFO_0007772	measurement	measurement	EFO_0007772	Other measurement	Skeletal
0.7558	1.08E-85	1.43E-82	PGS001984	EFO_0009805	whole body water mass	whole body water mass	EFO_0009805	Other measurement	Body measurement	PGS001159	EFO_0005106	body composition measurement	body composition measurement	EFO_0005106	Body measurement	Body measurement
0.7558	1.10E-85	1.46E-82	PGS001274	EFO_0003882	osteoporosis	osteoporosis	EFO_0003882	Other disease	Skeletal	PGS001273	EFO_0003882	osteoporosis	osteoporosis	EFO_0003882	Other disease	Skeletal
0.7557	1.11E-85	1.47E-82	PGS003235	EFO_0010472	creatine measurement	creatine measurement	EFO_0010472	Other measurement	Metabolic	PGS003230	EFO_0010472	creatine measurement	creatine measurement	EFO_0010472	Other measurement	Metabolic
0.7557	1.14E-85	1.51E-82	PGS001950	EFO_0004995	lean body mass	lean body mass	EFO_0004995	Other measurement	Skeletal	PGS001159	EFO_0005106	body composition measurement	body composition measurement	EFO_0005106	Body measurement	Body measurement
0.7557	1.19E-85	1.57E-82	PGS001264	EFO_0003907	deep vein thrombosis	deep vein thrombosis	EFO_0003907	Other trait	Cardiovascular	PGS001280	EFO_0003827	pulmonary embolism	pulmonary embolism	EFO_0003827	Cardiovascular disease	Cardiovascular
0.7556	1.24E-85	1.65E-82	PGS001989	EFO_0004527	mean corpuscular hemoglobin	mean corpuscular hemoglobin	EFO_0004527	Hematological measurement	Hematological	PGS002558	EFO_0004527	mean corpuscular hemoglobin	mean corpuscular hemoglobin	EFO_0004527	Hematological measurement	Hematological
0.7556	1.24E-85	1.65E-82	PGS003249	EFO_0010472	creatine measurement|glomerular filtration rate	creatine measurement	EFO_0010472	Other measurement	Metabolic	PGS003268	EFO_0010472	creatine measurement|glomerular filtration rate	creatine measurement	EFO_0010472	Other measurement	Metabolic
0.7556	1.25E-85	1.66E-82	PGS000169	EFO_0007986	reticulocyte count	reticulocyte count	EFO_0007986	Hematological measurement	Hematological	PGS000094	EFO_0007986	reticulocyte count	reticulocyte count	EFO_0007986	Hematological measurement	Hematological
0.7554	1.43E-85	1.90E-82	PGS000047	EFO_1000650	estrogen-receptor negative breast cancer	estrogen-receptor negative breast cancer	EFO_1000650	Cancer	Neoplasms	PGS000775	EFO_1000650	estrogen-receptor negative breast cancer	cancer	EFO_1000650	Cancer	Neoplasms
0.7554	1.45E-85	1.92E-82	PGS001477	EFO_0006930	brain volume measurement|neuroimaging measurement	brain volume measurement	EFO_0006930	Other measurement	Neurological	PGS001680	EFO_0006930	measurement	brain volume measurement	EFO_0006930	Other measurement	Neurological
0.7553	1.55E-85	2.06E-82	PGS000888	EFO_0004611	low density lipoprotein cholesterol measurement	low density lipoprotein cholesterol measurement	EFO_0004611	Lipid or lipoprotein measurement	Metabolic	PGS003034	EFO_0004611	low density lipoprotein cholesterol measurement	measurement	EFO_0004611	Lipid or lipoprotein measurement	Metabolic
0.7553	1.55E-85	2.06E-82	PGS000888	EFO_0004611	low density lipoprotein cholesterol measurement	low density lipoprotein cholesterol measurement	EFO_0004611	Cardiovascular measurement	Cardiovascular	PGS003034	EFO_0004611	low density lipoprotein cholesterol measurement	measurement	EFO_0004611	Cardiovascular measurement	Cardiovascular
0.7553	1.55E-85	2.06E-82	PGS000888	EFO_0004611	low density lipoprotein cholesterol measurement	low density lipoprotein cholesterol measurement	EFO_0004611	Lipid or lipoprotein measurement	Metabolic	PGS003034	EFO_0004611	low density lipoprotein cholesterol measurement	measurement	EFO_0004611	Cardiovascular measurement	Cardiovascular
0.7553	1.55E-85	2.06E-82	PGS000888	EFO_0004611	low density lipoprotein cholesterol measurement	low density lipoprotein cholesterol measurement	EFO_0004611	Cardiovascular measurement	Cardiovascular	PGS003034	EFO_0004611	low density lipoprotein cholesterol measurement	measurement	EFO_0004611	Lipid or lipoprotein measurement	Metabolic
0.7552	1.73E-85	2.29E-82	PGS000478	EFO_0000305	breast carcinoma	breast carcinoma	EFO_0000305	Cancer	Neoplasms	PGS000474	EFO_0000305	breast carcinoma	breast carcinoma	EFO_0000305	Cancer	Neoplasms
0.7552	1.74E-85	2.31E-82	PGS001825	MONDO_0003916	overnutrition|overweight body mass index status|obesity	overnutrition	MONDO_0003916	Metabolic disorder	Metabolic	PGS002033	MONDO_0003916	status|obesity	overnutrition	MONDO_0003916	Metabolic disorder	Metabolic
0.7552	1.77E-85	2.34E-82	PGS002353	EFO_0004530	triglyceride measurement	triglyceride measurement	EFO_0004530	Lipid or lipoprotein measurement	Metabolic	PGS003144	EFO_0004530	triglyceride measurement	triglyceride measurement	EFO_0004530	Lipid or lipoprotein measurement	Metabolic
0.7552	1.78E-85	2.35E-82	PGS000036	MONDO_0005148	type 2 diabetes mellitus	type 2 diabetes mellitus	MONDO_0005148	Digestive system disorder	Gastrointestinal	PGS000729	MONDO_0005148	type 2 diabetes mellitus	type 2 diabetes mellitus	MONDO_0005148	Metabolic disorder	Metabolic
0.7552	1.78E-85	2.35E-82	PGS000036	MONDO_0005148	type 2 diabetes mellitus	type 2 diabetes mellitus	MONDO_0005148	Metabolic disorder	Metabolic	PGS000729	MONDO_0005148	type 2 diabetes mellitus	type 2 diabetes mellitus	MONDO_0005148	Metabolic disorder	Metabolic
0.7552	1.78E-85	2.35E-82	PGS000036	MONDO_0005148	type 2 diabetes mellitus	type 2 diabetes mellitus	MONDO_0005148	Digestive system disorder	Gastrointestinal	PGS000729	MONDO_0005148	type 2 diabetes mellitus	type 2 diabetes mellitus	MONDO_0005148	Digestive system disorder	Gastrointestinal
0.7552	1.78E-85	2.35E-82	PGS000036	MONDO_0005148	type 2 diabetes mellitus	type 2 diabetes mellitus	MONDO_0005148	Metabolic disorder	Metabolic	PGS000729	MONDO_0005148	type 2 diabetes mellitus	type 2 diabetes mellitus	MONDO_0005148	Digestive system disorder	Gastrointestinal
0.755	2.00E-85	2.65E-82	PGS002913	EFO_0004329	alcohol drinking	alcohol drinking	EFO_0004329	Biological process	Psychiatric	PGS002909	EFO_0004329	alcohol drinking	alcohol drinking	EFO_0004329	Biological process	Psychiatric
0.755	2.01E-85	2.66E-82	PGS003189	EFO_0004631	vitamin D measurement	vitamin D measurement	EFO_0004631	Lipid or lipoprotein measurement	Metabolic	PGS002355	EFO_0004631	vitamin D measurement	vitamin D measurement	EFO_0004631	Lipid or lipoprotein measurement	Metabolic
0.755	2.03E-85	2.69E-82	PGS000700	EFO_0004531	urate measurement	urate measurement	EFO_0004531	Other measurement	Metabolic	PGS000711	EFO_0004274	gout	gout	EFO_0004274	Metabolic disorder	Metabolic
0.755	2.05E-85	2.72E-82	PGS001671	EFO_0006930	brain volume measurement|neuroimaging measurement	brain volume measurement	EFO_0006930	Other measurement	Neurological	PGS001481	EFO_0006930	measurement	brain volume measurement	EFO_0006930	Other measurement	Neurological
0.7549	2.14E-85	2.83E-82	PGS003315	EFO_0004617	cystatin C measurement|glomerular filtration rate	cystatin C measurement	EFO_0004617	Other measurement	Metabolic	PGS003311	EFO_0004617	cystatin C measurement|glomerular filtration rate	cystatin C measurement	EFO_0004617	Other measurement	Metabolic
0.7549	2.14E-85	2.83E-82	PGS003316	EFO_0004617	cystatin C measurement|glomerular filtration rate	cystatin C measurement	EFO_0004617	Other measurement	Metabolic	PGS003311	EFO_0004617	cystatin C measurement|glomerular filtration rate	cystatin C measurement	EFO_0004617	Other measurement	Metabolic
0.7549	2.20E-85	2.91E-82	PGS000049	EFO_0001663	prostate carcinoma	prostate carcinoma	EFO_0001663	Cancer	Neoplasms	PGS000742	EFO_0001663	prostate carcinoma	prostate carcinoma	EFO_0001663	Cancer	Neoplasms
0.7548	2.40E-85	3.17E-82	PGS003036	EFO_0004611	low density lipoprotein cholesterol measurement	low density lipoprotein cholesterol measurement	EFO_0004611	Cardiovascular measurement	Cardiovascular	PGS003032	EFO_0004611	low density lipoprotein cholesterol measurement	measurement	EFO_0004611	Cardiovascular measurement	Cardiovascular
0.7548	2.40E-85	3.17E-82	PGS003036	EFO_0004611	low density lipoprotein cholesterol measurement	low density lipoprotein cholesterol measurement	EFO_0004611	Cardiovascular measurement	Cardiovascular	PGS003032	EFO_0004611	low density lipoprotein cholesterol measurement	measurement	EFO_0004611	Lipid or lipoprotein measurement	Metabolic
0.7548	2.40E-85	3.17E-82	PGS003036	EFO_0004611	low density lipoprotein cholesterol measurement	low density lipoprotein cholesterol measurement	EFO_0004611	Lipid or lipoprotein measurement	Metabolic	PGS003032	EFO_0004611	low density lipoprotein cholesterol measurement	measurement	EFO_0004611	Cardiovascular measurement	Cardiovascular
0.7548	2.40E-85	3.17E-82	PGS003036	EFO_0004611	low density lipoprotein cholesterol measurement	low density lipoprotein cholesterol measurement	EFO_0004611	Lipid or lipoprotein measurement	Metabolic	PGS003032	EFO_0004611	low density lipoprotein cholesterol measurement	measurement	EFO_0004611	Lipid or lipoprotein measurement	Metabolic
0.7548	2.41E-85	3.18E-82	PGS001663	EFO_0006930	brain volume measurement|neuroimaging measurement	brain volume measurement	EFO_0006930	Other measurement	Neurological	PGS001672	EFO_0006930	measurement	brain volume measurement	EFO_0006930	Other measurement	Neurological
0.7548	2.43E-85	3.21E-82	PGS000433	MONDO_0002898	skin cancer	skin cancer	MONDO_0002898	Cancer	Neoplasms	PGS000402	EFO_0009259	skin carcinoma	skin carcinoma	EFO_0009259	Cancer	Neoplasms
0.7548	2.44E-85	3.23E-82	PGS003031	EFO_0004611	low density lipoprotein cholesterol measurement	low density lipoprotein cholesterol measurement	EFO_0004611	Cardiovascular measurement	Cardiovascular	PGS003032	EFO_0004611	low density lipoprotein cholesterol measurement	measurement	EFO_0004611	Cardiovascular measurement	Cardiovascular
0.7548	2.44E-85	3.23E-82	PGS003031	EFO_0004611	low density lipoprotein cholesterol measurement	low density lipoprotein cholesterol measurement	EFO_0004611	Lipid or lipoprotein measurement	Metabolic	PGS003032	EFO_0004611	low density lipoprotein cholesterol measurement	measurement	EFO_0004611	Lipid or lipoprotein measurement	Metabolic
0.7548	2.44E-85	3.23E-82	PGS003031	EFO_0004611	low density lipoprotein cholesterol measurement	low density lipoprotein cholesterol measurement	EFO_0004611	Cardiovascular measurement	Cardiovascular	PGS003032	EFO_0004611	low density lipoprotein cholesterol measurement	measurement	EFO_0004611	Lipid or lipoprotein measurement	Metabolic
0.7548	2.44E-85	3.23E-82	PGS003031	EFO_0004611	low density lipoprotein cholesterol measurement	low density lipoprotein cholesterol measurement	EFO_0004611	Lipid or lipoprotein measurement	Metabolic	PGS003032	EFO_0004611	low density lipoprotein cholesterol measurement	measurement	EFO_0004611	Cardiovascular measurement	Cardiovascular
0.7547	2.45E-85	3.24E-82	PGS003185	EFO_0004631	vitamin D measurement	vitamin D measurement	EFO_0004631	Lipid or lipoprotein measurement	Metabolic	PGS003191	EFO_0004631	vitamin D measurement	vitamin D measurement	EFO_0004631	Lipid or lipoprotein measurement	Metabolic
0.7547	2.47E-85	3.26E-82	PGS003145	EFO_0004530	triglyceride measurement	triglyceride measurement	EFO_0004530	Lipid or lipoprotein measurement	Metabolic	PGS003144	EFO_0004530	triglyceride measurement	triglyceride measurement	EFO_0004530	Lipid or lipoprotein measurement	Metabolic
0.7547	2.54E-85	3.35E-82	PGS001529	EFO_0007772	calcaneal bone quantitative ultrasound measurement	calcaneal bone quantitative ultrasound measurement	EFO_0007772	Other measurement	Skeletal	PGS001403	EFO_0009270	heel bone mineral density	heel bone mineral density	EFO_0009270	Other measurement	Skeletal
0.7547	2.58E-85	3.40E-82	PGS001951	EFO_0005409	fat body mass	fat body mass	EFO_0005409	Other measurement	Body measurement	PGS002313	EFO_0004340	body mass index	body mass index	EFO_0004340	Body measurement	Body measurement
0.7547	2.61E-85	3.43E-82	PGS000507	EFO_0000305	breast carcinoma	breast carcinoma	EFO_0000305	Cancer	Neoplasms	PGS000503	EFO_0000305	breast carcinoma	breast carcinoma	EFO_0000305	Cancer	Neoplasms
0.7547	2.65E-85	3.50E-82	PGS001485	EFO_0006930	brain volume measurement|neuroimaging measurement	brain volume measurement	EFO_0006930	Other measurement	Neurological	PGS001683	EFO_0006930	measurement	brain volume measurement	EFO_0006930	Other measurement	Neurological
0.7546	2.67E-85	3.51E-82	PGS002193	EFO_0004326	heart rate	heart rate	EFO_0004326	Cardiovascular measurement	Cardiovascular	PGS001233	EFO_0004326	heart rate	heart rate	EFO_0004326	Cardiovascular measurement	Cardiovascular
0.7546	2.74E-85	3.61E-82	PGS001336	EFO_0000305	breast carcinoma	breast carcinoma	EFO_0000305	Cancer	Neoplasms	PGS000483	EFO_0000305	breast carcinoma	breast carcinoma	EFO_0000305	Cancer	Neoplasms
0.7544	3.26E-85	4.29E-82	PGS002974	EFO_0004339	body height	body height	EFO_0004339	Body measurement	Body measurement	PGS002988	EFO_0004339	body height	body height	EFO_0004339	Body measurement	Body measurement
0.7544	3.36E-85	4.43E-82	PGS000391	MONDO_0001672	bronchus cancer|lung cancer|tracheal cancer	bronchus cancer	MONDO_0001672	Cancer	Neoplasms	PGS000880	EFO_0001071	lung carcinoma	lung carcinoma	EFO_0001071	Cancer	Neoplasms
0.7543	3.39E-85	4.45E-82	PGS001984	EFO_0009805	whole body water mass	whole body water mass	EFO_0009805	Other measurement	Body measurement	PGS001167	EFO_0004995	lean body mass	lean body mass	EFO_0004995	Other measurement	Skeletal
-0.7543	3.40E-85	4.47E-82	PGS002768	EFO_0003882	osteoporosis	osteoporosis	EFO_0003882	Other disease	Skeletal	PGS000951	EFO_0004514	bone quantitative ultrasound measurement	measurement	EFO_0004514	Other measurement	Skeletal
0.7543	3.59E-85	4.71E-82	PGS002775	EFO_0001645	coronary artery disease	coronary artery disease	EFO_0001645	Cardiovascular disease	Cardiovascular	PGS002776	EFO_0001645	coronary artery disease	coronary artery disease	EFO_0001645	Cardiovascular disease	Cardiovascular
0.7543	3.60E-85	4.73E-82	PGS001950	EFO_0004995	lean body mass	lean body mass	EFO_0004995	Other measurement	Skeletal	PGS001167	EFO_0004995	lean body mass	lean body mass	EFO_0004995	Other measurement	Skeletal
0.7542	3.70E-85	4.86E-82	PGS000001	EFO_0000305	breast carcinoma	breast carcinoma	EFO_0000305	Cancer	Neoplasms	PGS000487	EFO_0000305	breast carcinoma	breast carcinoma	EFO_0000305	Cancer	Neoplasms
0.7542	3.70E-85	4.86E-82	PGS000001	EFO_0000305	breast carcinoma	breast carcinoma	EFO_0000305	Cancer	Neoplasms	PGS000485	EFO_0000305	breast carcinoma	breast carcinoma	EFO_0000305	Cancer	Neoplasms
0.7542	3.80E-85	4.99E-82	PGS002554	EFO_0000537	hypertension	hypertension	EFO_0000537	Cardiovascular disease	Cardiovascular	PGS002590	EFO_0006336	diastolic blood pressure	diastolic blood pressure	EFO_0006336	Other measurement	Cardiovascular
0.7542	3.86E-85	5.07E-82	PGS000779	MONDO_0004975	Alzheimer disease	Alzheimer disease	MONDO_0004975	Neurological disorder	Neurological	PGS001347	EFO_0009268	family history of Alzheimer’s disease	family history of Alzheimer’s disease	EFO_0009268	Other measurement	Neurological
0.7541	4.02E-85	5.28E-82	PGS001671	EFO_0006930	brain volume measurement|neuroimaging measurement	brain volume measurement	EFO_0006930	Other measurement	Neurological	PGS001477	EFO_0006930	measurement	brain volume measurement	EFO_0006930	Other measurement	Neurological
0.7541	4.02E-85	5.28E-82	PGS002108	EFO_0004574	total cholesterol measurement	total cholesterol measurement	EFO_0004574	Lipid or lipoprotein measurement	Metabolic	PGS000677	EFO_0004574	total cholesterol measurement	total cholesterol measurement	EFO_0004574	Lipid or lipoprotein measurement	Metabolic
0.7541	4.25E-85	5.58E-82	PGS000312	EFO_0004530	triglyceride measurement	triglyceride measurement	EFO_0004530	Lipid or lipoprotein measurement	Metabolic	PGS000066	EFO_0004530	triglyceride measurement	triglyceride measurement	EFO_0004530	Lipid or lipoprotein measurement	Metabolic
0.754	4.35E-85	5.71E-82	PGS001737	EFO_0006930	brain volume measurement|neuroimaging measurement	brain volume measurement	EFO_0006930	Other measurement	Neurological	PGS001721	EFO_0006930	measurement	brain volume measurement	EFO_0006930	Other measurement	Neurological
0.754	4.49E-85	5.89E-82	PGS000488	EFO_0000305	breast carcinoma	breast carcinoma	EFO_0000305	Cancer	Neoplasms	PGS000873	EFO_0000305	breast carcinoma	breast carcinoma	EFO_0000305	Cancer	Neoplasms
0.7539	4.73E-85	6.21E-82	PGS001724	EFO_0006930	brain volume measurement|neuroimaging measurement	brain volume measurement	EFO_0006930	Other measurement	Neurological	PGS001722	EFO_0006930	measurement	brain volume measurement	EFO_0006930	Other measurement	Neurological
0.7539	4.79E-85	6.28E-82	PGS002169	EFO_0005409	fat body mass	fat body mass	EFO_0005409	Other measurement	Body measurement	PGS002859	EFO_0004340	body mass index	body mass index	EFO_0004340	Body measurement	Body measurement
0.7538	5.27E-85	6.91E-82	PGS001929	EFO_0004339	body height	body height	EFO_0004339	Body measurement	Body measurement	PGS002965	EFO_0004339	body height	body height	EFO_0004339	Body measurement	Body measurement
0.7538	5.41E-85	7.08E-82	PGS000540	EFO_0000305	breast carcinoma	breast carcinoma	EFO_0000305	Cancer	Neoplasms	PGS000503	EFO_0000305	breast carcinoma	breast carcinoma	EFO_0000305	Cancer	Neoplasms
0.7537	5.68E-85	7.44E-82	PGS000095	EFO_0007986	reticulocyte count	reticulocyte count	EFO_0007986	Hematological measurement	Hematological	PGS000170	EFO_0007986	reticulocyte count	reticulocyte count	EFO_0007986	Hematological measurement	Hematological
0.7537	5.77E-85	7.55E-82	PGS001484	EFO_0006930	brain volume measurement|neuroimaging measurement	brain volume measurement	EFO_0006930	Other measurement	Neurological	PGS001489	EFO_0006930	measurement	brain volume measurement	EFO_0006930	Other measurement	Neurological
0.7537	5.84E-85	7.64E-82	PGS001925	EFO_0004348	hematocrit	hematocrit	EFO_0004348	Hematological measurement	Hematological	PGS000092	EFO_0004348	hematocrit	hematocrit	EFO_0004348	Hematological measurement	Hematological
0.7537	5.85E-85	7.66E-82	PGS001225	EFO_0004348	hematocrit	hematocrit	EFO_0004348	Hematological measurement	Hematological	PGS001925	EFO_0004348	hematocrit	hematocrit	EFO_0004348	Hematological measurement	Hematological
0.7536	6.21E-85	8.13E-82	PGS000008	EFO_1000649	estrogen-receptor positive breast cancer	estrogen-receptor positive breast cancer	EFO_1000649	Cancer	Neoplasms	PGS000540	EFO_0000305	breast carcinoma	breast carcinoma	EFO_0000305	Cancer	Neoplasms
-0.7535	6.53E-85	8.55E-82	PGS000950	GO_0009314	response to radiation	response to radiation	GO_0009314	Biological process	Others	PGS001535	EFO_0010729	sun exposure measurement	sun exposure measurement	EFO_0010729	Other measurement	Activities
0.7535	6.57E-85	8.59E-82	PGS000499	EFO_0000305	breast carcinoma	breast carcinoma	EFO_0000305	Cancer	Neoplasms	PGS000536	EFO_0000305	breast carcinoma	breast carcinoma	EFO_0000305	Cancer	Neoplasms
0.7535	6.61E-85	8.64E-82	PGS002345	EFO_0004305	erythrocyte count	erythrocyte count	EFO_0004305	Hematological measurement	Hematological	PGS002662	EFO_0004305	erythrocyte count	erythrocyte count	EFO_0004305	Hematological measurement	Hematological
0.7535	6.64E-85	8.68E-82	PGS002802	EFO_0004339	body height	body height	EFO_0004339	Body measurement	Body measurement	PGS002968	EFO_0004339	body height	body height	EFO_0004339	Body measurement	Body measurement
0.7535	6.68E-85	8.72E-82	PGS000438	MONDO_0002898	skin cancer	skin cancer	MONDO_0002898	Cancer	Neoplasms	PGS000434	MONDO_0002898	skin cancer	skin cancer	MONDO_0002898	Cancer	Neoplasms
0.7535	6.89E-85	9.00E-82	PGS000688	EFO_0004611	low density lipoprotein cholesterol measurement	low density lipoprotein cholesterol measurement	EFO_0004611	Lipid or lipoprotein measurement	Metabolic	PGS002352	EFO_0004574	total cholesterol measurement	total cholesterol measurement	EFO_0004574	Lipid or lipoprotein measurement	Metabolic
0.7535	6.89E-85	9.00E-82	PGS000688	EFO_0004611	low density lipoprotein cholesterol measurement	low density lipoprotein cholesterol measurement	EFO_0004611	Cardiovascular measurement	Cardiovascular	PGS002352	EFO_0004574	total cholesterol measurement	total cholesterol measurement	EFO_0004574	Lipid or lipoprotein measurement	Metabolic
0.7534	6.92E-85	9.03E-82	PGS001479	EFO_0006930	brain volume measurement|neuroimaging measurement	brain volume measurement	EFO_0006930	Other measurement	Neurological	PGS001673	EFO_0006930	measurement	brain volume measurement	EFO_0006930	Other measurement	Neurological
0.7533	7.79E-85	1.02E-81	PGS000698	EFO_0004536	total blood protein measurement	total blood protein measurement	EFO_0004536	Hematological measurement	Hematological	PGS002001	EFO_0004536	total blood protein measurement	total blood protein measurement	EFO_0004536	Hematological measurement	Hematological
0.7533	8.07E-85	1.05E-81	PGS000002	EFO_1000649	estrogen-receptor positive breast cancer	estrogen-receptor positive breast cancer	EFO_1000649	Cancer	Neoplasms	PGS000773	EFO_0000305	breast carcinoma	breast carcinoma	EFO_0000305	Cancer	Neoplasms
0.7532	8.25E-85	1.08E-81	PGS000671	EFO_0004614	apolipoprotein A 1 measurement	apolipoprotein A 1 measurement	EFO_0004614	Cardiovascular measurement	Cardiovascular	PGS001888	EFO_0004614	apolipoprotein A 1 measurement	apolipoprotein A 1 measurement	EFO_0004614	Lipid or lipoprotein measurement	Metabolic
0.7532	8.25E-85	1.08E-81	PGS000671	EFO_0004614	apolipoprotein A 1 measurement	apolipoprotein A 1 measurement	EFO_0004614	Inflammatory measurement	Immunological	PGS001888	EFO_0004614	apolipoprotein A 1 measurement	apolipoprotein A 1 measurement	EFO_0004614	Lipid or lipoprotein measurement	Metabolic

0.7532	8.25E-85	1.08E-81	PGS000671	EFO_0004614	apolipoprotein A 1 measurement	apolipoprotein A 1 measurement	EFO_0004614	Cardiovascular measurement	Cardiovascular	PGS001888	EFO_0004614	apolipoprotein A 1 measurement	apolipoprotein A 1 measurement	EFO_0004614	Inflammatory measurement	Immunological
0.7532	8.25E-85	1.08E-81	PGS000671	EFO_0004614	apolipoprotein A 1 measurement	apolipoprotein A 1 measurement	EFO_0004614	Lipid or lipoprotein measurement	Metabolic	PGS001888	EFO_0004614	apolipoprotein A 1 measurement	apolipoprotein A 1 measurement	EFO_0004614	Inflammatory measurement	Immunological
0.7532	8.25E-85	1.08E-81	PGS000671	EFO_0004614	apolipoprotein A 1 measurement	apolipoprotein A 1 measurement	EFO_0004614	Inflammatory measurement	Immunological	PGS001888	EFO_0004614	apolipoprotein A 1 measurement	apolipoprotein A 1 measurement	EFO_0004614	Inflammatory measurement	Immunological
0.7532	8.25E-85	1.08E-81	PGS000671	EFO_0004614	apolipoprotein A 1 measurement	apolipoprotein A 1 measurement	EFO_0004614	Lipid or lipoprotein measurement	Metabolic	PGS001888	EFO_0004614	apolipoprotein A 1 measurement	apolipoprotein A 1 measurement	EFO_0004614	Cardiovascular measurement	Cardiovascular
0.7532	8.25E-85	1.08E-81	PGS000671	EFO_0004614	apolipoprotein A 1 measurement	apolipoprotein A 1 measurement	EFO_0004614	Lipid or lipoprotein measurement	Metabolic	PGS001888	EFO_0004614	apolipoprotein A 1 measurement	apolipoprotein A 1 measurement	EFO_0004614	Lipid or lipoprotein measurement	Metabolic
0.7532	8.25E-85	1.08E-81	PGS000671	EFO_0004614	apolipoprotein A 1 measurement	apolipoprotein A 1 measurement	EFO_0004614	Inflammatory measurement	Immunological	PGS001888	EFO_0004614	apolipoprotein A 1 measurement	apolipoprotein A 1 measurement	EFO_0004614	Cardiovascular measurement	Cardiovascular
0.7532	8.25E-85	1.08E-81	PGS000671	EFO_0004614	apolipoprotein A 1 measurement	apolipoprotein A 1 measurement	EFO_0004614	Cardiovascular measurement	Cardiovascular	PGS001888	EFO_0004614	apolipoprotein A 1 measurement	apolipoprotein A 1 measurement	EFO_0004614	Cardiovascular measurement	Cardiovascular
0.7532	8.61E-85	1.12E-81	PGS000310	EFO_0004611	low density lipoprotein cholesterol measurement	low density lipoprotein cholesterol measurement	EFO_0004611	Lipid or lipoprotein measurement	Metabolic	PGS002654	EFO_0004611	low density lipoprotein cholesterol measurement	measurement	EFO_0004611	Cardiovascular measurement	Cardiovascular
0.7532	8.61E-85	1.12E-81	PGS000310	EFO_0004611	low density lipoprotein cholesterol measurement	low density lipoprotein cholesterol measurement	EFO_0004611	Cardiovascular measurement	Cardiovascular	PGS002654	EFO_0004611	low density lipoprotein cholesterol measurement	measurement	EFO_0004611	Cardiovascular measurement	Cardiovascular
0.7532	8.61E-85	1.12E-81	PGS000310	EFO_0004611	low density lipoprotein cholesterol measurement	low density lipoprotein cholesterol measurement	EFO_0004611	Cardiovascular measurement	Cardiovascular	PGS002654	EFO_0004611	low density lipoprotein cholesterol measurement	measurement	EFO_0004611	Lipid or lipoprotein measurement	Metabolic
0.7532	8.61E-85	1.12E-81	PGS000310	EFO_0004611	low density lipoprotein cholesterol measurement	low density lipoprotein cholesterol measurement	EFO_0004611	Lipid or lipoprotein measurement	Metabolic	PGS002654	EFO_0004611	low density lipoprotein cholesterol measurement	measurement	EFO_0004611	Lipid or lipoprotein measurement	Metabolic
0.7532	8.75E-85	1.14E-81	PGS003398	EFO_0000305	breast carcinoma	breast carcinoma	EFO_0000305	Cancer	Neoplasms	PGS000488	EFO_0000305	breast carcinoma	breast carcinoma	EFO_0000305	Cancer	Neoplasms
0.7531	8.80E-85	1.15E-81	PGS000532	EFO_0000305	breast carcinoma	breast carcinoma	EFO_0000305	Cancer	Neoplasms	PGS000530	EFO_0000305	breast carcinoma	breast carcinoma	EFO_0000305	Cancer	Neoplasms
0.753	9.60E-85	1.25E-81	PGS002965	EFO_0004339	body height	body height	EFO_0004339	Body measurement	Body measurement	PGS002146	EFO_0004339	body height	body height	EFO_0004339	Body measurement	Body measurement
0.753	9.80E-85	1.28E-81	PGS002568	EFO_0006335	systolic blood pressure	systolic blood pressure	EFO_0006335	Other measurement	Cardiovascular	PGS002590	EFO_0006336	diastolic blood pressure	diastolic blood pressure	EFO_0006336	Other measurement	Cardiovascular
0.753	9.82E-85	1.28E-81	PGS003201	EFO_0004631	vitamin D measurement	vitamin D measurement	EFO_0004631	Lipid or lipoprotein measurement	Metabolic	PGS003196	EFO_0004631	vitamin D measurement	vitamin D measurement	EFO_0004631	Lipid or lipoprotein measurement	Metabolic
0.753	1.00E-84	1.30E-81	PGS003283	EFO_0004617	cystatin C measurement	cystatin C measurement	EFO_0004617	Other measurement	Metabolic	PGS003279	EFO_0004617	cystatin C measurement	cystatin C measurement	EFO_0004617	Other measurement	Metabolic
0.753	1.01E-84	1.31E-81	PGS002473	EFO_0004574	total cholesterol measurement	total cholesterol measurement	EFO_0004574	Lipid or lipoprotein measurement	Metabolic	PGS002571	EFO_0004574	total cholesterol measurement	total cholesterol measurement	EFO_0004574	Lipid or lipoprotein measurement	Metabolic
0.753	1.02E-84	1.32E-81	PGS002357	EFO_0004308	leukocyte count	leukocyte count	EFO_0004308	Hematological measurement	Hematological	PGS001969	EFO_0004833	neutrophil count	neutrophil count	EFO_0004833	Hematological measurement	Hematological
0.753	1.02E-84	1.32E-81	PGS001037	EFO_0009270	heel bone mineral density	heel bone mineral density	EFO_0009270	Other measurement	Skeletal	PGS000954	EFO_0004514	bone quantitative ultrasound measurement	measurement	EFO_0004514	Other measurement	Skeletal
0.7529	1.03E-84	1.34E-81	PGS000952	EFO_0004514	bone quantitative ultrasound measurement	bone quantitative ultrasound measurement	EFO_0004514	Other measurement	Skeletal	PGS000954	EFO_0004514	bone quantitative ultrasound measurement	measurement	EFO_0004514	Other measurement	Skeletal
0.7529	1.04E-84	1.35E-81	PGS001037	EFO_0009270	heel bone mineral density	heel bone mineral density	EFO_0009270	Other measurement	Skeletal	PGS001038	EFO_0009270	heel bone mineral density	heel bone mineral density	EFO_0009270	Other measurement	Skeletal
0.7529	1.06E-84	1.37E-81	PGS000952	EFO_0004514	bone quantitative ultrasound measurement	bone quantitative ultrasound measurement	EFO_0004514	Other measurement	Skeletal	PGS001038	EFO_0009270	heel bone mineral density	heel bone mineral density	EFO_0009270	Other measurement	Skeletal
0.7528	1.14E-84	1.48E-81	PGS003316	EFO_0004617	cystatin C measurement|glomerular filtration rate	cystatin C measurement	EFO_0004617	Other measurement	Metabolic	PGS003309	EFO_0004617	cystatin C measurement|glomerular filtration rate	cystatin C measurement	EFO_0004617	Other measurement	Metabolic
0.7528	1.14E-84	1.48E-81	PGS003315	EFO_0004617	cystatin C measurement|glomerular filtration rate	cystatin C measurement	EFO_0004617	Other measurement	Metabolic	PGS003309	EFO_0004617	cystatin C measurement|glomerular filtration rate	cystatin C measurement	EFO_0004617	Other measurement	Metabolic
0.7528	1.18E-84	1.53E-81	PGS001476	EFO_0006930	brain volume measurement|neuroimaging measurement	brain volume measurement	EFO_0006930	Other measurement	Neurological	PGS001680	EFO_0006930	measurement	brain volume measurement	EFO_0006930	Other measurement	Neurological
0.7528	1.19E-84	1.54E-81	PGS003145	EFO_0004530	triglyceride measurement	triglyceride measurement	EFO_0004530	Lipid or lipoprotein measurement	Metabolic	PGS003148	EFO_0004530	triglyceride measurement	triglyceride measurement	EFO_0004530	Lipid or lipoprotein measurement	Metabolic
0.7527	1.21E-84	1.57E-81	PGS000892	EFO_0004611	low density lipoprotein cholesterol measurement	low density lipoprotein cholesterol measurement	EFO_0004611	Lipid or lipoprotein measurement	Metabolic	PGS001933	EFO_0004611	low density lipoprotein cholesterol measurement	measurement	EFO_0004611	Cardiovascular measurement	Cardiovascular
0.7527	1.21E-84	1.57E-81	PGS000892	EFO_0004611	low density lipoprotein cholesterol measurement	low density lipoprotein cholesterol measurement	EFO_0004611	Cardiovascular measurement	Cardiovascular	PGS001933	EFO_0004611	low density lipoprotein cholesterol measurement	measurement	EFO_0004611	Cardiovascular measurement	Cardiovascular
0.7527	1.21E-84	1.57E-81	PGS000892	EFO_0004611	low density lipoprotein cholesterol measurement	low density lipoprotein cholesterol measurement	EFO_0004611	Cardiovascular measurement	Cardiovascular	PGS001933	EFO_0004611	low density lipoprotein cholesterol measurement	measurement	EFO_0004611	Lipid or lipoprotein measurement	Metabolic
0.7527	1.21E-84	1.57E-81	PGS000892	EFO_0004611	low density lipoprotein cholesterol measurement	low density lipoprotein cholesterol measurement	EFO_0004611	Lipid or lipoprotein measurement	Metabolic	PGS001933	EFO_0004611	low density lipoprotein cholesterol measurement	measurement	EFO_0004611	Lipid or lipoprotein measurement	Metabolic
0.7527	1.23E-84	1.60E-81	PGS002524	MONDO_0005148	type 2 diabetes mellitus	type 2 diabetes mellitus	MONDO_0005148	Digestive system disorder	Gastrointestinal	PGS002589	EFO_0000400	diabetes mellitus	diabetes mellitus	EFO_0000400	Metabolic disorder	Metabolic
0.7527	1.23E-84	1.60E-81	PGS002524	MONDO_0005148	type 2 diabetes mellitus	type 2 diabetes mellitus	MONDO_0005148	Digestive system disorder	Gastrointestinal	PGS002589	EFO_0000400	diabetes mellitus	diabetes mellitus	EFO_0000400	Digestive system disorder	Gastrointestinal
0.7527	1.23E-84	1.60E-81	PGS002524	MONDO_0005148	type 2 diabetes mellitus	type 2 diabetes mellitus	MONDO_0005148	Metabolic disorder	Metabolic	PGS002589	EFO_0000400	diabetes mellitus	diabetes mellitus	EFO_0000400	Metabolic disorder	Metabolic
0.7527	1.23E-84	1.60E-81	PGS002524	MONDO_0005148	type 2 diabetes mellitus	type 2 diabetes mellitus	MONDO_0005148	Metabolic disorder	Metabolic	PGS002589	EFO_0000400	diabetes mellitus	diabetes mellitus	EFO_0000400	Digestive system disorder	Gastrointestinal
0.7527	1.25E-84	1.62E-81	PGS003256	EFO_0010472	creatine measurement|glomerular filtration rate	creatine measurement	EFO_0010472	Other measurement	Metabolic	PGS003255	EFO_0010472	creatine measurement|glomerular filtration rate	creatine measurement	EFO_0010472	Other measurement	Metabolic
0.7526	1.35E-84	1.76E-81	PGS001623	EFO_0005420	measurement	grey matter volume measurement	EFO_0005420	Other measurement	Neurological	PGS001624	EFO_0005420	measurement	grey matter volume measurement	EFO_0005420	Other measurement	Neurological
0.7526	1.41E-84	1.82E-81	PGS001264	EFO_0003907	deep vein thrombosis	deep vein thrombosis	EFO_0003907	Other trait	Cardiovascular	PGS001277	EFO_0003827	pulmonary embolism|deep vein thrombosis	pulmonary embolism	EFO_0003827	Cardiovascular disease	Cardiovascular
0.7525	1.48E-84	1.91E-81	PGS003252	EFO_0010472	creatine measurement|glomerular filtration rate	creatine measurement	EFO_0010472	Other measurement	Metabolic	PGS003267	EFO_0010472	creatine measurement|glomerular filtration rate	creatine measurement	EFO_0010472	Other measurement	Metabolic
0.7524	1.54E-84	1.99E-81	PGS000185	EFO_0007984	platelet component distribution width	platelet component distribution width	EFO_0007984	Hematological measurement	Hematological	PGS000108	EFO_0007984	platelet component distribution width	platelet component distribution width	EFO_0007984	Hematological measurement	Hematological
0.7524	1.60E-84	2.07E-81	PGS002027	EFO_0003770	diabetic retinopathy	diabetic retinopathy	EFO_0003770	Metabolic disorder	Metabolic	PGS002025	MONDO_0005147	type 1 diabetes mellitus	type 1 diabetes mellitus	MONDO_0005147	Metabolic disorder	Metabolic
0.7524	1.60E-84	2.07E-81	PGS002027	EFO_0003770	diabetic retinopathy	diabetic retinopathy	EFO_0003770	Neurological disorder	Neurological	PGS002025	MONDO_0005147	type 1 diabetes mellitus	type 1 diabetes mellitus	MONDO_0005147	Digestive system disorder	Gastrointestinal
0.7524	1.60E-84	2.07E-81	PGS002027	EFO_0003770	diabetic retinopathy	diabetic retinopathy	EFO_0003770	Neurological disorder	Neurological	PGS002025	MONDO_0005147	type 1 diabetes mellitus	type 1 diabetes mellitus	MONDO_0005147	Immune system disorder	Immunological
0.7524	1.60E-84	2.07E-81	PGS002027	EFO_0003770	diabetic retinopathy	diabetic retinopathy	EFO_0003770	Cardiovascular disease	Cardiovascular	PGS002025	MONDO_0005147	type 1 diabetes mellitus	type 1 diabetes mellitus	MONDO_0005147	Metabolic disorder	Metabolic
0.7524	1.60E-84	2.07E-81	PGS002027	EFO_0003770	diabetic retinopathy	diabetic retinopathy	EFO_0003770	Metabolic disorder	Metabolic	PGS002025	MONDO_0005147	type 1 diabetes mellitus	type 1 diabetes mellitus	MONDO_0005147	Digestive system disorder	Gastrointestinal
0.7524	1.60E-84	2.07E-81	PGS002027	EFO_0003770	diabetic retinopathy	diabetic retinopathy	EFO_0003770	Cardiovascular disease	Cardiovascular	PGS002025	MONDO_0005147	type 1 diabetes mellitus	type 1 diabetes mellitus	MONDO_0005147	Immune system disorder	Immunological
0.7524	1.60E-84	2.07E-81	PGS002027	EFO_0003770	diabetic retinopathy	diabetic retinopathy	EFO_0003770	Cardiovascular disease	Cardiovascular	PGS002025	MONDO_0005147	type 1 diabetes mellitus	type 1 diabetes mellitus	MONDO_0005147	Digestive system disorder	Gastrointestinal
0.7524	1.60E-84	2.07E-81	PGS002027	EFO_0003770	diabetic retinopathy	diabetic retinopathy	EFO_0003770	Neurological disorder	Neurological	PGS002025	MONDO_0005147	type 1 diabetes mellitus	type 1 diabetes mellitus	MONDO_0005147	Metabolic disorder	Metabolic
0.7524	1.60E-84	2.07E-81	PGS002027	EFO_0003770	diabetic retinopathy	diabetic retinopathy	EFO_0003770	Metabolic disorder	Metabolic	PGS002025	MONDO_0005147	type 1 diabetes mellitus	type 1 diabetes mellitus	MONDO_0005147	Immune system disorder	Immunological
0.7524	1.64E-84	2.13E-81	PGS000476	EFO_0000305	breast carcinoma	breast carcinoma	EFO_0000305	Cancer	Neoplasms	PGS001337	EFO_0005606	family history of breast cancer	family history of breast cancer	EFO_0005606	Other trait	Neoplasms
0.7523	1.73E-84	2.23E-81	PGS003150	EFO_0004530	triglyceride measurement	triglyceride measurement	EFO_0004530	Lipid or lipoprotein measurement	Metabolic	PGS003149	EFO_0004530	triglyceride measurement	triglyceride measurement	EFO_0004530	Lipid or lipoprotein measurement	Metabolic
0.7523	1.74E-84	2.25E-81	PGS002802	EFO_0004339	body height	body height	EFO_0004339	Body measurement	Body measurement	PGS002973	EFO_0004339	body height	body height	EFO_0004339	Body measurement	Body measurement
0.7523	1.75E-84	2.27E-81	PGS002721	EFO_0004631	vitamin D measurement	vitamin D measurement	EFO_0004631	Lipid or lipoprotein measurement	Metabolic	PGS003194	EFO_0004631	vitamin D measurement	vitamin D measurement	EFO_0004631	Lipid or lipoprotein measurement	Metabolic
0.7522	1.79E-84	2.32E-81	PGS000500	EFO_0000305	breast carcinoma	breast carcinoma	EFO_0000305	Cancer	Neoplasms	PGS000007	EFO_0000305	breast carcinoma	breast carcinoma	EFO_0000305	Cancer	Neoplasms
0.7522	1.88E-84	2.43E-81	PGS000007	EFO_0000305	breast carcinoma	breast carcinoma	EFO_0000305	Cancer	Neoplasms	PGS000503	EFO_0000305	breast carcinoma	breast carcinoma	EFO_0000305	Cancer	Neoplasms
0.7521	2.03E-84	2.62E-81	PGS003316	EFO_0004617	cystatin C measurement|glomerular filtration rate	cystatin C measurement	EFO_0004617	Other measurement	Metabolic	PGS003298	EFO_0004617	cystatin C measurement|glomerular filtration rate	cystatin C measurement	EFO_0004617	Other measurement	Metabolic
0.7521	2.03E-84	2.62E-81	PGS003315	EFO_0004617	cystatin C measurement|glomerular filtration rate	cystatin C measurement	EFO_0004617	Other measurement	Metabolic	PGS003298	EFO_0004617	cystatin C measurement|glomerular filtration rate	cystatin C measurement	EFO_0004617	Other measurement	Metabolic
0.7521	2.08E-84	2.69E-81	PGS003031	EFO_0004611	low density lipoprotein cholesterol measurement	low density lipoprotein cholesterol measurement	EFO_0004611	Lipid or lipoprotein measurement	Metabolic	PGS002605	EFO_0004611	low density lipoprotein cholesterol measurement	measurement	EFO_0004611	Lipid or lipoprotein measurement	Metabolic
0.7521	2.08E-84	2.69E-81	PGS003031	EFO_0004611	low density lipoprotein cholesterol measurement	low density lipoprotein cholesterol measurement	EFO_0004611	Cardiovascular measurement	Cardiovascular	PGS002605	EFO_0004611	low density lipoprotein cholesterol measurement	measurement	EFO_0004611	Lipid or lipoprotein measurement	Metabolic
0.7521	2.08E-84	2.69E-81	PGS003031	EFO_0004611	low density lipoprotein cholesterol measurement	low density lipoprotein cholesterol measurement	EFO_0004611	Cardiovascular measurement	Cardiovascular	PGS002605	EFO_0004611	low density lipoprotein cholesterol measurement	measurement	EFO_0004611	Cardiovascular measurement	Cardiovascular
0.7521	2.08E-84	2.69E-81	PGS003031	EFO_0004611	low density lipoprotein cholesterol measurement	low density lipoprotein cholesterol measurement	EFO_0004611	Lipid or lipoprotein measurement	Metabolic	PGS002605	EFO_0004611	low density lipoprotein cholesterol measurement	measurement	EFO_0004611	Cardiovascular measurement	Cardiovascular
0.752	2.22E-84	2.87E-81	PGS000046	EFO_1000649	estrogen-receptor positive breast cancer	estrogen-receptor positive breast cancer	EFO_1000649	Cancer	Neoplasms	PGS003398	EFO_0000305	breast carcinoma	breast carcinoma	EFO_0000305	Cancer	Neoplasms
0.7518	2.61E-84	3.37E-81	PGS001280	EFO_0003827	pulmonary embolism	pulmonary embolism	EFO_0003827	Cardiovascular disease	Cardiovascular	PGS001844	EFO_1001395	Phlebitis|Thrombophlebitis	Phlebitis	EFO_1001395	Cardiovascular disease	Cardiovascular
0.7518	2.61E-84	3.37E-81	PGS000892	EFO_0004611	low density lipoprotein cholesterol measurement	low density lipoprotein cholesterol measurement	EFO_0004611	Cardiovascular measurement	Cardiovascular	PGS000889	EFO_0004611	low density lipoprotein cholesterol measurement	measurement	EFO_0004611	Cardiovascular measurement	Cardiovascular
0.7518	2.61E-84	3.37E-81	PGS000892	EFO_0004611	low density lipoprotein cholesterol measurement	low density lipoprotein cholesterol measurement	EFO_0004611	Lipid or lipoprotein measurement	Metabolic	PGS000889	EFO_0004611	low density lipoprotein cholesterol measurement	measurement	EFO_0004611	Lipid or lipoprotein measurement	Metabolic
0.7518	2.61E-84	3.37E-81	PGS000892	EFO_0004611	low density lipoprotein cholesterol measurement	low density lipoprotein cholesterol measurement	EFO_0004611	Cardiovascular measurement	Cardiovascular	PGS000889	EFO_0004611	low density lipoprotein cholesterol measurement	measurement	EFO_0004611	Lipid or lipoprotein measurement	Metabolic
0.7518	2.61E-84	3.37E-81	PGS000892	EFO_0004611	low density lipoprotein cholesterol measurement	low density lipoprotein cholesterol measurement	EFO_0004611	Lipid or lipoprotein measurement	Metabolic	PGS000889	EFO_0004611	low density lipoprotein cholesterol measurement	measurement	EFO_0004611	Cardiovascular measurement	Cardiovascular
0.7517	2.65E-84	3.42E-81	PGS002008	EFO_0004503	hematological measurement	hematological measurement	EFO_0004503	Hematological measurement	Hematological	PGS002207	EFO_0004526	mean corpuscular volume	mean corpuscular volume	EFO_0004526	Hematological measurement	Hematological
0.7517	2.77E-84	3.57E-81	PGS003036	EFO_0004611	low density lipoprotein cholesterol measurement	low density lipoprotein cholesterol measurement	EFO_0004611	Cardiovascular measurement	Cardiovascular	PGS003037	EFO_0004611	low density lipoprotein cholesterol measurement	measurement	EFO_0004611	Lipid or lipoprotein measurement	Metabolic
0.7517	2.77E-84	3.57E-81	PGS003036	EFO_0004611	low density lipoprotein cholesterol measurement	low density lipoprotein cholesterol measurement	EFO_0004611	Cardiovascular measurement	Cardiovascular	PGS003037	EFO_0004611	low density lipoprotein cholesterol measurement	measurement	EFO_0004611	Cardiovascular measurement	Cardiovascular
0.7517	2.77E-84	3.57E-81	PGS003036	EFO_0004611	low density lipoprotein cholesterol measurement	low density lipoprotein cholesterol measurement	EFO_0004611	Lipid or lipoprotein measurement	Metabolic	PGS003037	EFO_0004611	low density lipoprotein cholesterol measurement	measurement	EFO_0004611	Cardiovascular measurement	Cardiovascular
0.7517	2.77E-84	3.57E-81	PGS003036	EFO_0004611	low density lipoprotein cholesterol measurement	low density lipoprotein cholesterol measurement	EFO_0004611	Lipid or lipoprotein measurement	Metabolic	PGS003037	EFO_0004611	low density lipoprotein cholesterol measurement	measurement	EFO_0004611	Lipid or lipoprotein measurement	Metabolic
0.7516	3.02E-84	3.89E-81	PGS002648	EFO_0004541	HbA1c measurement	HbA1c measurement	EFO_0004541	Hematological measurement	Hematological	PGS001953	EFO_0004541	HbA1c measurement	HbA1c measurement	EFO_0004541	Hematological measurement	Hematological
0.7516	3.03E-84	3.91E-81	PGS001895	EFO_0004574	total cholesterol measurement	total cholesterol measurement	EFO_0004574	Lipid or lipoprotein measurement	Metabolic	PGS000677	EFO_0004574	total cholesterol measurement	total cholesterol measurement	EFO_0004574	Lipid or lipoprotein measurement	Metabolic
0.7515	3.15E-84	4.07E-81	PGS001493	EFO_0006930	brain volume measurement|neuroimaging measurement	brain volume measurement	EFO_0006930	Other measurement	Neurological	PGS001494	EFO_0006930	measurement	brain volume measurement	EFO_0006930	Other measurement	Neurological
0.7514	3.36E-84	4.33E-81	PGS003029	EFO_0004611	low density lipoprotein cholesterol measurement	low density lipoprotein cholesterol measurement	EFO_0004611	Cardiovascular measurement	Cardiovascular	PGS000888	EFO_0004611	low density lipoprotein cholesterol measurement	measurement	EFO_0004611	Cardiovascular measurement	Cardiovascular
0.7514	3.36E-84	4.33E-81	PGS003029	EFO_0004611	low density lipoprotein cholesterol measurement	low density lipoprotein cholesterol measurement	EFO_0004611	Lipid or lipoprotein measurement	Metabolic	PGS000888	EFO_0004611	low density lipoprotein cholesterol measurement	measurement	EFO_0004611	Lipid or lipoprotein measurement	Metabolic
0.7514	3.36E-84	4.33E-81	PGS003029	EFO_0004611	low density lipoprotein cholesterol measurement	low density lipoprotein cholesterol measurement	EFO_0004611	Lipid or lipoprotein measurement	Metabolic	PGS000888	EFO_0004611	low density lipoprotein cholesterol measurement	measurement	EFO_0004611	Cardiovascular measurement	Cardiovascular
0.7514	3.36E-84	4.33E-81	PGS003029	EFO_0004611	low density lipoprotein cholesterol measurement	low density lipoprotein cholesterol measurement	EFO_0004611	Cardiovascular measurement	Cardiovascular	PGS000888	EFO_0004611	low density lipoprotein cholesterol measurement	measurement	EFO_0004611	Lipid or lipoprotein measurement	Metabolic
0.7514	3.37E-84	4.35E-81	PGS000110	EFO_0004305	erythrocyte count	erythrocyte count	EFO_0004305	Hematological measurement	Hematological	PGS002123	EFO_0004305	erythrocyte count	erythrocyte count	EFO_0004305	Hematological measurement	Hematological
0.7514	3.54E-84	4.56E-81	PGS000537	EFO_0000305	breast carcinoma	breast carcinoma	EFO_0000305	Cancer	Neoplasms	PGS000506	EFO_0000305	breast carcinoma	breast carcinoma	EFO_0000305	Cancer	Neoplasms
0.7514	3.54E-84	4.56E-81	PGS000537	EFO_0000305	breast carcinoma	breast carcinoma	EFO_0000305	Cancer	Neoplasms	PGS000505	EFO_0000305	breast carcinoma	breast carcinoma	EFO_0000305	Cancer	Neoplasms
0.7513	3.76E-84	4.84E-81	PGS002524	MONDO_0005148	type 2 diabetes mellitus	type 2 diabetes mellitus	MONDO_0005148	Digestive system disorder	Gastrointestinal	PGS002501	EFO_0004541	HbA1c measurement	HbA1c measurement	EFO_0004541	Hematological measurement	Hematological
0.7513	3.76E-84	4.84E-81	PGS002524	MONDO_0005148	type 2 diabetes mellitus	type 2 diabetes mellitus	MONDO_0005148	Metabolic disorder	Metabolic	PGS002501	EFO_0004541	HbA1c measurement	HbA1c measurement	EFO_0004541	Hematological measurement	Hematological
0.7513	3.76E-84	4.84E-81	PGS001943	EFO_0004340	body mass index	body mass index	EFO_0004340	Body measurement	Body measurement	PGS002176	EFO_0005093	hip circumference	hip circumference	EFO_0005093	Body measurement	Body measurement
0.7513	3.82E-84	4.91E-81	PGS001943	EFO_0004340	body mass index	body mass index	EFO_0004340	Body measurement	Body measurement	PGS002133	EFO_0007800	body fat percentage	body fat percentage	EFO_0007800	Body measurement	Body measurement
0.7513	3.92E-84	5.04E-81	PGS003186	EFO_0004631	vitamin D measurement	vitamin D measurement	EFO_0004631	Lipid or lipoprotein measurement	Metabolic	PGS003196	EFO_0004631	vitamin D measurement	vitamin D measurement	EFO_0004631	Lipid or lipoprotein measurement	Metabolic
0.7511	4.35E-84	5.59E-81	PGS002513	EFO_0004309	platelet count	platelet count	EFO_0004309	Hematological measurement	Hematological	PGS002415	EFO_0004309	platelet count	platelet count	EFO_0004309	Hematological measurement	Hematological
0.7511	4.45E-84	5.72E-81	PGS002543	EFO_0001379	endocrine system disease|diabetes mellitus	endocrine system disease	EFO_0001379	Other disease	Metabolic	PGS002599	EFO_0004541	HbA1c measurement	HbA1c measurement	EFO_0004541	Hematological measurement	Hematological
0.751	4.62E-84	5.94E-81	PGS001147	EFO_0005409	fat body mass	fat body mass	EFO_0005409	Other measurement	Body measurement	PGS001105	EFO_0007800	body fat percentage	body fat percentage	EFO_0007800	Body measurement	Body measurement
0.751	4.92E-84	6.32E-81	PGS002404	EFO_0004339	body height	body height	EFO_0004339	Body measurement	Body measurement	PGS002968	EFO_0004339	body height	body height	EFO_0004339	Body measurement	Body measurement
0.7509	5.03E-84	6.46E-81	PGS001402	EFO_0009270	heel bone mineral density	heel bone mineral density	EFO_0009270	Other measurement	Skeletal	PGS000954	EFO_0004514	bone quantitative ultrasound measurement	measurement	EFO_0004514	Other measurement	Skeletal
0.7509	5.14E-84	6.61E-81	PGS001402	EFO_0009270	heel bone mineral density	heel bone mineral density	EFO_0009270	Other measurement	Skeletal	PGS001038	EFO_0009270	heel bone mineral density	heel bone mineral density	EFO_0009270	Other measurement	Skeletal
0.7508	5.42E-84	6.96E-81	PGS001684	EFO_0006930	brain volume measurement|neuroimaging measurement	brain volume measurement	EFO_0006930	Other measurement	Neurological	PGS001683	EFO_0006930	measurement	brain volume measurement	EFO_0006930	Other measurement	Neurological
0.7508	5.62E-84	7.21E-81	PGS001228	EFO_0004340	body mass index	body mass index	EFO_0004340	Body measurement	Body measurement	PGS001101	EFO_0007800	body fat percentage	body fat percentage	EFO_0007800	Body measurement	Body measurement
-0.7508	5.80E-84	7.44E-81	PGS001097	EFO_0007822	hair colour measurement	hair colour measurement	EFO_0007822	Other measurement	Dermatological	PGS001095	EFO_0007822	hair colour measurement	hair colour measurement	EFO_0007822	Other measurement	Dermatological
0.7507	5.99E-84	7.69E-81	PGS003069	EFO_0006335	systolic blood pressure	systolic blood pressure	EFO_0006335	Other measurement	Cardiovascular	PGS002009	EFO_0006335	systolic blood pressure	systolic blood pressure	EFO_0006335	Other measurement	Cardiovascular
0.7507	6.00E-84	7.70E-81	PGS002404	EFO_0004339	body height	body height	EFO_0004339	Body measurement	Body measurement	PGS002973	EFO_0004339	body height	body height	EFO_0004339	Body measurement	Body measurement
0.7506	6.39E-84	8.19E-81	PGS001673	EFO_0006930	brain volume measurement|neuroimaging measurement	brain volume measurement	EFO_0006930	Other measurement	Neurological	PGS001450	EFO_0006930	measurement	brain volume measurement	EFO_0006930	Other measurement	Neurological
0.7506	6.51E-84	8.35E-81	PGS001780	EFO_0001645	coronary artery disease	coronary artery disease	EFO_0001645	Cardiovascular disease	Cardiovascular	PGS000013	EFO_0001645	coronary artery disease	coronary artery disease	EFO_0001645	Cardiovascular disease	Cardiovascular
0.7506	6.65E-84	8.52E-81	PGS002501	EFO_0004541	HbA1c measurement	HbA1c measurement	EFO_0004541	Hematological measurement	Hematological	PGS002589	EFO_0000400	diabetes mellitus	diabetes mellitus	EFO_0000400	Metabolic disorder	Metabolic
0.7506	6.65E-84	8.52E-81	PGS002501	EFO_0004541	HbA1c measurement	HbA1c measurement	EFO_0004541	Hematological measurement	Hematological	PGS002589	EFO_0000400	diabetes mellitus	diabetes mellitus	EFO_0000400	Digestive system disorder	Gastrointestinal
0.7505	6.90E-84	8.84E-81	PGS002337	EFO_0004611	low density lipoprotein cholesterol measurement	low density lipoprotein cholesterol measurement	EFO_0004611	Lipid or lipoprotein measurement	Metabolic	PGS003138	EFO_0004574	total cholesterol measurement	total cholesterol measurement	EFO_0004574	Lipid or lipoprotein measurement	Metabolic

0.7505	6.90E-84	8.84E-81	PGS002337	EFO_0004611	low density lipoprotein cholesterol measurement	low density lipoprotein cholesterol measurement	EFO_0004611	Cardiovascular measurement	Cardiovascular	PGS003138	EFO_0004574	total cholesterol measurement	total cholesterol measurement	EFO_0004574	Lipid or lipoprotein measurement	Metabolic
0.7505	6.97E-84	8.92E-81	PGS001457	EFO_0006930	brain volume measurement|neuroimaging measurement	brain volume measurement	EFO_0006930	Other measurement	Neurological	PGS001665	EFO_0006930	measurement	brain volume measurement	EFO_0006930	Other measurement	Neurological
0.7505	6.98E-84	8.94E-81	PGS002781	EFO_0004612	high density lipoprotein cholesterol measurement	high density lipoprotein cholesterol measurement	EFO_0004612	Lipid or lipoprotein measurement	Metabolic	PGS002329	EFO_0004612	high density lipoprotein cholesterol measurement	measurement	EFO_0004612	Lipid or lipoprotein measurement	Metabolic
0.7505	7.19E-84	9.20E-81	PGS003295	EFO_0004617	cystatin C measurement|glomerular filtration rate	cystatin C measurement	EFO_0004617	Other measurement	Metabolic	PGS003312	EFO_0004617	cystatin C measurement|glomerular filtration rate	cystatin C measurement	EFO_0004617	Other measurement	Metabolic
0.7505	7.32E-84	9.36E-81	PGS000500	EFO_0000305	breast carcinoma	breast carcinoma	EFO_0000305	Cancer	Neoplasms	PGS000536	EFO_0000305	breast carcinoma	breast carcinoma	EFO_0000305	Cancer	Neoplasms
0.7504	7.35E-84	9.41E-81	PGS002672	EFO_0004631	vitamin D measurement	vitamin D measurement	EFO_0004631	Lipid or lipoprotein measurement	Metabolic	PGS003194	EFO_0004631	vitamin D measurement	vitamin D measurement	EFO_0004631	Lipid or lipoprotein measurement	Metabolic
0.7504	7.40E-84	9.47E-81	PGS000015	EFO_0000305	breast carcinoma	breast carcinoma	EFO_0000305	Cancer	Neoplasms	PGS000510	EFO_0000305	breast carcinoma	breast carcinoma	EFO_0000305	Cancer	Neoplasms
0.7504	7.46E-84	9.54E-81	PGS001173	EFO_0004833	neutrophil count	neutrophil count	EFO_0004833	Hematological measurement	Hematological	PGS002187	EFO_0004833	neutrophil count	neutrophil count	EFO_0004833	Hematological measurement	Hematological
0.7504	7.52E-84	9.62E-81	PGS000442	MONDO_0002898	skin cancer	skin cancer	MONDO_0002898	Cancer	Neoplasms	PGS000434	MONDO_0002898	skin cancer	skin cancer	MONDO_0002898	Cancer	Neoplasms
0.7504	7.71E-84	9.85E-81	PGS000758	EFO_0004339	body height	body height	EFO_0004339	Body measurement	Body measurement	PGS002969	EFO_0004339	body height	body height	EFO_0004339	Body measurement	Body measurement
0.7502	8.72E-84	1.11E-80	PGS001476	EFO_0006930	brain volume measurement|neuroimaging measurement	brain volume measurement	EFO_0006930	Other measurement	Neurological	PGS001481	EFO_0006930	measurement	brain volume measurement	EFO_0006930	Other measurement	Neurological
-0.7502	9.08E-84	1.16E-80	PGS003243	EFO_0010472	creatine measurement	creatine measurement	EFO_0010472	Other measurement	Metabolic	PGS003223	EFO_0005208	glomerular filtration rate	glomerular filtration rate	EFO_0005208	Other measurement	Genitourinary
0.7502	9.19E-84	1.17E-80	PGS002351	EFO_1000627	thyroid disease	thyroid disease	EFO_1000627	Other disease	Endocrine	PGS001181	EFO_0004705	hypothyroidism	hypothyroidism	EFO_0004705	Other disease	Endocrine
0.7501	9.31E-84	1.19E-80	PGS003239	EFO_0010472	creatine measurement	creatine measurement	EFO_0010472	Other measurement	Metabolic	PGS003240	EFO_0010472	creatine measurement	creatine measurement	EFO_0010472	Other measurement	Metabolic
0.75	1.05E-83	1.34E-80	PGS001320	EFO_0000537	hypertension	hypertension	EFO_0000537	Cardiovascular disease	Cardiovascular	PGS000957	MONDO_0001134	essential hypertension	essential hypertension	MONDO_0001134	Cardiovascular disease	Cardiovascular
0.75	1.06E-83	1.36E-80	PGS000888	EFO_0004611	low density lipoprotein cholesterol measurement	low density lipoprotein cholesterol measurement	EFO_0004611	Lipid or lipoprotein measurement	Metabolic	PGS000824	EFO_0004611	low density lipoprotein cholesterol measurement	measurement	EFO_0004611	Lipid or lipoprotein measurement	Metabolic
0.75	1.06E-83	1.36E-80	PGS000888	EFO_0004611	low density lipoprotein cholesterol measurement	low density lipoprotein cholesterol measurement	EFO_0004611	Lipid or lipoprotein measurement	Metabolic	PGS000824	EFO_0004611	low density lipoprotein cholesterol measurement	measurement	EFO_0004611	Cardiovascular measurement	Cardiovascular
0.75	1.06E-83	1.36E-80	PGS000888	EFO_0004611	low density lipoprotein cholesterol measurement	low density lipoprotein cholesterol measurement	EFO_0004611	Cardiovascular measurement	Cardiovascular	PGS000824	EFO_0004611	low density lipoprotein cholesterol measurement	measurement	EFO_0004611	Lipid or lipoprotein measurement	Metabolic
0.75	1.06E-83	1.36E-80	PGS000888	EFO_0004611	low density lipoprotein cholesterol measurement	low density lipoprotein cholesterol measurement	EFO_0004611	Cardiovascular measurement	Cardiovascular	PGS000824	EFO_0004611	low density lipoprotein cholesterol measurement	measurement	EFO_0004611	Cardiovascular measurement	Cardiovascular
0.7499	1.11E-83	1.41E-80	PGS003407	EFO_0003870	brain aneurysm|male	brain aneurysm	EFO_0003870	Neurological disorder	Neurological	PGS003408	EFO_0003870	brain aneurysm|female	brain aneurysm	EFO_0003870	Neurological disorder	Neurological
0.7499	1.11E-83	1.41E-80	PGS003407	EFO_0003870	brain aneurysm|male	brain aneurysm	EFO_0003870	Cardiovascular disease	Cardiovascular	PGS003408	EFO_0003870	brain aneurysm|female	brain aneurysm	EFO_0003870	Neurological disorder	Neurological
0.7499	1.11E-83	1.41E-80	PGS003407	EFO_0003870	brain aneurysm|male	brain aneurysm	EFO_0003870	Neurological disorder	Neurological	PGS003408	EFO_0003870	brain aneurysm|female	brain aneurysm	EFO_0003870	Cardiovascular disease	Cardiovascular
0.7499	1.11E-83	1.41E-80	PGS003407	EFO_0003870	brain aneurysm|male	brain aneurysm	EFO_0003870	Cardiovascular disease	Cardiovascular	PGS003408	EFO_0003870	brain aneurysm|female	brain aneurysm	EFO_0003870	Cardiovascular disease	Cardiovascular
0.7499	1.11E-83	1.41E-80	PGS001726	EFO_0006930	brain volume measurement|neuroimaging measurement	brain volume measurement	EFO_0006930	Other measurement	Neurological	PGS001755	EFO_0006930	measurement	brain volume measurement	EFO_0006930	Other measurement	Neurological
-0.7499	1.12E-83	1.42E-80	PGS002204	EFO_0007825	balding measurement	balding measurement	EFO_0007825	Other measurement	Activities	PGS002314	EFO_0007825	balding measurement	balding measurement	EFO_0007825	Other measurement	Activities
0.7499	1.12E-83	1.43E-80	PGS002501	EFO_0004541	HbA1c measurement	HbA1c measurement	EFO_0004541	Hematological measurement	Hematological	PGS002596	EFO_0004468	glucose measurement	glucose measurement	EFO_0004468	Other measurement	Metabolic
0.7499	1.16E-83	1.48E-80	PGS002970	EFO_0004339	body height	body height	EFO_0004339	Body measurement	Body measurement	PGS001929	EFO_0004339	body height	body height	EFO_0004339	Body measurement	Body measurement
0.7499	1.17E-83	1.49E-80	PGS000106	EFO_0007990	neutrophil percentage of leukocytes	neutrophil percentage of leukocytes	EFO_0007990	Hematological measurement	Hematological	PGS002214	EFO_0007990	neutrophil percentage of leukocytes	neutrophil percentage of leukocytes	EFO_0007990	Hematological measurement	Hematological
0.7498	1.18E-83	1.50E-80	PGS002174	EFO_0007772	calcaneal bone quantitative ultrasound measurement	calcaneal bone quantitative ultrasound measurement	EFO_0007772	Other measurement	Skeletal	PGS001529	EFO_0007772	measurement	measurement	EFO_0007772	Other measurement	Skeletal
0.7498	1.20E-83	1.53E-80	PGS003243	EFO_0010472	creatine measurement	creatine measurement	EFO_0010472	Other measurement	Metabolic	PGS000678	EFO_0004518	creatinine measurement	creatinine measurement	EFO_0004518	Other measurement	Metabolic
-0.7498	1.23E-83	1.57E-80	PGS000814	EFO_0004611	low density lipoprotein cholesterol measurement	low density lipoprotein cholesterol measurement	EFO_0004611	Lipid or lipoprotein measurement	Metabolic	PGS000061	EFO_0004611	low density lipoprotein cholesterol measurement	measurement	EFO_0004611	Lipid or lipoprotein measurement	Metabolic
0.7498	1.23E-83	1.57E-80	PGS000814	EFO_0004611	low density lipoprotein cholesterol measurement	low density lipoprotein cholesterol measurement	EFO_0004611	Lipid or lipoprotein measurement	Metabolic	PGS000061	EFO_0004611	low density lipoprotein cholesterol measurement	measurement	EFO_0004611	Cardiovascular measurement	Cardiovascular
-0.7498	1.23E-83	1.57E-80	PGS000814	EFO_0004611	low density lipoprotein cholesterol measurement	low density lipoprotein cholesterol measurement	EFO_0004611	Cardiovascular measurement	Cardiovascular	PGS000061	EFO_0004611	low density lipoprotein cholesterol measurement	measurement	EFO_0004611	Lipid or lipoprotein measurement	Metabolic
-0.7498	1.23E-83	1.57E-80	PGS000814	EFO_0004611	low density lipoprotein cholesterol measurement	low density lipoprotein cholesterol measurement	EFO_0004611	Cardiovascular measurement	Cardiovascular	PGS000061	EFO_0004611	low density lipoprotein cholesterol measurement	measurement	EFO_0004611	Cardiovascular measurement	Cardiovascular
-0.7498	1.23E-83	1.57E-80	PGS002534	EFO_0003923	bone density	bone density	EFO_0003923	Other measurement	Skeletal	PGS002768	EFO_0003882	osteoporosis	osteoporosis	EFO_0003882	Other disease	Skeletal
0.7498	1.23E-83	1.57E-80	PGS000774	EFO_1000649	estrogen-receptor positive breast cancer	estrogen-receptor positive breast cancer	EFO_1000649	Cancer	Neoplasms	PGS000046	EFO_1000649	estrogen-receptor positive breast cancer	cancer	EFO_1000649	Cancer	Neoplasms
0.7498	1.24E-83	1.57E-80	PGS003297	EFO_0004617	cystatin C measurement|glomerular filtration rate	cystatin C measurement	EFO_0004617	Other measurement	Metabolic	PGS003295	EFO_0004617	cystatin C measurement|glomerular filtration rate	cystatin C measurement	EFO_0004617	Other measurement	Metabolic
-0.7498	1.24E-83	1.58E-80	PGS001426	EFO_0006930	brain volume measurement|neuroimaging measurement	brain volume measurement	EFO_0006930	Other measurement	Neurological	PGS001499	EFO_0006930	measurement	brain volume measurement	EFO_0006930	Other measurement	Neurological
0.7497	1.29E-83	1.64E-80	PGS003033	EFO_0004611	low density lipoprotein cholesterol measurement	low density lipoprotein cholesterol measurement	EFO_0004611	Lipid or lipoprotein measurement	Metabolic	PGS002352	EFO_0004574	total cholesterol measurement	total cholesterol measurement	EFO_0004574	Lipid or lipoprotein measurement	Metabolic
0.7497	1.29E-83	1.64E-80	PGS003033	EFO_0004611	low density lipoprotein cholesterol measurement	low density lipoprotein cholesterol measurement	EFO_0004611	Cardiovascular measurement	Cardiovascular	PGS002352	EFO_0004574	total cholesterol measurement	total cholesterol measurement	EFO_0004574	Lipid or lipoprotein measurement	Metabolic
0.7497	1.35E-83	1.71E-80	PGS001958	EFO_0005093	hip circumference	hip circumference	EFO_0005093	Body measurement	Body measurement	PGS001162	EFO_0005093	hip circumference	hip circumference	EFO_0005093	Body measurement	Body measurement
0.7497	1.36E-83	1.72E-80	PGS003035	EFO_0004611	low density lipoprotein cholesterol measurement	low density lipoprotein cholesterol measurement	EFO_0004611	Cardiovascular measurement	Cardiovascular	PGS002605	EFO_0004611	low density lipoprotein cholesterol measurement	measurement	EFO_0004611	Cardiovascular measurement	Cardiovascular
0.7497	1.36E-83	1.72E-80	PGS003035	EFO_0004611	low density lipoprotein cholesterol measurement	low density lipoprotein cholesterol measurement	EFO_0004611	Lipid or lipoprotein measurement	Metabolic	PGS002605	EFO_0004611	low density lipoprotein cholesterol measurement	measurement	EFO_0004611	Lipid or lipoprotein measurement	Metabolic
0.7497	1.36E-83	1.72E-80	PGS003035	EFO_0004611	low density lipoprotein cholesterol measurement	low density lipoprotein cholesterol measurement	EFO_0004611	Cardiovascular measurement	Cardiovascular	PGS002605	EFO_0004611	low density lipoprotein cholesterol measurement	measurement	EFO_0004611	Lipid or lipoprotein measurement	Metabolic
0.7497	1.36E-83	1.72E-80	PGS003035	EFO_0004611	low density lipoprotein cholesterol measurement	low density lipoprotein cholesterol measurement	EFO_0004611	Lipid or lipoprotein measurement	Metabolic	PGS002605	EFO_0004611	low density lipoprotein cholesterol measurement	measurement	EFO_0004611	Cardiovascular measurement	Cardiovascular
0.7497	1.37E-83	1.74E-80	PGS001819	EFO_0003770	diabetic retinopathy	diabetic retinopathy	EFO_0003770	Cardiovascular disease	Cardiovascular	PGS001297	MONDO_0005147	type 1 diabetes mellitus	type 1 diabetes mellitus	MONDO_0005147	Immune system disorder	Immunological
0.7497	1.37E-83	1.74E-80	PGS001819	EFO_0003770	diabetic retinopathy	diabetic retinopathy	EFO_0003770	Neurological disorder	Neurological	PGS001297	MONDO_0005147	type 1 diabetes mellitus	type 1 diabetes mellitus	MONDO_0005147	Metabolic disorder	Metabolic
0.7497	1.37E-83	1.74E-80	PGS001819	EFO_0003770	diabetic retinopathy	diabetic retinopathy	EFO_0003770	Metabolic disorder	Metabolic	PGS001297	MONDO_0005147	type 1 diabetes mellitus	type 1 diabetes mellitus	MONDO_0005147	Digestive system disorder	Gastrointestinal
0.7497	1.37E-83	1.74E-80	PGS001819	EFO_0003770	diabetic retinopathy	diabetic retinopathy	EFO_0003770	Neurological disorder	Neurological	PGS001297	MONDO_0005147	type 1 diabetes mellitus	type 1 diabetes mellitus	MONDO_0005147	Digestive system disorder	Gastrointestinal
0.7497	1.37E-83	1.74E-80	PGS001819	EFO_0003770	diabetic retinopathy	diabetic retinopathy	EFO_0003770	Cardiovascular disease	Cardiovascular	PGS001297	MONDO_0005147	type 1 diabetes mellitus	type 1 diabetes mellitus	MONDO_0005147	Digestive system disorder	Gastrointestinal
0.7497	1.37E-83	1.74E-80	PGS001819	EFO_0003770	diabetic retinopathy	diabetic retinopathy	EFO_0003770	Metabolic disorder	Metabolic	PGS001297	MONDO_0005147	type 1 diabetes mellitus	type 1 diabetes mellitus	MONDO_0005147	Metabolic disorder	Metabolic
0.7497	1.37E-83	1.74E-80	PGS001819	EFO_0003770	diabetic retinopathy	diabetic retinopathy	EFO_0003770	Metabolic disorder	Metabolic	PGS001297	MONDO_0005147	type 1 diabetes mellitus	type 1 diabetes mellitus	MONDO_0005147	Immune system disorder	Immunological
0.7497	1.37E-83	1.74E-80	PGS001819	EFO_0003770	diabetic retinopathy	diabetic retinopathy	EFO_0003770	Cardiovascular disease	Cardiovascular	PGS001297	MONDO_0005147	type 1 diabetes mellitus	type 1 diabetes mellitus	MONDO_0005147	Metabolic disorder	Metabolic
0.7497	1.37E-83	1.74E-80	PGS001819	EFO_0003770	diabetic retinopathy	diabetic retinopathy	EFO_0003770	Neurological disorder	Neurological	PGS001297	MONDO_0005147	type 1 diabetes mellitus	type 1 diabetes mellitus	MONDO_0005147	Immune system disorder	Immunological
0.7496	1.40E-83	1.78E-80	PGS002970	EFO_0004339	body height	body height	EFO_0004339	Body measurement	Body measurement	PGS002146	EFO_0004339	body height	body height	EFO_0004339	Body measurement	Body measurement
0.7496	1.43E-83	1.81E-80	PGS000501	EFO_0000305	breast carcinoma	breast carcinoma	EFO_0000305	Cancer	Neoplasms	PGS000506	EFO_0000305	breast carcinoma	breast carcinoma	EFO_0000305	Cancer	Neoplasms
0.7496	1.43E-83	1.81E-80	PGS000501	EFO_0000305	breast carcinoma	breast carcinoma	EFO_0000305	Cancer	Neoplasms	PGS000505	EFO_0000305	breast carcinoma	breast carcinoma	EFO_0000305	Cancer	Neoplasms
-0.7495	1.55E-83	1.96E-80	PGS003068	EFO_0006527	smoking status measurement	smoking status measurement	EFO_0006527	Other measurement	Psychiatric	PGS003360	EFO_0005670	smoking initiation	smoking initiation	EFO_0005670	Biological process	Psychiatric
0.7494	1.61E-83	2.04E-80	PGS000950	GO_0009314	response to radiation	response to radiation	GO_0009314	Biological process	Others	PGS001072	EFO_0008006	skin aging measurement	skin aging measurement	EFO_0008006	Other measurement	Dermatological
0.7494	1.63E-83	2.06E-80	PGS002176	EFO_0005093	hip circumference	hip circumference	EFO_0005093	Body measurement	Body measurement	PGS002133	EFO_0007800	body fat percentage	body fat percentage	EFO_0007800	Body measurement	Body measurement
0.7494	1.73E-83	2.19E-80	PGS002337	EFO_0004611	low density lipoprotein cholesterol measurement	low density lipoprotein cholesterol measurement	EFO_0004611	Lipid or lipoprotein measurement	Metabolic	PGS000888	EFO_0004611	low density lipoprotein cholesterol measurement	measurement	EFO_0004611	Lipid or lipoprotein measurement	Metabolic
0.7494	1.73E-83	2.19E-80	PGS002337	EFO_0004611	low density lipoprotein cholesterol measurement	low density lipoprotein cholesterol measurement	EFO_0004611	Cardiovascular measurement	Cardiovascular	PGS000888	EFO_0004611	low density lipoprotein cholesterol measurement	measurement	EFO_0004611	Cardiovascular measurement	Cardiovascular
0.7494	1.73E-83	2.19E-80	PGS002337	EFO_0004611	low density lipoprotein cholesterol measurement	low density lipoprotein cholesterol measurement	EFO_0004611	Lipid or lipoprotein measurement	Metabolic	PGS000888	EFO_0004611	low density lipoprotein cholesterol measurement	measurement	EFO_0004611	Cardiovascular measurement	Cardiovascular
0.7494	1.73E-83	2.19E-80	PGS002337	EFO_0004611	low density lipoprotein cholesterol measurement	low density lipoprotein cholesterol measurement	EFO_0004611	Cardiovascular measurement	Cardiovascular	PGS000888	EFO_0004611	low density lipoprotein cholesterol measurement	measurement	EFO_0004611	Lipid or lipoprotein measurement	Metabolic
0.7493	1.74E-83	2.21E-80	PGS002656	EFO_0004527	mean corpuscular hemoglobin	mean corpuscular hemoglobin	EFO_0004527	Hematological measurement	Hematological	PGS000099	EFO_0004527	mean corpuscular hemoglobin	mean corpuscular hemoglobin	EFO_0004527	Hematological measurement	Hematological
0.7493	1.81E-83	2.29E-80	PGS003103	MONDO_0005148	type 2 diabetes mellitus	type 2 diabetes mellitus	MONDO_0005148	Metabolic disorder	Metabolic	PGS003114	MONDO_0005148	type 2 diabetes mellitus	type 2 diabetes mellitus	MONDO_0005148	Digestive system disorder	Gastrointestinal
0.7493	1.81E-83	2.29E-80	PGS003103	MONDO_0005148	type 2 diabetes mellitus	type 2 diabetes mellitus	MONDO_0005148	Digestive system disorder	Gastrointestinal	PGS003114	MONDO_0005148	type 2 diabetes mellitus	type 2 diabetes mellitus	MONDO_0005148	Metabolic disorder	Metabolic
0.7493	1.81E-83	2.29E-80	PGS003103	MONDO_0005148	type 2 diabetes mellitus	type 2 diabetes mellitus	MONDO_0005148	Digestive system disorder	Gastrointestinal	PGS003114	MONDO_0005148	type 2 diabetes mellitus	type 2 diabetes mellitus	MONDO_0005148	Digestive system disorder	Gastrointestinal
0.7493	1.81E-83	2.29E-80	PGS003103	MONDO_0005148	type 2 diabetes mellitus	type 2 diabetes mellitus	MONDO_0005148	Metabolic disorder	Metabolic	PGS003114	MONDO_0005148	type 2 diabetes mellitus	type 2 diabetes mellitus	MONDO_0005148	Metabolic disorder	Metabolic
0.7492	1.90E-83	2.40E-80	PGS000310	EFO_0004611	low density lipoprotein cholesterol measurement	low density lipoprotein cholesterol measurement	EFO_0004611	Cardiovascular measurement	Cardiovascular	PGS002337	EFO_0004611	low density lipoprotein cholesterol measurement	measurement	EFO_0004611	Cardiovascular measurement	Cardiovascular
0.7492	1.90E-83	2.40E-80	PGS000310	EFO_0004611	low density lipoprotein cholesterol measurement	low density lipoprotein cholesterol measurement	EFO_0004611	Lipid or lipoprotein measurement	Metabolic	PGS002337	EFO_0004611	low density lipoprotein cholesterol measurement	measurement	EFO_0004611	Cardiovascular measurement	Cardiovascular
0.7492	1.90E-83	2.40E-80	PGS000310	EFO_0004611	low density lipoprotein cholesterol measurement	low density lipoprotein cholesterol measurement	EFO_0004611	Cardiovascular measurement	Cardiovascular	PGS002337	EFO_0004611	low density lipoprotein cholesterol measurement	measurement	EFO_0004611	Lipid or lipoprotein measurement	Metabolic
0.7492	1.90E-83	2.40E-80	PGS000310	EFO_0004611	low density lipoprotein cholesterol measurement	low density lipoprotein cholesterol measurement	EFO_0004611	Lipid or lipoprotein measurement	Metabolic	PGS002337	EFO_0004611	low density lipoprotein cholesterol measurement	measurement	EFO_0004611	Lipid or lipoprotein measurement	Metabolic
0.7492	1.90E-83	2.41E-80	PGS000511	EFO_0000305	breast carcinoma	breast carcinoma	EFO_0000305	Cancer	Neoplasms	PGS000007	EFO_0000305	breast carcinoma	breast carcinoma	EFO_0000305	Cancer	Neoplasms
0.7491	2.17E-83	2.75E-80	PGS000539	EFO_0000305	breast carcinoma	breast carcinoma	EFO_0000305	Cancer	Neoplasms	PGS000534	EFO_0000305	breast carcinoma	breast carcinoma	EFO_0000305	Cancer	Neoplasms
0.7491	2.17E-83	2.75E-80	PGS000539	EFO_0000305	breast carcinoma	breast carcinoma	EFO_0000305	Cancer	Neoplasms	PGS000533	EFO_0000305	breast carcinoma	breast carcinoma	EFO_0000305	Cancer	Neoplasms
0.749	2.22E-83	2.81E-80	PGS002337	EFO_0004611	low density lipoprotein cholesterol measurement	low density lipoprotein cholesterol measurement	EFO_0004611	Cardiovascular measurement	Cardiovascular	PGS003143	EFO_0004574	total cholesterol measurement	total cholesterol measurement	EFO_0004574	Lipid or lipoprotein measurement	Metabolic
0.749	2.22E-83	2.81E-80	PGS002337	EFO_0004611	low density lipoprotein cholesterol measurement	low density lipoprotein cholesterol measurement	EFO_0004611	Lipid or lipoprotein measurement	Metabolic	PGS003143	EFO_0004574	total cholesterol measurement	total cholesterol measurement	EFO_0004574	Lipid or lipoprotein measurement	Metabolic
0.749	2.26E-83	2.86E-80	PGS001735	EFO_0006930	brain volume measurement|neuroimaging measurement	brain volume measurement	EFO_0006930	Other measurement	Neurological	PGS001723	EFO_0006930	measurement	brain volume measurement	EFO_0006930	Other measurement	Neurological
0.749	2.29E-83	2.90E-80	PGS001219	EFO_0004527	mean corpuscular hemoglobin	mean corpuscular hemoglobin	EFO_0004527	Hematological measurement	Hematological	PGS000099	EFO_0004527	mean corpuscular hemoglobin	mean corpuscular hemoglobin	EFO_0004527	Hematological measurement	Hematological
0.749	2.30E-83	2.91E-80	PGS001227	EFO_0004342	waist circumference	waist circumference	EFO_0004342	Body measurement	Body measurement	PGS001101	EFO_0007800	body fat percentage	body fat percentage	EFO_0007800	Body measurement	Body measurement
0.7489	2.44E-83	3.08E-80	PGS000401	EFO_0009259	skin carcinoma	skin carcinoma	EFO_0009259	Cancer	Neoplasms	PGS000405	EFO_0009259	skin carcinoma	skin carcinoma	EFO_0009259	Cancer	Neoplasms
0.7488	2.63E-83	3.32E-80	PGS001983	EFO_0004342	waist circumference	waist circumference	EFO_0004342	Body measurement	Body measurement	PGS002313	EFO_0004340	body mass index	body mass index	EFO_0004340	Body measurement	Body measurement
0.7488	2.63E-83	3.33E-80	PGS003380	EFO_0000305	breast carcinoma	breast carcinoma	EFO_0000305	Cancer	Neoplasms	PGS000332	EFO_0000305	breast carcinoma	breast carcinoma	EFO_0000305	Cancer	Neoplasms
0.7488	2.74E-83	3.45E-80	PGS003038	EFO_0004611	low density lipoprotein cholesterol measurement	low density lipoprotein cholesterol measurement	EFO_0004611	Cardiovascular measurement	Cardiovascular	PGS002352	EFO_0004574	total cholesterol measurement	total cholesterol measurement	EFO_0004574	Lipid or lipoprotein measurement	Metabolic
0.7488	2.74E-83	3.45E-80	PGS003038	EFO_0004611	low density lipoprotein cholesterol measurement	low density lipoprotein cholesterol measurement	EFO_0004611	Lipid or lipoprotein measurement	Metabolic	PGS002352	EFO_0004574	total cholesterol measurement	total cholesterol measurement	EFO_0004574	Lipid or lipoprotein measurement	Metabolic
0.7487	2.91E-83	3.68E-80	PGS002321	EFO_0000400	diabetes mellitus	diabetes mellitus	EFO_0000400	Metabolic disorder	Metabolic	PGS002638	EFO_0000400	diabetes mellitus	diabetes mellitus	EFO_0000400	Digestive system disorder	Gastrointestinal
0.7487	2.91E-83	3.68E-80	PGS002321	EFO_0000400	diabetes mellitus	diabetes mellitus	EFO_0000400	Digestive system disorder	Gastrointestinal	PGS002638	EFO_0000400	diabetes mellitus	diabetes mellitus	EFO_0000400	Digestive system disorder	Gastrointestinal
0.7487	2.91E-83	3.68E-80	PGS002321	EFO_0000400	diabetes mellitus	diabetes mellitus	EFO_0000400	Digestive system disorder	Gastrointestinal	PGS002638	EFO_0000400	diabetes mellitus	diabetes mellitus	EFO_0000400	Metabolic disorder	Metabolic
0.7487	2.91E-83	3.68E-80	PGS002321	EFO_0000400	diabetes mellitus	diabetes mellitus	EFO_0000400	Metabolic disorder	Metabolic	PGS002638	EFO_0000400	diabetes mellitus	diabetes mellitus	EFO_0000400	Metabolic disorder	Metabolic
0.7487	2.98E-83	3.76E-80	PGS002844	EFO_0004340	body mass index	body mass index	EFO_0004340	Body measurement	Body measurement	PGS002201	EFO_0004342	waist circumference	waist circumference	EFO_0004342	Body measurement	Body measurement
0.7486	3.10E-83	3.91E-80	PGS000437	MONDO_0002898	skin cancer	skin cancer	MONDO_0002898	Cancer	Neoplasms	PGS000404	EFO_0009259	skin carcinoma	skin carcinoma	EFO_0009259	Cancer	Neoplasms
0.7486	3.10E-83	3.91E-80	PGS001674	EFO_0006930	brain volume measurement|neuroimaging measurement	brain volume measurement	EFO_0006930	Other measurement	Neurological	PGS001476	EFO_0006930	measurement	brain volume measurement	EFO_0006930	Other measurement	Neurological
0.7486	3.14E-83	3.96E-80	PGS000051	EFO_0000305	breast carcinoma	breast carcinoma	EFO_0000305	Cancer	Neoplasms	PGS000774	EFO_1000649	estrogen-receptor positive breast cancer	cancer	EFO_1000649	Cancer	Neoplasms
0.7486	3.22E-83	4.06E-80	PGS000177	EFO_0005091	monocyte count	monocyte count	EFO_0005091	Hematological measurement	Hematological	PGS002658	EFO_0005091	monocyte count	monocyte count	EFO_0005091	Hematological measurement	Hematological
0.7485	3.24E-83	4.08E-80	PGS002242	EFO_0000305	breast carcinoma	breast carcinoma	EFO_0000305	Cancer	Neoplasms	PGS000332	EFO_0000305	breast carcinoma	breast carcinoma	EFO_0000305	Cancer	Neoplasms
0.7485	3.25E-83	4.09E-80	PGS002603	EFO_0000537	hypertension	hypertension	EFO_0000537	Cardiovascular disease	Cardiovascular	PGS002590	EFO_0006336	diastolic blood pressure	diastolic blood pressure	EFO_0006336	Other measurement	Cardiovascular
0.7485	3.27E-83	4.12E-80	PGS001817	MONDO_0005147	type 1 diabetes mellitus	type 1 diabetes mellitus	MONDO_0005147	Digestive system disorder	Gastrointestinal	PGS000023	MONDO_0005147	type 1 diabetes mellitus	type 1 diabetes mellitus	MONDO_0005147	Digestive system disorder	Gastrointestinal
0.7485	3.27E-83	4.12E-80	PGS001817	MONDO_0005147	type 1 diabetes mellitus	type 1 diabetes mellitus	MONDO_0005147	Metabolic disorder	Metabolic	PGS000023	MONDO_0005147	type 1 diabetes mellitus	type 1 diabetes mellitus	MONDO_0005147	Digestive system disorder	Gastrointestinal
0.7485	3.27E-83	4.12E-80	PGS001817	MONDO_0005147	type 1 diabetes mellitus	type 1 diabetes mellitus	MONDO_0005147	Metabolic disorder	Metabolic	PGS000023	MONDO_0005147	type 1 diabetes mellitus	type 1 diabetes mellitus	MONDO_0005147	Metabolic disorder	Metabolic
0.7485	3.27E-83	4.12E-80	PGS001817	MONDO_0005147	type 1 diabetes mellitus	type 1 diabetes mellitus	MONDO_0005147	Digestive system disorder	Gastrointestinal	PGS000023	MONDO_0005147	type 1 diabetes mellitus	type 1 diabetes mellitus	MONDO_0005147	Immune system disorder	Immunological
0.7485	3.27E-83	4.12E-80	PGS001817	MONDO_0005147	type 1 diabetes mellitus	type 1 diabetes mellitus	MONDO_0005147	Immune system disorder	Immunological	PGS000023	MONDO_0005147	type 1 diabetes mellitus	type 1 diabetes mellitus	MONDO_0005147	Immune system disorder	Immunological
0.7485	3.27E-83	4.12E-80	PGS001817	MONDO_0005147	type 1 diabetes mellitus	type 1 diabetes mellitus	MONDO_0005147	Metabolic disorder	Metabolic	PGS000023	MONDO_0005147	type 1 diabetes mellitus	type 1 diabetes mellitus	MONDO_0005147	Immune system disorder	Immunological

0.7485	3.27E-83	4.12E-80	PGS001817	MONDO_0005147	type 1 diabetes mellitus	type 1 diabetes mellitus	MONDO_0005147	Digestive system disorder	Gastrointestinal	PGS000023	MONDO_0005147	type 1 diabetes mellitus	type 1 diabetes mellitus	MONDO_0005147	Metabolic disorder	Metabolic
0.7485	3.27E-83	4.12E-80	PGS001817	MONDO_0005147	type 1 diabetes mellitus	type 1 diabetes mellitus	MONDO_0005147	Immune system disorder	Immunological	PGS000023	MONDO_0005147	type 1 diabetes mellitus	type 1 diabetes mellitus	MONDO_0005147	Digestive system disorder	Gastrointestinal
0.7485	3.27E-83	4.12E-80	PGS001817	MONDO_0005147	type 1 diabetes mellitus	type 1 diabetes mellitus	MONDO_0005147	Immune system disorder	Immunological	PGS000023	MONDO_0005147	type 1 diabetes mellitus	type 1 diabetes mellitus	MONDO_0005147	Metabolic disorder	Metabolic
0.7485	3.30E-83	4.16E-80	PGS000046	EFO_1000649	estrogen-receptor positive breast cancer	estrogen-receptor positive breast cancer	EFO_1000649	Cancer	Neoplasms	PGS000773	EFO_0000305	breast carcinoma	breast carcinoma	EFO_0000305	Cancer	Neoplasms
0.7485	3.31E-83	4.16E-80	PGS001152	EFO_0005192	red blood cell distribution width	red blood cell distribution width	EFO_0005192	Hematological measurement	Hematological	PGS002346	EFO_0005192	red blood cell distribution width	red blood cell distribution width	EFO_0005192	Hematological measurement	Hematological
0.7485	3.36E-83	4.22E-80	PGS003143	EFO_0004574	total cholesterol measurement	total cholesterol measurement	EFO_0004574	Lipid or lipoprotein measurement	Metabolic	PGS002782	EFO_0005689	measurement	measurement	EFO_0005689	Lipid or lipoprotein measurement	Metabolic
0.7485	3.36E-83	4.22E-80	PGS003143	EFO_0004574	total cholesterol measurement	total cholesterol measurement	EFO_0004574	Lipid or lipoprotein measurement	Metabolic	PGS002782	EFO_0005689	measurement	measurement	EFO_0005689	Cardiovascular measurement	Cardiovascular
0.7485	3.37E-83	4.24E-80	PGS001979	EFO_0004530	triglyceride measurement	triglyceride measurement	EFO_0004530	Lipid or lipoprotein measurement	Metabolic	PGS000699	EFO_0004530	triglyceride measurement	triglyceride measurement	EFO_0004530	Lipid or lipoprotein measurement	Metabolic
0.7484	3.75E-83	4.71E-80	PGS002227	EFO_0004503	hematological measurement	hematological measurement	EFO_0004503	Hematological measurement	Hematological	PGS002207	EFO_0004526	mean corpuscular volume	mean corpuscular volume	EFO_0004526	Hematological measurement	Hematological
0.7483	3.88E-83	4.88E-80	PGS002351	EFO_1000627	thyroid disease	thyroid disease	EFO_1000627	Other disease	Endocrine	PGS000965	EFO_1001055	myxedema|hypothyroidism	myxedema	EFO_1001055	Other disease	Endocrine
0.7483	3.88E-83	4.88E-80	PGS000539	EFO_0000305	breast carcinoma	breast carcinoma	EFO_0000305	Cancer	Neoplasms	PGS000007	EFO_0000305	breast carcinoma	breast carcinoma	EFO_0000305	Cancer	Neoplasms
0.7483	3.91E-83	4.92E-80	PGS000098	EFO_0007993	lymphocyte percentage of leukocytes	lymphocyte percentage of leukocytes	EFO_0007993	Hematological measurement	Hematological	PGS001986	EFO_0007993	lymphocyte percentage of leukocytes	lymphocyte percentage of leukocytes	EFO_0007993	Hematological measurement	Hematological
0.7482	4.15E-83	5.21E-80	PGS000539	EFO_0000305	breast carcinoma	breast carcinoma	EFO_0000305	Cancer	Neoplasms	PGS000505	EFO_0000305	breast carcinoma	breast carcinoma	EFO_0000305	Cancer	Neoplasms
0.7482	4.15E-83	5.21E-80	PGS000539	EFO_0000305	breast carcinoma	breast carcinoma	EFO_0000305	Cancer	Neoplasms	PGS000506	EFO_0000305	breast carcinoma	breast carcinoma	EFO_0000305	Cancer	Neoplasms
0.7481	4.44E-83	5.57E-80	PGS003200	EFO_0004631	vitamin D measurement	vitamin D measurement	EFO_0004631	Lipid or lipoprotein measurement	Metabolic	PGS003196	EFO_0004631	vitamin D measurement	vitamin D measurement	EFO_0004631	Lipid or lipoprotein measurement	Metabolic
0.7481	4.45E-83	5.59E-80	PGS001329	EFO_0000400	diabetes mellitus	diabetes mellitus	EFO_0000400	Digestive system disorder	Gastrointestinal	PGS001294	MONDO_0005148	type 2 diabetes mellitus	type 2 diabetes mellitus	MONDO_0005148	Digestive system disorder	Gastrointestinal
0.7481	4.45E-83	5.59E-80	PGS001329	EFO_0000400	diabetes mellitus	diabetes mellitus	EFO_0000400	Metabolic disorder	Metabolic	PGS001294	MONDO_0005148	type 2 diabetes mellitus	type 2 diabetes mellitus	MONDO_0005148	Digestive system disorder	Gastrointestinal
0.7481	4.45E-83	5.59E-80	PGS001329	EFO_0000400	diabetes mellitus	diabetes mellitus	EFO_0000400	Digestive system disorder	Gastrointestinal	PGS001294	MONDO_0005148	type 2 diabetes mellitus	type 2 diabetes mellitus	MONDO_0005148	Metabolic disorder	Metabolic
0.7481	4.45E-83	5.59E-80	PGS001329	EFO_0000400	diabetes mellitus	diabetes mellitus	EFO_0000400	Metabolic disorder	Metabolic	PGS001294	MONDO_0005148	type 2 diabetes mellitus	type 2 diabetes mellitus	MONDO_0005148	Metabolic disorder	Metabolic
0.7481	4.58E-83	5.74E-80	PGS002922	EFO_0004697	estradiol measurement	estradiol measurement	EFO_0004697	Other measurement	Endocrine	PGS002921	EFO_0004697	estradiol measurement	estradiol measurement	EFO_0004697	Other measurement	Endocrine
0.7481	4.69E-83	5.89E-80	PGS000539	EFO_0000305	breast carcinoma	breast carcinoma	EFO_0000305	Cancer	Neoplasms	PGS000511	EFO_0000305	breast carcinoma	breast carcinoma	EFO_0000305	Cancer	Neoplasms
-0.7481	4.70E-83	5.89E-80	PGS001994	HP_0000545	Myopia	Myopia	HP_0000545	Other trait	Ophthalmological	PGS002103	EFO_0008385	visual acuity measurement	visual acuity measurement	EFO_0008385	Other measurement	Ophthalmological
0.7479	5.26E-83	6.59E-80	PGS003099	MONDO_0005148	type 2 diabetes mellitus	type 2 diabetes mellitus	MONDO_0005148	Digestive system disorder	Gastrointestinal	PGS003117	MONDO_0005148	type 2 diabetes mellitus	type 2 diabetes mellitus	MONDO_0005148	Digestive system disorder	Gastrointestinal
0.7479	5.26E-83	6.59E-80	PGS003099	MONDO_0005148	type 2 diabetes mellitus	type 2 diabetes mellitus	MONDO_0005148	Digestive system disorder	Gastrointestinal	PGS003117	MONDO_0005148	type 2 diabetes mellitus	type 2 diabetes mellitus	MONDO_0005148	Metabolic disorder	Metabolic
0.7479	5.26E-83	6.59E-80	PGS003099	MONDO_0005148	type 2 diabetes mellitus	type 2 diabetes mellitus	MONDO_0005148	Metabolic disorder	Metabolic	PGS003117	MONDO_0005148	type 2 diabetes mellitus	type 2 diabetes mellitus	MONDO_0005148	Metabolic disorder	Metabolic
0.7479	5.26E-83	6.59E-80	PGS003099	MONDO_0005148	type 2 diabetes mellitus	type 2 diabetes mellitus	MONDO_0005148	Metabolic disorder	Metabolic	PGS003117	MONDO_0005148	type 2 diabetes mellitus	type 2 diabetes mellitus	MONDO_0005148	Digestive system disorder	Gastrointestinal
0.7479	5.26E-83	6.60E-80	PGS000382	EFO_1001950	colon carcinoma	colon carcinoma	EFO_1001950	Digestive system disorder	Gastrointestinal	PGS000381	EFO_1001950	colon carcinoma	colon carcinoma	EFO_1001950	Digestive system disorder	Gastrointestinal
0.7479	5.26E-83	6.60E-80	PGS000382	EFO_1001950	colon carcinoma	colon carcinoma	EFO_1001950	Cancer	Neoplasms	PGS000381	EFO_1001950	colon carcinoma	colon carcinoma	EFO_1001950	Cancer	Neoplasms
0.7479	5.26E-83	6.60E-80	PGS000382	EFO_1001950	colon carcinoma	colon carcinoma	EFO_1001950	Cancer	Neoplasms	PGS000381	EFO_1001950	colon carcinoma	colon carcinoma	EFO_1001950	Digestive system disorder	Gastrointestinal
0.7479	5.26E-83	6.60E-80	PGS000382	EFO_1001950	colon carcinoma	colon carcinoma	EFO_1001950	Digestive system disorder	Gastrointestinal	PGS000381	EFO_1001950	colon carcinoma	colon carcinoma	EFO_1001950	Cancer	Neoplasms
0.7479	5.40E-83	6.77E-80	PGS002150	EFO_0004611	low density lipoprotein cholesterol measurement	low density lipoprotein cholesterol measurement	EFO_0004611	Cardiovascular measurement	Cardiovascular	PGS003038	EFO_0004611	low density lipoprotein cholesterol measurement	measurement	EFO_0004611	Cardiovascular measurement	Cardiovascular
0.7479	5.40E-83	6.77E-80	PGS002150	EFO_0004611	low density lipoprotein cholesterol measurement	low density lipoprotein cholesterol measurement	EFO_0004611	Cardiovascular measurement	Cardiovascular	PGS003038	EFO_0004611	low density lipoprotein cholesterol measurement	measurement	EFO_0004611	Lipid or lipoprotein measurement	Metabolic
0.7479	5.40E-83	6.77E-80	PGS002150	EFO_0004611	low density lipoprotein cholesterol measurement	low density lipoprotein cholesterol measurement	EFO_0004611	Lipid or lipoprotein measurement	Metabolic	PGS003038	EFO_0004611	low density lipoprotein cholesterol measurement	measurement	EFO_0004611	Cardiovascular measurement	Cardiovascular
0.7479	5.40E-83	6.77E-80	PGS002150	EFO_0004611	low density lipoprotein cholesterol measurement	low density lipoprotein cholesterol measurement	EFO_0004611	Lipid or lipoprotein measurement	Metabolic	PGS003038	EFO_0004611	low density lipoprotein cholesterol measurement	measurement	EFO_0004611	Lipid or lipoprotein measurement	Metabolic
0.7479	5.40E-83	6.77E-80	PGS001991	EFO_0007989	monocyte percentage of leukocytes	monocyte percentage of leukocytes	EFO_0007989	Hematological measurement	Hematological	PGS001968	EFO_0005091	monocyte count	monocyte count	EFO_0005091	Hematological measurement	Hematological
0.7478	5.59E-83	7.00E-80	PGS001945	EFO_0004518	creatinine measurement	creatinine measurement	EFO_0004518	Other measurement	Metabolic	PGS003239	EFO_0010472	creatine measurement	creatine measurement	EFO_0010472	Other measurement	Metabolic
-0.7478	5.73E-83	7.18E-80	PGS003228	EFO_0005208	glomerular filtration rate	glomerular filtration rate	EFO_0005208	Other measurement	Genitourinary	PGS003243	EFO_0010472	creatine measurement	creatine measurement	EFO_0010472	Other measurement	Metabolic
0.7478	5.81E-83	7.27E-80	PGS003102	MONDO_0005148	type 2 diabetes mellitus	type 2 diabetes mellitus	MONDO_0005148	Metabolic disorder	Metabolic	PGS003099	MONDO_0005148	type 2 diabetes mellitus	type 2 diabetes mellitus	MONDO_0005148	Digestive system disorder	Gastrointestinal
0.7478	5.81E-83	7.27E-80	PGS003102	MONDO_0005148	type 2 diabetes mellitus	type 2 diabetes mellitus	MONDO_0005148	Digestive system disorder	Gastrointestinal	PGS003099	MONDO_0005148	type 2 diabetes mellitus	type 2 diabetes mellitus	MONDO_0005148	Digestive system disorder	Gastrointestinal
0.7478	5.81E-83	7.27E-80	PGS003102	MONDO_0005148	type 2 diabetes mellitus	type 2 diabetes mellitus	MONDO_0005148	Digestive system disorder	Gastrointestinal	PGS003099	MONDO_0005148	type 2 diabetes mellitus	type 2 diabetes mellitus	MONDO_0005148	Metabolic disorder	Metabolic
0.7478	5.81E-83	7.27E-80	PGS003102	MONDO_0005148	type 2 diabetes mellitus	type 2 diabetes mellitus	MONDO_0005148	Metabolic disorder	Metabolic	PGS003099	MONDO_0005148	type 2 diabetes mellitus	type 2 diabetes mellitus	MONDO_0005148	Metabolic disorder	Metabolic
0.7478	5.88E-83	7.35E-80	PGS002005	EFO_0011011	sitting height measurement	sitting height measurement	EFO_0011011	Other measurement	Skeletal	PGS002973	EFO_0004339	body height	body height	EFO_0004339	Body measurement	Body measurement
0.7478	5.98E-83	7.48E-80	PGS002656	EFO_0004527	mean corpuscular hemoglobin	mean corpuscular hemoglobin	EFO_0004527	Hematological measurement	Hematological	PGS001989	EFO_0004527	mean corpuscular hemoglobin	mean corpuscular hemoglobin	EFO_0004527	Hematological measurement	Hematological
0.7477	6.10E-83	7.63E-80	PGS002005	EFO_0011011	sitting height measurement	sitting height measurement	EFO_0011011	Other measurement	Skeletal	PGS000758	EFO_0004339	body height	body height	EFO_0004339	Body measurement	Body measurement
0.7477	6.13E-83	7.67E-80	PGS000729	MONDO_0005148	type 2 diabetes mellitus	type 2 diabetes mellitus	MONDO_0005148	Metabolic disorder	Metabolic	PGS002771	MONDO_0005148	type 2 diabetes mellitus	type 2 diabetes mellitus	MONDO_0005148	Digestive system disorder	Gastrointestinal
0.7477	6.13E-83	7.67E-80	PGS000729	MONDO_0005148	type 2 diabetes mellitus	type 2 diabetes mellitus	MONDO_0005148	Digestive system disorder	Gastrointestinal	PGS002771	MONDO_0005148	type 2 diabetes mellitus	type 2 diabetes mellitus	MONDO_0005148	Digestive system disorder	Gastrointestinal
0.7477	6.13E-83	7.67E-80	PGS000729	MONDO_0005148	type 2 diabetes mellitus	type 2 diabetes mellitus	MONDO_0005148	Metabolic disorder	Metabolic	PGS002771	MONDO_0005148	type 2 diabetes mellitus	type 2 diabetes mellitus	MONDO_0005148	Metabolic disorder	Metabolic
0.7477	6.13E-83	7.67E-80	PGS000729	MONDO_0005148	type 2 diabetes mellitus	type 2 diabetes mellitus	MONDO_0005148	Digestive system disorder	Gastrointestinal	PGS002771	MONDO_0005148	type 2 diabetes mellitus	type 2 diabetes mellitus	MONDO_0005148	Metabolic disorder	Metabolic
0.7477	6.19E-83	7.74E-80	PGS002150	EFO_0004611	low density lipoprotein cholesterol measurement	low density lipoprotein cholesterol measurement	EFO_0004611	Cardiovascular measurement	Cardiovascular	PGS000888	EFO_0004611	low density lipoprotein cholesterol measurement	measurement	EFO_0004611	Lipid or lipoprotein measurement	Metabolic
0.7477	6.19E-83	7.74E-80	PGS002150	EFO_0004611	low density lipoprotein cholesterol measurement	low density lipoprotein cholesterol measurement	EFO_0004611	Lipid or lipoprotein measurement	Metabolic	PGS000888	EFO_0004611	low density lipoprotein cholesterol measurement	measurement	EFO_0004611	Cardiovascular measurement	Cardiovascular
0.7477	6.19E-83	7.74E-80	PGS002150	EFO_0004611	low density lipoprotein cholesterol measurement	low density lipoprotein cholesterol measurement	EFO_0004611	Cardiovascular measurement	Cardiovascular	PGS000888	EFO_0004611	low density lipoprotein cholesterol measurement	measurement	EFO_0004611	Cardiovascular measurement	Cardiovascular
0.7477	6.19E-83	7.74E-80	PGS002150	EFO_0004611	low density lipoprotein cholesterol measurement	low density lipoprotein cholesterol measurement	EFO_0004611	Lipid or lipoprotein measurement	Metabolic	PGS000888	EFO_0004611	low density lipoprotein cholesterol measurement	measurement	EFO_0004611	Lipid or lipoprotein measurement	Metabolic
0.7477	6.31E-83	7.88E-80	PGS000044	EFO_0001663	prostate carcinoma	prostate carcinoma	EFO_0001663	Cancer	Neoplasms	PGS000580	EFO_0001663	prostate carcinoma	prostate carcinoma	EFO_0001663	Cancer	Neoplasms
0.7476	6.80E-83	8.50E-80	PGS000900	EFO_0006335	systolic blood pressure|female	systolic blood pressure	EFO_0006335	Other measurement	Cardiovascular	PGS002765	EFO_0000537	hypertension	hypertension	EFO_0000537	Cardiovascular disease	Cardiovascular
0.7476	6.91E-83	8.63E-80	PGS000783	EFO_0000305	breast carcinoma	breast carcinoma	EFO_0000305	Cancer	Neoplasms	PGS000212	MONDO_0021116	luminal A breast carcinoma	luminal A breast carcinoma	MONDO_0021116	Cancer	Neoplasms
0.7475	7.09E-83	8.86E-80	PGS002150	EFO_0004611	low density lipoprotein cholesterol measurement	low density lipoprotein cholesterol measurement	EFO_0004611	Cardiovascular measurement	Cardiovascular	PGS000892	EFO_0004611	low density lipoprotein cholesterol measurement	measurement	EFO_0004611	Cardiovascular measurement	Cardiovascular
0.7475	7.09E-83	8.86E-80	PGS002150	EFO_0004611	low density lipoprotein cholesterol measurement	low density lipoprotein cholesterol measurement	EFO_0004611	Cardiovascular measurement	Cardiovascular	PGS000892	EFO_0004611	low density lipoprotein cholesterol measurement	measurement	EFO_0004611	Lipid or lipoprotein measurement	Metabolic
0.7475	7.09E-83	8.86E-80	PGS002150	EFO_0004611	low density lipoprotein cholesterol measurement	low density lipoprotein cholesterol measurement	EFO_0004611	Lipid or lipoprotein measurement	Metabolic	PGS000892	EFO_0004611	low density lipoprotein cholesterol measurement	measurement	EFO_0004611	Cardiovascular measurement	Cardiovascular
0.7475	7.09E-83	8.86E-80	PGS002150	EFO_0004611	low density lipoprotein cholesterol measurement	low density lipoprotein cholesterol measurement	EFO_0004611	Lipid or lipoprotein measurement	Metabolic	PGS000892	EFO_0004611	low density lipoprotein cholesterol measurement	measurement	EFO_0004611	Lipid or lipoprotein measurement	Metabolic
0.7475	7.28E-83	9.10E-80	PGS001678	EFO_0006930	brain volume measurement|neuroimaging measurement	brain volume measurement	EFO_0006930	Other measurement	Neurological	PGS001686	EFO_0006930	measurement	brain volume measurement	EFO_0006930	Other measurement	Neurological
0.7475	7.54E-83	9.42E-80	PGS002315	EFO_0003923	bone density	bone density	EFO_0003923	Other measurement	Skeletal	PGS000951	EFO_0004514	bone quantitative ultrasound measurement	measurement	EFO_0004514	Other measurement	Skeletal
0.7474	7.80E-83	9.74E-80	PGS003189	EFO_0004631	vitamin D measurement	vitamin D measurement	EFO_0004631	Lipid or lipoprotein measurement	Metabolic	PGS002721	EFO_0004631	vitamin D measurement	vitamin D measurement	EFO_0004631	Lipid or lipoprotein measurement	Metabolic
0.7474	7.82E-83	9.75E-80	PGS003139	EFO_0004574	total cholesterol measurement	total cholesterol measurement	EFO_0004574	Lipid or lipoprotein measurement	Metabolic	PGS003038	EFO_0004611	low density lipoprotein cholesterol measurement	measurement	EFO_0004611	Cardiovascular measurement	Cardiovascular
0.7474	7.82E-83	9.75E-80	PGS003139	EFO_0004574	total cholesterol measurement	total cholesterol measurement	EFO_0004574	Lipid or lipoprotein measurement	Metabolic	PGS003038	EFO_0004611	low density lipoprotein cholesterol measurement	measurement	EFO_0004611	Lipid or lipoprotein measurement	Metabolic
0.7474	7.95E-83	9.91E-80	PGS001878	EFO_0008518	polymyalgia rheumatica	polymyalgia rheumatica	EFO_0008518	Other disease	Immunological	PGS002088	EFO_0000685	rheumatoid arthritis	rheumatoid arthritis	EFO_0000685	Immune system disorder	Immunological
0.7473	8.40E-83	1.05E-79	PGS001675	EFO_0006930	brain volume measurement|neuroimaging measurement	brain volume measurement	EFO_0006930	Other measurement	Neurological	PGS001676	EFO_0006930	measurement	brain volume measurement	EFO_0006930	Other measurement	Neurological
0.7472	9.12E-83	1.14E-79	PGS003036	EFO_0004611	low density lipoprotein cholesterol measurement	low density lipoprotein cholesterol measurement	EFO_0004611	Cardiovascular measurement	Cardiovascular	PGS002556	EFO_0004611	low density lipoprotein cholesterol measurement	measurement	EFO_0004611	Cardiovascular measurement	Cardiovascular
0.7472	9.12E-83	1.14E-79	PGS003036	EFO_0004611	low density lipoprotein cholesterol measurement	low density lipoprotein cholesterol measurement	EFO_0004611	Lipid or lipoprotein measurement	Metabolic	PGS002556	EFO_0004611	low density lipoprotein cholesterol measurement	measurement	EFO_0004611	Lipid or lipoprotein measurement	Metabolic
0.7472	9.12E-83	1.14E-79	PGS003036	EFO_0004611	low density lipoprotein cholesterol measurement	low density lipoprotein cholesterol measurement	EFO_0004611	Cardiovascular measurement	Cardiovascular	PGS002556	EFO_0004611	low density lipoprotein cholesterol measurement	measurement	EFO_0004611	Lipid or lipoprotein measurement	Metabolic
0.7472	9.12E-83	1.14E-79	PGS003036	EFO_0004611	low density lipoprotein cholesterol measurement	low density lipoprotein cholesterol measurement	EFO_0004611	Lipid or lipoprotein measurement	Metabolic	PGS002556	EFO_0004611	low density lipoprotein cholesterol measurement	measurement	EFO_0004611	Cardiovascular measurement	Cardiovascular
0.7472	9.18E-83	1.14E-79	PGS002186	EFO_0005091	monocyte count	monocyte count	EFO_0005091	Hematological measurement	Hematological	PGS001163	EFO_0005091	monocyte count	monocyte count	EFO_0005091	Hematological measurement	Hematological
0.7472	9.26E-83	1.15E-79	PGS001738	EFO_0006930	brain volume measurement|neuroimaging measurement	brain volume measurement	EFO_0006930	Other measurement	Neurological	PGS001722	EFO_0006930	measurement	brain volume measurement	EFO_0006930	Other measurement	Neurological
0.7472	9.34E-83	1.16E-79	PGS001662	EFO_0006930	brain volume measurement|neuroimaging measurement	brain volume measurement	EFO_0006930	Other measurement	Neurological	PGS001681	EFO_0006930	measurement	brain volume measurement	EFO_0006930	Other measurement	Neurological
0.7472	9.38E-83	1.17E-79	PGS002352	EFO_0004574	total cholesterol measurement	total cholesterol measurement	EFO_0004574	Lipid or lipoprotein measurement	Metabolic	PGS002654	EFO_0004611	low density lipoprotein cholesterol measurement	measurement	EFO_0004611	Cardiovascular measurement	Cardiovascular
0.7472	9.38E-83	1.17E-79	PGS002352	EFO_0004574	total cholesterol measurement	total cholesterol measurement	EFO_0004574	Lipid or lipoprotein measurement	Metabolic	PGS002654	EFO_0004611	low density lipoprotein cholesterol measurement	measurement	EFO_0004611	Lipid or lipoprotein measurement	Metabolic
0.7472	9.43E-83	1.17E-79	PGS002550	EFO_0004541	HbA1c measurement	HbA1c measurement	EFO_0004541	Hematological measurement	Hematological	PGS002622	MONDO_0005148	type 2 diabetes mellitus	type 2 diabetes mellitus	MONDO_0005148	Metabolic disorder	Metabolic
0.7472	9.43E-83	1.17E-79	PGS002550	EFO_0004541	HbA1c measurement	HbA1c measurement	EFO_0004541	Hematological measurement	Hematological	PGS002622	MONDO_0005148	type 2 diabetes mellitus	type 2 diabetes mellitus	MONDO_0005148	Digestive system disorder	Gastrointestinal
0.7472	9.52E-83	1.18E-79	PGS002964	EFO_0004339	body height	body height	EFO_0004339	Body measurement	Body measurement	PGS000758	EFO_0004339	body height	body height	EFO_0004339	Body measurement	Body measurement
0.7471	9.58E-83	1.19E-79	PGS003201	EFO_0004631	vitamin D measurement	vitamin D measurement	EFO_0004631	Lipid or lipoprotein measurement	Metabolic	PGS003190	EFO_0004631	vitamin D measurement	vitamin D measurement	EFO_0004631	Lipid or lipoprotein measurement	Metabolic
0.7471	9.93E-83	1.24E-79	PGS001738	EFO_0006930	brain volume measurement|neuroimaging measurement	brain volume measurement	EFO_0006930	Other measurement	Neurological	PGS001752	EFO_0006930	measurement	brain volume measurement	EFO_0006930	Other measurement	Neurological
0.7471	1.01E-82	1.26E-79	PGS003139	EFO_0004574	total cholesterol measurement	total cholesterol measurement	EFO_0004574	Lipid or lipoprotein measurement	Metabolic	PGS003033	EFO_0004611	low density lipoprotein cholesterol measurement	measurement	EFO_0004611	Lipid or lipoprotein measurement	Metabolic
0.7471	1.01E-82	1.26E-79	PGS003139	EFO_0004574	total cholesterol measurement	total cholesterol measurement	EFO_0004574	Lipid or lipoprotein measurement	Metabolic	PGS003033	EFO_0004611	low density lipoprotein cholesterol measurement	measurement	EFO_0004611	Cardiovascular measurement	Cardiovascular
0.747	1.04E-82	1.29E-79	PGS003134	EFO_0004574	total cholesterol measurement	total cholesterol measurement	EFO_0004574	Lipid or lipoprotein measurement	Metabolic	PGS003038	EFO_0004611	low density lipoprotein cholesterol measurement	measurement	EFO_0004611	Cardiovascular measurement	Cardiovascular
0.747	1.04E-82	1.29E-79	PGS003134	EFO_0004574	total cholesterol measurement	total cholesterol measurement	EFO_0004574	Lipid or lipoprotein measurement	Metabolic	PGS003038	EFO_0004611	low density lipoprotein cholesterol measurement	measurement	EFO_0004611	Lipid or lipoprotein measurement	Metabolic
0.747	1.08E-82	1.34E-79	PGS000001	EFO_0000305	breast carcinoma	breast carcinoma	EFO_0000305	Cancer	Neoplasms	PGS000052	EFO_0000305	breast carcinoma	breast carcinoma	EFO_0000305	Cancer	Neoplasms
0.747	1.08E-82	1.34E-79	PGS002596	EFO_0004468	glucose measurement	glucose measurement	EFO_0004468	Other measurement	Metabolic	PGS002491	EFO_0000400	diabetes mellitus	diabetes mellitus	EFO_0000400	Digestive system disorder	Gastrointestinal
0.747	1.08E-82	1.34E-79	PGS002596	EFO_0004468	glucose measurement	glucose measurement	EFO_0004468	Other measurement	Metabolic	PGS002491	EFO_0000400	diabetes mellitus	diabetes mellitus	EFO_0000400	Metabolic disorder	Metabolic
0.7469	1.11E-82	1.38E-79	PGS001926	EFO_0004509	hemoglobin measurement	hemoglobin measurement	EFO_0004509	Hematological measurement	Hematological	PGS000092	EFO_0004348	hematocrit	hematocrit	EFO_0004348	Hematological measurement	Hematological
0.7469	1.12E-82	1.39E-79	PGS001805	EFO_0001663	prostate carcinoma	prostate carcinoma	EFO_0001663	Cancer	Neoplasms	PGS000742	EFO_0001663	prostate carcinoma	prostate carcinoma	EFO_0001663	Cancer	Neoplasms
0.7469	1.14E-82	1.42E-79	PGS000532	EFO_0000305	breast carcinoma	breast carcinoma	EFO_0000305	Cancer	Neoplasms	PGS000529	EFO_0000305	breast carcinoma	breast carcinoma	EFO_0000305	Cancer	Neoplasms
0.7469	1.15E-82	1.43E-79	PGS000684	EFO_0004468	glucose measurement	glucose measurement	EFO_0004468	Other measurement	Metabolic	PGS002328	EFO_0004468	glucose measurement	glucose measurement	EFO_0004468	Other measurement	Metabolic
0.7469	1.15E-82	1.43E-79	PGS000114	EFO_0002609	juvenile idiopathic arthritis	juvenile idiopathic arthritis	EFO_0002609	Immune system disorder	Immunological	PGS000326	EFO_1002020	rheumatoid factor negative	rheumatoid factor negative	EFO_1002020	Immune system disorder	Immunological
0.7469	1.18E-82	1.47E-79	PGS001662	EFO_0006930	brain volume measurement|neuroimaging measurement	brain volume measurement	EFO_0006930	Other measurement	Neurological	PGS001672	EFO_0006930	measurement	brain volume measurement	EFO_0006930	Other measurement	Neurological
0.7468	1.21E-82	1.50E-79	PGS003134	EFO_0004574	total cholesterol measurement	total cholesterol measurement	EFO_0004574	Lipid or lipoprotein measurement	Metabolic	PGS003033	EFO_0004611	low density lipoprotein cholesterol measurement	measurement	EFO_0004611	Lipid or lipoprotein measurement	Metabolic
0.7468	1.21E-82	1.50E-79	PGS003134	EFO_0004574	total cholesterol measurement	total cholesterol measurement	EFO_0004574	Lipid or lipoprotein measurement	Metabolic	PGS003033	EFO_0004611	low density lipoprotein cholesterol measurement	measurement	EFO_0004611	Cardiovascular measurement	Cardiovascular
0.7468	1.23E-82	1.52E-79	PGS001844	EFO_1001395	Phlebitis|Thrombophlebitis	Phlebitis	EFO_1001395	Cardiovascular disease	Cardiovascular	PGS001279	EFO_0003827	pulmonary embolism|deep vein thrombosis	pulmonary embolism	EFO_0003827	Cardiovascular disease	Cardiovascular
0.7468	1.28E-82	1.59E-79	PGS000508	EFO_0000305	breast carcinoma	breast carcinoma	EFO_0000305	Cancer	Neoplasms	PGS000503	EFO_0000305	breast carcinoma	breast carcinoma	EFO_0000305	Cancer	Neoplasms
0.7467	1.31E-82	1.62E-79	PGS002150	EFO_0004611	low density lipoprotein cholesterol measurement	low density lipoprotein cholesterol measurement	EFO_0004611	Cardiovascular measurement	Cardiovascular	PGS003033	EFO_0004611	low density lipoprotein cholesterol measurement	measurement	EFO_0004611	Lipid or lipoprotein measurement	Metabolic
0.7467	1.31E-82	1.62E-79	PGS002150	EFO_0004611	low density lipoprotein cholesterol measurement	low density lipoprotein cholesterol measurement	EFO_0004611	Lipid or lipoprotein measurement	Metabolic	PGS003033	EFO_0004611	low density lipoprotein cholesterol measurement	measurement	EFO_0004611	Lipid or lipoprotein measurement	Metabolic
0.7467	1.31E-82	1.62E-79	PGS002150	EFO_0004611	low density lipoprotein cholesterol measurement	low density lipoprotein cholesterol measurement	EFO_0004611	Lipid or lipoprotein measurement	Metabolic	PGS003033	EFO_0004611	low density lipoprotein cholesterol measurement	measurement	EFO_0004611	Cardiovascular measurement	Cardiovascular
0.7467	1.31E-82	1.62E-79	PGS002150	EFO_0004611	low density lipoprotein cholesterol measurement	low density lipoprotein cholesterol measurement	EFO_0004611	Cardiovascular measurement	Cardiovascular	PGS003033	EFO_0004611	low density lipoprotein cholesterol measurement	measurement	EFO_0004611	Cardiovascular measurement	Cardiovascular
0.7467	1.32E-82	1.64E-79	PGS003184	EFO_0004631	vitamin D measurement	vitamin D measurement	EFO_0004631	Lipid or lipoprotein measurement	Metabolic	PGS003190	EFO_0004631	vitamin D measurement	vitamin D measurement	EFO_0004631	Lipid or lipoprotein measurement	Metabolic
0.7467	1.34E-82	1.66E-79	PGS000616	MONDO_0004986	urinary bladder carcinoma	urinary bladder carcinoma	MONDO_0004986	Cancer	Neoplasms	PGS000609	MONDO_0004986	urinary bladder carcinoma	urinary bladder carcinoma	MONDO_0004986	Cancer	Neoplasms

0.7467	1.37E-82	1.70E-79	PGS001945	EFO_0004518	creatinine measurement	creatinine measurement	EFO_0004518	Other measurement	Metabolic	PGS000678	EFO_0004518	creatinine measurement	creatinine measurement	EFO_0004518	Other measurement	Metabolic
0.7467	1.39E-82	1.71E-79	PGS001478	EFO_0006930	brain volume measurement|neuroimaging measurement	brain volume measurement	EFO_0006930	Other measurement	Neurological	PGS001683	EFO_0006930	measurement	brain volume measurement	EFO_0006930	Other measurement	Neurological
0.7467	1.39E-82	1.72E-79	PGS000525	EFO_0000305	breast carcinoma	breast carcinoma	EFO_0000305	Cancer	Neoplasms	PGS000474	EFO_0000305	breast carcinoma	breast carcinoma	EFO_0000305	Cancer	Neoplasms
0.7467	1.39E-82	1.72E-79	PGS000474	EFO_0000305	breast carcinoma	breast carcinoma	EFO_0000305	Cancer	Neoplasms	PGS000526	EFO_0000305	breast carcinoma	breast carcinoma	EFO_0000305	Cancer	Neoplasms
0.7466	1.43E-82	1.77E-79	PGS001844	EFO_1001395	Phlebitis|Thrombophlebitis	Phlebitis	EFO_1001395	Cardiovascular disease	Cardiovascular	PGS001277	EFO_0003827	pulmonary embolism|deep vein thrombosis	pulmonary embolism	EFO_0003827	Cardiovascular disease	Cardiovascular
0.7466	1.46E-82	1.81E-79	PGS001489	EFO_0006930	brain volume measurement|neuroimaging measurement	brain volume measurement	EFO_0006930	Other measurement	Neurological	PGS001673	EFO_0006930	measurement	brain volume measurement	EFO_0006930	Other measurement	Neurological
0.7466	1.48E-82	1.83E-79	PGS000533	EFO_0000305	breast carcinoma	breast carcinoma	EFO_0000305	Cancer	Neoplasms	PGS000002	EFO_1000649	estrogen-receptor positive breast cancer	cancer	EFO_1000649	Cancer	Neoplasms
0.7466	1.48E-82	1.83E-79	PGS000534	EFO_0000305	breast carcinoma	breast carcinoma	EFO_0000305	Cancer	Neoplasms	PGS000002	EFO_1000649	estrogen-receptor positive breast cancer	cancer	EFO_1000649	Cancer	Neoplasms
0.7465	1.52E-82	1.88E-79	PGS000578	EFO_0001663	prostate carcinoma	prostate carcinoma	EFO_0001663	Cancer	Neoplasms	PGS000044	EFO_0001663	prostate carcinoma	prostate carcinoma	EFO_0001663	Cancer	Neoplasms
0.7465	1.52E-82	1.88E-79	PGS000577	EFO_0001663	prostate carcinoma	prostate carcinoma	EFO_0001663	Cancer	Neoplasms	PGS000044	EFO_0001663	prostate carcinoma	prostate carcinoma	EFO_0001663	Cancer	Neoplasms
0.7465	1.55E-82	1.92E-79	PGS000065	EFO_0004611	low density lipoprotein cholesterol measurement	low density lipoprotein cholesterol measurement	EFO_0004611	Cardiovascular measurement	Cardiovascular	PGS002337	EFO_0004611	low density lipoprotein cholesterol measurement	measurement	EFO_0004611	Lipid or lipoprotein measurement	Metabolic
0.7465	1.55E-82	1.92E-79	PGS000065	EFO_0004611	low density lipoprotein cholesterol measurement	low density lipoprotein cholesterol measurement	EFO_0004611	Lipid or lipoprotein measurement	Metabolic	PGS002337	EFO_0004611	low density lipoprotein cholesterol measurement	measurement	EFO_0004611	Cardiovascular measurement	Cardiovascular
0.7465	1.55E-82	1.92E-79	PGS000065	EFO_0004611	low density lipoprotein cholesterol measurement	low density lipoprotein cholesterol measurement	EFO_0004611	Lipid or lipoprotein measurement	Metabolic	PGS002337	EFO_0004611	low density lipoprotein cholesterol measurement	measurement	EFO_0004611	Lipid or lipoprotein measurement	Metabolic
0.7465	1.55E-82	1.92E-79	PGS000065	EFO_0004611	low density lipoprotein cholesterol measurement	low density lipoprotein cholesterol measurement	EFO_0004611	Cardiovascular measurement	Cardiovascular	PGS002337	EFO_0004611	low density lipoprotein cholesterol measurement	measurement	EFO_0004611	Cardiovascular measurement	Cardiovascular
0.7465	1.56E-82	1.92E-79	PGS003199	EFO_0004631	vitamin D measurement	vitamin D measurement	EFO_0004631	Lipid or lipoprotein measurement	Metabolic	PGS003190	EFO_0004631	vitamin D measurement	vitamin D measurement	EFO_0004631	Lipid or lipoprotein measurement	Metabolic
0.7465	1.62E-82	2.00E-79	PGS000051	EFO_0000305	breast carcinoma	breast carcinoma	EFO_0000305	Cancer	Neoplasms	PGS000773	EFO_0000305	breast carcinoma	breast carcinoma	EFO_0000305	Cancer	Neoplasms
0.7464	1.67E-82	2.06E-79	PGS002171	EFO_0004541	HbA1c measurement	HbA1c measurement	EFO_0004541	Hematological measurement	Hematological	PGS002648	EFO_0004541	HbA1c measurement	HbA1c measurement	EFO_0004541	Hematological measurement	Hematological
0.7464	1.68E-82	2.07E-79	PGS001750	EFO_0006930	brain volume measurement|neuroimaging measurement	brain volume measurement	EFO_0006930	Other measurement	Neurological	PGS001751	EFO_0006930	measurement	brain volume measurement	EFO_0006930	Other measurement	Neurological
0.7464	1.74E-82	2.15E-79	PGS002241	EFO_0001663	prostate carcinoma	prostate carcinoma	EFO_0001663	Cancer	Neoplasms	PGS000588	EFO_0001663	prostate carcinoma	prostate carcinoma	EFO_0001663	Cancer	Neoplasms
0.7463	1.81E-82	2.23E-79	PGS002169	EFO_0005409	fat body mass	fat body mass	EFO_0005409	Other measurement	Body measurement	PGS002849	EFO_0004340	body mass index	body mass index	EFO_0004340	Body measurement	Body measurement
0.7463	1.84E-82	2.27E-79	PGS003199	EFO_0004631	vitamin D measurement	vitamin D measurement	EFO_0004631	Lipid or lipoprotein measurement	Metabolic	PGS002427	EFO_0004631	vitamin D measurement	vitamin D measurement	EFO_0004631	Lipid or lipoprotein measurement	Metabolic
0.7462	1.91E-82	2.36E-79	PGS002854	EFO_0004340	body mass index	body mass index	EFO_0004340	Body measurement	Body measurement	PGS002133	EFO_0007800	body fat percentage	body fat percentage	EFO_0007800	Body measurement	Body measurement
0.7462	2.00E-82	2.47E-79	PGS001163	EFO_0005091	monocyte count	monocyte count	EFO_0005091	Hematological measurement	Hematological	PGS001078	EFO_0007989	monocyte percentage of leukocytes	monocyte percentage of leukocytes	EFO_0007989	Hematological measurement	Hematological
0.7462	2.03E-82	2.50E-79	PGS002286	EFO_0004574	total cholesterol measurement	total cholesterol measurement	EFO_0004574	Lipid or lipoprotein measurement	Metabolic	PGS002284	EFO_0004612	high density lipoprotein cholesterol measurement	measurement	EFO_0004612	Lipid or lipoprotein measurement	Metabolic
0.7462	2.04E-82	2.51E-79	PGS002550	EFO_0004541	HbA1c measurement	HbA1c measurement	EFO_0004541	Hematological measurement	Hematological	PGS002445	EFO_0001379	endocrine system disease|diabetes mellitus	endocrine system disease	EFO_0001379	Other disease	Metabolic
-0.7462	2.04E-82	2.51E-79	PGS001535	EFO_0010729	sun exposure measurement	sun exposure measurement	EFO_0010729	Other measurement	Activities	PGS001257	EFO_0003958	sunburn	sunburn	EFO_0003958	Other disease	Dermatological
0.7462	2.04E-82	2.52E-79	PGS003152	EFO_0004530	triglyceride measurement	triglyceride measurement	EFO_0004530	Lipid or lipoprotein measurement	Metabolic	PGS003149	EFO_0004530	triglyceride measurement	triglyceride measurement	EFO_0004530	Lipid or lipoprotein measurement	Metabolic
0.7462	2.05E-82	2.52E-79	PGS002993	EFO_0004339	body height	body height	EFO_0004339	Body measurement	Body measurement	PGS002146	EFO_0004339	body height	body height	EFO_0004339	Body measurement	Body measurement
0.7461	2.16E-82	2.65E-79	PGS000685	EFO_0004541	HbA1c measurement	HbA1c measurement	EFO_0004541	Hematological measurement	Hematological	PGS000712	MONDO_0005148	type 2 diabetes mellitus	type 2 diabetes mellitus	MONDO_0005148	Metabolic disorder	Metabolic
0.7461	2.16E-82	2.65E-79	PGS000685	EFO_0004541	HbA1c measurement	HbA1c measurement	EFO_0004541	Hematological measurement	Hematological	PGS000712	MONDO_0005148	type 2 diabetes mellitus	type 2 diabetes mellitus	MONDO_0005148	Digestive system disorder	Gastrointestinal
0.7461	2.17E-82	2.67E-79	PGS001750	EFO_0006930	brain volume measurement|neuroimaging measurement	brain volume measurement	EFO_0006930	Other measurement	Neurological	PGS001723	EFO_0006930	measurement	brain volume measurement	EFO_0006930	Other measurement	Neurological
0.7461	2.18E-82	2.68E-79	PGS001750	EFO_0006930	brain volume measurement|neuroimaging measurement	brain volume measurement	EFO_0006930	Other measurement	Neurological	PGS001737	EFO_0006930	measurement	brain volume measurement	EFO_0006930	Other measurement	Neurological
0.746	2.26E-82	2.78E-79	PGS001753	EFO_0006930	brain volume measurement|neuroimaging measurement	brain volume measurement	EFO_0006930	Other measurement	Neurological	PGS001737	EFO_0006930	measurement	brain volume measurement	EFO_0006930	Other measurement	Neurological
0.7459	2.45E-82	3.02E-79	PGS000499	EFO_0000305	breast carcinoma	breast carcinoma	EFO_0000305	Cancer	Neoplasms	PGS000007	EFO_0000305	breast carcinoma	breast carcinoma	EFO_0000305	Cancer	Neoplasms
0.7459	2.49E-82	3.05E-79	PGS001814	MONDO_0000334	multinodular goiter|nontoxic goiter	multinodular goiter	MONDO_0000334	Other disease	Endocrine	PGS000928	MONDO_0001658	nontoxic goiter	nontoxic goiter	MONDO_0001658	Other disease	Endocrine
0.7459	2.50E-82	3.07E-79	PGS000507	EFO_0000305	breast carcinoma	breast carcinoma	EFO_0000305	Cancer	Neoplasms	PGS000527	EFO_0000305	breast carcinoma	breast carcinoma	EFO_0000305	Cancer	Neoplasms
0.7459	2.57E-82	3.15E-79	PGS002427	EFO_0004631	vitamin D measurement	vitamin D measurement	EFO_0004631	Lipid or lipoprotein measurement	Metabolic	PGS003184	EFO_0004631	vitamin D measurement	vitamin D measurement	EFO_0004631	Lipid or lipoprotein measurement	Metabolic
0.7458	2.60E-82	3.19E-79	PGS001667	EFO_0006930	brain volume measurement|neuroimaging measurement	brain volume measurement	EFO_0006930	Other measurement	Neurological	PGS001668	EFO_0006930	measurement	brain volume measurement	EFO_0006930	Other measurement	Neurological
0.7458	2.64E-82	3.23E-79	PGS003200	EFO_0004631	vitamin D measurement	vitamin D measurement	EFO_0004631	Lipid or lipoprotein measurement	Metabolic	PGS003190	EFO_0004631	vitamin D measurement	vitamin D measurement	EFO_0004631	Lipid or lipoprotein measurement	Metabolic
0.7458	2.64E-82	3.24E-79	PGS001038	EFO_0009270	heel bone mineral density	heel bone mineral density	EFO_0009270	Other measurement	Skeletal	PGS000951	EFO_0004514	bone quantitative ultrasound measurement	measurement	EFO_0004514	Other measurement	Skeletal
0.7458	2.64E-82	3.24E-79	PGS000951	EFO_0004514	bone quantitative ultrasound measurement	bone quantitative ultrasound measurement	EFO_0004514	Other measurement	Skeletal	PGS000954	EFO_0004514	bone quantitative ultrasound measurement	measurement	EFO_0004514	Other measurement	Skeletal
0.7458	2.70E-82	3.32E-79	PGS002015	EFO_0000305	breast carcinoma	breast carcinoma	EFO_0000305	Cancer	Neoplasms	PGS000516	EFO_0000305	breast carcinoma	breast carcinoma	EFO_0000305	Cancer	Neoplasms
0.7457	2.85E-82	3.49E-79	PGS000346	EFO_1000650	estrogen-receptor negative breast cancer	estrogen-receptor negative breast cancer	EFO_1000650	Cancer	Neoplasms	PGS000345	EFO_0000305	breast carcinoma	breast carcinoma	EFO_0000305	Cancer	Neoplasms
0.7457	2.95E-82	3.61E-79	PGS002501	EFO_0004541	HbA1c measurement	HbA1c measurement	EFO_0004541	Hematological measurement	Hematological	PGS002599	EFO_0004541	HbA1c measurement	HbA1c measurement	EFO_0004541	Hematological measurement	Hematological
0.7456	3.17E-82	3.88E-79	PGS002782	EFO_0005689	non-high density lipoprotein cholesterol measurement	non-high density lipoprotein cholesterol measurement	EFO_0005689	Lipid or lipoprotein measurement	Metabolic	PGS003138	EFO_0004574	total cholesterol measurement	total cholesterol measurement	EFO_0004574	Lipid or lipoprotein measurement	Metabolic
0.7456	3.17E-82	3.88E-79	PGS002782	EFO_0005689	non-high density lipoprotein cholesterol measurement	non-high density lipoprotein cholesterol measurement	EFO_0005689	Cardiovascular measurement	Cardiovascular	PGS003138	EFO_0004574	total cholesterol measurement	total cholesterol measurement	EFO_0004574	Lipid or lipoprotein measurement	Metabolic
0.7456	3.22E-82	3.94E-79	PGS000889	EFO_0004611	low density lipoprotein cholesterol measurement	low density lipoprotein cholesterol measurement	EFO_0004611	Lipid or lipoprotein measurement	Metabolic	PGS002654	EFO_0004611	low density lipoprotein cholesterol measurement	measurement	EFO_0004611	Cardiovascular measurement	Cardiovascular
0.7456	3.22E-82	3.94E-79	PGS000889	EFO_0004611	low density lipoprotein cholesterol measurement	low density lipoprotein cholesterol measurement	EFO_0004611	Lipid or lipoprotein measurement	Metabolic	PGS002654	EFO_0004611	low density lipoprotein cholesterol measurement	measurement	EFO_0004611	Lipid or lipoprotein measurement	Metabolic
0.7456	3.22E-82	3.94E-79	PGS000889	EFO_0004611	low density lipoprotein cholesterol measurement	low density lipoprotein cholesterol measurement	EFO_0004611	Cardiovascular measurement	Cardiovascular	PGS002654	EFO_0004611	low density lipoprotein cholesterol measurement	measurement	EFO_0004611	Cardiovascular measurement	Cardiovascular
0.7456	3.22E-82	3.94E-79	PGS000889	EFO_0004611	low density lipoprotein cholesterol measurement	low density lipoprotein cholesterol measurement	EFO_0004611	Cardiovascular measurement	Cardiovascular	PGS002654	EFO_0004611	low density lipoprotein cholesterol measurement	measurement	EFO_0004611	Lipid or lipoprotein measurement	Metabolic
0.7455	3.29E-82	4.03E-79	PGS002781	EFO_0004612	high density lipoprotein cholesterol measurement	high density lipoprotein cholesterol measurement	EFO_0004612	Lipid or lipoprotein measurement	Metabolic	PGS002172	EFO_0004612	high density lipoprotein cholesterol measurement	measurement	EFO_0004612	Lipid or lipoprotein measurement	Metabolic
0.7455	3.32E-82	4.07E-79	PGS001984	EFO_0009805	whole body water mass	whole body water mass	EFO_0009805	Other measurement	Body measurement	PGS001166	EFO_0004995	lean body mass	lean body mass	EFO_0004995	Other measurement	Skeletal
0.7455	3.40E-82	4.16E-79	PGS000675	EFO_0004458	C-reactive protein measurement	C-reactive protein measurement	EFO_0004458	Inflammatory measurement	Immunological	PGS002873	EFO_0004458	C-reactive protein measurement	C-reactive protein measurement	EFO_0004458	Inflammatory measurement	Immunological
0.7453	3.98E-82	4.87E-79	PGS002091	EFO_0008518	polymyalgia rheumatica	polymyalgia rheumatica	EFO_0008518	Other disease	Immunological	PGS001875	EFO_0000685	rheumatoid arthritis	rheumatoid arthritis	EFO_0000685	Immune system disorder	Immunological
0.7452	4.11E-82	5.03E-79	PGS003185	EFO_0004631	vitamin D measurement	vitamin D measurement	EFO_0004631	Lipid or lipoprotein measurement	Metabolic	PGS003196	EFO_0004631	vitamin D measurement	vitamin D measurement	EFO_0004631	Lipid or lipoprotein measurement	Metabolic
0.7452	4.13E-82	5.05E-79	PGS001680	EFO_0006930	brain volume measurement|neuroimaging measurement	brain volume measurement	EFO_0006930	Other measurement	Neurological	PGS001669	EFO_0006930	measurement	brain volume measurement	EFO_0006930	Other measurement	Neurological
0.7452	4.19E-82	5.12E-79	PGS002765	EFO_0000537	hypertension	hypertension	EFO_0000537	Cardiovascular disease	Cardiovascular	PGS002228	EFO_0006335	systolic blood pressure	systolic blood pressure	EFO_0006335	Other measurement	Cardiovascular
0.7451	4.73E-82	5.79E-79	PGS003254	EFO_0010472	creatine measurement|glomerular filtration rate	creatine measurement	EFO_0010472	Other measurement	Metabolic	PGS003255	EFO_0010472	creatine measurement|glomerular filtration rate	creatine measurement	EFO_0010472	Other measurement	Metabolic
-0.745	4.83E-82	5.91E-79	PGS003243	EFO_0010472	creatine measurement	creatine measurement	EFO_0010472	Other measurement	Metabolic	PGS002757	EFO_0003884	chronic kidney disease	chronic kidney disease	EFO_0003884	Other disease	Metabolic
0.745	4.99E-82	6.10E-79	PGS000591	EFO_0001663	prostate carcinoma	prostate carcinoma	EFO_0001663	Cancer	Neoplasms	PGS001805	EFO_0001663	prostate carcinoma	prostate carcinoma	EFO_0001663	Cancer	Neoplasms
0.7449	5.20E-82	6.35E-79	PGS000847	EFO_0004530	triglyceride measurement	triglyceride measurement	EFO_0004530	Lipid or lipoprotein measurement	Metabolic	PGS000312	EFO_0004530	triglyceride measurement	triglyceride measurement	EFO_0004530	Lipid or lipoprotein measurement	Metabolic
0.7449	5.39E-82	6.58E-79	PGS003399	EFO_0000305	breast carcinoma	breast carcinoma	EFO_0000305	Cancer	Neoplasms	PGS000004	EFO_0000305	breast carcinoma	breast carcinoma	EFO_0000305	Cancer	Neoplasms
0.7449	5.43E-82	6.64E-79	PGS003035	EFO_0004611	low density lipoprotein cholesterol measurement	low density lipoprotein cholesterol measurement	EFO_0004611	Lipid or lipoprotein measurement	Metabolic	PGS000889	EFO_0004611	low density lipoprotein cholesterol measurement	measurement	EFO_0004611	Lipid or lipoprotein measurement	Metabolic
0.7449	5.43E-82	6.64E-79	PGS003035	EFO_0004611	low density lipoprotein cholesterol measurement	low density lipoprotein cholesterol measurement	EFO_0004611	Lipid or lipoprotein measurement	Metabolic	PGS000889	EFO_0004611	low density lipoprotein cholesterol measurement	measurement	EFO_0004611	Cardiovascular measurement	Cardiovascular
0.7449	5.43E-82	6.64E-79	PGS003035	EFO_0004611	low density lipoprotein cholesterol measurement	low density lipoprotein cholesterol measurement	EFO_0004611	Cardiovascular measurement	Cardiovascular	PGS000889	EFO_0004611	low density lipoprotein cholesterol measurement	measurement	EFO_0004611	Lipid or lipoprotein measurement	Metabolic
0.7449	5.43E-82	6.64E-79	PGS003035	EFO_0004611	low density lipoprotein cholesterol measurement	low density lipoprotein cholesterol measurement	EFO_0004611	Cardiovascular measurement	Cardiovascular	PGS000889	EFO_0004611	low density lipoprotein cholesterol measurement	measurement	EFO_0004611	Cardiovascular measurement	Cardiovascular
0.7449	5.47E-82	6.68E-79	PGS001950	EFO_0004995	lean body mass	lean body mass	EFO_0004995	Other measurement	Skeletal	PGS001166	EFO_0004995	lean body mass	lean body mass	EFO_0004995	Other measurement	Skeletal
0.7449	5.50E-82	6.71E-79	PGS001682	EFO_0006930	brain volume measurement|neuroimaging measurement	brain volume measurement	EFO_0006930	Other measurement	Neurological	PGS001484	EFO_0006930	measurement	brain volume measurement	EFO_0006930	Other measurement	Neurological
0.7448	5.75E-82	7.02E-79	PGS000930	MONDO_0000831	thrombotic disease	thrombotic disease	MONDO_0000831	Cardiovascular disease	Cardiovascular	PGS001844	EFO_1001395	Phlebitis|Thrombophlebitis	Phlebitis	EFO_1001395	Cardiovascular disease	Cardiovascular
0.7448	5.92E-82	7.22E-79	PGS003198	EFO_0004631	vitamin D measurement	vitamin D measurement	EFO_0004631	Lipid or lipoprotein measurement	Metabolic	PGS002427	EFO_0004631	vitamin D measurement	vitamin D measurement	EFO_0004631	Lipid or lipoprotein measurement	Metabolic
0.7447	6.19E-82	7.54E-79	PGS001752	EFO_0006930	brain volume measurement|neuroimaging measurement	brain volume measurement	EFO_0006930	Other measurement	Neurological	PGS001707	EFO_0006930	measurement	brain volume measurement	EFO_0006930	Other measurement	Neurological
0.7447	6.23E-82	7.60E-79	PGS000046	EFO_1000649	estrogen-receptor positive breast cancer	estrogen-receptor positive breast cancer	EFO_1000649	Cancer	Neoplasms	PGS000488	EFO_0000305	breast carcinoma	breast carcinoma	EFO_0000305	Cancer	Neoplasms
0.7447	6.24E-82	7.61E-79	PGS000332	EFO_0000305	breast carcinoma	breast carcinoma	EFO_0000305	Cancer	Neoplasms	PGS000535	EFO_0000305	breast carcinoma	breast carcinoma	EFO_0000305	Cancer	Neoplasms
0.7447	6.35E-82	7.73E-79	PGS003288	EFO_0004617	cystatin C measurement	cystatin C measurement	EFO_0004617	Other measurement	Metabolic	PGS003279	EFO_0004617	cystatin C measurement	cystatin C measurement	EFO_0004617	Other measurement	Metabolic
0.7446	6.65E-82	8.10E-79	PGS003296	EFO_0004617	cystatin C measurement|glomerular filtration rate	cystatin C measurement	EFO_0004617	Other measurement	Metabolic	PGS003309	EFO_0004617	cystatin C measurement|glomerular filtration rate	cystatin C measurement	EFO_0004617	Other measurement	Metabolic
0.7445	7.01E-82	8.54E-79	PGS001488	EFO_0006930	brain volume measurement|neuroimaging measurement	brain volume measurement	EFO_0006930	Other measurement	Neurological	PGS001479	EFO_0006930	measurement	brain volume measurement	EFO_0006930	Other measurement	Neurological
0.7445	7.16E-82	8.72E-79	PGS000065	EFO_0004611	low density lipoprotein cholesterol measurement	low density lipoprotein cholesterol measurement	EFO_0004611	Cardiovascular measurement	Cardiovascular	PGS002102	EFO_0004615	apolipoprotein B measurement	apolipoprotein B measurement	EFO_0004615	Lipid or lipoprotein measurement	Metabolic
0.7445	7.16E-82	8.72E-79	PGS000065	EFO_0004611	low density lipoprotein cholesterol measurement	low density lipoprotein cholesterol measurement	EFO_0004611	Lipid or lipoprotein measurement	Metabolic	PGS002102	EFO_0004615	apolipoprotein B measurement	apolipoprotein B measurement	EFO_0004615	Lipid or lipoprotein measurement	Metabolic
0.7445	7.25E-82	8.83E-79	PGS002651	HP_0003124	Hypercholesterolemia	Hypercholesterolemia	HP_0003124	Other trait	Metabolic	PGS002334	HP_0003124	Hypercholesterolemia	Hypercholesterolemia	HP_0003124	Other trait	Metabolic
0.7445	7.37E-82	8.97E-79	PGS002201	EFO_0004342	waist circumference	waist circumference	EFO_0004342	Body measurement	Body measurement	PGS002859	EFO_0004340	body mass index	body mass index	EFO_0004340	Body measurement	Body measurement
0.7445	7.42E-82	9.03E-79	PGS000930	MONDO_0000831	thrombotic disease	thrombotic disease	MONDO_0000831	Cardiovascular disease	Cardiovascular	PGS000961	EFO_1001395	Phlebitis|Thrombophlebitis	Phlebitis	EFO_1001395	Cardiovascular disease	Cardiovascular
0.7444	7.77E-82	9.44E-79	PGS000124	EFO_0004695	intraocular pressure measurement	intraocular pressure measurement	EFO_0004695	Other measurement	Ophthalmological	PGS000879	EFO_0004695	intraocular pressure measurement	intraocular pressure measurement	EFO_0004695	Other measurement	Ophthalmological
0.7444	7.80E-82	9.49E-79	PGS002550	EFO_0004541	HbA1c measurement	HbA1c measurement	EFO_0004541	Hematological measurement	Hematological	PGS002442	EFO_0000400	diabetes mellitus	diabetes mellitus	EFO_0000400	Digestive system disorder	Gastrointestinal
0.7444	7.80E-82	9.49E-79	PGS002550	EFO_0004541	HbA1c measurement	HbA1c measurement	EFO_0004541	Hematological measurement	Hematological	PGS002442	EFO_0000400	diabetes mellitus	diabetes mellitus	EFO_0000400	Metabolic disorder	Metabolic
0.7444	7.88E-82	9.57E-79	PGS000018	EFO_0001645	coronary artery disease	coronary artery disease	EFO_0001645	Cardiovascular disease	Cardiovascular	PGS001780	EFO_0001645	coronary artery disease	coronary artery disease	EFO_0001645	Cardiovascular disease	Cardiovascular
0.7444	7.88E-82	9.58E-79	PGS001925	EFO_0004348	hematocrit	hematocrit	EFO_0004348	Hematological measurement	Hematological	PGS001400	EFO_0004509	hemoglobin measurement	hemoglobin measurement	EFO_0004509	Hematological measurement	Hematological
0.7443	8.23E-82	9.99E-79	PGS002965	EFO_0004339	body height	body height	EFO_0004339	Body measurement	Body measurement	PGS002332	EFO_0004339	body height	body height	EFO_0004339	Body measurement	Body measurement
0.7443	8.42E-82	1.02E-78	PGS000532	EFO_0000305	breast carcinoma	breast carcinoma	EFO_0000305	Cancer	Neoplasms	PGS000516	EFO_0000305	breast carcinoma	breast carcinoma	EFO_0000305	Cancer	Neoplasms
0.7443	8.49E-82	1.03E-78	PGS001158	EFO_0005106	body composition measurement	body composition measurement	EFO_0005106	Body measurement	Body measurement	PGS001984	EFO_0009805	whole body water mass	whole body water mass	EFO_0009805	Other measurement	Body measurement
0.7442	8.92E-82	1.08E-78	PGS003150	EFO_0004530	triglyceride measurement	triglyceride measurement	EFO_0004530	Lipid or lipoprotein measurement	Metabolic	PGS003148	EFO_0004530	triglyceride measurement	triglyceride measurement	EFO_0004530	Lipid or lipoprotein measurement	Metabolic
0.7441	9.69E-82	1.18E-78	PGS000002	EFO_1000649	estrogen-receptor positive breast cancer	estrogen-receptor positive breast cancer	EFO_1000649	Cancer	Neoplasms	PGS000526	EFO_0000305	breast carcinoma	breast carcinoma	EFO_0000305	Cancer	Neoplasms
0.7441	9.69E-82	1.18E-78	PGS000525	EFO_0000305	breast carcinoma	breast carcinoma	EFO_0000305	Cancer	Neoplasms	PGS000002	EFO_1000649	estrogen-receptor positive breast cancer	cancer	EFO_1000649	Cancer	Neoplasms
0.7441	9.72E-82	1.18E-78	PGS000467	EFO_0000707	squamous cell carcinoma	squamous cell carcinoma	EFO_0000707	Cancer	Neoplasms	PGS000454	EFO_0004193	basal cell carcinoma	basal cell carcinoma	EFO_0004193	Cancer	Neoplasms
0.7441	9.89E-82	1.20E-78	PGS000539	EFO_0000305	breast carcinoma	breast carcinoma	EFO_0000305	Cancer	Neoplasms	PGS000535	EFO_0000305	breast carcinoma	breast carcinoma	EFO_0000305	Cancer	Neoplasms
0.7441	9.90E-82	1.20E-78	PGS000478	EFO_0000305	breast carcinoma	breast carcinoma	EFO_0000305	Cancer	Neoplasms	PGS000472	EFO_0000305	breast carcinoma	breast carcinoma	EFO_0000305	Cancer	Neoplasms
0.744	1.06E-81	1.28E-78	PGS000774	EFO_1000649	estrogen-receptor positive breast cancer	estrogen-receptor positive breast cancer	EFO_1000649	Cancer	Neoplasms	PGS000505	EFO_0000305	breast carcinoma	breast carcinoma	EFO_0000305	Cancer	Neoplasms
0.744	1.06E-81	1.28E-78	PGS000774	EFO_1000649	estrogen-receptor positive breast cancer	estrogen-receptor positive breast cancer	EFO_1000649	Cancer	Neoplasms	PGS000506	EFO_0000305	breast carcinoma	breast carcinoma	EFO_0000305	Cancer	Neoplasms
0.744	1.07E-81	1.30E-78	PGS001984	EFO_0009805	whole body water mass	whole body water mass	EFO_0009805	Other measurement	Body measurement	PGS001235	EFO_0004324	body weights and measures	body weights and measures	EFO_0004324	Body measurement	Body measurement
0.744	1.09E-81	1.33E-78	PGS001529	EFO_0007772	calcaneal bone quantitative ultrasound measurement	calcaneal bone quantitative ultrasound measurement	EFO_0007772	Other measurement	Skeletal	PGS000954	EFO_0004514	bone quantitative ultrasound measurement	measurement	EFO_0004514	Other measurement	Skeletal
0.7439	1.12E-81	1.36E-78	PGS001529	EFO_0007772	calcaneal bone quantitative ultrasound measurement	calcaneal bone quantitative ultrasound measurement	EFO_0007772	Other measurement	Skeletal	PGS001038	EFO_0009270	heel bone mineral density	heel bone mineral density	EFO_0009270	Other measurement	Skeletal
0.7439	1.13E-81	1.36E-78	PGS002336	EFO_0004705	hypothyroidism	hypothyroidism	EFO_0004705	Other disease	Endocrine	PGS000965	EFO_1001055	myxedema|hypothyroidism	myxedema	EFO_1001055	Other disease	Endocrine
0.7439	1.13E-81	1.37E-78	PGS003150	EFO_0004530	triglyceride measurement	triglyceride measurement	EFO_0004530	Lipid or lipoprotein measurement	Metabolic	PGS003153	EFO_0004530	triglyceride measurement	triglyceride measurement	EFO_0004530	Lipid or lipoprotein measurement	Metabolic
0.7438	1.20E-81	1.46E-78	PGS002400	EFO_0004468	glucose measurement	glucose measurement	EFO_0004468	Other measurement	Metabolic	PGS002599	EFO_0004541	HbA1c measurement	HbA1c measurement	EFO_0004541	Hematological measurement	Hematological

0.7438	1.21E-81	1.47E-78	PGS001661	EFO_0006930	brain volume measurement|neuroimaging measurement	brain volume measurement	EFO_0006930	Other measurement	Neurological	PGS001673	EFO_0006930	measurement	brain volume measurement	EFO_0006930	Other measurement	Neurological
0.7438	1.25E-81	1.51E-78	PGS000515	EFO_0000305	breast carcinoma	breast carcinoma	EFO_0000305	Cancer	Neoplasms	PGS001336	EFO_0000305	breast carcinoma	breast carcinoma	EFO_0000305	Cancer	Neoplasms
0.7438	1.28E-81	1.54E-78	PGS003159	EFO_0004620	vitamin B12 measurement	vitamin B12 measurement	EFO_0004620	Other measurement	Metabolic	PGS003160	EFO_0004620	vitamin B12 measurement	vitamin B12 measurement	EFO_0004620	Other measurement	Metabolic
0.7438	1.28E-81	1.55E-78	PGS001158	EFO_0005106	body composition measurement	body composition measurement	EFO_0005106	Body measurement	Body measurement	PGS001950	EFO_0004995	lean body mass	lean body mass	EFO_0004995	Other measurement	Skeletal
0.7437	1.29E-81	1.56E-78	PGS001984	EFO_0009805	whole body water mass	whole body water mass	EFO_0009805	Other measurement	Body measurement	PGS001234	EFO_0004324	body weights and measures	body weights and measures	EFO_0004324	Body measurement	Body measurement
0.7437	1.33E-81	1.61E-78	PGS000488	EFO_0000305	breast carcinoma	breast carcinoma	EFO_0000305	Cancer	Neoplasms	PGS000045	EFO_0000305	breast carcinoma	breast carcinoma	EFO_0000305	Cancer	Neoplasms
0.7437	1.36E-81	1.64E-78	PGS002983	EFO_0004339	body height	body height	EFO_0004339	Body measurement	Body measurement	PGS002146	EFO_0004339	body height	body height	EFO_0004339	Body measurement	Body measurement
0.7436	1.45E-81	1.75E-78	PGS002970	EFO_0004339	body height	body height	EFO_0004339	Body measurement	Body measurement	PGS000758	EFO_0004339	body height	body height	EFO_0004339	Body measurement	Body measurement
0.7435	1.53E-81	1.85E-78	PGS000774	EFO_1000649	estrogen-receptor positive breast cancer	estrogen-receptor positive breast cancer	EFO_1000649	Cancer	Neoplasms	PGS000045	EFO_0000305	breast carcinoma	breast carcinoma	EFO_0000305	Cancer	Neoplasms
0.7435	1.54E-81	1.86E-78	PGS001682	EFO_0006930	brain volume measurement|neuroimaging measurement	brain volume measurement	EFO_0006930	Other measurement	Neurological	PGS001671	EFO_0006930	measurement	brain volume measurement	EFO_0006930	Other measurement	Neurological
0.7435	1.56E-81	1.88E-78	PGS002335	EFO_0000537	hypertension	hypertension	EFO_0000537	Cardiovascular disease	Cardiovascular	PGS002994	EFO_0000537	hypertension	hypertension	EFO_0000537	Cardiovascular disease	Cardiovascular
0.7435	1.58E-81	1.91E-78	PGS000758	EFO_0004339	body height	body height	EFO_0004339	Body measurement	Body measurement	PGS002983	EFO_0004339	body height	body height	EFO_0004339	Body measurement	Body measurement
0.7434	1.62E-81	1.96E-78	PGS001228	EFO_0004340	body mass index	body mass index	EFO_0004340	Body measurement	Body measurement	PGS002859	EFO_0004340	body mass index	body mass index	EFO_0004340	Body measurement	Body measurement
0.7434	1.64E-81	1.98E-78	PGS001554	EFO_0005420	measurement	grey matter volume measurement	EFO_0005420	Other measurement	Neurological	PGS001555	EFO_0005420	measurement	grey matter volume measurement	EFO_0005420	Other measurement	Neurological
0.7434	1.65E-81	1.99E-78	PGS001279	EFO_0003827	pulmonary embolism|deep vein thrombosis	pulmonary embolism	EFO_0003827	Cardiovascular disease	Cardiovascular	PGS000961	EFO_1001395	Phlebitis|Thrombophlebitis	Phlebitis	EFO_1001395	Cardiovascular disease	Cardiovascular
0.7434	1.73E-81	2.08E-78	PGS000065	EFO_0004611	low density lipoprotein cholesterol measurement	low density lipoprotein cholesterol measurement	EFO_0004611	Cardiovascular measurement	Cardiovascular	PGS000897	EFO_0004611	low density lipoprotein cholesterol measurement	measurement	EFO_0004611	Lipid or lipoprotein measurement	Metabolic
0.7434	1.73E-81	2.08E-78	PGS000065	EFO_0004611	low density lipoprotein cholesterol measurement	low density lipoprotein cholesterol measurement	EFO_0004611	Lipid or lipoprotein measurement	Metabolic	PGS000897	EFO_0004611	low density lipoprotein cholesterol measurement	measurement	EFO_0004611	Cardiovascular measurement	Cardiovascular
0.7434	1.73E-81	2.08E-78	PGS000065	EFO_0004611	low density lipoprotein cholesterol measurement	low density lipoprotein cholesterol measurement	EFO_0004611	Lipid or lipoprotein measurement	Metabolic	PGS000897	EFO_0004611	low density lipoprotein cholesterol measurement	measurement	EFO_0004611	Lipid or lipoprotein measurement	Metabolic
0.7434	1.73E-81	2.08E-78	PGS000065	EFO_0004611	low density lipoprotein cholesterol measurement	low density lipoprotein cholesterol measurement	EFO_0004611	Cardiovascular measurement	Cardiovascular	PGS000897	EFO_0004611	low density lipoprotein cholesterol measurement	measurement	EFO_0004611	Cardiovascular measurement	Cardiovascular
0.7433	1.80E-81	2.17E-78	PGS001724	EFO_0006930	brain volume measurement|neuroimaging measurement	brain volume measurement	EFO_0006930	Other measurement	Neurological	PGS001736	EFO_0006930	measurement	brain volume measurement	EFO_0006930	Other measurement	Neurological
0.7433	1.80E-81	2.17E-78	PGS000335	EFO_0000305	breast carcinoma	breast carcinoma	EFO_0000305	Cancer	Neoplasms	PGS000527	EFO_0000305	breast carcinoma	breast carcinoma	EFO_0000305	Cancer	Neoplasms
0.7433	1.81E-81	2.18E-78	PGS001162	EFO_0005093	hip circumference	hip circumference	EFO_0005093	Body measurement	Body measurement	PGS002176	EFO_0005093	hip circumference	hip circumference	EFO_0005093	Body measurement	Body measurement
0.7433	1.86E-81	2.24E-78	PGS002550	EFO_0004541	HbA1c measurement	HbA1c measurement	EFO_0004541	Hematological measurement	Hematological	PGS002449	EFO_0004468	glucose measurement	glucose measurement	EFO_0004468	Other measurement	Metabolic
0.7432	1.96E-81	2.36E-78	PGS002008	EFO_0004503	hematological measurement	hematological measurement	EFO_0004503	Hematological measurement	Hematological	PGS000987	EFO_0010701	mean reticulocyte volume	mean reticulocyte volume	EFO_0010701	Hematological measurement	Hematological
0.7431	2.04E-81	2.45E-78	PGS000331	EFO_0000275	atrial fibrillation	atrial fibrillation	EFO_0000275	Cardiovascular disease	Cardiovascular	PGS000016	EFO_0000275	atrial fibrillation	atrial fibrillation	EFO_0000275	Cardiovascular disease	Cardiovascular
0.7431	2.04E-81	2.45E-78	PGS001530	EFO_0007772	calcaneal bone quantitative ultrasound measurement	calcaneal bone quantitative ultrasound measurement	EFO_0007772	Other measurement	Skeletal	PGS000951	EFO_0004514	bone quantitative ultrasound measurement	measurement	EFO_0004514	Other measurement	Skeletal
0.7431	2.07E-81	2.49E-78	PGS000539	EFO_0000305	breast carcinoma	breast carcinoma	EFO_0000305	Cancer	Neoplasms	PGS000516	EFO_0000305	breast carcinoma	breast carcinoma	EFO_0000305	Cancer	Neoplasms
0.7431	2.13E-81	2.56E-78	PGS003243	EFO_0010472	creatine measurement	creatine measurement	EFO_0010472	Other measurement	Metabolic	PGS003240	EFO_0010472	creatine measurement	creatine measurement	EFO_0010472	Other measurement	Metabolic
0.743	2.19E-81	2.63E-78	PGS002993	EFO_0004339	body height	body height	EFO_0004339	Body measurement	Body measurement	PGS000758	EFO_0004339	body height	body height	EFO_0004339	Body measurement	Body measurement
0.7429	2.37E-81	2.84E-78	PGS003189	EFO_0004631	vitamin D measurement	vitamin D measurement	EFO_0004631	Lipid or lipoprotein measurement	Metabolic	PGS003196	EFO_0004631	vitamin D measurement	vitamin D measurement	EFO_0004631	Lipid or lipoprotein measurement	Metabolic
0.7428	2.56E-81	3.08E-78	PGS001488	EFO_0006930	brain volume measurement|neuroimaging measurement	brain volume measurement	EFO_0006930	Other measurement	Neurological	PGS001478	EFO_0006930	measurement	brain volume measurement	EFO_0006930	Other measurement	Neurological
0.7428	2.59E-81	3.11E-78	PGS000448	EFO_0004193	basal cell carcinoma	basal cell carcinoma	EFO_0004193	Cancer	Neoplasms	PGS000445	EFO_0004193	basal cell carcinoma	basal cell carcinoma	EFO_0004193	Cancer	Neoplasms
0.7427	2.76E-81	3.32E-78	PGS003385	EFO_1001516	ovarian serous carcinoma	ovarian serous carcinoma	EFO_1001516	Cancer	Neoplasms	PGS000552	EFO_0003893	ovarian neoplasm	ovarian neoplasm	EFO_0003893	Cancer	Neoplasms
0.7427	2.81E-81	3.37E-78	PGS002336	EFO_0004705	hypothyroidism	hypothyroidism	EFO_0004705	Other disease	Endocrine	PGS001181	EFO_0004705	hypothyroidism	hypothyroidism	EFO_0004705	Other disease	Endocrine
0.7427	2.85E-81	3.42E-78	PGS000110	EFO_0004305	erythrocyte count	erythrocyte count	EFO_0004305	Hematological measurement	Hematological	PGS001240	EFO_0004305	erythrocyte count	erythrocyte count	EFO_0004305	Hematological measurement	Hematological
0.7427	2.86E-81	3.44E-78	PGS002186	EFO_0005091	monocyte count	monocyte count	EFO_0005091	Hematological measurement	Hematological	PGS000102	EFO_0005091	monocyte count	monocyte count	EFO_0005091	Hematological measurement	Hematological
-0.7427	2.91E-81	3.49E-78	PGS003063	EFO_0006527	smoking status measurement	smoking status measurement	EFO_0006527	Other measurement	Psychiatric	PGS003360	EFO_0005670	smoking initiation	smoking initiation	EFO_0005670	Biological process	Psychiatric
0.7426	3.04E-81	3.64E-78	PGS001970	EFO_0007985	platelet crit	platelet crit	EFO_0007985	Hematological measurement	Hematological	PGS002191	EFO_0004309	platelet count	platelet count	EFO_0004309	Hematological measurement	Hematological
0.7426	3.11E-81	3.72E-78	PGS002339	EFO_0004527	mean corpuscular hemoglobin	mean corpuscular hemoglobin	EFO_0004527	Hematological measurement	Hematological	PGS002411	EFO_0004527	mean corpuscular hemoglobin	mean corpuscular hemoglobin	EFO_0004527	Hematological measurement	Hematological
0.7426	3.17E-81	3.80E-78	PGS000002	EFO_1000649	estrogen-receptor positive breast cancer	estrogen-receptor positive breast cancer	EFO_1000649	Cancer	Neoplasms	PGS000538	EFO_0000305	breast carcinoma	breast carcinoma	EFO_0000305	Cancer	Neoplasms
0.7425	3.19E-81	3.83E-78	PGS001973	EFO_0004309	platelet count	platelet count	EFO_0004309	Hematological measurement	Hematological	PGS002188	EFO_0007985	platelet crit	platelet crit	EFO_0007985	Hematological measurement	Hematological
0.7425	3.23E-81	3.86E-78	PGS002351	EFO_1000627	thyroid disease	thyroid disease	EFO_1000627	Other disease	Endocrine	PGS001816	EFO_0004705	hypothyroidism	hypothyroidism	EFO_0004705	Other disease	Endocrine
0.7425	3.30E-81	3.95E-78	PGS000090	EFO_0004842	eosinophil count	eosinophil count	EFO_0004842	Hematological measurement	Hematological	PGS002642	EFO_0004842	eosinophil count	eosinophil count	EFO_0004842	Hematological measurement	Hematological
0.7425	3.31E-81	3.96E-78	PGS002457	EFO_0004705	hypothyroidism	hypothyroidism	EFO_0004705	Other disease	Endocrine	PGS002472	EFO_1000627	thyroid disease	thyroid disease	EFO_1000627	Other disease	Endocrine
0.7425	3.36E-81	4.02E-78	PGS000539	EFO_0000305	breast carcinoma	breast carcinoma	EFO_0000305	Cancer	Neoplasms	PGS000536	EFO_0000305	breast carcinoma	breast carcinoma	EFO_0000305	Cancer	Neoplasms
0.7425	3.37E-81	4.04E-78	PGS001709	EFO_0006930	brain volume measurement|neuroimaging measurement	brain volume measurement	EFO_0006930	Other measurement	Neurological	PGS001754	EFO_0006930	measurement	brain volume measurement	EFO_0006930	Other measurement	Neurological
0.7424	3.59E-81	4.30E-78	PGS001908	EFO_0005192	red blood cell distribution width	red blood cell distribution width	EFO_0005192	Hematological measurement	Hematological	PGS001152	EFO_0005192	red blood cell distribution width	red blood cell distribution width	EFO_0005192	Hematological measurement	Hematological
0.7422	4.11E-81	4.91E-78	PGS001989	EFO_0004527	mean corpuscular hemoglobin	mean corpuscular hemoglobin	EFO_0004527	Hematological measurement	Hematological	PGS002607	EFO_0004527	mean corpuscular hemoglobin	mean corpuscular hemoglobin	EFO_0004527	Hematological measurement	Hematological
0.7422	4.13E-81	4.94E-78	PGS003030	EFO_0004611	low density lipoprotein cholesterol measurement	low density lipoprotein cholesterol measurement	EFO_0004611	Lipid or lipoprotein measurement	Metabolic	PGS000889	EFO_0004611	low density lipoprotein cholesterol measurement	measurement	EFO_0004611	Lipid or lipoprotein measurement	Metabolic
0.7422	4.13E-81	4.94E-78	PGS003030	EFO_0004611	low density lipoprotein cholesterol measurement	low density lipoprotein cholesterol measurement	EFO_0004611	Cardiovascular measurement	Cardiovascular	PGS000889	EFO_0004611	low density lipoprotein cholesterol measurement	measurement	EFO_0004611	Lipid or lipoprotein measurement	Metabolic
0.7422	4.13E-81	4.94E-78	PGS003030	EFO_0004611	low density lipoprotein cholesterol measurement	low density lipoprotein cholesterol measurement	EFO_0004611	Cardiovascular measurement	Cardiovascular	PGS000889	EFO_0004611	low density lipoprotein cholesterol measurement	measurement	EFO_0004611	Cardiovascular measurement	Cardiovascular
0.7422	4.13E-81	4.94E-78	PGS003030	EFO_0004611	low density lipoprotein cholesterol measurement	low density lipoprotein cholesterol measurement	EFO_0004611	Lipid or lipoprotein measurement	Metabolic	PGS000889	EFO_0004611	low density lipoprotein cholesterol measurement	measurement	EFO_0004611	Cardiovascular measurement	Cardiovascular
0.7422	4.17E-81	4.98E-78	PGS002844	EFO_0004340	body mass index	body mass index	EFO_0004340	Body measurement	Body measurement	PGS002133	EFO_0007800	body fat percentage	body fat percentage	EFO_0007800	Body measurement	Body measurement
0.7421	4.41E-81	5.27E-78	PGS001751	EFO_0006930	brain volume measurement|neuroimaging measurement	brain volume measurement	EFO_0006930	Other measurement	Neurological	PGS001753	EFO_0006930	measurement	brain volume measurement	EFO_0006930	Other measurement	Neurological
0.7421	4.57E-81	5.45E-78	PGS003147	EFO_0004530	triglyceride measurement	triglyceride measurement	EFO_0004530	Lipid or lipoprotein measurement	Metabolic	PGS002353	EFO_0004530	triglyceride measurement	triglyceride measurement	EFO_0004530	Lipid or lipoprotein measurement	Metabolic
0.7421	4.56E-81	5.45E-78	PGS001950	EFO_0004995	lean body mass	lean body mass	EFO_0004995	Other measurement	Skeletal	PGS001235	EFO_0004324	body weights and measures	body weights and measures	EFO_0004324	Body measurement	Body measurement
-0.742	4.73E-81	5.64E-78	PGS002330	EFO_0007822	hair colour measurement	hair colour measurement	EFO_0007822	Other measurement	Dermatological	PGS001092	EFO_0007822	hair colour measurement	hair colour measurement	EFO_0007822	Other measurement	Dermatological
0.742	4.77E-81	5.70E-78	PGS000002	EFO_1000649	estrogen-receptor positive breast cancer	estrogen-receptor positive breast cancer	EFO_1000649	Cancer	Neoplasms	PGS000540	EFO_0000305	breast carcinoma	breast carcinoma	EFO_0000305	Cancer	Neoplasms
0.742	4.78E-81	5.70E-78	PGS001229	EFO_0004339	body height	body height	EFO_0004339	Body measurement	Body measurement	PGS002969	EFO_0004339	body height	body height	EFO_0004339	Body measurement	Body measurement
0.742	4.89E-81	5.83E-78	PGS000669	EFO_0004535	serum albumin measurement	serum albumin measurement	EFO_0004535	Other measurement	Metabolic	PGS001886	EFO_0004535	serum albumin measurement	serum albumin measurement	EFO_0004535	Other measurement	Metabolic
0.742	4.95E-81	5.90E-78	PGS003186	EFO_0004631	vitamin D measurement	vitamin D measurement	EFO_0004631	Lipid or lipoprotein measurement	Metabolic	PGS003190	EFO_0004631	vitamin D measurement	vitamin D measurement	EFO_0004631	Lipid or lipoprotein measurement	Metabolic
0.7419	5.18E-81	6.17E-78	PGS000015	EFO_0000305	breast carcinoma	breast carcinoma	EFO_0000305	Cancer	Neoplasms	PGS000504	EFO_0000305	breast carcinoma	breast carcinoma	EFO_0000305	Cancer	Neoplasms
0.7418	5.42E-81	6.46E-78	PGS000103	EFO_0007989	monocyte percentage of leukocytes	monocyte percentage of leukocytes	EFO_0007989	Hematological measurement	Hematological	PGS001078	EFO_0007989	monocyte percentage of leukocytes	monocyte percentage of leukocytes	EFO_0007989	Hematological measurement	Hematological
0.7418	5.44E-81	6.49E-78	PGS001240	EFO_0004305	erythrocyte count	erythrocyte count	EFO_0004305	Hematological measurement	Hematological	PGS002662	EFO_0004305	erythrocyte count	erythrocyte count	EFO_0004305	Hematological measurement	Hematological
0.7418	5.51E-81	6.57E-78	PGS002090	HP_0003040	Arthropathy	Arthropathy	HP_0003040	Other trait	Skeletal	PGS001877	HP_0003040	Arthropathy	Arthropathy	HP_0003040	Other trait	Skeletal
0.7418	5.52E-81	6.58E-78	PGS002965	EFO_0004339	body height	body height	EFO_0004339	Body measurement	Body measurement	PGS000758	EFO_0004339	body height	body height	EFO_0004339	Body measurement	Body measurement
-0.7418	5.62E-81	6.69E-78	PGS000884	EFO_0005208	glomerular filtration rate	glomerular filtration rate	EFO_0005208	Other measurement	Genitourinary	PGS002237	EFO_0003884	chronic kidney disease	chronic kidney disease	EFO_0003884	Other disease	Metabolic
0.7418	5.69E-81	6.77E-78	PGS001911	EFO_0006527	smoking status measurement	smoking status measurement	EFO_0006527	Other measurement	Psychiatric	PGS003058	EFO_0006527	smoking status measurement	smoking status measurement	EFO_0006527	Other measurement	Psychiatric
0.7417	5.88E-81	7.00E-78	PGS000900	EFO_0006335	systolic blood pressure|female	systolic blood pressure	EFO_0006335	Other measurement	Cardiovascular	PGS002228	EFO_0006335	systolic blood pressure	systolic blood pressure	EFO_0006335	Other measurement	Cardiovascular
0.7417	5.93E-81	7.05E-78	PGS003031	EFO_0004611	low density lipoprotein cholesterol measurement	low density lipoprotein cholesterol measurement	EFO_0004611	Cardiovascular measurement	Cardiovascular	PGS003037	EFO_0004611	low density lipoprotein cholesterol measurement	measurement	EFO_0004611	Lipid or lipoprotein measurement	Metabolic
0.7417	5.93E-81	7.05E-78	PGS003031	EFO_0004611	low density lipoprotein cholesterol measurement	low density lipoprotein cholesterol measurement	EFO_0004611	Lipid or lipoprotein measurement	Metabolic	PGS003037	EFO_0004611	low density lipoprotein cholesterol measurement	measurement	EFO_0004611	Lipid or lipoprotein measurement	Metabolic
0.7417	5.93E-81	7.05E-78	PGS003031	EFO_0004611	low density lipoprotein cholesterol measurement	low density lipoprotein cholesterol measurement	EFO_0004611	Cardiovascular measurement	Cardiovascular	PGS003037	EFO_0004611	low density lipoprotein cholesterol measurement	measurement	EFO_0004611	Cardiovascular measurement	Cardiovascular
0.7417	5.93E-81	7.05E-78	PGS003031	EFO_0004611	low density lipoprotein cholesterol measurement	low density lipoprotein cholesterol measurement	EFO_0004611	Lipid or lipoprotein measurement	Metabolic	PGS003037	EFO_0004611	low density lipoprotein cholesterol measurement	measurement	EFO_0004611	Cardiovascular measurement	Cardiovascular
0.7417	5.94E-81	7.07E-78	PGS000455	EFO_0004193	basal cell carcinoma	basal cell carcinoma	EFO_0004193	Cancer	Neoplasms	PGS000467	EFO_0000707	squamous cell carcinoma	squamous cell carcinoma	EFO_0000707	Cancer	Neoplasms
0.7417	5.94E-81	7.07E-78	PGS000452	EFO_0004193	basal cell carcinoma	basal cell carcinoma	EFO_0004193	Cancer	Neoplasms	PGS000467	EFO_0000707	squamous cell carcinoma	squamous cell carcinoma	EFO_0000707	Cancer	Neoplasms
0.7417	5.94E-81	7.07E-78	PGS000467	EFO_0000707	squamous cell carcinoma	squamous cell carcinoma	EFO_0000707	Cancer	Neoplasms	PGS000453	EFO_0004193	basal cell carcinoma	basal cell carcinoma	EFO_0004193	Cancer	Neoplasms
0.7417	6.28E-81	7.46E-78	PGS001451	EFO_0006930	brain volume measurement|neuroimaging measurement	brain volume measurement	EFO_0006930	Other measurement	Neurological	PGS001673	EFO_0006930	measurement	brain volume measurement	EFO_0006930	Other measurement	Neurological
0.7416	6.54E-81	7.78E-78	PGS001480	EFO_0006930	brain volume measurement|neuroimaging measurement	brain volume measurement	EFO_0006930	Other measurement	Neurological	PGS001680	EFO_0006930	measurement	brain volume measurement	EFO_0006930	Other measurement	Neurological
0.7416	6.56E-81	7.80E-78	PGS002005	EFO_0011011	sitting height measurement	sitting height measurement	EFO_0011011	Other measurement	Skeletal	PGS002968	EFO_0004339	body height	body height	EFO_0004339	Body measurement	Body measurement
0.7416	6.71E-81	7.97E-78	PGS002840	EFO_0004340	body mass index	body mass index	EFO_0004340	Body measurement	Body measurement	PGS002851	EFO_0004340	body mass index	body mass index	EFO_0004340	Body measurement	Body measurement
0.7416	6.76E-81	8.03E-78	PGS002353	EFO_0004530	triglyceride measurement	triglyceride measurement	EFO_0004530	Lipid or lipoprotein measurement	Metabolic	PGS003152	EFO_0004530	triglyceride measurement	triglyceride measurement	EFO_0004530	Lipid or lipoprotein measurement	Metabolic
0.7415	6.87E-81	8.16E-78	PGS003147	EFO_0004530	triglyceride measurement	triglyceride measurement	EFO_0004530	Lipid or lipoprotein measurement	Metabolic	PGS002197	EFO_0004530	triglyceride measurement	triglyceride measurement	EFO_0004530	Lipid or lipoprotein measurement	Metabolic
0.7415	6.97E-81	8.27E-78	PGS002459	EFO_0004587	lymphocyte count	lymphocyte count	EFO_0004587	Hematological measurement	Hematological	PGS002508	EFO_0004587	lymphocyte count	lymphocyte count	EFO_0004587	Hematological measurement	Hematological
0.7415	6.97E-81	8.28E-78	PGS001943	EFO_0004340	body mass index	body mass index	EFO_0004340	Body measurement	Body measurement	PGS001228	EFO_0004340	body mass index	body mass index	EFO_0004340	Body measurement	Body measurement
0.7414	7.42E-81	8.80E-78	PGS003234	EFO_0010472	creatine measurement	creatine measurement	EFO_0010472	Other measurement	Metabolic	PGS003229	EFO_0010472	creatine measurement	creatine measurement	EFO_0010472	Other measurement	Metabolic
0.7414	7.55E-81	8.96E-78	PGS002150	EFO_0004611	low density lipoprotein cholesterol measurement	low density lipoprotein cholesterol measurement	EFO_0004611	Cardiovascular measurement	Cardiovascular	PGS000677	EFO_0004574	total cholesterol measurement	total cholesterol measurement	EFO_0004574	Lipid or lipoprotein measurement	Metabolic
0.7414	7.55E-81	8.96E-78	PGS002150	EFO_0004611	low density lipoprotein cholesterol measurement	low density lipoprotein cholesterol measurement	EFO_0004611	Lipid or lipoprotein measurement	Metabolic	PGS000677	EFO_0004574	total cholesterol measurement	total cholesterol measurement	EFO_0004574	Lipid or lipoprotein measurement	Metabolic
0.7414	7.71E-81	9.14E-78	PGS002150	EFO_0004611	low density lipoprotein cholesterol measurement	low density lipoprotein cholesterol measurement	EFO_0004611	Cardiovascular measurement	Cardiovascular	PGS000065	EFO_0004611	low density lipoprotein cholesterol measurement	measurement	EFO_0004611	Cardiovascular measurement	Cardiovascular
0.7414	7.71E-81	9.14E-78	PGS002150	EFO_0004611	low density lipoprotein cholesterol measurement	low density lipoprotein cholesterol measurement	EFO_0004611	Cardiovascular measurement	Cardiovascular	PGS000065	EFO_0004611	low density lipoprotein cholesterol measurement	measurement	EFO_0004611	Lipid or lipoprotein measurement	Metabolic
0.7414	7.71E-81	9.14E-78	PGS002150	EFO_0004611	low density lipoprotein cholesterol measurement	low density lipoprotein cholesterol measurement	EFO_0004611	Lipid or lipoprotein measurement	Metabolic	PGS000065	EFO_0004611	low density lipoprotein cholesterol measurement	measurement	EFO_0004611	Cardiovascular measurement	Cardiovascular
0.7414	7.71E-81	9.14E-78	PGS002150	EFO_0004611	low density lipoprotein cholesterol measurement	low density lipoprotein cholesterol measurement	EFO_0004611	Lipid or lipoprotein measurement	Metabolic	PGS000065	EFO_0004611	low density lipoprotein cholesterol measurement	measurement	EFO_0004611	Lipid or lipoprotein measurement	Metabolic
0.7413	8.30E-81	9.84E-78	PGS000499	EFO_0000305	breast carcinoma	breast carcinoma	EFO_0000305	Cancer	Neoplasms	PGS000535	EFO_0000305	breast carcinoma	breast carcinoma	EFO_0000305	Cancer	Neoplasms
0.7413	8.33E-81	9.88E-78	PGS002554	EFO_0000537	hypertension	hypertension	EFO_0000537	Cardiovascular disease	Cardiovascular	PGS002568	EFO_0006335	systolic blood pressure	systolic blood pressure	EFO_0006335	Other measurement	Cardiovascular
0.7413	8.43E-81	9.99E-78	PGS000699	EFO_0004530	triglyceride measurement	triglyceride measurement	EFO_0004530	Lipid or lipoprotein measurement	Metabolic	PGS002353	EFO_0004530	triglyceride measurement	triglyceride measurement	EFO_0004530	Lipid or lipoprotein measurement	Metabolic
0.7412	8.57E-81	1.01E-77	PGS001671	EFO_0006930	brain volume measurement|neuroimaging measurement	brain volume measurement	EFO_0006930	Other measurement	Neurological	PGS001450	EFO_0006930	measurement	brain volume measurement	EFO_0006930	Other measurement	Neurological
0.7412	8.73E-81	1.03E-77	PGS000687	EFO_0004627	IGF-1 measurement	IGF-1 measurement	EFO_0004627	Other measurement	Metabolic	PGS002178	EFO_0004627	IGF-1 measurement	IGF-1 measurement	EFO_0004627	Other measurement	Metabolic
0.7411	9.18E-81	1.09E-77	PGS003099	MONDO_0005148	type 2 diabetes mellitus	type 2 diabetes mellitus	MONDO_0005148	Metabolic disorder	Metabolic	PGS002308	MONDO_0005148	type 2 diabetes mellitus	type 2 diabetes mellitus	MONDO_0005148	Digestive system disorder	Gastrointestinal
0.7411	9.18E-81	1.09E-77	PGS003099	MONDO_0005148	type 2 diabetes mellitus	type 2 diabetes mellitus	MONDO_0005148	Digestive system disorder	Gastrointestinal	PGS002308	MONDO_0005148	type 2 diabetes mellitus	type 2 diabetes mellitus	MONDO_0005148	Metabolic disorder	Metabolic
0.7411	9.18E-81	1.09E-77	PGS003099	MONDO_0005148	type 2 diabetes mellitus	type 2 diabetes mellitus	MONDO_0005148	Digestive system disorder	Gastrointestinal	PGS002308	MONDO_0005148	type 2 diabetes mellitus	type 2 diabetes mellitus	MONDO_0005148	Digestive system disorder	Gastrointestinal
0.7411	9.18E-81	1.09E-77	PGS003099	MONDO_0005148	type 2 diabetes mellitus	type 2 diabetes mellitus	MONDO_0005148	Metabolic disorder	Metabolic	PGS002308	MONDO_0005148	type 2 diabetes mellitus	type 2 diabetes mellitus	MONDO_0005148	Metabolic disorder	Metabolic
0.7411	9.35E-81	1.11E-77	PGS001950	EFO_0004995	lean body mass	lean body mass	EFO_0004995	Other measurement	Skeletal	PGS001234	EFO_0004324	body weights and measures	body weights and measures	EFO_0004324	Body measurement	Body measurement
0.7411	9.48E-81	1.12E-77	PGS003401	EFO_0004530	triglyceride measurement	triglyceride measurement	EFO_0004530	Lipid or lipoprotein measurement	Metabolic	PGS000066	EFO_0004530	triglyceride measurement	triglyceride measurement	EFO_0004530	Lipid or lipoprotein measurement	Metabolic
0.7411	9.51E-81	1.13E-77	PGS001248	EFO_0004274	gout	gout	EFO_0004274	Metabolic disorder	Metabolic	PGS002030	EFO_0004274	gout	gout	EFO_0004274	Metabolic disorder	Metabolic

0.7411	9.76E-81	1.15E-77	PGS000069	EFO_1001516	ovarian serous carcinoma	ovarian serous carcinoma	EFO_1001516	Cancer	Neoplasms	PGS000549	EFO_0003893	ovarian neoplasm	ovarian neoplasm	EFO_0003893	Cancer	Neoplasms
0.741	9.86E-81	1.17E-77	PGS002347	EFO_0000684	respiratory system disease	respiratory system disease	EFO_0000684	Other disease	Skeletal	PGS002664	EFO_0000684	respiratory system disease	respiratory system disease	EFO_0000684	Other disease	Skeletal
0.741	1.01E-80	1.20E-77	PGS002571	EFO_0004574	total cholesterol measurement	total cholesterol measurement	EFO_0004574	Lipid or lipoprotein measurement	Metabolic	PGS002602	HP_0003124	Hypercholesterolemia	Hypercholesterolemia	HP_0003124	Other trait	Metabolic
0.741	1.02E-80	1.21E-77	PGS002201	EFO_0004342	waist circumference	waist circumference	EFO_0004342	Body measurement	Body measurement	PGS002849	EFO_0004340	body mass index	body mass index	EFO_0004340	Body measurement	Body measurement
0.741	1.04E-80	1.23E-77	PGS001724	EFO_0006930	brain volume measurement|neuroimaging measurement	brain volume measurement	EFO_0006930	Other measurement	Neurological	PGS001752	EFO_0006930	measurement	brain volume measurement	EFO_0006930	Other measurement	Neurological
0.741	1.05E-80	1.24E-77	PGS000482	EFO_0000305	breast carcinoma	breast carcinoma	EFO_0000305	Cancer	Neoplasms	PGS001336	EFO_0000305	breast carcinoma	breast carcinoma	EFO_0000305	Cancer	Neoplasms
-0.7409	1.07E-80	1.26E-77	PGS002330	EFO_0007822	hair colour measurement	hair colour measurement	EFO_0007822	Other measurement	Dermatological	PGS001094	EFO_0007822	hair colour measurement	hair colour measurement	EFO_0007822	Other measurement	Dermatological
0.7409	1.08E-80	1.27E-77	PGS000340	EFO_0004611	low density lipoprotein cholesterol measurement	low density lipoprotein cholesterol measurement	EFO_0004611	Cardiovascular measurement	Cardiovascular	PGS000115	EFO_0004611	low density lipoprotein cholesterol measurement	measurement	EFO_0004611	Cardiovascular measurement	Cardiovascular
0.7409	1.08E-80	1.27E-77	PGS003403	EFO_0004611	low density lipoprotein cholesterol measurement	low density lipoprotein cholesterol measurement	EFO_0004611	Cardiovascular measurement	Cardiovascular	PGS000115	EFO_0004611	low density lipoprotein cholesterol measurement	measurement	EFO_0004611	Cardiovascular measurement	Cardiovascular
0.7409	1.08E-80	1.27E-77	PGS000340	EFO_0004611	low density lipoprotein cholesterol measurement	low density lipoprotein cholesterol measurement	EFO_0004611	Lipid or lipoprotein measurement	Metabolic	PGS000115	EFO_0004611	low density lipoprotein cholesterol measurement	measurement	EFO_0004611	Lipid or lipoprotein measurement	Metabolic
0.7409	1.08E-80	1.27E-77	PGS003403	EFO_0004611	low density lipoprotein cholesterol measurement	low density lipoprotein cholesterol measurement	EFO_0004611	Lipid or lipoprotein measurement	Metabolic	PGS000115	EFO_0004611	low density lipoprotein cholesterol measurement	measurement	EFO_0004611	Cardiovascular measurement	Cardiovascular
0.7409	1.08E-80	1.27E-77	PGS003403	EFO_0004611	low density lipoprotein cholesterol measurement	low density lipoprotein cholesterol measurement	EFO_0004611	Cardiovascular measurement	Cardiovascular	PGS000115	EFO_0004611	low density lipoprotein cholesterol measurement	measurement	EFO_0004611	Lipid or lipoprotein measurement	Metabolic
0.7409	1.08E-80	1.27E-77	PGS003403	EFO_0004611	low density lipoprotein cholesterol measurement	low density lipoprotein cholesterol measurement	EFO_0004611	Lipid or lipoprotein measurement	Metabolic	PGS000115	EFO_0004611	low density lipoprotein cholesterol measurement	measurement	EFO_0004611	Lipid or lipoprotein measurement	Metabolic
0.7409	1.08E-80	1.27E-77	PGS000340	EFO_0004611	low density lipoprotein cholesterol measurement	low density lipoprotein cholesterol measurement	EFO_0004611	Lipid or lipoprotein measurement	Metabolic	PGS000115	EFO_0004611	low density lipoprotein cholesterol measurement	measurement	EFO_0004611	Cardiovascular measurement	Cardiovascular
0.7409	1.08E-80	1.27E-77	PGS000340	EFO_0004611	low density lipoprotein cholesterol measurement	low density lipoprotein cholesterol measurement	EFO_0004611	Cardiovascular measurement	Cardiovascular	PGS000115	EFO_0004611	low density lipoprotein cholesterol measurement	measurement	EFO_0004611	Lipid or lipoprotein measurement	Metabolic
0.7408	1.22E-80	1.43E-77	PGS002409	EFO_0004611	low density lipoprotein cholesterol measurement	low density lipoprotein cholesterol measurement	EFO_0004611	Lipid or lipoprotein measurement	Metabolic	PGS000889	EFO_0004611	low density lipoprotein cholesterol measurement	measurement	EFO_0004611	Lipid or lipoprotein measurement	Metabolic
0.7408	1.22E-80	1.43E-77	PGS002409	EFO_0004611	low density lipoprotein cholesterol measurement	low density lipoprotein cholesterol measurement	EFO_0004611	Lipid or lipoprotein measurement	Metabolic	PGS000889	EFO_0004611	low density lipoprotein cholesterol measurement	measurement	EFO_0004611	Cardiovascular measurement	Cardiovascular
0.7408	1.22E-80	1.43E-77	PGS002409	EFO_0004611	low density lipoprotein cholesterol measurement	low density lipoprotein cholesterol measurement	EFO_0004611	Cardiovascular measurement	Cardiovascular	PGS000889	EFO_0004611	low density lipoprotein cholesterol measurement	measurement	EFO_0004611	Lipid or lipoprotein measurement	Metabolic
0.7408	1.22E-80	1.43E-77	PGS002409	EFO_0004611	low density lipoprotein cholesterol measurement	low density lipoprotein cholesterol measurement	EFO_0004611	Lipid or lipoprotein measurement	Metabolic	PGS000889	EFO_0004611	low density lipoprotein cholesterol measurement	measurement	EFO_0004611	Cardiovascular measurement	Cardiovascular
0.7407	1.28E-80	1.52E-77	PGS000779	MONDO_0004975	Alzheimer disease	Alzheimer disease	MONDO_0004975	Neurological disorder	Neurological	PGS000929	MONDO_0001627	dementia	dementia	MONDO_0001627	Neurological disorder	Neurological
0.7407	1.29E-80	1.52E-77	PGS002162	EFO_0007934	creatinine clearance measurement	creatinine clearance measurement	EFO_0007934	Other measurement	Metabolic	PGS002192	EFO_0010952	urinary potassium measurement	urinary potassium measurement	EFO_0010952	Other measurement	Metabolic
0.7406	1.36E-80	1.60E-77	PGS000586	EFO_0001663	prostate carcinoma	prostate carcinoma	EFO_0001663	Cancer	Neoplasms	PGS000587	EFO_0001663	prostate carcinoma	prostate carcinoma	EFO_0001663	Cancer	Neoplasms
0.7405	1.48E-80	1.74E-77	PGS001984	EFO_0009805	whole body water mass	whole body water mass	EFO_0009805	Other measurement	Body measurement	PGS001146	EFO_0005409	fat body mass	fat body mass	EFO_0005409	Other measurement	Body measurement
0.7405	1.48E-80	1.75E-77	PGS000002	EFO_1000649	estrogen-receptor positive breast cancer	estrogen-receptor positive breast cancer	EFO_1000649	Cancer	Neoplasms	PGS000488	EFO_0000305	breast carcinoma	breast carcinoma	EFO_0000305	Cancer	Neoplasms
0.7404	1.55E-80	1.83E-77	PGS000526	EFO_0000305	breast carcinoma	breast carcinoma	EFO_0000305	Cancer	Neoplasms	PGS000513	EFO_0000305	breast carcinoma	breast carcinoma	EFO_0000305	Cancer	Neoplasms
0.7404	1.55E-80	1.83E-77	PGS000525	EFO_0000305	breast carcinoma	breast carcinoma	EFO_0000305	Cancer	Neoplasms	PGS000513	EFO_0000305	breast carcinoma	breast carcinoma	EFO_0000305	Cancer	Neoplasms
0.7404	1.56E-80	1.84E-77	PGS002554	EFO_0000537	hypertension	hypertension	EFO_0000537	Cardiovascular disease	Cardiovascular	PGS002541	EFO_0006336	diastolic blood pressure	diastolic blood pressure	EFO_0006336	Other measurement	Cardiovascular
0.7404	1.59E-80	1.87E-77	PGS002854	EFO_0004340	body mass index	body mass index	EFO_0004340	Body measurement	Body measurement	PGS002201	EFO_0004342	waist circumference	waist circumference	EFO_0004342	Body measurement	Body measurement
0.7404	1.65E-80	1.95E-77	PGS002603	EFO_0000537	hypertension	hypertension	EFO_0000537	Cardiovascular disease	Cardiovascular	PGS002568	EFO_0006335	systolic blood pressure	systolic blood pressure	EFO_0006335	Other measurement	Cardiovascular
0.7403	1.66E-80	1.96E-77	PGS001926	EFO_0004509	hemoglobin measurement	hemoglobin measurement	EFO_0004509	Hematological measurement	Hematological	PGS000093	EFO_0004509	hemoglobin measurement	hemoglobin measurement	EFO_0004509	Hematological measurement	Hematological
0.7402	1.92E-80	2.25E-77	PGS000479	EFO_0000305	breast carcinoma	breast carcinoma	EFO_0000305	Cancer	Neoplasms	PGS000513	EFO_0000305	breast carcinoma	breast carcinoma	EFO_0000305	Cancer	Neoplasms
0.7401	1.93E-80	2.27E-77	PGS000677	EFO_0004574	total cholesterol measurement	total cholesterol measurement	EFO_0004574	Lipid or lipoprotein measurement	Metabolic	PGS002654	EFO_0004611	low density lipoprotein cholesterol measurement	measurement	EFO_0004611	Cardiovascular measurement	Cardiovascular
0.7401	1.93E-80	2.27E-77	PGS000677	EFO_0004574	total cholesterol measurement	total cholesterol measurement	EFO_0004574	Lipid or lipoprotein measurement	Metabolic	PGS002654	EFO_0004611	low density lipoprotein cholesterol measurement	measurement	EFO_0004611	Lipid or lipoprotein measurement	Metabolic
0.7401	2.00E-80	2.35E-77	PGS000310	EFO_0004611	low density lipoprotein cholesterol measurement	low density lipoprotein cholesterol measurement	EFO_0004611	Lipid or lipoprotein measurement	Metabolic	PGS001933	EFO_0004611	low density lipoprotein cholesterol measurement	measurement	EFO_0004611	Lipid or lipoprotein measurement	Metabolic
0.7401	2.00E-80	2.35E-77	PGS000310	EFO_0004611	low density lipoprotein cholesterol measurement	low density lipoprotein cholesterol measurement	EFO_0004611	Lipid or lipoprotein measurement	Metabolic	PGS001933	EFO_0004611	low density lipoprotein cholesterol measurement	measurement	EFO_0004611	Cardiovascular measurement	Cardiovascular
0.7401	2.00E-80	2.35E-77	PGS000310	EFO_0004611	low density lipoprotein cholesterol measurement	low density lipoprotein cholesterol measurement	EFO_0004611	Cardiovascular measurement	Cardiovascular	PGS001933	EFO_0004611	low density lipoprotein cholesterol measurement	measurement	EFO_0004611	Lipid or lipoprotein measurement	Metabolic
0.7401	2.00E-80	2.35E-77	PGS000310	EFO_0004611	low density lipoprotein cholesterol measurement	low density lipoprotein cholesterol measurement	EFO_0004611	Cardiovascular measurement	Cardiovascular	PGS001933	EFO_0004611	low density lipoprotein cholesterol measurement	measurement	EFO_0004611	Cardiovascular measurement	Cardiovascular
0.7401	2.01E-80	2.36E-77	PGS001339	EFO_0000275	atrial fibrillation|atrial flutter	atrial fibrillation	EFO_0000275	Cardiovascular disease	Cardiovascular	PGS002050	EFO_0003911	atrial flutter|atrial fibrillation	atrial flutter	EFO_0003911	Cardiovascular disease	Cardiovascular
0.7401	2.05E-80	2.41E-77	PGS001661	EFO_0006930	brain volume measurement|neuroimaging measurement	brain volume measurement	EFO_0006930	Other measurement	Neurological	PGS001672	EFO_0006930	measurement	brain volume measurement	EFO_0006930	Other measurement	Neurological
0.7401	2.07E-80	2.44E-77	PGS002963	EFO_0004612	high density lipoprotein cholesterol measurement	high density lipoprotein cholesterol measurement	EFO_0004612	Lipid or lipoprotein measurement	Metabolic	PGS001888	EFO_0004614	apolipoprotein A 1 measurement	apolipoprotein A 1 measurement	EFO_0004614	Lipid or lipoprotein measurement	Metabolic
0.7401	2.07E-80	2.44E-77	PGS002963	EFO_0004612	high density lipoprotein cholesterol measurement	high density lipoprotein cholesterol measurement	EFO_0004612	Lipid or lipoprotein measurement	Metabolic	PGS001888	EFO_0004614	apolipoprotein A 1 measurement	apolipoprotein A 1 measurement	EFO_0004614	Inflammatory measurement	Immunological
0.7401	2.07E-80	2.44E-77	PGS002963	EFO_0004612	high density lipoprotein cholesterol measurement	high density lipoprotein cholesterol measurement	EFO_0004612	Lipid or lipoprotein measurement	Metabolic	PGS001888	EFO_0004614	apolipoprotein A 1 measurement	apolipoprotein A 1 measurement	EFO_0004614	Cardiovascular measurement	Cardiovascular
-0.74	2.10E-80	2.47E-77	PGS000708	EFO_1002048	kidney failure	kidney failure	EFO_1002048	Other disease	Genitourinary	PGS000682	EFO_0005208	glomerular filtration rate	glomerular filtration rate	EFO_0005208	Other measurement	Genitourinary
0.74	2.11E-80	2.48E-77	PGS002142	EFO_0004509	hemoglobin measurement	hemoglobin measurement	EFO_0004509	Hematological measurement	Hematological	PGS001400	EFO_0004509	hemoglobin measurement	hemoglobin measurement	EFO_0004509	Hematological measurement	Hematological
0.74	2.12E-80	2.49E-77	PGS002325	EFO_0004842	eosinophil count	eosinophil count	EFO_0004842	Hematological measurement	Hematological	PGS000091	EFO_0007991	eosinophil percentage of leukocytes	eosinophil percentage of leukocytes	EFO_0007991	Hematological measurement	Hematological
0.74	2.13E-80	2.50E-77	PGS000783	EFO_0000305	breast carcinoma	breast carcinoma	EFO_0000305	Cancer	Neoplasms	PGS000001	EFO_0000305	breast carcinoma	breast carcinoma	EFO_0000305	Cancer	Neoplasms
0.74	2.21E-80	2.60E-77	PGS002009	EFO_0006335	systolic blood pressure	systolic blood pressure	EFO_0006335	Other measurement	Cardiovascular	PGS003074	EFO_0006335	systolic blood pressure	systolic blood pressure	EFO_0006335	Other measurement	Cardiovascular
0.7399	2.28E-80	2.67E-77	PGS002106	EFO_0004838	calcium measurement	calcium measurement	EFO_0004838	Other measurement	Metabolic	PGS000676	EFO_0004838	calcium measurement	calcium measurement	EFO_0004838	Other measurement	Metabolic
0.7399	2.32E-80	2.73E-77	PGS000310	EFO_0004611	low density lipoprotein cholesterol measurement	low density lipoprotein cholesterol measurement	EFO_0004611	Cardiovascular measurement	Cardiovascular	PGS001889	EFO_0004615	apolipoprotein B measurement	apolipoprotein B measurement	EFO_0004615	Lipid or lipoprotein measurement	Metabolic
0.7399	2.32E-80	2.73E-77	PGS000310	EFO_0004611	low density lipoprotein cholesterol measurement	low density lipoprotein cholesterol measurement	EFO_0004611	Lipid or lipoprotein measurement	Metabolic	PGS001889	EFO_0004615	apolipoprotein B measurement	apolipoprotein B measurement	EFO_0004615	Lipid or lipoprotein measurement	Metabolic
0.7398	2.46E-80	2.89E-77	PGS003201	EFO_0004631	vitamin D measurement	vitamin D measurement	EFO_0004631	Lipid or lipoprotein measurement	Metabolic	PGS003195	EFO_0004631	vitamin D measurement	vitamin D measurement	EFO_0004631	Lipid or lipoprotein measurement	Metabolic
0.7398	2.50E-80	2.93E-77	PGS000008	EFO_1000649	estrogen-receptor positive breast cancer	estrogen-receptor positive breast cancer	EFO_1000649	Cancer	Neoplasms	PGS000505	EFO_0000305	breast carcinoma	breast carcinoma	EFO_0000305	Cancer	Neoplasms
0.7398	2.50E-80	2.93E-77	PGS000008	EFO_1000649	estrogen-receptor positive breast cancer	estrogen-receptor positive breast cancer	EFO_1000649	Cancer	Neoplasms	PGS000506	EFO_0000305	breast carcinoma	breast carcinoma	EFO_0000305	Cancer	Neoplasms
0.7398	2.54E-80	2.98E-77	PGS000517	EFO_0000305	breast carcinoma	breast carcinoma	EFO_0000305	Cancer	Neoplasms	PGS000524	EFO_0000305	breast carcinoma	breast carcinoma	EFO_0000305	Cancer	Neoplasms
0.7398	2.54E-80	2.98E-77	PGS000518	EFO_0000305	breast carcinoma	breast carcinoma	EFO_0000305	Cancer	Neoplasms	PGS000524	EFO_0000305	breast carcinoma	breast carcinoma	EFO_0000305	Cancer	Neoplasms
0.7397	2.65E-80	3.11E-77	PGS002453	EFO_0004339	body height	body height	EFO_0004339	Body measurement	Body measurement	PGS002968	EFO_0004339	body height	body height	EFO_0004339	Body measurement	Body measurement
0.7397	2.65E-80	3.11E-77	PGS003185	EFO_0004631	vitamin D measurement	vitamin D measurement	EFO_0004631	Lipid or lipoprotein measurement	Metabolic	PGS003190	EFO_0004631	vitamin D measurement	vitamin D measurement	EFO_0004631	Lipid or lipoprotein measurement	Metabolic
0.7397	2.68E-80	3.13E-77	PGS002837	EFO_0007878	alcohol consumption measurement	alcohol consumption measurement	EFO_0007878	Other measurement	Psychiatric	PGS002836	EFO_0007878	alcohol consumption measurement	alcohol consumption measurement	EFO_0007878	Other measurement	Psychiatric
0.7397	2.73E-80	3.19E-77	PGS000530	EFO_0000305	breast carcinoma	breast carcinoma	EFO_0000305	Cancer	Neoplasms	PGS000502	EFO_0000305	breast carcinoma	breast carcinoma	EFO_0000305	Cancer	Neoplasms
0.7397	2.74E-80	3.21E-77	PGS000437	MONDO_0002898	skin cancer	skin cancer	MONDO_0002898	Cancer	Neoplasms	PGS000443	MONDO_0002898	skin cancer	skin cancer	MONDO_0002898	Cancer	Neoplasms
0.7397	2.79E-80	3.27E-77	PGS000096	EFO_0007986	reticulocyte count	reticulocyte count	EFO_0007986	Hematological measurement	Hematological	PGS001930	EFO_0009253	Immature Reticulocyte Fraction Measurement	Measurement	EFO_0009253	Hematological measurement	Hematological
0.7396	2.87E-80	3.36E-77	PGS001621	EFO_0005420	measurement	grey matter volume measurement	EFO_0005420	Other measurement	Neurological	PGS001622	EFO_0005420	measurement	grey matter volume measurement	EFO_0005420	Other measurement	Neurological
0.7396	2.97E-80	3.48E-77	PGS001807	MONDO_0004986	urinary bladder carcinoma	urinary bladder carcinoma	MONDO_0004986	Cancer	Neoplasms	PGS002017	MONDO_0004986	urinary bladder carcinoma	urinary bladder carcinoma	MONDO_0004986	Cancer	Neoplasms
0.7395	3.05E-80	3.57E-77	PGS000775	EFO_1000650	estrogen-receptor negative breast cancer	estrogen-receptor negative breast cancer	EFO_1000650	Cancer	Neoplasms	PGS000052	EFO_0000305	breast carcinoma	breast carcinoma	EFO_0000305	Cancer	Neoplasms
0.7395	3.14E-80	3.67E-77	PGS001037	EFO_0009270	heel bone mineral density	heel bone mineral density	EFO_0009270	Other measurement	Skeletal	PGS001404	EFO_0009270	heel bone mineral density	heel bone mineral density	EFO_0009270	Other measurement	Skeletal
0.7395	3.21E-80	3.76E-77	PGS001531	EFO_0007772	calcaneal bone quantitative ultrasound measurement	calcaneal bone quantitative ultrasound measurement	EFO_0007772	Other measurement	Skeletal	PGS001529	EFO_0007772	measurement	measurement	EFO_0007772	Other measurement	Skeletal
0.7395	3.22E-80	3.76E-77	PGS000395	MONDO_0001672	bronchus cancer|lung cancer|tracheal cancer	bronchus cancer	MONDO_0001672	Cancer	Neoplasms	PGS000394	MONDO_0001672	bronchus cancer|lung cancer|tracheal cancer	bronchus cancer	MONDO_0001672	Cancer	Neoplasms
0.7395	3.22E-80	3.76E-77	PGS000395	MONDO_0001672	bronchus cancer|lung cancer|tracheal cancer	bronchus cancer	MONDO_0001672	Cancer	Neoplasms	PGS000393	MONDO_0001672	bronchus cancer|lung cancer|tracheal cancer	bronchus cancer	MONDO_0001672	Cancer	Neoplasms
0.7395	3.24E-80	3.79E-77	PGS000500	EFO_0000305	breast carcinoma	breast carcinoma	EFO_0000305	Cancer	Neoplasms	PGS000535	EFO_0000305	breast carcinoma	breast carcinoma	EFO_0000305	Cancer	Neoplasms
0.7394	3.29E-80	3.84E-77	PGS002409	EFO_0004611	low density lipoprotein cholesterol measurement	low density lipoprotein cholesterol measurement	EFO_0004611	Cardiovascular measurement	Cardiovascular	PGS003035	EFO_0004611	low density lipoprotein cholesterol measurement	measurement	EFO_0004611	Lipid or lipoprotein measurement	Metabolic
0.7394	3.29E-80	3.84E-77	PGS002409	EFO_0004611	low density lipoprotein cholesterol measurement	low density lipoprotein cholesterol measurement	EFO_0004611	Lipid or lipoprotein measurement	Metabolic	PGS003035	EFO_0004611	low density lipoprotein cholesterol measurement	measurement	EFO_0004611	Lipid or lipoprotein measurement	Metabolic
0.7394	3.29E-80	3.84E-77	PGS002409	EFO_0004611	low density lipoprotein cholesterol measurement	low density lipoprotein cholesterol measurement	EFO_0004611	Lipid or lipoprotein measurement	Metabolic	PGS003035	EFO_0004611	low density lipoprotein cholesterol measurement	measurement	EFO_0004611	Cardiovascular measurement	Cardiovascular
0.7394	3.29E-80	3.84E-77	PGS002409	EFO_0004611	low density lipoprotein cholesterol measurement	low density lipoprotein cholesterol measurement	EFO_0004611	Cardiovascular measurement	Cardiovascular	PGS003035	EFO_0004611	low density lipoprotein cholesterol measurement	measurement	EFO_0004611	Cardiovascular measurement	Cardiovascular
0.7394	3.29E-80	3.84E-77	PGS001234	EFO_0004324	body weights and measures	body weights and measures	EFO_0004324	Body measurement	Body measurement	PGS002202	EFO_0009805	whole body water mass	whole body water mass	EFO_0009805	Other measurement	Body measurement
0.7394	3.32E-80	3.87E-77	PGS000952	EFO_0004514	bone quantitative ultrasound measurement	bone quantitative ultrasound measurement	EFO_0004514	Other measurement	Skeletal	PGS001404	EFO_0009270	heel bone mineral density	heel bone mineral density	EFO_0009270	Other measurement	Skeletal
0.7394	3.32E-80	3.87E-77	PGS000938	HP_0002619	Varicose veins	Varicose veins	HP_0002619	Other trait	Cardiovascular	PGS000937	HP_0002619	Varicose veins	Varicose veins	HP_0002619	Other trait	Cardiovascular
0.7394	3.35E-80	3.91E-77	PGS000440	MONDO_0002898	skin cancer	skin cancer	MONDO_0002898	Cancer	Neoplasms	PGS000403	EFO_0009259	skin carcinoma	skin carcinoma	EFO_0009259	Cancer	Neoplasms
0.7394	3.45E-80	4.02E-77	PGS002244	EFO_0001645	coronary artery disease	coronary artery disease	EFO_0001645	Cardiovascular disease	Cardiovascular	PGS003355	EFO_0001645	coronary artery disease	coronary artery disease	EFO_0001645	Cardiovascular disease	Cardiovascular
0.7394	3.49E-80	4.07E-77	PGS002206	EFO_0004527	mean corpuscular hemoglobin	mean corpuscular hemoglobin	EFO_0004527	Hematological measurement	Hematological	PGS002558	EFO_0004527	mean corpuscular hemoglobin	mean corpuscular hemoglobin	EFO_0004527	Hematological measurement	Hematological
0.7392	3.78E-80	4.40E-77	PGS000525	EFO_0000305	breast carcinoma	breast carcinoma	EFO_0000305	Cancer	Neoplasms	PGS000540	EFO_0000305	breast carcinoma	breast carcinoma	EFO_0000305	Cancer	Neoplasms
0.7392	3.78E-80	4.40E-77	PGS000540	EFO_0000305	breast carcinoma	breast carcinoma	EFO_0000305	Cancer	Neoplasms	PGS000526	EFO_0000305	breast carcinoma	breast carcinoma	EFO_0000305	Cancer	Neoplasms
0.7392	3.80E-80	4.43E-77	PGS002241	EFO_0001663	prostate carcinoma	prostate carcinoma	EFO_0001663	Cancer	Neoplasms	PGS003383	EFO_0000673	prostate adenocarcinoma	prostate adenocarcinoma	EFO_0000673	Cancer	Neoplasms
0.7392	4.03E-80	4.70E-77	PGS002622	MONDO_0005148	type 2 diabetes mellitus	type 2 diabetes mellitus	MONDO_0005148	Metabolic disorder	Metabolic	PGS002599	EFO_0004541	HbA1c measurement	HbA1c measurement	EFO_0004541	Hematological measurement	Hematological
0.7392	4.03E-80	4.70E-77	PGS002622	MONDO_0005148	type 2 diabetes mellitus	type 2 diabetes mellitus	MONDO_0005148	Digestive system disorder	Gastrointestinal	PGS002599	EFO_0004541	HbA1c measurement	HbA1c measurement	EFO_0004541	Hematological measurement	Hematological
0.7391	4.18E-80	4.87E-77	PGS002970	EFO_0004339	body height	body height	EFO_0004339	Body measurement	Body measurement	PGS002332	EFO_0004339	body height	body height	EFO_0004339	Body measurement	Body measurement
0.7391	4.28E-80	4.99E-77	PGS003069	EFO_0006335	systolic blood pressure	systolic blood pressure	EFO_0006335	Other measurement	Cardiovascular	PGS000900	EFO_0006335	systolic blood pressure|female	systolic blood pressure	EFO_0006335	Other measurement	Cardiovascular
0.7391	4.31E-80	5.01E-77	PGS000824	EFO_0004611	low density lipoprotein cholesterol measurement	low density lipoprotein cholesterol measurement	EFO_0004611	Cardiovascular measurement	Cardiovascular	PGS000892	EFO_0004611	low density lipoprotein cholesterol measurement	measurement	EFO_0004611	Lipid or lipoprotein measurement	Metabolic
0.7391	4.31E-80	5.01E-77	PGS000824	EFO_0004611	low density lipoprotein cholesterol measurement	low density lipoprotein cholesterol measurement	EFO_0004611	Cardiovascular measurement	Cardiovascular	PGS000892	EFO_0004611	low density lipoprotein cholesterol measurement	measurement	EFO_0004611	Cardiovascular measurement	Cardiovascular
0.7391	4.31E-80	5.01E-77	PGS000824	EFO_0004611	low density lipoprotein cholesterol measurement	low density lipoprotein cholesterol measurement	EFO_0004611	Lipid or lipoprotein measurement	Metabolic	PGS000892	EFO_0004611	low density lipoprotein cholesterol measurement	measurement	EFO_0004611	Cardiovascular measurement	Cardiovascular
0.7391	4.31E-80	5.01E-77	PGS000824	EFO_0004611	low density lipoprotein cholesterol measurement	low density lipoprotein cholesterol measurement	EFO_0004611	Lipid or lipoprotein measurement	Metabolic	PGS000892	EFO_0004611	low density lipoprotein cholesterol measurement	measurement	EFO_0004611	Lipid or lipoprotein measurement	Metabolic
-0.7391	4.33E-80	5.04E-77	PGS003219	EFO_0005208	glomerular filtration rate	glomerular filtration rate	EFO_0005208	Other measurement	Genitourinary	PGS003377	EFO_0005208	glomerular filtration rate	glomerular filtration rate	EFO_0005208	Other measurement	Genitourinary
0.7391	4.37E-80	5.08E-77	PGS003149	EFO_0004530	triglyceride measurement	triglyceride measurement	EFO_0004530	Lipid or lipoprotein measurement	Metabolic	PGS003151	EFO_0004530	triglyceride measurement	triglyceride measurement	EFO_0004530	Lipid or lipoprotein measurement	Metabolic
0.739	4.48E-80	5.21E-77	PGS002524	MONDO_0005148	type 2 diabetes mellitus	type 2 diabetes mellitus	MONDO_0005148	Metabolic disorder	Metabolic	PGS002452	EFO_0004541	HbA1c measurement	HbA1c measurement	EFO_0004541	Hematological measurement	Hematological
0.739	4.48E-80	5.21E-77	PGS002524	MONDO_0005148	type 2 diabetes mellitus	type 2 diabetes mellitus	MONDO_0005148	Digestive system disorder	Gastrointestinal	PGS002452	EFO_0004541	HbA1c measurement	HbA1c measurement	EFO_0004541	Hematological measurement	Hematological
0.7389	4.74E-80	5.51E-77	PGS001340	EFO_0000275	atrial fibrillation	atrial fibrillation	EFO_0000275	Cardiovascular disease	Cardiovascular	PGS001841	EFO_0003911	atrial flutter|atrial fibrillation	atrial flutter	EFO_0003911	Cardiovascular disease	Cardiovascular
0.7389	4.82E-80	5.61E-77	PGS003189	EFO_0004631	vitamin D measurement	vitamin D measurement	EFO_0004631	Lipid or lipoprotein measurement	Metabolic	PGS003195	EFO_0004631	vitamin D measurement	vitamin D measurement	EFO_0004631	Lipid or lipoprotein measurement	Metabolic
0.7389	4.92E-80	5.71E-77	PGS001531	EFO_0007772	calcaneal bone quantitative ultrasound measurement	calcaneal bone quantitative ultrasound measurement	EFO_0007772	Other measurement	Skeletal	PGS001037	EFO_0009270	heel bone mineral density	heel bone mineral density	EFO_0009270	Other measurement	Skeletal
0.7388	5.16E-80	6.00E-77	PGS000952	EFO_0004514	bone quantitative ultrasound measurement	bone quantitative ultrasound measurement	EFO_0004514	Other measurement	Skeletal	PGS001531	EFO_0007772	measurement	measurement	EFO_0007772	Other measurement	Skeletal
0.7388	5.18E-80	6.02E-77	PGS002200	EFO_0004631	vitamin D measurement	vitamin D measurement	EFO_0004631	Lipid or lipoprotein measurement	Metabolic	PGS003189	EFO_0004631	vitamin D measurement	vitamin D measurement	EFO_0004631	Lipid or lipoprotein measurement	Metabolic
0.7388	5.30E-80	6.16E-77	PGS002656	EFO_0004527	mean corpuscular hemoglobin	mean corpuscular hemoglobin	EFO_0004527	Hematological measurement	Hematological	PGS001219	EFO_0004527	mean corpuscular hemoglobin	mean corpuscular hemoglobin	EFO_0004527	Hematological measurement	Hematological
0.7388	5.46E-80	6.34E-77	PGS001336	EFO_0000305	breast carcinoma	breast carcinoma	EFO_0000305	Cancer	Neoplasms	PGS000513	EFO_0000305	breast carcinoma	breast carcinoma	EFO_0000305	Cancer	Neoplasms
0.7387	5.57E-80	6.47E-77	PGS002338	EFO_0004587	lymphocyte count	lymphocyte count	EFO_0004587	Hematological measurement	Hematological	PGS002655	EFO_0004587	lymphocyte count	lymphocyte count	EFO_0004587	Hematological measurement	Hematological
0.7387	5.63E-80	6.53E-77	PGS002422	EFO_0004279	suntan	suntan	EFO_0004279	Other trait	Dermatological	PGS002520	EFO_0004279	suntan	suntan	EFO_0004279	Other trait	Dermatological

0.7387	5.69E-80	6.61E-77	PGS003069	EFO_0006335	systolic blood pressure	systolic blood pressure	EFO_0006335	Other measurement	Cardiovascular	PGS002349	EFO_0006335	systolic blood pressure	systolic blood pressure	EFO_0006335	Other measurement	Cardiovascular
0.7386	6.05E-80	7.02E-77	PGS002524	MONDO_0005148	type 2 diabetes mellitus	type 2 diabetes mellitus	MONDO_0005148	Metabolic disorder	Metabolic	PGS002403	EFO_0004541	HbA1c measurement	HbA1c measurement	EFO_0004541	Hematological measurement	Hematological
0.7386	6.05E-80	7.02E-77	PGS002524	MONDO_0005148	type 2 diabetes mellitus	type 2 diabetes mellitus	MONDO_0005148	Digestive system disorder	Gastrointestinal	PGS002403	EFO_0004541	HbA1c measurement	HbA1c measurement	EFO_0004541	Hematological measurement	Hematological
0.7385	6.75E-80	7.83E-77	PGS001950	EFO_0004995	lean body mass	lean body mass	EFO_0004995	Other measurement	Skeletal	PGS001146	EFO_0005409	fat body mass	fat body mass	EFO_0005409	Other measurement	Body measurement
0.7383	7.36E-80	8.54E-77	PGS003152	EFO_0004530	triglyceride measurement	triglyceride measurement	EFO_0004530	Lipid or lipoprotein measurement	Metabolic	PGS002197	EFO_0004530	triglyceride measurement	triglyceride measurement	EFO_0004530	Lipid or lipoprotein measurement	Metabolic
0.7383	7.43E-80	8.62E-77	PGS000540	EFO_0000305	breast carcinoma	breast carcinoma	EFO_0000305	Cancer	Neoplasms	PGS000873	EFO_0000305	breast carcinoma	breast carcinoma	EFO_0000305	Cancer	Neoplasms
0.7383	7.50E-80	8.69E-77	PGS001664	EFO_0006930	brain volume measurement|neuroimaging measurement	brain volume measurement	EFO_0006930	Other measurement	Neurological	PGS001672	EFO_0006930	measurement	brain volume measurement	EFO_0006930	Other measurement	Neurological
0.7383	7.59E-80	8.80E-77	PGS001682	EFO_0006930	brain volume measurement|neuroimaging measurement	brain volume measurement	EFO_0006930	Other measurement	Neurological	PGS001485	EFO_0006930	measurement	brain volume measurement	EFO_0006930	Other measurement	Neurological
0.7383	7.60E-80	8.80E-77	PGS002161	EFO_0004340	body mass index	body mass index	EFO_0004340	Body measurement	Body measurement	PGS001228	EFO_0004340	body mass index	body mass index	EFO_0004340	Body measurement	Body measurement
0.7382	8.48E-80	9.82E-77	PGS001991	EFO_0007989	monocyte percentage of leukocytes	monocyte percentage of leukocytes	EFO_0007989	Hematological measurement	Hematological	PGS002186	EFO_0005091	monocyte count	monocyte count	EFO_0005091	Hematological measurement	Hematological
0.7381	9.06E-80	1.05E-76	PGS001228	EFO_0004340	body mass index	body mass index	EFO_0004340	Body measurement	Body measurement	PGS002849	EFO_0004340	body mass index	body mass index	EFO_0004340	Body measurement	Body measurement
0.738	9.32E-80	1.08E-76	PGS002388	EFO_0000319	cardiovascular disease	cardiovascular disease	EFO_0000319	Cardiovascular disease	Cardiovascular	PGS002584	EFO_0000319	cardiovascular disease	cardiovascular disease	EFO_0000319	Cardiovascular disease	Cardiovascular
0.738	9.41E-80	1.09E-76	PGS001336	EFO_0000305	breast carcinoma	breast carcinoma	EFO_0000305	Cancer	Neoplasms	PGS000472	EFO_0000305	breast carcinoma	breast carcinoma	EFO_0000305	Cancer	Neoplasms
0.738	9.85E-80	1.14E-76	PGS002453	EFO_0004339	body height	body height	EFO_0004339	Body measurement	Body measurement	PGS002973	EFO_0004339	body height	body height	EFO_0004339	Body measurement	Body measurement
0.7379	1.03E-79	1.20E-76	PGS001681	EFO_0006930	brain volume measurement|neuroimaging measurement	brain volume measurement	EFO_0006930	Other measurement	Neurological	PGS001669	EFO_0006930	measurement	brain volume measurement	EFO_0006930	Other measurement	Neurological
0.7377	1.15E-79	1.33E-76	PGS001160	EFO_0005106	body composition measurement	body composition measurement	EFO_0005106	Body measurement	Body measurement	PGS001230	EFO_0004338	body weight	body weight	EFO_0004338	Body measurement	Body measurement
0.7377	1.16E-79	1.34E-76	PGS002780	MONDO_0005148	type 2 diabetes mellitus	type 2 diabetes mellitus	MONDO_0005148	Metabolic disorder	Metabolic	PGS002243	MONDO_0005148	type 2 diabetes mellitus	type 2 diabetes mellitus	MONDO_0005148	Metabolic disorder	Metabolic
0.7377	1.16E-79	1.34E-76	PGS002780	MONDO_0005148	type 2 diabetes mellitus	type 2 diabetes mellitus	MONDO_0005148	Metabolic disorder	Metabolic	PGS002243	MONDO_0005148	type 2 diabetes mellitus	type 2 diabetes mellitus	MONDO_0005148	Digestive system disorder	Gastrointestinal
0.7377	1.16E-79	1.34E-76	PGS002780	MONDO_0005148	type 2 diabetes mellitus	type 2 diabetes mellitus	MONDO_0005148	Digestive system disorder	Gastrointestinal	PGS002243	MONDO_0005148	type 2 diabetes mellitus	type 2 diabetes mellitus	MONDO_0005148	Metabolic disorder	Metabolic
0.7377	1.16E-79	1.34E-76	PGS002780	MONDO_0005148	type 2 diabetes mellitus	type 2 diabetes mellitus	MONDO_0005148	Digestive system disorder	Gastrointestinal	PGS002243	MONDO_0005148	type 2 diabetes mellitus	type 2 diabetes mellitus	MONDO_0005148	Digestive system disorder	Gastrointestinal
0.7377	1.19E-79	1.37E-76	PGS002409	EFO_0004611	low density lipoprotein cholesterol measurement	low density lipoprotein cholesterol measurement	EFO_0004611	Cardiovascular measurement	Cardiovascular	PGS003030	EFO_0004611	low density lipoprotein cholesterol measurement	measurement	EFO_0004611	Cardiovascular measurement	Cardiovascular
0.7377	1.19E-79	1.37E-76	PGS002409	EFO_0004611	low density lipoprotein cholesterol measurement	low density lipoprotein cholesterol measurement	EFO_0004611	Lipid or lipoprotein measurement	Metabolic	PGS003030	EFO_0004611	low density lipoprotein cholesterol measurement	measurement	EFO_0004611	Lipid or lipoprotein measurement	Metabolic
0.7377	1.19E-79	1.37E-76	PGS002409	EFO_0004611	low density lipoprotein cholesterol measurement	low density lipoprotein cholesterol measurement	EFO_0004611	Cardiovascular measurement	Cardiovascular	PGS003030	EFO_0004611	low density lipoprotein cholesterol measurement	measurement	EFO_0004611	Lipid or lipoprotein measurement	Metabolic
0.7377	1.19E-79	1.37E-76	PGS002409	EFO_0004611	low density lipoprotein cholesterol measurement	low density lipoprotein cholesterol measurement	EFO_0004611	Lipid or lipoprotein measurement	Metabolic	PGS003030	EFO_0004611	low density lipoprotein cholesterol measurement	measurement	EFO_0004611	Cardiovascular measurement	Cardiovascular
0.7375	1.35E-79	1.56E-76	PGS000783	EFO_0000305	breast carcinoma	breast carcinoma	EFO_0000305	Cancer	Neoplasms	PGS000345	EFO_0000305	breast carcinoma	breast carcinoma	EFO_0000305	Cancer	Neoplasms
0.7375	1.40E-79	1.62E-76	PGS002993	EFO_0004339	body height	body height	EFO_0004339	Body measurement	Body measurement	PGS002332	EFO_0004339	body height	body height	EFO_0004339	Body measurement	Body measurement
0.7374	1.44E-79	1.66E-76	PGS001900	EFO_0006336	diastolic blood pressure	diastolic blood pressure	EFO_0006336	Other measurement	Cardiovascular	PGS002889	EFO_0006336	diastolic blood pressure	diastolic blood pressure	EFO_0006336	Other measurement	Cardiovascular
0.7374	1.44E-79	1.67E-76	PGS002335	EFO_0000537	hypertension	hypertension	EFO_0000537	Cardiovascular disease	Cardiovascular	PGS001335	EFO_0000319	cardiovascular disease	cardiovascular disease	EFO_0000319	Cardiovascular disease	Cardiovascular
0.7374	1.49E-79	1.72E-76	PGS002141	EFO_0004348	hematocrit	hematocrit	EFO_0004348	Hematological measurement	Hematological	PGS000092	EFO_0004348	hematocrit	hematocrit	EFO_0004348	Hematological measurement	Hematological
0.7373	1.63E-79	1.89E-76	PGS000002	EFO_1000649	estrogen-receptor positive breast cancer	estrogen-receptor positive breast cancer	EFO_1000649	Cancer	Neoplasms	PGS000052	EFO_0000305	breast carcinoma	breast carcinoma	EFO_0000305	Cancer	Neoplasms
0.7372	1.74E-79	2.01E-76	PGS002396	EFO_0001379	endocrine system disease|diabetes mellitus	endocrine system disease	EFO_0001379	Other disease	Metabolic	PGS002599	EFO_0004541	HbA1c measurement	HbA1c measurement	EFO_0004541	Hematological measurement	Hematological
0.7372	1.77E-79	2.04E-76	PGS000539	EFO_0000305	breast carcinoma	breast carcinoma	EFO_0000305	Cancer	Neoplasms	PGS000538	EFO_0000305	breast carcinoma	breast carcinoma	EFO_0000305	Cancer	Neoplasms
0.7371	1.79E-79	2.06E-76	PGS001682	EFO_0006930	brain volume measurement|neuroimaging measurement	brain volume measurement	EFO_0006930	Other measurement	Neurological	PGS001670	EFO_0006930	measurement	brain volume measurement	EFO_0006930	Other measurement	Neurological
0.7371	1.80E-79	2.08E-76	PGS001230	EFO_0004338	body weight	body weight	EFO_0004338	Body measurement	Body measurement	PGS001168	EFO_0004995	lean body mass	lean body mass	EFO_0004995	Other measurement	Skeletal
0.7371	1.81E-79	2.08E-76	PGS003147	EFO_0004530	triglyceride measurement	triglyceride measurement	EFO_0004530	Lipid or lipoprotein measurement	Metabolic	PGS001979	EFO_0004530	triglyceride measurement	triglyceride measurement	EFO_0004530	Lipid or lipoprotein measurement	Metabolic
0.7371	1.81E-79	2.09E-76	PGS002387	EFO_0003923	bone density	bone density	EFO_0003923	Other measurement	Skeletal	PGS002485	EFO_0003923	bone density	bone density	EFO_0003923	Other measurement	Skeletal
0.7371	1.84E-79	2.12E-76	PGS001847	EFO_0004264	vascular disease	vascular disease	EFO_0004264	Cardiovascular disease	Cardiovascular	PGS000931	MONDO_0000831	thrombotic disease|deep vein thrombosis	thrombotic disease	MONDO_0000831	Cardiovascular disease	Cardiovascular
0.7371	1.85E-79	2.13E-76	PGS003145	EFO_0004530	triglyceride measurement	triglyceride measurement	EFO_0004530	Lipid or lipoprotein measurement	Metabolic	PGS003149	EFO_0004530	triglyceride measurement	triglyceride measurement	EFO_0004530	Lipid or lipoprotein measurement	Metabolic
0.7371	1.87E-79	2.15E-76	PGS001142	EFO_0005420	grey matter volume measurement	grey matter volume measurement	EFO_0005420	Other measurement	Neurological	PGS001571	EFO_0005420	measurement	grey matter volume measurement	EFO_0005420	Other measurement	Neurological
0.7371	1.87E-79	2.16E-76	PGS001682	EFO_0006930	brain volume measurement|neuroimaging measurement	brain volume measurement	EFO_0006930	Other measurement	Neurological	PGS001672	EFO_0006930	measurement	brain volume measurement	EFO_0006930	Other measurement	Neurological
0.7369	2.10E-79	2.41E-76	PGS002161	EFO_0004340	body mass index	body mass index	EFO_0004340	Body measurement	Body measurement	PGS002630	EFO_0004340	body mass index	body mass index	EFO_0004340	Body measurement	Body measurement
0.7369	2.19E-79	2.52E-76	PGS003060	EFO_0006527	smoking status measurement	smoking status measurement	EFO_0006527	Other measurement	Psychiatric	PGS003061	EFO_0006527	smoking status measurement	smoking status measurement	EFO_0006527	Other measurement	Psychiatric
0.7368	2.37E-79	2.73E-76	PGS002851	EFO_0004340	body mass index	body mass index	EFO_0004340	Body measurement	Body measurement	PGS002850	EFO_0004340	body mass index	body mass index	EFO_0004340	Body measurement	Body measurement
0.7367	2.48E-79	2.85E-76	PGS002313	EFO_0004340	body mass index	body mass index	EFO_0004340	Body measurement	Body measurement	PGS002630	EFO_0004340	body mass index	body mass index	EFO_0004340	Body measurement	Body measurement
0.7366	2.58E-79	2.97E-76	PGS000365	MONDO_0007576	esophageal cancer	esophageal cancer	MONDO_0007576	Cancer	Neoplasms	PGS000363	MONDO_0007576	esophageal cancer	esophageal cancer	MONDO_0007576	Digestive system disorder	Gastrointestinal
0.7366	2.58E-79	2.97E-76	PGS000365	MONDO_0007576	esophageal cancer	esophageal cancer	MONDO_0007576	Digestive system disorder	Gastrointestinal	PGS000363	MONDO_0007576	esophageal cancer	esophageal cancer	MONDO_0007576	Cancer	Neoplasms
0.7366	2.58E-79	2.97E-76	PGS000365	MONDO_0007576	esophageal cancer	esophageal cancer	MONDO_0007576	Cancer	Neoplasms	PGS000363	MONDO_0007576	esophageal cancer	esophageal cancer	MONDO_0007576	Cancer	Neoplasms
0.7366	2.58E-79	2.97E-76	PGS000365	MONDO_0007576	esophageal cancer	esophageal cancer	MONDO_0007576	Digestive system disorder	Gastrointestinal	PGS000363	MONDO_0007576	esophageal cancer	esophageal cancer	MONDO_0007576	Digestive system disorder	Gastrointestinal
0.7366	2.69E-79	3.09E-76	PGS000540	EFO_0000305	breast carcinoma	breast carcinoma	EFO_0000305	Cancer	Neoplasms	PGS001804	EFO_0000305	breast carcinoma	breast carcinoma	EFO_0000305	Cancer	Neoplasms
0.7365	2.83E-79	3.25E-76	PGS003200	EFO_0004631	vitamin D measurement	vitamin D measurement	EFO_0004631	Lipid or lipoprotein measurement	Metabolic	PGS003195	EFO_0004631	vitamin D measurement	vitamin D measurement	EFO_0004631	Lipid or lipoprotein measurement	Metabolic
0.7365	2.88E-79	3.31E-76	PGS000098	EFO_0007993	lymphocyte percentage of leukocytes	lymphocyte percentage of leukocytes	EFO_0007993	Hematological measurement	Hematological	PGS002203	EFO_0007993	lymphocyte percentage of leukocytes	lymphocyte percentage of leukocytes	EFO_0007993	Hematological measurement	Hematological
0.7365	2.88E-79	3.31E-76	PGS002775	EFO_0001645	coronary artery disease	coronary artery disease	EFO_0001645	Cardiovascular disease	Cardiovascular	PGS000746	EFO_0001645	coronary artery disease	coronary artery disease	EFO_0001645	Cardiovascular disease	Cardiovascular
0.7365	2.91E-79	3.34E-76	PGS003030	EFO_0004611	low density lipoprotein cholesterol measurement	low density lipoprotein cholesterol measurement	EFO_0004611	Cardiovascular measurement	Cardiovascular	PGS002571	EFO_0004574	total cholesterol measurement	total cholesterol measurement	EFO_0004574	Lipid or lipoprotein measurement	Metabolic
0.7365	2.91E-79	3.34E-76	PGS003030	EFO_0004611	low density lipoprotein cholesterol measurement	low density lipoprotein cholesterol measurement	EFO_0004611	Lipid or lipoprotein measurement	Metabolic	PGS002571	EFO_0004574	total cholesterol measurement	total cholesterol measurement	EFO_0004574	Lipid or lipoprotein measurement	Metabolic
0.7365	2.94E-79	3.38E-76	PGS000783	EFO_0000305	breast carcinoma	breast carcinoma	EFO_0000305	Cancer	Neoplasms	PGS003399	EFO_0000305	breast carcinoma	breast carcinoma	EFO_0000305	Cancer	Neoplasms
0.7365	2.97E-79	3.41E-76	PGS001235	EFO_0004324	body weights and measures	body weights and measures	EFO_0004324	Body measurement	Body measurement	PGS002202	EFO_0009805	whole body water mass	whole body water mass	EFO_0009805	Other measurement	Body measurement
0.7363	3.21E-79	3.68E-76	PGS001402	EFO_0009270	heel bone mineral density	heel bone mineral density	EFO_0009270	Other measurement	Skeletal	PGS001404	EFO_0009270	heel bone mineral density	heel bone mineral density	EFO_0009270	Other measurement	Skeletal
0.7363	3.21E-79	3.68E-76	PGS002409	EFO_0004611	low density lipoprotein cholesterol measurement	low density lipoprotein cholesterol measurement	EFO_0004611	Cardiovascular measurement	Cardiovascular	PGS003031	EFO_0004611	low density lipoprotein cholesterol measurement	measurement	EFO_0004611	Cardiovascular measurement	Cardiovascular
0.7363	3.21E-79	3.68E-76	PGS002409	EFO_0004611	low density lipoprotein cholesterol measurement	low density lipoprotein cholesterol measurement	EFO_0004611	Cardiovascular measurement	Cardiovascular	PGS003031	EFO_0004611	low density lipoprotein cholesterol measurement	measurement	EFO_0004611	Lipid or lipoprotein measurement	Metabolic
0.7363	3.21E-79	3.68E-76	PGS002409	EFO_0004611	low density lipoprotein cholesterol measurement	low density lipoprotein cholesterol measurement	EFO_0004611	Lipid or lipoprotein measurement	Metabolic	PGS003031	EFO_0004611	low density lipoprotein cholesterol measurement	measurement	EFO_0004611	Lipid or lipoprotein measurement	Metabolic
0.7363	3.21E-79	3.68E-76	PGS002409	EFO_0004611	low density lipoprotein cholesterol measurement	low density lipoprotein cholesterol measurement	EFO_0004611	Lipid or lipoprotein measurement	Metabolic	PGS003031	EFO_0004611	low density lipoprotein cholesterol measurement	measurement	EFO_0004611	Cardiovascular measurement	Cardiovascular
0.7363	3.23E-79	3.71E-76	PGS002894	EFO_0006336	diastolic blood pressure	diastolic blood pressure	EFO_0006336	Other measurement	Cardiovascular	PGS001900	EFO_0006336	diastolic blood pressure	diastolic blood pressure	EFO_0006336	Other measurement	Cardiovascular
0.7363	3.37E-79	3.87E-76	PGS001531	EFO_0007772	calcaneal bone quantitative ultrasound measurement	calcaneal bone quantitative ultrasound measurement	EFO_0007772	Other measurement	Skeletal	PGS001402	EFO_0009270	heel bone mineral density	heel bone mineral density	EFO_0009270	Other measurement	Skeletal
0.7363	3.38E-79	3.87E-76	PGS002163	EFO_0004518	creatinine measurement	creatinine measurement	EFO_0004518	Other measurement	Metabolic	PGS003239	EFO_0010472	creatine measurement	creatine measurement	EFO_0010472	Other measurement	Metabolic
0.7363	3.45E-79	3.95E-76	PGS000509	EFO_0000305	breast carcinoma	breast carcinoma	EFO_0000305	Cancer	Neoplasms	PGS000512	EFO_0000305	breast carcinoma	breast carcinoma	EFO_0000305	Cancer	Neoplasms
0.7362	3.50E-79	4.01E-76	PGS001292	EFO_0600083	family history of prostate cancer	family history of prostate cancer	EFO_0600083	Other trait	Neoplasms	PGS001291	EFO_0001663	prostate carcinoma	prostate carcinoma	EFO_0001663	Cancer	Neoplasms
0.7362	3.51E-79	4.02E-76	PGS001738	EFO_0006930	brain volume measurement|neuroimaging measurement	brain volume measurement	EFO_0006930	Other measurement	Neurological	PGS001735	EFO_0006930	measurement	brain volume measurement	EFO_0006930	Other measurement	Neurological
0.7362	3.67E-79	4.20E-76	PGS002656	EFO_0004527	mean corpuscular hemoglobin	mean corpuscular hemoglobin	EFO_0004527	Hematological measurement	Hematological	PGS002207	EFO_0004526	mean corpuscular volume	mean corpuscular volume	EFO_0004526	Hematological measurement	Hematological
0.7362	3.68E-79	4.21E-76	PGS000509	EFO_0000305	breast carcinoma	breast carcinoma	EFO_0000305	Cancer	Neoplasms	PGS000505	EFO_0000305	breast carcinoma	breast carcinoma	EFO_0000305	Cancer	Neoplasms
0.7362	3.68E-79	4.21E-76	PGS000506	EFO_0000305	breast carcinoma	breast carcinoma	EFO_0000305	Cancer	Neoplasms	PGS000509	EFO_0000305	breast carcinoma	breast carcinoma	EFO_0000305	Cancer	Neoplasms
0.7361	3.86E-79	4.42E-76	PGS002202	EFO_0009805	whole body water mass	whole body water mass	EFO_0009805	Other measurement	Body measurement	PGS001159	EFO_0005106	body composition measurement	body composition measurement	EFO_0005106	Body measurement	Body measurement
0.7361	3.87E-79	4.43E-76	PGS001478	EFO_0006930	brain volume measurement|neuroimaging measurement	brain volume measurement	EFO_0006930	Other measurement	Neurological	PGS001682	EFO_0006930	measurement	brain volume measurement	EFO_0006930	Other measurement	Neurological
0.7361	3.95E-79	4.52E-76	PGS002983	EFO_0004339	body height	body height	EFO_0004339	Body measurement	Body measurement	PGS002332	EFO_0004339	body height	body height	EFO_0004339	Body measurement	Body measurement
0.736	4.01E-79	4.59E-76	PGS001484	EFO_0006930	brain volume measurement|neuroimaging measurement	brain volume measurement	EFO_0006930	Other measurement	Neurological	PGS001683	EFO_0006930	measurement	brain volume measurement	EFO_0006930	Other measurement	Neurological
0.736	4.01E-79	4.59E-76	PGS000477	EFO_0000305	breast carcinoma	breast carcinoma	EFO_0000305	Cancer	Neoplasms	PGS000473	EFO_0000305	breast carcinoma	breast carcinoma	EFO_0000305	Cancer	Neoplasms
0.736	4.14E-79	4.73E-76	PGS001479	EFO_0006930	brain volume measurement|neuroimaging measurement	brain volume measurement	EFO_0006930	Other measurement	Neurological	PGS001682	EFO_0006930	measurement	brain volume measurement	EFO_0006930	Other measurement	Neurological
0.736	4.18E-79	4.78E-76	PGS001485	EFO_0006930	brain volume measurement|neuroimaging measurement	brain volume measurement	EFO_0006930	Other measurement	Neurological	PGS001472	EFO_0006930	measurement	brain volume measurement	EFO_0006930	Other measurement	Neurological
0.736	4.23E-79	4.83E-76	PGS001674	EFO_0006930	brain volume measurement|neuroimaging measurement	brain volume measurement	EFO_0006930	Other measurement	Neurological	PGS001670	EFO_0006930	measurement	brain volume measurement	EFO_0006930	Other measurement	Neurological
0.7359	4.57E-79	5.23E-76	PGS003184	EFO_0004631	vitamin D measurement	vitamin D measurement	EFO_0004631	Lipid or lipoprotein measurement	Metabolic	PGS002355	EFO_0004631	vitamin D measurement	vitamin D measurement	EFO_0004631	Lipid or lipoprotein measurement	Metabolic
0.7359	4.59E-79	5.24E-76	PGS001229	EFO_0004339	body height	body height	EFO_0004339	Body measurement	Body measurement	PGS002964	EFO_0004339	body height	body height	EFO_0004339	Body measurement	Body measurement
0.7358	4.65E-79	5.31E-76	PGS000001	EFO_0000305	breast carcinoma	breast carcinoma	EFO_0000305	Cancer	Neoplasms	PGS000533	EFO_0000305	breast carcinoma	breast carcinoma	EFO_0000305	Cancer	Neoplasms
0.7358	4.65E-79	5.31E-76	PGS000534	EFO_0000305	breast carcinoma	breast carcinoma	EFO_0000305	Cancer	Neoplasms	PGS000001	EFO_0000305	breast carcinoma	breast carcinoma	EFO_0000305	Cancer	Neoplasms
0.7358	4.74E-79	5.41E-76	PGS000572	EFO_0001663	prostate carcinoma	prostate carcinoma	EFO_0001663	Cancer	Neoplasms	PGS001805	EFO_0001663	prostate carcinoma	prostate carcinoma	EFO_0001663	Cancer	Neoplasms
0.7358	4.82E-79	5.50E-76	PGS000530	EFO_0000305	breast carcinoma	breast carcinoma	EFO_0000305	Cancer	Neoplasms	PGS000540	EFO_0000305	breast carcinoma	breast carcinoma	EFO_0000305	Cancer	Neoplasms
0.7358	4.89E-79	5.58E-76	PGS001737	EFO_0006930	brain volume measurement|neuroimaging measurement	brain volume measurement	EFO_0006930	Other measurement	Neurological	PGS001736	EFO_0006930	measurement	brain volume measurement	EFO_0006930	Other measurement	Neurological
0.7358	4.89E-79	5.58E-76	PGS002550	EFO_0004541	HbA1c measurement	HbA1c measurement	EFO_0004541	Hematological measurement	Hematological	PGS002475	MONDO_0005148	type 2 diabetes mellitus	type 2 diabetes mellitus	MONDO_0005148	Digestive system disorder	Gastrointestinal
0.7358	4.89E-79	5.58E-76	PGS002550	EFO_0004541	HbA1c measurement	HbA1c measurement	EFO_0004541	Hematological measurement	Hematological	PGS002475	MONDO_0005148	type 2 diabetes mellitus	type 2 diabetes mellitus	MONDO_0005148	Metabolic disorder	Metabolic
0.7358	4.92E-79	5.60E-76	PGS001228	EFO_0004340	body mass index	body mass index	EFO_0004340	Body measurement	Body measurement	PGS002313	EFO_0004340	body mass index	body mass index	EFO_0004340	Body measurement	Body measurement
0.7358	4.97E-79	5.66E-76	PGS002337	EFO_0004611	low density lipoprotein cholesterol measurement	low density lipoprotein cholesterol measurement	EFO_0004611	Cardiovascular measurement	Cardiovascular	PGS002556	EFO_0004611	low density lipoprotein cholesterol measurement	measurement	EFO_0004611	Cardiovascular measurement	Cardiovascular
0.7358	4.97E-79	5.66E-76	PGS002337	EFO_0004611	low density lipoprotein cholesterol measurement	low density lipoprotein cholesterol measurement	EFO_0004611	Lipid or lipoprotein measurement	Metabolic	PGS002556	EFO_0004611	low density lipoprotein cholesterol measurement	measurement	EFO_0004611	Lipid or lipoprotein measurement	Metabolic
0.7358	4.97E-79	5.66E-76	PGS002337	EFO_0004611	low density lipoprotein cholesterol measurement	low density lipoprotein cholesterol measurement	EFO_0004611	Lipid or lipoprotein measurement	Metabolic	PGS002556	EFO_0004611	low density lipoprotein cholesterol measurement	measurement	EFO_0004611	Cardiovascular measurement	Cardiovascular
0.7358	4.97E-79	5.66E-76	PGS002337	EFO_0004611	low density lipoprotein cholesterol measurement	low density lipoprotein cholesterol measurement	EFO_0004611	Cardiovascular measurement	Cardiovascular	PGS002556	EFO_0004611	low density lipoprotein cholesterol measurement	measurement	EFO_0004611	Lipid or lipoprotein measurement	Metabolic
-0.7357	5.10E-79	5.82E-76	PGS002583	EFO_0003923	bone density	bone density	EFO_0003923	Other measurement	Skeletal	PGS002768	EFO_0003882	osteoporosis	osteoporosis	EFO_0003882	Other disease	Skeletal
0.7357	5.31E-79	6.05E-76	PGS002042	MONDO_0018102	corneal dystrophy	corneal dystrophy	MONDO_0018102	Other disease	Ophthalmological	PGS001029	EFO_0009464	corneal disease	corneal disease	EFO_0009464	Other disease	Ophthalmological
0.7357	5.32E-79	6.06E-76	PGS000539	EFO_0000305	breast carcinoma	breast carcinoma	EFO_0000305	Cancer	Neoplasms	PGS000479	EFO_0000305	breast carcinoma	breast carcinoma	EFO_0000305	Cancer	Neoplasms
0.7356	5.45E-79	6.21E-76	PGS003199	EFO_0004631	vitamin D measurement	vitamin D measurement	EFO_0004631	Lipid or lipoprotein measurement	Metabolic	PGS002355	EFO_0004631	vitamin D measurement	vitamin D measurement	EFO_0004631	Lipid or lipoprotein measurement	Metabolic
0.7356	5.54E-79	6.30E-76	PGS000421	EFO_0000756	melanoma	melanoma	EFO_0000756	Cancer	Neoplasms	PGS000079	EFO_0000756	melanoma	melanoma	EFO_0000756	Cancer	Neoplasms
0.7356	5.54E-79	6.30E-76	PGS003099	MONDO_0005148	type 2 diabetes mellitus	type 2 diabetes mellitus	MONDO_0005148	Metabolic disorder	Metabolic	PGS002771	MONDO_0005148	type 2 diabetes mellitus	type 2 diabetes mellitus	MONDO_0005148	Digestive system disorder	Gastrointestinal
0.7356	5.54E-79	6.30E-76	PGS000422	EFO_0000756	melanoma	melanoma	EFO_0000756	Cancer	Neoplasms	PGS000079	EFO_0000756	melanoma	melanoma	EFO_0000756	Cancer	Neoplasms
0.7356	5.54E-79	6.30E-76	PGS003099	MONDO_0005148	type 2 diabetes mellitus	type 2 diabetes mellitus	MONDO_0005148	Digestive system disorder	Gastrointestinal	PGS002771	MONDO_0005148	type 2 diabetes mellitus	type 2 diabetes mellitus	MONDO_0005148	Digestive system disorder	Gastrointestinal
0.7356	5.54E-79	6.30E-76	PGS003099	MONDO_0005148	type 2 diabetes mellitus	type 2 diabetes mellitus	MONDO_0005148	Metabolic disorder	Metabolic	PGS002771	MONDO_0005148	type 2 diabetes mellitus	type 2 diabetes mellitus	MONDO_0005148	Metabolic disorder	Metabolic
0.7356	5.54E-79	6.30E-76	PGS003099	MONDO_0005148	type 2 diabetes mellitus	type 2 diabetes mellitus	MONDO_0005148	Digestive system disorder	Gastrointestinal	PGS002771	MONDO_0005148	type 2 diabetes mellitus	type 2 diabetes mellitus	MONDO_0005148	Metabolic disorder	Metabolic
0.7356	5.61E-79	6.38E-76	PGS000893	EFO_0004611	low density lipoprotein cholesterol measurement	low density lipoprotein cholesterol measurement	EFO_0004611	Cardiovascular measurement	Cardiovascular	PGS002783	EFO_0004574	total cholesterol measurement	total cholesterol measurement	EFO_0004574	Lipid or lipoprotein measurement	Metabolic

0.7356	5.61E-79	6.38E-76	PGS000893	EFO_0004611	low density lipoprotein cholesterol measurement	low density lipoprotein cholesterol measurement	EFO_0004611	Lipid or lipoprotein measurement	Metabolic	PGS002783	EFO_0004574	total cholesterol measurement	total cholesterol measurement	EFO_0004574	Lipid or lipoprotein measurement	Metabolic
0.7355	5.89E-79	6.70E-76	PGS001167	EFO_0004995	lean body mass	lean body mass	EFO_0004995	Other measurement	Skeletal	PGS002202	EFO_0009805	whole body water mass	whole body water mass	EFO_0009805	Other measurement	Body measurement
0.7355	6.17E-79	7.01E-76	PGS000873	EFO_0000305	breast carcinoma	breast carcinoma	EFO_0000305	Cancer	Neoplasms	PGS000004	EFO_0000305	breast carcinoma	breast carcinoma	EFO_0000305	Cancer	Neoplasms
0.7354	6.27E-79	7.13E-76	PGS001234	EFO_0004324	body weights and measures	body weights and measures	EFO_0004324	Body measurement	Body measurement	PGS002168	EFO_0004995	lean body mass	lean body mass	EFO_0004995	Other measurement	Skeletal
0.7354	6.32E-79	7.19E-76	PGS000956	EFO_0004514	bone quantitative ultrasound measurement	bone quantitative ultrasound measurement	EFO_0004514	Other measurement	Skeletal	PGS001037	EFO_0009270	heel bone mineral density	heel bone mineral density	EFO_0009270	Other measurement	Skeletal
0.7354	6.37E-79	7.24E-76	PGS001039	EFO_0009270	heel bone mineral density	heel bone mineral density	EFO_0009270	Other measurement	Skeletal	PGS001037	EFO_0009270	heel bone mineral density	heel bone mineral density	EFO_0009270	Other measurement	Skeletal
0.7354	6.40E-79	7.27E-76	PGS001173	EFO_0004833	neutrophil count	neutrophil count	EFO_0004833	Hematological measurement	Hematological	PGS001969	EFO_0004833	neutrophil count	neutrophil count	EFO_0004833	Hematological measurement	Hematological
0.7353	6.68E-79	7.59E-76	PGS000952	EFO_0004514	bone quantitative ultrasound measurement	bone quantitative ultrasound measurement	EFO_0004514	Other measurement	Skeletal	PGS000956	EFO_0004514	bone quantitative ultrasound measurement	measurement	EFO_0004514	Other measurement	Skeletal
0.7353	6.73E-79	7.64E-76	PGS000952	EFO_0004514	bone quantitative ultrasound measurement	bone quantitative ultrasound measurement	EFO_0004514	Other measurement	Skeletal	PGS001039	EFO_0009270	heel bone mineral density	heel bone mineral density	EFO_0009270	Other measurement	Skeletal
0.7352	7.41E-79	8.41E-76	PGS003186	EFO_0004631	vitamin D measurement	vitamin D measurement	EFO_0004631	Lipid or lipoprotein measurement	Metabolic	PGS003195	EFO_0004631	vitamin D measurement	vitamin D measurement	EFO_0004631	Lipid or lipoprotein measurement	Metabolic
0.7352	7.47E-79	8.48E-76	PGS001746	EFO_0006930	brain volume measurement|neuroimaging measurement	brain volume measurement	EFO_0006930	Other measurement	Neurological	PGS001747	EFO_0006930	measurement	brain volume measurement	EFO_0006930	Other measurement	Neurological
0.7352	7.51E-79	8.53E-76	PGS001492	EFO_0006930	brain volume measurement|neuroimaging measurement	brain volume measurement	EFO_0006930	Other measurement	Neurological	PGS001686	EFO_0006930	measurement	brain volume measurement	EFO_0006930	Other measurement	Neurological
0.7352	7.57E-79	8.59E-76	PGS002170	EFO_0004468	glucose measurement	glucose measurement	EFO_0004468	Other measurement	Metabolic	PGS002934	EFO_0004468	glucose measurement	glucose measurement	EFO_0004468	Other measurement	Metabolic
0.7352	7.61E-79	8.63E-76	PGS002354	MONDO_0005148	type 2 diabetes mellitus	type 2 diabetes mellitus	MONDO_0005148	Metabolic disorder	Metabolic	PGS002638	EFO_0000400	diabetes mellitus	diabetes mellitus	EFO_0000400	Digestive system disorder	Gastrointestinal
0.7352	7.61E-79	8.63E-76	PGS002354	MONDO_0005148	type 2 diabetes mellitus	type 2 diabetes mellitus	MONDO_0005148	Metabolic disorder	Metabolic	PGS002638	EFO_0000400	diabetes mellitus	diabetes mellitus	EFO_0000400	Metabolic disorder	Metabolic
0.7352	7.61E-79	8.63E-76	PGS002354	MONDO_0005148	type 2 diabetes mellitus	type 2 diabetes mellitus	MONDO_0005148	Digestive system disorder	Gastrointestinal	PGS002638	EFO_0000400	diabetes mellitus	diabetes mellitus	EFO_0000400	Digestive system disorder	Gastrointestinal
0.7352	7.61E-79	8.63E-76	PGS002354	MONDO_0005148	type 2 diabetes mellitus	type 2 diabetes mellitus	MONDO_0005148	Digestive system disorder	Gastrointestinal	PGS002638	EFO_0000400	diabetes mellitus	diabetes mellitus	EFO_0000400	Metabolic disorder	Metabolic
0.7352	7.67E-79	8.70E-76	PGS002458	EFO_0004611	low density lipoprotein cholesterol measurement	low density lipoprotein cholesterol measurement	EFO_0004611	Cardiovascular measurement	Cardiovascular	PGS002620	EFO_0004574	total cholesterol measurement	total cholesterol measurement	EFO_0004574	Lipid or lipoprotein measurement	Metabolic
0.7352	7.67E-79	8.70E-76	PGS002458	EFO_0004611	low density lipoprotein cholesterol measurement	low density lipoprotein cholesterol measurement	EFO_0004611	Lipid or lipoprotein measurement	Metabolic	PGS002620	EFO_0004574	total cholesterol measurement	total cholesterol measurement	EFO_0004574	Lipid or lipoprotein measurement	Metabolic
0.7352	7.70E-79	8.73E-76	PGS002311	MONDO_0004979	asthma	asthma	MONDO_0004979	Other disease	Respiratory	PGS001341	MONDO_0004979	asthma	asthma	MONDO_0004979	Other disease	Respiratory
0.7351	7.74E-79	8.77E-76	PGS002840	EFO_0004340	body mass index	body mass index	EFO_0004340	Body measurement	Body measurement	PGS002841	EFO_0004340	body mass index	body mass index	EFO_0004340	Body measurement	Body measurement
0.7351	7.77E-79	8.80E-76	PGS002123	EFO_0004305	erythrocyte count	erythrocyte count	EFO_0004305	Hematological measurement	Hematological	PGS002662	EFO_0004305	erythrocyte count	erythrocyte count	EFO_0004305	Hematological measurement	Hematological
0.7351	7.85E-79	8.90E-76	PGS002865	EFO_0004458	C-reactive protein measurement	C-reactive protein measurement	EFO_0004458	Inflammatory measurement	Immunological	PGS002868	EFO_0004458	C-reactive protein measurement	C-reactive protein measurement	EFO_0004458	Inflammatory measurement	Immunological
0.7351	8.07E-79	9.13E-76	PGS002465	EFO_0000676	psoriasis	psoriasis	EFO_0000676	Immune system disorder	Immunological	PGS002482	EFO_0005140	autoimmune disease	autoimmune disease	EFO_0005140	Immune system disorder	Immunological
0.735	8.72E-79	9.87E-76	PGS001922	EFO_0004312	vital capacity	vital capacity	EFO_0004312	Other measurement	Activities	PGS001237	EFO_0004312	vital capacity	vital capacity	EFO_0004312	Other measurement	Activities
0.7348	1.01E-78	1.14E-75	PGS003201	EFO_0004631	vitamin D measurement	vitamin D measurement	EFO_0004631	Lipid or lipoprotein measurement	Metabolic	PGS002672	EFO_0004631	vitamin D measurement	vitamin D measurement	EFO_0004631	Lipid or lipoprotein measurement	Metabolic
0.7347	1.04E-78	1.18E-75	PGS001951	EFO_0005409	fat body mass	fat body mass	EFO_0005409	Other measurement	Body measurement	PGS001148	EFO_0005409	fat body mass	fat body mass	EFO_0005409	Other measurement	Body measurement
0.7347	1.05E-78	1.19E-75	PGS001344	MONDO_0004979	asthma	asthma	MONDO_0004979	Other disease	Respiratory	PGS002347	EFO_0000684	respiratory system disease	respiratory system disease	EFO_0000684	Other disease	Skeletal
0.7347	1.06E-78	1.19E-75	PGS000515	EFO_0000305	breast carcinoma	breast carcinoma	EFO_0000305	Cancer	Neoplasms	PGS000479	EFO_0000305	breast carcinoma	breast carcinoma	EFO_0000305	Cancer	Neoplasms
0.7346	1.12E-78	1.26E-75	PGS003035	EFO_0004611	low density lipoprotein cholesterol measurement	low density lipoprotein cholesterol measurement	EFO_0004611	Lipid or lipoprotein measurement	Metabolic	PGS003029	EFO_0004611	low density lipoprotein cholesterol measurement	measurement	EFO_0004611	Lipid or lipoprotein measurement	Metabolic
0.7346	1.12E-78	1.26E-75	PGS003035	EFO_0004611	low density lipoprotein cholesterol measurement	low density lipoprotein cholesterol measurement	EFO_0004611	Cardiovascular measurement	Cardiovascular	PGS003029	EFO_0004611	low density lipoprotein cholesterol measurement	measurement	EFO_0004611	Cardiovascular measurement	Cardiovascular
0.7346	1.12E-78	1.26E-75	PGS003035	EFO_0004611	low density lipoprotein cholesterol measurement	low density lipoprotein cholesterol measurement	EFO_0004611	Cardiovascular measurement	Cardiovascular	PGS003029	EFO_0004611	low density lipoprotein cholesterol measurement	measurement	EFO_0004611	Lipid or lipoprotein measurement	Metabolic
0.7346	1.12E-78	1.26E-75	PGS003035	EFO_0004611	low density lipoprotein cholesterol measurement	low density lipoprotein cholesterol measurement	EFO_0004611	Lipid or lipoprotein measurement	Metabolic	PGS003029	EFO_0004611	low density lipoprotein cholesterol measurement	measurement	EFO_0004611	Cardiovascular measurement	Cardiovascular
0.7346	1.13E-78	1.28E-75	PGS002322	EFO_0006336	diastolic blood pressure	diastolic blood pressure	EFO_0006336	Other measurement	Cardiovascular	PGS002114	EFO_0006336	diastolic blood pressure	diastolic blood pressure	EFO_0006336	Other measurement	Cardiovascular
0.7346	1.13E-78	1.28E-75	PGS001159	EFO_0005106	body composition measurement	body composition measurement	EFO_0005106	Body measurement	Body measurement	PGS002168	EFO_0004995	lean body mass	lean body mass	EFO_0004995	Other measurement	Skeletal
0.7346	1.18E-78	1.33E-75	PGS002721	EFO_0004631	vitamin D measurement	vitamin D measurement	EFO_0004631	Lipid or lipoprotein measurement	Metabolic	PGS002427	EFO_0004631	vitamin D measurement	vitamin D measurement	EFO_0004631	Lipid or lipoprotein measurement	Metabolic
0.7346	1.19E-78	1.34E-75	PGS002524	MONDO_0005148	type 2 diabetes mellitus	type 2 diabetes mellitus	MONDO_0005148	Metabolic disorder	Metabolic	PGS002596	EFO_0004468	glucose measurement	glucose measurement	EFO_0004468	Other measurement	Metabolic
0.7346	1.19E-78	1.34E-75	PGS002524	MONDO_0005148	type 2 diabetes mellitus	type 2 diabetes mellitus	MONDO_0005148	Digestive system disorder	Gastrointestinal	PGS002596	EFO_0004468	glucose measurement	glucose measurement	EFO_0004468	Other measurement	Metabolic
-0.7345	1.20E-78	1.36E-75	PGS001094	EFO_0007822	hair colour measurement	hair colour measurement	EFO_0007822	Other measurement	Dermatological	PGS002647	EFO_0007822	hair colour measurement	hair colour measurement	EFO_0007822	Other measurement	Dermatological
0.7345	1.22E-78	1.38E-75	PGS000759	EFO_0004705	hypothyroidism	hypothyroidism	EFO_0004705	Other disease	Endocrine	PGS002668	EFO_1000627	thyroid disease	thyroid disease	EFO_1000627	Other disease	Endocrine
0.7345	1.23E-78	1.39E-75	PGS002876	EFO_0004458	C-reactive protein measurement	C-reactive protein measurement	EFO_0004458	Inflammatory measurement	Immunological	PGS002874	EFO_0004458	C-reactive protein measurement	C-reactive protein measurement	EFO_0004458	Inflammatory measurement	Immunological
0.7345	1.23E-78	1.39E-75	PGS002874	EFO_0004458	C-reactive protein measurement	C-reactive protein measurement	EFO_0004458	Inflammatory measurement	Immunological	PGS002875	EFO_0004458	C-reactive protein measurement	C-reactive protein measurement	EFO_0004458	Inflammatory measurement	Immunological
0.7345	1.24E-78	1.40E-75	PGS001674	EFO_0006930	brain volume measurement|neuroimaging measurement	brain volume measurement	EFO_0006930	Other measurement	Neurological	PGS001662	EFO_0006930	measurement	brain volume measurement	EFO_0006930	Other measurement	Neurological
0.7345	1.25E-78	1.41E-75	PGS001451	EFO_0006930	brain volume measurement|neuroimaging measurement	brain volume measurement	EFO_0006930	Other measurement	Neurological	PGS001466	EFO_0006930	measurement	brain volume measurement	EFO_0006930	Other measurement	Neurological
0.7344	1.30E-78	1.47E-75	PGS001149	EFO_0005409	fat body mass	fat body mass	EFO_0005409	Other measurement	Body measurement	PGS001104	EFO_0007800	body fat percentage	body fat percentage	EFO_0007800	Body measurement	Body measurement
0.7344	1.31E-78	1.48E-75	PGS002869	EFO_0004458	C-reactive protein measurement	C-reactive protein measurement	EFO_0004458	Inflammatory measurement	Immunological	PGS001946	EFO_0004458	C-reactive protein measurement	C-reactive protein measurement	EFO_0004458	Inflammatory measurement	Immunological
0.7344	1.32E-78	1.49E-75	PGS001670	EFO_0006930	brain volume measurement|neuroimaging measurement	brain volume measurement	EFO_0006930	Other measurement	Neurological	PGS001683	EFO_0006930	measurement	brain volume measurement	EFO_0006930	Other measurement	Neurological
0.7343	1.38E-78	1.56E-75	PGS002161	EFO_0004340	body mass index	body mass index	EFO_0004340	Body measurement	Body measurement	PGS001958	EFO_0005093	hip circumference	hip circumference	EFO_0005093	Body measurement	Body measurement
0.7343	1.43E-78	1.61E-75	PGS001404	EFO_0009270	heel bone mineral density	heel bone mineral density	EFO_0009270	Other measurement	Skeletal	PGS000951	EFO_0004514	bone quantitative ultrasound measurement	measurement	EFO_0004514	Other measurement	Skeletal
0.7343	1.46E-78	1.64E-75	PGS000002	EFO_1000649	estrogen-receptor positive breast cancer	estrogen-receptor positive breast cancer	EFO_1000649	Cancer	Neoplasms	PGS000487	EFO_0000305	breast carcinoma	breast carcinoma	EFO_0000305	Cancer	Neoplasms
0.7343	1.46E-78	1.64E-75	PGS000002	EFO_1000649	estrogen-receptor positive breast cancer	estrogen-receptor positive breast cancer	EFO_1000649	Cancer	Neoplasms	PGS000485	EFO_0000305	breast carcinoma	breast carcinoma	EFO_0000305	Cancer	Neoplasms
0.7343	1.48E-78	1.67E-75	PGS002390	EFO_0008328	chronotype measurement	chronotype measurement	EFO_0008328	Other measurement	Psychiatric	PGS002586	EFO_0008328	chronotype measurement	chronotype measurement	EFO_0008328	Other measurement	Psychiatric
0.7341	1.60E-78	1.81E-75	PGS000537	EFO_0000305	breast carcinoma	breast carcinoma	EFO_0000305	Cancer	Neoplasms	PGS000498	EFO_0000305	breast carcinoma	breast carcinoma	EFO_0000305	Cancer	Neoplasms
0.7341	1.60E-78	1.81E-75	PGS000537	EFO_0000305	breast carcinoma	breast carcinoma	EFO_0000305	Cancer	Neoplasms	PGS000497	EFO_0000305	breast carcinoma	breast carcinoma	EFO_0000305	Cancer	Neoplasms
0.7341	1.61E-78	1.82E-75	PGS001167	EFO_0004995	lean body mass	lean body mass	EFO_0004995	Other measurement	Skeletal	PGS002168	EFO_0004995	lean body mass	lean body mass	EFO_0004995	Other measurement	Skeletal
0.7341	1.63E-78	1.83E-75	PGS000515	EFO_0000305	breast carcinoma	breast carcinoma	EFO_0000305	Cancer	Neoplasms	PGS000516	EFO_0000305	breast carcinoma	breast carcinoma	EFO_0000305	Cancer	Neoplasms
0.7341	1.63E-78	1.83E-75	PGS002785	MONDO_0005090	schizophrenia	schizophrenia	MONDO_0005090	Neurological disorder	Neurological	PGS000134	MONDO_0005090	schizophrenia	schizophrenia	MONDO_0005090	Neurological disorder	Neurological
0.7341	1.66E-78	1.86E-75	PGS000215	EFO_1000294	HER2 Positive Breast Carcinoma	HER2 Positive Breast Carcinoma	EFO_1000294	Cancer	Neoplasms	PGS000214	MONDO_0021115	luminal B breast carcinoma	luminal B breast carcinoma	MONDO_0021115	Cancer	Neoplasms
0.734	1.83E-78	2.05E-75	PGS003035	EFO_0004611	low density lipoprotein cholesterol measurement	low density lipoprotein cholesterol measurement	EFO_0004611	Cardiovascular measurement	Cardiovascular	PGS003034	EFO_0004611	low density lipoprotein cholesterol measurement	measurement	EFO_0004611	Lipid or lipoprotein measurement	Metabolic
0.734	1.83E-78	2.05E-75	PGS003035	EFO_0004611	low density lipoprotein cholesterol measurement	low density lipoprotein cholesterol measurement	EFO_0004611	Cardiovascular measurement	Cardiovascular	PGS003034	EFO_0004611	low density lipoprotein cholesterol measurement	measurement	EFO_0004611	Cardiovascular measurement	Cardiovascular
0.734	1.83E-78	2.05E-75	PGS003035	EFO_0004611	low density lipoprotein cholesterol measurement	low density lipoprotein cholesterol measurement	EFO_0004611	Lipid or lipoprotein measurement	Metabolic	PGS003034	EFO_0004611	low density lipoprotein cholesterol measurement	measurement	EFO_0004611	Cardiovascular measurement	Cardiovascular
0.734	1.83E-78	2.05E-75	PGS003035	EFO_0004611	low density lipoprotein cholesterol measurement	low density lipoprotein cholesterol measurement	EFO_0004611	Lipid or lipoprotein measurement	Metabolic	PGS003034	EFO_0004611	low density lipoprotein cholesterol measurement	measurement	EFO_0004611	Lipid or lipoprotein measurement	Metabolic
0.734	1.84E-78	2.06E-75	PGS003147	EFO_0004530	triglyceride measurement	triglyceride measurement	EFO_0004530	Lipid or lipoprotein measurement	Metabolic	PGS003144	EFO_0004530	triglyceride measurement	triglyceride measurement	EFO_0004530	Lipid or lipoprotein measurement	Metabolic
0.7339	1.86E-78	2.09E-75	PGS001478	EFO_0006930	brain volume measurement|neuroimaging measurement	brain volume measurement	EFO_0006930	Other measurement	Neurological	PGS001481	EFO_0006930	measurement	brain volume measurement	EFO_0006930	Other measurement	Neurological
0.7339	1.89E-78	2.12E-75	PGS001745	EFO_0006930	brain volume measurement|neuroimaging measurement	brain volume measurement	EFO_0006930	Other measurement	Neurological	PGS001731	EFO_0006930	measurement	brain volume measurement	EFO_0006930	Other measurement	Neurological
0.7339	1.89E-78	2.13E-75	PGS000508	EFO_0000305	breast carcinoma	breast carcinoma	EFO_0000305	Cancer	Neoplasms	PGS000527	EFO_0000305	breast carcinoma	breast carcinoma	EFO_0000305	Cancer	Neoplasms
0.7339	1.98E-78	2.23E-75	PGS003198	EFO_0004631	vitamin D measurement	vitamin D measurement	EFO_0004631	Lipid or lipoprotein measurement	Metabolic	PGS003184	EFO_0004631	vitamin D measurement	vitamin D measurement	EFO_0004631	Lipid or lipoprotein measurement	Metabolic
0.7338	2.02E-78	2.27E-75	PGS000005	EFO_1000649	estrogen-receptor positive breast cancer	estrogen-receptor positive breast cancer	EFO_1000649	Cancer	Neoplasms	PGS000873	EFO_0000305	breast carcinoma	breast carcinoma	EFO_0000305	Cancer	Neoplasms
0.7338	2.02E-78	2.27E-75	PGS000510	EFO_0000305	breast carcinoma	breast carcinoma	EFO_0000305	Cancer	Neoplasms	PGS000498	EFO_0000305	breast carcinoma	breast carcinoma	EFO_0000305	Cancer	Neoplasms
0.7338	2.02E-78	2.27E-75	PGS000510	EFO_0000305	breast carcinoma	breast carcinoma	EFO_0000305	Cancer	Neoplasms	PGS000497	EFO_0000305	breast carcinoma	breast carcinoma	EFO_0000305	Cancer	Neoplasms
0.7338	2.04E-78	2.29E-75	PGS002764	HP_0003124	Hypercholesterolemia	Hypercholesterolemia	HP_0003124	Other trait	Metabolic	PGS002352	EFO_0004574	total cholesterol measurement	total cholesterol measurement	EFO_0004574	Lipid or lipoprotein measurement	Metabolic
0.7338	2.07E-78	2.32E-75	PGS003199	EFO_0004631	vitamin D measurement	vitamin D measurement	EFO_0004631	Lipid or lipoprotein measurement	Metabolic	PGS003198	EFO_0004631	vitamin D measurement	vitamin D measurement	EFO_0004631	Lipid or lipoprotein measurement	Metabolic
0.7338	2.09E-78	2.34E-75	PGS003118	MONDO_0005148	type 2 diabetes mellitus	type 2 diabetes mellitus	MONDO_0005148	Metabolic disorder	Metabolic	PGS002026	MONDO_0005148	type 2 diabetes mellitus	type 2 diabetes mellitus	MONDO_0005148	Metabolic disorder	Metabolic
0.7338	2.09E-78	2.34E-75	PGS003118	MONDO_0005148	type 2 diabetes mellitus	type 2 diabetes mellitus	MONDO_0005148	Metabolic disorder	Metabolic	PGS002026	MONDO_0005148	type 2 diabetes mellitus	type 2 diabetes mellitus	MONDO_0005148	Digestive system disorder	Gastrointestinal
0.7338	2.09E-78	2.34E-75	PGS003118	MONDO_0005148	type 2 diabetes mellitus	type 2 diabetes mellitus	MONDO_0005148	Digestive system disorder	Gastrointestinal	PGS002026	MONDO_0005148	type 2 diabetes mellitus	type 2 diabetes mellitus	MONDO_0005148	Digestive system disorder	Gastrointestinal
0.7338	2.09E-78	2.34E-75	PGS003118	MONDO_0005148	type 2 diabetes mellitus	type 2 diabetes mellitus	MONDO_0005148	Digestive system disorder	Gastrointestinal	PGS002026	MONDO_0005148	type 2 diabetes mellitus	type 2 diabetes mellitus	MONDO_0005148	Metabolic disorder	Metabolic
0.7337	2.19E-78	2.45E-75	PGS000591	EFO_0001663	prostate carcinoma	prostate carcinoma	EFO_0001663	Cancer	Neoplasms	PGS000573	EFO_0001663	prostate carcinoma	prostate carcinoma	EFO_0001663	Cancer	Neoplasms
0.7337	2.21E-78	2.47E-75	PGS002313	EFO_0004340	body mass index	body mass index	EFO_0004340	Body measurement	Body measurement	PGS002133	EFO_0007800	body fat percentage	body fat percentage	EFO_0007800	Body measurement	Body measurement
0.7336	2.40E-78	2.69E-75	PGS003146	EFO_0004530	triglyceride measurement	triglyceride measurement	EFO_0004530	Lipid or lipoprotein measurement	Metabolic	PGS003147	EFO_0004530	triglyceride measurement	triglyceride measurement	EFO_0004530	Lipid or lipoprotein measurement	Metabolic
0.7336	2.42E-78	2.72E-75	PGS000539	EFO_0000305	breast carcinoma	breast carcinoma	EFO_0000305	Cancer	Neoplasms	PGS000507	EFO_0000305	breast carcinoma	breast carcinoma	EFO_0000305	Cancer	Neoplasms
0.7335	2.49E-78	2.79E-75	PGS001979	EFO_0004530	triglyceride measurement	triglyceride measurement	EFO_0004530	Lipid or lipoprotein measurement	Metabolic	PGS003152	EFO_0004530	triglyceride measurement	triglyceride measurement	EFO_0004530	Lipid or lipoprotein measurement	Metabolic
0.7335	2.59E-78	2.91E-75	PGS002764	HP_0003124	Hypercholesterolemia	Hypercholesterolemia	HP_0003124	Other trait	Metabolic	PGS001889	EFO_0004615	apolipoprotein B measurement	apolipoprotein B measurement	EFO_0004615	Lipid or lipoprotein measurement	Metabolic
0.7335	2.62E-78	2.93E-75	PGS002327	EFO_0004312	vital capacity	vital capacity	EFO_0004312	Other measurement	Activities	PGS002644	EFO_0004312	vital capacity	vital capacity	EFO_0004312	Other measurement	Activities
0.7335	2.63E-78	2.94E-75	PGS003031	EFO_0004611	low density lipoprotein cholesterol measurement	low density lipoprotein cholesterol measurement	EFO_0004611	Lipid or lipoprotein measurement	Metabolic	PGS002571	EFO_0004574	total cholesterol measurement	total cholesterol measurement	EFO_0004574	Lipid or lipoprotein measurement	Metabolic
0.7335	2.63E-78	2.94E-75	PGS003031	EFO_0004611	low density lipoprotein cholesterol measurement	low density lipoprotein cholesterol measurement	EFO_0004611	Cardiovascular measurement	Cardiovascular	PGS002571	EFO_0004574	total cholesterol measurement	total cholesterol measurement	EFO_0004574	Lipid or lipoprotein measurement	Metabolic
0.7334	2.65E-78	2.97E-75	PGS002432	MONDO_0004979	asthma	asthma	MONDO_0004979	Other disease	Respiratory	PGS002517	EFO_0000684	respiratory system disease	respiratory system disease	EFO_0000684	Other disease	Skeletal
0.7334	2.66E-78	2.98E-75	PGS000824	EFO_0004611	low density lipoprotein cholesterol measurement	low density lipoprotein cholesterol measurement	EFO_0004611	Lipid or lipoprotein measurement	Metabolic	PGS002620	EFO_0004574	total cholesterol measurement	total cholesterol measurement	EFO_0004574	Lipid or lipoprotein measurement	Metabolic
0.7334	2.66E-78	2.98E-75	PGS000824	EFO_0004611	low density lipoprotein cholesterol measurement	low density lipoprotein cholesterol measurement	EFO_0004611	Cardiovascular measurement	Cardiovascular	PGS002620	EFO_0004574	total cholesterol measurement	total cholesterol measurement	EFO_0004574	Lipid or lipoprotein measurement	Metabolic
0.7334	2.73E-78	3.05E-75	PGS001451	EFO_0006930	brain volume measurement|neuroimaging measurement	brain volume measurement	EFO_0006930	Other measurement	Neurological	PGS001672	EFO_0006930	measurement	brain volume measurement	EFO_0006930	Other measurement	Neurological
0.7334	2.74E-78	3.06E-75	PGS003029	EFO_0004611	low density lipoprotein cholesterol measurement	low density lipoprotein cholesterol measurement	EFO_0004611	Lipid or lipoprotein measurement	Metabolic	PGS003030	EFO_0004611	low density lipoprotein cholesterol measurement	measurement	EFO_0004611	Cardiovascular measurement	Cardiovascular
0.7334	2.74E-78	3.06E-75	PGS003029	EFO_0004611	low density lipoprotein cholesterol measurement	low density lipoprotein cholesterol measurement	EFO_0004611	Cardiovascular measurement	Cardiovascular	PGS003030	EFO_0004611	low density lipoprotein cholesterol measurement	measurement	EFO_0004611	Lipid or lipoprotein measurement	Metabolic
0.7334	2.74E-78	3.06E-75	PGS003029	EFO_0004611	low density lipoprotein cholesterol measurement	low density lipoprotein cholesterol measurement	EFO_0004611	Cardiovascular measurement	Cardiovascular	PGS003030	EFO_0004611	low density lipoprotein cholesterol measurement	measurement	EFO_0004611	Cardiovascular measurement	Cardiovascular
0.7334	2.74E-78	3.06E-75	PGS003029	EFO_0004611	low density lipoprotein cholesterol measurement	low density lipoprotein cholesterol measurement	EFO_0004611	Lipid or lipoprotein measurement	Metabolic	PGS003030	EFO_0004611	low density lipoprotein cholesterol measurement	measurement	EFO_0004611	Lipid or lipoprotein measurement	Metabolic
0.7334	2.75E-78	3.07E-75	PGS001228	EFO_0004340	body mass index	body mass index	EFO_0004340	Body measurement	Body measurement	PGS001104	EFO_0007800	body fat percentage	body fat percentage	EFO_0007800	Body measurement	Body measurement
0.7334	2.84E-78	3.17E-75	PGS000515	EFO_0000305	breast carcinoma	breast carcinoma	EFO_0000305	Cancer	Neoplasms	PGS000472	EFO_0000305	breast carcinoma	breast carcinoma	EFO_0000305	Cancer	Neoplasms
0.7333	2.89E-78	3.22E-75	PGS000001	EFO_0000305	breast carcinoma	breast carcinoma	EFO_0000305	Cancer	Neoplasms	PGS000540	EFO_0000305	breast carcinoma	breast carcinoma	EFO_0000305	Cancer	Neoplasms
0.7333	3.00E-78	3.35E-75	PGS000951	EFO_0004514	bone quantitative ultrasound measurement	bone quantitative ultrasound measurement	EFO_0004514	Other measurement	Skeletal	PGS000953	EFO_0004514	bone quantitative ultrasound measurement	measurement	EFO_0004514	Other measurement	Skeletal
0.7332	3.13E-78	3.49E-75	PGS000585	EFO_0001663	prostate carcinoma	prostate carcinoma	EFO_0001663	Cancer	Neoplasms	PGS000587	EFO_0001663	prostate carcinoma	prostate carcinoma	EFO_0001663	Cancer	Neoplasms
0.7332	3.29E-78	3.68E-75	PGS001248	EFO_0004274	gout	gout	EFO_0004274	Metabolic disorder	Metabolic	PGS000711	EFO_0004274	gout	gout	EFO_0004274	Metabolic disorder	Metabolic
0.7331	3.49E-78	3.90E-75	PGS000133	MONDO_0005090	schizophrenia	schizophrenia	MONDO_0005090	Neurological disorder	Neurological	PGS002785	MONDO_0005090	schizophrenia	schizophrenia	MONDO_0005090	Neurological disorder	Neurological
0.7331	3.50E-78	3.90E-75	PGS002122	EFO_0005192	red blood cell distribution width	red blood cell distribution width	EFO_0005192	Hematological measurement	Hematological	PGS001152	EFO_0005192	red blood cell distribution width	red blood cell distribution width	EFO_0005192	Hematological measurement	Hematological
0.733	3.61E-78	4.03E-75	PGS001479	EFO_0006930	brain volume measurement|neuroimaging measurement	brain volume measurement	EFO_0006930	Other measurement	Neurological	PGS001477	EFO_0006930	measurement	brain volume measurement	EFO_0006930	Other measurement	Neurological
0.733	3.66E-78	4.08E-75	PGS001961	EFO_0005106	body composition measurement	body composition measurement	EFO_0005106	Body measurement	Body measurement	PGS001154	EFO_0005106	body composition measurement	body composition measurement	EFO_0005106	Body measurement	Body measurement

0.733	3.69E-78	4.12E-75	PGS001529	EFO_0007772	calcaneal bone quantitative ultrasound measurement	calcaneal bone quantitative ultrasound measurement	EFO_0007772	Other measurement	Skeletal	PGS001404	EFO_0009270	heel bone mineral density	heel bone mineral density	EFO_0009270	Other measurement	Skeletal
0.733	3.72E-78	4.15E-75	PGS001235	EFO_0004324	body weights and measures	body weights and measures	EFO_0004324	Body measurement	Body measurement	PGS002168	EFO_0004995	lean body mass	lean body mass	EFO_0004995	Other measurement	Skeletal
0.733	3.72E-78	4.15E-75	PGS001146	EFO_0005409	fat body mass	fat body mass	EFO_0005409	Other measurement	Body measurement	PGS002202	EFO_0009805	whole body water mass	whole body water mass	EFO_0009805	Other measurement	Body measurement
0.733	3.78E-78	4.21E-75	PGS000479	EFO_0000305	breast carcinoma	breast carcinoma	EFO_0000305	Cancer	Neoplasms	PGS000472	EFO_0000305	breast carcinoma	breast carcinoma	EFO_0000305	Cancer	Neoplasms
0.7329	3.91E-78	4.36E-75	PGS003088	EFO_0006335	systolic blood pressure	systolic blood pressure	EFO_0006335	Other measurement	Cardiovascular	PGS003084	EFO_0006335	systolic blood pressure	systolic blood pressure	EFO_0006335	Other measurement	Cardiovascular
0.7329	3.91E-78	4.36E-75	PGS003151	EFO_0004530	triglyceride measurement	triglyceride measurement	EFO_0004530	Lipid or lipoprotein measurement	Metabolic	PGS003148	EFO_0004530	triglyceride measurement	triglyceride measurement	EFO_0004530	Lipid or lipoprotein measurement	Metabolic
0.7329	3.95E-78	4.40E-75	PGS002963	EFO_0004612	high density lipoprotein cholesterol measurement	high density lipoprotein cholesterol measurement	EFO_0004612	Lipid or lipoprotein measurement	Metabolic	PGS002101	EFO_0004614	apolipoprotein A 1 measurement	apolipoprotein A 1 measurement	EFO_0004614	Cardiovascular measurement	Cardiovascular
0.7329	3.95E-78	4.40E-75	PGS002963	EFO_0004612	high density lipoprotein cholesterol measurement	high density lipoprotein cholesterol measurement	EFO_0004612	Lipid or lipoprotein measurement	Metabolic	PGS002101	EFO_0004614	apolipoprotein A 1 measurement	apolipoprotein A 1 measurement	EFO_0004614	Lipid or lipoprotein measurement	Metabolic
0.7329	3.95E-78	4.40E-75	PGS002963	EFO_0004612	high density lipoprotein cholesterol measurement	high density lipoprotein cholesterol measurement	EFO_0004612	Lipid or lipoprotein measurement	Metabolic	PGS002101	EFO_0004614	apolipoprotein A 1 measurement	apolipoprotein A 1 measurement	EFO_0004614	Inflammatory measurement	Immunological
0.7329	3.99E-78	4.44E-75	PGS000153	EFO_0000305	breast carcinoma	breast carcinoma	EFO_0000305	Cancer	Neoplasms	PGS000045	EFO_0000305	breast carcinoma	breast carcinoma	EFO_0000305	Cancer	Neoplasms
0.7329	4.03E-78	4.48E-75	PGS001241	EFO_0004302	anthropometric measurement	anthropometric measurement	EFO_0004302	Other measurement	Body measurement	PGS001887	EFO_0004302	anthropometric measurement	anthropometric measurement	EFO_0004302	Other measurement	Body measurement
0.7328	4.22E-78	4.70E-75	PGS000540	EFO_0000305	breast carcinoma	breast carcinoma	EFO_0000305	Cancer	Neoplasms	PGS000007	EFO_0000305	breast carcinoma	breast carcinoma	EFO_0000305	Cancer	Neoplasms
0.7328	4.36E-78	4.86E-75	PGS003244	EFO_0010472	creatine measurement	creatine measurement	EFO_0010472	Other measurement	Metabolic	PGS003245	EFO_0010472	creatine measurement	creatine measurement	EFO_0010472	Other measurement	Metabolic
0.7327	4.42E-78	4.92E-75	PGS000533	EFO_0000305	breast carcinoma	breast carcinoma	EFO_0000305	Cancer	Neoplasms	PGS000529	EFO_0000305	breast carcinoma	breast carcinoma	EFO_0000305	Cancer	Neoplasms
0.7327	4.42E-78	4.92E-75	PGS000534	EFO_0000305	breast carcinoma	breast carcinoma	EFO_0000305	Cancer	Neoplasms	PGS000529	EFO_0000305	breast carcinoma	breast carcinoma	EFO_0000305	Cancer	Neoplasms
0.7327	4.48E-78	4.98E-75	PGS002131	EFO_0004703	age at menarche	age at menarche	EFO_0004703	Other measurement	Genitourinary	PGS002310	EFO_0004703	age at menarche	age at menarche	EFO_0004703	Other measurement	Genitourinary
-0.7327	4.52E-78	5.02E-75	PGS000883	EFO_0005208	glomerular filtration rate	glomerular filtration rate	EFO_0005208	Other measurement	Genitourinary	PGS003219	EFO_0005208	glomerular filtration rate	glomerular filtration rate	EFO_0005208	Other measurement	Genitourinary
0.7326	4.82E-78	5.36E-75	PGS000956	EFO_0004514	bone quantitative ultrasound measurement	bone quantitative ultrasound measurement	EFO_0004514	Other measurement	Skeletal	PGS001402	EFO_0009270	heel bone mineral density	heel bone mineral density	EFO_0009270	Other measurement	Skeletal
0.7326	4.85E-78	5.39E-75	PGS001039	EFO_0009270	heel bone mineral density	heel bone mineral density	EFO_0009270	Other measurement	Skeletal	PGS001402	EFO_0009270	heel bone mineral density	heel bone mineral density	EFO_0009270	Other measurement	Skeletal
0.7326	4.91E-78	5.46E-75	PGS001464	EFO_0006930	brain volume measurement|neuroimaging measurement	brain volume measurement	EFO_0006930	Other measurement	Neurological	PGS001463	EFO_0006930	measurement	brain volume measurement	EFO_0006930	Other measurement	Neurological
0.7326	4.98E-78	5.53E-75	PGS001280	EFO_0003827	pulmonary embolism	pulmonary embolism	EFO_0003827	Cardiovascular disease	Cardiovascular	PGS000961	EFO_1001395	Phlebitis|Thrombophlebitis	Phlebitis	EFO_1001395	Cardiovascular disease	Cardiovascular
0.7325	5.31E-78	5.90E-75	PGS003103	MONDO_0005148	type 2 diabetes mellitus	type 2 diabetes mellitus	MONDO_0005148	Digestive system disorder	Gastrointestinal	PGS002026	MONDO_0005148	type 2 diabetes mellitus	type 2 diabetes mellitus	MONDO_0005148	Metabolic disorder	Metabolic
0.7325	5.31E-78	5.90E-75	PGS003103	MONDO_0005148	type 2 diabetes mellitus	type 2 diabetes mellitus	MONDO_0005148	Metabolic disorder	Metabolic	PGS002026	MONDO_0005148	type 2 diabetes mellitus	type 2 diabetes mellitus	MONDO_0005148	Digestive system disorder	Gastrointestinal
0.7325	5.31E-78	5.90E-75	PGS003103	MONDO_0005148	type 2 diabetes mellitus	type 2 diabetes mellitus	MONDO_0005148	Metabolic disorder	Metabolic	PGS002026	MONDO_0005148	type 2 diabetes mellitus	type 2 diabetes mellitus	MONDO_0005148	Metabolic disorder	Metabolic
0.7325	5.31E-78	5.90E-75	PGS003103	MONDO_0005148	type 2 diabetes mellitus	type 2 diabetes mellitus	MONDO_0005148	Digestive system disorder	Gastrointestinal	PGS002026	MONDO_0005148	type 2 diabetes mellitus	type 2 diabetes mellitus	MONDO_0005148	Digestive system disorder	Gastrointestinal
0.7325	5.42E-78	6.02E-75	PGS001984	EFO_0009805	whole body water mass	whole body water mass	EFO_0009805	Other measurement	Body measurement	PGS001165	EFO_0004995	lean body mass	lean body mass	EFO_0004995	Other measurement	Skeletal
0.7324	5.54E-78	6.14E-75	PGS002328	EFO_0004468	glucose measurement	glucose measurement	EFO_0004468	Other measurement	Metabolic	PGS002943	EFO_0004468	glucose measurement	glucose measurement	EFO_0004468	Other measurement	Metabolic
0.7324	5.54E-78	6.15E-75	PGS002425	EFO_0004530	triglyceride measurement	triglyceride measurement	EFO_0004530	Lipid or lipoprotein measurement	Metabolic	PGS000826	EFO_0004530	triglyceride measurement	triglyceride measurement	EFO_0004530	Lipid or lipoprotein measurement	Metabolic
0.7324	5.72E-78	6.35E-75	PGS001166	EFO_0004995	lean body mass	lean body mass	EFO_0004995	Other measurement	Skeletal	PGS002202	EFO_0009805	whole body water mass	whole body water mass	EFO_0009805	Other measurement	Body measurement
0.7324	5.84E-78	6.47E-75	PGS002620	EFO_0004574	total cholesterol measurement	total cholesterol measurement	EFO_0004574	Lipid or lipoprotein measurement	Metabolic	PGS000889	EFO_0004611	low density lipoprotein cholesterol measurement	measurement	EFO_0004611	Lipid or lipoprotein measurement	Metabolic
0.7324	5.84E-78	6.47E-75	PGS002620	EFO_0004574	total cholesterol measurement	total cholesterol measurement	EFO_0004574	Lipid or lipoprotein measurement	Metabolic	PGS000889	EFO_0004611	low density lipoprotein cholesterol measurement	measurement	EFO_0004611	Cardiovascular measurement	Cardiovascular
0.7323	6.21E-78	6.88E-75	PGS002672	EFO_0004631	vitamin D measurement	vitamin D measurement	EFO_0004631	Lipid or lipoprotein measurement	Metabolic	PGS003186	EFO_0004631	vitamin D measurement	vitamin D measurement	EFO_0004631	Lipid or lipoprotein measurement	Metabolic
0.7323	6.30E-78	6.98E-75	PGS000018	EFO_0001645	coronary artery disease	coronary artery disease	EFO_0001645	Cardiovascular disease	Cardiovascular	PGS001355	EFO_0001645	coronary artery disease	coronary artery disease	EFO_0001645	Cardiovascular disease	Cardiovascular
0.7322	6.34E-78	7.02E-75	PGS000895	EFO_0004611	low density lipoprotein cholesterol measurement	low density lipoprotein cholesterol measurement	EFO_0004611	Cardiovascular measurement	Cardiovascular	PGS002654	EFO_0004611	low density lipoprotein cholesterol measurement	measurement	EFO_0004611	Cardiovascular measurement	Cardiovascular
0.7322	6.34E-78	7.02E-75	PGS000895	EFO_0004611	low density lipoprotein cholesterol measurement	low density lipoprotein cholesterol measurement	EFO_0004611	Lipid or lipoprotein measurement	Metabolic	PGS002654	EFO_0004611	low density lipoprotein cholesterol measurement	measurement	EFO_0004611	Lipid or lipoprotein measurement	Metabolic
0.7322	6.34E-78	7.02E-75	PGS000895	EFO_0004611	low density lipoprotein cholesterol measurement	low density lipoprotein cholesterol measurement	EFO_0004611	Lipid or lipoprotein measurement	Metabolic	PGS002654	EFO_0004611	low density lipoprotein cholesterol measurement	measurement	EFO_0004611	Cardiovascular measurement	Cardiovascular
0.7322	6.34E-78	7.02E-75	PGS000895	EFO_0004611	low density lipoprotein cholesterol measurement	low density lipoprotein cholesterol measurement	EFO_0004611	Cardiovascular measurement	Cardiovascular	PGS002654	EFO_0004611	low density lipoprotein cholesterol measurement	measurement	EFO_0004611	Lipid or lipoprotein measurement	Metabolic
0.7322	6.44E-78	7.13E-75	PGS000900	EFO_0006335	systolic blood pressure|female	systolic blood pressure	EFO_0006335	Other measurement	Cardiovascular	PGS003074	EFO_0006335	systolic blood pressure	systolic blood pressure	EFO_0006335	Other measurement	Cardiovascular
0.7322	6.48E-78	7.18E-75	PGS001440	EFO_0006930	brain volume measurement|neuroimaging measurement	brain volume measurement	EFO_0006930	Other measurement	Neurological	PGS001439	EFO_0006930	measurement	brain volume measurement	EFO_0006930	Other measurement	Neurological
0.7322	6.58E-78	7.29E-75	PGS003036	EFO_0004611	low density lipoprotein cholesterol measurement	low density lipoprotein cholesterol measurement	EFO_0004611	Lipid or lipoprotein measurement	Metabolic	PGS002605	EFO_0004611	low density lipoprotein cholesterol measurement	measurement	EFO_0004611	Cardiovascular measurement	Cardiovascular
0.7322	6.58E-78	7.29E-75	PGS003036	EFO_0004611	low density lipoprotein cholesterol measurement	low density lipoprotein cholesterol measurement	EFO_0004611	Lipid or lipoprotein measurement	Metabolic	PGS002605	EFO_0004611	low density lipoprotein cholesterol measurement	measurement	EFO_0004611	Lipid or lipoprotein measurement	Metabolic
0.7322	6.58E-78	7.29E-75	PGS003036	EFO_0004611	low density lipoprotein cholesterol measurement	low density lipoprotein cholesterol measurement	EFO_0004611	Cardiovascular measurement	Cardiovascular	PGS002605	EFO_0004611	low density lipoprotein cholesterol measurement	measurement	EFO_0004611	Lipid or lipoprotein measurement	Metabolic
0.7322	6.58E-78	7.29E-75	PGS003036	EFO_0004611	low density lipoprotein cholesterol measurement	low density lipoprotein cholesterol measurement	EFO_0004611	Cardiovascular measurement	Cardiovascular	PGS002605	EFO_0004611	low density lipoprotein cholesterol measurement	measurement	EFO_0004611	Cardiovascular measurement	Cardiovascular
0.7322	6.67E-78	7.38E-75	PGS000051	EFO_0000305	breast carcinoma	breast carcinoma	EFO_0000305	Cancer	Neoplasms	PGS000052	EFO_0000305	breast carcinoma	breast carcinoma	EFO_0000305	Cancer	Neoplasms
0.7322	6.72E-78	7.44E-75	PGS001719	EFO_0006930	brain volume measurement|neuroimaging measurement	brain volume measurement	EFO_0006930	Other measurement	Neurological	PGS001748	EFO_0006930	measurement	brain volume measurement	EFO_0006930	Other measurement	Neurological
0.7322	6.72E-78	7.44E-75	PGS002640	HP_0000964	Eczema	Eczema	HP_0000964	Other trait	Dermatological	PGS002542	HP_0000964	Eczema	Eczema	HP_0000964	Other trait	Dermatological
0.7321	7.17E-78	7.94E-75	PGS000482	EFO_0000305	breast carcinoma	breast carcinoma	EFO_0000305	Cancer	Neoplasms	PGS000479	EFO_0000305	breast carcinoma	breast carcinoma	EFO_0000305	Cancer	Neoplasms
0.732	7.61E-78	8.42E-75	PGS002435	EFO_0007825	balding measurement	balding measurement	EFO_0007825	Other measurement	Activities	PGS002533	EFO_0007825	balding measurement	balding measurement	EFO_0007825	Other measurement	Activities
0.732	7.79E-78	8.62E-75	PGS002856	EFO_0004340	body mass index	body mass index	EFO_0004340	Body measurement	Body measurement	PGS002850	EFO_0004340	body mass index	body mass index	EFO_0004340	Body measurement	Body measurement
0.7319	7.91E-78	8.75E-75	PGS002874	EFO_0004458	C-reactive protein measurement	C-reactive protein measurement	EFO_0004458	Inflammatory measurement	Immunological	PGS002885	EFO_0004458	C-reactive protein measurement	C-reactive protein measurement	EFO_0004458	Inflammatory measurement	Immunological
0.7319	7.91E-78	8.75E-75	PGS002886	EFO_0004458	C-reactive protein measurement	C-reactive protein measurement	EFO_0004458	Inflammatory measurement	Immunological	PGS002874	EFO_0004458	C-reactive protein measurement	C-reactive protein measurement	EFO_0004458	Inflammatory measurement	Immunological
0.7319	7.96E-78	8.79E-75	PGS000310	EFO_0004611	low density lipoprotein cholesterol measurement	low density lipoprotein cholesterol measurement	EFO_0004611	Cardiovascular measurement	Cardiovascular	PGS002102	EFO_0004615	apolipoprotein B measurement	apolipoprotein B measurement	EFO_0004615	Lipid or lipoprotein measurement	Metabolic
0.7319	7.96E-78	8.79E-75	PGS000310	EFO_0004611	low density lipoprotein cholesterol measurement	low density lipoprotein cholesterol measurement	EFO_0004611	Lipid or lipoprotein measurement	Metabolic	PGS002102	EFO_0004615	apolipoprotein B measurement	apolipoprotein B measurement	EFO_0004615	Lipid or lipoprotein measurement	Metabolic
0.7319	8.30E-78	9.17E-75	PGS000160	EFO_0001663	prostate carcinoma	prostate carcinoma	EFO_0001663	Cancer	Neoplasms	PGS000795	EFO_0001663	prostate carcinoma	prostate carcinoma	EFO_0001663	Cancer	Neoplasms
0.7318	8.42E-78	9.30E-75	PGS002971	EFO_0004339	body height	body height	EFO_0004339	Body measurement	Body measurement	PGS002969	EFO_0004339	body height	body height	EFO_0004339	Body measurement	Body measurement
0.7318	8.46E-78	9.34E-75	PGS003151	EFO_0004530	triglyceride measurement	triglyceride measurement	EFO_0004530	Lipid or lipoprotein measurement	Metabolic	PGS003153	EFO_0004530	triglyceride measurement	triglyceride measurement	EFO_0004530	Lipid or lipoprotein measurement	Metabolic
0.7318	8.49E-78	9.38E-75	PGS000018	EFO_0001645	coronary artery disease	coronary artery disease	EFO_0001645	Cardiovascular disease	Cardiovascular	PGS000013	EFO_0001645	coronary artery disease	coronary artery disease	EFO_0001645	Cardiovascular disease	Cardiovascular
0.7318	8.61E-78	9.51E-75	PGS000873	EFO_0000305	breast carcinoma	breast carcinoma	EFO_0000305	Cancer	Neoplasms	PGS001804	EFO_0000305	breast carcinoma	breast carcinoma	EFO_0000305	Cancer	Neoplasms
0.7318	8.73E-78	9.64E-75	PGS000018	EFO_0001645	coronary artery disease	coronary artery disease	EFO_0001645	Cardiovascular disease	Cardiovascular	PGS000296	EFO_0001645	coronary artery disease	coronary artery disease	EFO_0001645	Cardiovascular disease	Cardiovascular
0.7318	8.94E-78	9.86E-75	PGS000590	EFO_0001663	prostate carcinoma	prostate carcinoma	EFO_0001663	Cancer	Neoplasms	PGS000570	EFO_0001663	prostate carcinoma	prostate carcinoma	EFO_0001663	Cancer	Neoplasms
0.7318	8.95E-78	9.87E-75	PGS003030	EFO_0004611	low density lipoprotein cholesterol measurement	low density lipoprotein cholesterol measurement	EFO_0004611	Lipid or lipoprotein measurement	Metabolic	PGS003034	EFO_0004611	low density lipoprotein cholesterol measurement	measurement	EFO_0004611	Lipid or lipoprotein measurement	Metabolic
0.7318	8.95E-78	9.87E-75	PGS003030	EFO_0004611	low density lipoprotein cholesterol measurement	low density lipoprotein cholesterol measurement	EFO_0004611	Cardiovascular measurement	Cardiovascular	PGS003034	EFO_0004611	low density lipoprotein cholesterol measurement	measurement	EFO_0004611	Cardiovascular measurement	Cardiovascular
0.7318	8.95E-78	9.87E-75	PGS003030	EFO_0004611	low density lipoprotein cholesterol measurement	low density lipoprotein cholesterol measurement	EFO_0004611	Cardiovascular measurement	Cardiovascular	PGS003034	EFO_0004611	low density lipoprotein cholesterol measurement	measurement	EFO_0004611	Lipid or lipoprotein measurement	Metabolic
0.7318	8.95E-78	9.87E-75	PGS003030	EFO_0004611	low density lipoprotein cholesterol measurement	low density lipoprotein cholesterol measurement	EFO_0004611	Lipid or lipoprotein measurement	Metabolic	PGS003034	EFO_0004611	low density lipoprotein cholesterol measurement	measurement	EFO_0004611	Cardiovascular measurement	Cardiovascular
0.7318	9.01E-78	9.94E-75	PGS002958	EFO_0004612	high density lipoprotein cholesterol measurement	high density lipoprotein cholesterol measurement	EFO_0004612	Lipid or lipoprotein measurement	Metabolic	PGS001888	EFO_0004614	apolipoprotein A 1 measurement	apolipoprotein A 1 measurement	EFO_0004614	Lipid or lipoprotein measurement	Metabolic
0.7318	9.01E-78	9.94E-75	PGS002958	EFO_0004612	high density lipoprotein cholesterol measurement	high density lipoprotein cholesterol measurement	EFO_0004612	Lipid or lipoprotein measurement	Metabolic	PGS001888	EFO_0004614	apolipoprotein A 1 measurement	apolipoprotein A 1 measurement	EFO_0004614	Cardiovascular measurement	Cardiovascular
0.7318	9.01E-78	9.94E-75	PGS002958	EFO_0004612	high density lipoprotein cholesterol measurement	high density lipoprotein cholesterol measurement	EFO_0004612	Lipid or lipoprotein measurement	Metabolic	PGS001888	EFO_0004614	apolipoprotein A 1 measurement	apolipoprotein A 1 measurement	EFO_0004614	Inflammatory measurement	Immunological
0.7317	9.38E-78	1.03E-74	PGS001489	EFO_0006930	brain volume measurement|neuroimaging measurement	brain volume measurement	EFO_0006930	Other measurement	Neurological	PGS001480	EFO_0006930	measurement	brain volume measurement	EFO_0006930	Other measurement	Neurological
0.7317	9.40E-78	1.04E-74	PGS002571	EFO_0004574	total cholesterol measurement	total cholesterol measurement	EFO_0004574	Lipid or lipoprotein measurement	Metabolic	PGS000889	EFO_0004611	low density lipoprotein cholesterol measurement	measurement	EFO_0004611	Cardiovascular measurement	Cardiovascular
0.7317	9.40E-78	1.04E-74	PGS002571	EFO_0004574	total cholesterol measurement	total cholesterol measurement	EFO_0004574	Lipid or lipoprotein measurement	Metabolic	PGS000889	EFO_0004611	low density lipoprotein cholesterol measurement	measurement	EFO_0004611	Lipid or lipoprotein measurement	Metabolic
0.7317	9.43E-78	1.04E-74	PGS000538	EFO_0000305	breast carcinoma	breast carcinoma	EFO_0000305	Cancer	Neoplasms	PGS000513	EFO_0000305	breast carcinoma	breast carcinoma	EFO_0000305	Cancer	Neoplasms
0.7316	1.01E-77	1.11E-74	PGS001684	EFO_0006930	brain volume measurement|neuroimaging measurement	brain volume measurement	EFO_0006930	Other measurement	Neurological	PGS001682	EFO_0006930	measurement	brain volume measurement	EFO_0006930	Other measurement	Neurological
0.7316	1.02E-77	1.12E-74	PGS003035	EFO_0004611	low density lipoprotein cholesterol measurement	low density lipoprotein cholesterol measurement	EFO_0004611	Lipid or lipoprotein measurement	Metabolic	PGS003037	EFO_0004611	low density lipoprotein cholesterol measurement	measurement	EFO_0004611	Cardiovascular measurement	Cardiovascular
0.7316	1.02E-77	1.12E-74	PGS003035	EFO_0004611	low density lipoprotein cholesterol measurement	low density lipoprotein cholesterol measurement	EFO_0004611	Cardiovascular measurement	Cardiovascular	PGS003037	EFO_0004611	low density lipoprotein cholesterol measurement	measurement	EFO_0004611	Lipid or lipoprotein measurement	Metabolic
0.7316	1.02E-77	1.12E-74	PGS003035	EFO_0004611	low density lipoprotein cholesterol measurement	low density lipoprotein cholesterol measurement	EFO_0004611	Lipid or lipoprotein measurement	Metabolic	PGS003037	EFO_0004611	low density lipoprotein cholesterol measurement	measurement	EFO_0004611	Lipid or lipoprotein measurement	Metabolic
0.7316	1.02E-77	1.12E-74	PGS003035	EFO_0004611	low density lipoprotein cholesterol measurement	low density lipoprotein cholesterol measurement	EFO_0004611	Cardiovascular measurement	Cardiovascular	PGS003037	EFO_0004611	low density lipoprotein cholesterol measurement	measurement	EFO_0004611	Cardiovascular measurement	Cardiovascular
0.7316	1.04E-77	1.14E-74	PGS002840	EFO_0004340	body mass index	body mass index	EFO_0004340	Body measurement	Body measurement	PGS002856	EFO_0004340	body mass index	body mass index	EFO_0004340	Body measurement	Body measurement
0.7316	1.04E-77	1.15E-74	PGS002049	MONDO_0004596	cor pulmonale	cor pulmonale	MONDO_0004596	Cardiovascular disease	Cardiovascular	PGS001280	EFO_0003827	pulmonary embolism	pulmonary embolism	EFO_0003827	Cardiovascular disease	Cardiovascular
0.7315	1.07E-77	1.18E-74	PGS002808	EFO_0001071	lung carcinoma	lung carcinoma	EFO_0001071	Cancer	Neoplasms	PGS000390	MONDO_0001672	bronchus cancer|lung cancer|tracheal cancer	bronchus cancer	MONDO_0001672	Cancer	Neoplasms
0.7315	1.07E-77	1.18E-74	PGS002808	EFO_0001071	lung carcinoma	lung carcinoma	EFO_0001071	Cancer	Neoplasms	PGS000389	MONDO_0001672	bronchus cancer|lung cancer|tracheal cancer	bronchus cancer	MONDO_0001672	Cancer	Neoplasms
0.7315	1.10E-77	1.21E-74	PGS001162	EFO_0005093	hip circumference	hip circumference	EFO_0005093	Body measurement	Body measurement	PGS001102	EFO_0007800	body fat percentage	body fat percentage	EFO_0007800	Body measurement	Body measurement
-0.7314	1.14E-77	1.25E-74	PGS000309	EFO_0004612	high density lipoprotein cholesterol measurement	high density lipoprotein cholesterol measurement	EFO_0004612	Lipid or lipoprotein measurement	Metabolic	PGS000060	EFO_0004612	high density lipoprotein cholesterol measurement	measurement	EFO_0004612	Lipid or lipoprotein measurement	Metabolic
0.7314	1.14E-77	1.25E-74	PGS001154	EFO_0005106	body composition measurement	body composition measurement	EFO_0005106	Body measurement	Body measurement	PGS002179	EFO_0005106	body composition measurement	body composition measurement	EFO_0005106	Body measurement	Body measurement
0.7314	1.14E-77	1.26E-74	PGS002672	EFO_0004631	vitamin D measurement	vitamin D measurement	EFO_0004631	Lipid or lipoprotein measurement	Metabolic	PGS003200	EFO_0004631	vitamin D measurement	vitamin D measurement	EFO_0004631	Lipid or lipoprotein measurement	Metabolic
0.7314	1.16E-77	1.27E-74	PGS000411	EFO_0000756	melanoma	melanoma	EFO_0000756	Cancer	Neoplasms	PGS000410	EFO_0000756	melanoma	melanoma	EFO_0000756	Cancer	Neoplasms
0.7314	1.16E-77	1.27E-74	PGS001180	EFO_0004713	FEV|FEC ratio	FEV|FEC ratio	EFO_0004713	Other measurement	Respiratory	PGS002326	EFO_0004713	FEV|FEC ratio	FEV|FEC ratio	EFO_0004713	Other measurement	Respiratory
0.7314	1.17E-77	1.28E-74	PGS002326	EFO_0004713	FEV|FEC ratio	FEV|FEC ratio	EFO_0004713	Other measurement	Respiratory	PGS001010	EFO_0009718	peak expiratory flow	peak expiratory flow	EFO_0009718	Other measurement	Respiratory
0.7314	1.19E-77	1.31E-74	PGS001158	EFO_0005106	body composition measurement	body composition measurement	EFO_0005106	Body measurement	Body measurement	PGS002202	EFO_0009805	whole body water mass	whole body water mass	EFO_0009805	Other measurement	Body measurement
0.7314	1.20E-77	1.32E-74	PGS003142	EFO_0004574	total cholesterol measurement	total cholesterol measurement	EFO_0004574	Lipid or lipoprotein measurement	Metabolic	PGS003141	EFO_0004574	total cholesterol measurement	total cholesterol measurement	EFO_0004574	Lipid or lipoprotein measurement	Metabolic
0.7313	1.25E-77	1.37E-74	PGS000551	EFO_0003893	ovarian neoplasm	ovarian neoplasm	EFO_0003893	Cancer	Neoplasms	PGS000068	EFO_0001075	ovarian carcinoma	ovarian carcinoma	EFO_0001075	Cancer	Neoplasms
0.7313	1.27E-77	1.39E-74	PGS001165	EFO_0004995	lean body mass	lean body mass	EFO_0004995	Other measurement	Skeletal	PGS002202	EFO_0009805	whole body water mass	whole body water mass	EFO_0009805	Other measurement	Body measurement
0.7313	1.27E-77	1.39E-74	PGS002603	EFO_0000537	hypertension	hypertension	EFO_0000537	Cardiovascular disease	Cardiovascular	PGS002541	EFO_0006336	diastolic blood pressure	diastolic blood pressure	EFO_0006336	Other measurement	Cardiovascular
0.7313	1.27E-77	1.40E-74	PGS001145	EFO_0005409	fat body mass	fat body mass	EFO_0005409	Other measurement	Body measurement	PGS001103	EFO_0007800	body fat percentage	body fat percentage	EFO_0007800	Body measurement	Body measurement
0.7313	1.27E-77	1.40E-74	PGS001686	EFO_0006930	brain volume measurement|neuroimaging measurement	brain volume measurement	EFO_0006930	Other measurement	Neurological	PGS001461	EFO_0006930	measurement	brain volume measurement	EFO_0006930	Other measurement	Neurological
0.7313	1.29E-77	1.41E-74	PGS000773	EFO_0000305	breast carcinoma	breast carcinoma	EFO_0000305	Cancer	Neoplasms	PGS000506	EFO_0000305	breast carcinoma	breast carcinoma	EFO_0000305	Cancer	Neoplasms
0.7313	1.29E-77	1.41E-74	PGS000773	EFO_0000305	breast carcinoma	breast carcinoma	EFO_0000305	Cancer	Neoplasms	PGS000505	EFO_0000305	breast carcinoma	breast carcinoma	EFO_0000305	Cancer	Neoplasms
0.7311	1.41E-77	1.54E-74	PGS001166	EFO_0004995	lean body mass	lean body mass	EFO_0004995	Other measurement	Skeletal	PGS002168	EFO_0004995	lean body mass	lean body mass	EFO_0004995	Other measurement	Skeletal
0.7311	1.48E-77	1.62E-74	PGS002859	EFO_0004340	body mass index	body mass index	EFO_0004340	Body measurement	Body measurement	PGS002133	EFO_0007800	body fat percentage	body fat percentage	EFO_0007800	Body measurement	Body measurement
0.731	1.49E-77	1.64E-74	PGS000023	MONDO_0005147	type 1 diabetes mellitus	type 1 diabetes mellitus	MONDO_0005147	Digestive system disorder	Gastrointestinal	PGS001014	EFO_0009605	pancreas disease	pancreas disease	EFO_0009605	Digestive system disorder	Gastrointestinal
0.731	1.49E-77	1.64E-74	PGS000023	MONDO_0005147	type 1 diabetes mellitus	type 1 diabetes mellitus	MONDO_0005147	Immune system disorder	Immunological	PGS001014	EFO_0009605	pancreas disease	pancreas disease	EFO_0009605	Digestive system disorder	Gastrointestinal
0.731	1.49E-77	1.64E-74	PGS000023	MONDO_0005147	type 1 diabetes mellitus	type 1 diabetes mellitus	MONDO_0005147	Metabolic disorder	Metabolic	PGS001014	EFO_0009605	pancreas disease	pancreas disease	EFO_0009605	Digestive system disorder	Gastrointestinal
0.731	1.50E-77	1.64E-74	PGS003189	EFO_0004631	vitamin D measurement	vitamin D measurement	EFO_0004631	Lipid or lipoprotein measurement	Metabolic	PGS003191	EFO_0004631	vitamin D measurement	vitamin D measurement	EFO_0004631	Lipid or lipoprotein measurement	Metabolic
0.731	1.59E-77	1.74E-74	PGS000987	EFO_0010701	mean reticulocyte volume	mean reticulocyte volume	EFO_0010701	Hematological measurement	Hematological	PGS002227	EFO_0004503	hematological measurement	hematological measurement	EFO_0004503	Hematological measurement	Hematological

0.7309	1.61E-77	1.76E-74	PGS002774	EFO_0000275	atrial fibrillation	atrial fibrillation	EFO_0000275	Cardiovascular disease	Cardiovascular	PGS000016	EFO_0000275	atrial fibrillation	atrial fibrillation	EFO_0000275	Cardiovascular disease	Cardiovascular
0.7309	1.65E-77	1.81E-74	PGS003185	EFO_0004631	vitamin D measurement	vitamin D measurement	EFO_0004631	Lipid or lipoprotein measurement	Metabolic	PGS003195	EFO_0004631	vitamin D measurement	vitamin D measurement	EFO_0004631	Lipid or lipoprotein measurement	Metabolic
0.7309	1.68E-77	1.84E-74	PGS000747	EFO_0001645	coronary artery disease	coronary artery disease	EFO_0001645	Cardiovascular disease	Cardiovascular	PGS001780	EFO_0001645	coronary artery disease	coronary artery disease	EFO_0001645	Cardiovascular disease	Cardiovascular
0.7309	1.70E-77	1.86E-74	PGS002970	EFO_0004339	body height	body height	EFO_0004339	Body measurement	Body measurement	PGS002453	EFO_0004339	body height	body height	EFO_0004339	Body measurement	Body measurement
0.7308	1.74E-77	1.90E-74	PGS003194	EFO_0004631	vitamin D measurement	vitamin D measurement	EFO_0004631	Lipid or lipoprotein measurement	Metabolic	PGS002574	EFO_0004631	vitamin D measurement	vitamin D measurement	EFO_0004631	Lipid or lipoprotein measurement	Metabolic
0.7308	1.75E-77	1.91E-74	PGS003399	EFO_0000305	breast carcinoma	breast carcinoma	EFO_0000305	Cancer	Neoplasms	PGS000005	EFO_1000649	estrogen-receptor positive breast cancer	cancer	EFO_1000649	Cancer	Neoplasms
0.7307	1.96E-77	2.14E-74	PGS000525	EFO_0000305	breast carcinoma	breast carcinoma	EFO_0000305	Cancer	Neoplasms	PGS000537	EFO_0000305	breast carcinoma	breast carcinoma	EFO_0000305	Cancer	Neoplasms
0.7307	1.96E-77	2.14E-74	PGS000537	EFO_0000305	breast carcinoma	breast carcinoma	EFO_0000305	Cancer	Neoplasms	PGS000526	EFO_0000305	breast carcinoma	breast carcinoma	EFO_0000305	Cancer	Neoplasms
0.7307	1.98E-77	2.17E-74	PGS001476	EFO_0006930	brain volume measurement|neuroimaging measurement	brain volume measurement	EFO_0006930	Other measurement	Neurological	PGS001472	EFO_0006930	measurement	brain volume measurement	EFO_0006930	Other measurement	Neurological
0.7306	2.05E-77	2.24E-74	PGS002840	EFO_0004340	body mass index	body mass index	EFO_0004340	Body measurement	Body measurement	PGS002846	EFO_0004340	body mass index	body mass index	EFO_0004340	Body measurement	Body measurement
0.7306	2.05E-77	2.24E-74	PGS000921	EFO_0004340	body mass index	body mass index	EFO_0004340	Body measurement	Body measurement	PGS002201	EFO_0004342	waist circumference	waist circumference	EFO_0004342	Body measurement	Body measurement
0.7306	2.05E-77	2.24E-74	PGS000170	EFO_0007986	reticulocyte count	reticulocyte count	EFO_0007986	Hematological measurement	Hematological	PGS000094	EFO_0007986	reticulocyte count	reticulocyte count	EFO_0007986	Hematological measurement	Hematological
0.7306	2.08E-77	2.27E-74	PGS003147	EFO_0004530	triglyceride measurement	triglyceride measurement	EFO_0004530	Lipid or lipoprotein measurement	Metabolic	PGS003151	EFO_0004530	triglyceride measurement	triglyceride measurement	EFO_0004530	Lipid or lipoprotein measurement	Metabolic
0.7306	2.10E-77	2.29E-74	PGS001480	EFO_0006930	brain volume measurement|neuroimaging measurement	brain volume measurement	EFO_0006930	Other measurement	Neurological	PGS001681	EFO_0006930	measurement	brain volume measurement	EFO_0006930	Other measurement	Neurological
0.7306	2.11E-77	2.30E-74	PGS000482	EFO_0000305	breast carcinoma	breast carcinoma	EFO_0000305	Cancer	Neoplasms	PGS001337	EFO_0005606	family history of breast cancer	family history of breast cancer	EFO_0005606	Other trait	Neoplasms
0.7306	2.12E-77	2.31E-74	PGS000888	EFO_0004611	low density lipoprotein cholesterol measurement	low density lipoprotein cholesterol measurement	EFO_0004611	Cardiovascular measurement	Cardiovascular	PGS002556	EFO_0004611	low density lipoprotein cholesterol measurement	measurement	EFO_0004611	Cardiovascular measurement	Cardiovascular
0.7306	2.12E-77	2.31E-74	PGS000888	EFO_0004611	low density lipoprotein cholesterol measurement	low density lipoprotein cholesterol measurement	EFO_0004611	Cardiovascular measurement	Cardiovascular	PGS002556	EFO_0004611	low density lipoprotein cholesterol measurement	measurement	EFO_0004611	Lipid or lipoprotein measurement	Metabolic
0.7306	2.12E-77	2.31E-74	PGS000888	EFO_0004611	low density lipoprotein cholesterol measurement	low density lipoprotein cholesterol measurement	EFO_0004611	Lipid or lipoprotein measurement	Metabolic	PGS002556	EFO_0004611	low density lipoprotein cholesterol measurement	measurement	EFO_0004611	Lipid or lipoprotein measurement	Metabolic
0.7306	2.12E-77	2.31E-74	PGS000888	EFO_0004611	low density lipoprotein cholesterol measurement	low density lipoprotein cholesterol measurement	EFO_0004611	Lipid or lipoprotein measurement	Metabolic	PGS002556	EFO_0004611	low density lipoprotein cholesterol measurement	measurement	EFO_0004611	Cardiovascular measurement	Cardiovascular
-0.7306	2.12E-77	2.32E-74	PGS001819	EFO_0003770	diabetic retinopathy	diabetic retinopathy	EFO_0003770	Neurological disorder	Neurological	PGS001371	EFO_0004918	age at diagnosis|diabetes mellitus	age at diagnosis	EFO_0004918	Other measurement	Metabolic
-0.7306	2.12E-77	2.32E-74	PGS001819	EFO_0003770	diabetic retinopathy	diabetic retinopathy	EFO_0003770	Cardiovascular disease	Cardiovascular	PGS001371	EFO_0004918	age at diagnosis|diabetes mellitus	age at diagnosis	EFO_0004918	Other measurement	Metabolic
-0.7306	2.12E-77	2.32E-74	PGS001819	EFO_0003770	diabetic retinopathy	diabetic retinopathy	EFO_0003770	Metabolic disorder	Metabolic	PGS001371	EFO_0004918	age at diagnosis|diabetes mellitus	age at diagnosis	EFO_0004918	Other measurement	Metabolic
0.7305	2.20E-77	2.40E-74	PGS002170	EFO_0004468	glucose measurement	glucose measurement	EFO_0004468	Other measurement	Metabolic	PGS002943	EFO_0004468	glucose measurement	glucose measurement	EFO_0004468	Other measurement	Metabolic
0.7303	2.49E-77	2.71E-74	PGS002427	EFO_0004631	vitamin D measurement	vitamin D measurement	EFO_0004631	Lipid or lipoprotein measurement	Metabolic	PGS003193	EFO_0004631	vitamin D measurement	vitamin D measurement	EFO_0004631	Lipid or lipoprotein measurement	Metabolic
0.7303	2.64E-77	2.87E-74	PGS003191	EFO_0004631	vitamin D measurement	vitamin D measurement	EFO_0004631	Lipid or lipoprotein measurement	Metabolic	PGS001982	EFO_0004631	vitamin D measurement	vitamin D measurement	EFO_0004631	Lipid or lipoprotein measurement	Metabolic
-0.7302	2.71E-77	2.95E-74	PGS000471	MONDO_0004641	skin carcinoma in situ	skin carcinoma in situ	MONDO_0004641	Cancer	Neoplasms	PGS001535	EFO_0010729	sun exposure measurement	sun exposure measurement	EFO_0010729	Other measurement	Activities
0.7302	2.82E-77	3.07E-74	PGS001158	EFO_0005106	body composition measurement	body composition measurement	EFO_0005106	Body measurement	Body measurement	PGS002168	EFO_0004995	lean body mass	lean body mass	EFO_0004995	Other measurement	Skeletal
0.7302	2.83E-77	3.08E-74	PGS000940	HP_0002024	Malabsorption	Malabsorption	HP_0002024	Other trait	Gastrointestinal	PGS002067	EFO_0001060	celiac disease	celiac disease	EFO_0001060	Immune system disorder	Immunological
0.7302	2.83E-77	3.08E-74	PGS000940	HP_0002024	Malabsorption	Malabsorption	HP_0002024	Other trait	Gastrointestinal	PGS002067	EFO_0001060	celiac disease	celiac disease	EFO_0001060	Digestive system disorder	Gastrointestinal
0.7301	3.00E-77	3.26E-74	PGS000001	EFO_0000305	breast carcinoma	breast carcinoma	EFO_0000305	Cancer	Neoplasms	PGS000538	EFO_0000305	breast carcinoma	breast carcinoma	EFO_0000305	Cancer	Neoplasms
0.73	3.07E-77	3.34E-74	PGS000956	EFO_0004514	bone quantitative ultrasound measurement	bone quantitative ultrasound measurement	EFO_0004514	Other measurement	Skeletal	PGS000951	EFO_0004514	bone quantitative ultrasound measurement	measurement	EFO_0004514	Other measurement	Skeletal
0.73	3.10E-77	3.37E-74	PGS000153	EFO_0000305	breast carcinoma	breast carcinoma	EFO_0000305	Cancer	Neoplasms	PGS000001	EFO_0000305	breast carcinoma	breast carcinoma	EFO_0000305	Cancer	Neoplasms
0.73	3.11E-77	3.38E-74	PGS001039	EFO_0009270	heel bone mineral density	heel bone mineral density	EFO_0009270	Other measurement	Skeletal	PGS000951	EFO_0004514	bone quantitative ultrasound measurement	measurement	EFO_0004514	Other measurement	Skeletal
0.7299	3.36E-77	3.66E-74	PGS003030	EFO_0004611	low density lipoprotein cholesterol measurement	low density lipoprotein cholesterol measurement	EFO_0004611	Cardiovascular measurement	Cardiovascular	PGS003037	EFO_0004611	low density lipoprotein cholesterol measurement	measurement	EFO_0004611	Lipid or lipoprotein measurement	Metabolic
0.7299	3.36E-77	3.66E-74	PGS003030	EFO_0004611	low density lipoprotein cholesterol measurement	low density lipoprotein cholesterol measurement	EFO_0004611	Cardiovascular measurement	Cardiovascular	PGS003037	EFO_0004611	low density lipoprotein cholesterol measurement	measurement	EFO_0004611	Cardiovascular measurement	Cardiovascular
0.7299	3.36E-77	3.66E-74	PGS003030	EFO_0004611	low density lipoprotein cholesterol measurement	low density lipoprotein cholesterol measurement	EFO_0004611	Lipid or lipoprotein measurement	Metabolic	PGS003037	EFO_0004611	low density lipoprotein cholesterol measurement	measurement	EFO_0004611	Cardiovascular measurement	Cardiovascular
0.7299	3.36E-77	3.66E-74	PGS003030	EFO_0004611	low density lipoprotein cholesterol measurement	low density lipoprotein cholesterol measurement	EFO_0004611	Lipid or lipoprotein measurement	Metabolic	PGS003037	EFO_0004611	low density lipoprotein cholesterol measurement	measurement	EFO_0004611	Lipid or lipoprotein measurement	Metabolic
0.7298	3.66E-77	3.98E-74	PGS001950	EFO_0004995	lean body mass	lean body mass	EFO_0004995	Other measurement	Skeletal	PGS001165	EFO_0004995	lean body mass	lean body mass	EFO_0004995	Other measurement	Skeletal
0.7298	3.76E-77	4.09E-74	PGS000538	EFO_0000305	breast carcinoma	breast carcinoma	EFO_0000305	Cancer	Neoplasms	PGS000008	EFO_1000649	estrogen-receptor positive breast cancer	cancer	EFO_1000649	Cancer	Neoplasms
0.7297	3.85E-77	4.19E-74	PGS001990	EFO_0004526	mean corpuscular volume	mean corpuscular volume	EFO_0004526	Hematological measurement	Hematological	PGS002227	EFO_0004503	hematological measurement	hematological measurement	EFO_0004503	Hematological measurement	Hematological
0.7297	3.89E-77	4.23E-74	PGS002993	EFO_0004339	body height	body height	EFO_0004339	Body measurement	Body measurement	PGS001929	EFO_0004339	body height	body height	EFO_0004339	Body measurement	Body measurement
0.7297	3.97E-77	4.31E-74	PGS001343	MONDO_0004979	asthma	asthma	MONDO_0004979	Other disease	Respiratory	PGS002311	MONDO_0004979	asthma	asthma	MONDO_0004979	Other disease	Respiratory
0.7297	3.97E-77	4.31E-74	PGS000188	EFO_0005192	red blood cell distribution width	red blood cell distribution width	EFO_0005192	Hematological measurement	Hematological	PGS002663	EFO_0005192	red blood cell distribution width	red blood cell distribution width	EFO_0005192	Hematological measurement	Hematological
0.7297	4.03E-77	4.38E-74	PGS002433	EFO_0005140	autoimmune disease	autoimmune disease	EFO_0005140	Immune system disorder	Immunological	PGS002465	EFO_0000676	psoriasis	psoriasis	EFO_0000676	Immune system disorder	Immunological
0.7296	4.08E-77	4.43E-74	PGS003283	EFO_0004617	cystatin C measurement	cystatin C measurement	EFO_0004617	Other measurement	Metabolic	PGS003284	EFO_0004617	cystatin C measurement	cystatin C measurement	EFO_0004617	Other measurement	Metabolic
0.7296	4.13E-77	4.49E-74	PGS001917	EFO_0007800	body fat percentage	body fat percentage	EFO_0007800	Body measurement	Body measurement	PGS001958	EFO_0005093	hip circumference	hip circumference	EFO_0005093	Body measurement	Body measurement
-0.7296	4.27E-77	4.64E-74	PGS002318	EFO_0008328	chronotype measurement	chronotype measurement	EFO_0008328	Other measurement	Psychiatric	PGS001992	EFO_0008328	chronotype measurement	chronotype measurement	EFO_0008328	Other measurement	Psychiatric
0.7295	4.52E-77	4.91E-74	PGS001146	EFO_0005409	fat body mass	fat body mass	EFO_0005409	Other measurement	Body measurement	PGS002168	EFO_0004995	lean body mass	lean body mass	EFO_0004995	Other measurement	Skeletal
0.7295	4.53E-77	4.91E-74	PGS002556	EFO_0004611	low density lipoprotein cholesterol measurement	low density lipoprotein cholesterol measurement	EFO_0004611	Cardiovascular measurement	Cardiovascular	PGS002654	EFO_0004611	low density lipoprotein cholesterol measurement	measurement	EFO_0004611	Lipid or lipoprotein measurement	Metabolic
0.7295	4.53E-77	4.91E-74	PGS002556	EFO_0004611	low density lipoprotein cholesterol measurement	low density lipoprotein cholesterol measurement	EFO_0004611	Cardiovascular measurement	Cardiovascular	PGS002654	EFO_0004611	low density lipoprotein cholesterol measurement	measurement	EFO_0004611	Cardiovascular measurement	Cardiovascular
0.7295	4.53E-77	4.91E-74	PGS002556	EFO_0004611	low density lipoprotein cholesterol measurement	low density lipoprotein cholesterol measurement	EFO_0004611	Lipid or lipoprotein measurement	Metabolic	PGS002654	EFO_0004611	low density lipoprotein cholesterol measurement	measurement	EFO_0004611	Cardiovascular measurement	Cardiovascular
0.7295	4.53E-77	4.91E-74	PGS002556	EFO_0004611	low density lipoprotein cholesterol measurement	low density lipoprotein cholesterol measurement	EFO_0004611	Lipid or lipoprotein measurement	Metabolic	PGS002654	EFO_0004611	low density lipoprotein cholesterol measurement	measurement	EFO_0004611	Lipid or lipoprotein measurement	Metabolic
-0.7294	4.88E-77	5.29E-74	PGS000173	EFO_0007993	lymphocyte percentage of leukocytes	lymphocyte percentage of leukocytes	EFO_0007993	Hematological measurement	Hematological	PGS000183	EFO_0007990	neutrophil percentage of leukocytes	neutrophil percentage of leukocytes	EFO_0007990	Hematological measurement	Hematological
0.7294	4.95E-77	5.37E-74	PGS003150	EFO_0004530	triglyceride measurement	triglyceride measurement	EFO_0004530	Lipid or lipoprotein measurement	Metabolic	PGS003144	EFO_0004530	triglyceride measurement	triglyceride measurement	EFO_0004530	Lipid or lipoprotein measurement	Metabolic
0.7294	4.96E-77	5.38E-74	PGS001952	EFO_0004468	glucose measurement	glucose measurement	EFO_0004468	Other measurement	Metabolic	PGS002934	EFO_0004468	glucose measurement	glucose measurement	EFO_0004468	Other measurement	Metabolic
0.7294	5.01E-77	5.43E-74	PGS001517	EFO_0007985	platelet crit	platelet crit	EFO_0007985	Hematological measurement	Hematological	PGS000107	EFO_0007985	platelet crit	platelet crit	EFO_0007985	Hematological measurement	Hematological
0.7293	5.19E-77	5.62E-74	PGS002785	MONDO_0005090	schizophrenia	schizophrenia	MONDO_0005090	Neurological disorder	Neurological	PGS000136	MONDO_0005090	schizophrenia	schizophrenia	MONDO_0005090	Neurological disorder	Neurological
0.7293	5.19E-77	5.62E-74	PGS002628	MONDO_0004979	asthma	asthma	MONDO_0004979	Other disease	Respiratory	PGS001344	MONDO_0004979	asthma	asthma	MONDO_0004979	Other disease	Respiratory
0.7293	5.24E-77	5.68E-74	PGS000528	EFO_0000305	breast carcinoma	breast carcinoma	EFO_0000305	Cancer	Neoplasms	PGS000507	EFO_0000305	breast carcinoma	breast carcinoma	EFO_0000305	Cancer	Neoplasms
0.7292	5.39E-77	5.83E-74	PGS001663	EFO_0006930	brain volume measurement|neuroimaging measurement	brain volume measurement	EFO_0006930	Other measurement	Neurological	PGS001671	EFO_0006930	measurement	brain volume measurement	EFO_0006930	Other measurement	Neurological
0.7292	5.69E-77	6.16E-74	PGS000900	EFO_0006335	systolic blood pressure|female	systolic blood pressure	EFO_0006335	Other measurement	Cardiovascular	PGS002349	EFO_0006335	systolic blood pressure	systolic blood pressure	EFO_0006335	Other measurement	Cardiovascular
0.7292	5.73E-77	6.20E-74	PGS001661	EFO_0006930	brain volume measurement|neuroimaging measurement	brain volume measurement	EFO_0006930	Other measurement	Neurological	PGS001680	EFO_0006930	measurement	brain volume measurement	EFO_0006930	Other measurement	Neurological
0.7292	5.75E-77	6.22E-74	PGS000090	EFO_0004842	eosinophil count	eosinophil count	EFO_0004842	Hematological measurement	Hematological	PGS001172	EFO_0004842	eosinophil count	eosinophil count	EFO_0004842	Hematological measurement	Hematological
0.7292	5.76E-77	6.23E-74	PGS000774	EFO_1000649	estrogen-receptor positive breast cancer	estrogen-receptor positive breast cancer	EFO_1000649	Cancer	Neoplasms	PGS000488	EFO_0000305	breast carcinoma	breast carcinoma	EFO_0000305	Cancer	Neoplasms
0.7291	5.86E-77	6.34E-74	PGS003189	EFO_0004631	vitamin D measurement	vitamin D measurement	EFO_0004631	Lipid or lipoprotein measurement	Metabolic	PGS001982	EFO_0004631	vitamin D measurement	vitamin D measurement	EFO_0004631	Lipid or lipoprotein measurement	Metabolic
-0.7291	5.97E-77	6.45E-74	PGS003224	EFO_0005208	glomerular filtration rate	glomerular filtration rate	EFO_0005208	Other measurement	Genitourinary	PGS003377	EFO_0005208	glomerular filtration rate	glomerular filtration rate	EFO_0005208	Other measurement	Genitourinary
0.729	6.22E-77	6.72E-74	PGS002049	MONDO_0004596	cor pulmonale	cor pulmonale	MONDO_0004596	Cardiovascular disease	Cardiovascular	PGS001277	EFO_0003827	pulmonary embolism|deep vein thrombosis	pulmonary embolism	EFO_0003827	Cardiovascular disease	Cardiovascular
0.729	6.32E-77	6.83E-74	PGS000731	EFO_0000707	squamous cell carcinoma	squamous cell carcinoma	EFO_0000707	Cancer	Neoplasms	PGS001072	EFO_0008006	skin aging measurement	skin aging measurement	EFO_0008006	Other measurement	Dermatological
0.729	6.40E-77	6.92E-74	PGS000378	EFO_1001950	colon carcinoma	colon carcinoma	EFO_1001950	Digestive system disorder	Gastrointestinal	PGS000376	EFO_1001950	colon carcinoma	colon carcinoma	EFO_1001950	Cancer	Neoplasms
0.729	6.40E-77	6.92E-74	PGS000378	EFO_1001950	colon carcinoma	colon carcinoma	EFO_1001950	Cancer	Neoplasms	PGS000376	EFO_1001950	colon carcinoma	colon carcinoma	EFO_1001950	Digestive system disorder	Gastrointestinal
0.729	6.40E-77	6.92E-74	PGS000378	EFO_1001950	colon carcinoma	colon carcinoma	EFO_1001950	Digestive system disorder	Gastrointestinal	PGS000376	EFO_1001950	colon carcinoma	colon carcinoma	EFO_1001950	Digestive system disorder	Gastrointestinal
0.729	6.40E-77	6.92E-74	PGS000378	EFO_1001950	colon carcinoma	colon carcinoma	EFO_1001950	Cancer	Neoplasms	PGS000376	EFO_1001950	colon carcinoma	colon carcinoma	EFO_1001950	Cancer	Neoplasms
0.729	6.58E-77	7.11E-74	PGS002024	EFO_0004705	hypothyroidism	hypothyroidism	EFO_0004705	Other disease	Endocrine	PGS002351	EFO_1000627	thyroid disease	thyroid disease	EFO_1000627	Other disease	Endocrine
0.729	6.63E-77	7.16E-74	PGS000499	EFO_0000305	breast carcinoma	breast carcinoma	EFO_0000305	Cancer	Neoplasms	PGS000531	EFO_0000305	breast carcinoma	breast carcinoma	EFO_0000305	Cancer	Neoplasms
0.7289	6.96E-77	7.52E-74	PGS001244	EFO_0004279	suntan	suntan	EFO_0004279	Other trait	Dermatological	PGS001257	EFO_0003958	sunburn	sunburn	EFO_0003958	Other disease	Dermatological
0.7288	7.18E-77	7.76E-74	PGS002169	EFO_0005409	fat body mass	fat body mass	EFO_0005409	Other measurement	Body measurement	PGS000921	EFO_0004340	body mass index	body mass index	EFO_0004340	Body measurement	Body measurement
0.7288	7.35E-77	7.93E-74	PGS003198	EFO_0004631	vitamin D measurement	vitamin D measurement	EFO_0004631	Lipid or lipoprotein measurement	Metabolic	PGS002574	EFO_0004631	vitamin D measurement	vitamin D measurement	EFO_0004631	Lipid or lipoprotein measurement	Metabolic
0.7287	7.98E-77	8.61E-74	PGS003184	EFO_0004631	vitamin D measurement	vitamin D measurement	EFO_0004631	Lipid or lipoprotein measurement	Metabolic	PGS000702	EFO_0004631	vitamin D measurement	vitamin D measurement	EFO_0004631	Lipid or lipoprotein measurement	Metabolic
0.7287	8.03E-77	8.67E-74	PGS000049	EFO_0001663	prostate carcinoma	prostate carcinoma	EFO_0001663	Cancer	Neoplasms	PGS002791	EFO_0001663	prostate carcinoma	prostate carcinoma	EFO_0001663	Cancer	Neoplasms
0.7287	8.14E-77	8.78E-74	PGS002850	EFO_0004340	body mass index	body mass index	EFO_0004340	Body measurement	Body measurement	PGS002841	EFO_0004340	body mass index	body mass index	EFO_0004340	Body measurement	Body measurement
0.7287	8.20E-77	8.84E-74	PGS000956	EFO_0004514	bone quantitative ultrasound measurement	bone quantitative ultrasound measurement	EFO_0004514	Other measurement	Skeletal	PGS001529	EFO_0007772	measurement	measurement	EFO_0007772	Other measurement	Skeletal
0.7287	8.21E-77	8.85E-74	PGS002337	EFO_0004611	low density lipoprotein cholesterol measurement	low density lipoprotein cholesterol measurement	EFO_0004611	Cardiovascular measurement	Cardiovascular	PGS000889	EFO_0004611	low density lipoprotein cholesterol measurement	measurement	EFO_0004611	Cardiovascular measurement	Cardiovascular
0.7287	8.21E-77	8.85E-74	PGS002337	EFO_0004611	low density lipoprotein cholesterol measurement	low density lipoprotein cholesterol measurement	EFO_0004611	Lipid or lipoprotein measurement	Metabolic	PGS000889	EFO_0004611	low density lipoprotein cholesterol measurement	measurement	EFO_0004611	Lipid or lipoprotein measurement	Metabolic
0.7287	8.21E-77	8.85E-74	PGS002337	EFO_0004611	low density lipoprotein cholesterol measurement	low density lipoprotein cholesterol measurement	EFO_0004611	Lipid or lipoprotein measurement	Metabolic	PGS000889	EFO_0004611	low density lipoprotein cholesterol measurement	measurement	EFO_0004611	Cardiovascular measurement	Cardiovascular
0.7287	8.21E-77	8.85E-74	PGS002337	EFO_0004611	low density lipoprotein cholesterol measurement	low density lipoprotein cholesterol measurement	EFO_0004611	Cardiovascular measurement	Cardiovascular	PGS000889	EFO_0004611	low density lipoprotein cholesterol measurement	measurement	EFO_0004611	Lipid or lipoprotein measurement	Metabolic
0.7287	8.21E-77	8.85E-74	PGS001039	EFO_0009270	heel bone mineral density	heel bone mineral density	EFO_0009270	Other measurement	Skeletal	PGS001529	EFO_0007772	measurement	measurement	EFO_0007772	Other measurement	Skeletal
0.7286	8.29E-77	8.93E-74	PGS002780	MONDO_0005148	type 2 diabetes mellitus	type 2 diabetes mellitus	MONDO_0005148	Digestive system disorder	Gastrointestinal	PGS003104	MONDO_0005148	type 2 diabetes mellitus	type 2 diabetes mellitus	MONDO_0005148	Metabolic disorder	Metabolic
0.7286	8.29E-77	8.93E-74	PGS002780	MONDO_0005148	type 2 diabetes mellitus	type 2 diabetes mellitus	MONDO_0005148	Metabolic disorder	Metabolic	PGS003104	MONDO_0005148	type 2 diabetes mellitus	type 2 diabetes mellitus	MONDO_0005148	Metabolic disorder	Metabolic
0.7286	8.29E-77	8.93E-74	PGS002780	MONDO_0005148	type 2 diabetes mellitus	type 2 diabetes mellitus	MONDO_0005148	Metabolic disorder	Metabolic	PGS003104	MONDO_0005148	type 2 diabetes mellitus	type 2 diabetes mellitus	MONDO_0005148	Digestive system disorder	Gastrointestinal
0.7286	8.29E-77	8.93E-74	PGS002780	MONDO_0005148	type 2 diabetes mellitus	type 2 diabetes mellitus	MONDO_0005148	Digestive system disorder	Gastrointestinal	PGS003104	MONDO_0005148	type 2 diabetes mellitus	type 2 diabetes mellitus	MONDO_0005148	Digestive system disorder	Gastrointestinal
0.7286	8.42E-77	9.07E-74	PGS001778	EFO_0000305	breast carcinoma	breast carcinoma	EFO_0000305	Cancer	Neoplasms	PGS000472	EFO_0000305	breast carcinoma	breast carcinoma	EFO_0000305	Cancer	Neoplasms
0.7285	9.48E-77	1.02E-73	PGS000018	EFO_0001645	coronary artery disease	coronary artery disease	EFO_0001645	Cardiovascular disease	Cardiovascular	PGS002776	EFO_0001645	coronary artery disease	coronary artery disease	EFO_0001645	Cardiovascular disease	Cardiovascular
0.7284	9.58E-77	1.03E-73	PGS000528	EFO_0000305	breast carcinoma	breast carcinoma	EFO_0000305	Cancer	Neoplasms	PGS000335	EFO_0000305	breast carcinoma	breast carcinoma	EFO_0000305	Cancer	Neoplasms
0.7284	9.79E-77	1.05E-73	PGS002049	MONDO_0004596	cor pulmonale	cor pulmonale	MONDO_0004596	Cardiovascular disease	Cardiovascular	PGS001847	EFO_0004264	vascular disease	vascular disease	EFO_0004264	Cardiovascular disease	Cardiovascular
0.7284	1.01E-76	1.09E-73	PGS000310	EFO_0004611	low density lipoprotein cholesterol measurement	low density lipoprotein cholesterol measurement	EFO_0004611	Cardiovascular measurement	Cardiovascular	PGS000895	EFO_0004611	low density lipoprotein cholesterol measurement	measurement	EFO_0004611	Lipid or lipoprotein measurement	Metabolic
0.7284	1.01E-76	1.09E-73	PGS000310	EFO_0004611	low density lipoprotein cholesterol measurement	low density lipoprotein cholesterol measurement	EFO_0004611	Lipid or lipoprotein measurement	Metabolic	PGS000895	EFO_0004611	low density lipoprotein cholesterol measurement	measurement	EFO_0004611	Lipid or lipoprotein measurement	Metabolic
0.7284	1.01E-76	1.09E-73	PGS000310	EFO_0004611	low density lipoprotein cholesterol measurement	low density lipoprotein cholesterol measurement	EFO_0004611	Lipid or lipoprotein measurement	Metabolic	PGS000895	EFO_0004611	low density lipoprotein cholesterol measurement	measurement	EFO_0004611	Cardiovascular measurement	Cardiovascular
0.7284	1.01E-76	1.09E-73	PGS000310	EFO_0004611	low density lipoprotein cholesterol measurement	low density lipoprotein cholesterol measurement	EFO_0004611	Cardiovascular measurement	Cardiovascular	PGS000895	EFO_0004611	low density lipoprotein cholesterol measurement	measurement	EFO_0004611	Cardiovascular measurement	Cardiovascular
0.7284	1.02E-76	1.10E-73	PGS002172	EFO_0004612	high density lipoprotein cholesterol measurement	high density lipoprotein cholesterol measurement	EFO_0004612	Lipid or lipoprotein measurement	Metabolic	PGS000686	EFO_0004612	high density lipoprotein cholesterol measurement	measurement	EFO_0004612	Lipid or lipoprotein measurement	Metabolic
0.7283	1.06E-76	1.14E-73	PGS003199	EFO_0004631	vitamin D measurement	vitamin D measurement	EFO_0004631	Lipid or lipoprotein measurement	Metabolic	PGS000702	EFO_0004631	vitamin D measurement	vitamin D measurement	EFO_0004631	Lipid or lipoprotein measurement	Metabolic
0.7283	1.06E-76	1.14E-73	PGS002015	EFO_0000305	breast carcinoma	breast carcinoma	EFO_0000305	Cancer	Neoplasms	PGS001336	EFO_0000305	breast carcinoma	breast carcinoma	EFO_0000305	Cancer	Neoplasms
0.7283	1.08E-76	1.16E-73	PGS002501	EFO_0004541	HbA1c measurement	HbA1c measurement	EFO_0004541	Hematological measurement	Hematological	PGS002449	EFO_0004468	glucose measurement	glucose measurement	EFO_0004468	Other measurement	Metabolic
0.7282	1.09E-76	1.18E-73	PGS002502	EFO_0004339	body height	body height	EFO_0004339	Body measurement	Body measurement	PGS002600	EFO_0004339	body height	body height	EFO_0004339	Body measurement	Body measurement

0.7282	1.13E-76	1.21E-73	PGS001103	EFO_0007800	body fat percentage	body fat percentage	EFO_0007800	Body measurement	Body measurement	PGS001144	EFO_0005409	fat body mass	fat body mass	EFO_0005409	Other measurement	Body measurement
0.7281	1.19E-76	1.27E-73	PGS001165	EFO_0004995	lean body mass	lean body mass	EFO_0004995	Other measurement	Skeletal	PGS002168	EFO_0004995	lean body mass	lean body mass	EFO_0004995	Other measurement	Skeletal
0.7281	1.19E-76	1.28E-73	PGS000532	EFO_0000305	breast carcinoma	breast carcinoma	EFO_0000305	Cancer	Neoplasms	PGS000008	EFO_1000649	estrogen-receptor positive breast cancer	cancer	EFO_1000649	Cancer	Neoplasms
0.7281	1.20E-76	1.29E-73	PGS000507	EFO_0000305	breast carcinoma	breast carcinoma	EFO_0000305	Cancer	Neoplasms	PGS000540	EFO_0000305	breast carcinoma	breast carcinoma	EFO_0000305	Cancer	Neoplasms
0.728	1.27E-76	1.36E-73	PGS000536	EFO_0000305	breast carcinoma	breast carcinoma	EFO_0000305	Cancer	Neoplasms	PGS000531	EFO_0000305	breast carcinoma	breast carcinoma	EFO_0000305	Cancer	Neoplasms
0.728	1.27E-76	1.37E-73	PGS000476	EFO_0000305	breast carcinoma	breast carcinoma	EFO_0000305	Cancer	Neoplasms	PGS001336	EFO_0000305	breast carcinoma	breast carcinoma	EFO_0000305	Cancer	Neoplasms
0.728	1.35E-76	1.45E-73	PGS000513	EFO_0000305	breast carcinoma	breast carcinoma	EFO_0000305	Cancer	Neoplasms	PGS001337	EFO_0005606	family history of breast cancer	family history of breast cancer	EFO_0005606	Other trait	Neoplasms
0.7279	1.36E-76	1.46E-73	PGS002049	MONDO_0004596	cor pulmonale	cor pulmonale	MONDO_0004596	Cardiovascular disease	Cardiovascular	PGS001844	EFO_1001395	Phlebitis|Thrombophlebitis	Phlebitis	EFO_1001395	Cardiovascular disease	Cardiovascular
0.7279	1.42E-76	1.53E-73	PGS000738	EFO_0004208	Vitiligo	Vitiligo	EFO_0004208	Immune system disorder	Immunological	PGS000760	EFO_0004208	Vitiligo	Vitiligo	EFO_0004208	Immune system disorder	Immunological
0.7279	1.43E-76	1.53E-73	PGS002964	EFO_0004339	body height	body height	EFO_0004339	Body measurement	Body measurement	PGS002971	EFO_0004339	body height	body height	EFO_0004339	Body measurement	Body measurement
0.7279	1.44E-76	1.54E-73	PGS002672	EFO_0004631	vitamin D measurement	vitamin D measurement	EFO_0004631	Lipid or lipoprotein measurement	Metabolic	PGS003185	EFO_0004631	vitamin D measurement	vitamin D measurement	EFO_0004631	Lipid or lipoprotein measurement	Metabolic
0.7279	1.44E-76	1.55E-73	PGS001805	EFO_0001663	prostate carcinoma	prostate carcinoma	EFO_0001663	Cancer	Neoplasms	PGS000795	EFO_0001663	prostate carcinoma	prostate carcinoma	EFO_0001663	Cancer	Neoplasms
0.7279	1.45E-76	1.55E-73	PGS000097	EFO_0004587	lymphocyte count	lymphocyte count	EFO_0004587	Hematological measurement	Hematological	PGS002338	EFO_0004587	lymphocyte count	lymphocyte count	EFO_0004587	Hematological measurement	Hematological
0.7278	1.45E-76	1.56E-73	PGS001990	EFO_0004526	mean corpuscular volume	mean corpuscular volume	EFO_0004526	Hematological measurement	Hematological	PGS002411	EFO_0004527	mean corpuscular hemoglobin	mean corpuscular hemoglobin	EFO_0004527	Hematological measurement	Hematological
0.7278	1.46E-76	1.57E-73	PGS002628	MONDO_0004979	asthma	asthma	MONDO_0004979	Other disease	Respiratory	PGS002347	EFO_0000684	respiratory system disease	respiratory system disease	EFO_0000684	Other disease	Skeletal
0.7278	1.51E-76	1.62E-73	PGS001488	EFO_0006930	brain volume measurement|neuroimaging measurement	brain volume measurement	EFO_0006930	Other measurement	Neurological	PGS001671	EFO_0006930	measurement	brain volume measurement	EFO_0006930	Other measurement	Neurological
0.7278	1.51E-76	1.62E-73	PGS000961	EFO_1001395	Phlebitis|Thrombophlebitis	Phlebitis	EFO_1001395	Cardiovascular disease	Cardiovascular	PGS001277	EFO_0003827	pulmonary embolism|deep vein thrombosis	pulmonary embolism	EFO_0003827	Cardiovascular disease	Cardiovascular
0.7277	1.57E-76	1.68E-73	PGS003254	EFO_0010472	creatine measurement|glomerular filtration rate	creatine measurement	EFO_0010472	Other measurement	Metabolic	PGS003270	EFO_0010472	creatine measurement|glomerular filtration rate	creatine measurement	EFO_0010472	Other measurement	Metabolic
0.7277	1.59E-76	1.70E-73	PGS002351	EFO_1000627	thyroid disease	thyroid disease	EFO_1000627	Other disease	Endocrine	PGS000761	EFO_0004705	hypothyroidism	hypothyroidism	EFO_0004705	Other disease	Endocrine
0.7277	1.64E-76	1.75E-73	PGS002481	MONDO_0004979	asthma	asthma	MONDO_0004979	Other disease	Respiratory	PGS002468	EFO_0000684	respiratory system disease	respiratory system disease	EFO_0000684	Other disease	Skeletal
0.7277	1.67E-76	1.79E-73	PGS002200	EFO_0004631	vitamin D measurement	vitamin D measurement	EFO_0004631	Lipid or lipoprotein measurement	Metabolic	PGS003191	EFO_0004631	vitamin D measurement	vitamin D measurement	EFO_0004631	Lipid or lipoprotein measurement	Metabolic
0.7276	1.69E-76	1.81E-73	PGS002322	EFO_0006336	diastolic blood pressure	diastolic blood pressure	EFO_0006336	Other measurement	Cardiovascular	PGS002349	EFO_0006335	systolic blood pressure	systolic blood pressure	EFO_0006335	Other measurement	Cardiovascular
0.7276	1.70E-76	1.82E-73	PGS002311	MONDO_0004979	asthma	asthma	MONDO_0004979	Other disease	Respiratory	PGS002664	EFO_0000684	respiratory system disease	respiratory system disease	EFO_0000684	Other disease	Skeletal
0.7276	1.72E-76	1.84E-73	PGS003184	EFO_0004631	vitamin D measurement	vitamin D measurement	EFO_0004631	Lipid or lipoprotein measurement	Metabolic	PGS001982	EFO_0004631	vitamin D measurement	vitamin D measurement	EFO_0004631	Lipid or lipoprotein measurement	Metabolic
0.7276	1.79E-76	1.91E-73	PGS003142	EFO_0004574	total cholesterol measurement	total cholesterol measurement	EFO_0004574	Lipid or lipoprotein measurement	Metabolic	PGS003136	EFO_0004574	total cholesterol measurement	total cholesterol measurement	EFO_0004574	Lipid or lipoprotein measurement	Metabolic
0.7275	1.80E-76	1.93E-73	PGS002409	EFO_0004611	low density lipoprotein cholesterol measurement	low density lipoprotein cholesterol measurement	EFO_0004611	Lipid or lipoprotein measurement	Metabolic	PGS000824	EFO_0004611	low density lipoprotein cholesterol measurement	measurement	EFO_0004611	Cardiovascular measurement	Cardiovascular
0.7275	1.80E-76	1.93E-73	PGS002409	EFO_0004611	low density lipoprotein cholesterol measurement	low density lipoprotein cholesterol measurement	EFO_0004611	Cardiovascular measurement	Cardiovascular	PGS000824	EFO_0004611	low density lipoprotein cholesterol measurement	measurement	EFO_0004611	Lipid or lipoprotein measurement	Metabolic
0.7275	1.80E-76	1.93E-73	PGS002409	EFO_0004611	low density lipoprotein cholesterol measurement	low density lipoprotein cholesterol measurement	EFO_0004611	Cardiovascular measurement	Cardiovascular	PGS000824	EFO_0004611	low density lipoprotein cholesterol measurement	measurement	EFO_0004611	Cardiovascular measurement	Cardiovascular
0.7275	1.80E-76	1.93E-73	PGS002409	EFO_0004611	low density lipoprotein cholesterol measurement	low density lipoprotein cholesterol measurement	EFO_0004611	Lipid or lipoprotein measurement	Metabolic	PGS000824	EFO_0004611	low density lipoprotein cholesterol measurement	measurement	EFO_0004611	Lipid or lipoprotein measurement	Metabolic
0.7275	1.81E-76	1.93E-73	PGS002322	EFO_0006336	diastolic blood pressure	diastolic blood pressure	EFO_0006336	Other measurement	Cardiovascular	PGS001900	EFO_0006336	diastolic blood pressure	diastolic blood pressure	EFO_0006336	Other measurement	Cardiovascular
0.7274	1.93E-76	2.06E-73	PGS000532	EFO_0000305	breast carcinoma	breast carcinoma	EFO_0000305	Cancer	Neoplasms	PGS000499	EFO_0000305	breast carcinoma	breast carcinoma	EFO_0000305	Cancer	Neoplasms
0.7274	1.94E-76	2.07E-73	PGS000097	EFO_0004587	lymphocyte count	lymphocyte count	EFO_0004587	Hematological measurement	Hematological	PGS001965	EFO_0004587	lymphocyte count	lymphocyte count	EFO_0004587	Hematological measurement	Hematological
0.7274	1.95E-76	2.08E-73	PGS003030	EFO_0004611	low density lipoprotein cholesterol measurement	low density lipoprotein cholesterol measurement	EFO_0004611	Cardiovascular measurement	Cardiovascular	PGS002620	EFO_0004574	total cholesterol measurement	total cholesterol measurement	EFO_0004574	Lipid or lipoprotein measurement	Metabolic
0.7274	1.95E-76	2.08E-73	PGS003030	EFO_0004611	low density lipoprotein cholesterol measurement	low density lipoprotein cholesterol measurement	EFO_0004611	Lipid or lipoprotein measurement	Metabolic	PGS002620	EFO_0004574	total cholesterol measurement	total cholesterol measurement	EFO_0004574	Lipid or lipoprotein measurement	Metabolic
0.7274	1.96E-76	2.09E-73	PGS003145	EFO_0004530	triglyceride measurement	triglyceride measurement	EFO_0004530	Lipid or lipoprotein measurement	Metabolic	PGS003153	EFO_0004530	triglyceride measurement	triglyceride measurement	EFO_0004530	Lipid or lipoprotein measurement	Metabolic
0.7274	2.00E-76	2.13E-73	PGS003199	EFO_0004631	vitamin D measurement	vitamin D measurement	EFO_0004631	Lipid or lipoprotein measurement	Metabolic	PGS001982	EFO_0004631	vitamin D measurement	vitamin D measurement	EFO_0004631	Lipid or lipoprotein measurement	Metabolic
0.7274	2.01E-76	2.14E-73	PGS001145	EFO_0005409	fat body mass	fat body mass	EFO_0005409	Other measurement	Body measurement	PGS001105	EFO_0007800	body fat percentage	body fat percentage	EFO_0007800	Body measurement	Body measurement
0.7274	2.03E-76	2.17E-73	PGS000572	EFO_0001663	prostate carcinoma	prostate carcinoma	EFO_0001663	Cancer	Neoplasms	PGS000568	EFO_0001663	prostate carcinoma	prostate carcinoma	EFO_0001663	Cancer	Neoplasms
0.7273	2.09E-76	2.23E-73	PGS003147	EFO_0004530	triglyceride measurement	triglyceride measurement	EFO_0004530	Lipid or lipoprotein measurement	Metabolic	PGS003148	EFO_0004530	triglyceride measurement	triglyceride measurement	EFO_0004530	Lipid or lipoprotein measurement	Metabolic
0.7273	2.10E-76	2.24E-73	PGS001778	EFO_0000305	breast carcinoma	breast carcinoma	EFO_0000305	Cancer	Neoplasms	PGS001336	EFO_0000305	breast carcinoma	breast carcinoma	EFO_0000305	Cancer	Neoplasms
0.7273	2.16E-76	2.30E-73	PGS002388	EFO_0000319	cardiovascular disease	cardiovascular disease	EFO_0000319	Cardiovascular disease	Cardiovascular	PGS002437	EFO_0000319	cardiovascular disease	cardiovascular disease	EFO_0000319	Cardiovascular disease	Cardiovascular
0.7272	2.35E-76	2.51E-73	PGS002856	EFO_0004340	body mass index	body mass index	EFO_0004340	Body measurement	Body measurement	PGS002845	EFO_0004340	body mass index	body mass index	EFO_0004340	Body measurement	Body measurement
0.7271	2.38E-76	2.53E-73	PGS002844	EFO_0004340	body mass index	body mass index	EFO_0004340	Body measurement	Body measurement	PGS001228	EFO_0004340	body mass index	body mass index	EFO_0004340	Body measurement	Body measurement
0.7271	2.38E-76	2.53E-73	PGS000471	MONDO_0004641	skin carcinoma in situ	skin carcinoma in situ	MONDO_0004641	Cancer	Neoplasms	PGS000470	MONDO_0004641	skin carcinoma in situ	skin carcinoma in situ	MONDO_0004641	Cancer	Neoplasms
0.7271	2.43E-76	2.59E-73	PGS000671	EFO_0004614	apolipoprotein A 1 measurement	apolipoprotein A 1 measurement	EFO_0004614	Cardiovascular measurement	Cardiovascular	PGS002101	EFO_0004614	apolipoprotein A 1 measurement	apolipoprotein A 1 measurement	EFO_0004614	Cardiovascular measurement	Cardiovascular
0.7271	2.43E-76	2.59E-73	PGS000671	EFO_0004614	apolipoprotein A 1 measurement	apolipoprotein A 1 measurement	EFO_0004614	Lipid or lipoprotein measurement	Metabolic	PGS002101	EFO_0004614	apolipoprotein A 1 measurement	apolipoprotein A 1 measurement	EFO_0004614	Lipid or lipoprotein measurement	Metabolic
0.7271	2.43E-76	2.59E-73	PGS003135	EFO_0004574	total cholesterol measurement	total cholesterol measurement	EFO_0004574	Lipid or lipoprotein measurement	Metabolic	PGS002571	EFO_0004574	total cholesterol measurement	total cholesterol measurement	EFO_0004574	Lipid or lipoprotein measurement	Metabolic
0.7271	2.43E-76	2.59E-73	PGS000671	EFO_0004614	apolipoprotein A 1 measurement	apolipoprotein A 1 measurement	EFO_0004614	Inflammatory measurement	Immunological	PGS002101	EFO_0004614	apolipoprotein A 1 measurement	apolipoprotein A 1 measurement	EFO_0004614	Inflammatory measurement	Immunological
0.7271	2.43E-76	2.59E-73	PGS000671	EFO_0004614	apolipoprotein A 1 measurement	apolipoprotein A 1 measurement	EFO_0004614	Cardiovascular measurement	Cardiovascular	PGS002101	EFO_0004614	apolipoprotein A 1 measurement	apolipoprotein A 1 measurement	EFO_0004614	Inflammatory measurement	Immunological
0.7271	2.43E-76	2.59E-73	PGS000671	EFO_0004614	apolipoprotein A 1 measurement	apolipoprotein A 1 measurement	EFO_0004614	Lipid or lipoprotein measurement	Metabolic	PGS002101	EFO_0004614	apolipoprotein A 1 measurement	apolipoprotein A 1 measurement	EFO_0004614	Cardiovascular measurement	Cardiovascular
0.7271	2.43E-76	2.59E-73	PGS000671	EFO_0004614	apolipoprotein A 1 measurement	apolipoprotein A 1 measurement	EFO_0004614	Cardiovascular measurement	Cardiovascular	PGS002101	EFO_0004614	apolipoprotein A 1 measurement	apolipoprotein A 1 measurement	EFO_0004614	Lipid or lipoprotein measurement	Metabolic
0.7271	2.43E-76	2.59E-73	PGS000671	EFO_0004614	apolipoprotein A 1 measurement	apolipoprotein A 1 measurement	EFO_0004614	Inflammatory measurement	Immunological	PGS002101	EFO_0004614	apolipoprotein A 1 measurement	apolipoprotein A 1 measurement	EFO_0004614	Lipid or lipoprotein measurement	Metabolic
0.7271	2.43E-76	2.59E-73	PGS000671	EFO_0004614	apolipoprotein A 1 measurement	apolipoprotein A 1 measurement	EFO_0004614	Lipid or lipoprotein measurement	Metabolic	PGS002101	EFO_0004614	apolipoprotein A 1 measurement	apolipoprotein A 1 measurement	EFO_0004614	Inflammatory measurement	Immunological
0.7271	2.43E-76	2.59E-73	PGS000671	EFO_0004614	apolipoprotein A 1 measurement	apolipoprotein A 1 measurement	EFO_0004614	Inflammatory measurement	Immunological	PGS002101	EFO_0004614	apolipoprotein A 1 measurement	apolipoprotein A 1 measurement	EFO_0004614	Cardiovascular measurement	Cardiovascular
0.7271	2.47E-76	2.63E-73	PGS000401	EFO_0009259	skin carcinoma	skin carcinoma	EFO_0009259	Cancer	Neoplasms	PGS000442	MONDO_0002898	skin cancer	skin cancer	MONDO_0002898	Cancer	Neoplasms
0.727	2.55E-76	2.71E-73	PGS000504	EFO_0000305	breast carcinoma	breast carcinoma	EFO_0000305	Cancer	Neoplasms	PGS000505	EFO_0000305	breast carcinoma	breast carcinoma	EFO_0000305	Cancer	Neoplasms
0.727	2.55E-76	2.71E-73	PGS000504	EFO_0000305	breast carcinoma	breast carcinoma	EFO_0000305	Cancer	Neoplasms	PGS000506	EFO_0000305	breast carcinoma	breast carcinoma	EFO_0000305	Cancer	Neoplasms
0.727	2.60E-76	2.77E-73	PGS002091	EFO_0008518	polymyalgia rheumatica	polymyalgia rheumatica	EFO_0008518	Other disease	Immunological	PGS002088	EFO_0000685	rheumatoid arthritis	rheumatoid arthritis	EFO_0000685	Immune system disorder	Immunological
0.727	2.67E-76	2.84E-73	PGS002633	EFO_0000319	cardiovascular disease	cardiovascular disease	EFO_0000319	Cardiovascular disease	Cardiovascular	PGS002316	EFO_0000319	cardiovascular disease	cardiovascular disease	EFO_0000319	Cardiovascular disease	Cardiovascular
0.727	2.72E-76	2.89E-73	PGS000049	EFO_0001663	prostate carcinoma	prostate carcinoma	EFO_0001663	Cancer	Neoplasms	PGS000580	EFO_0001663	prostate carcinoma	prostate carcinoma	EFO_0001663	Cancer	Neoplasms
0.7269	2.81E-76	2.99E-73	PGS002964	EFO_0004339	body height	body height	EFO_0004339	Body measurement	Body measurement	PGS002966	EFO_0004339	body height	body height	EFO_0004339	Body measurement	Body measurement
0.7269	2.81E-76	2.99E-73	PGS002186	EFO_0005091	monocyte count	monocyte count	EFO_0005091	Hematological measurement	Hematological	PGS002208	EFO_0007989	monocyte percentage of leukocytes	monocyte percentage of leukocytes	EFO_0007989	Hematological measurement	Hematological
0.7269	2.90E-76	3.08E-73	PGS002237	EFO_0003884	chronic kidney disease	chronic kidney disease	EFO_0003884	Other disease	Metabolic	PGS003377	EFO_0005208	glomerular filtration rate	glomerular filtration rate	EFO_0005208	Other measurement	Genitourinary
-0.7268	3.01E-76	3.20E-73	PGS003253	EFO_0010472	creatine measurement|glomerular filtration rate	creatine measurement	EFO_0010472	Other measurement	Metabolic	PGS003263	EFO_0010472	creatine measurement|glomerular filtration rate	creatine measurement	EFO_0010472	Other measurement	Metabolic
0.7266	3.39E-76	3.61E-73	PGS000488	EFO_0000305	breast carcinoma	breast carcinoma	EFO_0000305	Cancer	Neoplasms	PGS000052	EFO_0000305	breast carcinoma	breast carcinoma	EFO_0000305	Cancer	Neoplasms
0.7266	3.46E-76	3.67E-73	PGS002958	EFO_0004612	high density lipoprotein cholesterol measurement	high density lipoprotein cholesterol measurement	EFO_0004612	Lipid or lipoprotein measurement	Metabolic	PGS002101	EFO_0004614	apolipoprotein A 1 measurement	apolipoprotein A 1 measurement	EFO_0004614	Inflammatory measurement	Immunological
0.7266	3.46E-76	3.67E-73	PGS002958	EFO_0004612	high density lipoprotein cholesterol measurement	high density lipoprotein cholesterol measurement	EFO_0004612	Lipid or lipoprotein measurement	Metabolic	PGS002101	EFO_0004614	apolipoprotein A 1 measurement	apolipoprotein A 1 measurement	EFO_0004614	Cardiovascular measurement	Cardiovascular
0.7266	3.46E-76	3.67E-73	PGS002958	EFO_0004612	high density lipoprotein cholesterol measurement	high density lipoprotein cholesterol measurement	EFO_0004612	Lipid or lipoprotein measurement	Metabolic	PGS002101	EFO_0004614	apolipoprotein A 1 measurement	apolipoprotein A 1 measurement	EFO_0004614	Lipid or lipoprotein measurement	Metabolic
0.7266	3.58E-76	3.81E-73	PGS002553	HP_0003124	Hypercholesterolemia	Hypercholesterolemia	HP_0003124	Other trait	Metabolic	PGS002605	EFO_0004611	low density lipoprotein cholesterol measurement	measurement	EFO_0004611	Cardiovascular measurement	Cardiovascular
0.7266	3.58E-76	3.81E-73	PGS002553	HP_0003124	Hypercholesterolemia	Hypercholesterolemia	HP_0003124	Other trait	Metabolic	PGS002605	EFO_0004611	low density lipoprotein cholesterol measurement	measurement	EFO_0004611	Lipid or lipoprotein measurement	Metabolic
0.7265	3.68E-76	3.91E-73	PGS001106	EFO_0007800	body fat percentage	body fat percentage	EFO_0007800	Body measurement	Body measurement	PGS001162	EFO_0005093	hip circumference	hip circumference	EFO_0005093	Body measurement	Body measurement
-0.7265	3.68E-76	3.91E-73	PGS002768	EFO_0003882	osteoporosis	osteoporosis	EFO_0003882	Other disease	Skeletal	PGS001403	EFO_0009270	heel bone mineral density	heel bone mineral density	EFO_0009270	Other measurement	Skeletal
0.7265	3.71E-76	3.94E-73	PGS001278	EFO_0003827	pulmonary embolism|deep vein thrombosis	pulmonary embolism	EFO_0003827	Cardiovascular disease	Cardiovascular	PGS001847	EFO_0004264	vascular disease	vascular disease	EFO_0004264	Cardiovascular disease	Cardiovascular
0.7265	3.74E-76	3.97E-73	PGS000589	EFO_0001663	prostate carcinoma	prostate carcinoma	EFO_0001663	Cancer	Neoplasms	PGS000571	EFO_0001663	prostate carcinoma	prostate carcinoma	EFO_0001663	Cancer	Neoplasms
0.7265	3.74E-76	3.97E-73	PGS003285	EFO_0004617	cystatin C measurement	cystatin C measurement	EFO_0004617	Other measurement	Metabolic	PGS003280	EFO_0004617	cystatin C measurement	cystatin C measurement	EFO_0004617	Other measurement	Metabolic
0.7265	3.75E-76	3.98E-73	PGS001263	EFO_0003911	atrial flutter	atrial flutter	EFO_0003911	Cardiovascular disease	Cardiovascular	PGS002050	EFO_0003911	atrial flutter|atrial fibrillation	atrial flutter	EFO_0003911	Cardiovascular disease	Cardiovascular
0.7265	3.77E-76	4.00E-73	PGS002953	EFO_0004465	fasting blood glucose measurement	fasting blood glucose measurement	EFO_0004465	Other measurement	Metabolic	PGS002929	EFO_0004465	fasting blood glucose measurement	fasting blood glucose measurement	EFO_0004465	Other measurement	Metabolic
0.7265	3.79E-76	4.02E-73	PGS002336	EFO_0004705	hypothyroidism	hypothyroidism	EFO_0004705	Other disease	Endocrine	PGS001816	EFO_0004705	hypothyroidism	hypothyroidism	EFO_0004705	Other disease	Endocrine
0.7265	3.81E-76	4.03E-73	PGS001971	EFO_0004584	mean platelet volume	mean platelet volume	EFO_0004584	Hematological measurement	Hematological	PGS002412	EFO_0004584	mean platelet volume	mean platelet volume	EFO_0004584	Hematological measurement	Hematological
0.7264	4.07E-76	4.31E-73	PGS000774	EFO_1000649	estrogen-receptor positive breast cancer	estrogen-receptor positive breast cancer	EFO_1000649	Cancer	Neoplasms	PGS000005	EFO_1000649	estrogen-receptor positive breast cancer	cancer	EFO_1000649	Cancer	Neoplasms
0.7264	4.11E-76	4.35E-73	PGS001961	EFO_0005106	body composition measurement	body composition measurement	EFO_0005106	Body measurement	Body measurement	PGS001156	EFO_0005106	body composition measurement	body composition measurement	EFO_0005106	Body measurement	Body measurement
0.7264	4.14E-76	4.38E-73	PGS000578	EFO_0001663	prostate carcinoma	prostate carcinoma	EFO_0001663	Cancer	Neoplasms	PGS000049	EFO_0001663	prostate carcinoma	prostate carcinoma	EFO_0001663	Cancer	Neoplasms
0.7264	4.14E-76	4.38E-73	PGS000577	EFO_0001663	prostate carcinoma	prostate carcinoma	EFO_0001663	Cancer	Neoplasms	PGS000049	EFO_0001663	prostate carcinoma	prostate carcinoma	EFO_0001663	Cancer	Neoplasms
0.7263	4.23E-76	4.48E-73	PGS002999	EFO_0000537	hypertension	hypertension	EFO_0000537	Cardiovascular disease	Cardiovascular	PGS003000	EFO_0000537	hypertension	hypertension	EFO_0000537	Cardiovascular disease	Cardiovascular
0.7263	4.27E-76	4.52E-73	PGS003035	EFO_0004611	low density lipoprotein cholesterol measurement	low density lipoprotein cholesterol measurement	EFO_0004611	Lipid or lipoprotein measurement	Metabolic	PGS002571	EFO_0004574	total cholesterol measurement	total cholesterol measurement	EFO_0004574	Lipid or lipoprotein measurement	Metabolic
0.7263	4.27E-76	4.52E-73	PGS003035	EFO_0004611	low density lipoprotein cholesterol measurement	low density lipoprotein cholesterol measurement	EFO_0004611	Cardiovascular measurement	Cardiovascular	PGS002571	EFO_0004574	total cholesterol measurement	total cholesterol measurement	EFO_0004574	Lipid or lipoprotein measurement	Metabolic
0.7263	4.28E-76	4.53E-73	PGS001882	MONDO_0005178	osteoarthritis	osteoarthritis	MONDO_0005178	Other disease	Skeletal	PGS002094	MONDO_0005178	osteoarthritis	osteoarthritis	MONDO_0005178	Other disease	Skeletal
0.7263	4.37E-76	4.63E-73	PGS002453	EFO_0004339	body height	body height	EFO_0004339	Body measurement	Body measurement	PGS002965	EFO_0004339	body height	body height	EFO_0004339	Body measurement	Body measurement
0.7263	4.37E-76	4.63E-73	PGS001694	EFO_0006930	brain volume measurement|neuroimaging measurement	brain volume measurement	EFO_0006930	Other measurement	Neurological	PGS001696	EFO_0006930	measurement	brain volume measurement	EFO_0006930	Other measurement	Neurological
0.7262	4.49E-76	4.75E-73	PGS001478	EFO_0006930	brain volume measurement|neuroimaging measurement	brain volume measurement	EFO_0006930	Other measurement	Neurological	PGS001476	EFO_0006930	measurement	brain volume measurement	EFO_0006930	Other measurement	Neurological
0.7262	4.52E-76	4.78E-73	PGS002966	EFO_0004339	body height	body height	EFO_0004339	Body measurement	Body measurement	PGS002969	EFO_0004339	body height	body height	EFO_0004339	Body measurement	Body measurement
-0.7262	4.54E-76	4.80E-73	PGS002667	EFO_0004279	suntan	suntan	EFO_0004279	Other trait	Dermatological	PGS002647	EFO_0007822	hair colour measurement	hair colour measurement	EFO_0007822	Other measurement	Dermatological
0.7262	4.56E-76	4.82E-73	PGS000476	EFO_0000305	breast carcinoma	breast carcinoma	EFO_0000305	Cancer	Neoplasms	PGS000479	EFO_0000305	breast carcinoma	breast carcinoma	EFO_0000305	Cancer	Neoplasms
0.7262	4.72E-76	4.99E-73	PGS000684	EFO_0004468	glucose measurement	glucose measurement	EFO_0004468	Other measurement	Metabolic	PGS002938	EFO_0004468	glucose measurement	glucose measurement	EFO_0004468	Other measurement	Metabolic
0.7261	4.86E-76	5.14E-73	PGS002550	EFO_0004541	HbA1c measurement	HbA1c measurement	EFO_0004541	Hematological measurement	Hematological	PGS002589	EFO_0000400	diabetes mellitus	diabetes mellitus	EFO_0000400	Metabolic disorder	Metabolic
0.7261	4.86E-76	5.14E-73	PGS002550	EFO_0004541	HbA1c measurement	HbA1c measurement	EFO_0004541	Hematological measurement	Hematological	PGS002589	EFO_0000400	diabetes mellitus	diabetes mellitus	EFO_0000400	Digestive system disorder	Gastrointestinal
0.7261	5.08E-76	5.36E-73	PGS002545	EFO_0004713	FEV|FEC ratio	FEV|FEC ratio	EFO_0004713	Other measurement	Respiratory	PGS002447	EFO_0004713	FEV|FEC ratio	FEV|FEC ratio	EFO_0004713	Other measurement	Respiratory
0.726	5.23E-76	5.52E-73	PGS002999	EFO_0000537	hypertension	hypertension	EFO_0000537	Cardiovascular disease	Cardiovascular	PGS003001	EFO_0000537	hypertension	hypertension	EFO_0000537	Cardiovascular disease	Cardiovascular
0.726	5.29E-76	5.58E-73	PGS002856	EFO_0004340	body mass index	body mass index	EFO_0004340	Body measurement	Body measurement	PGS002855	EFO_0004340	body mass index	body mass index	EFO_0004340	Body measurement	Body measurement
0.726	5.34E-76	5.64E-73	PGS002412	EFO_0004584	mean platelet volume	mean platelet volume	EFO_0004584	Hematological measurement	Hematological	PGS002189	EFO_0004584	mean platelet volume	mean platelet volume	EFO_0004584	Hematological measurement	Hematological
0.7259	5.58E-76	5.89E-73	PGS000955	EFO_0004514	bone quantitative ultrasound measurement	bone quantitative ultrasound measurement	EFO_0004514	Other measurement	Skeletal	PGS000951	EFO_0004514	bone quantitative ultrasound measurement	measurement	EFO_0004514	Other measurement	Skeletal
0.7259	5.65E-76	5.96E-73	PGS001971	EFO_0004584	mean platelet volume	mean platelet volume	EFO_0004584	Hematological measurement	Hematological	PGS002559	EFO_0004584	mean platelet volume	mean platelet volume	EFO_0004584	Hematological measurement	Hematological
0.7259	5.68E-76	6.00E-73	PGS002721	EFO_0004631	vitamin D measurement	vitamin D measurement	EFO_0004631	Lipid or lipoprotein measurement	Metabolic	PGS003184	EFO_0004631	vitamin D measurement	vitamin D measurement	EFO_0004631	Lipid or lipoprotein measurement	Metabolic

0.7259	5.69E-76	6.00E-73	PGS003074	EFO_0006335	systolic blood pressure	systolic blood pressure	EFO_0006335	Other measurement	Cardiovascular	PGS002349	EFO_0006335	systolic blood pressure	systolic blood pressure	EFO_0006335	Other measurement	Cardiovascular
0.7259	5.86E-76	6.18E-73	PGS003198	EFO_0004631	vitamin D measurement	vitamin D measurement	EFO_0004631	Lipid or lipoprotein measurement	Metabolic	PGS003190	EFO_0004631	vitamin D measurement	vitamin D measurement	EFO_0004631	Lipid or lipoprotein measurement	Metabolic
0.7258	6.00E-76	6.32E-73	PGS001662	EFO_0006930	brain volume measurement|neuroimaging measurement	brain volume measurement	EFO_0006930	Other measurement	Neurological	PGS001673	EFO_0006930	measurement	brain volume measurement	EFO_0006930	Other measurement	Neurological
0.7258	6.11E-76	6.43E-73	PGS000532	EFO_0000305	breast carcinoma	breast carcinoma	EFO_0000305	Cancer	Neoplasms	PGS000526	EFO_0000305	breast carcinoma	breast carcinoma	EFO_0000305	Cancer	Neoplasms
0.7258	6.11E-76	6.43E-73	PGS000525	EFO_0000305	breast carcinoma	breast carcinoma	EFO_0000305	Cancer	Neoplasms	PGS000532	EFO_0000305	breast carcinoma	breast carcinoma	EFO_0000305	Cancer	Neoplasms
0.7258	6.12E-76	6.45E-73	PGS000424	EFO_0000756	melanoma	melanoma	EFO_0000756	Cancer	Neoplasms	PGS000079	EFO_0000756	melanoma	melanoma	EFO_0000756	Cancer	Neoplasms
0.7257	6.62E-76	6.97E-73	PGS000511	EFO_0000305	breast carcinoma	breast carcinoma	EFO_0000305	Cancer	Neoplasms	PGS000510	EFO_0000305	breast carcinoma	breast carcinoma	EFO_0000305	Cancer	Neoplasms
0.7257	6.63E-76	6.98E-73	PGS003231	EFO_0010472	creatine measurement	creatine measurement	EFO_0010472	Other measurement	Metabolic	PGS003236	EFO_0010472	creatine measurement	creatine measurement	EFO_0010472	Other measurement	Metabolic
0.7257	6.68E-76	7.03E-73	PGS002721	EFO_0004631	vitamin D measurement	vitamin D measurement	EFO_0004631	Lipid or lipoprotein measurement	Metabolic	PGS003199	EFO_0004631	vitamin D measurement	vitamin D measurement	EFO_0004631	Lipid or lipoprotein measurement	Metabolic
0.7255	7.50E-76	7.90E-73	PGS000471	MONDO_0004641	skin carcinoma in situ	skin carcinoma in situ	MONDO_0004641	Cancer	Neoplasms	PGS001257	EFO_0003958	sunburn	sunburn	EFO_0003958	Other disease	Dermatological
0.7255	7.53E-76	7.93E-73	PGS000783	EFO_0000305	breast carcinoma	breast carcinoma	EFO_0000305	Cancer	Neoplasms	PGS000344	EFO_0000305	breast carcinoma	breast carcinoma	EFO_0000305	Cancer	Neoplasms
0.7255	7.80E-76	8.20E-73	PGS003194	EFO_0004631	vitamin D measurement	vitamin D measurement	EFO_0004631	Lipid or lipoprotein measurement	Metabolic	PGS002623	EFO_0004631	vitamin D measurement	vitamin D measurement	EFO_0004631	Lipid or lipoprotein measurement	Metabolic
0.7254	7.92E-76	8.33E-73	PGS003035	EFO_0004611	low density lipoprotein cholesterol measurement	low density lipoprotein cholesterol measurement	EFO_0004611	Cardiovascular measurement	Cardiovascular	PGS003032	EFO_0004611	low density lipoprotein cholesterol measurement	measurement	EFO_0004611	Cardiovascular measurement	Cardiovascular
0.7254	7.92E-76	8.33E-73	PGS003035	EFO_0004611	low density lipoprotein cholesterol measurement	low density lipoprotein cholesterol measurement	EFO_0004611	Cardiovascular measurement	Cardiovascular	PGS003032	EFO_0004611	low density lipoprotein cholesterol measurement	measurement	EFO_0004611	Lipid or lipoprotein measurement	Metabolic
0.7254	7.92E-76	8.33E-73	PGS003035	EFO_0004611	low density lipoprotein cholesterol measurement	low density lipoprotein cholesterol measurement	EFO_0004611	Lipid or lipoprotein measurement	Metabolic	PGS003032	EFO_0004611	low density lipoprotein cholesterol measurement	measurement	EFO_0004611	Cardiovascular measurement	Cardiovascular
0.7254	7.92E-76	8.33E-73	PGS003035	EFO_0004611	low density lipoprotein cholesterol measurement	low density lipoprotein cholesterol measurement	EFO_0004611	Lipid or lipoprotein measurement	Metabolic	PGS003032	EFO_0004611	low density lipoprotein cholesterol measurement	measurement	EFO_0004611	Lipid or lipoprotein measurement	Metabolic
0.7254	8.05E-76	8.46E-73	PGS000045	EFO_0000305	breast carcinoma	breast carcinoma	EFO_0000305	Cancer	Neoplasms	PGS000052	EFO_0000305	breast carcinoma	breast carcinoma	EFO_0000305	Cancer	Neoplasms
0.7254	8.24E-76	8.66E-73	PGS002559	EFO_0004584	mean platelet volume	mean platelet volume	EFO_0004584	Hematological measurement	Hematological	PGS002189	EFO_0004584	mean platelet volume	mean platelet volume	EFO_0004584	Hematological measurement	Hematological
0.7254	8.31E-76	8.74E-73	PGS001753	EFO_0006930	brain volume measurement|neuroimaging measurement	brain volume measurement	EFO_0006930	Other measurement	Neurological	PGS001723	EFO_0006930	measurement	brain volume measurement	EFO_0006930	Other measurement	Neurological
0.7254	8.37E-76	8.79E-73	PGS002476	EFO_0004631	vitamin D measurement	vitamin D measurement	EFO_0004631	Lipid or lipoprotein measurement	Metabolic	PGS002427	EFO_0004631	vitamin D measurement	vitamin D measurement	EFO_0004631	Lipid or lipoprotein measurement	Metabolic
0.7253	8.89E-76	9.34E-73	PGS001289	EFO_0002892	thyroid carcinoma	thyroid carcinoma	EFO_0002892	Cancer	Neoplasms	PGS000208	EFO_0002892	thyroid carcinoma	thyroid carcinoma	EFO_0002892	Cancer	Neoplasms
0.7252	9.06E-76	9.51E-73	PGS003150	EFO_0004530	triglyceride measurement	triglyceride measurement	EFO_0004530	Lipid or lipoprotein measurement	Metabolic	PGS003147	EFO_0004530	triglyceride measurement	triglyceride measurement	EFO_0004530	Lipid or lipoprotein measurement	Metabolic
0.7252	9.07E-76	9.53E-73	PGS001990	EFO_0004526	mean corpuscular volume	mean corpuscular volume	EFO_0004526	Hematological measurement	Hematological	PGS002656	EFO_0004527	mean corpuscular hemoglobin	mean corpuscular hemoglobin	EFO_0004527	Hematological measurement	Hematological
0.7252	9.27E-76	9.74E-73	PGS001487	EFO_0006930	brain volume measurement|neuroimaging measurement	brain volume measurement	EFO_0006930	Other measurement	Neurological	PGS001486	EFO_0006930	measurement	brain volume measurement	EFO_0006930	Other measurement	Neurological
0.7252	9.39E-76	9.86E-73	PGS002802	EFO_0004339	body height	body height	EFO_0004339	Body measurement	Body measurement	PGS001229	EFO_0004339	body height	body height	EFO_0004339	Body measurement	Body measurement
0.7251	9.79E-76	1.03E-72	PGS000333	EFO_0001663	prostate carcinoma	prostate carcinoma	EFO_0001663	Cancer	Neoplasms	PGS003383	EFO_0000673	prostate adenocarcinoma	prostate adenocarcinoma	EFO_0000673	Cancer	Neoplasms
0.7251	1.03E-75	1.08E-72	PGS001962	EFO_0004308	leukocyte count	leukocyte count	EFO_0004308	Hematological measurement	Hematological	PGS001239	EFO_0004308	leukocyte count	leukocyte count	EFO_0004308	Hematological measurement	Hematological
-0.7251	1.03E-75	1.08E-72	PGS000309	EFO_0004612	high density lipoprotein cholesterol measurement	high density lipoprotein cholesterol measurement	EFO_0004612	Lipid or lipoprotein measurement	Metabolic	PGS000825	EFO_0004612	high density lipoprotein cholesterol measurement	measurement	EFO_0004612	Lipid or lipoprotein measurement	Metabolic
0.7249	1.14E-75	1.20E-72	PGS002774	EFO_0000275	atrial fibrillation	atrial fibrillation	EFO_0000275	Cardiovascular disease	Cardiovascular	PGS000035	EFO_0000275	atrial fibrillation	atrial fibrillation	EFO_0000275	Cardiovascular disease	Cardiovascular
0.7249	1.18E-75	1.23E-72	PGS002846	EFO_0004340	body mass index	body mass index	EFO_0004340	Body measurement	Body measurement	PGS002845	EFO_0004340	body mass index	body mass index	EFO_0004340	Body measurement	Body measurement
0.7248	1.19E-75	1.25E-72	PGS001480	EFO_0006930	brain volume measurement|neuroimaging measurement	brain volume measurement	EFO_0006930	Other measurement	Neurological	PGS001450	EFO_0006930	measurement	brain volume measurement	EFO_0006930	Other measurement	Neurological
0.7248	1.20E-75	1.25E-72	PGS001943	EFO_0004340	body mass index	body mass index	EFO_0004340	Body measurement	Body measurement	PGS002630	EFO_0004340	body mass index	body mass index	EFO_0004340	Body measurement	Body measurement
0.7248	1.20E-75	1.25E-72	PGS000164	EFO_0007992	basophil percentage of leukocytes	basophil percentage of leukocytes	EFO_0007992	Inflammatory measurement	Immunological	PGS000089	EFO_0007992	basophil percentage of leukocytes	basophil percentage of leukocytes	EFO_0007992	Inflammatory measurement	Immunological
0.7248	1.20E-75	1.25E-72	PGS000164	EFO_0007992	basophil percentage of leukocytes	basophil percentage of leukocytes	EFO_0007992	Hematological measurement	Hematological	PGS000089	EFO_0007992	basophil percentage of leukocytes	basophil percentage of leukocytes	EFO_0007992	Hematological measurement	Hematological
0.7248	1.20E-75	1.25E-72	PGS000164	EFO_0007992	basophil percentage of leukocytes	basophil percentage of leukocytes	EFO_0007992	Inflammatory measurement	Immunological	PGS000089	EFO_0007992	basophil percentage of leukocytes	basophil percentage of leukocytes	EFO_0007992	Hematological measurement	Hematological
0.7248	1.20E-75	1.25E-72	PGS000164	EFO_0007992	basophil percentage of leukocytes	basophil percentage of leukocytes	EFO_0007992	Hematological measurement	Hematological	PGS000089	EFO_0007992	basophil percentage of leukocytes	basophil percentage of leukocytes	EFO_0007992	Inflammatory measurement	Immunological
0.7248	1.20E-75	1.26E-72	PGS002241	EFO_0001663	prostate carcinoma	prostate carcinoma	EFO_0001663	Cancer	Neoplasms	PGS000587	EFO_0001663	prostate carcinoma	prostate carcinoma	EFO_0001663	Cancer	Neoplasms
0.7248	1.22E-75	1.28E-72	PGS002846	EFO_0004340	body mass index	body mass index	EFO_0004340	Body measurement	Body measurement	PGS002850	EFO_0004340	body mass index	body mass index	EFO_0004340	Body measurement	Body measurement
0.7248	1.22E-75	1.28E-72	PGS000207	EFO_0002892	thyroid carcinoma	thyroid carcinoma	EFO_0002892	Cancer	Neoplasms	PGS001809	EFO_0002892	thyroid carcinoma	thyroid carcinoma	EFO_0002892	Cancer	Neoplasms
0.7247	1.28E-75	1.34E-72	PGS002632	EFO_0003923	bone density	bone density	EFO_0003923	Other measurement	Skeletal	PGS001037	EFO_0009270	heel bone mineral density	heel bone mineral density	EFO_0009270	Other measurement	Skeletal
0.7247	1.30E-75	1.36E-72	PGS002551	EFO_0004339	body height	body height	EFO_0004339	Body measurement	Body measurement	PGS002973	EFO_0004339	body height	body height	EFO_0004339	Body measurement	Body measurement
0.7247	1.30E-75	1.36E-72	PGS000952	EFO_0004514	bone quantitative ultrasound measurement	bone quantitative ultrasound measurement	EFO_0004514	Other measurement	Skeletal	PGS002632	EFO_0003923	bone density	bone density	EFO_0003923	Other measurement	Skeletal
0.7247	1.33E-75	1.39E-72	PGS002642	EFO_0004842	eosinophil count	eosinophil count	EFO_0004842	Hematological measurement	Hematological	PGS001172	EFO_0004842	eosinophil count	eosinophil count	EFO_0004842	Hematological measurement	Hematological
0.7247	1.33E-75	1.39E-72	PGS002443	EFO_0006336	diastolic blood pressure	diastolic blood pressure	EFO_0006336	Other measurement	Cardiovascular	PGS002541	EFO_0006336	diastolic blood pressure	diastolic blood pressure	EFO_0006336	Other measurement	Cardiovascular
0.7247	1.34E-75	1.40E-72	PGS000023	MONDO_0005147	type 1 diabetes mellitus	type 1 diabetes mellitus	MONDO_0005147	Digestive system disorder	Gastrointestinal	PGS001296	MONDO_0005147	type 1 diabetes mellitus	type 1 diabetes mellitus	MONDO_0005147	Digestive system disorder	Gastrointestinal
0.7247	1.34E-75	1.40E-72	PGS000023	MONDO_0005147	type 1 diabetes mellitus	type 1 diabetes mellitus	MONDO_0005147	Metabolic disorder	Metabolic	PGS001296	MONDO_0005147	type 1 diabetes mellitus	type 1 diabetes mellitus	MONDO_0005147	Metabolic disorder	Metabolic
0.7247	1.34E-75	1.40E-72	PGS000023	MONDO_0005147	type 1 diabetes mellitus	type 1 diabetes mellitus	MONDO_0005147	Metabolic disorder	Metabolic	PGS001296	MONDO_0005147	type 1 diabetes mellitus	type 1 diabetes mellitus	MONDO_0005147	Immune system disorder	Immunological
0.7247	1.34E-75	1.40E-72	PGS000023	MONDO_0005147	type 1 diabetes mellitus	type 1 diabetes mellitus	MONDO_0005147	Digestive system disorder	Gastrointestinal	PGS001296	MONDO_0005147	type 1 diabetes mellitus	type 1 diabetes mellitus	MONDO_0005147	Immune system disorder	Immunological
0.7247	1.34E-75	1.40E-72	PGS000023	MONDO_0005147	type 1 diabetes mellitus	type 1 diabetes mellitus	MONDO_0005147	Immune system disorder	Immunological	PGS001296	MONDO_0005147	type 1 diabetes mellitus	type 1 diabetes mellitus	MONDO_0005147	Immune system disorder	Immunological
0.7247	1.34E-75	1.40E-72	PGS000023	MONDO_0005147	type 1 diabetes mellitus	type 1 diabetes mellitus	MONDO_0005147	Immune system disorder	Immunological	PGS001296	MONDO_0005147	type 1 diabetes mellitus	type 1 diabetes mellitus	MONDO_0005147	Digestive system disorder	Gastrointestinal
0.7247	1.34E-75	1.40E-72	PGS000023	MONDO_0005147	type 1 diabetes mellitus	type 1 diabetes mellitus	MONDO_0005147	Metabolic disorder	Metabolic	PGS001296	MONDO_0005147	type 1 diabetes mellitus	type 1 diabetes mellitus	MONDO_0005147	Digestive system disorder	Gastrointestinal
0.7247	1.34E-75	1.40E-72	PGS000023	MONDO_0005147	type 1 diabetes mellitus	type 1 diabetes mellitus	MONDO_0005147	Immune system disorder	Immunological	PGS001296	MONDO_0005147	type 1 diabetes mellitus	type 1 diabetes mellitus	MONDO_0005147	Metabolic disorder	Metabolic
0.7247	1.34E-75	1.40E-72	PGS000023	MONDO_0005147	type 1 diabetes mellitus	type 1 diabetes mellitus	MONDO_0005147	Digestive system disorder	Gastrointestinal	PGS001296	MONDO_0005147	type 1 diabetes mellitus	type 1 diabetes mellitus	MONDO_0005147	Metabolic disorder	Metabolic
0.7247	1.34E-75	1.40E-72	PGS003186	EFO_0004631	vitamin D measurement	vitamin D measurement	EFO_0004631	Lipid or lipoprotein measurement	Metabolic	PGS002623	EFO_0004631	vitamin D measurement	vitamin D measurement	EFO_0004631	Lipid or lipoprotein measurement	Metabolic
0.7246	1.37E-75	1.44E-72	PGS002133	EFO_0007800	body fat percentage	body fat percentage	EFO_0007800	Body measurement	Body measurement	PGS001101	EFO_0007800	body fat percentage	body fat percentage	EFO_0007800	Body measurement	Body measurement
0.7246	1.43E-75	1.49E-72	PGS002024	EFO_0004705	hypothyroidism	hypothyroidism	EFO_0004705	Other disease	Endocrine	PGS002336	EFO_0004705	hypothyroidism	hypothyroidism	EFO_0004705	Other disease	Endocrine
0.7245	1.51E-75	1.58E-72	PGS000333	EFO_0001663	prostate carcinoma	prostate carcinoma	EFO_0001663	Cancer	Neoplasms	PGS000588	EFO_0001663	prostate carcinoma	prostate carcinoma	EFO_0001663	Cancer	Neoplasms
0.7245	1.52E-75	1.59E-72	PGS000096	EFO_0007986	reticulocyte count	reticulocyte count	EFO_0007986	Hematological measurement	Hematological	PGS001408	EFO_0009253	Immature Reticulocyte Fraction Measurement	Measurement	EFO_0009253	Hematological measurement	Hematological
0.7245	1.54E-75	1.61E-72	PGS000713	MONDO_0005148	type 2 diabetes mellitus	type 2 diabetes mellitus	MONDO_0005148	Digestive system disorder	Gastrointestinal	PGS001329	EFO_0000400	diabetes mellitus	diabetes mellitus	EFO_0000400	Digestive system disorder	Gastrointestinal
0.7245	1.54E-75	1.61E-72	PGS000713	MONDO_0005148	type 2 diabetes mellitus	type 2 diabetes mellitus	MONDO_0005148	Digestive system disorder	Gastrointestinal	PGS001329	EFO_0000400	diabetes mellitus	diabetes mellitus	EFO_0000400	Metabolic disorder	Metabolic
0.7245	1.54E-75	1.61E-72	PGS000713	MONDO_0005148	type 2 diabetes mellitus	type 2 diabetes mellitus	MONDO_0005148	Metabolic disorder	Metabolic	PGS001329	EFO_0000400	diabetes mellitus	diabetes mellitus	EFO_0000400	Digestive system disorder	Gastrointestinal
0.7245	1.54E-75	1.61E-72	PGS000713	MONDO_0005148	type 2 diabetes mellitus	type 2 diabetes mellitus	MONDO_0005148	Metabolic disorder	Metabolic	PGS001329	EFO_0000400	diabetes mellitus	diabetes mellitus	EFO_0000400	Metabolic disorder	Metabolic
-0.7245	1.56E-75	1.63E-72	PGS001987	EFO_0007825	balding measurement	balding measurement	EFO_0007825	Other measurement	Activities	PGS002631	EFO_0007825	balding measurement	balding measurement	EFO_0007825	Other measurement	Activities
0.7244	1.59E-75	1.66E-72	PGS002206	EFO_0004527	mean corpuscular hemoglobin	mean corpuscular hemoglobin	EFO_0004527	Hematological measurement	Hematological	PGS002607	EFO_0004527	mean corpuscular hemoglobin	mean corpuscular hemoglobin	EFO_0004527	Hematological measurement	Hematological
0.7244	1.61E-75	1.68E-72	PGS000174	EFO_0004527	mean corpuscular hemoglobin	mean corpuscular hemoglobin	EFO_0004527	Hematological measurement	Hematological	PGS000099	EFO_0004527	mean corpuscular hemoglobin	mean corpuscular hemoglobin	EFO_0004527	Hematological measurement	Hematological
0.7244	1.61E-75	1.68E-72	PGS001909	EFO_0004305	erythrocyte count	erythrocyte count	EFO_0004305	Hematological measurement	Hematological	PGS002662	EFO_0004305	erythrocyte count	erythrocyte count	EFO_0004305	Hematological measurement	Hematological
0.7244	1.65E-75	1.73E-72	PGS002632	EFO_0003923	bone density	bone density	EFO_0003923	Other measurement	Skeletal	PGS001402	EFO_0009270	heel bone mineral density	heel bone mineral density	EFO_0009270	Other measurement	Skeletal
0.7243	1.69E-75	1.77E-72	PGS002194	EFO_0007986	reticulocyte count	reticulocyte count	EFO_0007986	Hematological measurement	Hematological	PGS001406	EFO_0010700	reticulocyte measurement	reticulocyte measurement	EFO_0010700	Hematological measurement	Hematological
0.7243	1.75E-75	1.83E-72	PGS000594	EFO_0007355	male reproductive organ cancer	male reproductive organ cancer	EFO_0007355	Cancer	Neoplasms	PGS000589	EFO_0001663	prostate carcinoma	prostate carcinoma	EFO_0001663	Cancer	Neoplasms
0.7243	1.76E-75	1.84E-72	PGS003144	EFO_0004530	triglyceride measurement	triglyceride measurement	EFO_0004530	Lipid or lipoprotein measurement	Metabolic	PGS003152	EFO_0004530	triglyceride measurement	triglyceride measurement	EFO_0004530	Lipid or lipoprotein measurement	Metabolic
0.7242	1.85E-75	1.92E-72	PGS000438	MONDO_0002898	skin cancer	skin cancer	MONDO_0002898	Cancer	Neoplasms	PGS000401	EFO_0009259	skin carcinoma	skin carcinoma	EFO_0009259	Cancer	Neoplasms
0.7242	1.87E-75	1.95E-72	PGS000831	EFO_0004574	total cholesterol measurement	total cholesterol measurement	EFO_0004574	Lipid or lipoprotein measurement	Metabolic	PGS002620	EFO_0004574	total cholesterol measurement	total cholesterol measurement	EFO_0004574	Lipid or lipoprotein measurement	Metabolic
0.7241	1.95E-75	2.04E-72	PGS003245	EFO_0010472	creatine measurement	creatine measurement	EFO_0010472	Other measurement	Metabolic	PGS003240	EFO_0010472	creatine measurement	creatine measurement	EFO_0010472	Other measurement	Metabolic
0.7241	1.97E-75	2.05E-72	PGS000044	EFO_0001663	prostate carcinoma	prostate carcinoma	EFO_0001663	Cancer	Neoplasms	PGS000579	EFO_0001663	prostate carcinoma	prostate carcinoma	EFO_0001663	Cancer	Neoplasms
0.7241	1.98E-75	2.06E-72	PGS001928	EFO_0009550	headache disorder	headache disorder	EFO_0009550	Neurological disorder	Neurological	PGS002145	EFO_0009550	headache disorder	headache disorder	EFO_0009550	Neurological disorder	Neurological
0.7241	2.06E-75	2.15E-72	PGS001805	EFO_0001663	prostate carcinoma	prostate carcinoma	EFO_0001663	Cancer	Neoplasms	PGS000571	EFO_0001663	prostate carcinoma	prostate carcinoma	EFO_0001663	Cancer	Neoplasms
0.724	2.09E-75	2.18E-72	PGS001929	EFO_0004339	body height	body height	EFO_0004339	Body measurement	Body measurement	PGS002983	EFO_0004339	body height	body height	EFO_0004339	Body measurement	Body measurement
0.724	2.15E-75	2.24E-72	PGS001336	EFO_0000305	breast carcinoma	breast carcinoma	EFO_0000305	Cancer	Neoplasms	PGS000474	EFO_0000305	breast carcinoma	breast carcinoma	EFO_0000305	Cancer	Neoplasms
0.724	2.18E-75	2.27E-72	PGS001671	EFO_0006930	brain volume measurement|neuroimaging measurement	brain volume measurement	EFO_0006930	Other measurement	Neurological	PGS001661	EFO_0006930	measurement	brain volume measurement	EFO_0006930	Other measurement	Neurological
0.7239	2.28E-75	2.37E-72	PGS001479	EFO_0006930	brain volume measurement|neuroimaging measurement	brain volume measurement	EFO_0006930	Other measurement	Neurological	PGS001683	EFO_0006930	measurement	brain volume measurement	EFO_0006930	Other measurement	Neurological
0.7239	2.28E-75	2.38E-72	PGS000883	EFO_0005208	glomerular filtration rate	glomerular filtration rate	EFO_0005208	Other measurement	Genitourinary	PGS002237	EFO_0003884	chronic kidney disease	chronic kidney disease	EFO_0003884	Other disease	Metabolic
-0.7239	2.31E-75	2.40E-72	PGS003228	EFO_0005208	glomerular filtration rate	glomerular filtration rate	EFO_0005208	Other measurement	Genitourinary	PGS003248	EFO_0010472	creatine measurement	creatine measurement	EFO_0010472	Other measurement	Metabolic
0.7239	2.36E-75	2.45E-72	PGS002409	EFO_0004611	low density lipoprotein cholesterol measurement	low density lipoprotein cholesterol measurement	EFO_0004611	Cardiovascular measurement	Cardiovascular	PGS003036	EFO_0004611	low density lipoprotein cholesterol measurement	measurement	EFO_0004611	Cardiovascular measurement	Cardiovascular
0.7239	2.36E-75	2.45E-72	PGS002409	EFO_0004611	low density lipoprotein cholesterol measurement	low density lipoprotein cholesterol measurement	EFO_0004611	Lipid or lipoprotein measurement	Metabolic	PGS003036	EFO_0004611	low density lipoprotein cholesterol measurement	measurement	EFO_0004611	Lipid or lipoprotein measurement	Metabolic
0.7239	2.36E-75	2.45E-72	PGS002409	EFO_0004611	low density lipoprotein cholesterol measurement	low density lipoprotein cholesterol measurement	EFO_0004611	Lipid or lipoprotein measurement	Metabolic	PGS003036	EFO_0004611	low density lipoprotein cholesterol measurement	measurement	EFO_0004611	Cardiovascular measurement	Cardiovascular
0.7239	2.36E-75	2.45E-72	PGS002409	EFO_0004611	low density lipoprotein cholesterol measurement	low density lipoprotein cholesterol measurement	EFO_0004611	Cardiovascular measurement	Cardiovascular	PGS003036	EFO_0004611	low density lipoprotein cholesterol measurement	measurement	EFO_0004611	Lipid or lipoprotein measurement	Metabolic
0.7237	2.57E-75	2.67E-72	PGS000048	EFO_0001075	ovarian carcinoma	ovarian carcinoma	EFO_0001075	Cancer	Neoplasms	PGS000563	EFO_0003893	ovarian neoplasm	ovarian neoplasm	EFO_0003893	Cancer	Neoplasms
0.7237	2.58E-75	2.68E-72	PGS000824	EFO_0004611	low density lipoprotein cholesterol measurement	low density lipoprotein cholesterol measurement	EFO_0004611	Cardiovascular measurement	Cardiovascular	PGS002571	EFO_0004574	total cholesterol measurement	total cholesterol measurement	EFO_0004574	Lipid or lipoprotein measurement	Metabolic
0.7237	2.58E-75	2.68E-72	PGS000824	EFO_0004611	low density lipoprotein cholesterol measurement	low density lipoprotein cholesterol measurement	EFO_0004611	Lipid or lipoprotein measurement	Metabolic	PGS002571	EFO_0004574	total cholesterol measurement	total cholesterol measurement	EFO_0004574	Lipid or lipoprotein measurement	Metabolic
0.7237	2.63E-75	2.74E-72	PGS002335	EFO_0000537	hypertension	hypertension	EFO_0000537	Cardiovascular disease	Cardiovascular	PGS002652	EFO_0000537	hypertension	hypertension	EFO_0000537	Cardiovascular disease	Cardiovascular
0.7237	2.63E-75	2.74E-72	PGS002970	EFO_0004339	body height	body height	EFO_0004339	Body measurement	Body measurement	PGS002404	EFO_0004339	body height	body height	EFO_0004339	Body measurement	Body measurement
0.7237	2.68E-75	2.79E-72	PGS001672	EFO_0006930	brain volume measurement|neuroimaging measurement	brain volume measurement	EFO_0006930	Other measurement	Neurological	PGS001450	EFO_0006930	measurement	brain volume measurement	EFO_0006930	Other measurement	Neurological
0.7237	2.70E-75	2.80E-72	PGS001908	EFO_0005192	red blood cell distribution width	red blood cell distribution width	EFO_0005192	Hematological measurement	Hematological	PGS002565	EFO_0005192	red blood cell distribution width	red blood cell distribution width	EFO_0005192	Hematological measurement	Hematological
0.7236	2.89E-75	3.00E-72	PGS001751	EFO_0006930	brain volume measurement|neuroimaging measurement	brain volume measurement	EFO_0006930	Other measurement	Neurological	PGS001752	EFO_0006930	measurement	brain volume measurement	EFO_0006930	Other measurement	Neurological
0.7235	2.93E-75	3.05E-72	PGS003147	EFO_0004530	triglyceride measurement	triglyceride measurement	EFO_0004530	Lipid or lipoprotein measurement	Metabolic	PGS003149	EFO_0004530	triglyceride measurement	triglyceride measurement	EFO_0004530	Lipid or lipoprotein measurement	Metabolic
0.7235	2.94E-75	3.05E-72	PGS002721	EFO_0004631	vitamin D measurement	vitamin D measurement	EFO_0004631	Lipid or lipoprotein measurement	Metabolic	PGS003191	EFO_0004631	vitamin D measurement	vitamin D measurement	EFO_0004631	Lipid or lipoprotein measurement	Metabolic
0.7235	2.96E-75	3.08E-72	PGS002337	EFO_0004611	low density lipoprotein cholesterol measurement	low density lipoprotein cholesterol measurement	EFO_0004611	Lipid or lipoprotein measurement	Metabolic	PGS002605	EFO_0004611	low density lipoprotein cholesterol measurement	measurement	EFO_0004611	Cardiovascular measurement	Cardiovascular
0.7235	2.96E-75	3.08E-72	PGS002337	EFO_0004611	low density lipoprotein cholesterol measurement	low density lipoprotein cholesterol measurement	EFO_0004611	Cardiovascular measurement	Cardiovascular	PGS002605	EFO_0004611	low density lipoprotein cholesterol measurement	measurement	EFO_0004611	Cardiovascular measurement	Cardiovascular
0.7235	2.96E-75	3.08E-72	PGS002337	EFO_0004611	low density lipoprotein cholesterol measurement	low density lipoprotein cholesterol measurement	EFO_0004611	Lipid or lipoprotein measurement	Metabolic	PGS002605	EFO_0004611	low density lipoprotein cholesterol measurement	measurement	EFO_0004611	Lipid or lipoprotein measurement	Metabolic
0.7235	2.96E-75	3.08E-72	PGS002337	EFO_0004611	low density lipoprotein cholesterol measurement	low density lipoprotein cholesterol measurement	EFO_0004611	Cardiovascular measurement	Cardiovascular	PGS002605	EFO_0004611	low density lipoprotein cholesterol measurement	measurement	EFO_0004611	Lipid or lipoprotein measurement	Metabolic
0.7235	3.10E-75	3.21E-72	PGS000332	EFO_0000305	breast carcinoma	breast carcinoma	EFO_0000305	Cancer	Neoplasms	PGS000504	EFO_0000305	breast carcinoma	breast carcinoma	EFO_0000305	Cancer	Neoplasms
0.7234	3.22E-75	3.34E-72	PGS002551	EFO_0004339	body height	body height	EFO_0004339	Body measurement	Body measurement	PGS002968	EFO_0004339	body height	body height	EFO_0004339	Body measurement	Body measurement
0.7234	3.27E-75	3.39E-72	PGS000503	EFO_0000305	breast carcinoma	breast carcinoma	EFO_0000305	Cancer	Neoplasms	PGS000505	EFO_0000305	breast carcinoma	breast carcinoma	EFO_0000305	Cancer	Neoplasms

0.7234	3.27E-75	3.39E-72	PGS000506	EFO_0000305	breast carcinoma	breast carcinoma	EFO_0000305	Cancer	Neoplasms	PGS000503	EFO_0000305	breast carcinoma	breast carcinoma	EFO_0000305	Cancer	Neoplasms
0.7234	3.29E-75	3.41E-72	PGS000163	EFO_0005090	basophil count	basophil count	EFO_0005090	Inflammatory measurement	Immunological	PGS000164	EFO_0007992	basophil percentage of leukocytes	basophil percentage of leukocytes	EFO_0007992	Hematological measurement	Hematological
0.7234	3.29E-75	3.41E-72	PGS000163	EFO_0005090	basophil count	basophil count	EFO_0005090	Hematological measurement	Hematological	PGS000164	EFO_0007992	basophil percentage of leukocytes	basophil percentage of leukocytes	EFO_0007992	Hematological measurement	Hematological
0.7234	3.29E-75	3.41E-72	PGS000163	EFO_0005090	basophil count	basophil count	EFO_0005090	Hematological measurement	Hematological	PGS000164	EFO_0007992	basophil percentage of leukocytes	basophil percentage of leukocytes	EFO_0007992	Inflammatory measurement	Immunological
0.7234	3.29E-75	3.41E-72	PGS000163	EFO_0005090	basophil count	basophil count	EFO_0005090	Inflammatory measurement	Immunological	PGS000164	EFO_0007992	basophil percentage of leukocytes	basophil percentage of leukocytes	EFO_0007992	Inflammatory measurement	Immunological
0.7234	3.34E-75	3.46E-72	PGS003032	EFO_0004611	low density lipoprotein cholesterol measurement	low density lipoprotein cholesterol measurement	EFO_0004611	Cardiovascular measurement	Cardiovascular	PGS003030	EFO_0004611	low density lipoprotein cholesterol measurement	measurement	EFO_0004611	Lipid or lipoprotein measurement	Metabolic
0.7234	3.34E-75	3.46E-72	PGS003032	EFO_0004611	low density lipoprotein cholesterol measurement	low density lipoprotein cholesterol measurement	EFO_0004611	Lipid or lipoprotein measurement	Metabolic	PGS003030	EFO_0004611	low density lipoprotein cholesterol measurement	measurement	EFO_0004611	Lipid or lipoprotein measurement	Metabolic
0.7234	3.34E-75	3.46E-72	PGS003032	EFO_0004611	low density lipoprotein cholesterol measurement	low density lipoprotein cholesterol measurement	EFO_0004611	Lipid or lipoprotein measurement	Metabolic	PGS003030	EFO_0004611	low density lipoprotein cholesterol measurement	measurement	EFO_0004611	Cardiovascular measurement	Cardiovascular
0.7234	3.34E-75	3.46E-72	PGS003032	EFO_0004611	low density lipoprotein cholesterol measurement	low density lipoprotein cholesterol measurement	EFO_0004611	Cardiovascular measurement	Cardiovascular	PGS003030	EFO_0004611	low density lipoprotein cholesterol measurement	measurement	EFO_0004611	Cardiovascular measurement	Cardiovascular
0.7233	3.45E-75	3.58E-72	PGS000731	EFO_0000707	squamous cell carcinoma	squamous cell carcinoma	EFO_0000707	Cancer	Neoplasms	PGS001257	EFO_0003958	sunburn	sunburn	EFO_0003958	Other disease	Dermatological
0.7233	3.52E-75	3.65E-72	PGS001149	EFO_0005409	fat body mass	fat body mass	EFO_0005409	Other measurement	Body measurement	PGS001951	EFO_0005409	fat body mass	fat body mass	EFO_0005409	Other measurement	Body measurement
0.7231	4.00E-75	4.14E-72	PGS001751	EFO_0006930	brain volume measurement|neuroimaging measurement	brain volume measurement	EFO_0006930	Other measurement	Neurological	PGS001738	EFO_0006930	measurement	brain volume measurement	EFO_0006930	Other measurement	Neurological
0.7231	4.04E-75	4.18E-72	PGS003089	MONDO_0005148	type 2 diabetes mellitus	type 2 diabetes mellitus	MONDO_0005148	Metabolic disorder	Metabolic	PGS002354	MONDO_0005148	type 2 diabetes mellitus	type 2 diabetes mellitus	MONDO_0005148	Digestive system disorder	Gastrointestinal
0.7231	4.04E-75	4.18E-72	PGS003089	MONDO_0005148	type 2 diabetes mellitus	type 2 diabetes mellitus	MONDO_0005148	Metabolic disorder	Metabolic	PGS002354	MONDO_0005148	type 2 diabetes mellitus	type 2 diabetes mellitus	MONDO_0005148	Metabolic disorder	Metabolic
0.7231	4.04E-75	4.18E-72	PGS003089	MONDO_0005148	type 2 diabetes mellitus	type 2 diabetes mellitus	MONDO_0005148	Digestive system disorder	Gastrointestinal	PGS002354	MONDO_0005148	type 2 diabetes mellitus	type 2 diabetes mellitus	MONDO_0005148	Metabolic disorder	Metabolic
0.7231	4.04E-75	4.18E-72	PGS003089	MONDO_0005148	type 2 diabetes mellitus	type 2 diabetes mellitus	MONDO_0005148	Digestive system disorder	Gastrointestinal	PGS002354	MONDO_0005148	type 2 diabetes mellitus	type 2 diabetes mellitus	MONDO_0005148	Digestive system disorder	Gastrointestinal
0.7231	4.07E-75	4.21E-72	PGS003065	EFO_0006527	smoking status measurement	smoking status measurement	EFO_0006527	Other measurement	Psychiatric	PGS003059	EFO_0006527	smoking status measurement	smoking status measurement	EFO_0006527	Other measurement	Psychiatric
0.7231	4.09E-75	4.23E-72	PGS003136	EFO_0004574	total cholesterol measurement	total cholesterol measurement	EFO_0004574	Lipid or lipoprotein measurement	Metabolic	PGS003137	EFO_0004574	total cholesterol measurement	total cholesterol measurement	EFO_0004574	Lipid or lipoprotein measurement	Metabolic
0.723	4.18E-75	4.33E-72	PGS000783	EFO_0000305	breast carcinoma	breast carcinoma	EFO_0000305	Cancer	Neoplasms	PGS000002	EFO_1000649	estrogen-receptor positive breast cancer	cancer	EFO_1000649	Cancer	Neoplasms
0.723	4.36E-75	4.51E-72	PGS002854	EFO_0004340	body mass index	body mass index	EFO_0004340	Body measurement	Body measurement	PGS001228	EFO_0004340	body mass index	body mass index	EFO_0004340	Body measurement	Body measurement
0.723	4.36E-75	4.51E-72	PGS002660	EFO_0004309	platelet count	platelet count	EFO_0004309	Hematological measurement	Hematological	PGS000186	EFO_0004309	platelet count	platelet count	EFO_0004309	Hematological measurement	Hematological
0.723	4.38E-75	4.53E-72	PGS001229	EFO_0004339	body height	body height	EFO_0004339	Body measurement	Body measurement	PGS000998	EFO_0009819	comparative body size at age 10, self-reported	reported	EFO_0009819	Other trait	Body measurement
0.723	4.42E-75	4.57E-72	PGS002989	EFO_0004339	body height	body height	EFO_0004339	Body measurement	Body measurement	PGS002990	EFO_0004339	body height	body height	EFO_0004339	Body measurement	Body measurement
0.723	4.43E-75	4.58E-72	PGS000538	EFO_0000305	breast carcinoma	breast carcinoma	EFO_0000305	Cancer	Neoplasms	PGS000529	EFO_0000305	breast carcinoma	breast carcinoma	EFO_0000305	Cancer	Neoplasms
0.7229	4.58E-75	4.73E-72	PGS001457	EFO_0006930	brain volume measurement|neuroimaging measurement	brain volume measurement	EFO_0006930	Other measurement	Neurological	PGS001683	EFO_0006930	measurement	brain volume measurement	EFO_0006930	Other measurement	Neurological
0.7229	4.59E-75	4.75E-72	PGS003198	EFO_0004631	vitamin D measurement	vitamin D measurement	EFO_0004631	Lipid or lipoprotein measurement	Metabolic	PGS002623	EFO_0004631	vitamin D measurement	vitamin D measurement	EFO_0004631	Lipid or lipoprotein measurement	Metabolic
0.7229	4.62E-75	4.77E-72	PGS002150	EFO_0004611	low density lipoprotein cholesterol measurement	low density lipoprotein cholesterol measurement	EFO_0004611	Cardiovascular measurement	Cardiovascular	PGS000310	EFO_0004611	low density lipoprotein cholesterol measurement	measurement	EFO_0004611	Lipid or lipoprotein measurement	Metabolic
0.7229	4.62E-75	4.77E-72	PGS002150	EFO_0004611	low density lipoprotein cholesterol measurement	low density lipoprotein cholesterol measurement	EFO_0004611	Lipid or lipoprotein measurement	Metabolic	PGS000310	EFO_0004611	low density lipoprotein cholesterol measurement	measurement	EFO_0004611	Lipid or lipoprotein measurement	Metabolic
0.7229	4.62E-75	4.77E-72	PGS002150	EFO_0004611	low density lipoprotein cholesterol measurement	low density lipoprotein cholesterol measurement	EFO_0004611	Lipid or lipoprotein measurement	Metabolic	PGS000310	EFO_0004611	low density lipoprotein cholesterol measurement	measurement	EFO_0004611	Cardiovascular measurement	Cardiovascular
0.7229	4.62E-75	4.77E-72	PGS002150	EFO_0004611	low density lipoprotein cholesterol measurement	low density lipoprotein cholesterol measurement	EFO_0004611	Cardiovascular measurement	Cardiovascular	PGS000310	EFO_0004611	low density lipoprotein cholesterol measurement	measurement	EFO_0004611	Cardiovascular measurement	Cardiovascular
0.7228	4.77E-75	4.93E-72	PGS000516	EFO_0000305	breast carcinoma	breast carcinoma	EFO_0000305	Cancer	Neoplasms	PGS000513	EFO_0000305	breast carcinoma	breast carcinoma	EFO_0000305	Cancer	Neoplasms
0.7228	4.90E-75	5.06E-72	PGS003031	EFO_0004611	low density lipoprotein cholesterol measurement	low density lipoprotein cholesterol measurement	EFO_0004611	Lipid or lipoprotein measurement	Metabolic	PGS002620	EFO_0004574	total cholesterol measurement	total cholesterol measurement	EFO_0004574	Lipid or lipoprotein measurement	Metabolic
0.7228	4.90E-75	5.06E-72	PGS003031	EFO_0004611	low density lipoprotein cholesterol measurement	low density lipoprotein cholesterol measurement	EFO_0004611	Cardiovascular measurement	Cardiovascular	PGS002620	EFO_0004574	total cholesterol measurement	total cholesterol measurement	EFO_0004574	Lipid or lipoprotein measurement	Metabolic
0.7228	4.92E-75	5.08E-72	PGS000536	EFO_0000305	breast carcinoma	breast carcinoma	EFO_0000305	Cancer	Neoplasms	PGS000512	EFO_0000305	breast carcinoma	breast carcinoma	EFO_0000305	Cancer	Neoplasms
0.7227	5.24E-75	5.40E-72	PGS000539	EFO_0000305	breast carcinoma	breast carcinoma	EFO_0000305	Cancer	Neoplasms	PGS000526	EFO_0000305	breast carcinoma	breast carcinoma	EFO_0000305	Cancer	Neoplasms
0.7227	5.24E-75	5.40E-72	PGS000539	EFO_0000305	breast carcinoma	breast carcinoma	EFO_0000305	Cancer	Neoplasms	PGS000525	EFO_0000305	breast carcinoma	breast carcinoma	EFO_0000305	Cancer	Neoplasms
-0.7227	5.28E-75	5.45E-72	PGS002780	MONDO_0005148	type 2 diabetes mellitus	type 2 diabetes mellitus	MONDO_0005148	Digestive system disorder	Gastrointestinal	PGS001357	MONDO_0005148	type 2 diabetes mellitus	type 2 diabetes mellitus	MONDO_0005148	Metabolic disorder	Metabolic
-0.7227	5.28E-75	5.45E-72	PGS002780	MONDO_0005148	type 2 diabetes mellitus	type 2 diabetes mellitus	MONDO_0005148	Metabolic disorder	Metabolic	PGS001357	MONDO_0005148	type 2 diabetes mellitus	type 2 diabetes mellitus	MONDO_0005148	Metabolic disorder	Metabolic
-0.7227	5.28E-75	5.45E-72	PGS002780	MONDO_0005148	type 2 diabetes mellitus	type 2 diabetes mellitus	MONDO_0005148	Digestive system disorder	Gastrointestinal	PGS001357	MONDO_0005148	type 2 diabetes mellitus	type 2 diabetes mellitus	MONDO_0005148	Digestive system disorder	Gastrointestinal
-0.7227	5.28E-75	5.45E-72	PGS002780	MONDO_0005148	type 2 diabetes mellitus	type 2 diabetes mellitus	MONDO_0005148	Metabolic disorder	Metabolic	PGS001357	MONDO_0005148	type 2 diabetes mellitus	type 2 diabetes mellitus	MONDO_0005148	Digestive system disorder	Gastrointestinal
0.7227	5.41E-75	5.58E-72	PGS000893	EFO_0004611	low density lipoprotein cholesterol measurement	low density lipoprotein cholesterol measurement	EFO_0004611	Lipid or lipoprotein measurement	Metabolic	PGS000892	EFO_0004611	low density lipoprotein cholesterol measurement	measurement	EFO_0004611	Lipid or lipoprotein measurement	Metabolic
0.7227	5.41E-75	5.58E-72	PGS000893	EFO_0004611	low density lipoprotein cholesterol measurement	low density lipoprotein cholesterol measurement	EFO_0004611	Cardiovascular measurement	Cardiovascular	PGS000892	EFO_0004611	low density lipoprotein cholesterol measurement	measurement	EFO_0004611	Lipid or lipoprotein measurement	Metabolic
0.7227	5.41E-75	5.58E-72	PGS000893	EFO_0004611	low density lipoprotein cholesterol measurement	low density lipoprotein cholesterol measurement	EFO_0004611	Cardiovascular measurement	Cardiovascular	PGS000892	EFO_0004611	low density lipoprotein cholesterol measurement	measurement	EFO_0004611	Cardiovascular measurement	Cardiovascular
0.7227	5.41E-75	5.58E-72	PGS000893	EFO_0004611	low density lipoprotein cholesterol measurement	low density lipoprotein cholesterol measurement	EFO_0004611	Lipid or lipoprotein measurement	Metabolic	PGS000892	EFO_0004611	low density lipoprotein cholesterol measurement	measurement	EFO_0004611	Cardiovascular measurement	Cardiovascular
0.7226	5.50E-75	5.67E-72	PGS001488	EFO_0006930	brain volume measurement|neuroimaging measurement	brain volume measurement	EFO_0006930	Other measurement	Neurological	PGS001672	EFO_0006930	measurement	brain volume measurement	EFO_0006930	Other measurement	Neurological
0.7226	5.51E-75	5.68E-72	PGS000102	EFO_0005091	monocyte count	monocyte count	EFO_0005091	Hematological measurement	Hematological	PGS001163	EFO_0005091	monocyte count	monocyte count	EFO_0005091	Hematological measurement	Hematological
0.7226	5.75E-75	5.93E-72	PGS000500	EFO_0000305	breast carcinoma	breast carcinoma	EFO_0000305	Cancer	Neoplasms	PGS000531	EFO_0000305	breast carcinoma	breast carcinoma	EFO_0000305	Cancer	Neoplasms
0.7226	5.82E-75	5.99E-72	PGS003137	EFO_0004574	total cholesterol measurement	total cholesterol measurement	EFO_0004574	Lipid or lipoprotein measurement	Metabolic	PGS003141	EFO_0004574	total cholesterol measurement	total cholesterol measurement	EFO_0004574	Lipid or lipoprotein measurement	Metabolic
0.7225	5.93E-75	6.11E-72	PGS001105	EFO_0007800	body fat percentage	body fat percentage	EFO_0007800	Body measurement	Body measurement	PGS001144	EFO_0005409	fat body mass	fat body mass	EFO_0005409	Other measurement	Body measurement
0.7225	5.99E-75	6.17E-72	PGS001477	EFO_0006930	brain volume measurement|neuroimaging measurement	brain volume measurement	EFO_0006930	Other measurement	Neurological	PGS001473	EFO_0006930	measurement	brain volume measurement	EFO_0006930	Other measurement	Neurological
0.7225	5.99E-75	6.17E-72	PGS000529	EFO_0000305	breast carcinoma	breast carcinoma	EFO_0000305	Cancer	Neoplasms	PGS000505	EFO_0000305	breast carcinoma	breast carcinoma	EFO_0000305	Cancer	Neoplasms
0.7225	5.99E-75	6.17E-72	PGS000506	EFO_0000305	breast carcinoma	breast carcinoma	EFO_0000305	Cancer	Neoplasms	PGS000529	EFO_0000305	breast carcinoma	breast carcinoma	EFO_0000305	Cancer	Neoplasms
0.7225	6.04E-75	6.22E-72	PGS001480	EFO_0006930	brain volume measurement|neuroimaging measurement	brain volume measurement	EFO_0006930	Other measurement	Neurological	PGS001683	EFO_0006930	measurement	brain volume measurement	EFO_0006930	Other measurement	Neurological
0.7225	6.08E-75	6.26E-72	PGS002548	EFO_0004612	high density lipoprotein cholesterol measurement	high density lipoprotein cholesterol measurement	EFO_0004612	Lipid or lipoprotein measurement	Metabolic	PGS002781	EFO_0004612	high density lipoprotein cholesterol measurement	measurement	EFO_0004612	Lipid or lipoprotein measurement	Metabolic
0.7225	6.22E-75	6.40E-72	PGS002632	EFO_0003923	bone density	bone density	EFO_0003923	Other measurement	Skeletal	PGS001529	EFO_0007772	measurement	measurement	EFO_0007772	Other measurement	Skeletal
0.7224	6.43E-75	6.61E-72	PGS000940	HP_0002024	Malabsorption	Malabsorption	HP_0002024	Other trait	Gastrointestinal	PGS001065	EFO_0008111	diet measurement	diet measurement	EFO_0008111	Other measurement	Activities
0.7224	6.58E-75	6.77E-72	PGS000514	EFO_0000305	breast carcinoma	breast carcinoma	EFO_0000305	Cancer	Neoplasms	PGS000473	EFO_0000305	breast carcinoma	breast carcinoma	EFO_0000305	Cancer	Neoplasms
0.7223	6.73E-75	6.92E-72	PGS002850	EFO_0004340	body mass index	body mass index	EFO_0004340	Body measurement	Body measurement	PGS002169	EFO_0005409	fat body mass	fat body mass	EFO_0005409	Other measurement	Body measurement
0.7223	6.86E-75	7.05E-72	PGS001989	EFO_0004527	mean corpuscular hemoglobin	mean corpuscular hemoglobin	EFO_0004527	Hematological measurement	Hematological	PGS000174	EFO_0004527	mean corpuscular hemoglobin	mean corpuscular hemoglobin	EFO_0004527	Hematological measurement	Hematological
0.7223	6.90E-75	7.09E-72	PGS000008	EFO_1000649	estrogen-receptor positive breast cancer	estrogen-receptor positive breast cancer	EFO_1000649	Cancer	Neoplasms	PGS000510	EFO_0000305	breast carcinoma	breast carcinoma	EFO_0000305	Cancer	Neoplasms
0.7223	6.92E-75	7.11E-72	PGS001781	MONDO_0005148	type 2 diabetes mellitus	type 2 diabetes mellitus	MONDO_0005148	Digestive system disorder	Gastrointestinal	PGS002771	MONDO_0005148	type 2 diabetes mellitus	type 2 diabetes mellitus	MONDO_0005148	Metabolic disorder	Metabolic
0.7223	6.92E-75	7.11E-72	PGS001781	MONDO_0005148	type 2 diabetes mellitus	type 2 diabetes mellitus	MONDO_0005148	Metabolic disorder	Metabolic	PGS002771	MONDO_0005148	type 2 diabetes mellitus	type 2 diabetes mellitus	MONDO_0005148	Metabolic disorder	Metabolic
0.7223	6.92E-75	7.11E-72	PGS001781	MONDO_0005148	type 2 diabetes mellitus	type 2 diabetes mellitus	MONDO_0005148	Digestive system disorder	Gastrointestinal	PGS002771	MONDO_0005148	type 2 diabetes mellitus	type 2 diabetes mellitus	MONDO_0005148	Digestive system disorder	Gastrointestinal
0.7223	6.92E-75	7.11E-72	PGS001781	MONDO_0005148	type 2 diabetes mellitus	type 2 diabetes mellitus	MONDO_0005148	Metabolic disorder	Metabolic	PGS002771	MONDO_0005148	type 2 diabetes mellitus	type 2 diabetes mellitus	MONDO_0005148	Digestive system disorder	Gastrointestinal
0.7223	7.08E-75	7.27E-72	PGS001336	EFO_0000305	breast carcinoma	breast carcinoma	EFO_0000305	Cancer	Neoplasms	PGS000480	EFO_0000305	breast carcinoma	breast carcinoma	EFO_0000305	Cancer	Neoplasms
0.7222	7.34E-75	7.54E-72	PGS000176	EFO_0004526	mean corpuscular volume	mean corpuscular volume	EFO_0004526	Hematological measurement	Hematological	PGS001989	EFO_0004527	mean corpuscular hemoglobin	mean corpuscular hemoglobin	EFO_0004527	Hematological measurement	Hematological
0.7222	7.58E-75	7.78E-72	PGS002844	EFO_0004340	body mass index	body mass index	EFO_0004340	Body measurement	Body measurement	PGS002841	EFO_0004340	body mass index	body mass index	EFO_0004340	Body measurement	Body measurement
0.7222	7.61E-75	7.82E-72	PGS003185	EFO_0004631	vitamin D measurement	vitamin D measurement	EFO_0004631	Lipid or lipoprotein measurement	Metabolic	PGS000702	EFO_0004631	vitamin D measurement	vitamin D measurement	EFO_0004631	Lipid or lipoprotein measurement	Metabolic
0.7221	7.77E-75	7.98E-72	PGS003198	EFO_0004631	vitamin D measurement	vitamin D measurement	EFO_0004631	Lipid or lipoprotein measurement	Metabolic	PGS003191	EFO_0004631	vitamin D measurement	vitamin D measurement	EFO_0004631	Lipid or lipoprotein measurement	Metabolic
0.7221	8.03E-75	8.24E-72	PGS000705	EFO_0004210	gallstones	gallstones	EFO_0004210	Digestive system disorder	Gastrointestinal	PGS001256	EFO_0004210	gallstones	gallstones	EFO_0004210	Digestive system disorder	Gastrointestinal
0.722	8.34E-75	8.56E-72	PGS003135	EFO_0004574	total cholesterol measurement	total cholesterol measurement	EFO_0004574	Lipid or lipoprotein measurement	Metabolic	PGS003134	EFO_0004574	total cholesterol measurement	total cholesterol measurement	EFO_0004574	Lipid or lipoprotein measurement	Metabolic
0.722	8.44E-75	8.65E-72	PGS001664	EFO_0006930	brain volume measurement|neuroimaging measurement	brain volume measurement	EFO_0006930	Other measurement	Neurological	PGS001662	EFO_0006930	measurement	brain volume measurement	EFO_0006930	Other measurement	Neurological
0.722	8.57E-75	8.78E-72	PGS002624	EFO_0004343	waist-hip ratio	waist-hip ratio	EFO_0004343	Body measurement	Body measurement	PGS002477	EFO_0004343	waist-hip ratio	waist-hip ratio	EFO_0004343	Body measurement	Body measurement
0.722	8.73E-75	8.95E-72	PGS000699	EFO_0004530	triglyceride measurement	triglyceride measurement	EFO_0004530	Lipid or lipoprotein measurement	Metabolic	PGS002197	EFO_0004530	triglyceride measurement	triglyceride measurement	EFO_0004530	Lipid or lipoprotein measurement	Metabolic
0.7219	8.84E-75	9.06E-72	PGS000831	EFO_0004574	total cholesterol measurement	total cholesterol measurement	EFO_0004574	Lipid or lipoprotein measurement	Metabolic	PGS002571	EFO_0004574	total cholesterol measurement	total cholesterol measurement	EFO_0004574	Lipid or lipoprotein measurement	Metabolic
0.7219	8.97E-75	9.19E-72	PGS002169	EFO_0005409	fat body mass	fat body mass	EFO_0005409	Other measurement	Body measurement	PGS001148	EFO_0005409	fat body mass	fat body mass	EFO_0005409	Other measurement	Body measurement
0.7219	8.97E-75	9.19E-72	PGS002133	EFO_0007800	body fat percentage	body fat percentage	EFO_0007800	Body measurement	Body measurement	PGS002849	EFO_0004340	body mass index	body mass index	EFO_0004340	Body measurement	Body measurement
0.7219	9.38E-75	9.60E-72	PGS002632	EFO_0003923	bone density	bone density	EFO_0003923	Other measurement	Skeletal	PGS001403	EFO_0009270	heel bone mineral density	heel bone mineral density	EFO_0009270	Other measurement	Skeletal
-0.7218	9.54E-75	9.77E-72	PGS002835	EFO_0007878	alcohol consumption measurement	alcohol consumption measurement	EFO_0007878	Other measurement	Psychiatric	PGS003376	EFO_0007878	alcohol consumption measurement	alcohol consumption measurement	EFO_0007878	Other measurement	Psychiatric
0.7218	9.88E-75	1.01E-71	PGS000873	EFO_0000305	breast carcinoma	breast carcinoma	EFO_0000305	Cancer	Neoplasms	PGS000487	EFO_0000305	breast carcinoma	breast carcinoma	EFO_0000305	Cancer	Neoplasms
0.7218	9.88E-75	1.01E-71	PGS000873	EFO_0000305	breast carcinoma	breast carcinoma	EFO_0000305	Cancer	Neoplasms	PGS000485	EFO_0000305	breast carcinoma	breast carcinoma	EFO_0000305	Cancer	Neoplasms
0.7217	1.07E-74	1.10E-71	PGS001980	EFO_0011005	urea measurement	urea measurement	EFO_0011005	Other measurement	Metabolic	PGS000701	EFO_0009795	serum urea measurement	serum urea measurement	EFO_0009795	Other measurement	Metabolic
0.7216	1.09E-74	1.11E-71	PGS000538	EFO_0000305	breast carcinoma	breast carcinoma	EFO_0000305	Cancer	Neoplasms	PGS001804	EFO_0000305	breast carcinoma	breast carcinoma	EFO_0000305	Cancer	Neoplasms
0.7216	1.09E-74	1.12E-71	PGS003200	EFO_0004631	vitamin D measurement	vitamin D measurement	EFO_0004631	Lipid or lipoprotein measurement	Metabolic	PGS000702	EFO_0004631	vitamin D measurement	vitamin D measurement	EFO_0004631	Lipid or lipoprotein measurement	Metabolic
0.7216	1.13E-74	1.15E-71	PGS000505	EFO_0000305	breast carcinoma	breast carcinoma	EFO_0000305	Cancer	Neoplasms	PGS000052	EFO_0000305	breast carcinoma	breast carcinoma	EFO_0000305	Cancer	Neoplasms
0.7216	1.13E-74	1.15E-71	PGS000506	EFO_0000305	breast carcinoma	breast carcinoma	EFO_0000305	Cancer	Neoplasms	PGS000052	EFO_0000305	breast carcinoma	breast carcinoma	EFO_0000305	Cancer	Neoplasms
0.7216	1.13E-74	1.16E-71	PGS001678	EFO_0006930	brain volume measurement|neuroimaging measurement	brain volume measurement	EFO_0006930	Other measurement	Neurological	PGS001679	EFO_0006930	measurement	brain volume measurement	EFO_0006930	Other measurement	Neurological
0.7215	1.20E-74	1.22E-71	PGS001709	EFO_0006930	brain volume measurement|neuroimaging measurement	brain volume measurement	EFO_0006930	Other measurement	Neurological	PGS001710	EFO_0006930	measurement	brain volume measurement	EFO_0006930	Other measurement	Neurological
0.7215	1.20E-74	1.22E-71	PGS003408	EFO_0003870	brain aneurysm|female	brain aneurysm	EFO_0003870	Cardiovascular disease	Cardiovascular	PGS003410	EFO_0003870	brain aneurysm|male	brain aneurysm	EFO_0003870	Cardiovascular disease	Cardiovascular
0.7215	1.20E-74	1.22E-71	PGS003408	EFO_0003870	brain aneurysm|female	brain aneurysm	EFO_0003870	Neurological disorder	Neurological	PGS003410	EFO_0003870	brain aneurysm|male	brain aneurysm	EFO_0003870	Cardiovascular disease	Cardiovascular
0.7215	1.20E-74	1.22E-71	PGS003408	EFO_0003870	brain aneurysm|female	brain aneurysm	EFO_0003870	Neurological disorder	Neurological	PGS003410	EFO_0003870	brain aneurysm|male	brain aneurysm	EFO_0003870	Neurological disorder	Neurological
0.7215	1.20E-74	1.22E-71	PGS003408	EFO_0003870	brain aneurysm|female	brain aneurysm	EFO_0003870	Cardiovascular disease	Cardiovascular	PGS003410	EFO_0003870	brain aneurysm|male	brain aneurysm	EFO_0003870	Neurological disorder	Neurological
0.7214	1.25E-74	1.28E-71	PGS003065	EFO_0006527	smoking status measurement	smoking status measurement	EFO_0006527	Other measurement	Psychiatric	PGS003066	EFO_0006527	smoking status measurement	smoking status measurement	EFO_0006527	Other measurement	Psychiatric
0.7214	1.26E-74	1.29E-71	PGS000069	EFO_1001516	ovarian serous carcinoma	ovarian serous carcinoma	EFO_1001516	Cancer	Neoplasms	PGS000551	EFO_0003893	ovarian neoplasm	ovarian neoplasm	EFO_0003893	Cancer	Neoplasms
0.7214	1.28E-74	1.31E-71	PGS000215	EFO_1000294	HER2 Positive Breast Carcinoma	HER2 Positive Breast Carcinoma	EFO_1000294	Cancer	Neoplasms	PGS000344	EFO_0000305	breast carcinoma	breast carcinoma	EFO_0000305	Cancer	Neoplasms
0.7214	1.30E-74	1.32E-71	PGS002965	EFO_0004339	body height	body height	EFO_0004339	Body measurement	Body measurement	PGS002404	EFO_0004339	body height	body height	EFO_0004339	Body measurement	Body measurement
0.7214	1.30E-74	1.32E-71	PGS000795	EFO_0001663	prostate carcinoma	prostate carcinoma	EFO_0001663	Cancer	Neoplasms	PGS002796	EFO_0001663	prostate carcinoma	prostate carcinoma	EFO_0001663	Cancer	Neoplasms
0.7214	1.31E-74	1.33E-71	PGS001735	EFO_0006930	brain volume measurement|neuroimaging measurement	brain volume measurement	EFO_0006930	Other measurement	Neurological	PGS001722	EFO_0006930	measurement	brain volume measurement	EFO_0006930	Other measurement	Neurological
0.7214	1.32E-74	1.34E-71	PGS003036	EFO_0004611	low density lipoprotein cholesterol measurement	low density lipoprotein cholesterol measurement	EFO_0004611	Cardiovascular measurement	Cardiovascular	PGS000889	EFO_0004611	low density lipoprotein cholesterol measurement	measurement	EFO_0004611	Lipid or lipoprotein measurement	Metabolic
0.7214	1.32E-74	1.34E-71	PGS003036	EFO_0004611	low density lipoprotein cholesterol measurement	low density lipoprotein cholesterol measurement	EFO_0004611	Lipid or lipoprotein measurement	Metabolic	PGS000889	EFO_0004611	low density lipoprotein cholesterol measurement	measurement	EFO_0004611	Cardiovascular measurement	Cardiovascular
0.7214	1.32E-74	1.34E-71	PGS003036	EFO_0004611	low density lipoprotein cholesterol measurement	low density lipoprotein cholesterol measurement	EFO_0004611	Lipid or lipoprotein measurement	Metabolic	PGS000889	EFO_0004611	low density lipoprotein cholesterol measurement	measurement	EFO_0004611	Lipid or lipoprotein measurement	Metabolic
0.7214	1.32E-74	1.34E-71	PGS003036	EFO_0004611	low density lipoprotein cholesterol measurement	low density lipoprotein cholesterol measurement	EFO_0004611	Cardiovascular measurement	Cardiovascular	PGS000889	EFO_0004611	low density lipoprotein cholesterol measurement	measurement	EFO_0004611	Cardiovascular measurement	Cardiovascular
0.7214	1.32E-74	1.35E-71	PGS000312	EFO_0004530	triglyceride measurement	triglyceride measurement	EFO_0004530	Lipid or lipoprotein measurement	Metabolic	PGS002425	EFO_0004530	triglyceride measurement	triglyceride measurement	EFO_0004530	Lipid or lipoprotein measurement	Metabolic

0.7214	1.33E-74	1.35E-71	PGS002534	EFO_0003923	bone density	bone density	EFO_0003923	Other measurement	Skeletal	PGS002632	EFO_0003923	bone density	bone density	EFO_0003923	Other measurement	Skeletal
-0.7213	1.37E-74	1.39E-71	PGS003268	EFO_0010472	creatine measurement|glomerular filtration rate	creatine measurement	EFO_0010472	Other measurement	Metabolic	PGS003263	EFO_0010472	creatine measurement|glomerular filtration rate	creatine measurement	EFO_0010472	Other measurement	Metabolic
0.7213	1.37E-74	1.40E-71	PGS000688	EFO_0004611	low density lipoprotein cholesterol measurement	low density lipoprotein cholesterol measurement	EFO_0004611	Lipid or lipoprotein measurement	Metabolic	PGS002108	EFO_0004574	total cholesterol measurement	total cholesterol measurement	EFO_0004574	Lipid or lipoprotein measurement	Metabolic
0.7213	1.37E-74	1.40E-71	PGS000688	EFO_0004611	low density lipoprotein cholesterol measurement	low density lipoprotein cholesterol measurement	EFO_0004611	Cardiovascular measurement	Cardiovascular	PGS002108	EFO_0004574	total cholesterol measurement	total cholesterol measurement	EFO_0004574	Lipid or lipoprotein measurement	Metabolic
0.7213	1.40E-74	1.43E-71	PGS001488	EFO_0006930	brain volume measurement|neuroimaging measurement	brain volume measurement	EFO_0006930	Other measurement	Neurological	PGS001480	EFO_0006930	measurement	brain volume measurement	EFO_0006930	Other measurement	Neurological
0.7213	1.41E-74	1.43E-71	PGS000535	EFO_0000305	breast carcinoma	breast carcinoma	EFO_0000305	Cancer	Neoplasms	PGS000531	EFO_0000305	breast carcinoma	breast carcinoma	EFO_0000305	Cancer	Neoplasms
0.7213	1.41E-74	1.44E-71	PGS003034	EFO_0004611	low density lipoprotein cholesterol measurement	low density lipoprotein cholesterol measurement	EFO_0004611	Cardiovascular measurement	Cardiovascular	PGS000892	EFO_0004611	low density lipoprotein cholesterol measurement	measurement	EFO_0004611	Cardiovascular measurement	Cardiovascular
0.7213	1.41E-74	1.44E-71	PGS003034	EFO_0004611	low density lipoprotein cholesterol measurement	low density lipoprotein cholesterol measurement	EFO_0004611	Lipid or lipoprotein measurement	Metabolic	PGS000892	EFO_0004611	low density lipoprotein cholesterol measurement	measurement	EFO_0004611	Lipid or lipoprotein measurement	Metabolic
0.7213	1.41E-74	1.44E-71	PGS003034	EFO_0004611	low density lipoprotein cholesterol measurement	low density lipoprotein cholesterol measurement	EFO_0004611	Cardiovascular measurement	Cardiovascular	PGS000892	EFO_0004611	low density lipoprotein cholesterol measurement	measurement	EFO_0004611	Lipid or lipoprotein measurement	Metabolic
0.7213	1.41E-74	1.44E-71	PGS003034	EFO_0004611	low density lipoprotein cholesterol measurement	low density lipoprotein cholesterol measurement	EFO_0004611	Lipid or lipoprotein measurement	Metabolic	PGS000892	EFO_0004611	low density lipoprotein cholesterol measurement	measurement	EFO_0004611	Cardiovascular measurement	Cardiovascular
0.7213	1.42E-74	1.44E-71	PGS002840	EFO_0004340	body mass index	body mass index	EFO_0004340	Body measurement	Body measurement	PGS002169	EFO_0005409	fat body mass	fat body mass	EFO_0005409	Other measurement	Body measurement
0.7213	1.43E-74	1.45E-71	PGS001683	EFO_0006930	brain volume measurement|neuroimaging measurement	brain volume measurement	EFO_0006930	Other measurement	Neurological	PGS001672	EFO_0006930	measurement	brain volume measurement	EFO_0006930	Other measurement	Neurological
0.7212	1.44E-74	1.47E-71	PGS000014	MONDO_0005148	type 2 diabetes mellitus	type 2 diabetes mellitus	MONDO_0005148	Metabolic disorder	Metabolic	PGS002779	MONDO_0005148	type 2 diabetes mellitus	type 2 diabetes mellitus	MONDO_0005148	Metabolic disorder	Metabolic
0.7212	1.44E-74	1.47E-71	PGS000014	MONDO_0005148	type 2 diabetes mellitus	type 2 diabetes mellitus	MONDO_0005148	Digestive system disorder	Gastrointestinal	PGS002779	MONDO_0005148	type 2 diabetes mellitus	type 2 diabetes mellitus	MONDO_0005148	Metabolic disorder	Metabolic
0.7212	1.44E-74	1.47E-71	PGS000014	MONDO_0005148	type 2 diabetes mellitus	type 2 diabetes mellitus	MONDO_0005148	Digestive system disorder	Gastrointestinal	PGS002779	MONDO_0005148	type 2 diabetes mellitus	type 2 diabetes mellitus	MONDO_0005148	Digestive system disorder	Gastrointestinal
0.7212	1.44E-74	1.47E-71	PGS000014	MONDO_0005148	type 2 diabetes mellitus	type 2 diabetes mellitus	MONDO_0005148	Metabolic disorder	Metabolic	PGS002779	MONDO_0005148	type 2 diabetes mellitus	type 2 diabetes mellitus	MONDO_0005148	Digestive system disorder	Gastrointestinal
0.7212	1.45E-74	1.47E-71	PGS002540	EFO_0000400	diabetes mellitus	diabetes mellitus	EFO_0000400	Metabolic disorder	Metabolic	PGS002599	EFO_0004541	HbA1c measurement	HbA1c measurement	EFO_0004541	Hematological measurement	Hematological
0.7212	1.45E-74	1.47E-71	PGS002540	EFO_0000400	diabetes mellitus	diabetes mellitus	EFO_0000400	Digestive system disorder	Gastrointestinal	PGS002599	EFO_0004541	HbA1c measurement	HbA1c measurement	EFO_0004541	Hematological measurement	Hematological
0.7212	1.45E-74	1.48E-71	PGS002556	EFO_0004611	low density lipoprotein cholesterol measurement	low density lipoprotein cholesterol measurement	EFO_0004611	Cardiovascular measurement	Cardiovascular	PGS003038	EFO_0004611	low density lipoprotein cholesterol measurement	measurement	EFO_0004611	Lipid or lipoprotein measurement	Metabolic
0.7212	1.45E-74	1.48E-71	PGS002556	EFO_0004611	low density lipoprotein cholesterol measurement	low density lipoprotein cholesterol measurement	EFO_0004611	Cardiovascular measurement	Cardiovascular	PGS003038	EFO_0004611	low density lipoprotein cholesterol measurement	measurement	EFO_0004611	Cardiovascular measurement	Cardiovascular
0.7212	1.45E-74	1.48E-71	PGS002556	EFO_0004611	low density lipoprotein cholesterol measurement	low density lipoprotein cholesterol measurement	EFO_0004611	Lipid or lipoprotein measurement	Metabolic	PGS003038	EFO_0004611	low density lipoprotein cholesterol measurement	measurement	EFO_0004611	Cardiovascular measurement	Cardiovascular
0.7212	1.45E-74	1.48E-71	PGS002556	EFO_0004611	low density lipoprotein cholesterol measurement	low density lipoprotein cholesterol measurement	EFO_0004611	Lipid or lipoprotein measurement	Metabolic	PGS003038	EFO_0004611	low density lipoprotein cholesterol measurement	measurement	EFO_0004611	Lipid or lipoprotein measurement	Metabolic
0.7212	1.46E-74	1.49E-71	PGS000534	EFO_0000305	breast carcinoma	breast carcinoma	EFO_0000305	Cancer	Neoplasms	PGS000008	EFO_1000649	estrogen-receptor positive breast cancer	cancer	EFO_1000649	Cancer	Neoplasms
0.7212	1.46E-74	1.49E-71	PGS000533	EFO_0000305	breast carcinoma	breast carcinoma	EFO_0000305	Cancer	Neoplasms	PGS000008	EFO_1000649	estrogen-receptor positive breast cancer	cancer	EFO_1000649	Cancer	Neoplasms
0.7212	1.52E-74	1.55E-71	PGS003185	EFO_0004631	vitamin D measurement	vitamin D measurement	EFO_0004631	Lipid or lipoprotein measurement	Metabolic	PGS002623	EFO_0004631	vitamin D measurement	vitamin D measurement	EFO_0004631	Lipid or lipoprotein measurement	Metabolic
0.7211	1.55E-74	1.58E-71	PGS000532	EFO_0000305	breast carcinoma	breast carcinoma	EFO_0000305	Cancer	Neoplasms	PGS000500	EFO_0000305	breast carcinoma	breast carcinoma	EFO_0000305	Cancer	Neoplasms
0.7211	1.58E-74	1.61E-71	PGS002118	EFO_0004462	PR interval	PR interval	EFO_0004462	Cardiovascular measurement	Cardiovascular	PGS001521	EFO_0004462	PR interval	PR interval	EFO_0004462	Cardiovascular measurement	Cardiovascular
0.7211	1.59E-74	1.61E-71	PGS002780	MONDO_0005148	type 2 diabetes mellitus	type 2 diabetes mellitus	MONDO_0005148	Digestive system disorder	Gastrointestinal	PGS003119	MONDO_0005148	type 2 diabetes mellitus	type 2 diabetes mellitus	MONDO_0005148	Digestive system disorder	Gastrointestinal
0.7211	1.59E-74	1.61E-71	PGS002780	MONDO_0005148	type 2 diabetes mellitus	type 2 diabetes mellitus	MONDO_0005148	Digestive system disorder	Gastrointestinal	PGS003119	MONDO_0005148	type 2 diabetes mellitus	type 2 diabetes mellitus	MONDO_0005148	Metabolic disorder	Metabolic
0.7211	1.59E-74	1.61E-71	PGS002780	MONDO_0005148	type 2 diabetes mellitus	type 2 diabetes mellitus	MONDO_0005148	Metabolic disorder	Metabolic	PGS003119	MONDO_0005148	type 2 diabetes mellitus	type 2 diabetes mellitus	MONDO_0005148	Metabolic disorder	Metabolic
0.7211	1.59E-74	1.61E-71	PGS002780	MONDO_0005148	type 2 diabetes mellitus	type 2 diabetes mellitus	MONDO_0005148	Metabolic disorder	Metabolic	PGS003119	MONDO_0005148	type 2 diabetes mellitus	type 2 diabetes mellitus	MONDO_0005148	Digestive system disorder	Gastrointestinal
0.7211	1.60E-74	1.62E-71	PGS000888	EFO_0004611	low density lipoprotein cholesterol measurement	low density lipoprotein cholesterol measurement	EFO_0004611	Lipid or lipoprotein measurement	Metabolic	PGS001895	EFO_0004574	total cholesterol measurement	total cholesterol measurement	EFO_0004574	Lipid or lipoprotein measurement	Metabolic
0.7211	1.60E-74	1.62E-71	PGS000888	EFO_0004611	low density lipoprotein cholesterol measurement	low density lipoprotein cholesterol measurement	EFO_0004611	Cardiovascular measurement	Cardiovascular	PGS001895	EFO_0004574	total cholesterol measurement	total cholesterol measurement	EFO_0004574	Lipid or lipoprotein measurement	Metabolic
0.721	1.73E-74	1.76E-71	PGS001684	EFO_0006930	brain volume measurement|neuroimaging measurement	brain volume measurement	EFO_0006930	Other measurement	Neurological	PGS001450	EFO_0006930	measurement	brain volume measurement	EFO_0006930	Other measurement	Neurological
0.721	1.75E-74	1.78E-71	PGS001822	EFO_0004274	gout	gout	EFO_0004274	Metabolic disorder	Metabolic	PGS001248	EFO_0004274	gout	gout	EFO_0004274	Metabolic disorder	Metabolic
0.7209	1.76E-74	1.79E-71	PGS002357	EFO_0004308	leukocyte count	leukocyte count	EFO_0004308	Hematological measurement	Hematological	PGS001239	EFO_0004308	leukocyte count	leukocyte count	EFO_0004308	Hematological measurement	Hematological
0.7209	1.77E-74	1.79E-71	PGS002645	EFO_0004468	glucose measurement	glucose measurement	EFO_0004468	Other measurement	Metabolic	PGS002938	EFO_0004468	glucose measurement	glucose measurement	EFO_0004468	Other measurement	Metabolic
0.7209	1.78E-74	1.80E-71	PGS000002	EFO_1000649	estrogen-receptor positive breast cancer	estrogen-receptor positive breast cancer	EFO_1000649	Cancer	Neoplasms	PGS001804	EFO_0000305	breast carcinoma	breast carcinoma	EFO_0000305	Cancer	Neoplasms
0.7209	1.80E-74	1.83E-71	PGS003140	EFO_0004574	total cholesterol measurement	total cholesterol measurement	EFO_0004574	Lipid or lipoprotein measurement	Metabolic	PGS003139	EFO_0004574	total cholesterol measurement	total cholesterol measurement	EFO_0004574	Lipid or lipoprotein measurement	Metabolic
0.7209	1.84E-74	1.87E-71	PGS000741	EFO_0001663	prostate carcinoma	prostate carcinoma	EFO_0001663	Cancer	Neoplasms	PGS001805	EFO_0001663	prostate carcinoma	prostate carcinoma	EFO_0001663	Cancer	Neoplasms
0.7208	1.88E-74	1.91E-71	PGS000297	EFO_0004339	body height	body height	EFO_0004339	Body measurement	Body measurement	PGS002146	EFO_0004339	body height	body height	EFO_0004339	Body measurement	Body measurement
0.7208	1.91E-74	1.93E-71	PGS002791	EFO_0001663	prostate carcinoma	prostate carcinoma	EFO_0001663	Cancer	Neoplasms	PGS002796	EFO_0001663	prostate carcinoma	prostate carcinoma	EFO_0001663	Cancer	Neoplasms
0.7208	1.91E-74	1.94E-71	PGS000774	EFO_1000649	estrogen-receptor positive breast cancer	estrogen-receptor positive breast cancer	EFO_1000649	Cancer	Neoplasms	PGS000004	EFO_0000305	breast carcinoma	breast carcinoma	EFO_0000305	Cancer	Neoplasms
0.7208	1.97E-74	2.00E-71	PGS002426	MONDO_0005148	type 2 diabetes mellitus	type 2 diabetes mellitus	MONDO_0005148	Digestive system disorder	Gastrointestinal	PGS002599	EFO_0004541	HbA1c measurement	HbA1c measurement	EFO_0004541	Hematological measurement	Hematological
0.7208	1.97E-74	2.00E-71	PGS002426	MONDO_0005148	type 2 diabetes mellitus	type 2 diabetes mellitus	MONDO_0005148	Metabolic disorder	Metabolic	PGS002599	EFO_0004541	HbA1c measurement	HbA1c measurement	EFO_0004541	Hematological measurement	Hematological
0.7206	2.20E-74	2.23E-71	PGS000783	EFO_0000305	breast carcinoma	breast carcinoma	EFO_0000305	Cancer	Neoplasms	PGS000214	MONDO_0021115	luminal B breast carcinoma	luminal B breast carcinoma	MONDO_0021115	Cancer	Neoplasms
0.7206	2.20E-74	2.23E-71	PGS001731	EFO_0006930	brain volume measurement|neuroimaging measurement	brain volume measurement	EFO_0006930	Other measurement	Neurological	PGS001715	EFO_0006930	measurement	brain volume measurement	EFO_0006930	Other measurement	Neurological
0.7206	2.23E-74	2.26E-71	PGS000893	EFO_0004611	low density lipoprotein cholesterol measurement	low density lipoprotein cholesterol measurement	EFO_0004611	Lipid or lipoprotein measurement	Metabolic	PGS003030	EFO_0004611	low density lipoprotein cholesterol measurement	measurement	EFO_0004611	Lipid or lipoprotein measurement	Metabolic
0.7206	2.23E-74	2.26E-71	PGS000893	EFO_0004611	low density lipoprotein cholesterol measurement	low density lipoprotein cholesterol measurement	EFO_0004611	Cardiovascular measurement	Cardiovascular	PGS003030	EFO_0004611	low density lipoprotein cholesterol measurement	measurement	EFO_0004611	Cardiovascular measurement	Cardiovascular
0.7206	2.23E-74	2.26E-71	PGS000893	EFO_0004611	low density lipoprotein cholesterol measurement	low density lipoprotein cholesterol measurement	EFO_0004611	Lipid or lipoprotein measurement	Metabolic	PGS003030	EFO_0004611	low density lipoprotein cholesterol measurement	measurement	EFO_0004611	Cardiovascular measurement	Cardiovascular
0.7206	2.23E-74	2.26E-71	PGS000893	EFO_0004611	low density lipoprotein cholesterol measurement	low density lipoprotein cholesterol measurement	EFO_0004611	Cardiovascular measurement	Cardiovascular	PGS003030	EFO_0004611	low density lipoprotein cholesterol measurement	measurement	EFO_0004611	Lipid or lipoprotein measurement	Metabolic
0.7205	2.31E-74	2.34E-71	PGS000189	EFO_0007986	reticulocyte count	reticulocyte count	EFO_0007986	Hematological measurement	Hematological	PGS000111	EFO_0007986	reticulocyte count	reticulocyte count	EFO_0007986	Hematological measurement	Hematological
0.7205	2.36E-74	2.39E-71	PGS002556	EFO_0004611	low density lipoprotein cholesterol measurement	low density lipoprotein cholesterol measurement	EFO_0004611	Cardiovascular measurement	Cardiovascular	PGS001933	EFO_0004611	low density lipoprotein cholesterol measurement	measurement	EFO_0004611	Cardiovascular measurement	Cardiovascular
0.7205	2.36E-74	2.39E-71	PGS002556	EFO_0004611	low density lipoprotein cholesterol measurement	low density lipoprotein cholesterol measurement	EFO_0004611	Lipid or lipoprotein measurement	Metabolic	PGS001933	EFO_0004611	low density lipoprotein cholesterol measurement	measurement	EFO_0004611	Cardiovascular measurement	Cardiovascular
0.7205	2.36E-74	2.39E-71	PGS002556	EFO_0004611	low density lipoprotein cholesterol measurement	low density lipoprotein cholesterol measurement	EFO_0004611	Lipid or lipoprotein measurement	Metabolic	PGS001933	EFO_0004611	low density lipoprotein cholesterol measurement	measurement	EFO_0004611	Lipid or lipoprotein measurement	Metabolic
0.7205	2.36E-74	2.39E-71	PGS002556	EFO_0004611	low density lipoprotein cholesterol measurement	low density lipoprotein cholesterol measurement	EFO_0004611	Cardiovascular measurement	Cardiovascular	PGS001933	EFO_0004611	low density lipoprotein cholesterol measurement	measurement	EFO_0004611	Lipid or lipoprotein measurement	Metabolic
0.7204	2.50E-74	2.53E-71	PGS000190	EFO_0007986	reticulocyte count	reticulocyte count	EFO_0007986	Hematological measurement	Hematological	PGS000112	EFO_0007986	reticulocyte count	reticulocyte count	EFO_0007986	Hematological measurement	Hematological
0.7204	2.51E-74	2.54E-71	PGS001940	EFO_0004735	serum alanine aminotransferase measurement	serum alanine aminotransferase measurement	EFO_0004735	Liver enzyme measurement	Gastrointestinal	PGS000668	EFO_0004735	serum alanine aminotransferase measurement	measurement	EFO_0004735	Liver enzyme measurement	Gastrointestinal
0.7204	2.60E-74	2.64E-71	PGS001895	EFO_0004574	total cholesterol measurement	total cholesterol measurement	EFO_0004574	Lipid or lipoprotein measurement	Metabolic	PGS002654	EFO_0004611	low density lipoprotein cholesterol measurement	measurement	EFO_0004611	Cardiovascular measurement	Cardiovascular
0.7204	2.60E-74	2.64E-71	PGS001895	EFO_0004574	total cholesterol measurement	total cholesterol measurement	EFO_0004574	Lipid or lipoprotein measurement	Metabolic	PGS002654	EFO_0004611	low density lipoprotein cholesterol measurement	measurement	EFO_0004611	Lipid or lipoprotein measurement	Metabolic
0.7204	2.62E-74	2.65E-71	PGS003194	EFO_0004631	vitamin D measurement	vitamin D measurement	EFO_0004631	Lipid or lipoprotein measurement	Metabolic	PGS001982	EFO_0004631	vitamin D measurement	vitamin D measurement	EFO_0004631	Lipid or lipoprotein measurement	Metabolic
0.7203	2.70E-74	2.74E-71	PGS000779	MONDO_0004975	Alzheimer disease	Alzheimer disease	MONDO_0004975	Neurological disorder	Neurological	PGS001349	MONDO_0004975	Alzheimer disease	Alzheimer disease	MONDO_0004975	Neurological disorder	Neurological
0.7203	2.73E-74	2.76E-71	PGS003184	EFO_0004631	vitamin D measurement	vitamin D measurement	EFO_0004631	Lipid or lipoprotein measurement	Metabolic	PGS003193	EFO_0004631	vitamin D measurement	vitamin D measurement	EFO_0004631	Lipid or lipoprotein measurement	Metabolic
0.7203	2.79E-74	2.82E-71	PGS001479	EFO_0006930	brain volume measurement|neuroimaging measurement	brain volume measurement	EFO_0006930	Other measurement	Neurological	PGS001672	EFO_0006930	measurement	brain volume measurement	EFO_0006930	Other measurement	Neurological
0.7203	2.82E-74	2.86E-71	PGS003228	EFO_0005208	glomerular filtration rate	glomerular filtration rate	EFO_0005208	Other measurement	Genitourinary	PGS003227	EFO_0005208	glomerular filtration rate	glomerular filtration rate	EFO_0005208	Other measurement	Genitourinary
0.7202	2.84E-74	2.87E-71	PGS000023	MONDO_0005147	type 1 diabetes mellitus	type 1 diabetes mellitus	MONDO_0005147	Metabolic disorder	Metabolic	PGS002025	MONDO_0005147	type 1 diabetes mellitus	type 1 diabetes mellitus	MONDO_0005147	Digestive system disorder	Gastrointestinal
0.7202	2.84E-74	2.87E-71	PGS000023	MONDO_0005147	type 1 diabetes mellitus	type 1 diabetes mellitus	MONDO_0005147	Metabolic disorder	Metabolic	PGS002025	MONDO_0005147	type 1 diabetes mellitus	type 1 diabetes mellitus	MONDO_0005147	Immune system disorder	Immunological
0.7202	2.84E-74	2.87E-71	PGS000023	MONDO_0005147	type 1 diabetes mellitus	type 1 diabetes mellitus	MONDO_0005147	Immune system disorder	Immunological	PGS002025	MONDO_0005147	type 1 diabetes mellitus	type 1 diabetes mellitus	MONDO_0005147	Immune system disorder	Immunological
0.7202	2.84E-74	2.87E-71	PGS000023	MONDO_0005147	type 1 diabetes mellitus	type 1 diabetes mellitus	MONDO_0005147	Digestive system disorder	Gastrointestinal	PGS002025	MONDO_0005147	type 1 diabetes mellitus	type 1 diabetes mellitus	MONDO_0005147	Metabolic disorder	Metabolic
0.7202	2.84E-74	2.87E-71	PGS000023	MONDO_0005147	type 1 diabetes mellitus	type 1 diabetes mellitus	MONDO_0005147	Immune system disorder	Immunological	PGS002025	MONDO_0005147	type 1 diabetes mellitus	type 1 diabetes mellitus	MONDO_0005147	Metabolic disorder	Metabolic
0.7202	2.84E-74	2.87E-71	PGS000023	MONDO_0005147	type 1 diabetes mellitus	type 1 diabetes mellitus	MONDO_0005147	Metabolic disorder	Metabolic	PGS002025	MONDO_0005147	type 1 diabetes mellitus	type 1 diabetes mellitus	MONDO_0005147	Metabolic disorder	Metabolic
0.7202	2.84E-74	2.87E-71	PGS000023	MONDO_0005147	type 1 diabetes mellitus	type 1 diabetes mellitus	MONDO_0005147	Immune system disorder	Immunological	PGS002025	MONDO_0005147	type 1 diabetes mellitus	type 1 diabetes mellitus	MONDO_0005147	Digestive system disorder	Gastrointestinal
0.7202	2.84E-74	2.87E-71	PGS000023	MONDO_0005147	type 1 diabetes mellitus	type 1 diabetes mellitus	MONDO_0005147	Digestive system disorder	Gastrointestinal	PGS002025	MONDO_0005147	type 1 diabetes mellitus	type 1 diabetes mellitus	MONDO_0005147	Digestive system disorder	Gastrointestinal
0.7202	2.84E-74	2.87E-71	PGS000023	MONDO_0005147	type 1 diabetes mellitus	type 1 diabetes mellitus	MONDO_0005147	Digestive system disorder	Gastrointestinal	PGS002025	MONDO_0005147	type 1 diabetes mellitus	type 1 diabetes mellitus	MONDO_0005147	Immune system disorder	Immunological
0.7202	2.84E-74	2.87E-71	PGS003199	EFO_0004631	vitamin D measurement	vitamin D measurement	EFO_0004631	Lipid or lipoprotein measurement	Metabolic	PGS003193	EFO_0004631	vitamin D measurement	vitamin D measurement	EFO_0004631	Lipid or lipoprotein measurement	Metabolic
0.7202	3.02E-74	3.05E-71	PGS002354	MONDO_0005148	type 2 diabetes mellitus	type 2 diabetes mellitus	MONDO_0005148	Digestive system disorder	Gastrointestinal	PGS002771	MONDO_0005148	type 2 diabetes mellitus	type 2 diabetes mellitus	MONDO_0005148	Digestive system disorder	Gastrointestinal
0.7202	3.02E-74	3.05E-71	PGS002354	MONDO_0005148	type 2 diabetes mellitus	type 2 diabetes mellitus	MONDO_0005148	Digestive system disorder	Gastrointestinal	PGS002771	MONDO_0005148	type 2 diabetes mellitus	type 2 diabetes mellitus	MONDO_0005148	Metabolic disorder	Metabolic
0.7202	3.02E-74	3.05E-71	PGS002354	MONDO_0005148	type 2 diabetes mellitus	type 2 diabetes mellitus	MONDO_0005148	Metabolic disorder	Metabolic	PGS002771	MONDO_0005148	type 2 diabetes mellitus	type 2 diabetes mellitus	MONDO_0005148	Digestive system disorder	Gastrointestinal
0.7202	3.02E-74	3.05E-71	PGS002354	MONDO_0005148	type 2 diabetes mellitus	type 2 diabetes mellitus	MONDO_0005148	Metabolic disorder	Metabolic	PGS002771	MONDO_0005148	type 2 diabetes mellitus	type 2 diabetes mellitus	MONDO_0005148	Metabolic disorder	Metabolic
0.7202	3.03E-74	3.06E-71	PGS001320	EFO_0000537	hypertension	hypertension	EFO_0000537	Cardiovascular disease	Cardiovascular	PGS000958	MONDO_0001134	essential hypertension	essential hypertension	MONDO_0001134	Cardiovascular disease	Cardiovascular
0.7201	3.19E-74	3.22E-71	PGS003136	EFO_0004574	total cholesterol measurement	total cholesterol measurement	EFO_0004574	Lipid or lipoprotein measurement	Metabolic	PGS002571	EFO_0004574	total cholesterol measurement	total cholesterol measurement	EFO_0004574	Lipid or lipoprotein measurement	Metabolic
-0.72	3.33E-74	3.36E-71	PGS003243	EFO_0010472	creatine measurement	creatine measurement	EFO_0010472	Other measurement	Metabolic	PGS000682	EFO_0005208	glomerular filtration rate	glomerular filtration rate	EFO_0005208	Other measurement	Genitourinary
0.72	3.36E-74	3.39E-71	PGS003135	EFO_0004574	total cholesterol measurement	total cholesterol measurement	EFO_0004574	Lipid or lipoprotein measurement	Metabolic	PGS003139	EFO_0004574	total cholesterol measurement	total cholesterol measurement	EFO_0004574	Lipid or lipoprotein measurement	Metabolic
0.72	3.47E-74	3.50E-71	PGS002979	EFO_0004339	body height	body height	EFO_0004339	Body measurement	Body measurement	PGS002990	EFO_0004339	body height	body height	EFO_0004339	Body measurement	Body measurement
0.7199	3.49E-74	3.52E-71	PGS002099	EFO_0004535	serum albumin measurement	serum albumin measurement	EFO_0004535	Other measurement	Metabolic	PGS000669	EFO_0004535	serum albumin measurement	serum albumin measurement	EFO_0004535	Other measurement	Metabolic
0.7199	3.60E-74	3.64E-71	PGS000065	EFO_0004611	low density lipoprotein cholesterol measurement	low density lipoprotein cholesterol measurement	EFO_0004611	Cardiovascular measurement	Cardiovascular	PGS000672	EFO_0004615	apolipoprotein B measurement	apolipoprotein B measurement	EFO_0004615	Lipid or lipoprotein measurement	Metabolic
0.7199	3.60E-74	3.64E-71	PGS000065	EFO_0004611	low density lipoprotein cholesterol measurement	low density lipoprotein cholesterol measurement	EFO_0004611	Lipid or lipoprotein measurement	Metabolic	PGS000672	EFO_0004615	apolipoprotein B measurement	apolipoprotein B measurement	EFO_0004615	Lipid or lipoprotein measurement	Metabolic
0.7199	3.63E-74	3.66E-71	PGS000005	EFO_1000649	estrogen-receptor positive breast cancer	estrogen-receptor positive breast cancer	EFO_1000649	Cancer	Neoplasms	PGS000505	EFO_0000305	breast carcinoma	breast carcinoma	EFO_0000305	Cancer	Neoplasms
0.7199	3.63E-74	3.66E-71	PGS000506	EFO_0000305	breast carcinoma	breast carcinoma	EFO_0000305	Cancer	Neoplasms	PGS000005	EFO_1000649	estrogen-receptor positive breast cancer	cancer	EFO_1000649	Cancer	Neoplasms
0.7199	3.64E-74	3.67E-71	PGS002854	EFO_0004340	body mass index	body mass index	EFO_0004340	Body measurement	Body measurement	PGS001951	EFO_0005409	fat body mass	fat body mass	EFO_0005409	Other measurement	Body measurement
0.7199	3.66E-74	3.68E-71	PGS003135	EFO_0004574	total cholesterol measurement	total cholesterol measurement	EFO_0004574	Lipid or lipoprotein measurement	Metabolic	PGS002424	EFO_0004574	total cholesterol measurement	total cholesterol measurement	EFO_0004574	Lipid or lipoprotein measurement	Metabolic
0.7199	3.68E-74	3.71E-71	PGS002573	MONDO_0005148	type 2 diabetes mellitus	type 2 diabetes mellitus	MONDO_0005148	Metabolic disorder	Metabolic	PGS002599	EFO_0004541	HbA1c measurement	HbA1c measurement	EFO_0004541	Hematological measurement	Hematological
0.7199	3.68E-74	3.71E-71	PGS002573	MONDO_0005148	type 2 diabetes mellitus	type 2 diabetes mellitus	MONDO_0005148	Digestive system disorder	Gastrointestinal	PGS002599	EFO_0004541	HbA1c measurement	HbA1c measurement	EFO_0004541	Hematological measurement	Hematological
0.7198	3.78E-74	3.81E-71	PGS001674	EFO_0006930	brain volume measurement|neuroimaging measurement	brain volume measurement	EFO_0006930	Other measurement	Neurological	PGS001684	EFO_0006930	measurement	brain volume measurement	EFO_0006930	Other measurement	Neurological
0.7198	3.83E-74	3.86E-71	PGS002200	EFO_0004631	vitamin D measurement	vitamin D measurement	EFO_0004631	Lipid or lipoprotein measurement	Metabolic	PGS003194	EFO_0004631	vitamin D measurement	vitamin D measurement	EFO_0004631	Lipid or lipoprotein measurement	Metabolic
0.7198	3.92E-74	3.95E-71	PGS000015	EFO_0000305	breast carcinoma	breast carcinoma	EFO_0000305	Cancer	Neoplasms	PGS000508	EFO_0000305	breast carcinoma	breast carcinoma	EFO_0000305	Cancer	Neoplasms
0.7197	4.07E-74	4.09E-71	PGS000532	EFO_0000305	breast carcinoma	breast carcinoma	EFO_0000305	Cancer	Neoplasms	PGS000479	EFO_0000305	breast carcinoma	breast carcinoma	EFO_0000305	Cancer	Neoplasms
0.7197	4.12E-74	4.15E-71	PGS000534	EFO_0000305	breast carcinoma	breast carcinoma	EFO_0000305	Cancer	Neoplasms	PGS000513	EFO_0000305	breast carcinoma	breast carcinoma	EFO_0000305	Cancer	Neoplasms
0.7197	4.12E-74	4.15E-71	PGS000533	EFO_0000305	breast carcinoma	breast carcinoma	EFO_0000305	Cancer	Neoplasms	PGS000513	EFO_0000305	breast carcinoma	breast carcinoma	EFO_0000305	Cancer	Neoplasms
0.7197	4.16E-74	4.19E-71	PGS000988	EFO_0010700	reticulocyte measurement	reticulocyte measurement	EFO_0010700	Hematological measurement	Hematological	PGS001959	EFO_0010700	reticulocyte measurement	reticulocyte measurement	EFO_0010700	Hematological measurement	Hematological
0.7197	4.21E-74	4.24E-71	PGS002764	HP_0003124	Hypercholesterolemia	Hypercholesterolemia	HP_0003124	Other trait	Metabolic	PGS002108	EFO_0004574	total cholesterol measurement	total cholesterol measurement	EFO_0004574	Lipid or lipoprotein measurement	Metabolic
-0.7197	4.24E-74	4.27E-71	PGS003248	EFO_0010472	creatine measurement	creatine measurement	EFO_0010472	Other measurement	Metabolic	PGS003223	EFO_0005208	glomerular filtration rate	glomerular filtration rate	EFO_0005208	Other measurement	Genitourinary
0.7196	4.27E-74	4.29E-71	PGS000008	EFO_1000649	estrogen-receptor positive breast cancer	estrogen-receptor positive breast cancer	EFO_1000649	Cancer	Neoplasms	PGS000873	EFO_0000305	breast carcinoma	breast carcinoma	EFO_0000305	Cancer	Neoplasms

0.7196	4.31E-74	4.33E-71	PGS001971	EFO_0004584	mean platelet volume	mean platelet volume	EFO_0004584	Hematological measurement	Hematological	PGS002461	EFO_0004584	mean platelet volume	mean platelet volume	EFO_0004584	Hematological measurement	Hematological
0.7196	4.34E-74	4.37E-71	PGS001671	EFO_0006930	brain volume measurement|neuroimaging measurement	brain volume measurement	EFO_0006930	Other measurement	Neurological	PGS001451	EFO_0006930	measurement	brain volume measurement	EFO_0006930	Other measurement	Neurological
0.7196	4.37E-74	4.39E-71	PGS002846	EFO_0004340	body mass index	body mass index	EFO_0004340	Body measurement	Body measurement	PGS002844	EFO_0004340	body mass index	body mass index	EFO_0004340	Body measurement	Body measurement
0.7196	4.39E-74	4.41E-71	PGS000532	EFO_0000305	breast carcinoma	breast carcinoma	EFO_0000305	Cancer	Neoplasms	PGS000506	EFO_0000305	breast carcinoma	breast carcinoma	EFO_0000305	Cancer	Neoplasms
0.7196	4.39E-74	4.41E-71	PGS000532	EFO_0000305	breast carcinoma	breast carcinoma	EFO_0000305	Cancer	Neoplasms	PGS000505	EFO_0000305	breast carcinoma	breast carcinoma	EFO_0000305	Cancer	Neoplasms
0.7196	4.45E-74	4.47E-71	PGS001531	EFO_0007772	calcaneal bone quantitative ultrasound measurement	calcaneal bone quantitative ultrasound measurement	EFO_0007772	Other measurement	Skeletal	PGS000951	EFO_0004514	bone quantitative ultrasound measurement	measurement	EFO_0004514	Other measurement	Skeletal
-0.7196	4.50E-74	4.51E-71	PGS002768	EFO_0003882	osteoporosis	osteoporosis	EFO_0003882	Other disease	Skeletal	PGS000954	EFO_0004514	bone quantitative ultrasound measurement	measurement	EFO_0004514	Other measurement	Skeletal
-0.7196	4.55E-74	4.57E-71	PGS002768	EFO_0003882	osteoporosis	osteoporosis	EFO_0003882	Other disease	Skeletal	PGS001038	EFO_0009270	heel bone mineral density	heel bone mineral density	EFO_0009270	Other measurement	Skeletal
0.7195	4.62E-74	4.63E-71	PGS002589	EFO_0000400	diabetes mellitus	diabetes mellitus	EFO_0000400	Metabolic disorder	Metabolic	PGS002599	EFO_0004541	HbA1c measurement	HbA1c measurement	EFO_0004541	Hematological measurement	Hematological
0.7195	4.62E-74	4.63E-71	PGS002589	EFO_0000400	diabetes mellitus	diabetes mellitus	EFO_0000400	Digestive system disorder	Gastrointestinal	PGS002599	EFO_0004541	HbA1c measurement	HbA1c measurement	EFO_0004541	Hematological measurement	Hematological
0.7195	4.69E-74	4.71E-71	PGS001805	EFO_0001663	prostate carcinoma	prostate carcinoma	EFO_0001663	Cancer	Neoplasms	PGS000049	EFO_0001663	prostate carcinoma	prostate carcinoma	EFO_0001663	Cancer	Neoplasms
0.7195	4.78E-74	4.79E-71	PGS002850	EFO_0004340	body mass index	body mass index	EFO_0004340	Body measurement	Body measurement	PGS000921	EFO_0004340	body mass index	body mass index	EFO_0004340	Body measurement	Body measurement
0.7195	4.85E-74	4.86E-71	PGS000532	EFO_0000305	breast carcinoma	breast carcinoma	EFO_0000305	Cancer	Neoplasms	PGS000540	EFO_0000305	breast carcinoma	breast carcinoma	EFO_0000305	Cancer	Neoplasms
0.7194	4.92E-74	4.94E-71	PGS001076	EFO_0007991	eosinophil percentage of leukocytes	eosinophil percentage of leukocytes	EFO_0007991	Hematological measurement	Hematological	PGS000091	EFO_0007991	eosinophil percentage of leukocytes	eosinophil percentage of leukocytes	EFO_0007991	Hematological measurement	Hematological
0.7194	5.10E-74	5.11E-71	PGS001488	EFO_0006930	brain volume measurement|neuroimaging measurement	brain volume measurement	EFO_0006930	Other measurement	Neurological	PGS001472	EFO_0006930	measurement	brain volume measurement	EFO_0006930	Other measurement	Neurological
0.7193	5.49E-74	5.50E-71	PGS001220	EFO_0004526	mean corpuscular volume	mean corpuscular volume	EFO_0004526	Hematological measurement	Hematological	PGS002656	EFO_0004527	mean corpuscular hemoglobin	mean corpuscular hemoglobin	EFO_0004527	Hematological measurement	Hematological
0.7193	5.56E-74	5.57E-71	PGS000532	EFO_0000305	breast carcinoma	breast carcinoma	EFO_0000305	Cancer	Neoplasms	PGS000002	EFO_1000649	estrogen-receptor positive breast cancer	cancer	EFO_1000649	Cancer	Neoplasms
0.7192	5.66E-74	5.67E-71	PGS003198	EFO_0004631	vitamin D measurement	vitamin D measurement	EFO_0004631	Lipid or lipoprotein measurement	Metabolic	PGS003195	EFO_0004631	vitamin D measurement	vitamin D measurement	EFO_0004631	Lipid or lipoprotein measurement	Metabolic
0.7192	5.68E-74	5.69E-71	PGS002556	EFO_0004611	low density lipoprotein cholesterol measurement	low density lipoprotein cholesterol measurement	EFO_0004611	Lipid or lipoprotein measurement	Metabolic	PGS003033	EFO_0004611	low density lipoprotein cholesterol measurement	measurement	EFO_0004611	Lipid or lipoprotein measurement	Metabolic
0.7192	5.68E-74	5.69E-71	PGS002556	EFO_0004611	low density lipoprotein cholesterol measurement	low density lipoprotein cholesterol measurement	EFO_0004611	Cardiovascular measurement	Cardiovascular	PGS003033	EFO_0004611	low density lipoprotein cholesterol measurement	measurement	EFO_0004611	Lipid or lipoprotein measurement	Metabolic
0.7192	5.68E-74	5.69E-71	PGS002556	EFO_0004611	low density lipoprotein cholesterol measurement	low density lipoprotein cholesterol measurement	EFO_0004611	Cardiovascular measurement	Cardiovascular	PGS003033	EFO_0004611	low density lipoprotein cholesterol measurement	measurement	EFO_0004611	Cardiovascular measurement	Cardiovascular
0.7192	5.68E-74	5.69E-71	PGS002556	EFO_0004611	low density lipoprotein cholesterol measurement	low density lipoprotein cholesterol measurement	EFO_0004611	Lipid or lipoprotein measurement	Metabolic	PGS003033	EFO_0004611	low density lipoprotein cholesterol measurement	measurement	EFO_0004611	Cardiovascular measurement	Cardiovascular
0.7192	5.72E-74	5.73E-71	PGS002142	EFO_0004509	hemoglobin measurement	hemoglobin measurement	EFO_0004509	Hematological measurement	Hematological	PGS000092	EFO_0004348	hematocrit	hematocrit	EFO_0004348	Hematological measurement	Hematological
0.7192	5.72E-74	5.73E-71	PGS003186	EFO_0004631	vitamin D measurement	vitamin D measurement	EFO_0004631	Lipid or lipoprotein measurement	Metabolic	PGS000702	EFO_0004631	vitamin D measurement	vitamin D measurement	EFO_0004631	Lipid or lipoprotein measurement	Metabolic
0.7192	5.73E-74	5.74E-71	PGS000539	EFO_0000305	breast carcinoma	breast carcinoma	EFO_0000305	Cancer	Neoplasms	PGS000508	EFO_0000305	breast carcinoma	breast carcinoma	EFO_0000305	Cancer	Neoplasms
0.7192	5.76E-74	5.76E-71	PGS002840	EFO_0004340	body mass index	body mass index	EFO_0004340	Body measurement	Body measurement	PGS000921	EFO_0004340	body mass index	body mass index	EFO_0004340	Body measurement	Body measurement
0.7191	6.18E-74	6.18E-71	PGS003140	EFO_0004574	total cholesterol measurement	total cholesterol measurement	EFO_0004574	Lipid or lipoprotein measurement	Metabolic	PGS003134	EFO_0004574	total cholesterol measurement	total cholesterol measurement	EFO_0004574	Lipid or lipoprotein measurement	Metabolic
0.7191	6.30E-74	6.31E-71	PGS000508	EFO_0000305	breast carcinoma	breast carcinoma	EFO_0000305	Cancer	Neoplasms	PGS000540	EFO_0000305	breast carcinoma	breast carcinoma	EFO_0000305	Cancer	Neoplasms
0.7191	6.35E-74	6.36E-71	PGS003035	EFO_0004611	low density lipoprotein cholesterol measurement	low density lipoprotein cholesterol measurement	EFO_0004611	Lipid or lipoprotein measurement	Metabolic	PGS002620	EFO_0004574	total cholesterol measurement	total cholesterol measurement	EFO_0004574	Lipid or lipoprotein measurement	Metabolic
0.7191	6.35E-74	6.36E-71	PGS003035	EFO_0004611	low density lipoprotein cholesterol measurement	low density lipoprotein cholesterol measurement	EFO_0004611	Cardiovascular measurement	Cardiovascular	PGS002620	EFO_0004574	total cholesterol measurement	total cholesterol measurement	EFO_0004574	Lipid or lipoprotein measurement	Metabolic
0.7191	6.38E-74	6.38E-71	PGS002009	EFO_0006335	systolic blood pressure	systolic blood pressure	EFO_0006335	Other measurement	Cardiovascular	PGS002765	EFO_0000537	hypertension	hypertension	EFO_0000537	Cardiovascular disease	Cardiovascular
-0.719	6.44E-74	6.44E-71	PGS003248	EFO_0010472	creatine measurement	creatine measurement	EFO_0010472	Other measurement	Metabolic	PGS002757	EFO_0003884	chronic kidney disease	chronic kidney disease	EFO_0003884	Other disease	Metabolic
0.719	6.53E-74	6.53E-71	PGS002108	EFO_0004574	total cholesterol measurement	total cholesterol measurement	EFO_0004574	Lipid or lipoprotein measurement	Metabolic	PGS002654	EFO_0004611	low density lipoprotein cholesterol measurement	measurement	EFO_0004611	Cardiovascular measurement	Cardiovascular
0.719	6.53E-74	6.53E-71	PGS002108	EFO_0004574	total cholesterol measurement	total cholesterol measurement	EFO_0004574	Lipid or lipoprotein measurement	Metabolic	PGS002654	EFO_0004611	low density lipoprotein cholesterol measurement	measurement	EFO_0004611	Lipid or lipoprotein measurement	Metabolic
0.719	6.54E-74	6.54E-71	PGS003147	EFO_0004530	triglyceride measurement	triglyceride measurement	EFO_0004530	Lipid or lipoprotein measurement	Metabolic	PGS003145	EFO_0004530	triglyceride measurement	triglyceride measurement	EFO_0004530	Lipid or lipoprotein measurement	Metabolic
0.719	6.75E-74	6.75E-71	PGS002605	EFO_0004611	low density lipoprotein cholesterol measurement	low density lipoprotein cholesterol measurement	EFO_0004611	Cardiovascular measurement	Cardiovascular	PGS002654	EFO_0004611	low density lipoprotein cholesterol measurement	measurement	EFO_0004611	Lipid or lipoprotein measurement	Metabolic
0.719	6.75E-74	6.75E-71	PGS002605	EFO_0004611	low density lipoprotein cholesterol measurement	low density lipoprotein cholesterol measurement	EFO_0004611	Lipid or lipoprotein measurement	Metabolic	PGS002654	EFO_0004611	low density lipoprotein cholesterol measurement	measurement	EFO_0004611	Cardiovascular measurement	Cardiovascular
0.719	6.75E-74	6.75E-71	PGS002605	EFO_0004611	low density lipoprotein cholesterol measurement	low density lipoprotein cholesterol measurement	EFO_0004611	Lipid or lipoprotein measurement	Metabolic	PGS002654	EFO_0004611	low density lipoprotein cholesterol measurement	measurement	EFO_0004611	Lipid or lipoprotein measurement	Metabolic
0.719	6.75E-74	6.75E-71	PGS002605	EFO_0004611	low density lipoprotein cholesterol measurement	low density lipoprotein cholesterol measurement	EFO_0004611	Cardiovascular measurement	Cardiovascular	PGS002654	EFO_0004611	low density lipoprotein cholesterol measurement	measurement	EFO_0004611	Cardiovascular measurement	Cardiovascular
0.7189	7.07E-74	7.07E-71	PGS002608	EFO_0004584	mean platelet volume	mean platelet volume	EFO_0004584	Hematological measurement	Hematological	PGS002189	EFO_0004584	mean platelet volume	mean platelet volume	EFO_0004584	Hematological measurement	Hematological
0.7189	7.08E-74	7.07E-71	PGS001991	EFO_0007989	monocyte percentage of leukocytes	monocyte percentage of leukocytes	EFO_0007989	Hematological measurement	Hematological	PGS002341	EFO_0005091	monocyte count	monocyte count	EFO_0005091	Hematological measurement	Hematological
-0.7189	7.19E-74	7.18E-71	PGS003228	EFO_0005208	glomerular filtration rate	glomerular filtration rate	EFO_0005208	Other measurement	Genitourinary	PGS003253	EFO_0010472	creatine measurement|glomerular filtration rate	creatine measurement	EFO_0010472	Other measurement	Metabolic
0.7189	7.19E-74	7.18E-71	PGS001037	EFO_0009270	heel bone mineral density	heel bone mineral density	EFO_0009270	Other measurement	Skeletal	PGS000953	EFO_0004514	bone quantitative ultrasound measurement	measurement	EFO_0004514	Other measurement	Skeletal
0.7188	7.36E-74	7.35E-71	PGS000952	EFO_0004514	bone quantitative ultrasound measurement	bone quantitative ultrasound measurement	EFO_0004514	Other measurement	Skeletal	PGS000953	EFO_0004514	bone quantitative ultrasound measurement	measurement	EFO_0004514	Other measurement	Skeletal
0.7188	7.47E-74	7.45E-71	PGS002846	EFO_0004340	body mass index	body mass index	EFO_0004340	Body measurement	Body measurement	PGS002855	EFO_0004340	body mass index	body mass index	EFO_0004340	Body measurement	Body measurement
0.7188	7.49E-74	7.48E-71	PGS001488	EFO_0006930	brain volume measurement|neuroimaging measurement	brain volume measurement	EFO_0006930	Other measurement	Neurological	PGS001670	EFO_0006930	measurement	brain volume measurement	EFO_0006930	Other measurement	Neurological
0.7188	7.70E-74	7.69E-71	PGS000511	EFO_0000305	breast carcinoma	breast carcinoma	EFO_0000305	Cancer	Neoplasms	PGS000015	EFO_0000305	breast carcinoma	breast carcinoma	EFO_0000305	Cancer	Neoplasms
0.7187	8.30E-74	8.28E-71	PGS003136	EFO_0004574	total cholesterol measurement	total cholesterol measurement	EFO_0004574	Lipid or lipoprotein measurement	Metabolic	PGS002424	EFO_0004574	total cholesterol measurement	total cholesterol measurement	EFO_0004574	Lipid or lipoprotein measurement	Metabolic
0.7186	8.51E-74	8.48E-71	PGS000532	EFO_0000305	breast carcinoma	breast carcinoma	EFO_0000305	Cancer	Neoplasms	PGS000538	EFO_0000305	breast carcinoma	breast carcinoma	EFO_0000305	Cancer	Neoplasms
0.7186	8.56E-74	8.53E-71	PGS002409	EFO_0004611	low density lipoprotein cholesterol measurement	low density lipoprotein cholesterol measurement	EFO_0004611	Lipid or lipoprotein measurement	Metabolic	PGS000893	EFO_0004611	low density lipoprotein cholesterol measurement	measurement	EFO_0004611	Cardiovascular measurement	Cardiovascular
0.7186	8.56E-74	8.53E-71	PGS002409	EFO_0004611	low density lipoprotein cholesterol measurement	low density lipoprotein cholesterol measurement	EFO_0004611	Cardiovascular measurement	Cardiovascular	PGS000893	EFO_0004611	low density lipoprotein cholesterol measurement	measurement	EFO_0004611	Cardiovascular measurement	Cardiovascular
0.7186	8.56E-74	8.53E-71	PGS002427	EFO_0004631	vitamin D measurement	vitamin D measurement	EFO_0004631	Lipid or lipoprotein measurement	Metabolic	PGS002355	EFO_0004631	vitamin D measurement	vitamin D measurement	EFO_0004631	Lipid or lipoprotein measurement	Metabolic
0.7186	8.56E-74	8.53E-71	PGS002409	EFO_0004611	low density lipoprotein cholesterol measurement	low density lipoprotein cholesterol measurement	EFO_0004611	Lipid or lipoprotein measurement	Metabolic	PGS000893	EFO_0004611	low density lipoprotein cholesterol measurement	measurement	EFO_0004611	Lipid or lipoprotein measurement	Metabolic
0.7186	8.56E-74	8.53E-71	PGS002409	EFO_0004611	low density lipoprotein cholesterol measurement	low density lipoprotein cholesterol measurement	EFO_0004611	Cardiovascular measurement	Cardiovascular	PGS000893	EFO_0004611	low density lipoprotein cholesterol measurement	measurement	EFO_0004611	Lipid or lipoprotein measurement	Metabolic
0.7186	8.64E-74	8.61E-71	PGS003198	EFO_0004631	vitamin D measurement	vitamin D measurement	EFO_0004631	Lipid or lipoprotein measurement	Metabolic	PGS003196	EFO_0004631	vitamin D measurement	vitamin D measurement	EFO_0004631	Lipid or lipoprotein measurement	Metabolic
0.7185	9.44E-74	9.41E-71	PGS003029	EFO_0004611	low density lipoprotein cholesterol measurement	low density lipoprotein cholesterol measurement	EFO_0004611	Lipid or lipoprotein measurement	Metabolic	PGS000892	EFO_0004611	low density lipoprotein cholesterol measurement	measurement	EFO_0004611	Lipid or lipoprotein measurement	Metabolic
0.7185	9.44E-74	9.41E-71	PGS003029	EFO_0004611	low density lipoprotein cholesterol measurement	low density lipoprotein cholesterol measurement	EFO_0004611	Cardiovascular measurement	Cardiovascular	PGS000892	EFO_0004611	low density lipoprotein cholesterol measurement	measurement	EFO_0004611	Lipid or lipoprotein measurement	Metabolic
0.7185	9.44E-74	9.41E-71	PGS003029	EFO_0004611	low density lipoprotein cholesterol measurement	low density lipoprotein cholesterol measurement	EFO_0004611	Lipid or lipoprotein measurement	Metabolic	PGS000892	EFO_0004611	low density lipoprotein cholesterol measurement	measurement	EFO_0004611	Cardiovascular measurement	Cardiovascular
0.7185	9.44E-74	9.41E-71	PGS003029	EFO_0004611	low density lipoprotein cholesterol measurement	low density lipoprotein cholesterol measurement	EFO_0004611	Cardiovascular measurement	Cardiovascular	PGS000892	EFO_0004611	low density lipoprotein cholesterol measurement	measurement	EFO_0004611	Cardiovascular measurement	Cardiovascular
0.7185	9.55E-74	9.52E-71	PGS000596	EFO_0005088	testicular carcinoma	testicular carcinoma	EFO_0005088	Cancer	Neoplasms	PGS001806	EFO_0004281	testicular neoplasm	testicular neoplasm	EFO_0004281	Other disease	Neoplasms
0.7184	9.80E-74	9.76E-71	PGS003248	EFO_0010472	creatine measurement	creatine measurement	EFO_0010472	Other measurement	Metabolic	PGS003245	EFO_0010472	creatine measurement	creatine measurement	EFO_0010472	Other measurement	Metabolic
0.7184	1.01E-73	1.01E-70	PGS002242	EFO_0000305	breast carcinoma	breast carcinoma	EFO_0000305	Cancer	Neoplasms	PGS000507	EFO_0000305	breast carcinoma	breast carcinoma	EFO_0000305	Cancer	Neoplasms
-0.7184	1.01E-73	1.01E-70	PGS002768	EFO_0003882	osteoporosis	osteoporosis	EFO_0003882	Other disease	Skeletal	PGS001530	EFO_0007772	measurement	measurement	EFO_0007772	Other measurement	Skeletal
0.7184	1.01E-73	1.01E-70	PGS001860	EFO_0001421	liver disease	liver disease	EFO_0001421	Digestive system disorder	Gastrointestinal	PGS000776	EFO_0001422	cirrhosis of liver	cirrhosis of liver	EFO_0001422	Digestive system disorder	Gastrointestinal
0.7184	1.01E-73	1.01E-70	PGS001860	EFO_0001421	liver disease	liver disease	EFO_0001421	Digestive system disorder	Gastrointestinal	PGS000776	EFO_0001422	cirrhosis of liver	cirrhosis of liver	EFO_0001422	Cancer	Neoplasms
0.7184	1.01E-73	1.01E-70	PGS003146	EFO_0004530	triglyceride measurement	triglyceride measurement	EFO_0004530	Lipid or lipoprotein measurement	Metabolic	PGS003144	EFO_0004530	triglyceride measurement	triglyceride measurement	EFO_0004530	Lipid or lipoprotein measurement	Metabolic
0.7184	1.02E-73	1.02E-70	PGS002840	EFO_0004340	body mass index	body mass index	EFO_0004340	Body measurement	Body measurement	PGS002630	EFO_0004340	body mass index	body mass index	EFO_0004340	Body measurement	Body measurement
0.7183	1.08E-73	1.07E-70	PGS000297	EFO_0004339	body height	body height	EFO_0004339	Body measurement	Body measurement	PGS002332	EFO_0004339	body height	body height	EFO_0004339	Body measurement	Body measurement
0.7183	1.09E-73	1.08E-70	PGS002605	EFO_0004611	low density lipoprotein cholesterol measurement	low density lipoprotein cholesterol measurement	EFO_0004611	Lipid or lipoprotein measurement	Metabolic	PGS000888	EFO_0004611	low density lipoprotein cholesterol measurement	measurement	EFO_0004611	Cardiovascular measurement	Cardiovascular
0.7183	1.09E-73	1.08E-70	PGS002605	EFO_0004611	low density lipoprotein cholesterol measurement	low density lipoprotein cholesterol measurement	EFO_0004611	Cardiovascular measurement	Cardiovascular	PGS000888	EFO_0004611	low density lipoprotein cholesterol measurement	measurement	EFO_0004611	Lipid or lipoprotein measurement	Metabolic
0.7183	1.09E-73	1.08E-70	PGS002605	EFO_0004611	low density lipoprotein cholesterol measurement	low density lipoprotein cholesterol measurement	EFO_0004611	Cardiovascular measurement	Cardiovascular	PGS000888	EFO_0004611	low density lipoprotein cholesterol measurement	measurement	EFO_0004611	Cardiovascular measurement	Cardiovascular
0.7183	1.09E-73	1.08E-70	PGS002605	EFO_0004611	low density lipoprotein cholesterol measurement	low density lipoprotein cholesterol measurement	EFO_0004611	Lipid or lipoprotein measurement	Metabolic	PGS000888	EFO_0004611	low density lipoprotein cholesterol measurement	measurement	EFO_0004611	Lipid or lipoprotein measurement	Metabolic
0.7183	1.10E-73	1.09E-70	PGS003256	EFO_0010472	creatine measurement|glomerular filtration rate	creatine measurement	EFO_0010472	Other measurement	Metabolic	PGS003270	EFO_0010472	creatine measurement|glomerular filtration rate	creatine measurement	EFO_0010472	Other measurement	Metabolic
0.7182	1.13E-73	1.12E-70	PGS001917	EFO_0007800	body fat percentage	body fat percentage	EFO_0007800	Body measurement	Body measurement	PGS001101	EFO_0007800	body fat percentage	body fat percentage	EFO_0007800	Body measurement	Body measurement
0.7182	1.16E-73	1.16E-70	PGS002764	HP_0003124	Hypercholesterolemia	Hypercholesterolemia	HP_0003124	Other trait	Metabolic	PGS002102	EFO_0004615	apolipoprotein B measurement	apolipoprotein B measurement	EFO_0004615	Lipid or lipoprotein measurement	Metabolic
0.7181	1.22E-73	1.22E-70	PGS000824	EFO_0004611	low density lipoprotein cholesterol measurement	low density lipoprotein cholesterol measurement	EFO_0004611	Lipid or lipoprotein measurement	Metabolic	PGS003033	EFO_0004611	low density lipoprotein cholesterol measurement	measurement	EFO_0004611	Lipid or lipoprotein measurement	Metabolic
0.7181	1.22E-73	1.22E-70	PGS000824	EFO_0004611	low density lipoprotein cholesterol measurement	low density lipoprotein cholesterol measurement	EFO_0004611	Cardiovascular measurement	Cardiovascular	PGS003033	EFO_0004611	low density lipoprotein cholesterol measurement	measurement	EFO_0004611	Cardiovascular measurement	Cardiovascular
0.7181	1.22E-73	1.22E-70	PGS000824	EFO_0004611	low density lipoprotein cholesterol measurement	low density lipoprotein cholesterol measurement	EFO_0004611	Cardiovascular measurement	Cardiovascular	PGS003033	EFO_0004611	low density lipoprotein cholesterol measurement	measurement	EFO_0004611	Lipid or lipoprotein measurement	Metabolic
0.7181	1.22E-73	1.22E-70	PGS000824	EFO_0004611	low density lipoprotein cholesterol measurement	low density lipoprotein cholesterol measurement	EFO_0004611	Lipid or lipoprotein measurement	Metabolic	PGS003033	EFO_0004611	low density lipoprotein cholesterol measurement	measurement	EFO_0004611	Cardiovascular measurement	Cardiovascular
0.7181	1.26E-73	1.25E-70	PGS001908	EFO_0005192	red blood cell distribution width	red blood cell distribution width	EFO_0005192	Hematological measurement	Hematological	PGS002614	EFO_0005192	red blood cell distribution width	red blood cell distribution width	EFO_0005192	Hematological measurement	Hematological
0.718	1.28E-73	1.27E-70	PGS000539	EFO_0000305	breast carcinoma	breast carcinoma	EFO_0000305	Cancer	Neoplasms	PGS000335	EFO_0000305	breast carcinoma	breast carcinoma	EFO_0000305	Cancer	Neoplasms
0.718	1.29E-73	1.28E-70	PGS002179	EFO_0005106	body composition measurement	body composition measurement	EFO_0005106	Body measurement	Body measurement	PGS001156	EFO_0005106	body composition measurement	body composition measurement	EFO_0005106	Body measurement	Body measurement
0.718	1.32E-73	1.31E-70	PGS000400	EFO_0009259	skin carcinoma	skin carcinoma	EFO_0009259	Cancer	Neoplasms	PGS000435	MONDO_0002898	skin cancer	skin cancer	MONDO_0002898	Cancer	Neoplasms
0.718	1.34E-73	1.33E-70	PGS002244	EFO_0001645	coronary artery disease	coronary artery disease	EFO_0001645	Cardiovascular disease	Cardiovascular	PGS001780	EFO_0001645	coronary artery disease	coronary artery disease	EFO_0001645	Cardiovascular disease	Cardiovascular
0.718	1.34E-73	1.33E-70	PGS002869	EFO_0004458	C-reactive protein measurement	C-reactive protein measurement	EFO_0004458	Inflammatory measurement	Immunological	PGS002870	EFO_0004458	C-reactive protein measurement	C-reactive protein measurement	EFO_0004458	Inflammatory measurement	Immunological
0.7179	1.35E-73	1.34E-70	PGS000893	EFO_0004611	low density lipoprotein cholesterol measurement	low density lipoprotein cholesterol measurement	EFO_0004611	Lipid or lipoprotein measurement	Metabolic	PGS002654	EFO_0004611	low density lipoprotein cholesterol measurement	measurement	EFO_0004611	Cardiovascular measurement	Cardiovascular
0.7179	1.35E-73	1.34E-70	PGS000893	EFO_0004611	low density lipoprotein cholesterol measurement	low density lipoprotein cholesterol measurement	EFO_0004611	Cardiovascular measurement	Cardiovascular	PGS002654	EFO_0004611	low density lipoprotein cholesterol measurement	measurement	EFO_0004611	Cardiovascular measurement	Cardiovascular
0.7179	1.35E-73	1.34E-70	PGS000893	EFO_0004611	low density lipoprotein cholesterol measurement	low density lipoprotein cholesterol measurement	EFO_0004611	Lipid or lipoprotein measurement	Metabolic	PGS002654	EFO_0004611	low density lipoprotein cholesterol measurement	measurement	EFO_0004611	Lipid or lipoprotein measurement	Metabolic
0.7179	1.35E-73	1.34E-70	PGS000893	EFO_0004611	low density lipoprotein cholesterol measurement	low density lipoprotein cholesterol measurement	EFO_0004611	Cardiovascular measurement	Cardiovascular	PGS002654	EFO_0004611	low density lipoprotein cholesterol measurement	measurement	EFO_0004611	Lipid or lipoprotein measurement	Metabolic
0.7179	1.38E-73	1.37E-70	PGS003123	MONDO_0005148	type 2 diabetes mellitus	type 2 diabetes mellitus	MONDO_0005148	Digestive system disorder	Gastrointestinal	PGS002780	MONDO_0005148	type 2 diabetes mellitus	type 2 diabetes mellitus	MONDO_0005148	Metabolic disorder	Metabolic
0.7179	1.38E-73	1.37E-70	PGS003123	MONDO_0005148	type 2 diabetes mellitus	type 2 diabetes mellitus	MONDO_0005148	Digestive system disorder	Gastrointestinal	PGS002780	MONDO_0005148	type 2 diabetes mellitus	type 2 diabetes mellitus	MONDO_0005148	Digestive system disorder	Gastrointestinal
0.7179	1.38E-73	1.37E-70	PGS003123	MONDO_0005148	type 2 diabetes mellitus	type 2 diabetes mellitus	MONDO_0005148	Metabolic disorder	Metabolic	PGS002780	MONDO_0005148	type 2 diabetes mellitus	type 2 diabetes mellitus	MONDO_0005148	Metabolic disorder	Metabolic
0.7179	1.38E-73	1.37E-70	PGS003123	MONDO_0005148	type 2 diabetes mellitus	type 2 diabetes mellitus	MONDO_0005148	Metabolic disorder	Metabolic	PGS002780	MONDO_0005148	type 2 diabetes mellitus	type 2 diabetes mellitus	MONDO_0005148	Digestive system disorder	Gastrointestinal
0.7179	1.40E-73	1.39E-70	PGS000888	EFO_0004611	low density lipoprotein cholesterol measurement	low density lipoprotein cholesterol measurement	EFO_0004611	Lipid or lipoprotein measurement	Metabolic	PGS001889	EFO_0004615	apolipoprotein B measurement	apolipoprotein B measurement	EFO_0004615	Lipid or lipoprotein measurement	Metabolic
0.7179	1.40E-73	1.39E-70	PGS000888	EFO_0004611	low density lipoprotein cholesterol measurement	low density lipoprotein cholesterol measurement	EFO_0004611	Cardiovascular measurement	Cardiovascular	PGS001889	EFO_0004615	apolipoprotein B measurement	apolipoprotein B measurement	EFO_0004615	Lipid or lipoprotein measurement	Metabolic
0.7178	1.44E-73	1.43E-70	PGS002242	EFO_0000305	breast carcinoma	breast carcinoma	EFO_0000305	Cancer	Neoplasms	PGS000508	EFO_0000305	breast carcinoma	breast carcinoma	EFO_0000305	Cancer	Neoplasms
0.7178	1.45E-73	1.44E-70	PGS000688	EFO_0004611	low density lipoprotein cholesterol measurement	low density lipoprotein cholesterol measurement	EFO_0004611	Lipid or lipoprotein measurement	Metabolic	PGS001895	EFO_0004574	total cholesterol measurement	total cholesterol measurement	EFO_0004574	Lipid or lipoprotein measurement	Metabolic
0.7178	1.45E-73	1.44E-70	PGS000688	EFO_0004611	low density lipoprotein cholesterol measurement	low density lipoprotein cholesterol measurement	EFO_0004611	Cardiovascular measurement	Cardiovascular	PGS001895	EFO_0004574	total cholesterol measurement	total cholesterol measurement	EFO_0004574	Lipid or lipoprotein measurement	Metabolic
0.7178	1.50E-73	1.49E-70	PGS003201	EFO_0004631	vitamin D measurement	vitamin D measurement	EFO_0004631	Lipid or lipoprotein measurement	Metabolic	PGS002623	EFO_0004631	vitamin D measurement	vitamin D measurement	EFO_0004631	Lipid or lipoprotein measurement	Metabolic
0.7178	1.51E-73	1.49E-70	PGS003201	EFO_0004631	vitamin D measurement	vitamin D measurement	EFO_0004631	Lipid or lipoprotein measurement	Metabolic	PGS000702	EFO_0004631	vitamin D measurement	vitamin D measurement	EFO_0004631	Lipid or lipoprotein measurement	Metabolic
0.7178	1.51E-73	1.49E-70	PGS000001	EFO_0000305	breast carcinoma	breast carcinoma	EFO_0000305	Cancer	Neoplasms	PGS000028	EFO_0000305	breast carcinoma	breast carcinoma	EFO_0000305	Cancer	Neoplasms
-0.7177	1.55E-73	1.54E-70	PGS002768	EFO_0003882	osteoporosis	osteoporosis	EFO_0003882	Other disease	Skeletal	PGS001404	EFO_0009270	heel bone mineral density	heel bone mineral density	EFO_0009270	Other measurement	Skeletal
0.7177	1.60E-73	1.59E-70	PGS001402	EFO_0009270	heel bone mineral density	heel bone mineral density	EFO_0009270	Other measurement	Skeletal	PGS000953	EFO_0004514	bone quantitative ultrasound measurement	measurement	EFO_0004514	Other measurement	Skeletal

0.7177	1.65E-73	1.63E-70	PGS003144	EFO_0004530	triglyceride measurement	triglyceride measurement	EFO_0004530	Lipid or lipoprotein measurement	Metabolic	PGS003151	EFO_0004530	triglyceride measurement	triglyceride measurement	EFO_0004530	Lipid or lipoprotein measurement	Metabolic
0.7176	1.69E-73	1.68E-70	PGS002785	MONDO_0005090	schizophrenia	schizophrenia	MONDO_0005090	Neurological disorder	Neurological	PGS000135	MONDO_0005090	schizophrenia	schizophrenia	MONDO_0005090	Neurological disorder	Neurological
0.7176	1.72E-73	1.70E-70	PGS000474	EFO_0000305	breast carcinoma	breast carcinoma	EFO_0000305	Cancer	Neoplasms	PGS000497	EFO_0000305	breast carcinoma	breast carcinoma	EFO_0000305	Cancer	Neoplasms
0.7176	1.72E-73	1.70E-70	PGS000474	EFO_0000305	breast carcinoma	breast carcinoma	EFO_0000305	Cancer	Neoplasms	PGS000498	EFO_0000305	breast carcinoma	breast carcinoma	EFO_0000305	Cancer	Neoplasms
0.7176	1.74E-73	1.72E-70	PGS003134	EFO_0004574	total cholesterol measurement	total cholesterol measurement	EFO_0004574	Lipid or lipoprotein measurement	Metabolic	PGS002352	EFO_0004574	total cholesterol measurement	total cholesterol measurement	EFO_0004574	Lipid or lipoprotein measurement	Metabolic
0.7176	1.76E-73	1.74E-70	PGS000296	EFO_0001645	coronary artery disease	coronary artery disease	EFO_0001645	Cardiovascular disease	Cardiovascular	PGS002776	EFO_0001645	coronary artery disease	coronary artery disease	EFO_0001645	Cardiovascular disease	Cardiovascular
0.7175	1.78E-73	1.76E-70	PGS003146	EFO_0004530	triglyceride measurement	triglyceride measurement	EFO_0004530	Lipid or lipoprotein measurement	Metabolic	PGS003148	EFO_0004530	triglyceride measurement	triglyceride measurement	EFO_0004530	Lipid or lipoprotein measurement	Metabolic
0.7175	1.78E-73	1.76E-70	PGS000215	EFO_1000294	HER2 Positive Breast Carcinoma	HER2 Positive Breast Carcinoma	EFO_1000294	Cancer	Neoplasms	PGS000006	EFO_1000650	estrogen-receptor negative breast cancer	cancer	EFO_1000650	Cancer	Neoplasms
0.7175	1.79E-73	1.77E-70	PGS001918	EFO_0004314	forced expiratory volume	forced expiratory volume	EFO_0004314	Other measurement	Respiratory	PGS001236	EFO_0004314	forced expiratory volume	forced expiratory volume	EFO_0004314	Other measurement	Respiratory
-0.7175	1.79E-73	1.77E-70	PGS002768	EFO_0003882	osteoporosis	osteoporosis	EFO_0003882	Other disease	Skeletal	PGS002387	EFO_0003923	bone density	bone density	EFO_0003923	Other measurement	Skeletal
0.7175	1.81E-73	1.79E-70	PGS001929	EFO_0004339	body height	body height	EFO_0004339	Body measurement	Body measurement	PGS000297	EFO_0004339	body height	body height	EFO_0004339	Body measurement	Body measurement
0.7175	1.81E-73	1.79E-70	PGS002764	HP_0003124	Hypercholesterolemia	Hypercholesterolemia	HP_0003124	Other trait	Metabolic	PGS001895	EFO_0004574	total cholesterol measurement	total cholesterol measurement	EFO_0004574	Lipid or lipoprotein measurement	Metabolic
0.7173	2.04E-73	2.01E-70	PGS001492	EFO_0006930	brain volume measurement|neuroimaging measurement	brain volume measurement	EFO_0006930	Other measurement	Neurological	PGS001491	EFO_0006930	measurement	brain volume measurement	EFO_0006930	Other measurement	Neurological
0.7173	2.06E-73	2.04E-70	PGS002869	EFO_0004458	C-reactive protein measurement	C-reactive protein measurement	EFO_0004458	Inflammatory measurement	Immunological	PGS002164	EFO_0004458	C-reactive protein measurement	C-reactive protein measurement	EFO_0004458	Inflammatory measurement	Immunological
0.7173	2.07E-73	2.04E-70	PGS001687	EFO_0006930	brain volume measurement|neuroimaging measurement	brain volume measurement	EFO_0006930	Other measurement	Neurological	PGS001672	EFO_0006930	measurement	brain volume measurement	EFO_0006930	Other measurement	Neurological
0.7173	2.08E-73	2.05E-70	PGS000160	EFO_0001663	prostate carcinoma	prostate carcinoma	EFO_0001663	Cancer	Neoplasms	PGS000030	EFO_0001663	prostate carcinoma	prostate carcinoma	EFO_0001663	Cancer	Neoplasms
0.7173	2.10E-73	2.07E-70	PGS002150	EFO_0004611	low density lipoprotein cholesterol measurement	low density lipoprotein cholesterol measurement	EFO_0004611	Cardiovascular measurement	Cardiovascular	PGS002782	EFO_0005689	measurement	measurement	EFO_0005689	Lipid or lipoprotein measurement	Metabolic
0.7173	2.10E-73	2.07E-70	PGS002150	EFO_0004611	low density lipoprotein cholesterol measurement	low density lipoprotein cholesterol measurement	EFO_0004611	Lipid or lipoprotein measurement	Metabolic	PGS002782	EFO_0005689	measurement	measurement	EFO_0005689	Lipid or lipoprotein measurement	Metabolic
0.7173	2.10E-73	2.07E-70	PGS002150	EFO_0004611	low density lipoprotein cholesterol measurement	low density lipoprotein cholesterol measurement	EFO_0004611	Lipid or lipoprotein measurement	Metabolic	PGS002782	EFO_0005689	measurement	measurement	EFO_0005689	Cardiovascular measurement	Cardiovascular
0.7173	2.10E-73	2.07E-70	PGS002150	EFO_0004611	low density lipoprotein cholesterol measurement	low density lipoprotein cholesterol measurement	EFO_0004611	Cardiovascular measurement	Cardiovascular	PGS002782	EFO_0005689	measurement	measurement	EFO_0005689	Cardiovascular measurement	Cardiovascular
0.7173	2.11E-73	2.08E-70	PGS003140	EFO_0004574	total cholesterol measurement	total cholesterol measurement	EFO_0004574	Lipid or lipoprotein measurement	Metabolic	PGS002571	EFO_0004574	total cholesterol measurement	total cholesterol measurement	EFO_0004574	Lipid or lipoprotein measurement	Metabolic
0.7173	2.12E-73	2.09E-70	PGS002856	EFO_0004340	body mass index	body mass index	EFO_0004340	Body measurement	Body measurement	PGS002844	EFO_0004340	body mass index	body mass index	EFO_0004340	Body measurement	Body measurement
-0.7172	2.19E-73	2.16E-70	PGS003295	EFO_0004617	cystatin C measurement|glomerular filtration rate	cystatin C measurement	EFO_0004617	Other measurement	Metabolic	PGS003304	EFO_0004617	cystatin C measurement|glomerular filtration rate	cystatin C measurement	EFO_0004617	Other measurement	Metabolic
0.7172	2.21E-73	2.18E-70	PGS000417	EFO_0000756	melanoma	melanoma	EFO_0000756	Cancer	Neoplasms	PGS000430	EFO_0000756	melanoma	melanoma	EFO_0000756	Cancer	Neoplasms
0.7172	2.29E-73	2.26E-70	PGS000051	EFO_0000305	breast carcinoma	breast carcinoma	EFO_0000305	Cancer	Neoplasms	PGS000488	EFO_0000305	breast carcinoma	breast carcinoma	EFO_0000305	Cancer	Neoplasms
0.7171	2.32E-73	2.28E-70	PGS001971	EFO_0004584	mean platelet volume	mean platelet volume	EFO_0004584	Hematological measurement	Hematological	PGS002608	EFO_0004584	mean platelet volume	mean platelet volume	EFO_0004584	Hematological measurement	Hematological
0.7171	2.33E-73	2.29E-70	PGS002206	EFO_0004527	mean corpuscular hemoglobin	mean corpuscular hemoglobin	EFO_0004527	Hematological measurement	Hematological	PGS000174	EFO_0004527	mean corpuscular hemoglobin	mean corpuscular hemoglobin	EFO_0004527	Hematological measurement	Hematological
0.7171	2.33E-73	2.30E-70	PGS001752	EFO_0006930	brain volume measurement|neuroimaging measurement	brain volume measurement	EFO_0006930	Other measurement	Neurological	PGS001735	EFO_0006930	measurement	brain volume measurement	EFO_0006930	Other measurement	Neurological
0.7171	2.34E-73	2.30E-70	PGS000344	EFO_0000305	breast carcinoma	breast carcinoma	EFO_0000305	Cancer	Neoplasms	PGS000006	EFO_1000650	estrogen-receptor negative breast cancer	cancer	EFO_1000650	Cancer	Neoplasms
0.7171	2.34E-73	2.31E-70	PGS001983	EFO_0004342	waist circumference	waist circumference	EFO_0004342	Body measurement	Body measurement	PGS001227	EFO_0004342	waist circumference	waist circumference	EFO_0004342	Body measurement	Body measurement
0.7171	2.39E-73	2.35E-70	PGS002463	EFO_0007660	neuroticism measurement	neuroticism measurement	EFO_0007660	Other measurement	Psychiatric	PGS002414	EFO_0007660	neuroticism measurement	neuroticism measurement	EFO_0007660	Other measurement	Psychiatric
0.7171	2.43E-73	2.39E-70	PGS000510	EFO_0000305	breast carcinoma	breast carcinoma	EFO_0000305	Cancer	Neoplasms	PGS000512	EFO_0000305	breast carcinoma	breast carcinoma	EFO_0000305	Cancer	Neoplasms
0.717	2.49E-73	2.45E-70	PGS001403	EFO_0009270	heel bone mineral density	heel bone mineral density	EFO_0009270	Other measurement	Skeletal	PGS002175	EFO_0005654	velocity of sound measurement	velocity of sound measurement	EFO_0005654	Other measurement	Others
-0.717	2.50E-73	2.46E-70	PGS000116	EFO_0001645	coronary artery disease	coronary artery disease	EFO_0001645	Cardiovascular disease	Cardiovascular	PGS003355	EFO_0001645	coronary artery disease	coronary artery disease	EFO_0001645	Cardiovascular disease	Cardiovascular
0.717	2.52E-73	2.48E-70	PGS001682	EFO_0006930	brain volume measurement|neuroimaging measurement	brain volume measurement	EFO_0006930	Other measurement	Neurological	PGS001480	EFO_0006930	measurement	brain volume measurement	EFO_0006930	Other measurement	Neurological
0.717	2.54E-73	2.50E-70	PGS003243	EFO_0010472	creatine measurement	creatine measurement	EFO_0010472	Other measurement	Metabolic	PGS003242	EFO_0010472	creatine measurement	creatine measurement	EFO_0010472	Other measurement	Metabolic
0.7169	2.65E-73	2.61E-70	PGS002851	EFO_0004340	body mass index	body mass index	EFO_0004340	Body measurement	Body measurement	PGS002844	EFO_0004340	body mass index	body mass index	EFO_0004340	Body measurement	Body measurement
0.7169	2.75E-73	2.70E-70	PGS001753	EFO_0006930	brain volume measurement|neuroimaging measurement	brain volume measurement	EFO_0006930	Other measurement	Neurological	PGS001708	EFO_0006930	measurement	brain volume measurement	EFO_0006930	Other measurement	Neurological
0.7168	2.85E-73	2.80E-70	PGS003186	EFO_0004631	vitamin D measurement	vitamin D measurement	EFO_0004631	Lipid or lipoprotein measurement	Metabolic	PGS002574	EFO_0004631	vitamin D measurement	vitamin D measurement	EFO_0004631	Lipid or lipoprotein measurement	Metabolic
0.7168	2.86E-73	2.81E-70	PGS001970	EFO_0007985	platelet crit	platelet crit	EFO_0007985	Hematological measurement	Hematological	PGS000107	EFO_0007985	platelet crit	platelet crit	EFO_0007985	Hematological measurement	Hematological
0.7168	2.88E-73	2.83E-70	PGS000569	EFO_0001663	prostate carcinoma	prostate carcinoma	EFO_0001663	Cancer	Neoplasms	PGS001805	EFO_0001663	prostate carcinoma	prostate carcinoma	EFO_0001663	Cancer	Neoplasms
0.7168	2.94E-73	2.89E-70	PGS002780	MONDO_0005148	type 2 diabetes mellitus	type 2 diabetes mellitus	MONDO_0005148	Metabolic disorder	Metabolic	PGS002779	MONDO_0005148	type 2 diabetes mellitus	type 2 diabetes mellitus	MONDO_0005148	Digestive system disorder	Gastrointestinal
0.7168	2.94E-73	2.89E-70	PGS002780	MONDO_0005148	type 2 diabetes mellitus	type 2 diabetes mellitus	MONDO_0005148	Digestive system disorder	Gastrointestinal	PGS002779	MONDO_0005148	type 2 diabetes mellitus	type 2 diabetes mellitus	MONDO_0005148	Digestive system disorder	Gastrointestinal
0.7168	2.94E-73	2.89E-70	PGS002780	MONDO_0005148	type 2 diabetes mellitus	type 2 diabetes mellitus	MONDO_0005148	Metabolic disorder	Metabolic	PGS002779	MONDO_0005148	type 2 diabetes mellitus	type 2 diabetes mellitus	MONDO_0005148	Metabolic disorder	Metabolic
0.7168	2.94E-73	2.89E-70	PGS002780	MONDO_0005148	type 2 diabetes mellitus	type 2 diabetes mellitus	MONDO_0005148	Digestive system disorder	Gastrointestinal	PGS002779	MONDO_0005148	type 2 diabetes mellitus	type 2 diabetes mellitus	MONDO_0005148	Metabolic disorder	Metabolic
0.7167	3.06E-73	3.01E-70	PGS000049	EFO_0001663	prostate carcinoma	prostate carcinoma	EFO_0001663	Cancer	Neoplasms	PGS000719	EFO_0001663	prostate carcinoma	prostate carcinoma	EFO_0001663	Cancer	Neoplasms
0.7167	3.10E-73	3.04E-70	PGS001929	EFO_0004339	body height	body height	EFO_0004339	Body measurement	Body measurement	PGS002966	EFO_0004339	body height	body height	EFO_0004339	Body measurement	Body measurement
0.7167	3.18E-73	3.13E-70	PGS002854	EFO_0004340	body mass index	body mass index	EFO_0004340	Body measurement	Body measurement	PGS002856	EFO_0004340	body mass index	body mass index	EFO_0004340	Body measurement	Body measurement
-0.7166	3.33E-73	3.27E-70	PGS003228	EFO_0005208	glomerular filtration rate	glomerular filtration rate	EFO_0005208	Other measurement	Genitourinary	PGS003268	EFO_0010472	creatine measurement|glomerular filtration rate	creatine measurement	EFO_0010472	Other measurement	Metabolic
0.7166	3.38E-73	3.32E-70	PGS001752	EFO_0006930	brain volume measurement|neuroimaging measurement	brain volume measurement	EFO_0006930	Other measurement	Neurological	PGS001721	EFO_0006930	measurement	brain volume measurement	EFO_0006930	Other measurement	Neurological
0.7166	3.39E-73	3.32E-70	PGS000347	EFO_1000649	estrogen-receptor positive breast cancer	estrogen-receptor positive breast cancer	EFO_1000649	Cancer	Neoplasms	PGS000783	EFO_0000305	breast carcinoma	breast carcinoma	EFO_0000305	Cancer	Neoplasms
0.7166	3.39E-73	3.33E-70	PGS000065	EFO_0004611	low density lipoprotein cholesterol measurement	low density lipoprotein cholesterol measurement	EFO_0004611	Lipid or lipoprotein measurement	Metabolic	PGS000311	EFO_0004574	total cholesterol measurement	total cholesterol measurement	EFO_0004574	Lipid or lipoprotein measurement	Metabolic
0.7166	3.39E-73	3.33E-70	PGS000065	EFO_0004611	low density lipoprotein cholesterol measurement	low density lipoprotein cholesterol measurement	EFO_0004611	Cardiovascular measurement	Cardiovascular	PGS000311	EFO_0004574	total cholesterol measurement	total cholesterol measurement	EFO_0004574	Lipid or lipoprotein measurement	Metabolic
-0.7165	3.48E-73	3.41E-70	PGS003278	EFO_0010472	creatine measurement|glomerular filtration rate	creatine measurement	EFO_0010472	Other measurement	Metabolic	PGS003268	EFO_0010472	creatine measurement|glomerular filtration rate	creatine measurement	EFO_0010472	Other measurement	Metabolic
0.7165	3.51E-73	3.44E-70	PGS001105	EFO_0007800	body fat percentage	body fat percentage	EFO_0007800	Body measurement	Body measurement	PGS002133	EFO_0007800	body fat percentage	body fat percentage	EFO_0007800	Body measurement	Body measurement
0.7165	3.53E-73	3.46E-70	PGS002844	EFO_0004340	body mass index	body mass index	EFO_0004340	Body measurement	Body measurement	PGS001951	EFO_0005409	fat body mass	fat body mass	EFO_0005409	Other measurement	Body measurement
0.7165	3.58E-73	3.50E-70	PGS000065	EFO_0004611	low density lipoprotein cholesterol measurement	low density lipoprotein cholesterol measurement	EFO_0004611	Cardiovascular measurement	Cardiovascular	PGS000688	EFO_0004611	low density lipoprotein cholesterol measurement	measurement	EFO_0004611	Cardiovascular measurement	Cardiovascular
0.7165	3.58E-73	3.50E-70	PGS000065	EFO_0004611	low density lipoprotein cholesterol measurement	low density lipoprotein cholesterol measurement	EFO_0004611	Lipid or lipoprotein measurement	Metabolic	PGS000688	EFO_0004611	low density lipoprotein cholesterol measurement	measurement	EFO_0004611	Lipid or lipoprotein measurement	Metabolic
0.7165	3.58E-73	3.50E-70	PGS000065	EFO_0004611	low density lipoprotein cholesterol measurement	low density lipoprotein cholesterol measurement	EFO_0004611	Lipid or lipoprotein measurement	Metabolic	PGS000688	EFO_0004611	low density lipoprotein cholesterol measurement	measurement	EFO_0004611	Cardiovascular measurement	Cardiovascular
0.7165	3.58E-73	3.50E-70	PGS000065	EFO_0004611	low density lipoprotein cholesterol measurement	low density lipoprotein cholesterol measurement	EFO_0004611	Cardiovascular measurement	Cardiovascular	PGS000688	EFO_0004611	low density lipoprotein cholesterol measurement	measurement	EFO_0004611	Lipid or lipoprotein measurement	Metabolic
0.7165	3.60E-73	3.52E-70	PGS002869	EFO_0004458	C-reactive protein measurement	C-reactive protein measurement	EFO_0004458	Inflammatory measurement	Immunological	PGS002868	EFO_0004458	C-reactive protein measurement	C-reactive protein measurement	EFO_0004458	Inflammatory measurement	Immunological
-0.7165	3.60E-73	3.53E-70	PGS003243	EFO_0010472	creatine measurement	creatine measurement	EFO_0010472	Other measurement	Metabolic	PGS000885	EFO_0005208	glomerular filtration rate	glomerular filtration rate	EFO_0005208	Other measurement	Genitourinary
0.7165	3.62E-73	3.54E-70	PGS000893	EFO_0004611	low density lipoprotein cholesterol measurement	low density lipoprotein cholesterol measurement	EFO_0004611	Cardiovascular measurement	Cardiovascular	PGS000888	EFO_0004611	low density lipoprotein cholesterol measurement	measurement	EFO_0004611	Lipid or lipoprotein measurement	Metabolic
0.7165	3.62E-73	3.54E-70	PGS000893	EFO_0004611	low density lipoprotein cholesterol measurement	low density lipoprotein cholesterol measurement	EFO_0004611	Lipid or lipoprotein measurement	Metabolic	PGS000888	EFO_0004611	low density lipoprotein cholesterol measurement	measurement	EFO_0004611	Cardiovascular measurement	Cardiovascular
0.7165	3.62E-73	3.54E-70	PGS000893	EFO_0004611	low density lipoprotein cholesterol measurement	low density lipoprotein cholesterol measurement	EFO_0004611	Lipid or lipoprotein measurement	Metabolic	PGS000888	EFO_0004611	low density lipoprotein cholesterol measurement	measurement	EFO_0004611	Lipid or lipoprotein measurement	Metabolic
0.7165	3.62E-73	3.54E-70	PGS000893	EFO_0004611	low density lipoprotein cholesterol measurement	low density lipoprotein cholesterol measurement	EFO_0004611	Cardiovascular measurement	Cardiovascular	PGS000888	EFO_0004611	low density lipoprotein cholesterol measurement	measurement	EFO_0004611	Cardiovascular measurement	Cardiovascular
0.7165	3.66E-73	3.58E-70	PGS002776	EFO_0001645	coronary artery disease	coronary artery disease	EFO_0001645	Cardiovascular disease	Cardiovascular	PGS002244	EFO_0001645	coronary artery disease	coronary artery disease	EFO_0001645	Cardiovascular disease	Cardiovascular
0.7164	3.75E-73	3.67E-70	PGS000347	EFO_1000649	estrogen-receptor positive breast cancer	estrogen-receptor positive breast cancer	EFO_1000649	Cancer	Neoplasms	PGS000346	EFO_1000650	estrogen-receptor negative breast cancer	cancer	EFO_1000650	Cancer	Neoplasms
0.7164	3.88E-73	3.79E-70	PGS003201	EFO_0004631	vitamin D measurement	vitamin D measurement	EFO_0004631	Lipid or lipoprotein measurement	Metabolic	PGS001982	EFO_0004631	vitamin D measurement	vitamin D measurement	EFO_0004631	Lipid or lipoprotein measurement	Metabolic
0.7164	3.90E-73	3.82E-70	PGS001917	EFO_0007800	body fat percentage	body fat percentage	EFO_0007800	Body measurement	Body measurement	PGS002313	EFO_0004340	body mass index	body mass index	EFO_0004340	Body measurement	Body measurement
0.7164	3.96E-73	3.87E-70	PGS001479	EFO_0006930	brain volume measurement|neuroimaging measurement	brain volume measurement	EFO_0006930	Other measurement	Neurological	PGS001451	EFO_0006930	measurement	brain volume measurement	EFO_0006930	Other measurement	Neurological
0.7163	3.99E-73	3.90E-70	PGS000503	EFO_0000305	breast carcinoma	breast carcinoma	EFO_0000305	Cancer	Neoplasms	PGS000497	EFO_0000305	breast carcinoma	breast carcinoma	EFO_0000305	Cancer	Neoplasms
0.7163	3.99E-73	3.90E-70	PGS000503	EFO_0000305	breast carcinoma	breast carcinoma	EFO_0000305	Cancer	Neoplasms	PGS000498	EFO_0000305	breast carcinoma	breast carcinoma	EFO_0000305	Cancer	Neoplasms
-0.7163	4.01E-73	3.93E-70	PGS003278	EFO_0010472	creatine measurement|glomerular filtration rate	creatine measurement	EFO_0010472	Other measurement	Metabolic	PGS003253	EFO_0010472	creatine measurement|glomerular filtration rate	creatine measurement	EFO_0010472	Other measurement	Metabolic
0.7163	4.03E-73	3.94E-70	PGS000004	EFO_0000305	breast carcinoma	breast carcinoma	EFO_0000305	Cancer	Neoplasms	PGS000052	EFO_0000305	breast carcinoma	breast carcinoma	EFO_0000305	Cancer	Neoplasms
0.7163	4.03E-73	3.94E-70	PGS001645	EFO_0006930	brain volume measurement|neuroimaging measurement	brain volume measurement	EFO_0006930	Other measurement	Neurological	PGS001646	EFO_0006930	measurement	brain volume measurement	EFO_0006930	Other measurement	Neurological
0.7163	4.20E-73	4.10E-70	PGS003031	EFO_0004611	low density lipoprotein cholesterol measurement	low density lipoprotein cholesterol measurement	EFO_0004611	Cardiovascular measurement	Cardiovascular	PGS000889	EFO_0004611	low density lipoprotein cholesterol measurement	measurement	EFO_0004611	Cardiovascular measurement	Cardiovascular
0.7163	4.20E-73	4.10E-70	PGS003031	EFO_0004611	low density lipoprotein cholesterol measurement	low density lipoprotein cholesterol measurement	EFO_0004611	Cardiovascular measurement	Cardiovascular	PGS000889	EFO_0004611	low density lipoprotein cholesterol measurement	measurement	EFO_0004611	Lipid or lipoprotein measurement	Metabolic
0.7163	4.20E-73	4.10E-70	PGS003031	EFO_0004611	low density lipoprotein cholesterol measurement	low density lipoprotein cholesterol measurement	EFO_0004611	Lipid or lipoprotein measurement	Metabolic	PGS000889	EFO_0004611	low density lipoprotein cholesterol measurement	measurement	EFO_0004611	Cardiovascular measurement	Cardiovascular
0.7163	4.20E-73	4.10E-70	PGS003031	EFO_0004611	low density lipoprotein cholesterol measurement	low density lipoprotein cholesterol measurement	EFO_0004611	Lipid or lipoprotein measurement	Metabolic	PGS000889	EFO_0004611	low density lipoprotein cholesterol measurement	measurement	EFO_0004611	Lipid or lipoprotein measurement	Metabolic
0.7163	4.20E-73	4.11E-70	PGS001833	EFO_0005773	retinal detachment	retinal detachment	EFO_0005773	Neurological disorder	Neurological	PGS002040	EFO_0005773	retinal detachment	retinal detachment	EFO_0005773	Neurological disorder	Neurological
-0.7162	4.32E-73	4.22E-70	PGS000116	EFO_0001645	coronary artery disease	coronary artery disease	EFO_0001645	Cardiovascular disease	Cardiovascular	PGS001355	EFO_0001645	coronary artery disease	coronary artery disease	EFO_0001645	Cardiovascular disease	Cardiovascular
0.7162	4.39E-73	4.28E-70	PGS000533	EFO_0000305	breast carcinoma	breast carcinoma	EFO_0000305	Cancer	Neoplasms	PGS000532	EFO_0000305	breast carcinoma	breast carcinoma	EFO_0000305	Cancer	Neoplasms
0.7162	4.39E-73	4.28E-70	PGS000534	EFO_0000305	breast carcinoma	breast carcinoma	EFO_0000305	Cancer	Neoplasms	PGS000532	EFO_0000305	breast carcinoma	breast carcinoma	EFO_0000305	Cancer	Neoplasms
0.7162	4.41E-73	4.31E-70	PGS003189	EFO_0004631	vitamin D measurement	vitamin D measurement	EFO_0004631	Lipid or lipoprotein measurement	Metabolic	PGS003190	EFO_0004631	vitamin D measurement	vitamin D measurement	EFO_0004631	Lipid or lipoprotein measurement	Metabolic
0.7161	4.68E-73	4.57E-70	PGS001751	EFO_0006930	brain volume measurement|neuroimaging measurement	brain volume measurement	EFO_0006930	Other measurement	Neurological	PGS001721	EFO_0006930	measurement	brain volume measurement	EFO_0006930	Other measurement	Neurological
0.7161	4.84E-73	4.72E-70	PGS003223	EFO_0005208	glomerular filtration rate	glomerular filtration rate	EFO_0005208	Other measurement	Genitourinary	PGS003227	EFO_0005208	glomerular filtration rate	glomerular filtration rate	EFO_0005208	Other measurement	Genitourinary
0.716	4.91E-73	4.79E-70	PGS000478	EFO_0000305	breast carcinoma	breast carcinoma	EFO_0000305	Cancer	Neoplasms	PGS001336	EFO_0000305	breast carcinoma	breast carcinoma	EFO_0000305	Cancer	Neoplasms
0.716	4.93E-73	4.80E-70	PGS002315	EFO_0003923	bone density	bone density	EFO_0003923	Other measurement	Skeletal	PGS001403	EFO_0009270	heel bone mineral density	heel bone mineral density	EFO_0009270	Other measurement	Skeletal
0.716	4.94E-73	4.82E-70	PGS000434	MONDO_0002898	skin cancer	skin cancer	MONDO_0002898	Cancer	Neoplasms	PGS000405	EFO_0009259	skin carcinoma	skin carcinoma	EFO_0009259	Cancer	Neoplasms
0.716	4.97E-73	4.84E-70	PGS002534	EFO_0003923	bone density	bone density	EFO_0003923	Other measurement	Skeletal	PGS002315	EFO_0003923	bone density	bone density	EFO_0003923	Other measurement	Skeletal
0.716	5.05E-73	4.93E-70	PGS002934	EFO_0004468	glucose measurement	glucose measurement	EFO_0004468	Other measurement	Metabolic	PGS002943	EFO_0004468	glucose measurement	glucose measurement	EFO_0004468	Other measurement	Metabolic
0.7159	5.36E-73	5.23E-70	PGS003034	EFO_0004611	low density lipoprotein cholesterol measurement	low density lipoprotein cholesterol measurement	EFO_0004611	Lipid or lipoprotein measurement	Metabolic	PGS003037	EFO_0004611	low density lipoprotein cholesterol measurement	measurement	EFO_0004611	Cardiovascular measurement	Cardiovascular
0.7159	5.36E-73	5.23E-70	PGS003034	EFO_0004611	low density lipoprotein cholesterol measurement	low density lipoprotein cholesterol measurement	EFO_0004611	Lipid or lipoprotein measurement	Metabolic	PGS003037	EFO_0004611	low density lipoprotein cholesterol measurement	measurement	EFO_0004611	Lipid or lipoprotein measurement	Metabolic
0.7159	5.36E-73	5.23E-70	PGS003034	EFO_0004611	low density lipoprotein cholesterol measurement	low density lipoprotein cholesterol measurement	EFO_0004611	Cardiovascular measurement	Cardiovascular	PGS003037	EFO_0004611	low density lipoprotein cholesterol measurement	measurement	EFO_0004611	Lipid or lipoprotein measurement	Metabolic
0.7159	5.36E-73	5.23E-70	PGS003034	EFO_0004611	low density lipoprotein cholesterol measurement	low density lipoprotein cholesterol measurement	EFO_0004611	Cardiovascular measurement	Cardiovascular	PGS003037	EFO_0004611	low density lipoprotein cholesterol measurement	measurement	EFO_0004611	Cardiovascular measurement	Cardiovascular
0.7159	5.46E-73	5.32E-70	PGS002339	EFO_0004527	mean corpuscular hemoglobin	mean corpuscular hemoglobin	EFO_0004527	Hematological measurement	Hematological	PGS002558	EFO_0004527	mean corpuscular hemoglobin	mean corpuscular hemoglobin	EFO_0004527	Hematological measurement	Hematological
0.7158	5.56E-73	5.42E-70	PGS001687	EFO_0006930	brain volume measurement|neuroimaging measurement	brain volume measurement	EFO_0006930	Other measurement	Neurological	PGS001492	EFO_0006930	measurement	brain volume measurement	EFO_0006930	Other measurement	Neurological
0.7158	5.59E-73	5.44E-70	PGS000008	EFO_1000649	estrogen-receptor positive breast cancer	estrogen-receptor positive breast cancer	EFO_1000649	Cancer	Neoplasms	PGS000212	MONDO_0021116	luminal A breast carcinoma	luminal A breast carcinoma	MONDO_0021116	Cancer	Neoplasms
0.7158	5.62E-73	5.47E-70	PGS000127	EFO_0004541	HbA1c measurement	HbA1c measurement	EFO_0004541	Hematological measurement	Hematological	PGS000304	EFO_0004541	HbA1c measurement	HbA1c measurement	EFO_0004541	Hematological measurement	Hematological
0.7158	5.63E-73	5.48E-70	PGS001489	EFO_0006930	brain volume measurement|neuroimaging measurement	brain volume measurement	EFO_0006930	Other measurement	Neurological	PGS001481	EFO_0006930	measurement	brain volume measurement	EFO_0006930	Other measurement	Neurological
0.7158	5.63E-73	5.49E-70	PGS000046	EFO_1000649	estrogen-receptor positive breast cancer	estrogen-receptor positive breast cancer	EFO_1000649	Cancer	Neoplasms	PGS000052	EFO_0000305	breast carcinoma	breast carcinoma	EFO_0000305	Cancer	Neoplasms
0.7158	5.66E-73	5.51E-70	PGS002391	EFO_0011015	educational attainment	educational attainment	EFO_0011015	Other measurement	Activities	PGS002587	EFO_0011015	educational attainment	educational attainment	EFO_0011015	Other measurement	Activities

0.7158	5.67E-73	5.52E-70	PGS003328	EFO_0004698	insomnia	insomnia	EFO_0004698	Neurological disorder	Neurological	PGS001932	EFO_0004698	insomnia	insomnia	EFO_0004698	Neurological disorder	Neurological
0.7158	5.80E-73	5.64E-70	PGS001333	EFO_0000341	chronic obstructive pulmonary disease	chronic obstructive pulmonary disease	EFO_0000341	Other disease	Respiratory	PGS001332	EFO_0000341	chronic obstructive pulmonary disease	chronic obstructive pulmonary disease	EFO_0000341	Other disease	Respiratory
0.7158	5.91E-73	5.75E-70	PGS001476	EFO_0006930	brain volume measurement|neuroimaging measurement	brain volume measurement	EFO_0006930	Other measurement	Neurological	PGS001671	EFO_0006930	measurement	brain volume measurement	EFO_0006930	Other measurement	Neurological
0.7157	5.98E-73	5.82E-70	PGS002846	EFO_0004340	body mass index	body mass index	EFO_0004340	Body measurement	Body measurement	PGS002854	EFO_0004340	body mass index	body mass index	EFO_0004340	Body measurement	Body measurement
0.7157	6.07E-73	5.90E-70	PGS000540	EFO_0000305	breast carcinoma	breast carcinoma	EFO_0000305	Cancer	Neoplasms	PGS001336	EFO_0000305	breast carcinoma	breast carcinoma	EFO_0000305	Cancer	Neoplasms
-0.7157	6.10E-73	5.93E-70	PGS001945	EFO_0004518	creatinine measurement	creatinine measurement	EFO_0004518	Other measurement	Metabolic	PGS000682	EFO_0005208	glomerular filtration rate	glomerular filtration rate	EFO_0005208	Other measurement	Genitourinary
0.7157	6.13E-73	5.96E-70	PGS000889	EFO_0004611	low density lipoprotein cholesterol measurement	low density lipoprotein cholesterol measurement	EFO_0004611	Cardiovascular measurement	Cardiovascular	PGS001933	EFO_0004611	low density lipoprotein cholesterol measurement	measurement	EFO_0004611	Lipid or lipoprotein measurement	Metabolic
0.7157	6.13E-73	5.96E-70	PGS000889	EFO_0004611	low density lipoprotein cholesterol measurement	low density lipoprotein cholesterol measurement	EFO_0004611	Cardiovascular measurement	Cardiovascular	PGS001933	EFO_0004611	low density lipoprotein cholesterol measurement	measurement	EFO_0004611	Cardiovascular measurement	Cardiovascular
0.7157	6.13E-73	5.96E-70	PGS000889	EFO_0004611	low density lipoprotein cholesterol measurement	low density lipoprotein cholesterol measurement	EFO_0004611	Lipid or lipoprotein measurement	Metabolic	PGS001933	EFO_0004611	low density lipoprotein cholesterol measurement	measurement	EFO_0004611	Cardiovascular measurement	Cardiovascular
0.7157	6.13E-73	5.96E-70	PGS000889	EFO_0004611	low density lipoprotein cholesterol measurement	low density lipoprotein cholesterol measurement	EFO_0004611	Lipid or lipoprotein measurement	Metabolic	PGS001933	EFO_0004611	low density lipoprotein cholesterol measurement	measurement	EFO_0004611	Lipid or lipoprotein measurement	Metabolic
0.7157	6.25E-73	6.08E-70	PGS002782	EFO_0005689	non-high density lipoprotein cholesterol measurement	non-high density lipoprotein cholesterol measurement	EFO_0005689	Cardiovascular measurement	Cardiovascular	PGS001933	EFO_0004611	low density lipoprotein cholesterol measurement	measurement	EFO_0004611	Cardiovascular measurement	Cardiovascular
0.7157	6.25E-73	6.08E-70	PGS002782	EFO_0005689	non-high density lipoprotein cholesterol measurement	non-high density lipoprotein cholesterol measurement	EFO_0005689	Lipid or lipoprotein measurement	Metabolic	PGS001933	EFO_0004611	low density lipoprotein cholesterol measurement	measurement	EFO_0004611	Cardiovascular measurement	Cardiovascular
0.7157	6.25E-73	6.08E-70	PGS002782	EFO_0005689	non-high density lipoprotein cholesterol measurement	non-high density lipoprotein cholesterol measurement	EFO_0005689	Cardiovascular measurement	Cardiovascular	PGS001933	EFO_0004611	low density lipoprotein cholesterol measurement	measurement	EFO_0004611	Lipid or lipoprotein measurement	Metabolic
0.7157	6.25E-73	6.08E-70	PGS002782	EFO_0005689	non-high density lipoprotein cholesterol measurement	non-high density lipoprotein cholesterol measurement	EFO_0005689	Lipid or lipoprotein measurement	Metabolic	PGS001933	EFO_0004611	low density lipoprotein cholesterol measurement	measurement	EFO_0004611	Lipid or lipoprotein measurement	Metabolic
0.7157	6.31E-73	6.13E-70	PGS002721	EFO_0004631	vitamin D measurement	vitamin D measurement	EFO_0004631	Lipid or lipoprotein measurement	Metabolic	PGS002574	EFO_0004631	vitamin D measurement	vitamin D measurement	EFO_0004631	Lipid or lipoprotein measurement	Metabolic
0.7156	6.47E-73	6.29E-70	PGS001489	EFO_0006930	brain volume measurement|neuroimaging measurement	brain volume measurement	EFO_0006930	Other measurement	Neurological	PGS001472	EFO_0006930	measurement	brain volume measurement	EFO_0006930	Other measurement	Neurological
0.7156	6.55E-73	6.36E-70	PGS003248	EFO_0010472	creatine measurement	creatine measurement	EFO_0010472	Other measurement	Metabolic	PGS000678	EFO_0004518	creatinine measurement	creatinine measurement	EFO_0004518	Other measurement	Metabolic
0.7156	6.61E-73	6.42E-70	PGS002393	EFO_0000400	diabetes mellitus	diabetes mellitus	EFO_0000400	Metabolic disorder	Metabolic	PGS002599	EFO_0004541	HbA1c measurement	HbA1c measurement	EFO_0004541	Hematological measurement	Hematological
0.7156	6.61E-73	6.42E-70	PGS002393	EFO_0000400	diabetes mellitus	diabetes mellitus	EFO_0000400	Digestive system disorder	Gastrointestinal	PGS002599	EFO_0004541	HbA1c measurement	HbA1c measurement	EFO_0004541	Hematological measurement	Hematological
0.7155	6.78E-73	6.58E-70	PGS000841	EFO_0004340	body mass index	body mass index	EFO_0004340	Body measurement	Body measurement	PGS002251	EFO_0004340	body mass index	body mass index	EFO_0004340	Body measurement	Body measurement
0.7155	6.79E-73	6.58E-70	PGS000587	EFO_0001663	prostate carcinoma	prostate carcinoma	EFO_0001663	Cancer	Neoplasms	PGS000582	EFO_0001663	prostate carcinoma	prostate carcinoma	EFO_0001663	Cancer	Neoplasms
0.7155	6.79E-73	6.58E-70	PGS000587	EFO_0001663	prostate carcinoma	prostate carcinoma	EFO_0001663	Cancer	Neoplasms	PGS000581	EFO_0001663	prostate carcinoma	prostate carcinoma	EFO_0001663	Cancer	Neoplasms
0.7155	6.92E-73	6.71E-70	PGS002623	EFO_0004631	vitamin D measurement	vitamin D measurement	EFO_0004631	Lipid or lipoprotein measurement	Metabolic	PGS003200	EFO_0004631	vitamin D measurement	vitamin D measurement	EFO_0004631	Lipid or lipoprotein measurement	Metabolic
0.7155	6.96E-73	6.75E-70	PGS001264	EFO_0003907	deep vein thrombosis	deep vein thrombosis	EFO_0003907	Other trait	Cardiovascular	PGS001847	EFO_0004264	vascular disease	vascular disease	EFO_0004264	Cardiovascular disease	Cardiovascular
0.7155	7.23E-73	7.01E-70	PGS002173	EFO_0009270	heel bone mineral density	heel bone mineral density	EFO_0009270	Other measurement	Skeletal	PGS001403	EFO_0009270	heel bone mineral density	heel bone mineral density	EFO_0009270	Other measurement	Skeletal
0.7154	7.33E-73	7.11E-70	PGS002416	EFO_0000676	psoriasis	psoriasis	EFO_0000676	Immune system disorder	Immunological	PGS002563	EFO_0000676	psoriasis	psoriasis	EFO_0000676	Immune system disorder	Immunological
0.7154	7.35E-73	7.13E-70	PGS000215	EFO_1000294	HER2 Positive Breast Carcinoma	HER2 Positive Breast Carcinoma	EFO_1000294	Cancer	Neoplasms	PGS003399	EFO_0000305	breast carcinoma	breast carcinoma	EFO_0000305	Cancer	Neoplasms
0.7154	7.36E-73	7.13E-70	PGS000176	EFO_0004526	mean corpuscular volume	mean corpuscular volume	EFO_0004526	Hematological measurement	Hematological	PGS002206	EFO_0004527	mean corpuscular hemoglobin	mean corpuscular hemoglobin	EFO_0004527	Hematological measurement	Hematological
0.7154	7.59E-73	7.36E-70	PGS000528	EFO_0000305	breast carcinoma	breast carcinoma	EFO_0000305	Cancer	Neoplasms	PGS000532	EFO_0000305	breast carcinoma	breast carcinoma	EFO_0000305	Cancer	Neoplasms
-0.7154	7.68E-73	7.44E-70	PGS002204	EFO_0007825	balding measurement	balding measurement	EFO_0007825	Other measurement	Activities	PGS002631	EFO_0007825	balding measurement	balding measurement	EFO_0007825	Other measurement	Activities
0.7153	7.80E-73	7.55E-70	PGS000751	EFO_0001663	prostate carcinoma	prostate carcinoma	EFO_0001663	Cancer	Neoplasms	PGS000795	EFO_0001663	prostate carcinoma	prostate carcinoma	EFO_0001663	Cancer	Neoplasms
0.7153	7.91E-73	7.66E-70	PGS001804	EFO_0000305	breast carcinoma	breast carcinoma	EFO_0000305	Cancer	Neoplasms	PGS000483	EFO_0000305	breast carcinoma	breast carcinoma	EFO_0000305	Cancer	Neoplasms
0.7153	7.95E-73	7.70E-70	PGS001652	EFO_0006930	brain volume measurement|neuroimaging measurement	brain volume measurement	EFO_0006930	Other measurement	Neurological	PGS001653	EFO_0006930	measurement	brain volume measurement	EFO_0006930	Other measurement	Neurological
0.7153	8.12E-73	7.86E-70	PGS000741	EFO_0001663	prostate carcinoma	prostate carcinoma	EFO_0001663	Cancer	Neoplasms	PGS000593	EFO_0007355	male reproductive organ cancer	male reproductive organ cancer	EFO_0007355	Cancer	Neoplasms
0.7152	8.43E-73	8.16E-70	PGS002330	EFO_0007822	hair colour measurement	hair colour measurement	EFO_0007822	Other measurement	Dermatological	PGS001096	EFO_0007822	hair colour measurement	hair colour measurement	EFO_0007822	Other measurement	Dermatological
0.7152	8.81E-73	8.52E-70	PGS002183	EFO_0004587	lymphocyte count	lymphocyte count	EFO_0004587	Hematological measurement	Hematological	PGS002655	EFO_0004587	lymphocyte count	lymphocyte count	EFO_0004587	Hematological measurement	Hematological
0.7151	8.88E-73	8.59E-70	PGS001751	EFO_0006930	brain volume measurement|neuroimaging measurement	brain volume measurement	EFO_0006930	Other measurement	Neurological	PGS001724	EFO_0006930	measurement	brain volume measurement	EFO_0006930	Other measurement	Neurological
0.7151	9.11E-73	8.81E-70	PGS003380	EFO_0000305	breast carcinoma	breast carcinoma	EFO_0000305	Cancer	Neoplasms	PGS000008	EFO_1000649	estrogen-receptor positive breast cancer	cancer	EFO_1000649	Cancer	Neoplasms
0.7151	9.20E-73	8.90E-70	PGS002668	EFO_1000627	thyroid disease	thyroid disease	EFO_1000627	Other disease	Endocrine	PGS000761	EFO_0004705	hypothyroidism	hypothyroidism	EFO_0004705	Other disease	Endocrine
0.7151	9.35E-73	9.04E-70	PGS000105	EFO_0004833	neutrophil count	neutrophil count	EFO_0004833	Hematological measurement	Hematological	PGS001969	EFO_0004833	neutrophil count	neutrophil count	EFO_0004833	Hematological measurement	Hematological
0.7151	9.37E-73	9.05E-70	PGS002929	EFO_0004465	fasting blood glucose measurement	fasting blood glucose measurement	EFO_0004465	Other measurement	Metabolic	PGS002933	EFO_0004465	fasting blood glucose measurement	fasting blood glucose measurement	EFO_0004465	Other measurement	Metabolic
0.7149	1.02E-72	9.84E-70	PGS001673	EFO_0006930	brain volume measurement|neuroimaging measurement	brain volume measurement	EFO_0006930	Other measurement	Neurological	PGS001669	EFO_0006930	measurement	brain volume measurement	EFO_0006930	Other measurement	Neurological
0.7149	1.04E-72	1.00E-69	PGS002983	EFO_0004339	body height	body height	EFO_0004339	Body measurement	Body measurement	PGS002980	EFO_0004339	body height	body height	EFO_0004339	Body measurement	Body measurement
0.7149	1.05E-72	1.02E-69	PGS000589	EFO_0001663	prostate carcinoma	prostate carcinoma	EFO_0001663	Cancer	Neoplasms	PGS001805	EFO_0001663	prostate carcinoma	prostate carcinoma	EFO_0001663	Cancer	Neoplasms
0.7149	1.06E-72	1.03E-69	PGS001489	EFO_0006930	brain volume measurement|neuroimaging measurement	brain volume measurement	EFO_0006930	Other measurement	Neurological	PGS001473	EFO_0006930	measurement	brain volume measurement	EFO_0006930	Other measurement	Neurological
0.7149	1.07E-72	1.03E-69	PGS000791	EFO_0005952	non-Hodgkins lymphoma	non-Hodgkins lymphoma	EFO_0005952	Cancer	Neoplasms	PGS000641	EFO_0005952	non-Hodgkins lymphoma	non-Hodgkins lymphoma	EFO_0005952	Cancer	Neoplasms
-0.7148	1.09E-72	1.05E-69	PGS002781	EFO_0004612	high density lipoprotein cholesterol measurement	high density lipoprotein cholesterol measurement	EFO_0004612	Lipid or lipoprotein measurement	Metabolic	PGS000825	EFO_0004612	high density lipoprotein cholesterol measurement	measurement	EFO_0004612	Lipid or lipoprotein measurement	Metabolic
0.7148	1.09E-72	1.05E-69	PGS003094	MONDO_0005148	type 2 diabetes mellitus	type 2 diabetes mellitus	MONDO_0005148	Digestive system disorder	Gastrointestinal	PGS003098	MONDO_0005148	type 2 diabetes mellitus	type 2 diabetes mellitus	MONDO_0005148	Digestive system disorder	Gastrointestinal
0.7148	1.09E-72	1.05E-69	PGS003094	MONDO_0005148	type 2 diabetes mellitus	type 2 diabetes mellitus	MONDO_0005148	Digestive system disorder	Gastrointestinal	PGS003098	MONDO_0005148	type 2 diabetes mellitus	type 2 diabetes mellitus	MONDO_0005148	Metabolic disorder	Metabolic
0.7148	1.09E-72	1.05E-69	PGS003094	MONDO_0005148	type 2 diabetes mellitus	type 2 diabetes mellitus	MONDO_0005148	Metabolic disorder	Metabolic	PGS003098	MONDO_0005148	type 2 diabetes mellitus	type 2 diabetes mellitus	MONDO_0005148	Metabolic disorder	Metabolic
0.7148	1.09E-72	1.05E-69	PGS003094	MONDO_0005148	type 2 diabetes mellitus	type 2 diabetes mellitus	MONDO_0005148	Metabolic disorder	Metabolic	PGS003098	MONDO_0005148	type 2 diabetes mellitus	type 2 diabetes mellitus	MONDO_0005148	Digestive system disorder	Gastrointestinal
0.7148	1.14E-72	1.10E-69	PGS002208	EFO_0007989	monocyte percentage of leukocytes	monocyte percentage of leukocytes	EFO_0007989	Hematological measurement	Hematological	PGS001968	EFO_0005091	monocyte count	monocyte count	EFO_0005091	Hematological measurement	Hematological
0.7147	1.16E-72	1.12E-69	PGS000015	EFO_0000305	breast carcinoma	breast carcinoma	EFO_0000305	Cancer	Neoplasms	PGS000335	EFO_0000305	breast carcinoma	breast carcinoma	EFO_0000305	Cancer	Neoplasms
0.7147	1.16E-72	1.12E-69	PGS000875	EFO_0004611	low density lipoprotein cholesterol measurement	low density lipoprotein cholesterol measurement	EFO_0004611	Lipid or lipoprotein measurement	Metabolic	PGS002605	EFO_0004611	low density lipoprotein cholesterol measurement	measurement	EFO_0004611	Lipid or lipoprotein measurement	Metabolic
0.7147	1.16E-72	1.12E-69	PGS000875	EFO_0004611	low density lipoprotein cholesterol measurement	low density lipoprotein cholesterol measurement	EFO_0004611	Lipid or lipoprotein measurement	Metabolic	PGS002605	EFO_0004611	low density lipoprotein cholesterol measurement	measurement	EFO_0004611	Cardiovascular measurement	Cardiovascular
0.7147	1.16E-72	1.12E-69	PGS000875	EFO_0004611	low density lipoprotein cholesterol measurement	low density lipoprotein cholesterol measurement	EFO_0004611	Cardiovascular measurement	Cardiovascular	PGS002605	EFO_0004611	low density lipoprotein cholesterol measurement	measurement	EFO_0004611	Lipid or lipoprotein measurement	Metabolic
0.7147	1.16E-72	1.12E-69	PGS000875	EFO_0004611	low density lipoprotein cholesterol measurement	low density lipoprotein cholesterol measurement	EFO_0004611	Cardiovascular measurement	Cardiovascular	PGS002605	EFO_0004611	low density lipoprotein cholesterol measurement	measurement	EFO_0004611	Cardiovascular measurement	Cardiovascular
0.7147	1.18E-72	1.14E-69	PGS000048	EFO_0001075	ovarian carcinoma	ovarian carcinoma	EFO_0001075	Cancer	Neoplasms	PGS000559	EFO_0003893	ovarian neoplasm	ovarian neoplasm	EFO_0003893	Cancer	Neoplasms
0.7147	1.18E-72	1.14E-69	PGS000048	EFO_0001075	ovarian carcinoma	ovarian carcinoma	EFO_0001075	Cancer	Neoplasms	PGS000560	EFO_0003893	ovarian neoplasm	ovarian neoplasm	EFO_0003893	Cancer	Neoplasms
0.7147	1.22E-72	1.18E-69	PGS000510	EFO_0000305	breast carcinoma	breast carcinoma	EFO_0000305	Cancer	Neoplasms	PGS000007	EFO_0000305	breast carcinoma	breast carcinoma	EFO_0000305	Cancer	Neoplasms
0.7146	1.24E-72	1.19E-69	PGS000528	EFO_0000305	breast carcinoma	breast carcinoma	EFO_0000305	Cancer	Neoplasms	PGS000508	EFO_0000305	breast carcinoma	breast carcinoma	EFO_0000305	Cancer	Neoplasms
0.7146	1.24E-72	1.20E-69	PGS001323	MONDO_0005041	glaucoma	glaucoma	MONDO_0005041	Other disease	Ophthalmological	PGS001322	MONDO_0005041	glaucoma	glaucoma	MONDO_0005041	Other disease	Ophthalmological
0.7146	1.25E-72	1.20E-69	PGS002461	EFO_0004584	mean platelet volume	mean platelet volume	EFO_0004584	Hematological measurement	Hematological	PGS002189	EFO_0004584	mean platelet volume	mean platelet volume	EFO_0004584	Hematological measurement	Hematological
0.7146	1.25E-72	1.21E-69	PGS000160	EFO_0001663	prostate carcinoma	prostate carcinoma	EFO_0001663	Cancer	Neoplasms	PGS000580	EFO_0001663	prostate carcinoma	prostate carcinoma	EFO_0001663	Cancer	Neoplasms
0.7146	1.26E-72	1.21E-69	PGS001933	EFO_0004611	low density lipoprotein cholesterol measurement	low density lipoprotein cholesterol measurement	EFO_0004611	Lipid or lipoprotein measurement	Metabolic	PGS003138	EFO_0004574	total cholesterol measurement	total cholesterol measurement	EFO_0004574	Lipid or lipoprotein measurement	Metabolic
0.7146	1.26E-72	1.21E-69	PGS001933	EFO_0004611	low density lipoprotein cholesterol measurement	low density lipoprotein cholesterol measurement	EFO_0004611	Cardiovascular measurement	Cardiovascular	PGS003138	EFO_0004574	total cholesterol measurement	total cholesterol measurement	EFO_0004574	Lipid or lipoprotein measurement	Metabolic
0.7146	1.26E-72	1.22E-69	PGS000002	EFO_1000649	estrogen-receptor positive breast cancer	estrogen-receptor positive breast cancer	EFO_1000649	Cancer	Neoplasms	PGS000530	EFO_0000305	breast carcinoma	breast carcinoma	EFO_0000305	Cancer	Neoplasms
0.7146	1.27E-72	1.22E-69	PGS002993	EFO_0004339	body height	body height	EFO_0004339	Body measurement	Body measurement	PGS002980	EFO_0004339	body height	body height	EFO_0004339	Body measurement	Body measurement
0.7146	1.30E-72	1.25E-69	PGS001662	EFO_0006930	brain volume measurement|neuroimaging measurement	brain volume measurement	EFO_0006930	Other measurement	Neurological	PGS001680	EFO_0006930	measurement	brain volume measurement	EFO_0006930	Other measurement	Neurological
0.7146	1.31E-72	1.26E-69	PGS000008	EFO_1000649	estrogen-receptor positive breast cancer	estrogen-receptor positive breast cancer	EFO_1000649	Cancer	Neoplasms	PGS000526	EFO_0000305	breast carcinoma	breast carcinoma	EFO_0000305	Cancer	Neoplasms
0.7146	1.31E-72	1.26E-69	PGS000525	EFO_0000305	breast carcinoma	breast carcinoma	EFO_0000305	Cancer	Neoplasms	PGS000008	EFO_1000649	estrogen-receptor positive breast cancer	cancer	EFO_1000649	Cancer	Neoplasms
-0.7145	1.33E-72	1.28E-69	PGS000674	EFO_0010934	ratio	ratio	EFO_0010934	Other measurement	Metabolic	PGS000668	EFO_0004735	serum alanine aminotransferase measurement	measurement	EFO_0004735	Liver enzyme measurement	Gastrointestinal
0.7145	1.36E-72	1.31E-69	PGS000703	EFO_0003913	angina pectoris	angina pectoris	EFO_0003913	Other trait	Cardiovascular	PGS001260	EFO_0003913	angina pectoris	angina pectoris	EFO_0003913	Other trait	Cardiovascular
0.7145	1.36E-72	1.31E-69	PGS003186	EFO_0004631	vitamin D measurement	vitamin D measurement	EFO_0004631	Lipid or lipoprotein measurement	Metabolic	PGS001982	EFO_0004631	vitamin D measurement	vitamin D measurement	EFO_0004631	Lipid or lipoprotein measurement	Metabolic
0.7145	1.39E-72	1.33E-69	PGS000594	EFO_0007355	male reproductive organ cancer	male reproductive organ cancer	EFO_0007355	Cancer	Neoplasms	PGS001805	EFO_0001663	prostate carcinoma	prostate carcinoma	EFO_0001663	Cancer	Neoplasms
0.7144	1.43E-72	1.37E-69	PGS000486	EFO_0000305	breast carcinoma	breast carcinoma	EFO_0000305	Cancer	Neoplasms	PGS000873	EFO_0000305	breast carcinoma	breast carcinoma	EFO_0000305	Cancer	Neoplasms
0.7144	1.44E-72	1.39E-69	PGS002850	EFO_0004340	body mass index	body mass index	EFO_0004340	Body measurement	Body measurement	PGS002630	EFO_0004340	body mass index	body mass index	EFO_0004340	Body measurement	Body measurement
0.7144	1.48E-72	1.43E-69	PGS002936	EFO_0004468	glucose measurement	glucose measurement	EFO_0004468	Other measurement	Metabolic	PGS002938	EFO_0004468	glucose measurement	glucose measurement	EFO_0004468	Other measurement	Metabolic
0.7144	1.49E-72	1.43E-69	PGS000007	EFO_0000305	breast carcinoma	breast carcinoma	EFO_0000305	Cancer	Neoplasms	PGS000505	EFO_0000305	breast carcinoma	breast carcinoma	EFO_0000305	Cancer	Neoplasms
0.7144	1.49E-72	1.43E-69	PGS000506	EFO_0000305	breast carcinoma	breast carcinoma	EFO_0000305	Cancer	Neoplasms	PGS000007	EFO_0000305	breast carcinoma	breast carcinoma	EFO_0000305	Cancer	Neoplasms
0.7144	1.49E-72	1.44E-69	PGS002556	EFO_0004611	low density lipoprotein cholesterol measurement	low density lipoprotein cholesterol measurement	EFO_0004611	Cardiovascular measurement	Cardiovascular	PGS000892	EFO_0004611	low density lipoprotein cholesterol measurement	measurement	EFO_0004611	Lipid or lipoprotein measurement	Metabolic
0.7144	1.49E-72	1.44E-69	PGS002556	EFO_0004611	low density lipoprotein cholesterol measurement	low density lipoprotein cholesterol measurement	EFO_0004611	Lipid or lipoprotein measurement	Metabolic	PGS000892	EFO_0004611	low density lipoprotein cholesterol measurement	measurement	EFO_0004611	Lipid or lipoprotein measurement	Metabolic
0.7144	1.49E-72	1.44E-69	PGS002556	EFO_0004611	low density lipoprotein cholesterol measurement	low density lipoprotein cholesterol measurement	EFO_0004611	Cardiovascular measurement	Cardiovascular	PGS000892	EFO_0004611	low density lipoprotein cholesterol measurement	measurement	EFO_0004611	Cardiovascular measurement	Cardiovascular
0.7144	1.49E-72	1.44E-69	PGS002556	EFO_0004611	low density lipoprotein cholesterol measurement	low density lipoprotein cholesterol measurement	EFO_0004611	Lipid or lipoprotein measurement	Metabolic	PGS000892	EFO_0004611	low density lipoprotein cholesterol measurement	measurement	EFO_0004611	Cardiovascular measurement	Cardiovascular
0.7143	1.51E-72	1.45E-69	PGS000169	EFO_0007986	reticulocyte count	reticulocyte count	EFO_0007986	Hematological measurement	Hematological	PGS000111	EFO_0007986	reticulocyte count	reticulocyte count	EFO_0007986	Hematological measurement	Hematological
0.7143	1.51E-72	1.45E-69	PGS001400	EFO_0004509	hemoglobin measurement	hemoglobin measurement	EFO_0004509	Hematological measurement	Hematological	PGS002141	EFO_0004348	hematocrit	hematocrit	EFO_0004348	Hematological measurement	Hematological
0.7143	1.51E-72	1.45E-69	PGS000441	MONDO_0002898	skin cancer	skin cancer	MONDO_0002898	Cancer	Neoplasms	PGS000406	EFO_0009259	skin carcinoma	skin carcinoma	EFO_0009259	Cancer	Neoplasms
0.7143	1.52E-72	1.46E-69	PGS001476	EFO_0006930	brain volume measurement|neuroimaging measurement	brain volume measurement	EFO_0006930	Other measurement	Neurological	PGS001479	EFO_0006930	measurement	brain volume measurement	EFO_0006930	Other measurement	Neurological
0.7143	1.53E-72	1.47E-69	PGS001951	EFO_0005409	fat body mass	fat body mass	EFO_0005409	Other measurement	Body measurement	PGS002859	EFO_0004340	body mass index	body mass index	EFO_0004340	Body measurement	Body measurement
0.7143	1.59E-72	1.52E-69	PGS000892	EFO_0004611	low density lipoprotein cholesterol measurement	low density lipoprotein cholesterol measurement	EFO_0004611	Lipid or lipoprotein measurement	Metabolic	PGS001889	EFO_0004615	apolipoprotein B measurement	apolipoprotein B measurement	EFO_0004615	Lipid or lipoprotein measurement	Metabolic
0.7143	1.59E-72	1.52E-69	PGS000892	EFO_0004611	low density lipoprotein cholesterol measurement	low density lipoprotein cholesterol measurement	EFO_0004611	Cardiovascular measurement	Cardiovascular	PGS001889	EFO_0004615	apolipoprotein B measurement	apolipoprotein B measurement	EFO_0004615	Lipid or lipoprotein measurement	Metabolic
0.7142	1.61E-72	1.55E-69	PGS000921	EFO_0004340	body mass index	body mass index	EFO_0004340	Body measurement	Body measurement	PGS001951	EFO_0005409	fat body mass	fat body mass	EFO_0005409	Other measurement	Body measurement
0.7142	1.67E-72	1.60E-69	PGS000113	EFO_0004308	leukocyte count	leukocyte count	EFO_0004308	Hematological measurement	Hematological	PGS001962	EFO_0004308	leukocyte count	leukocyte count	EFO_0004308	Hematological measurement	Hematological
0.7142	1.69E-72	1.62E-69	PGS002147	EFO_0009253	Immature Reticulocyte Fraction Measurement	Immature Reticulocyte Fraction Measurement	EFO_0009253	Hematological measurement	Hematological	PGS000096	EFO_0007986	reticulocyte count	reticulocyte count	EFO_0007986	Hematological measurement	Hematological
0.7142	1.70E-72	1.62E-69	PGS000824	EFO_0004611	low density lipoprotein cholesterol measurement	low density lipoprotein cholesterol measurement	EFO_0004611	Cardiovascular measurement	Cardiovascular	PGS000889	EFO_0004611	low density lipoprotein cholesterol measurement	measurement	EFO_0004611	Cardiovascular measurement	Cardiovascular
0.7142	1.70E-72	1.62E-69	PGS000824	EFO_0004611	low density lipoprotein cholesterol measurement	low density lipoprotein cholesterol measurement	EFO_0004611	Lipid or lipoprotein measurement	Metabolic	PGS000889	EFO_0004611	low density lipoprotein cholesterol measurement	measurement	EFO_0004611	Lipid or lipoprotein measurement	Metabolic
0.7142	1.70E-72	1.62E-69	PGS000824	EFO_0004611	low density lipoprotein cholesterol measurement	low density lipoprotein cholesterol measurement	EFO_0004611	Cardiovascular measurement	Cardiovascular	PGS000889	EFO_0004611	low density lipoprotein cholesterol measurement	measurement	EFO_0004611	Lipid or lipoprotein measurement	Metabolic
0.7142	1.70E-72	1.62E-69	PGS000824	EFO_0004611	low density lipoprotein cholesterol measurement	low density lipoprotein cholesterol measurement	EFO_0004611	Lipid or lipoprotein measurement	Metabolic	PGS000889	EFO_0004611	low density lipoprotein cholesterol measurement	measurement	EFO_0004611	Cardiovascular measurement	Cardiovascular
0.7141	1.78E-72	1.70E-69	PGS000515	EFO_0000305	breast carcinoma	breast carcinoma	EFO_0000305	Cancer	Neoplasms	PGS000526	EFO_0000305	breast carcinoma	breast carcinoma	EFO_0000305	Cancer	Neoplasms
0.7141	1.78E-72	1.70E-69	PGS000525	EFO_0000305	breast carcinoma	breast carcinoma	EFO_0000305	Cancer	Neoplasms	PGS000515	EFO_0000305	breast carcinoma	breast carcinoma	EFO_0000305	Cancer	Neoplasms
0.7141	1.79E-72	1.72E-69	PGS003287	EFO_0004617	cystatin C measurement	cystatin C measurement	EFO_0004617	Other measurement	Metabolic	PGS003286	EFO_0004617	cystatin C measurement	cystatin C measurement	EFO_0004617	Other measurement	Metabolic
0.7141	1.80E-72	1.72E-69	PGS002005	EFO_0011011	sitting height measurement	sitting height measurement	EFO_0011011	Other measurement	Skeletal	PGS001229	EFO_0004339	body height	body height	EFO_0004339	Body measurement	Body measurement
0.714	1.87E-72	1.79E-69	PGS001476	EFO_0006930	brain volume measurement|neuroimaging measurement	brain volume measurement	EFO_0006930	Other measurement	Neurological	PGS001672	EFO_0006930	measurement	brain volume measurement	EFO_0006930	Other measurement	Neurological

0.714	1.88E-72	1.80E-69	PGS002321	EFO_0000400	diabetes mellitus	diabetes mellitus	EFO_0000400	Digestive system disorder	Gastrointestinal	PGS002308	MONDO_0005148	type 2 diabetes mellitus	type 2 diabetes mellitus	MONDO_0005148	Digestive system disorder	Gastrointestinal
0.714	1.88E-72	1.80E-69	PGS002321	EFO_0000400	diabetes mellitus	diabetes mellitus	EFO_0000400	Digestive system disorder	Gastrointestinal	PGS002308	MONDO_0005148	type 2 diabetes mellitus	type 2 diabetes mellitus	MONDO_0005148	Metabolic disorder	Metabolic
0.714	1.88E-72	1.80E-69	PGS002321	EFO_0000400	diabetes mellitus	diabetes mellitus	EFO_0000400	Metabolic disorder	Metabolic	PGS002308	MONDO_0005148	type 2 diabetes mellitus	type 2 diabetes mellitus	MONDO_0005148	Digestive system disorder	Gastrointestinal
0.714	1.88E-72	1.80E-69	PGS002321	EFO_0000400	diabetes mellitus	diabetes mellitus	EFO_0000400	Metabolic disorder	Metabolic	PGS002308	MONDO_0005148	type 2 diabetes mellitus	type 2 diabetes mellitus	MONDO_0005148	Metabolic disorder	Metabolic
0.714	1.91E-72	1.82E-69	PGS001806	EFO_0004281	testicular neoplasm	testicular neoplasm	EFO_0004281	Other disease	Neoplasms	PGS000597	EFO_0005088	testicular carcinoma	testicular carcinoma	EFO_0005088	Cancer	Neoplasms
0.714	1.95E-72	1.86E-69	PGS002321	EFO_0000400	diabetes mellitus	diabetes mellitus	EFO_0000400	Digestive system disorder	Gastrointestinal	PGS002771	MONDO_0005148	type 2 diabetes mellitus	type 2 diabetes mellitus	MONDO_0005148	Metabolic disorder	Metabolic
0.714	1.95E-72	1.86E-69	PGS002321	EFO_0000400	diabetes mellitus	diabetes mellitus	EFO_0000400	Metabolic disorder	Metabolic	PGS002771	MONDO_0005148	type 2 diabetes mellitus	type 2 diabetes mellitus	MONDO_0005148	Metabolic disorder	Metabolic
0.714	1.95E-72	1.86E-69	PGS002321	EFO_0000400	diabetes mellitus	diabetes mellitus	EFO_0000400	Metabolic disorder	Metabolic	PGS002771	MONDO_0005148	type 2 diabetes mellitus	type 2 diabetes mellitus	MONDO_0005148	Digestive system disorder	Gastrointestinal
0.714	1.95E-72	1.86E-69	PGS002321	EFO_0000400	diabetes mellitus	diabetes mellitus	EFO_0000400	Digestive system disorder	Gastrointestinal	PGS002771	MONDO_0005148	type 2 diabetes mellitus	type 2 diabetes mellitus	MONDO_0005148	Digestive system disorder	Gastrointestinal
0.7139	1.99E-72	1.90E-69	PGS002966	EFO_0004339	body height	body height	EFO_0004339	Body measurement	Body measurement	PGS002146	EFO_0004339	body height	body height	EFO_0004339	Body measurement	Body measurement
0.7139	2.04E-72	1.95E-69	PGS002632	EFO_0003923	bone density	bone density	EFO_0003923	Other measurement	Skeletal	PGS000954	EFO_0004514	bone quantitative ultrasound measurement	measurement	EFO_0004514	Other measurement	Skeletal
0.7139	2.05E-72	1.96E-69	PGS000111	EFO_0007986	reticulocyte count	reticulocyte count	EFO_0007986	Hematological measurement	Hematological	PGS001976	EFO_0007986	reticulocyte count	reticulocyte count	EFO_0007986	Hematological measurement	Hematological
0.7139	2.08E-72	1.98E-69	PGS001753	EFO_0006930	brain volume measurement|neuroimaging measurement	brain volume measurement	EFO_0006930	Other measurement	Neurological	PGS001736	EFO_0006930	measurement	brain volume measurement	EFO_0006930	Other measurement	Neurological
0.7138	2.10E-72	2.01E-69	PGS001805	EFO_0001663	prostate carcinoma	prostate carcinoma	EFO_0001663	Cancer	Neoplasms	PGS000593	EFO_0007355	male reproductive organ cancer	male reproductive organ cancer	EFO_0007355	Cancer	Neoplasms
0.7138	2.12E-72	2.02E-69	PGS002632	EFO_0003923	bone density	bone density	EFO_0003923	Other measurement	Skeletal	PGS001038	EFO_0009270	heel bone mineral density	heel bone mineral density	EFO_0009270	Other measurement	Skeletal
0.7138	2.13E-72	2.03E-69	PGS001895	EFO_0004574	total cholesterol measurement	total cholesterol measurement	EFO_0004574	Lipid or lipoprotein measurement	Metabolic	PGS003033	EFO_0004611	low density lipoprotein cholesterol measurement	measurement	EFO_0004611	Lipid or lipoprotein measurement	Metabolic
0.7138	2.13E-72	2.03E-69	PGS001895	EFO_0004574	total cholesterol measurement	total cholesterol measurement	EFO_0004574	Lipid or lipoprotein measurement	Metabolic	PGS003033	EFO_0004611	low density lipoprotein cholesterol measurement	measurement	EFO_0004611	Cardiovascular measurement	Cardiovascular
0.7138	2.18E-72	2.08E-69	PGS000818	EFO_0001645	coronary artery disease	coronary artery disease	EFO_0001645	Cardiovascular disease	Cardiovascular	PGS000899	EFO_0001645	coronary artery disease	coronary artery disease	EFO_0001645	Cardiovascular disease	Cardiovascular
0.7138	2.19E-72	2.09E-69	PGS001130	EFO_0006525	cigarettes per day measurement	cigarettes per day measurement	EFO_0006525	Other measurement	Psychiatric	PGS001515	EFO_0006525	cigarettes per day measurement	cigarettes per day measurement	EFO_0006525	Other measurement	Psychiatric
0.7138	2.20E-72	2.10E-69	PGS001477	EFO_0006930	brain volume measurement|neuroimaging measurement	brain volume measurement	EFO_0006930	Other measurement	Neurological	PGS001472	EFO_0006930	measurement	brain volume measurement	EFO_0006930	Other measurement	Neurological
0.7136	2.40E-72	2.29E-69	PGS003031	EFO_0004611	low density lipoprotein cholesterol measurement	low density lipoprotein cholesterol measurement	EFO_0004611	Cardiovascular measurement	Cardiovascular	PGS000893	EFO_0004611	low density lipoprotein cholesterol measurement	measurement	EFO_0004611	Cardiovascular measurement	Cardiovascular
0.7136	2.40E-72	2.29E-69	PGS003031	EFO_0004611	low density lipoprotein cholesterol measurement	low density lipoprotein cholesterol measurement	EFO_0004611	Lipid or lipoprotein measurement	Metabolic	PGS000893	EFO_0004611	low density lipoprotein cholesterol measurement	measurement	EFO_0004611	Lipid or lipoprotein measurement	Metabolic
0.7136	2.40E-72	2.29E-69	PGS003031	EFO_0004611	low density lipoprotein cholesterol measurement	low density lipoprotein cholesterol measurement	EFO_0004611	Cardiovascular measurement	Cardiovascular	PGS000893	EFO_0004611	low density lipoprotein cholesterol measurement	measurement	EFO_0004611	Lipid or lipoprotein measurement	Metabolic
0.7136	2.40E-72	2.29E-69	PGS003031	EFO_0004611	low density lipoprotein cholesterol measurement	low density lipoprotein cholesterol measurement	EFO_0004611	Lipid or lipoprotein measurement	Metabolic	PGS000893	EFO_0004611	low density lipoprotein cholesterol measurement	measurement	EFO_0004611	Cardiovascular measurement	Cardiovascular
0.7136	2.41E-72	2.30E-69	PGS002854	EFO_0004340	body mass index	body mass index	EFO_0004340	Body measurement	Body measurement	PGS002841	EFO_0004340	body mass index	body mass index	EFO_0004340	Body measurement	Body measurement
0.7136	2.46E-72	2.35E-69	PGS002049	MONDO_0004596	cor pulmonale	cor pulmonale	MONDO_0004596	Cardiovascular disease	Cardiovascular	PGS002056	EFO_1001395	Phlebitis|Thrombophlebitis	Phlebitis	EFO_1001395	Cardiovascular disease	Cardiovascular
0.7136	2.51E-72	2.39E-69	PGS000507	EFO_0000305	breast carcinoma	breast carcinoma	EFO_0000305	Cancer	Neoplasms	PGS000005	EFO_1000649	estrogen-receptor positive breast cancer	cancer	EFO_1000649	Cancer	Neoplasms
0.7136	2.56E-72	2.44E-69	PGS000474	EFO_0000305	breast carcinoma	breast carcinoma	EFO_0000305	Cancer	Neoplasms	PGS001337	EFO_0005606	family history of breast cancer	family history of breast cancer	EFO_0005606	Other trait	Neoplasms
0.7135	2.60E-72	2.48E-69	PGS003150	EFO_0004530	triglyceride measurement	triglyceride measurement	EFO_0004530	Lipid or lipoprotein measurement	Metabolic	PGS002425	EFO_0004530	triglyceride measurement	triglyceride measurement	EFO_0004530	Lipid or lipoprotein measurement	Metabolic
0.7135	2.66E-72	2.53E-69	PGS001615	EFO_0005420	measurement	grey matter volume measurement	EFO_0005420	Other measurement	Neurological	PGS001572	EFO_0005420	measurement	grey matter volume measurement	EFO_0005420	Other measurement	Neurological
0.7135	2.67E-72	2.54E-69	PGS002142	EFO_0004509	hemoglobin measurement	hemoglobin measurement	EFO_0004509	Hematological measurement	Hematological	PGS000093	EFO_0004509	hemoglobin measurement	hemoglobin measurement	EFO_0004509	Hematological measurement	Hematological
0.7135	2.70E-72	2.57E-69	PGS000510	EFO_0000305	breast carcinoma	breast carcinoma	EFO_0000305	Cancer	Neoplasms	PGS000503	EFO_0000305	breast carcinoma	breast carcinoma	EFO_0000305	Cancer	Neoplasms
0.7135	2.70E-72	2.57E-69	PGS000684	EFO_0004468	glucose measurement	glucose measurement	EFO_0004468	Other measurement	Metabolic	PGS002645	EFO_0004468	glucose measurement	glucose measurement	EFO_0004468	Other measurement	Metabolic
0.7134	2.82E-72	2.69E-69	PGS003029	EFO_0004611	low density lipoprotein cholesterol measurement	low density lipoprotein cholesterol measurement	EFO_0004611	Cardiovascular measurement	Cardiovascular	PGS003037	EFO_0004611	low density lipoprotein cholesterol measurement	measurement	EFO_0004611	Lipid or lipoprotein measurement	Metabolic
0.7134	2.82E-72	2.69E-69	PGS003029	EFO_0004611	low density lipoprotein cholesterol measurement	low density lipoprotein cholesterol measurement	EFO_0004611	Lipid or lipoprotein measurement	Metabolic	PGS003037	EFO_0004611	low density lipoprotein cholesterol measurement	measurement	EFO_0004611	Cardiovascular measurement	Cardiovascular
0.7134	2.82E-72	2.69E-69	PGS003029	EFO_0004611	low density lipoprotein cholesterol measurement	low density lipoprotein cholesterol measurement	EFO_0004611	Lipid or lipoprotein measurement	Metabolic	PGS003037	EFO_0004611	low density lipoprotein cholesterol measurement	measurement	EFO_0004611	Lipid or lipoprotein measurement	Metabolic
0.7134	2.82E-72	2.69E-69	PGS003029	EFO_0004611	low density lipoprotein cholesterol measurement	low density lipoprotein cholesterol measurement	EFO_0004611	Cardiovascular measurement	Cardiovascular	PGS003037	EFO_0004611	low density lipoprotein cholesterol measurement	measurement	EFO_0004611	Cardiovascular measurement	Cardiovascular
0.7134	2.90E-72	2.76E-69	PGS000015	EFO_0000305	breast carcinoma	breast carcinoma	EFO_0000305	Cancer	Neoplasms	PGS000507	EFO_0000305	breast carcinoma	breast carcinoma	EFO_0000305	Cancer	Neoplasms
0.7134	2.92E-72	2.78E-69	PGS001220	EFO_0004526	mean corpuscular volume	mean corpuscular volume	EFO_0004526	Hematological measurement	Hematological	PGS000099	EFO_0004527	mean corpuscular hemoglobin	mean corpuscular hemoglobin	EFO_0004527	Hematological measurement	Hematological
0.7133	2.94E-72	2.80E-69	PGS002315	EFO_0003923	bone density	bone density	EFO_0003923	Other measurement	Skeletal	PGS000954	EFO_0004514	bone quantitative ultrasound measurement	measurement	EFO_0004514	Other measurement	Skeletal
-0.7133	2.99E-72	2.85E-69	PGS003253	EFO_0010472	creatine measurement|glomerular filtration rate	creatine measurement	EFO_0010472	Other measurement	Metabolic	PGS003223	EFO_0005208	glomerular filtration rate	glomerular filtration rate	EFO_0005208	Other measurement	Genitourinary
0.7133	3.02E-72	2.87E-69	PGS002315	EFO_0003923	bone density	bone density	EFO_0003923	Other measurement	Skeletal	PGS001038	EFO_0009270	heel bone mineral density	heel bone mineral density	EFO_0009270	Other measurement	Skeletal
0.7133	3.03E-72	2.88E-69	PGS001296	MONDO_0005147	type 1 diabetes mellitus	type 1 diabetes mellitus	MONDO_0005147	Metabolic disorder	Metabolic	PGS001014	EFO_0009605	pancreas disease	pancreas disease	EFO_0009605	Digestive system disorder	Gastrointestinal
0.7133	3.03E-72	2.88E-69	PGS001296	MONDO_0005147	type 1 diabetes mellitus	type 1 diabetes mellitus	MONDO_0005147	Digestive system disorder	Gastrointestinal	PGS001014	EFO_0009605	pancreas disease	pancreas disease	EFO_0009605	Digestive system disorder	Gastrointestinal
0.7133	3.03E-72	2.88E-69	PGS001296	MONDO_0005147	type 1 diabetes mellitus	type 1 diabetes mellitus	MONDO_0005147	Immune system disorder	Immunological	PGS001014	EFO_0009605	pancreas disease	pancreas disease	EFO_0009605	Digestive system disorder	Gastrointestinal
0.7133	3.07E-72	2.92E-69	PGS000955	EFO_0004514	bone quantitative ultrasound measurement	bone quantitative ultrasound measurement	EFO_0004514	Other measurement	Skeletal	PGS001037	EFO_0009270	heel bone mineral density	heel bone mineral density	EFO_0009270	Other measurement	Skeletal
-0.7133	3.09E-72	2.93E-69	PGS000883	EFO_0005208	glomerular filtration rate	glomerular filtration rate	EFO_0005208	Other measurement	Genitourinary	PGS003224	EFO_0005208	glomerular filtration rate	glomerular filtration rate	EFO_0005208	Other measurement	Genitourinary
-0.7132	3.16E-72	3.00E-69	PGS000471	MONDO_0004641	skin carcinoma in situ	skin carcinoma in situ	MONDO_0004641	Cancer	Neoplasms	PGS001245	EFO_0004279	suntan	suntan	EFO_0004279	Other trait	Dermatological
0.7132	3.18E-72	3.02E-69	PGS000955	EFO_0004514	bone quantitative ultrasound measurement	bone quantitative ultrasound measurement	EFO_0004514	Other measurement	Skeletal	PGS000952	EFO_0004514	bone quantitative ultrasound measurement	measurement	EFO_0004514	Other measurement	Skeletal
0.7132	3.21E-72	3.05E-69	PGS000525	EFO_0000305	breast carcinoma	breast carcinoma	EFO_0000305	Cancer	Neoplasms	PGS000001	EFO_0000305	breast carcinoma	breast carcinoma	EFO_0000305	Cancer	Neoplasms
0.7132	3.21E-72	3.05E-69	PGS000001	EFO_0000305	breast carcinoma	breast carcinoma	EFO_0000305	Cancer	Neoplasms	PGS000526	EFO_0000305	breast carcinoma	breast carcinoma	EFO_0000305	Cancer	Neoplasms
0.7132	3.21E-72	3.05E-69	PGS001451	EFO_0006930	brain volume measurement|neuroimaging measurement	brain volume measurement	EFO_0006930	Other measurement	Neurological	PGS001454	EFO_0006930	measurement	brain volume measurement	EFO_0006930	Other measurement	Neurological
0.7132	3.22E-72	3.05E-69	PGS001840	MONDO_0004596	cor pulmonale	cor pulmonale	MONDO_0004596	Cardiovascular disease	Cardiovascular	PGS002056	EFO_1001395	Phlebitis|Thrombophlebitis	Phlebitis	EFO_1001395	Cardiovascular disease	Cardiovascular
0.7131	3.38E-72	3.21E-69	PGS000534	EFO_0000305	breast carcinoma	breast carcinoma	EFO_0000305	Cancer	Neoplasms	PGS001804	EFO_0000305	breast carcinoma	breast carcinoma	EFO_0000305	Cancer	Neoplasms
0.7131	3.38E-72	3.21E-69	PGS000533	EFO_0000305	breast carcinoma	breast carcinoma	EFO_0000305	Cancer	Neoplasms	PGS001804	EFO_0000305	breast carcinoma	breast carcinoma	EFO_0000305	Cancer	Neoplasms
0.7131	3.38E-72	3.21E-69	PGS000090	EFO_0004842	eosinophil count	eosinophil count	EFO_0004842	Hematological measurement	Hematological	PGS001076	EFO_0007991	eosinophil percentage of leukocytes	eosinophil percentage of leukocytes	EFO_0007991	Hematological measurement	Hematological
0.7131	3.41E-72	3.23E-69	PGS002856	EFO_0004340	body mass index	body mass index	EFO_0004340	Body measurement	Body measurement	PGS002859	EFO_0004340	body mass index	body mass index	EFO_0004340	Body measurement	Body measurement
0.7131	3.46E-72	3.28E-69	PGS002846	EFO_0004340	body mass index	body mass index	EFO_0004340	Body measurement	Body measurement	PGS002849	EFO_0004340	body mass index	body mass index	EFO_0004340	Body measurement	Body measurement
0.7131	3.48E-72	3.30E-69	PGS001753	EFO_0006930	brain volume measurement|neuroimaging measurement	brain volume measurement	EFO_0006930	Other measurement	Neurological	PGS001722	EFO_0006930	measurement	brain volume measurement	EFO_0006930	Other measurement	Neurological
0.713	3.76E-72	3.57E-69	PGS002473	EFO_0004574	total cholesterol measurement	total cholesterol measurement	EFO_0004574	Lipid or lipoprotein measurement	Metabolic	PGS002620	EFO_0004574	total cholesterol measurement	total cholesterol measurement	EFO_0004574	Lipid or lipoprotein measurement	Metabolic
0.713	3.77E-72	3.57E-69	PGS003380	EFO_0000305	breast carcinoma	breast carcinoma	EFO_0000305	Cancer	Neoplasms	PGS000007	EFO_0000305	breast carcinoma	breast carcinoma	EFO_0000305	Cancer	Neoplasms
0.713	3.80E-72	3.60E-69	PGS002783	EFO_0004574	total cholesterol measurement	total cholesterol measurement	EFO_0004574	Lipid or lipoprotein measurement	Metabolic	PGS002782	EFO_0005689	measurement	measurement	EFO_0005689	Lipid or lipoprotein measurement	Metabolic
0.713	3.80E-72	3.60E-69	PGS002783	EFO_0004574	total cholesterol measurement	total cholesterol measurement	EFO_0004574	Lipid or lipoprotein measurement	Metabolic	PGS002782	EFO_0005689	measurement	measurement	EFO_0005689	Cardiovascular measurement	Cardiovascular
0.7129	3.84E-72	3.64E-69	PGS002851	EFO_0004340	body mass index	body mass index	EFO_0004340	Body measurement	Body measurement	PGS002845	EFO_0004340	body mass index	body mass index	EFO_0004340	Body measurement	Body measurement
0.7129	3.89E-72	3.68E-69	PGS003228	EFO_0005208	glomerular filtration rate	glomerular filtration rate	EFO_0005208	Other measurement	Genitourinary	PGS003222	EFO_0005208	glomerular filtration rate	glomerular filtration rate	EFO_0005208	Other measurement	Genitourinary
0.7129	3.96E-72	3.75E-69	PGS001684	EFO_0006930	brain volume measurement|neuroimaging measurement	brain volume measurement	EFO_0006930	Other measurement	Neurological	PGS001484	EFO_0006930	measurement	brain volume measurement	EFO_0006930	Other measurement	Neurological
0.7129	3.96E-72	3.75E-69	PGS002831	EFO_0007878	alcohol consumption measurement	alcohol consumption measurement	EFO_0007878	Other measurement	Psychiatric	PGS002837	EFO_0007878	alcohol consumption measurement	alcohol consumption measurement	EFO_0007878	Other measurement	Psychiatric
0.7129	4.03E-72	3.82E-69	PGS002315	EFO_0003923	bone density	bone density	EFO_0003923	Other measurement	Skeletal	PGS001404	EFO_0009270	heel bone mineral density	heel bone mineral density	EFO_0009270	Other measurement	Skeletal
0.7128	4.07E-72	3.85E-69	PGS002651	HP_0003124	Hypercholesterolemia	Hypercholesterolemia	HP_0003124	Other trait	Metabolic	PGS002654	EFO_0004611	low density lipoprotein cholesterol measurement	measurement	EFO_0004611	Cardiovascular measurement	Cardiovascular
0.7128	4.07E-72	3.85E-69	PGS002651	HP_0003124	Hypercholesterolemia	Hypercholesterolemia	HP_0003124	Other trait	Metabolic	PGS002654	EFO_0004611	low density lipoprotein cholesterol measurement	measurement	EFO_0004611	Lipid or lipoprotein measurement	Metabolic
0.7128	4.15E-72	3.93E-69	PGS003035	EFO_0004611	low density lipoprotein cholesterol measurement	low density lipoprotein cholesterol measurement	EFO_0004611	Lipid or lipoprotein measurement	Metabolic	PGS000893	EFO_0004611	low density lipoprotein cholesterol measurement	measurement	EFO_0004611	Cardiovascular measurement	Cardiovascular
0.7128	4.15E-72	3.93E-69	PGS003035	EFO_0004611	low density lipoprotein cholesterol measurement	low density lipoprotein cholesterol measurement	EFO_0004611	Cardiovascular measurement	Cardiovascular	PGS000893	EFO_0004611	low density lipoprotein cholesterol measurement	measurement	EFO_0004611	Lipid or lipoprotein measurement	Metabolic
0.7128	4.15E-72	3.93E-69	PGS003035	EFO_0004611	low density lipoprotein cholesterol measurement	low density lipoprotein cholesterol measurement	EFO_0004611	Cardiovascular measurement	Cardiovascular	PGS000893	EFO_0004611	low density lipoprotein cholesterol measurement	measurement	EFO_0004611	Cardiovascular measurement	Cardiovascular
0.7128	4.15E-72	3.93E-69	PGS003035	EFO_0004611	low density lipoprotein cholesterol measurement	low density lipoprotein cholesterol measurement	EFO_0004611	Lipid or lipoprotein measurement	Metabolic	PGS000893	EFO_0004611	low density lipoprotein cholesterol measurement	measurement	EFO_0004611	Lipid or lipoprotein measurement	Metabolic
0.7128	4.15E-72	3.93E-69	PGS000594	EFO_0007355	male reproductive organ cancer	male reproductive organ cancer	EFO_0007355	Cancer	Neoplasms	PGS000573	EFO_0001663	prostate carcinoma	prostate carcinoma	EFO_0001663	Cancer	Neoplasms
0.7128	4.21E-72	3.98E-69	PGS002108	EFO_0004574	total cholesterol measurement	total cholesterol measurement	EFO_0004574	Lipid or lipoprotein measurement	Metabolic	PGS003033	EFO_0004611	low density lipoprotein cholesterol measurement	measurement	EFO_0004611	Lipid or lipoprotein measurement	Metabolic
0.7128	4.21E-72	3.98E-69	PGS002108	EFO_0004574	total cholesterol measurement	total cholesterol measurement	EFO_0004574	Lipid or lipoprotein measurement	Metabolic	PGS003033	EFO_0004611	low density lipoprotein cholesterol measurement	measurement	EFO_0004611	Cardiovascular measurement	Cardiovascular
0.7128	4.30E-72	4.06E-69	PGS003193	EFO_0004631	vitamin D measurement	vitamin D measurement	EFO_0004631	Lipid or lipoprotein measurement	Metabolic	PGS003190	EFO_0004631	vitamin D measurement	vitamin D measurement	EFO_0004631	Lipid or lipoprotein measurement	Metabolic
-0.7128	4.30E-72	4.07E-69	PGS003268	EFO_0010472	creatine measurement|glomerular filtration rate	creatine measurement	EFO_0010472	Other measurement	Metabolic	PGS003223	EFO_0005208	glomerular filtration rate	glomerular filtration rate	EFO_0005208	Other measurement	Genitourinary
0.7128	4.32E-72	4.09E-69	PGS002916	EFO_0004697	estradiol measurement	estradiol measurement	EFO_0004697	Other measurement	Endocrine	PGS002915	EFO_0004697	estradiol measurement	estradiol measurement	EFO_0004697	Other measurement	Endocrine
0.7127	4.39E-72	4.15E-69	PGS002981	EFO_0004339	body height	body height	EFO_0004339	Body measurement	Body measurement	PGS002983	EFO_0004339	body height	body height	EFO_0004339	Body measurement	Body measurement
0.7127	4.41E-72	4.17E-69	PGS003033	EFO_0004611	low density lipoprotein cholesterol measurement	low density lipoprotein cholesterol measurement	EFO_0004611	Lipid or lipoprotein measurement	Metabolic	PGS001889	EFO_0004615	apolipoprotein B measurement	apolipoprotein B measurement	EFO_0004615	Lipid or lipoprotein measurement	Metabolic
0.7127	4.41E-72	4.17E-69	PGS003033	EFO_0004611	low density lipoprotein cholesterol measurement	low density lipoprotein cholesterol measurement	EFO_0004611	Cardiovascular measurement	Cardiovascular	PGS001889	EFO_0004615	apolipoprotein B measurement	apolipoprotein B measurement	EFO_0004615	Lipid or lipoprotein measurement	Metabolic
0.7127	4.47E-72	4.22E-69	PGS000310	EFO_0004611	low density lipoprotein cholesterol measurement	low density lipoprotein cholesterol measurement	EFO_0004611	Lipid or lipoprotein measurement	Metabolic	PGS000897	EFO_0004611	low density lipoprotein cholesterol measurement	measurement	EFO_0004611	Lipid or lipoprotein measurement	Metabolic
0.7127	4.47E-72	4.22E-69	PGS000310	EFO_0004611	low density lipoprotein cholesterol measurement	low density lipoprotein cholesterol measurement	EFO_0004611	Cardiovascular measurement	Cardiovascular	PGS000897	EFO_0004611	low density lipoprotein cholesterol measurement	measurement	EFO_0004611	Cardiovascular measurement	Cardiovascular
0.7127	4.47E-72	4.22E-69	PGS000310	EFO_0004611	low density lipoprotein cholesterol measurement	low density lipoprotein cholesterol measurement	EFO_0004611	Cardiovascular measurement	Cardiovascular	PGS000897	EFO_0004611	low density lipoprotein cholesterol measurement	measurement	EFO_0004611	Lipid or lipoprotein measurement	Metabolic
0.7127	4.47E-72	4.22E-69	PGS000310	EFO_0004611	low density lipoprotein cholesterol measurement	low density lipoprotein cholesterol measurement	EFO_0004611	Lipid or lipoprotein measurement	Metabolic	PGS000897	EFO_0004611	low density lipoprotein cholesterol measurement	measurement	EFO_0004611	Cardiovascular measurement	Cardiovascular
0.7126	4.80E-72	4.54E-69	PGS000539	EFO_0000305	breast carcinoma	breast carcinoma	EFO_0000305	Cancer	Neoplasms	PGS000002	EFO_1000649	estrogen-receptor positive breast cancer	cancer	EFO_1000649	Cancer	Neoplasms
0.7126	4.81E-72	4.55E-69	PGS000539	EFO_0000305	breast carcinoma	breast carcinoma	EFO_0000305	Cancer	Neoplasms	PGS000873	EFO_0000305	breast carcinoma	breast carcinoma	EFO_0000305	Cancer	Neoplasms
0.7126	4.84E-72	4.57E-69	PGS000002	EFO_1000649	estrogen-receptor positive breast cancer	estrogen-receptor positive breast cancer	EFO_1000649	Cancer	Neoplasms	PGS001778	EFO_0000305	breast carcinoma	breast carcinoma	EFO_0000305	Cancer	Neoplasms
0.7126	4.94E-72	4.66E-69	PGS002413	EFO_0005091	monocyte count	monocyte count	EFO_0005091	Hematological measurement	Hematological	PGS002341	EFO_0005091	monocyte count	monocyte count	EFO_0005091	Hematological measurement	Hematological
0.7126	4.96E-72	4.68E-69	PGS001670	EFO_0006930	brain volume measurement|neuroimaging measurement	brain volume measurement	EFO_0006930	Other measurement	Neurological	PGS001454	EFO_0006930	measurement	brain volume measurement	EFO_0006930	Other measurement	Neurological
0.7125	4.98E-72	4.70E-69	PGS002108	EFO_0004574	total cholesterol measurement	total cholesterol measurement	EFO_0004574	Lipid or lipoprotein measurement	Metabolic	PGS003038	EFO_0004611	low density lipoprotein cholesterol measurement	measurement	EFO_0004611	Cardiovascular measurement	Cardiovascular
0.7125	4.98E-72	4.70E-69	PGS002108	EFO_0004574	total cholesterol measurement	total cholesterol measurement	EFO_0004574	Lipid or lipoprotein measurement	Metabolic	PGS003038	EFO_0004611	low density lipoprotein cholesterol measurement	measurement	EFO_0004611	Lipid or lipoprotein measurement	Metabolic
0.7125	4.99E-72	4.71E-69	PGS000088	EFO_0005090	basophil count	basophil count	EFO_0005090	Hematological measurement	Hematological	PGS000089	EFO_0007992	basophil percentage of leukocytes	basophil percentage of leukocytes	EFO_0007992	Inflammatory measurement	Immunological
0.7125	4.99E-72	4.71E-69	PGS000088	EFO_0005090	basophil count	basophil count	EFO_0005090	Inflammatory measurement	Immunological	PGS000089	EFO_0007992	basophil percentage of leukocytes	basophil percentage of leukocytes	EFO_0007992	Hematological measurement	Hematological
0.7125	4.99E-72	4.71E-69	PGS000088	EFO_0005090	basophil count	basophil count	EFO_0005090	Hematological measurement	Hematological	PGS000089	EFO_0007992	basophil percentage of leukocytes	basophil percentage of leukocytes	EFO_0007992	Hematological measurement	Hematological
0.7125	4.99E-72	4.71E-69	PGS000088	EFO_0005090	basophil count	basophil count	EFO_0005090	Inflammatory measurement	Immunological	PGS000089	EFO_0007992	basophil percentage of leukocytes	basophil percentage of leukocytes	EFO_0007992	Inflammatory measurement	Immunological
0.7125	4.99E-72	4.71E-69	PGS003019	EFO_0000537	hypertension	hypertension	EFO_0000537	Cardiovascular disease	Cardiovascular	PGS003024	EFO_0000537	hypertension	hypertension	EFO_0000537	Cardiovascular disease	Cardiovascular
0.7125	5.00E-72	4.72E-69	PGS000005	EFO_1000649	estrogen-receptor positive breast cancer	estrogen-receptor positive breast cancer	EFO_1000649	Cancer	Neoplasms	PGS000052	EFO_0000305	breast carcinoma	breast carcinoma	EFO_0000305	Cancer	Neoplasms
0.7125	5.24E-72	4.94E-69	PGS002605	EFO_0004611	low density lipoprotein cholesterol measurement	low density lipoprotein cholesterol measurement	EFO_0004611	Lipid or lipoprotein measurement	Metabolic	PGS003038	EFO_0004611	low density lipoprotein cholesterol measurement	measurement	EFO_0004611	Cardiovascular measurement	Cardiovascular
0.7125	5.24E-72	4.94E-69	PGS002605	EFO_0004611	low density lipoprotein cholesterol measurement	low density lipoprotein cholesterol measurement	EFO_0004611	Lipid or lipoprotein measurement	Metabolic	PGS003038	EFO_0004611	low density lipoprotein cholesterol measurement	measurement	EFO_0004611	Lipid or lipoprotein measurement	Metabolic
0.7125	5.24E-72	4.94E-69	PGS002605	EFO_0004611	low density lipoprotein cholesterol measurement	low density lipoprotein cholesterol measurement	EFO_0004611	Cardiovascular measurement	Cardiovascular	PGS003038	EFO_0004611	low density lipoprotein cholesterol measurement	measurement	EFO_0004611	Cardiovascular measurement	Cardiovascular

0.7125	5.24E-72	4.94E-69	PGS002605	EFO_0004611	low density lipoprotein cholesterol measurement	low density lipoprotein cholesterol measurement	EFO_0004611	Cardiovascular measurement	Cardiovascular	PGS003038	EFO_0004611	low density lipoprotein cholesterol measurement	measurement	EFO_0004611	Lipid or lipoprotein measurement	Metabolic
0.7124	5.32E-72	5.02E-69	PGS000352	EFO_1001958	high grade ovarian serous adenocarcinoma	high grade ovarian serous adenocarcinoma	EFO_1001958	Cancer	Neoplasms	PGS000551	EFO_0003893	ovarian neoplasm	ovarian neoplasm	EFO_0003893	Cancer	Neoplasms
0.7124	5.36E-72	5.05E-69	PGS001229	EFO_0004339	body height	body height	EFO_0004339	Body measurement	Body measurement	PGS002224	EFO_0011011	sitting height measurement	sitting height measurement	EFO_0011011	Other measurement	Skeletal
0.7124	5.37E-72	5.06E-69	PGS001895	EFO_0004574	total cholesterol measurement	total cholesterol measurement	EFO_0004574	Lipid or lipoprotein measurement	Metabolic	PGS003038	EFO_0004611	low density lipoprotein cholesterol measurement	measurement	EFO_0004611	Cardiovascular measurement	Cardiovascular
0.7124	5.37E-72	5.06E-69	PGS001895	EFO_0004574	total cholesterol measurement	total cholesterol measurement	EFO_0004574	Lipid or lipoprotein measurement	Metabolic	PGS003038	EFO_0004611	low density lipoprotein cholesterol measurement	measurement	EFO_0004611	Lipid or lipoprotein measurement	Metabolic
0.7124	5.51E-72	5.20E-69	PGS003108	MONDO_0005148	type 2 diabetes mellitus	type 2 diabetes mellitus	MONDO_0005148	Metabolic disorder	Metabolic	PGS002771	MONDO_0005148	type 2 diabetes mellitus	type 2 diabetes mellitus	MONDO_0005148	Metabolic disorder	Metabolic
0.7124	5.51E-72	5.20E-69	PGS003108	MONDO_0005148	type 2 diabetes mellitus	type 2 diabetes mellitus	MONDO_0005148	Digestive system disorder	Gastrointestinal	PGS002771	MONDO_0005148	type 2 diabetes mellitus	type 2 diabetes mellitus	MONDO_0005148	Digestive system disorder	Gastrointestinal
0.7124	5.51E-72	5.20E-69	PGS003108	MONDO_0005148	type 2 diabetes mellitus	type 2 diabetes mellitus	MONDO_0005148	Metabolic disorder	Metabolic	PGS002771	MONDO_0005148	type 2 diabetes mellitus	type 2 diabetes mellitus	MONDO_0005148	Digestive system disorder	Gastrointestinal
0.7124	5.51E-72	5.20E-69	PGS003108	MONDO_0005148	type 2 diabetes mellitus	type 2 diabetes mellitus	MONDO_0005148	Digestive system disorder	Gastrointestinal	PGS002771	MONDO_0005148	type 2 diabetes mellitus	type 2 diabetes mellitus	MONDO_0005148	Metabolic disorder	Metabolic
0.7124	5.58E-72	5.26E-69	PGS002792	EFO_0001663	prostate carcinoma	prostate carcinoma	EFO_0001663	Cancer	Neoplasms	PGS002791	EFO_0001663	prostate carcinoma	prostate carcinoma	EFO_0001663	Cancer	Neoplasms
0.7124	5.62E-72	5.29E-69	PGS002939	EFO_0004468	glucose measurement	glucose measurement	EFO_0004468	Other measurement	Metabolic	PGS002943	EFO_0004468	glucose measurement	glucose measurement	EFO_0004468	Other measurement	Metabolic
0.7123	5.69E-72	5.36E-69	PGS002605	EFO_0004611	low density lipoprotein cholesterol measurement	low density lipoprotein cholesterol measurement	EFO_0004611	Cardiovascular measurement	Cardiovascular	PGS003033	EFO_0004611	low density lipoprotein cholesterol measurement	measurement	EFO_0004611	Cardiovascular measurement	Cardiovascular
0.7123	5.69E-72	5.36E-69	PGS002605	EFO_0004611	low density lipoprotein cholesterol measurement	low density lipoprotein cholesterol measurement	EFO_0004611	Cardiovascular measurement	Cardiovascular	PGS003033	EFO_0004611	low density lipoprotein cholesterol measurement	measurement	EFO_0004611	Lipid or lipoprotein measurement	Metabolic
0.7123	5.69E-72	5.36E-69	PGS002605	EFO_0004611	low density lipoprotein cholesterol measurement	low density lipoprotein cholesterol measurement	EFO_0004611	Lipid or lipoprotein measurement	Metabolic	PGS003033	EFO_0004611	low density lipoprotein cholesterol measurement	measurement	EFO_0004611	Cardiovascular measurement	Cardiovascular
0.7123	5.69E-72	5.36E-69	PGS002605	EFO_0004611	low density lipoprotein cholesterol measurement	low density lipoprotein cholesterol measurement	EFO_0004611	Lipid or lipoprotein measurement	Metabolic	PGS003033	EFO_0004611	low density lipoprotein cholesterol measurement	measurement	EFO_0004611	Lipid or lipoprotein measurement	Metabolic
0.7123	5.75E-72	5.41E-69	PGS000013	EFO_0001645	coronary artery disease	coronary artery disease	EFO_0001645	Cardiovascular disease	Cardiovascular	PGS000746	EFO_0001645	coronary artery disease	coronary artery disease	EFO_0001645	Cardiovascular disease	Cardiovascular
0.7123	5.85E-72	5.50E-69	PGS002437	EFO_0000319	cardiovascular disease	cardiovascular disease	EFO_0000319	Cardiovascular disease	Cardiovascular	PGS002486	EFO_0000319	cardiovascular disease	cardiovascular disease	EFO_0000319	Cardiovascular disease	Cardiovascular
0.7123	5.87E-72	5.52E-69	PGS000824	EFO_0004611	low density lipoprotein cholesterol measurement	low density lipoprotein cholesterol measurement	EFO_0004611	Lipid or lipoprotein measurement	Metabolic	PGS003038	EFO_0004611	low density lipoprotein cholesterol measurement	measurement	EFO_0004611	Lipid or lipoprotein measurement	Metabolic
0.7123	5.87E-72	5.52E-69	PGS000824	EFO_0004611	low density lipoprotein cholesterol measurement	low density lipoprotein cholesterol measurement	EFO_0004611	Lipid or lipoprotein measurement	Metabolic	PGS003038	EFO_0004611	low density lipoprotein cholesterol measurement	measurement	EFO_0004611	Cardiovascular measurement	Cardiovascular
0.7123	5.87E-72	5.52E-69	PGS000824	EFO_0004611	low density lipoprotein cholesterol measurement	low density lipoprotein cholesterol measurement	EFO_0004611	Cardiovascular measurement	Cardiovascular	PGS003038	EFO_0004611	low density lipoprotein cholesterol measurement	measurement	EFO_0004611	Cardiovascular measurement	Cardiovascular
0.7123	5.87E-72	5.52E-69	PGS000824	EFO_0004611	low density lipoprotein cholesterol measurement	low density lipoprotein cholesterol measurement	EFO_0004611	Cardiovascular measurement	Cardiovascular	PGS003038	EFO_0004611	low density lipoprotein cholesterol measurement	measurement	EFO_0004611	Lipid or lipoprotein measurement	Metabolic
0.7123	5.95E-72	5.60E-69	PGS002308	MONDO_0005148	type 2 diabetes mellitus	type 2 diabetes mellitus	MONDO_0005148	Digestive system disorder	Gastrointestinal	PGS002354	MONDO_0005148	type 2 diabetes mellitus	type 2 diabetes mellitus	MONDO_0005148	Digestive system disorder	Gastrointestinal
0.7123	5.95E-72	5.60E-69	PGS002308	MONDO_0005148	type 2 diabetes mellitus	type 2 diabetes mellitus	MONDO_0005148	Digestive system disorder	Gastrointestinal	PGS002354	MONDO_0005148	type 2 diabetes mellitus	type 2 diabetes mellitus	MONDO_0005148	Metabolic disorder	Metabolic
0.7123	5.95E-72	5.60E-69	PGS002308	MONDO_0005148	type 2 diabetes mellitus	type 2 diabetes mellitus	MONDO_0005148	Metabolic disorder	Metabolic	PGS002354	MONDO_0005148	type 2 diabetes mellitus	type 2 diabetes mellitus	MONDO_0005148	Digestive system disorder	Gastrointestinal
0.7123	5.95E-72	5.60E-69	PGS002308	MONDO_0005148	type 2 diabetes mellitus	type 2 diabetes mellitus	MONDO_0005148	Metabolic disorder	Metabolic	PGS002354	MONDO_0005148	type 2 diabetes mellitus	type 2 diabetes mellitus	MONDO_0005148	Metabolic disorder	Metabolic
0.7123	5.98E-72	5.63E-69	PGS002556	EFO_0004611	low density lipoprotein cholesterol measurement	low density lipoprotein cholesterol measurement	EFO_0004611	Lipid or lipoprotein measurement	Metabolic	PGS001889	EFO_0004615	apolipoprotein B measurement	apolipoprotein B measurement	EFO_0004615	Lipid or lipoprotein measurement	Metabolic
0.7123	5.98E-72	5.63E-69	PGS002556	EFO_0004611	low density lipoprotein cholesterol measurement	low density lipoprotein cholesterol measurement	EFO_0004611	Cardiovascular measurement	Cardiovascular	PGS001889	EFO_0004615	apolipoprotein B measurement	apolipoprotein B measurement	EFO_0004615	Lipid or lipoprotein measurement	Metabolic
0.7123	6.00E-72	5.64E-69	PGS002568	EFO_0006335	systolic blood pressure	systolic blood pressure	EFO_0006335	Other measurement	Cardiovascular	PGS002394	EFO_0006336	diastolic blood pressure	diastolic blood pressure	EFO_0006336	Other measurement	Cardiovascular
0.7123	6.00E-72	5.65E-69	PGS001340	EFO_0000275	atrial fibrillation	atrial fibrillation	EFO_0000275	Cardiovascular disease	Cardiovascular	PGS002050	EFO_0003911	atrial flutter|atrial fibrillation	atrial flutter	EFO_0003911	Cardiovascular disease	Cardiovascular
0.7122	6.23E-72	5.86E-69	PGS002516	EFO_0005192	red blood cell distribution width	red blood cell distribution width	EFO_0005192	Hematological measurement	Hematological	PGS002418	EFO_0005192	red blood cell distribution width	red blood cell distribution width	EFO_0005192	Hematological measurement	Hematological
0.7122	6.37E-72	5.99E-69	PGS000400	EFO_0009259	skin carcinoma	skin carcinoma	EFO_0009259	Cancer	Neoplasms	PGS000443	MONDO_0002898	skin cancer	skin cancer	MONDO_0002898	Cancer	Neoplasms
0.7121	6.67E-72	6.27E-69	PGS000423	EFO_0000756	melanoma	melanoma	EFO_0000756	Cancer	Neoplasms	PGS000079	EFO_0000756	melanoma	melanoma	EFO_0000756	Cancer	Neoplasms
0.7121	6.85E-72	6.44E-69	PGS002757	EFO_0003884	chronic kidney disease	chronic kidney disease	EFO_0003884	Other disease	Metabolic	PGS003227	EFO_0005208	glomerular filtration rate	glomerular filtration rate	EFO_0005208	Other measurement	Genitourinary
0.712	6.94E-72	6.52E-69	PGS002900	EFO_0006336	diastolic blood pressure	diastolic blood pressure	EFO_0006336	Other measurement	Cardiovascular	PGS002901	EFO_0006336	diastolic blood pressure	diastolic blood pressure	EFO_0006336	Other measurement	Cardiovascular
0.712	7.11E-72	6.68E-69	PGS003196	EFO_0004631	vitamin D measurement	vitamin D measurement	EFO_0004631	Lipid or lipoprotein measurement	Metabolic	PGS001982	EFO_0004631	vitamin D measurement	vitamin D measurement	EFO_0004631	Lipid or lipoprotein measurement	Metabolic
0.7119	7.69E-72	7.22E-69	PGS001819	EFO_0003770	diabetic retinopathy	diabetic retinopathy	EFO_0003770	Cardiovascular disease	Cardiovascular	PGS001296	MONDO_0005147	type 1 diabetes mellitus	type 1 diabetes mellitus	MONDO_0005147	Digestive system disorder	Gastrointestinal
0.7119	7.69E-72	7.22E-69	PGS001819	EFO_0003770	diabetic retinopathy	diabetic retinopathy	EFO_0003770	Cardiovascular disease	Cardiovascular	PGS001296	MONDO_0005147	type 1 diabetes mellitus	type 1 diabetes mellitus	MONDO_0005147	Immune system disorder	Immunological
0.7119	7.69E-72	7.22E-69	PGS001819	EFO_0003770	diabetic retinopathy	diabetic retinopathy	EFO_0003770	Cardiovascular disease	Cardiovascular	PGS001296	MONDO_0005147	type 1 diabetes mellitus	type 1 diabetes mellitus	MONDO_0005147	Metabolic disorder	Metabolic
0.7119	7.69E-72	7.22E-69	PGS001819	EFO_0003770	diabetic retinopathy	diabetic retinopathy	EFO_0003770	Metabolic disorder	Metabolic	PGS001296	MONDO_0005147	type 1 diabetes mellitus	type 1 diabetes mellitus	MONDO_0005147	Digestive system disorder	Gastrointestinal
0.7119	7.69E-72	7.22E-69	PGS001819	EFO_0003770	diabetic retinopathy	diabetic retinopathy	EFO_0003770	Metabolic disorder	Metabolic	PGS001296	MONDO_0005147	type 1 diabetes mellitus	type 1 diabetes mellitus	MONDO_0005147	Immune system disorder	Immunological
0.7119	7.69E-72	7.22E-69	PGS001819	EFO_0003770	diabetic retinopathy	diabetic retinopathy	EFO_0003770	Metabolic disorder	Metabolic	PGS001296	MONDO_0005147	type 1 diabetes mellitus	type 1 diabetes mellitus	MONDO_0005147	Metabolic disorder	Metabolic
0.7119	7.69E-72	7.22E-69	PGS001819	EFO_0003770	diabetic retinopathy	diabetic retinopathy	EFO_0003770	Neurological disorder	Neurological	PGS001296	MONDO_0005147	type 1 diabetes mellitus	type 1 diabetes mellitus	MONDO_0005147	Digestive system disorder	Gastrointestinal
0.7119	7.69E-72	7.22E-69	PGS001819	EFO_0003770	diabetic retinopathy	diabetic retinopathy	EFO_0003770	Neurological disorder	Neurological	PGS001296	MONDO_0005147	type 1 diabetes mellitus	type 1 diabetes mellitus	MONDO_0005147	Immune system disorder	Immunological
0.7119	7.69E-72	7.22E-69	PGS001819	EFO_0003770	diabetic retinopathy	diabetic retinopathy	EFO_0003770	Neurological disorder	Neurological	PGS001296	MONDO_0005147	type 1 diabetes mellitus	type 1 diabetes mellitus	MONDO_0005147	Metabolic disorder	Metabolic
0.7119	7.77E-72	7.29E-69	PGS000538	EFO_0000305	breast carcinoma	breast carcinoma	EFO_0000305	Cancer	Neoplasms	PGS000873	EFO_0000305	breast carcinoma	breast carcinoma	EFO_0000305	Cancer	Neoplasms
0.7119	7.86E-72	7.38E-69	PGS002846	EFO_0004340	body mass index	body mass index	EFO_0004340	Body measurement	Body measurement	PGS002859	EFO_0004340	body mass index	body mass index	EFO_0004340	Body measurement	Body measurement
0.7118	7.88E-72	7.40E-69	PGS003185	EFO_0004631	vitamin D measurement	vitamin D measurement	EFO_0004631	Lipid or lipoprotein measurement	Metabolic	PGS002574	EFO_0004631	vitamin D measurement	vitamin D measurement	EFO_0004631	Lipid or lipoprotein measurement	Metabolic
0.7118	8.12E-72	7.62E-69	PGS002993	EFO_0004339	body height	body height	EFO_0004339	Body measurement	Body measurement	PGS002981	EFO_0004339	body height	body height	EFO_0004339	Body measurement	Body measurement
0.7118	8.17E-72	7.67E-69	PGS002808	EFO_0001071	lung carcinoma	lung carcinoma	EFO_0001071	Cancer	Neoplasms	PGS000392	MONDO_0001672	bronchus cancer|lung cancer|tracheal cancer	bronchus cancer	MONDO_0001672	Cancer	Neoplasms
0.7118	8.35E-72	7.83E-69	PGS003098	MONDO_0005148	type 2 diabetes mellitus	type 2 diabetes mellitus	MONDO_0005148	Metabolic disorder	Metabolic	PGS003109	MONDO_0005148	type 2 diabetes mellitus	type 2 diabetes mellitus	MONDO_0005148	Digestive system disorder	Gastrointestinal
0.7118	8.35E-72	7.83E-69	PGS003098	MONDO_0005148	type 2 diabetes mellitus	type 2 diabetes mellitus	MONDO_0005148	Metabolic disorder	Metabolic	PGS003109	MONDO_0005148	type 2 diabetes mellitus	type 2 diabetes mellitus	MONDO_0005148	Metabolic disorder	Metabolic
0.7118	8.35E-72	7.83E-69	PGS003098	MONDO_0005148	type 2 diabetes mellitus	type 2 diabetes mellitus	MONDO_0005148	Digestive system disorder	Gastrointestinal	PGS003109	MONDO_0005148	type 2 diabetes mellitus	type 2 diabetes mellitus	MONDO_0005148	Metabolic disorder	Metabolic
0.7118	8.35E-72	7.83E-69	PGS003098	MONDO_0005148	type 2 diabetes mellitus	type 2 diabetes mellitus	MONDO_0005148	Digestive system disorder	Gastrointestinal	PGS003109	MONDO_0005148	type 2 diabetes mellitus	type 2 diabetes mellitus	MONDO_0005148	Digestive system disorder	Gastrointestinal
0.7117	8.44E-72	7.92E-69	PGS002851	EFO_0004340	body mass index	body mass index	EFO_0004340	Body measurement	Body measurement	PGS002854	EFO_0004340	body mass index	body mass index	EFO_0004340	Body measurement	Body measurement
0.7117	8.51E-72	7.98E-69	PGS001738	EFO_0006930	brain volume measurement|neuroimaging measurement	brain volume measurement	EFO_0006930	Other measurement	Neurological	PGS001721	EFO_0006930	measurement	brain volume measurement	EFO_0006930	Other measurement	Neurological
0.7117	8.54E-72	8.00E-69	PGS000540	EFO_0000305	breast carcinoma	breast carcinoma	EFO_0000305	Cancer	Neoplasms	PGS000529	EFO_0000305	breast carcinoma	breast carcinoma	EFO_0000305	Cancer	Neoplasms
0.7117	8.67E-72	8.12E-69	PGS001225	EFO_0004348	hematocrit	hematocrit	EFO_0004348	Hematological measurement	Hematological	PGS002141	EFO_0004348	hematocrit	hematocrit	EFO_0004348	Hematological measurement	Hematological
0.7117	8.70E-72	8.16E-69	PGS000305	EFO_0004465	fasting blood glucose measurement	fasting blood glucose measurement	EFO_0004465	Other measurement	Metabolic	PGS002951	EFO_0004465	fasting blood glucose measurement	fasting blood glucose measurement	EFO_0004465	Other measurement	Metabolic
0.7117	8.70E-72	8.16E-69	PGS000305	EFO_0004465	fasting blood glucose measurement	fasting blood glucose measurement	EFO_0004465	Other measurement	Metabolic	PGS002950	EFO_0004465	fasting blood glucose measurement	fasting blood glucose measurement	EFO_0004465	Other measurement	Metabolic
0.7117	8.74E-72	8.19E-69	PGS001929	EFO_0004339	body height	body height	EFO_0004339	Body measurement	Body measurement	PGS002971	EFO_0004339	body height	body height	EFO_0004339	Body measurement	Body measurement
0.7117	8.75E-72	8.20E-69	PGS001674	EFO_0006930	brain volume measurement|neuroimaging measurement	brain volume measurement	EFO_0006930	Other measurement	Neurological	PGS001661	EFO_0006930	measurement	brain volume measurement	EFO_0006930	Other measurement	Neurological
0.7116	9.05E-72	8.47E-69	PGS000539	EFO_0000305	breast carcinoma	breast carcinoma	EFO_0000305	Cancer	Neoplasms	PGS000530	EFO_0000305	breast carcinoma	breast carcinoma	EFO_0000305	Cancer	Neoplasms
0.7116	9.29E-72	8.69E-69	PGS001682	EFO_0006930	brain volume measurement|neuroimaging measurement	brain volume measurement	EFO_0006930	Other measurement	Neurological	PGS001472	EFO_0006930	measurement	brain volume measurement	EFO_0006930	Other measurement	Neurological
0.7116	9.34E-72	8.75E-69	PGS002910	EFO_0004329	alcohol drinking	alcohol drinking	EFO_0004329	Biological process	Psychiatric	PGS002911	EFO_0004329	alcohol drinking	alcohol drinking	EFO_0004329	Biological process	Psychiatric
0.7115	9.62E-72	9.01E-69	PGS002460	EFO_0004527	mean corpuscular hemoglobin	mean corpuscular hemoglobin	EFO_0004527	Hematological measurement	Hematological	PGS001989	EFO_0004527	mean corpuscular hemoglobin	mean corpuscular hemoglobin	EFO_0004527	Hematological measurement	Hematological
0.7115	9.83E-72	9.20E-69	PGS003036	EFO_0004611	low density lipoprotein cholesterol measurement	low density lipoprotein cholesterol measurement	EFO_0004611	Cardiovascular measurement	Cardiovascular	PGS000893	EFO_0004611	low density lipoprotein cholesterol measurement	measurement	EFO_0004611	Cardiovascular measurement	Cardiovascular
0.7115	9.83E-72	9.20E-69	PGS003036	EFO_0004611	low density lipoprotein cholesterol measurement	low density lipoprotein cholesterol measurement	EFO_0004611	Cardiovascular measurement	Cardiovascular	PGS000893	EFO_0004611	low density lipoprotein cholesterol measurement	measurement	EFO_0004611	Lipid or lipoprotein measurement	Metabolic
0.7115	9.83E-72	9.20E-69	PGS003036	EFO_0004611	low density lipoprotein cholesterol measurement	low density lipoprotein cholesterol measurement	EFO_0004611	Lipid or lipoprotein measurement	Metabolic	PGS000893	EFO_0004611	low density lipoprotein cholesterol measurement	measurement	EFO_0004611	Lipid or lipoprotein measurement	Metabolic
0.7115	9.83E-72	9.20E-69	PGS003036	EFO_0004611	low density lipoprotein cholesterol measurement	low density lipoprotein cholesterol measurement	EFO_0004611	Lipid or lipoprotein measurement	Metabolic	PGS000893	EFO_0004611	low density lipoprotein cholesterol measurement	measurement	EFO_0004611	Cardiovascular measurement	Cardiovascular
0.7115	1.02E-71	9.51E-69	PGS001450	EFO_0006930	brain volume measurement|neuroimaging measurement	brain volume measurement	EFO_0006930	Other measurement	Neurological	PGS001472	EFO_0006930	measurement	brain volume measurement	EFO_0006930	Other measurement	Neurological
0.7114	1.06E-71	9.91E-69	PGS001489	EFO_0006930	brain volume measurement|neuroimaging measurement	brain volume measurement	EFO_0006930	Other measurement	Neurological	PGS001672	EFO_0006930	measurement	brain volume measurement	EFO_0006930	Other measurement	Neurological
0.7114	1.07E-71	1.00E-68	PGS002632	EFO_0003923	bone density	bone density	EFO_0003923	Other measurement	Skeletal	PGS002583	EFO_0003923	bone density	bone density	EFO_0003923	Other measurement	Skeletal
0.7114	1.08E-71	1.01E-68	PGS003084	EFO_0006335	systolic blood pressure	systolic blood pressure	EFO_0006335	Other measurement	Cardiovascular	PGS003083	EFO_0006335	systolic blood pressure	systolic blood pressure	EFO_0006335	Other measurement	Cardiovascular
0.7114	1.09E-71	1.02E-68	PGS002845	EFO_0004340	body mass index	body mass index	EFO_0004340	Body measurement	Body measurement	PGS002630	EFO_0004340	body mass index	body mass index	EFO_0004340	Body measurement	Body measurement
0.7113	1.12E-71	1.05E-68	PGS002845	EFO_0004340	body mass index	body mass index	EFO_0004340	Body measurement	Body measurement	PGS002841	EFO_0004340	body mass index	body mass index	EFO_0004340	Body measurement	Body measurement
0.7113	1.13E-71	1.05E-68	PGS001678	EFO_0006930	brain volume measurement|neuroimaging measurement	brain volume measurement	EFO_0006930	Other measurement	Neurological	PGS001459	EFO_0006930	measurement	brain volume measurement	EFO_0006930	Other measurement	Neurological
0.7112	1.17E-71	1.10E-68	PGS000212	MONDO_0021116	luminal A breast carcinoma	luminal A breast carcinoma	MONDO_0021116	Cancer	Neoplasms	PGS000540	EFO_0000305	breast carcinoma	breast carcinoma	EFO_0000305	Cancer	Neoplasms
0.7112	1.18E-71	1.10E-68	PGS000113	EFO_0004308	leukocyte count	leukocyte count	EFO_0004308	Hematological measurement	Hematological	PGS002357	EFO_0004308	leukocyte count	leukocyte count	EFO_0004308	Hematological measurement	Hematological
0.7112	1.18E-71	1.11E-68	PGS001451	EFO_0006930	brain volume measurement|neuroimaging measurement	brain volume measurement	EFO_0006930	Other measurement	Neurological	PGS001480	EFO_0006930	measurement	brain volume measurement	EFO_0006930	Other measurement	Neurological
0.7112	1.18E-71	1.11E-68	PGS002534	EFO_0003923	bone density	bone density	EFO_0003923	Other measurement	Skeletal	PGS002175	EFO_0005654	velocity of sound measurement	velocity of sound measurement	EFO_0005654	Other measurement	Others
0.7112	1.19E-71	1.11E-68	PGS003200	EFO_0004631	vitamin D measurement	vitamin D measurement	EFO_0004631	Lipid or lipoprotein measurement	Metabolic	PGS001982	EFO_0004631	vitamin D measurement	vitamin D measurement	EFO_0004631	Lipid or lipoprotein measurement	Metabolic
0.7112	1.20E-71	1.12E-68	PGS003123	MONDO_0005148	type 2 diabetes mellitus	type 2 diabetes mellitus	MONDO_0005148	Metabolic disorder	Metabolic	PGS002779	MONDO_0005148	type 2 diabetes mellitus	type 2 diabetes mellitus	MONDO_0005148	Metabolic disorder	Metabolic
0.7112	1.20E-71	1.12E-68	PGS003123	MONDO_0005148	type 2 diabetes mellitus	type 2 diabetes mellitus	MONDO_0005148	Metabolic disorder	Metabolic	PGS002779	MONDO_0005148	type 2 diabetes mellitus	type 2 diabetes mellitus	MONDO_0005148	Digestive system disorder	Gastrointestinal
0.7112	1.20E-71	1.12E-68	PGS003123	MONDO_0005148	type 2 diabetes mellitus	type 2 diabetes mellitus	MONDO_0005148	Digestive system disorder	Gastrointestinal	PGS002779	MONDO_0005148	type 2 diabetes mellitus	type 2 diabetes mellitus	MONDO_0005148	Metabolic disorder	Metabolic
0.7112	1.20E-71	1.12E-68	PGS003123	MONDO_0005148	type 2 diabetes mellitus	type 2 diabetes mellitus	MONDO_0005148	Digestive system disorder	Gastrointestinal	PGS002779	MONDO_0005148	type 2 diabetes mellitus	type 2 diabetes mellitus	MONDO_0005148	Digestive system disorder	Gastrointestinal
0.7112	1.21E-71	1.13E-68	PGS002242	EFO_0000305	breast carcinoma	breast carcinoma	EFO_0000305	Cancer	Neoplasms	PGS000335	EFO_0000305	breast carcinoma	breast carcinoma	EFO_0000305	Cancer	Neoplasms
0.7112	1.22E-71	1.14E-68	PGS000677	EFO_0004574	total cholesterol measurement	total cholesterol measurement	EFO_0004574	Lipid or lipoprotein measurement	Metabolic	PGS001889	EFO_0004615	apolipoprotein B measurement	apolipoprotein B measurement	EFO_0004615	Lipid or lipoprotein measurement	Metabolic
0.7112	1.23E-71	1.15E-68	PGS000509	EFO_0000305	breast carcinoma	breast carcinoma	EFO_0000305	Cancer	Neoplasms	PGS000503	EFO_0000305	breast carcinoma	breast carcinoma	EFO_0000305	Cancer	Neoplasms
0.7111	1.26E-71	1.18E-68	PGS002569	EFO_0004279	suntan	suntan	EFO_0004279	Other trait	Dermatological	PGS002520	EFO_0004279	suntan	suntan	EFO_0004279	Other trait	Dermatological
0.7111	1.30E-71	1.21E-68	PGS001230	EFO_0004338	body weight	body weight	EFO_0004338	Body measurement	Body measurement	PGS002176	EFO_0005093	hip circumference	hip circumference	EFO_0005093	Body measurement	Body measurement
0.711	1.37E-71	1.28E-68	PGS003036	EFO_0004611	low density lipoprotein cholesterol measurement	low density lipoprotein cholesterol measurement	EFO_0004611	Cardiovascular measurement	Cardiovascular	PGS002571	EFO_0004574	total cholesterol measurement	total cholesterol measurement	EFO_0004574	Lipid or lipoprotein measurement	Metabolic
0.711	1.37E-71	1.28E-68	PGS003036	EFO_0004611	low density lipoprotein cholesterol measurement	low density lipoprotein cholesterol measurement	EFO_0004611	Lipid or lipoprotein measurement	Metabolic	PGS002571	EFO_0004574	total cholesterol measurement	total cholesterol measurement	EFO_0004574	Lipid or lipoprotein measurement	Metabolic
0.711	1.38E-71	1.29E-68	PGS002315	EFO_0003923	bone density	bone density	EFO_0003923	Other measurement	Skeletal	PGS001530	EFO_0007772	measurement	measurement	EFO_0007772	Other measurement	Skeletal
0.711	1.39E-71	1.30E-68	PGS003034	EFO_0004611	low density lipoprotein cholesterol measurement	low density lipoprotein cholesterol measurement	EFO_0004611	Cardiovascular measurement	Cardiovascular	PGS002782	EFO_0005689	measurement	measurement	EFO_0005689	Cardiovascular measurement	Cardiovascular
0.711	1.39E-71	1.30E-68	PGS003034	EFO_0004611	low density lipoprotein cholesterol measurement	low density lipoprotein cholesterol measurement	EFO_0004611	Cardiovascular measurement	Cardiovascular	PGS002782	EFO_0005689	measurement	measurement	EFO_0005689	Lipid or lipoprotein measurement	Metabolic
0.711	1.39E-71	1.30E-68	PGS003034	EFO_0004611	low density lipoprotein cholesterol measurement	low density lipoprotein cholesterol measurement	EFO_0004611	Lipid or lipoprotein measurement	Metabolic	PGS002782	EFO_0005689	measurement	measurement	EFO_0005689	Cardiovascular measurement	Cardiovascular
0.711	1.39E-71	1.30E-68	PGS003034	EFO_0004611	low density lipoprotein cholesterol measurement	low density lipoprotein cholesterol measurement	EFO_0004611	Lipid or lipoprotein measurement	Metabolic	PGS002782	EFO_0005689	measurement	measurement	EFO_0005689	Lipid or lipoprotein measurement	Metabolic
0.711	1.40E-71	1.31E-68	PGS002461	EFO_0004584	mean platelet volume	mean platelet volume	EFO_0004584	Hematological measurement	Hematological	PGS002340	EFO_0004584	mean platelet volume	mean platelet volume	EFO_0004584	Hematological measurement	Hematological
0.7108	1.52E-71	1.42E-68	PGS003193	EFO_0004631	vitamin D measurement	vitamin D measurement	EFO_0004631	Lipid or lipoprotein measurement	Metabolic	PGS002574	EFO_0004631	vitamin D measurement	vitamin D measurement	EFO_0004631	Lipid or lipoprotein measurement	Metabolic
0.7108	1.53E-71	1.43E-68	PGS002780	MONDO_0005148	type 2 diabetes mellitus	type 2 diabetes mellitus	MONDO_0005148	Digestive system disorder	Gastrointestinal	PGS003108	MONDO_0005148	type 2 diabetes mellitus	type 2 diabetes mellitus	MONDO_0005148	Metabolic disorder	Metabolic
0.7108	1.53E-71	1.43E-68	PGS002780	MONDO_0005148	type 2 diabetes mellitus	type 2 diabetes mellitus	MONDO_0005148	Metabolic disorder	Metabolic	PGS003108	MONDO_0005148	type 2 diabetes mellitus	type 2 diabetes mellitus	MONDO_0005148	Metabolic disorder	Metabolic
0.7108	1.53E-71	1.43E-68	PGS002780	MONDO_0005148	type 2 diabetes mellitus	type 2 diabetes mellitus	MONDO_0005148	Metabolic disorder	Metabolic	PGS003108	MONDO_0005148	type 2 diabetes mellitus	type 2 diabetes mellitus	MONDO_0005148	Digestive system disorder	Gastrointestinal
0.7108	1.53E-71	1.43E-68	PGS002780	MONDO_0005148	type 2 diabetes mellitus	type 2 diabetes mellitus	MONDO_0005148	Digestive system disorder	Gastrointestinal	PGS003108	MONDO_0005148	type 2 diabetes mellitus	type 2 diabetes mellitus	MONDO_0005148	Digestive system disorder	Gastrointestinal

0.7107	1.63E-71	1.52E-68	PGS003356	EFO_0001645	coronary artery disease	coronary artery disease	EFO_0001645	Cardiovascular disease	Cardiovascular	PGS001780	EFO_0001645	coronary artery disease	coronary artery disease	EFO_0001645	Cardiovascular disease	Cardiovascular
0.7107	1.69E-71	1.57E-68	PGS001944	EFO_0007934	creatinine clearance measurement	creatinine clearance measurement	EFO_0007934	Other measurement	Metabolic	PGS003233	EFO_0010472	creatine measurement	creatine measurement	EFO_0010472	Other measurement	Metabolic
0.7107	1.71E-71	1.59E-68	PGS000758	EFO_0004339	body height	body height	EFO_0004339	Body measurement	Body measurement	PGS002971	EFO_0004339	body height	body height	EFO_0004339	Body measurement	Body measurement
0.7107	1.72E-71	1.60E-68	PGS000746	EFO_0001645	coronary artery disease	coronary artery disease	EFO_0001645	Cardiovascular disease	Cardiovascular	PGS003355	EFO_0001645	coronary artery disease	coronary artery disease	EFO_0001645	Cardiovascular disease	Cardiovascular
0.7106	1.79E-71	1.67E-68	PGS003143	EFO_0004574	total cholesterol measurement	total cholesterol measurement	EFO_0004574	Lipid or lipoprotein measurement	Metabolic	PGS001933	EFO_0004611	low density lipoprotein cholesterol measurement	measurement	EFO_0004611	Cardiovascular measurement	Cardiovascular
0.7106	1.79E-71	1.67E-68	PGS003143	EFO_0004574	total cholesterol measurement	total cholesterol measurement	EFO_0004574	Lipid or lipoprotein measurement	Metabolic	PGS001933	EFO_0004611	low density lipoprotein cholesterol measurement	measurement	EFO_0004611	Lipid or lipoprotein measurement	Metabolic
0.7106	1.80E-71	1.67E-68	PGS001957	EFO_0005654	velocity of sound measurement	velocity of sound measurement	EFO_0005654	Other measurement	Others	PGS001403	EFO_0009270	heel bone mineral density	heel bone mineral density	EFO_0009270	Other measurement	Skeletal
0.7106	1.80E-71	1.68E-68	PGS003038	EFO_0004611	low density lipoprotein cholesterol measurement	low density lipoprotein cholesterol measurement	EFO_0004611	Cardiovascular measurement	Cardiovascular	PGS001889	EFO_0004615	apolipoprotein B measurement	apolipoprotein B measurement	EFO_0004615	Lipid or lipoprotein measurement	Metabolic
0.7106	1.80E-71	1.68E-68	PGS003038	EFO_0004611	low density lipoprotein cholesterol measurement	low density lipoprotein cholesterol measurement	EFO_0004611	Lipid or lipoprotein measurement	Metabolic	PGS001889	EFO_0004615	apolipoprotein B measurement	apolipoprotein B measurement	EFO_0004615	Lipid or lipoprotein measurement	Metabolic
0.7106	1.81E-71	1.68E-68	PGS002721	EFO_0004631	vitamin D measurement	vitamin D measurement	EFO_0004631	Lipid or lipoprotein measurement	Metabolic	PGS003196	EFO_0004631	vitamin D measurement	vitamin D measurement	EFO_0004631	Lipid or lipoprotein measurement	Metabolic
0.7105	1.88E-71	1.75E-68	PGS001671	EFO_0006930	brain volume measurement|neuroimaging measurement	brain volume measurement	EFO_0006930	Other measurement	Neurological	PGS001683	EFO_0006930	measurement	brain volume measurement	EFO_0006930	Other measurement	Neurological
-0.7105	1.89E-71	1.76E-68	PGS001986	EFO_0007993	lymphocyte percentage of leukocytes	lymphocyte percentage of leukocytes	EFO_0007993	Hematological measurement	Hematological	PGS001077	EFO_0007990	neutrophil percentage of leukocytes	neutrophil percentage of leukocytes	EFO_0007990	Hematological measurement	Hematological
0.7105	1.91E-71	1.77E-68	PGS001609	EFO_0005420	measurement	grey matter volume measurement	EFO_0005420	Other measurement	Neurological	PGS001608	EFO_0005420	measurement	grey matter volume measurement	EFO_0005420	Other measurement	Neurological
-0.7105	1.93E-71	1.79E-68	PGS001039	EFO_0009270	heel bone mineral density	heel bone mineral density	EFO_0009270	Other measurement	Skeletal	PGS002768	EFO_0003882	osteoporosis	osteoporosis	EFO_0003882	Other disease	Skeletal
-0.7105	1.93E-71	1.80E-68	PGS000956	EFO_0004514	bone quantitative ultrasound measurement	bone quantitative ultrasound measurement	EFO_0004514	Other measurement	Skeletal	PGS002768	EFO_0003882	osteoporosis	osteoporosis	EFO_0003882	Other disease	Skeletal
0.7105	1.95E-71	1.81E-68	PGS003030	EFO_0004611	low density lipoprotein cholesterol measurement	low density lipoprotein cholesterol measurement	EFO_0004611	Lipid or lipoprotein measurement	Metabolic	PGS003033	EFO_0004611	low density lipoprotein cholesterol measurement	measurement	EFO_0004611	Cardiovascular measurement	Cardiovascular
0.7105	1.95E-71	1.81E-68	PGS003030	EFO_0004611	low density lipoprotein cholesterol measurement	low density lipoprotein cholesterol measurement	EFO_0004611	Lipid or lipoprotein measurement	Metabolic	PGS003033	EFO_0004611	low density lipoprotein cholesterol measurement	measurement	EFO_0004611	Lipid or lipoprotein measurement	Metabolic
0.7105	1.95E-71	1.81E-68	PGS003030	EFO_0004611	low density lipoprotein cholesterol measurement	low density lipoprotein cholesterol measurement	EFO_0004611	Cardiovascular measurement	Cardiovascular	PGS003033	EFO_0004611	low density lipoprotein cholesterol measurement	measurement	EFO_0004611	Cardiovascular measurement	Cardiovascular
0.7105	1.95E-71	1.81E-68	PGS003030	EFO_0004611	low density lipoprotein cholesterol measurement	low density lipoprotein cholesterol measurement	EFO_0004611	Cardiovascular measurement	Cardiovascular	PGS003033	EFO_0004611	low density lipoprotein cholesterol measurement	measurement	EFO_0004611	Lipid or lipoprotein measurement	Metabolic
0.7104	1.99E-71	1.84E-68	PGS003201	EFO_0004631	vitamin D measurement	vitamin D measurement	EFO_0004631	Lipid or lipoprotein measurement	Metabolic	PGS002574	EFO_0004631	vitamin D measurement	vitamin D measurement	EFO_0004631	Lipid or lipoprotein measurement	Metabolic
0.7104	2.07E-71	1.92E-68	PGS000956	EFO_0004514	bone quantitative ultrasound measurement	bone quantitative ultrasound measurement	EFO_0004514	Other measurement	Skeletal	PGS002315	EFO_0003923	bone density	bone density	EFO_0003923	Other measurement	Skeletal
0.7104	2.07E-71	1.92E-68	PGS002412	EFO_0004584	mean platelet volume	mean platelet volume	EFO_0004584	Hematological measurement	Hematological	PGS002340	EFO_0004584	mean platelet volume	mean platelet volume	EFO_0004584	Hematological measurement	Hematological
0.7104	2.07E-71	1.92E-68	PGS001039	EFO_0009270	heel bone mineral density	heel bone mineral density	EFO_0009270	Other measurement	Skeletal	PGS002315	EFO_0003923	bone density	bone density	EFO_0003923	Other measurement	Skeletal
0.7104	2.10E-71	1.95E-68	PGS000988	EFO_0010700	reticulocyte measurement	reticulocyte measurement	EFO_0010700	Hematological measurement	Hematological	PGS002333	EFO_0007986	reticulocyte count	reticulocyte count	EFO_0007986	Hematological measurement	Hematological
0.7104	2.10E-71	1.95E-68	PGS000789	EFO_0001071	lung carcinoma	lung carcinoma	EFO_0001071	Cancer	Neoplasms	PGS003392	EFO_0000708	squamous cell lung carcinoma	squamous cell lung carcinoma	EFO_0000708	Cancer	Neoplasms
0.7103	2.15E-71	1.99E-68	PGS003243	EFO_0010472	creatine measurement	creatine measurement	EFO_0010472	Other measurement	Metabolic	PGS003245	EFO_0010472	creatine measurement	creatine measurement	EFO_0010472	Other measurement	Metabolic
0.7103	2.15E-71	1.99E-68	PGS003398	EFO_0000305	breast carcinoma	breast carcinoma	EFO_0000305	Cancer	Neoplasms	PGS000485	EFO_0000305	breast carcinoma	breast carcinoma	EFO_0000305	Cancer	Neoplasms
0.7103	2.15E-71	1.99E-68	PGS003398	EFO_0000305	breast carcinoma	breast carcinoma	EFO_0000305	Cancer	Neoplasms	PGS000487	EFO_0000305	breast carcinoma	breast carcinoma	EFO_0000305	Cancer	Neoplasms
0.7103	2.19E-71	2.03E-68	PGS000080	EFO_0005952	non-Hodgkins lymphoma	non-Hodgkins lymphoma	EFO_0005952	Cancer	Neoplasms	PGS000641	EFO_0005952	non-Hodgkins lymphoma	non-Hodgkins lymphoma	EFO_0005952	Cancer	Neoplasms
0.7103	2.23E-71	2.07E-68	PGS001489	EFO_0006930	brain volume measurement|neuroimaging measurement	brain volume measurement	EFO_0006930	Other measurement	Neurological	PGS001670	EFO_0006930	measurement	brain volume measurement	EFO_0006930	Other measurement	Neurological
0.7103	2.23E-71	2.07E-68	PGS000818	EFO_0001645	coronary artery disease	coronary artery disease	EFO_0001645	Cardiovascular disease	Cardiovascular	PGS000058	EFO_0001645	coronary artery disease	coronary artery disease	EFO_0001645	Cardiovascular disease	Cardiovascular
0.7102	2.25E-71	2.09E-68	PGS002796	EFO_0001663	prostate carcinoma	prostate carcinoma	EFO_0001663	Cancer	Neoplasms	PGS000742	EFO_0001663	prostate carcinoma	prostate carcinoma	EFO_0001663	Cancer	Neoplasms
0.7102	2.26E-71	2.09E-68	PGS001162	EFO_0005093	hip circumference	hip circumference	EFO_0005093	Body measurement	Body measurement	PGS001101	EFO_0007800	body fat percentage	body fat percentage	EFO_0007800	Body measurement	Body measurement
0.7102	2.26E-71	2.09E-68	PGS002949	EFO_0004465	fasting blood glucose measurement	fasting blood glucose measurement	EFO_0004465	Other measurement	Metabolic	PGS002951	EFO_0004465	fasting blood glucose measurement	fasting blood glucose measurement	EFO_0004465	Other measurement	Metabolic
0.7102	2.26E-71	2.09E-68	PGS002949	EFO_0004465	fasting blood glucose measurement	fasting blood glucose measurement	EFO_0004465	Other measurement	Metabolic	PGS002950	EFO_0004465	fasting blood glucose measurement	fasting blood glucose measurement	EFO_0004465	Other measurement	Metabolic
0.7102	2.34E-71	2.16E-68	PGS002200	EFO_0004631	vitamin D measurement	vitamin D measurement	EFO_0004631	Lipid or lipoprotein measurement	Metabolic	PGS003184	EFO_0004631	vitamin D measurement	vitamin D measurement	EFO_0004631	Lipid or lipoprotein measurement	Metabolic
0.7102	2.35E-71	2.17E-68	PGS002632	EFO_0003923	bone density	bone density	EFO_0003923	Other measurement	Skeletal	PGS000951	EFO_0004514	bone quantitative ultrasound measurement	measurement	EFO_0004514	Other measurement	Skeletal
0.7102	2.36E-71	2.18E-68	PGS000889	EFO_0004611	low density lipoprotein cholesterol measurement	low density lipoprotein cholesterol measurement	EFO_0004611	Lipid or lipoprotein measurement	Metabolic	PGS001889	EFO_0004615	apolipoprotein B measurement	apolipoprotein B measurement	EFO_0004615	Lipid or lipoprotein measurement	Metabolic
0.7102	2.36E-71	2.18E-68	PGS000889	EFO_0004611	low density lipoprotein cholesterol measurement	low density lipoprotein cholesterol measurement	EFO_0004611	Cardiovascular measurement	Cardiovascular	PGS001889	EFO_0004615	apolipoprotein B measurement	apolipoprotein B measurement	EFO_0004615	Lipid or lipoprotein measurement	Metabolic
0.7101	2.40E-71	2.22E-68	PGS001478	EFO_0006930	brain volume measurement|neuroimaging measurement	brain volume measurement	EFO_0006930	Other measurement	Neurological	PGS001477	EFO_0006930	measurement	brain volume measurement	EFO_0006930	Other measurement	Neurological
0.7101	2.42E-71	2.24E-68	PGS001674	EFO_0006930	brain volume measurement|neuroimaging measurement	brain volume measurement	EFO_0006930	Other measurement	Neurological	PGS001687	EFO_0006930	measurement	brain volume measurement	EFO_0006930	Other measurement	Neurological
0.7101	2.44E-71	2.26E-68	PGS001478	EFO_0006930	brain volume measurement|neuroimaging measurement	brain volume measurement	EFO_0006930	Other measurement	Neurological	PGS001672	EFO_0006930	measurement	brain volume measurement	EFO_0006930	Other measurement	Neurological
0.7101	2.48E-71	2.30E-68	PGS000103	EFO_0007989	monocyte percentage of leukocytes	monocyte percentage of leukocytes	EFO_0007989	Hematological measurement	Hematological	PGS000102	EFO_0005091	monocyte count	monocyte count	EFO_0005091	Hematological measurement	Hematological
0.7101	2.49E-71	2.30E-68	PGS003035	EFO_0004611	low density lipoprotein cholesterol measurement	low density lipoprotein cholesterol measurement	EFO_0004611	Cardiovascular measurement	Cardiovascular	PGS003038	EFO_0004611	low density lipoprotein cholesterol measurement	measurement	EFO_0004611	Lipid or lipoprotein measurement	Metabolic
0.7101	2.49E-71	2.30E-68	PGS003035	EFO_0004611	low density lipoprotein cholesterol measurement	low density lipoprotein cholesterol measurement	EFO_0004611	Cardiovascular measurement	Cardiovascular	PGS003038	EFO_0004611	low density lipoprotein cholesterol measurement	measurement	EFO_0004611	Cardiovascular measurement	Cardiovascular
0.7101	2.49E-71	2.30E-68	PGS003035	EFO_0004611	low density lipoprotein cholesterol measurement	low density lipoprotein cholesterol measurement	EFO_0004611	Lipid or lipoprotein measurement	Metabolic	PGS003038	EFO_0004611	low density lipoprotein cholesterol measurement	measurement	EFO_0004611	Lipid or lipoprotein measurement	Metabolic
0.7101	2.49E-71	2.30E-68	PGS003035	EFO_0004611	low density lipoprotein cholesterol measurement	low density lipoprotein cholesterol measurement	EFO_0004611	Lipid or lipoprotein measurement	Metabolic	PGS003038	EFO_0004611	low density lipoprotein cholesterol measurement	measurement	EFO_0004611	Cardiovascular measurement	Cardiovascular
0.7101	2.50E-71	2.31E-68	PGS003140	EFO_0004574	total cholesterol measurement	total cholesterol measurement	EFO_0004574	Lipid or lipoprotein measurement	Metabolic	PGS002424	EFO_0004574	total cholesterol measurement	total cholesterol measurement	EFO_0004574	Lipid or lipoprotein measurement	Metabolic
0.7101	2.52E-71	2.33E-68	PGS001149	EFO_0005409	fat body mass	fat body mass	EFO_0005409	Other measurement	Body measurement	PGS002169	EFO_0005409	fat body mass	fat body mass	EFO_0005409	Other measurement	Body measurement
0.71	2.61E-71	2.42E-68	PGS000440	MONDO_0002898	skin cancer	skin cancer	MONDO_0002898	Cancer	Neoplasms	PGS000439	MONDO_0002898	skin cancer	skin cancer	MONDO_0002898	Cancer	Neoplasms
0.71	2.64E-71	2.44E-68	PGS003030	EFO_0004611	low density lipoprotein cholesterol measurement	low density lipoprotein cholesterol measurement	EFO_0004611	Lipid or lipoprotein measurement	Metabolic	PGS003038	EFO_0004611	low density lipoprotein cholesterol measurement	measurement	EFO_0004611	Lipid or lipoprotein measurement	Metabolic
0.71	2.64E-71	2.44E-68	PGS003030	EFO_0004611	low density lipoprotein cholesterol measurement	low density lipoprotein cholesterol measurement	EFO_0004611	Lipid or lipoprotein measurement	Metabolic	PGS003038	EFO_0004611	low density lipoprotein cholesterol measurement	measurement	EFO_0004611	Cardiovascular measurement	Cardiovascular
0.71	2.64E-71	2.44E-68	PGS003030	EFO_0004611	low density lipoprotein cholesterol measurement	low density lipoprotein cholesterol measurement	EFO_0004611	Cardiovascular measurement	Cardiovascular	PGS003038	EFO_0004611	low density lipoprotein cholesterol measurement	measurement	EFO_0004611	Lipid or lipoprotein measurement	Metabolic
0.71	2.64E-71	2.44E-68	PGS003030	EFO_0004611	low density lipoprotein cholesterol measurement	low density lipoprotein cholesterol measurement	EFO_0004611	Cardiovascular measurement	Cardiovascular	PGS003038	EFO_0004611	low density lipoprotein cholesterol measurement	measurement	EFO_0004611	Cardiovascular measurement	Cardiovascular
0.7099	2.84E-71	2.63E-68	PGS002200	EFO_0004631	vitamin D measurement	vitamin D measurement	EFO_0004631	Lipid or lipoprotein measurement	Metabolic	PGS003199	EFO_0004631	vitamin D measurement	vitamin D measurement	EFO_0004631	Lipid or lipoprotein measurement	Metabolic
0.7099	2.85E-71	2.63E-68	PGS000507	EFO_0000305	breast carcinoma	breast carcinoma	EFO_0000305	Cancer	Neoplasms	PGS000004	EFO_0000305	breast carcinoma	breast carcinoma	EFO_0000305	Cancer	Neoplasms
0.7098	3.08E-71	2.84E-68	PGS000333	EFO_0001663	prostate carcinoma	prostate carcinoma	EFO_0001663	Cancer	Neoplasms	PGS000587	EFO_0001663	prostate carcinoma	prostate carcinoma	EFO_0001663	Cancer	Neoplasms
0.7097	3.13E-71	2.89E-68	PGS000008	EFO_1000649	estrogen-receptor positive breast cancer	estrogen-receptor positive breast cancer	EFO_1000649	Cancer	Neoplasms	PGS000497	EFO_0000305	breast carcinoma	breast carcinoma	EFO_0000305	Cancer	Neoplasms
0.7097	3.13E-71	2.89E-68	PGS000008	EFO_1000649	estrogen-receptor positive breast cancer	estrogen-receptor positive breast cancer	EFO_1000649	Cancer	Neoplasms	PGS000498	EFO_0000305	breast carcinoma	breast carcinoma	EFO_0000305	Cancer	Neoplasms
0.7097	3.15E-71	2.90E-68	PGS000515	EFO_0000305	breast carcinoma	breast carcinoma	EFO_0000305	Cancer	Neoplasms	PGS000538	EFO_0000305	breast carcinoma	breast carcinoma	EFO_0000305	Cancer	Neoplasms
0.7097	3.20E-71	2.96E-68	PGS003314	EFO_0004617	cystatin C measurement|glomerular filtration rate	cystatin C measurement	EFO_0004617	Other measurement	Metabolic	PGS003301	EFO_0004617	cystatin C measurement|glomerular filtration rate	cystatin C measurement	EFO_0004617	Other measurement	Metabolic
0.7097	3.21E-71	2.96E-68	PGS001663	EFO_0006930	brain volume measurement|neuroimaging measurement	brain volume measurement	EFO_0006930	Other measurement	Neurological	PGS001670	EFO_0006930	measurement	brain volume measurement	EFO_0006930	Other measurement	Neurological
0.7097	3.25E-71	3.00E-68	PGS002632	EFO_0003923	bone density	bone density	EFO_0003923	Other measurement	Skeletal	PGS001530	EFO_0007772	measurement	measurement	EFO_0007772	Other measurement	Skeletal
-0.7097	3.25E-71	3.00E-68	PGS001531	EFO_0007772	calcaneal bone quantitative ultrasound measurement	calcaneal bone quantitative ultrasound measurement	EFO_0007772	Other measurement	Skeletal	PGS002768	EFO_0003882	osteoporosis	osteoporosis	EFO_0003882	Other disease	Skeletal
0.7097	3.32E-71	3.06E-68	PGS001093	EFO_0007822	hair colour measurement	hair colour measurement	EFO_0007822	Other measurement	Dermatological	PGS002647	EFO_0007822	hair colour measurement	hair colour measurement	EFO_0007822	Other measurement	Dermatological
0.7096	3.42E-71	3.15E-68	PGS000002	EFO_1000649	estrogen-receptor positive breast cancer	estrogen-receptor positive breast cancer	EFO_1000649	Cancer	Neoplasms	PGS000505	EFO_0000305	breast carcinoma	breast carcinoma	EFO_0000305	Cancer	Neoplasms
0.7096	3.42E-71	3.15E-68	PGS000002	EFO_1000649	estrogen-receptor positive breast cancer	estrogen-receptor positive breast cancer	EFO_1000649	Cancer	Neoplasms	PGS000506	EFO_0000305	breast carcinoma	breast carcinoma	EFO_0000305	Cancer	Neoplasms
0.7096	3.44E-71	3.17E-68	PGS000051	EFO_0000305	breast carcinoma	breast carcinoma	EFO_0000305	Cancer	Neoplasms	PGS001778	EFO_0000305	breast carcinoma	breast carcinoma	EFO_0000305	Cancer	Neoplasms
0.7096	3.45E-71	3.18E-68	PGS003147	EFO_0004530	triglyceride measurement	triglyceride measurement	EFO_0004530	Lipid or lipoprotein measurement	Metabolic	PGS003153	EFO_0004530	triglyceride measurement	triglyceride measurement	EFO_0004530	Lipid or lipoprotein measurement	Metabolic
0.7096	3.52E-71	3.24E-68	PGS000153	EFO_0000305	breast carcinoma	breast carcinoma	EFO_0000305	Cancer	Neoplasms	PGS000046	EFO_1000649	estrogen-receptor positive breast cancer	cancer	EFO_1000649	Cancer	Neoplasms
0.7095	3.57E-71	3.29E-68	PGS002781	EFO_0004612	high density lipoprotein cholesterol measurement	high density lipoprotein cholesterol measurement	EFO_0004612	Lipid or lipoprotein measurement	Metabolic	PGS002597	EFO_0004612	high density lipoprotein cholesterol measurement	measurement	EFO_0004612	Lipid or lipoprotein measurement	Metabolic
0.7095	3.59E-71	3.30E-68	PGS000955	EFO_0004514	bone quantitative ultrasound measurement	bone quantitative ultrasound measurement	EFO_0004514	Other measurement	Skeletal	PGS001402	EFO_0009270	heel bone mineral density	heel bone mineral density	EFO_0009270	Other measurement	Skeletal
0.7095	3.63E-71	3.34E-68	PGS002339	EFO_0004527	mean corpuscular hemoglobin	mean corpuscular hemoglobin	EFO_0004527	Hematological measurement	Hematological	PGS000174	EFO_0004527	mean corpuscular hemoglobin	mean corpuscular hemoglobin	EFO_0004527	Hematological measurement	Hematological
0.7095	3.65E-71	3.36E-68	PGS002348	EFO_0006527	smoking status measurement	smoking status measurement	EFO_0006527	Other measurement	Psychiatric	PGS003068	EFO_0006527	smoking status measurement	smoking status measurement	EFO_0006527	Other measurement	Psychiatric
0.7095	3.65E-71	3.36E-68	PGS000478	EFO_0000305	breast carcinoma	breast carcinoma	EFO_0000305	Cancer	Neoplasms	PGS001804	EFO_0000305	breast carcinoma	breast carcinoma	EFO_0000305	Cancer	Neoplasms
0.7095	3.69E-71	3.39E-68	PGS003134	EFO_0004574	total cholesterol measurement	total cholesterol measurement	EFO_0004574	Lipid or lipoprotein measurement	Metabolic	PGS001895	EFO_0004574	total cholesterol measurement	total cholesterol measurement	EFO_0004574	Lipid or lipoprotein measurement	Metabolic
0.7094	3.82E-71	3.51E-68	PGS000630	EFO_0002892	thyroid carcinoma	thyroid carcinoma	EFO_0002892	Cancer	Neoplasms	PGS000087	EFO_0002892	thyroid carcinoma	thyroid carcinoma	EFO_0002892	Cancer	Neoplasms
0.7094	3.82E-71	3.51E-68	PGS000087	EFO_0002892	thyroid carcinoma	thyroid carcinoma	EFO_0002892	Cancer	Neoplasms	PGS000631	EFO_0002892	thyroid carcinoma	thyroid carcinoma	EFO_0002892	Cancer	Neoplasms
0.7094	3.85E-71	3.54E-68	PGS002845	EFO_0004340	body mass index	body mass index	EFO_0004340	Body measurement	Body measurement	PGS002169	EFO_0005409	fat body mass	fat body mass	EFO_0005409	Other measurement	Body measurement
0.7094	3.88E-71	3.57E-68	PGS002628	MONDO_0004979	asthma	asthma	MONDO_0004979	Other disease	Respiratory	PGS002754	MONDO_0004979	asthma	asthma	MONDO_0004979	Other disease	Respiratory
0.7094	3.93E-71	3.61E-68	PGS000176	EFO_0004526	mean corpuscular volume	mean corpuscular volume	EFO_0004526	Hematological measurement	Hematological	PGS000099	EFO_0004527	mean corpuscular hemoglobin	mean corpuscular hemoglobin	EFO_0004527	Hematological measurement	Hematological
0.7094	3.98E-71	3.65E-68	PGS002721	EFO_0004631	vitamin D measurement	vitamin D measurement	EFO_0004631	Lipid or lipoprotein measurement	Metabolic	PGS002623	EFO_0004631	vitamin D measurement	vitamin D measurement	EFO_0004631	Lipid or lipoprotein measurement	Metabolic
0.7093	4.08E-71	3.75E-68	PGS002605	EFO_0004611	low density lipoprotein cholesterol measurement	low density lipoprotein cholesterol measurement	EFO_0004611	Cardiovascular measurement	Cardiovascular	PGS001933	EFO_0004611	low density lipoprotein cholesterol measurement	measurement	EFO_0004611	Cardiovascular measurement	Cardiovascular
0.7093	4.08E-71	3.75E-68	PGS002605	EFO_0004611	low density lipoprotein cholesterol measurement	low density lipoprotein cholesterol measurement	EFO_0004611	Cardiovascular measurement	Cardiovascular	PGS001933	EFO_0004611	low density lipoprotein cholesterol measurement	measurement	EFO_0004611	Lipid or lipoprotein measurement	Metabolic
0.7093	4.08E-71	3.75E-68	PGS002605	EFO_0004611	low density lipoprotein cholesterol measurement	low density lipoprotein cholesterol measurement	EFO_0004611	Lipid or lipoprotein measurement	Metabolic	PGS001933	EFO_0004611	low density lipoprotein cholesterol measurement	measurement	EFO_0004611	Lipid or lipoprotein measurement	Metabolic
0.7093	4.08E-71	3.75E-68	PGS002605	EFO_0004611	low density lipoprotein cholesterol measurement	low density lipoprotein cholesterol measurement	EFO_0004611	Lipid or lipoprotein measurement	Metabolic	PGS001933	EFO_0004611	low density lipoprotein cholesterol measurement	measurement	EFO_0004611	Cardiovascular measurement	Cardiovascular
0.7093	4.12E-71	3.79E-68	PGS000773	EFO_0000305	breast carcinoma	breast carcinoma	EFO_0000305	Cancer	Neoplasms	PGS000487	EFO_0000305	breast carcinoma	breast carcinoma	EFO_0000305	Cancer	Neoplasms
0.7093	4.12E-71	3.79E-68	PGS000773	EFO_0000305	breast carcinoma	breast carcinoma	EFO_0000305	Cancer	Neoplasms	PGS000485	EFO_0000305	breast carcinoma	breast carcinoma	EFO_0000305	Cancer	Neoplasms
0.7093	4.18E-71	3.83E-68	PGS001230	EFO_0004338	body weight	body weight	EFO_0004338	Body measurement	Body measurement	PGS001958	EFO_0005093	hip circumference	hip circumference	EFO_0005093	Body measurement	Body measurement
0.7093	4.21E-71	3.87E-68	PGS000578	EFO_0001663	prostate carcinoma	prostate carcinoma	EFO_0001663	Cancer	Neoplasms	PGS000160	EFO_0001663	prostate carcinoma	prostate carcinoma	EFO_0001663	Cancer	Neoplasms
0.7093	4.21E-71	3.87E-68	PGS000577	EFO_0001663	prostate carcinoma	prostate carcinoma	EFO_0001663	Cancer	Neoplasms	PGS000160	EFO_0001663	prostate carcinoma	prostate carcinoma	EFO_0001663	Cancer	Neoplasms
0.7093	4.28E-71	3.93E-68	PGS002434	EFO_0004340	body mass index	body mass index	EFO_0004340	Body measurement	Body measurement	PGS002385	EFO_0004340	body mass index	body mass index	EFO_0004340	Body measurement	Body measurement
0.7092	4.38E-71	4.01E-68	PGS001451	EFO_0006930	brain volume measurement|neuroimaging measurement	brain volume measurement	EFO_0006930	Other measurement	Neurological	PGS001472	EFO_0006930	measurement	brain volume measurement	EFO_0006930	Other measurement	Neurological
0.7092	4.39E-71	4.03E-68	PGS002782	EFO_0005689	non-high density lipoprotein cholesterol measurement	non-high density lipoprotein cholesterol measurement	EFO_0005689	Lipid or lipoprotein measurement	Metabolic	PGS000824	EFO_0004611	low density lipoprotein cholesterol measurement	measurement	EFO_0004611	Lipid or lipoprotein measurement	Metabolic
0.7092	4.39E-71	4.03E-68	PGS002782	EFO_0005689	non-high density lipoprotein cholesterol measurement	non-high density lipoprotein cholesterol measurement	EFO_0005689	Lipid or lipoprotein measurement	Metabolic	PGS000824	EFO_0004611	low density lipoprotein cholesterol measurement	measurement	EFO_0004611	Cardiovascular measurement	Cardiovascular
0.7092	4.39E-71	4.03E-68	PGS002782	EFO_0005689	non-high density lipoprotein cholesterol measurement	non-high density lipoprotein cholesterol measurement	EFO_0005689	Cardiovascular measurement	Cardiovascular	PGS000824	EFO_0004611	low density lipoprotein cholesterol measurement	measurement	EFO_0004611	Cardiovascular measurement	Cardiovascular
0.7092	4.39E-71	4.03E-68	PGS002782	EFO_0005689	non-high density lipoprotein cholesterol measurement	non-high density lipoprotein cholesterol measurement	EFO_0005689	Cardiovascular measurement	Cardiovascular	PGS000824	EFO_0004611	low density lipoprotein cholesterol measurement	measurement	EFO_0004611	Lipid or lipoprotein measurement	Metabolic
0.7092	4.46E-71	4.09E-68	PGS002173	EFO_0009270	heel bone mineral density	heel bone mineral density	EFO_0009270	Other measurement	Skeletal	PGS002534	EFO_0003923	bone density	bone density	EFO_0003923	Other measurement	Skeletal
0.7092	4.47E-71	4.10E-68	PGS002764	HP_0003124	Hypercholesterolemia	Hypercholesterolemia	HP_0003124	Other trait	Metabolic	PGS002556	EFO_0004611	low density lipoprotein cholesterol measurement	measurement	EFO_0004611	Lipid or lipoprotein measurement	Metabolic
0.7092	4.47E-71	4.10E-68	PGS002764	HP_0003124	Hypercholesterolemia	Hypercholesterolemia	HP_0003124	Other trait	Metabolic	PGS002556	EFO_0004611	low density lipoprotein cholesterol measurement	measurement	EFO_0004611	Cardiovascular measurement	Cardiovascular
0.7091	4.64E-71	4.25E-68	PGS001683	EFO_0006930	brain volume measurement|neuroimaging measurement	brain volume measurement	EFO_0006930	Other measurement	Neurological	PGS001685	EFO_0006930	measurement	brain volume measurement	EFO_0006930	Other measurement	Neurological
-0.7091	4.74E-71	4.35E-68	PGS000884	EFO_0005208	glomerular filtration rate	glomerular filtration rate	EFO_0005208	Other measurement	Genitourinary	PGS003243	EFO_0010472	creatine measurement	creatine measurement	EFO_0010472	Other measurement	Metabolic
0.7091	4.76E-71	4.36E-68	PGS003191	EFO_0004631	vitamin D measurement	vitamin D measurement	EFO_0004631	Lipid or lipoprotein measurement	Metabolic	PGS003193	EFO_0004631	vitamin D measurement	vitamin D measurement	EFO_0004631	Lipid or lipoprotein measurement	Metabolic

0.7091	4.87E-71	4.46E-68	PGS001105	EFO_0007800	body fat percentage	body fat percentage	EFO_0007800	Body measurement	Body measurement	PGS001227	EFO_0004342	waist circumference	waist circumference	EFO_0004342	Body measurement	Body measurement
0.7091	4.88E-71	4.47E-68	PGS001687	EFO_0006930	brain volume measurement|neuroimaging measurement	brain volume measurement	EFO_0006930	Other measurement	Neurological	PGS001664	EFO_0006930	measurement	brain volume measurement	EFO_0006930	Other measurement	Neurological
0.7091	4.90E-71	4.49E-68	PGS000860	EFO_0005208	glomerular filtration rate	glomerular filtration rate	EFO_0005208	Other measurement	Genitourinary	PGS000885	EFO_0005208	glomerular filtration rate	glomerular filtration rate	EFO_0005208	Other measurement	Genitourinary
[truncated: 20,009,811 more chars]
